# Supplementary material for: Neutrophil extracellular trap formation and gene programs distinguish TST/IGRA sensitization outcomes among Mycobacterium tuberculosis exposed persons living with HIV
Source: PLoS Genet. 2023 Aug 24;19(8):e1010888. doi: 10.1371/journal.pgen.1010888 (PMC10470897; doi:10.1371/journal.pgen.1010888)
Supplement: S1 Table — (PDF) [file pgen.1010888.s001.pdf]

S1 Table: Antibodies used for Flow Cytometry Analysis of Contaminating Cell Populations

Staining step	Fluorophore	Specificity	Supplier	Clone	Catalogue Number
**Surface Staining**	BUV496	CD16	BD	3G8	612945
	V450	CD66b	BD	G10F5	561649
**Staining After Perm/Wash Incubation**	BV650	CD15	BioLegend	W6D3	323034
	Spark Blue 550	CD45	BioLegend	2D1	368549
	PE-eFluor-610	CD14	Thermo Fisher (Invitrogen)	61D3	61-0149-42
	PE-Cy5	CD3	BD	UCHT1	561007
