# Supplementary material for: Neutrophil extracellular trap formation and gene programs distinguish TST/IGRA sensitization outcomes among Mycobacterium tuberculosis exposed persons living with HIV
Source: PLoS Genet. 2023 Aug 24;19(8):e1010888. doi: 10.1371/journal.pgen.1010888 (PMC10470897; doi:10.1371/journal.pgen.1010888)
Supplement: S2 Table — (PDF) [file pgen.1010888.s002.pdf]

S2 Table: Cell population distribution of isolated PMN from HITTIN and HIT, as determined by flow cytometry

**Characteristic**	**HITTIN[a], N = 14 (3 missing values)[*]**	**HIT[b], N = 10 (1 missing value)[*]**	**p-value[**]**
**CD15+ CD66b+**	92.70% (87.05, 93.53)	94.35% (93.65, 97.08)	0.02**
**CD16+ (Neutrophils)**	86.90 (80.28, 92.90)	90.45 (86.10, 93.25)	0.28
**CD16- $CD14_{low}$ (Eosinophils)**	4.33 (1.51, 7.23)	3.63 (2.11, 6.60)	0.93
**CD15- CD66b-**	7.31% (6.44, 12.95)	5.67% (2.92, 6.32)	0.03**
**CD3- CD14- (Other)**	0.30 (0.17, 0.74)	0.39 (0.18, 0.58)	0.66
**CD3- CD14+ (Monocytes)**	0.07 (0.04, 0.12)	0.11 (0.07, 0.19)	0.22
**CD3+ (T-cells)**	6.70 (6.10, 12.43)	4.55 (2.27, 6.00)	0.02**

[*]Median (IQR); [**]Wilcoxon rank sum test

[a] HITTIN (HIV-1-infected persistently TB, tuberculin and IGRA negative), [b] HIT (HIV-1-infected IGRA positive tuberculin positive)
