# Supplementary material for: Neutrophil extracellular trap formation and gene programs distinguish TST/IGRA sensitization outcomes among Mycobacterium tuberculosis exposed persons living with HIV
Source: PLoS Genet. 2023 Aug 24;19(8):e1010888. doi: 10.1371/journal.pgen.1010888 (PMC10470897; doi:10.1371/journal.pgen.1010888)
Supplement: S3 Table — (PDF) [file pgen.1010888.s003.pdf]

<u>Supplementary table 3</u> : Differential gene expression testing results

		Average Expression	Log2FC Expression values			Adj.P.Value	Log2FC Expression values			Adj.P.Value
Ensembl_ID	Gene	All	HITTIN 1h inf	HIT 1h inf	HITTINxHIT 1h inf	HITTINxHIT 1h inf	HITTIN 6h inf	HIT 6h inf	HITTINxHIT 6h inf	HITTINxHIT 6h inf
ENSG00000013810	*TACC3*	6.290964045	-0.002851691	-0.012062479	0.009210788	0.999933887	-0.920121901	-2.064248081	1.14412618	4.0e-08
ENSG00000198728	*LDB1*	6.566089821	-0.018328008	-0.022618037	0.004290029	0.999933887	-0.537086423	-1.179098795	0.642012372	4.1e-08
ENSG00000163950	*SLBP*	4.391521096	0.007184897	-0.067021321	0.074206218	0.999933887	-1.092490973	-2.271706574	1.179215602	4.1e-08
ENSG00000114770	*ABCC5*	4.598229697	-0.04577187	-0.007035407	-0.038736463	0.999933887	-0.38288356	-1.107346016	0.724462456	1.55e-07
ENSG00000154889	*MPPE1*	4.986227645	-0.060510226	-0.04892043	-0.011589796	0.999933887	-0.956374604	-2.120342821	1.163968216	1.55e-07
ENSG00000148411	*NACC2*	4.429865479	0.028911995	-0.028333317	0.057245312	0.999933887	-0.686865258	-1.720275735	1.033410477	1.55e-07
ENSG00000115525	*ST3GAL5*	3.212199503	0.024936133	0.056258708	-0.031322575	0.999933887	-0.909640132	-2.028942914	1.119302783	1.55e-07
ENSG00000114650	*SCAP*	5.894223641	0.00407177	0.000219602	0.003852167	0.999933887	-0.693582353	-1.491069581	0.797487227	2.04e-07
ENSG00000186469	*GNG2*	7.636126174	-0.086645275	-0.039984134	-0.046661142	0.999933887	1.689878598	2.786825127	-1.096946529	2.83e-07
ENSG00000136490	*LIMD2*	8.224782826	0.046063834	0.020552663	0.02551117	0.999933887	-0.334981953	-1.088005044	0.753023091	3.77e-07
ENSG00000175115	*PACS1*	7.156963363	0.017294307	0.023147182	-0.005852875	0.999933887	-0.389540031	-1.115206526	0.725666495	4.56e-07
ENSG00000149177	*PTPRJ*	8.105117631	0.003543127	-0.02488696	0.028430087	0.999933887	0.993095255	1.778970509	-0.785875254	5.85e-07
ENSG00000176454	*LPCAT4*	3.751378867	-0.076903478	0.084847201	-0.161750679	0.999933887	-0.215394507	-0.926666514	0.711272007	7.08e-07
ENSG00000097033	*SH3GLB1*	7.561685911	-0.037566006	-0.061047052	0.023481046	0.999933887	0.055818994	0.609377741	-0.553558747	7.28e-07
ENSG00000142599	*RERE*	7.111115304	-0.001125043	0.025483521	-0.026608564	0.999933887	-0.879698007	-1.831043409	0.951345402	7.28e-07
ENSG00000126882	*FAM78A*	4.354335938	0.035313925	0.020032206	0.015281719	0.999933887	-0.360049992	-1.140014822	0.77996483	8.67e-07
ENSG00000077420	*APBB1IP*	7.011032353	0.068260876	0.038463138	0.029797738	0.999933887	-0.644671435	-1.358051197	0.713379763	1.247e-06
ENSG00000148660	*CAMK2G*	6.00175391	-0.019914597	0.019206591	-0.039121189	0.999933887	-0.485044854	-1.247596411	0.762551557	1.247e-06
ENSG00000130475	*FCHO1*	5.369262812	0.067658605	0.054474871	0.013183734	0.999933887	-0.62047067	-1.578634166	0.958163497	1.403e-06
ENSG00000168906	*MAT2A*	5.788810601	-0.12898742	-0.053301496	-0.075685924	0.999933887	-0.404587622	-1.0040006	0.599412978	1.405e-06
ENSG00000132359	*RAP1GAP2*	6.965216034	-0.055247579	0.034752219	-0.089999798	0.999933887	-0.5328804	-1.334797713	0.801917313	2.145e-06
ENSG00000198909	*MAP3K3*	6.925593001	0.006309852	0.032385554	-0.026075702	0.999933887	-0.923243447	-1.751441154	0.828197708	2.245e-06
ENSG00000065809	*FAM107B*	7.626215891	-0.049455645	-0.058092221	0.008636576	0.999933887	0.950935916	1.58013648	-0.629200565	2.469e-06
ENSG00000173020	*GRK2*	8.540296719	0.045699309	0.06557417	-0.019874861	0.999933887	-0.358906437	-0.959921588	0.601015151	2.469e-06
ENSG00000169220	*RGS14*	6.20992342	0.041852281	0.115571347	-0.073719066	0.999933887	-0.548504028	-1.459109152	0.910605124	2.469e-06
ENSG00000172375	*C2CD2L*	3.480366741	0.013879221	0.174006408	-0.160127187	0.999933887	-0.334832564	-1.034191303	0.699358739	2.469e-06
ENSG00000082212	*ME2*	4.529195555	0.022371314	0.023416201	-0.001044887	0.999933887	-0.846288138	-1.593121487	0.746833348	3.069e-06
ENSG00000106608	*URGCP*	3.230348518	-0.04418902	0.036972839	-0.081161858	0.999933887	1.405187847	2.295210356	-0.890022509	3.854e-06
ENSG00000130699	*TAF4*	3.786223363	0.015236879	0.008683517	0.006553362	0.999933887	-0.455188327	-1.220182054	0.764993727	3.854e-06
ENSG00000168918	*INPP5D*	7.718757251	0.005114308	0.009818274	-0.004703966	0.999933887	-0.323458024	-0.807702399	0.484244374	4.0e-06
ENSG00000178104	*PDE4DIP*	4.264688646	0.032511927	-0.143211394	0.175723321	0.999933887	1.015127346	1.794306972	-0.779179626	4.628e-06
ENSG00000198231	*DDX42*	5.363265192	-0.034808736	-0.074098825	0.039290089	0.999933887	-0.321010591	-0.881219363	0.560208773	4.65e-06
ENSG00000146083	*RNF44*	7.444290972	0.008016684	0.030679566	-0.022662882	0.999933887	-0.499370932	-1.077023227	0.577652295	4.65e-06
ENSG00000167895	*TMC8*	6.777299354	-0.001634845	0.034679133	-0.036313978	0.999933887	-0.472444916	-1.143139929	0.670695014	5.27e-06
ENSG00000133943	*DGLUCY*	6.132360015	-0.001855521	0.005003189	-0.006858711	0.999933887	-0.218912398	-0.742483871	0.523571473	5.325e-06
ENSG00000123329	*ARHGAP9*	8.165973367	-0.007638712	-0.00276802	-0.004870692	0.999933887	-0.66738706	-1.185433618	0.518046558	5.845e-06
ENSG00000119844	*AFTPH*	7.209865128	-0.046276039	-0.115350946	0.069074907	0.999933887	0.439159178	0.924488316	-0.485329138	6.046e-06
ENSG00000088833	*NSFL1C*	5.661026915	0.002455419	-0.011287075	0.013742495	0.999933887	-0.458833826	-0.987479167	0.528645342	6.357e-06
ENSG00000075420	*FNDC3B*	8.237662676	-0.0133526	0.004402784	-0.017755384	0.999933887	1.197128388	1.898007669	-0.700879281	6.798e-06
ENSG00000169231	*THBS3*	3.535581999	-0.001929168	0.006431848	-0.008361016	0.999933887	-0.389862815	-1.030149936	0.640287121	7.538e-06
ENSG00000172932	*ANKRD13D*	7.146472498	0.057239192	0.097639191	-0.040399999	0.999933887	-0.490647134	-1.121310456	0.630663322	7.652e-06
ENSG00000172794	*RAB37*	5.656575342	0.017405499	-0.017140746	0.034546245	0.999933887	-0.393957541	-1.127790842	0.733833302	7.652e-06
ENSG00000197142	*ACSL5*	6.375869596	0.011310521	-0.055101964	0.066412485	0.999933887	1.221588824	1.841295156	-0.619706332	1.005e-05
ENSG00000136280	*CCM2*	6.106257077	-0.03415799	0.054801455	-0.088959445	0.999933887	-0.777418987	-1.50719577	0.729776783	1.0254e-05
ENSG00000084070	*SMAP2*	8.502520365	0.021460197	0.014435331	0.007024866	0.999933887	-0.571759356	-1.248351251	0.676591896	1.045e-05
ENSG00000061938	*TNK2*	6.416439929	0.03391351	0.075237887	-0.041324376	0.999933887	-0.456161307	-1.179131658	0.722970352	1.057e-05
ENSG00000072736	*NFATC3*	4.746903342	0.005816827	0.016761045	-0.010944217	0.999933887	-0.708766503	-1.584622945	0.875856442	1.0571e-05
ENSG00000143418	*CERS2*	5.242249155	0.076751762	0.088002973	-0.011251211	0.999933887	-0.503960036	-1.108604218	0.604644181	1.0619e-05
ENSG00000122515	*ZMIZ2*	6.094058829	0.056423391	0.023522079	0.032901312	0.999933887	0.987495903	1.606907823	-0.619411921	1.0619e-05
ENSG00000127663	*KDM4B*	6.902984478	-0.036326011	0.021069235	-0.057395246	0.999933887	-0.3991895	-1.045417531	0.646228031	1.0619e-05
ENSG00000149289	*ZC3H12C*	4.31250693	-0.02985739	-0.04336922	0.01351183	0.999933887	1.120532112	1.944860009	-0.824327897	1.0619e-05
ENSG00000170776	*AKAP13*	7.855959673	-0.033706668	-0.039203124	0.005496456	0.999933887	0.404817359	1.014002547	-0.609185187	1.1278e-05
ENSG00000124496	*TRERF1*	5.660039947	-0.002085982	0.036342486	-0.038428468	0.999933887	-0.2960214	-0.862413658	0.566392257	1.1278e-05
ENSG00000164938	*TP53INP1*	5.951823499	-0.03912612	-0.012460146	-0.026665974	0.999933887	-0.821019442	-1.388679632	0.56766019	1.1755e-05
ENSG00000108479	*GALK1*	2.938215054	0.035637383	0.010113401	0.025523982	0.999933887	-0.840456606	-1.821683638	0.981227032	1.1755e-05
ENSG00000114446	*IFT57*	3.614690425	-0.067203241	-0.15600716	0.088803919	0.999933887	1.22506003	2.118661648	-0.893601618	1.3402e-05
ENSG00000197024	*ZNF398*	3.661984467	-0.060360522	-0.013476937	-0.046883585	0.999933887	-1.060979036	-2.001919911	0.940940875	1.4634e-05
ENSG00000124067	*SLC12A4*	4.159691512	0.051831849	0.03428606	0.017545789	0.999933887	-0.736896536	-1.559236391	0.822339855	1.4772e-05
ENSG00000187109	*NAP1L1*	6.509610016	-0.077393259	-0.114708373	0.037315114	0.999933887	0.227178643	0.685234027	-0.458055384	1.7612e-05
ENSG00000129911	*KLF16*	4.350480746	0.113846363	0.167121941	-0.053275578	0.999933887	-1.068546571	-2.102400418	1.033853847	1.8637e-05
ENSG00000072274	*TFRC*	6.715511047	-0.052872077	-0.072672126	0.019800048	0.999933887	1.066976556	1.83596925	-0.768992693	1.8637e-05
ENSG00000167522	*ANKRD11*	7.652069794	-0.008438045	-0.025374392	0.016936347	0.999933887	-0.403440064	-0.784640018	0.381199955	1.8637e-05
ENSG00000178764	*ZHX2*	6.062754902	-0.022794807	-0.014171798	-0.008623009	0.999933887	1.048461858	1.636783233	-0.588321375	1.9029e-05
ENSG00000261221	*ZNF865*	4.581461258	0.088814707	-0.017832338	0.106647045	0.999933887	-0.670236674	-1.443582102	0.773345428	1.934e-05
ENSG00000170266	*GLB1*	3.989794072	-0.010154649	-0.029577025	0.019422376	0.999933887	-0.574528202	-1.170385833	0.595857631	1.934e-05
ENSG00000185963	*BICD2*	5.246868351	0.003997328	0.074577185	-0.070579856	0.999933887	-0.857017	-1.604623298	0.747606298	1.934e-05
ENSG00000161980	*POLR3K*	1.896842638	0.069435916	-0.155579532	0.225015448	0.999933887	-1.102369579	-2.147970051	1.045600472	2.0533e-05
ENSG00000158470	*B4GALT5*	7.967916792	0.027272438	0.03112881	-0.003856373	0.999933887	1.582174739	2.163203298	-0.581028559	2.1043e-05
ENSG00000175463	*TBC1D10C*	6.640421337	0.046697737	0.091331436	-0.044633699	0.999933887	-0.37231127	-1.134078509	0.761767239	2.1522e-05
ENSG00000164088	*PPM1M*	5.089046667	-0.035798282	-0.056602142	0.020803861	0.999933887	-0.692823402	-1.333613478	0.640790076	2.4268e-05
ENSG00000133874	*RNF122*	3.512510895	-0.069419139	0.005922269	-0.075341408	0.999933887	-0.651886704	-1.364651695	0.712764991	2.5203e-05
ENSG00000100605	*ITPK1*	7.041676962	-0.014329097	-0.000833266	-0.013495831	0.999933887	-0.625830863	-1.479689141	0.853858278	2.5203e-05
ENSG00000171522	*PTGER4*	9.112463595	0.09305502	0.028950047	0.064104972	0.999933887	0.985578739	1.515878348	-0.530299609	2.5572e-05
ENSG00000136161	*RCBTB2*	4.270111034	-0.1322119	-0.03888944	-0.09332246	0.999933887	-1.075706597	-1.952215626	0.876509029	2.6108e-05
ENSG00000107099	*DOCK8*	8.241036158	-0.039974702	-0.043777231	0.003802529	0.999933887	-0.254823142	-0.724429524	0.469606382	2.6108e-05
ENSG00000148158	*SNX30*	4.459368284	-0.05897294	-0.128370587	0.069397646	0.999933887	-0.632186835	-1.321057384	0.688870549	2.6108e-05
ENSG00000170265	*ZNF282*	3.061189479	-0.024343724	-0.032856269	0.008512545	0.999933887	-0.268789324	-1.028135314	0.759345989	2.6108e-05
ENSG00000262370	*AC108134.3*	4.170000055	-0.020055202	-0.016020937	-0.004034265	0.999933887	-0.117373798	-0.71117137	0.593797572	2.689e-05
ENSG00000104312	*RIPK2*	5.982029547	0.059481317	0.029719938	0.029761379	0.999933887	1.48382956	2.091243852	-0.607414292	2.7693e-05
ENSG00000111554	*MDM1*	2.958056966	-0.090142581	-0.177234868	0.087092287	0.999933887	0.674620612	1.68400263	-1.009382018	2.8417e-05
ENSG00000178927	*CYBC1*	6.512412249	0.009366682	-0.002301262	0.011667944	0.999933887	-0.454298837	-1.019453827	0.56515499	3.1216e-05
ENSG00000185033	*SEMA4B*	4.65768804	0.008379295	0.028968947	-0.020589652	0.999933887	-0.976389375	-1.809334944	0.832945568	3.1467e-05
ENSG00000142208	*AKT1*	6.856448615	0.038309326	0.050906596	-0.01259727	0.999933887	-0.428805041	-0.999983904	0.571178863	3.1468e-05
ENSG00000182095	*TNRC18*	7.292012628	0.029069332	0.087921854	-0.058852522	0.999933887	-0.715395605	-1.540606797	0.825211193	3.3674e-05
ENSG00000010322	*NISCH*	5.941426818	-0.007555315	0.032740067	-0.040295382	0.999933887	-0.287112182	-0.699489625	0.412377443	3.4586e-05
ENSG00000196535	*MYO18A*	5.31585968	0.052711226	-0.000782399	0.053493624	0.999933887	-0.211186292	-0.726004074	0.514817782	3.739e-05
ENSG00000105063	*PPP6R1*	6.873124656	0.02717147	0.028572972	-0.001401502	0.999933887	-0.359550968	-0.845333588	0.48578262	3.739e-05
ENSG00000197852	*INKA2*	5.611720601	-0.059848755	-0.039763265	-0.02008549	0.999933887	-1.519672723	-2.384656789	0.864984065	4.0313e-05
ENSG00000165671	*NSD1*	6.08950729	-0.02836576	-0.077824133	0.049458372	0.999933887	-0.612874938	-1.15168642	0.538811483	4.2202e-05
ENSG00000183323	*CCDC125*	3.439679479	-0.129614031	-0.040640955	-0.088973076	0.999933887	-0.853773109	-1.891981439	1.038208329	4.3566e-05
ENSG00000161203	*AP2M1*	5.99528036	0.022793665	-0.024275211	0.047068876	0.999933887	-0.303901376	-0.726249466	0.422348091	4.676e-05
ENSG00000110911	*SLC11A2*	5.095201037	0.000682727	-0.126628398	0.127311125	0.999933887	2.077431823	2.955633337	-0.878201515	4.7381e-05
ENSG00000168936	*TMEM129*	2.87590367	0.116278274	0.086295423	0.029982852	0.999933887	-0.220205142	-0.94808347	0.727878328	4.8639e-05
ENSG00000137642	*SORL1*	10.159646271	-0.052035552	-0.038401783	-0.013633769	0.999933887	-0.289240127	-0.748679516	0.459439388	5.021e-05
ENSG00000128791	*TWSG1*	1.764435395	-0.097533723	-0.095952561	-0.001581162	0.999933887	0.666664111	1.526086403	-0.859422292	5.021e-05
ENSG00000068024	*HDAC4*	6.01381678	-0.009347047	0.021609004	-0.030956051	0.999933887	-0.334793181	-0.841529845	0.506736664	5.054e-05
ENSG00000103569	*AQP9*	11.438079296	-0.062899547	-0.099385257	0.03648571	0.999933887	1.430266544	1.994875776	-0.564609232	5.1462e-05
ENSG00000206344	*HCG27*	3.853383578	-0.253277799	-0.151009364	-0.102268436	0.999933887	-1.647544731	-2.68769071	1.040145979	5.1573e-05
ENSG00000168887	*C2orf68*	4.376881428	-0.115748588	-0.122008639	0.006260051	0.999933887	-0.512220704	-1.039203996	0.526983292	5.1573e-05
ENSG00000127314	*RAP1B*	6.617679922	-0.060923537	-0.09583743	0.034913893	0.999933887	0.388562847	0.877727719	-0.489164872	5.1573e-05
ENSG00000141524	*TMC6*	6.747463663	0.071311779	0.09011674	-0.01880496	0.999933887	-0.310375703	-0.933315519	0.622939816	5.1573e-05

ENSG00000116685	*KIAA2013*	5.196244863	0.072549181	0.014480546	0.058068635	0.999933887	-0.601427321	-1.324402741	0.722975421	5.1573e-05
ENSG00000106144	*CASP2*	4.334164006	-0.011239216	-0.071730569	0.060491353	0.999933887	-0.218176748	-0.77462884	0.556452093	5.1573e-05
ENSG00000031081	*ARHGAP31*	2.971786361	0.017044277	0.028884149	-0.011839871	0.999933887	1.852292041	2.916988934	-1.064696893	5.1573e-05
ENSG00000091542	*ALKBH5*	5.248493509	0.058733965	0.016972187	0.041761778	0.999933887	-0.318851633	-0.883247477	0.564395844	5.2874e-05
ENSG00000181192	*DHTKD1*	3.810553508	-0.024623433	-0.032545574	0.007922141	0.999933887	-0.86826476	-1.660973322	0.792708562	5.3299e-05
ENSG00000122359	*ANXA11*	8.184550833	-0.017245939	0.001235212	-0.018481151	0.999933887	-0.32552756	-0.774590883	0.449063322	5.4974e-05
ENSG00000180992	*MRPL14*	2.299509152	0.016393501	0.096547399	-0.080153898	0.999933887	1.293195975	2.057455967	-0.764259992	5.4974e-05
ENSG00000070413	*DGCR2*	5.979323245	0.009887424	-0.007767047	0.017654471	0.999933887	-0.453665715	-1.14662462	0.692958905	5.4974e-05
ENSG00000275131	*AC241952.1*	2.358888181	-0.104409698	-0.013737424	-0.090672273	0.999933887	0.435835947	1.033259323	-0.597423376	5.6213e-05
ENSG00000023892	*DEF6*	6.218974363	0.029219153	0.069277476	-0.040058323	0.999933887	-0.238078285	-0.77677757	0.538699284	5.6213e-05
ENSG00000279369	*AC046185.3*	2.630421058	0.02849853	0.062661143	-0.034162613	0.999933887	-0.449390993	-1.335433753	0.886042759	5.6213e-05
ENSG00000104915	*STX10*	5.332392688	0.04363254	0.026144329	0.017488211	0.999933887	-0.562711525	-1.398504423	0.835792898	5.6302e-05
ENSG00000152804	*HHEX*	3.003777197	-0.119276793	0.011368368	-0.130645161	0.999933887	-1.346938269	-2.434663502	1.087725233	5.6527e-05
ENSG00000108306	*FBXL20*	4.689349271	-0.029796973	0.109142475	-0.138939448	0.999933887	-0.663461024	-1.302255225	0.638794201	5.959e-05
ENSG00000089159	*PXN*	7.629933757	0.010099607	-0.024240581	0.034340189	0.999933887	-0.823778252	-1.376827166	0.553048914	6.0851e-05
ENSG00000189339	*SLC35E2B*	5.428709483	0.026469614	-0.055181045	0.081650659	0.999933887	-0.221047606	-0.593639688	0.372592082	6.1934e-05
ENSG00000263528	*IKBKE*	3.867450284	-0.013214348	-0.075093642	0.061879295	0.999933887	1.181051084	2.188857082	-1.007805997	6.3123e-05
ENSG00000242247	*ARFGAP3*	6.822814623	0.005662773	-0.090122351	0.095785125	0.999933887	0.886759053	1.357889561	-0.471130508	6.3676e-05
ENSG00000141401	*IMPA2*	4.690593141	-0.007905947	-0.021418954	0.013513007	0.999933887	-1.158802175	-2.345322103	1.186519927	6.3676e-05
ENSG00000144597	*EAF1*	6.603504729	-0.007865281	-0.019469745	0.011604464	0.999933887	1.198690172	1.736121725	-0.537431553	6.5127e-05
ENSG00000112576	*CCND3*	6.348766398	0.089999466	0.072026484	0.017972981	0.999933887	-0.450282406	-1.068861119	0.618578713	6.6879e-05
ENSG00000153029	*MR1*	5.037120279	-0.008860535	-0.018056652	0.009196117	0.999933887	0.435331069	0.913862978	-0.478531909	6.6908e-05
ENSG00000123610	*TNFAIP6*	7.671591934	-0.006760387	0.057575992	-0.064336379	0.999933887	3.099319565	4.070842057	-0.971522492	6.6908e-05
ENSG00000110697	*PITPNM1*	6.636056624	0.112352551	0.053770388	0.058582163	0.999933887	-0.351277263	-0.893326514	0.542049251	6.8159e-05
ENSG00000166523	*CLEC4E*	7.983767091	-0.01256623	0.027780944	-0.040347174	0.999933887	1.993090714	2.5572695	-0.564178786	6.8159e-05
ENSG00000271601	*LIX1L*	3.074129974	-0.01591519	0.009927599	-0.025842789	0.999933887	-0.188866361	-0.823331048	0.634464686	7.1027e-05
ENSG00000125730	*C3*	4.290919655	0.059465496	-0.049403284	0.10886878	0.999933887	2.166974178	3.034212042	-0.867237864	7.2381e-05
ENSG00000204713	*TRIM27*	4.667633302	0.036192169	-0.086008445	0.122200614	0.999933887	-0.388922443	-0.956906405	0.567983961	7.6819e-05
ENSG00000187257	*RSBN1L*	4.572085456	0.011871371	0.023859848	-0.011988476	0.999933887	-0.822891909	-1.548249568	0.725357658	7.9437e-05
ENSG00000121578	*B4GALT4*	3.361789483	-0.03500484	-0.083963073	0.048958233	0.999933887	0.925886906	1.481551297	-0.555664391	7.9813e-05
ENSG00000123240	*OPTN*	4.964283456	0.019374357	-0.155232538	0.174606895	0.999933887	0.548645245	1.086644648	-0.537999403	8.1278e-05
ENSG00000167552	*TUBA1A*	7.94142701	-0.042793738	-0.088039737	0.045245998	0.999933887	-0.715743104	-1.398062696	0.682319592	8.14e-05
ENSG00000139613	*SMARCC2*	5.539934014	0.019094683	0.074318512	-0.055223829	0.999933887	-0.406135569	-0.837696565	0.431560997	8.14e-05
ENSG00000160285	*LSS*	4.285526782	-0.018440629	0.090762359	-0.109202988	0.999933887	2.109705668	3.229635513	-1.119929846	8.14e-05
ENSG00000104974	*LILRA1*	4.290693708	0.001177589	-0.038137169	0.039314758	0.999933887	-1.28278991	-2.261615323	0.978825413	8.14e-05
ENSG00000196782	*MAML3*	4.001320772	-0.073558127	0.031949396	-0.105507522	0.999933887	-0.393930238	-0.98721309	0.593282852	8.1771e-05
ENSG00000113368	*LMNB1*	6.680646682	-0.046376509	-0.065779898	0.01940339	0.999933887	0.651980356	1.147768305	-0.495787949	8.2386e-05
ENSG00000110934	*BIN2*	7.406867867	0.003393237	-0.010796694	0.014189931	0.999933887	-0.184319121	-0.681826507	0.497507386	8.2386e-05
ENSG00000163050	*COQ8A*	5.021133219	-0.018896949	0.026221704	-0.045118654	0.999933887	-0.586293826	-1.238320704	0.652026878	8.3487e-05
ENSG00000197555	*SIPA1L1*	6.957835753	0.003095838	-0.049461715	0.052557552	0.999933887	0.338868748	0.820855588	-0.48198684	8.3487e-05
ENSG00000091640	*SPAG7*	4.472679426	0.051224162	0.041107685	0.010116477	0.999933887	0.730351348	1.203792348	-0.473441	8.444e-05
ENSG00000099991	*CABIN1*	5.67584602	0.020933027	0.049385252	-0.028452225	0.999933887	-0.098379209	-0.617258916	0.518879707	8.5579e-05
ENSG00000105939	*ZC3HAV1*	6.109419084	0.012615292	-0.09398765	0.106602941	0.999933887	-0.385110172	-0.838310077	0.453199905	8.5579e-05
ENSG00000066294	*CD84*	5.440471952	-0.018768682	-0.120810176	0.102041494	0.999933887	0.294825836	0.940508819	-0.645682983	8.6003e-05
ENSG00000240849	*PEDS1*	2.338072948	-0.009268574	0.041826672	-0.051095246	0.999933887	0.399813848	1.120367051	-0.720553203	8.7406e-05
ENSG00000103502	*CDIPT*	5.141461283	0.051635765	-0.026973659	0.078609424	0.999933887	-0.4831706	-1.110995899	0.627825299	8.9675e-05
ENSG00000124570	*SERPINB6*	3.394423387	-0.068544206	-0.071909973	0.003365768	0.999933887	0.272745849	1.061919284	-0.789173434	8.9843e-05
ENSG00000186298	*PPP1CC*	5.197340673	-0.039660488	0.033208108	-0.072868596	0.999933887	-0.376431953	-0.832881125	0.456449171	9.0564e-05
ENSG00000100342	*APOL1*	5.066475479	0.020492725	-0.06172811	0.082220835	0.999933887	-0.881600206	-1.511286315	0.629686109	9.1108e-05
ENSG00000088832	*FKBP1A*	5.905197263	-0.068163957	-0.041480064	-0.026683893	0.999933887	-0.383000097	-0.965413562	0.582413465	9.2691e-05
ENSG00000124256	*ZBP1*	5.318297747	0.015451319	0.028634268	-0.013182949	0.999933887	-0.367032979	-0.786119739	0.41908676	9.2691e-05
ENSG00000080371	*RAB21*	8.036346702	0.011944323	0.037905317	-0.025960994	0.999933887	1.516672385	2.010171464	-0.49349908	9.516e-05
ENSG00000214753	*HNRNPUL2*	3.586903115	-0.075287413	-0.055959908	-0.019327505	0.999933887	-0.457184203	-1.098218715	0.641034512	0.000100004
ENSG00000100299	*ARSA*	5.837957626	0.032980544	0.010816811	0.022163733	0.999933887	-0.288934212	-0.747442864	0.458508652	0.000100004
ENSG00000170542	*SERPINB9*	6.820558712	0.003676961	0.113572317	-0.109895357	0.999933887	2.565527197	3.518728957	-0.95320176	0.000100004
ENSG00000178719	*GRINA*	7.662975626	0.038646411	0.033642533	0.005003877	0.999933887	1.500660243	2.082451341	-0.581791098	0.000101059
ENSG00000125731	*SH2D3A*	3.661076595	0.020906176	0.150335957	-0.129429781	0.999933887	1.626789012	2.519512345	-0.892723333	0.000101059
ENSG00000197912	*SPG7*	5.831660434	-0.030046924	0.030674696	-0.06072162	0.999933887	-0.220672906	-0.565327021	0.344654114	0.000102073
ENSG00000180891	*CUEDC1*	3.634562755	0.009476964	0.065754293	-0.056277329	0.999933887	-0.392863375	-1.136433405	0.74357003	0.000102073
ENSG00000096996	*IL12RB1*	4.505343621	0.093402075	0.010827342	0.082574734	0.999933887	-0.38277599	-0.975666368	0.592890378	0.000104397
ENSG00000111912	*NCOA7*	3.700613356	0.089662344	-0.082227976	0.171890321	0.999933887	-0.707243749	-1.593417553	0.886173805	0.000104397
ENSG00000100307	*CBX7*	5.363080207	0.057896487	-0.004923848	0.062820336	0.999933887	-0.638016176	-1.264594038	0.626577862	0.000109289
ENSG00000023445	*BIRC3*	6.421204193	-0.088746623	-0.144759827	0.056013204	0.999933887	0.779340429	1.798279315	-1.018938886	0.000114085
ENSG00000163082	*SGPP2*	3.402664208	0.072169955	0.134228532	-0.062058577	0.999933887	2.267611196	3.136170412	-0.868559217	0.000114085
ENSG00000116213	*WRAP73*	3.532723107	0.131608409	0.107729019	0.02387939	0.999933887	-0.323011888	-0.824377784	0.501365896	0.000118976
ENSG00000197183	*NOL4L*	5.112875044	0.071452455	0.056459665	0.01499279	0.999933887	-0.393018451	-0.836542711	0.44334426	0.000118976
ENSG00000104880	*ARHGEF18*	5.135943592	0.064915441	0.033448324	0.031467117	0.999933887	-0.26551078	-0.786976691	0.521465911	0.000121859
ENSG00000140443	*IGF1R*	6.948582491	-0.060027299	-0.01588316	-0.044144139	0.999933887	-0.780791648	-1.275370769	0.494579121	0.000123754
ENSG00000232934	*AL157786.1*	3.026417215	0.041150464	0.133862996	-0.092712532	0.999933887	1.576550003	2.268449334	-0.691899331	0.000123754
ENSG00000010295	*IFFO1*	3.970754318	-0.011498515	0.019905397	-0.031403912	0.999933887	-0.221712888	-0.763315457	0.541602569	0.000136424
ENSG00000132819	*RBM38*	5.991726698	0.062907836	0.021624543	0.041283293	0.999933887	-0.344132886	-0.824140168	0.480007282	0.000139671
ENSG00000235750	*KIAA0040*	5.364402475	0.100591763	0.032912034	0.067679729	0.999933887	-0.753789444	-1.375603873	0.62181443	0.000142944
ENSG00000121281	*ADCY7*	6.004016262	0.05978705	0.076236836	-0.016449786	0.999933887	-0.508883676	-0.92914088	0.420257204	0.000142944
ENSG00000074319	*TSG101*	6.097345071	0.022472082	-0.039837091	0.062309174	0.999933887	0.205614885	0.567317354	-0.361702469	0.000143164
ENSG00000115896	*PLCL1*	3.646926842	0.013248703	-0.053434519	0.066683222	0.999933887	0.151010362	0.671340931	-0.520330569	0.000146958
ENSG00000048828	*FAM120A*	6.34425053	0.011503481	-0.022970873	0.034474354	0.999933887	-0.239483254	-0.599813164	0.36032991	0.000149532
ENSG00000198863	*RUNDC1*	3.648199713	-0.062143697	0.016964836	-0.079108532	0.999933887	-0.638412895	-1.267145479	0.628732584	0.000152314
ENSG00000135439	*AGAP2*	5.161490322	0.060030877	0.056880968	0.00314991	0.999933887	-0.20874256	-0.750502793	0.541760233	0.000153201
ENSG00000172725	*CORO1B*	4.762069943	0.08879845	0.058170796	0.030627654	0.999933887	-0.747969281	-1.433017059	0.685047778	0.000156565
ENSG00000197965	*MPZL1*	4.888727371	0.011504512	-0.076379593	0.087884104	0.999933887	-0.933464548	-1.992756526	1.059291978	0.000158508
ENSG00000180354	*MTURN*	4.277437722	-0.080573611	-0.039359851	-0.04121376	0.999933887	-0.43242999	-0.926530197	0.494100207	0.000172528
ENSG00000165169	*DYNLT3*	4.023396564	-0.114430981	-0.06350791	-0.050923071	0.999933887	0.901744338	1.602722374	-0.700978036	0.000175985
ENSG00000010818	*HIVEP2*	7.533875946	0.024267247	-0.002534529	0.026801777	0.999933887	1.346339833	1.994733087	-0.648393254	0.000180899
ENSG00000167613	*LAIR1*	3.499649536	-0.012112946	0.128921423	-0.141034369	0.999933887	1.149612424	2.109860592	-0.960248168	0.000184766
ENSG00000111276	*CDKN1B*	6.332204871	-0.045795577	-0.200279819	0.154484241	0.999933887	-0.773917318	-1.415389256	0.641471939	0.000185381
ENSG00000141905	*NFIC*	4.712526922	0.025646017	-0.040679834	0.066325851	0.999933887	-0.80936435	-1.438005626	0.628641276	0.000186201
ENSG00000103490	*PYCARD*	4.3769037	0.033902641	-0.005391161	0.039293802	0.999933887	-0.758729503	-1.643990439	0.885260936	0.000186782
ENSG00000100068	*LRP5L*	3.100461582	0.006849981	-0.048149771	0.054999753	0.999933887	0.677048315	1.324148887	-0.647100572	0.000186782
ENSG00000124587	*PEX6*	3.578012649	0.003161306	0.086497605	-0.0833363	0.999933887	-0.248720005	-0.712705445	0.46398544	0.000186782
ENSG00000186687	*LYRM7*	2.29513875	0.02550131	-0.067437314	0.092938624	0.999933887	-0.079707415	0.687291178	-0.766998593	0.000191707
ENSG00000124702	*KLHDC3*	3.790460894	0.010600706	-0.060145579	0.070746285	0.999933887	-0.239328912	-0.741042468	0.501713556	0.000192577
ENSG00000143507	*DUSP10*	4.982359534	-0.031765178	-0.005886149	-0.025879029	0.999933887	0.910562651	1.459647185	-0.549084535	0.000197108
ENSG00000119950	*MXI1*	4.822318728	0.09962565	0.020452526	0.079173124	0.999933887	-1.05002184	-1.832587214	0.782565374	0.00019954
ENSG00000127666	*TICAM1*	6.008258797	0.078513534	0.042936764	0.03557677	0.999933887	2.014155525	2.632645442	-0.618489917	0.00019954
ENSG00000133805	*AMPD3*	6.947313633	-0.083981354	-0.009213496	-0.074767858	0.999933887	1.872941879	2.502154351	-0.629212472	0.000201389
ENSG00000142197	*DOP1B*	4.418206273	-0.035808556	0.048208942	-0.084017497	0.999933887	-0.719442141	-1.264569401	0.545127261	0.000201389
ENSG00000130340	*SNX9*	4.1904169	0.032847287	-0.078637676	0.111484963	0.999933887	0.781112874	1.466774496	-0.685661622	0.000201389
ENSG00000076242	*MLH1*	3.731248342	-0.058820782	0.079538146	-0.138358928	0.999933887	1.097328401	1.790026518	-0.692698116	0.000205097
ENSG00000197779	*ZNF81*	2.839199618	-0.063000261	-0.058524756	-0.004475504	0.999933887	0.105650043	0.810010081	-0.704360038	0.000211925
ENSG00000144401	*METTL21A*	3.543826341	0.042827908	0.117403515	-0.074575607	0.999933887	0.59696454	1.118630297	-0.521665757	0.000211925
ENSG00000090020	*SLC9A1*	6.281828726	0.009281339	0.026961341	-0.017680002	0.999933887	-0.18598371	-0.599601541	0.413617831	0.000211925
ENSG00000263264	*AC119396.1*	3.21210731	0.077452603	-0.002382725	0.079835327	0.999933887	-0.137448377	-0.788321593	0.650873216	0.000213046
ENSG00000254087	*LYN*	10.502904072	-0.011637782	-0.039977884	0.028340103	0.999933887	0.852734655	1.302740101	-0.450005446	0.000213046
ENSG00000171443	*ZNF524*	2.22337691	0.152550734	0.016627653	0.135923081	0.999933887	-0.376794998	-1.315647808	0.93885281	0.000213281
ENSG00000122733	*PHF24*	1.577472214	-0.06604745	0.011915885	-0.077963335	0.999933887	1.334187996	2.14514802	-0.810960024	0.000214438

ENSG00000238227	*TMEM250*	3.747208982	0.065702932	-0.023351946	0.089054878	0.999933887	-0.504419353	-1.249701776	0.745282423	0.000215256
ENSG00000166477	*LEO1*	2.066744886	-0.071753102	-0.064423821	-0.007329281	0.999933887	0.566903971	1.284676792	-0.717772821	0.000223747
ENSG00000262246	*CORO7*	5.21504005	0.015307925	0.038076806	-0.02276888	0.999933887	-0.173068285	-0.497947414	0.324879129	0.000223747
ENSG00000164032	*H2AZ1*	5.766563792	0.002520891	-0.07981449	0.082335381	0.999933887	-0.20648341	-0.67029474	0.46381133	0.000223747
ENSG00000204839	*MROH6*	3.579884365	0.116811623	0.060763397	0.056048226	0.999933887	-0.78492525	-1.56131998	0.77639473	0.000225643
ENSG00000166398	*GARRE1*	3.657842062	0.027310631	0.11871207	-0.091401439	0.999933887	-0.139213279	-0.593874348	0.454661069	0.000225643
ENSG00000196209	*SIRPB2*	4.433157527	-0.03982362	-0.001358977	-0.038464643	0.999933887	-0.722619928	-1.461170698	0.73855077	0.000227422
ENSG00000100060	*MFNG*	6.055318048	-0.020099496	-0.028167576	0.008068079	0.999933887	-0.902707106	-1.534194412	0.631487306	0.000227699
ENSG00000165474	*GJB2*	4.018471658	0.184063499	0.070394379	0.11366912	0.999933887	1.678213457	2.502046137	-0.82383268	0.000228996
ENSG00000175137	*SH3BP5L*	4.99787884	-0.065102059	-0.010215797	-0.054886262	0.999933887	-0.847646549	-1.458054322	0.610407773	0.000228996
ENSG00000123416	*TUBA1B*	3.855245703	0.054544998	0.022145335	0.032399663	0.999933887	-0.354632706	-1.025497907	0.670865201	0.000228996
ENSG00000114353	*GNAI2*	9.158639486	0.04023449	0.01855277	0.02168172	0.999933887	-0.476032843	-1.002195513	0.52616267	0.000228996
ENSG00000108406	*DHX40*	5.550744809	-0.024207967	-0.090557877	0.06634991	0.999933887	0.36465064	0.764964832	-0.400314192	0.000228996
ENSG00000122986	*HVCN1*	3.250315266	-0.00690454	0.004096068	-0.011000608	0.999933887	-0.731850894	-1.461740116	0.729889222	0.000230904
ENSG00000124659	*TBCC*	4.457067302	-0.058493622	-0.175751266	0.117257644	0.999933887	-0.427415617	-0.890889827	0.46347421	0.000233644
ENSG00000021355	*SERPINB1*	7.089210047	0.051527359	-0.044536822	0.096064181	0.999933887	1.493446749	2.117840147	-0.624393398	0.000239943
ENSG00000048740	*CELF2*	7.782255196	0.055473523	-0.027244395	0.082717919	0.999933887	-0.726154195	-1.298467065	0.572312869	0.000241325
ENSG00000135842	*NIBAN1*	9.195632183	0.019526462	-0.028262603	0.047789065	0.999933887	1.126985936	1.717986609	-0.591000673	0.000241325
ENSG00000275302	*CCL4*	9.130925942	0.379507849	0.38471106	-0.005203211	0.999933887	3.717792632	4.862261055	-1.144468424	0.000246643
ENSG00000119335	*SET*	6.321347653	-0.066031836	-0.04504822	-0.020983616	0.999933887	-0.356648609	-0.826923858	0.470275249	0.000246643
ENSG00000164620	*RELL2*	2.201792452	-0.095371238	0.099071222	-0.19444246	0.999933887	-0.768903198	-1.521731526	0.752828328	0.000246643
ENSG00000146828	*SLC12A9*	6.695554705	0.03973703	0.074704003	-0.034966973	0.999933887	-0.120766451	-0.687442116	0.566675665	0.000246643
ENSG00000138964	*PARVG*	5.878723171	0.018340927	0.041109021	-0.022768095	0.999933887	-0.183683898	-0.561071913	0.377388016	0.000246643
ENSG00000145779	*TNFAIP8*	5.4141109	-0.032590625	-0.008176978	-0.024413647	0.999933887	1.0323358	1.664727325	-0.632391525	0.000246901
ENSG00000198740	*ZNF652*	4.731758649	0.001417028	-0.037529531	0.038946559	0.999933887	-1.01688726	-1.774359002	0.757471741	0.000250265
ENSG00000115956	*PLEK*	12.023675126	0.075079335	0.037220306	0.037859029	0.999933887	1.597892467	2.061758473	-0.463866005	0.000252492
ENSG00000196684	*HSH2D*	6.88649446	-0.042401097	-0.001750812	-0.040650285	0.999933887	-0.437522359	-0.900319364	0.462797006	0.000252492
ENSG00000180198	*RCC1*	2.912076758	0.040071405	0.173637518	-0.133566114	0.999933887	0.577798494	1.307192435	-0.729393941	0.000252492
ENSG00000205189	*ZBTB10*	3.938061568	0.024748339	-0.012201382	0.036949721	0.999933887	1.089135579	1.985584247	-0.896448668	0.000252492
ENSG00000100647	*SUSD6*	9.45735636	0.010436892	0.036888069	-0.026451177	0.999933887	0.91140039	1.309033844	-0.397633454	0.000252492
ENSG00000136026	*CKAP4*	7.607937659	0.004202874	0.014878684	-0.010675811	0.999933887	1.270295966	1.965786258	-0.695490292	0.000252492
ENSG00000198911	*SREBF2*	5.854413947	0.02169526	-0.037149774	0.058845034	0.999933887	0.565988174	1.027213129	-0.461224955	0.000257374
ENSG00000169925	*BRD3*	4.164455573	0.010431938	0.020369462	-0.009937525	0.999933887	-0.651337506	-1.327132039	0.675794534	0.000257374
ENSG00000164251	*F2RL1*	2.759032672	-0.037616033	0.117203937	-0.15481997	0.999933887	-2.413552025	-4.098744666	1.685192641	0.000257464
ENSG00000159314	*ARHGAP27*	7.668155691	0.047550806	0.088952478	-0.041401672	0.999933887	-0.595777113	-1.089746916	0.493969803	0.000262944
ENSG00000109654	*TRIM2*	0.00120883	-0.078697902	0.451130405	-0.529828307	0.999933887	0.532934931	1.580905084	-1.047970153	0.000263444
ENSG00000130560	*UBAC1*	3.695429311	0.032003507	0.043271158	-0.011267651	0.999933887	-0.216301756	-0.784712087	0.568410332	0.000263767
ENSG00000163110	*PDLIM5*	4.578542113	0.03823955	0.112531303	-0.074291753	0.999933887	0.756530142	1.166392234	-0.409862092	0.000272337
ENSG00000102871	*TRADD*	4.735924686	0.071324524	0.013234666	0.058089857	0.999933887	-0.290282686	-0.894060355	0.603777668	0.000276518
ENSG00000110844	*PRPF40B*	-0.122007712	-0.108806516	0.201079919	-0.309886435	0.999933887	1.484003775	3.008363348	-1.524359573	0.000290446
ENSG00000165168	*CYBB*	6.738982697	-0.070108547	-0.031186093	-0.038922455	0.999933887	0.602982377	1.080449674	-0.477467297	0.00029364
ENSG00000112339	*HBS1L*	4.4047058	-0.061257071	-0.064107555	0.002850484	0.999933887	0.574541671	1.0764278	-0.50188613	0.00029364
ENSG00000145569	*OTULINL*	3.706960469	-0.094460687	0.005058795	-0.099519482	0.999933887	0.263474523	0.833424946	-0.569950423	0.000295879
ENSG00000157514	*TSC22D3*	9.134040049	-0.011916777	-0.104292187	0.09237541	0.999933887	-0.77628887	-1.228715918	0.452427048	0.000296261
ENSG00000168246	*UBTD2*	4.18125607	-0.075763025	-0.228083976	0.152320952	0.999933887	1.503379877	2.098187896	-0.594808019	0.000297103
ENSG00000118046	*STK11*	5.313184241	0.064348163	0.112190306	-0.047842143	0.999933887	-0.308829625	-0.871492187	0.562662562	0.000310927
ENSG00000178623	*GPR35*	2.1171776	-0.008914785	-0.167741273	0.158826488	0.999933887	0.347240485	1.175361781	-0.828121297	0.00031148
ENSG00000127481	*UBR4*	9.070915414	0.018468391	-0.00327772	0.021746111	0.999933887	0.825319599	1.29239217	-0.467072571	0.0003116
ENSG00000226479	*TMEM185B*	6.284323945	0.045320704	0.028668138	0.016652566	0.999933887	1.311359819	1.763462082	-0.452102263	0.000320855
ENSG00000275183	*LENG9*	-0.003696788	0.407066343	0.36159922	0.045467124	0.999933887	0.923062846	2.367513803	-1.444450957	0.000322891
ENSG00000155307	*SAMSN1*	7.361672702	-0.035232391	-0.093150808	0.057918417	0.999933887	1.375488844	2.23342943	-0.857940586	0.000328361
ENSG00000120913	*PDLIM2*	5.763933627	0.046706991	0.058095803	-0.011388813	0.999933887	-0.592193905	-1.071276454	0.479082548	0.000328961
ENSG00000177963	*RIC8A*	6.06040413	0.049903089	0.051216703	-0.001313614	0.999933887	-0.27344288	-0.673720428	0.400277548	0.000336036
ENSG00000127311	*HELB*	3.07806817	-0.074746483	-0.080984999	0.006238516	0.999933887	0.920101277	1.56364098	-0.643539703	0.000340246
ENSG00000108651	*UTP6*	3.753831768	-0.101602368	-0.011021808	-0.090580559	0.999933887	-0.338873643	-0.905998575	0.567124931	0.000342831
ENSG00000064687	*ABCA7*	7.140526705	0.053165691	0.070036064	-0.016870373	0.999933887	-0.21182583	-0.662920736	0.451094905	0.000342831
ENSG00000162894	*FCMR*	5.566905152	0.018916731	-0.040110136	0.059026867	0.999933887	-0.379283865	-0.980961236	0.601677372	0.000342831
ENSG00000063854	*HAGH*	3.37349388	-0.015526495	0.121366734	-0.136893229	0.999933887	-0.235978833	-0.815820986	0.579842153	0.000342831
ENSG00000096968	*JAK2*	4.829666692	-0.065669532	-0.074804238	0.009134706	0.999933887	0.140770073	0.872101852	-0.731331779	0.00034346
ENSG00000171206	*TRIM8*	6.149454054	0.051304978	0.039997705	0.011307272	0.999933887	-0.375453055	-0.832709426	0.457256371	0.000347638
ENSG00000185010	*F8*	2.899725049	-0.049423081	0.015389995	-0.064813076	0.999933887	1.183654891	1.908150233	-0.724495342	0.000351263
ENSG00000163545	*NUAK2*	4.734755249	-0.116094494	-0.094025629	-0.022068865	0.999933887	-1.312199132	-2.114633122	0.80243399	0.000363508
ENSG00000247596	*TWF2*	4.720285522	-0.00883377	0.057236771	-0.06607054	0.999933887	-0.942269909	-1.618419363	0.676149455	0.000363508
ENSG00000163513	*TGFBR2*	6.746729634	-0.037999402	-0.006431059	-0.031568343	0.999933887	-0.320455523	-0.787632824	0.467177301	0.000365152
ENSG00000078804	*TP53INP2*	7.470845163	0.101793777	0.016483737	0.08531004	0.999933887	1.015564483	1.625605549	-0.610041065	0.000380181
ENSG00000130723	*PRRC2B*	6.421379404	0.039470274	0.024987753	0.01448252	0.999933887	-0.153940435	-0.480676553	0.326736118	0.000380941
ENSG00000104518	*GSDMD*	5.285067435	0.0620982	0.110069298	-0.047971099	0.999933887	-0.260582224	-0.955633255	0.695051032	0.000380941
ENSG00000124466	*LYPD3*	0.952918453	0.142397702	0.154981695	-0.012583993	0.999933887	1.587694586	2.685839716	-1.09814513	0.000382875
ENSG00000177666	*PNPLA2*	6.112612197	0.023720823	0.030934588	-0.007213765	0.999933887	-0.357530952	-0.767364966	0.409834014	0.000382875
ENSG00000188559	*RALGAPA2*	6.850085421	0.017017115	0.034169465	-0.01715235	0.999933887	0.382574072	0.771778747	-0.389204675	0.000382875
ENSG00000163393	*SLC22A15*	3.934525979	-0.019241434	-0.028810187	0.009568752	0.999933887	-0.92340487	-1.49638136	0.57297649	0.000382875
ENSG00000007376	*RPUSD1*	2.748897741	0.144232633	0.072775968	0.071456665	0.999933887	-0.219763514	-0.866349871	0.646586357	0.000383359
ENSG00000198771	*RCSD1*	5.690393357	-0.037845445	0.053356796	-0.091202241	0.999933887	-0.379265409	-0.884299882	0.505034473	0.000383359
ENSG00000166527	*CLEC4D*	4.227749523	0.030030717	-0.128974488	0.159005205	0.999933887	1.594116308	2.412598667	-0.818482359	0.000383359
ENSG00000196954	*CASP4*	7.761161173	0.004543991	-0.084567162	0.089111153	0.999933887	0.53897292	0.959991855	-0.421018934	0.000383359
ENSG00000171720	*HDAC3*	3.999238741	0.053194648	-0.021468723	0.074663371	0.999933887	-0.404073892	-0.810007824	0.405933932	0.000384019
ENSG00000186407	*CD300E*	2.944192076	-0.069054116	-0.051933632	-0.017120484	0.999933887	-0.659544443	-1.434269886	0.774725444	0.000386294
ENSG00000115594	*IL1R1*	7.790224633	-0.023434844	-0.015918881	-0.007515963	0.999933887	0.379607241	0.77954311	-0.399935869	0.000389323
ENSG00000152518	*ZFP36L2*	7.932307048	-0.022566771	0.005889456	-0.028456227	0.999933887	-0.439024626	-0.861787134	0.422762508	0.000389323
ENSG00000128245	*YWHAH*	3.545677759	-0.010536197	0.077245447	-0.087781645	0.999933887	-0.352069516	-0.856682704	0.504613188	0.000398451
ENSG00000104522	*GFUS*	3.637358339	0.082537477	-0.0002963	0.082833777	0.999933887	-0.127333287	-0.660884069	0.533550781	0.000419257
ENSG00000116815	*CD58*	6.27287418	-0.034811517	-0.045229629	0.010418111	0.999933887	1.036876948	1.528359243	-0.491482295	0.000424593
ENSG00000169032	*MAP2K1*	4.963971073	0.022843882	0.001441474	0.021402408	0.999933887	0.535697411	0.957248545	-0.421551134	0.000429549
ENSG00000027075	*PRKCH*	5.93754361	0.002291963	0.002888777	-0.000596814	0.999933887	0.748574278	1.351965659	-0.603391382	0.00043842
ENSG00000128815	*WDFY4*	5.418324845	0.004615865	0.024106198	-0.019490333	0.999933887	-0.273189742	-0.696552277	0.423362535	0.000441885
ENSG00000100030	*MAPK1*	6.920482394	-0.046051535	-0.028984524	-0.017067011	0.999933887	-0.656273187	-1.077412142	0.421138955	0.000442001
ENSG00000101350	*KIF3B*	4.550306827	-0.047295356	-0.032874933	-0.014420423	0.999933887	0.516258302	1.032159192	-0.515900889	0.000442001
ENSG00000127948	*POR*	6.064997002	0.079536915	0.010435683	0.069101232	0.999933887	0.789430653	1.261225254	-0.471794602	0.000443174
ENSG00000102172	*SMS*	4.029263704	0.052803808	0.028452808	0.024351	0.999933887	0.304335439	0.810299267	-0.505963828	0.000443174
ENSG00000180233	*ZNRF2*	4.063599814	0.171745845	-0.028537575	0.200283419	0.999933887	-0.450071691	-1.074345674	0.624273983	0.000449751
ENSG00000128487	*SPECC1*	2.78044192	0.002909515	-0.003160547	0.006070062	0.999933887	0.23153459	0.95709718	-0.72556259	0.000449751
ENSG00000173221	*GLRX*	5.513715977	-0.104411118	-0.168002616	0.063591498	0.999933887	-0.43955077	-0.921778461	0.482227691	0.000451956
ENSG00000105323	*HNRNPUL1*	7.201004861	0.022898354	-0.015782767	0.038681121	0.999933887	-0.503150111	-0.873394095	0.370243984	0.000452872
ENSG00000068650	*ATP11A*	6.540336762	0.00547934	0.011911333	-0.006431993	0.999933887	-0.28324549	-0.648754681	0.365509191	0.000453076
ENSG00000235385	*LINC02154*	2.068111595	-0.127646427	-0.173474972	0.045828545	0.999933887	1.699931992	2.378956931	-0.679024938	0.000453222
ENSG00000166797	*CIAO2A*	3.629525476	0.038839121	-0.068898878	0.107737999	0.999933887	-0.939918514	-1.69863732	0.758718807	0.000453571
ENSG00000140931	*CMTM3*	5.116688532	0.013726457	-0.002136896	0.015863354	0.999933887	-0.504204894	-1.038553627	0.534348732	0.000453593
ENSG00000162616	*DNAJB4*	2.139147296	-0.222427696	-0.08489688	-0.137530816	0.999933887	1.3540377	2.165484192	-0.811446492	0.000455605
ENSG00000105771	*SMG9*	4.473063719	0.081676359	0.083399173	-0.001722813	0.999933887	2.235076587	2.966552158	-0.73147557	0.000455605
ENSG00000125384	*PTGER2*	5.015057251	0.061045575	0.035178339	0.025867236	0.999933887	1.680499444	2.564656976	-0.884157532	0.000455605
ENSG00000156675	*RAB11FIP1*	7.228766406	-0.03905698	-0.023485442	-0.015571538	0.999933887	-1.426057154	-2.078969782	0.652912628	0.000456712
ENSG00000129245	*FXR2*	3.982898192	0.066477037	0.16269059	-0.096213553	0.999933887	-0.349901564	-0.79284361	0.442942046	0.000456921
ENSG00000110400	*NECTIN1*	2.975497652	-0.098099612	-0.049537529	-0.048562083	0.999933887	1.479229522	2.286187203	-0.806957681	0.00046129
ENSG00000059378	*PARP12*	5.767711384	0.016420195	0.025727682	-0.009307487	0.999933887	-0.151420814	-0.515575294	0.36415448	0.000461673
ENSG00000100644	*HIF1A*	9.375688509	-0.084711337	-0.146426113	0.061714776	0.999933887	1.147411103	1.90057234	-0.753161237	0.000463771

ENSG00000136059	*VILL*	3.904656882	0.040150453	0.06683193	-0.026681477	0.999933887	2.086237209	2.910918988	-0.824681779	0.000463771
ENSG00000173868	*PHOSPHO1*	7.338655545	0.008237831	-0.00488136	0.013119191	0.999933887	-1.088414248	-1.700667664	0.612251416	0.000463771
ENSG00000079459	*FDFT1*	4.697173366	0.035090254	-0.024609319	0.059699572	0.999933887	-0.236137191	-0.651858652	0.415721461	0.000465902
ENSG00000099860	*GADD45B*	10.247449495	0.171428636	0.155579217	0.015849419	0.999933887	1.363104249	1.928093589	-0.56498934	0.000474137
ENSG00000173011	*TADA2B*	5.235598144	0.03179577	0.003956989	0.027838781	0.999933887	-0.624137195	-1.105875984	0.481738788	0.000474137
ENSG00000117298	*ECE1*	9.089812894	0.014221799	0.005735823	0.008485975	0.999933887	1.742953283	2.317089955	-0.574136672	0.000474137
ENSG00000162433	*AK4*	0.490796962	-0.235465689	-0.327402117	0.091936428	0.999933887	2.188280806	2.949740164	-0.761459358	0.000478436
ENSG00000182511	*FES*	5.072112503	0.018858534	0.024954152	-0.006095617	0.999933887	-0.570558208	-1.254934828	0.684376619	0.000478436
ENSG00000118503	*TNFAIP3*	10.401396196	0.131013551	0.131600428	-0.000586877	0.999933887	2.325472362	2.980843456	-0.655371093	0.000481081
ENSG00000120217	*CD274*	4.631863574	0.049397087	0.064695207	-0.015298121	0.999933887	1.997103329	2.703446219	-0.70634289	0.000493276
ENSG00000099910	*KLHL22*	2.8548099	-0.057505516	0.005317983	-0.062823499	0.999933887	-0.243420278	-0.708316686	0.464896409	0.00049429
ENSG00000232442	*MHENCR*	2.703767444	0.01094313	-0.051906132	0.062849262	0.999933887	-0.1354225	-0.729809852	0.594387352	0.000498713
ENSG00000166501	*PRKCB*	7.655081114	-0.068956297	-0.041310712	-0.027645584	0.999933887	-0.423642147	-0.757976277	0.33433413	0.00049883
ENSG00000130313	*PGLS*	4.008544025	0.074734564	-0.010460198	0.085194763	0.999933887	-0.532247002	-1.150565308	0.618318307	0.00049883
ENSG00000198315	*ZKSCAN8*	4.353249745	-0.012552264	-0.106695115	0.094142851	0.999933887	0.168265699	0.822822848	-0.654557149	0.00049918
ENSG00000110080	*ST3GAL4*	4.883979989	0.120873664	0.139814247	-0.018940583	0.999933887	0.923438431	1.416935402	-0.493496971	0.000509158
ENSG00000073849	*ST6GAL1*	5.137139756	0.008299139	-0.032144187	0.040443326	0.999933887	-0.315752399	-0.703174092	0.387421693	0.000509158
ENSG00000115649	*CNPPD1*	5.224900151	-0.036621252	0.000187896	-0.036809148	0.999933887	-0.706268412	-1.173552934	0.467284523	0.000512618
ENSG00000055208	*TAB2*	8.063379728	-0.023003317	-0.074135522	0.051132205	0.999933887	0.699293902	1.101446521	-0.402152619	0.00051355
ENSG00000160191	*PDE9A*	0.723217692	0.049836408	0.064928413	-0.015092004	0.999933887	0.076881875	1.118035375	-1.041153499	0.000521709
ENSG00000095015	*MAP3K1*	6.831575066	-0.030440972	-0.049034851	0.018593878	0.999933887	-0.168520606	0.297427913	-0.46594852	0.000525996
ENSG00000116729	*WLS*	5.197853349	0.028390501	-0.010487496	0.038877997	0.999933887	-0.787430261	-1.367410468	0.579980207	0.000525996
ENSG00000102554	*KLF5*	4.238786433	0.020212749	0.013776478	0.006436271	0.999933887	0.814897465	1.343313048	-0.528415583	0.000525996
ENSG00000167778	*SPRYD3*	4.086048572	-0.004189092	0.04094156	-0.045130652	0.999933887	-0.392752689	-0.927242874	0.534490185	0.000525996
ENSG00000005844	*ITGAL*	7.968619303	0.026285021	0.022364862	0.003920159	0.999933887	-0.054442639	-0.349665831	0.295223192	0.000528974
ENSG00000142405	*NLRP12*	4.998051598	-0.076259096	-0.083419673	0.007160577	0.999933887	-1.253327554	-1.91626857	0.662941016	0.000531247
ENSG00000277406	*SEC22B4P*	2.696943022	-0.117219606	0.132610968	-0.249830574	0.999933887	1.625679534	2.193104622	-0.567425088	0.000531247
ENSG00000125656	*CLPP*	3.563830608	0.053177859	-0.09749213	0.150669989	0.999933887	-0.166994519	-0.830337775	0.663343257	0.00054406
ENSG00000147394	*ZNF185*	4.281441854	0.031550595	-0.018498934	0.05004953	0.999933887	-0.82086849	-1.364822976	0.543954487	0.000553891
ENSG00000125910	*S1PR4*	6.306404756	0.016717606	-0.043721018	0.060438625	0.999933887	-0.697309509	-1.412671686	0.715362178	0.000558687
ENSG00000099817	*POLR2E*	5.730153765	0.0226226	0.020026596	0.002596004	0.999933887	-0.28996169	-0.677846517	0.387884827	0.000558834
ENSG00000111087	*GLI1*	2.624007187	0.049798876	-0.044227185	0.094026061	0.999933887	-0.553569614	-1.140575758	0.587006145	0.000564584
ENSG00000119403	*PHF19*	3.831820289	-0.018326151	0.030036577	-0.048362727	0.999933887	0.603947943	1.258501589	-0.654553646	0.000564584
ENSG00000163931	*TKT*	8.272076849	0.010518431	-0.002207398	0.012725829	0.999933887	-0.336796986	-0.847302542	0.510505556	0.00056899
ENSG00000266173	*STRADA*	3.326938532	-0.010214006	-0.134750543	0.124536537	0.999933887	-0.518326979	-1.100155504	0.581828525	0.00056899
ENSG00000138614	*INTS14*	2.460627094	-0.006507736	0.02537633	-0.031884066	0.999933887	0.685669629	1.341883935	-0.656214306	0.000569404
ENSG00000286162	*AL162253.2*	1.64577002	0.13322837	0.087289541	0.045938829	0.999933887	1.57063582	2.467061202	-0.896425382	0.000581413
ENSG00000151465	*CDC123*	4.252510867	-0.053746532	-0.030487713	-0.023258819	0.999933887	-0.306430671	-0.771693294	0.465262623	0.000581413
ENSG00000068028	*RASSF1*	4.891462629	-0.037162803	0.02853351	-0.065696313	0.999933887	-0.13494986	-0.562207847	0.427257987	0.000581947
ENSG00000110324	*IL10RA*	7.422934672	0.072740418	0.102350273	-0.029609855	0.999933887	1.131844978	1.822963674	-0.691118696	0.000582956
ENSG00000116574	*RHOU*	2.726233695	-0.102730842	-0.214213816	0.111482974	0.999933887	0.78114959	1.839404265	-1.058254675	0.000582956
ENSG00000169756	*LIMS1*	6.915249345	-0.058639423	0.005851231	-0.064490654	0.999933887	1.638769898	2.220986789	-0.582216891	0.000582956
ENSG00000100596	*SPTLC2*	4.29971387	-0.041050623	-0.036920042	-0.004130581	0.999933887	-0.599764306	-1.122627777	0.522863471	0.000582956
ENSG00000154957	*ZNF18*	3.24696529	-0.051323353	0.011618395	-0.062941748	0.999933887	-0.523975766	-1.111147023	0.587171258	0.00059238
ENSG00000173276	*ZBTB21*	5.822255805	0.036906955	-0.091802073	0.128709028	0.999933887	0.541520042	1.044856748	-0.503336707	0.00059238
ENSG00000162924	*REL*	6.703556129	-0.03931007	-0.006483538	-0.032826532	0.999933887	1.469172903	2.266581537	-0.797408634	0.00059238
ENSG00000167077	*MEI1*	3.664762043	0.065662383	0.03245316	0.033209223	0.999933887	0.640884165	1.308964043	-0.668079877	0.000592893
ENSG00000150054	*MPP7*	4.126406659	-0.033362275	0.015469826	-0.048832101	0.999933887	0.002872586	0.497844836	-0.494972249	0.000598825
ENSG00000198920	*KIAA0753*	2.833926859	-0.071690608	-0.122054894	0.050364285	0.999933887	-0.641371467	-1.201962916	0.56059145	0.000599905
ENSG00000143207	*COP1*	6.046359176	-0.044050497	0.022748502	-0.066798999	0.999933887	-0.463585687	-0.90060972	0.437024033	0.000599905
ENSG00000023041	*ZDHHC6*	5.006196152	-0.005896617	0.003052504	-0.00894912	0.999933887	0.653613196	1.038760046	-0.385146849	0.000599905
ENSG00000187994	*RINL*	3.434543152	0.093907251	0.16280181	-0.068894559	0.999933887	-0.102796442	-0.660473923	0.557677481	0.000600908
ENSG00000163156	*SCNM1*	4.239916091	-0.04965325	-0.039081249	-0.010572001	0.999933887	-0.215464228	-0.634315967	0.418851739	0.000600908
ENSG00000168763	*CNNM3*	4.382311719	0.050485755	0.029042453	0.021443301	0.999933887	-0.337315054	-0.735048783	0.397733729	0.000602361
ENSG00000197070	*ARRDC1*	4.802213759	0.045818734	0.096673527	-0.050854792	0.999933887	-0.12106694	-0.608540687	0.487473747	0.000602361
ENSG00000172830	*SSH3*	4.489237102	0.025402329	0.071246487	-0.045844158	0.999933887	-0.977209232	-1.613043141	0.635833909	0.000602361
ENSG00000134070	*IRAK2*	8.465396421	0.007435236	-0.058204457	0.065639693	0.999933887	2.076817628	2.654400998	-0.577583369	0.000602361
ENSG00000167261	*DPEP2*	6.704467166	-0.051407234	-0.039027158	-0.012380076	0.999933887	-1.456076039	-2.074524735	0.618448696	0.000607851
ENSG00000112419	*PHACTR2*	3.486976267	-0.028876467	-0.085627665	0.056751197	0.999933887	0.029435882	0.904048215	-0.874612333	0.000607851
ENSG00000186074	*CD300LF*	5.549016988	-0.015508064	0.051273333	-0.066781397	0.999933887	-0.803353928	-1.3474114	0.544057472	0.000607851
ENSG00000173120	*KDM2A*	7.885612119	0.011230504	0.031453871	-0.020223368	0.999933887	-0.255324258	-0.587376362	0.332052105	0.000625152
ENSG00000105514	*RAB3D*	4.855767503	-0.086710129	-0.093705679	0.006995551	0.999933887	-1.256174382	-2.184413015	0.928238633	0.000636876
ENSG00000138821	*SLC39A8*	4.32104294	-0.034530771	-0.005733192	-0.028797579	0.999933887	1.769492516	2.698565707	-0.92907319	0.000638366
ENSG00000205339	*IPO7*	4.123677741	-0.065744837	-0.17258003	0.106835193	0.999933887	0.322654136	0.908415517	-0.585761381	0.000639185
ENSG00000173281	*PPP1R3B*	5.408562413	-0.096006164	-0.062085398	-0.033920766	0.999933887	-0.891823591	-1.415668102	0.52384451	0.000651613
ENSG00000107771	*CCSER2*	5.053372282	-0.039765717	-0.062880182	0.023114465	0.999933887	0.083338967	0.673996645	-0.590657678	0.000651613
ENSG00000075131	*TIPIN*	-1.022044372	-0.19702859	-0.060792726	-0.136235864	0.999933887	1.07889118	2.704298128	-1.625406948	0.000665217
ENSG00000144566	*RAB5A*	6.8954384	0.026013049	0.060220051	-0.034207002	0.999933887	0.419443151	0.726072029	-0.306628878	0.000668512
ENSG00000104660	*LEPROTL1*	5.133251522	0.023259172	-0.038540737	0.061799909	0.999933887	-0.248828957	-0.706030205	0.457201247	0.000668512
ENSG00000187147	*RNF220*	4.278335805	-0.048115165	-0.030773336	-0.017341829	0.999933887	-0.307283804	-0.664629197	0.357345393	0.000670753
ENSG00000176393	*RNPEP*	3.849141579	0.040557681	0.042538101	-0.00198042	0.999933887	-0.567800472	-1.205495463	0.637694991	0.000670753
ENSG00000138670	*RASGEF1B*	6.587929909	0.075328306	0.052262228	0.023066078	0.999933887	0.57346098	1.022968733	-0.449507753	0.000673004
ENSG00000151116	*UEVLD*	2.383167553	-0.050634726	-0.051910165	0.001275439	0.999933887	0.328326911	0.950051241	-0.62172433	0.000676611
ENSG00000026751	*SLAMF7*	9.659828825	-0.007269608	0.053395258	-0.060664866	0.999933887	1.051754266	1.549022172	-0.497267905	0.000677698
ENSG00000179119	*SPTY2D1*	6.269354632	0.058595202	-0.045067015	0.103662217	0.999933887	0.047757819	0.386816999	-0.33905918	0.000678012
ENSG00000162104	*ADCY9*	3.335054775	0.113220092	0.113348982	-0.00012889	0.999933887	2.573194043	3.638345053	-1.06515101	0.000678012
ENSG00000261222	*AC064805.1*	1.645408369	0.03630679	0.015269093	0.021037697	0.999933887	-0.847062844	-1.84769918	1.000636336	0.000685315
ENSG00000143815	*LBR*	6.380973389	0.00315357	-0.021314533	0.024468103	0.999933887	-0.897072195	-1.427534376	0.530462181	0.000691622
ENSG00000196547	*MAN2A2*	7.556823292	-0.024102457	0.018677027	-0.042779484	0.999933887	-0.89009938	-1.310174435	0.420075056	0.000691744
ENSG00000149547	*EI24*	2.555658236	0.017082948	0.154428479	-0.137345531	0.999933887	0.529022279	1.148405792	-0.619383512	0.000710166
ENSG00000177663	*IL17RA*	7.262781875	-0.023919452	-0.011395111	-0.012524341	0.999933887	-0.570077093	-1.012984885	0.442907792	0.000721705
ENSG00000180251	*SLC9A4*	1.371732711	0.207078331	-0.070057644	0.277135975	0.999933887	2.079929732	2.951919755	-0.871990023	0.000724211
ENSG00000197943	*PLCG2*	7.266836129	-0.026802566	0.007147802	-0.033950368	0.999933887	-0.507252827	-0.83656692	0.329314093	0.000728783
ENSG00000171314	*PGAM1*	4.506436938	0.055101634	-0.016881678	0.071983311	0.999933887	-0.339366049	-0.811000454	0.471634405	0.000731801
ENSG00000143772	*ITPKB*	7.056114795	0.035405451	0.020196007	0.015209444	0.999933887	0.209327187	0.579802154	-0.370474967	0.000735093
ENSG00000159339	*PADI4*	4.882174685	-0.025149354	0.006251592	-0.031400946	0.999933887	-0.205395259	-0.595604274	0.390209015	0.000735093
ENSG00000129566	*TEP1*	6.232780882	0.014995888	0.061454487	-0.046458599	0.999933887	-0.556279079	-0.973157754	0.416878675	0.000736532
ENSG00000170458	*CD14*	8.828296574	0.007846503	-0.018929823	0.026776326	0.999933887	-0.329386631	0.265528554	-0.594915184	0.000736532
ENSG00000143401	*ANP32E*	4.531938743	-0.050462953	-0.124863838	0.074400885	0.999933887	0.375289572	0.871837943	-0.496548371	0.000749858
ENSG00000135250	*SRPK2*	5.891751389	-0.033463949	-0.001353639	-0.03211031	0.999933887	-0.413705414	-0.918399866	0.504694452	0.000749858
ENSG00000265491	*RNF115*	3.803413278	-0.04844467	-0.003376867	-0.045067804	0.999933887	0.638162188	1.315038408	-0.67687622	0.000749858
ENSG00000134108	*ARL8B*	7.026558315	-0.092369359	-0.055571024	-0.036798335	0.999933887	0.44095787	0.785664713	-0.344706844	0.000750407
ENSG00000123908	*AGO2*	7.766068705	-0.049449539	-0.047085628	-0.00236391	0.999933887	0.366467311	0.721729036	-0.355261725	0.000776341
ENSG00000112146	*FBXO9*	4.362978734	0.02911889	0.086214681	-0.057095791	0.999933887	-0.364287062	-0.852573109	0.488286048	0.000779597
ENSG00000150977	*RILPL2*	9.535149527	0.009226912	0.051580092	-0.042353179	0.999933887	2.180197083	2.756574651	-0.576377568	0.000779597
ENSG00000011243	*AKAP8L*	4.658927034	-0.031900512	-0.020904691	-0.010995821	0.999933887	-0.314531712	-0.635171815	0.320640103	0.000782741
ENSG00000179630	*LACC1*	2.352570178	0.051173076	-0.164210185	0.215383261	0.999933887	0.705301748	1.667036183	-0.961734435	0.00078338
ENSG00000141956	*PRDM15*	2.547260076	0.034325687	0.048150192	-0.013824506	0.999933887	0.23418849	0.744319459	-0.510130969	0.000791789
ENSG00000168067	*MAP4K2*	5.304019328	0.053344371	0.046629137	0.006715233	0.999933887	-0.062772511	-0.393381702	0.330609191	0.000792826
ENSG00000078967	*UBE2D4*	1.073428295	-0.090216824	0.092820023	-0.183036848	0.999933887	0.724312503	1.614624345	-0.890311842	0.000792826
ENSG00000104081	*BMF*	5.535541113	0.009683369	-0.000739349	0.010422717	0.999933887	1.416592799	2.09209501	-0.675502211	0.000802845
ENSG00000047056	*WDR37*	5.040737169	-0.006846276	-0.027311885	0.020465609	0.999933887	-0.454609042	-0.84878803	0.394178988	0.000806064
ENSG00000138834	*MAPK8IP3*	7.416784767	0.024973187	0.087226296	-0.062253109	0.999933887	-0.515945747	-0.993374984	0.477429238	0.000815355
ENSG00000085449	*WDFY1*	5.472178937	-0.073871905	-0.049301209	-0.024570696	0.999933887	0.318503369	0.743729148	-0.425225779	0.000816688
ENSG00000157764	*BRAF*	6.945800454	0.025834561	0.015216423	0.010618139	0.999933887	0.433873071	0.829533951	-0.395660879	0.000816688

ENSG00000213398	*LCAT*	1.799986222	0.080204867	0.068330226	0.011874641	0.999933887	-0.654952177	-1.504975605	0.850023428	0.000831212
ENSG00000160326	*SLC2A6*	2.282232105	0.161355104	0.009933379	0.151421726	0.999933887	3.271499775	4.489742289	-1.218242514	0.000834147
ENSG00000115602	*IL1RL1*	2.905921682	-0.068124385	-0.009300448	-0.058823937	0.999933887	-0.018412791	0.525336114	-0.543748904	0.000851884
ENSG00000151702	*FLI1*	5.65072938	0.04251064	0.048062083	-0.005551444	0.999933887	-0.266649821	-0.702765479	0.436115658	0.000851884
ENSG00000213625	*LEPROT*	6.756932198	-0.06183044	-0.023409061	-0.038421379	0.999933887	-0.294947933	0.156533862	-0.451481795	0.000855648
ENSG00000102879	*CORO1A*	9.099271042	0.029189919	0.023617169	0.00557275	0.999933887	-0.628412864	-1.155227762	0.526814898	0.000855648
ENSG00000080815	*PSEN1*	7.967791916	-0.089330885	-0.046127572	-0.043203314	0.999933887	1.167122172	1.552611796	-0.385489624	0.000859511
ENSG00000095319	*NUP188*	4.088413094	-0.034942913	-0.010234276	-0.024708637	0.999933887	0.578225307	1.132824986	-0.554599679	0.000859531
ENSG00000151694	*ADAM17*	6.846157601	-0.081503171	-0.043276138	-0.038227033	0.999933887	0.945624397	1.271658452	-0.326034055	0.000861223
ENSG00000131242	*RAB11FIP4*	7.029733623	-0.096982499	-0.052327907	-0.044654592	0.999933887	-1.253543634	-1.794640701	0.541097067	0.000861223
ENSG00000108262	*GIT1*	4.285374505	0.03413015	0.020001222	0.014128928	0.999933887	-0.165869188	-0.568420673	0.402551486	0.000861223
ENSG00000215861	*AC245297.1*	1.500980679	-0.129264089	0.413986395	-0.543250484	0.999933887	1.258602537	1.912291656	-0.653689119	0.000861223
ENSG00000175471	*MCTP1*	5.880030353	-0.014736746	-0.023190288	0.008453543	0.999933887	1.225932422	1.82747085	-0.601538428	0.000861223
ENSG00000104973	*MED25*	6.499680055	-0.004589642	0.023811591	-0.028401233	0.999933887	-0.907996878	-1.381358628	0.473361751	0.000861223
ENSG00000196498	*NCOR2*	7.457743169	0.090605118	0.061155803	0.029449315	0.999933887	1.383891049	1.951659956	-0.567768907	0.000865401
ENSG00000198879	*SFMBT2*	6.935850546	-0.024493193	0.006517866	-0.031011058	0.999933887	1.280894524	1.802098047	-0.521203523	0.00086938
ENSG00000026508	*CD44*	10.012102911	-0.008263367	0.011141126	-0.019404493	0.999933887	1.173283413	1.669825102	-0.496541689	0.00086938
ENSG00000105967	*TFEC*	3.262959541	0.134275547	-0.016004502	0.150280049	0.999933887	2.592349255	3.634482164	-1.042132909	0.000869393
ENSG00000170448	*NFXL1*	3.001643868	-0.117824547	-0.023345124	-0.094479423	0.999933887	-0.429349902	-1.080396134	0.651046232	0.000873123
ENSG00000172732	*MUS81*	4.0947931	0.082357227	0.14626158	-0.063904353	0.999933887	-0.144765866	-0.543353734	0.398587868	0.000873123
ENSG00000137757	*CASP5*	3.749760443	-0.08609178	-0.182235994	0.096144214	0.999933887	1.512608821	2.273230805	-0.760621985	0.000873123
ENSG00000100811	*YY1*	6.093847832	-0.005007753	0.044118022	-0.049125775	0.999933887	-0.478857033	-0.840571919	0.361714886	0.000873123
ENSG00000160766	*GBAP1*	0.903382235	0.107460104	-0.254622086	0.36208219	0.999933887	-0.698924426	-1.772565537	1.073641111	0.000886957
ENSG00000156873	*PHKG2*	4.013398329	0.032914119	0.020865958	0.012048161	0.999933887	-0.26334179	-0.675186363	0.411844573	0.000886957
ENSG00000189319	*FAM53B*	5.863330618	-0.035389795	0.00251613	-0.037905926	0.999933887	-0.545307394	-1.078350952	0.533043558	0.000886957
ENSG00000182287	*AP1S2*	4.709134929	0.012312835	-0.171067645	0.18338048	0.999933887	1.070466726	1.564622083	-0.494155356	0.000886957
ENSG00000261804	*AC007342.4*	1.84888396	-0.016519493	-0.075906134	0.059386641	0.999933887	-0.846757813	-1.973536691	1.126778878	0.000888641
ENSG00000169621	*APLF*	0.236054238	-0.175551456	0.099786736	-0.275338192	0.999933887	1.203355123	2.106635919	-0.903280796	0.000892752
ENSG00000102882	*MAPK3*	6.17902598	0.029810834	0.017738284	0.012072551	0.999933887	-0.780748739	-1.254757156	0.474008417	0.000903596
ENSG00000111348	*ARHGDIB*	9.144516412	0.014736594	-0.05683368	0.071570274	0.999933887	-0.206022716	-0.658814279	0.452791563	0.000904363
ENSG00000151012	*SLC7A11*	6.158364357	-0.064944797	-0.057411143	-0.007533654	0.999933887	-0.073271427	0.432089152	-0.50536058	0.000909441
ENSG00000138646	*HERC5*	5.026477572	-0.083749164	-0.058704985	-0.025044179	0.999933887	-0.721078821	-1.275293766	0.554214945	0.00091068
ENSG00000084733	*RAB10*	7.088847968	0.047778287	0.063341354	-0.015563067	0.999933887	0.491744298	0.876478915	-0.384734617	0.000915528
ENSG00000100106	*TRIOBP*	6.021055611	0.054558684	0.063812048	-0.009253365	0.999933887	-0.618894151	-1.088074406	0.469180255	0.000915649
ENSG00000104960	*PTOV1*	5.037908963	0.049903056	0.10887393	-0.058970874	0.999933887	-0.278479871	-0.770054262	0.491574391	0.000926398
ENSG00000111732	*AICDA*	-1.068427222	0.311359986	-0.549775674	0.86113566	0.999933887	1.557603969	2.740640977	-1.183037008	0.000949765
ENSG00000182973	*CNOT10*	3.548674091	-0.0387956	-0.026976516	-0.011819084	0.999933887	-0.29947756	-0.80101289	0.501535331	0.000949765
ENSG00000146826	*MAP11*	4.819692171	0.107134509	0.116923024	-0.009788516	0.999933887	-0.386154864	-0.863227827	0.477072963	0.000952845
ENSG00000274265	*AC245297.3*	2.355286057	0.062817848	0.074202031	-0.011384183	0.999933887	0.783963714	1.313909565	-0.529945851	0.000960953
ENSG00000056558	*TRAF1*	6.65872219	0.043222643	0.027058333	0.01616431	0.999933887	2.604150443	3.449710752	-0.845560309	0.000960953
ENSG00000154237	*LRRK1*	4.15203469	-0.025959454	0.027358265	-0.053317719	0.999933887	-0.370903319	-0.93291908	0.562015761	0.000967348
ENSG00000108861	*DUSP3*	5.991821097	-0.058160242	-0.087420092	0.029259849	0.999933887	0.935414221	1.362509467	-0.427095247	0.000969831
ENSG00000038210	*PI4K2B*	2.971242188	-0.17442804	0.014339162	-0.188767203	0.999933887	0.884343533	1.736433205	-0.852089673	0.00097215
ENSG00000126216	*TUBGCP3*	4.009229963	-0.013935024	0.050176444	-0.064111468	0.999933887	-0.284708781	-0.723243092	0.43853431	0.00097215
ENSG00000139651	*ZNF740*	3.556275446	-0.03534286	-0.001449762	-0.033893097	0.999933887	-0.167301476	-0.551628541	0.384327066	0.00097297
ENSG00000123728	*RAP2C*	6.740101708	0.019185788	0.010604213	0.008581576	0.999933887	0.757529754	1.114401871	-0.356872117	0.000987054
ENSG00000122958	*VPS26A*	6.245553559	-0.065660389	-0.152938486	0.087278097	0.999933887	-0.075710106	0.29289067	-0.368600776	0.001006552
ENSG00000160310	*PRMT2*	6.084208728	0.022518318	-0.038198299	0.060716617	0.999933887	0.804957681	1.311656198	-0.506698517	0.001006552
ENSG00000179743	*FLJ37453*	2.086985545	-0.07488705	0.045696202	-0.120583252	0.999933887	-0.311897845	-0.915954757	0.604056912	0.001045357
ENSG00000085978	*ATG16L1*	4.460021644	-0.05552266	-0.017953022	-0.037569637	0.999933887	-0.107672693	-0.432284006	0.324611313	0.001057199
ENSG00000131979	*GCH1*	5.598911626	-0.050802097	0.024590322	-0.075392419	0.999933887	2.958338265	3.869760131	-0.911421867	0.001063286
ENSG00000272501	*AL662844.4*	4.527859513	-0.087503388	-0.089438793	0.001935404	0.999933887	-0.883104971	-1.433593599	0.550488628	0.001072907
ENSG00000116478	*HDAC1*	5.126536128	0.025448329	-0.042998062	0.068446392	0.999933887	-0.126543867	-0.50503495	0.378491083	0.001089166
ENSG00000137161	*CNPY3*	7.11298908	0.001965625	-0.010919968	0.012885593	0.999933887	-0.360322446	-0.729515254	0.369192808	0.001089166
ENSG00000120690	*ELF1*	9.074939424	-0.061614246	-0.144267855	0.082653609	0.999933887	-0.053605827	0.293197105	-0.346802932	0.001092888
ENSG00000108622	*ICAM2*	3.194515294	0.071919981	0.012510454	0.059409527	0.999933887	0.368944739	0.810031012	-0.441086273	0.00110226
ENSG00000166145	*SPINT1*	2.364680459	0.021940463	0.063219973	-0.041279509	0.999933887	-0.958334028	-1.938115495	0.979781468	0.00110226
ENSG00000049239	*H6PD*	4.55803922	0.010937577	-0.039203117	0.050140694	0.999933887	-0.348712024	-0.738936312	0.390224288	0.001113003
ENSG00000185338	*SOCS1*	3.75060889	0.164057176	0.11755812	0.046499056	0.999933887	1.072436563	1.656482596	-0.584046033	0.001126632
ENSG00000146070	*PLA2G7*	1.070755343	0.17249263	0.184580699	-0.012088069	0.999933887	0.488889488	1.141170228	-0.65228074	0.001132075
ENSG00000171163	*ZNF692*	3.702623715	0.028704578	0.056240757	-0.027536179	0.999933887	-0.356602666	-0.827265932	0.470663267	0.001132075
ENSG00000103415	*HMOX2*	4.43750536	-0.036498042	0.001993042	-0.038491084	0.999933887	-0.43704414	-1.004070863	0.567026722	0.001132167
ENSG00000105122	*RASAL3*	6.389077992	0.021825805	0.043155708	-0.021329902	0.999933887	-0.23921628	-0.574251043	0.335034762	0.001132167
ENSG00000160214	*RRP1*	4.524148065	0.063491947	0.082693821	-0.019201874	0.999933887	0.911728836	1.332408532	-0.420679696	0.001136208
ENSG00000141506	*PIK3R5*	9.557875607	-0.007780963	-0.001920783	-0.00586018	0.999933887	0.527022804	0.78487653	-0.257853726	0.00114191
ENSG00000167034	*NKX3-1*	1.007559537	0.019750232	-0.026367036	0.046117268	0.999933887	-1.049529014	-2.125213217	1.075684203	0.00114191
ENSG00000017260	*ATP2C1*	4.291924181	-0.052775512	-0.092500352	0.03972484	0.999933887	0.274393355	0.783468934	-0.509075578	0.001143123
ENSG00000040275	*SPDL1*	1.757446492	-0.151275343	-0.371452951	0.220177608	0.999933887	0.234800241	0.839891104	-0.605090863	0.001146891
ENSG00000162222	*TTC9C*	2.044275448	-0.020349162	-0.071342337	0.050993175	0.999933887	-0.312251873	-0.937431068	0.625179195	0.001148212
ENSG00000180539	*C9orf139*	1.824751912	0.008731282	0.130369365	-0.121638083	0.999933887	-0.376075397	-1.331684145	0.955608747	0.00115915
ENSG00000147813	*NAPRT*	4.931008226	0.056295556	0.015218807	0.041076749	0.999933887	-1.496180663	-2.196773914	0.700593251	0.001163393
ENSG00000196839	*ADA*	2.861694027	0.07900337	0.007658935	0.071344435	0.999933887	0.913933388	1.601717051	-0.687783663	0.001173433
ENSG00000163171	*CDC42EP3*	10.39180724	0.010058429	0.012642135	-0.002583705	0.999933887	0.272568063	0.587857049	-0.315288986	0.001173433
ENSG00000100883	*SRP54*	6.023390761	-0.067691779	0.025815955	-0.093507734	0.999933887	1.328188011	1.77615185	-0.447963839	0.001178705
ENSG00000075426	*FOSL2*	10.768004632	0.075277701	0.026471606	0.048806096	0.999933887	0.630111172	1.056156876	-0.426045704	0.001193821
ENSG00000168961	*LGALS9*	4.896452645	0.116125076	0.05471894	0.061406136	0.999933887	-0.694263001	-1.283483768	0.589220767	0.0012016
ENSG00000114738	*MAPKAPK3*	5.897413329	-0.020361045	-0.014909332	-0.005451713	0.999933887	-1.091721876	-1.593351009	0.501629134	0.001202766
ENSG00000163154	*TNFAIP8L2*	2.470249233	-0.203317114	-0.204203308	0.000886194	0.999933887	-0.633611626	-1.405861342	0.772249716	0.001203375
ENSG00000197646	*PDCD1LG2*	0.479990533	0.005199718	-0.365802241	0.371001958	0.999933887	0.247597036	1.132495571	-0.884898535	0.001204161
ENSG00000147421	*HMBOX1*	4.24244837	-0.018652999	0.004600376	-0.023253375	0.999933887	0.159688325	0.59708195	-0.437393625	0.001212255
ENSG00000128340	*RAC2*	9.934842254	0.028523023	-0.028474883	0.056997906	0.999933887	0.379539484	0.681054536	-0.301515052	0.001214153
ENSG00000074416	*MGLL*	4.795307858	0.047369024	0.213577207	-0.166208183	0.999933887	0.664667768	1.15282506	-0.488157292	0.001219929
ENSG00000137710	*RDX*	2.830110131	-0.150893298	-0.178397965	0.027504667	0.999933887	-0.012227297	0.521926042	-0.534153339	0.001257358
ENSG00000288156	*AC104530.1*	3.758414408	-0.175033884	-0.097406403	-0.077627481	0.999933887	-0.723844839	-1.453764954	0.729920115	0.00125987
ENSG00000204842	*ATXN2*	4.007151881	0.029050896	0.027529968	0.001520928	0.999933887	-0.545126031	-0.944641456	0.399515425	0.001263396
ENSG00000145911	*N4BP3*	2.847106788	0.120893809	0.002678365	0.118215444	0.999933887	1.204535875	1.844475502	-0.639939627	0.001280367
ENSG00000104976	*SNAPC2*	2.844501046	0.037631356	-0.112204886	0.149836242	0.999933887	-0.722880718	-1.244402894	0.521522175	0.001280706
ENSG00000138663	*COPS4*	3.675239641	-0.037775963	-0.124585966	0.086810003	0.999933887	-0.170443096	0.312967338	-0.483410434	0.001286398
ENSG00000149476	*TKFC*	2.355303561	0.098123379	-0.021821671	0.11994505	0.999933887	0.684391297	1.198409423	-0.514018126	0.001288706
ENSG00000213024	*NUP62*	4.876136998	-0.043611442	-0.001391667	-0.042219774	0.999933887	0.539259448	0.93562044	-0.396360992	0.001288706
ENSG00000157570	*TSPAN18*	2.208190651	0.115394062	0.096638306	0.018755756	0.999933887	0.637038653	1.370704979	-0.733666326	0.001299133
ENSG00000116731	*PRDM2*	7.153867851	-0.032073041	-0.080275769	0.048202728	0.999933887	0.482056475	0.803322457	-0.321265981	0.001299133
ENSG00000035687	*ADSS2*	4.642206822	-0.032814184	-0.054405914	0.02159173	0.999933887	-1.015124797	-1.59823131	0.583106514	0.001299882
ENSG00000082397	*EPB41L3*	1.667384647	0.073630332	-0.050037975	0.123668307	0.999933887	0.570728719	1.248389218	-0.677660499	0.001304861
ENSG00000197119	*SLC25A29*	4.7285602	0.039892733	-0.030781565	0.070674299	0.999933887	-0.760956199	-1.207032119	0.44607592	0.001306118
ENSG00000105520	*PLPPR2*	6.544207108	0.006071057	0.041838214	-0.035767157	0.999933887	-0.544997432	-1.044841099	0.499843667	0.001306118
ENSG00000155115	*GTF3C6*	2.655219403	-0.138237231	0.019047033	-0.157284264	0.999933887	2.001974612	2.838323193	-0.836348582	0.001321337
ENSG00000171860	*C3AR1*	7.852069919	-0.001817445	-0.054265615	0.05244817	0.999933887	1.060265875	1.63146335	-0.571197476	0.001331046
ENSG00000152558	*TMEM123*	7.716180565	-0.032923437	-0.115619034	0.082695597	0.999933887	0.028713314	0.474168258	-0.445454945	0.001332219
ENSG00000037637	*FBXO42*	4.781856494	-0.024074987	-0.010881739	-0.013193247	0.999933887	0.722491813	1.204036415	-0.481544603	0.001347282
ENSG00000165516	*KLHDC2*	3.849440126	-0.101499024	-0.032446414	-0.069052611	0.999933887	-0.241229743	-0.660661795	0.419432052	0.001351096
ENSG00000140030	*GPR65*	6.292342717	0.025927402	-0.031517234	0.057444636	0.999933887	0.962411716	1.506886099	-0.544474383	0.001351096
ENSG00000136560	*TANK*	8.228200072	-0.014876622	0.022539326	-0.037415949	0.999933887	0.801659315	1.333963831	-0.532304516	0.001351096
ENSG00000008516	*MMP25*	8.770077893	0.005702505	0.009882424	-0.004179919	0.999933887	-0.387193003	-0.807040034	0.419847031	0.001351096
ENSG00000110955	*ATP5F1B*	6.351470874	-0.013482022	-0.020443148	0.006961126	0.999933887	-0.11678379	-0.387918829	0.271135039	0.001360905

ENSG00000140932	*CMTM2*	6.650287658	-0.038273753	-0.064978863	0.02670511	0.999933887	-0.43487889	-1.038086622	0.603207733	0.001370793
ENSG00000158201	*ABHD3*	5.36684154	-0.029278807	-0.032717582	0.003438775	0.999933887	-0.444824932	-0.859422976	0.414598044	0.001373997
ENSG00000127328	*RAB3IP*	2.200302749	-0.049835669	-0.143462422	0.093626753	0.999933887	0.451126445	1.097024438	-0.645897993	0.001390956
ENSG00000067955	*CBFB*	4.357709591	-0.037838468	0.003283496	-0.041121963	0.999933887	-0.490513422	-0.865207711	0.374694289	0.001397599
ENSG00000146476	*ARMT1*	4.057822996	-0.06616615	-0.065403908	-0.000762242	0.999933887	0.115188619	0.489564042	-0.374375422	0.001398212
ENSG00000166128	*RAB8B*	8.422073549	-0.082839163	-0.138378184	0.055539021	0.999933887	0.468493465	0.953413518	-0.484920052	0.001410206
ENSG00000125779	*PANK2*	4.416017883	0.021439671	0.046380965	-0.024941294	0.999933887	-0.404803605	-0.808672361	0.403868756	0.001417129
ENSG00000015285	*WAS*	7.942319352	3.4829e-05	-0.004711243	0.004746072	0.999933887	-0.466864243	-0.813630026	0.346765783	0.001419612
ENSG00000104320	*NBN*	8.290914724	-0.108007119	-0.175803556	0.067796437	0.999933887	1.745953824	2.511749664	-0.765795841	0.001421977
ENSG00000155096	*AZIN1*	7.989130461	-0.092308378	-0.049106277	-0.043202101	0.999933887	1.601183406	2.073971957	-0.47278855	0.00142668
ENSG00000104866	*PPP1R37*	3.434734634	0.075795769	0.058308487	0.017487282	0.999933887	-0.304170148	-0.788976893	0.484806745	0.001427275
ENSG00000197860	*SGTB*	7.708349632	-0.043899454	-0.114931342	0.071031888	0.999933887	0.372939021	0.818817598	-0.445878577	0.001427275
ENSG00000257027	*AC010186.3*	2.200414169	-0.034075521	0.087204356	-0.121279877	0.999933887	0.839351153	1.729461296	-0.890110143	0.001427275
ENSG00000148290	*SURF1*	3.860321104	0.003662941	-0.05473195	0.05839489	0.999933887	-0.177253533	-0.560297813	0.383044281	0.001427275
ENSG00000128564	*VGF*	0.157970524	-0.145556878	0.299168243	-0.444725121	0.999933887	-0.575043985	-1.638403287	1.063359303	0.001445882
ENSG00000103495	*MAZ*	4.029472511	0.032788355	0.104761989	-0.071973634	0.999933887	-0.413368229	-0.956025557	0.542657328	0.001445882
ENSG00000113916	*BCL6*	9.921030222	0.009469631	-0.011243693	0.020713324	0.999933887	0.510006039	0.751846485	-0.241840446	0.001445882
ENSG00000035664	*DAPK2*	4.990465506	-0.012765326	0.055061394	-0.067826721	0.999933887	-0.289636685	-0.719050706	0.429414021	0.001445882
ENSG00000043462	*LCP2*	10.941819185	-0.006795142	-0.001225155	-0.005569987	0.999933887	0.921980152	1.258023786	-0.336043634	0.001445882
ENSG00000170638	*TRABD*	6.035562848	0.051131951	0.041558172	0.009573778	0.999933887	-0.082725185	-0.460201401	0.377476216	0.001447743
ENSG00000134802	*SLC43A3*	6.398534944	0.036693287	-0.006944367	0.043637654	0.999933887	1.589710385	2.031190276	-0.441479891	0.001447743
ENSG00000187266	*EPOR*	2.460028448	0.019134689	0.073763276	-0.054628588	0.999933887	-0.657894064	-1.255716873	0.597822809	0.001459735
ENSG00000112096	*SOD2*	12.211699964	-0.040862904	0.022933325	-0.063796229	0.999933887	2.031100578	2.529030648	-0.49793007	0.001465509
ENSG00000128594	*LRRC4*	5.863742671	0.020438484	0.039057027	-0.018618544	0.999933887	-1.568306981	-2.163316753	0.595009773	0.001470514
ENSG00000079805	*DNM2*	7.996709507	0.002575392	0.032184455	-0.029609062	0.999933887	-0.286296091	-0.562380583	0.276084492	0.001524737
ENSG00000148737	*TCF7L2*	4.718575352	-0.038280177	0.105987416	-0.144267593	0.999933887	0.571546136	0.941747091	-0.370200954	0.001530305
ENSG00000205356	*TECPR1*	5.493905349	0.037977813	0.053550041	-0.015572228	0.999933887	-0.289714419	-0.681834273	0.392119854	0.00154636
ENSG00000164603	*BMT2*	3.933299743	-0.00577163	-0.106498648	0.100727018	0.999933887	1.248532369	1.79381925	-0.54528688	0.00154636
ENSG00000165030	*NFIL3*	7.816060722	0.055898231	-0.03946434	0.095362571	0.999933887	0.794924028	1.233732985	-0.438808957	0.001546463
ENSG00000069956	*MAPK6*	7.628388574	-0.062497878	-0.127723171	0.065225294	0.999933887	1.132372757	1.65203032	-0.519657563	0.001546463
ENSG00000119638	*NEK9*	4.793638018	-0.03039132	-0.024971584	-0.005419736	0.999933887	-0.203087138	-0.47305837	0.269971232	0.001546463
ENSG00000140990	*NDUFB10*	3.527518084	0.049236136	0.01203903	0.037197106	0.999933887	-0.131783698	-0.684326066	0.552542367	0.001546463
ENSG00000156587	*UBE2L6*	7.024718755	0.039792329	-0.011170747	0.050963076	0.999933887	-0.423122335	-0.846973328	0.423850993	0.001546463
ENSG00000120437	*ACAT2*	4.236828779	-0.037995823	-0.032032712	-0.005963112	0.999933887	1.672567215	2.152756971	-0.480189756	0.001546463
ENSG00000196588	*MRTFA*	5.977646559	-0.041794038	0.05183244	-0.093626477	0.999933887	-0.230960892	-0.624934249	0.393973358	0.001570613
ENSG00000040933	*INPP4A*	5.824525316	0.016065675	0.006324758	0.009740917	0.999933887	-0.323715569	-0.72077693	0.397061361	0.001570613
ENSG00000104154	*SLC30A4*	2.419907804	-0.180495187	-0.159962524	-0.020532663	0.999933887	2.055480905	2.960444347	-0.904963443	0.00160586
ENSG00000067334	*DNTTIP2*	7.334978894	-0.06754697	-0.188661127	0.121114158	0.999933887	0.428847143	0.965127258	-0.536280115	0.001615311
ENSG00000119979	*DENND10*	4.309155494	0.061249476	-0.028646492	0.089895968	0.999933887	-0.359094367	-0.742223497	0.38312913	0.001635524
ENSG00000155099	*PIP4P2*	3.862429569	0.005404049	0.128901532	-0.123497483	0.999933887	-0.991107923	-1.560253105	0.569145182	0.001640256
ENSG00000198839	*ZNF277*	4.004704648	-0.013454063	-0.1308227	0.117368637	0.999933887	0.171511473	0.612101459	-0.440589986	0.001649672
ENSG00000279024	*AC112255.1*	2.743371378	-0.127262741	-0.124491866	-0.002770875	0.999933887	0.209670404	0.713410165	-0.503739762	0.001651684
ENSG00000078081	*LAMP3*	2.401064945	-0.004454341	0.107302014	-0.111756354	0.999933887	1.57349036	2.452852864	-0.879362504	0.001652104
ENSG00000167508	*MVD*	4.498744565	0.054299967	0.109066419	-0.054766452	0.999933887	1.592507882	2.415950217	-0.823442335	0.00167496
ENSG00000140995	*DEF8*	6.36772318	-0.002342184	0.054379808	-0.056721992	0.999933887	-0.173883061	-0.694981503	0.521098442	0.001675025
ENSG00000274536	*MIR223HG*	4.723661018	-0.117188132	-0.057324685	-0.059863447	0.999933887	-0.251007326	-0.771001632	0.519994305	0.001676007
ENSG00000169598	*DFFB*	1.503030199	-0.042271011	0.012382708	-0.054653719	0.999933887	-0.052257893	-0.68361393	0.631356037	0.001682235
ENSG00000163516	*ANKZF1*	4.564474014	0.016028951	0.056994774	-0.040965823	0.999933887	-0.123431033	-0.478604096	0.355173063	0.001682235
ENSG00000172590	*MRPL52*	2.630810029	-0.098945318	-0.145437301	0.046491983	0.999933887	1.187636568	1.873629861	-0.685993293	0.001682453
ENSG00000161542	*PRPSAP1*	3.282556893	0.056994165	-0.216489195	0.273483359	0.999933887	1.197885226	1.804721453	-0.606836228	0.001682453
ENSG00000236939	*BAALC-AS2*	-1.645688777	-0.032896537	-0.411022322	0.378125785	0.999933887	1.576544307	2.401167077	-0.82462277	0.001682453
ENSG00000109787	*KLF3*	6.818771052	-0.032585855	-0.019248699	-0.013337156	0.999933887	-1.122306337	-1.564926625	0.442620288	0.00168609
ENSG00000172531	*PPP1CA*	6.283589868	0.001727259	-0.018459099	0.020186358	0.999933887	-0.619176009	-1.059594449	0.44041844	0.00168609
ENSG00000213445	*SIPA1*	8.006244098	0.058689015	0.078346631	-0.019657616	0.999933887	-0.344905388	-0.721700001	0.376794613	0.001693511
ENSG00000213889	*PPM1N*	1.437522558	-0.074964866	0.096032198	-0.170997064	0.999933887	0.525201017	1.232943334	-0.707742318	0.001698453
ENSG00000137413	*TAF8*	3.562354095	-0.01151045	-0.038874328	0.027363878	0.999933887	-0.565437652	-1.049828056	0.484390404	0.001734874
ENSG00000068831	*RASGRP2*	7.032998288	0.053206782	0.051136045	0.002070737	0.999933887	-0.031169255	-0.428350816	0.397181561	0.001742917
ENSG00000127922	*SEM1*	3.33066589	0.001532115	-0.061587509	0.063119624	0.999933887	-0.034290185	0.337048787	-0.371338973	0.001742917
ENSG00000137497	*NUMA1*	6.810838926	0.110860981	0.063729587	0.047131394	0.999933887	0.573070869	0.92102668	-0.347955811	0.001744885
ENSG00000155304	*HSPA13*	4.779867695	-0.020014688	-0.155868825	0.135854137	0.999933887	0.316748662	0.812179621	-0.495430959	0.001744885
ENSG00000129071	*MBD4*	4.974586653	-0.095242732	-0.140746988	0.045504256	0.999933887	-0.842956781	-1.234858448	0.391901667	0.001745406
ENSG00000220008	*LINGO3*	3.976532118	-0.030148705	-0.072090778	0.041942073	0.999933887	-0.778475064	-1.582952969	0.804477905	0.001754444
ENSG00000237522	*NONOP2*	1.334090777	-0.125249046	-0.045135825	-0.080113221	0.999933887	0.635544109	1.648867575	-1.013323466	0.001769364
ENSG00000115275	*MOGS*	4.361345817	0.016988322	0.066874978	-0.049886655	0.999933887	-0.045448379	-0.349613572	0.304165193	0.001770791
ENSG00000183091	*NEB*	1.243723659	-0.143458762	-0.160537684	0.017078922	0.999933887	0.218144842	1.139046996	-0.920902154	0.001815722
ENSG00000115825	*PRKD3*	3.82092395	-0.086586725	-0.097296291	0.010709566	0.999933887	0.219408738	0.848403476	-0.628994738	0.001815722
ENSG00000114450	*GNB4*	3.349327964	-0.094815655	0.050125658	-0.144941313	0.999933887	-1.364311479	-2.068364665	0.704053187	0.00181681
ENSG00000287626	*AL021978.1*	4.553455939	-0.090241026	-0.050632908	-0.039608118	0.999933887	0.387408228	0.748345576	-0.360937348	0.001818128
ENSG00000130309	*COLGALT1*	4.986161427	0.040774316	-0.020313227	0.061087542	0.999933887	-0.103504597	-0.426274886	0.322770289	0.001845295
ENSG00000139178	*C1RL*	4.969060745	-0.021411455	-0.000540953	-0.020870501	0.999933887	-0.244497221	-0.575430012	0.330932792	0.001897128
ENSG00000213390	*ARHGAP19*	3.207387212	0.027416621	-0.017191398	0.044608018	0.999933887	-0.52487679	-1.072028802	0.547152013	0.001924838
ENSG00000170260	*ZNF212*	2.518703359	-0.096102545	0.015412133	-0.111514678	0.999933887	-0.436481145	-0.977777484	0.54129634	0.001928964
ENSG00000235531	*MSC-AS1*	1.188589313	-0.180192987	-0.249021129	0.068828142	0.999933887	0.882304661	1.588051113	-0.705746452	0.001954053
ENSG00000122299	*ZC3H7A*	5.562581208	-0.062432777	-0.070877402	0.008444625	0.999933887	0.266084778	0.638643423	-0.372558646	0.001967748
ENSG00000136167	*LCP1*	11.817379217	-0.010931158	-0.073358519	0.062427361	0.999933887	0.583538933	0.889995011	-0.306456078	0.001972101
ENSG00000031823	*RANBP3*	4.894394209	-0.000882473	0.048524833	-0.049407305	0.999933887	-0.236444128	-0.591476413	0.355032285	0.001978477
ENSG00000113615	*SEC24A*	5.567674198	-0.015896149	-0.074655685	0.058759536	0.999933887	1.355859991	1.929300502	-0.573440511	0.001983796
ENSG00000106012	*IQCE*	4.196000849	0.025557008	0.054303213	-0.028746205	0.999933887	-0.594492432	-1.092800004	0.498307572	0.002009638
ENSG00000131149	*GSE1*	5.676094554	0.019070902	0.035535445	-0.016464543	0.999933887	0.155162625	0.453772912	-0.298610287	0.00203286
ENSG00000076604	*TRAF4*	3.898016263	0.023472077	0.118726649	-0.095254573	0.999933887	0.97498407	1.706579944	-0.731595874	0.002036845
ENSG00000161981	*SNRNP25*	2.454530324	-0.010734354	-0.056464322	0.045729968	0.999933887	-1.598158662	-2.357919646	0.759760984	0.002039838
ENSG00000186591	*UBE2H*	7.384523721	0.0198901	0.004662352	0.015227748	0.999933887	0.760144646	1.116039636	-0.355894991	0.002040707
ENSG00000060138	*YBX3*	6.474325988	0.042843029	0.011879399	0.03096363	0.999933887	0.175458859	0.464249087	-0.288790228	0.002045257
ENSG00000139926	*FRMD6*	-0.173424563	-0.160899015	-0.063105811	-0.097793205	0.999933887	0.955739611	2.344841447	-1.389101836	0.002051251
ENSG00000164663	*USP49*	4.170606807	-0.116472479	-0.053365587	-0.063106892	0.999933887	0.854068353	1.286387371	-0.432319018	0.002069859
ENSG00000187260	*WDR86*	0.741192358	0.119564893	0.189999136	-0.069434243	0.999933887	-0.012949353	-0.762074215	0.749124862	0.002069859
ENSG00000119048	*UBE2B*	8.280011437	-0.008320474	0.03861762	-0.046938094	0.999933887	0.291688005	0.544771809	-0.253083805	0.002069859
ENSG00000198719	*DLL1*	1.955332716	0.051584403	0.41679618	-0.365211777	0.999933887	0.972495429	1.47933969	-0.506844261	0.002074453
ENSG00000198018	*ENTPD7*	2.931317446	-0.031513051	-0.009228969	-0.022284082	0.999933887	-0.085248294	0.518062498	-0.603310792	0.002086623
ENSG00000123358	*NR4A1*	6.017573788	0.113020453	0.159754566	-0.046734113	0.999933887	0.921034983	1.517281211	-0.596246228	0.002093108
ENSG00000204231	*RXRB*	5.032917834	0.011054953	0.035551703	-0.024496749	0.999933887	-0.395799134	-0.744731811	0.348932677	0.002093108
ENSG00000105642	*KCNN1*	-0.838271118	-0.555806841	-1.045159858	0.489353017	0.999933887	2.491481303	3.57200263	-1.080521326	0.002132646
ENSG00000174485	*DENND4A*	6.184723224	-0.178570857	-0.135404243	-0.043166614	0.999933887	2.455678968	3.509553571	-1.053874603	0.002132646
ENSG00000177674	*AGTRAP*	6.729311301	0.031345516	-0.019898506	0.051244022	0.999933887	1.130373771	1.565700437	-0.435326666	0.002142636
ENSG00000054983	*GALC*	6.727970803	-0.00468086	0.00064332	-0.005324179	0.999933887	1.070646535	1.502392566	-0.431746031	0.002142636
ENSG00000158669	*GPAT4*	5.417057677	-0.002828899	0.008255858	-0.011084756	0.999933887	0.636646093	0.98759597	-0.350949877	0.002142636
ENSG00000265975	*AC002091.1*	4.313103649	0.059894745	0.036353533	0.023541212	0.999933887	1.165251613	1.567638308	-0.402386695	0.002148805
ENSG00000224660	*SH3BP5-AS1*	6.557635944	0.00262507	0.014991054	-0.012365984	0.999933887	0.735195924	1.151534479	-0.416338555	0.002175136
ENSG00000175857	*GAPT*	5.657648423	-0.062486687	-0.187784735	0.125298048	0.999933887	0.650090363	1.223817364	-0.573727001	0.00217758
ENSG00000104412	*EMC2*	3.666936231	-0.110638244	-0.099091639	-0.011546605	0.999933887	0.422805377	0.951856687	-0.52905131	0.002197043
ENSG00000147202	*DIAPH2*	2.483957723	-0.105999874	0.093352573	-0.199352447	0.999933887	0.359764581	0.93787448	-0.578109899	0.002221417
ENSG00000119471	*HSDL2*	4.054889922	-0.031345789	-0.08494699	0.053601202	0.999933887	-0.476417327	-1.060097106	0.58367978	0.002266275
ENSG00000141971	*MVB12A*	2.455715945	0.058243705	-0.153097263	0.211340968	0.999933887	-0.166544809	-0.769240219	0.60269541	0.002297268
ENSG00000104695	*PPP2CB*	3.475097998	0.038940457	-0.077957879	0.116898336	0.999933887	0.286037128	0.635422303	-0.349385176	0.002302011
ENSG00000132507	*EIF5A*	7.050762932	0.047168854	0.034667849	0.012501004	0.999933887	1.209421965	1.642158968	-0.432737004	0.002310861

ENSG00000108100	*CCNY*	6.394786948	-0.023028271	-0.012109427	-0.010918843	0.999933887	-0.733654533	-1.128856564	0.395202031	0.002313026
ENSG00000166548	*TK2*	2.963674087	0.006032504	0.082158369	-0.076125865	0.999933887	-0.575855302	-1.266070035	0.690214733	0.002317187
ENSG00000279833	*AL031846.2*	2.427553033	-0.057611873	-0.143792497	0.086180624	0.999933887	-0.715010818	-1.379967683	0.664956865	0.002320205
ENSG00000135926	*TMBIM1*	7.588919248	-0.012112756	-0.005020194	-0.007092562	0.999933887	0.493458359	0.857228672	-0.363770313	0.002329934
ENSG00000066583	*ISOC1*	2.769033518	-0.138152847	0.083158771	-0.221311618	0.999933887	-0.237037245	-0.83263441	0.595597165	0.002357699
ENSG00000123374	*CDK2*	2.639281403	-0.097077101	0.069074622	-0.166151723	0.999933887	0.50529125	1.098018678	-0.592727429	0.002383735
ENSG00000266094	*RASSF5*	9.961578567	0.022709938	0.006430665	0.016279274	0.999933887	0.442366128	0.71643687	-0.274070742	0.002383735
ENSG00000160211	*G6PD*	6.814452304	0.011346899	0.005126583	0.006220316	0.999933887	-0.667288606	-1.087587773	0.420299167	0.002392071
ENSG00000188986	*NELFB*	4.148545652	0.068294079	0.100060453	-0.031766374	0.999933887	-0.276523665	-0.73414223	0.457618565	0.002394411
ENSG00000125733	*TRIP10*	2.551926679	0.071028259	-0.082433836	0.153462095	0.999933887	2.354667286	3.163407895	-0.808740609	0.002394411
ENSG00000092421	*SEMA6A*	0.600104887	-0.047206097	0.378426768	-0.425632865	0.999933887	0.63777397	1.636308074	-0.998534105	0.002394411
ENSG00000062194	*GPBP1*	6.751214588	-0.080300895	-0.121577691	0.041276796	0.999933887	0.551872992	0.982767261	-0.430894269	0.002397925
ENSG00000008513	*ST3GAL1*	7.796866626	0.010942557	0.000621332	0.010321225	0.999933887	0.916748062	1.329552178	-0.412804116	0.002403977
ENSG00000261971	*MMP25-AS1*	5.511641175	-0.028590294	-0.032033542	0.003443248	0.999933887	-0.92362772	-1.560659627	0.637031907	0.002418034
ENSG00000133048	*CHI3L1*	7.298293087	0.07447316	0.012835717	0.061637443	0.999933887	1.787883781	2.443479662	-0.65559588	0.002444117
ENSG00000115604	*IL18R1*	3.944306597	-0.062059126	-0.119712524	0.057653398	0.999933887	0.055128983	0.490487167	-0.435358184	0.002447732
ENSG00000115840	*SLC25A12*	1.890968647	-0.097724548	-0.068900312	-0.028824236	0.999933887	0.817886687	1.544617615	-0.726730928	0.002447732
ENSG00000135476	*ESPL1*	2.381463282	-0.036280769	-0.054239259	0.01795849	0.999933887	2.333748068	3.165769852	-0.832021784	0.002447732
ENSG00000095794	*CREM*	5.925942737	0.029096978	-0.067529032	0.09662601	0.999933887	0.438736382	0.906444263	-0.467707881	0.002449348
ENSG00000118260	*CREB1*	6.037625303	-0.019437578	-0.080532162	0.061094584	0.999933887	0.251395108	0.682392041	-0.430996934	0.002457077
ENSG00000232713	*RPS12P3*	0.299621248	-0.137212722	-0.180441551	0.043228828	0.999933887	0.612072476	1.671274223	-1.059201747	0.002494288
ENSG00000165097	*KDM1B*	5.730215038	-0.045918313	-0.01787136	-0.028046952	0.999933887	0.505307154	0.874465178	-0.369158024	0.002494288
ENSG00000137364	*TPMT*	2.959678014	0.04057513	-0.007217559	0.047792689	0.999933887	1.024746576	1.555289262	-0.530542686	0.002494288
ENSG00000166189	*HPS6*	2.755354464	0.043906145	0.077237947	-0.033331801	0.999933887	-0.464453677	-1.023353598	0.558899922	0.002545761
ENSG00000163964	*PIGX*	4.874886232	0.011288634	0.033529869	-0.022241235	0.999933887	-0.66474325	-1.223278077	0.558534828	0.002546999
ENSG00000178149	*DALRD3*	2.935961504	0.084829019	-0.058414468	0.143243487	0.999933887	-0.227892376	-0.660299131	0.432406754	0.002551886
ENSG00000092094	*OSGEP*	3.993841008	-0.114124466	0.057205296	-0.171329762	0.999933887	-0.347597496	-0.77540424	0.427806744	0.002564032
ENSG00000160209	*PDXK*	7.056255017	-0.033226711	-0.034768376	0.001541665	0.999933887	1.253242723	1.678335101	-0.425092378	0.002564032
ENSG00000160602	*NEK8*	0.975743057	0.040079949	-0.047853426	0.087933375	0.999933887	0.387880868	1.112954394	-0.725073526	0.002564032
ENSG00000206562	*METTL6*	2.106586494	0.029820063	-0.280162645	0.309982708	0.999933887	0.970812388	1.418153252	-0.447340864	0.002564032
ENSG00000124508	*BTN2A2*	5.712504717	0.037384723	0.026732331	0.010652392	0.999933887	2.008161977	2.479059055	-0.470897078	0.002580204
ENSG00000159840	*ZYX*	8.790142027	0.017056816	0.018616718	-0.001559902	0.999933887	-0.590649411	-0.940623749	0.349974338	0.002592451
ENSG00000139192	*TAPBPL*	4.596463977	0.043448015	-0.002827944	0.046275959	0.999933887	-0.312975242	-0.754779517	0.441804275	0.002606478
ENSG00000133313	*CNDP2*	4.608996209	0.022401523	-0.05800886	0.080410383	0.999933887	-0.253418521	-0.591840123	0.338421602	0.002617961
ENSG00000213918	*DNASE1*	3.599285017	0.013305263	0.056268572	-0.042963309	0.999933887	-0.191752147	-0.568086273	0.376334126	0.002632533
ENSG00000118564	*FBXL5*	8.633766787	-0.064202114	-0.123585657	0.059383543	0.999933887	-0.092244007	0.215494442	-0.307738449	0.002632813
ENSG00000146802	*TMEM168*	3.201490893	-0.133095364	-0.148639298	0.015543934	0.999933887	0.32454347	0.826422399	-0.501878928	0.002648581
ENSG00000056586	*RC3H2*	5.353354228	-0.005702929	-0.048992279	0.04328935	0.999933887	-0.259572243	0.137624426	-0.397196669	0.002672593
ENSG00000264364	*DYNLL2*	5.602166541	0.026052106	-0.00544764	0.031499746	0.999933887	-0.157730073	-0.497280085	0.339550012	0.00268074
ENSG00000155629	*PIK3AP1*	9.084389879	-0.028339643	-0.009231798	-0.019107845	0.999933887	2.063245188	2.617862778	-0.55461759	0.002684578
ENSG00000148331	*ASB6*	3.84151362	-0.016636622	-0.041763586	0.025126964	0.999933887	-0.156243358	-0.529227845	0.372984487	0.002684578
ENSG00000065613	*SLK*	6.710355186	-0.113524791	-0.142769087	0.029244296	0.999933887	0.91109654	1.704582594	-0.793486054	0.002698269
ENSG00000163932	*PRKCD*	9.270942921	-0.033901815	-0.017170908	-0.016730907	0.999933887	0.597591874	0.916654917	-0.319063043	0.002701117
ENSG00000166188	*ZNF319*	4.129823931	0.108138889	0.033630597	0.074508293	0.999933887	-0.623621009	-1.08359353	0.459972521	0.002712351
ENSG00000203880	*PCMTD2*	4.362727894	-0.056751708	-0.076231235	0.019479527	0.999933887	-0.283602314	-0.647479749	0.363877436	0.002712351
ENSG00000090376	*IRAK3*	7.08762894	-0.109851147	-0.088190348	-0.021660799	0.999933887	1.462172614	2.085982742	-0.623810128	0.002712351
ENSG00000137221	*TJAP1*	5.307584713	0.034370377	0.022032165	0.012338212	0.999933887	0.880159243	1.182856853	-0.302697609	0.002712351
ENSG00000185989	*RASA3*	6.326004302	0.02276308	-0.007824158	0.030587238	0.999933887	-0.193152516	-0.442968951	0.249816435	0.002712351
ENSG00000115738	*ID2*	7.060543458	0.011864998	0.093869521	-0.082004522	0.999933887	1.898915125	2.670421608	-0.771506483	0.002729525
ENSG00000112679	*DUSP22*	5.039557682	0.033037801	0.024506447	0.008531353	0.999933887	-0.290657429	-0.633342924	0.342785495	0.002746223
ENSG00000105483	*CARD8*	7.231027526	-0.032960024	-0.05330916	0.020349136	0.999933887	-1.027603823	-1.460434015	0.432830192	0.002746223
ENSG00000100591	*AHSA1*	3.724631672	-0.026611221	0.008534942	-0.035146163	0.999933887	-0.170148821	-0.520793799	0.350644978	0.002754402
ENSG00000173825	*TIGD3*	1.957627318	-0.050490603	-0.004024942	-0.046465661	0.999933887	-1.512773431	-2.567321881	1.05454845	0.002780513
ENSG00000133612	*AGAP3*	7.041055357	0.002562884	-0.010903673	0.013466557	0.999933887	0.657611621	1.000854662	-0.343243041	0.002780513
ENSG00000182446	*NPLOC4*	6.591044263	0.021428801	-0.024278539	0.045707341	0.999933887	0.686240662	1.041123049	-0.354882387	0.002785955
ENSG00000204396	*VWA7*	0.454746738	-0.413431864	-0.136013284	-0.27741858	0.999933887	-0.364773797	-1.273658951	0.908885154	0.002793785
ENSG00000101187	*SLCO4A1*	4.093211452	0.113688839	0.153083988	-0.039395149	0.999933887	0.978266326	1.489573685	-0.511307359	0.002793785
ENSG00000100024	*UPB1*	5.38810352	-0.033903264	-0.042398388	0.008495123	0.999933887	1.719014537	2.151491105	-0.432476568	0.002793785
ENSG00000215041	*NEURL4*	3.178217245	0.114275605	-0.017132127	0.131407732	0.999933887	0.044001523	-0.388557283	0.432558806	0.002806915
ENSG00000160593	*JAML*	8.583024809	-0.064255283	-0.044343219	-0.019912064	0.999933887	-0.285417048	-0.706663836	0.421246788	0.002806915
ENSG00000078269	*SYNJ2*	4.466846953	0.036701493	0.048406484	-0.011704991	0.999933887	1.715611824	2.370756344	-0.65514452	0.002806915
ENSG00000154822	*PLCL2*	5.178191039	-0.024172011	-0.039577334	0.015405323	0.999933887	0.204718951	0.697552665	-0.492833714	0.002886956
ENSG00000271856	*LINC01215*	4.957943114	-0.017005053	-0.024492749	0.007487695	0.999933887	1.040497298	1.495126879	-0.454629581	0.002886956
ENSG00000130402	*ACTN4*	7.554141173	0.054999819	0.009640658	0.045359161	0.999933887	-0.331311565	-0.677221251	0.345909686	0.002888658
ENSG00000118217	*ATF6*	5.161307879	0.020329401	-0.059750836	0.080080237	0.999933887	0.266660409	0.629129001	-0.362468593	0.002898289
ENSG00000072110	*ACTN1*	8.613777521	0.03723982	0.011204435	0.026035385	0.999933887	-0.646361342	-0.963597903	0.317236561	0.002906618
ENSG00000103769	*RAB11A*	5.851917115	-0.025978462	0.004799202	-0.030777663	0.999933887	0.338164488	0.666725785	-0.328561298	0.002906618
ENSG00000070081	*NUCB2*	3.000649652	-0.180351207	-0.212883365	0.032532158	0.999933887	-0.009387797	0.678264262	-0.687652058	0.00290939
ENSG00000154642	*C21orf91*	3.842250963	-0.074562692	-0.008271299	-0.066291393	0.999933887	0.397970982	1.03188628	-0.633915298	0.002940265
ENSG00000188186	*LAMTOR4*	6.434991117	-0.005298481	0.001762322	-0.007060802	0.999933887	-0.211772971	-0.600557219	0.388784248	0.002968487
ENSG00000135269	*TES*	5.089620949	-0.00682557	-0.038205	0.03137943	0.999933887	0.504052215	0.931774202	-0.427721987	0.002977149
ENSG00000102886	*GDPD3*	2.299829629	0.111139961	0.130704226	-0.019564265	0.999933887	-0.844810002	-1.470506	0.625695997	0.002977589
ENSG00000147408	*CSGALNACT1*	4.797858225	0.006213115	-0.015581149	0.021794264	0.999933887	0.401673126	0.836522243	-0.434849117	0.002987845
ENSG00000197467	*COL13A1*	0.763487812	-0.153074966	-0.012745611	-0.140329355	0.999933887	1.176948209	1.945655645	-0.768707437	0.002995387
ENSG00000121858	*TNFSF10*	4.619030921	-0.033122707	-0.113683054	0.080560347	0.999933887	-0.582761117	-1.243798343	0.661037227	0.003040071
ENSG00000178127	*NDUFV2*	1.709878155	-0.074690731	0.003440681	-0.078131412	0.999933887	0.634286374	1.366922359	-0.732635985	0.003059272
ENSG00000100077	*GRK3*	3.876261178	-0.037920486	0.090502284	-0.12842277	0.999933887	0.276887705	0.707388804	-0.430501099	0.003071059
ENSG00000141096	*DPEP3*	2.671454211	-0.007766466	-0.037632852	0.029866386	0.999933887	-1.353168404	-2.048699102	0.695530698	0.003080084
ENSG00000160948	*VPS28*	5.874456191	0.010584045	0.068292243	-0.057708198	0.999933887	-0.122094578	-0.485779459	0.36368488	0.003097567
ENSG00000275023	*MLLT6*	8.40973129	0.073291844	0.006825311	0.066466532	0.999933887	0.818478099	1.193393885	-0.374915786	0.00311743
ENSG00000138433	*CIR1*	8.27537217	-0.065367651	-0.165627261	0.10025961	0.999933887	0.397014631	0.774458044	-0.377443413	0.003165265
ENSG00000103642	*LACTB*	5.475542665	-0.0531549	0.008622308	-0.061777208	0.999933887	0.261726119	0.56072236	-0.298996241	0.003176267
ENSG00000096070	*BRPF3*	6.501035093	0.012494675	-0.021748329	0.034243004	0.999933887	0.751801616	1.093676909	-0.341875293	0.003192123
ENSG00000114796	*KLHL24*	7.170897649	-0.043724696	-0.113506495	0.0697818	0.999933887	-0.281756457	0.01450277	-0.296259227	0.003221368
ENSG00000197380	*DACT3*	-1.193473833	0.072252194	0.399155029	-0.326902835	0.999933887	1.3165085	2.905758237	-1.589249737	0.003221368
ENSG00000178252	*WDR6*	5.075886236	0.01572241	0.014607375	0.001115034	0.999933887	-0.063414644	-0.362846932	0.299432288	0.003232836
ENSG00000113163	*CERT1*	6.343021297	-0.017651804	-0.094503903	0.076852098	0.999933887	0.385857939	0.798932882	-0.413074943	0.0032334
ENSG00000165732	*DDX21*	6.978354673	-0.01303621	-0.120825888	0.107789678	0.999933887	0.656034543	1.168429553	-0.51239501	0.003248952
ENSG00000155329	*ZCCHC10*	2.957910642	-0.139469138	-0.009393906	-0.130075232	0.999933887	0.615212194	1.020348128	-0.405135934	0.003254615
ENSG00000143067	*ZNF697*	3.057995518	0.058195358	-0.08059986	0.138795218	0.999933887	0.792876165	1.3328386	-0.539962435	0.003254615
ENSG00000104365	*IKBKB*	6.754770093	-0.006032322	-0.034892098	0.028859776	0.999933887	0.639318151	0.951773271	-0.312455119	0.003254615
ENSG00000173801	*JUP*	2.716297688	-0.022078172	0.060050328	-0.0821285	0.999933887	0.073372832	0.58065293	-0.507280098	0.003260597
ENSG00000111144	*LTA4H*	5.674792975	-0.079206354	-0.093469441	0.014263087	0.999933887	-0.546439098	-0.976553341	0.430114242	0.003305158
ENSG00000080345	*RIF1*	4.845051105	-0.102258899	-0.093815265	-0.008443635	0.999933887	0.419699241	1.300946769	-0.881247528	0.003305158
ENSG00000132388	*UBE2G1*	4.957725699	-0.033190513	0.040701246	-0.073891759	0.999933887	-0.706390996	-1.109323293	0.402932297	0.003305158
ENSG00000139636	*LMBR1L*	5.604191905	-0.005821937	0.004587629	-0.010409566	0.999933887	-0.132107108	-0.391469493	0.259362385	0.003305158
ENSG00000162927	*PUS10*	1.923272167	-0.120393565	-0.215949266	0.095555701	0.999933887	0.511981395	1.446347168	-0.934365772	0.003324819
ENSG00000253167	*WASHC5-AS1*	0.363966404	0.196889281	0.000755321	0.19613396	0.999933887	-0.143662675	0.636176088	-0.779838763	0.003333652
ENSG00000068366	*ACSL4*	5.59638782	-0.099485818	-0.133516279	0.034030462	0.999933887	0.165783074	0.856668519	-0.690885445	0.003333652
ENSG00000114648	*KLHL18*	4.698582137	0.01182571	-0.002845558	0.014671268	0.999933887	-0.309832624	-0.679101443	0.369268819	0.003333652
ENSG00000168002	*POLR2G*	3.836999916	0.001551403	0.033751502	-0.032200099	0.999933887	-0.126766252	-0.514486662	0.38772041	0.003333652
ENSG00000114388	*NPRL2*	3.365776518	0.028387123	-0.028130939	0.056518062	0.999933887	-0.193973414	-0.589351858	0.395378444	0.003341921
ENSG00000135124	*P2RX4*	5.847521242	0.000287346	-0.053285032	0.053572378	0.999933887	1.055208232	1.482963237	-0.427755004	0.003341921
ENSG00000152291	*TGOLN2*	7.950654793	-0.034978369	-0.027632514	-0.007345854	0.999933887	-0.448969617	-0.700380856	0.251411239	0.003354118
ENSG00000122042	*UBL3*	6.168465714	-0.043256924	-0.056891521	0.013634598	0.999933887	0.112907707	0.423689139	-0.310781433	0.003354118

ENSG00000134352	*IL6ST*	5.132124922	-0.041119672	-0.212383357	0.171263685	0.999933887	0.191960766	0.959876934	-0.767916168	0.003354118
ENSG00000035403	*VCL*	5.590052861	0.008567832	-0.052107086	0.060674918	0.999933887	0.217201353	0.513499747	-0.296298394	0.003354118
ENSG00000253256	*AC134043.2*	-0.390743575	0.018032636	0.191306471	-0.173273835	0.999933887	0.572276871	1.409781174	-0.837504304	0.003354118
ENSG00000138600	*SPPL2A*	6.255438196	-0.041055486	-0.052152663	0.011097177	0.999933887	0.741317454	1.10603271	-0.364715256	0.00335678
ENSG00000168438	*CDC40*	3.741498982	-0.084566015	-0.013359504	-0.071206511	0.999933887	-0.61641209	-1.091365564	0.474953474	0.00339547
ENSG00000164142	*FAM160A1*	0.212239124	-0.01797775	0.084684194	-0.102661944	0.999933887	1.335667954	2.489065458	-1.153397504	0.003397039
ENSG00000176619	*LMNB2*	3.384593781	0.138846783	0.021949729	0.116897054	0.999933887	1.008870369	1.658325873	-0.649455504	0.003422121
ENSG00000088888	*MAVS*	4.804922006	-0.00439618	0.009784215	-0.014180396	0.999933887	-0.205763358	-0.581770556	0.376007199	0.003422121
ENSG00000138760	*SCARB2*	3.176147309	-0.010322383	-0.0063092	-0.004013183	0.999933887	-0.482817267	-0.982895718	0.500078452	0.003477086
ENSG00000144283	*PKP4*	3.580449543	-0.031581971	0.089436059	-0.12101803	0.999933887	-0.887849375	-1.371557804	0.483708429	0.003477624
ENSG00000033800	*PIAS1*	6.778675487	-0.029146011	-0.039512698	0.010366687	0.999933887	0.078456966	0.465827764	-0.387370798	0.00350511
ENSG00000142751	*GPN2*	3.604075023	-0.060285754	0.131968099	-0.192253853	0.944094537	-0.146047919	-0.453790895	0.307742977	0.003507203
ENSG00000115091	*ACTR3*	9.497681864	-0.016939671	-0.004535351	-0.01240432	0.999933887	0.744096652	1.016079013	-0.271982361	0.003507203
ENSG00000110756	*HPS5*	5.660160961	-0.015438068	-0.024960759	0.009522691	0.999933887	0.535529523	0.94244861	-0.406919087	0.003520319
ENSG00000286281	*AL353152.2*	1.380117748	-0.0526744	0.381520884	-0.434195283	0.999933887	0.525299865	1.118843439	-0.593543575	0.003527351
ENSG00000154310	*TNIK*	4.472668381	-0.02589935	-0.023064169	-0.002835181	0.999933887	0.01825107	0.515539388	-0.497288318	0.003530991
ENSG00000234616	*JRK*	3.054569016	-0.088559343	-0.001256303	-0.08730304	0.999933887	-0.188885187	-0.545397507	0.356512319	0.003548404
ENSG00000160446	*ZDHHC12*	3.830894813	0.095166956	-0.03379384	0.128960797	0.999933887	-0.467119713	-1.017273593	0.55015388	0.003548404
ENSG00000151726	*ACSL1*	10.068648973	-0.061427846	-0.051561798	-0.009866048	0.999933887	1.739207772	2.242398127	-0.503190356	0.003548404
ENSG00000186088	*GSAP*	5.36394477	-0.025236702	-0.003094197	-0.022142505	0.999933887	1.081895612	1.611017854	-0.529122243	0.003548404
ENSG00000126561	*STAT5A*	8.046770933	-0.028086008	0.004867381	-0.03295339	0.999933887	2.540926236	3.164184309	-0.623258073	0.00355842
ENSG00000184640	*SEPTIN9*	7.773937361	0.012953249	-0.018900762	0.031854011	0.999933887	-0.303133668	-0.665599737	0.362466069	0.003589577
ENSG00000252172	*RNU6-720P*	0.092766874	-0.406520971	0.488079678	-0.894600649	0.944094537	-0.484273349	0.189759258	-0.674032606	0.003601601
ENSG00000173465	*ZNRD2*	2.968275222	-0.021048251	-0.063113503	0.042065251	0.999933887	0.524675721	1.001415574	-0.476739853	0.003601601
ENSG00000121552	*CSTA*	4.267479694	0.002654431	0.0322774	-0.029622969	0.999933887	0.628914949	1.066158298	-0.437243349	0.003652967
ENSG00000258926	*AL355916.2*	2.935949204	0.030359452	-0.001884067	0.032243519	0.999933887	0.957629468	1.687258534	-0.729629066	0.003668712
ENSG00000112773	*TENT5A*	3.868766283	-0.065597842	-0.07544464	0.009846798	0.999933887	-0.620939851	-1.082991046	0.462051196	0.003675416
ENSG00000196663	*TECPR2*	8.165887867	-0.017928469	-0.018410393	0.000481923	0.999933887	0.779724339	1.081531458	-0.301807119	0.003675416
ENSG00000065413	*ANKRD44*	7.109570287	-0.032583703	-0.053911519	0.021327816	0.999933887	-0.477124701	-0.980648931	0.50352423	0.00369443
ENSG00000158773	*USF1*	6.062661124	-0.041874777	-0.002393976	-0.039480801	0.999933887	-0.422877948	-0.773367211	0.350489263	0.003745502
ENSG00000176597	*B3GNT5*	7.391567201	0.036345042	-0.031855029	0.068200071	0.999933887	0.261578409	0.72214835	-0.460569941	0.003745502
ENSG00000065526	*SPEN*	6.913763594	-0.033829708	-0.061404342	0.027574634	0.999933887	-0.860188755	-1.307759648	0.447570892	0.003748876
ENSG00000104093	*DMXL2*	7.115953014	-0.166292375	-0.144452313	-0.021840062	0.999933887	1.793565171	2.763992888	-0.970427717	0.003762073
ENSG00000163444	*TMEM183A*	4.730113702	0.000413074	0.007900736	-0.007487662	0.999933887	-0.248013912	-0.556390926	0.308377014	0.003762073
ENSG00000131899	*LLGL1*	3.125475589	0.030769752	-0.003366149	0.0341359	0.999933887	-0.019131111	-0.387669327	0.368538217	0.003765578
ENSG00000167264	*DUS2*	3.472864256	-0.134978211	-0.016552567	-0.118425643	0.999933887	-1.691043981	-2.380853979	0.689809998	0.003818742
ENSG00000142046	*TMEM91*	3.430651765	0.055121604	0.03969476	0.015426844	0.999933887	0.048527165	-0.456729106	0.505256271	0.003818742
ENSG00000154153	*RETREG1*	3.045126496	-0.096098633	-0.006397073	-0.08970156	0.999933887	0.524573059	1.080640489	-0.55606743	0.003839672
ENSG00000111371	*SLC38A1*	6.985376936	-0.066231091	-0.099041068	0.032809977	0.999933887	0.076549323	0.583517323	-0.506967999	0.003884438
ENSG00000163539	*CLASP2*	3.570597366	-0.066273293	-0.001564242	-0.064709051	0.999933887	0.146597484	0.680268622	-0.533671138	0.003888643
ENSG00000159556	*ISL2*	0.587985461	-0.176985224	0.290239474	-0.467224698	0.944094537	-0.986376159	-2.088896472	1.102520314	0.003906181
ENSG00000179163	*FUCA1*	3.328650257	-0.201737404	0.028779466	-0.230516869	0.999933887	-0.757927105	-1.25424809	0.496320984	0.003910623
ENSG00000136044	*APPL2*	5.0402989	-0.075619822	-0.039592297	-0.036027525	0.999933887	-0.568732858	-0.906048438	0.337315581	0.003910623
ENSG00000153048	*CARHSP1*	3.003723714	0.089445724	0.048796532	0.040649192	0.999933887	-0.139235879	-0.675678001	0.536442122	0.003910623
ENSG00000197948	*FCHSD1*	5.986270261	0.028791371	0.107656866	-0.078865495	0.999933887	-0.317715367	-0.66094634	0.343230973	0.003910623
ENSG00000100612	*DHRS7*	6.150034897	-0.030013777	-0.094386588	0.064372811	0.999933887	-1.024403615	-1.458835851	0.434432237	0.003910623
ENSG00000142327	*RNPEPL1*	6.492476826	-0.015655563	0.009361198	-0.025016761	0.999933887	-0.475347543	-0.826474284	0.351126741	0.003910623
ENSG00000067992	*PDK3*	5.215305824	-0.091912754	-0.09192784	1.5085e-05	0.999933887	-1.322713757	-1.808945742	0.486231985	0.003917996
ENSG00000171368	*TPPP*	2.237900123	-0.101150927	-0.022793786	-0.078357141	0.999933887	1.036882267	2.148356758	-1.111474491	0.003927935
ENSG00000255823	*MTRNR2L8*	1.76121405	-0.19908938	-0.122806857	-0.076282523	0.999933887	2.036025584	2.829976688	-0.793951103	0.003940125
ENSG00000132589	*FLOT2*	7.777917681	0.023389402	-0.046249787	0.06963919	0.999933887	0.419295446	0.7762401	-0.356944655	0.003966922
ENSG00000104450	*SPAG1*	2.417431086	-0.00974323	0.027593931	-0.037337161	0.999933887	0.574549554	1.233261211	-0.658711656	0.003966922
ENSG00000268581	*SIGLEC18P*	-1.192952733	0.135592842	-0.082993914	0.218586756	0.999933887	1.225787282	2.091602133	-0.86581485	0.003990503
ENSG00000115325	*DOK1*	4.17051304	0.015960695	0.008617542	0.007343153	0.999933887	-0.68774336	-1.170507141	0.482763781	0.003999376
ENSG00000166670	*MMP10*	0.244724504	0.244365067	0.025311803	0.219053264	0.999933887	1.086922871	2.180863596	-1.093940725	0.004012324
ENSG00000188483	*IER5L*	2.012554191	-0.226874126	-0.133483449	-0.093390678	0.999933887	-1.463953544	-2.238153769	0.774200225	0.004030639
ENSG00000159055	*MIS18A*	0.059401798	0.076541772	0.07703551	-0.000493738	0.999933887	1.051614328	2.093900411	-1.042286083	0.004030639
ENSG00000163625	*WDFY3*	7.528504268	-0.034786371	-0.0603605	0.025574129	0.999933887	0.566372105	0.963652467	-0.397280362	0.004054681
ENSG00000188921	*HACD4*	4.641438929	0.079396831	-0.048243659	0.12764049	0.999933887	-0.264116932	-0.760208373	0.49609144	0.004056109
ENSG00000139146	*SINHCAF*	3.611462925	-0.067886744	0.016128765	-0.084015508	0.999933887	0.132495295	0.477273128	-0.344777833	0.00406207
ENSG00000137486	*ARRB1*	5.343553556	-0.014590191	-0.00429512	-0.010295071	0.999933887	-0.866264838	-1.336572911	0.470308073	0.00406207
ENSG00000114268	*PFKFB4*	4.440363198	-0.042253388	0.038297665	-0.080551053	0.999933887	-0.328299458	-0.961046495	0.632747037	0.004077578
ENSG00000131100	*ATP6V1E1*	7.064348553	-0.033403212	-0.051873715	0.018470503	0.999933887	0.157149539	0.42973883	-0.272589291	0.004088839
ENSG00000198189	*HSD17B11*	6.348771611	-0.06404765	-0.079305914	0.015258263	0.999933887	-0.481993316	-0.935804481	0.453811166	0.004091068
ENSG00000115234	*SNX17*	5.046035121	0.060397094	0.031490657	0.028906437	0.999933887	-0.166106617	-0.498674125	0.332567508	0.004091108
ENSG00000142765	*SYTL1*	5.983392265	0.043349882	0.060517577	-0.017167694	0.999933887	-0.009747115	-0.338940648	0.329193533	0.004102848
ENSG00000167703	*SLC43A2*	9.386692836	0.030732194	0.007083172	0.023649022	0.999933887	0.392319238	0.668043361	-0.275724123	0.00413189
ENSG00000092964	*DPYSL2*	1.516399616	-0.081106434	-0.05257484	-0.028531594	0.999933887	-0.199799268	-0.81111552	0.611316251	0.004137556
ENSG00000197208	*SLC22A4*	4.683140869	0.062518543	0.0028196	0.059698942	0.999933887	-0.517467547	-0.891277953	0.373810406	0.004141236
ENSG00000110660	*SLC35F2*	1.950890288	0.153761983	-0.087485099	0.241247082	0.999933887	1.64940502	2.24336956	-0.59396454	0.004141236
ENSG00000160213	*CSTB*	7.738179036	0.114107384	0.051101105	0.063006278	0.999933887	0.955346839	1.325025608	-0.369678769	0.004174658
ENSG00000108510	*MED13*	7.115606893	-0.03125877	-0.078293178	0.047034408	0.999933887	0.076062433	0.524697804	-0.448635371	0.004179127
ENSG00000265808	*SEC22B*	7.664608061	-0.041581753	-0.096160434	0.05457868	0.999933887	0.579136989	0.886647462	-0.307510473	0.004196175
ENSG00000263956	*NBPF11*	2.608090504	-0.028086504	-0.109324031	0.081237527	0.999933887	0.174767289	0.546358298	-0.371591009	0.004196175
ENSG00000064012	*CASP8*	7.594893196	-0.097866557	-0.018276108	-0.07959045	0.999933887	-0.11313839	-0.37227454	0.25913615	0.004248238
ENSG00000166949	*SMAD3*	5.458869924	-0.042584509	-0.005281068	-0.037303441	0.999933887	0.320188827	0.693702865	-0.373514038	0.004250116
ENSG00000169504	*CLIC4*	4.510078149	-0.055897519	-0.161506464	0.105608945	0.999933887	3.977491053	4.861878502	-0.884387449	0.004250116
ENSG00000124813	*RUNX2*	4.055972266	0.005427641	0.045927809	-0.040500168	0.999933887	-0.468720616	-1.018654057	0.549933441	0.004250116
ENSG00000126003	*PLAGL2*	6.139464567	-0.093536277	-0.063655651	-0.029880626	0.999933887	1.192170031	1.618603005	-0.426432974	0.00427278
ENSG00000137496	*IL18BP*	6.635925811	0.085186088	0.107141144	-0.021955056	0.999933887	0.96717305	1.288500838	-0.321327788	0.004297179
ENSG00000099785	*MARCHF2*	4.2917905	0.008016814	-0.001471297	0.009488111	0.999933887	-0.546377095	-0.963721227	0.417344132	0.004297179
ENSG00000164880	*INTS1*	5.872242565	0.05663493	0.080148389	-0.023513459	0.999933887	-0.159006709	-0.483690572	0.324683863	0.004304393
ENSG00000140044	*JDP2*	4.48773761	0.026874471	0.056237175	-0.029362703	0.999933887	-0.980221363	-1.601571592	0.621350229	0.004304393
ENSG00000116752	*BCAS2*	4.77376395	-0.021440885	-0.108810876	0.087369991	0.999933887	-0.048579552	0.349243503	-0.397823055	0.004310283
ENSG00000144843	*ADPRH*	1.892505495	-0.141071941	0.00040303	-0.141474972	0.999933887	0.669709639	1.196083552	-0.526373914	0.004353579
ENSG00000163399	*ATP1A1*	7.030357997	-0.009724812	-0.00335873	-0.006366081	0.999933887	0.379876411	0.679011034	-0.299134623	0.004353579
ENSG00000119508	*NR4A3*	7.634489989	0.107843938	0.051490008	0.056353931	0.999933887	1.347395886	2.0845948	-0.737198914	0.004370285
ENSG00000120889	*TNFRSF10B*	6.237771822	-0.016734895	0.052416505	-0.0691514	0.999933887	0.058645806	-0.314190666	0.372836472	0.004374451
ENSG00000016864	*GLT8D1*	2.388476932	-0.093258683	-0.156709066	0.063450383	0.999933887	-0.152731674	-0.525334853	0.372603179	0.004375551
ENSG00000103150	*MLYCD*	2.481426595	0.033698574	0.08310556	-0.049406986	0.999933887	-0.150284989	-0.62660356	0.476318571	0.004375551
ENSG00000152684	*PELO*	4.171883559	-0.213959925	-0.242257023	0.028297098	0.999933887	-1.052612467	-1.585578293	0.532965826	0.004390737
ENSG00000164062	*APEH*	4.137031787	0.053189145	-0.103094772	0.156283917	0.999933887	-0.089372063	-0.416364729	0.326992666	0.004460469
ENSG00000100296	*THOC5*	4.557633942	0.024873011	-0.011795149	0.03666816	0.999933887	-0.10737941	-0.553705598	0.446326188	0.004460469
ENSG00000120438	*TCP1*	5.868573604	-0.05577555	-0.076042602	0.020267052	0.999933887	0.559316754	0.805410749	-0.246093995	0.004472924
ENSG00000196151	*WDSUB1*	1.948942152	0.002467891	-0.163075699	0.16554359	0.999933887	-0.012151442	0.467285015	-0.479436457	0.004486055
ENSG00000261371	*PECAM1*	7.336203771	-0.009643566	-0.093243474	0.083599908	0.999933887	-0.619472616	-1.261653589	0.642180973	0.004488162
ENSG00000138279	*ANXA7*	6.43172484	-0.033341708	-0.048094076	0.014752368	0.999933887	0.683394696	0.977500258	-0.294105562	0.004490805
ENSG00000164068	*RNF123*	4.020266248	0.040683789	0.094729911	-0.054046122	0.999933887	-0.190072408	-0.52365785	0.333585442	0.004494147
ENSG00000111110	*PPM1H*	0.897314112	-0.070395096	0.130588205	-0.200983301	0.999933887	-1.117987119	-1.932987576	0.815000457	0.00450868
ENSG00000109685	*NSD2*	4.207080316	-0.016022124	-0.069629645	0.053607521	0.999933887	-0.335927815	-0.714886254	0.378958439	0.004546145
ENSG00000183020	*AP2A2*	4.30327739	0.022641418	0.019349055	0.003292363	0.999933887	-0.221987335	-0.550747118	0.328759783	0.004574549
ENSG00000183597	*TANGO2*	4.441327121	0.017579204	-0.08774071	0.105319914	0.999933887	-0.568725213	-0.88807401	0.319348797	0.004634346
ENSG00000110713	*NUP98*	8.422234241	0.005848158	0.002818203	0.003029955	0.999933887	0.527991686	0.799590386	-0.2715987	0.004639536
ENSG00000102178	*UBL4A*	2.731043992	-0.017341608	-0.035717436	0.018375827	0.999933887	-0.486665219	-1.043201482	0.556536263	0.004659789
ENSG00000118495	*PLAGL1*	5.401622104	-0.016320392	-0.086380684	0.070060292	0.999933887	0.25294981	0.674063877	-0.421114066	0.004673418

ENSG00000103489	*XYLT1*	5.062924553	-0.026349581	0.003066153	-0.029415734	0.999933887	-0.344490256	-0.752094056	0.4076038	0.004722737
ENSG00000187609	*EXD3*	2.602745193	-0.002493101	0.063450139	-0.06594324	0.999933887	0.300914588	0.732263144	-0.431348556	0.004733331
ENSG00000101040	*ZMYND8*	3.945362033	-0.022805364	-0.047364055	0.024558692	0.999933887	-0.333446741	-0.739928545	0.406481804	0.004746279
ENSG00000100297	*MCM5*	3.852564574	-0.008402128	0.03451027	-0.042912399	0.999933887	-0.132403369	-0.476554511	0.344151142	0.004746279
ENSG00000135604	*STX11*	8.553671189	0.089947586	0.0614051	0.028542487	0.999933887	1.000738584	1.294368115	-0.293629531	0.004758562
ENSG00000014216	*CAPN1*	5.698581057	0.050832359	-0.008112252	0.058944611	0.999933887	-0.231761412	-0.592428705	0.360667293	0.004775836
ENSG00000196814	*MVB12B*	2.794815731	0.013962397	0.035165084	-0.021202688	0.999933887	0.549326375	1.153857527	-0.604531152	0.004775836
ENSG00000141569	*TRIM65*	2.189479855	0.099983792	0.001262756	0.098721036	0.999933887	0.13433308	-0.315475577	0.449808657	0.004791884
ENSG00000124102	*PI3*	9.348772601	-0.043228343	0.037985421	-0.081213764	0.999933887	2.287084983	2.981097968	-0.694012985	0.004824025
ENSG00000185475	*TMEM179B*	2.298995691	0.064466574	-0.018624372	0.083090946	0.999933887	-0.364158307	-1.040802671	0.676644363	0.004858186
ENSG00000004660	*CAMKK1*	4.438083525	-0.052362933	-0.022991985	-0.029370948	0.999933887	-1.459370694	-1.95858571	0.499215015	0.004910284
ENSG00000175634	*RPS6KB2*	4.327590061	0.038621863	0.044990272	-0.006368409	0.999933887	-0.282808719	-0.698659893	0.415851173	0.004910284
ENSG00000196776	*CD47*	6.739019085	-0.030342216	-0.030120892	-0.000221324	0.999933887	-0.122540245	0.229744233	-0.352284478	0.004945664
ENSG00000074800	*ENO1*	7.584134227	-0.020785647	-0.052975652	0.032190005	0.999933887	-0.300560977	-0.608818529	0.308257553	0.004946575
ENSG00000104903	*LYL1*	3.990935002	0.019354455	-0.05267793	0.072032385	0.999933887	-1.575269475	-2.38737183	0.812102355	0.004957942
ENSG00000113638	*TTC33*	3.042753618	-0.039524406	0.030252124	-0.06977653	0.999933887	0.376347768	0.902533259	-0.526185491	0.00495894
ENSG00000087087	*SRRT*	6.263304862	0.020761633	0.042152569	-0.021390935	0.999933887	-0.097689933	-0.34503947	0.247349537	0.004988891
ENSG00000213380	*COG8*	0.64598216	-0.08124276	-0.027090226	-0.054152535	0.999933887	0.069711273	-0.607446735	0.677158008	0.00498895
ENSG00000089916	*GPATCH2L*	6.783258397	-0.035667778	-0.05151284	0.015845062	0.999933887	0.342370139	0.623886087	-0.281515948	0.00501099
ENSG00000205220	*PSMB10*	4.469337967	0.043623405	0.035179032	0.008444372	0.999933887	-0.657672371	-1.044847027	0.387174656	0.005021317
ENSG00000250565	*ATP6V1E2*	0.846054354	0.204333262	-0.13460923	0.338942492	0.999933887	1.108724505	1.881508856	-0.77278435	0.00502718
ENSG00000223949	*ROR1-AS1*	-1.203378618	0.398380387	0.206633096	0.191747291	0.999933887	1.618015552	2.446288972	-0.828273421	0.005034651
ENSG00000213937	*CLDN9*	1.360828733	-0.012605435	0.134656573	-0.147262009	0.999933887	-1.344044502	-2.275761333	0.931716832	0.005055411
ENSG00000101337	*TM9SF4*	6.256751411	0.00238706	-0.047819395	0.050206455	0.999933887	0.630395769	0.908810459	-0.27841469	0.00508912
ENSG00000111596	*CNOT2*	5.858534382	-0.051334684	-0.044537199	-0.006797485	0.999933887	0.177032163	0.539233136	-0.362200973	0.005090606
ENSG00000171853	*TRAPPC12*	4.462001277	0.051588132	0.075693626	-0.024105494	0.999933887	-0.285771865	-0.670155245	0.38438338	0.005134543
ENSG00000110057	*UNC93B1*	6.421020481	0.046436902	0.025301735	0.021135167	0.999933887	-1.065129749	-1.480489186	0.415359437	0.00514807
ENSG00000166920	*C15orf48*	6.50602043	0.278275277	0.396552714	-0.118277438	0.999933887	2.314660568	2.970865014	-0.656204446	0.005152533
ENSG00000139718	*SETD1B*	6.480721792	-0.030280919	0.097577012	-0.127857931	0.999933887	-0.776909253	-1.182227947	0.405318694	0.005200615
ENSG00000111786	*SRSF9*	5.180880374	0.013910436	0.028053443	-0.014143007	0.999933887	-0.272762627	-0.604899107	0.33213648	0.005200615
ENSG00000242048	*AC093583.1*	-0.008556875	0.25594421	0.509314154	-0.253369944	0.999933887	3.493669328	4.881390955	-1.387721627	0.005202514
ENSG00000196976	*LAGE3*	2.174520282	0.167482042	-0.032455655	0.199937697	0.999933887	0.949864622	1.508699022	-0.5588344	0.005208077
ENSG00000010404	*IDS*	6.336953002	0.039868501	-0.047712751	0.087581253	0.999933887	-0.475901297	-0.764996092	0.289094795	0.005208077
ENSG00000182141	*ZNF708*	4.139606838	-0.045324515	-0.083826364	0.038501849	0.999933887	-0.351467609	0.18128536	-0.532752969	0.005208077
ENSG00000175895	*PLEKHF2*	5.891265971	-0.024245039	-0.051368796	0.027123757	0.999933887	1.191822121	1.616971808	-0.425149686	0.005208077
ENSG00000100284	*TOM1*	9.589696082	-0.024398324	-0.014290426	-0.010107898	0.999933887	0.501629205	0.750741119	-0.249111914	0.005225811
ENSG00000174151	*CYB561D1*	3.960124052	-0.12061952	-0.078980802	-0.041638718	0.999933887	-0.597616031	-0.973349434	0.375733403	0.005282123
ENSG00000069702	*TGFBR3*	4.715804446	-0.033891681	-0.005112945	-0.028778737	0.999933887	0.364925695	0.848786971	-0.483861276	0.005282123
ENSG00000259668	*AC066613.1*	2.437570041	-0.040137432	-0.274346903	0.23420947	0.999933887	2.009144258	3.040766815	-1.031622557	0.005282898
ENSG00000138069	*RAB1A*	8.65850465	-0.023636826	-0.07208387	0.048447044	0.999933887	0.285795616	0.535041202	-0.249245586	0.005285536
ENSG00000172493	*AFF1*	6.683264873	-0.026585459	-0.008480053	-0.018105407	0.999933887	-1.176661978	-1.652167671	0.475505693	0.005288241
ENSG00000226328	*NUP50-DT*	2.95954831	-0.002343468	-0.001804447	-0.000539021	0.999933887	-1.126971128	-1.698032742	0.571061613	0.005302994
ENSG00000156313	*RPGR*	5.407542787	-0.116947971	-0.099300454	-0.017647517	0.999933887	0.672142225	1.348900846	-0.676758621	0.005307062
ENSG00000109062	*SLC9A3R1*	6.632480186	0.064486622	0.065570487	-0.001083864	0.999933887	-0.28309175	-0.642926982	0.359835232	0.005308939
ENSG00000136715	*SAP130*	4.761810787	0.040341284	0.010911476	0.029429808	0.999933887	-0.437181741	-0.891045045	0.453863304	0.005308939
ENSG00000187051	*RPS19BP1*	4.884794567	-0.038896066	0.011782855	-0.050678921	0.999933887	-0.498200594	-0.876364414	0.378163821	0.005308939
ENSG00000251194	*AL133330.1*	3.281279122	0.018666898	0.017352564	0.001314334	0.999933887	1.427727303	2.252329696	-0.824602393	0.005308939
ENSG00000014138	*POLA2*	0.774821026	-0.018873262	0.222194233	-0.241067494	0.999933887	0.839955881	1.649312695	-0.809356814	0.005308939
ENSG00000102921	*N4BP1*	8.735485799	-0.028986074	-0.041621432	0.012635357	0.999933887	1.332331149	1.646515449	-0.3141843	0.005326083
ENSG00000185379	*RAD51D*	1.624291782	-0.015850999	-0.071258067	0.055407068	0.999933887	0.408256077	0.954542745	-0.546286668	0.005326083
ENSG00000180316	*PNPLA1*	4.765212967	-0.063467594	0.060722817	-0.124190411	0.999933887	2.059766114	2.540005319	-0.480239206	0.00532927
ENSG00000214413	*BBIP1*	4.917252797	-0.069823367	0.034097678	-0.103921045	0.999933887	0.307657982	0.664464477	-0.356806496	0.00533851
ENSG00000127483	*HP1BP3*	6.388703735	0.007512906	0.04896427	-0.041451363	0.999933887	-0.318180067	-0.569134251	0.250954185	0.005394487
ENSG00000146457	*WTAP*	7.933392765	-0.050631115	-0.035136722	-0.015494393	0.999933887	1.746649698	2.131544034	-0.384894336	0.005445704
ENSG00000113657	*DPYSL3*	0.842000877	-0.265497182	-0.228131427	-0.037365755	0.999933887	0.297884286	0.865800418	-0.567916131	0.005479273
ENSG00000091592	*NLRP1*	7.560174275	0.024294482	0.02152258	0.002771902	0.999933887	-0.081421315	-0.291994279	0.210572964	0.005479273
ENSG00000253315	*LINC01932*	1.000142392	0.015737775	0.136530126	-0.120792351	0.999933887	0.702911326	1.283046568	-0.580135242	0.005479273
ENSG00000088930	*XRN2*	6.068564492	-0.051425286	-0.041878584	-0.009546702	0.999933887	-0.466919356	-0.731876863	0.264957507	0.005497848
ENSG00000102096	*PIM2*	8.078718708	0.001125791	-0.005720787	0.006846577	0.999933887	2.265407375	2.751990091	-0.486582716	0.005497848
ENSG00000154640	*BTG3*	2.356511009	-0.030187071	-0.11559977	0.085412699	0.999933887	1.003240743	1.667529146	-0.664288403	0.00551054
ENSG00000166900	*STX3*	9.246851234	-0.044557061	-0.034825861	-0.0097312	0.999933887	0.220139424	0.414591919	-0.194452495	0.00553225
ENSG00000157191	*NECAP2*	5.789217535	-0.011894609	-0.000312918	-0.011581691	0.999933887	0.45836293	0.71924131	-0.26087838	0.005533342
ENSG00000156411	*ATP5MPL*	4.167915537	-0.029391496	-0.098006863	0.068615367	0.999933887	-0.34273701	-0.671971341	0.329234331	0.005567498
ENSG00000163735	*CXCL5*	0.55621579	0.334881485	0.152685604	0.182195881	0.999933887	0.46985402	1.385194906	-0.915340886	0.00557473
ENSG00000058272	*PPP1R12A*	7.432293738	-0.109493413	-0.167538337	0.058044924	0.999933887	0.87583261	1.680997194	-0.805164583	0.005632858
ENSG00000125912	*NCLN*	5.482451299	0.046668222	0.02683189	0.019836332	0.999933887	-0.097106581	-0.406826483	0.309719901	0.005661506
ENSG00000133872	*SARAF*	8.221332769	0.025206086	-0.039590122	0.064796208	0.999933887	-0.339655099	-0.649245259	0.30959016	0.005670121
ENSG00000154767	*XPC*	4.655192273	-0.023746302	-0.038454358	0.014708056	0.999933887	-0.261022726	-0.567704338	0.306681611	0.005676414
ENSG00000130244	*FAM98C*	3.580396406	0.092606895	0.118000995	-0.0253941	0.999933887	0.013983647	-0.384718591	0.398702239	0.005709642
ENSG00000176390	*CRLF3*	4.733318426	0.042248276	0.067630508	-0.025382232	0.999933887	-0.646921196	-0.987037255	0.340116059	0.005796659
ENSG00000140455	*USP3*	5.072247854	-0.060419862	-0.064646145	0.004226282	0.999933887	-0.331253005	-0.593502926	0.262249922	0.005805739
ENSG00000169926	*KLF13*	7.123561532	0.037730676	-0.046750743	0.084481419	0.999933887	-0.384847731	-0.730830253	0.345982522	0.005818621
ENSG00000185973	*TMLHE*	1.735407472	0.008160366	-0.051200615	0.059360981	0.999933887	-0.47619982	-1.114144073	0.637944252	0.005818621
ENSG00000163006	*CCDC138*	0.448494754	0.00419948	0.142019684	-0.137820204	0.999933887	0.84140655	1.65459536	-0.81318881	0.005911906
ENSG00000065883	*CDK13*	5.57941409	0.050054387	0.0559366	-0.005882213	0.999933887	-0.434771711	-0.723161629	0.288389917	0.005932049
ENSG00000144802	*NFKBIZ*	9.553633073	0.278243582	0.233821579	0.044422003	0.999933887	3.006350961	3.588112948	-0.581761987	0.005949376
ENSG00000131473	*ACLY*	5.233086493	0.070239256	0.000716176	0.06952308	0.999933887	-0.226950404	-0.585909942	0.358959538	0.005963792
ENSG00000072135	*PTPN18*	4.717568983	0.030505659	0.015615377	0.014890282	0.999933887	-0.068002823	-0.343944218	0.275941395	0.005963792
ENSG00000123562	*MORF4L2*	6.954519264	-0.014519255	-0.107629731	0.093110476	0.999933887	0.336008323	0.672907562	-0.33689924	0.005963792
ENSG00000170142	*UBE2E1*	4.711799277	-0.0227354	0.019905899	-0.042641298	0.999933887	1.620806958	2.169583568	-0.54877661	0.006012132
ENSG00000136869	*TLR4*	7.731158654	-0.04920278	-0.071148254	0.021945474	0.999933887	0.841575215	1.223873474	-0.382298259	0.006031687
ENSG00000127824	*TUBA4A*	6.307991246	0.049632619	-0.028040523	0.077673142	0.999933887	-0.209618964	-0.54196969	0.332350725	0.006079579
ENSG00000222009	*BTBD19*	6.602120534	0.269965037	0.231341641	0.038623396	0.999933887	1.696406856	2.21741472	-0.521007863	0.006091781
ENSG00000080802	*CNOT4*	3.981804883	0.022664666	-0.099831643	0.122496309	0.999933887	0.185644758	0.573714632	-0.388069874	0.006095358
ENSG00000165410	*CFL2*	1.006135279	-0.225101433	-0.032819812	-0.192281622	0.999933887	0.487421059	1.177480661	-0.690059602	0.006105698
ENSG00000107140	*TESK1*	3.963576036	0.108690531	0.031537769	0.077152762	0.999933887	0.439545119	0.884534888	-0.444989769	0.006126175
ENSG00000166788	*SAAL1*	1.241209936	-0.235972238	-0.106793151	-0.129179087	0.999933887	1.037635674	1.732287964	-0.694652289	0.006135294
ENSG00000099337	*KCNK6*	3.289405662	-0.033921569	-0.031639834	-0.002281735	0.999933887	-0.435430844	-0.941348246	0.505917401	0.006135294
ENSG00000073169	*SELENOO*	4.631852765	0.06509843	-0.004914372	0.070012802	0.999933887	-0.23994688	-0.632822473	0.392875593	0.006138384
ENSG00000237499	*WAKMAR2*	4.565138736	0.24840286	0.239789802	0.008613059	0.999933887	1.707198569	2.292091171	-0.584892602	0.006150254
ENSG00000163975	*MELTF*	4.905739832	0.068323046	0.204646558	-0.136323512	0.999933887	0.484281926	0.978786493	-0.494504567	0.006150254
ENSG00000183044	*ABAT*	3.505722259	-0.043020226	0.070485399	-0.113505625	0.999933887	-0.156422096	-0.656935391	0.500513294	0.006150254
ENSG00000165775	*FUNDC2*	5.134838445	0.025429247	-0.061685899	0.087115146	0.999933887	0.222025126	0.534908799	-0.312883672	0.006169686
ENSG00000133624	*ZNF767P*	3.70912865	0.039541142	-0.004550521	0.044091662	0.999933887	-0.206884816	-0.502287792	0.295402977	0.00618531
ENSG00000180871	*CXCR2*	8.82889996	-0.007982065	-0.002784613	-0.005197452	0.999933887	-1.472834917	-2.069921889	0.597086971	0.00619039
ENSG00000146067	*FAM193B*	6.63448988	0.04672546	0.089590485	-0.042865025	0.999933887	-0.218256862	-0.472880713	0.254623851	0.006230361
ENSG00000015475	*BID*	8.520241359	0.034486041	-0.054724785	0.089210827	0.999933887	1.008709351	1.316515849	-0.307806499	0.006239497
ENSG00000135912	*TTLL4*	5.560865639	-0.017194627	-0.04049637	0.023301743	0.999933887	0.625576054	1.050312437	-0.424736383	0.006242407
ENSG00000151502	*VPS26B*	4.359905571	0.037588386	0.000491753	0.037096633	0.999933887	-0.157401015	-0.518040561	0.360639546	0.006272701
ENSG00000162521	*RBBP4*	3.741384568	-0.082105547	-0.109806641	0.027701094	0.999933887	-0.065362114	-0.364362829	0.299000715	0.006282121
ENSG00000286067	*AC004263.2*	4.168369937	-0.018211212	0.027902863	-0.046114075	0.999933887	-0.681350146	-1.135474236	0.45412409	0.006303087
ENSG00000162645	*GBP2*	9.789131942	-0.037781691	-0.192598971	0.15481728	0.999933887	0.711320768	1.16483777	-0.453517002	0.00631032
ENSG00000095951	*HIVEP1*	7.540204778	-0.029606514	-0.018033641	-0.011572873	0.999933887	0.840474149	1.407458116	-0.566983967	0.006311685
ENSG00000178567	*EPM2AIP1*	5.145081989	-0.229107687	-0.159851236	-0.069256451	0.999933887	2.049703894	2.861380254	-0.81167636	0.006344947
ENSG00000164144	*ARFIP1*	5.723284681	-0.096612354	-0.093660386	-0.002951967	0.999933887	0.541817387	0.944196409	-0.402379022	0.006344947

ENSG00000074621	*SLC24A1*	2.884744766	-0.128811803	-0.039682358	-0.089129445	0.999933887	2.658607755	3.659176392	-1.000568637	0.006344947
ENSG00000007202	*KIAA0100*	5.474601498	0.010026177	0.008110114	0.001916062	0.999933887	-0.173589481	-0.451498986	0.277909506	0.006361713
ENSG00000069399	*BCL3*	7.881679314	0.125306381	0.134548139	-0.009241758	0.999933887	1.925062756	2.37272177	-0.447659014	0.006372831
ENSG00000103042	*SLC38A7*	2.995340358	0.009747041	0.095443595	-0.085696554	0.999933887	0.218942103	0.549492454	-0.330550351	0.006375408
ENSG00000272886	*DCP1A*	5.855619853	-0.029705772	-0.039819823	0.010114051	0.999933887	0.415712134	0.665406204	-0.24969407	0.006446897
ENSG00000253372	*AC016405.1*	3.021059484	0.092433168	0.151734304	-0.059301136	0.999933887	1.713509559	2.326621798	-0.613112239	0.006447994
ENSG00000090060	*PAPOLA*	7.338860248	-0.04728022	-0.012213238	-0.035066982	0.999933887	-0.126597492	0.154879372	-0.281476864	0.006447994
ENSG00000107882	*SUFU*	2.275292503	-0.011786178	0.086915048	-0.098701227	0.999933887	-0.016284632	-0.577332596	0.561047964	0.006447994
ENSG00000113068	*PFDN1*	3.782800012	-0.058923265	-0.02215063	-0.036772635	0.999933887	0.184487694	0.487496413	-0.303008719	0.006477778
ENSG00000198830	*HMGN2*	6.590506442	0.010273405	-0.029921467	0.040194872	0.999933887	-0.195271736	-0.466271173	0.270999437	0.006496696
ENSG00000177946	*CENPBD1*	0.497518121	-0.048792623	-0.140549499	0.091756877	0.999933887	-0.488398765	-1.289487003	0.801088238	0.0065302
ENSG00000188886	*ASTL*	2.171631879	0.120680216	0.181303217	-0.060623001	0.999933887	0.857349925	1.533352123	-0.676002198	0.00654793
ENSG00000129465	*RIPK3*	3.687668293	-0.020641767	0.066828483	-0.08747025	0.999933887	-0.424291035	-0.794782866	0.370491831	0.006553929
ENSG00000164086	*DUSP7*	2.242424788	0.079707057	0.040002275	0.039704781	0.999933887	-0.442252279	-0.973264984	0.531012704	0.006590214
ENSG00000039319	*ZFYVE16*	8.201307998	-0.036642853	-0.071754875	0.035112022	0.999933887	-0.05030401	0.647873602	-0.698177612	0.006620652
ENSG00000247982	*LINC00926*	1.768623798	0.140282598	-0.119649286	0.259931884	0.999933887	0.280657055	0.802388519	-0.521731464	0.006663577
ENSG00000277443	*MARCKS*	10.588034292	-0.050743024	0.006325812	-0.057068836	0.999933887	0.91064083	1.275207291	-0.364566461	0.006700829
ENSG00000237803	*LINC00211*	5.468515434	0.027517138	-0.041337656	0.068854794	0.999933887	0.428366891	0.754362868	-0.325995977	0.00674196
ENSG00000152700	*SAR1B*	3.963489258	-0.059083323	0.064936384	-0.124019707	0.999933887	0.634157938	1.028427128	-0.39426919	0.006748846
ENSG00000101544	*ADNP2*	6.552911507	-0.049785368	-0.038895238	-0.01089013	0.999933887	0.610806431	0.881984617	-0.271178186	0.006782173
ENSG00000267080	*ASB16-AS1*	1.495934419	0.100179724	-0.030623523	0.130803247	0.999933887	-0.154324931	-0.82595192	0.671626989	0.006782173
ENSG00000198791	*CNOT7*	4.598208731	-0.065191945	-0.043110503	-0.022081442	0.999933887	-0.412769357	-0.709590916	0.296821559	0.006819588
ENSG00000214106	*PAXIP1-AS2*	1.74221589	-0.118981792	-0.121252789	0.002270997	0.999933887	0.192442822	0.864665147	-0.672222326	0.006832012
ENSG00000163412	*EIF4E3*	5.265294779	-0.024502009	-0.010713307	-0.013788703	0.999933887	-0.35539035	-0.63432577	0.27893542	0.006848499
ENSG00000257354	*AC048341.1*	1.699784257	-0.237111503	-0.155169062	-0.081942441	0.999933887	0.873040515	1.586036886	-0.712996371	0.006854837
ENSG00000106123	*EPHB6*	3.530529706	0.043263208	0.088080226	-0.044817019	0.999933887	-0.09260776	-0.540944759	0.448336999	0.006854837
ENSG00000204560	*DHX16*	4.686322628	0.025036849	0.01708145	0.0079554	0.999933887	-0.161776749	-0.418173501	0.256396752	0.006854837
ENSG00000144040	*SFXN5*	3.147121299	-0.076149705	-0.027947486	-0.048202219	0.999933887	-0.193229946	-0.587800113	0.394570167	0.006909551
ENSG00000160305	*DIP2A*	5.817257846	0.043035339	0.034397579	0.008637761	0.999933887	0.458866881	0.911538112	-0.452671231	0.006909551
ENSG00000158062	*UBXN11*	4.33920628	0.014163768	0.045851489	-0.031687721	0.999933887	-0.030003181	-0.337840501	0.30783732	0.006909551
ENSG00000160584	*SIK3*	6.680228321	0.011602072	0.006360971	0.005241101	0.999933887	0.467667737	0.748908931	-0.281241194	0.006909551
ENSG00000138449	*SLC40A1*	3.486121028	-0.147445441	-0.038140701	-0.109304739	0.999933887	-0.885323251	-1.45188929	0.566566038	0.006926248
ENSG00000120742	*SERP1*	7.314791898	0.050324643	-0.003048867	0.05337351	0.999933887	0.464237213	0.720348895	-0.256111682	0.007013498
ENSG00000085514	*PILRA*	8.545638083	0.072087313	0.042200261	0.029887052	0.999933887	1.72852057	2.157631989	-0.429111419	0.007032458
ENSG00000110711	*AIP*	4.541860858	0.020898758	0.0083998	0.012498958	0.999933887	-0.119983724	-0.510772832	0.390789107	0.007043205
ENSG00000145088	*EAF2*	0.982045682	0.033362525	0.00998713	0.023375395	0.999933887	0.19693704	0.882585902	-0.685648862	0.007051166
ENSG00000197818	*SLC9A8*	7.725213442	-0.024100132	0.024466359	-0.048566491	0.999933887	1.019430285	1.327790824	-0.308360538	0.007057086
ENSG00000258919	*AL049836.1*	1.878473129	-0.23338285	0.061368113	-0.294750964	0.999933887	0.966110402	1.429399711	-0.463289309	0.007084522
ENSG00000175582	*RAB6A*	4.658521134	-0.017938953	0.012459433	-0.030398386	0.999933887	-0.442529278	-0.791492406	0.348963127	0.007149497
ENSG00000237732	*CT75*	1.067569736	0.326871203	0.370437968	-0.043566765	0.999933887	3.098517158	3.760554862	-0.662037704	0.007192328
ENSG00000115307	*AUP1*	5.391924138	-0.002282138	0.038072387	-0.040354525	0.999933887	0.090390154	-0.149489116	0.23987927	0.007225737
ENSG00000163346	*PBXIP1*	8.071159004	0.018397679	0.03246097	-0.014063291	0.999933887	-0.150090464	-0.512718386	0.362627922	0.007244265
ENSG00000133731	*IMPA1*	3.390049008	-0.051834431	-0.137686795	0.085852364	0.999933887	-0.169772319	0.22073057	-0.390502889	0.007250413
ENSG00000254999	*BRK1*	5.84704191	0.064009315	-0.019108719	0.083118033	0.999933887	0.629275872	0.940793637	-0.311517764	0.007252815
ENSG00000116701	*NCF2*	11.05621892	-0.01562602	-0.018236708	0.002610688	0.999933887	-0.104238514	0.158283462	-0.262521976	0.007252815
ENSG00000185483	*ROR1*	-0.932285495	-0.013569945	0.630553648	-0.644123594	0.999933887	1.895819651	2.687452698	-0.791633046	0.007252815
ENSG00000179833	*SERTAD2*	6.295920189	-0.001014281	-0.079093034	0.078078754	0.999933887	0.82606723	1.339529626	-0.513462396	0.007252815
ENSG00000197837	*H4-16*	1.428911281	0.087275109	0.217087744	-0.129812635	0.999933887	-0.067138335	-0.670405377	0.603267042	0.007280029
ENSG00000250571	*GLI4*	1.974715723	-0.014462424	0.044077972	-0.058540396	0.999933887	-0.005822672	-0.467228325	0.461405653	0.007280029
ENSG00000075975	*MKRN2*	4.028781084	-0.001215932	0.036264121	-0.037480053	0.999933887	-0.554881418	-0.870486006	0.315604589	0.007280029
ENSG00000147416	*ATP6V1B2*	10.461574598	-0.076140959	-0.066405538	-0.009735422	0.999933887	0.215486371	0.492345461	-0.27685909	0.007281496
ENSG00000115486	*GGCX*	3.407708285	0.031573035	-0.061205202	0.092778237	0.999933887	-0.195001762	-0.539611429	0.344609668	0.007295388
ENSG00000115306	*SPTBN1*	7.142172345	-0.004804384	-0.057770385	0.052966001	0.999933887	-0.009753102	0.23734287	-0.247095972	0.007295388
ENSG00000112659	*CUL9*	4.849573357	0.054484356	0.058748227	-0.004263871	0.999933887	0.207333594	0.483460414	-0.276126821	0.007336609
ENSG00000127947	*PTPN12*	7.617749838	-0.013053284	-0.047316211	0.034262927	0.999933887	0.370199349	0.71470769	-0.344508341	0.00735351
ENSG00000067048	*DDX3Y*	-1.64642325	0.048116252	0.079064675	-0.030948423	0.999933887	0.408752915	0.958617198	-0.549864283	0.007357975
ENSG00000026297	*RNASET2*	7.893252123	0.050599568	0.069568919	-0.018969351	0.999933887	-0.340269927	-0.799778813	0.459508886	0.007382221
ENSG00000170892	*TSEN34*	5.501151433	-0.046696609	-0.046828413	0.000131804	0.999933887	-1.227307582	-1.728692947	0.501385365	0.007416857
ENSG00000111671	*SPSB2*	1.749860455	0.034793305	0.021895294	0.01289801	0.999933887	-0.322212775	-0.853584348	0.531371573	0.007500167
ENSG00000112110	*MRPL18*	3.457235575	-0.045668324	0.045991678	-0.091660002	0.999933887	1.019323114	1.439728846	-0.420405733	0.00754233
ENSG00000116337	*AMPD2*	7.984183341	0.03185726	0.083039707	-0.051182447	0.999933887	-0.400728105	-0.830252893	0.429524788	0.007563416
ENSG00000120008	*WDR11*	3.280297343	-0.078646644	-0.074039744	-0.0046069	0.999933887	0.362658897	0.818372611	-0.455713714	0.007653018
ENSG00000138303	*ASCC1*	2.683066665	0.015202489	0.045827804	-0.030625315	0.999933887	0.474291858	0.877612843	-0.403320986	0.007653018
ENSG00000102580	*DNAJC3*	7.73492038	-0.058551561	-0.066996207	0.008444646	0.999933887	0.623167815	1.012883588	-0.389715773	0.007663374
ENSG00000129625	*REEP5*	4.68815165	-0.015369739	-0.040943088	0.02557335	0.999933887	-0.068925719	-0.464082739	0.39515702	0.007675356
ENSG00000103197	*TSC2*	5.616914762	0.051372522	0.09892222	-0.047549698	0.999933887	-0.165759329	-0.444242681	0.278483352	0.007684693
ENSG00000069493	*CLEC2D*	5.275837277	-0.08070252	-0.021057853	-0.059644667	0.999933887	0.109765046	0.584422387	-0.474657342	0.007684693
ENSG00000185722	*ANKFY1*	5.601852858	-0.014399458	0.070770167	-0.085169625	0.999933887	-0.328760717	-0.633044376	0.304283659	0.007684693
ENSG00000169891	*REPS2*	5.38951638	-0.02983419	-0.00514755	-0.02468664	0.999933887	0.537120177	0.817268918	-0.280148741	0.007698726
ENSG00000180767	*CHST13*	1.723275454	-0.01917439	0.043256669	-0.062431059	0.999933887	-1.393519558	-2.280809698	0.887290141	0.007698726
ENSG00000114383	*TUSC2*	4.560981395	-0.024819705	0.007623866	-0.032443571	0.999933887	-0.502928932	-0.854307704	0.351378772	0.007701037
ENSG00000113742	*CPEB4*	8.135905938	0.001536958	-0.023837838	0.025374796	0.999933887	0.656624727	1.060495094	-0.403870367	0.007708398
ENSG00000197774	*EME2*	2.892750048	0.046284251	0.166379475	-0.120095224	0.999933887	-0.050534085	-0.518325547	0.467791462	0.007708851
ENSG00000135862	*LAMC1*	0.741234602	-0.046436732	-0.090859175	0.044422444	0.999933887	0.956361629	1.591466213	-0.635104584	0.007711634
ENSG00000188056	*TREML4*	5.850977593	0.083740387	-0.015807792	0.099548179	0.999933887	0.787773689	1.177304059	-0.389530371	0.007788685
ENSG00000065357	*DGKA*	6.339863522	-0.039048387	0.011776475	-0.050824862	0.999933887	-0.061200757	-0.249721082	0.188520324	0.007788685
ENSG00000142227	*EMP3*	7.379945103	0.021853365	-0.029390585	0.051243951	0.999933887	0.207941289	0.465236593	-0.257295303	0.007788685
ENSG00000101745	*ANKRD12*	7.616499703	-0.223477793	-0.194490488	-0.028987304	0.999933887	-0.271871632	0.466464365	-0.738335997	0.007791588
ENSG00000246705	*H2AJ*	2.502137642	0.073882861	-0.075985569	0.14986843	0.999933887	0.074491981	-0.391767161	0.466259143	0.007791588
ENSG00000115866	*DARS1*	4.717697135	-0.110450237	-0.09873641	-0.011713827	0.999933887	-0.14740984	0.204208875	-0.351618714	0.007792764
ENSG00000166002	*SMCO4*	1.649482553	0.086452561	0.300408062	-0.213955501	0.999933887	0.665315003	1.393601334	-0.728286331	0.007871046
ENSG00000124224	*PPP4R1L*	5.413614204	0.00027608	0.033558623	-0.033282543	0.999933887	0.019492476	0.36184914	-0.342356665	0.00789778
ENSG00000198604	*BAZ1A*	7.627105422	-0.111590618	-0.11888956	0.007298941	0.999933887	1.276620776	1.772810568	-0.496189791	0.007901347
ENSG00000006634	*DBF4*	4.231712367	-0.12276275	-0.081076047	-0.041686703	0.999933887	0.623144204	1.170856112	-0.547711908	0.007901347
ENSG00000130363	*RSPH3*	1.89835293	-0.053921012	0.066101241	-0.120022253	0.999933887	-0.701122201	-1.312836778	0.611714576	0.007901347
ENSG00000189308	*LIN54*	5.220006374	-0.037683838	-0.067130834	0.029446995	0.999933887	-0.413354687	-0.033074289	-0.380280398	0.007901347
ENSG00000108639	*SYNGR2*	5.986150791	0.044440783	-0.020447443	0.064888226	0.999933887	0.851842974	1.153051224	-0.30120825	0.007909253
ENSG00000139832	*RAB20*	6.321999612	0.027702875	-0.052422961	0.080125836	0.999933887	0.627383219	1.097166336	-0.469783116	0.007919104
ENSG00000100084	*HIRA*	2.950524813	0.09221321	-0.06853663	0.16074984	0.999933887	-0.843250188	-1.52319878	0.679948592	0.007971083
ENSG00000011422	*PLAUR*	10.36394921	0.263083404	0.149132799	0.113950606	0.999933887	1.465440783	1.931537037	-0.466096254	0.007987076
ENSG00000130764	*LRRC47*	3.446088508	0.019268954	0.073730564	-0.05446161	0.999933887	-0.119459384	-0.467908804	0.348449421	0.007992253
ENSG00000028528	*SNX1*	5.531543624	-0.088315639	0.012386117	-0.100701756	0.999933887	-0.78452461	-1.125269412	0.340744801	0.008007374
ENSG00000083799	*CYLD*	8.195987782	-0.058994762	-0.055966368	-0.003028393	0.999933887	0.750196834	1.06666821	-0.316471376	0.008013885
ENSG00000101384	*JAG1*	5.3647317	0.074031906	-0.048235084	0.122266989	0.999933887	2.874130023	3.632928836	-0.758798813	0.00802485
ENSG00000139163	*ETNK1*	4.8069626	-0.032137168	-0.050177931	0.018040763	0.999933887	0.148142585	0.626403696	-0.47826111	0.00802485
ENSG00000158122	*PRXL2C*	2.748814205	-0.031437195	-0.023070609	-0.008366586	0.999933887	0.383216743	0.841380847	-0.458164104	0.008102961
ENSG00000115310	*RTN4*	8.570093176	0.003816174	-0.00017732	0.003993493	0.999933887	0.242921413	0.472488965	-0.229567551	0.008103924
ENSG00000108669	*CYTH1*	8.970131961	-0.025739655	-0.005774955	-0.0199647	0.999933887	0.464294605	0.721399684	-0.25710508	0.008134299
ENSG00000110315	*RNF141*	5.72532245	-0.058814435	-0.027778406	-0.031036029	0.999933887	0.887723933	1.423184991	-0.535461059	0.008142285
ENSG00000102858	*MGRN1*	7.223779947	0.006459862	0.000424374	0.006035489	0.999933887	-0.247268279	-0.483711001	0.236442721	0.008188397
ENSG00000141968	*VAV1*	7.48727601	0.020721786	0.021766063	-0.001044277	0.999933887	0.754881636	1.114934685	-0.360053048	0.00823616
ENSG00000137185	*ZSCAN9*	1.454835106	0.000427955	-0.269245257	0.269673212	0.999933887	0.236410659	0.723619081	-0.487208422	0.008260023
ENSG00000188167	*TMPPE*	2.817871281	-0.032266097	0.033500703	-0.065766799	0.999933887	-0.820148509	-1.287297206	0.467148698	0.008336163
ENSG00000172113	*NME6*	2.422622624	0.045015251	0.096766577	-0.051751326	0.999933887	-0.304773696	-0.755948744	0.451175048	0.0083739
ENSG00000198876	*DCAF12*	3.983717148	-0.092292788	-0.039720018	-0.052572771	0.999933887	-0.444370503	-0.89343553	0.449065028	0.008384176

ENSG00000111913	*RIPOR2*	8.685730181	-0.010690329	-0.012049778	0.001359449	0.999933887	0.190258877	0.474323939	-0.284065063	0.008384176
ENSG00000118689	*FOXO3*	7.415723562	-0.071213079	-0.007058266	-0.064154813	0.999933887	-0.455215748	-0.770087462	0.314871714	0.008436443
ENSG00000267344	*AC003070.1*	0.819858953	0.125340914	0.12361689	0.001724024	0.999933887	-0.424995069	-1.172749924	0.747754855	0.008445986
ENSG00000197724	*PHF2*	5.552758163	0.015954168	0.054487198	-0.03853303	0.999933887	-0.204575333	-0.489490494	0.284915161	0.008475058
ENSG00000137075	*RNF38*	5.334514625	0.069113905	0.083392858	-0.014278952	0.999933887	-0.740417306	-1.13255531	0.392138004	0.008477772
ENSG00000198690	*FAN1*	3.878633311	0.029439222	0.03671495	-0.007275729	0.999933887	-0.285490501	-0.610817983	0.325327482	0.008477772
ENSG00000163867	*ZMYM6*	2.278822682	-0.048520732	-0.078308422	0.029787689	0.999933887	-0.3568653	0.290111348	-0.646976648	0.008488436
ENSG00000164576	*SAP30L*	3.844219984	0.022565829	-0.047387652	0.069953482	0.999933887	-0.396230067	-0.732024485	0.335794418	0.008521696
ENSG00000240057	*AC078785.1*	-1.105478127	-0.221132418	0.14248819	-0.363620608	0.999933887	0.430922284	1.523295352	-1.092373068	0.008526681
ENSG00000164091	*WDR82*	6.69767817	-0.054472185	-0.03603354	-0.018438645	0.999933887	-0.527502749	-0.805395269	0.277892519	0.008535052
ENSG00000137752	*CASP1*	7.729227293	-0.073118065	-0.166153155	0.09303509	0.999933887	1.027983393	1.400422697	-0.372439303	0.008535052
ENSG00000105732	*ZNF574*	3.26246829	0.047699629	-0.051022353	0.098721982	0.999933887	-0.171753679	-0.518705708	0.346952029	0.008543685
ENSG00000129968	*ABHD17A*	7.111969279	0.097078644	0.085275464	0.01180318	0.999933887	-0.402052509	-0.809047039	0.40699453	0.008574397
ENSG00000087589	*CASS4*	5.548015034	-0.023578992	0.118452036	-0.142031028	0.999933887	-1.36212561	-1.987303183	0.625177573	0.008581508
ENSG00000105705	*SUGP1*	3.752765879	0.029609197	0.028195013	0.001414184	0.999933887	-0.268066994	-0.626477605	0.358410611	0.008582344
ENSG00000099622	*CIRBP*	7.344196253	0.009293952	-0.018678479	0.027972431	0.999933887	-0.358698205	-0.617325433	0.258627228	0.00861444
ENSG00000186141	*POLR3C*	3.129255218	-0.071712083	-0.123959647	0.052247564	0.999933887	0.984257653	1.660109197	-0.675851544	0.008626883
ENSG00000137574	*TGS1*	3.470335178	-0.086385404	-0.153104433	0.066719029	0.999933887	0.446737108	0.792291122	-0.345554014	0.008643361
ENSG00000140379	*BCL2A1*	9.502010607	0.067646164	0.018391678	0.049254486	0.999933887	1.112657558	1.519824694	-0.407167136	0.008663522
ENSG00000103381	*CPPED1*	7.383857608	-0.022206055	-0.028700114	0.006494059	0.999933887	-0.797530593	-1.235235777	0.437705184	0.008682849
ENSG00000197283	*SYNGAP1*	3.433721363	0.054618727	0.022598673	0.032020053	0.999933887	0.554118236	1.012174742	-0.458056506	0.008758927
ENSG00000112851	*ERBIN*	8.867037715	-0.059411405	-0.113003686	0.053592281	0.999933887	0.280364547	0.920821974	-0.640457427	0.008758927
ENSG00000153936	*HS2ST1*	3.51864175	-0.073638513	0.024600588	-0.098239101	0.999933887	-0.361557913	0.0930506	-0.454608513	0.008791191
ENSG00000112149	*CD83*	10.188261769	0.065820514	0.008129367	0.057691147	0.999933887	1.28479596	1.645788295	-0.360992335	0.008821656
ENSG00000144895	*EIF2A*	3.932884115	-0.036175946	-0.09252491	0.056348964	0.999933887	0.143724542	0.465548796	-0.321824255	0.008821656
ENSG00000108061	*SHOC2*	6.82873088	-0.044717839	-0.039424798	-0.005293041	0.999933887	-0.232896546	0.169893842	-0.402790388	0.008821656
ENSG00000015133	*CCDC88C*	6.049497525	0.025536269	0.080039596	-0.054503327	0.999933887	-0.102276724	-0.33244024	0.230163516	0.008821656
ENSG00000196544	*BORCS6*	1.568606373	0.053862687	0.045777657	0.00808503	0.999933887	-0.498062414	-1.145372366	0.647309952	0.008821656
ENSG00000123815	*COQ8B*	4.074781103	0.021585558	0.076854979	-0.055269421	0.999933887	-0.071429719	-0.40464554	0.333215821	0.008821656
ENSG00000180398	*MCFD2*	4.230836099	0.019559114	0.028920252	-0.009361138	0.999933887	0.433165359	0.747074417	-0.313909058	0.008821656
ENSG00000145901	*TNIP1*	9.417293046	-0.015926756	-0.019990994	0.004064238	0.999933887	1.564320029	1.959873311	-0.395553282	0.008821656
ENSG00000172053	*QARS1*	4.834482721	0.004996353	-0.018015692	0.023012045	0.999933887	0.045931339	-0.227504647	0.273435986	0.008860491
ENSG00000109320	*NFKB1*	8.123360281	-0.052092572	0.000575656	-0.052668228	0.999933887	2.135915491	2.602510778	-0.466595287	0.008890492
ENSG00000260279	*AC137932.1*	3.19618909	-0.102602773	-0.076685242	-0.025917531	0.999933887	-0.366181806	-0.818728769	0.452546963	0.008895359
ENSG00000172724	*CCL19*	-2.234237265	0.034191327	0.203420777	-0.169229451	0.999933887	1.591513298	3.180011971	-1.588498672	0.008895359
ENSG00000093134	*VNN3*	6.520946651	-0.080894637	-0.108275103	0.027380466	0.999933887	0.389035417	0.788922221	-0.399886805	0.008907669
ENSG00000063244	*U2AF2*	6.950693277	0.029493899	0.041455657	-0.011961759	0.999933887	-0.289845887	-0.550767859	0.260921972	0.008928526
ENSG00000110925	*CSRNP2*	3.550893026	0.015878059	0.08850649	-0.072628431	0.999933887	0.546140224	0.972949395	-0.42680917	0.008964674
ENSG00000196411	*EPHB4*	2.842045315	0.023721696	0.019943046	0.003778651	0.999933887	-0.110639994	-0.699281402	0.588641408	0.008967352
ENSG00000241258	*CRCP*	3.876769748	-0.086555022	-0.003539725	-0.083015297	0.999933887	-0.194022562	-0.557695036	0.363672474	0.008970024
ENSG00000138073	*PREB*	3.542669637	0.010939319	0.03501886	-0.024079542	0.999933887	-0.172041623	-0.486477305	0.314435682	0.009005826
ENSG00000066697	*MSANTD3*	1.582945023	-0.157287979	-0.056899737	-0.100388242	0.999933887	1.603539138	2.266420058	-0.66288092	0.009015869
ENSG00000184602	*SNN*	8.934781355	-0.023253671	-0.083323711	0.06007004	0.999933887	0.83123069	1.175244419	-0.344013729	0.009017292
ENSG00000164691	*TAGAP*	9.80561271	0.143195625	0.114807415	0.02838821	0.999933887	1.761879135	2.236951365	-0.475072229	0.009058621
ENSG00000089057	*SLC23A2*	6.329707518	-0.098653133	-0.047446687	-0.051206446	0.999933887	-1.094861131	-1.405946363	0.311085231	0.00906111
ENSG00000083937	*CHMP2B*	7.402111048	-0.018762642	-0.145378767	0.126616124	0.999933887	0.222524808	0.540164844	-0.317640036	0.009074981
ENSG00000108312	*UBTF*	5.342946152	0.01437657	0.043977123	-0.029600553	0.999933887	-0.090624339	-0.358642992	0.268018653	0.009074981
ENSG00000090316	*MAEA*	7.771152537	0.003000138	-0.049523546	0.052523683	0.999933887	0.463651358	0.709301727	-0.245650369	0.009133618
ENSG00000168175	*MAPK1IP1L*	6.489435749	-0.042010296	-0.019640576	-0.022369721	0.999933887	0.170908961	0.394934502	-0.22402554	0.009195328
ENSG00000096060	*FKBP5*	5.063923616	0.009505567	-0.024003858	0.033509425	0.999933887	0.42109077	0.857416008	-0.436325238	0.009195328
ENSG00000182676	*PPP1R27*	0.657344415	0.257614549	0.329162542	-0.071547994	0.999933887	1.630097755	2.605651107	-0.975553352	0.009202286
ENSG00000121749	*TBC1D15*	6.885800741	-0.074316788	-0.081202211	0.006885423	0.999933887	-0.07454639	0.387762523	-0.462308913	0.009228434
ENSG00000198818	*SFT2D1*	5.545389777	-0.001707519	0.007279126	-0.008986644	0.999933887	0.278317621	0.514375997	-0.236058375	0.009253392
ENSG00000049249	*TNFRSF9*	3.321266406	0.040620289	-0.08480377	0.125424059	0.999933887	1.173616968	1.669775228	-0.49615826	0.009264876
ENSG00000232912	*RERE-AS1*	0.073829276	0.058165456	-0.097011972	0.155177428	0.999933887	-0.772210625	-1.712912145	0.940701521	0.009275755
ENSG00000078369	*GNB1*	8.965924571	0.015812432	0.007155988	0.008656445	0.999933887	0.34639291	0.568311968	-0.221919058	0.009341579
ENSG00000197548	*ATG7*	6.633400633	0.036268487	0.094143831	-0.057875344	0.999933887	1.452725447	1.80803993	-0.355314483	0.009398771
ENSG00000176542	*USF3*	7.836563386	-0.049044488	-0.086995249	0.037950761	0.999933887	0.666176245	1.197499062	-0.531322816	0.009402896
ENSG00000103222	*ABCC1*	6.02604141	0.032594555	0.037043395	-0.004448841	0.999933887	0.713192601	1.039850184	-0.326657583	0.009406642
ENSG00000166164	*BRD7*	4.193765859	-0.01204484	-0.078326298	0.066281458	0.999933887	-0.153738624	-0.463833156	0.310094531	0.009406642
ENSG00000089818	*NECAP1*	7.677512757	-0.000935435	0.022632537	-0.023567972	0.999933887	0.905468036	1.209450868	-0.303982831	0.009406642
ENSG00000144824	*PHLDB2*	1.021912735	-0.134666333	-0.00339066	-0.131275673	0.999933887	-0.198426138	0.574878096	-0.773304235	0.009414184
ENSG00000226137	*BAIAP2-DT*	1.071123398	0.079335706	0.130551061	-0.051215355	0.999933887	-0.752480221	-1.64244499	0.889964769	0.009482901
ENSG00000143851	*PTPN7*	5.978877678	0.005731372	-0.003760213	0.009491584	0.999933887	-0.278638068	-0.520342213	0.241704145	0.009482901
ENSG00000213073	*CHP1P2*	5.514519925	0.006988579	0.027512921	-0.020524342	0.999933887	-0.043870424	0.229529526	-0.27339995	0.009494612
ENSG00000157020	*SEC13*	5.281488213	0.026031187	-0.007305728	0.033336915	0.999933887	0.921007004	1.210820858	-0.289813854	0.00949591
ENSG00000140553	*UNC45A*	3.843271187	-0.066109764	0.012404157	-0.078513921	0.999933887	-0.403809327	-0.748808291	0.344998964	0.009540465
ENSG00000133703	*KRAS*	5.461825027	-0.032985494	-0.023658962	-0.009326532	0.999933887	0.238819641	0.578703356	-0.339883715	0.009570827
ENSG00000229314	*ORM1*	6.265369327	-0.083518107	-0.0129869	-0.070531207	0.999933887	1.906484722	2.454878383	-0.54839366	0.009583212
ENSG00000107679	*PLEKHA1*	3.111521934	-0.06132005	-0.098659563	0.037339513	0.999933887	-0.13251171	0.270249838	-0.402761548	0.009654826
ENSG00000124391	*IL17C*	-0.923840129	-0.263117486	0.091984826	-0.355102313	0.999933887	2.19907867	3.682139989	-1.483061318	0.009667701
ENSG00000214078	*CPNE1*	5.931749481	-0.013730516	-0.009508467	-0.004222049	0.999933887	-0.226117658	-0.448782654	0.222664996	0.009670933
ENSG00000160190	*SLC37A1*	4.079318244	0.091788901	0.022445373	0.069343528	0.999933887	0.2144093	0.496587174	-0.282177874	0.009770337
ENSG00000177542	*SLC25A22*	2.636201158	0.078608303	0.051326041	0.027282263	0.999933887	-0.221748663	-0.588552586	0.366803924	0.009770337
ENSG00000153560	*UBP1*	4.650206307	-0.043071164	-0.041290543	-0.001780621	0.999933887	-0.13505712	-0.357305131	0.222248012	0.009770337
ENSG00000028137	*TNFRSF1B*	11.174381683	-0.021473395	-0.067566405	0.04609301	0.999933887	0.367778706	0.626660999	-0.258882293	0.009808343
ENSG00000167323	*STIM1*	5.270823792	0.027328536	-0.015312105	0.042640641	0.999933887	-0.082401828	-0.398636699	0.316234871	0.009809565
ENSG00000110958	*PTGES3*	5.462545915	-0.046095511	-0.066941526	0.020846015	0.999933887	-0.296022828	-0.533195599	0.237172771	0.009842122
ENSG00000130584	*ZBTB46*	1.858074077	-0.037413913	-0.087096973	0.049683059	0.999933887	0.905896053	1.521510084	-0.615614031	0.009842122
ENSG00000160404	*TOR2A*	3.543413824	0.08592025	0.033963457	0.051956793	0.999933887	-0.488581821	-0.923544837	0.434963015	0.009848846
ENSG00000104904	*OAZ1*	8.742387652	0.030133638	0.007685578	0.02244806	0.999933887	-0.120888419	-0.36036131	0.239472891	0.00988455
ENSG00000125863	*MKKS*	1.770474631	0.18033562	0.117344542	0.062991077	0.999933887	1.379000312	1.985456542	-0.606456231	0.009893026
ENSG00000125304	*TM9SF2*	6.717711858	-0.076129994	-0.068458879	-0.007671115	0.999933887	-0.599544822	-0.903973644	0.304428822	0.009944651
ENSG00000152409	*JMY*	5.745621033	-0.046111212	-0.119029985	0.072918773	0.999933887	0.127976507	0.518526867	-0.39055036	0.009953104
ENSG00000120063	*GNA13*	8.876704312	-0.049292756	-0.074489612	0.025196855	0.999933887	-0.348288931	-0.073262741	-0.27502619	0.00997099
ENSG00000129646	*QRICH2*	1.398124915	0.077687199	0.16529589	-0.087608691	0.999933887	0.827455381	1.568663662	-0.741208281	0.009975333
ENSG00000122694	*GLIPR2*	7.010974532	-0.011302969	-0.027946205	0.016643236	0.999933887	-0.340342749	-0.675142287	0.334799538	0.010041767
ENSG00000236790	*LINC00299*	1.799289496	0.041228566	-0.010158516	0.051387082	0.999933887	0.918919667	1.64162158	-0.722701913	0.010088935
ENSG00000152270	*PDE3B*	5.647294421	0.01997071	-0.009712254	0.029682964	0.999933887	-0.58367611	-1.029507394	0.445831284	0.010090507
ENSG00000278195	*SSTR3*	4.186693269	0.190153606	0.125898613	0.064254993	0.999933887	0.54445333	0.982243936	-0.437790607	0.010133444
ENSG00000251615	*AC104825.1*	-0.023591424	-0.253317254	-0.029516397	-0.223800857	0.999933887	0.759468217	1.552275775	-0.792807558	0.010133444
ENSG00000176788	*BASP1*	10.901470166	0.058799905	0.0649927	-0.006192795	0.999933887	0.42565455	0.759005232	-0.333350682	0.010133444
ENSG00000258867	*LINC01146*	0.154541979	-0.129548017	0.092530116	-0.222078133	0.999933887	0.342286967	1.274965253	-0.932678286	0.010148721
ENSG00000103479	*RBL2*	6.146165119	-0.011579048	-0.036353435	0.024774388	0.999933887	-0.322176561	-0.581388196	0.259211635	0.010148721
ENSG00000251023	*AC114980.1*	0.254325548	0.097403908	-0.316571353	0.413975261	0.999933887	-0.647488076	0.225279144	-0.87276722	0.010153306
ENSG00000068383	*INPP5A*	5.847255056	0.016687967	0.017312662	-0.000624695	0.999933887	0.616073765	0.861324896	-0.245251132	0.010229382
ENSG00000129667	*RHBDF2*	7.101719398	0.007965546	0.019476173	-0.011510627	0.999933887	1.157807091	1.564998487	-0.407191396	0.010239963
ENSG00000136731	*UGGT1*	5.686446829	-0.046944151	-0.055159896	0.008215744	0.999933887	-0.150149625	-0.377716083	0.227566458	0.010363449
ENSG00000277130	*AC073569.3*	0.175215792	-0.010036518	0.030645543	-0.040682061	0.999933887	1.168727557	2.119671905	-0.950944349	0.010363449
ENSG00000102547	*CAB39L*	3.289958063	-0.043269032	-0.061952555	0.018683523	0.999933887	-0.390628454	-0.103091743	-0.287536711	0.010372544
ENSG00000185947	*ZNF267*	8.982942632	-0.103472242	-0.221857872	0.11838563	0.999933887	0.226989291	0.756069777	-0.529080486	0.010401281
ENSG00000109065	*NAT9*	4.689996478	0.037694899	0.048041382	-0.010346483	0.999933887	-0.294480414	-0.612633296	0.318152882	0.010494747
ENSG00000185728	*YTHDF3*	7.315253888	-0.048455314	-0.078716134	0.03026082	0.999933887	0.007688322	0.357356537	-0.349668215	0.010506339
ENSG00000162961	*DPY30*	2.505445363	0.058762763	-0.068587064	0.127349827	0.999933887	0.092053026	-0.44978681	0.541839836	0.010506339
ENSG00000091527	*CDV3*	7.072038252	-0.007418973	-0.000884811	-0.006534162	0.999933887	-0.572257877	-0.832656247	0.26039837	0.010506339

ENSG00000251136	*AF117829.1*	3.511940203	0.10538667	0.185128174	-0.079741504	0.999933887	1.834988462	2.363214407	-0.528225946	0.01050666
ENSG00000131504	*DIAPH1*	7.842246107	-0.028113308	-0.01402163	-0.014091678	0.999933887	-0.412681204	-0.648918313	0.236237109	0.010509856
ENSG00000145860	*RNF145*	7.240198162	-0.02325847	-0.00711028	-0.01614819	0.999933887	0.00213308	0.212190768	-0.210057689	0.010509856
ENSG00000197442	*MAP3K5*	5.858460184	-0.011324201	-0.027934916	0.016610715	0.999933887	0.709733419	1.118590911	-0.408857492	0.010509856
ENSG00000158161	*EYA3*	5.182168039	-0.032539437	-0.11595749	0.083418053	0.999933887	1.047202629	1.505236559	-0.45803393	0.010524484
ENSG00000163348	*PYGO2*	3.671006864	-0.051331624	-0.059485585	0.008153961	0.999933887	-0.056798566	-0.350647826	0.29384926	0.010541794
ENSG00000101160	*CTSZ*	6.369313139	0.064040072	0.080247127	-0.016207055	0.999933887	-0.571192573	-1.058611308	0.487418736	0.010541794
ENSG00000152503	*TRIM36*	-0.328209948	-0.077627758	0.287528113	-0.365155871	0.999933887	1.381463354	2.314578902	-0.933115549	0.010541794
ENSG00000185650	*ZFP36L1*	10.227713044	0.158110291	0.27203099	-0.113920699	0.999933887	-0.051854803	0.213379747	-0.26523455	0.010563592
ENSG00000213281	*NRAS*	5.33815715	-0.055862738	-0.062162689	0.006299951	0.999933887	-0.134181905	0.122195606	-0.256377511	0.010563592
ENSG00000181790	*ADGRB1*	2.663837468	0.206042661	-0.012829977	0.218872638	0.999933887	1.110438731	1.545072711	-0.43463398	0.010563592
ENSG00000185049	*NELFA*	3.177133043	0.060862976	0.020056231	0.040806745	0.999933887	-0.002577353	-0.354905388	0.352328035	0.010563592
ENSG00000177105	*RHOG*	8.561865451	0.020831145	-0.016672371	0.037503516	0.999933887	0.624062492	0.943118165	-0.319055672	0.010617206
ENSG00000115762	*PLEKHB2*	8.825658248	-0.077090917	-0.02254532	-0.054545597	0.999933887	0.661424032	0.90188612	-0.240462087	0.010632742
ENSG00000240065	*PSMB9*	6.759828514	0.07329268	-0.002422189	0.075714869	0.999933887	-0.275809712	-0.573482424	0.297672712	0.010632742
ENSG00000005889	*ZFX*	6.097715613	-0.072119743	-0.052820689	-0.019299053	0.999933887	-0.102311731	0.268264034	-0.370575766	0.010632742
ENSG00000125430	*HS3ST3B1*	4.056506548	0.041534981	0.071983571	-0.03044859	0.999933887	1.557413477	2.442758688	-0.885345211	0.010632742
ENSG00000167642	*SPINT2*	3.587560123	0.066144615	0.070899075	-0.00475446	0.999933887	0.727382751	1.145069414	-0.417686663	0.010688145
ENSG00000113575	*PPP2CA*	6.793789624	-0.025398411	-0.059779993	0.034381583	0.999933887	0.082997606	0.2831465	-0.200148894	0.010760612
ENSG00000237765	*FAM200B*	5.135756596	0.029675396	0.121716292	-0.092040896	0.999933887	0.109943254	0.448373787	-0.338430534	0.010799218
ENSG00000171490	*RSL1D1*	4.470106222	-0.036120295	-0.144780274	0.108659979	0.999933887	0.268775929	0.563937984	-0.295162055	0.010807398
ENSG00000090924	*PLEKHG2*	5.728202768	0.137574351	0.072999346	0.064575004	0.999933887	0.573412475	0.897490486	-0.324078011	0.010832781
ENSG00000126883	*NUP214*	6.1816606	-0.028549281	0.021537208	-0.050086489	0.999933887	-0.447257297	-0.764967384	0.317710087	0.010861629
ENSG00000160791	*CCR5*	3.190003322	-0.007802735	-0.030219511	0.022416776	0.999933887	0.383318144	0.721141981	-0.337823837	0.010946562
ENSG00000112242	*E2F3*	6.98068466	-0.073683169	-0.059241002	-0.014442167	0.999933887	0.007584752	0.237992072	-0.23040732	0.010954779
ENSG00000143878	*RHOB*	6.048418309	0.117438298	0.087354419	0.030083879	0.999933887	-0.475420707	-0.991000708	0.515580001	0.010997122
ENSG00000153291	*SLC25A27*	1.941049561	-0.062693327	-0.157886435	0.095193108	0.999933887	0.243143129	0.683686901	-0.440543772	0.010997122
ENSG00000185753	*CXorf38*	4.915340699	-0.086399895	-0.031077622	-0.055322273	0.999933887	-0.746023256	-1.111652666	0.36562941	0.011009003
ENSG00000139645	*ANKRD52*	4.376852408	0.029742661	-0.038055304	0.067797965	0.999933887	-0.121205068	-0.394055309	0.272850241	0.011009003
ENSG00000128335	*APOL2*	6.351305992	-0.023231552	-0.040004792	0.016773239	0.999933887	-0.759563268	-1.074906867	0.315343599	0.011071997
ENSG00000130305	*NSUN5*	3.237321084	0.022182889	-0.106924196	0.129107085	0.999933887	-0.293165105	-0.671021667	0.377856562	0.011074296
ENSG00000143390	*RFX5*	4.640711344	0.00883039	0.012053425	-0.003223036	0.999933887	0.605288565	0.942140271	-0.336851706	0.011104238
ENSG00000173171	*MTX1*	3.917368937	-0.058354905	-0.059166409	0.000811504	0.999933887	-0.783360805	-1.252815606	0.469454801	0.011149479
ENSG00000170035	*UBE2E3*	3.908368844	-0.05525784	-0.020217478	-0.035040363	0.999933887	-0.295338707	-0.636020747	0.34068204	0.011149825
ENSG00000102125	*TAZ*	4.775907872	0.02224218	0.04563642	-0.023394241	0.999933887	-0.183394325	-0.469043099	0.285648774	0.011149825
ENSG00000148516	*ZEB1*	5.856201958	-0.045590037	-0.025684079	-0.019905958	0.999933887	-0.514545561	0.087885488	-0.602431048	0.011151931
ENSG00000172578	*KLHL6*	7.012129582	-0.032206794	-0.063599164	0.03139237	0.999933887	-0.175123658	0.07533749	-0.250461148	0.011153532
ENSG00000076053	*RBM7*	4.310456205	-0.00887466	0.032067019	-0.040941679	0.999933887	0.055396993	0.341338199	-0.285941207	0.011199192
ENSG00000113595	*TRIM23*	3.685239511	-0.027321072	-0.178534929	0.151213857	0.999933887	-0.338639944	0.11749439	-0.456134334	0.01121816
ENSG00000184831	*APOO*	0.156078099	0.008194999	0.120595694	-0.112400695	0.999933887	0.422874392	1.400346424	-0.977472032	0.011242017
ENSG00000084110	*HAL*	5.695709964	-0.030051023	0.019022067	-0.04907309	0.999933887	-0.50368555	-0.77137753	0.26769198	0.011255493
ENSG00000149187	*CELF1*	7.327662126	-0.060125646	0.000134557	-0.060260203	0.999933887	0.608466972	0.920380812	-0.31191384	0.011258303
ENSG00000156127	*BATF*	3.396478853	0.051996095	0.038844835	0.01315126	0.999933887	0.541433306	1.0393963	-0.497962994	0.01128075
ENSG00000115590	*IL1R2*	7.753039878	0.043217129	0.018033767	0.025183362	0.999933887	0.780421163	1.065272306	-0.284851143	0.011351218
ENSG00000150961	*SEC24D*	4.44030428	-0.01869759	-0.019608248	0.000910658	0.999933887	-0.416295045	-0.729255341	0.312960297	0.011367767
ENSG00000110330	*BIRC2*	6.057995984	-0.010533721	-0.06241607	0.051882349	0.999933887	0.673331849	1.033310622	-0.359978773	0.011376317
ENSG00000152484	*USP12*	4.168754619	-0.051422108	-0.067100269	0.015678161	0.999933887	0.452887055	0.827403671	-0.374516617	0.01140366
ENSG00000234191	*LINC01283*	-1.860567304	-0.131239876	-0.377429623	0.246189746	0.999933887	0.988421698	1.675927248	-0.68750555	0.01140366
ENSG00000263847	*AP005899.1*	0.166121991	-0.103884757	0.294984807	-0.398869564	0.999933887	0.479986905	1.286430532	-0.806443627	0.011406925
ENSG00000144711	*IQSEC1*	9.045221391	-0.003316012	-0.037473737	0.034157725	0.999933887	0.310579568	0.492637418	-0.182057851	0.011531948
ENSG00000105352	*CEACAM4*	3.518691199	0.035218863	0.003707399	0.031511465	0.999933887	-0.218814357	-0.851654138	0.632839781	0.011554888
ENSG00000139629	*GALNT6*	3.066037494	0.013376745	0.108963584	-0.095586839	0.999933887	0.202425564	0.513430819	-0.311005255	0.011556622
ENSG00000131323	*TRAF3*	7.46318198	-0.052816301	-0.045652897	-0.007163403	0.999933887	0.634785433	0.893741738	-0.258956305	0.011556807
ENSG00000272079	*AC004233.2*	1.339885517	0.019292209	0.062170771	-0.042878562	0.999933887	-1.315140047	-2.101058578	0.785918531	0.011576407
ENSG00000197632	*SERPINB2*	5.538252544	0.143361014	0.198751284	-0.05539027	0.999933887	0.877009034	1.407894885	-0.530885851	0.011709949
ENSG00000146205	*ANO7*	-2.348720182	-0.019785324	0.465266785	-0.485052109	0.999933887	-0.875806674	0.924822962	-1.800629636	0.011709949
ENSG00000172977	*KAT5*	4.726366166	0.000621441	0.03773906	-0.037117619	0.999933887	0.159889125	0.407412697	-0.247523572	0.011709949
ENSG00000075651	*PLD1*	4.008056231	-0.010642916	-0.023742894	0.013099978	0.999933887	2.862938065	3.656083757	-0.793145692	0.011722331
ENSG00000101190	*TCFL5*	3.844685828	0.117050929	0.075564003	0.041486926	0.999933887	3.325746097	4.011249057	-0.685502959	0.011722647
ENSG00000173575	*CHD2*	9.155684252	-0.087246768	-0.061866589	-0.025380178	0.999933887	0.58640516	0.909698626	-0.323293467	0.011737192
ENSG00000273812	*BX640514.2*	3.673831059	0.183881758	0.266708982	-0.082827223	0.999933887	1.345592779	1.875113966	-0.529521188	0.011750009
ENSG00000256771	*ZNF253*	0.101054558	0.127940137	-0.12304191	0.250982048	0.999933887	-0.383632841	0.299534375	-0.683167216	0.011792753
ENSG00000138413	*IDH1*	1.690641087	-0.060699693	0.031933731	-0.092633424	0.999933887	-0.376482319	-0.841612536	0.465130217	0.011797255
ENSG00000042445	*RETSAT*	3.093894669	-0.028548546	0.088169596	-0.116718142	0.999933887	-0.065055047	-0.430469624	0.365414577	0.011797255
ENSG00000008226	*DLEC1*	3.238949677	-0.089540199	0.021738152	-0.111278351	0.999933887	0.836721362	1.575353356	-0.738631994	0.011862587
ENSG00000178385	*PLEKHM3*	5.823228419	0.075183411	0.089173644	-0.013990233	0.999933887	1.496096554	1.924641339	-0.428544785	0.011887427
ENSG00000204852	*TCTN1*	2.13737168	-0.107884891	-0.019194182	-0.08869071	0.999933887	-0.692186726	-1.250062052	0.557875327	0.011909977
ENSG00000233578	*EIF4EP1*	3.168272062	-0.020807394	0.098122156	-0.11892955	0.999933887	-0.139265927	0.455845357	-0.595111284	0.011917758
ENSG00000133059	*DSTYK*	3.602250813	0.028138586	-0.006084825	0.034223411	0.999933887	-0.494435267	-0.959232194	0.464796927	0.011931489
ENSG00000151715	*TMEM45B*	2.51879103	0.023122651	0.033327269	-0.010204618	0.999933887	-0.809321269	-1.351506015	0.542184746	0.01195218
ENSG00000147894	*C9orf72*	8.228645741	-0.079204303	-0.083149461	0.003945157	0.999933887	0.215626813	0.654578968	-0.438952155	0.011961847
ENSG00000144560	*VGLL4*	2.250880804	0.05659976	0.032246939	0.024352821	0.999933887	0.062687756	0.49084828	-0.428160525	0.011961847
ENSG00000158545	*ZC3H18*	6.232387196	0.046870524	0.058222232	-0.011351708	0.999933887	0.677823455	1.057625599	-0.379802144	0.011961847
ENSG00000131503	*ANKHD1*	3.085394198	-0.082840432	-0.014066653	-0.06877378	0.999933887	-0.109060126	0.278109584	-0.387169711	0.011997124
ENSG00000130939	*UBE4B*	5.722794814	-0.013660968	0.004921279	-0.018582247	0.999933887	-0.489003254	-0.707440305	0.218437051	0.012004854
ENSG00000103152	*MPG*	2.260185302	0.162801136	0.150530253	0.012270883	0.999933887	-0.66827048	-1.296693545	0.628423064	0.012135237
ENSG00000169554	*ZEB2*	9.28070514	0.029194384	0.124775573	-0.095581189	0.999933887	1.203070252	1.55313599	-0.350065738	0.012266245
ENSG00000103510	*KAT8*	5.308247131	-0.039821947	-0.020185569	-0.019636379	0.999933887	-0.230297527	-0.481281858	0.250984331	0.012276005
ENSG00000241886	*AC112496.1*	3.520233279	-0.110503506	-0.034189323	-0.076314184	0.999933887	0.067392579	0.580081475	-0.512688896	0.012282594
ENSG00000105221	*AKT2*	6.423437025	0.059159866	0.024092124	0.035067742	0.999933887	-0.199226542	-0.411609669	0.212383127	0.012297505
ENSG00000116017	*ARID3A*	5.930210051	0.091386017	0.096936953	-0.005550936	0.999933887	0.558969554	0.854016172	-0.295046618	0.012309628
ENSG00000147533	*GOLGA7*	4.881614707	0.080935032	-0.017521754	0.098456786	0.999933887	0.908377052	1.240924794	-0.332547743	0.01231622
ENSG00000107897	*ACBD5*	3.387071544	0.010718137	0.039105214	-0.028387077	0.999933887	-0.486648973	-0.885015981	0.398367008	0.012355476
ENSG00000166938	*DIS3L*	2.059533307	-0.042643307	0.063471157	-0.106114465	0.999933887	0.054856683	0.417181952	-0.362325269	0.01243997
ENSG00000172366	*MCRIP2*	3.532468191	0.071251005	0.130110436	-0.058859431	0.999933887	1.578141102	2.113796327	-0.535655225	0.012448108
ENSG00000165219	*GAPVD1*	5.304796898	0.01115629	-0.07973968	0.090895969	0.999933887	-0.396204603	-0.708157437	0.311952834	0.012453929
ENSG00000141232	*TOB1*	4.91367584	0.088243049	0.166141401	-0.077898352	0.999933887	-0.186265208	-0.478733031	0.292467824	0.012499695
ENSG00000144635	*DYNC1LI1*	5.232149402	-0.03399081	0.042004799	-0.075995609	0.999933887	-0.829334255	-1.113533274	0.284199019	0.012583477
ENSG00000139436	*GIT2*	5.338432749	-0.060501593	-0.058751343	-0.00175025	0.999933887	-0.335012682	-0.562582187	0.227569505	0.012589684
ENSG00000215644	*GCGR*	-2.711519817	0.192334257	-0.6118585	0.804192756	0.999933887	3.756058346	5.510160314	-1.754101968	0.012589684
ENSG00000125457	*MIF4GD*	2.418931233	-0.121716603	0.084437411	-0.206154014	0.999933887	-0.171402944	-0.617052548	0.445649604	0.012634613
ENSG00000076928	*ARHGEF1*	8.675629157	0.045045061	0.066361138	-0.021316077	0.999933887	-0.116954625	-0.372541575	0.25558695	0.012634613
ENSG00000001084	*GCLC*	4.639552566	-0.055968176	-0.01418645	-0.041781726	0.999933887	0.936289066	1.424820087	-0.488531021	0.012634613
ENSG00000247853	*AC006064.2*	0.349552401	0.131784787	0.006005442	0.125779345	0.999933887	0.042480658	0.909395985	-0.866915327	0.012643189
ENSG00000154359	*LONRF1*	5.709431304	0.091855171	0.102907852	-0.01105268	0.999933887	0.129105435	-0.210379564	0.339484999	0.012644379
ENSG00000153201	*RANBP2*	8.240332071	-0.197514877	-0.149126969	-0.048387908	0.999933887	0.358045071	1.132569633	-0.774524562	0.012644379
ENSG00000114302	*PRKAR2A*	4.164728642	-0.057249133	-0.000882371	-0.056366762	0.999933887	-0.189111892	-0.516971604	0.327859711	0.012644379
ENSG00000204397	*CARD16*	6.968261626	0.060163279	-0.033339035	0.093502314	0.999933887	1.079579167	1.416385788	-0.33680662	0.012644379
ENSG00000182541	*LIMK2*	9.839064238	0.022723855	-0.005119695	0.027843551	0.999933887	1.449915822	1.717432614	-0.267516792	0.012644379
ENSG00000204308	*RNF5*	3.082459261	-0.01645846	0.046878031	-0.063336491	0.999933887	-0.042228247	-0.453744212	0.411515965	0.012644379
ENSG00000099968	*BCL2L13*	4.366125375	0.022047595	0.05758192	-0.035534325	0.999933887	0.360947505	0.620755781	-0.259808276	0.012654086
ENSG00000059915	*PSD*	2.435739418	0.245408713	0.299338864	-0.053930151	0.999933887	1.730772705	2.306519998	-0.575747293	0.012663439
ENSG00000188786	*MTF1*	7.053393435	-0.055756272	-0.032230066	-0.023526206	0.999933887	2.209454543	2.681010067	-0.471555524	0.012663439
ENSG00000196396	*PTPN1*	6.106365671	-0.007997898	-0.017392393	0.009394495	0.999933887	0.377877589	0.714916587	-0.337038998	0.012663439
ENSG00000007168	*PAFAH1B1*	7.433795082	-0.043818491	-0.031120466	-0.012698026	0.999933887	0.364209187	0.590393965	-0.226184778	0.012750032

ENSG00000105825	*TFPI2*	-1.826989997	-0.198870711	0.081314654	-0.280185365	0.999933887	0.815095839	1.743640737	-0.928544898	0.012831371
ENSG00000116525	*TRIM62*	3.631261264	0.141556324	0.080426244	0.06113008	0.999933887	-0.316363662	-0.664986702	0.34862304	0.012900138
ENSG00000186431	*FCAR*	8.125259563	0.157449278	0.136168643	0.021280635	0.999933887	1.82801463	2.226175811	-0.398161181	0.012900138
ENSG00000185112	*FAM43A*	1.714431669	-0.061971234	-0.05237038	-0.009600854	0.999933887	-0.069812518	-0.544380571	0.474568053	0.012919341
ENSG00000110436	*SLC1A2*	-0.610501627	0.025844316	0.469128589	-0.443284273	0.999933887	0.498128198	1.6035353	-1.105407102	0.013040327
ENSG00000148356	*LRSAM1*	3.905584776	-0.026697711	0.10499416	-0.131691871	0.999933887	-0.13452494	-0.485645377	0.351120437	0.013050847
ENSG00000225422	*RBMS1P1*	1.967570959	-0.109990479	-0.176211863	0.066221384	0.999933887	1.518252441	2.191621258	-0.673368818	0.013073263
ENSG00000151689	*INPP1*	2.618727644	-0.053628484	-0.063140363	0.009511879	0.999933887	-0.06118912	0.29969403	-0.36088315	0.013119632
ENSG00000143149	*ALDH9A1*	3.858763117	-0.129485151	0.072154846	-0.201639996	0.969206975	-0.390335497	-0.688606519	0.298271021	0.013241847
ENSG00000111802	*TDP2*	8.224673971	-0.034839047	-0.101299432	0.066460384	0.999933887	0.06272639	0.302388638	-0.239662247	0.013248345
ENSG00000163519	*TRAT1*	3.600766461	-0.05711455	-0.156843984	0.099729434	0.999933887	-0.017975896	0.441237722	-0.459213619	0.013248345
ENSG00000139190	*VAMP1*	4.865581627	0.012092209	-0.03882217	0.050914379	0.999933887	-0.141649874	-0.439095165	0.297445291	0.013254284
ENSG00000010361	*FUZ*	3.109482473	0.059110494	-0.053564727	0.112675221	0.999933887	-0.290151694	-0.717195444	0.427043749	0.013510771
ENSG00000171467	*ZNF318*	5.448708698	0.019378325	-0.104715805	0.12409413	0.999933887	0.358033471	0.646776074	-0.288742603	0.013534104
ENSG00000205208	*C4orf46*	2.301216159	-0.12156041	-0.103145916	-0.018414494	0.999933887	-0.051112871	0.376669152	-0.427782023	0.013555936
ENSG00000176101	*SSNA1*	4.178158686	0.111742571	0.080319349	0.031423223	0.999933887	-0.233473815	-0.607981136	0.374507321	0.013555936
ENSG00000168528	*SERINC2*	1.185925047	-0.015113147	-0.123136798	0.108023651	0.999933887	-0.377488068	0.017266953	-0.394755021	0.013559372
ENSG00000108798	*ABI3*	4.28440785	0.042817407	0.039928519	0.002888888	0.999933887	-0.211565507	-0.5397714	0.328205892	0.013563059
ENSG00000165997	*ARL5B*	7.614909991	0.11578085	-0.036761365	0.152542215	0.999933887	1.620074593	2.219936846	-0.599862253	0.013574174
ENSG00000203644	*AC083799.1*	4.886542407	-0.088546309	-0.110623317	0.022077008	0.999933887	-0.043355902	0.238575944	-0.281931845	0.013575728
ENSG00000214194	*SMIM30*	0.008631567	-0.047772845	-0.278872805	0.23109996	0.999933887	-0.003152548	0.855045822	-0.85819837	0.013660005
ENSG00000173846	*PLK3*	8.676020753	0.187103647	0.094618976	0.092484672	0.999933887	1.84232184	2.268848973	-0.426527134	0.013666711
ENSG00000183513	*COA5*	3.080959882	-0.023887789	-0.230872137	0.206984348	0.999933887	-0.295419809	-0.608113333	0.312693524	0.013716153
ENSG00000101266	*CSNK2A1*	4.22192711	-0.055050817	0.000551998	-0.055602815	0.999933887	-0.2914478	-0.574945141	0.283497341	0.013770704
ENSG00000138495	*COX17*	3.798060225	-0.008113505	-0.033528736	0.025415232	0.999933887	0.671133903	0.936164405	-0.265030502	0.013770704
ENSG00000166716	*ZNF592*	6.431577473	-0.004139076	-0.002162699	-0.001976377	0.999933887	-0.548080853	-0.784711919	0.236631066	0.013770704
ENSG00000149679	*CABLES2*	3.479721356	0.028012312	0.022101308	0.005911004	0.999933887	0.504382016	0.862741485	-0.358359468	0.013851156
ENSG00000142347	*MYO1F*	10.339761823	-0.011919759	0.020869143	-0.032788902	0.999933887	-0.732455661	-1.048125121	0.31566946	0.013891473
ENSG00000091972	*CD200*	-0.025862591	0.212285212	-0.415687584	0.627972796	0.999933887	1.907100073	2.744887274	-0.837787201	0.013933931
ENSG00000114904	*NEK4*	1.560331549	0.030464031	-0.07362812	0.104092151	0.999933887	0.279077899	0.763116694	-0.484038795	0.013933931
ENSG00000077150	*NFKB2*	9.661266513	0.0569005	0.119924853	-0.063024353	0.999933887	2.142827091	2.524923529	-0.382096438	0.013938749
ENSG00000100439	*ABHD4*	6.294281111	-0.005065012	-0.018673714	0.013608702	0.999933887	0.305518615	0.557029806	-0.251511191	0.014001786
ENSG00000090863	*GLG1*	6.171215998	-0.022471069	-0.060152466	0.037681397	0.999933887	-0.181495332	-0.406223598	0.224728266	0.014039157
ENSG00000105835	*NAMPT*	12.55791678	-0.012940281	-0.070751979	0.057811699	0.999933887	0.352708477	0.658821444	-0.306112967	0.014085244
ENSG00000099942	*CRKL*	5.746057521	-0.118666986	-0.032895113	-0.085771872	0.999933887	-0.512913172	-0.790977943	0.278064771	0.014088054
ENSG00000164609	*SLU7*	6.647631096	-0.061002597	-0.095386246	0.034383649	0.999933887	0.004973742	0.29442794	-0.289454198	0.014088054
ENSG00000155961	*RAB39B*	2.91797255	-0.041521502	-0.09986885	0.058347348	0.999933887	0.407896818	0.834561771	-0.426664954	0.014156326
ENSG00000135679	*MDM2*	6.095659033	-0.024178395	0.046473336	-0.070651731	0.999933887	0.269246703	0.617810098	-0.348563395	0.014164296
ENSG00000160013	*PTGIR*	0.184208693	0.056070289	-0.236905926	0.292976215	0.999933887	2.112590223	3.206958746	-1.094368523	0.014167611
ENSG00000134452	*FBH1*	4.187715419	0.02505346	0.024787722	0.000265737	0.999933887	-0.118455227	-0.401722522	0.283267295	0.014219225
ENSG00000178028	*DMAP1*	3.971919006	-0.073080324	-0.007018206	-0.066062119	0.999933887	0.066433665	-0.177568112	0.244001778	0.014262378
ENSG00000136048	*DRAM1*	5.486728626	-0.007692011	-0.063379066	0.055687054	0.999933887	1.825290721	2.347123607	-0.521832886	0.014262378
ENSG00000161204	*ABCF3*	4.539952492	-0.043404046	0.012957997	-0.056362044	0.999933887	-0.05849678	-0.37330926	0.314812479	0.014306387
ENSG00000106628	*POLD2*	2.309096681	0.055568851	-0.070504216	0.126073067	0.999933887	0.354669295	0.814513982	-0.459844687	0.014306387
ENSG00000118496	*FBXO30*	4.373865764	-0.123343248	-0.081851281	-0.041491967	0.999933887	0.131062452	0.468501807	-0.337439355	0.014361194
ENSG00000229337	*AC079305.2*	2.502359385	0.247419817	0.00685206	0.240567757	0.999933887	0.815790147	0.175588664	0.640201483	0.014365099
ENSG00000185043	*CIB1*	5.70124605	0.009008074	0.017635034	-0.00862696	0.999933887	-0.082936328	-0.297298598	0.21436227	0.014395085
ENSG00000196502	*SULT1A1*	5.791296186	-0.112840811	-0.059684733	-0.053156078	0.999933887	-1.124476633	-1.471605528	0.347128895	0.014402877
ENSG00000111186	*WNT5B*	-1.764529404	0.374535542	0.183505859	0.191029683	0.999933887	2.560481492	4.272027757	-1.711546265	0.014402877
ENSG00000218565	*AL592429.1*	-1.178158512	-0.091449694	0.474436729	-0.565886424	0.999933887	1.465527278	2.556874894	-1.091347616	0.014402877
ENSG00000184384	*MAML2*	6.886173578	0.041396872	0.081048547	-0.039651675	0.999933887	0.689266616	0.983666194	-0.294399577	0.014413097
ENSG00000109381	*ELF2*	6.122993817	-0.021792107	-0.053518585	0.031726478	0.999933887	0.474902619	0.771583961	-0.296681342	0.014425974
ENSG00000161267	*BDH1*	1.128901392	0.021378715	0.099330027	-0.077951312	0.999933887	0.079632253	0.579335273	-0.49970302	0.014459402
ENSG00000118985	*ELL2*	6.098135726	-0.022700552	-0.030158119	0.007457567	0.999933887	0.537534687	0.848326162	-0.310791475	0.014513967
ENSG00000164332	*UBLCP1*	4.992347883	-0.079690337	-0.148260745	0.068570408	0.999933887	-0.07444164	0.467770122	-0.542211762	0.014548207
ENSG00000116266	*STXBP3*	5.500003717	-0.072745559	-0.117450856	0.044705297	0.999933887	-0.177978362	0.219159316	-0.397137678	0.014563595
ENSG00000169299	*PGM2*	3.35978018	-0.052553489	-0.08827763	0.035724141	0.999933887	-0.117854231	-0.556121739	0.438267507	0.014611988
ENSG00000242732	*RTL5*	1.716497299	-0.100216677	0.260746142	-0.360962819	0.944094537	-0.221982354	-0.918634462	0.696652108	0.014636942
ENSG00000141458	*NPC1*	6.851303176	-0.001284045	-0.024381072	0.023097027	0.999933887	0.329886351	0.560811495	-0.230925143	0.014636942
ENSG00000183495	*EP400*	5.300325502	0.042002401	-0.001438515	0.043440916	0.999933887	-0.078888422	-0.299883096	0.220994674	0.014663879
ENSG00000168286	*THAP11*	2.729223279	0.009633889	0.034010145	-0.024376256	0.999933887	-0.042043874	-0.439847027	0.397803154	0.014665368
ENSG00000180773	*SLC36A4*	4.082084686	-0.019563208	0.013377082	-0.03294029	0.999933887	0.431054067	0.862179146	-0.431125079	0.014747037
ENSG00000075413	*MARK3*	6.773547546	-0.006157124	0.020864359	-0.027021483	0.999933887	0.205003126	0.397912638	-0.192909512	0.014865658
ENSG00000134627	*PIWIL4*	2.549170873	0.00346155	-0.096042674	0.099504224	0.999933887	1.13534203	1.566389885	-0.431047855	0.014865658
ENSG00000138092	*CENPO*	1.132166257	-0.000129372	0.161600734	-0.161730106	0.999933887	0.834334737	1.406881487	-0.57254675	0.014865658
ENSG00000173621	*LRFN4*	0.091436031	0.090888531	0.060937401	0.02995113	0.999933887	-0.491221959	-1.249819434	0.758597475	0.014909641
ENSG00000148296	*SURF6*	3.672569983	0.021804498	0.100119749	-0.078315251	0.999933887	-0.085413446	-0.48727741	0.401863964	0.014973278
ENSG00000011007	*ELOA*	3.578408208	-0.020624119	-0.100218615	0.079594497	0.999933887	0.222571127	0.485562374	-0.262991246	0.014990028
ENSG00000185745	*IFIT1*	5.486816474	-0.15952655	-0.231432483	0.071905933	0.999933887	-1.137975363	-1.675818018	0.537842655	0.014996076
ENSG00000133884	*DPF2*	5.089303583	-0.079802132	0.011581994	-0.091384126	0.999933887	-0.428248725	-0.724012848	0.295764123	0.015016873
ENSG00000133063	*CHIT1*	2.89009541	0.028179297	0.07187853	-0.043699233	0.999933887	0.831752175	1.241969428	-0.410217252	0.015016873
ENSG00000105855	*ITGB8*	-0.208703392	-0.031175201	0.316308357	-0.347483558	0.999933887	2.034246776	2.850108641	-0.815861866	0.015016873
ENSG00000087302	*RTRAF*	4.355835822	0.021748919	-0.092372072	0.114120991	0.999933887	1.005803963	1.45437842	-0.448574457	0.01504698
ENSG00000269836	*AC020913.3*	-0.08573025	-0.014705484	0.098827715	-0.1135332	0.999933887	-0.592143895	0.126811654	-0.718955549	0.01504698
ENSG00000090621	*PABPC4*	5.194573098	0.049420982	0.030170125	0.019250858	0.999933887	0.215479159	0.439545485	-0.224066326	0.015049948
ENSG00000108883	*EFTUD2*	4.609530947	-0.031260013	-0.093238315	0.061978302	0.999933887	-0.088424579	-0.320028779	0.2316042	0.015049948
ENSG00000196812	*ZSCAN16*	2.557499248	-0.179131877	-0.215776902	0.036645024	0.999933887	-0.134789146	0.357249398	-0.492038544	0.01510437
ENSG00000171608	*PIK3CD*	8.374602765	0.024684011	0.034115873	-0.009431862	0.999933887	-0.091795383	-0.35545425	0.263658868	0.01510437
ENSG00000126456	*IRF3*	4.801855358	0.054777255	0.130915265	-0.07613801	0.999933887	-0.050310525	-0.421933064	0.37162254	0.01525338
ENSG00000136250	*AOAH*	5.74365993	-0.020439828	-0.039907651	0.019467823	0.999933887	0.205889066	0.490670039	-0.284780972	0.01525338
ENSG00000186111	*PIP5K1C*	4.535792988	0.047539498	0.043480772	0.004058726	0.999933887	-0.383720364	-0.682972058	0.299251694	0.015273117
ENSG00000110876	*SELPLG*	8.413014359	0.029160618	-0.05473248	0.083893099	0.999933887	-1.523520912	-2.018286703	0.494765791	0.015273117
ENSG00000145214	*DGKQ*	4.375252855	0.071434371	0.082666874	-0.011232502	0.999933887	0.035427425	-0.358423992	0.393851417	0.015274048
ENSG00000129933	*MAU2*	6.79062712	0.023920879	0.009521405	0.014399475	0.999933887	-0.488631542	-0.726837025	0.238205483	0.015282567
ENSG00000164929	*BAALC*	-0.910292163	-0.715765615	-0.158072801	-0.557692814	0.999933887	1.523038148	2.249406651	-0.726368503	0.015292463
ENSG00000225572	*DOCK4-AS1*	1.689672934	0.113224996	0.363311404	-0.250086409	0.999933887	0.496251091	1.033179091	-0.536928001	0.015292463
ENSG00000101421	*CHMP4B*	8.709476274	-0.030208547	-0.018669829	-0.011538718	0.999933887	0.69271367	0.939915688	-0.247202018	0.015302451
ENSG00000126945	*HNRNPH2*	7.65538199	-0.08006137	-0.08879204	0.00873067	0.999933887	0.299526404	0.525307544	-0.22578114	0.015305919
ENSG00000111261	*MANSC1*	3.731976424	-0.021175088	-0.047782463	0.026607375	0.999933887	-0.591810814	-1.067733108	0.475922294	0.015333609
ENSG00000137462	*TLR2*	9.797260779	-0.079425299	-0.129966127	0.050540828	0.999933887	0.346705065	0.755677563	-0.408972498	0.015383854
ENSG00000271821	*AL662844.3*	-0.986104636	-0.418844981	-0.422675195	0.003830214	0.999933887	-1.317887596	-2.968712084	1.650824488	0.015450215
ENSG00000185813	*PCYT2*	3.807973297	0.115990165	0.160557858	-0.044567692	0.999933887	0.615791382	0.992185422	-0.376394039	0.015494584
ENSG00000197457	*STMN3*	3.518820389	0.013836933	0.025893147	-0.012056214	0.999933887	0.06250467	-0.274299538	0.336804208	0.015494584
ENSG00000052795	*FNIP2*	6.622923636	-0.09038131	0.063127703	-0.153509013	0.999933887	-0.152057477	0.1421583	-0.294215776	0.015571179
ENSG00000105499	*PLA2G4C*	1.4309254	-0.078510991	-0.198338659	0.119827668	0.999933887	0.943603093	1.422081948	-0.478478855	0.015571179
ENSG00000211450	*SELENOH*	1.03792792	-0.111210765	-0.119017681	0.007806916	0.999933887	-0.054445233	-0.493167422	0.43872219	0.015634624
ENSG00000078237	*TIGAR*	0.940634458	-0.064135974	0.143519583	-0.207655557	0.999933887	-0.386742987	-1.136095364	0.749352377	0.015637747
ENSG00000105576	*TNPO2*	5.083962922	0.025122471	0.036794733	-0.011672262	0.999933887	0.663565947	0.978370677	-0.31480473	0.015637747
ENSG00000174405	*LIG4*	4.738603718	-0.132017511	-0.226316597	0.094299087	0.999933887	0.073090033	0.684315988	-0.611225955	0.015677493
ENSG00000255398	*HCAR3*	11.072643251	0.114728099	0.049456042	0.065272057	0.999933887	0.706859952	1.032292916	-0.325432964	0.015692258
ENSG00000125637	*PSD4*	7.162385062	0.017198438	-0.000113646	0.017312083	0.999933887	0.103068846	-0.186290863	0.28935971	0.015705526
ENSG00000167670	*CHAF1A*	2.961129301	0.045573172	-0.046228168	0.09180134	0.999933887	0.728069926	1.128767217	-0.400697291	0.015748188
ENSG00000005302	*MSL3*	6.199370397	-0.045302236	-0.063926472	0.018624236	0.999933887	-0.049824417	-0.250027899	0.200203482	0.015851672
ENSG00000151092	*NGLY1*	4.666161151	0.043310463	0.017374281	0.025936182	0.999933887	-0.65570063	-1.038644909	0.382944279	0.015851672
ENSG00000182768	*NGRN*	2.024485534	0.03826814	-0.164851399	0.20311954	0.999933887	-0.031751526	-0.439731685	0.407980159	0.015851672

ENSG00000115828	*QPCT*	6.840130742	0.014180721	-0.043042696	0.057223417	0.999933887	0.121890955	0.513026845	-0.391135891	0.015851672
ENSG00000135519	*KCNH3*	3.203373382	0.080351999	0.009527872	0.070824126	0.999933887	-0.151962922	-0.622121734	0.470158812	0.015915181
ENSG00000119922	*IFIT2*	8.328032815	-0.196746265	-0.197820648	0.001074383	0.999933887	-1.368412893	-1.863504737	0.495091844	0.015989053
ENSG00000124107	*SLPI*	6.813368997	-0.028954057	-0.034994363	0.006040306	0.999933887	0.983239957	1.339382842	-0.356142886	0.015989053
ENSG00000126391	*FRMD8*	6.331794645	-0.014128532	-0.031278923	0.01715039	0.999933887	-0.594312079	-0.847964957	0.253652879	0.015989053
ENSG00000155363	*MOV10*	4.915073189	0.061468894	0.052946739	0.008522154	0.999933887	-0.001514948	-0.281975899	0.280460951	0.016008536
ENSG00000105612	*DNASE2*	4.662103394	-0.112064597	-0.033335094	-0.078729503	0.999933887	-1.416570992	-1.837733544	0.421162552	0.016012638
ENSG00000110696	*C11orf58*	5.749405505	-0.107889139	-0.084057877	-0.023831262	0.999933887	-0.018184571	0.179593712	-0.197778283	0.016013602
ENSG00000174165	*ZDHHC24*	2.648838928	0.075005597	0.071588671	0.003416926	0.999933887	-0.051483155	-0.441920734	0.390437579	0.016013602
ENSG00000125753	*VASP*	10.04952972	0.044570694	0.050189151	-0.005618457	0.999933887	0.459158402	0.725919663	-0.26676126	0.016013602
ENSG00000124151	*NCOA3*	6.082704409	0.033001457	-0.011041119	0.044042575	0.999933887	-0.509322951	-0.760087628	0.250764677	0.016013602
ENSG00000145868	*FBXO38*	5.894349417	-0.03782571	-0.050351197	0.012525487	0.999933887	0.235534032	0.533319114	-0.297785083	0.016013602
ENSG00000072518	*MARK2*	6.943436178	0.030518088	0.085191611	-0.054673523	0.999933887	-0.422675553	-0.69194266	0.269267107	0.016066721
ENSG00000151304	*SRFBP1*	0.003955739	-0.049500459	0.108101361	-0.15760182	0.999933887	-0.076491916	0.748828255	-0.825320171	0.016156499
ENSG00000173638	*SLC19A1*	6.737395798	0.069371539	0.015028005	0.054343533	0.999933887	-0.755512772	-1.096506008	0.340993236	0.016156612
ENSG00000259354	*AC025580.2*	2.960912065	0.124413873	0.099786014	0.024627859	0.999933887	2.395114657	3.022808836	-0.627694179	0.016156612
ENSG00000188419	*CHM*	1.829254738	0.072001805	-0.095278522	0.167280327	0.999933887	-0.014676482	0.560406618	-0.5750831	0.016156612
ENSG00000107104	*KANK1*	1.07576382	-0.099505923	-0.046004811	-0.053501112	0.999933887	1.205516245	2.004090324	-0.798574078	0.016156612
ENSG00000163162	*RNF149*	8.929363348	0.019836431	0.040636421	-0.020799991	0.999933887	-0.554935669	-0.782577434	0.227641765	0.016156612
ENSG00000161791	*FMNL3*	5.105190266	0.044147563	-0.057340276	0.101487839	0.999933887	2.35342265	2.965421585	-0.611998934	0.016156612
ENSG00000125734	*GPR108*	7.261163676	-0.003924045	0.000686722	-0.004610766	0.999933887	1.471987199	1.861429118	-0.389441919	0.016156612
ENSG00000257923	*CUX1*	5.905766741	-0.001754593	-0.012852926	0.011098333	0.999933887	0.193382697	0.424406137	-0.231023439	0.016156612
ENSG00000225828	*FAM229A*	2.4399994	0.026082457	0.055627329	-0.029544872	0.999933887	-0.015288881	-0.417227233	0.401938352	0.016237211
ENSG00000106009	*BRAT1*	5.861094439	0.004256916	0.070492218	-0.066235302	0.999933887	-0.468171609	-0.809824665	0.341653056	0.016237211
ENSG00000165879	*FRAT1*	5.450188905	-0.140478018	-0.254616366	0.114138348	0.999933887	-1.371269236	-1.89390903	0.522639794	0.01627742
ENSG00000182054	*IDH2*	4.695546007	-0.041028251	0.012529639	-0.05355789	0.999933887	-0.187284705	-0.411879515	0.22459481	0.01627742
ENSG00000113448	*PDE4D*	4.836060579	0.020903217	-0.111704883	0.1326081	0.999933887	-0.233968444	0.155372849	-0.389341293	0.01627742
ENSG00000059728	*MXD1*	10.981261541	-0.000416339	0.016621724	-0.017038063	0.999933887	0.508145395	0.7098321	-0.201686705	0.016342314
ENSG00000122482	*ZNF644*	4.248658072	-0.048165372	-0.124529102	0.07636373	0.999933887	0.197954536	0.822675753	-0.624721217	0.016395869
ENSG00000178537	*SLC25A20*	1.854661903	0.037109463	-0.012096124	0.049205586	0.999933887	-0.090795239	-0.567765795	0.476970556	0.016423264
ENSG00000184207	*PGP*	2.888588982	0.162993421	0.040087041	0.12290638	0.999933887	-0.115288938	-0.573950823	0.458661884	0.016445691
ENSG00000145365	*TIFA*	3.621265918	0.079263216	0.026075106	0.05318811	0.999933887	1.970353794	2.546078695	-0.575724902	0.016475589
ENSG00000166619	*BLCAP*	4.422926105	0.002151323	-0.00855692	0.010708243	0.999933887	-0.266515493	-0.5187875	0.252272007	0.016475589
ENSG00000090487	*SPG21*	6.88275736	-0.031637023	-0.038834618	0.007197595	0.999933887	0.333704091	0.514903269	-0.181199178	0.016570464
ENSG00000001617	*SEMA3F*	-0.173194469	0.201201913	0.003480034	0.19772188	0.999933887	-0.108484604	0.659965718	-0.768450322	0.016570464
ENSG00000064201	*TSPAN32*	4.239712826	0.044326904	0.057071902	-0.012744998	0.999933887	-0.150884414	-0.480377953	0.329493539	0.016570464
ENSG00000125510	*OPRL1*	0.168755098	0.010442415	0.26458645	-0.254144035	0.999933887	-0.881322709	-1.825348711	0.944026002	0.016570464
ENSG00000237576	*LINC01888*	2.605887108	0.091985328	0.000230014	0.091755314	0.999933887	0.958816549	1.378621278	-0.419804729	0.016575242
ENSG00000133961	*NUMB*	8.53167444	0.00194292	-0.029724439	0.031667359	0.999933887	0.470019789	0.677308987	-0.207289198	0.01673452
ENSG00000198554	*WDHD1*	-0.661910829	-0.139569474	-0.225340589	0.085771114	0.999933887	-0.324777012	0.385015325	-0.709792337	0.016786919
ENSG00000175643	*RMI2*	1.948856457	-0.038255995	-0.190172232	0.151916236	0.999933887	0.656975492	1.155920742	-0.498945249	0.016822687
ENSG00000130830	*MPP1*	8.812309549	-0.078402632	-0.083795004	0.005392372	0.999933887	-0.118565628	0.117058417	-0.235624045	0.016829965
ENSG00000126804	*ZBTB1*	6.172508566	-0.050980696	-0.095836325	0.044855629	0.999933887	0.022874045	0.338783135	-0.315909089	0.016851768
ENSG00000197930	*ERO1A*	5.199945674	-0.017416136	-0.039981362	0.022565226	0.999933887	-0.424933454	-0.693356035	0.268422581	0.016904787
ENSG00000149182	*ARFGAP2*	5.528844315	-0.015513088	-0.027389125	0.011876037	0.999933887	-0.169587082	-0.359192929	0.189605846	0.016918715
ENSG00000063978	*RNF4*	5.574145689	-0.016257166	-0.034002243	0.017745077	0.999933887	-0.36239631	-0.594772221	0.232375911	0.016918715
ENSG00000214253	*FIS1*	4.376267212	0.02662835	0.001440691	0.02518766	0.999933887	-0.077098246	-0.354709493	0.277611246	0.016931578
ENSG00000103591	*AAGAB*	3.745645738	-0.055794411	-0.110008666	0.054214255	0.999933887	0.38656415	0.70485943	-0.31829528	0.016933643
ENSG00000117713	*ARID1A*	7.170482015	0.008019646	0.052705716	-0.04468607	0.999933887	-0.274785511	-0.518151416	0.243365905	0.016933643
ENSG00000165006	*UBAP1*	7.781948812	-0.012110561	-0.069685313	0.057574752	0.999933887	0.290591212	0.503935565	-0.213344353	0.016964
ENSG00000167967	*E4F1*	4.805199585	0.085181787	0.092829385	-0.007647598	0.999933887	-0.179818919	-0.514802009	0.334983091	0.016972079
ENSG00000224597	*SVIL-AS1*	1.70998374	0.003028045	-0.118747502	0.121775547	0.999933887	0.105964687	0.622290856	-0.516326169	0.016972079
ENSG00000111490	*TBC1D30*	1.87205188	-0.012105418	-0.14981231	0.137706892	0.999933887	1.050055357	1.501257733	-0.451202376	0.017072827
ENSG00000157227	*MMP14*	1.862174206	0.097212361	0.079961439	0.017250922	0.999933887	2.2346455	3.017633171	-0.782987671	0.017124722
ENSG00000122882	*ECD*	4.671038551	-0.001766268	-0.112570655	0.110804387	0.999933887	0.253211017	0.492171546	-0.23896053	0.017169896
ENSG00000161921	*CXCL16*	9.6272892	0.042644582	0.048879361	-0.006234779	0.999933887	-0.228239394	0.064873768	-0.293113162	0.017376857
ENSG00000171503	*ETFDH*	2.364607084	-0.047931326	0.016445283	-0.064376609	0.999933887	-0.150254984	0.238246953	-0.388501937	0.017393786
ENSG00000196189	*SEMA4A*	6.862010081	0.018567925	-0.019950122	0.038518047	0.999933887	0.799364886	1.187636678	-0.388271792	0.017393786
ENSG00000269293	*ZSCAN16-AS1*	1.14159728	-0.134611632	-0.302356866	0.167745234	0.999933887	0.533580464	1.026241622	-0.492661158	0.017401471
ENSG00000255443	*CD44-AS1*	3.248959645	-0.061606083	0.055920638	-0.11752672	0.999933887	1.535030452	2.164282915	-0.629252463	0.017401471
ENSG00000126822	*PLEKHG3*	7.404829493	0.001353658	0.090749738	-0.08939608	0.999933887	-0.396495094	-0.70787563	0.311380536	0.017401471
ENSG00000172575	*RASGRP1*	4.515447625	0.001058812	-0.054238212	0.055297024	0.999933887	0.098281327	0.441789741	-0.343508415	0.017401471
ENSG00000153551	*CMTM7*	3.876838142	0.069963296	0.116582498	-0.046619202	0.999933887	-0.271184947	-0.679435879	0.408250931	0.017431346
ENSG00000160877	*NACC1*	4.529563634	0.023301524	-0.009608228	0.032909753	0.999933887	-0.543444317	-0.856592906	0.31314859	0.017433638
ENSG00000172273	*HINFP*	2.717924283	-0.068306521	-0.035797478	-0.032509043	0.999933887	-0.2501128	-0.645782191	0.395669391	0.017467316
ENSG00000281420	*AP001052.1*	-0.453497025	0.052120443	0.177935291	-0.125814849	0.999933887	1.239290162	2.096161941	-0.856871779	0.017500888
ENSG00000122008	*POLK*	2.434520606	-0.115156035	-0.261966835	0.1468108	0.999933887	-0.205110224	0.514134463	-0.719244687	0.017577709
ENSG00000154978	*VOPP1*	6.858034054	-0.040094085	-0.053341108	0.013247023	0.999933887	0.270587053	0.582389862	-0.311802809	0.01765558
ENSG00000175029	*CTBP2*	4.996736822	-0.011806026	-0.00616824	-0.005637786	0.999933887	-0.416636703	-0.693509083	0.27687238	0.017685912
ENSG00000172939	*OXSR1*	7.87765093	-0.031916936	-0.0516001	0.019683164	0.999933887	0.189895921	0.456424442	-0.266528521	0.017696496
ENSG00000155097	*ATP6V1C1*	7.205479725	-0.081636388	-0.014927913	-0.066708475	0.999933887	0.372425954	0.660704255	-0.288278301	0.017717231
ENSG00000128563	*PRKRIP1*	4.097600251	-0.014884517	0.017426728	-0.032311245	0.999933887	-0.113923448	-0.403689236	0.289765788	0.017717231
ENSG00000156110	*ADK*	2.582987346	-0.005675743	-0.124096954	0.11842121	0.999933887	-0.385282968	-0.675858379	0.290575411	0.017738962
ENSG00000188820	*CALHM6*	2.124001631	0.16216525	0.110056527	0.052108723	0.999933887	-1.151540648	-2.251474981	1.099934333	0.017758889
ENSG00000137171	*KLC4*	3.145504221	-0.056033527	0.050499838	-0.106533365	0.999933887	-0.32509515	-0.664873351	0.339778202	0.017772396
ENSG00000136816	*TOR1B*	5.135532336	-0.067266225	-0.100347618	0.033081393	0.999933887	-0.638563714	-0.930785155	0.29222144	0.017810927
ENSG00000162613	*FUBP1*	5.981012589	-0.039026643	-0.054864539	0.015837896	0.999933887	-0.11744795	0.136777776	-0.254225726	0.01782574
ENSG00000143157	*POGK*	4.120039319	-0.027601324	-0.074709917	0.047108594	0.999933887	0.507917453	0.818211639	-0.310294186	0.01782574
ENSG00000078699	*CBFA2T2*	4.067294005	-0.019212497	-0.001148747	-0.01806375	0.999933887	-0.45719375	-0.762912163	0.305718413	0.01782574
ENSG00000285639	*AL133259.1*	1.824221931	0.010297942	0.269661259	-0.259363317	0.999933887	0.69138229	1.328502344	-0.637120054	0.01782574
ENSG00000278090	*LUNAR1*	0.35169648	-0.167373807	-0.209354704	0.041980896	0.999933887	-1.24497685	-1.966716365	0.721739515	0.017857807
ENSG00000068120	*COASY*	4.264885701	-0.115130216	-0.15098125	0.035851034	0.999933887	-0.357051961	-0.638278065	0.281226104	0.017879039
ENSG00000107551	*RASSF4*	1.080527406	-0.031720701	0.006091444	-0.037812145	0.999933887	0.563711784	1.403698113	-0.839986328	0.017924782
ENSG00000117143	*UAP1*	3.542033472	-0.05234677	-0.114407276	0.062060507	0.999933887	0.047896916	0.416666262	-0.368769346	0.01796644
ENSG00000164889	*SLC4A2*	4.866644893	0.08783217	0.090051551	-0.00221938	0.999933887	0.489399499	0.800085693	-0.310686194	0.018012382
ENSG00000253522	*MIR3142HG*	1.317249424	0.219109798	0.439303628	-0.22019383	0.999933887	2.01661299	2.892408301	-0.875795311	0.018095581
ENSG00000133816	*MICAL2*	5.651343333	-0.011764062	-0.01127276	-0.000491302	0.999933887	-0.382967366	-0.707790791	0.324823425	0.018095581
ENSG00000169230	*PRELID1*	5.614936646	0.028672632	-0.003922359	0.03259499	0.999933887	-0.07308246	-0.388124234	0.315041774	0.01810546
ENSG00000155330	*C16orf87*	2.166986434	-0.043446289	0.031054592	-0.074500882	0.999933887	0.135204171	0.498497023	-0.363292853	0.01810546
ENSG00000169692	*AGPAT2*	3.890326544	0.055667388	0.031581208	0.024086181	0.999933887	-0.668779122	-1.082692815	0.413913693	0.018120765
ENSG00000070961	*ATP2B1*	9.657755321	-0.047125124	-0.093966328	0.046841204	0.999933887	1.011433213	1.605007617	-0.593574404	0.01813517
ENSG00000123595	*RAB9A*	3.855979721	-0.104640928	-0.142568932	0.037928004	0.999933887	1.079195001	1.4330866	-0.353891599	0.018230834
ENSG00000145675	*PIK3R1*	6.481332601	-0.077963167	-0.144467835	0.066504668	0.999933887	-0.112871612	0.253040668	-0.36591228	0.018230834
ENSG00000115919	*KYNU*	2.733567677	0.024913732	0.062581105	-0.037667373	0.999933887	1.141604746	1.628621634	-0.487016888	0.018230834
ENSG00000169375	*SIN3A*	6.87773346	-0.002487782	-0.064342248	0.061854466	0.999933887	0.705715134	0.963865819	-0.258150685	0.018232605
ENSG00000181631	*P2RY13*	6.166215201	-0.090281674	-0.137658718	0.047377044	0.999933887	-1.922379216	-2.424072464	0.501693248	0.018268492
ENSG00000228382	*ITPKB-IT1*	2.490660025	0.127012555	-0.180811338	0.307823893	0.999933887	0.838489288	1.218322119	-0.37983283	0.018279956
ENSG00000175550	*DRAP1*	5.680569199	0.08709309	0.024292417	0.062800673	0.999933887	-0.184460677	-0.684249082	0.499788405	0.018291641
ENSG00000174738	*NR1D2*	5.569859303	-0.072836478	-0.163098196	0.090261718	0.999933887	0.245009468	0.618813782	-0.373804313	0.018366142
ENSG00000135940	*COX5B*	5.103062838	-0.009787786	-0.056378118	0.046590332	0.999933887	-0.106944863	-0.408146322	0.301201459	0.018366142
ENSG00000127125	*PPCS*	4.050701423	-0.116660122	-0.135955439	0.019295317	0.999933887	-0.416858776	-0.716052524	0.299193748	0.01837878
ENSG00000168769	*TET2*	7.714501632	-0.026975585	-0.090125314	0.063149728	0.999933887	-0.586737885	-0.102202549	-0.484535335	0.018441867
ENSG00000150867	*PIP4K2A*	6.12008218	-0.004094582	-0.033256216	0.029161634	0.999933887	-0.192870685	-0.414441512	0.221570827	0.018441867
ENSG00000135541	*AHI1*	2.089545794	0.007519495	0.168680333	-0.161160838	0.999933887	-0.129412531	0.363382732	-0.492795263	0.018441867
ENSG00000104497	*SNX16*	3.176357007	-0.074732528	0.019291255	-0.094023783	0.999933887	-0.25611046	0.15432947	-0.41043993	0.018457725

ENSG00000169660	*HEXD*	3.870144027	0.04377214	0.087020576	-0.043248436	0.999933887	-0.058997893	-0.447669	0.388671107	0.018457725
ENSG00000121716	*PILRB*	3.114439211	-0.017252151	0.079942538	-0.097194689	0.999933887	0.771906259	1.338368339	-0.56646208	0.018495435
ENSG00000183484	*GPR132*	5.279336332	0.145827322	0.167252862	-0.021425539	0.999933887	1.351945106	1.910675124	-0.558730018	0.018521393
ENSG00000108788	*MLX*	5.117458866	-0.085929284	-0.018074333	-0.067854951	0.999933887	-0.933021953	-1.27039623	0.337374277	0.018521393
ENSG00000108604	*SMARCD2*	5.5600537	0.044645688	0.020965683	0.023680005	0.999933887	-0.367725566	-0.637940443	0.270214877	0.018545522
ENSG00000140153	*WDR20*	4.604527072	-0.032978282	-0.044198009	0.011219727	0.999933887	0.169654961	0.411168957	-0.241513997	0.018551277
ENSG00000102572	*STK24*	5.898806274	-0.034504682	-0.031770308	-0.002734373	0.999933887	-0.816577347	-1.136658678	0.320081331	0.018630133
ENSG00000102401	*ARMCX3*	5.158048029	0.072420393	-0.06754099	0.139961383	0.999933887	0.50467542	0.818798303	-0.314122883	0.018648127
ENSG00000149532	*CPSF7*	7.191010307	-0.041304772	-0.042953966	0.001649194	0.999933887	0.100761667	0.277655837	-0.176894171	0.018648472
ENSG00000149480	*MTA2*	5.014892398	0.051551782	0.050843344	0.000708438	0.999933887	0.100172448	-0.138180798	0.238353246	0.018648472
ENSG00000181649	*PHLDA2*	3.362493764	0.451824118	0.412295341	0.039528777	0.999933887	1.84595073	2.353553145	-0.507602414	0.018695933
ENSG00000139437	*TCHP*	3.812637021	-0.001608103	-0.126650989	0.125042885	0.999933887	-1.346660438	-1.864483208	0.517822771	0.018716206
ENSG00000137801	*THBS1*	4.933753946	0.068990931	0.086414886	-0.017423955	0.999933887	0.424581297	1.035330095	-0.610748798	0.018726434
ENSG00000131236	*CAP1*	8.234651079	-0.022291912	-0.06093648	0.038644568	0.999933887	-0.578626669	-0.883084927	0.304458258	0.018873697
ENSG00000166295	*ANAPC16*	5.049145075	0.022437962	0.031697254	-0.009259292	0.999933887	0.331555964	0.574081909	-0.242525945	0.018942994
ENSG00000070476	*ZXDC*	5.964922511	-0.005664819	-0.006277239	0.00061242	0.999933887	-0.611377648	-0.842060319	0.230682671	0.018955865
ENSG00000072310	*SREBF1*	6.705017434	0.037467879	0.041821256	-0.004353376	0.999933887	0.451288066	0.700960448	-0.249672382	0.018959397
ENSG00000071553	*ATP6AP1*	8.547507946	-0.049416281	-0.043532103	-0.005884179	0.999933887	0.613830034	0.828749808	-0.214919774	0.018972246
ENSG00000165131	*LLCFC1*	-0.403338406	0.164377422	-0.267319254	0.431696676	0.999933887	-0.766688144	-1.547469807	0.780781664	0.019007582
ENSG00000112511	*PHF1*	7.623057645	0.028664365	0.058208193	-0.029543828	0.999933887	0.536606046	0.756219155	-0.219613109	0.019064876
ENSG00000140853	*NLRC5*	8.360759594	0.053785191	0.032723777	0.021061414	0.999933887	-0.321709437	-0.551475334	0.229765897	0.01909151
ENSG00000149231	*CCDC82*	5.163056616	-0.107776268	-0.154585757	0.046809489	0.999933887	0.638824301	1.375021965	-0.736197664	0.01909151
ENSG00000270457	*AC093424.1*	-0.47289177	-0.112905812	-0.037430392	-0.07547542	0.999933887	1.017316664	1.867021903	-0.849705239	0.01909151
ENSG00000197147	*LRRC8B*	3.011017673	-0.041704958	0.002070592	-0.04377555	0.999933887	0.484503593	0.932848152	-0.448344559	0.01909151
ENSG00000120314	*WDR55*	4.628243982	-0.01678596	-0.00478506	-0.012000899	0.999933887	-0.230349892	-0.468609535	0.238259644	0.01909151
ENSG00000013275	*PSMC4*	5.338842624	-0.011339558	0.011590991	-0.022930549	0.999933887	0.392373193	0.652396881	-0.260023688	0.01909151
ENSG00000181135	*ZNF707*	2.467970492	0.059802304	0.133406838	-0.073604535	0.999933887	-0.034971636	-0.418819493	0.383847857	0.019144384
ENSG00000159388	*BTG2*	9.768035326	0.364412936	0.369364415	-0.004951478	0.999933887	1.006843983	1.428214917	-0.421370934	0.019165546
ENSG00000046653	*GPM6B*	0.625083964	-0.20935902	-0.14486986	-0.06448916	0.999933887	0.099428037	0.88067843	-0.781250393	0.019165546
ENSG00000161036	*LRWD1*	5.100663282	0.044671423	0.110745287	-0.066073863	0.999933887	-0.112165853	-0.402792394	0.290626541	0.019165546
ENSG00000196387	*ZNF140*	2.997554288	-0.040229198	-0.073584611	0.033355413	0.999933887	0.326560611	0.639966281	-0.31340567	0.019165546
ENSG00000036054	*TBC1D23*	5.500642358	-0.042190038	-0.092685936	0.050495898	0.999933887	-0.023184439	0.335968075	-0.359152515	0.019207797
ENSG00000064703	*DDX20*	3.402223121	-0.03289515	-0.026514664	-0.006380486	0.999933887	-0.111538102	-0.435629225	0.324091123	0.019207797
ENSG00000164054	*SHISA5*	7.866543889	0.079431504	0.011970259	0.067461245	0.999933887	-0.467220802	-0.802588581	0.335367778	0.019248971
ENSG00000165915	*SLC39A13*	4.070149649	0.075108475	-0.021643629	0.096752104	0.999933887	0.280941034	0.594202237	-0.313261203	0.019248971
ENSG00000118308	*IRAG2*	6.522404716	-0.061007314	-0.118640624	0.05763331	0.999933887	-0.574142323	-0.886398668	0.312256345	0.019248971
ENSG00000100461	*RBM23*	7.169319836	-0.021093341	0.005499167	-0.026592508	0.999933887	0.596925805	0.828713909	-0.231788104	0.019248971
ENSG00000188191	*PRKAR1B*	1.351835343	0.059136973	0.120212394	-0.061075421	0.999933887	0.336267742	0.961478547	-0.625210805	0.019248971
ENSG00000175470	*PPP2R2D*	5.813431072	-0.012599575	-0.024096375	0.0114968	0.999933887	0.192814972	0.373324792	-0.18050982	0.019248971
ENSG00000172869	*DMXL1*	3.953068142	-0.0036904	-0.13805764	0.13436724	0.999933887	-0.195860582	0.328307403	-0.524167985	0.019248971
ENSG00000159348	*CYB5R1*	5.022593551	-0.015590881	-0.02116686	0.00557598	0.999933887	-0.636745273	-0.920631769	0.283886496	0.019253943
ENSG00000114030	*KPNA1*	5.57091542	-0.053229444	-0.084659394	0.031429949	0.999933887	-0.202870508	0.005202331	-0.208072839	0.01929538
ENSG00000273604	*EPOP*	1.39809035	0.086009837	0.176167135	-0.090157298	0.999933887	2.092359671	2.719842871	-0.6274832	0.019296374
ENSG00000111837	*MAK*	3.678491864	-0.045635743	0.02153147	-0.067167213	0.999933887	0.184171303	0.495434492	-0.311263189	0.019300053
ENSG00000141337	*ARSG*	4.065019449	-0.084239974	-0.074205444	-0.01003453	0.999933887	-0.149541873	-0.466557528	0.317015655	0.019322219
ENSG00000238018	*AC093110.1*	1.027340254	-0.099935186	-0.396057772	0.296122586	0.999933887	0.020164712	0.441353918	-0.421189206	0.019402191
ENSG00000047249	*ATP6V1H*	4.996827613	-0.034360211	0.021861437	-0.056221648	0.999933887	0.662150731	0.964425358	-0.302274627	0.019402191
ENSG00000116044	*NFE2L2*	8.495838126	-0.016574664	-0.03301314	0.016438476	0.999933887	0.736253006	1.043966152	-0.307713145	0.019402191
ENSG00000137767	*SQOR*	5.041867824	0.01163546	-0.05801786	0.06965332	0.999933887	0.828033237	1.188550845	-0.360517607	0.019402191
ENSG00000136485	*DCAF7*	4.995975259	-0.08825825	0.022044737	-0.110302987	0.999933887	-0.337501475	-0.613610075	0.2761086	0.019440913
ENSG00000073417	*PDE8A*	2.616537674	0.013637068	0.008546076	0.005090993	0.999933887	0.285013193	0.702565637	-0.417552445	0.019552784
ENSG00000138835	*RGS3*	4.11939002	0.093105624	0.000956858	0.092148766	0.999933887	-0.250446524	-0.654597281	0.404150757	0.019589301
ENSG00000075618	*FSCN1*	2.919385715	0.064231206	-0.089866135	0.154097342	0.999933887	2.475703757	3.221965262	-0.746261504	0.019589301
ENSG00000051523	*CYBA*	9.178312278	0.068204279	0.015301427	0.052902851	0.999933887	0.985319545	1.324408758	-0.339089214	0.01961462
ENSG00000226648	*PLCG1-AS1*	2.020068743	0.093300693	0.005745698	0.087554994	0.999933887	-0.080930057	0.468117651	-0.549047708	0.019636955
ENSG00000215458	*AATBC*	1.060080035	0.036298143	0.040859884	-0.004561742	0.999933887	0.82532022	1.495409542	-0.670089322	0.019730598
ENSG00000107738	*VSIR*	9.442960556	-0.009362462	-0.008176713	-0.001185749	0.999933887	-0.430183561	-0.734444608	0.304261047	0.019730598
ENSG00000164111	*ANXA5*	7.443725353	0.002587578	-0.040585495	0.043173073	0.999933887	1.346094054	1.687759226	-0.341665173	0.019730598
ENSG00000112379	*ARFGEF3*	-1.240230178	0.098290083	0.625327037	-0.527036955	0.999933887	0.874449681	1.59444942	-0.71999974	0.019746995
ENSG00000171298	*GAA*	6.184341239	0.048233169	0.033168487	0.015064682	0.999933887	-0.244606991	-0.50848487	0.263877879	0.019752552
ENSG00000213639	*PPP1CB*	7.529225162	-0.087492396	-0.063194267	-0.024298129	0.999933887	-0.084130884	0.220868428	-0.304999311	0.01975449
ENSG00000213347	*MXD3*	3.663905161	0.050537919	0.059588258	-0.00905034	0.999933887	-0.107534301	-0.53860925	0.43107495	0.019770664
ENSG00000105127	*AKAP8*	5.390287569	-0.000665917	0.022641165	-0.023307082	0.999933887	-0.161694085	-0.366675515	0.204981429	0.019770664
ENSG00000255921	*AC026310.1*	-2.384324374	-0.409115763	-0.083318626	-0.325797137	0.999933887	2.102207969	3.430342166	-1.328134197	0.019790423
ENSG00000047315	*POLR2B*	5.469007736	-0.110725692	-0.109447225	-0.001278466	0.999933887	-0.296412061	-0.536326266	0.239914205	0.019801029
ENSG00000128294	*TPST2*	6.104632222	0.023371912	0.00956458	0.013807332	0.999933887	-0.745557514	-1.055479409	0.309921895	0.019801029
ENSG00000146376	*ARHGAP18*	3.063857264	-0.147628357	-0.033345322	-0.114283035	0.999933887	-0.455504539	-0.081703464	-0.373801076	0.019808519
ENSG00000224609	*HSD52*	3.092397928	0.252506261	0.129432837	0.123073424	0.999933887	1.271381906	1.79488294	-0.523501034	0.019829039
ENSG00000103876	*FAH*	1.600446303	-0.187019501	0.315149629	-0.502169129	0.944094537	-0.432121033	-0.814786413	0.38266538	0.019829039
ENSG00000114861	*FOXP1*	4.799667441	-0.063940731	0.028919249	-0.09285998	0.999933887	0.383399453	0.785204656	-0.401805204	0.019829039
ENSG00000146063	*TRIM41*	4.494919472	0.076656442	0.052599808	0.024056634	0.999933887	-0.216844162	-0.50711072	0.290266558	0.019960797
ENSG00000156508	*EEF1A1*	10.934526471	-0.033064696	-0.177567366	0.14450267	0.999933887	0.304104004	0.590076109	-0.285972106	0.0199651
ENSG00000205155	*PSENEN*	2.960210957	0.077489544	0.064292578	0.013196966	0.999933887	-0.295833015	-0.676107393	0.380274379	0.020010856
ENSG00000153827	*TRIP12*	7.827206781	-0.047762142	-0.072044065	0.024281924	0.999933887	0.144853835	0.377125703	-0.232271868	0.020010856
ENSG00000165650	*PDZD8*	5.182799183	0.062851676	-0.011950824	0.074802499	0.999933887	-0.663255004	-1.116759472	0.453504468	0.020023433
ENSG00000087470	*DNM1L*	3.235622457	-0.13247971	-0.202723808	0.070244098	0.999933887	0.173111658	0.733913738	-0.56080208	0.020075975
ENSG00000224531	*SMIM13*	3.081667061	-0.074616869	0.008938266	-0.083555134	0.999933887	-0.165802097	0.144762117	-0.310564213	0.020075975
ENSG00000188313	*PLSCR1*	5.755929162	-0.019445299	-0.041224873	0.021779575	0.999933887	0.560610483	0.838077857	-0.277467373	0.020075975
ENSG00000166401	*SERPINB8*	3.913431967	-0.03297302	-0.008258085	-0.024714935	0.999933887	0.75610363	1.195009891	-0.438906261	0.020075975
ENSG00000008282	*SYPL1*	3.655534044	-0.014008712	-0.067564074	0.053555363	0.999933887	0.187919412	0.481829362	-0.29390995	0.020075975
ENSG00000136950	*ARPC5L*	3.997404512	-0.009336957	-0.018396651	0.009059693	0.999933887	-0.04601525	-0.31466831	0.268653059	0.020075975
ENSG00000138031	*ADCY3*	4.51306348	-0.003038673	-0.016775283	0.01373661	0.999933887	0.935513868	1.277800921	-0.342287053	0.020075975
ENSG00000142512	*SIGLEC10*	6.485211668	0.035629744	0.034243942	0.001385802	0.999933887	0.503827034	0.825163316	-0.321336282	0.020077272
ENSG00000250510	*GPR162*	1.194064892	0.017838049	-0.027193643	0.045031692	0.999933887	-0.597128838	-1.440928942	0.843800103	0.020091685
ENSG00000079387	*SENP1*	3.544185974	0.002023954	-0.011707187	0.013731141	0.999933887	0.385858661	0.671694014	-0.285835353	0.020091685
ENSG00000082014	*SMARCD3*	3.685998976	0.029157404	0.022443815	0.006713589	0.999933887	0.100598502	-0.244528115	0.345126617	0.020118608
ENSG00000100994	*PYGB*	4.003969774	-0.019738212	0.01860471	-0.038342922	0.999933887	-0.061629173	-0.322423956	0.260794783	0.020118608
ENSG00000168216	*LMBRD1*	5.230369397	-0.037352683	-0.049353221	0.012000538	0.999933887	-0.324918392	-0.62040295	0.295484558	0.02012639
ENSG00000181444	*ZNF467*	6.066515759	0.04910024	0.088357531	-0.039257291	0.999933887	-0.950207923	-1.386350653	0.43614273	0.020164442
ENSG00000075303	*SLC25A40*	3.662648893	-0.029617852	-0.119588279	0.089970427	0.999933887	0.159516654	0.559277187	-0.399760533	0.020164442
ENSG00000269713	*NBPF9*	2.322581916	0.003103494	-0.097797007	0.100900501	0.999933887	0.425631082	0.846542927	-0.420911844	0.020164442
ENSG00000137575	*SDCBP*	10.809163669	-0.091083008	-0.125275727	0.034192719	0.999933887	0.044818186	0.295292549	-0.250474363	0.020254093
ENSG00000141503	*MINK1*	6.44440546	0.026865534	0.019415549	0.007449985	0.999933887	-0.152751992	-0.361293676	0.208541684	0.020297625
ENSG00000137193	*PIM1*	8.492363704	-0.100666548	-0.103544323	0.002877775	0.999933887	0.134409866	0.45186177	-0.317451904	0.02044054
ENSG00000126070	*AGO3*	4.94584566	-0.001417687	-0.012006222	0.010588536	0.999933887	0.218024818	0.515477541	-0.297452723	0.020453532
ENSG00000095139	*ARCN1*	6.012466305	-0.032118458	-0.005210168	-0.02690829	0.999933887	0.009782335	0.213826789	-0.204044454	0.020456012
ENSG00000178803	*ADORA2A-AS*	5.952960649	0.230950781	0.146156546	0.084794236	0.999933887	1.873465116	2.281641848	-0.408176732	0.020471595
ENSG00000131203	*IDO1*	1.019822559	-0.093396073	-0.008443149	-0.084952924	0.999933887	0.381876303	1.034919864	-0.653043561	0.02049399
ENSG00000261324	*AC010168.2*	1.680549125	-0.056586218	0.070793249	-0.127379467	0.999933887	-0.084035071	-0.60231618	0.518281109	0.02049399
ENSG00000115137	*DNAJC27*	2.617162893	-0.030207911	-0.032721976	0.002514065	0.999933887	0.114532724	0.542345169	-0.427812446	0.02049399
ENSG00000261546	*AC135782.3*	-0.255661167	-0.00080661	-0.044059023	0.043252413	0.999933887	-0.619699209	-1.563644579	0.94394537	0.020581865
ENSG00000104064	*GABPB1*	3.955777106	-0.00275484	0.073498877	-0.076253716	0.999933887	0.883797244	1.20828844	-0.324491196	0.020589562
ENSG00000139625	*MAP3K12*	3.526309563	-0.004323837	0.060417548	-0.064741384	0.999933887	-0.519118669	-0.84398728	0.324868611	0.02061159
ENSG00000159202	*UBE2Z*	6.26912915	-0.004969917	-0.095335599	0.090365682	0.999933887	0.560531518	0.766413441	-0.205881923	0.020651994
ENSG00000135766	*EGLN1*	4.181372167	0.116029719	0.090419305	0.025610414	0.999933887	-0.641712598	-1.204682769	0.562970172	0.020769419

ENSG00000071054	*MAP4K4*	9.783929495	-0.044631056	-0.007489439	-0.037141617	0.999933887	0.40181685	0.601935254	-0.200118403	0.020769419
ENSG00000185404	*SP140L*	4.27168288	-0.045178488	-0.066552537	0.021374049	0.999933887	-0.290678511	-0.526930075	0.236251565	0.020769419
ENSG00000148985	*PGAP2*	3.920419495	0.045041952	-0.028538742	0.073580694	0.999933887	0.742987668	1.128182164	-0.385194496	0.020769419
ENSG00000158850	*B4GALT3*	4.142032927	0.021300574	0.052098421	-0.030797847	0.999933887	-0.115279216	-0.351928715	0.236649499	0.020769419
ENSG00000163482	*STK36*	2.510255551	0.032198235	-0.080244451	0.112442686	0.999933887	0.53284275	0.877792783	-0.344950033	0.020769419
ENSG00000246575	*AC093162.1*	2.611078876	-0.017235689	-0.097210843	0.079975154	0.999933887	-0.317062831	-0.691655654	0.374592822	0.020769419
ENSG00000102531	*FNDC3A*	6.345639422	-0.021782003	-0.035760261	0.013978258	0.999933887	-0.463675762	-0.089479343	-0.374196419	0.020769419
ENSG00000212907	*MT-ND4L*	6.03153988	0.001316329	-0.082926244	0.084242573	0.999933887	-0.02058827	0.310825815	-0.331414085	0.020769419
ENSG00000250616	*AC012645.1*	1.644984498	-0.009308995	0.008481018	-0.017790013	0.999933887	-1.157019153	-2.020096229	0.863077077	0.020839212
ENSG00000244405	*ETV5*	-0.218188178	-0.025154275	-0.087542271	0.062387996	0.999933887	0.756971673	1.800482725	-1.043511052	0.021072907
ENSG00000070423	*RNF126*	4.699187934	-0.002497975	0.02920998	-0.031707956	0.999933887	-0.078912277	-0.338690204	0.259777928	0.02109467
ENSG00000219891	*ZSCAN12P1*	0.065464981	0.147285497	0.187875615	-0.040590118	0.999933887	-0.548424548	0.209671003	-0.758095552	0.021123327
ENSG00000198873	*GRK5*	4.720698901	0.016751839	0.004927354	0.011824485	0.999933887	0.24316487	0.489236397	-0.246071528	0.021139399
ENSG00000180448	*ARHGAP45*	9.123306221	0.052783902	0.08581531	-0.033031408	0.999933887	-0.359422	-0.661418438	0.301996438	0.02114403
ENSG00000105928	*GSDME*	-0.544871663	-0.218137776	0.162879203	-0.381016978	0.999933887	0.713177304	1.388482027	-0.675304724	0.021255058
ENSG00000204681	*GABBR1*	3.845949769	0.052095781	0.059581284	-0.007485503	0.999933887	0.286308036	0.595234641	-0.308926605	0.021255058
ENSG00000077585	*GPR137B*	2.701959553	0.030320366	0.028306093	0.002014273	0.999933887	0.219838828	0.541872023	-0.322033195	0.021255058
ENSG00000241764	*AC002467.1*	0.050713245	-0.029911955	-0.323573787	0.293661833	0.999933887	-0.262838269	-1.097446111	0.834607842	0.021255058
ENSG00000085511	*MAP3K4*	3.022467502	-0.009244642	-0.074602375	0.065357733	0.999933887	0.170160987	0.536403125	-0.366242138	0.021308422
ENSG00000127616	*SMARCA4*	4.824265169	-0.007692931	0.020414668	-0.028107599	0.999933887	-0.027579585	-0.232042432	0.204462847	0.021378552
ENSG00000168229	*PTGDR*	0.791261722	0.1080188	-0.04982437	0.157843169	0.999933887	0.677828123	1.343511889	-0.665683766	0.021407437
ENSG00000166507	*NDST2*	0.288285456	0.046565782	-0.215610204	0.262175986	0.999933887	0.008933907	-0.728433878	0.737367785	0.021407437
ENSG00000173320	*STOX2*	2.779059777	0.003941399	0.134053974	-0.130112575	0.999933887	-0.139410994	0.159902871	-0.299313865	0.021436896
ENSG00000249115	*HAUS5*	2.170714725	0.035473096	-0.068864405	0.104337501	0.999933887	0.351757243	0.844424059	-0.492666816	0.021481812
ENSG00000165102	*HGSNAT*	4.588575037	-0.054612007	-0.023279478	-0.031332529	0.999933887	-0.095670335	-0.353234957	0.257564621	0.021484232
ENSG00000170852	*KBTBD2*	6.855151098	-0.039398921	-0.00852532	-0.030873601	0.999933887	0.130081246	0.304000662	-0.173919416	0.021484232
ENSG00000175970	*UNC119B*	2.807857932	-0.014445896	-0.110075215	0.095629319	0.999933887	-0.335259134	-0.697238201	0.361979067	0.021484232
ENSG00000241058	*NSUN6*	2.345770631	0.01485619	0.099131033	-0.084274843	0.999933887	0.679020859	1.2047099	-0.525689041	0.021484232
ENSG00000109270	*LAMTOR3*	6.517294642	0.004573746	-0.029917696	0.034491442	0.999933887	0.299794765	0.514633092	-0.214838327	0.021484232
ENSG00000102265	*TIMP1*	6.445366441	0.016434206	-0.016491347	0.032925553	0.999933887	0.271437901	0.517914342	-0.246476442	0.021562148
ENSG00000167797	*CDK2AP2*	3.506216841	0.050184372	0.06763557	-0.017451198	0.999933887	0.023619068	-0.283367207	0.306986274	0.021624344
ENSG00000023330	*ALAS1*	3.604017252	0.057615818	0.030958125	0.026657693	0.999933887	-0.106602742	-0.432366432	0.325763689	0.021624344
ENSG00000243927	*MRPS6*	3.281086532	0.019242015	0.047232809	-0.027990794	0.999933887	0.591195143	1.04876631	-0.457571167	0.021624344
ENSG00000204131	*NHSL2*	5.999584909	-0.025985246	-0.020970814	-0.005014432	0.999933887	-1.004109881	-1.470713647	0.466603766	0.021656055
ENSG00000001561	*ENPP4*	2.023684473	-0.088408923	-0.104755889	0.016346966	0.999933887	0.001978517	0.569760979	-0.567782462	0.0217522
ENSG00000126653	*NSRP1*	4.364150075	-0.093979464	-0.124873884	0.03089442	0.999933887	-0.125414723	0.349594937	-0.47500966	0.02181991
ENSG00000112531	*QKI*	8.194449323	-0.030407263	-0.027399598	-0.003007665	0.999933887	0.776022792	1.139704357	-0.363681565	0.021821932
ENSG00000119681	*LTBP2*	2.956072599	0.059205244	0.013441881	0.045763363	0.999933887	-0.812510906	-1.206811421	0.394300515	0.021822793
ENSG00000011405	*PIK3C2A*	4.437591582	-0.05368187	-0.176731135	0.123049265	0.999933887	-0.315104799	0.475191649	-0.790296448	0.021822793
ENSG00000113302	*IL12B*	0.678584304	-0.204672788	0.373749128	-0.578421916	0.999933887	1.03756536	1.640018116	-0.602452756	0.021879525
ENSG00000124541	*RRP36*	2.988451499	-0.081537929	-0.021287675	-0.060250255	0.999933887	-0.193790047	-0.497782269	0.303992221	0.021953682
ENSG00000096384	*HSP90AB1*	7.255156533	-0.050095198	-0.106557232	0.056462034	0.999933887	0.45118936	0.695090194	-0.243900835	0.021958818
ENSG00000171606	*ZNF274*	6.261814262	0.014549656	0.028574265	-0.014024609	0.999933887	0.146737937	0.351404573	-0.204666637	0.022073026
ENSG00000160813	*PPP1R35*	3.852824149	0.105103188	0.170213245	-0.065110057	0.999933887	-0.316687053	-0.651396564	0.334709512	0.022078172
ENSG00000196739	*COL27A1*	-0.479095701	0.036736729	0.143804012	-0.107067283	0.999933887	1.377179921	2.39786742	-1.020687498	0.022078172
ENSG00000066135	*KDM4A*	2.612522408	0.060567217	0.051651955	0.008915262	0.999933887	-0.325120079	-0.735039329	0.40991925	0.02215695
ENSG00000183864	*TOB2*	4.493294148	0.069320089	0.036251837	0.033068253	0.999933887	-0.316665908	-0.603701672	0.287035764	0.022173102
ENSG00000160679	*CHTOP*	5.661422984	-0.026156336	0.013228891	-0.039385227	0.999933887	-0.294392337	-0.514056642	0.219664306	0.022222936
ENSG00000082996	*RNF13*	8.300169145	-0.078166101	-0.088397071	0.01023097	0.999933887	-0.392877959	-0.152635265	-0.240242694	0.022354492
ENSG00000167962	*ZNF598*	4.450833575	0.098908892	0.123692617	-0.024783724	0.999933887	0.539089484	0.863503265	-0.324413782	0.02236655
ENSG00000009413	*REV3L*	5.588997551	-0.134714366	-0.095028015	-0.039686351	0.999933887	0.358497964	1.00773918	-0.649241216	0.02236655
ENSG00000183621	*ZNF438*	6.039782024	-0.036065171	-0.019890823	-0.016174348	0.999933887	0.054664148	0.279559375	-0.224895227	0.02236655
ENSG00000215790	*SLC35E2A*	2.416355402	0.095850559	0.119124067	-0.023273508	0.999933887	-0.392157106	-0.787862036	0.395704929	0.022407411
ENSG00000006007	*GDE1*	3.867410195	0.009006753	-0.138521262	0.147528016	0.999933887	-0.321898368	-0.757698201	0.435799832	0.022407411
ENSG00000169026	*SLC49A3*	2.620983215	0.081721998	-0.050630519	0.132352516	0.999933887	-0.995474448	-1.536828682	0.541354233	0.022428371
ENSG00000123268	*ATF1*	3.520753755	-0.079287758	-0.108913591	0.029625833	0.999933887	-0.064298541	0.198735964	-0.263034505	0.022446524
ENSG00000242125	*SNHG3*	3.73929552	-0.072843018	0.048216262	-0.12105928	0.999933887	0.125647109	-0.149683118	0.275330227	0.022447436
ENSG00000115053	*NCL*	6.815759375	-0.039705205	-0.084097448	0.044392243	0.999933887	0.25689313	0.510360455	-0.253467325	0.022618078
ENSG00000163249	*CCNYL1*	3.706411568	0.037807874	0.06128459	-0.023476715	0.999933887	0.147952109	0.451111073	-0.303158964	0.022653606
ENSG00000149716	*LTO1*	3.052593633	-0.066488355	-0.100238924	0.033750569	0.999933887	-0.08628084	-0.339105077	0.252824237	0.02266467
ENSG00000076770	*MBNL3*	2.48803395	-0.097642002	-0.063386232	-0.034255771	0.999933887	-0.243690347	0.406415708	-0.650106054	0.02266467
ENSG00000178761	*FAM219B*	2.015898794	0.007895325	-0.109823563	0.117718888	0.999933887	0.07226678	-0.317547201	0.389813981	0.022676565
ENSG00000159128	*IFNGR2*	7.826886407	-0.002372084	0.010233647	-0.012605731	0.999933887	1.449021766	1.770795556	-0.32177379	0.022723894
ENSG00000124762	*CDKN1A*	7.414633029	0.243007898	0.262830201	-0.019822303	0.999933887	1.183007137	1.540861108	-0.357853971	0.022730871
ENSG00000101493	*ZNF516*	6.640030957	0.017393572	0.055325585	-0.037932013	0.999933887	-0.653520203	-0.924181689	0.270661486	0.022761432
ENSG00000137547	*MRPL15*	1.208977996	-0.085466993	0.182904216	-0.268371209	0.999933887	-0.043229626	0.44719916	-0.490428786	0.022799205
ENSG00000141497	*ZMYND15*	5.375091627	-0.001911084	-0.010651286	0.008740202	0.999933887	-0.426785109	-0.123576616	-0.303208494	0.022806892
ENSG00000164823	*OSGIN2*	6.233409878	-0.053112935	0.018068846	-0.07118178	0.999933887	0.048070076	0.327222011	-0.279151935	0.022819217
ENSG00000159069	*FBXW5*	5.802320848	0.05153199	0.018852072	0.032679918	0.999933887	0.00502816	-0.25907571	0.26410387	0.022827491
ENSG00000135114	*OASL*	5.275420502	0.030987304	-0.035576628	0.066563932	0.999933887	-0.157731873	-0.495024982	0.337293109	0.022878301
ENSG00000134243	*SORT1*	3.661349691	-0.024932795	-0.042876248	0.017943454	0.999933887	-0.801399596	-1.199217137	0.397817542	0.022878301
ENSG00000105290	*APLP1*	0.725012185	0.194861567	0.304887418	-0.110025852	0.999933887	2.411641629	3.287586503	-0.875944873	0.023066406
ENSG00000242259	*C22orf39*	3.175329976	0.044108197	-0.048755075	0.092863272	0.999933887	-0.588707889	-0.945364265	0.356656376	0.023067299
ENSG00000113194	*FAF2*	4.822265855	-0.015152997	-0.02742058	0.012267583	0.999933887	0.002833555	0.31658866	-0.313755105	0.023101173
ENSG00000120539	*MASTL*	3.672999739	-0.036897065	0.071812292	-0.108709357	0.999933887	0.707753333	1.019064258	-0.311310925	0.023193251
ENSG00000048649	*RSF1*	5.567964676	-0.083939181	-0.119560051	0.03562087	0.999933887	-0.45735586	-0.008451585	-0.448904275	0.023212559
ENSG00000113648	*MACROH2A1*	7.579486308	-0.015912061	-0.025743892	0.009831831	0.999933887	-1.246224936	-1.561348324	0.315123388	0.023364746
ENSG00000013374	*NUB1*	5.919378907	0.015787953	-0.003834453	0.019622406	0.999933887	-0.31121456	-0.568308187	0.257093626	0.023383096
ENSG00000138029	*HADHB*	5.104268526	-0.074352791	-0.020468811	-0.05388398	0.999933887	-0.325841628	-0.525591621	0.199749994	0.023406732
ENSG00000091009	*RBM27*	3.700437637	-0.063595366	-0.153291425	0.089696058	0.999933887	-0.413628414	-0.045636258	-0.367992157	0.023452472
ENSG00000198952	*SMG5*	6.06932519	0.029951477	0.013375473	0.016576004	0.999933887	-0.236970062	-0.428213389	0.191243327	0.02347914
ENSG00000144815	*NXPE3*	3.53666304	0.027201637	-0.012749593	0.039951229	0.999933887	-0.128193676	-0.499199306	0.37100563	0.023696902
ENSG00000159023	*EPB41*	7.439547853	-0.076569878	-0.032642082	-0.043927797	0.999933887	-0.414858038	-0.656030747	0.241172709	0.02370665
ENSG00000166592	*RRAD*	0.618266061	0.415859511	0.792326547	-0.376467035	0.999933887	2.013966698	2.797266411	-0.783299713	0.023725344
ENSG00000090520	*DNAJB11*	4.773925218	-0.022375785	0.034118561	-0.056494345	0.999933887	0.268103294	0.526954707	-0.258851413	0.023845456
ENSG00000070047	*PHRF1*	5.784357864	0.012228217	0.063743662	-0.051515445	0.999933887	-0.17026656	-0.441692527	0.271425967	0.023892146
ENSG00000214046	*SMIM7*	2.867963938	-0.057099051	-0.033082712	-0.024016339	0.999933887	-0.200258001	-0.480363225	0.280105223	0.02397898
ENSG00000136379	*ABHD17C*	-0.240883853	0.007284352	-0.254379116	0.261663468	0.999933887	-0.383617892	-1.21582658	0.832208688	0.023984236
ENSG00000157954	*WIPI2*	6.274227127	-0.045360442	-0.036979276	-0.008381166	0.999933887	-0.53293127	-0.758319664	0.225388394	0.024041665
ENSG00000104714	*ERICH1*	5.257304794	0.044361065	0.045498978	-0.001137913	0.999933887	-0.244205625	-0.54732976	0.303124135	0.024072729
ENSG00000142552	*RCN3*	4.062651025	0.03880702	0.161420454	-0.122613433	0.999933887	-0.315592516	-0.830184151	0.514591635	0.024108155
ENSG00000074695	*LMAN1*	2.930005217	-0.051396618	-0.096119752	0.044723134	0.999933887	-0.062863372	0.406444029	-0.469307401	0.024182826
ENSG00000103066	*PLA2G15*	1.304072589	0.054542052	-0.141388685	0.195930737	0.999933887	-0.174511275	-0.790180397	0.615669122	0.024182826
ENSG00000171448	*ZBTB26*	3.033684326	-0.091835958	-0.218526758	0.126690801	0.999933887	0.662779614	1.002445169	-0.339665555	0.024187191
ENSG00000197045	*GMFB*	4.667228332	-0.075979189	-0.223853785	0.147874596	0.999933887	-0.331373736	0.066158649	-0.397532385	0.024231784
ENSG00000286555	*AP003065.1*	4.476968629	-0.046724989	-0.071317791	0.024592803	0.999933887	-0.167168068	0.136720992	-0.30388906	0.024231784
ENSG00000100281	*HMGXB4*	4.420952603	-0.015679245	0.067842731	-0.083521976	0.999933887	0.345409503	0.605331974	-0.259922471	0.024250928
ENSG00000186827	*TNFRSF4*	2.459879159	-0.02871214	0.023056595	-0.051768735	0.999933887	1.079071174	1.67783039	-0.598759216	0.024268317
ENSG00000110768	*GTF2H1*	5.701562729	-0.036467983	0.029123121	-0.065591104	0.999933887	0.283908188	0.529987578	-0.24607939	0.024326791
ENSG00000110047	*EHD1*	8.626710668	-0.054442244	0.009863708	-0.064305951	0.999933887	2.400045175	2.979681881	-0.579636705	0.024368204
ENSG00000148187	*MRRF*	2.981826754	-0.064903674	-0.015596269	-0.049307405	0.999933887	0.196286277	0.508070731	-0.311784453	0.024412527
ENSG00000054965	*FAM168A*	5.719181154	0.007809861	0.015528093	-0.007718232	0.999933887	-0.76251863	-1.053271983	0.290753353	0.024412527
ENSG00000256229	*ZNF486*	1.633290229	-0.058559498	0.033707005	-0.092266503	0.999933887	-0.823452282	-1.374648588	0.551196306	0.024421223
ENSG00000148297	*MED22*	4.57165746	-0.082613131	-0.018959592	-0.063653538	0.999933887	-0.319671922	-0.613415387	0.293743465	0.024437538
ENSG00000204310	*AGPAT1*	3.329253403	-0.021677647	0.001974505	-0.023652152	0.999933887	-0.286832738	-0.639073079	0.35224034	0.024462764

ENSG00000156042	*CFAP70*	1.161647234	-0.010034756	-0.089791407	0.079756651	0.999933887	0.644948646	1.263096133	-0.618147488	0.024556015
ENSG00000166016	*ABTB2*	3.60282547	-0.009854434	-0.016361941	0.006507507	0.999933887	0.398362422	0.822377569	-0.424015147	0.024557495
ENSG00000011304	*PTBP1*	6.846958324	0.006967807	0.021464646	-0.014496839	0.999933887	-0.142386932	-0.322286109	0.179899176	0.024619635
ENSG00000178234	*GALNT11*	4.369141516	-0.003557189	0.010278807	-0.013835996	0.999933887	0.200912848	0.4191982	-0.218285352	0.024630167
ENSG00000067798	*NAV3*	0.293640316	-0.059439534	-0.117392842	0.057953308	0.999933887	2.101244205	2.906460765	-0.80521656	0.024644695
ENSG00000134780	*DAGLA*	1.161775735	-0.052288096	0.169576294	-0.22186439	0.999933887	-0.129467061	0.391969574	-0.521436635	0.024644695
ENSG00000197136	*PCNX3*	6.586279521	0.005037677	0.048708832	-0.043671156	0.999933887	-0.203493045	-0.405888726	0.202395681	0.024685697
ENSG00000143933	*CALM2*	7.088392152	-0.089079777	-0.116892058	0.02781228	0.999933887	-0.459278338	-0.698757111	0.239478773	0.024687679
ENSG00000068079	*IFI35*	4.696236008	0.017791075	-0.071643147	0.089434221	0.999933887	-0.439368513	-0.776158311	0.336789798	0.024687679
ENSG00000072062	*PRKACA*	5.547969984	0.006991971	0.030958846	-0.023966875	0.999933887	-0.104862117	-0.377898683	0.273036566	0.024744546
ENSG00000160741	*CRTC2*	7.621386629	0.018632686	0.051251198	-0.032618512	0.999933887	0.902106845	1.120626418	-0.218519573	0.024799411
ENSG00000171867	*PRNP*	5.786261098	-0.000415512	0.017275544	-0.017691057	0.999933887	0.267889901	0.51280759	-0.244917689	0.024799411
ENSG00000066336	*SPI1*	10.297134615	0.061479693	0.03714822	0.024331473	0.999933887	0.340383037	0.539251966	-0.19886893	0.02480964
ENSG00000197302	*ZNF720*	2.722967887	0.04151557	0.056571857	-0.015056287	0.999933887	-0.031808697	0.382888186	-0.414696883	0.02480964
ENSG00000140391	*TSPAN3*	4.592059049	0.007433155	-0.008509737	0.015942892	0.999933887	0.361016531	0.655999559	-0.294983028	0.02480964
ENSG00000273611	*ZNHIT3*	2.855074334	-0.018159318	-0.072632352	0.054473033	0.999933887	-0.12761981	-0.43579885	0.30817904	0.024997937
ENSG00000143409	*MINDY1*	4.822238837	-0.056047948	0.071428513	-0.127476461	0.999933887	-0.660291252	-1.066521575	0.406230322	0.025037515
ENSG00000196371	*FUT4*	4.040090106	0.08140056	0.025137139	0.056263421	0.999933887	0.985194358	1.322342881	-0.337148523	0.025081883
ENSG00000128463	*EMC4*	3.49932858	0.016813819	-0.131740147	0.148553966	0.999933887	-0.029529075	-0.294678355	0.26514928	0.025105783
ENSG00000154832	*CXXC1*	4.318801307	0.054639303	0.018798127	0.035841175	0.999933887	0.008369355	-0.28380952	0.292178875	0.025153686
ENSG00000092330	*TINF2*	6.683428219	0.039240232	0.034832234	0.004407998	0.999933887	0.476429883	0.682701315	-0.206271432	0.025153686
ENSG00000120899	*PTK2B*	9.265902746	0.031668734	0.027924544	0.003744191	0.999933887	0.887982091	1.198641481	-0.310659389	0.025153686
ENSG00000277224	*H2BC7*	0.430432395	0.084342343	0.20558706	-0.121244717	0.999933887	-0.601941891	-1.606067436	1.004125546	0.025242413
ENSG00000171421	*MRPL36*	1.002463798	0.001378873	0.257687748	-0.256308875	0.999933887	0.053143699	-0.598120633	0.651264332	0.025250244
ENSG00000006062	*MAP3K14*	4.129330585	-0.006898154	0.061534008	-0.068432162	0.999933887	-0.149399982	-0.437084362	0.28768438	0.025259189
ENSG00000104529	*EEF1D*	6.389684508	0.002939793	0.00534265	-0.002402857	0.999933887	-0.229188093	-0.454157324	0.224969231	0.025259189
ENSG00000198900	*TOP1*	7.290191045	-0.039742497	-0.063337699	0.023595201	0.999933887	0.20006838	0.446973934	-0.246905554	0.025310409
ENSG00000147383	*NSDHL*	0.633414954	-0.053566345	0.01284078	-0.066407125	0.999933887	0.034674094	-0.553339635	0.588013729	0.025310409
ENSG00000273284	*AP001033.2*	2.582458378	-0.148558747	-0.25165716	0.103098413	0.999933887	-0.259445645	0.362898458	-0.622344103	0.025339684
ENSG00000070540	*WIPI1*	3.663910632	-0.050732165	0.075745919	-0.126478085	0.999933887	-0.685082634	-1.023906478	0.338823843	0.025381782
ENSG00000023909	*GCLM*	5.028918478	-0.070366451	0.007311233	-0.077677683	0.999933887	0.418167465	0.726408467	-0.308241002	0.025381782
ENSG00000282851	*BISPR*	1.852966571	0.172313679	0.213505488	-0.041191809	0.999933887	-0.140055942	-0.61258099	0.472525048	0.025498332
ENSG00000242485	*MRPL20*	3.404949719	-0.085871701	-0.122411677	0.036539976	0.999933887	-0.158064891	-0.391634629	0.233569737	0.025534178
ENSG00000106261	*ZKSCAN1*	5.125820913	-0.061029748	-0.078371533	0.017341785	0.999933887	-0.003401401	0.230651109	-0.23405251	0.025534178
ENSG00000083814	*ZNF671*	1.783826485	0.065676555	0.1017377	-0.036061144	0.999933887	0.007907934	-0.390084826	0.39799276	0.025534178
ENSG00000133657	*ATP13A3*	6.866518609	-0.049299833	-0.029377399	-0.019922434	0.999933887	0.49193398	0.922473504	-0.430539525	0.025534178
ENSG00000078403	*MLLT10*	3.845262964	-0.034187032	-0.059592825	0.025405793	0.999933887	-0.498429571	-0.823051345	0.324621774	0.025534178
ENSG00000169429	*CXCL8*	13.85870056	0.547314938	0.527723852	0.019591086	0.999933887	2.034739193	2.530100787	-0.495361595	0.025544397
ENSG00000164081	*TEX264*	2.808516155	0.036228811	-0.074145854	0.110374665	0.999933887	0.028325383	-0.350160232	0.378485616	0.025559094
ENSG00000198743	*SLC5A3*	3.949093873	-0.034692545	-0.111916861	0.077224316	0.999933887	0.653963885	1.319041524	-0.665077639	0.025644012
ENSG00000105717	*PBX4*	2.156319287	-0.034802808	0.013291502	-0.048094311	0.999933887	0.35594664	0.74859507	-0.39264843	0.025681919
ENSG00000106460	*TMEM106B*	4.160864273	0.048769767	-0.163182297	0.211952064	0.999933887	-0.473407925	0.030549015	-0.50395694	0.025681919
ENSG00000116793	*PHTF1*	5.017663339	-0.043605556	0.017584691	-0.061190247	0.999933887	-0.359403324	-0.585409858	0.226006535	0.025726915
ENSG00000110848	*CD69*	5.737848119	-0.031999641	-0.079268009	0.047268368	0.999933887	0.132295768	0.484214393	-0.351918625	0.025726915
ENSG00000140519	*RHCG*	-1.645547746	-0.001035673	0.246876825	-0.247912497	0.999933887	3.830612409	5.173221195	-1.342608786	0.025726915
ENSG00000197405	*C5AR1*	11.056494624	0.038543226	0.018102939	0.020440286	0.999933887	-0.221799028	-0.005561784	-0.216237244	0.025760935
ENSG00000126698	*DNAJC8*	6.503319004	-0.04719119	-0.122167909	0.07497672	0.999933887	0.547045452	0.785856474	-0.238811022	0.025850622
ENSG00000171310	*CHST11*	7.557069469	0.078594715	0.105614376	-0.027019661	0.999933887	-0.197590086	-0.47382233	0.276232245	0.025875988
ENSG00000175505	*CLCF1*	1.986654196	0.155913208	0.105429302	0.050483906	0.999933887	1.505106886	2.136462712	-0.631355827	0.025875988
ENSG00000072958	*AP1M1*	5.254712139	0.042091884	-0.013114231	0.055206116	0.999933887	-0.298687981	-0.523272583	0.224584602	0.025875988
ENSG00000092036	*HAUS4*	2.236151178	0.057606191	-0.02113264	0.07873883	0.999933887	-0.232154156	-0.825730575	0.593576419	0.025875988
ENSG00000187713	*TMEM203*	2.546677114	0.047105592	-0.090257756	0.137363348	0.999933887	-0.033294763	-0.425732028	0.392437265	0.025875988
ENSG00000070759	*TESK2*	4.538527994	0.014112336	-0.010094773	0.024207109	0.999933887	0.248167706	0.513597817	-0.26543011	0.025894986
ENSG00000176973	*FAM89B*	1.169459282	0.1319522	-0.119335002	0.251287202	0.999933887	0.247245192	-0.455877809	0.703123001	0.025907486
ENSG00000227502	*MROCKI*	2.482655805	-0.036971025	-0.053111843	0.016140817	0.999933887	0.841189536	1.446872428	-0.605682892	0.025907486
ENSG00000132141	*CCT6B*	-0.201177554	0.005317428	0.186358207	-0.181040779	0.999933887	0.136415499	1.052950539	-0.916535039	0.025907486
ENSG00000224051	*CPTP*	3.024342933	0.00708862	0.005591613	0.001497006	0.999933887	-0.53060376	-0.930373513	0.399769753	0.025930573
ENSG00000179044	*EXOC3L1*	-0.214381433	-0.097852028	0.091570051	-0.189422079	0.999933887	-1.080882254	-2.640032112	1.559149857	0.026032014
ENSG00000231074	*HCG18*	4.381739555	0.063537359	0.035003279	0.02853408	0.999933887	0.145869753	0.45530225	-0.309432498	0.02607577
ENSG00000072163	*LIMS2*	-0.015467429	0.051930046	0.088574169	-0.036644123	0.999933887	-0.246007841	-1.169751623	0.923743782	0.026155447
ENSG00000118454	*ANKRD13C*	2.574310966	-0.014943729	0.011404743	-0.026348472	0.999933887	-0.10913838	0.245136607	-0.354274987	0.026253579
ENSG00000139990	*DCAF5*	5.522377349	0.005721116	0.013808126	-0.00808701	0.999933887	-0.437852177	-0.669358515	0.231506338	0.026253579
ENSG00000272084	*AL137127.1*	1.888004171	0.080529295	-0.012294855	0.09282415	0.999933887	0.833972639	1.307727784	-0.473755145	0.026286115
ENSG00000131669	*NINJ1*	10.103021777	0.041006428	-0.002092013	0.043098442	0.999933887	1.087995885	1.315806062	-0.227810177	0.026299314
ENSG00000124209	*RAB22A*	5.745629625	-0.053004702	-0.102104103	0.049099401	0.999933887	-0.180266063	-0.016125804	-0.164140259	0.026316651
ENSG00000198093	*ZNF649*	0.33017136	0.010278495	-0.241823414	0.252101909	0.999933887	0.039804853	0.639460624	-0.599655771	0.026316651
ENSG00000071243	*ING3*	5.490253882	-0.023889175	-0.054958687	0.031069512	0.999933887	0.413601964	0.690553763	-0.276951799	0.026436753
ENSG00000102974	*CTCF*	4.913413067	-0.082976308	-0.015175104	-0.067801204	0.999933887	-0.382114775	-0.661204919	0.279090144	0.02644315
ENSG00000262884	*AC015921.1*	-1.048490788	0.043664211	0.052605055	-0.008940844	0.999933887	-0.537573753	-1.952912812	1.41533906	0.02644315
ENSG00000107021	*TBC1D13*	3.741818305	-0.023407089	-0.026295925	0.002888835	0.999933887	-0.347592589	-0.652061348	0.304468759	0.026472065
ENSG00000247081	*BAALC-AS1*	-0.60122028	-0.500550673	0.232060799	-0.732611472	0.999933887	1.90344695	2.750698431	-0.847251481	0.026631524
ENSG00000113621	*TXNDC15*	2.443594812	0.002463546	-0.159146024	0.161609571	0.999933887	-0.161398383	-0.535753581	0.374355198	0.026638275
ENSG00000153406	*NMRAL1*	3.856645127	-0.026796152	-0.091056091	0.064259939	0.999933887	-0.518192576	-0.855299937	0.337107361	0.026675481
ENSG00000005100	*DHX33*	1.788411771	-0.155842197	0.13116633	-0.287008527	0.999933887	0.096387763	-0.280797292	0.377185056	0.026791545
ENSG00000151151	*IPMK*	6.728415477	-0.049204988	-0.088970953	0.039765965	0.999933887	-0.512739097	-0.131420161	-0.381318936	0.026820293
ENSG00000146278	*PNRC1*	10.459089763	0.109929652	0.07259832	0.037331331	0.999933887	1.371562797	1.658290075	-0.286727278	0.026902793
ENSG00000105856	*HBP1*	8.117233362	-0.085388418	-0.123681719	0.038293301	0.999933887	0.124165855	0.350220747	-0.226054892	0.026902793
ENSG00000154370	*TRIM11*	4.509733445	0.016130051	0.075294788	-0.059164737	0.999933887	-0.314261085	-0.613455879	0.299194794	0.026902793
ENSG00000138814	*PPP3CA*	5.680245235	0.008010606	0.048530679	-0.040520072	0.999933887	-0.323485953	-0.624826418	0.301340464	0.026903095
ENSG00000107968	*MAP3K8*	7.591966985	0.062296146	0.048671303	0.013624843	0.999933887	1.76873989	2.194677308	-0.425937418	0.026942319
ENSG00000169105	*CHST14*	1.091991654	0.012836983	-0.047801299	0.060638283	0.999933887	0.262053607	-0.296006858	0.558060465	0.026942319
ENSG00000162444	*RBP7*	2.358970921	-0.04277004	-0.078648212	0.035878172	0.999933887	-0.590756145	-1.171690857	0.580934713	0.026971504
ENSG00000160219	*GAB3*	5.503338752	-0.021016642	-0.073889777	0.052873134	0.999933887	-0.016563766	0.222251048	-0.238814815	0.026971504
ENSG00000204520	*MICA*	2.551691045	0.019675998	-0.080160418	0.099836415	0.999933887	-0.294649024	-0.691647521	0.396998497	0.026971504
ENSG00000134851	*TMEM165*	5.128588089	-0.013729915	0.104786961	-0.118516876	0.999933887	1.029178462	1.343504284	-0.314325822	0.026971504
ENSG00000166747	*AP1G1*	7.860590566	-0.069164987	-0.066192801	-0.002972186	0.999933887	0.098607458	0.284901199	-0.186293741	0.026993043
ENSG00000114902	*SPCS1*	4.278573917	-0.004801375	-0.03759499	0.032793615	0.999933887	-0.031234419	-0.294295768	0.263061349	0.026993043
ENSG00000124731	*TREM1*	10.885557178	0.054139734	0.02914062	0.024999114	0.999933887	0.379856177	0.610148168	-0.230291991	0.027052382
ENSG00000118257	*NRP2*	-0.610079471	-0.248380656	0.214974495	-0.46335515	0.999933887	0.562641771	1.348427311	-0.78578554	0.027077296
ENSG00000043093	*DCUN1D1*	4.438150416	-0.042943384	-0.117947206	0.075003822	0.999933887	-0.03039731	0.308020942	-0.338418252	0.027081253
ENSG00000114354	*TFG*	5.135269487	-0.050984538	-0.063541182	0.012556645	0.999933887	0.387722278	0.610147048	-0.222424771	0.027087473
ENSG00000135316	*SYNCRIP*	5.503108504	-0.018135577	-0.059278408	0.041142831	0.999933887	-0.052593017	0.178016238	-0.230609255	0.027087473
ENSG00000170921	*TANC2*	5.22522522	-0.076949965	-0.003914107	-0.073035858	0.999933887	-0.593731716	-0.889864145	0.296132429	0.027106315
ENSG00000277632	*CCL3*	6.970312984	0.541102414	0.593214974	-0.05211256	0.999933887	3.172144794	3.837581046	-0.665436251	0.027136165
ENSG00000131370	*SH3BP5*	6.625175386	0.080974846	0.048867211	0.032107635	0.999933887	0.472542427	0.75698316	-0.284440733	0.027136165
ENSG00000110075	*PPP6R3*	6.395514127	-0.038109171	-0.015056086	-0.023053085	0.999933887	0.040994802	0.290705017	-0.249710215	0.027166212
ENSG00000111679	*PTPN6*	9.479280143	0.000594374	3.2031e-05	0.000562342	0.999933887	0.20051351	0.430133755	-0.229620246	0.027166212
ENSG00000055147	*FAM114A2*	2.558892233	-0.031340753	-0.069781272	0.038440519	0.999933887	-0.324657957	-0.023287826	-0.301370132	0.027177008
ENSG00000163870	*TPRA1*	6.002320644	-0.030557018	0.062552528	-0.093109546	0.999933887	1.192727646	1.5079946	-0.315266954	0.027177008
ENSG00000126767	*ELK1*	4.325625707	-0.008917352	0.016551807	-0.025469159	0.999933887	0.929261502	1.28210434	-0.352842838	0.027228949
ENSG00000168090	*COPS6*	3.461055164	0.013009222	-0.000578588	0.01358781	0.999933887	0.130268819	-0.158207134	0.288475953	0.027254581
ENSG00000120705	*ETF1*	8.804603343	-0.010529974	-0.080043392	0.069513418	0.999933887	0.387608409	0.631896222	-0.244287813	0.027254581
ENSG00000100425	*BRD1*	5.951272799	-0.015032943	0.021334551	-0.036367494	0.999933887	-0.240114742	-0.435912664	0.195797921	0.027258939
ENSG00000101199	*ARFGAP1*	6.023487788	-0.01145438	0.07517744	-0.086631821	0.999933887	-0.12324538	-0.353292367	0.230046987	0.027258939
ENSG00000149294	*NCAM1*	-0.207077974	-0.032731394	0.116311079	-0.149042473	0.999933887	-0.65253443	0.157747801	-0.810282231	0.027283129

ENSG00000168421	*RHOH*	5.40316308	0.054389957	0.068127777	-0.013737821	0.999933887	1.062607681	1.552281386	-0.489673705	0.027340245
ENSG00000136603	*SKIL*	7.349384158	-0.030730424	-0.100450071	0.069719647	0.999933887	0.707214195	1.1687041	-0.461489905	0.027342257
ENSG00000148834	*GSTO1*	6.091533262	-0.075993072	-0.063553838	-0.012439233	0.999933887	0.311680183	0.570153739	-0.258473556	0.027406255
ENSG00000139641	*ESYT1*	5.564356861	-0.032744769	0.023775868	-0.056520637	0.999933887	-0.120556724	-0.318812088	0.198255364	0.027542216
ENSG00000125827	*TMX4*	6.158531538	0.00388676	-0.038646253	0.042533014	0.999933887	-0.249618507	-0.5186946	0.269076093	0.027611924
ENSG00000137726	*FXYD6*	-0.216868585	0.084992056	-0.405310786	0.490302841	0.999933887	-0.093404796	0.425676389	-0.519081185	0.027723641
ENSG00000160325	*CACFD1*	1.427055126	0.148589717	-0.017495681	0.166085398	0.999933887	1.680826764	2.248976041	-0.568149277	0.02772805
ENSG00000141452	*RMC1*	4.939689969	0.022249148	0.018942518	0.003306631	0.999933887	-0.291330898	-0.492384792	0.201053894	0.02772805
ENSG00000104140	*RHOV*	1.952570912	-0.060530652	0.174096466	-0.234627118	0.999933887	2.043778145	2.512495086	-0.468716941	0.027763728
ENSG00000167604	*NFKBID*	8.809550491	0.106863916	0.127620512	-0.020756595	0.999933887	2.398350392	2.784424553	-0.386074161	0.027770019
ENSG00000049541	*RFC2*	2.745078108	-0.04851762	-0.097061657	0.048544038	0.999933887	-0.014174973	-0.347154229	0.332979257	0.02782417
ENSG00000013288	*MAN2B2*	5.497002783	0.051292712	0.039515064	0.011777647	0.999933887	-0.266264832	-0.509802992	0.24353816	0.027824341
ENSG00000003402	*CFLAR*	9.557156309	-0.012662443	0.003943402	-0.016605845	0.999933887	1.15427913	1.400873582	-0.246594452	0.027825157
ENSG00000135924	*DNAJB2*	5.640683026	0.054836178	0.077009433	-0.022173256	0.999933887	-0.162562632	-0.476474085	0.313911453	0.027858324
ENSG00000095787	*WAC*	8.223328264	0.00997741	-0.027289734	0.037267144	0.999933887	-0.012335491	0.169346438	-0.181681929	0.027858324
ENSG00000074370	*ATP2A3*	8.534718474	0.030157918	0.011710801	0.018447117	0.999933887	-0.541342727	-0.782863321	0.241520594	0.027936005
ENSG00000166130	*IKBIP*	3.439451933	0.048479382	-0.032290152	0.080769534	0.999933887	-0.602752585	-1.064880189	0.462127604	0.028020988
ENSG00000010244	*ZNF207*	6.992243118	-0.058766135	-0.051635428	-0.007130707	0.999933887	0.015039012	0.208463403	-0.193424391	0.028031789
ENSG00000184887	*BTBD6*	3.541812218	0.012339247	0.048293042	-0.035953795	0.999933887	0.147792988	-0.173905464	0.321698452	0.028040745
ENSG00000115520	*COQ10B*	5.994261081	0.110059368	0.123296811	-0.013237443	0.999933887	0.249369898	0.446253059	-0.196883161	0.028060422
ENSG00000250771	*AC106865.1*	4.448528007	0.044058202	0.134306684	-0.090248482	0.999933887	0.884946589	1.327256973	-0.442310384	0.028060422
ENSG00000134222	*PSRC1*	0.343393473	0.066309649	0.376637615	-0.310327965	0.999933887	-0.510188468	-1.722279833	1.212091365	0.028101224
ENSG00000131238	*PPT1*	6.27248025	0.035783463	-0.061847244	0.097630707	0.999933887	-0.614297314	-0.938035364	0.323738051	0.028112042
ENSG00000089775	*ZBTB25*	4.522176567	-0.063782218	0.104141037	-0.167923255	0.999933887	0.232247385	0.479930202	-0.247682817	0.028215892
ENSG00000236213	*AC006369.1*	1.994725247	0.135233136	0.180665245	-0.045432109	0.999933887	1.201677449	1.729393947	-0.527716498	0.028215892
ENSG00000198216	*CACNA1E*	5.032366565	0.021060631	0.020392424	0.000668207	0.999933887	0.803465737	1.042391003	-0.238925266	0.028215892
ENSG00000242299	*AC073861.1*	0.205001331	-0.022864523	-0.042374357	0.019509834	0.999933887	-0.272102461	0.367696506	-0.639798967	0.028340164
ENSG00000133055	*MYBPH*	1.694343932	0.133173781	-0.092445742	0.225619523	0.999933887	1.712189398	2.199746854	-0.487557456	0.028452773
ENSG00000114416	*FXR1*	5.306143983	-0.116557524	-0.155696737	0.039139214	0.999933887	0.031239161	0.292158111	-0.26091895	0.028480497
ENSG00000101367	*MAPRE1*	7.956613587	-0.028235899	-0.082003284	0.053767385	0.999933887	0.196021641	0.422548761	-0.226527121	0.028480497
ENSG00000123415	*SMUG1*	1.889630448	-0.076763986	0.009895813	-0.086659799	0.999933887	-0.222850855	-0.629354801	0.406503946	0.028520165
ENSG00000101158	*NELFCD*	4.171445121	-0.005479111	-0.010453755	0.004974645	0.999933887	-0.10846592	-0.350165671	0.241699751	0.028521233
ENSG00000198925	*ATG9A*	3.773298005	0.057750658	-0.042903769	0.100654427	0.999933887	-0.392024758	-0.704474304	0.312449546	0.02867175
ENSG00000250033	*SLC7A11-AS1*	2.610803083	-0.030247365	0.185742071	-0.215989437	0.999933887	-0.019635046	0.426647312	-0.446282358	0.028718519
ENSG00000126246	*IGFLR1*	1.322443301	-0.137328229	-0.239556649	0.102228421	0.999933887	-0.379627241	-0.826703857	0.447076616	0.02875246
ENSG00000135525	*MAP7*	0.807114741	-0.099615267	-0.093235051	-0.006380217	0.999933887	-0.500134181	-1.243541429	0.743407247	0.02875246
ENSG00000251022	*THAP9-AS1*	6.430508475	0.075661395	0.00098762	0.074673776	0.999933887	0.666483766	1.165797116	-0.49931335	0.02875246
ENSG00000148841	*ITPRIP*	8.966548808	0.096305839	0.071171126	0.025134714	0.999933887	0.762385575	0.983205361	-0.220819786	0.028765847
ENSG00000123545	*NDUFAF4*	0.407645214	-0.114470336	-0.153485062	0.039014726	0.999933887	0.122645475	0.778750452	-0.656104977	0.028772679
ENSG00000265982	*AC103810.3*	2.143310667	-0.039830038	-0.070380988	0.03055095	0.999933887	-0.273220889	-0.716563744	0.443342855	0.028772679
ENSG00000247400	*DNAJC3-DT*	4.816031175	0.02726392	-0.050817099	0.078081019	0.999933887	1.467904279	1.825182538	-0.357278258	0.028776839
ENSG00000151876	*FBXO4*	0.212189291	-0.303611683	-0.104915471	-0.198696211	0.999933887	-0.122787779	0.518851501	-0.64163928	0.028840794
ENSG00000105246	*EBI3*	-1.74598186	0.301893463	-0.068250629	0.370144092	0.999933887	4.701810297	6.261026685	-1.559216388	0.028840794
ENSG00000017797	*RALBP1*	6.356855476	-0.000493404	0.012410464	-0.012903867	0.999933887	-0.812255612	-1.172699427	0.360443815	0.028840794
ENSG00000173905	*GOLIM4*	0.835105923	0.07346057	-0.20003845	0.27349902	0.999933887	-0.193257221	0.41198753	-0.605244752	0.028923733
ENSG00000276317	*AL357033.3*	2.221139989	0.062012133	-0.037249821	0.099261954	0.999933887	0.435852168	0.811986432	-0.376134264	0.028979131
ENSG00000221926	*TRIM16*	-0.448005131	0.137178699	0.020268362	0.116910337	0.999933887	0.590216499	1.249948749	-0.659732249	0.029002709
ENSG00000148154	*UGCG*	5.618767513	-0.075993108	0.007474217	-0.083467325	0.999933887	-0.034195366	0.191698586	-0.225893952	0.02904515
ENSG00000151327	*FAM177A1*	5.803380246	0.090084916	0.056450104	0.033634813	0.999933887	0.583986037	0.830689317	-0.24670328	0.029182524
ENSG00000101773	*RBBP8*	2.209892558	-0.05774038	-0.158761993	0.101021614	0.999933887	0.308066307	0.768575795	-0.460509488	0.029182524
ENSG00000176170	*SPHK1*	6.75625957	0.037925572	0.11779101	-0.079865437	0.999933887	1.800685557	2.182460614	-0.381775057	0.029182524
ENSG00000204574	*ABCF1*	6.851088902	-0.002733976	0.004786921	-0.007520897	0.999933887	0.589862089	0.807216923	-0.217354835	0.029182524
ENSG00000197249	*SERPINA1*	10.491007963	0.036663322	-0.01320071	0.049864032	0.999933887	1.548245535	1.876318792	-0.328073256	0.029258576
ENSG00000168010	*ATG16L2*	9.118356401	-0.010168396	0.06009889	-0.070267285	0.999933887	-0.667749244	-0.942173032	0.274423788	0.02929866
ENSG00000130038	*CRACR2A*	3.324695522	0.052579586	0.025619637	0.026959948	0.999933887	0.116585435	-0.230685424	0.347270859	0.029375059
ENSG00000197562	*RAB40C*	3.626183309	0.038360712	0.002539285	0.035821427	0.999933887	-0.18926136	-0.53945635	0.35019499	0.029392546
ENSG00000106615	*RHEB*	6.581974685	-0.003394579	0.030440336	-0.033834914	0.999933887	0.111824145	0.291762063	-0.179937919	0.029392546
ENSG00000125772	*GPCPD1*	9.430215003	-0.082600497	-0.061042364	-0.021558133	0.999933887	-0.279509257	0.031249494	-0.310758751	0.029397973
ENSG00000092020	*PPP2R3C*	4.126277672	-0.021405736	-0.093431351	0.072025614	0.999933887	-0.071162042	0.156418194	-0.227580236	0.029397973
ENSG00000091436	*MAP3K20*	2.469289441	0.037203941	0.030037119	0.007166822	0.999933887	0.898134845	1.43438978	-0.536254936	0.029397973
ENSG00000070159	*PTPN3*	-3.252609989	-0.226081784	-0.060462637	-0.165619147	0.999933887	-0.874714948	-0.0262616	-0.848453348	0.029401057
ENSG00000149308	*NPAT*	4.477799457	-0.057919657	-0.169817927	0.11189827	0.999933887	-0.067039551	0.534800028	-0.601839579	0.029401057
ENSG00000182871	*COL18A1*	6.51769146	0.081875897	0.129682583	-0.047806686	0.999933887	-0.130906709	-0.496900174	0.365993465	0.029551184
ENSG00000134287	*ARF3*	5.811557704	0.010137517	0.00081584	0.009321677	0.999933887	-0.243067545	-0.466805499	0.223737954	0.029551184
ENSG00000142186	*SCYL1*	6.407137758	0.011207648	0.048934971	-0.037727323	0.999933887	-0.430725319	-0.665578534	0.234853216	0.029551184
ENSG00000269246	*AC011445.1*	-0.178264623	0.0071602	-0.694551223	0.701711422	0.594252256	-0.553123744	-1.444404263	0.891280519	0.029551184
ENSG00000266389	*AC002091.2*	4.476780084	0.080422154	0.002702195	0.07771996	0.999933887	1.158755984	1.472927459	-0.314171474	0.029564592
ENSG00000077157	*PPP1R12B*	5.824619487	-0.066193398	-0.04551162	-0.020681778	0.999933887	-1.381305562	-1.814788695	0.433483133	0.029575298
ENSG00000276953	*TRBV12-4*	-0.149253739	-0.017828485	0.059664381	-0.077492865	0.999933887	0.130304524	-0.4568163	0.587120824	0.029601018
ENSG00000133104	*SPART*	6.195975559	-0.080226997	-0.145966327	0.06573933	0.999933887	-0.057823996	0.190310638	-0.248134633	0.029635691
ENSG00000111670	*GNPTAB*	4.368380081	-0.035893417	-0.090560055	0.054666638	0.999933887	0.069703811	0.393384332	-0.323680521	0.029635691
ENSG00000154813	*DPH3*	4.449071074	0.012722233	-0.052572351	0.065294584	0.999933887	0.048559684	0.276748098	-0.228188414	0.029635691
ENSG00000145287	*PLAC8*	3.630684071	-0.022136938	0.043792665	-0.065929603	0.999933887	0.528729085	1.136379666	-0.607650581	0.029688437
ENSG00000141527	*CARD14*	2.982068116	0.005844668	0.184450045	-0.178605377	0.999933887	0.131466918	0.413491802	-0.282024884	0.029724969
ENSG00000175727	*MLXIP*	5.404973492	-0.018095759	-0.042370617	0.024274858	0.999933887	-0.133270035	-0.355548616	0.222278581	0.029798453
ENSG00000213676	*ATF6B*	6.046270069	0.026934465	0.055763758	-0.028829293	0.999933887	-0.299661885	-0.491454895	0.19179301	0.029831133
ENSG00000226945	*AC098935.1*	-0.811114854	0.04535464	0.364131553	-0.318776913	0.999933887	-0.307645279	-1.21920951	0.91156423	0.029831133
ENSG00000184588	*PDE4B*	7.627776399	0.088417281	0.035841002	0.052576279	0.999933887	1.377118239	1.687446562	-0.310328323	0.029837734
ENSG00000100241	*SBF1*	7.207655609	0.050587285	0.098301886	-0.047714601	0.999933887	-0.088471547	-0.299379203	0.210907656	0.029837734
ENSG00000253276	*CCDC71L*	6.93111786	0.139728299	0.115192033	0.024536266	0.999933887	0.423775035	0.738480908	-0.314705873	0.029994791
ENSG00000100749	*VRK1*	1.86790528	-0.140481854	-0.155012976	0.014531122	0.999933887	0.077558959	0.486441878	-0.408882919	0.030131048
ENSG00000136997	*MYC*	3.066514224	0.061635595	-0.010331072	0.071966667	0.999933887	0.38478685	0.799750985	-0.414964135	0.030131048
ENSG00000143624	*INTS3*	5.138486273	-0.03022332	0.002861462	-0.033084782	0.999933887	-0.22560212	-0.468376462	0.242774343	0.030131048
ENSG00000188404	*SELL*	9.535069683	-0.026502114	-0.046908192	0.020406078	0.999933887	-0.237825055	-0.551318987	0.313493932	0.030174511
ENSG00000123609	*NMI*	6.347427027	-0.068132668	-0.095599105	0.027466437	0.999933887	0.440616639	0.67899953	-0.238382891	0.030186564
ENSG00000162881	*OXER1*	3.070812076	-0.181535865	-0.06548604	-0.116050825	0.999933887	-0.861488752	-1.432416763	0.570928011	0.030189651
ENSG00000127054	*INTS11*	5.398276242	0.051869323	0.067333203	-0.01546388	0.999933887	-0.061292297	-0.28378137	0.222489073	0.03024247
ENSG00000100902	*PSMA6*	2.695934081	-0.045047747	-0.14949503	0.104447283	0.999933887	1.099580844	1.567779088	-0.468198244	0.03024247
ENSG00000129993	*CBFA2T3*	4.638933919	0.00426477	0.071959525	-0.067694754	0.999933887	-0.601249183	-0.976873605	0.375624422	0.03024247
ENSG00000132912	*DCTN4*	5.644158704	-0.04662814	-0.083676408	0.037048268	0.999933887	0.236247978	0.452947497	-0.216699519	0.030249847
ENSG00000132003	*ZSWIM4*	3.652604341	0.045345148	0.050436892	-0.005091744	0.999933887	1.347839812	1.693327065	-0.345487253	0.030262795
ENSG00000138386	*NAB1*	5.220743104	-0.04882057	-0.023211196	-0.025609374	0.999933887	0.003470625	0.238141591	-0.234670965	0.030273009
ENSG00000090615	*GOLGA3*	5.03234074	-0.008448793	0.037997909	-0.046446702	0.999933887	-0.13111075	-0.330263048	0.199152298	0.030324857
ENSG00000173928	*SWSAP1*	0.380528576	-0.029835021	0.046122941	-0.075957962	0.999933887	-0.185752882	-0.962765475	0.777012593	0.030367456
ENSG00000197321	*SVIL*	8.20503359	0.01862287	0.007849457	0.010773413	0.999933887	0.287176911	0.450437436	-0.163260525	0.030377514
ENSG00000188315	*C3orf62*	5.002783116	-0.045950064	-0.028993483	-0.016956582	0.999933887	-0.354579222	-0.539853473	0.185274251	0.030413302
ENSG00000126934	*MAP2K2*	6.406683396	0.044918935	0.006352133	0.038566803	0.999933887	-0.281771037	-0.516884937	0.2351139	0.030413302
ENSG00000131626	*PPFIA1*	5.784713007	0.000470384	0.009807997	-0.009337613	0.999933887	0.138639082	0.347934825	-0.209295742	0.030420229
ENSG00000189164	*ZNF527*	0.027149899	-0.195944944	-0.212663552	0.016718608	0.999933887	-0.218234077	0.503902691	-0.722136767	0.03045706
ENSG00000178860	*MSC*	3.355388297	0.028019461	0.009911545	0.018107916	0.999933887	1.312478375	1.71803912	-0.405560745	0.030466237
ENSG00000163823	*CCR1*	9.887855017	0.116845512	0.135949091	-0.019103579	0.999933887	0.200112948	0.405687156	-0.205574208	0.030561833
ENSG00000117174	*ZNHIT6*	1.435039469	-0.05986946	-0.209476509	0.149607049	0.999933887	-0.084431053	0.386950219	-0.471381272	0.030561833
ENSG00000165288	*BRWD3*	6.071382739	-0.056201544	-0.163365213	0.107163668	0.999933887	-0.224603342	0.286555526	-0.511158868	0.030572177
ENSG00000272333	*KMT2B*	7.266641007	0.014651933	0.05511462	-0.040462688	0.999933887	-0.429195755	-0.639089096	0.209893341	0.030572177
ENSG00000068097	*HEATR6*	2.78811974	-0.032165173	-0.02765741	-0.004507763	0.999933887	-0.400587076	-0.706329957	0.305742881	0.030640215

ENSG00000143486	*EIF2D*	5.125092421	0.10296422	0.073975873	0.028988347	0.999933887	0.522330802	0.768106375	-0.245775573	0.030661016
ENSG00000113597	*TRAPPC13*	3.158021068	-0.216399067	-0.171866865	-0.044532203	0.999933887	-0.078333601	0.396424622	-0.474758223	0.030674304
ENSG00000180113	*TDRD6*	-0.951157947	-0.062327903	-0.328412333	0.26608443	0.999933887	0.269340226	1.077118459	-0.807778234	0.030674304
ENSG00000064490	*RFXANK*	3.648508049	-0.002236937	-0.058191314	0.055954377	0.999933887	-0.04459746	-0.346583776	0.301986316	0.030717464
ENSG00000158104	*HPD*	1.880739506	0.057423179	0.032056591	0.025366588	0.999933887	0.776056846	1.223339651	-0.447282804	0.030756729
ENSG00000164463	*CREBRF*	8.284095001	-0.100230442	-0.165004254	0.064773812	0.999933887	-0.378218187	-0.044683075	-0.333535112	0.030875324
ENSG00000126353	*CCR7*	4.728193281	0.061610991	-0.020233436	0.081844427	0.999933887	0.56544941	0.854919357	-0.289469947	0.030875406
ENSG00000265206	*AC004687.1*	4.43902005	0.120426656	0.175505343	-0.055078687	0.999933887	-0.539431129	-1.157966436	0.618535307	0.030875406
ENSG00000116161	*CACYBP*	3.591687865	-0.032662175	-0.079585947	0.046923771	0.999933887	-0.161750035	-0.449492074	0.287742039	0.030875406
ENSG00000260404	*AC110079.1*	4.037165187	0.022747009	0.010467008	0.012280001	0.999933887	0.205612093	0.4931569	-0.287544807	0.030875406
ENSG00000100416	*TRMU*	3.525792501	0.013391232	0.051018317	-0.037627086	0.999933887	0.029476253	-0.261710638	0.291186891	0.030875406
ENSG00000023287	*RB1CC1*	7.404155789	-0.068859107	-0.12460343	0.055744323	0.999933887	-0.23895776	0.340688184	-0.579645944	0.030970209
ENSG00000221978	*CCNL2*	6.739280948	-0.000862874	0.04715874	-0.048021614	0.999933887	-0.176847378	-0.342706621	0.165859242	0.031038883
ENSG00000068976	*PYGM*	2.971480952	0.087189577	0.071150922	0.016038655	0.999933887	-0.254880934	-0.605500097	0.350619163	0.031071888
ENSG00000173812	*EIF1*	10.560953674	0.163682669	0.149047151	0.014635518	0.999933887	0.86208882	1.128233456	-0.266144636	0.031091103
ENSG00000110002	*VWA5A*	2.782872511	0.000537552	-0.103613162	0.104150715	0.999933887	0.435053775	0.770811872	-0.335758097	0.031091934
ENSG00000137996	*RTCA*	3.524186051	0.010809353	-0.073147185	0.083956539	0.999933887	-0.391354878	-0.691003084	0.299648206	0.031143373
ENSG00000051620	*HEBP2*	4.738362949	0.036307674	-0.015966665	0.052274339	0.999933887	-0.222960836	-0.492732819	0.269771983	0.031157431
ENSG00000102699	*PARP4*	6.344026168	-0.090863095	-0.108051751	0.017188656	0.999933887	-0.753251001	-1.025124539	0.271873538	0.031199273
ENSG00000197182	*MIRLET7BHG*	0.675226229	0.126010172	-0.062958627	0.188968799	0.999933887	-0.549343585	-1.296481909	0.747138323	0.031204046
ENSG00000111581	*NUP107*	2.670833011	-0.04484749	-0.172126003	0.127278513	0.999933887	0.058208546	0.444786009	-0.386577463	0.031214639
ENSG00000183049	*CAMK1D*	6.495419366	-0.036428086	0.024764079	-0.061192165	0.999933887	-0.107108287	-0.303018094	0.195909807	0.031253072
ENSG00000158825	*CDA*	5.845519777	0.058583924	-0.022348429	0.080932352	0.999933887	-0.411154587	-0.701087279	0.289932692	0.031440161
ENSG00000137200	*CMTR1*	5.649555729	-0.007925756	0.071506616	-0.079432372	0.999933887	-0.080518023	-0.30512602	0.224607996	0.031586254
ENSG00000182810	*DDX28*	3.15567132	-0.327471802	-0.412901535	0.085429733	0.999933887	-1.592551503	-2.31114578	0.718594277	0.031615872
ENSG00000279741	*AC007342.8*	-0.261931872	-0.208995225	-0.032124115	-0.17687111	0.999933887	-0.949662134	-2.133972132	1.184309998	0.031651392
ENSG00000122643	*NT5C3A*	4.043036051	0.027639687	-0.018872736	0.046512422	0.999933887	-0.315047657	-0.622061424	0.307013766	0.031689765
ENSG00000139572	*GPR84*	5.074439977	0.353099694	0.46972728	-0.116627587	0.999933887	2.721309665	3.137253606	-0.415943941	0.031706079
ENSG00000142546	*NOSIP*	5.149252803	0.062804639	0.003325159	0.059479479	0.999933887	-0.110214957	-0.363184067	0.25296911	0.031753288
ENSG00000270231	*NBPF8*	3.205712594	-0.120043311	-0.158822818	0.038779507	0.999933887	0.228881887	0.576100562	-0.347218675	0.031774918
ENSG00000136021	*SCYL2*	5.940746255	-0.020927549	-0.067878976	0.046951427	0.999933887	-0.003997967	0.396921447	-0.400919413	0.031774918
ENSG00000196917	*HCAR1*	5.471291761	0.12171565	0.119124437	0.002591213	0.999933887	1.898639183	2.388828955	-0.490189771	0.031814115
ENSG00000052749	*RRP12*	7.667383879	0.011831932	0.039310446	-0.027478514	0.999933887	0.114536402	-0.096999275	0.211535677	0.031814115
ENSG00000127527	*EPS15L1*	7.788438959	-0.007028247	0.016542213	-0.02357046	0.999933887	0.152030242	0.324624316	-0.172594075	0.031814115
ENSG00000196352	*CD55*	9.525884635	0.090040482	0.047575783	0.042464699	0.999933887	1.222835069	1.556775375	-0.333940306	0.031842452
ENSG00000117616	*RSRP1*	7.134819469	-0.019460637	0.04944082	-0.068901457	0.999933887	0.08340127	-0.103622188	0.187023458	0.031868235
ENSG00000172602	*RND1*	-0.720960056	-0.05473168	-0.086013215	0.031281536	0.999933887	1.338412735	2.507995608	-1.169582873	0.031870719
ENSG00000166439	*RNF169*	5.298875956	-0.042162694	-0.038978111	-0.003184582	0.999933887	-0.921481219	-1.216154283	0.294673064	0.031890046
ENSG00000171703	*TCEA2*	2.483083681	0.100829968	0.007469584	0.093360384	0.999933887	-0.237913719	-0.620111147	0.382197428	0.031899961
ENSG00000163510	*CWC22*	4.446005931	-0.098252348	-0.187918676	0.089666328	0.999933887	-0.234931996	0.243265804	-0.4781978	0.031922949
ENSG00000187650	*VMAC*	1.636787854	-0.070969114	-0.037020537	-0.033948576	0.999933887	0.049434905	-0.435003347	0.484438251	0.031922949
ENSG00000257093	*DENND11*	4.382207543	-0.003812484	-0.022270113	0.018457629	0.999933887	0.022601894	-0.182899578	0.205501472	0.031958267
ENSG00000105568	*PPP2R1A*	5.543344637	-0.020066416	0.031055605	-0.051122021	0.999933887	-0.218771884	-0.440261081	0.221489197	0.032132022
ENSG00000129235	*TXNDC17*	2.610314683	-0.031772634	0.043728313	-0.075500948	0.999933887	-0.579734628	-0.954316034	0.374581406	0.032235007
ENSG00000258860	*AL133163.2*	-1.260133867	0.389372033	0.289717709	0.099654324	0.999933887	2.784121999	3.725865026	-0.941743027	0.032292883
ENSG00000141522	*ARHGDIA*	8.407799192	0.103066505	0.041205167	0.061861337	0.999933887	0.544344274	0.783712167	-0.239367893	0.032294642
ENSG00000124795	*DEK*	5.779506005	-0.055497458	-0.146996148	0.09149869	0.999933887	-0.191547385	0.221420496	-0.412967881	0.032294642
ENSG00000169180	*XPO6*	10.244333267	0.005452308	0.020243984	-0.014791677	0.999933887	-0.770925672	-0.99514492	0.224219247	0.032379945
ENSG00000176715	*ACSF3*	3.936286557	0.058841824	-0.017182052	0.076023876	0.999933887	-0.194806724	-0.479468604	0.28466188	0.032399414
ENSG00000198886	*MT-ND4*	9.598860048	0.040284852	-0.071142358	0.111427209	0.999933887	-0.094365939	0.286135772	-0.380501711	0.032424679
ENSG00000115008	*IL1A*	4.112924074	0.537032283	0.432105003	0.10492728	0.999933887	3.002128554	4.011219782	-1.009091228	0.03245272
ENSG00000234484	*AL032821.1*	0.696247802	-0.343246624	-0.061391628	-0.281854996	0.999933887	0.049330925	0.810385937	-0.761055012	0.032521781
ENSG00000062716	*VMP1*	8.492880863	0.014124036	-0.016068283	0.03019232	0.999933887	-0.167925934	-0.467285809	0.299359875	0.032543379
ENSG00000184634	*MED12*	7.353043176	0.026411981	0.047130675	-0.020718695	0.999933887	0.476077344	0.670108672	-0.194031327	0.032563459
ENSG00000225830	*ERCC6*	3.715701941	-0.084142813	-0.107300281	0.023157468	0.999933887	-0.438211507	-0.690101586	0.251890079	0.032579325
ENSG00000115556	*PLCD4*	0.892268742	-0.049759841	-0.096199646	0.046439805	0.999933887	0.396276966	0.945523415	-0.54924645	0.032600032
ENSG00000179456	*ZBTB18*	6.223004576	-0.152154276	-0.094001906	-0.058152371	0.999933887	-0.848993684	-1.16332353	0.314329846	0.032650145
ENSG00000099381	*SETD1A*	4.55505046	-0.012512798	0.02853925	-0.041052048	0.999933887	-0.254744506	-0.457453163	0.202708656	0.032668259
ENSG00000158050	*DUSP2*	7.121739564	0.356820646	0.474767661	-0.117947015	0.999933887	1.059084881	1.628936835	-0.569851954	0.032705545
ENSG00000119414	*PPP6C*	5.63716158	-0.049590635	-0.04284541	-0.006745225	0.999933887	-0.11901644	0.056684873	-0.175701314	0.032711833
ENSG00000104325	*DECR1*	4.705664546	-0.05541657	-0.074814458	0.019397888	0.999933887	-0.218317898	-0.43392291	0.215605012	0.03278014
ENSG00000115548	*KDM3A*	7.353734537	-0.029364699	-0.047568495	0.018203796	0.999933887	-0.824220284	-1.08170249	0.257482206	0.03278014
ENSG00000103126	*AXIN1*	5.682043402	0.021190607	0.105824695	-0.084634087	0.999933887	-0.411480538	-0.688987814	0.277507275	0.032781629
ENSG00000115839	*RAB3GAP1*	4.509472726	-0.109668939	-0.148926654	0.039257715	0.999933887	-0.034963939	0.167377077	-0.202341016	0.032791564
ENSG00000126814	*TRMT5*	2.573919286	-0.006153883	-0.215041568	0.208887684	0.999933887	0.023098533	0.3332367	-0.310138168	0.032791564
ENSG00000279716	*AC006128.1*	0.55966482	-0.172170397	-0.187511661	0.015341264	0.999933887	-0.131025913	-0.618148239	0.487122326	0.032920806
ENSG00000005483	*KMT2E*	7.784650824	-0.016152491	-0.080589709	0.064437218	0.999933887	-0.107479115	0.313810347	-0.421289462	0.032920806
ENSG00000145907	*G3BP1*	5.206032487	-0.084883817	-0.049574079	-0.035309738	0.999933887	0.211245804	0.449328012	-0.238082208	0.032921696
ENSG00000104859	*CLASRP*	6.272203908	0.056697649	0.069195389	-0.01249774	0.999933887	-0.017802431	-0.266337189	0.248534758	0.033183932
ENSG00000116852	*KIF21B*	6.983516068	0.040140616	0.024676104	0.015464512	0.999933887	-0.075506137	-0.280133955	0.204627818	0.033223384
ENSG00000265298	*AC132812.1*	2.283757366	0.06709234	0.049026855	0.018065485	0.999933887	-0.068573999	-0.437998504	0.369424506	0.033337589
ENSG00000170525	*PFKFB3*	10.320894231	0.079238496	0.069699578	0.009538918	0.999933887	0.280513497	0.580569397	-0.3000559	0.033650337
ENSG00000164164	*OTUD4*	4.699515268	-0.01810288	-0.13790587	0.11980299	0.999933887	0.14363249	0.563580262	-0.419947771	0.033650337
ENSG00000283050	*GTF2IP12*	2.120218574	0.122512026	-0.040241754	0.162753779	0.999933887	0.337817375	0.852839335	-0.515021959	0.03368597
ENSG00000186496	*ZNF396*	0.718600898	0.003424146	-0.211543047	0.214967193	0.999933887	0.978419485	1.397509507	-0.419090022	0.03375094
ENSG00000169379	*ARL13B*	1.535103426	-0.080747459	-0.060937594	-0.019809864	0.999933887	0.460998631	0.940870161	-0.47987153	0.03377842
ENSG00000117000	*RLF*	6.773840677	-0.092585283	-0.171904625	0.079319342	0.999933887	0.015431915	0.470239387	-0.454807472	0.033812514
ENSG00000204843	*DCTN1*	5.304422027	-0.005661576	0.044225917	-0.049887492	0.999933887	-0.294241772	-0.571410843	0.27716907	0.033823386
ENSG00000141380	*SS18*	3.659771253	-0.061829748	0.034058362	-0.095888109	0.999933887	0.024596371	0.264426669	-0.239830297	0.033829313
ENSG00000177951	*BET1L*	4.613604417	-0.047387222	0.000344978	-0.0477322	0.999933887	-0.379055823	-0.628836822	0.249780998	0.033864645
ENSG00000231881	*AL109615.2*	-0.718744561	0.356431339	0.479236488	-0.122805149	0.999933887	1.457342726	2.405896081	-0.948553355	0.033876274
ENSG00000183401	*CCDC159*	3.963065832	0.04498864	-0.034121733	0.079110374	0.999933887	0.446259191	0.692444992	-0.246185801	0.033943928
ENSG00000186222	*BLOC1S4*	2.563751902	0.046272157	0.042268113	0.004004044	0.999933887	-0.27501136	-0.702998873	0.427987514	0.033993912
ENSG00000163785	*RYK*	3.757428399	-0.092157685	0.052109101	-0.144266786	0.999933887	-0.431535419	-0.697841662	0.266306243	0.03410294
ENSG00000161533	*ACOX1*	5.536474023	-0.093248206	-0.028797616	-0.064450591	0.999933887	-0.925636175	-1.269990123	0.344353948	0.03410294
ENSG00000185825	*BCAP31*	6.805333697	-0.025051967	-0.078659242	0.053607275	0.999933887	-0.540617823	-0.777063939	0.236446116	0.034121331
ENSG00000172831	*CES2*	3.410937211	0.026244797	0.094952837	-0.06870804	0.999933887	-0.216607689	-0.480394977	0.263787288	0.034167854
ENSG00000168152	*THAP9*	3.11556583	0.037303917	-0.199346689	0.236650605	0.999933887	0.818472363	1.420647525	-0.602175162	0.034232015
ENSG00000276600	*RAB7B*	-1.338277705	0.22714424	-0.283729729	0.510873969	0.999933887	-1.97347026	-3.121916597	1.148446336	0.034285671
ENSG00000139218	*SCAF11*	7.614144916	-0.065241804	-0.06917817	0.003936367	0.999933887	-0.665888666	-0.151371608	-0.514517058	0.03433521
ENSG00000122862	*SRGN*	12.531072875	0.016650551	-0.035627056	0.052277608	0.999933887	0.619955581	0.845272171	-0.22531659	0.034362135
ENSG00000164134	*NAA15*	3.305556749	-0.002808893	-0.134892528	0.132083636	0.999933887	-0.037690135	0.339037735	-0.37672787	0.034378872
ENSG00000089351	*GRAMD1A*	7.963173787	0.094810986	0.119646003	-0.024835017	0.999933887	2.713272322	3.167904002	-0.45463168	0.034390081
ENSG00000154265	*ABCA5*	3.728682701	-0.172871752	-0.059093517	-0.113778235	0.999933887	0.221031046	0.842818016	-0.62178697	0.034402516
ENSG00000091164	*TXNL1*	5.330234733	-0.039259066	-0.007628648	-0.031630418	0.999933887	0.057231768	0.239781699	-0.182549931	0.034402516
ENSG00000134571	*MYBPC3*	5.196233955	0.025833465	0.038962581	-0.013129115	0.999933887	0.748319979	1.026578085	-0.278258106	0.034446167
ENSG00000131471	*AOC3*	2.939038824	0.063774986	0.030605585	0.0331694	0.999933887	1.269166022	0.618975502	0.65019052	0.034503966
ENSG00000101336	*HCK*	8.739991495	0.083492994	0.042541808	0.040951186	0.999933887	1.334102286	1.62566326	-0.291560975	0.03451867
ENSG00000120694	*HSPH1*	4.19923435	-0.071188027	-0.145410315	0.074222288	0.999933887	0.068296555	0.515727572	-0.447431017	0.034540634
ENSG00000160703	*NLRX1*	3.201496224	-0.041157476	-0.068072757	0.026915281	0.999933887	-0.414472557	-0.803304453	0.388831895	0.034540634
ENSG00000077044	*DGKD*	6.053586367	0.015700919	0.035389359	-0.01968844	0.999933887	-0.092609564	-0.27443432	0.181824756	0.034540634
ENSG00000197121	*PGAP1*	1.864337581	-0.060931634	-0.058581943	-0.002349691	0.999933887	0.60829257	1.276565648	-0.668273077	0.034540634
ENSG00000165029	*ABCA1*	8.964618122	-0.028826346	-0.086289809	0.057463463	0.999933887	0.058624941	0.402301722	-0.343676781	0.034540634
ENSG00000267317	*AC027307.2*	0.891423977	-0.037728309	0.069902752	-0.107631061	0.999933887	-0.179872025	0.278388702	-0.458260727	0.034540634
ENSG00000279672	*AP006621.5*	-0.084844248	0.156512865	0.086192183	0.070320682	0.999933887	-0.375462024	-1.036845194	0.66138317	0.034540983

ENSG00000185420	*SMYD3*	0.477878309	0.035779436	-0.057722036	0.093501472	0.999933887	0.235599299	-0.499172626	0.734771925	0.034540983
ENSG00000175482	*POLD4*	3.013585577	0.155009581	0.000511213	0.154498368	0.999933887	0.133661211	-0.197964158	0.331625369	0.034559396
ENSG00000138767	*CNOT6L*	6.054222846	0.048142846	-0.031513635	0.079656481	0.999933887	-0.436054445	-0.772943971	0.336889526	0.034559396
ENSG00000068724	*TTC7A*	4.181492799	0.091762807	0.029968056	0.06179475	0.999933887	0.462171696	0.724922122	-0.262750427	0.034637154
ENSG00000123143	*PKN1*	6.435825321	0.044488761	0.052645064	-0.008156303	0.999933887	-0.12661696	-0.417004351	0.290387391	0.034637154
ENSG00000174837	*ADGRE1*	6.026691148	-0.014774505	-0.003589188	-0.011185317	0.999933887	1.346116092	1.785783328	-0.439667236	0.034637154
ENSG00000122547	*EEPD1*	5.792467514	0.003449602	-0.021936231	0.025385833	0.999933887	-0.309655773	-0.091284854	-0.21837092	0.034836165
ENSG00000174132	*FAM174A*	3.530542798	0.12366471	0.144910919	-0.021246209	0.999933887	-0.933442756	-1.371725408	0.438282652	0.034872204
ENSG00000214872	*SMTNL1*	2.270307061	-0.02850355	-0.004194898	-0.024308652	0.999933887	-0.276190826	-0.768276116	0.49208529	0.034931127
ENSG00000256683	*ZNF350*	4.437753193	-0.106549857	-0.146722158	0.040172301	0.999933887	-0.191468285	0.056552041	-0.248020326	0.034962796
ENSG00000133773	*CCDC59*	4.497016273	-0.043168173	-0.024759242	-0.018408932	0.999933887	0.113044172	0.351820066	-0.238775894	0.034962796
ENSG00000162695	*SLC30A7*	3.945889345	-0.075361611	0.018844274	-0.094205886	0.999933887	0.431873255	0.791464411	-0.359591156	0.034962796
ENSG00000269556	*TMEM185A*	1.352592215	-0.034706914	0.090487902	-0.125194816	0.999933887	-0.292723892	-0.726549092	0.4338252	0.034962796
ENSG00000136286	*MYO1G*	6.6781501	0.029811128	0.048349565	-0.018538437	0.999933887	0.010008911	-0.208898372	0.218907283	0.03502988
ENSG00000108671	*PSMD11*	4.709674895	-0.09816317	-0.128349121	0.030185951	0.999933887	0.027365935	0.237892553	-0.210526618	0.035039773
ENSG00000271614	*ATP2B1-AS1*	8.017007494	0.271278348	0.111808059	0.15947029	0.999933887	1.649722673	2.115827284	-0.46610461	0.035040639
ENSG00000134086	*VHL*	6.142697693	-0.059179605	0.01132719	-0.070506795	0.999933887	1.098531806	1.392351407	-0.293819601	0.035040639
ENSG00000111269	*CREBL2*	3.599416038	0.047184659	-0.13863836	0.185823019	0.999933887	0.198584233	0.502718124	-0.304133891	0.035040639
ENSG00000132612	*VPS4A*	3.69762588	-0.037034851	0.056003207	-0.093038059	0.999933887	-0.084275621	-0.352253013	0.267977391	0.035040639
ENSG00000118707	*TGIF2*	5.280637076	-0.015865584	0.014623008	-0.030488593	0.999933887	1.031059356	1.306356335	-0.27529698	0.035040639
ENSG00000203993	*ARRDC1-AS1*	2.092810245	0.022294256	0.136361612	-0.114067356	0.999933887	-0.13425199	-0.488870865	0.354618875	0.035040639
ENSG00000030419	*IKZF2*	2.520092419	-0.111976588	-0.192373227	0.080396639	0.999933887	-0.220495237	0.268445855	-0.488941091	0.035204207
ENSG00000160932	*LY6E*	5.971800443	0.064501432	0.004006975	0.060494457	0.999933887	-0.189086245	-0.556943462	0.367857217	0.035204207
ENSG00000104856	*RELB*	7.532186948	0.037629261	0.014075023	0.023554238	0.999933887	0.945722556	1.17267544	-0.226952883	0.035266601
ENSG00000001167	*NFYA*	5.913879378	0.065310351	-0.078970987	0.144281338	0.944094537	0.196279932	0.384912608	-0.188632676	0.03534404
ENSG00000136381	*IREB2*	5.257687957	-0.056852794	-0.084254932	0.027402137	0.999933887	-0.306885286	-0.041820142	-0.265065144	0.03534404
ENSG00000135677	*GNS*	7.936315518	-0.042442186	-0.019985209	-0.022456977	0.999933887	0.070503485	0.280657235	-0.210153751	0.03534404
ENSG00000284690	*CD300H*	1.944277336	-0.047599805	-0.017067398	-0.030532407	0.999933887	-0.98094749	-1.808529744	0.827582254	0.03534404
ENSG00000213976	*AC010615.1*	0.285649835	-0.22032318	-0.222168732	0.001845553	0.999933887	-0.69834931	0.207028189	-0.905377499	0.035426851
ENSG00000003400	*CASP10*	3.297098696	-0.057325526	0.03504834	-0.092373866	0.999933887	-0.330042601	-0.663929466	0.333886864	0.035426851
ENSG00000285865	*AC010285.3*	2.26729959	-0.009801275	0.182595943	-0.192397218	0.999933887	-0.019101319	0.38428057	-0.403381889	0.035426851
ENSG00000119139	*TJP2*	-0.019803427	0.097834984	-0.120801504	0.218636488	0.999933887	1.296947239	2.043029297	-0.746082058	0.035528714
ENSG00000133243	*BTBD2*	4.478071572	0.020887573	0.069286165	-0.048398592	0.999933887	-0.040058721	-0.308441956	0.268383235	0.035528714
ENSG00000134265	*NAPG*	4.309746782	-0.054853765	-0.072512149	0.017658384	0.999933887	-0.691309802	-1.001970832	0.31066103	0.035627819
ENSG00000071889	*FAM3A*	3.601735092	0.018272186	0.069484334	-0.051212148	0.999933887	0.119743769	-0.129668508	0.249412277	0.035714739
ENSG00000177683	*THAP5*	3.753969918	0.081750803	-0.089778389	0.171529192	0.999933887	-0.346486915	0.096842308	-0.443329224	0.035730428
ENSG00000140612	*SEC11A*	4.864168437	-0.016919666	-0.034753261	0.017833595	0.999933887	-0.305723815	-0.54953751	0.243813696	0.035808948
ENSG00000111859	*NEDD9*	8.238145784	0.000857952	0.011489001	-0.010631049	0.999933887	0.100388419	0.286211538	-0.185823119	0.035880303
ENSG00000254979	*AP000781.2*	-1.148804937	0.28139041	0.387950834	-0.106560424	0.999933887	1.05840113	1.857192495	-0.798791366	0.035889278
ENSG00000183307	*TMEM121B*	1.674257012	0.02159567	-0.142906208	0.164501878	0.999933887	-0.941508449	-1.738557024	0.797048575	0.035889278
ENSG00000113387	*SUB1*	6.633090459	-0.032801491	-0.024302802	-0.008498689	0.999933887	0.186601636	0.382796276	-0.19619464	0.035922918
ENSG00000250274	*AC034199.1*	5.529156836	0.204361681	0.236763877	-0.032402196	0.999933887	1.64185734	2.021444266	-0.379586926	0.035996623
ENSG00000179361	*ARID3B*	5.929178922	0.054177983	0.019986111	0.034191872	0.999933887	-1.172068958	-1.483901204	0.311832246	0.036021068
ENSG00000145016	*RUBCN*	4.005101322	0.084715372	0.082029669	0.002685703	0.999933887	-0.316068437	-0.619552957	0.30348452	0.036028327
ENSG00000172663	*TMEM134*	2.916224148	0.056838364	-0.016105916	0.07294428	0.999933887	-0.03281855	-0.288132898	0.255314348	0.036028327
ENSG00000115652	*UXS1*	3.174509607	-0.033771729	-0.123583631	0.089811902	0.999933887	0.363190923	0.691846286	-0.328655363	0.036028327
ENSG00000276980	*AC008760.2*	0.50598261	-0.144570551	0.213278408	-0.357848959	0.999933887	3.059205157	3.915064563	-0.855859405	0.036078161
ENSG00000266053	*NDUFV2-AS1*	2.860557894	-0.095096892	0.015888762	-0.110985655	0.999933887	0.662034539	0.986039439	-0.3240049	0.036153348
ENSG00000102962	*CCL22*	0.521486115	-0.230904106	-0.123525459	-0.107378647	0.999933887	0.726268078	1.766411885	-1.040143807	0.036153348
ENSG00000166197	*NOLC1*	4.705767196	0.024608924	-0.056283014	0.080891938	0.999933887	0.529167617	0.842695843	-0.313528226	0.036153348
ENSG00000169155	*ZBTB43*	5.679438911	-0.058623939	-0.048168772	-0.010455167	0.999933887	0.274996469	0.501018509	-0.22602204	0.036164762
ENSG00000103168	*TAF1C*	4.586824292	0.083043367	0.057875238	0.025168129	0.999933887	0.5624122	0.989613973	-0.427201773	0.036164762
ENSG00000185670	*ZBTB3*	0.082383335	-0.046094893	-0.044493556	-0.001601338	0.999933887	-0.34654289	-1.011851223	0.665308333	0.036164762
ENSG00000152689	*RASGRP3*	1.680447094	-0.052669597	-0.042734105	-0.009935492	0.999933887	0.334135012	0.76617972	-0.432044709	0.036222941
ENSG00000126266	*FFAR1*	0.172151599	0.04515907	0.256191935	-0.211032865	0.999933887	0.407099685	0.889673025	-0.48257334	0.03633022
ENSG00000135090	*TAOK3*	5.936358036	-0.002199146	-0.028337809	0.026138663	0.999933887	-0.470718084	-0.694907128	0.224189045	0.03633022
ENSG00000125871	*MGME1*	3.763804335	-0.089433184	-0.098920113	0.009486929	0.999933887	-1.124019113	-1.452533352	0.328514239	0.036380737
ENSG00000135953	*MFSD9*	2.587313217	0.068627474	0.103057294	-0.03442982	0.999933887	-0.22034721	-0.594181169	0.373833959	0.036389234
ENSG00000126759	*CFP*	4.488146278	0.074533103	0.048286599	0.026246504	0.999933887	1.446841454	1.866351101	-0.419509647	0.036389234
ENSG00000174197	*MGA*	6.421327088	-0.054877192	-0.027579936	-0.027297255	0.999933887	-0.229358407	0.084761406	-0.314119813	0.036389234
ENSG00000173511	*VEGFB*	3.619746456	-0.006003471	-0.069466866	0.063463394	0.999933887	-0.540024674	-0.835290049	0.295265375	0.036389234
ENSG00000144840	*RABL3*	2.488460914	-0.053857976	-0.025689467	-0.028168509	0.999933887	0.215836649	0.573196891	-0.357360242	0.036410497
ENSG00000231925	*TAPBP*	8.897363357	0.029527643	0.011752425	0.017775218	0.999933887	0.686997601	0.887085924	-0.200088324	0.036410497
ENSG00000128513	*POT1*	1.89655697	-0.030783386	-0.249492271	0.218708885	0.999933887	-0.070708989	0.462083685	-0.532792674	0.036477901
ENSG00000160785	*SLC25A44*	5.641907676	-0.061547147	-0.009324	-0.052223147	0.999933887	-0.470715149	-0.743813038	0.273097888	0.036628458
ENSG00000127957	*PMS2P3*	0.977790349	0.004147857	0.165128816	-0.160980959	0.999933887	-0.061103414	-0.494325185	0.433221771	0.036632372
ENSG00000126705	*AHDC1*	3.264292264	0.02905989	-0.060801364	0.089861255	0.999933887	0.28565415	0.589093628	-0.303439479	0.036694605
ENSG00000136630	*HLX*	3.406635524	-0.07872767	-0.025147541	-0.053580129	0.999933887	-0.409245426	-0.840642713	0.431397286	0.036873835
ENSG00000110048	*OSBP*	5.742851804	-0.000743092	-0.058363664	0.057620572	0.999933887	0.223695706	0.404522254	-0.180826548	0.03690754
ENSG00000198431	*TXNRD1*	8.74840389	-0.045867875	-0.040116862	-0.005751013	0.999933887	-0.032461986	0.210828298	-0.243290284	0.036916268
ENSG00000167645	*YIF1B*	2.81328713	0.085374992	0.063660788	0.021714203	0.999933887	-0.007212787	-0.338898987	0.331686201	0.036980533
ENSG00000197150	*ABCB8*	3.118203869	0.019737541	0.064351855	-0.044614314	0.999933887	0.219133144	0.494513059	-0.275379915	0.036980533
ENSG00000130254	*SAFB2*	6.619125599	0.01148201	0.062609902	-0.051127893	0.999933887	-0.298713028	-0.518879644	0.220166616	0.036983222
ENSG00000162302	*RPS6KA4*	4.117005598	0.110373939	0.079230294	0.031143645	0.999933887	-0.059731316	-0.381497837	0.321766522	0.037149672
ENSG00000123684	*LPGAT1*	6.082704723	-0.038910041	-0.032265897	-0.006644144	0.999933887	-0.442041553	-0.675698936	0.233657383	0.037164507
ENSG00000078401	*EDN1*	6.663003544	0.382842799	0.335520828	0.047321972	0.999933887	1.167521557	1.735906169	-0.568384612	0.03723299
ENSG00000137817	*PARP6*	5.194070126	0.004952859	-0.001800615	0.006753474	0.999933887	0.547521367	0.771977225	-0.224455858	0.037246429
ENSG00000137601	*NEK1*	2.070152974	-0.020538074	0.069118967	-0.089657041	0.999933887	-0.136056341	0.337958091	-0.474014432	0.037346794
ENSG00000145740	*SLC30A5*	3.544827299	-0.070862044	-0.000474833	-0.070387211	0.999933887	0.361722207	0.619078802	-0.257356595	0.037351418
ENSG00000081041	*CXCL2*	8.448447411	0.70231544	0.853543872	-0.151228432	0.999933887	2.433314688	3.089181656	-0.655866968	0.037392822
ENSG00000287691	*AL031602.2*	2.413446423	-0.102938852	-0.066711133	-0.036227719	0.999933887	1.180267817	0.708150622	0.472117195	0.037392822
ENSG00000135932	*CAB39*	6.231970294	0.014117076	-0.00782669	0.021943767	0.999933887	-0.570453483	-0.82634509	0.255891607	0.037392822
ENSG00000105186	*ANKRD27*	3.876808217	0.007166047	0.053791371	-0.046625323	0.999933887	-0.323668628	-0.578426908	0.25475828	0.037392822
ENSG00000099804	*CDC34*	5.400532216	0.034355363	0.042491285	-0.008135922	0.999933887	-0.167408147	-0.454955927	0.28754778	0.037435286
ENSG00000169136	*ATF5*	2.622096221	0.013088227	0.027529441	-0.014441215	0.999933887	0.455693876	0.802411059	-0.346717183	0.037594335
ENSG00000119866	*BCL11A*	2.624993466	-0.011388697	0.016274719	-0.027663417	0.999933887	1.906474438	2.53014438	-0.623669942	0.037613669
ENSG00000160991	*ORAI2*	7.204821415	-0.004219047	0.022653503	-0.02687255	0.999933887	-0.526423444	-0.789114828	0.262691384	0.037613669
ENSG00000113593	*PPWD1*	4.23542264	-0.122979855	-0.158752668	0.035772813	0.999933887	-0.325432457	0.11833213	-0.443764587	0.037650585
ENSG00000172292	*CERS6*	2.083345583	0.093267572	-0.020820414	0.114087986	0.999933887	0.253664774	0.590191397	-0.336526623	0.037728313
ENSG00000099875	*MKNK2*	8.508315248	0.033212001	0.049671519	-0.016459518	0.999933887	0.013142593	-0.176586005	0.189728598	0.037728313
ENSG00000259717	*LINC00677*	1.542234371	0.046171118	-0.026493873	0.072664991	0.999933887	0.540492697	0.095519413	0.444973284	0.037728313
ENSG00000232915	*RNF14P1*	-1.075489474	0.20097768	0.237311018	-0.036333338	0.999933887	0.97907046	1.772780049	-0.793709589	0.037728313
ENSG00000115165	*CYTIP*	9.00177402	0.017558581	-0.024233434	0.041792016	0.999933887	-0.539795758	-0.304115887	-0.235679871	0.037728313
ENSG00000136930	*PSMB7*	4.881497601	0.01618617	-0.013252777	0.029438947	0.999933887	-0.340164902	-0.565999213	0.225834311	0.037728313
ENSG00000181704	*YIPF6*	4.680317866	0.060812235	0.068596366	-0.007784131	0.999933887	0.406197415	0.632018642	-0.225821227	0.03777165
ENSG00000109689	*STIM2*	4.75232685	0.008854757	-0.000827891	0.009682648	0.999933887	-0.320580698	-0.629934186	0.309353488	0.03777165
ENSG00000197102	*DYNC1H1*	7.208123998	-0.046294563	-0.061795863	0.0155013	0.999933887	-0.477248359	-0.67232722	0.195078861	0.037804339
ENSG00000285165	*AC124014.1*	0.414543395	-0.102672942	-0.022461855	-0.080211087	0.999933887	-0.06789036	0.613328824	-0.681219183	0.037804339
ENSG00000262152	*LINC00514*	0.13297036	-0.093489591	-0.08147739	-0.012012201	0.999933887	-1.63405975	-2.517579286	0.883519536	0.037804339
ENSG00000173517	*PEAK1*	3.091997903	-0.123028776	0.016478223	-0.139506999	0.999933887	-0.287492525	0.048584113	-0.336076638	0.037859091
ENSG00000187605	*TET3*	8.197580847	-0.019470546	-0.009426605	-0.010043941	0.999933887	0.409646747	0.60882552	-0.199178773	0.037859091
ENSG00000136052	*SLC41A2*	-0.383998529	-0.033658306	0.15133915	-0.184997457	0.999933887	0.144511724	0.727497304	-0.58298558	0.037859091
ENSG00000030110	*BAK1*	3.274567674	0.000667453	-0.070380232	0.071047685	0.999933887	-0.210152146	-0.525876034	0.315723888	0.037859091
ENSG00000105404	*RABAC1*	2.449520563	-0.044156136	0.046810855	-0.090966991	0.999933887	-0.192730226	-0.620667288	0.427937062	0.037872775
ENSG00000075785	*RAB7A*	9.459309857	0.012884341	-0.036303167	0.049187508	0.999933887	0.205268462	0.398078566	-0.192810104	0.038036248

ENSG00000005187	*ACSM3*	0.277885133	-0.074689603	-0.097587878	0.022898275	0.999933887	0.226338802	-0.35005954	0.576398342	0.038049241
ENSG00000109079	*TNFAIP1*	2.867449544	-0.109706168	-0.029920669	-0.079785499	0.999933887	-0.057215478	-0.304894116	0.247678639	0.038097499
ENSG00000111737	*RAB35*	7.255998033	0.028381388	0.017071335	0.011310052	0.999933887	-0.083484494	0.110281069	-0.193765564	0.038097499
ENSG00000124074	*ENKD1*	1.655704725	0.024813061	0.20978052	-0.184967459	0.999933887	-0.277730142	-0.809097991	0.531367849	0.038097499
ENSG00000124784	*RIOK1*	2.874801743	-0.06170514	0.081176438	-0.142881578	0.999933887	0.377240329	0.726311089	-0.34907076	0.038105685
ENSG00000135999	*EPC2*	5.309796269	-0.034101474	-0.013550215	-0.020551259	0.999933887	-0.294927607	-0.080822101	-0.214105506	0.038171621
ENSG00000092439	*TRPM7*	5.988256836	-0.083190281	-0.057643561	-0.02554672	0.999933887	0.124626766	0.536724422	-0.412097656	0.038227686
ENSG00000108588	*CCDC47*	4.757043993	-0.033140555	-0.099564472	0.066423917	0.999933887	-0.573334514	-0.837558986	0.264224472	0.038262277
ENSG00000112053	*SLC26A8*	3.53621163	-0.029877158	0.032416122	-0.062293281	0.999933887	-0.138848953	0.19098233	-0.329831283	0.038262277
ENSG00000165684	*SNAPC4*	3.326377313	0.073049617	0.105210833	-0.032161216	0.999933887	0.095078717	-0.234356016	0.329434734	0.038292207
ENSG00000234925	*ATP5PDP4*	0.030319068	0.11989642	0.193801045	-0.073904625	0.999933887	0.246397199	1.136873845	-0.890476646	0.038320123
ENSG00000061337	*LZTS1*	0.969788753	0.06612284	0.284414972	-0.218292133	0.999933887	0.690449165	1.427043334	-0.73659417	0.038376024
ENSG00000076003	*MCM6*	4.36610633	-0.177817119	-0.10643899	-0.071378129	0.999933887	-1.268824971	-1.631702366	0.362877394	0.03841788
ENSG00000110011	*DNAJC4*	3.193641641	0.018225868	-0.021447406	0.039673275	0.999933887	-0.132822533	-0.417226511	0.284403978	0.038454421
ENSG00000187808	*SOWAHD*	-0.384144231	-0.017584507	0.059172054	-0.076756561	0.999933887	-0.641646846	-1.572986781	0.931339935	0.038476081
ENSG00000101972	*STAG2*	7.445464208	-0.097877795	-0.15957699	0.061699195	0.999933887	-0.315180883	0.185017066	-0.500197949	0.038519063
ENSG00000280620	*SCAANT1*	-0.368163024	-0.002841994	0.10922265	-0.112064644	0.999933887	0.495862184	-0.46919347	0.965055654	0.038519063
ENSG00000117385	*P3H1*	2.940931826	0.083303322	0.134230498	-0.050927176	0.999933887	0.059625707	-0.293165534	0.352791241	0.038657756
ENSG00000172548	*NIPAL4*	1.320426973	0.074932376	-0.173419924	0.248352299	0.999933887	2.763948884	3.391276579	-0.627327695	0.038657756
ENSG00000117748	*RPA2*	5.148720862	0.000549882	-0.055592321	0.056142203	0.999933887	0.235506869	0.451948847	-0.216441978	0.03874718
ENSG00000152056	*AP1S3*	1.516415363	-0.090446299	-0.44014185	0.349695552	0.999933887	2.966835908	3.466615723	-0.499779815	0.03876282
ENSG00000177606	*JUN*	8.300620413	0.263597669	0.269293096	-0.005695427	0.999933887	0.260876615	0.585337295	-0.32446068	0.038862418
ENSG00000276070	*CCL4L2*	9.240247983	0.517400761	0.444964577	0.072436184	0.999933887	3.149059254	3.782706858	-0.633647604	0.03888051
ENSG00000168288	*MMADHC*	5.654450953	-0.06632344	-0.016317388	-0.050006052	0.999933887	0.049563731	0.228385176	-0.178821446	0.038936861
ENSG00000054267	*ARID4B*	7.163592179	-0.073857167	-0.146021202	0.072164036	0.999933887	-0.132874137	0.375958723	-0.508832861	0.038961611
ENSG00000169972	*PUSL1*	1.550363113	0.01057681	0.106495885	-0.095919076	0.999933887	-0.003280717	-0.513436373	0.510155656	0.039009377
ENSG00000175354	*PTPN2*	4.530904234	-0.035098984	-0.077724741	0.042625757	0.999933887	0.046125063	0.326145077	-0.280020014	0.039078823
ENSG00000135052	*GOLM1*	0.787334351	-0.053172483	-0.240511138	0.187338655	0.999933887	-0.096344011	0.507783495	-0.604127506	0.039112766
ENSG00000197498	*RPF2*	2.251992715	-0.033161832	-0.113851404	0.080689572	0.999933887	0.775751418	1.322788314	-0.547036896	0.039118603
ENSG00000186891	*TNFRSF18*	1.083264534	0.265002688	-0.030257316	0.295260003	0.999933887	1.010703254	1.650913468	-0.640210214	0.039187757
ENSG00000166200	*COPS2*	4.10972635	-0.104013038	-0.237506536	0.133493497	0.999933887	-0.295140462	0.049870507	-0.345010968	0.039187757
ENSG00000121481	*RNF2*	2.550880347	-0.014746844	-0.05758981	0.042842966	0.999933887	0.050683897	0.316687059	-0.266003162	0.039276597
ENSG00000196187	*TMEM63A*	5.725801727	0.008805595	0.031972157	-0.023166562	0.999933887	-0.037845172	-0.222946161	0.185100989	0.039299501
ENSG00000112658	*SRF*	6.896233605	0.196875694	0.172283555	0.024592139	0.999933887	0.387309731	0.687307433	-0.299997702	0.039460153
ENSG00000033100	*CHPF2*	6.39921544	-0.036790984	-0.021051681	-0.015739303	0.999933887	-0.643125454	-0.88271561	0.239590155	0.039460153
ENSG00000104852	*SNRNP70*	7.50641917	0.043014542	0.124523575	-0.081509033	0.999933887	0.000879929	-0.241256966	0.242136894	0.039525813
ENSG00000255274	*SMIM35*	1.026464795	0.104423562	-0.095536376	0.199959937	0.999933887	0.780888859	1.544843022	-0.763954163	0.039525813
ENSG00000078114	*NEBL*	2.500892749	0.054074988	-0.129794906	0.183869893	0.999933887	-0.035889176	0.354707982	-0.390597158	0.039525813
ENSG00000177173	*NAP1L4P1*	3.911429808	0.00785226	0.008097347	-0.000245087	0.999933887	1.205954837	1.738760705	-0.532805868	0.039525813
ENSG00000169508	*GPR183*	4.446049788	0.039562246	-0.000854527	0.040416772	0.999933887	0.014480125	0.309135286	-0.294655161	0.039553484
ENSG00000160949	*TONSL*	1.390734075	-0.014192159	0.209249135	-0.223441294	0.999933887	0.455052638	1.008601101	-0.553548463	0.039553484
ENSG00000153395	*LPCAT1*	10.223361221	-0.017967587	-0.01711852	-0.000849067	0.999933887	0.852770588	1.093707331	-0.240936743	0.039596599
ENSG00000140474	*ULK3*	3.777135799	-0.04683857	0.037897435	-0.084736005	0.999933887	0.079243137	-0.147815891	0.227059027	0.03979001
ENSG00000117475	*BLZF1*	3.659908892	-0.134053568	-0.240320817	0.106267249	0.999933887	-0.519389397	-0.176528346	-0.342861051	0.039810051
ENSG00000275560	*AC008115.3*	1.815142288	-0.119468404	-0.175743955	0.056275551	0.999933887	-0.438434603	-0.923041191	0.484606587	0.039810051
ENSG00000142082	*SIRT3*	2.54198914	0.082936543	0.021188376	0.061748167	0.999933887	0.03813146	-0.287196679	0.32532814	0.039810051
ENSG00000178585	*CTNNBIP1*	1.804158121	0.099011135	0.126232964	-0.027221829	0.999933887	-0.491415817	-1.099654782	0.608238965	0.039810051
ENSG00000087301	*TXNDC16*	3.366498884	-0.068294982	0.064814373	-0.133109355	0.999933887	0.415022303	0.937670102	-0.5226478	0.039810051
ENSG00000225783	*MIAT*	5.870358628	-0.024328177	0.035715832	-0.060044009	0.999933887	-0.114608284	-0.352350962	0.237742678	0.039810051
ENSG00000169403	*PTAFR*	9.741022084	-0.087680156	-0.09846753	0.010787373	0.999933887	1.351019414	1.686256763	-0.335237349	0.040017099
ENSG00000285103	*AL451123.1*	4.148794353	-0.060817246	-0.039467755	-0.021349491	0.999933887	-0.032888036	0.500748183	-0.533636219	0.040106542
ENSG00000065882	*TBC1D1*	5.883739899	-0.012693322	0.003110894	-0.015804216	0.999933887	0.051768636	-0.135383789	0.187152425	0.040106542
ENSG00000124422	*USP22*	6.019585763	-0.037312767	-0.017598012	-0.019714755	0.999933887	-0.423346415	-0.626751949	0.203405534	0.040125712
ENSG00000150995	*ITPR1*	5.652554442	-0.036004531	-0.104596996	0.068592464	0.999933887	0.222244421	0.425384611	-0.20314019	0.040125712
ENSG00000198915	*RASGEF1A*	3.360194456	0.03388617	-0.13370993	0.1675961	0.999933887	-1.467139227	-1.869881802	0.402742575	0.040125712
ENSG00000165609	*NUDT5*	3.774675915	-0.011491062	-0.07671821	0.065227149	0.999933887	-0.150423325	-0.423882323	0.273458998	0.040170964
ENSG00000162888	*C1orf147*	-0.473412741	-0.034344238	0.289298661	-0.323642899	0.999933887	1.052139932	2.082189607	-1.030049674	0.040170964
ENSG00000156860	*FBRS*	9.474317995	0.099483077	0.085439843	0.014043234	0.999933887	0.696879323	0.903928994	-0.207049671	0.04018161
ENSG00000089041	*P2RX7*	2.902054572	-0.010810949	-0.028747524	0.017936575	0.999933887	1.274496291	1.740922545	-0.466426254	0.040233858
ENSG00000128159	*TUBGCP6*	5.877894949	0.026893456	0.07149006	-0.044596604	0.999933887	0.042204039	-0.146773238	0.188977277	0.040364118
ENSG00000059377	*TBXAS1*	7.293073321	-0.048148296	-0.042745835	-0.005402461	0.999933887	-1.096822545	-1.451976683	0.355154138	0.040412442
ENSG00000130150	*MOSPD2*	5.224793381	-0.034408722	-0.117237553	0.082828831	0.999933887	-0.60468588	-0.242697153	-0.361988728	0.040511434
ENSG00000137312	*FLOT1*	8.472147632	0.097559232	0.053794224	0.043765008	0.999933887	1.135471223	1.434217035	-0.298745812	0.040607178
ENSG00000136819	*C9orf78*	5.47815194	-0.034609451	-0.076253557	0.041644106	0.999933887	-0.380176138	-0.576043252	0.195867114	0.040607178
ENSG00000164236	*ANKRD33B*	5.06283045	-0.06874268	-0.029948871	-0.038793809	0.999933887	2.255693094	2.762951176	-0.507258081	0.040607178
ENSG00000109736	*MFSD10*	5.380968908	0.048373649	0.035107985	0.013265664	0.999933887	0.038475094	-0.17764839	0.216123484	0.040647603
ENSG00000110665	*C11orf21*	5.518938847	0.047746241	0.052266176	-0.004519935	0.999933887	-0.391188664	-0.678628482	0.287439819	0.040647603
ENSG00000144747	*TMF1*	5.419610713	-0.033117495	-0.169523961	0.136406467	0.999933887	-0.377501143	0.15714931	-0.534650453	0.040647603
ENSG00000182782	*HCAR2*	11.4216375	0.065221714	0.06651399	-0.001292277	0.999933887	0.119994925	0.400234183	-0.280239257	0.040719783
ENSG00000103496	*STX4*	7.97558332	0.109626848	0.092625179	0.017001669	0.999933887	0.590246755	0.820935123	-0.230688368	0.040817686
ENSG00000198646	*NCOA6*	5.659842401	-0.038127725	-0.013376733	-0.024750992	0.999933887	-0.639110015	-0.888171946	0.249061931	0.040817686
ENSG00000156026	*MCU*	3.28620708	0.018158975	0.085619482	-0.067460507	0.999933887	-0.099652294	-0.339761305	0.240109011	0.040838871
ENSG00000178971	*CTC1*	6.138302612	-0.021386618	0.044121042	-0.06550766	0.999933887	0.123461422	0.302170354	-0.178708932	0.040876562
ENSG00000175130	*MARCKSL1*	6.435479167	-0.000290789	0.090286978	-0.090577767	0.999933887	-0.210781271	-0.515645197	0.304863926	0.041059821
ENSG00000284669	*AC092053.3*	5.7601866	0.388172045	0.422271128	-0.034099083	0.999933887	1.040390791	1.477586486	-0.437195695	0.041151811
ENSG00000198961	*PJA2*	6.985176333	-0.054287115	-0.063099139	0.008812024	0.999933887	-0.19583257	0.104610256	-0.300442826	0.041151811
ENSG00000197746	*PSAP*	11.381219877	-0.030049026	-0.045489602	0.015440577	0.999933887	0.402119662	0.584366104	-0.182246442	0.041309234
ENSG00000107864	*CPEB3*	1.80383323	0.027257167	0.086117832	-0.058860665	0.999933887	-0.199990608	-0.752845905	0.552855297	0.041311718
ENSG00000141627	*DYM*	3.958456809	0.027795238	-0.035306407	0.063101645	0.999933887	-0.089090164	0.139560551	-0.228650715	0.041323539
ENSG00000227155	*AL161725.1*	3.230775142	-0.00319414	0.010787857	-0.013981997	0.999933887	-0.237488248	-0.795480351	0.557992103	0.041327894
ENSG00000171617	*ENC1*	4.515763518	-0.065841858	-0.083333078	0.017491219	0.999933887	-0.075048599	-0.314985868	0.239937269	0.041433282
ENSG00000167258	*CDK12*	6.408223858	-0.013358219	-0.04043177	0.02707355	0.999933887	0.061125108	0.226328028	-0.16520292	0.041433282
ENSG00000044090	*CUL7*	2.445426605	0.054847625	0.119763833	-0.064916207	0.999933887	-0.013541614	-0.366265876	0.352724262	0.041487595
ENSG00000112378	*PERP*	0.990973951	-0.038567534	-0.018933281	-0.019634254	0.999933887	0.440666202	1.060795386	-0.620129184	0.041497961
ENSG00000183576	*SETD3*	3.922019932	0.012988405	-0.058791039	0.071779444	0.999933887	-0.223572605	-0.474581897	0.251009291	0.041497961
ENSG00000234511	*C5orf58*	4.663176417	0.072624111	0.083489742	-0.010865631	0.999933887	0.806155386	1.357717869	-0.551562483	0.041511416
ENSG00000203364	*AL390760.1*	0.269913999	0.211292672	-0.43385886	0.645151532	0.999933887	2.994154164	3.586146014	-0.59199185	0.041565071
ENSG00000169567	*HINT1*	5.288763982	-0.007480124	-0.021592412	0.014112289	0.999933887	0.271582298	0.512981638	-0.241399341	0.041613456
ENSG00000287839	*AL353807.5*	1.976298596	0.006766694	-0.175316196	0.18208289	0.999933887	0.036504191	-0.500827686	0.537331877	0.041640203
ENSG00000187796	*CARD9*	0.454261603	-0.063389598	0.060138392	-0.12352799	0.999933887	0.026343447	-0.691553498	0.717896945	0.041696353
ENSG00000007129	*CEACAM21*	2.076305347	0.019230896	0.010929591	0.008301305	0.999933887	0.017638232	-0.425155102	0.442793334	0.041696353
ENSG00000139233	*LLPH*	3.314720467	-0.196260489	-0.120420872	-0.075839617	0.999933887	0.045885072	0.310680096	-0.264795024	0.041749447
ENSG00000133318	*RTN3*	7.918592188	-0.016514861	-0.028373333	0.011858472	0.999933887	-0.462611103	-0.731191964	0.26858086	0.041764887
ENSG00000103994	*ZNF106*	5.417171986	-0.070924131	-0.061253971	-0.00967016	0.999933887	-0.3725747	-0.699473117	0.326898417	0.041824148
ENSG00000012211	*PRICKLE3*	1.241142614	0.111866935	0.069043274	0.042823661	0.999933887	-0.07907766	-0.627656603	0.548578942	0.042071161
ENSG00000120616	*EPC1*	5.655848772	-0.041155517	-0.025305819	-0.015849698	0.999933887	0.009949519	0.201371792	-0.191422273	0.042284865
ENSG00000204569	*PPP1R10*	8.718473652	0.040093391	0.056842573	-0.016749182	0.999933887	0.44696004	0.6544902	-0.207530159	0.042527292
ENSG00000149948	*HMGA2*	-2.193312488	-0.423295874	-0.156691933	-0.266603941	0.999933887	1.347664301	2.169750613	-0.822086312	0.042561331
ENSG00000198580	*AC073343.1*	0.899769816	-0.149546398	-0.552858026	0.403311628	0.999933887	0.11634482	0.554120479	-0.437775659	0.042566563
ENSG00000173535	*TNFRSF10C*	6.938295405	-0.005392919	-0.034560313	0.029167395	0.999933887	-2.068368088	-2.669798513	0.601430425	0.042631783
ENSG00000166377	*ATP9B*	3.493387421	-0.041414379	0.081855682	-0.123270061	0.999933887	-0.023550944	0.217042545	-0.240593489	0.042674702
ENSG00000121297	*TSHZ3*	2.944628722	0.124548412	0.074319329	0.050229083	0.999933887	-0.83828668	-1.201354928	0.363068249	0.042750877
ENSG00000116014	*KISS1R*	0.435498909	0.191605297	0.016634567	0.17497073	0.999933887	-0.677187433	-1.434049569	0.756862136	0.042750877
ENSG00000048471	*SNX29*	3.373083399	0.023295265	0.052364651	-0.029069386	0.999933887	0.023941678	-0.238958716	0.262900395	0.042750877
ENSG00000157259	*GATAD1*	3.10234984	-0.025332726	0.05655556	-0.081888286	0.999933887	-0.382690419	-0.631015526	0.248325107	0.042780108

ENSG00000116698	SMG7	6.780309579	-0.014122558	-0.032144402	0.018021845	0.999933887	0.61049898	0.945984195	-0.335485215	0.042780108
ENSG00000132823	OSER1	7.970656719	0.00393199	-0.044270387	0.048202377	0.999933887	-0.203902735	0.015535781	-0.219438516	0.04278054
ENSG00000078061	ARAF	5.95607212	0.016045076	0.023514593	-0.007469517	0.999933887	0.282239561	0.472312813	-0.190073252	0.04287799
ENSG00000010671	BTK	4.041103786	-0.024621428	-0.015977413	-0.008644015	0.999933887	-0.47656056	-0.764407097	0.287846537	0.042950139
ENSG00000278097	AC243829.5	-1.935916471	0.225928777	-0.002664324	0.228593101	0.999933887	0.239785708	1.37622597	-1.136440262	0.042950446
ENSG00000175220	ARHGAP1	5.207279429	-0.040977191	0.032875474	-0.073852665	0.999933887	-0.293623189	-0.491915007	0.198291819	0.042950446
ENSG00000100031	GGT1	2.883322375	0.070739862	0.233337945	-0.162598083	0.999933887	0.493660883	0.895931686	-0.402270802	0.042960923
ENSG00000197301	HMGA2-AS1	-1.367110607	-0.229260357	0.496529772	-0.725790128	0.999933887	2.04930821	2.893890908	-0.844582698	0.042963042
ENSG00000100364	KIAA0930	5.141469307	0.018700582	0.035960739	-0.017260157	0.999933887	-1.49714068	-1.915285135	0.418144455	0.042963042
ENSG00000101868	POLA1	1.441280555	-0.002162327	0.067895217	-0.070057544	0.999933887	0.025382781	0.544556946	-0.519174165	0.042979407
ENSG00000100523	DDHD1	6.236118623	-0.021337935	0.014702172	-0.036040107	0.999933887	0.199240486	0.542090777	-0.342850291	0.042983266
ENSG00000057704	TMCC3	7.215542075	-0.045500985	-0.037836118	-0.007664866	0.999933887	-0.411069536	-0.156850575	-0.254218961	0.043169705
ENSG00000239998	LILRA2	7.609432247	0.027143989	-0.032795729	0.059939719	0.999933887	-1.167770882	-1.477903421	0.310132539	0.043169705
ENSG00000167083	GNGT2	1.491630986	-0.164642653	-0.11746818	-0.047174473	0.999933887	0.171156972	-0.329730679	0.500887651	0.04322391
ENSG00000113761	ZNF346	2.472520811	0.065326534	-0.039346659	0.104673193	0.999933887	-0.445587603	-0.842636184	0.397048582	0.04322391
ENSG00000164330	EBF1	-1.075755845	0.080478653	0.263860735	-0.183382082	0.999933887	0.340206499	1.207275516	-0.867069016	0.04322391
ENSG00000237181	PRKAR1B-AS	2.053977532	-0.004095619	0.023939762	-0.028035381	0.999933887	1.384145957	1.739732637	-0.35558668	0.04322391
ENSG00000148291	SURF2	1.558657488	-0.001694903	0.062934369	-0.064629272	0.999933887	-0.000492855	-0.355780732	0.355287876	0.04322391
ENSG00000167487	KLHL26	1.981485431	0.14092495	0.065570358	0.075354593	0.999933887	0.149792127	-0.247382109	0.397174235	0.043265754
ENSG00000116830	TTF2	2.293861749	-0.052204493	-0.021616599	-0.030587894	0.999933887	0.337960999	0.676004733	-0.338043735	0.043265754
ENSG00000258302	AC025034.1	3.877425882	-0.13273152	0.128494752	-0.261226272	0.999933887	0.971725681	1.794604255	-0.822878574	0.043353676
ENSG00000271383	NBPF19	1.169091639	0.020472862	0.088513376	-0.068040514	0.999933887	0.513238441	0.977705295	-0.464466854	0.043353676
ENSG00000281195	AC007878.1	2.030327627	0.176123952	0.030995166	0.145128786	0.999933887	-0.324263194	0.241470436	-0.56573363	0.04341981
ENSG00000101966	XIAP	5.914549093	-0.049429348	0.009526519	-0.058955867	0.999933887	0.14882588	0.470647033	-0.321821153	0.043452148
ENSG00000066322	ELOVL1	4.974844228	-0.017291843	-0.020040746	0.002748902	0.999933887	-0.48685857	-0.728003704	0.241145134	0.043467769
ENSG00000089505	CMTM1	1.079746636	0.013423225	-0.204641967	0.218065192	0.999933887	-1.593468287	-2.354038098	0.760569812	0.043467769
ENSG00000100288	CHKB	0.631385614	-0.202596846	-0.004651301	-0.197945545	0.999933887	-0.11497576	-0.663521588	0.548545828	0.043492498
ENSG00000085185	BCORL1	3.196569822	-0.053831981	0.080568469	-0.13440045	0.999933887	-0.291551226	-0.60745644	0.315905215	0.043533756
ENSG00000183723	CMTM4	0.406033936	-0.112866308	-0.130062669	0.017196361	0.999933887	-0.402854131	-1.159651242	0.756797111	0.043533756
ENSG00000105991	HOXA1	-0.005153012	-0.035050038	-0.238484222	0.203434185	0.999933887	-0.646180473	-1.707354965	1.061174493	0.043583401
ENSG00000130382	MLLT1	5.9353508	0.050020788	-0.006276064	0.056296852	0.999933887	-0.248452442	-0.464348365	0.215895923	0.04360068
ENSG00000139132	FGD4	4.928647815	-0.044075148	-0.076927391	0.032852243	0.999933887	0.264216124	0.681679462	-0.417463338	0.043643491
ENSG00000130741	EIF2S3	7.076446039	-0.021503115	-0.011130792	-0.010372323	0.999933887	0.126750062	0.279626851	-0.152876789	0.043689744
ENSG00000132661	NXT1	3.533508754	0.128372431	0.048413551	0.07995888	0.999933887	0.832906137	1.127578774	-0.294672638	0.04376953
ENSG00000108405	P2RX1	5.805197829	-0.018994933	0.032489071	-0.051484004	0.999933887	-0.610594125	-0.82984244	0.219248315	0.043782057
ENSG00000123353	ORMDL2	2.465185426	-0.0632595	0.042595947	-0.105855447	0.999933887	-0.568808512	-0.901307519	0.332499008	0.043860227
ENSG00000165417	GTF2A1	6.865212048	-0.066378187	-0.114379214	0.048001027	0.999933887	0.195441258	0.491331739	-0.295890481	0.043921649
ENSG00000198742	SMURF1	6.248955236	0.002605556	-0.011875343	0.014480899	0.999933887	0.395095374	0.556665501	-0.161570127	0.043945351
ENSG00000183648	NDUFB1	3.880279182	-0.034166009	-0.05370381	0.019537801	0.999933887	-0.101959294	-0.386359674	0.28440038	0.043999532
ENSG00000198804	MT-CO1	9.841663293	0.013682868	-0.040331145	0.054014013	0.999933887	-0.01870394	0.267416739	-0.286120679	0.043999532
ENSG00000144504	ANKMY1	3.032870954	-0.062728075	0.056366114	-0.119094188	0.999933887	-0.071237815	-0.335481114	0.2642433	0.04405078
ENSG00000171862	PTEN	8.361259958	-0.020467225	-0.003695929	-0.016771295	0.999933887	-0.535005858	-0.78138172	0.246375862	0.04405078
ENSG00000258730	ITPK1-AS1	-0.258670459	-0.041694112	0.0731622	-0.114856312	0.999933887	-0.874726852	-2.086118417	1.211391565	0.044136921
ENSG00000141404	GNAL	1.041975506	-0.149843888	-0.115118064	-0.034725824	0.999933887	-0.450530018	-0.967333042	0.516803025	0.044137619
ENSG00000111863	ADTRP	0.435522556	0.00217819	-0.254536302	0.256714492	0.999933887	0.420273068	0.957453269	-0.537180202	0.044171545
ENSG00000112624	BICRAL	5.267308708	-0.037638237	0.053980712	-0.091618949	0.999933887	-0.668835456	-0.9531906	0.284355143	0.04421323
ENSG00000143622	RIT1	8.047026347	-0.050731513	-0.062851897	0.012120383	0.999933887	0.222614286	0.410353187	-0.187738901	0.044217906
ENSG00000090989	EXOC1	4.694782506	-0.018336331	-0.120156912	0.101820581	0.999933887	-0.375228062	-0.001755258	-0.373472804	0.04431757
ENSG00000232810	TNF	5.930030268	0.687892381	1.050933967	-0.363041587	0.999933887	2.553222565	3.198183223	-0.644960658	0.044483039
ENSG00000134698	AGO4	7.280138115	-0.077307954	-0.059283571	-0.018024384	0.999933887	-0.824228945	-1.056013759	0.231784813	0.044483039
ENSG00000115155	OTOF	-0.009304876	0.05642628	-0.106249811	0.162676091	0.999933887	0.255988738	-0.363061033	0.61904977	0.044505018
ENSG00000233791	LINC01136	3.777709075	0.172309065	0.155760525	0.016548539	0.999933887	1.497606107	1.864800244	-0.367194137	0.044652564
ENSG00000228109	MELTF-AS1	-0.7551082	0.24720064	0.300757892	-0.053557252	0.999933887	0.714677141	1.516765598	-0.802088457	0.044652564
ENSG00000232630	PRPS1P2	0.88203665	0.013813381	0.067782386	-0.053969005	0.999933887	-0.313016898	0.198442352	-0.51145925	0.044652564
ENSG00000178952	TUFM	4.835918286	-0.005053668	-0.047040929	0.041987261	0.999933887	-7.1678e-05	-0.222227563	0.222155886	0.044652564
ENSG00000163874	ZC3H12A	8.688105141	0.047867219	0.070284444	-0.022417225	0.999933887	1.896313348	2.168763163	-0.272449815	0.044936541
ENSG00000183087	GAS6	1.784914318	0.036808615	0.098090424	-0.061281809	0.999933887	0.432303092	0.81777841	-0.385475318	0.044936541
ENSG00000128604	IRF5	4.108829803	0.031044539	0.023270531	0.007774008	0.999933887	1.507344905	1.915962913	-0.408618009	0.044936541
ENSG00000129673	AANAT	0.64440941	-0.098876011	0.089573085	-0.188449097	0.999933887	1.180587235	1.970686721	-0.790099485	0.045018285
ENSG00000075624	ACTB	12.44988677	0.026838974	0.038609065	-0.011770091	0.999933887	-0.328817951	-0.548319841	0.21950189	0.045018285
ENSG00000166946	CCNDBP1	6.460661125	-0.049790836	-0.057645206	0.007854371	0.999933887	-0.569150033	-0.759571828	0.190421795	0.045038269
ENSG00000118515	SGK1	11.37549727	0.191871894	0.215341141	-0.023469246	0.999933887	0.564336437	0.800392981	-0.236056544	0.04523552
ENSG00000109066	TMEM104	4.24186213	-0.053816124	0.001142311	-0.054958435	0.999933887	-0.664368731	-0.907865812	0.24349708	0.04523552
ENSG00000133985	TTC9	3.531508557	0.012858941	0.05453478	-0.041675839	0.999933887	-0.536905941	-0.850363693	0.313457751	0.04523552
ENSG00000174796	THAP6	3.063657549	-0.040913657	0.038711838	-0.079625495	0.999933887	-0.152312074	0.179915692	-0.332227766	0.045409382
ENSG00000138592	USP8	5.58333298	-0.041939716	-0.124472991	0.082533276	0.999933887	-0.172056626	0.233511833	-0.405568459	0.045409382
ENSG00000141027	NCOR1	6.306823779	0.003800716	-0.023901953	0.027702669	0.999933887	-0.221060688	-0.43319951	0.212138821	0.045409382
ENSG00000139154	AEBP2	3.781429483	0.000984588	-0.06767354	0.068658127	0.999933887	0.203920629	0.499897608	-0.295976979	0.045409382
ENSG00000168884	TNIP2	5.094220714	0.024980721	-0.005825097	0.030805818	0.999933887	0.668452453	0.950538717	-0.282086264	0.045416465
ENSG00000084734	GCKR	-2.911788345	-0.146755024	0.23942716	-0.386182184	0.999933887	2.759667504	4.397955088	-1.638287584	0.045545305
ENSG00000101940	WDR13	4.576929576	0.02969057	0.049295147	-0.019604578	0.999933887	-0.112924411	-0.356417312	0.243492901	0.045654959
ENSG00000243273	AC020636.1	0.164783832	0.22429215	-0.030373179	0.254665328	0.999933887	0.983021256	1.761370522	-0.778349267	0.045691083
ENSG00000237513	AC007384.1	2.85878649	-0.079167391	0.052961528	-0.132128919	0.999933887	2.156506838	2.77645028	-0.619943442	0.045691083
ENSG00000114745	GORASP1	4.452039726	0.010462471	0.065999486	-0.055537015	0.999933887	-0.289175568	-0.542424442	0.253248873	0.045691083
ENSG00000172232	AZU1	-0.079639871	-0.006215748	0.184764804	-0.190980552	0.999933887	-0.233399971	0.461469018	-0.69486899	0.045691083
ENSG00000164941	INTS8	3.891068676	-0.032607811	-0.018059469	-0.014548342	0.999933887	-0.375624917	-0.653090398	0.277465481	0.045722637
ENSG00000141179	PCTP	2.525486715	-0.018255938	-0.034063271	0.015807333	0.999933887	-0.037755691	0.272206449	-0.30996214	0.0457937
ENSG00000250929	LINC01181	-1.567563909	-0.473363029	-0.539547284	0.066184255	0.999933887	1.620705892	2.33712723	-0.716421337	0.046029611
ENSG00000168209	DDIT4	4.651289441	0.276332987	0.387251737	-0.11091875	0.999933887	1.499252784	1.905457821	-0.406205037	0.046057631
ENSG00000181896	ZNF101	3.415425365	-0.048003477	-0.042992133	-0.005011344	0.999933887	-0.084667301	-0.386560687	0.301893387	0.046057631
ENSG00000119242	CCDC92	3.359938415	0.014865038	-0.10268675	0.117551788	0.999933887	-0.130974617	-0.387910527	0.25693591	0.046057631
ENSG00000217128	FNIP1	7.978963376	0.000404566	0.0141069	-0.013702334	0.999933887	-0.117374474	0.145620573	-0.262995047	0.046057631
ENSG00000141574	SECTM1	7.663564564	0.136716431	0.039525343	0.097191088	0.999933887	-0.691202933	-1.08507282	0.393869887	0.04616368
ENSG00000105726	ATP13A1	4.5918479	0.06036476	0.025646671	0.034718089	0.999933887	0.020292637	-0.169817379	0.190110017	0.046161368
ENSG00000117091	CD48	7.436993403	-0.019378942	-0.061265795	0.041886853	0.999933887	0.437884948	0.645204974	-0.207320026	0.046161368
ENSG00000121316	PLBD1	5.734694769	-0.014429214	-0.028607408	0.014178194	0.999933887	-0.207493931	-0.54143926	0.333945329	0.046161368
ENSG00000265241	RBM8A	4.61866475	0.009770268	-0.006122555	0.015892823	0.999933887	-0.414117073	-0.685696284	0.271579211	0.046161368
ENSG00000050130	JKAMP	3.34039957	-0.003203252	0.01077817	-0.013981422	0.999933887	-0.9136072	-1.282318712	0.368711512	0.046219461
ENSG00000076382	SPAG5	2.646110792	0.070356377	-0.126139293	0.196495671	0.999933887	0.823931888	1.242880229	-0.418948341	0.046328859
ENSG00000106603	COA1	4.030076828	-0.005560436	-0.200339895	0.194779459	0.999933887	-0.150620176	-0.461159747	0.310539572	0.046328859
ENSG00000164754	RAD21	6.928248211	-0.093396345	-0.060867152	-0.032529193	0.999933887	-0.099785057	0.165464313	-0.26524937	0.046417923
ENSG00000126107	HECTD3	4.168072975	0.007213387	0.037939757	-0.03072637	0.999933887	-0.28112436	-0.49806896	0.2169446	0.046442352
ENSG00000254995	STX16-NPEPL	1.85243559	0.014479812	0.073049586	-0.058569774	0.999933887	0.183439908	-0.174461389	0.357901297	0.046450375
ENSG00000177427	MIEF2	1.679408453	0.126944608	0.03817168	0.088772928	0.999933887	-0.605518021	-1.125496586	0.519978565	0.046636977
ENSG00000123106	CCDC91	2.052130892	-0.137623635	-0.21681568	0.079192045	0.999933887	-0.196389811	0.228442709	-0.42483252	0.04668796
ENSG00000136888	ATP6V1G1	7.207268603	0.001241401	-0.000874143	0.002115544	0.999933887	0.32706051	0.485293978	-0.158233468	0.04668796
ENSG00000100075	SLC25A1	3.027263624	0.043994409	0.016275867	0.027718543	0.999933887	0.083476689	-0.281116536	0.364593225	0.046708851
ENSG00000176018	LYSMD3	4.615081479	-0.046420553	-0.214218955	0.167798402	0.999933887	-0.401177927	0.012471812	-0.413649738	0.046708851
ENSG00000131042	LILRB2	7.738276012	0.017401226	0.007478702	0.009922524	0.999933887	-0.268551746	-0.562834962	0.294283216	0.046881621
ENSG00000101577	LPIN2	7.608265381	-0.088332408	0.005167674	-0.093500082	0.999933887	-1.139101814	-1.45386169	0.314759876	0.046922633
ENSG00000185515	BRCC3	2.084378555	0.034021499	-0.056362361	0.09038386	0.999933887	-0.553100929	-1.053278791	0.500177862	0.046922633
ENSG00000125741	OPA3	4.215085021	0.0215921	0.179975956	-0.158383857	0.999933887	0.689277312	0.962497215	-0.273219903	0.046985169
ENSG00000196911	KPNA5	2.095440248	-0.067474505	-0.193060316	0.125585811	0.999933887	-0.326036639	0.176978449	-0.503015089	0.046988989
ENSG00000037749	MFAP3	3.127671731	-0.124800619	0.011401095	-0.136201713	0.999933887	-0.259645764	-0.010018542	-0.249627222	0.047061096
ENSG00000126226	PCID2	3.342197638	-0.020654666	-0.007443942	-0.013210724	0.999933887	0.23877773	0.520322009	-0.281544279	0.047061096

ENSG00000162144	*CYB561A3*	5.300086359	0.001255323	0.024489747	-0.023234424	0.999933887	0.916961119	1.116526792	-0.199565674	0.047061096
ENSG00000174004	*NRROS*	5.067230672	-0.1051133	-0.093628305	-0.011484994	0.999933887	-0.972843666	-1.362366133	0.389522468	0.047104943
ENSG00000238260	*AL513320.1*	-0.518662995	-0.155600461	-0.166374774	0.010774314	0.999933887	-1.114105587	-2.045305257	0.93119967	0.04716322
ENSG00000141867	*BRD4*	8.377072076	-0.008693065	0.005296715	-0.01398978	0.999933887	0.133205577	0.273760347	-0.14055477	0.04716322
ENSG00000103966	*EHD4*	4.74215198	0.020607194	-0.008227129	0.028834323	0.999933887	0.135043417	0.391523254	-0.256479837	0.047177372
ENSG00000164323	*CFAP97*	2.10949713	-0.10004251	-0.050057124	-0.049985386	0.999933887	-0.065950307	0.381681306	-0.447631613	0.047254931
ENSG00000132471	*WBP2*	9.519211088	0.016723075	0.021244353	-0.004521279	0.999933887	0.473790041	0.632864451	-0.15907441	0.04728428
ENSG00000223959	*AFG3L1P*	2.795168548	-0.007012911	0.008094993	-0.015107904	0.999933887	0.012610819	-0.26896092	0.281571739	0.04728428
ENSG00000077327	*SPAG6*	-0.367941606	0.148451158	0.07993435	0.068516809	0.999933887	0.301953033	1.054128788	-0.752175755	0.047379211
ENSG00000100426	*ZBED4*	3.679444776	0.007242534	0.053791885	-0.046549351	0.999933887	-0.062379338	-0.329830589	0.267451251	0.04758416
ENSG00000228340	*MIR646HG*	3.623064964	0.003654146	0.019739623	-0.016085477	0.999933887	0.513434589	0.843111811	-0.329677223	0.047706502
ENSG00000198727	*MT-CYB*	9.151402594	0.080402483	-0.035546663	0.115949146	0.999933887	-0.009882913	0.344440332	-0.354323245	0.047803569
ENSG00000003056	*M6PR*	7.474442343	0.031374118	0.017348409	0.014025709	0.999933887	0.160747357	0.327821935	-0.167074579	0.047803569
ENSG00000086758	*HUWE1*	7.16821569	-0.024681082	0.004142226	-0.028823308	0.999933887	0.162985201	0.331707766	-0.168722565	0.047803569
ENSG00000111652	*COPS7A*	3.74238591	-0.003943742	-0.073728146	0.069784403	0.999933887	-0.322723572	-0.530340754	0.207617183	0.047803569
ENSG00000169733	*RFNG*	3.139860979	0.00346737	0.05016977	-0.0467024	0.999933887	-0.128773767	-0.425910248	0.297136481	0.048180143
ENSG00000198846	*TOX*	2.041559218	0.012258472	-0.128935747	0.141194219	0.999933887	-0.102684074	0.270618712	-0.373302786	0.048222927
ENSG00000148175	*STOM*	5.059972923	-0.013161915	-0.005111025	-0.00805089	0.999933887	0.119374824	0.32559125	-0.206216425	0.048264664
ENSG00000108771	*DHX58*	3.663925604	0.083633842	0.012780102	0.070853741	0.999933887	0.027460193	-0.261918154	0.289378346	0.048334771
ENSG00000173334	*TRIB1*	10.381047556	0.04976718	0.063750853	-0.013983673	0.999933887	-0.051617686	0.170612473	-0.222230159	0.048342036
ENSG00000140090	*SLC24A4*	2.943963967	-0.027411589	-0.085769686	0.058358096	0.999933887	-0.354882377	-0.754845	0.399962624	0.048342036
ENSG00000168883	*USP39*	3.89166192	0.018388869	-0.077723961	0.09611283	0.999933887	-0.30556877	-0.582218458	0.276649688	0.048342036
ENSG00000164985	*PSIP1*	4.511276463	0.030709915	-0.026855017	0.057564931	0.999933887	-0.38616712	-0.757169656	0.371002535	0.048361051
ENSG00000153006	*SREK1IP1*	2.458937929	0.011679292	-0.117089309	0.128768601	0.999933887	-0.38805638	0.116791581	-0.504847961	0.048361051
ENSG00000111897	*SERINC1*	8.567560347	-0.053689999	-0.032183098	-0.021506901	0.999933887	-0.008597734	0.197705239	-0.206302973	0.0484995
ENSG00000108587	*GOSR1*	4.328880127	0.010204383	0.061448473	-0.051244091	0.999933887	0.274218705	0.473088761	-0.198870055	0.048534931
ENSG00000181274	*FRAT2*	7.225521319	-0.250853326	-0.290088326	0.039235	0.999933887	-1.874752034	-2.327573639	0.452821605	0.048778683
ENSG00000100908	*EMC9*	1.414606591	-0.294786903	0.13568295	-0.430469852	0.944094537	0.097049108	-0.356419312	0.45346842	0.048892328
ENSG00000065548	*ZC3H15*	5.121514541	-0.104502725	-0.129278751	0.024776026	0.999933887	-0.172334128	0.10829455	-0.280628677	0.048892328
ENSG00000130517	*PGPEP1*	5.468642327	-0.088236159	-0.046572068	-0.041664091	0.999933887	-0.799122887	-1.081587123	0.282464236	0.048892328
ENSG00000117523	*PRRC2C*	8.51473349	-0.0738817	-0.11336149	0.03947979	0.999933887	0.32082656	0.673625121	-0.352798561	0.048892328
ENSG00000106268	*NUDT1*	1.729920579	0.105610125	0.047666714	0.057943412	0.999933887	0.573661963	0.98734053	-0.413678568	0.048918155
ENSG00000169957	*ZNF768*	1.735534689	0.02547368	0.08021655	-0.05474287	0.999933887	-0.944592976	-1.421630438	0.477037463	0.048918155
ENSG00000168310	*IRF2*	7.560315469	0.013650505	-0.019022695	0.0326732	0.999933887	0.040226599	0.221542504	-0.181315905	0.048919709
ENSG00000113811	*SELENOK*	5.953729116	0.096384056	0.121898405	-0.025514349	0.999933887	0.716452088	0.995644513	-0.279192425	0.048927348
ENSG00000196865	*NHLRC2*	2.352462155	0.045495273	-0.059165458	0.104660732	0.999933887	-0.215180992	0.182001237	-0.397182229	0.048927348
ENSG00000164266	*SPINK1*	2.573579337	0.097167712	0.192352218	-0.095184505	0.999933887	0.505411904	0.862924989	-0.357513085	0.049028853
ENSG00000136040	*PLXNC1*	9.420029698	-0.009451468	-0.005930145	-0.003521324	0.999933887	0.424671222	0.620311858	-0.195640636	0.049083263
ENSG00000140262	*TCF12*	3.0136615	-0.006195564	-0.159994643	0.153799078	0.999933887	-0.138388999	0.238762716	-0.377151716	0.049132456
ENSG00000183486	*MX2*	8.055989464	0.015507967	0.023085139	-0.007577172	0.999933887	-0.123696007	-0.297553039	0.173857032	0.049228998
ENSG00000149657	*LSM14B*	5.158423979	-0.007166976	0.014379013	-0.02154599	0.999933887	-0.876733338	-1.139237737	0.262504399	0.049557793
ENSG00000223901	*AP001469.1*	-0.973255627	0.1723289	-0.092751104	0.265080004	0.999933887	2.088912395	3.186518783	-1.097606389	0.049581726
ENSG00000196455	*PIK3R4*	3.90388956	-0.049304072	-0.016121777	-0.033182296	0.999933887	0.109057573	0.340943536	-0.231885963	0.049712897
ENSG00000135185	*TMEM243*	2.761260828	-0.108401287	0.028343802	-0.136745089	0.999933887	-0.027491296	-0.280743954	0.253252658	0.049783801
ENSG00000153922	*CHD1*	7.910178756	-0.062483515	-0.177849775	0.11536626	0.999933887	-0.108444358	0.430309701	-0.538754059	0.049820842
ENSG00000128311	*TST*	1.377177948	-0.067935233	0.087280535	-0.155215768	0.999933887	-0.813111716	-1.54479975	0.731688035	0.049897614
ENSG00000117036	*ETV3*	5.230587146	0.008069824	-0.018645102	0.026714926	0.999933887	0.153144422	0.333471466	-0.180327044	0.049897614
ENSG00000128829	*EIF2AK4*	2.300442152	-0.005157486	-0.083522027	0.078364541	0.999933887	-0.173496608	-0.534922667	0.361426059	0.049897614
ENSG00000174840	*PDE12*	4.345892594	0.041210417	-0.056035462	0.097245879	0.999933887	0.264800424	0.482852959	-0.218052535	0.049990821
ENSG00000187608	*ISG15*	6.241678647	0.091983984	0.04396306	0.048020924	0.999933887	-0.123894872	-0.447884564	0.323989692	0.050039717
ENSG00000109956	*B3GAT1*	1.774042872	-0.013763835	0.065645116	-0.07940895	0.999933887	0.22969448	-0.137006594	0.366701074	0.05008292
ENSG00000117450	*PRDX1*	3.801123387	-0.07470135	0.095382215	-0.170083565	0.999933887	0.381484651	0.643592853	-0.262108202	0.050255923
ENSG00000287630	*AC011370.1*	-0.30648871	0.196848111	0.60011255	-0.403264439	0.999933887	-0.37449303	0.268196733	-0.642689763	0.050255923
ENSG00000184203	*PPP1R2*	6.427636436	-0.036945867	-0.011554925	-0.025390942	0.999933887	-0.04555947	0.101438401	-0.146997871	0.050352977
ENSG00000244945	*RUFY1-AS1*	0.724580004	0.012259057	0.080169622	-0.067910565	0.999933887	0.105089102	0.627265703	-0.5221766	0.050352977
ENSG00000142875	*PRKACB*	3.645933806	-0.121887728	-0.215691563	0.093803835	0.999933887	-0.187931513	0.256189763	-0.444121276	0.050427846
ENSG00000198951	*NAGA*	2.292099656	-0.031050967	-8.7561e-05	-0.030963406	0.999933887	-0.292593946	-0.648706921	0.356112975	0.050427846
ENSG00000143554	*SLC27A3*	3.441086698	0.101911894	0.013684606	0.088227288	0.999933887	-0.196339363	-0.529690366	0.333351004	0.050534741
ENSG00000182957	*SPATA13*	6.424455962	-0.054223613	-0.021679194	-0.032544418	0.999933887	0.148491697	0.347010274	-0.198518577	0.050613572
ENSG00000162976	*SLC66A3*	2.598453744	-0.006578875	-0.100823832	0.094244957	0.999933887	-0.131632928	-0.520417027	0.388784099	0.050654531
ENSG00000176783	*RUFY1*	5.331423743	0.003799377	0.042507572	-0.038708195	0.999933887	0.114839129	0.303305107	-0.188465979	0.050732996
ENSG00000241839	*PLEKHO2*	9.493031998	-0.036100647	-0.115169344	0.079068697	0.999933887	0.485994078	0.697272911	-0.211278833	0.050853094
ENSG00000139826	*ABHD13*	4.702317779	-0.023257989	-0.098394447	0.075136457	0.999933887	0.009060852	0.260019349	-0.250958497	0.0508586
ENSG00000115568	*ZNF142*	3.921087696	0.072585848	0.029855992	0.042729856	0.999933887	0.329975618	0.599845096	-0.269869478	0.050948782
ENSG00000145414	*NAF1*	2.015618337	-0.01446949	-0.075673363	0.061203873	0.999933887	0.831917113	1.357102756	-0.525185644	0.051018243
ENSG00000125970	*RALY*	7.232923295	0.044485067	0.02483719	0.019647878	0.999933887	-0.199825469	-0.400939252	0.201113784	0.05103601
ENSG00000198160	*MIER1*	6.326949891	-0.050829477	-0.124412764	0.073583287	0.999933887	-0.436129983	-0.122336583	-0.3137934	0.05103601
ENSG00000173473	*SMARCC1*	5.302324921	-0.012126765	0.010011522	-0.022138287	0.999933887	-0.086855972	-0.255285421	0.168429449	0.051200849
ENSG00000152359	*POC5*	1.157453994	-0.036636595	-0.325697365	0.28906077	0.999933887	0.560497348	1.064507062	-0.504009714	0.051264542
ENSG00000162702	*ZNF281*	6.661315861	-0.101038568	-0.123673386	0.022634818	0.999933887	0.336542022	0.552312887	-0.215770866	0.051526891
ENSG00000176371	*ZSCAN2*	-0.267324889	-0.056214277	0.172249915	-0.228464192	0.999933887	-0.004578705	-0.59955887	0.594980166	0.051569377
ENSG00000158869	*FCER1G*	8.704431339	-0.013404366	0.0005016	-0.013905966	0.999933887	0.63233732	0.835128183	-0.202790863	0.051611659
ENSG00000104419	*NDRG1*	7.085507121	0.00283816	0.045228203	-0.042390043	0.999933887	0.077282016	0.293418743	-0.216136727	0.051611827
ENSG00000144655	*CSRNP1*	10.034656736	0.243753309	0.198314262	0.045439047	0.999933887	0.927867988	1.2112019	-0.283333912	0.051664385
ENSG00000071189	*SNX13*	5.762580947	-0.009619321	-0.071980059	0.062360738	0.999933887	-0.63747624	-0.240873017	-0.396603222	0.051666486
ENSG00000132294	*EFR3A*	5.843832749	-0.073740485	-0.102972208	0.029231724	0.999933887	-0.303434278	0.052178702	-0.35561298	0.051695458
ENSG00000197329	*PELI1*	9.664967774	-0.00908764	-0.024756146	0.015668507	0.999933887	-0.058714516	0.205546118	-0.264260634	0.051695458
ENSG00000287644	*AC080112.4*	2.390280989	0.11214846	0.097714841	0.014433619	0.999933887	0.407975423	0.860204904	-0.452229481	0.051756348
ENSG00000224596	*ZMIZ1-AS1*	0.792868125	0.121049838	-0.095637207	0.216687045	0.999933887	0.507006502	0.97157474	-0.464568238	0.051863609
ENSG00000114933	*INO80D*	5.182191741	0.022002309	-0.106668607	0.128670916	0.999933887	-0.178007261	0.346827791	-0.524835052	0.051863609
ENSG00000116668	*SWT1*	4.394725726	-0.033630138	-0.086248147	0.052618009	0.999933887	-0.084520246	0.343934865	-0.428455111	0.051873362
ENSG00000189430	*NCR1*	1.590654029	-0.024755405	0.019862262	-0.044617667	0.999933887	1.042405698	1.497153665	-0.454747968	0.052068414
ENSG00000119878	*CRIPT*	3.505607037	-0.11961115	-0.057645038	-0.061966112	0.999933887	-0.277826635	-0.534431754	0.256605119	0.052095091
ENSG00000008018	*PSMB1*	5.870794534	-0.040730408	-0.074131927	0.033401519	0.999933887	0.101978825	0.273815691	-0.171836867	0.052176045
ENSG00000198833	*UBE2J1*	6.552147375	-0.005425091	-0.022021572	0.016596482	0.999933887	-0.305223538	-0.523799321	0.218575783	0.052186915
ENSG00000286276	*AL390066.2*	0.159607746	0.224919808	0.22360431	0.001315499	0.999933887	0.935287719	1.378691987	-0.443404268	0.052233773
ENSG00000151229	*SLC2A13*	1.720252382	-0.029676227	-0.083840155	0.054163928	0.999933887	-1.065434428	-1.456126555	0.390692127	0.052233773
ENSG00000026103	*FAS*	6.317371794	-0.07413799	-0.086911565	0.012773575	0.999933887	0.90493047	1.21396481	-0.30903434	0.052354556
ENSG00000104164	*BLOC1S6*	6.384361551	-0.021615165	-0.007991319	-0.013623845	0.999933887	0.033860504	0.332066161	-0.298205657	0.052413982
ENSG00000080189	*SLC35C2*	5.629643629	0.011139793	0.009264147	0.001875647	0.999933887	0.664663818	0.869144906	-0.204481088	0.052413982
ENSG00000155093	*PTPRN2*	3.59733287	-0.066031813	-0.036689721	-0.029342092	0.999933887	-0.447469504	-0.765658105	0.318188601	0.052432139
ENSG00000241163	*LINC00877*	6.377744484	0.116579694	0.083714081	0.032865613	0.999933887	1.184126084	1.483113201	-0.298987117	0.052477597
ENSG00000125735	*TNFSF14*	8.594179704	0.069622742	0.049294359	0.020328384	0.999933887	0.461058597	0.716204328	-0.255145731	0.052477597
ENSG00000144791	*LIMD1*	1.87943653	-0.056292128	-0.050423317	-0.00586881	0.999933887	0.02458704	-0.318898425	0.343485465	0.052477597
ENSG00000214087	*ARL16*	2.344258265	-0.024702063	-0.058239965	0.033537902	0.999933887	0.178158494	-0.097200387	0.275358881	0.052477597
ENSG00000124120	*TTPAL*	7.664524649	-0.075111077	-0.04654091	-0.028570167	0.999933887	-0.100831888	0.114313275	-0.215145163	0.052501405
ENSG00000178691	*SUZ12*	5.020785406	-0.003706997	-0.132976519	0.129269522	0.999933887	-0.243247108	0.175793796	-0.419040903	0.052569827
ENSG00000130726	*TRIM28*	6.491569653	0.007614903	0.064951619	-0.057336717	0.999933887	0.0489872	-0.12374605	0.172733251	0.052840694
ENSG00000141504	*SAT2*	3.454582581	0.037800853	-0.028893386	0.066694239	0.999933887	-0.295207045	-0.564976642	0.269769597	0.052854784
ENSG00000095209	*TMEM38B*	3.094639042	0.013546794	-0.048230262	0.061777055	0.999933887	0.759232393	1.063750535	-0.304518141	0.052854784
ENSG00000172062	*SMN1*	0.450256412	-0.123857331	0.22704352	-0.350900851	0.999933887	-0.053545771	0.535605071	-0.589150842	0.052900039
ENSG00000254837	*AP001372.2*	-1.219411234	0.203992354	0.006857003	0.197135351	0.999933887	1.645326416	2.41004182	-0.764715405	0.05305383
ENSG00000115109	*EPB41L5*	1.222015092	0.042964439	-0.062065295	0.105029734	0.999933887	0.209845248	0.785834384	-0.575989136	0.05305383
ENSG00000196205	*EEF1A1P5*	2.598757535	-0.036607184	-0.305332335	0.26872515	0.999933887	0.264120373	0.641421469	-0.377301096	0.05305383
ENSG00000101574	*METTL4*	2.568911343	0.033929178	-0.069676443	0.103605621	0.999933887	-0.736535693	-1.090459623	0.353923931	0.053105871

ENSG00000151849	*CENPJ*	0.652631864	0.00256264	-0.101060109	0.103622749	0.999933887	-0.276201233	0.299297092	-0.575498325	0.05310712
ENSG00000182087	*TMEM259*	7.577772022	0.076335158	0.105522884	-0.029187726	0.999933887	0.042463853	-0.16092943	0.203393283	0.053119877
ENSG00000160959	*LRRC14*	2.771841464	0.061822032	0.060381635	0.001440397	0.999933887	0.068765002	-0.256058421	0.324823423	0.053119877
ENSG00000198689	*SLC9A6*	2.975293095	-0.068659154	0.019017174	-0.087676328	0.999933887	-0.208356773	-0.57808077	0.369723997	0.053194228
ENSG00000075240	*GRAMD4*	3.054086348	-0.046450065	0.107880258	-0.154330323	0.999933887	-0.152027892	-0.456635304	0.304607411	0.053194228
ENSG00000147457	*CHMP7*	4.403693108	-0.024283274	0.048481323	-0.072764597	0.999933887	-0.115079056	-0.315150384	0.200071328	0.053194228
ENSG00000198964	*SGMS1*	4.401766027	0.021939439	-0.030475329	0.052414768	0.999933887	0.303228557	0.535118787	-0.23189023	0.053194228
ENSG00000174373	*RALGAPA1*	3.650137724	-0.019080613	-0.094252732	0.075172119	0.999933887	0.143280552	0.511937293	-0.368656741	0.053217547
ENSG00000100092	*SH3BP1*	4.651959345	0.067552662	0.08645631	-0.018903649	0.999933887	-0.246452649	-0.458441315	0.211988666	0.053305413
ENSG00000140416	*TPM1*	1.006970665	-0.069148709	-0.048323848	-0.020824861	0.999933887	-0.574855332	-1.019603793	0.444748462	0.053319898
ENSG00000144554	*FANCD2*	3.484704765	-0.029917277	-0.139049911	0.109132634	0.999933887	0.244955462	0.498078461	-0.253123	0.053783269
ENSG00000171316	*CHD7*	6.423295397	-0.054083891	-0.06876365	0.01467976	0.999933887	-0.881012875	-1.142051007	0.261038132	0.053915951
ENSG00000071626	*DAZAP1*	5.922739242	0.010182218	0.020189776	-0.010007558	0.999933887	-0.226677598	-0.384206973	0.157529375	0.053948756
ENSG00000255857	*PXN-AS1*	1.406685792	0.11945469	0.035868108	0.083586583	0.999933887	-0.501795201	-1.032623874	0.530828672	0.054067939
ENSG00000168615	*ADAM9*	5.655770341	-0.026468694	-0.096707057	0.070238363	0.999933887	-0.442232311	-0.129525751	-0.31270656	0.054277053
ENSG00000164163	*ABCE1*	2.882033722	-0.077009682	-0.154205937	0.077196255	0.999933887	0.006153135	0.373691648	-0.367538513	0.054330591
ENSG00000178694	*NSUN3*	3.317965317	-0.098904387	0.039728827	-0.138633213	0.999933887	-0.135302912	0.100352415	-0.235655327	0.054513498
ENSG00000175782	*SLC35E3*	4.936154474	-0.065306985	0.0143419	-0.079648885	0.999933887	0.330519829	0.528447984	-0.197928156	0.054513498
ENSG00000168944	*CEP120*	3.463259953	0.067512717	0.027160596	0.040352121	0.999933887	-0.138418733	0.288404746	-0.426823479	0.054513498
ENSG00000105849	*POLR1F*	3.311850866	-0.101532351	-0.182794359	0.081262008	0.999933887	0.780337456	1.264345698	-0.484008243	0.054516861
ENSG00000064999	*ANKS1A*	4.483797205	0.003460537	0.04228548	-0.038824943	0.999933887	-0.200406437	-0.42152649	0.221120052	0.054516861
ENSG00000149091	*DGKZ*	6.854598258	0.002375279	0.050531351	-0.048156072	0.999933887	-0.172165501	-0.363649387	0.191483886	0.054516861
ENSG00000105559	*PLEKHA4*	-1.04025098	0.269203329	0.101450252	0.167753077	0.999933887	1.081428147	1.859304355	-0.777876208	0.054634561
ENSG00000124772	*CPNE5*	1.342462746	0.020443506	0.026109939	-0.005666433	0.999933887	0.825598889	1.223025838	-0.397426949	0.054651279
ENSG00000064601	*CTSA*	7.662847139	0.008250334	-0.021847132	0.030097466	0.999933887	-0.476560481	-0.654516655	0.177956174	0.054713018
ENSG00000136536	*MARCHF7*	6.573976308	-0.070911901	-0.149637756	0.078725855	0.999933887	-0.503571124	-0.183049613	-0.32052151	0.054847274
ENSG00000149418	*ST14*	1.498269261	0.06452191	0.027452508	0.037069402	0.999933887	-0.603242911	-1.104933896	0.501690985	0.054847274
ENSG00000119673	*ACOT2*	2.373118212	0.031499187	0.207917673	-0.176418486	0.999933887	0.644812129	0.993994958	-0.349182829	0.054847274
ENSG00000255050	*AC067930.4*	-0.572477713	-0.019864737	0.107472827	-0.127337564	0.999933887	-0.611350141	-1.333107358	0.721757218	0.054847274
ENSG00000083838	*ZNF446*	1.038575445	0.007440298	-0.030390531	0.037830828	0.999933887	0.171161791	-0.471410048	0.642571839	0.05492458
ENSG00000130940	*CASZ1*	3.843583096	0.037857362	0.035479255	0.002378107	0.999933887	0.534650804	0.843602593	-0.30895179	0.054961631
ENSG00000087053	*MTMR2*	1.938353696	-0.030188435	0.024737317	-0.054925752	0.999933887	0.222832865	0.525326192	-0.302493327	0.054978312
ENSG00000117862	*TXNDC12*	2.489721768	-0.080078895	-0.033413421	-0.046665474	0.999933887	-0.066099848	-0.447164663	0.381064815	0.055089803
ENSG00000174574	*AKIRIN1*	7.269584277	0.053764322	0.024605071	0.029159251	0.999933887	0.202563667	0.383415615	-0.180851948	0.055117093
ENSG00000243968	*RN7SL402P*	-1.352339622	-0.259440709	0.083714176	-0.343154885	0.999933887	2.253106898	3.274636765	-1.021529867	0.055203062
ENSG00000155463	*OXA1L*	5.506829012	-0.009843274	-0.046493573	0.0366503	0.999933887	-0.087154481	-0.265560044	0.178405563	0.055223615
ENSG00000160111	*CPAMD8*	-1.025569332	-0.097868235	0.193199092	-0.291067327	0.999933887	0.476986476	1.556943154	-1.079956678	0.055358491
ENSG00000170909	*OSCAR*	5.681153696	0.055746294	-0.057739341	0.113485635	0.999933887	0.248105873	0.474160375	-0.226054502	0.055365144
ENSG00000169826	*CSGALNACT2*	8.16244016	-0.036717952	-0.075705159	0.038987206	0.999933887	0.14042351	0.372265503	-0.231841994	0.055365144
ENSG00000137073	*UBAP2*	4.233170427	0.028844993	0.029346735	-0.000501742	0.999933887	0.063711072	0.251411737	-0.187700665	0.055405514
ENSG00000182185	*RAD51B*	1.4116626	0.026939146	-0.009698736	0.036637882	0.999933887	0.054668392	0.473886462	-0.41921807	0.055464425
ENSG00000077097	*TOP2B*	5.679757567	-0.049508867	-0.165953373	0.116444506	0.999933887	-0.104952086	0.378393376	-0.483345462	0.055482981
ENSG00000171475	*WIPF2*	7.313967506	-0.044797432	-0.001045281	-0.043752151	0.999933887	0.00027506	0.149793442	-0.149518382	0.055528601
ENSG00000102103	*PQBP1*	4.09068335	0.077588681	0.033358549	0.044230133	0.999933887	0.643756548	0.924546618	-0.28079007	0.055528601
ENSG00000171262	*FAM98B*	1.650059192	-0.051054548	-0.040997693	-0.010056855	0.999933887	-0.141828266	0.305452723	-0.447280989	0.055528601
ENSG00000077235	*GTF3C1*	5.112989462	0.002027249	0.01699451	-0.01496726	0.999933887	-0.03680285	-0.222098238	0.185295388	0.055528601
ENSG00000128656	*CHN1*	-0.020622396	-0.08253555	-0.084155765	0.001620215	0.999933887	-0.148199261	0.591692294	-0.739891555	0.055532034
ENSG00000073803	*MAP3K13*	3.651710779	0.045790684	0.039570209	0.006220475	0.999933887	0.599419467	0.923730522	-0.324311055	0.055532034
ENSG00000139687	*RB1*	4.295975762	0.054843032	-0.030432018	0.08527505	0.999933887	-0.401471124	-0.085963342	-0.315507782	0.055584601
ENSG00000287263	*AC008875.3*	4.28471554	0.022401968	0.014456598	0.00794537	0.999933887	-0.111818245	-0.378698814	0.266880569	0.055724824
ENSG00000158828	*PINK1*	3.831985217	-0.026826156	-0.010440398	-0.016385758	0.999933887	-0.767382041	-1.083027246	0.315645205	0.055773375
ENSG00000169592	*INO80E*	4.077652121	0.050658412	0.037056969	0.013601443	0.999933887	-0.055850784	-0.34294899	0.287098206	0.055789596
ENSG00000148248	*SURF4*	6.458532051	0.019490333	0.002222414	0.01726792	0.999933887	-0.227600852	-0.420369528	0.192768676	0.055808397
ENSG00000090238	*YPEL3*	8.624407456	0.008251654	0.030405041	-0.022153387	0.999933887	-0.639444794	-0.857167501	0.217722707	0.055808397
ENSG00000162869	*PPP1R21*	4.340895246	-0.13104745	-0.013891713	-0.117155737	0.999933887	-0.540449306	-0.745052675	0.204603369	0.055916462
ENSG00000166750	*SLFN5*	5.07551299	-0.037350959	-0.185070607	0.147719647	0.999933887	0.066835766	0.464842962	-0.398007196	0.055916462
ENSG00000172594	*SMPDL3A*	1.034254371	-0.142979176	0.004516152	-0.147495327	0.999933887	0.704185133	1.177565086	-0.473379953	0.056233726
ENSG00000198918	*RPL39*	4.933699128	0.057427201	0.00735854	0.050068661	0.999933887	0.065603166	0.390708377	-0.32510521	0.056365902
ENSG00000148926	*ADM*	7.395216228	0.23413581	0.259541285	-0.025405475	0.999933887	0.920498274	1.248923524	-0.32842525	0.056413352
ENSG00000163738	*MTHFD2L*	1.982457128	-0.166630292	-0.082683314	-0.083946977	0.999933887	0.088824149	0.455935172	-0.367111023	0.056413352
ENSG00000085117	*CD82*	8.463865003	0.007617665	0.019258567	-0.011640902	0.999933887	1.254014903	1.525047389	-0.271032486	0.056431498
ENSG00000275238	*MIR4734*	-0.387867907	0.487061001	0.363342339	0.123718662	0.999933887	2.269259792	2.984181056	-0.714921264	0.05644008
ENSG00000137501	*SYTL2*	3.474862502	-0.112470622	-0.043797682	-0.06867294	0.999933887	-0.140998379	0.187885476	-0.328883855	0.056640463
ENSG00000154582	*ELOC*	4.567270518	-0.029951847	-0.000720524	-0.029231324	0.999933887	-0.10654878	0.098117421	-0.204666201	0.056640463
ENSG00000196204	*RNF216P1*	2.158779526	0.018531307	0.016257787	0.00227352	0.999933887	0.007298867	-0.32757826	0.334877127	0.056672243
ENSG00000138678	*GPAT3*	4.75795477	-0.109398836	-0.076300956	-0.03309788	0.999933887	-1.283046291	-1.646056388	0.363010097	0.056754077
ENSG00000133056	*PIK3C2B*	4.816532107	-0.02152344	-0.066698916	0.045175476	0.999933887	-2.521256914	-3.033679205	0.512422291	0.056754338
ENSG00000102908	*NFAT5*	6.950908662	-0.009639308	-0.059577518	0.049938211	0.999933887	-0.208126006	0.227266491	-0.435392496	0.056943583
ENSG00000203747	*FCGR3A*	7.701159835	-0.063133178	-0.121541177	0.058408	0.999933887	-0.453908537	-0.743933777	0.290025241	0.057003094
ENSG00000164758	*MED30*	3.877650103	0.033073336	0.122470665	-0.089397329	0.999933887	0.138072833	0.37083399	-0.232761157	0.057003094
ENSG00000107262	*BAG1*	5.747967864	-0.002149587	0.095434099	-0.097583686	0.999933887	-0.169056252	-0.472857407	0.303801155	0.057003094
ENSG00000130312	*MRPL34*	2.222071245	0.099031646	-0.063168039	0.162199686	0.999933887	0.003783639	-0.351238524	0.355022163	0.057029923
ENSG00000134308	*YWHAQ*	5.740743064	-0.038411568	-0.087381648	0.04897008	0.999933887	0.074266525	0.241346224	-0.167079699	0.05710324
ENSG00000176444	*CLK2*	4.5040483	-0.014807597	-0.074918764	0.060111167	0.999933887	-0.2823464	-0.466053865	0.183707465	0.057199923
ENSG00000236453	*AC003092.1*	-2.3908742	-0.101619594	-0.503311854	0.40169226	0.999933887	-0.036750786	0.638906904	-0.67565769	0.057261634
ENSG00000184371	*CSF1*	9.030867871	0.045765964	0.042896212	0.002869752	0.999933887	0.786239235	1.048009321	-0.261770085	0.057263221
ENSG00000155545	*MIER3*	2.372875115	-0.02901318	0.002629084	-0.031642264	0.999933887	-0.129548177	0.346909221	-0.476457398	0.057282506
ENSG00000184992	*BRI3BP*	2.270142437	-0.088797034	0.085032463	-0.173829497	0.999933887	-0.362022503	-0.774825686	0.412803183	0.057521037
ENSG00000165264	*NDUFB6*	3.530533971	0.004147485	0.061454884	-0.057307399	0.999933887	-0.046518287	-0.450429533	0.403911246	0.057641643
ENSG00000164683	*HEY1*	0.750912366	0.053071212	-0.151967071	0.205038283	0.999933887	-0.072434924	-0.677148107	0.604713183	0.057708403
ENSG00000093000	*NUP50*	6.170466448	-0.118567325	-0.078960531	-0.039606794	0.999933887	-0.879394659	-1.192893157	0.313498498	0.0577424
ENSG00000123933	*MXD4*	5.381072371	0.027585334	0.08166725	-0.054081916	0.999933887	0.033404653	-0.186632894	0.220037547	0.057756447
ENSG00000135968	*GCC2*	5.587919935	-0.135034739	-0.204013051	0.068978312	0.999933887	-0.1569041	0.467361343	-0.624265444	0.057769986
ENSG00000196730	*DAPK1*	5.095533678	-0.055058549	0.095049557	-0.150108106	0.999933887	-0.940050027	-1.21476644	0.274716413	0.057769986
ENSG00000260256	*AP001063.1*	0.70755853	0.254698388	0.112217777	0.142480611	0.999933887	1.743547929	2.285673705	-0.542125775	0.057799751
ENSG00000158864	*NDUFS2*	4.626938733	-0.077165754	-0.020133219	-0.057032535	0.999933887	-0.109893603	-0.317255255	0.207361652	0.057799751
ENSG00000287387	*AC010883.3*	-0.136598131	-0.087028069	-0.180539401	0.093511332	0.999933887	-0.433309472	-1.085288702	0.651979229	0.057799751
ENSG00000141446	*ESCO1*	5.754684402	-0.084445752	-0.112032629	0.027586877	0.999933887	-0.386451983	0.070326031	-0.456778014	0.057908471
ENSG00000054967	*RELT*	8.051449405	0.042475726	0.037559181	0.004916545	0.999933887	-0.28695498	-0.541642757	0.254687777	0.057908471
ENSG00000280739	*EIF1B-AS1*	0.219216351	-0.065104165	-0.132694894	0.067590729	0.999933887	0.037132445	0.739674586	-0.702542141	0.057908471
ENSG00000248527	*MTATP6P1*	6.138805734	-0.000451118	-0.149131914	0.148680796	0.999933887	-0.017138576	0.231542556	-0.248681132	0.057909756
ENSG00000081087	*OSTM1*	6.277784545	0.060412217	0.046956253	0.013455965	0.999933887	-0.043426756	0.182416642	-0.225843397	0.057918481
ENSG00000132716	*DCAF8*	5.477334221	-0.002868816	-0.028471555	0.025602739	0.999933887	0.142095925	0.35207725	-0.209981326	0.057977055
ENSG00000088205	*DDX18*	6.044616019	-0.011347628	-0.060308746	0.048961119	0.999933887	0.281397832	0.560079578	-0.278681746	0.058033055
ENSG00000239697	*TNFSF12*	2.605713151	0.070475823	0.075748304	-0.005272481	0.999933887	-0.038380836	-0.3869828	0.348601963	0.058311889
ENSG00000100221	*JOSD1*	7.530871127	0.031884976	0.025765319	0.006119658	0.999933887	0.116398954	0.306518821	-0.190119867	0.058538736
ENSG00000100242	*SUN2*	7.798020492	0.048428293	0.035651015	0.012777278	0.999933887	-0.123319149	-0.334540391	0.211221241	0.058604376
ENSG00000153317	*ASAP1*	8.61782687	0.006297766	-0.024828156	0.031125922	0.999933887	0.107658215	0.27782427	-0.170166055	0.058617999
ENSG00000120832	*MTERF2*	1.57760094	-0.051166349	-0.106020852	0.054854503	0.999933887	-0.232177152	-0.652752783	0.420575631	0.058673449
ENSG00000277496	*AL357033.4*	4.127884059	0.021945686	0.017394364	0.004551323	0.999933887	0.413327944	0.695638389	-0.282310445	0.058676144
ENSG00000176563	*CNTD1*	0.849921622	-0.024523219	-0.063970762	0.039447543	0.999933887	-0.165159997	0.375820703	-0.5409807	0.058676144
ENSG00000130827	*PLXNA3*	3.509834435	0.032325222	0.106290811	-0.073965588	0.999933887	0.17022771	0.397839203	-0.227611493	0.058688994
ENSG00000101888	*NXT2*	1.771333879	-0.027098827	-0.159631627	0.1325328	0.999933887	0.498995051	0.901013812	-0.402018761	0.058688994
ENSG00000023608	*SNAPC1*	5.926061147	0.144434737	-0.080947383	0.225382119	0.999933887	0.744101137	1.094634169	-0.350533032	0.058793625
ENSG00000081377	*CDC14B*	1.584956802	-0.130861296	0.015745982	-0.146607279	0.999933887	0.438471812	0.815797886	-0.377326074	0.058807619

ENSG00000279149	*AL356750.1*	-0.071284424	-0.047617387	0.237706467	-0.285323854	0.999933887	-0.868424058	-0.347563752	-0.520860306	0.058807619
ENSG00000179094	*PER1*	8.641527567	0.080245304	0.052149885	0.028095419	0.999933887	0.313926714	0.514776128	-0.200849415	0.058877162
ENSG00000158552	*ZFAND2B*	4.77322461	-0.024760051	0.016034699	-0.04079475	0.999933887	-0.271473565	-0.476774195	0.20530063	0.059033122
ENSG00000176208	*ATAD5*	0.264543424	-0.161064572	-0.068061804	-0.093002768	0.999933887	-0.373565535	0.305764052	-0.679329587	0.059073313
ENSG00000182093	*GET1*	1.409492335	-0.093981941	-0.009135318	-0.084846623	0.999933887	-0.853551784	-1.281076065	0.427524281	0.059073313
ENSG00000165929	*TC2N*	4.354385192	-0.066664959	-0.19106189	0.124396931	0.999933887	-0.162567801	0.182388014	-0.344955815	0.059073313
ENSG00000175984	*DENND2C*	0.209043659	0.071236502	-0.102262672	0.173499175	0.999933887	-0.455287315	0.215557297	-0.670844612	0.059073313
ENSG00000251661	*AC136475.1*	1.731188977	0.062797212	-0.022953705	0.085750917	0.999933887	-0.424980628	-1.086388283	0.661407655	0.059073313
ENSG00000047188	*YTHDC2*	4.427827909	-0.061869945	-0.048199342	-0.013670602	0.999933887	-0.160237745	0.222999637	-0.383237382	0.059076565
ENSG00000175573	*C11orf68*	3.857983646	0.087698865	-0.106897307	0.194596172	0.999933887	-0.592447242	-0.912026554	0.319579312	0.059078515
ENSG00000183255	*PTTG1IP*	6.834363011	0.022574501	-0.006096969	0.028671469	0.999933887	0.973924311	1.337779868	-0.363855557	0.059078515
ENSG00000134852	*CLOCK*	1.600355806	-0.040162725	-0.170432162	0.130269437	0.999933887	0.116818648	0.675487317	-0.558668669	0.059130661
ENSG00000100354	*TNRC6B*	6.717597879	0.014115646	0.001631295	0.012484351	0.999933887	-0.68225419	-0.93803592	0.25578173	0.059208073
ENSG00000269066	*AC020908.3*	0.96262286	0.126830351	0.150408749	-0.023578398	0.999933887	-0.241356266	0.272447491	-0.513803757	0.05930969
ENSG00000118900	*UBN1*	7.550583366	-0.033784487	0.011173709	-0.044958196	0.999933887	-0.536780577	-0.718964422	0.182183845	0.05930969
ENSG00000231233	*CFAP58-DT*	5.903288991	0.03209865	0.025203626	0.006895024	0.999933887	1.963455767	2.285593584	-0.322137817	0.05930969
ENSG00000115902	*SLC1A4*	3.295663791	-0.015346539	-0.016131719	0.00078518	0.999933887	-0.370539958	-0.757236061	0.386696103	0.05930969
ENSG00000100711	*ZFYVE21*	1.766824361	-0.064056724	0.137703845	-0.201760569	0.999933887	-0.403852337	-0.738175829	0.334323492	0.059325353
ENSG00000173926	*MARCHF3*	1.925062733	0.068118591	0.035044296	0.033074295	0.999933887	0.902695372	1.401086018	-0.498390646	0.059325353
ENSG00000145354	*CISD2*	1.566738669	-0.060688559	-0.020650572	-0.040037988	0.999933887	-0.324232549	-0.819050364	0.494817815	0.059325451
ENSG00000175155	*YPEL2*	3.81132491	-0.058360686	0.004940291	-0.063300977	0.999933887	-0.672330398	-0.950266183	0.277935785	0.05934181
ENSG00000188938	*FAM120AOS*	5.022446332	0.040705592	0.040306736	0.000398857	0.999933887	-0.16688055	-0.347450332	0.180569782	0.059368466
ENSG00000071794	*HLTF*	0.119667686	-0.210778101	-0.110268155	-0.100509946	0.999933887	-0.327912543	0.38075324	-0.708665783	0.059443278
ENSG00000163812	*ZDHHC3*	5.217216036	0.022688487	0.030963834	-0.008275347	0.999933887	-0.169245755	-0.394255391	0.225009636	0.059500683
ENSG00000182578	*CSF1R*	2.40575839	0.024587521	-0.06350981	0.088097331	0.999933887	-1.205076514	-1.794991626	0.589915112	0.059501464
ENSG00000164305	*CASP3*	4.915918345	-0.116647922	-0.102034427	-0.014613495	0.999933887	-0.082145712	0.109940098	-0.192085809	0.05953527
ENSG00000175215	*CTDSP2*	7.336803709	-0.052589477	-0.002983176	-0.0496063	0.999933887	-0.538839596	-0.758125628	0.219286032	0.059590792
ENSG00000058668	*ATP2B4*	6.562464284	-0.007567808	-0.014802512	0.007234704	0.999933887	-0.33572792	-0.54821205	0.21248413	0.059590792
ENSG00000225339	*AL354740.1*	1.257309833	-0.010997442	-0.197609543	0.186612101	0.999933887	0.160436594	0.526692052	-0.366255458	0.059590792
ENSG00000196591	*HDAC2*	4.061753944	-0.070064912	-0.019521204	-0.050543708	0.999933887	0.220312752	0.478997598	-0.258684845	0.059621239
ENSG00000228329	*LINC01890*	2.665614179	0.038105368	0.103566294	-0.065460927	0.999933887	0.327155756	0.660088035	-0.33293228	0.059621239
ENSG00000141424	*SLC39A6*	4.363878776	-0.055141139	-0.083246253	0.028105114	0.999933887	-0.453100435	-0.687528984	0.234428549	0.059662123
ENSG00000146556	*WASH2P*	2.389745298	-0.012313511	0.099925176	-0.112238687	0.999933887	-0.145159127	-0.447861882	0.302702756	0.059677596
ENSG00000277978	*AC010542.5*	-0.377246622	-0.468900118	-0.548373881	0.079473762	0.999933887	-1.014536205	-2.016157428	1.001621224	0.059927367
ENSG00000148719	*DNAJB12*	6.018189657	-0.008033895	-0.001736088	-0.006297807	0.999933887	0.129844225	0.279090308	-0.149246083	0.059927367
ENSG00000172771	*EFCAB12*	1.340594791	-0.108349377	0.048021651	-0.156371028	0.999933887	-0.317642809	-0.825833032	0.508190223	0.059938379
ENSG00000121742	*GJB6*	6.108709385	-0.077026718	-0.049863282	-0.027163436	0.999933887	-0.381388392	-0.15304301	-0.228345382	0.060141788
ENSG00000105662	*CRTC1*	2.991979198	0.059246964	0.082563185	-0.023316221	0.999933887	-0.130375431	-0.392034684	0.261659253	0.060141788
ENSG00000119574	*ZBTB45*	2.334401111	-0.00680056	0.010675979	-0.017476539	0.999933887	-0.252941612	-0.57252562	0.319584007	0.060161669
ENSG00000159335	*PTMS*	3.336328121	0.014928685	0.047920844	-0.032992159	0.999933887	-0.018212076	-0.381293418	0.363081342	0.060295248
ENSG00000188677	*PARVB*	1.108770068	0.02018897	0.148732501	-0.128543531	0.999933887	0.652263689	1.133926251	-0.481662562	0.060349298
ENSG00000179588	*ZFPM1*	3.106251715	0.047874259	0.086336772	-0.038462513	0.999933887	0.050449841	-0.278723089	0.32917293	0.06038871
ENSG00000266709	*AC005224.3*	1.884970272	0.032779881	-0.102734786	0.135514667	0.999933887	1.37056786	2.01456329	-0.64399543	0.060395897
ENSG00000073050	*XRCC1*	3.332548986	0.049064257	-0.004050494	0.053114751	0.999933887	-0.222049534	-0.506263683	0.284214148	0.060403903
ENSG00000170903	*MSANTD4*	1.5203384	-0.120254979	-0.129417407	0.009162428	0.999933887	-0.179694661	0.240286525	-0.419981186	0.060477256
ENSG00000124222	*STX16*	5.924803208	0.023943197	0.065028378	-0.041085181	0.999933887	-0.249247001	-0.426046178	0.176799177	0.060477256
ENSG00000127220	*ABHD8*	1.750607468	0.212177091	0.070909801	0.14126729	0.999933887	-0.094233932	-0.566682365	0.472448433	0.060519482
ENSG00000150347	*ARID5B*	4.556726981	-0.015117552	-0.100523154	0.085405602	0.999933887	0.17693684	0.488694731	-0.31175789	0.060519482
ENSG00000125538	*IL1B*	8.683670028	0.66009871	0.850344427	-0.190245718	0.999933887	2.548933538	3.265567783	-0.716634245	0.060538246
ENSG00000250138	*AC139495.2*	2.197596364	0.160502695	0.099587209	0.060915486	0.999933887	-0.755420314	-1.389545355	0.634125041	0.060538246
ENSG00000100239	*PPP6R2*	5.810256303	-0.011297873	0.070474753	-0.081772626	0.999933887	-0.181690343	-0.357334309	0.175643966	0.060538246
ENSG00000182179	*UBA7*	5.608369997	0.040243296	0.073311008	-0.033067712	0.999933887	0.039652991	-0.198074864	0.237727855	0.060551554
ENSG00000146425	*DYNLT1*	4.352696149	0.017400013	0.02135904	-0.003959028	0.999933887	-0.468014974	-0.720083731	0.252068757	0.060551554
ENSG00000182749	*PAQR7*	0.572458915	0.025969926	0.00784789	0.018122036	0.999933887	-0.366922071	-1.004772425	0.637850354	0.060578053
ENSG00000231507	*LINC01353*	3.610751096	0.18960387	0.171578043	0.018025827	0.999933887	1.402807182	1.778674741	-0.375867559	0.060601738
ENSG00000159792	*PSKH1*	2.8525903	0.018182074	-0.076360673	0.094542747	0.999933887	-0.286671522	-0.561967789	0.275296267	0.060601738
ENSG00000165995	*CACNB2*	0.165553225	-0.241059025	-0.308194942	0.067135917	0.999933887	0.09943097	1.067271244	-0.967840274	0.060672815
ENSG00000034713	*GABARAPL2*	8.43051702	-0.017708119	0.007722552	-0.02543067	0.999933887	1.026861504	1.243662058	-0.216800553	0.060672815
ENSG00000246526	*LINC02481*	1.437897349	-0.119060651	-0.009033255	-0.110027396	0.999933887	0.220635007	0.616993155	-0.396358148	0.060687333
ENSG00000140749	*IGSF6*	8.869906985	-0.012274821	-0.078176395	0.065901574	0.999933887	0.820771416	1.02768063	-0.206909213	0.060806593
ENSG00000131848	*ZSCAN5A*	-0.195292778	-0.010956336	0.07503412	-0.085990456	0.999933887	0.241956475	0.798766566	-0.55681009	0.060853553
ENSG00000100911	*PSME2*	5.345907174	0.019665832	-0.013377404	0.033043236	0.999933887	0.042229809	-0.162291051	0.20452086	0.060862777
ENSG00000267321	*SNHG30*	2.718914315	-0.010911206	-0.041379873	0.030468667	0.999933887	-0.148417387	-0.477749699	0.329332313	0.060903003
ENSG00000162298	*SYVN1*	5.081001471	0.093643021	0.025164175	0.068478846	0.999933887	-0.271716833	-0.45967847	0.187961637	0.060907198
ENSG00000155090	*KLF10*	8.313400343	0.239500191	0.253011262	-0.013511072	0.999933887	1.318958424	1.583954447	-0.264996022	0.060932122
ENSG00000234883	*MIR155HG*	1.846814388	0.052004178	0.325287796	-0.273283618	0.999933887	1.971293789	2.57518998	-0.603896192	0.061152639
ENSG00000166347	*CYB5A*	0.826570296	-0.076275552	0.127260684	-0.203536237	0.999933887	0.423429165	0.86971814	-0.446288975	0.061285755
ENSG00000100083	*GGA1*	7.82245223	0.051177969	0.059422161	-0.008244193	0.999933887	0.637421663	0.834469221	-0.197047559	0.061288802
ENSG00000080503	*SMARCA2*	5.978365671	0.006325543	-0.034239412	0.040564954	0.999933887	-0.115076107	-0.333245019	0.218168912	0.061567397
ENSG00000184489	*PTP4A3*	5.746185341	0.092380725	0.058994316	0.033386409	0.999933887	0.450161708	0.108965686	0.341196023	0.061809249
ENSG00000188295	*ZNF669*	2.28710075	0.015459547	-0.000536503	0.01599605	0.999933887	-0.071026612	-0.40721334	0.336186728	0.061917301
ENSG00000008294	*SPAG9*	9.449316124	-0.013737472	-0.01223278	-0.001504692	0.999933887	0.266964441	0.483989832	-0.21702539	0.062054893
ENSG00000114850	*SSR3*	3.888865677	-0.057028044	-0.036375637	-0.020652407	0.999933887	0.032382713	-0.187120307	0.21950302	0.06211776
ENSG00000153094	*BCL2L11*	5.622478607	-0.095559337	-0.052279805	-0.043279531	0.999933887	-0.79861379	-1.092414635	0.293800845	0.062167971
ENSG00000230417	*LINC00595*	1.272081954	0.257115039	0.126436137	0.130678902	0.999933887	0.674703145	1.020756039	-0.346052895	0.062167971
ENSG00000230417	*LINC00595*	1.272081954	0.257115039	0.126436137	0.130678902	0.999933887	0.674703145	1.020756039	-0.346052895	0.062167971
ENSG00000115211	*EIF2B4*	3.313277565	-0.051147733	0.055478429	-0.106626162	0.999933887	-0.374607667	-0.678723584	0.304115916	0.062420794
ENSG00000004864	*SLC25A13*	6.782723919	0.134327356	0.140721792	-0.006394436	0.999933887	0.282036104	0.512504997	-0.230468893	0.062498977
ENSG00000103154	*NECAB2*	3.260732849	-0.038761461	-0.030218691	-0.00854277	0.999933887	-0.874634895	-1.235108699	0.360473804	0.062498977
ENSG00000087502	*ERGIC2*	4.389767642	-0.022843129	-0.128712245	0.105869116	0.999933887	-0.571958298	-0.289907729	-0.282050569	0.06256791
ENSG00000100906	*NFKBIA*	11.251387314	0.470374894	0.522283926	-0.051909033	0.999933887	2.712002909	3.187404676	-0.475401767	0.062675731
ENSG00000175538	*KCNE3*	4.313408472	-0.153082849	-0.226663899	0.07358105	0.999933887	-0.244331782	-0.501584142	0.25725236	0.062831367
ENSG00000254004	*ZNF260*	0.438637006	-0.05563473	-0.054402562	-0.001232168	0.999933887	-0.209234017	0.496086934	-0.705320952	0.06294127
ENSG00000228010	*AC073343.2*	0.988839419	-0.160879602	0.16797998	-0.328859582	0.999933887	0.216724954	0.602072265	-0.385347311	0.0629444
ENSG00000120306	*CYSTM1*	7.372798632	0.02405108	0.025963434	-0.001912355	0.999933887	0.43995024	0.606805442	-0.166855202	0.063171682
ENSG00000140987	*ZSCAN32*	2.927204902	-0.131448721	-0.166725041	0.03527632	0.999933887	-0.284092214	-0.536028633	0.251936419	0.063180108
ENSG00000130203	*APOE*	0.25643152	-0.075793886	0.462671406	-0.538465292	0.999933887	0.05832458	1.041053425	-0.982728845	0.06318655
ENSG00000182923	*CEP63*	6.652284312	-0.049254209	-0.116633292	0.067379083	0.999933887	-0.827539733	-0.547015573	-0.28052416	0.063208645
ENSG00000123338	*NCKAP1L*	6.590561902	-0.008639435	0.008522736	-0.017162171	0.999933887	-0.174446953	-0.349364726	0.174917773	0.063270918
ENSG00000125351	*UPF3B*	2.945801199	-0.133075387	-0.051107149	-0.081968237	0.999933887	0.804146823	1.15201042	-0.347863597	0.063338109
ENSG00000230869	*AGAP10P*	-0.590573215	0.022676326	0.037862732	-0.015186406	0.999933887	-0.240078925	0.445508375	-0.6855873	0.063864309
ENSG00000100150	*DEPDC5*	3.024660622	0.038134981	-0.039975921	0.078110902	0.999933887	-0.285954258	-0.593073517	0.307119259	0.063913323
ENSG00000137714	*FDX1*	2.671646483	0.030075001	0.065394341	-0.03531934	0.999933887	-0.084826671	0.226281983	-0.311108654	0.063913323
ENSG00000125945	*ZNF436*	1.602633013	-0.025169369	0.133683561	-0.15885293	0.999933887	-0.190812984	-0.540983428	0.350170443	0.063916453
ENSG00000133574	*GIMAP4*	5.138412501	0.000216319	-0.059433956	0.059650275	0.999933887	-0.121720985	-0.40442461	0.282703625	0.063916453
ENSG00000109861	*CTSC*	6.443100407	0.001412165	-0.036070765	0.03748293	0.999933887	0.434904938	0.853773356	-0.418868418	0.063928689
ENSG00000157540	*DYRK1A*	7.624490451	-0.048232857	-0.059447629	0.011214772	0.999933887	-0.027453171	0.113999822	-0.141452993	0.064266208
ENSG00000237036	*ZEB1-AS1*	4.010593004	0.015376482	0.202256437	-0.186879955	0.999291887	0.193448049	0.420489099	-0.227041049	0.06429652
ENSG00000172530	*BANP*	6.442021183	0.033840827	-0.010775577	0.044616404	0.999933887	0.633515275	0.820004672	-0.186489397	0.064496971
ENSG00000111642	*CHD4*	4.826496749	-0.002753366	0.014723027	-0.017476393	0.999933887	0.550538658	0.75350433	-0.202965672	0.064501612
ENSG00000106605	*BLVRA*	3.057824942	-0.179882601	-0.034989737	-0.144892864	0.999933887	-1.161597823	-1.563889726	0.402291903	0.064542267
ENSG00000245148	*ARAP1-AS2*	4.514584002	0.000969875	0.006520644	-0.005550768	0.999933887	-0.052672646	0.294766626	-0.347439271	0.064542267
ENSG00000042980	*ADAM28*	2.914264954	-0.056227042	-0.063752983	0.007525941	0.999933887	-0.111212705	0.321796766	-0.433009472	0.064697908
ENSG00000273188	*AL022328.3*	1.251493974	-0.040213659	0.022970995	-0.063184654	0.999933887	-0.070806227	-0.58449514	0.513688913	0.064751551
ENSG00000074071	*MRPS34*	1.996562375	0.056894089	-0.086207985	0.143102074	0.999933887	-0.037868847	-0.410849023	0.372980176	0.064769938

ENSG00000196562	*SULF2*	7.051126062	0.01116693	-0.000967723	0.012134653	0.999933887	-1.201882384	-1.462112946	0.260230562	0.064769938
ENSG00000111328	*CDK2AP1*	2.645059649	0.078011274	0.004342571	0.073668703	0.999933887	0.079054215	-0.205182868	0.284237082	0.064912997
ENSG00000204283	*LINC01973*	1.901602316	0.039281032	0.103922899	-0.064641866	0.999933887	1.307732354	1.895051043	-0.587318689	0.065341411
ENSG00000257702	*LBX2-AS1*	0.346913954	0.198657578	0.415056248	-0.216398671	0.999933887	-0.184387257	0.169420756	-0.353808013	0.065532953
ENSG00000161996	*WDR90*	1.639236179	0.081301832	0.193669342	-0.11236751	0.999933887	0.29350306	0.654595828	-0.361092768	0.065532953
ENSG00000164190	*NIPBL*	7.179741251	-0.084149157	-0.15815503	0.074005874	0.999933887	-0.695228691	-0.179230471	-0.51599822	0.065589326
ENSG00000125462	*MIR9-1HG*	3.490534373	0.02238218	0.008609951	0.01377223	0.999933887	2.041030033	2.414326996	-0.373296963	0.065589326
ENSG00000079819	*EPB41L2*	0.405182601	0.030756294	-0.123925913	0.154682207	0.999933887	0.166791381	0.711585116	-0.544793735	0.06559643
ENSG00000258404	*LINC02320*	1.9138707	0.119505023	0.122788796	-0.003283773	0.999933887	1.268199254	1.616847782	-0.348648527	0.065630025
ENSG00000100266	*PACSIN2*	9.250750944	-0.000326068	0.002279681	-0.002605749	0.999933887	0.032936566	0.186703377	-0.153766812	0.065728378
ENSG00000183604	*SMG1P5*	2.255164223	0.070898558	0.059737128	0.01116143	0.999933887	-0.265296806	-0.603593059	0.338296253	0.065828176
ENSG00000196968	*FUT11*	2.801028062	-0.156019291	0.000587806	-0.156607097	0.999933887	-0.179621764	-0.419172179	0.239550416	0.065868662
ENSG00000261218	*AC099524.1*	0.051414871	-0.150756764	0.163483982	-0.314240746	0.999933887	-0.606827765	-1.435978367	0.829150602	0.065974568
ENSG00000165678	*GHITM*	6.851173528	-0.02351366	-0.036781502	0.013267841	0.999933887	0.188395506	0.344406631	-0.156011125	0.065990783
ENSG00000234127	*TRIM26*	5.23955782	0.033917977	0.043485172	-0.009567195	0.999933887	-0.125735141	-0.369031476	0.243296336	0.066085134
ENSG00000104756	*KCTD9*	3.841392539	-0.124289741	-0.065028862	-0.059260879	0.999933887	-0.4488999	-0.212354109	-0.236545791	0.066097896
ENSG00000167595	*PROSER3*	3.081079205	-0.051181652	-0.039813695	-0.011367958	0.999933887	-0.275730876	-0.57293647	0.297205594	0.066097896
ENSG00000176142	*TMEM39A*	3.423392654	-0.042118087	0.022129419	-0.064247506	0.999933887	0.22878986	0.471380159	-0.242590298	0.066097896
ENSG00000237943	*PRKCQ-AS1*	3.114383538	-0.127614745	-0.191511984	0.063897238	0.999933887	0.134137967	0.389307659	-0.255169691	0.066131955
ENSG00000168439	*STIP1*	5.416357058	-0.053103375	-0.054361791	0.001258417	0.999933887	0.257675668	0.452766506	-0.195090838	0.066131955
ENSG00000102738	*MRPS31*	1.071371923	-0.088877199	-0.147316381	0.058439181	0.999933887	-0.032911299	0.34224374	-0.375155039	0.066131955
ENSG00000180530	*NRIP1*	2.935064038	-0.087748355	-0.154412389	0.066664034	0.999933887	0.339165213	0.918290314	-0.5791251	0.066131955
ENSG00000154529	*CNTNAP3B*	0.164338136	-0.045204698	0.044197634	-0.089402332	0.999933887	-0.841090328	-1.512343899	0.67125357	0.066131955
ENSG00000141854	*MISP3*	-0.218478687	0.036532576	-0.069508272	0.106040848	0.999933887	0.240340463	-0.391906096	0.632246559	0.066131955
ENSG00000159640	*ACE*	-0.097848197	0.021114042	0.00977857	0.011335472	0.999933887	-0.120522284	0.490756946	-0.611279231	0.066131955
ENSG00000116991	*SIPA1L2*	7.059765456	-0.002465766	-0.003095441	0.000629675	0.999933887	-0.722332292	-0.948477267	0.226144975	0.066131955
ENSG00000136897	*MRPL50*	1.808272665	0.070278626	-0.096709158	0.166987784	0.999933887	0.183995357	0.539572285	-0.355576928	0.066150787
ENSG00000121774	*KHDRBS1*	6.541624255	-0.041004416	0.003454652	-0.044459067	0.999933887	0.113627072	0.261051673	-0.147424601	0.066165184
ENSG00000072364	*AFF4*	8.045205172	-0.042744971	-0.107255514	0.064510544	0.999933887	-0.051182006	0.218082019	-0.269264025	0.066353345
ENSG00000105963	*ADAP1*	3.930915143	0.057806309	0.079872752	-0.022066443	0.999933887	-0.153165749	-0.490374273	0.337208524	0.066395632
ENSG00000234518	*PTGES3P1*	3.509969296	-0.170689144	-0.187617196	0.016928052	0.999933887	0.973024903	1.518535905	-0.545511002	0.066449356
ENSG00000131873	*CHSY1*	6.11151017	0.051567797	0.128321269	-0.076753471	0.999933887	-0.438070194	-0.706946803	0.268876609	0.066449356
ENSG00000160179	*ABCG1*	5.906283735	0.094765889	0.030084932	0.064680957	0.999933887	-0.262173079	0.102352325	-0.364525404	0.066513155
ENSG00000149557	*FEZ1*	-0.107759909	0.01380753	0.013545309	0.000262222	0.999933887	0.559559603	1.149006274	-0.589446671	0.066541392
ENSG00000101346	*POFUT1*	2.366953622	0.029307104	0.017982233	0.011324871	0.999933887	0.312721323	0.63023067	-0.317509347	0.066708357
ENSG00000104290	*FZD3*	-0.289785452	-0.04854393	-0.052333323	0.003789393	0.999933887	-0.552032707	0.18293912	-0.734971828	0.066812067
ENSG00000258289	*CHURC1*	5.236348311	-0.060218	-0.148289308	0.088071307	0.999933887	-0.11637824	0.139920283	-0.256298523	0.066864349
ENSG00000264522	*OTUD7B*	1.398080655	0.060173302	0.070896856	-0.010723555	0.999933887	-0.190182929	0.198183883	-0.388366813	0.066864349
ENSG00000014164	*ZC3H3*	5.205053215	0.02215315	0.019218869	0.002934281	0.999933887	-0.255357502	-0.465219423	0.20986192	0.066864349
ENSG00000156804	*FBXO32*	2.964133122	-0.100449807	-0.001483316	-0.09896649	0.999933887	-0.202630785	-0.475027904	0.272397119	0.066903895
ENSG00000133789	*SWAP70*	4.098286265	0.017338611	0.059155479	-0.041816868	0.999933887	-0.021769304	0.197579917	-0.219349221	0.066940078
ENSG00000198814	*GK*	8.2581606	-0.027820714	0.000365881	-0.028186594	0.999933887	0.248390072	0.501679908	-0.253289836	0.067211581
ENSG00000227268	*KLLN*	1.746037093	-0.123505445	-0.083330769	-0.040174676	0.999933887	-0.266519989	-0.791532211	0.525012222	0.067228411
ENSG00000076641	*PAG1*	8.053093852	-0.040026465	-0.040498131	0.000471666	0.999933887	-0.274732765	-0.071643118	-0.203089646	0.06724967
ENSG00000087008	*ACOX3*	2.826783796	0.138576311	-0.094523924	0.233100235	0.999933887	1.111083131	1.472174008	-0.361090877	0.067398475
ENSG00000074660	*SCARF1*	9.461748187	0.064796023	0.09041919	-0.025623167	0.999933887	0.962387772	1.183461021	-0.221073248	0.067398475
ENSG00000145632	*PLK2*	2.687443616	0.194982484	0.083302375	0.111680109	0.999933887	1.604479365	2.034844136	-0.430364771	0.067404541
ENSG00000137700	*SLC37A4*	1.184019635	0.155248845	0.124247728	0.031001117	0.999933887	0.204449469	-0.133656298	0.338105767	0.067404541
ENSG00000197885	*NKIRAS1*	1.717916596	0.1108849	0.119576378	-0.008691478	0.999933887	1.428841164	1.856735448	-0.427894284	0.067404541
ENSG00000225648	*SBDSP1*	2.985684641	-0.10642342	-0.145239261	0.038815841	0.999933887	0.191795876	0.488435998	-0.296640123	0.067493997
ENSG00000182606	*TRAK1*	4.695660797	-0.025041292	0.002158429	-0.027199721	0.999933887	0.575320503	0.881403324	-0.306082821	0.067493997
ENSG00000070190	*DAPP1*	5.673982029	-0.002708992	0.015632137	-0.018341129	0.999933887	0.584962349	0.84256917	-0.257606821	0.067493997
ENSG00000117153	*KLHL12*	4.642522108	-0.049396822	0.022798255	-0.072195077	0.999933887	0.283019322	0.553768277	-0.270748954	0.067585486
ENSG00000198756	*COLGALT2*	0.298282343	0.070961274	-0.021321014	0.092282288	0.999933887	0.018032324	0.600678962	-0.582646638	0.067927742
ENSG00000154188	*ANGPT1*	0.418991699	0.192276723	-0.021370044	0.213646766	0.999933887	-0.696817123	-1.592553252	0.895736129	0.068006062
ENSG00000168137	*SETD5*	7.135247208	-0.037151764	-0.03940406	0.002252296	0.999933887	0.187749084	0.351823874	-0.16407479	0.068263371
ENSG00000130487	*KLHDC7B*	1.289505553	0.09213815	-0.003453631	0.095591781	0.999933887	-0.290740631	-0.73643318	0.445692549	0.068271276
ENSG00000143373	*ZNF687*	6.110674874	-0.0215566	-0.017190585	-0.004366015	0.999933887	0.006641405	-0.162553357	0.169194762	0.068313406
ENSG00000162747	*FCGR3B*	10.584646055	-0.026751794	-0.136641597	0.109889804	0.999933887	-0.618652933	-0.890860845	0.272207912	0.068313406
ENSG00000121104	*FAM117A*	4.372921863	0.049366921	-0.046185674	0.095552594	0.999933887	-0.163061246	-0.430290841	0.267229595	0.068376463
ENSG00000159692	*CTBP1*	5.325010077	0.057153131	-0.026092891	0.083246022	0.999933887	-0.086725778	-0.248701187	0.161975409	0.068383165
ENSG00000100784	*RPS6KA5*	4.66171742	-0.043597818	-0.024316522	-0.019281296	0.999933887	-0.521283241	-0.858617162	0.337333922	0.068383165
ENSG00000140465	*CYP1A1*	0.319149994	-0.14962225	0.090113133	-0.239735384	0.999933887	0.59449895	0.967644837	-0.373145888	0.068505494
ENSG00000153234	*NR4A2*	7.492828573	0.120028068	0.116934472	0.003093596	0.999933887	0.376978055	0.658115654	-0.2811376	0.068518743
ENSG00000249786	*EAF1-AS1*	-0.118307768	-0.067962055	-0.047624817	-0.020337238	0.999933887	0.886008462	1.561386676	-0.675378214	0.068518743
ENSG00000071994	*PDCD2*	3.557510001	-0.011248017	-0.023765891	0.012517875	0.999933887	-0.068387427	-0.269703744	0.201316316	0.068537483
ENSG00000215845	*TSTD1*	3.255420379	-0.055131423	-0.024627705	-0.030503717	0.999933887	-0.118328229	-0.40064237	0.282314141	0.068546903
ENSG00000138185	*ENTPD1*	5.474851589	-0.045025579	-0.086672159	0.04164658	0.999933887	-0.633944499	-0.92761732	0.29367282	0.068870989
ENSG00000178093	*TSSK6*	0.683746446	-0.182457443	-0.077456298	-0.105001145	0.999933887	0.093787619	-0.44930928	0.543096899	0.068914124
ENSG00000120278	*PLEKHG1*	1.320949263	-0.053169694	0.106419311	-0.159589005	0.999933887	0.319169905	0.721999651	-0.402829746	0.069040763
ENSG00000082641	*NFE2L1*	5.273086283	-0.008822494	-0.007575427	-0.001247067	0.999933887	-0.457477787	-0.695842076	0.238364289	0.069074164
ENSG00000160551	*TAOK1*	6.403524069	0.002193909	-0.089381076	0.091574984	0.999933887	-0.549027765	-0.212553298	-0.336474468	0.069234986
ENSG00000182327	*GLTPD2*	0.835674144	0.134093381	0.071259209	0.062834173	0.999933887	0.175732775	0.780715073	-0.604982299	0.069381298
ENSG00000101986	*ABCD1*	4.26413475	0.059732319	0.043190455	0.016541865	0.999933887	-0.865975827	-1.138432502	0.272456674	0.069381298
ENSG00000211694	*TRGV10*	0.564055262	-0.005502933	-0.044847427	0.039344494	0.999933887	0.032616409	0.497256998	-0.464640589	0.069391441
ENSG00000163788	*SNRK*	6.810766155	-0.035188081	-0.009358166	-0.025829915	0.999933887	-0.255890041	-0.064346719	-0.191543322	0.069398446
ENSG00000125107	*CNOT1*	7.218253436	-0.045717655	-0.118661175	0.07294352	0.999933887	0.165524027	0.347409773	-0.181885746	0.069461114
ENSG00000243055	*GK-AS1*	3.721928708	-0.084790452	0.087975006	-0.172765458	0.999933887	-0.027431929	0.378722097	-0.406154026	0.069461114
ENSG00000181404	*WASHC1*	1.984190246	0.10136423	-0.001021592	0.102385822	0.999933887	-0.318254315	-0.709016109	0.390761794	0.069493162
ENSG00000119927	*GPAM*	0.841642061	-0.020775352	-0.256741605	0.235966253	0.999933887	0.191877394	0.690123006	-0.498245612	0.069502599
ENSG00000071967	*CYBRD1*	2.167408865	-0.061847844	0.130366962	-0.192214806	0.999933887	-0.756422003	-1.272440575	0.516018571	0.069575649
ENSG00000113555	*PCDH12*	1.253882536	0.21874856	-0.128495584	0.347244144	0.981460244	-0.01841572	0.424392484	-0.442808205	0.069586432
ENSG00000107872	*FBXL15*	2.613797311	0.164675494	0.087117814	0.07755768	0.999933887	0.72008978	1.073954364	-0.353864585	0.069693775
ENSG00000203710	*CR1*	7.451891882	-0.038194792	-0.072688619	0.034493827	0.999933887	-0.182761159	-0.328035014	0.145273855	0.069737276
ENSG00000158769	*F11R*	5.482735667	-0.046735145	0.006949321	-0.053684467	0.999933887	-0.469346427	-0.777001258	0.307654832	0.069741859
ENSG00000137857	*DUOX1*	-0.086912018	-0.298894389	0.180803186	-0.479697575	0.999933887	0.475478579	1.013450121	-0.537971542	0.069806796
ENSG00000153774	*CFDP1*	3.142035822	0.005000587	0.002580002	0.002420585	0.999933887	0.019649114	0.235795579	-0.216146466	0.069957981
ENSG00000215305	*VPS16*	4.60060343	0.01649529	0.054276105	-0.037780815	0.999933887	-0.070171989	-0.276835098	0.206663109	0.070051184
ENSG00000038358	*EDC4*	5.057720152	0.024360808	0.093196651	-0.068835844	0.999933887	-0.092684995	-0.272005111	0.179320115	0.070060142
ENSG00000171603	*CLSTN1*	5.285193977	0.004854657	0.013274761	-0.008420104	0.999933887	-0.071435096	-0.250303145	0.178868049	0.070217106
ENSG00000132953	*XPO4*	2.977932849	-0.099282193	-0.197662795	0.098380602	0.999933887	-0.108248552	0.249552998	-0.35780155	0.070335538
ENSG00000143319	*ISG20L2*	5.689154658	0.041365023	0.022853958	0.018511066	0.999933887	0.112164554	-0.05127491	0.163439464	0.070335538
ENSG00000131002	*TXLNGY*	-3.058917679	0.060247978	0.286962011	-0.226714033	0.999933887	-0.601699178	0.101350652	-0.70304983	0.070388792
ENSG00000156471	*PTDSS1*	4.272446362	-0.004076968	-0.027652869	0.0235759	0.999933887	-0.114090083	-0.334290365	0.220200282	0.070532836
ENSG00000276644	*DACH1*	1.701148365	0.019550347	0.049184455	-0.029634108	0.999933887	-0.331397749	-0.760377237	0.428979488	0.070533343
ENSG00000121797	*CCRL2*	9.27961563	0.177088224	0.150688287	0.026399937	0.999933887	0.968519999	1.220824993	-0.252304994	0.070614513
ENSG00000140948	*ZCCHC14*	4.406145007	-0.002775292	0.060360712	-0.063136003	0.999933887	0.164161574	0.359367897	-0.195206323	0.070629268
ENSG00000116670	*MAD2L2*	3.319725389	0.085765228	0.074380516	0.011384711	0.999933887	0.269117426	0.585012771	-0.315895345	0.070665163
ENSG00000279080	*AL022322.2*	0.663344599	-0.026206628	-0.025828465	-0.000378162	0.999933887	0.574202654	1.100419569	-0.526216914	0.070678741
ENSG00000089693	*MLF2*	7.258258098	0.031915006	0.020386153	0.011528853	0.999933887	-0.150060393	-0.31182961	0.161769218	0.070776572
ENSG00000100422	*CERK*	6.788342663	0.020748829	0.010059439	0.01068939	0.999933887	0.352702	0.540414495	-0.187712494	0.070997003
ENSG00000272645	*GTF2IP20*	2.214093417	0.045021252	-0.019615347	0.064636599	0.999933887	0.468902894	0.857676745	-0.38877385	0.071193228
ENSG00000257122	*RRN3P3*	3.173837298	-0.044007268	-0.005300036	-0.038707232	0.999933887	-0.150561425	0.244477461	-0.395038886	0.071193228
ENSG00000196358	*NTNG2*	6.080018809	-0.013336111	0.083847647	-0.097183758	0.999933887	0.049422385	-0.125319618	0.174742003	0.071239573
ENSG00000183431	*SF3A3*	4.41264409	-0.068105899	-0.027742753	-0.040363145	0.999933887	-0.211604512	-0.414504645	0.202900133	0.07137411

ENSG00000179134	*SAMD4B*	7.366603729	0.021938984	-0.041086181	0.063025165	0.999933887	0.343874946	0.519650456	-0.17577551	0.07154832
ENSG00000261416	*AC012645.3*	1.106662166	-0.073589172	-0.063084925	-0.010504246	0.999933887	-0.605006662	-1.197425773	0.59241911	0.071577488
ENSG00000186130	*ZBTB6*	2.904495848	-0.245662808	-0.384441136	0.138778327	0.999933887	-0.287314073	0.033118342	-0.320432415	0.07184797
ENSG00000111817	*DSE*	6.164804694	-0.067888782	-0.018787702	-0.04910108	0.999933887	0.891811264	1.199093922	-0.307282658	0.07184797
ENSG00000085063	*CD59*	5.803927278	-0.040911262	-0.068781911	0.027870649	0.999933887	0.831704217	1.082961044	-0.251256827	0.071929796
ENSG00000115685	*PPP1R7*	3.698670207	0.043128307	-0.055368816	0.098497123	0.999933887	-0.050618514	-0.322122406	0.271503893	0.072081434
ENSG00000099821	*POLRMT*	4.063876085	0.05097835	0.046033752	0.004944598	0.999933887	0.160683922	-0.085386887	0.246070808	0.072140107
ENSG00000232618	*AL355304.1*	2.088166132	-0.025589664	0.043146526	-0.06873619	0.999933887	2.139970189	2.540792917	-0.400822728	0.072149212
ENSG00000198898	*CAPZA2*	7.305577892	-0.079863647	-0.117014271	0.037150624	0.999933887	-0.222859873	-0.021441723	-0.20141815	0.072213057
ENSG00000119321	*FKBP15*	7.252149514	-0.046142154	-0.068968468	0.022826314	0.999933887	-0.241034771	-0.058492706	-0.182542064	0.072220829
ENSG00000122033	*MTIF3*	2.262526335	0.04057186	0.003471688	0.037100172	0.999933887	-0.2831393	-0.655736333	0.372597032	0.072445183
ENSG00000070495	*JMJD6*	7.979107113	0.175145053	0.128947379	0.046197674	0.999933887	0.620035392	0.902184003	-0.282148611	0.072508371
ENSG00000050327	*ARHGEF5*	0.22209739	0.007491473	0.221246224	-0.21375475	0.999933887	-0.265472352	0.341776403	-0.607248755	0.072645451
ENSG00000132330	*SCLY*	0.317544245	0.120925085	-0.211760808	0.332685894	0.999933887	0.013799822	-0.512138533	0.525938355	0.072665162
ENSG00000198888	*MT-ND1*	8.980604584	0.061688084	-0.010728153	0.072416237	0.999933887	0.107772864	0.384820907	-0.277048043	0.072686694
ENSG00000233429	*HOTAIRM1*	2.456812701	-0.234744469	-0.084648198	-0.15009627	0.999933887	-1.4480322	-1.900113827	0.452081627	0.072696158
ENSG00000196428	*TSC22D2*	7.372765204	0.096551867	0.168098114	-0.071546247	0.999933887	0.575365664	0.77003258	-0.194666916	0.072696158
ENSG00000272837	*OR10AE3P*	2.291622168	0.247987111	0.214418516	0.033568595	0.999933887	0.96438017	1.327085401	-0.362705232	0.07273399
ENSG00000136754	*ABI1*	6.584156638	-0.03510435	-0.04447133	0.00936698	0.999933887	-0.108298421	0.040619585	-0.148918006	0.07273399
ENSG00000060642	*PIGV*	2.855831322	-0.047773484	0.059576829	-0.107350313	0.999933887	1.339954065	1.694561156	-0.354607091	0.07273399
ENSG00000270170	*NCBP2AS2*	2.415080808	-0.04205049	-0.157743622	0.115693132	0.999933887	-0.140623621	-0.439569253	0.298945632	0.072838902
ENSG00000134330	*IAH1*	3.265824252	-0.039019056	-0.115412425	0.07639337	0.999933887	0.618152737	0.85794467	-0.239791933	0.072878109
ENSG00000122218	*COPA*	6.949514049	-0.022279094	-0.018796175	-0.003482919	0.999933887	0.04704086	0.184343895	-0.137303035	0.072938647
ENSG00000253218	*KLF3P1*	-0.989694179	0.687726242	-0.011133039	0.698859281	0.999933887	1.600797469	2.39031111	-0.789513641	0.073060658
ENSG00000129675	*ARHGEF6*	5.500274411	-0.021174216	-0.039543986	0.018369771	0.999933887	-0.181040082	-0.354928575	0.173888492	0.073153573
ENSG00000173821	*RNF213*	9.941437057	0.039610917	-0.015700628	0.055311545	0.999933887	-0.307940691	-0.521540085	0.213599394	0.073249396
ENSG00000053254	*FOXN3*	6.752509	0.002875589	-0.013346438	0.016222027	0.999933887	-0.536452324	-0.726608199	0.190155875	0.073273741
ENSG00000134463	*ECHDC3*	-0.444256528	-0.004895119	0.112052951	-0.11694807	0.999933887	-0.353689731	0.562846825	-0.916536557	0.073273741
ENSG00000269837	*IPO5P1*	0.594013458	-0.166938211	0.001104878	-0.168043089	0.999933887	-0.047137889	-0.544971255	0.497833367	0.073431992
ENSG00000151743	*AMN1*	3.357948148	-0.090433068	-0.108767199	0.018334131	0.999933887	-0.03704372	0.174914099	-0.211957819	0.073458979
ENSG00000119596	*YLPM1*	4.899197273	0.001063039	-0.028603281	0.02966632	0.999933887	-0.228919562	-0.412635856	0.183716294	0.073458979
ENSG00000141568	*FOXK2*	3.735550946	-0.004356859	0.078720285	-0.083077143	0.999933887	-0.169626842	-0.390840354	0.221213512	0.073474449
ENSG00000105701	*FKBP8*	8.357538712	0.061255898	0.039608028	0.02164787	0.999933887	-0.213838173	-0.492695008	0.278856835	0.073671867
ENSG00000159082	*SYNJ1*	5.448584922	-0.022065995	-0.061512126	0.03944613	0.999933887	-0.533135464	-0.21222609	-0.320909374	0.073767647
ENSG00000163902	*RPN1*	6.83426712	-0.037010222	-0.038553427	0.001543205	0.999933887	-0.501773186	-0.695053381	0.193280195	0.073775017
ENSG00000000938	*FGR*	10.396319838	0.040178539	0.040135454	4.3084e-05	0.999933887	0.511760109	0.681251555	-0.169491446	0.073815165
ENSG00000162601	*MYSM1*	5.69542748	-0.060743138	-0.182370784	0.121627646	0.999933887	-0.315077013	0.30471781	-0.619794824	0.073815165
ENSG00000149483	*TMEM138*	3.860073476	-0.019330892	0.033903958	-0.05323485	0.999933887	0.543039504	0.762450798	-0.219411294	0.073815165
ENSG00000120071	*KANSL1*	6.399923103	-0.026818833	0.040208966	-0.067027798	0.999933887	-0.491182444	-0.668418272	0.177235829	0.074005086
ENSG00000130755	*GMFG*	8.405344904	-0.01303103	-0.036528928	0.023497898	0.999933887	-0.240672261	-0.428161413	0.187489152	0.074066285
ENSG00000072818	*ACAP1*	7.015680698	0.028126674	0.049471298	-0.021344625	0.999933887	0.002294416	-0.201398283	0.203692699	0.074182239
ENSG00000167100	*SAMD14*	-0.864445926	0.471203907	-0.226028913	0.69723282	0.999933887	2.169822174	2.85126605	-0.681443876	0.07430869
ENSG00000166483	*WEE1*	2.2445774	-0.072734927	-0.082541169	0.009806242	0.999933887	0.232013659	0.531322509	-0.29930885	0.07430869
ENSG00000158195	*WASF2*	7.06743894	-0.021695868	-0.011115247	-0.010580621	0.999933887	-0.408441015	-0.575557124	0.167116109	0.07430869
ENSG00000135272	*MDFIC*	3.323635118	-0.015200505	-0.016133097	0.000932591	0.999933887	0.065329167	0.427024631	-0.361695464	0.07430869
ENSG00000130813	*SHFL*	4.308028998	0.068001986	-0.016736792	0.084738778	0.999933887	-0.351618557	-0.617174196	0.265555639	0.074498924
ENSG00000136169	*SETDB2*	5.339848715	0.067023573	0.078218672	-0.011195099	0.999933887	0.238403055	0.484422764	-0.246019708	0.074583932
ENSG00000255112	*CHMP1B*	7.148321043	-0.025998632	-0.041697754	0.015699122	0.999933887	0.024480259	-0.150731191	0.175211451	0.074583932
ENSG00000099795	*NDUFB7*	2.732125879	0.102822402	-0.02256411	0.125386512	0.999933887	0.120633971	-0.234372566	0.355006537	0.074611564
ENSG00000117408	*IPO13*	2.822253328	0.068887036	0.102941	-0.034053964	0.999933887	-0.082619999	-0.369149355	0.286529357	0.074668978
ENSG00000140983	*RHOT2*	6.55917094	0.052413787	0.101870332	-0.049456545	0.999933887	0.030285365	-0.16844624	0.198731604	0.074724517
ENSG00000103657	*HERC1*	6.84917979	-0.033184562	-0.045035569	0.011851007	0.999933887	-0.154248738	-0.349203254	0.194954516	0.074724517
ENSG00000138942	*RNF185*	5.844334453	-0.022637705	-0.043848405	0.0212107	0.999933887	0.074744146	0.261504334	-0.186760188	0.074819991
ENSG00000081237	*PTPRC*	10.370218142	-0.061863045	-0.2108921	0.149029054	0.999933887	-0.138102866	0.294877193	-0.432980059	0.074849221
ENSG00000124882	*EREG*	2.92901325	0.504787474	0.614340346	-0.109552872	0.999933887	1.810807195	2.571385346	-0.760578151	0.075073322
ENSG00000275052	*PPP4R3B*	5.96618128	-0.025842373	-0.071460146	0.045617772	0.999933887	-0.370746443	-0.127657736	-0.243088707	0.07519984
ENSG00000076984	*MAP2K7*	5.688874687	0.044865145	0.063293853	-0.018428708	0.999933887	-0.273008545	-0.490920206	0.217911661	0.075231217
ENSG00000010610	*CD4*	4.440126905	0.036688251	-0.008103776	0.044792027	0.999933887	0.04887548	-0.196424263	0.245299743	0.075231217
ENSG00000161904	*LEMD2*	5.719522373	-0.029906107	0.046504779	-0.076410886	0.999933887	-0.238395697	-0.427898939	0.189503242	0.075989295
ENSG00000237190	*CDKN2AIPNL*	1.769277323	0.087156143	0.097675652	-0.010519509	0.999933887	0.084903311	0.471711899	-0.386808588	0.07602411
ENSG00000121964	*GTDC1*	4.478742104	-0.015865023	-0.064216941	0.048351917	0.999933887	0.066823864	0.242631743	-0.17580788	0.07614709
ENSG00000171988	*JMJD1C*	9.327119403	-0.098373818	-0.140726147	0.042352329	0.999933887	-0.593018379	-0.054917122	-0.538101256	0.07616359
ENSG00000174720	*LARP7*	4.421011863	-0.06067613	-0.28457339	0.22389726	0.999933887	-0.096673663	0.315686815	-0.412360478	0.076222275
ENSG00000234678	*ELF3-AS1*	0.481730902	0.032210124	-0.135786607	0.167996731	0.999933887	-0.603076058	-1.193070401	0.589994343	0.076222275
ENSG00000172348	*RCAN2*	-0.698716885	-0.110778764	-0.149540095	0.038761331	0.999933887	-0.056061834	0.565994847	-0.622056681	0.07641288
ENSG00000130734	*ATG4D*	4.135295663	0.095296745	0.117231643	-0.021934898	0.999933887	-0.064768038	-0.337784829	0.273016791	0.076568709
ENSG00000144021	*CIAO1*	5.019215158	-0.005791715	-0.010122979	0.004331264	0.999933887	-0.189326838	-0.36593209	0.176605252	0.076624773
ENSG00000103266	*STUB1*	3.198104171	0.059864966	0.072720801	-0.012855835	0.999933887	-0.114736702	-0.479937084	0.365200382	0.076714828
ENSG00000276809	*AL138955.1*	0.899839553	-0.016205076	0.377377247	-0.393582323	0.999933887	1.072956572	1.563495584	-0.490539012	0.076714828
ENSG00000090339	*ICAM1*	9.146844484	0.173766103	0.147916766	0.025849337	0.999933887	2.317859041	2.639269358	-0.321410317	0.076906361
ENSG00000243660	*ZNF487*	2.596871708	-0.025615216	-0.064460399	0.038845183	0.999933887	-0.518445665	-0.795867986	0.277422321	0.077295655
ENSG00000173674	*EIF1AX*	4.046784186	-0.078805595	-0.118842905	0.04003731	0.999933887	0.027851706	0.271514083	-0.243662376	0.077339698
ENSG00000180573	*H2AC6*	6.886647227	0.012679842	-0.022369214	0.035049056	0.999933887	-0.630424632	-0.915873476	0.285448843	0.077369867
ENSG00000125744	*RTN2*	4.148278245	0.141908103	0.039477945	0.102430158	0.999933887	0.761752953	1.085140285	-0.323387333	0.077399235
ENSG00000174276	*ZNHIT2*	0.128115771	0.158668241	-0.105402393	0.264070634	0.999933887	0.298557404	-0.3412088	0.639766204	0.077399235
ENSG00000166888	*STAT6*	9.146789959	-0.015089137	6.4151e-05	-0.015153287	0.999933887	-0.572907873	-0.733745303	0.16083743	0.077403716
ENSG00000104679	*R3HCC1*	2.451684981	-0.075927902	-0.12291581	0.046987908	0.999933887	0.017833877	-0.285776984	0.303610861	0.077426312
ENSG00000175309	*PHYKPL*	5.21525258	0.009226611	0.036098707	-0.026872096	0.999933887	-0.263396565	-0.438745362	0.175348798	0.077426312
ENSG00000170779	*CDCA4*	2.315532622	0.044566231	-0.152196314	0.196762545	0.999933887	-0.273827152	-0.616372241	0.342545089	0.077549176
ENSG00000137841	*PLCB2*	7.382253873	0.028722786	0.088618105	-0.059895319	0.999933887	-0.033669505	-0.216234395	0.18256489	0.077643225
ENSG00000172840	*PDP2*	1.528740082	0.039720892	0.133606672	-0.09388578	0.999933887	-0.477360626	-0.849174009	0.371813383	0.077643225
ENSG00000187239	*FNBP1*	7.054889915	0.001043335	-0.06917665	0.070219985	0.999933887	0.324839846	0.554379396	-0.229539551	0.077762462
ENSG00000110321	*EIF4G2*	8.590448758	-0.037156532	-0.073417835	0.036261304	0.999933887	-0.29872794	-0.433882908	0.135154968	0.077776622
ENSG00000111845	*PAK1IP1*	1.241078461	-0.10851592	-0.093116066	-0.015399854	0.999933887	0.18734766	0.546475515	-0.359127856	0.077986305
ENSG00000165406	*MARCHF8*	6.574376188	-0.058806815	-0.005671192	-0.053135623	0.999933887	-0.755098746	-0.957387644	0.202288899	0.077986305
ENSG00000070061	*ELP1*	3.751935374	-0.032207858	-0.124613799	0.09240594	0.999933887	-0.050407611	0.183923255	-0.234330866	0.078056865
ENSG00000220305	*HNRNPH1P1*	0.317824866	-0.126559879	-0.082064847	-0.044495032	0.999933887	1.357770108	1.927671583	-0.569901475	0.078416354
ENSG00000256660	*CLEC12B*	2.080038988	-0.020939656	-0.042797711	0.021858055	0.999933887	0.754332448	1.296692756	-0.542360308	0.07848573
ENSG00000111696	*NT5DC3*	4.202468875	0.059073263	0.06638582	-0.007312557	0.999933887	0.567697706	0.792858478	-0.225160772	0.078895267
ENSG00000157600	*TMEM164*	6.61257457	-0.050604252	0.024345122	-0.074949374	0.999933887	-0.70895676	-1.024396739	0.31543998	0.078970192
ENSG00000197362	*ZNF786*	0.62073953	-0.079746469	-0.013722224	-0.066024245	0.999933887	0.084258502	-0.351308222	0.435566724	0.079169705
ENSG00000182685	*BRICD5*	0.868216506	0.090052601	0.357993216	-0.267940615	0.999933887	-0.060914622	-0.541170355	0.480255733	0.079203204
ENSG00000132024	*CC2D1A*	3.74215623	0.029993653	0.012677526	0.017316127	0.999933887	-0.075364735	-0.313406746	0.238042011	0.079324044
ENSG00000140259	*MFAP1*	4.204191911	-0.089559535	-0.108332146	0.018772611	0.999933887	0.197870075	0.431626569	-0.233756494	0.079460411
ENSG00000144455	*SUMF1*	2.915190422	0.048645955	-0.072057604	0.120703559	0.999933887	-0.25617649	-0.505139227	0.248962737	0.079514304
ENSG00000134058	*CDK7*	4.168742207	-0.061521808	-0.098708245	0.037186437	0.999933887	1.102229399	1.371777237	-0.269547838	0.079680012
ENSG00000167106	*FAM102A*	5.941288506	-0.000464001	0.049390922	-0.049854923	0.999933887	0.032887467	-0.105163308	0.138050775	0.079800417
ENSG00000115816	*CEBPZ*	3.437103359	-0.095766593	-0.245708572	0.149941979	0.999933887	-0.112163045	0.271251438	-0.383414484	0.079816791
ENSG00000197956	*S100A6*	8.381663824	0.001174624	-0.064886265	0.066060889	0.999933887	-0.182461194	-0.370647602	0.188186409	0.079816791
ENSG00000204923	*FBXO48*	1.404790874	-0.010391505	0.286705644	-0.297097149	0.999933887	0.746488372	1.120590098	-0.374101727	0.079876883
ENSG00000142634	*EFHD2*	9.996269323	0.069768899	0.031463843	0.038305056	0.999933887	0.367497661	0.57751811	-0.210020449	0.079952386
ENSG00000116497	*S100PBP*	3.52929275	0.016101024	0.000421156	0.015679868	0.999933887	-0.20026535	-0.462163319	0.26189797	0.079956638
ENSG00000119929	*CUTC*	2.806771337	0.015545314	-0.125004961	0.140550274	0.999933887	-0.428359708	-0.727989967	0.299630259	0.079956638
ENSG00000145919	*BOD1*	3.194586688	-0.021794819	-0.057043807	0.035248988	0.999933887	0.521687458	0.791952684	-0.270265226	0.080018524
ENSG00000189050	*RNFT1*	3.012647519	-0.103849497	-0.036577048	-0.067272449	0.999933887	-0.236339486	0.002627496	-0.238966983	0.080060434

ENSG00000198087	*CD2AP*	2.686988846	-0.000932304	-0.117649063	0.116716759	0.999933887	-0.22751151	0.260131587	-0.487643097	0.0801872
ENSG00000061656	*SPAG4*	2.004534627	0.072406894	0.114275313	-0.041868419	0.999933887	0.221997833	0.59905615	-0.377058317	0.080306088
ENSG00000177731	*FLII*	7.498768741	0.005267815	0.025317196	-0.020049382	0.999933887	-0.642334497	-0.821461315	0.179126818	0.080383142
ENSG00000077684	*JADE1*	4.571211898	0.053855725	-0.018476889	0.072332614	0.999933887	-0.288506498	-0.545468496	0.256961998	0.080467073
ENSG00000235106	*BRD3OS*	0.932210158	0.085334473	0.024219058	0.061115415	0.999933887	-0.094634623	-0.526002508	0.431367885	0.080467073
ENSG00000136146	*MED4*	3.953036812	-0.058017245	-0.038861204	-0.019156041	0.999933887	-0.190486492	-0.002985107	-0.187501386	0.080478592
ENSG00000144567	*RETREG2*	7.051682774	0.008661852	-0.023822357	0.032484209	0.999933887	-0.763320863	-0.974511308	0.211190445	0.080515465
ENSG00000119729	*RHOQ*	6.195673601	-0.057281236	-0.003845815	-0.053435421	0.999933887	0.168863224	0.402521662	-0.233658439	0.080529093
ENSG00000103051	*COG4*	3.329783634	0.00708321	-0.068885835	0.075969046	0.999933887	-0.168392979	-0.396572072	0.228179093	0.080529093
ENSG00000092820	*EZR*	7.131316091	-0.056622886	-0.002219225	-0.054403661	0.999933887	0.153936248	0.314446013	-0.160509765	0.081117656
ENSG00000186105	*LRRC70*	3.683260443	-0.035197006	0.059887039	-0.095084045	0.999933887	0.084967859	0.385981583	-0.301013723	0.081152743
ENSG00000187840	*EIF4EBP1*	1.667288106	-0.015865632	0.083895867	-0.099761499	0.999933887	-0.654515459	-1.147169897	0.492654438	0.081288345
ENSG00000100368	*CSF2RB*	9.710640926	0.055330881	0.072076888	-0.016746008	0.999933887	0.48371395	0.298007924	0.185706026	0.08156402
ENSG00000287561	*AL121899.4*	3.272192498	0.26357207	0.309588619	-0.046016549	0.999933887	0.349657261	0.721902816	-0.372245555	0.08166956
ENSG00000109332	*UBE2D3*	9.397169272	0.009847078	-0.011103509	0.020950587	0.999933887	-0.001379167	0.109683862	-0.111063029	0.08166956
ENSG00000143384	*MCL1*	10.51752024	0.108846884	0.150557153	-0.041710269	0.999933887	0.079473914	-0.115695362	0.195169276	0.081825545
ENSG00000141098	*GFOD2*	2.900155792	0.00270307	0.005995311	-0.003292241	0.999933887	-0.753156152	-1.168049753	0.414893601	0.081825545
ENSG00000185361	*TNFAIP8L1*	2.939060593	-0.079655452	-0.065420604	-0.014234848	0.999933887	-0.198959234	-0.508986163	0.310026929	0.081854666
ENSG00000231856	*AL162377.1*	1.789461482	0.058255695	0.127719853	-0.069464157	0.999933887	1.954160106	2.470049666	-0.51588956	0.081870335
ENSG00000157557	*ETS2*	8.946814827	0.214395643	0.213153731	0.001241911	0.999933887	1.67698242	1.990346957	-0.313364537	0.08214628
ENSG00000236200	*KDM4A-AS1*	-0.197930636	0.22451804	-0.081067386	0.305585426	0.999933887	0.082071219	-0.764452852	0.846524071	0.08214628
ENSG00000066117	*SMARCD1*	4.412487987	-0.010667003	-0.052085073	0.04141807	0.999933887	-0.208363912	-0.410127913	0.201764001	0.082181858
ENSG00000186652	*PRG2*	-0.507773972	-0.088927638	0.254983194	-0.343910832	0.999933887	1.282486089	1.880609956	-0.598123867	0.082182978
ENSG00000177889	*UBE2N*	4.960509159	-0.01141612	-0.026341868	0.014925747	0.999933887	0.187744273	0.358970817	-0.171226544	0.082223984
ENSG00000104870	*FCGRT*	7.909274817	0.024688019	0.013796872	0.010891148	0.999933887	-0.312748289	-0.531243604	0.218495315	0.082261214
ENSG00000144674	*GOLGA4*	4.923088039	-0.117445064	-0.123472306	0.006027242	0.999933887	-0.52232597	0.041152561	-0.563478531	0.082277946
ENSG00000123124	*WWP1*	3.569825502	-0.033217861	-0.147278919	0.114061058	0.999933887	-0.15121154	0.084352417	-0.235563957	0.082395481
ENSG00000113303	*BTNL8*	4.548861581	-0.009758908	0.025805147	-0.035564056	0.999933887	0.209237907	0.412921575	-0.203683668	0.082440874
ENSG00000280080	*TBC1D22A-AS*	0.471353466	0.172912929	-0.035900997	0.208813926	0.999933887	0.391274956	0.993539971	-0.602265015	0.082556861
ENSG00000198712	*MT-CO2*	9.16940856	0.029562096	-0.06510153	0.094663626	0.999933887	0.03050244	0.334493705	-0.303991265	0.082754203
ENSG00000160117	*ANKLE1*	-0.026168927	0.036440327	0.067806619	-0.031366292	0.999933887	1.208506802	1.939734284	-0.731227482	0.082754203
ENSG00000134900	*TPP2*	5.572023732	-0.004872675	-0.12901651	0.124143835	0.999933887	-1.02345795	-1.380551728	0.357093778	0.082791095
ENSG00000198218	*QRICH1*	5.816359665	0.001554505	0.027994966	-0.026440461	0.999933887	-0.192056939	-0.351381765	0.159324826	0.082875323
ENSG00000112357	*PEX7*	-0.358042132	-0.163638657	0.397443768	-0.561082425	0.999933887	1.0627258	1.700354596	-0.637628796	0.082907765
ENSG00000102226	*USP11*	4.285975856	-0.037045354	-0.030711686	-0.006333668	0.999933887	0.149261265	0.358081406	-0.208820141	0.082907765
ENSG00000178053	*MLF1*	0.437521251	0.05422811	-0.256521854	0.310749964	0.999933887	-0.008622347	0.566781099	-0.575403446	0.082907765
ENSG00000176595	*KBTBD11*	2.243324219	-0.037603149	-0.004630741	-0.032972407	0.999933887	-0.089532365	-0.396455953	0.306923588	0.082907765
ENSG00000135778	*NTPCR*	1.806208424	-0.02734669	0.168911178	-0.196257868	0.999933887	-0.021889787	-0.345189647	0.32329986	0.082907765
ENSG00000089006	*SNX5*	4.269915971	-0.002942856	-0.044786741	0.041843886	0.999933887	-0.08157075	-0.255715614	0.174144864	0.082907765
ENSG00000064763	*FAR2*	3.506073837	-0.001255331	-0.033530275	0.032274945	0.999933887	-0.267257954	-0.525444973	0.258187019	0.08291344
ENSG00000226942	*IL9RP3*	1.298032608	-0.278987558	-0.184600442	-0.094387115	0.999933887	-1.427244212	-1.850926553	0.423682342	0.083022978
ENSG00000247287	*AL359220.1*	1.094922586	0.029104504	-0.183806454	0.212910958	0.999933887	-0.257263065	0.192675844	-0.449938909	0.083022978
ENSG00000152495	*CAMK4*	4.043641136	0.022203031	-0.075820435	0.098023466	0.999933887	-0.114408153	0.222616486	-0.337024639	0.083057628
ENSG00000140525	*FANCI*	2.030753733	-0.043458828	-0.036236897	-0.007221932	0.999933887	-0.009929975	0.363876519	-0.373806494	0.083221995
ENSG00000203879	*GDI1*	8.219053409	0.010629496	0.029870833	-0.019241337	0.999933887	0.232696597	0.373503877	-0.14080728	0.083221995
ENSG00000101138	*CSTF1*	3.364221654	-0.047493821	-0.018007558	-0.029486263	0.999933887	-0.374038342	-0.633127375	0.259089034	0.083246855
ENSG00000261884	*AC040162.1*	3.140688962	-0.076903795	0.017850477	-0.094754272	0.999933887	-0.529870579	-0.785930816	0.256060237	0.083323161
ENSG00000167207	*NOD2*	5.469518198	0.059432602	-0.051807107	0.111239709	0.999933887	1.122940991	1.488205568	-0.365264578	0.083338462
ENSG00000105723	*GSK3A*	5.843319629	0.025656807	0.070250273	-0.044593466	0.999933887	-0.015700424	0.156631161	-0.172331586	0.083338462
ENSG00000009335	*UBE3C*	4.292297605	-0.043915731	-0.001126936	-0.042788795	0.999933887	-0.100668063	-0.317114129	0.216446065	0.083353739
ENSG00000039650	*PNKP*	5.17091932	0.03330873	0.060419503	-0.027110773	0.999933887	-0.204555824	-0.428825733	0.224269909	0.083444817
ENSG00000132017	*DCAF15*	5.864203973	0.039693071	0.059255422	-0.019562351	0.999933887	-0.302793659	-0.523005471	0.220211812	0.083482224
ENSG00000112249	*ASCC3*	2.894962664	-0.012395598	-0.110511834	0.098116236	0.999933887	-0.100371057	0.261722297	-0.362093354	0.083489954
ENSG00000237886	*NALT1*	1.158609236	-0.065784912	-0.068556144	0.002771232	0.999933887	-0.768516006	-1.195598729	0.427082723	0.083559587
ENSG00000105401	*CDC37*	6.819609154	0.05427355	0.013319353	0.040954196	0.999933887	0.376559703	0.564263571	-0.187703868	0.083636891
ENSG00000127445	*PIN1*	3.950836791	0.11381173	0.074537933	0.039273797	0.999933887	-0.077319747	-0.340091362	0.262771615	0.083648635
ENSG00000037757	*MRI1*	1.895176818	0.082927915	0.134916776	-0.051988862	0.999933887	0.060485656	-0.269531977	0.330017633	0.083648635
ENSG00000197114	*ZGPAT*	3.015301907	0.075508254	0.053815022	0.021693232	0.999933887	-0.048509735	-0.410876605	0.362366871	0.083893348
ENSG00000183734	*ASCL2*	1.832329269	0.085935327	0.1461795	-0.060244173	0.999933887	0.045615353	0.515644965	-0.470029612	0.083893348
ENSG00000130052	*STARD8*	4.148521086	0.112318429	-0.032082129	0.144400558	0.999933887	1.09305787	1.3994452	-0.306387329	0.084033495
ENSG00000065135	*GNAI3*	7.187336532	-0.039390804	-0.047534362	0.008143558	0.999933887	-0.311429103	-0.137020662	-0.174408441	0.084033495
ENSG00000136999	*CCN3*	4.097613987	-0.069675944	-0.064893561	-0.004782383	0.999933887	-1.383094703	-1.708306339	0.325211636	0.084033495
ENSG00000077782	*FGFR1*	2.274791447	0.045288088	0.11391372	-0.068625633	0.999933887	-0.292823583	-0.553281825	0.260458242	0.084039884
ENSG00000168066	*SF1*	8.494133603	0.008912047	0.007744666	0.001167381	0.999933887	-0.179347406	-0.310901592	0.131554186	0.0840681
ENSG00000105245	*NUMBL*	1.736837282	0.135673508	-0.042421207	0.178094714	0.999933887	-0.139479823	-0.49779702	0.358317198	0.084115399
ENSG00000127022	*CANX*	7.366698983	-0.044612927	-0.1185419	0.073928973	0.999933887	0.04050678	0.182780708	-0.142273928	0.084115399
ENSG00000186283	*TOR3A*	2.987429518	0.027149507	0.098076073	-0.070926567	0.999933887	0.783039127	1.078374144	-0.295335017	0.084126529
ENSG00000130714	*POMT1*	3.432068489	-0.031963791	-0.033660285	0.001696494	0.999933887	0.180472598	-0.037547367	0.218019965	0.084292877
ENSG00000142959	*BEST4*	0.155447837	-0.164572686	0.11947917	-0.284051855	0.999933887	0.486964309	0.930265645	-0.443301337	0.084318919
ENSG00000189159	*JPT1*	5.397265201	0.056692193	-0.036291698	0.092983891	0.999933887	-0.04362089	-0.338317526	0.294696637	0.084497382
ENSG00000252391	*RNU6-638P*	0.389448081	-0.033951239	0.054137164	-0.088088403	0.999933887	0.639894168	1.096086051	-0.456191883	0.084497382
ENSG00000287763	*AC068279.2*	-1.755103097	0.236873647	0.36027218	-0.123398533	0.999933887	0.092675251	-0.535017711	0.627692963	0.084635225
ENSG00000077238	*IL4R*	8.288266041	0.015960666	0.010128904	0.005831762	0.999933887	0.54726258	0.737906114	-0.190643534	0.08477547
ENSG00000091483	*FH*	1.499630153	-0.044373129	-0.014140578	-0.030232552	0.999933887	0.395189952	0.762293701	-0.367103749	0.084783483
ENSG00000101901	*ALG13*	3.617102817	0.007906474	-0.008180272	0.016086747	0.999933887	-0.022696678	0.24591389	-0.268610568	0.084894001
ENSG00000065559	*MAP2K4*	5.123879678	-0.068807945	-0.036008148	-0.032799798	0.999933887	-0.87979774	-1.118506319	0.238708578	0.085233296
ENSG00000171700	*RGS19*	7.027455144	0.07873626	0.070417591	0.008318669	0.999933887	-0.917106208	-1.21384323	0.296737022	0.085233296
ENSG00000163251	*FZD5*	1.71807898	-0.096780579	-0.075956592	-0.020823987	0.999933887	0.841261465	1.178185707	-0.336924242	0.085233296
ENSG00000165175	*MID1IP1*	7.636377249	0.192086709	0.200498357	-0.008411648	0.999933887	0.342724381	0.599531066	-0.256806685	0.085313828
ENSG00000131759	*RARA*	7.83384247	0.06646499	0.044774969	0.021690021	0.999933887	0.363792601	0.563663251	-0.19987065	0.085313828
ENSG00000189350	*TOGARAM2*	2.242874555	0.239588827	0.268730512	-0.029141685	0.999933887	-0.455264305	-0.819621617	0.364357312	0.085459525
ENSG00000068697	*LAPTM4A*	6.517842602	0.033750133	-0.003613446	0.03736358	0.999933887	-0.332042909	-0.54355991	0.211517001	0.085459525
ENSG00000082805	*ERC1*	3.291203974	0.000524979	0.098625769	-0.09810079	0.999933887	0.015613372	0.250970047	-0.235356674	0.085459525
ENSG00000188396	*TCTEX1D4*	3.561811665	0.30507307	0.328285347	-0.023212277	0.999933887	1.644528092	2.074508364	-0.429980273	0.08557155
ENSG00000125740	*FOSB*	9.268129828	0.295840982	0.154454419	0.141386563	0.999933887	0.750744756	1.144415125	-0.39367037	0.08557155
ENSG00000186660	*ZFP91*	5.43012791	-0.053392183	-0.041623029	-0.011769155	0.999933887	-0.154369089	-0.320278224	0.165909135	0.08557155
ENSG00000101654	*RNMT*	5.814616322	-0.077067281	-0.13317749	0.056110209	0.999933887	0.056170775	0.326709491	-0.270538717	0.08557155
ENSG00000138629	*UBL7*	4.01029996	0.053560632	0.064819846	-0.011259215	0.999933887	-0.688130276	-0.973765733	0.285635457	0.08557155
ENSG00000060558	*GNA15*	4.451363642	0.029700536	0.047430033	-0.017729496	0.999933887	0.921132569	1.168029504	-0.246896935	0.08557155
ENSG00000230943	*LINC02541*	0.626471896	0.045234287	0.212500784	-0.167266497	0.999933887	3.207116675	3.87581984	-0.668703164	0.08557155
ENSG00000167977	*KCTD5*	4.332639431	-0.044129092	0.019334159	-0.063463251	0.999933887	-0.638216064	-0.852204164	0.2139881	0.085852636
ENSG00000135047	*CTSL*	4.212821983	-0.044001743	-0.176011734	0.132009991	0.999933887	0.439264876	0.692594878	-0.253330002	0.085852636
ENSG00000071051	*NCK2*	4.971065671	0.095027908	0.087577957	0.00744995	0.999933887	0.313370711	0.502362915	-0.188992204	0.085855344
ENSG00000120860	*WASHC3*	2.228454668	-0.068431388	-0.085763673	0.017332285	0.999933887	-0.005682976	0.301560028	-0.307243004	0.085855344
ENSG00000131480	*AOC2*	3.666327952	0.155779891	-0.022740414	0.178520304	0.999933887	1.195670213	0.708357118	0.487313095	0.085940877
ENSG00000203326	*ZNF525*	0.535340903	-0.36049767	-0.140027219	-0.220470451	0.999933887	-0.152669637	0.420344532	-0.573014169	0.086094428
ENSG00000159322	*ADPGK*	7.363904595	-0.035886774	0.015655736	-0.051542509	0.999933887	0.345737428	0.509539054	-0.163801626	0.086096989
ENSG00000166546	*BEAN1*	-0.111386644	-0.108198872	-0.005248847	-0.102950026	0.999933887	-0.545399766	-1.271531705	0.726131939	0.086290805
ENSG00000173786	*CNP*	3.465769853	0.033453197	-0.007807179	0.041260376	0.999933887	-0.301166245	-0.563316867	0.262150622	0.08630493
ENSG00000110931	*CAMKK2*	9.036289411	-0.009693254	-0.006050991	-0.003642262	0.999933887	0.490227931	0.651638725	-0.161410794	0.08630493
ENSG00000277895	*AC135279.3*	-0.448223445	0.127511309	0.122679781	0.004831528	0.999933887	2.643360535	3.532218941	-0.888858406	0.086325192
ENSG00000181800	*CELF2-AS1*	3.613900728	0.062499023	-0.04500544	0.107504463	0.999933887	-0.885972036	-1.475947266	0.589975231	0.086588726
ENSG00000254685	*FPGT*	0.75724841	-0.130674769	-0.217610615	0.086935846	0.999933887	-0.488809894	-1.049570767	0.560760873	0.086691886
ENSG00000228278	*ORM2*	2.99308938	-0.438702892	0.233496986	-0.672199878	0.999933887	2.031898618	2.542636247	-0.510737629	0.086928715
ENSG00000177054	*ZDHHC13*	3.314836524	-0.07492564	0.081571246	-0.156496886	0.999933887	-0.308967283	-0.074053329	-0.234913954	0.086937213

ENSG00000100504	*PYGL*	6.318384433	-0.031690825	-0.02669161	-0.004999215	0.999933887	-0.151296991	-0.339263069	0.187966078	0.086951674
ENSG00000165804	*ZNF219*	2.957213228	0.152697593	0.184427605	-0.031730012	0.999933887	-0.640155872	-0.975628714	0.335472842	0.087048498
ENSG00000183291	*SELENOF*	5.979156874	0.023402258	-0.053207708	0.076609966	0.999933887	-0.047122014	0.137499997	-0.184622011	0.087048498
ENSG00000070950	*RAD18*	1.969332891	-0.069279147	-0.190498354	0.121219207	0.999933887	0.217481389	0.650061604	-0.432580216	0.087199454
ENSG00000163563	*MNDA*	8.468525323	-0.101381442	-0.221746493	0.120365051	0.999933887	-0.876606644	-1.32301839	0.446411747	0.087283987
ENSG00000081320	*STK17B*	8.238268932	0.054049476	-0.005581058	0.059630533	0.999933887	-0.453984461	-0.650332284	0.196347824	0.087317925
ENSG00000111530	*CAND1*	4.267539911	-0.065740702	-0.043911165	-0.021829537	0.999933887	-0.049041378	0.178660593	-0.227701971	0.087341644
ENSG00000268119	*AC010615.2*	1.682647559	0.008241365	0.081528896	-0.073287531	0.999933887	-0.259694216	0.149392409	-0.409086625	0.087405274
ENSG00000154001	*PPP2R5E*	3.93301641	0.023185229	-0.058055289	0.081240518	0.999933887	-0.266133615	-0.47379398	0.207660365	0.08746701
ENSG00000272668	*AL590560.3*	1.325103375	0.008228972	-0.00141398	0.009642953	0.999933887	0.009792	-0.425868412	0.435660412	0.08746701
ENSG00000182858	*ALG12*	2.358087855	0.065166911	-0.047474628	0.112641539	0.999933887	-0.191877237	-0.497355262	0.305478026	0.087519354
ENSG00000139618	*BRCA2*	1.449268476	-0.083068891	-0.265767105	0.182698214	0.999933887	0.08196565	0.848282725	-0.766317075	0.087519354
ENSG00000185022	*MAFF*	9.144944873	0.328448665	0.271996597	0.056452068	0.999933887	1.88116178	2.228695099	-0.347533319	0.087603193
ENSG00000138744	*NAAA*	3.255639582	-0.024157249	-0.01092079	-0.013236459	0.999933887	-0.197655301	-0.472000222	0.274344921	0.087603193
ENSG00000133112	*TPT1*	9.553028321	0.002512479	0.019844908	-0.017332429	0.999933887	0.188972715	0.371375457	-0.182402742	0.087603193
ENSG00000063169	*BICRA*	4.646743574	0.056282975	0.044000192	0.012282783	0.999933887	-0.413190663	-0.671721568	0.258530905	0.087847671
ENSG00000122873	*CISD1*	0.233628907	0.017157827	0.064739177	-0.04758135	0.999933887	-0.124218873	0.521058823	-0.645277697	0.088022826
ENSG00000143420	*ENSA*	6.762572882	-0.005601015	-0.009522418	0.003921404	0.999933887	0.252232321	0.423209241	-0.17097692	0.088022826
ENSG00000238241	*CCR12P*	-0.240717369	0.222866372	-0.146653267	0.369519639	0.999933887	0.789078288	1.453431284	-0.664352996	0.088122923
ENSG00000230982	*DSTNP1*	0.074032771	-0.064314488	-0.179471161	0.115156674	0.999933887	0.750577112	1.364783219	-0.614206107	0.08819215
ENSG00000179218	*CALR*	6.207791914	0.004102573	-0.046264975	0.050367548	0.999933887	-0.016193251	-0.183517189	0.167323938	0.08819215
ENSG00000123130	*ACOT9*	6.536666868	-0.062354019	-0.018819011	-0.043535008	0.999933887	-0.000695858	0.169927371	-0.170623229	0.088237579
ENSG00000088298	*EDEM2*	4.845591586	-0.02311061	-0.030554474	0.007443864	0.999933887	0.037544034	-0.156118478	0.193662512	0.088321368
ENSG00000108389	*MTMR4*	5.422287547	-0.042267387	-0.054745123	0.012477735	0.999933887	1.011105753	1.280183046	-0.269077293	0.088534992
ENSG00000197961	*ZNF121*	3.070451416	-0.066260355	-0.031224185	-0.03503617	0.999933887	-0.138748731	0.131233911	-0.269982642	0.088539038
ENSG00000135828	*RNASEL*	4.5408833	-0.037752291	-0.110557579	0.072805288	0.999933887	-0.408446071	-0.603183824	0.194737753	0.088701205
ENSG00000140943	*MBTPS1*	5.050620205	-0.01037912	-0.030222983	0.019843863	0.999933887	-0.011178352	-0.174288595	0.163110243	0.088978636
ENSG00000237753	*AC079922.2*	1.79009424	-0.161920255	0.182895939	-0.344816194	0.999933887	-0.409228467	-0.771835712	0.362607245	0.088990362
ENSG00000196453	*ZNF777*	2.797200448	0.063508239	0.00481799	0.058690249	0.999933887	-0.196972077	-0.455910377	0.2589383	0.08906901
ENSG00000279164	*AL118508.2*	0.551915841	0.108551741	0.108291826	0.000259915	0.999933887	-0.315987554	0.484317213	-0.800304767	0.089170294
ENSG00000263731	*AC145207.5*	-0.188896838	0.11989764	0.117930163	0.001967477	0.999933887	-0.110450036	0.696064938	-0.806514974	0.089270559
ENSG00000053524	*MCF2L2*	2.946356251	0.053334642	0.131575344	-0.078240702	0.999933887	0.670413675	0.980372833	-0.309959159	0.089762208
ENSG00000105711	*SCN1B*	3.623993305	0.058506056	-0.004946064	0.063452121	0.999933887	3.492270411	4.04487305	-0.552602639	0.089762208
ENSG00000230149	*AL021707.3*	1.529365595	0.031526486	-0.027096315	0.058622801	0.999933887	0.195573393	-0.23500353	0.430576923	0.089762208
ENSG00000234389	*AC007278.1*	2.914828906	-0.030341417	-0.066142068	0.035800651	0.999933887	0.049413305	0.547953046	-0.498539741	0.089762208
ENSG00000096401	*CDC5L*	4.917919814	-0.010678049	-0.090058944	0.079380895	0.999933887	-0.100151041	0.120908751	-0.221059792	0.089762208
ENSG00000112137	*PHACTR1*	10.351358763	0.088815031	0.069130819	0.019684211	0.999933887	0.699661313	0.924008172	-0.224346858	0.08991288
ENSG00000170322	*NFRKB*	4.857745056	-0.025415965	-0.045559528	0.020143563	0.999933887	-0.226148803	-0.399845266	0.173696463	0.089937542
ENSG00000253738	*OTUD6B-AS1*	2.473322769	-0.001126183	-0.24097798	0.239851797	0.999933887	-0.351062026	0.069784877	-0.420846903	0.089951539
ENSG00000009307	*CSDE1*	8.674625145	-0.032351173	-0.119305898	0.086954725	0.999933887	-0.087297807	0.047423302	-0.134721109	0.089991863
ENSG00000163840	*DTX3L*	5.966129666	-0.066292395	-0.033269648	-0.033022748	0.999933887	-0.364394459	-0.55547802	0.19108356	0.090019787
ENSG00000124164	*VAPB*	4.031419193	-0.055228392	-0.046301275	-0.008927116	0.999933887	-0.628809838	-0.855291391	0.226481553	0.090039665
ENSG00000120370	*GORAB*	0.778272495	0.005452714	-0.290465393	0.295918107	0.999933887	0.023807292	0.53974989	-0.515942597	0.090243096
ENSG00000155508	*CNOT8*	5.493679624	-0.094770652	0.009110961	-0.103881613	0.999933887	-0.323441318	-0.487381218	0.1639399	0.09033252
ENSG00000068001	*HYAL2*	1.933998517	0.109418553	-0.09510989	0.204528443	0.999933887	-0.674773836	-1.066158794	0.391384958	0.09033252
ENSG00000133246	*PRAM1*	6.155998462	-0.045200235	-0.06062861	0.015428374	0.999933887	-1.475403663	-1.725075466	0.249671803	0.09033252
ENSG00000132424	*PNISR*	7.413861153	-0.06309474	-0.224391852	0.161297112	0.999933887	-0.378608442	0.098712495	-0.477320937	0.090358251
ENSG00000163512	*AZI2*	2.531876806	-0.027385576	-0.043874154	0.016488578	0.999933887	-0.2892801	0.011034041	-0.30031414	0.090358251
ENSG00000185220	*PGBD2*	0.832764551	-0.030878969	-0.045957174	0.015078205	0.999933887	-0.167849525	-0.545791671	0.377942146	0.090358251
ENSG00000196268	*ZNF493*	4.852968065	-0.015436322	-0.039440921	0.024004598	0.999933887	-0.258633958	0.126687454	-0.385321412	0.090358251
ENSG00000178188	*SH2B1*	4.559238735	0.010280034	0.069766109	-0.059486075	0.999933887	-0.166242052	-0.377684311	0.211442259	0.090571907
ENSG00000167536	*DHRS13*	2.622486563	0.141763218	0.019965794	0.121797424	0.999933887	1.457779025	2.110556467	-0.652777441	0.090588411
ENSG00000006530	*AGK*	2.665845581	0.049124657	0.042255627	0.00686903	0.999933887	0.240369937	0.480220536	-0.239850599	0.090609722
ENSG00000271795	*AC011337.1*	-1.179465613	-0.035244288	-0.497647692	0.462403404	0.999933887	-0.847470378	-0.195824411	-0.651645968	0.090627104
ENSG00000089127	*OAS1*	3.70051178	-0.043613849	0.041076881	-0.08469073	0.999933887	-0.504848416	-0.834344847	0.329496431	0.091073872
ENSG00000207736	*MIR657*	0.579412334	0.069200125	0.070572683	-0.001372558	0.999933887	0.182042666	0.664737387	-0.48269472	0.09110639
ENSG00000181481	*RNF135*	2.279561254	-0.019171589	0.047783648	-0.066955237	0.999933887	-0.523837164	-0.951405968	0.427568804	0.091140191
ENSG00000181634	*TNFSF15*	7.665598257	-0.071498384	0.017729375	-0.089227759	0.999933887	-0.112559446	0.172191334	-0.28475078	0.091469857
ENSG00000174243	*DDX23*	5.083236712	-0.026895481	-0.060768342	0.033872861	0.999933887	-0.083473571	-0.227233091	0.143759521	0.091469857
ENSG00000155313	*USP25*	6.062414917	-0.008866381	-0.099959985	0.091093604	0.999933887	-0.252934055	0.054827576	-0.307761631	0.09149167
ENSG00000092199	*HNRNPC*	9.154834639	-0.053195403	-0.057639236	0.004443833	0.999933887	1.383206203	1.635651983	-0.25244578	0.09159359
ENSG00000141219	*C17orf80*	0.782073795	-0.110170056	-0.110674316	0.000504259	0.999933887	-0.196759619	-0.587162725	0.390403106	0.09159359
ENSG00000134884	*ARGLU1*	7.404164894	-0.023082305	0.037694179	-0.060776483	0.999933887	-0.173774629	-0.329056799	0.15528217	0.09159359
ENSG00000221821	*C6orf226*	1.270861471	0.025432119	-0.02425519	0.049687309	0.999933887	0.000512318	-0.448982681	0.449494999	0.09159359
ENSG00000237172	*B3GNT9*	1.299667997	-0.008292321	0.065132017	-0.073424338	0.999933887	-0.503006764	-0.924238256	0.421231492	0.09159359
ENSG00000100767	*PAPLN*	1.649276662	0.008559188	0.087111729	-0.078552541	0.999933887	0.472387404	0.886878036	-0.414490632	0.091947522
ENSG00000006459	*KDM7A*	6.147640473	0.042077682	-0.057446177	0.09952386	0.999933887	-1.272448623	-1.597863491	0.325414868	0.091948898
ENSG00000213923	*CSNK1E*	4.102602611	0.01995183	0.099942035	-0.079990205	0.999933887	0.232412304	0.442322411	-0.209910107	0.092267053
ENSG00000125818	*PSMF1*	5.31567053	0.016349185	0.017242055	-0.00089287	0.999933887	-0.203150527	-0.407303285	0.204152759	0.092329192
ENSG00000188549	*CCDC9B*	2.805171277	0.134099817	0.23459394	-0.100494123	0.999933887	0.839651488	1.106801604	-0.267150116	0.092348208
ENSG00000270120	*AC007728.3*	0.455027542	0.089746593	-0.038004546	0.127751139	0.999933887	1.061841014	1.730919304	-0.66907829	0.092396489
ENSG00000111676	*ATN1*	3.575550111	-0.013532575	0.13121085	-0.144743425	0.999933887	0.098098696	0.305292638	-0.207193943	0.092396489
ENSG00000176903	*PNMA1*	3.653377072	0.089150429	-0.009384193	0.098534622	0.999933887	-0.090704038	-0.272378606	0.181674568	0.092440851
ENSG00000135070	*ISCA1*	4.593878285	0.052463932	0.032917989	0.019545943	0.999933887	-0.043049705	0.173075794	-0.216125499	0.092440851
ENSG00000083896	*YTHDC1*	7.933972162	-0.056279957	-0.096408769	0.040128812	0.999933887	0.048786995	0.266658109	-0.217871114	0.092440851
ENSG00000286691	*AL590009.1*	-0.74970361	-0.118771123	-0.130283626	0.011512503	0.999933887	-1.25239567	-2.088908805	0.836513134	0.092440851
ENSG00000229931	*ATXN1-AS1*	2.410712214	0.04624186	-0.151758654	0.198000514	0.999933887	0.28304282	0.710103622	-0.427060802	0.092440851
ENSG00000228794	*LINC01128*	3.988855069	-0.01512257	-0.047115903	0.031993333	0.999933887	-0.010896801	0.227857096	-0.238753897	0.092440851
ENSG00000197429	*IPP*	1.412838649	0.015213695	-0.167847147	0.183060842	0.999933887	0.1012437	0.475464844	-0.374221144	0.092440851
ENSG00000088247	*KHSRP*	5.689901986	0.05151325	0.046244594	0.005268655	0.999933887	-0.190920717	-0.37207408	0.181153363	0.092462752
ENSG00000156171	*DRAM2*	3.00712037	-0.081056093	-0.093415373	0.01235928	0.999933887	-0.496549499	-0.790489774	0.293940275	0.092462752
ENSG00000173480	*ZNF417*	3.482883203	-0.023403885	-0.108426144	0.085022259	0.999933887	-0.032422157	0.179563175	-0.211985332	0.092462752
ENSG00000132646	*PCNA*	3.332702106	0.084777543	-0.039322014	0.124099556	0.999933887	-0.493175906	-0.786166316	0.292990409	0.092589075
ENSG00000259342	*AC025580.1*	1.999852423	0.334767457	0.530590798	-0.195823342	0.999933887	2.557732959	3.114255154	-0.556522196	0.092661109
ENSG00000179295	*PTPN11*	4.965621254	-0.052919131	-0.00411878	-0.048800351	0.999933887	-0.087523832	0.122191379	-0.209715211	0.092855332
ENSG00000138443	*ABI2*	1.871263499	-0.052663516	-0.11120747	0.058543954	0.999933887	-0.21604967	0.094451494	-0.310501164	0.092951852
ENSG00000011454	*RABGAP1*	5.075294561	-0.037679966	-0.061091761	0.023411795	0.999933887	-0.06404191	0.127146124	-0.191188034	0.09301689
ENSG00000005436	*GCFC2*	0.568024012	-0.230892284	-0.116575491	-0.114316793	0.999933887	-0.118744779	0.442258948	-0.561003727	0.093231597
ENSG00000165629	*ATP5F1C*	3.638367857	-0.070056832	-0.098372887	0.028316055	0.999933887	-0.259681844	-0.473583713	0.213901868	0.093231597
ENSG00000128512	*DOCK4*	7.502603755	-0.026394478	-0.018279606	-0.008114872	0.999933887	0.259394685	0.454950993	-0.195556308	0.093312078
ENSG00000026652	*AGPAT4*	3.977001153	-0.051907217	0.024695373	-0.07660259	0.999933887	0.196220984	0.411096956	-0.214875972	0.093416232
ENSG00000104897	*SF3A2*	4.719644423	0.023148368	0.017793175	0.005355193	0.999933887	-0.020778193	-0.215170231	0.194392038	0.093666764
ENSG00000117868	*ESYT2*	6.427061375	0.003993112	0.033003564	-0.029010451	0.999933887	0.287515837	0.445975091	-0.158459254	0.093666764
ENSG00000087111	*PIGS*	4.087104545	0.002410183	0.041707684	-0.039297501	0.999933887	-0.043865478	-0.248450464	0.204584986	0.093672335
ENSG00000197223	*C1D*	3.62669313	-0.076070678	-0.077381929	0.001311251	0.999933887	-0.067901598	0.140403938	-0.208305535	0.093685139
ENSG00000272402	*AL031775.2*	0.305558403	0.130815322	0.038078722	0.0927366	0.999933887	0.355983565	0.810446437	-0.454462871	0.093685139
ENSG00000072042	*RDH11*	2.640970535	-0.084899255	-0.047647739	-0.037251515	0.999933887	-0.017299231	0.251172713	-0.268471944	0.093685139
ENSG00000127511	*SIN3B*	4.927775759	0.040747143	0.039223793	0.00152335	0.999933887	0.032548149	-0.151707252	0.184255401	0.093685139
ENSG00000113532	*ST8SIA4*	5.876836957	-0.045251672	-0.034280742	-0.01097093	0.999933887	-0.819411208	-1.112860694	0.293449486	0.093685139
ENSG00000164654	*MIOS*	3.293479152	0.050348725	0.004586948	0.045761777	0.999933887	0.165481171	0.372575243	-0.207094072	0.093733497
ENSG00000177425	*PAWR*	-0.604456649	0.022364334	-0.055284772	0.077649106	0.999933887	1.106508722	1.906763419	-0.800254697	0.093806051
ENSG00000111271	*ACAD10*	2.920032175	0.068794197	0.008128722	0.060665475	0.999933887	-0.187167485	-0.472109238	0.284941753	0.093809819
ENSG00000086232	*EIF2AK1*	4.250688544	-0.031148309	-0.105536624	0.074388316	0.999933887	-0.1310363	-0.337250181	0.206213881	0.093984336
ENSG00000127838	*PNKD*	3.984407768	0.058987163	-0.131979723	0.190966887	0.999933887	0.48314524	0.89342585	-0.41028061	0.093984336
ENSG00000156273	*BACH1*	9.437785403	-0.166775077	-0.177109372	0.010334296	0.999933887	-0.342811743	-0.064260641	-0.278551102	0.094107056

ENSG00000236778	*INTS6-AS1*	0.290461703	-0.252003603	0.123774574	-0.375778177	0.999933887	-0.431153134	0.019023393	-0.450176527	0.094450608
ENSG00000197969	*VPS13A*	4.026424359	-0.128316881	-0.219097447	0.090780566	0.999933887	-0.272381536	0.398667213	-0.671048749	0.094492521
ENSG00000122884	*P4HA1*	3.643410485	-0.051649268	-0.100905237	0.049255968	0.999933887	0.625710551	0.907672192	-0.281961641	0.094492521
ENSG00000164949	*GEM*	0.665028597	-0.006432961	0.266874822	-0.273307784	0.999933887	0.767816211	1.140260521	-0.37244431	0.09458739
ENSG00000134516	*DOCK2*	7.865379873	-0.019831525	-0.053524027	0.033692502	0.999933887	-0.053244419	-0.178727057	0.125482638	0.094651574
ENSG00000108846	*ABCC3*	0.101634695	0.034093354	0.304372881	-0.270279527	0.999933887	0.8597978	1.587614478	-0.727816678	0.09474837
ENSG00000110422	*HIPK3*	6.732459062	-0.031152672	-0.071810566	0.040657894	0.999933887	-0.134842092	0.152190865	-0.287032957	0.094942326
ENSG00000166024	*R3HCC1L*	4.176826355	-0.124675646	-0.093360278	-0.031315369	0.999933887	-0.162627525	0.069619262	-0.232246788	0.095002543
ENSG00000173545	*ZNF622*	4.840045161	-0.029491642	-0.094735407	0.065243765	0.999933887	-0.008285229	0.166781285	-0.175066514	0.095075505
ENSG00000126821	*SGPP1*	3.211976783	-0.107408507	0.072541563	-0.17995007	0.999933887	-1.154872742	-1.545313245	0.390440503	0.095076984
ENSG00000138286	*FAM149B1*	2.240286873	-0.01629435	0.02794232	-0.04423667	0.999933887	0.025293765	0.314209741	-0.288915977	0.095086117
ENSG00000170017	*ALCAM*	3.903786421	-0.079526946	-0.061828073	-0.017698873	0.999933887	0.072403744	0.467708137	-0.395304392	0.095373679
ENSG00000084092	*NOA1*	2.45579761	0.053602794	0.207500528	-0.153897734	0.999933887	-0.011780098	-0.294998607	0.283218509	0.095419669
ENSG00000169951	*ZNF764*	2.280349269	0.051769044	0.162133436	-0.110364392	0.999933887	-0.227652421	-0.537574093	0.309921672	0.095419669
ENSG00000114374	*USP9Y*	-2.967694079	0.000108542	0.057759265	-0.057650723	0.999933887	-0.459474312	0.147072155	-0.606546467	0.095419669
ENSG00000198899	*MT-ATP6*	8.353753722	0.047786475	-0.044887061	0.092673536	0.999933887	-0.054255463	0.250990568	-0.305246032	0.09556972
ENSG00000156256	*USP16*	5.116454494	-0.120639711	-0.202623358	0.081983647	0.999933887	-0.168825876	0.256233814	-0.42505969	0.095605699
ENSG00000111231	*GPN3*	1.10578999	0.024922914	0.087024933	-0.062102019	0.999933887	-0.230541238	-0.616507199	0.385965961	0.095802876
ENSG00000136295	*TTYH3*	5.98846648	0.008029791	-0.020129042	0.028158833	0.999933887	-0.292648339	-0.076073929	-0.216574409	0.095802876
ENSG00000136051	*WASHC4*	6.718639487	-0.038908703	-0.163854431	0.124945728	0.999933887	-0.563163707	-0.08841809	-0.474745617	0.095860651
ENSG00000168159	*RNF187*	4.054361953	0.021920548	0.139216963	-0.117296415	0.999933887	0.037364025	-0.189931686	0.227295711	0.095903008
ENSG00000285999	*AC025442.2*	1.448000068	-0.195962555	0.101114154	-0.297076709	0.999933887	0.730530561	1.078503457	-0.347972896	0.095911327
ENSG00000108515	*ENO3*	3.416585685	0.241749631	0.151084759	0.090664872	0.999933887	0.797960125	1.159582514	-0.361622389	0.095970813
ENSG00000164329	*TENT2*	5.533276934	-0.010673067	-0.047728865	0.037055799	0.999933887	-0.131367093	0.116522742	-0.247889835	0.095970813
ENSG00000263072	*ZNF213-AS1*	1.450124884	0.015086916	-0.073051813	0.088138729	0.999933887	-0.096903566	-0.474161774	0.377258207	0.095970813
ENSG00000087303	*NID2*	-0.753821211	-0.024386926	0.302717604	-0.327104529	0.999933887	1.0680676	1.998055583	-0.929987984	0.095970813
ENSG00000169439	*SDC2*	2.66486533	0.012676756	-0.031800789	0.044477545	0.999933887	-1.169928433	-1.632591102	0.462662669	0.095970813
ENSG00000138658	*ZGRF1*	1.042846616	-0.089534897	-0.04258977	-0.046945127	0.999933887	-0.225332778	0.376209756	-0.601542535	0.096050224
ENSG00000065675	*PRKCQ*	4.133615597	-0.036795722	0.050107601	-0.086903323	0.999933887	0.163616813	0.374521393	-0.21090458	0.096050224
ENSG00000103343	*ZNF174*	1.002791562	0.062502816	-0.045576481	0.108079296	0.999933887	-0.310759635	-0.756339555	0.445579919	0.096050224
ENSG00000229390	*MICD*	0.501214167	-0.031986317	-0.135578904	0.103592587	0.999933887	-0.672317634	-1.138090257	0.465772623	0.096050224
ENSG00000260007	*AC107871.1*	2.910131436	-0.000561538	-0.104534053	0.103972515	0.999933887	0.064679161	0.461433882	-0.396754721	0.096050224
ENSG00000140740	*UQCRC2*	7.304196335	-0.03395154	-0.055719898	0.021768359	0.999933887	-0.182144329	-0.018217525	-0.163926804	0.096361706
ENSG00000116030	*SUMO1*	5.598185831	-0.020055406	-0.086726488	0.066671082	0.999933887	-0.096264609	0.07340171	-0.169666319	0.096361706
ENSG00000204482	*LST1*	7.957469376	0.025562592	0.020968904	0.004593688	0.999933887	0.090348289	-0.129850618	0.220198907	0.096593298
ENSG00000172661	*WASHC2C*	7.209985843	-0.065168454	-0.006300457	-0.058867996	0.999933887	0.251431814	0.397505973	-0.14607416	0.096620053
ENSG00000116791	*CRYZ*	0.477653871	-0.051588077	-0.138670636	0.087082559	0.999933887	-0.094494497	0.399766286	-0.494260783	0.096620053
ENSG00000132357	*CARD6*	2.606612013	-0.115064211	0.0007363	-0.115800511	0.999933887	-0.674220129	-0.965477297	0.291257168	0.09684281
ENSG00000256235	*SMIM3*	3.031289098	-0.027609618	0.062421485	-0.090031103	0.999933887	1.351340888	1.711743096	-0.360402208	0.09684281
ENSG00000272341	*AL137003.1*	2.564537445	-0.2610983	-0.375408659	0.11431036	0.999933887	0.157550369	0.710773065	-0.553222696	0.096854897
ENSG00000233058	*LINC00884*	1.739806234	0.153859547	0.084933913	0.068925634	0.999933887	1.351877746	1.714682054	-0.362804309	0.096962178
ENSG00000183475	*ASB7*	3.117235902	-0.08384783	-0.034954342	-0.048893488	0.999933887	-0.472477395	-0.718692106	0.246214711	0.096962178
ENSG00000228140	*AL031283.1*	1.862085304	-0.042890492	-0.015157932	-0.027732561	0.999933887	1.543134004	1.962036022	-0.418902017	0.096990606
ENSG00000146094	*DOK3*	9.302720956	0.004112653	-0.003551277	0.00766393	0.999933887	-0.321653571	-0.150806702	-0.170846869	0.097048151
ENSG00000101665	*SMAD7*	5.02683054	0.053878065	0.077868489	-0.023990424	0.999933887	0.457084695	0.716280851	-0.259196156	0.0971222
ENSG00000158805	*ZNF276*	5.843887401	0.058369395	0.077920076	-0.019550681	0.999933887	-0.114452998	-0.287907884	0.173454886	0.097165628
ENSG00000108239	*TBC1D12*	-0.85972803	-0.291742491	0.082878915	-0.374621406	0.999933887	0.721358331	1.475339272	-0.753980941	0.097172089
ENSG00000196967	*ZNF585A*	1.082473898	-0.190367089	-0.175925621	-0.014441468	0.999933887	-0.325077553	0.063586716	-0.388664269	0.097425944
ENSG00000188994	*ZNF292*	7.41644241	-0.109838947	-0.131164372	0.021325426	0.999933887	-0.427166042	0.055607728	-0.48277377	0.097506193
ENSG00000152465	*NMT2*	2.412663356	-0.032322691	0.08123548	-0.113558172	0.999933887	0.045016072	0.31007812	-0.265062048	0.097506193
ENSG00000280434	*AL031595.3*	-0.802601088	-0.059346197	0.483179019	-0.542525216	0.999933887	0.304844226	1.034323133	-0.729478907	0.097614188
ENSG00000270049	*AC009061.2*	0.999450363	-0.0656029	-0.212306766	0.146703866	0.999933887	-0.272301174	-0.791326116	0.519024942	0.097618946
ENSG00000161179	*YDJC*	3.305755489	0.017163657	0.071775859	-0.054612202	0.999933887	-0.246719507	-0.506227811	0.259508304	0.097809228
ENSG00000163558	*PRKCI*	2.426375083	-0.008097508	-0.016684227	0.008586719	0.999933887	-0.092731731	0.20639284	-0.299124571	0.09781528
ENSG00000255874	*PRECSIT*	0.66845516	0.455774392	0.117452972	0.33832142	0.999933887	2.546752047	3.20354061	-0.656788563	0.097980503
ENSG00000150779	*TIMM8B*	2.060495484	-0.112285971	0.007011122	-0.119297093	0.999933887	-0.06325891	-0.429977322	0.366718412	0.09798863
ENSG00000186020	*ZNF529*	2.267035064	-0.087656065	-0.07542715	-0.012228915	0.999933887	-0.188643049	0.16479766	-0.353440708	0.09798863
ENSG00000127980	*PEX1*	2.301354782	0.048464701	-0.117663214	0.166127914	0.999933887	-0.052252211	-0.380403704	0.328151493	0.09811011
ENSG00000166508	*MCM7*	5.23007153	-0.031726298	0.032618002	-0.0643443	0.999933887	0.076642128	-0.095132806	0.171774933	0.098118059
ENSG00000055483	*USP36*	7.017357851	-0.029954151	0.002942386	-0.032896537	0.999933887	0.043636648	0.234912582	-0.191275934	0.098118059
ENSG00000171100	*MTM1*	3.07575815	-0.024250767	-0.02620929	0.001958523	0.999933887	-0.587445786	-0.870437444	0.282991658	0.098118059
ENSG00000106105	*GARS1*	5.959167649	0.019690584	-0.034713011	0.054403595	0.999933887	0.347782102	0.568572541	-0.220790439	0.098118059
ENSG00000157617	*C2CD2*	0.609823098	-0.030098307	-0.031590114	0.001491807	0.999933887	0.047940963	0.547756105	-0.499815141	0.098266235
ENSG00000266320	*MIR3909*	2.10736128	-0.080034188	-0.004263736	-0.075770453	0.999933887	0.593860923	0.950182748	-0.356321825	0.098284619
ENSG00000161202	*DVL3*	5.83102012	0.015889805	0.033861923	-0.017972118	0.999933887	-0.139176202	-0.288008764	0.148832563	0.098416817
ENSG00000054219	*LY75*	2.640404814	-0.033083203	-0.091406302	0.058323099	0.999933887	-0.4358761	-0.864938627	0.429062526	0.098548689
ENSG00000198848	*CES1*	-0.573756927	0.018908957	0.313443773	-0.294534817	0.999933887	0.887795053	1.487109245	-0.599314192	0.098577044
ENSG00000120137	*PANK3*	4.456594851	0.012253721	-0.14668509	0.158938811	0.999933887	-0.377006577	0.022293258	-0.399299835	0.098706841
ENSG00000038382	*TRIO*	3.385034663	0.080968156	-0.007239462	0.088207618	0.999933887	0.189123233	0.421128908	-0.232005675	0.098733673
ENSG00000116906	*GNPAT*	3.267593809	-0.054846484	-0.052048592	-0.002797892	0.999933887	-0.281735765	-0.526773352	0.245037587	0.098733673
ENSG00000130724	*CHMP2A*	7.814680744	0.027564828	-0.014879121	0.042443948	0.999933887	0.418708607	0.580047711	-0.161339104	0.098733673
ENSG00000133794	*ARNTL*	5.964463607	0.005194723	-0.019856023	0.025050746	0.999933887	-0.620276249	-0.796574765	0.176298516	0.098733673
ENSG00000188486	*H2AX*	2.728019269	0.066765858	0.12479021	-0.058024352	0.999933887	0.137845829	-0.125860676	0.263706506	0.098805333
ENSG00000137747	*TMPRSS13*	-0.292601772	0.012960954	-0.274895978	0.287856932	0.999933887	0.472220167	1.052268819	-0.580048652	0.098936256
ENSG00000230551	*AC021078.1*	5.034773524	-0.001951428	-0.008835343	0.006883915	0.999933887	-0.160388471	0.184610521	-0.344998991	0.099008835
ENSG00000124875	*CXCL6*	1.62289214	0.040419894	0.066427881	-0.026007987	0.999933887	-0.838372319	-1.253426061	0.415053742	0.099239851
ENSG00000143653	*SCCPDH*	0.855216616	-0.010855157	0.075089242	-0.085944399	0.999933887	-0.095812607	-0.460695804	0.364883198	0.099278641
ENSG00000137563	*GGH*	1.990900711	-0.03435326	0.086634092	-0.120987352	0.999933887	0.59676925	0.945385065	-0.348615815	0.099522068
ENSG00000157045	*NTAN1*	2.848145999	0.192468998	0.012743409	0.179725589	0.999933887	-0.070380931	-0.370665439	0.300284508	0.099523943
ENSG00000163607	*GTPBP8*	1.362157272	0.03139998	0.019246804	0.012153176	0.999933887	-0.08583765	0.29940946	-0.38524711	0.099543943
ENSG00000186806	*VSIG10L*	-0.055326846	0.087055212	0.344845168	-0.257789956	0.999933887	0.721635082	1.294276366	-0.572641284	0.099638048
ENSG00000149743	*TRPT1*	2.42715873	0.084799201	-0.074848621	0.159647822	0.999933887	0.051504661	-0.237371521	0.288876183	0.099827201
ENSG00000283839	*AC096667.1*	2.892391438	-0.123780378	-0.080765498	-0.04301488	0.999933887	0.407961266	0.679353603	-0.271392338	0.09993016
ENSG00000227398	*KIF9-AS1*	-0.224121762	0.191584208	0.230421506	-0.038837299	0.999933887	-0.192967037	-0.953220141	0.760253105	0.09993016
ENSG00000274180	*NATD1*	6.582120851	-0.082973691	-0.125056902	0.042083211	0.999933887	-1.549317605	-1.856553507	0.307235901	0.09993016
ENSG00000070831	*CDC42*	8.690592154	-0.017616243	-0.013555697	-0.004060547	0.999933887	0.098751205	0.241599755	-0.14284855	0.099991931
ENSG00000279838	*AL356273.3*	4.365345061	-0.062979227	0.061166666	-0.124145893	0.999933887	-0.055108442	0.457254602	-0.512363044	0.100122643
ENSG00000164400	*CSF2*	-0.57053444	1.028429173	1.07997508	-0.051545908	0.999933887	3.973544366	4.933436777	-0.959892411	0.100211801
ENSG00000141759	*TXNL4A*	3.616474125	-0.025882924	-0.008305118	-0.017577806	0.999933887	-0.063365523	-0.27595641	0.212590887	0.100244648
ENSG00000005022	*SLC25A5*	4.906690288	-0.043603027	-0.052216869	0.008613842	0.999933887	-0.120077535	-0.309583274	0.189505739	0.100382707
ENSG00000116679	*IVNS1ABP*	11.599785201	-0.023883288	-0.051322551	0.027439263	0.999933887	0.289374829	0.482591992	-0.193217164	0.10047511
ENSG00000149761	*NUDT22*	2.63002669	0.024646594	0.128416508	-0.103769914	0.999933887	-0.292183584	-0.616516721	0.324333137	0.100751261
ENSG00000121931	*LRIF1*	3.932228252	-0.078068544	-0.056109052	-0.021959492	0.999933887	-0.068124508	0.187266146	-0.255390654	0.100789514
ENSG00000111880	*RNGTT*	3.490096367	-0.047474639	-0.08623958	0.038764941	0.999933887	0.102370837	0.334801979	-0.232431142	0.100789514
ENSG00000163904	*SENP2*	4.853951957	-0.017607972	0.045482821	-0.063090794	0.999933887	0.119026977	0.284763318	-0.165736341	0.100803808
ENSG00000160570	*DEDD2*	8.489739363	0.014377287	-0.002637832	0.01701512	0.999933887	-0.119216533	0.072171605	-0.191388137	0.100870811
ENSG00000229017	*LINC01277*	-0.41983696	-0.179390532	0.095748975	-0.275139507	0.999933887	1.291414622	1.974970831	-0.68355621	0.100936722
ENSG00000172890	*NADSYN1*	6.335727214	-0.016150965	0.000625387	-0.016776352	0.999933887	-0.092161406	-0.222700564	0.130539159	0.100983938
ENSG00000100813	*ACIN1*	7.298529526	-0.028921805	0.028077587	-0.056999392	0.999933887	-0.209628623	-0.345375349	0.135746726	0.101075262
ENSG00000286219	*NOTCH2NLC*	3.744162951	-0.035590308	-0.001514076	-0.034076233	0.999933887	0.715273783	1.096388563	-0.381114779	0.101126903
ENSG00000168374	*ARF4*	6.544945982	-0.015064447	-0.013326754	-0.001737692	0.999933887	0.260192102	0.420350668	-0.160158565	0.101278962
ENSG00000076662	*ICAM3*	6.277953409	-0.006469568	-0.029749551	0.023279982	0.999933887	-0.198944332	-0.413182232	0.2142379	0.101278962
ENSG00000177556	*ATOX1*	3.260593556	0.005104356	0.039338732	-0.034234377	0.999933887	0.600649527	0.840355852	-0.239706325	0.101278962
ENSG00000285888	*Z85996.2*	-0.982587404	0.034390928	-0.226075683	0.260466611	0.999933887	0.82531145	1.389033977	-0.563722527	0.101347999
ENSG00000266074	*BAHCC1*	-0.274883097	0.012840642	-0.020223763	0.033064404	0.999933887	-0.018156006	0.629828247	-0.647984253	0.101518518

ENSG00000065911	*MTHFD2*	3.761201318	0.012861256	0.121635325	-0.108774068	0.999933887	0.234656109	0.495647936	-0.260991827	0.101581224
ENSG00000184307	*ZDHHC23*	1.968467142	-0.26224443	0.064925674	-0.327170104	0.999933887	0.011141919	0.256430442	-0.245288524	0.1015866
ENSG00000056972	*TRAF3IP2*	2.99670158	0.051588521	0.043912837	0.007675684	0.999933887	0.873772338	1.202212692	-0.328440354	0.1015866
ENSG00000028277	*POU2F2*	6.296189466	0.000193189	0.013530669	-0.01333748	0.999933887	0.164196838	0.429723951	-0.265527113	0.101748948
ENSG00000242294	*STAG3L5P*	2.437003002	0.041325004	0.092551711	-0.051226707	0.999933887	0.461665567	0.822210567	-0.360545	0.101764703
ENSG00000172403	*SYNPO2*	-0.648960019	0.030646303	0.160645039	-0.129998736	0.999933887	-0.125910483	0.73824921	-0.864159693	0.101940346
ENSG00000164181	*ELOVL7*	-0.361962519	0.201953563	0.34620934	-0.144255777	0.999933887	2.305640725	3.091062431	-0.785421705	0.102156738
ENSG00000164366	*CCDC127*	1.347470768	0.006013577	0.070264369	-0.064250792	0.999933887	0.100463974	-0.266295005	0.366758979	0.102234553
ENSG00000150045	*KLRF1*	0.804985155	-0.089058073	-0.23154668	0.142488607	0.999933887	-0.211577308	0.173199653	-0.384776961	0.102255258
ENSG00000064419	*TNPO3*	4.906314681	-0.049765502	-0.010968837	-0.038796666	0.999933887	-0.193220194	-0.39078936	0.197569166	0.102335121
ENSG00000257285	*AL132780.1*	0.817126754	-0.020533404	0.019624359	-0.040157763	0.999933887	-0.468386775	-1.109736033	0.641349258	0.102335121
ENSG00000135387	*CAPRIN1*	5.716031418	-0.01855653	-0.049316897	0.030760367	0.999933887	-0.473422624	-0.625368107	0.151945483	0.102628661
ENSG00000286190	*AC055839.2*	3.104578688	0.047263218	0.007419954	0.039843263	0.999933887	-0.121033489	-0.454226102	0.333192613	0.102794244
ENSG00000165813	*CCDC186*	5.178159628	-0.040436457	-0.15279249	0.112356033	0.999933887	-0.813151138	-0.253150418	-0.56000072	0.102794244
ENSG00000205890	*AC108134.1*	-0.679375612	0.229886812	0.04796903	0.181917783	0.999933887	-0.443874124	-1.397670953	0.953796829	0.102853273
ENSG00000135899	*SP110*	7.040199518	-0.033905682	-0.089189072	0.05528339	0.999933887	-0.599694432	-0.770668849	0.170974417	0.102853273
ENSG00000112701	*SENP6*	5.463626279	-0.007512322	-0.096024513	0.088512191	0.999933887	-0.57203874	-0.989734849	0.417696109	0.102853273
ENSG00000230438	*SERPINB9P1*	4.701156391	-0.072610442	-0.008410658	-0.064199784	0.999933887	2.583924887	3.006617752	-0.422692864	0.10303842
ENSG00000198265	*HELZ*	5.620476042	-0.002069772	-0.114576619	0.112506847	0.999933887	-0.309516641	0.002634715	-0.312151355	0.103201787
ENSG00000113749	*HRH2*	4.467795348	0.02604853	0.012049613	0.013998917	0.999933887	0.053876476	-0.242830195	0.296706671	0.103225921
ENSG00000151014	*NOCT*	1.941522227	0.028378979	0.056998608	-0.028619629	0.999933887	1.034160754	1.419806069	-0.385645315	0.103323198
ENSG00000113504	*SLC12A7*	4.047554905	0.086707404	-0.002655205	0.089362609	0.999933887	0.75895985	1.111851579	-0.35289173	0.103329704
ENSG00000174799	*CEP135*	2.881172232	-0.177229616	-0.164004532	-0.013225084	0.999933887	-0.14555432	0.415735082	-0.561289402	0.103510089
ENSG00000064393	*HIPK2*	5.830765813	-0.058502689	-0.035676767	-0.022825922	0.999933887	-0.961264916	-1.19114111	0.229876194	0.103510089
ENSG00000260135	*MMP2-AS1*	1.026550495	0.083909551	-0.020963692	0.104873243	0.999933887	2.505556154	3.122178889	-0.616622736	0.103510089
ENSG00000114209	*PDCD10*	3.871921466	-0.026384063	-0.110285726	0.083901663	0.999933887	-0.350848599	-0.123823019	-0.22702558	0.103630863
ENSG00000009780	*FAM76A*	2.384779895	-0.05765018	0.135456674	-0.193106854	0.999933887	-0.210226157	-0.442606222	0.232380066	0.103694869
ENSG00000157933	*SKI*	7.213083218	0.006571805	-0.029313918	0.035885722	0.999933887	0.387002806	0.567869749	-0.180866943	0.103694869
ENSG00000186047	*DLEU7*	0.835749623	-0.09578177	-0.139147363	0.043365593	0.999933887	1.209089208	1.747620196	-0.538530988	0.103724509
ENSG00000228300	*FAM174C*	3.608013101	0.048422117	0.082576669	-0.034154552	0.999933887	-0.150920735	-0.406366145	0.25544541	0.103724509
ENSG00000196072	*BLOC1S2*	5.816562349	-0.08919446	-0.038127778	-0.051066681	0.999933887	-0.253713143	-0.084905512	-0.168807631	0.103805489
ENSG00000184545	*DUSP8*	4.084314304	0.053659961	0.073640753	-0.019980791	0.999933887	0.721510881	1.036577775	-0.315066894	0.103805489
ENSG00000170412	*GPRC5C*	0.600508248	-0.086532575	0.023959952	-0.110492527	0.999933887	0.555786095	-0.079466731	0.635252826	0.103805489
ENSG00000173039	*RELA*	7.449891307	0.032568881	0.016607561	0.015961321	0.999933887	0.809018934	1.004253149	-0.195234215	0.103805489
ENSG00000110799	*VWF*	2.348192963	-0.106861318	-0.079372792	-0.027488526	0.999933887	-2.347241049	-2.795237036	0.447995987	0.103805489
ENSG00000287562	*AL109615.4*	-0.530871267	-0.130226632	0.335758168	-0.4659848	0.999933887	1.12461336	1.744571447	-0.619958087	0.103906175
ENSG00000144029	*MRPS5*	2.71926261	-0.072607812	-0.123906991	0.05129918	0.999933887	0.022741978	-0.194335045	0.217077024	0.104048867
ENSG00000143515	*ATP8B2*	5.060379196	0.036727807	0.05999308	-0.023265274	0.999933887	0.14851783	0.351254835	-0.202737005	0.104048867
ENSG00000243147	*MRPL33*	3.432199424	0.062869835	0.08499511	-0.022125275	0.999933887	0.121133232	0.335959247	-0.214826015	0.104211557
ENSG00000184900	*SUMO3*	4.489361116	-0.012282905	-0.088461279	0.076178375	0.999933887	-0.049797013	0.132936721	-0.182733735	0.104214947
ENSG00000162378	*ZYG11B*	3.841452702	0.037532211	0.043824414	-0.006292202	0.999933887	-0.898126012	-1.179063871	0.280937859	0.104224408
ENSG00000232118	*BACH1-AS1*	1.434472517	-0.054151366	0.094430521	-0.148581887	0.999933887	-0.253278925	0.139887034	-0.393165959	0.104224408
ENSG00000274286	*ADRA2B*	0.694589978	0.06796318	0.170919564	-0.102956384	0.999933887	0.398822776	1.209197778	-0.810375002	0.104252176
ENSG00000135686	*KLHL36*	5.748572382	-0.019795261	-0.043907931	0.02411267	0.999933887	-0.356910196	-0.546805022	0.189894825	0.104440668
ENSG00000218283	*MORF4L1P1*	2.845615247	-0.093897222	-0.024950317	-0.068946906	0.999933887	-0.212501082	0.060228815	-0.272729896	0.104452239
ENSG00000166275	*BORCS7*	2.816186233	0.042520407	0.032125506	0.010394901	0.999933887	0.012021222	0.282636827	-0.270615605	0.104520941
ENSG00000122861	*PLAU*	10.763219043	0.146545597	0.240247849	-0.093702252	0.999933887	1.419441181	1.692566859	-0.273125678	0.104532299
ENSG00000267121	*AC008105.3*	4.83199317	-0.086160657	-0.025877074	-0.060283582	0.999933887	-0.04265716	-0.217328985	0.174671824	0.104541654
ENSG00000121671	*CRY2*	4.500061676	-0.011924893	0.023727304	-0.035652197	0.999933887	0.002229263	-0.208735026	0.21096429	0.104541654
ENSG00000237892	*KLF7-IT1*	1.453089024	0.011659634	0.034644265	-0.022984631	0.999933887	0.581360522	1.124344946	-0.542984424	0.104541654
ENSG00000130119	*GNL3L*	4.996265598	-0.051534607	-0.048171014	-0.003363593	0.999933887	-0.277670367	-0.486405708	0.208735341	0.104783203
ENSG00000165801	*ARHGEF40*	6.624190349	0.071026825	0.093923248	-0.022896422	0.999933887	-0.408157897	-0.618006107	0.20984821	0.104789883
ENSG00000116863	*ADPRS*	3.1059031	-0.054479832	0.082841915	-0.137321747	0.999933887	-0.148068876	-0.434235727	0.28616685	0.104791612
ENSG00000163714	*U2SURP*	5.091456213	-0.075668727	-0.149761282	0.074092555	0.999933887	-0.435479152	-0.04405112	-0.391428032	0.104840181
ENSG00000141429	*GALNT1*	4.927113348	-0.046457732	-0.157587831	0.1111301	0.999933887	-0.053404041	0.153969	-0.207373041	0.105015042
ENSG00000082258	*CCNT2*	5.918120195	-0.052078409	-0.135292823	0.083214414	0.999933887	-0.379651133	-0.008465218	-0.371185915	0.105105042
ENSG00000287978	*AC245407.2*	2.565957116	0.018633028	0.051877185	-0.033244158	0.999933887	1.067255598	1.413994939	-0.346739342	0.105105042
ENSG00000204103	*MAFB*	6.969120217	0.249003476	0.327514823	-0.078511346	0.999933887	0.265223995	0.466692269	-0.201468274	0.105200848
ENSG00000198763	*MT-ND2*	8.951473459	0.114766957	0.001876953	0.112890004	0.999933887	0.048301938	0.381645467	-0.333343529	0.105323806
ENSG00000135637	*CCDC142*	1.390873516	-0.048858867	0.210606003	-0.25946487	0.999933887	-0.109000139	-0.475244565	0.366244426	0.105323806
ENSG00000273729	*AC007686.3*	0.610574574	0.006499488	0.012534639	-0.006035151	0.999933887	-0.227412407	-0.772932493	0.545520086	0.105369064
ENSG00000270210	*AC104695.2*	0.841900565	0.15009608	0.209212239	-0.059116159	0.999933887	0.90850828	1.390126755	-0.481618475	0.105393171
ENSG00000162407	*PLPP3*	6.264472817	-0.072405286	-0.026390436	-0.04601485	0.999933887	-0.048715458	0.132004158	-0.180719616	0.105629691
ENSG00000118873	*RAB3GAP2*	4.665852705	0.005568292	-0.10529624	0.110864532	0.999933887	-0.274794034	-0.008203333	-0.266590701	0.105629691
ENSG00000101974	*ATP11C*	3.598742026	0.009402048	-0.041861892	0.05126394	0.999933887	0.319044422	0.714862342	-0.39581792	0.10570842
ENSG00000217275	*AL031777.1*	-0.959199157	0.099752619	0.229326131	-0.129573512	0.999933887	-0.638642206	-1.392275299	0.753633093	0.105798883
ENSG00000198382	*UVRAG*	5.72821318	-0.02288402	0.034839331	-0.057723351	0.999933887	0.340872898	0.48603329	-0.145160392	0.105798883
ENSG00000148341	*SH3GLB2*	4.503806908	0.056704073	0.06268359	-0.005979517	0.999933887	0.110067948	-0.086041864	0.196109812	0.105825566
ENSG00000224950	*AL390066.1*	2.345103325	-0.055371942	0.029707292	-0.085079234	0.999933887	0.892442114	1.169178371	-0.276736257	0.105993293
ENSG00000136448	*NMT1*	4.503496155	-0.033669259	-0.093764609	0.060095349	0.999933887	-0.17232479	-0.362929711	0.19060492	0.106348994
ENSG00000131378	*RFTN1*	3.731653675	-0.040045266	0.100501283	-0.140546548	0.999933887	0.296445161	0.540639711	-0.24419455	0.10648322
ENSG00000174953	*DHX36*	5.031566476	-0.061858983	-0.091699335	0.029840351	0.999933887	-0.066988639	0.259075131	-0.32606377	0.106519599
ENSG00000032444	*PNPLA6*	6.400332511	0.00210606	0.033841738	-0.031735679	0.999933887	-0.098321177	-0.249050548	0.150729371	0.106544659
ENSG00000122390	*NAA60*	5.120171656	-0.008574607	0.024259702	-0.032834309	0.999933887	-0.403372457	-0.593471779	0.190099322	0.106670166
ENSG00000169914	*OTUD3*	2.366331723	-0.016738795	0.097340455	-0.114079249	0.999933887	0.001013545	-0.232218352	0.233231897	0.106672634
ENSG00000144228	*SPOPL*	6.900455426	0.015441196	0.00061603	0.014825166	0.999933887	0.051910078	0.324789981	-0.272879902	0.106825355
ENSG00000225402	*AC010878.1*	1.300061228	0.061937853	0.096326598	-0.034388745	0.999933887	0.564993039	1.01204296	-0.447049922	0.106876444
ENSG00000260233	*ZNRD2-AS1*	2.484646571	0.133472416	-0.038796338	0.172268754	0.999933887	1.366402924	1.655567309	-0.289164385	0.106910591
ENSG00000162129	*CLPB*	1.258396911	0.111854107	0.072198537	0.03965557	0.999933887	0.12235993	0.503615291	-0.381255362	0.106931995
ENSG00000142867	*BCL10*	5.513804878	0.021956646	0.064836104	-0.042879458	0.999933887	0.042485947	0.204243265	-0.161757318	0.107137774
ENSG00000163395	*IGFN1*	-2.873480007	-0.234616251	-0.440740014	0.206123763	0.999933887	-0.427970682	-1.228157374	0.800186692	0.107137774
ENSG00000270980	*AC060764.1*	-2.261467299	-0.171065064	0.308620799	-0.479685863	0.999933887	0.619563332	1.22144941	-0.601886078	0.107137774
ENSG00000260361	*AC106028.3*	0.758694522	0.168022596	0.034635479	0.133387118	0.999933887	0.442014484	0.794706506	-0.352692021	0.107560395
ENSG00000169087	*HSPBAP1*	5.746218373	-0.028630888	-0.062788131	0.034157243	0.999933887	-0.312853608	-0.12055209	-0.192301518	0.107679315
ENSG00000108010	*GLRX3*	2.952102907	-0.007638483	-0.038164498	0.030526016	0.999933887	0.112915969	0.318839961	-0.205923991	0.10786858
ENSG00000015676	*NUDCD3*	3.920216863	-0.063780487	0.082242364	-0.146022851	0.999933887	-0.06175419	-0.245704434	0.183950244	0.10792566
ENSG00000178988	*MRFAP1L1*	4.357368939	-0.076104699	-0.176454398	0.100349699	0.999933887	-0.320338895	-0.543871251	0.223532356	0.108130518
ENSG00000244038	*DDOST*	4.751065769	0.026354538	-0.00601123	0.032365768	0.999933887	-0.048837953	-0.227428991	0.178591038	0.108377548
ENSG00000170043	*TRAPPC1*	4.488984369	0.034834241	-0.017860513	0.052694754	0.999933887	-0.133734678	-0.35183625	0.218101572	0.108397447
ENSG00000032219	*ARID4A*	6.007065076	-0.03439945	-0.159365421	0.12496597	0.999933887	-0.789782819	-0.287183765	-0.502599054	0.108877923
ENSG00000152229	*PSTPIP2*	5.315719079	0.010755345	-0.027148026	0.037903371	0.999933887	1.19856329	1.61038969	-0.4118264	0.108877923
ENSG00000138101	*DTNB*	2.587516576	0.029743655	0.194588823	-0.164845168	0.999933887	0.599254362	0.887490695	-0.288236333	0.108965914
ENSG00000025039	*RRAGD*	7.06243667	-0.059583306	0.000908161	-0.060491467	0.999933887	-0.089214611	0.075360799	-0.16457541	0.108993262
ENSG00000183562	*AC131971.1*	-0.563875964	-0.077789623	0.556412769	-0.634202392	0.999933887	0.26828017	-0.390828077	0.659108247	0.109062076
ENSG00000113013	*HSPA9*	7.4816381	0.010598182	-0.009965593	0.020563774	0.999933887	0.373368828	0.574388961	-0.201020133	0.109440359
ENSG00000215196	*BASP1-AS1*	2.836485205	0.123129635	0.053913095	0.069216539	0.999933887	0.878664622	1.182360533	-0.303695911	0.1098193
ENSG00000226979	*LTA*	0.990898492	0.046153505	-0.185768041	0.231921546	0.999933887	0.765195601	1.149214292	-0.384018692	0.1098193
ENSG00000167695	*TLCD3A*	0.465197457	0.069176136	0.009913332	0.059262804	0.999933887	1.416231658	2.05064836	-0.634416702	0.1098193
ENSG00000182919	*C11orf54*	2.586380121	-0.050828674	-0.02817495	-0.022653724	0.999933887	-0.476388361	-0.7656831	0.289294738	0.11001911
ENSG00000196646	*ZNF136*	3.185743919	-0.106185062	-0.12344821	0.017263148	0.999933887	-0.09608644	0.23244791	-0.32853435	0.110148302
ENSG00000170385	*SLC30A1*	5.063648413	-0.075301858	-0.068810281	-0.006491577	0.999933887	-0.508799145	-0.219926335	-0.28887281	0.110148302
ENSG00000121879	*PIK3CA*	4.928721561	-0.031898959	-0.124860107	0.092961148	0.999933887	-0.50199291	-0.157159895	-0.344833015	0.110148302
ENSG00000175206	*NPPA*	0.140840348	0.058465498	-0.386782505	0.445248003	0.999933887	-0.299156794	0.096489403	-0.395646197	0.110148302
ENSG00000172673	*THEMIS*	3.384420302	-0.012705777	-0.041379904	0.028674127	0.999933887	-0.036409318	0.293510881	-0.329920198	0.110148302
ENSG00000164970	*FAM219A*	3.857188601	0.050759674	-0.029630308	0.080389981	0.999933887	-0.472944673	-0.673305444	0.200360771	0.110156155

ENSG00000233223	AC016876.1	3.660824166	0.378420177	0.359423413	0.018996764	0.999933887	0.550242915	0.805419856	-0.255176941	0.110181365
ENSG00000261207	AL031717.1	0.836140432	0.00914956	-0.051709311	0.060858871	0.999933887	-0.787098426	-1.25866879	0.471570365	0.110181365
ENSG00000137478	FCHSD2	6.890564274	-0.030571481	-0.0318562	0.001284719	0.999933887	-0.080849982	0.116388652	-0.197238634	0.110211669
ENSG00000014824	SLC30A9	3.271794986	-0.082787392	-0.050500601	-0.032286791	0.999933887	-0.164905944	0.095844972	-0.260750916	0.110405925
ENSG00000183386	FHL3	4.920374048	0.032778862	0.061411531	-0.028632669	0.999933887	-0.84978757	-1.131941331	0.282153761	0.110405925
ENSG00000153214	TMEM87B	2.918030614	0.028953596	0.049650786	-0.02069719	0.999933887	-0.28890184	-0.612778139	0.323876298	0.110405925
ENSG00000168385	SEPTIN2	6.233345626	-0.042161024	-0.031032651	-0.011128373	0.999933887	-0.071753721	0.062335973	-0.134089693	0.11049517
ENSG00000078140	UBE2K	4.363035835	-0.049388245	-0.130522636	0.08113439	0.999933887	-0.427612325	-0.625095909	0.197483584	0.110609072
ENSG00000273033	LINC02035	4.810917527	-0.105109355	-0.075671636	-0.029437719	0.999933887	-0.378899021	-0.144380543	-0.234518479	0.110699653
ENSG00000050730	TNIP3	0.169333978	-0.012169778	-0.108310603	0.096140825	0.999933887	0.047606857	0.599397094	-0.551790236	0.110699653
ENSG00000109790	KLHL5	4.384320875	-0.030842313	0.013928157	-0.044770471	0.999933887	0.137357056	0.377096715	-0.239739659	0.110869099
ENSG00000151422	FER	0.478874277	0.117194526	-0.257041627	0.374236153	0.999933887	-0.260374452	0.215852969	-0.476227422	0.111137067
ENSG00000103363	ELOB	5.685184878	0.027019645	0.006089802	0.020929843	0.999933887	-0.082990002	-0.266713712	0.18372371	0.111160707
ENSG00000109756	RAPGEF2	7.829495897	0.009582703	-0.027999562	0.037582266	0.999933887	0.196264153	0.388926247	-0.192662094	0.111160707
ENSG00000011132	APBA3	4.779723626	0.104257389	0.022951952	0.081305437	0.999933887	0.673329912	0.886179149	-0.212849237	0.111620517
ENSG00000217930	PAM16	1.09121647	-0.062807923	-0.095620657	0.032812734	0.999933887	-0.14928443	-0.590824758	0.441540328	0.111620517
ENSG00000171681	ATF7IP	5.943906405	-0.041501467	-0.074737688	0.033236221	0.999933887	-0.069319448	0.19389197	-0.263211418	0.111708108
ENSG00000091039	OSBPL8	7.531291714	-0.067868098	-0.107680213	0.039812115	0.999933887	-0.712736498	-0.331458939	-0.381277559	0.111793028
ENSG00000037474	NSUN2	4.469048293	-0.026648009	0.052148985	-0.078796994	0.999933887	0.133500942	0.342302395	-0.208801453	0.11208977
ENSG00000253227	AC090192.2	-0.115192263	-0.288310978	0.471425983	-0.75973696	0.999933887	0.606824135	1.035049636	-0.428225501	0.112148816
ENSG00000008952	SEC62	6.535544668	-0.089747461	-0.19524102	0.105493559	0.999933887	-0.550821175	-0.338058716	-0.212762458	0.11227655
ENSG00000184164	CRELD2	3.434099262	0.007178729	0.002554225	0.004624504	0.999933887	0.29770171	0.553108671	-0.255406962	0.112568189
ENSG00000272391	POM121C	5.677768602	0.003920197	0.029620606	-0.025700409	0.999933887	-0.341751487	-0.498757998	0.157006511	0.112568189
ENSG00000135241	PNPLA8	7.006515735	-0.003730426	-0.194700366	0.19096994	0.999933887	0.744741367	1.112081238	-0.36733987	0.112568189
ENSG00000196937	FAM3C	1.115694249	-0.029530422	-0.057716159	0.028185737	0.999933887	-0.112249877	0.240739654	-0.35298953	0.112613591
ENSG00000089820	ARHGAP4	7.81702536	0.076235726	0.147424682	-0.071188956	0.999933887	0.077423984	-0.137539022	0.214963006	0.112669217
ENSG00000112592	TBP	2.862370974	-0.028872875	0.046100621	-0.074973496	0.999933887	-0.142942658	-0.360483042	0.217540384	0.112669217
ENSG00000112584	FAM120B	4.650717992	-0.048440581	0.069004658	-0.117445239	0.999933887	-0.003276535	0.158538267	-0.161814803	0.11277243
ENSG00000262580	AC087741.1	4.39139774	-0.126334924	-0.033012308	-0.093322616	0.999933887	0.167349858	0.336358239	-0.16900838	0.112785701
ENSG00000263001	GTF2I	2.820368037	-0.102430306	0.041583139	-0.144013445	0.999933887	0.01141702	0.224124682	-0.212707662	0.112785701
ENSG00000175121	WFDC5	0.634233891	0.310748449	0.465579372	-0.154830924	0.999933887	1.873114845	2.302515202	-0.429400358	0.112785701
ENSG00000210082	MT-RNR2	12.320455824	0.046262892	0.041245334	0.005017557	0.999933887	0.248459487	0.444125573	-0.195666086	0.112785701
ENSG00000204345	CD300LD	-0.534601288	-0.146760872	0.098473165	-0.245234037	0.999933887	-0.739123083	-1.678522084	0.939399001	0.112893745
ENSG00000170801	HTRA3	1.184604328	-0.132653743	-0.134937776	0.002284033	0.999933887	1.401777336	1.711636665	-0.309859329	0.11289729
ENSG00000072415	MPP5	5.39270516	0.052214272	0.039080396	0.013133876	0.999933887	0.06707224	0.467059586	-0.399987346	0.112999135
ENSG00000256806	C17orf100	-0.327904654	0.25153681	-0.045490045	0.297026856	0.999933887	-0.152679831	-0.858731489	0.706051657	0.112999436
ENSG00000138674	SEC31A	5.227983544	-0.027897622	0.019287704	-0.047185326	0.999933887	0.099825357	0.275213036	-0.175387679	0.112999436
ENSG00000120686	UFM1	4.348353054	-0.073731004	-0.158967445	0.085236442	0.999933887	-0.091414006	0.207551821	-0.298965827	0.113079494
ENSG00000137094	DNAJB5	2.332854321	0.065585205	0.036060407	0.029524798	0.999933887	0.21662051	0.647730182	-0.431109672	0.113098302
ENSG00000151725	CENPU	1.024069018	0.115056645	0.277765436	-0.162708791	0.999933887	0.190492239	0.784510779	-0.59401854	0.113119668
ENSG00000285448	AL136961.1	0.600972381	-0.073695427	0.164440131	-0.238135558	0.999933887	2.318479039	2.789347938	-0.470868899	0.113126736
ENSG00000261654	AL360270.2	0.307071748	0.047135025	-0.065224772	0.112359798	0.999933887	-0.313279902	0.622178175	-0.935458077	0.113126736
ENSG00000174839	DENND6A	3.679382537	0.046952314	0.013256054	0.03369626	0.999933887	-0.330827934	-0.595045765	0.264217832	0.11346909
ENSG00000007541	PIGQ	3.006422365	0.020254808	0.135703397	-0.11544859	0.999933887	0.081342482	-0.180521928	0.26186441	0.11346909
ENSG00000115170	ACVR1	2.063273656	-0.026914259	0.054476638	-0.081390897	0.999933887	-0.397265245	-0.695731824	0.298466579	0.11346909
ENSG00000112640	PPP2R5D	2.653597712	0.017670034	-0.009477865	0.027147899	0.999933887	0.084243992	-0.197261235	0.281505226	0.11346909
ENSG00000111850	SMIM8	2.295305739	-0.061591981	-0.054107546	-0.007484435	0.999933887	-1.288402903	-1.640221954	0.351819051	0.113481492
ENSG00000163683	SMIM14	3.491566521	-0.042094873	0.14982454	-0.191919414	0.999933887	-0.308265138	-0.606603284	0.298338146	0.113543144
ENSG00000155465	SLC7A7	5.762588532	-0.107568458	-0.01764452	-0.089923938	0.999933887	1.627320799	1.97909545	-0.351774651	0.11360349
ENSG00000145782	ATG12	5.899528268	-0.040070063	-0.060460557	0.020390494	0.999933887	-0.356088873	-0.187970624	-0.168118249	0.113628219
ENSG00000078142	PIK3C3	4.769875582	-0.0816068	-0.095484226	0.013877426	0.999933887	-0.138552579	0.069966763	-0.208519342	0.113643433
ENSG00000159461	AMFR	4.503398507	-0.00862869	-0.045993378	0.037364689	0.999933887	-0.434199391	-0.686771967	0.252572576	0.113711981
ENSG00000163464	CXCR1	8.205008192	-0.029982155	-0.055179184	0.025197029	0.999933887	-1.820009999	-2.180362392	0.360352393	0.113731991
ENSG00000171222	SCAND1	4.457766775	0.006970128	-0.062495696	0.069465823	0.999933887	-0.32863218	-0.578680636	0.250048456	0.113731991
ENSG00000226950	DANCR	1.234572119	-0.0012077	-0.086539454	0.085331754	0.999933887	0.215575104	-0.183688711	0.399263815	0.113731991
ENSG00000176155	CCDC57	4.868394551	0.026985239	0.023349157	0.003636083	0.999933887	0.320094136	0.503125434	-0.183031298	0.113753072
ENSG00000169964	TMEM42	1.31049505	-0.098212761	-0.01290935	-0.085303411	0.999933887	0.193309447	0.493223762	-0.299914315	0.113909042
ENSG00000221823	PPP3R1	5.542557954	-0.022569376	0.058836518	-0.081405895	0.999933887	-0.529246458	-0.713534367	0.184287909	0.113909042
ENSG00000159164	SV2A	0.335051631	-0.101073211	-0.183002542	0.081929332	0.999933887	-0.016244035	0.535823977	-0.552068012	0.113994411
ENSG00000170456	DENND5B	-0.506293463	-0.179577025	-0.292563223	0.112986198	0.999933887	-0.819809819	-0.200745832	-0.619063986	0.114103687
ENSG00000166855	CLPX	3.787588881	-0.037843678	-0.146301844	0.108458165	0.999933887	-0.444349713	-0.6313678	0.187018088	0.114187916
ENSG00000134996	OSTF1	6.390401761	0.015098344	0.027163017	-0.012064673	0.999933887	-0.702025686	-0.894614452	0.192588766	0.114232591
ENSG00000100228	RAB36	0.627775682	0.067810306	0.086607616	-0.01879731	0.999933887	-0.434486731	-1.12172548	0.687238748	0.114234562
ENSG00000187231	SESTD1	3.707620512	-0.042496221	-0.060806297	0.018310075	0.999933887	0.495091361	0.803353269	-0.308261907	0.114337998
ENSG00000251474	RPL32P3	3.433069941	-0.018940171	-0.001815526	-0.017124645	0.999933887	-0.219359798	-0.410366424	0.191006626	0.114337998
ENSG00000086288	NME8	0.246417147	-0.006479885	-0.179843866	0.173363981	0.999933887	-0.108543679	0.56097914	-0.669522819	0.114337998
ENSG00000227500	SCAMP4	5.218576285	-0.035090245	-0.034353245	-0.000737	0.999933887	-1.105269281	-1.339040135	0.233770854	0.114383477
ENSG00000112877	CEP72	0.17787788	0.189210489	-0.172970673	0.362181162	0.999933887	0.083057313	0.664559568	-0.581502256	0.114468581
ENSG00000163389	POGLUT1	2.0906616	0.059210714	-0.143612983	0.202823697	0.999933887	0.159935715	0.45419608	-0.294260366	0.114580358
ENSG00000169727	GPS1	4.056080178	0.016686048	0.065167943	-0.048481895	0.999933887	0.05231531	-0.15056261	0.20287792	0.114649318
ENSG00000198840	MT-ND3	7.473521289	0.02495813	-0.0113073	0.03626543	0.999933887	0.090838485	0.315985104	-0.225146618	0.1148211
ENSG00000256249	AC026333.3	3.984240447	0.222397882	0.236686052	-0.01428817	0.999933887	0.826055666	1.197644468	-0.371588802	0.114822464
ENSG00000112234	FBXL4	2.34361323	0.051342282	-0.005026056	0.056368338	0.999933887	-0.539801211	-0.846346493	0.306545282	0.115076184
ENSG00000144445	KANSL1L	3.682882328	-0.015430501	-0.080078955	0.064648454	0.999933887	-0.254323324	-0.04364622	-0.210677104	0.115141866
ENSG00000183741	CBX6	6.631648737	-0.060034926	0.0037768	-0.063811726	0.999933887	-0.260589429	-0.423757345	0.163167915	0.115196061
ENSG00000112972	HMGCS1	5.336666201	0.021437244	-0.067867076	0.08930432	0.999933887	0.623750801	0.840382515	-0.216631714	0.11529042
ENSG00000131143	COX4I1	6.931286124	-0.013636083	-0.052796031	0.039159948	0.999933887	0.368488343	0.513974112	-0.145485769	0.11529042
ENSG00000248309	MEF2C-AS1	1.228413207	-0.103944252	0.106316384	-0.210260636	0.999933887	0.554268505	0.908987651	-0.354719146	0.115355374
ENSG00000005007	UPF1	7.46308019	-0.020755513	0.024480154	-0.045235667	0.999933887	-0.288009775	-0.438532546	0.150522771	0.115357968
ENSG00000115267	IFIH1	7.090559157	0.018666317	-0.085713328	0.104379645	0.999933887	0.69538453	0.933188942	-0.237804412	0.115359428
ENSG00000184708	EIF4ENIF1	4.004948955	-0.067559084	0.026474152	-0.094033236	0.999933887	0.289221305	0.525987176	-0.236765872	0.115652477
ENSG00000143127	ITGA10	-0.91991926	-0.071749916	-0.348553862	0.276803945	0.999933887	-0.002807369	-0.68300258	0.680195211	0.115652477
ENSG00000006555	TTC22	1.532210898	0.102245464	0.054021303	0.048224161	0.999933887	0.052457384	-0.262366016	0.3148234	0.115753855
ENSG00000123689	G0S2	10.673364821	0.732704413	0.667785604	0.064918809	0.999933887	2.008472164	2.480931412	-0.472459249	0.115891391
ENSG00000155111	CDK19	4.784567117	-0.055078664	-0.033510269	-0.021568395	0.999933887	-0.530944473	-0.773541354	0.242596881	0.115922268
ENSG00000126067	PSMB2	3.860238894	-0.040851322	-0.029494694	-0.011356628	0.999933887	-0.163694821	-0.389294164	0.225599343	0.11604271
ENSG00000136868	SLC31A1	3.444551085	0.000549608	-0.0662549	0.066804508	0.999933887	-0.325054659	-0.050732758	-0.274321901	0.116156376
ENSG00000084073	ZMPSTE24	3.534886526	-0.014036763	-0.124564903	0.11052814	0.999933887	-0.246919419	-0.511964528	0.265045109	0.11626822
ENSG00000134317	GRHL1	-0.041770919	-0.040339304	-0.134773827	0.094434524	0.999933887	-0.050412869	0.619511458	-0.669924327	0.116281335
ENSG00000197620	EOLA1	2.899850695	0.029224884	-0.070772414	0.099997298	0.999933887	-0.148370614	-0.424374174	0.27600356	0.116315983
ENSG00000182004	SNRPE	3.058413431	-0.092414845	-0.075618541	-0.016796303	0.999933887	0.021696651	-0.245979762	0.267676412	0.116319501
ENSG00000115966	ATF2	4.473920342	-0.044587258	-0.099266777	0.054679519	0.999933887	-0.465077912	-0.215931037	-0.249146875	0.116357077
ENSG00000077454	LRCH4	5.657185688	0.054073513	0.043337023	0.01073649	0.999933887	-0.071609998	-0.232780096	0.161170098	0.116450708
ENSG00000164327	RICTOR	8.003805841	-0.08356935	-0.165234651	0.081665301	0.999933887	-0.961512853	-0.453763222	-0.507749631	0.116450708
ENSG00000070614	NDST1	6.727488658	-0.103779661	-0.085586266	-0.018193395	0.999933887	-1.099946737	-1.333008051	0.233061313	0.116462746
ENSG00000130449	ZSWIM6	6.300179647	0.036714912	0.046279738	-0.009564826	0.999933887	0.097881447	0.291093857	-0.19321241	0.116462746
ENSG00000100105	PATZ1	3.641496379	-0.003084522	0.011377568	-0.014462089	0.999933887	0.068847982	-0.122470838	0.191318819	0.11654885
ENSG00000171208	NETO2	0.362967757	0.016462312	0.024538449	-0.008076137	0.999933887	0.175521863	0.625205276	-0.449683413	0.116607313
ENSG00000125810	CD93	6.676611179	-0.045486309	0.00088145	-0.046367758	0.999933887	-0.968233226	-1.322725953	0.354492727	0.116698242
ENSG00000127507	ADGRE2	7.506315674	-0.016564153	0.022274796	-0.03883895	0.999933887	-0.382893322	-0.611438553	0.228545231	0.116698242
ENSG00000166340	TPP1	8.294950498	0.016495132	-0.020465266	0.036960398	0.999933887	0.357594312	0.52466894	-0.167074628	0.116959882
ENSG00000270195	AC016773.1	-0.865655797	-0.204895667	-0.017988713	-0.186906954	0.999933887	-0.748293916	-1.616329649	0.868035733	0.117103212
ENSG00000136758	YME1L1	6.531551556	-0.01816096	-0.034643278	0.016482318	0.999933887	0.236689878	0.374758872	-0.138068994	0.117146087
ENSG00000271913	AL035530.2	5.509090239	0.46910624	0.605547938	-0.136441697	0.999933887	1.666360957	2.083465256	-0.417104299	0.117480881
ENSG00000132824	SERINC3	8.935073608	-0.043198946	-0.007933643	-0.035265302	0.999933887	-0.103780578	0.028206377	-0.131986956	0.117507751

ENSG00000167434	*CA4*	3.729393979	-0.039517196	-0.079013496	0.0394963	0.999933887	-0.668355551	-0.964359726	0.296004175	0.117985742
ENSG00000100324	*TAB1*	2.553588187	0.020203002	-0.006747209	0.026950211	0.999933887	-0.007083853	-0.296931654	0.289847801	0.117997583
ENSG00000100603	*SNW1*	5.750011995	-0.041104167	-0.101880772	0.060776604	0.999933887	0.106905108	0.283276221	-0.176371113	0.118098305
ENSG00000100325	*ASCC2*	4.750249535	0.022630382	-0.034394755	0.057025137	0.999933887	0.148261559	0.324684578	-0.176423018	0.118205289
ENSG00000157916	*RER1*	5.856336708	-0.01039251	-0.044901954	0.034509444	0.999933887	-0.188262047	-0.321097203	0.132835156	0.118212941
ENSG00000158716	*DUSP23*	3.403485437	-0.160628492	-0.107711805	-0.052916687	0.999933887	-0.811743954	-1.166659096	0.354915142	0.118325264
ENSG00000134871	*COL4A2*	1.373641028	0.176641993	0.175227075	0.001414918	0.999933887	1.006639236	1.444798517	-0.43815928	0.118709616
ENSG00000138448	*ITGAV*	2.220588531	-0.139942874	-0.217858981	0.077916108	0.999933887	-0.300649995	0.041099114	-0.341749109	0.118709616
ENSG00000198301	*SDAD1*	2.343323978	-0.087351087	0.035682385	-0.123033472	0.999933887	-0.105631729	0.194172701	-0.29980443	0.118774083
ENSG00000132549	*VPS13B*	5.753288366	0.004423632	-0.011932594	0.016356226	0.999933887	-0.129000362	0.187870765	-0.316871127	0.118837325
ENSG00000148634	*HERC4*	4.974059769	-0.024902361	-0.107824556	0.082922195	0.999933887	-0.450086912	-0.635228583	0.185141671	0.119085351
ENSG00000108829	*LRRC59*	5.163162916	0.000682046	-0.069929412	0.070611458	0.999933887	0.168477442	0.32522437	-0.156746928	0.119118959
ENSG00000126858	*RHOT1*	3.862463662	0.009893814	-0.065371054	0.075264869	0.999933887	-0.558945563	-0.811185024	0.252239461	0.119216505
ENSG00000101608	*MYL12A*	6.949736387	0.012890738	0.019034616	-0.006143878	0.999933887	-0.095868989	-0.270299474	0.174430486	0.119484671
ENSG00000260807	*CEROX1*	0.362540587	-0.24810013	0.024403391	-0.272503521	0.999933887	0.194331068	-0.270902117	0.465233185	0.119509992
ENSG00000089060	*SLC8B1*	5.214992296	0.025445345	0.010544347	0.014900998	0.999933887	-0.288182658	-0.460476186	0.172293528	0.119509992
ENSG00000127124	*HIVEP3*	3.531866482	0.023956258	0.057834926	-0.033878668	0.999933887	0.225707144	0.46964876	-0.243941616	0.119509992
ENSG00000171552	*BCL2L1*	3.583933089	0.017241206	0.102048653	-0.084807447	0.999933887	0.939756126	1.344793234	-0.405037109	0.119509992
ENSG00000137845	*ADAM10*	7.436953638	-0.056059614	-0.047090092	-0.008969523	0.999933887	-0.773832575	-0.546579757	-0.227252818	0.119667688
ENSG00000159496	*RGL4*	5.396603887	0.12766711	0.086856237	0.040810873	0.999933887	0.133102615	0.320950745	-0.18784813	0.119813409
ENSG00000232803	*SLCO4A1-AS1*	-0.167468148	0.331691172	-0.146082882	0.477774054	0.999933887	1.041656168	1.617383157	-0.57572699	0.119813409
ENSG00000287737	*AC132872.5*	1.813576146	0.066912215	-0.055995038	0.122907253	0.999933887	-0.13183397	-0.469593726	0.337759756	0.119813409
ENSG00000137337	*MDC1*	3.194617918	-0.027642408	-0.025856721	-0.001785687	0.999933887	0.149162449	0.338709951	-0.189547502	0.119813409
ENSG00000092067	*CEBPE*	1.053903268	-0.21540638	0.256476136	-0.471882516	0.999933887	-0.324062568	-0.717710702	0.393648135	0.119859893
ENSG00000281530	*AC004461.2*	0.371182788	-0.086128573	0.089830993	-0.175959565	0.999933887	-0.388319209	-1.006418177	0.618098968	0.119887643
ENSG00000159111	*MRPL10*	3.972241305	-0.054673226	-0.031245228	-0.023427997	0.999933887	0.00758924	-0.191612864	0.199202104	0.11989341
ENSG00000173588	*CEP83*	1.820153925	-0.082865221	-0.130345415	0.047480194	0.999933887	0.062300851	0.545037504	-0.482736653	0.119900285
ENSG00000138593	*SECISBP2L*	4.881867393	0.042501183	-0.02063151	0.063132693	0.999933887	-0.538980712	-0.188227555	-0.350753157	0.120000158
ENSG00000110344	*UBE4A*	5.58505566	-0.028494776	-0.064019994	0.035525219	0.999933887	-0.13463349	0.043848541	-0.178482031	0.120012602
ENSG00000172197	*MBOAT1*	5.312908633	-0.016256604	0.010091958	-0.026348562	0.999933887	-0.367742256	-0.528010196	0.160267939	0.120012602
ENSG00000125503	*PPP1R12C*	7.246324362	0.089012699	0.146203705	-0.057191006	0.999933887	0.001775699	-0.211147282	0.212922981	0.120039527
ENSG00000159885	*ZNF222*	2.904360319	0.042684225	-0.016992228	0.059676453	0.999933887	0.15585936	0.39697669	-0.24111733	0.12004278
ENSG00000162959	*MEMO1*	1.161129539	0.156436031	0.230458861	-0.07402283	0.999933887	-0.330201583	-0.761820022	0.431618439	0.120088869
ENSG00000153815	*CMIP*	6.817618086	-0.021884901	0.055196879	-0.07708178	0.999933887	-0.12972986	-0.305734533	0.176004673	0.120088869
ENSG00000160796	*NBEAL2*	9.322782875	0.054918114	0.054145514	0.000772599	0.999933887	0.349401008	0.500083846	-0.150682839	0.120134619
ENSG00000177697	*CD151*	2.190454583	0.055352121	-0.001647225	0.056999346	0.999933887	-0.34512672	-0.682436571	0.337309851	0.120227403
ENSG00000152492	*CCDC50*	1.893495936	0.023366368	0.013288358	0.01007801	0.999933887	0.124134336	0.445944899	-0.321810563	0.120289173
ENSG00000186767	*SPIN4*	-0.315830061	0.107209126	-0.086222607	0.193431733	0.999933887	-0.439231224	0.212168931	-0.651400156	0.120497302
ENSG00000057608	*GDI2*	5.913505141	-0.070063299	-0.067747369	-0.002315931	0.999933887	-0.451985293	-0.637340194	0.185354901	0.12061387
ENSG00000178467	*P4HTM*	3.183517709	0.077807975	0.128454426	-0.050646451	0.999933887	0.134102909	-0.092182367	0.226285276	0.12061387
ENSG00000152219	*ARL14EP*	2.18345939	-0.070989757	-0.19099261	0.120002853	0.999933887	0.106574199	0.401941003	-0.295366804	0.120720268
ENSG00000261460	*AC009690.2*	2.188807273	0.037553073	0.069893111	-0.032340038	0.999933887	0.027946678	0.347281763	-0.319335085	0.121462023
ENSG00000165682	*CLEC1B*	1.502572721	-0.141190599	0.089992857	-0.231183456	0.999933887	1.098146157	1.606350808	-0.508204651	0.121721554
ENSG00000225300	*AL591623.1*	-0.350796777	0.063661399	0.175474941	-0.111813542	0.999933887	0.615355179	1.168734239	-0.553379059	0.121721554
ENSG00000138381	*ASNSD1*	1.525422418	-0.072749039	-0.30529865	0.232549611	0.999933887	0.238072857	0.58702157	-0.348948713	0.121799923
ENSG00000074356	*NCBP3*	5.296766589	-0.045916045	0.002018515	-0.04793456	0.999933887	-0.303627858	-0.460135571	0.156507713	0.121976844
ENSG00000197122	*SRC*	4.106528664	0.016412994	0.06364315	-0.047230155	0.999933887	1.920281813	2.34901685	-0.428735036	0.122040223
ENSG00000165458	*INPPL1*	6.238909568	0.059545181	0.085250632	-0.025705451	0.999933887	-0.077652725	-0.221604792	0.143952066	0.122116566
ENSG00000163719	*MTMR14*	7.491575328	0.004624077	-0.006129748	0.010753825	0.999933887	0.129633268	0.291107524	-0.161474257	0.122116566
ENSG00000184056	*VPS33B*	-0.179381988	-0.318174323	0.007439429	-0.325613752	0.999933887	-0.172129893	0.357864602	-0.529994495	0.122137955
ENSG00000113272	*THG1L*	0.747638845	0.060477137	-0.150287888	0.210765025	0.999933887	0.598606589	1.044186045	-0.445579456	0.122137955
ENSG00000197483	*ZNF628*	3.184382574	0.092036266	0.096070683	-0.004034417	0.999933887	-0.251943855	-0.562280624	0.310336768	0.122164108
ENSG00000172780	*RAB43*	3.720159638	0.118620494	0.063611055	0.055009438	0.999933887	-0.373197815	-0.693190717	0.319992902	0.122233267
ENSG00000170604	*IRF2BP1*	2.805846596	0.098572973	0.156455525	-0.057882552	0.999933887	-0.070612724	-0.38498728	0.314374556	0.122233267
ENSG00000137270	*GCM1*	0.384653523	-0.235911847	0.046666676	-0.282578523	0.999933887	-0.969342252	-1.527602801	0.558260548	0.122297954
ENSG00000143258	*USP21*	3.198374122	0.044817942	0.08560087	-0.040782929	0.999933887	-0.02390551	-0.262468374	0.238562864	0.122297954
ENSG00000151612	*ZNF827*	2.050253717	-0.110395013	0.037168489	-0.147563501	0.999933887	0.020580433	0.324559509	-0.303979076	0.122556499
ENSG00000136738	*STAM*	3.608347036	0.050229211	0.119649867	-0.069420656	0.999933887	0.057074745	0.253699679	-0.196624934	0.122556679
ENSG00000108244	*KRT23*	5.329220898	0.042241054	-0.008598858	0.050839913	0.999933887	0.936603936	1.135458371	-0.198854435	0.122577457
ENSG00000114127	*XRN1*	6.410894427	0.000321724	-0.094745882	0.095067606	0.999933887	-0.6464633	-0.206220427	-0.440242873	0.122667394
ENSG00000047644	*WWC3*	6.508034863	0.049888175	0.085609656	-0.035721482	0.999933887	-0.005489586	-0.181627711	0.176138125	0.122695023
ENSG00000278129	*ZNF8*	2.347624806	0.0160897	-0.118805203	0.134894903	0.999933887	-0.187246474	-0.451666887	0.264420413	0.122816649
ENSG00000189051	*RNF222*	0.037985613	0.142243053	0.358691137	-0.216448084	0.999933887	0.293111765	-0.31146535	0.604577115	0.122917817
ENSG00000171488	*LRRC8C*	3.883878815	-0.014079541	-0.019313015	0.005233474	0.999933887	-0.084700795	0.16924424	-0.253945034	0.122972913
ENSG00000226330	*AL606489.1*	-1.440908946	0.348672111	-0.54952387	0.898195981	0.999933887	-1.784388165	-2.552461228	0.768073063	0.1230916
ENSG00000105519	*CAPS*	1.593572459	0.019020341	-0.068231659	0.087252	0.999933887	0.062702943	-0.341662071	0.404365015	0.123154166
ENSG00000176749	*CDK5R1*	0.981814328	-0.047371117	0.092191669	-0.139562786	0.999933887	-0.216385695	-0.614402358	0.398016663	0.123194813
ENSG00000126351	*THRA*	5.865209306	-0.025212714	-0.038021426	0.012808712	0.999933887	-0.331571737	-0.131635786	-0.199935951	0.12325158
ENSG00000073969	*NSF*	2.453768485	-0.077613176	-0.015031932	-0.062581244	0.999933887	-0.234341662	-0.520341626	0.285999964	0.123255393
ENSG00000155876	*RRAGA*	4.446580459	0.015567976	0.002394815	0.013173161	0.999933887	-0.337177374	-0.505065445	0.167888071	0.123255393
ENSG00000170876	*TMEM43*	6.427766037	-0.030722534	-0.026105101	-0.004617433	0.999933887	-0.534354775	-0.694457483	0.160102707	0.123392767
ENSG00000173681	*BCLAF3*	3.678848619	0.054571443	-0.033714368	0.088285811	0.999933887	-0.308119315	-0.017253638	-0.290865676	0.123454877
ENSG00000176171	*BNIP3*	2.893382246	0.036134796	0.059472339	-0.023337543	0.999933887	-0.098118069	0.134737048	-0.232855117	0.123477181
ENSG00000198040	*ZNF84*	2.553145993	-0.079019401	0.007312201	-0.086331602	0.999933887	-0.258643615	0.044428377	-0.303071992	0.123679931
ENSG00000111911	*HINT3*	5.03302609	-0.042687528	0.007624048	-0.050311576	0.999933887	-0.699799731	-0.465820373	-0.233979358	0.123679931
ENSG00000103888	*CEMIP*	0.04687767	0.210884225	-0.058875006	0.269759231	0.999933887	-0.450945839	-0.979575542	0.528629703	0.123855634
ENSG00000205138	*SDHAF1*	1.199423486	0.1350885	0.176526033	-0.041437532	0.999933887	-0.26409815	-0.57236338	0.30826523	0.123855634
ENSG00000215301	*DDX3X*	10.432557352	-0.006548367	-0.040315765	0.033767397	0.999933887	0.203263693	0.394001603	-0.190737911	0.123855634
ENSG00000229644	*NAMPTP1*	5.791907432	-0.037209598	-0.073466027	0.036256429	0.999933887	0.398090742	0.660810495	-0.262719753	0.12408535
ENSG00000183891	*TTC32*	4.740078707	0.114175858	0.140186395	-0.026010537	0.999933887	0.808379813	0.998755218	-0.190375405	0.124209501
ENSG00000135503	*ACVR1B*	5.381305577	-0.058748512	-0.0094097	-0.049338812	0.999933887	-0.355864465	-0.148011607	-0.207852858	0.124209501
ENSG00000178177	*LCORL*	2.487515958	-0.025514513	0.007242191	-0.032756704	0.999933887	-0.40130367	-0.06831464	-0.33298903	0.124252284
ENSG00000229152	*ANKRD10-IT1*	2.576500469	-0.095461784	-0.213967241	0.118505457	0.999933887	-0.083388719	0.347608841	-0.43099756	0.12448161
ENSG00000104951	*IL4I1*	0.776125445	-0.037357313	-0.0086906	-0.028666713	0.999933887	0.851614352	1.367354283	-0.515739931	0.12448161
ENSG00000086300	*SNX10*	6.076169835	0.023417526	-0.054550881	0.077968407	0.999933887	1.255849854	1.634383104	-0.37853325	0.124531881
ENSG00000076067	*RBMS2*	1.03192729	-0.065966194	-0.09506159	0.029095397	0.999933887	-0.246714164	0.260176222	-0.506890386	0.124603374
ENSG00000153310	*CYRIB*	8.949346205	-0.012526417	-0.004039553	-0.008486865	0.999933887	-0.085189498	0.046999818	-0.132189316	0.124635694
ENSG00000092203	*TOX4*	6.911098384	-0.009618842	-0.030565012	0.02094617	0.999933887	0.111141639	0.255913178	-0.144771538	0.124818959
ENSG00000287733	*AC083862.2*	5.69974831	-0.002033641	-0.082895961	0.08086232	0.999933887	-0.166379132	0.045099772	-0.211478904	0.124867556
ENSG00000279946	*AC244034.3*	-1.994152684	0.187937642	1.230400671	-1.042463029	0.999933887	2.186828089	3.463783671	-1.276955582	0.12487743
ENSG00000104549	*SQLE*	2.7344362	-0.019778537	0.081450212	-0.101228749	0.999933887	0.648918498	0.907465255	-0.258546757	0.12487743
ENSG00000122729	*ACO1*	2.870524653	0.049613809	0.085881414	-0.036267605	0.999933887	-0.35709593	0.00502292	-0.36211885	0.125059525
ENSG00000129128	*SPCS3*	5.522900165	0.030978521	0.016608281	0.014370241	0.999933887	-0.569379291	-0.813837933	0.244458641	0.125084836
ENSG00000183726	*TMEM50A*	6.923694609	0.0070499	-0.014582823	0.021632723	0.999933887	-0.340124536	-0.498718269	0.158593733	0.125317285
ENSG00000185507	*IRF7*	6.798625497	0.113788095	0.104614472	0.009173623	0.999933887	-0.025547709	-0.245653608	0.220105899	0.125375505
ENSG00000170340	*B3GNT2*	4.304435825	0.056731054	0.226863845	-0.170132791	0.999933887	-0.377480906	-0.588644604	0.210803697	0.125375505
ENSG00000142039	*CCDC97*	4.968793952	-0.001492755	0.001422461	-0.002915216	0.999933887	-0.474606557	-0.703541528	0.228934971	0.125449982
ENSG00000010256	*UQCRC1*	4.196434723	0.00807474	-0.038600444	0.046675184	0.999933887	-0.092044193	-0.293480055	0.201435862	0.125467634
ENSG00000152193	*OBI1*	0.752511207	0.078990135	-0.509127099	0.588117234	0.944094537	-0.407149661	-0.019453623	-0.387696038	0.125481234
ENSG00000075415	*SLC25A3*	6.681885632	0.00834399	-0.027624494	0.035968484	0.999933887	-0.122925924	-0.248113319	0.125187395	0.125548004
ENSG00000272221	*AL645933.2*	0.63350834	-0.176138446	0.010443114	-0.18658156	0.999933887	-0.04086628	-0.458140733	0.417274453	0.125643803
ENSG00000067369	*TP53BP1*	5.85228135	-0.019713109	-0.048907372	0.029194263	0.999933887	0.263187721	0.434318295	-0.171130574	0.125643803
ENSG00000226200	*SGMS1-AS1*	2.176194754	-0.139434116	0.100142662	-0.239576779	0.999933887	0.604880918	0.847949607	-0.243068688	0.125933618
ENSG00000142002	*DPP9*	5.173207347	-0.017992288	-0.005436855	-0.012555433	0.999933887	-0.055031437	-0.193302616	0.13827118	0.125933618
ENSG00000112343	*TRIM38*	6.411593152	-0.0460308	-0.030106914	-0.015923885	0.999933887	-0.288325687	-0.427889036	0.13956335	0.126191015

ENSG00000156639	*ZFAND3*	7.214211626	0.006610564	0.023516595	-0.016906031	0.999933887	0.240333999	0.370423521	-0.130089522	0.126266535
ENSG00000078487	*ZCWPW1*	4.326463881	-0.053189431	0.045043541	-0.098232972	0.999933887	1.132330655	1.369946577	-0.237615922	0.126302178
ENSG00000080493	*SLC4A4*	-0.160883269	-0.095503326	0.071823765	-0.16732709	0.999933887	-0.155997287	0.473219339	-0.629216626	0.126373718
ENSG00000160131	*VMA21*	2.163593343	-0.001678249	0.108642776	-0.110321025	0.999933887	-0.07584337	-0.38612195	0.310278579	0.12648322
ENSG00000244687	*UBE2V1*	1.549623715	0.035640765	-0.136514602	0.172155367	0.999933887	-0.07310535	0.239060664	-0.312166014	0.126573736
ENSG00000124134	*KCNS1*	4.528710425	-0.028965899	0.044618162	-0.073584061	0.999933887	-0.348721883	-0.139691435	-0.209030448	0.12661267
ENSG00000235217	*TSPY26P*	1.766328587	-0.003606131	0.037109222	-0.040715353	0.999933887	1.084031931	1.487351816	-0.403319885	0.126723203
ENSG00000139323	*POC1B*	3.602069194	-0.001984684	-0.02223919	0.020254506	0.999933887	0.349124294	0.595700994	-0.246576699	0.12672697
ENSG00000285976	*AL135905.2*	2.127978175	0.171775961	0.317866608	-0.146090647	0.999933887	0.573605562	0.071557883	0.50204768	0.126733605
ENSG00000167548	*KMT2D*	8.637677011	0.03651769	0.080580248	-0.044062558	0.999933887	-0.611332038	-0.788263005	0.176930966	0.126756457
ENSG00000128590	*DNAJB9*	6.224200412	0.10073216	0.055291017	0.045441143	0.999933887	0.067764614	0.298301587	-0.230536973	0.126864727
ENSG00000260565	*ERVK13-1*	4.714616374	0.037054177	-0.027725234	0.064779411	0.999933887	-0.626571018	-0.85900771	0.232436693	0.127040523
ENSG00000186654	*PRR5*	2.894947679	0.056291349	0.023972909	0.03231844	0.999933887	0.041023906	0.312987135	-0.271963229	0.127171507
ENSG00000063176	*SPHK2*	1.740831198	0.099272755	0.20452201	-0.105249256	0.999933887	-0.105469862	-0.457551852	0.35208199	0.127260287
ENSG00000172331	*BPGM*	2.962354501	-0.130888383	-0.089240504	-0.041647879	0.999933887	-0.433292148	-0.676963091	0.243670942	0.127266471
ENSG00000175352	*NRIP3*	6.401416981	0.020522648	0.062522086	-0.041999438	0.999933887	0.056936364	0.317429145	-0.26049278	0.127266471
ENSG00000155636	*RBM45*	0.683339674	-0.02717538	-0.171207263	0.144031883	0.999933887	-0.118073969	-0.572696659	0.454622689	0.127266471
ENSG00000157014	*TATDN2*	4.852805979	0.010411284	0.063885565	-0.053474281	0.999933887	1.06415544	1.289574046	-0.225418606	0.127266471
ENSG00000196139	*AKR1C3*	1.081396691	0.023084231	-0.132599633	0.155683864	0.999933887	-0.727798151	-1.097617308	0.369819157	0.127266471
ENSG00000155957	*TMBIM4*	4.11418331	-0.00229534	-0.064020946	0.061725607	0.999933887	0.768891679	0.992704027	-0.223812348	0.127268375
ENSG00000187498	*COL4A1*	0.123499182	-0.056384177	0.060338061	-0.116722238	0.999933887	-0.1415484	0.351441584	-0.492989984	0.127335856
ENSG00000260542	*AL499627.1*	-1.59499495	0.160106973	0.019437953	0.140669021	0.999933887	0.411862166	1.058649204	-0.646787039	0.127379246
ENSG00000196154	*S100A4*	7.825153019	-0.044917626	-0.046970787	0.002053161	0.999933887	-0.111006861	-0.345437556	0.234430695	0.127552893
ENSG00000125386	*FAM193A*	5.508951095	-0.020636176	0.007459833	-0.028096009	0.999933887	-0.307267199	-0.482361627	0.175094428	0.127945108
ENSG00000198912	*C1orf174*	2.987633172	-0.051081659	-0.113004926	0.061923267	0.999933887	-0.321812608	-0.521849674	0.200037066	0.127981302
ENSG00000112078	*KCTD20*	6.152374737	-0.00974303	-0.065419089	0.055676059	0.999933887	1.126465074	1.458337203	-0.331872129	0.127981302
ENSG00000170275	*CRTAP*	3.868689879	0.049477115	-0.024640183	0.074117298	0.999933887	-0.116137541	-0.369160585	0.253023045	0.128068898
ENSG00000276570	*AC010327.6*	1.442659056	0.067847396	0.279970729	-0.212123334	0.999933887	-0.501029183	-0.894215068	0.393185885	0.128068898
ENSG00000255284	*AP006621.3*	-0.121487646	0.106587987	-0.220393247	0.326981233	0.999933887	-0.272908853	-0.837503993	0.56459514	0.128068898
ENSG00000132274	*TRIM22*	7.514967907	0.024560744	-0.01671227	0.041273014	0.999933887	-0.131559058	-0.299569799	0.168010741	0.128068898
ENSG00000157106	*SMG1*	7.837289213	-0.022713456	-0.071597924	0.048884468	0.999933887	-0.167742153	0.162921092	-0.330663245	0.128068898
ENSG00000120318	*ARAP3*	7.222514552	-0.020525293	0.071188324	-0.091713617	0.999933887	-0.591895063	-0.792481674	0.200586611	0.128201787
ENSG00000186529	*CYP4F3*	7.467335148	-0.044079009	0.036661131	-0.08074014	0.999933887	-0.178479239	-0.294710877	0.116231638	0.128330097
ENSG00000140299	*BNIP2*	6.602111143	-0.079895771	-0.041637799	-0.038257973	0.999933887	-0.396052492	-0.197797665	-0.198254827	0.128426789
ENSG00000005075	*POLR2J*	4.645984888	0.057589283	0.048694112	0.008895171	0.999933887	-0.105656032	-0.27287402	0.167217988	0.128426789
ENSG00000281357	*ARRDC3-AS1*	1.625839308	-0.141415272	-0.156059867	0.014644595	0.999933887	0.38745389	0.670640445	-0.283186555	0.128436425
ENSG00000167904	*TMEM68*	1.335502744	-0.054201772	0.003582249	-0.057784022	0.999933887	0.464231722	0.840326592	-0.37609487	0.128436425
ENSG00000172466	*ZNF24*	6.955472574	-0.108054665	-0.164950182	0.056895517	0.999933887	0.195595694	0.388549736	-0.192954042	0.128480911
ENSG00000134049	*IER3IP1*	0.325758836	-0.2228851	0.413371991	-0.636257091	0.944094537	-0.142969908	0.429828666	-0.572798574	0.128480911
ENSG00000160014	*CALM3*	7.205837862	0.058209273	0.005671651	0.052537621	0.999933887	0.071695922	0.212750451	-0.141054529	0.128480911
ENSG00000118965	*WDR35*	2.11696405	-0.126302274	-0.178937256	0.052634981	0.999933887	0.040962465	0.366148594	-0.325186129	0.128480911
ENSG00000196456	*ZNF775*	1.048743641	0.07027043	0.104832203	-0.034561772	0.999933887	0.844170621	1.218512309	-0.374341688	0.128517344
ENSG00000196182	*STK40*	9.47251234	0.013898282	0.001221273	0.012677009	0.999933887	-0.058162395	0.064450965	-0.12261336	0.128568144
ENSG00000169992	*NLGN2*	-0.030924254	-0.094417408	0.240788258	-0.335205665	0.999933887	-0.101034346	0.559695039	-0.660729385	0.12872819
ENSG00000126749	*EMG1*	2.315759619	-0.034145299	-0.008601703	-0.025543596	0.999933887	0.1865438	0.455109489	-0.268565689	0.12872819
ENSG00000135336	*ORC3*	2.216645271	-0.021781226	-0.146310998	0.124529771	0.999933887	0.023759642	0.338523624	-0.314763982	0.12872819
ENSG00000070444	*MNT*	6.319272009	0.007264432	-0.006902931	0.014167363	0.999933887	-0.583014427	-0.739634679	0.156620252	0.12872819
ENSG00000101182	*PSMA7*	6.982103095	0.006989953	-0.023724707	0.03071466	0.999933887	-0.176552451	-0.324997166	0.148444716	0.12872819
ENSG00000124198	*ARFGEF2*	5.193353861	-0.042238716	-0.115850947	0.073612231	0.999933887	-0.108574721	0.050602463	-0.159177184	0.128743221
ENSG00000177575	*CD163*	3.447776666	0.129118925	0.136399039	-0.007280114	0.999933887	-0.864948989	-1.177190594	0.312241605	0.128796951
ENSG00000138378	*STAT4*	4.011873062	-0.063358556	-0.156763356	0.0934048	0.999933887	0.201896809	0.435352974	-0.233456165	0.128965966
ENSG00000165898	*ISCA2*	1.786777652	-0.114842049	-0.01228848	-0.102553569	0.999933887	-0.324208396	-0.672525975	0.348317579	0.129040936
ENSG00000170242	*USP47*	4.737544032	-0.078778362	-0.080642656	0.001864294	0.999933887	-0.125232609	0.278616663	-0.403849272	0.129040936
ENSG00000198959	*TGM2*	1.000979611	0.07462047	0.207658658	-0.133038188	0.999933887	-0.018518915	0.526096898	-0.544615814	0.129040936
ENSG00000139597	*N4BP2L1*	3.130795266	-0.069874303	-0.07444161	0.004567307	0.999933887	-0.021194802	0.249085831	-0.270280633	0.129082391
ENSG00000085872	*CHERP*	5.863898734	0.033087862	-0.002774038	0.0358619	0.999933887	0.267299987	0.453212586	-0.185912599	0.129252705
ENSG00000004059	*ARF5*	6.134953715	-0.009490695	0.010321079	-0.019811774	0.999933887	-0.211317829	-0.402081814	0.190763985	0.129318773
ENSG00000169413	*RNASE6*	2.512410198	-0.020035459	-0.001971856	-0.018063603	0.999933887	-0.419305668	-1.111567927	0.692262259	0.12944918
ENSG00000092098	*RNF31*	1.292182792	0.002828231	0.076500739	-0.073672508	0.999933887	-0.675605442	-1.115729066	0.440123624	0.129464244
ENSG00000263766	*AC025682.1*	0.126890341	0.068979682	0.079464278	-0.010484596	0.999933887	0.653170609	1.285239328	-0.632068719	0.129482371
ENSG00000186501	*TMEM222*	4.734278283	-0.022023911	-0.001530502	-0.020493409	0.999933887	-0.395459069	-0.564842142	0.169383072	0.129650883
ENSG00000175938	*ORAI3*	3.551547386	-0.104640299	-0.014881632	-0.089758666	0.999933887	-0.899234623	-1.149974915	0.250740292	0.129668394
ENSG00000130811	*EIF3G*	5.430054565	0.014017152	-0.026430214	0.040447366	0.999933887	-0.020612994	-0.186449657	0.165836662	0.129745363
ENSG00000254777	*AC022182.1*	1.19997669	0.102202167	0.202567937	-0.10036577	0.999933887	0.176484111	-0.335238586	0.511722698	0.129815573
ENSG00000137393	*RNF144B*	6.344176609	-0.231192418	-0.080807504	-0.150384914	0.999933887	2.547298785	2.935105338	-0.387806552	0.129868743
ENSG00000167637	*ZNF283*	-0.1826437	0.251031366	-0.156016155	0.407047522	0.999933887	-0.372553059	0.091083127	-0.463636186	0.129868743
ENSG00000226711	*FAM66C*	-0.401006565	-0.005206181	0.137440296	-0.142646477	0.999933887	0.29638198	0.97268069	-0.676298711	0.129868743
ENSG00000262879	*AC005670.3*	1.45184196	-0.0090604	0.066230175	-0.075290576	0.999933887	-0.335893239	-0.618466204	0.282572965	0.129879904
ENSG00000106290	*TAF6*	2.51076887	-0.061584097	0.047165391	-0.108749488	0.999933887	0.075831968	-0.159676219	0.235508186	0.129889885
ENSG00000287494	*AC069368.2*	2.852478223	0.107962457	-0.010893156	0.118855613	0.999933887	1.043433935	1.375075108	-0.331641173	0.129917072
ENSG00000217555	*CKLF*	-0.140377018	-0.259004946	-0.255373284	-0.003631662	0.999933887	-0.969873655	-1.80103026	0.831156605	0.129948683
ENSG00000177225	*GATD1*	3.408721708	-0.084639268	0.015407357	-0.100046625	0.999933887	-0.328638921	-0.553911912	0.225272991	0.129948683
ENSG00000188185	*LINC00265*	1.275627006	-0.136774235	0.082228708	-0.219002943	0.999933887	0.256357713	0.612075579	-0.355717867	0.129998969
ENSG00000118922	*KLF12*	4.009126158	-0.079427935	-0.185924413	0.106496478	0.999933887	-0.285724475	0.105525235	-0.39124971	0.130203859
ENSG00000205084	*TMEM231*	2.217942222	0.059242963	0.006059831	0.053183132	0.999933887	0.860286337	1.122932844	-0.262646507	0.130203859
ENSG00000165689	*ENTR1*	3.052230359	0.031675917	-0.060728955	0.092404873	0.999933887	-0.077597332	-0.321567401	0.243970069	0.130203859
ENSG00000111860	*CEP85L*	3.862350065	-0.015257309	-0.099674258	0.084416949	0.999933887	-0.650911752	-1.112249971	0.461338218	0.130203859
ENSG00000153391	*INO80C*	2.279743937	-0.1182441	0.045381886	-0.163625986	0.999933887	0.367753567	0.659861362	-0.292107795	0.13023857
ENSG00000095397	*WHRN*	4.327268714	0.095328438	0.039772355	0.055556083	0.999933887	0.321990874	0.543339328	-0.221348454	0.13023857
ENSG00000228223	*HCG11*	0.926832871	0.045455666	0.225114179	-0.179658512	0.999933887	-0.397516275	-0.750413033	0.352896758	0.13023857
ENSG00000079393	*DUSP13*	1.247732767	-0.023959064	0.241034002	-0.264993066	0.999933887	-1.160476406	-1.514536477	0.354060071	0.13023857
ENSG00000114378	*HYAL1*	-0.008695784	-0.120102016	-0.281284862	0.161182846	0.999933887	-0.532431315	-1.046365268	0.513933953	0.130261133
ENSG00000143061	*IGSF3*	-1.754068161	0.034270158	-0.223967682	0.258237841	0.999933887	3.101752406	4.086322624	-0.984570218	0.130261133
ENSG00000163472	*TMEM79*	1.937069025	-0.007880689	-0.270868884	0.262988195	0.999933887	-0.379116009	-0.660534122	0.281418113	0.130341498
ENSG00000169905	*TOR1AIP2*	5.79741532	-0.04365056	-0.028565437	-0.015085123	0.999933887	-0.465069014	-0.2812242	-0.183844813	0.130649991
ENSG00000150527	*MIA2*	4.667996678	-0.03857174	-0.096897144	0.058325405	0.999933887	-0.687501004	-0.898133249	0.210632245	0.130686288
ENSG00000177125	*ZBTB34*	5.388058924	-0.124174401	0.025661432	-0.149835833	0.999933887	-1.023734534	-1.310041349	0.286306815	0.130810051
ENSG00000228526	*MIR34AHG*	-1.620714339	-0.202798565	0.270849776	-0.473648341	0.999933887	-0.307907815	0.257916829	-0.565824644	0.131129847
ENSG00000022976	*ZNF839*	2.808647423	0.087161946	0.059143852	0.028018094	0.999933887	0.096941208	-0.128638379	0.225579587	0.131190484
ENSG00000177239	*MAN1B1*	4.929367383	0.026382565	-0.003870931	0.030253496	0.999933887	-0.028784425	-0.189920648	0.161136223	0.131190484
ENSG00000151176	*PLBD2*	2.733617583	0.03609334	-0.128847719	0.164941058	0.999933887	-0.464077119	-0.700728854	0.236651735	0.131190484
ENSG00000249096	*LINC02362*	4.090340097	0.015130344	0.049510166	-0.034379822	0.999933887	0.690147375	0.947720026	-0.25757265	0.131190484
ENSG00000138750	*NUP54*	2.855109564	-0.00565533	-0.188075765	0.182420434	0.999933887	-0.113848406	0.145714965	-0.259563371	0.131190484
ENSG00000149930	*TAOK2*	5.206888725	0.029454027	0.057917798	-0.028463771	0.999933887	-0.220090486	-0.400636585	0.180546099	0.131251106
ENSG00000214595	*EML6*	1.563589044	-0.000967813	0.220865923	-0.221833736	0.999933887	0.041305825	0.561891586	-0.520585761	0.131251106
ENSG00000080822	*CLDND1*	5.283815571	-0.05847197	-0.089517573	0.031045602	0.999933887	-0.031008577	0.159059362	-0.190067939	0.131289285
ENSG00000196668	*LINC00173*	4.494682693	-0.155259386	-0.072016712	-0.083242674	0.999933887	-0.488322951	-0.699938851	0.2116159	0.13207783
ENSG00000138398	*PPIG*	5.421675251	-0.079879968	-0.221649562	0.141769594	0.999933887	-0.523584135	-0.147047276	-0.376536859	0.13207783
ENSG00000244459	*AC147067.1*	-0.460760805	0.076370873	-0.143870462	0.220241335	0.999933887	-0.318092708	-1.114947731	0.796855022	0.132181639
ENSG00000183784	*DOCK8-AS1*	0.521218348	0.182086111	0.037623889	0.144462222	0.999933887	-0.813124734	-1.620885336	0.807760602	0.13227524
ENSG00000124831	*LRRFIP1*	8.219934114	0.007229309	-0.073483002	0.08071231	0.999933887	-0.574306603	-0.826329226	0.252022622	0.13227524
ENSG00000134285	*FKBP11*	3.253046772	-0.038851852	-0.059054592	0.02020274	0.999933887	-0.064102248	-0.242348348	0.1782461	0.132463306
ENSG00000241489	*AC244197.3*	1.47965341	-0.028900347	-0.077159448	0.048259101	0.999933887	-0.270904923	-0.615515424	0.344610501	0.132463306
ENSG00000099139	*PCSK5*	0.804771807	0.167716005	-0.167701715	0.335417721	0.944094537	-0.202030417	0.2582565	-0.460286918	0.132528275
ENSG00000232811	*AL360270.1*	-0.118326532	0.134924699	0.199995477	-0.065070777	0.999933887	-0.301183044	0.217755331	-0.518938375	0.132528275

ENSG00000160993	*ALKBH4*	2.759762912	0.026086163	0.019040244	0.007045919	0.999933887	0.03462345	-0.258098171	0.292721621	0.132528275
ENSG00000058673	*ZC3H11A*	0.581727556	-0.016962795	-0.122779754	0.105816958	0.999933887	0.362222023	0.734030447	-0.371808423	0.132528275
ENSG00000163703	*CRELD1*	0.077445357	-0.178625614	0.083381793	-0.262007407	0.999933887	-0.390302127	-0.77305136	0.382749233	0.132773397
ENSG00000262089	*AC040977.1*	3.26622783	-0.136735128	0.001998743	-0.138733872	0.999933887	0.112095524	0.306352903	-0.194257379	0.132801888
ENSG00000260630	*SNAI3-AS1*	1.721312188	0.142419484	0.338820218	-0.196400734	0.999933887	0.674076767	1.005096892	-0.331020125	0.132801888
ENSG00000271032	*AC020907.4*	1.244271949	0.05109105	0.274306228	-0.223215177	0.999933887	2.840051532	3.331257289	-0.491205757	0.132801888
ENSG00000135636	*DYSF*	8.336059033	-0.00099744	0.092194126	-0.093191565	0.999933887	-0.179602329	-0.40289625	0.223293922	0.132801888
ENSG00000242086	*MUC20-OT1*	3.177317491	-0.035124684	0.096695623	-0.131820307	0.999933887	-0.153408965	0.068644259	-0.222053224	0.13307667
ENSG00000279884	*AC010894.4*	0.430662464	-0.213933738	-0.117219786	-0.096713952	0.999933887	-0.661346978	-1.346496939	0.685149961	0.133161831
ENSG00000237491	*LINC01409*	0.428551243	-0.100966491	-0.073270002	-0.027696489	0.999933887	-0.213553223	0.261372174	-0.474925398	0.133394046
ENSG00000244879	*GABPB1-AS1*	3.902909026	-0.089790565	-0.026376115	-0.063414451	0.999933887	0.086333141	0.266261328	-0.179928186	0.133568236
ENSG00000198176	*TFDP1*	5.884116711	-0.048715423	0.024717465	-0.073432889	0.999933887	0.639754473	0.817893137	-0.178138664	0.133568236
ENSG00000198053	*SIRPA*	9.537137177	-0.015404544	-0.033628563	0.018224019	0.999933887	0.412521106	0.576382536	-0.163861431	0.133568236
ENSG00000100280	*AP1B1*	4.815556136	0.01552863	0.024626652	-0.009098022	0.999933887	0.316862162	0.48682299	-0.169960828	0.133621717
ENSG00000154548	*SRSF12*	1.626549311	-0.003664021	0.073657774	-0.077321795	0.999933887	0.750611589	1.066516513	-0.315904924	0.133672733
ENSG00000121775	*TMEM39B*	1.719513883	0.090714883	0.028406413	0.06230847	0.999933887	-0.007592861	-0.369502795	0.361909933	0.133740731
ENSG00000163155	*LYSMD1*	0.916708229	-0.16957122	-0.07838524	-0.09118598	0.999933887	-0.419881611	-0.834178323	0.414296712	0.133891865
ENSG00000120051	*CFAP58*	2.537757996	-0.113549425	-0.163201775	0.049652349	0.999933887	1.019468645	1.346311234	-0.326842589	0.133891865
ENSG00000014919	*COX15*	2.313961564	-0.182206293	-0.109104375	-0.073101918	0.999933887	-0.247105816	-0.477306997	0.23020118	0.133938626
ENSG00000185252	*ZNF74*	0.598594202	0.022712594	0.147804756	-0.125092163	0.999933887	0.158724787	0.502844531	-0.344119744	0.133938626
ENSG00000186810	*CXCR3*	2.885020942	-0.032441074	-0.074117189	0.041676115	0.999933887	0.156949996	0.395798898	-0.238848902	0.134000893
ENSG00000163961	*RNF168*	5.254341029	-0.132657266	-0.025044729	-0.107612537	0.999933887	-0.194671517	-0.348456343	0.153784826	0.134046448
ENSG00000261094	*AC007066.2*	1.143656285	-0.041258654	-0.156592505	0.115333851	0.999933887	0.844841621	1.171544408	-0.326702786	0.134046448
ENSG00000236675	*MTX1P1*	3.118909719	0.011688455	-0.074104512	0.085792967	0.999933887	-0.43062122	-0.678542084	0.247920864	0.134053369
ENSG00000160255	*ITGB2*	9.443084137	0.044067352	0.000313822	0.043753531	0.999933887	-0.188274821	-0.359416227	0.171141406	0.134081898
ENSG00000156052	*GNAQ*	6.245718581	0.02670285	0.015869573	0.010833277	0.999933887	-0.615772998	-0.856830784	0.241057786	0.13414887
ENSG00000143498	*TAF1A*	-0.368117839	-0.285792875	0.430676588	-0.716469462	0.944094537	0.03700242	0.58089257	-0.54389015	0.134169863
ENSG00000185621	*LMLN*	0.53395075	-0.120001583	-0.03594989	-0.084051693	0.999933887	-0.210158998	0.24051623	-0.450675229	0.134169863
ENSG00000280332	*AC020917.4*	3.153820298	0.052518208	0.045101756	0.007416451	0.999933887	-0.021097579	0.183852427	-0.204950005	0.134227808
ENSG00000112715	*VEGFA*	10.559120951	0.180800346	0.244035953	-0.063235607	0.999933887	0.523144879	0.720675023	-0.197530144	0.13439979
ENSG00000110274	*CEP164*	4.367150057	0.046245247	0.05550337	-0.009258123	0.999933887	-0.054729858	-0.223290555	0.168560697	0.134556605
ENSG00000029153	*ARNTL2*	-1.167599415	-0.489639976	-0.427815317	-0.06182466	0.999933887	0.215859399	0.861454478	-0.645595079	0.134775773
ENSG00000168291	*PDHB*	2.522311904	0.024566792	0.052609009	-0.028042217	0.999933887	-0.07900096	-0.307192759	0.2281918	0.134845697
ENSG00000111653	*ING4*	4.07438491	-0.113018279	-0.067582746	-0.045435533	0.999933887	-0.310369486	-0.499044645	0.18867516	0.13493934
ENSG00000221817	*PPP3CB-AS1*	0.978100848	-0.026642791	-0.004655369	-0.021987421	0.999933887	-0.111647192	0.220520539	-0.332167731	0.13493934
ENSG00000115827	*DCAF17*	1.595629323	0.010869541	-0.133621004	0.144490545	0.999933887	-0.176933624	0.129828355	-0.306761979	0.135125876
ENSG00000130222	*GADD45G*	3.985131317	-0.086415047	-0.149488632	0.063073586	0.999933887	-0.122620507	-0.489115461	0.366494954	0.135467069
ENSG00000087074	*PPP1R15A*	11.72942688	0.369578552	0.351393575	0.018184977	0.999933887	1.579993507	1.866464287	-0.28647078	0.13547322
ENSG00000178026	*LRRC75B*	2.853921439	0.111005281	0.099493297	0.011511985	0.999933887	-0.361319219	-0.107926449	-0.25339277	0.135578709
ENSG00000156735	*BAG4*	2.592036828	0.028911164	0.073369356	-0.044458192	0.999933887	-0.475047909	-0.799809296	0.324761387	0.135578709
ENSG00000236977	*ANKRD44-IT1*	1.237834319	0.069805865	0.05438269	0.015423174	0.999933887	-0.241670038	-0.775766931	0.534096893	0.135578876
ENSG00000159110	*IFNAR2*	2.229065544	-0.154876879	-0.074713303	-0.080163576	0.999933887	-0.175650049	-0.532496883	0.356846835	0.135739602
ENSG00000108528	*SLC25A11*	4.009575119	0.071485044	0.03035484	0.041130204	0.999933887	0.087189182	-0.100246048	0.18743523	0.135739602
ENSG00000130303	*BST2*	4.281188282	0.125738856	0.059083924	0.066654931	0.999933887	-0.22738765	-0.536007063	0.308619413	0.135861121
ENSG00000253653	*AC009185.1*	0.652740873	0.040184357	0.043989104	-0.003804747	0.999933887	-0.412761856	0.080322248	-0.493084104	0.13608266
ENSG00000164978	*NUDT2*	0.934040835	-0.065567815	0.053728723	-0.119296538	0.999933887	-0.036011281	-0.417964934	0.381953653	0.136363487
ENSG00000187824	*TMEM220*	-0.430853442	-0.119705049	-0.10074403	-0.018961019	0.999933887	-0.36944169	0.306913974	-0.676355664	0.136482813
ENSG00000092871	*RFFL*	6.074820896	-0.021982877	0.029310392	-0.051293269	0.999933887	0.965651102	1.162230794	-0.196579691	0.136731506
ENSG00000156697	*UTP14A*	1.724496752	-0.24934154	-0.140156482	-0.109185058	0.999933887	0.01952046	0.285520463	-0.266000003	0.136970844
ENSG00000095906	*NUBP2*	3.35565564	0.066795627	0.10735296	-0.040557333	0.999933887	0.0158313	-0.226631603	0.242462904	0.136970844
ENSG00000136891	*TEX10*	4.342956417	-0.057716566	0.059144951	-0.116861517	0.999933887	0.100145809	0.280509766	-0.180363958	0.136970844
ENSG00000107566	*ERLIN1*	4.859993219	-0.04306147	-0.049077707	0.006016237	0.999933887	0.04741962	0.283191047	-0.235771427	0.136970844
ENSG00000100949	*RABGGTA*	4.878714127	0.042845769	0.076789911	-0.033944142	0.999933887	0.736451782	0.929057931	-0.19260615	0.136970844
ENSG00000151650	*VENTX*	1.262636503	0.107422569	-0.010792323	0.118214892	0.999933887	0.016788799	0.372694735	-0.355905936	0.13726186
ENSG00000119669	*IRF2BPL*	4.530304604	0.058959834	0.029292896	0.029666938	0.999933887	-0.101066683	-0.338145823	0.23707914	0.13726186
ENSG00000105355	*PLIN3*	5.947348757	0.051226435	0.002892852	0.048333583	0.999933887	-0.067646016	0.159865303	-0.22751132	0.13726186
ENSG00000184840	*TMED9*	5.044349752	-0.01630641	-0.008957138	-0.007349272	0.999933887	-0.307658168	-0.525526332	0.217868163	0.13726186
ENSG00000100726	*TELO2*	3.469145562	0.109455638	0.126656443	-0.017200805	0.999933887	-0.00530964	-0.237955581	0.232645941	0.137408886
ENSG00000149084	*HSD17B12*	2.763426408	0.15395625	0.01076256	0.14319369	0.999933887	-0.223390694	0.018693066	-0.24208376	0.137572723
ENSG00000125633	*CCDC93*	7.468202066	-0.064488947	-0.126792732	0.062303785	0.999933887	-0.018412677	0.147996141	-0.166408818	0.137572723
ENSG00000121644	*DESI2*	3.40390332	-0.03247766	-0.03994517	0.00746751	0.999933887	-0.336551392	-0.539016416	0.202465024	0.137572723
ENSG00000144224	*UBXN4*	7.536896441	-0.031166283	-0.101720446	0.070554162	0.999933887	-0.412422082	-0.184675168	-0.227746914	0.137572723
ENSG00000146192	*FGD2*	1.28033119	0.014145002	0.142488132	-0.12834313	0.999933887	0.081510578	0.425173841	-0.343663263	0.137572723
ENSG00000273133	*AC116651.1*	1.292931858	-0.01739245	0.324931345	-0.342323796	0.944094537	0.050485044	0.473584405	-0.42309936	0.137625439
ENSG00000138175	*ARL3*	2.12508656	0.023309243	0.130862622	-0.107553379	0.999933887	-0.604371398	-0.954080423	0.349709025	0.137694325
ENSG00000123091	*RNF11*	6.679097485	0.0338894	0.010617584	0.023271816	0.999933887	0.011124579	0.157222128	-0.146097549	0.137855401
ENSG00000165118	*C9orf64*	0.217458789	-0.11995271	-0.070592968	-0.049359741	0.999933887	-0.295135204	-0.676894508	0.381759304	0.13787568
ENSG00000170236	*USP50*	0.820689404	-0.09287602	-0.092911563	3.5543e-05	0.999933887	-0.213492722	0.305512286	-0.519005009	0.13787568
ENSG00000198346	*ZNF813*	-0.208523807	-0.044946905	-0.194728469	0.149781565	0.999933887	-0.332980687	0.210135546	-0.543116233	0.13787568
ENSG00000187479	*C11orf96*	1.670980594	0.288223575	0.626835928	-0.338612353	0.999933887	2.049082065	2.369273385	-0.320191321	0.137974392
ENSG00000072840	*EVC*	-0.00882689	0.112292717	-0.007704542	0.119997259	0.999933887	-0.202814783	0.347566484	-0.550381267	0.137974392
ENSG00000237550	*AC243919.1*	3.029823346	-0.042830438	-0.068326861	0.025496423	0.999933887	0.148484845	0.398641822	-0.250156978	0.137974392
ENSG00000249859	*PVT1*	2.075569112	-0.085472112	0.125409826	-0.210881937	0.999933887	0.101008209	0.36358493	-0.262576721	0.138120408
ENSG00000169955	*ZNF747*	0.568794224	-0.145202388	0.013244778	-0.158447165	0.999933887	-0.091964092	-0.540504259	0.448540167	0.138207123
ENSG00000149792	*MRPL49*	3.412825928	-0.028966385	0.053115627	-0.082082013	0.999933887	-0.081335407	-0.281234733	0.199899327	0.138210925
ENSG00000213341	*CHUK*	5.338522925	-0.022238511	0.034564484	-0.056802995	0.999933887	-0.152552167	0.04576285	-0.198315017	0.13828656
ENSG00000174885	*NLRP6*	6.051756986	-0.002701213	-0.017378865	0.014677652	0.999933887	-2.145375254	-2.550280354	0.404905099	0.138303754
ENSG00000115271	*GCA*	7.274958247	-0.024516002	-0.017806509	-0.006709493	0.999933887	-0.183886009	-0.380285335	0.196399326	0.138357057
ENSG00000129355	*CDKN2D*	6.103489354	0.222234888	0.258276815	-0.036041927	0.999933887	-0.060177913	-0.343737096	0.283559183	0.138360342
ENSG00000120458	*MSANTD2*	0.778770198	-0.180253456	-0.036516582	-0.143736874	0.999933887	0.270959552	-0.131291986	0.402251538	0.13838791
ENSG00000102796	*DHRS12*	2.84528177	0.059090423	-0.069443828	0.128534252	0.999933887	0.553741362	0.816240232	-0.26249887	0.138447819
ENSG00000102292	*NCAPD2*	4.197328252	-0.018446233	-0.026692249	0.008246016	0.999933887	-0.440799984	-0.686178219	0.245378235	0.138544243
ENSG00000248476	*BACH1-IT1*	2.68500061	-0.049362959	-0.010893208	-0.038469751	0.999933887	-0.043537239	0.189939241	-0.23347648	0.138899699
ENSG00000170144	*HNRNPA3*	8.183357398	0.002436671	0.016886056	-0.014449385	0.999933887	0.307564585	0.479601218	-0.172036633	0.138899699
ENSG00000162910	*MRPL55*	2.668379198	0.062761571	0.062721485	4.0086e-05	0.999933887	0.104383784	-0.164209669	0.268593453	0.139029983
ENSG00000121486	*TRMT1L*	3.344901711	-0.067208911	0.005010852	-0.072219763	0.999933887	-0.038802414	0.139674397	-0.178476811	0.139029983
ENSG00000182240	*BACE2*	-0.089471435	0.008355684	-0.152016197	0.160371881	0.999933887	-0.270521127	0.302243467	-0.572764593	0.139029983
ENSG00000179523	*EIF3J-DT*	1.597223008	0.053589263	-0.135621326	0.189210589	0.999933887	-0.356272678	-0.684002717	0.32773004	0.13909779
ENSG00000070731	*ST6GALNAC2*	3.615279447	0.064685445	0.048900409	0.015785036	0.999933887	-0.781470094	-1.038267088	0.256796993	0.139108408
ENSG00000120733	*KDM3B*	6.923641358	-0.027510556	0.012336563	-0.039847119	0.999933887	-0.638390923	-0.814748837	0.176357915	0.139108408
ENSG00000015153	*YAF2*	4.055061239	-0.018966933	-0.007542441	-0.011424492	0.999933887	0.089429877	0.255382619	-0.165952742	0.139119391
ENSG00000198938	*MT-CO3*	9.470977802	0.085656512	0.005170785	0.080485727	0.999933887	0.045109932	0.325128598	-0.280018666	0.139285365
ENSG00000215908	*CROCCP2*	4.246040413	0.05691688	0.097875882	-0.040959002	0.999933887	0.050126002	-0.135854151	0.185980153	0.139285365
ENSG00000183718	*TRIM52*	3.19516821	-0.042089318	-0.021922909	-0.020166408	0.999933887	-0.215757944	-0.419978094	0.20422015	0.139285365
ENSG00000089048	*ESF1*	1.402579167	-0.276099591	-0.245610531	-0.03048906	0.999933887	-0.215096903	0.209883233	-0.424980136	0.139450653
ENSG00000100815	*TRIP11*	3.298431415	-0.132631488	-0.103139318	-0.029492171	0.999933887	-0.356319975	0.017833862	-0.374153837	0.139525219
ENSG00000268043	*NBPF12*	3.144927478	-0.076392259	-0.132762654	0.056370395	0.999933887	-0.008342069	0.229181256	-0.237523325	0.139525219
ENSG00000111731	*C2CD5*	3.970800127	-0.064583867	-0.000732726	-0.06385114	0.999933887	0.037422804	0.318353069	-0.280930265	0.139525219
ENSG00000134508	*CABLES1*	0.529093955	-0.040736731	0.00526158	-0.045998311	0.999933887	0.163122622	0.515561115	-0.352438493	0.139525219
ENSG00000002330	*BAD*	1.785394999	0.012887839	0.158137998	-0.145250159	0.999933887	0.026734962	-0.308241154	0.334976116	0.139525219
ENSG00000273344	*PAXIP1-AS1*	1.242121707	0.015538826	0.217480854	-0.201942028	0.999933887	0.509493186	0.88203889	-0.372545704	0.139525219
ENSG00000105829	*BET1*	0.985429567	-0.00715525	-0.019178129	0.012022879	0.999933887	0.035055451	0.390822612	-0.355767162	0.139525219
ENSG00000158636	*EMSY*	4.514410906	0.00285356	-0.073427114	0.076280674	0.999933887	-0.427615056	-0.19776106	-0.229853995	0.139525219
ENSG00000241370	*RPP21*	-0.111392783	0.147626299	0.217832003	-0.070205704	0.999933887	0.320843355	-0.221673794	0.542517149	0.139639796
ENSG00000244036	*AC073320.1*	-0.070612438	0.09416649	0.110311695	-0.016145205	0.999933887	0.526425215	1.027649447	-0.501224232	0.139639796

ENSG00000188342	*GTF2F2*	2.783457724	0.009636157	-0.076887162	0.086523319	0.999933887	-0.000302815	0.215642746	-0.215945561	0.139808268
ENSG00000092931	*MFSD11*	4.182747901	0.069309247	0.184215983	-0.114906736	0.999933887	0.517515846	0.695877651	-0.178361805	0.140112267
ENSG00000163161	*ERCC3*	3.990156306	0.037421372	-0.064009245	0.101430617	0.999933887	0.008907647	-0.14781382	0.156721467	0.140112267
ENSG00000184381	*PLA2G6*	4.521988687	0.13611294	0.1686793	-0.03256636	0.999933887	0.757773159	0.975993013	-0.218219853	0.140157435
ENSG00000183844	*FAM3B*	-1.163815916	0.165891311	-0.131960912	0.297852223	0.999933887	-0.056525824	-0.778898335	0.722372511	0.140162169
ENSG00000169223	*LMAN2*	6.053745775	0.024797787	-0.02343405	0.048231837	0.999933887	-0.194908622	-0.349323414	0.154414792	0.140393419
ENSG00000183309	*ZNF623*	2.278664015	0.040652844	-0.035743079	0.076395923	0.999933887	-0.298687389	-0.556003283	0.257315894	0.140437846
ENSG00000130856	*ZNF236*	3.255730603	0.052689342	-0.028041636	0.080730978	0.999933887	-0.310534777	-0.491393226	0.180858449	0.140461428
ENSG00000287878	*AC022382.2*	3.603950793	-0.026291515	0.07769475	-0.103986265	0.999933887	0.078205865	0.319185582	-0.240979717	0.140496983
ENSG00000033178	*UBA6*	4.595281828	-0.059054887	-0.067606604	0.008551717	0.999933887	-0.286130633	0.124732986	-0.410863619	0.14049984
ENSG00000115718	*PROC*	0.652521018	-0.187141105	-0.187793303	0.000652198	0.999933887	-1.919772235	-2.525106684	0.605334448	0.140776085
ENSG00000117020	*AKT3*	3.037839795	-0.040493895	0.022623215	-0.06311711	0.999933887	0.041009229	0.327079244	-0.286070015	0.140803705
ENSG00000203872	*C6orf163*	0.240677534	0.105640513	-0.148564447	0.25420496	0.999933887	-1.624021617	-2.193559335	0.569537719	0.141025084
ENSG00000127578	*WFIKKN1*	-0.516718571	0.049033548	0.160003165	-0.110969617	0.999933887	0.050636287	-0.579428436	0.630064723	0.141209706
ENSG00000266028	*SRGAP2*	4.078647114	0.00138314	0.071910609	-0.070527469	0.999933887	-0.082496031	0.149148191	-0.231644222	0.141263664
ENSG00000111424	*VDR*	6.248811632	0.003266135	0.063274755	-0.06000862	0.999933887	0.070512989	-0.106221261	0.176734249	0.141362506
ENSG00000182010	*RTKN2*	0.374971205	0.164051911	-0.419039941	0.583091852	0.999933887	0.263071928	0.698920686	-0.435848758	0.141588505
ENSG00000074696	*HACD3*	1.75368762	0.048560389	-0.215486903	0.264047292	0.999933887	0.038085522	0.367996381	-0.329910859	0.141648559
ENSG00000287631	*AC005071.1*	2.169389223	-0.246314285	-0.043004352	-0.203309933	0.999933887	2.34995168	2.875744035	-0.525792355	0.141858036
ENSG00000139514	*SLC7A1*	4.037865879	0.043440765	-0.068128643	0.111569408	0.999933887	0.282929013	0.473433564	-0.190504551	0.14202508
ENSG00000139278	*GLIPR1*	5.573927046	-0.009848429	-0.123417721	0.113569292	0.999933887	-0.386118521	-0.657704453	0.271585931	0.142070682
ENSG00000214765	*SEPTIN7P2*	2.961335468	-0.007914022	-0.00432555	-0.003588472	0.999933887	0.115026343	0.380367207	-0.265340863	0.142072279
ENSG00000106952	*TNFSF8*	5.499256268	0.00657426	-0.017911786	0.024486046	0.999933887	0.081168354	0.27676141	-0.195593056	0.142072279
ENSG00000232254	*CSF2RBP1*	-0.727710449	-0.080689636	0.315802856	-0.396492492	0.999933887	0.159510485	-0.849090091	1.008600576	0.142240927
ENSG00000256594	*AC010186.2*	1.889345922	0.020229086	0.059739717	-0.039510631	0.999933887	0.563297136	0.857732322	-0.294435186	0.142240927
ENSG00000127995	*CASD1*	2.00833274	-0.078438879	-0.226344942	0.147906063	0.999933887	-0.271708117	0.056394073	-0.32810219	0.142246568
ENSG00000164010	*ERMAP*	1.710025654	0.069581676	-0.054790031	0.124371706	0.999933887	-0.153775595	0.151472161	-0.305247756	0.142246568
ENSG00000174945	*AMZ1*	-0.750762502	0.124316045	0.257487528	-0.133171483	0.999933887	0.22390381	0.823216977	-0.599313167	0.142246568
ENSG00000125686	*MED1*	4.799972147	-0.006422021	-0.149839645	0.143417623	0.999933887	0.064266688	0.226855404	-0.162588716	0.142246568
ENSG00000124523	*SIRT5*	2.008190978	-0.006365252	0.080846382	-0.087211634	0.999933887	-0.418568546	-0.653177671	0.234609125	0.142348562
ENSG00000157212	*PAXIP1*	2.261001357	-0.102291359	0.022900799	-0.125192158	0.999933887	0.381897285	0.729372266	-0.347474981	0.142623682
ENSG00000079385	*CEACAM1*	6.326475404	-0.019097549	0.020866054	-0.039963603	0.999933887	-0.087473895	0.122348469	-0.209822363	0.142840806
ENSG00000100567	*PSMA3*	5.142588473	-0.089563977	-0.126625221	0.037061245	0.999933887	-0.122771505	0.023649311	-0.146420816	0.142900798
ENSG00000204084	*INPP5B*	3.570999346	-0.007590236	0.006494841	-0.014085076	0.999933887	0.091157657	-0.077561866	0.168719523	0.142900798
ENSG00000186480	*INSIG1*	7.975238772	0.088006264	0.087347059	0.000659205	0.999933887	1.422541426	1.763653474	-0.341112048	0.142929833
ENSG00000130640	*TUBGCP2*	5.601491917	0.007855603	0.024189866	-0.016334262	0.999933887	-0.046689733	-0.170361318	0.123671585	0.142929833
ENSG00000105248	*YJU2*	3.319818632	-0.009832628	0.032594792	-0.04242742	0.999933887	0.261802996	0.525212966	-0.26340997	0.14332617
ENSG00000177732	*SOX12*	1.738215156	0.068117506	0.027807441	0.040310065	0.999933887	-0.188738824	-0.545519755	0.356780932	0.143390962
ENSG00000158882	*TOMM40L*	4.611589771	0.028147904	0.028062083	8.5821e-05	0.999933887	0.822277854	1.022693511	-0.200415657	0.143461402
ENSG00000065150	*IPO5*	3.575927972	-0.000722936	-0.123764308	0.123041373	0.999933887	0.354042396	0.564371644	-0.210329248	0.144197887
ENSG00000260051	*AL031600.1*	3.494174835	-0.010015434	-0.029159417	0.019143983	0.999933887	0.110086577	0.364303547	-0.254216971	0.144255914
ENSG00000118292	*C1orf54*	-0.147427492	0.026046142	0.00064285	0.025403291	0.999933887	-0.021760697	0.417806216	-0.439566913	0.144859094
ENSG00000178726	*THBD*	4.389051964	0.136350752	0.12240289	0.013947862	0.999933887	-1.863862586	-2.267850553	0.403987967	0.145069471
ENSG00000136156	*ITM2B*	10.528344329	-0.006818302	-0.028151972	0.02133367	0.999933887	-0.384752341	-0.548812471	0.16406013	0.145069471
ENSG00000163359	*COL6A3*	-0.362695309	-0.030113286	0.157382564	-0.18749585	0.999933887	-0.457138934	0.368675879	-0.825814813	0.145794852
ENSG00000272758	*AC083798.2*	1.106787324	-0.168285745	-0.053468978	-0.114816768	0.999933887	-0.139295351	0.203830901	-0.343126252	0.145812278
ENSG00000196655	*TRAPPC4*	3.666361242	-0.073657909	-0.033872414	-0.039785495	0.999933887	-0.077358215	-0.242745512	0.165387297	0.145812278
ENSG00000168488	*ATXN2L*	6.988827128	0.001896722	0.066737557	-0.064840835	0.999933887	0.050858064	0.173206353	-0.122348289	0.145812278
ENSG00000273243	*Z82243.1*	-0.388000008	-0.219778063	-0.042818919	-0.176959144	0.999933887	-0.954380154	-1.600775784	0.64639563	0.146062252
ENSG00000196705	*ZNF431*	3.10753668	-0.042565277	-0.199519841	0.156954564	0.999933887	-0.395219567	-0.057168074	-0.338051494	0.146077263
ENSG00000197037	*ZSCAN25*	3.358136008	0.061737111	0.051414846	0.010322264	0.999933887	-0.111075331	-0.345399747	0.234324416	0.146126903
ENSG00000149541	*B3GAT3*	3.985501211	0.081157829	-0.018922864	0.100080693	0.999933887	0.798830258	1.130114336	-0.331284078	0.146157273
ENSG00000135835	*KIAA1614*	0.220540814	0.031644886	-0.30459832	0.336243207	0.999933887	-0.525700533	-1.014482599	0.488782066	0.146157273
ENSG00000148840	*PPRC1*	3.821765316	-0.046108856	-0.026081642	-0.020027214	0.999933887	0.548949393	0.752013309	-0.203063916	0.146422222
ENSG00000067646	*ZFY*	-3.421724204	-0.159379079	0.051879907	-0.211258986	0.999933887	-0.051130051	0.444249078	-0.495379129	0.146438709
ENSG00000253320	*MAILR*	6.694393316	0.095042724	0.204637545	-0.109594821	0.999933887	3.71131949	4.21966943	-0.508349941	0.146870927
ENSG00000149346	*SLX4IP*	1.858564461	0.047212635	-0.09686463	0.144077265	0.999933887	0.63569404	0.984268058	-0.348574017	0.146870927
ENSG00000215267	*AKR1C7P*	0.75359138	0.177093798	-0.046449941	0.223543739	0.999933887	0.100644369	0.421256519	-0.32061215	0.147214245
ENSG00000173065	*FAM222B*	2.918427611	-0.006509284	-0.068266155	0.061756871	0.999933887	0.14005393	0.355245231	-0.2151913	0.147315614
ENSG00000261526	*AC012615.1*	1.277912123	-0.106910105	-0.032433863	-0.074476242	0.999933887	-0.855123582	-1.305235273	0.450111691	0.147484265
ENSG00000071242	*RPS6KA2*	3.116842561	-0.010720909	-0.017570746	0.006849837	0.999933887	-1.077911473	-1.384341998	0.306430525	0.147714616
ENSG00000112983	*BRD8*	3.914468345	-0.114571813	-0.111517193	-0.00305462	0.999933887	-0.351786509	-0.571751574	0.219965065	0.147724171
ENSG00000113649	*TCERG1*	3.728845496	-0.041451185	-0.101912495	0.06046131	0.999933887	-0.049108403	0.2026182	-0.251726603	0.147724171
ENSG00000180758	*GPR157*	3.014800567	-0.090871641	0.102623371	-0.193495012	0.999933887	0.895894371	1.286194902	-0.390300531	0.147762332
ENSG00000084774	*CAD*	3.287447343	0.027047107	0.062829344	-0.035782237	0.999933887	0.108998084	-0.087712185	0.196710269	0.147838936
ENSG00000258921	*AC009779.4*	-0.468997016	0.012021549	-0.041235269	0.053256818	0.999933887	1.382002741	1.860580973	-0.478578232	0.148081502
ENSG00000146776	*ATXN7L1*	2.834423794	-0.003192605	-0.019505838	0.016313233	0.999933887	0.176331324	0.412933378	-0.236602054	0.148081502
ENSG00000204619	*PPP1R11*	6.701932121	0.036891942	0.002369565	0.034522377	0.999933887	0.392686255	0.545109069	-0.152422814	0.148230089
ENSG00000187741	*FANCA*	2.16837568	0.139861035	0.127600826	0.01226021	0.999933887	-0.349793865	-0.735488403	0.385694538	0.148467486
ENSG00000142961	*MOB3C*	4.149834255	-0.011959976	0.014032988	-0.025992964	0.999933887	0.247278649	0.524634397	-0.277355748	0.148477574
ENSG00000224614	*TNK2-AS1*	1.204128271	-0.039474785	-0.096668373	0.057193588	0.999933887	-1.019960189	-1.455728118	0.435767929	0.148802784
ENSG00000224307	*AL161785.1*	1.046517319	-0.069731287	-0.055466387	-0.0142649	0.999933887	-1.250705784	-1.758363507	0.507657723	0.148811419
ENSG00000151914	*DST*	0.218376341	-0.066742865	0.118075888	-0.184818753	0.999933887	-0.351176333	0.269785729	-0.620962062	0.148811419
ENSG00000174886	*NDUFA11*	2.138171414	0.135647394	0.041149959	0.094497435	0.999933887	0.071841549	-0.207796338	0.279637888	0.148907387
ENSG00000133106	*EPSTI1*	5.078541582	-0.051252457	-0.053637751	0.002385294	0.999933887	-0.092079263	-0.251696518	0.159617256	0.148907387
ENSG00000170759	*KIF5B*	6.027375994	-0.021209917	-0.119199359	0.097989442	0.999933887	-0.606170831	-0.273674155	-0.332496675	0.149035621
ENSG00000165185	*KIAA1958*	1.212158406	0.003484773	-0.041903446	0.045388219	0.999933887	-0.268935113	-0.753131985	0.484196872	0.149147597
ENSG00000165832	*TRUB1*	0.509468608	-0.056417937	-0.08226133	0.025843393	0.999933887	-0.101111682	0.352376325	-0.453488007	0.149172154
ENSG00000149591	*TAGLN*	2.791973582	0.027395027	0.073067965	-0.045672938	0.999933887	0.072069965	-0.173300407	0.245370372	0.149220517
ENSG00000146904	*EPHA1*	2.31095607	0.155011538	0.184463181	-0.029451643	0.999933887	-0.359921471	-0.720556818	0.360635346	0.149241409
ENSG00000258441	*LINC00641*	5.410605811	-0.017380388	-0.00412999	-0.013250398	0.999933887	1.424545687	1.825250569	-0.400704883	0.149345453
ENSG00000100162	*CENPM*	-0.30335497	-0.052664362	0.1269947	-0.179659062	0.999933887	0.243433425	-0.29731733	0.540750755	0.149368536
ENSG00000041802	*LSG1*	3.903682982	-0.041803079	0.033647901	-0.07545098	0.999933887	0.108573017	0.271980454	-0.163407437	0.149415071
ENSG00000012171	*SEMA3B*	1.924123934	0.031617733	0.126048154	-0.094430421	0.999933887	0.035797365	-0.373521239	0.409318605	0.149415071
ENSG00000084090	*STARD7*	4.364613919	-0.021897715	-0.00799051	-0.013907205	0.999933887	0.086117062	0.296648695	-0.210531633	0.149415071
ENSG00000115295	*CLIP4*	3.174598706	-0.025105148	-0.107899561	0.082794413	0.999933887	-0.357711611	-0.657787092	0.300075481	0.149415071
ENSG00000206418	*RAB12*	2.252509941	0.02034683	0.059974552	-0.039627722	0.999933887	0.688321589	0.972555913	-0.284234325	0.149415071
ENSG00000107929	*LARP4B*	6.25400346	-0.02697087	0.039314438	-0.066285307	0.999933887	-0.387562036	-0.513093928	0.125531892	0.149449412
ENSG00000049759	*NEDD4L*	0.431386481	-0.121021079	0.021935766	-0.142956845	0.999933887	0.446794213	0.797255404	-0.350461191	0.149504886
ENSG00000269825	*AC022150.4*	0.00640683	0.115518317	0.163749634	-0.048231317	0.999933887	-0.297035945	0.187146734	-0.484182679	0.149622169
ENSG00000164442	*CITED2*	5.352821438	0.03123852	0.033277527	-0.002039007	0.999933887	0.089501721	0.283157883	-0.193656162	0.149622169
ENSG00000130881	*LRP3*	-0.11347176	-0.052803439	0.005972903	-0.058776342	0.999933887	-0.192770608	-0.836897309	0.644126701	0.149877134
ENSG00000021762	*OSBPL5*	2.906424413	0.004837567	0.212897096	-0.208059528	0.999933887	-0.074409324	-0.323604564	0.24919524	0.149877134
ENSG00000253710	*ALG11*	1.84230728	-0.004637557	-0.089155394	0.084517837	0.999933887	-0.003856283	-0.311319286	0.307463003	0.149974297
ENSG00000147403	*RPL10*	8.351173928	0.050950151	-0.02223084	0.073180991	0.999933887	0.218747023	0.380282685	-0.161535662	0.149992749
ENSG00000153363	*LINC00467*	0.021148686	-0.03065179	0.021115409	-0.051767199	0.999933887	-0.025961508	0.552265197	-0.578226705	0.150094166
ENSG00000101310	*SEC23B*	4.747441944	0.007053917	-0.019303234	0.026357151	0.999933887	0.37551035	0.547531126	-0.172020776	0.150421826
ENSG00000221963	*APOL6*	6.592990245	0.007715664	-0.021401894	0.029117558	0.999933887	-0.470156965	-0.655229342	0.185072376	0.150469436
ENSG00000159842	*ABR*	7.716961895	0.017980145	0.015460894	0.002519251	0.999933887	0.088143649	0.215024756	-0.126881107	0.150725338
ENSG00000179921	*GPBAR1*	-0.046382135	0.017932836	0.031812831	-0.013879995	0.999933887	0.029406699	-0.596165352	0.625572051	0.15083128
ENSG00000285730	*Z94721.3*	1.232922958	0.097197639	-0.067030901	0.16422854	0.999933887	-0.419029569	-0.85914107	0.440111502	0.150947639
ENSG00000162819	*BROX*	5.46184249	-0.159535924	-0.066106363	-0.093429561	0.999933887	-0.439449321	-0.226959757	-0.212489565	0.151086304
ENSG00000167674	*HDGFL2*	3.932342824	0.082412231	0.143606855	-0.061194624	0.999933887	-0.133258456	-0.41079045	0.277531994	0.151396732
ENSG00000230521	*AL645929.1*	-0.100610568	-0.048128548	0.393815375	-0.441943922	0.999933887	0.901275595	0.374856367	0.526419228	0.151446913
ENSG00000172667	*ZMAT3*	3.121094356	0.006858628	0.018169542	-0.011310913	0.999933887	-0.01267997	0.287998586	-0.300678556	0.151446913

ENSG00000114013	*CD86*	0.673401877	0.00913733	-0.126100337	0.135237667	0.999933887	-0.443048895	0.119798658	-0.562847553	0.1514647
ENSG00000138768	*USO1*	4.003340524	-0.035216224	-0.141015422	0.105799198	0.999933887	-0.314016846	-0.012112226	-0.301904619	0.151591045
ENSG00000244754	*N4BP2L2*	6.805177846	-0.054101043	-0.087926797	0.033825754	0.999933887	-0.009535533	0.334410172	-0.343945704	0.151595764
ENSG00000168101	*NUDT16L1*	3.594020695	0.026956641	-0.050846072	0.077802714	0.999933887	0.032576912	-0.224299411	0.256876323	0.151595764
ENSG00000113296	*THBS4*	-0.527727599	0.066951114	0.175814023	-0.10886291	0.999933887	-0.300920432	0.384795981	-0.685716413	0.151669291
ENSG00000157353	*FCSK*	2.938484096	0.043634017	0.198958257	-0.155324241	0.999933887	0.049225936	-0.162527649	0.211753585	0.151707712
ENSG00000124145	*SDC4*	1.148010384	-0.170208506	-0.090136005	-0.080072501	0.999933887	1.631012368	2.185558833	-0.554546465	0.151707712
ENSG00000099624	*ATP5F1D*	3.556715219	0.041838934	-0.043865414	0.085704347	0.999933887	-0.10002802	-0.360475789	0.260447769	0.151707712
ENSG00000198862	*LTN1*	4.792231937	-0.072562746	-0.156003679	0.083440933	0.999933887	-0.519230108	-0.068053579	-0.451176529	0.151707712
ENSG00000204946	*ZNF783*	2.589317188	0.037625243	0.085821404	-0.048196161	0.999933887	0.065434593	-0.167174654	0.232609247	0.151707712
ENSG00000034152	*MAP2K3*	8.701344868	-0.027327068	-0.008627197	-0.01869987	0.999933887	1.187668351	1.379308672	-0.191640321	0.151707712
ENSG00000021776	*AQR*	3.869758218	0.00664505	-0.064060038	0.070705088	0.999933887	-0.188786981	0.052867161	-0.241654141	0.151707712
ENSG00000118246	*FASTKD2*	2.180896683	-0.139784545	-0.054460519	-0.085324026	0.999933887	0.010457991	0.321010773	-0.310552782	0.151726246
ENSG00000166889	*PATL1*	8.121588279	0.00631347	0.016578376	-0.010264906	0.999933887	0.499430378	0.671088004	-0.171657627	0.151726246
ENSG00000179144	*GIMAP7*	3.890955126	-0.094982377	-0.184467733	0.089485356	0.999933887	0.105867724	0.298529501	-0.192661777	0.151749122
ENSG00000204960	*BLACE*	-0.653758995	0.154014417	0.39985454	-0.245840122	0.999933887	2.456220623	3.046439932	-0.59021931	0.151749122
ENSG00000114626	*ABTB1*	9.436386599	0.007573989	0.008718563	-0.001144574	0.999933887	-0.17417909	-0.327015395	0.152836304	0.151762201
ENSG00000121988	*ZRANB3*	-0.277020161	-0.02285781	-0.133721572	0.110863762	0.999933887	-0.476237899	0.178065063	-0.654302962	0.151762201
ENSG00000108785	*HSD17B1P1*	0.009302307	0.132507825	0.107915098	0.024592727	0.999933887	-0.171767182	-0.725015488	0.553248306	0.151778607
ENSG00000225630	*MTND2P28*	4.056775559	0.128388373	0.059963539	0.068424834	0.999933887	0.051851245	0.319968375	-0.26811713	0.151882137
ENSG00000111052	*LIN7A*	4.387342231	-0.061882457	-0.021415076	-0.040467381	0.999933887	-0.894723967	-1.218201664	0.323477697	0.151891014
ENSG00000181019	*NQO1*	0.354869481	0.137111385	0.220508367	-0.083396982	0.999933887	0.189303015	0.549205263	-0.359902248	0.15194206
ENSG00000175826	*CTDNEP1*	5.282275677	-0.043685466	0.007161791	-0.050847256	0.999933887	-0.075330702	-0.21792476	0.142594058	0.152046131
ENSG00000157601	*MX1*	7.284294973	-0.021050288	0.008557263	-0.029607551	0.999933887	-0.397725436	-0.571056469	0.173331033	0.152046131
ENSG00000164073	*MFSD8*	2.474373876	-0.027836406	-0.027578418	-0.000257988	0.999933887	-0.357786571	-0.616794282	0.259007711	0.152101527
ENSG00000223482	*NUTM2A-AS1*	3.172183016	-0.057249379	-0.022921007	-0.034328372	0.999933887	-0.312196108	-0.521828518	0.20963241	0.152108116
ENSG00000204673	*AKT1S1*	3.883269879	0.044609642	0.068746069	-0.024136427	0.999933887	-0.558933771	-0.748614289	0.189680518	0.152108116
ENSG00000055044	*NOP58*	3.828811234	-0.067039957	-0.192678232	0.125638275	0.999933887	0.124353193	0.329846694	-0.2054935	0.152278364
ENSG00000205744	*DENND1C*	6.127017033	0.027342512	0.04894585	-0.021603338	0.999933887	-0.068754732	-0.189915522	0.121160791	0.152334828
ENSG00000129353	*SLC44A2*	8.597467412	-0.029043713	-0.035990207	0.006946494	0.999933887	-0.826639463	-1.022499341	0.195859878	0.152334828
ENSG00000011275	*RNF216*	5.520553874	-0.018176687	0.021570393	-0.039747081	0.999933887	-0.046778773	-0.160562475	0.113783702	0.152368068
ENSG00000124713	*GNMT*	0.234554024	-0.023547174	0.014275398	-0.037822572	0.999933887	-0.172013251	-0.713432246	0.541418996	0.152530602
ENSG00000172046	*USP19*	6.397972773	-0.049047254	0.037970375	-0.087017629	0.999933887	-0.376954854	-0.51686125	0.139906397	0.152675819
ENSG00000276085	*CCL3L3*	10.130244606	0.59802638	0.609265036	-0.011238656	0.999933887	2.721293567	3.188856757	-0.46756319	0.152728443
ENSG00000276085	*CCL3L3*	10.130244606	0.59802638	0.609265036	-0.011238656	0.999933887	2.721293567	3.188856757	-0.46756319	0.152728443
ENSG00000120265	*PCMT1*	3.954563164	0.032137706	-0.038246744	0.07038445	0.999933887	-0.38017006	-0.600791512	0.220621452	0.153014005
ENSG00000280020	*AC116407.3*	-0.766404744	0.012214854	-0.091310154	0.103525008	0.999933887	-0.164928069	-1.115771801	0.950843732	0.153104927
ENSG00000286584	*AC023494.1*	0.244078491	-0.223678348	0.070663066	-0.294341415	0.999933887	0.354960722	0.781380646	-0.426419924	0.153249624
ENSG00000248323	*LUCAT1*	9.124534462	0.057227236	0.090927037	-0.033699801	0.999933887	0.511284116	0.691762481	-0.180478365	0.153609554
ENSG00000287269	*Z82215.1*	0.546144657	0.057646813	0.171729683	-0.11408287	0.999933887	0.431416961	0.951149132	-0.519732171	0.153609554
ENSG00000286004	*AC124944.3*	1.177819361	-0.055365738	0.058790567	-0.114156305	0.999933887	-0.465449283	-0.902739659	0.437290376	0.153674043
ENSG00000175467	*SART1*	5.581016468	0.029547096	0.058382572	-0.028835476	0.999933887	-0.20981851	-0.460757476	0.250938966	0.153674043
ENSG00000167182	*SP2*	5.646117354	0.006760707	-0.041727227	0.048487934	0.999933887	0.068089155	0.202288257	-0.134199102	0.153873003
ENSG00000142669	*SH3BGRL3*	8.131526663	0.028368968	-0.009126834	0.037495802	0.999933887	-0.258981221	-0.418968699	0.159987479	0.15399776
ENSG00000166685	*COG1*	3.635495506	0.082450654	0.065197069	0.017253586	0.999933887	-0.085424461	-0.293218646	0.207794185	0.15404243
ENSG00000151748	*SAV1*	1.563265394	0.087167958	0.216625307	-0.129457349	0.999933887	0.463921358	0.766937069	-0.303015711	0.15404243
ENSG00000251127	*AC091173.1*	3.878099471	0.186001847	0.115971139	0.070030708	0.999933887	1.333414354	1.684441882	-0.351027529	0.154227046
ENSG00000041988	*THAP3*	1.95074799	-0.146466958	-0.280729935	0.134262977	0.999933887	-0.105169321	-0.34025731	0.235087989	0.154290925
ENSG00000173918	*C1QTNF1*	-1.95464785	-0.245324469	1.227233421	-1.47255789	0.999933887	1.389338773	2.048938096	-0.659599323	0.154290925
ENSG00000005339	*CREBBP*	9.07465888	0.01301701	0.055141958	-0.042124948	0.999933887	0.142783755	0.253549032	-0.110765276	0.154290925
ENSG00000047634	*SCML1*	3.685666347	-0.012169954	-0.143482909	0.131312955	0.999933887	0.263988876	0.64597006	-0.381981184	0.154290925
ENSG00000104408	*EIF3E*	5.177948861	-0.091901411	-0.168020589	0.076119178	0.999933887	0.023322058	0.245786228	-0.22246417	0.154315413
ENSG00000156515	*HK1*	5.143489744	0.00934078	-0.015477108	0.024817889	0.999933887	-0.089716563	-0.238144214	0.148427652	0.154315413
ENSG00000247828	*TMEM161B-AS*	1.880861043	0.046217251	-0.286979613	0.333196865	0.999933887	-0.319242289	0.117936384	-0.437178674	0.154347596
ENSG00000259807	*AC009093.1*	-0.44836692	-0.041817265	-0.184559782	0.142742516	0.999933887	-0.194503638	0.461110267	-0.655613905	0.154365792
ENSG00000198836	*OPA1*	4.224676961	0.016086383	-0.056679371	0.072765754	0.999933887	-0.229767758	0.10723014	-0.336997897	0.15480416
ENSG00000100429	*HDAC10*	0.646843116	0.110474341	-0.011525399	0.121999739	0.999933887	0.010971574	-0.447321645	0.458293219	0.15482473
ENSG00000100258	*LMF2*	6.226127874	0.048660097	0.057130157	-0.008470061	0.999933887	-0.03578996	-0.188155283	0.152365323	0.154970282
ENSG00000255801	*AC092746.1*	1.394257435	0.019160521	-0.009191044	0.028351565	0.999933887	1.456565728	1.881262933	-0.424697205	0.154970282
ENSG00000092201	*SUPT16H*	4.685347861	-0.087112192	-0.226182846	0.139070654	0.999933887	-0.203205232	-0.365023944	0.161818712	0.155038369
ENSG00000147168	*IL2RG*	6.762997652	0.042140148	0.067793001	-0.025652853	0.999933887	0.522407263	0.670185422	-0.147778159	0.155038369
ENSG00000187595	*ZNF385C*	-2.18342944	-0.030292859	-0.098525627	0.068232768	0.999933887	2.588301905	3.806247951	-1.217946046	0.155038369
ENSG00000018189	*RUFY3*	2.040368243	-0.034657498	-0.036295122	0.001637624	0.999933887	-0.118425193	0.164007441	-0.282432634	0.155114557
ENSG00000266338	*NBPF15*	2.826045287	-0.014248325	-0.024786482	0.010538157	0.999933887	0.169203645	0.416690078	-0.247486433	0.15513726
ENSG00000271335	*AL117336.2*	-0.659708531	-0.03010294	0.257710781	-0.287813722	0.999933887	-0.417632668	-1.038948647	0.621315979	0.15513726
ENSG00000077254	*USP33*	4.517318309	0.005449516	-0.01985044	0.025299956	0.999933887	-0.337666302	-0.563950478	0.226284176	0.15513726
ENSG00000103202	*NME4*	2.716243728	0.074165161	0.016036722	0.058128439	0.999933887	0.006598049	-0.240380568	0.246978617	0.155144254
ENSG00000133997	*MED6*	3.294227428	-0.034260593	0.00966443	-0.043925023	0.999933887	-0.117467911	0.05169929	-0.169167201	0.155144254
ENSG00000091181	*IL5RA*	2.501150156	-0.047341981	0.113288668	-0.160630649	0.999933887	-0.084557564	0.137031143	-0.221588707	0.155221971
ENSG00000167291	*TBC1D16*	0.974213991	0.023226386	0.084776977	-0.061550591	0.999933887	0.920245079	1.35443853	-0.434193451	0.155327453
ENSG00000264304	*AC024267.3*	0.349406715	-0.007961114	0.001765034	-0.009726148	0.999933887	0.605748611	1.254219186	-0.648470575	0.155327453
ENSG00000168476	*REEP4*	3.005349507	0.013288897	0.012702384	0.000586514	0.999933887	-0.075622408	-0.338572021	0.262949613	0.155477574
ENSG00000204632	*HLA-G*	4.029116289	0.084878431	0.002572141	0.082306291	0.999933887	0.369554809	-0.058701957	0.428256766	0.155504213
ENSG00000165891	*E2F7*	-0.261287748	-0.046421309	0.088666731	-0.13508804	0.999933887	0.438500981	1.033397873	-0.594896892	0.155524118
ENSG00000179981	*TSHZ1*	4.860394747	-0.054344968	0.006175215	-0.060520183	0.999933887	-0.972771009	-1.215728194	0.242957185	0.155550206
ENSG00000224397	*PELATON*	7.620070107	0.043806584	0.026847143	0.016959441	0.999933887	0.432356074	0.585752751	-0.153396677	0.155569838
ENSG00000131355	*ADGRE3*	7.77703752	-0.026165933	-0.016402108	-0.009763825	0.999933887	-0.850618601	-1.047633364	0.197014763	0.155569838
ENSG00000136810	*TXN*	6.690016368	-0.011809805	-0.022832305	0.0110225	0.999933887	0.580951686	0.755161859	-0.174210173	0.155621151
ENSG00000164022	*AIMP1*	3.14102872	-0.094980914	-0.106394342	0.011413427	0.999933887	-0.109223696	0.132067323	-0.241291019	0.155927184
ENSG00000087206	*UIMC1*	5.902255081	-0.039474593	-0.027952259	-0.011522334	0.999933887	-0.707375589	-0.862135956	0.154760367	0.155927184
ENSG00000189042	*ZNF567*	0.370423957	-0.136636615	-0.311551981	0.174915366	0.999933887	-0.053820223	0.470745589	-0.524565812	0.156028452
ENSG00000196460	*RFX8*	-0.715842515	0.278384773	0.141795824	0.136588949	0.999933887	2.674441585	3.366557712	-0.692116127	0.156368636
ENSG00000196628	*TCF4*	0.673379441	-0.078693869	0.054070535	-0.132764404	0.999933887	-0.450191378	-0.016546556	-0.433644821	0.156368636
ENSG00000160208	*RRP1B*	3.2357012	-0.070371261	-0.045065779	-0.025305482	0.999933887	0.221545741	0.41666163	-0.195115888	0.156394541
ENSG00000204186	*ZDBF2*	2.05327961	-0.110411882	-0.196275524	0.085863642	0.999933887	-0.223313442	0.345803119	-0.569116561	0.156394541
ENSG00000179362	*HMGN2P46*	-1.270099294	-0.237811123	-0.419797901	0.181986778	0.999933887	1.807241129	2.54231196	-0.735070831	0.156414972
ENSG00000116539	*ASH1L*	6.242810068	-0.040374979	-0.053699799	0.013324819	0.999933887	-0.271494354	-0.013428117	-0.258066236	0.156420525
ENSG00000168283	*BMI1*	2.866783005	0.016142493	-0.005820737	0.02196323	0.999933887	-0.275885663	-0.005038806	-0.270846857	0.156429367
ENSG00000178695	*KCTD12*	4.969390161	-0.198750392	-0.198503916	-0.000246476	0.999933887	-1.015567247	-1.311927711	0.296360464	0.156520031
ENSG00000185561	*TLCD2*	1.388787297	-0.019390551	0.040480747	-0.059871298	0.999933887	-0.086787395	0.225867235	-0.31265463	0.156827783
ENSG00000087338	*GMCL1*	3.864549791	0.054920533	7.6742e-05	0.054843791	0.999933887	-0.422874248	-0.647652181	0.224777933	0.156929443
ENSG00000237596	*AL138828.1*	-1.366475113	0.611443938	0.782307903	-0.170863964	0.999933887	1.03247625	1.526944803	-0.494468553	0.157028935
ENSG00000103035	*PSMD7*	4.66242009	-0.059121326	-0.128217954	0.069096628	0.999933887	-0.036856997	0.103317436	-0.140174433	0.157028935
ENSG00000177868	*SVBP*	2.145013755	-0.098173815	-0.100780532	0.002606717	0.999933887	-0.29171672	-0.005962299	-0.285754421	0.157278212
ENSG00000157873	*TNFRSF14*	10.030765685	0.047919465	0.058877755	-0.01095829	0.999933887	0.670235919	0.824220639	-0.153984721	0.157278212
ENSG00000135951	*TSGA10*	0.670992292	0.027391532	0.073280815	-0.045889283	0.999933887	0.46962932	0.845137924	-0.375508604	0.15730306
ENSG00000141367	*CLTC*	8.024607357	-0.118822703	-0.143035243	0.02421254	0.999933887	-0.0783	0.111258642	-0.189558643	0.157395135
ENSG00000214706	*IFRD2*	2.348946332	0.020211129	-0.069383034	0.089594163	0.999933887	0.665540229	0.948013366	-0.282473137	0.157404456
ENSG00000286940	*MICB-DT*	3.041256122	0.02156592	0.029987668	-0.008421748	0.999933887	0.993359037	1.274442146	-0.28108311	0.157534564
ENSG00000069667	*RORA*	5.224014197	-0.111580684	-0.117101547	0.005520863	0.999933887	-0.293750872	0.124357058	-0.41810793	0.158052651
ENSG00000116095	*PLEKHA3*	3.463683804	0.003164076	0.104807712	-0.101643635	0.999933887	-0.251817649	-0.454894424	0.203076775	0.158052651
ENSG00000171497	*PPID*	4.23761206	-0.14178747	-0.119071574	-0.022715896	0.999933887	0.180273521	0.337768444	-0.157494922	0.158099731
ENSG00000186265	*BTLA*	0.678265216	-0.11603346	-0.489204884	0.373171424	0.999933887	-0.014553071	0.351356447	-0.365909518	0.158205299
ENSG00000239948	*RN7SL368P*	3.55141622	0.286173169	0.160684242	0.125488927	0.999933887	1.322763319	1.807406303	-0.484642984	0.158580596
ENSG00000160712	*IL6R*	9.268635389	-0.032447104	-0.031001381	-0.001445723	0.999933887	-0.205172332	-0.038959103	-0.166213229	0.158580596

ENSG00000119720	*NRDE2*	3.836205892	-0.081261059	-0.051178732	-0.030082327	0.999933887	-0.231096543	-0.404782801	0.173686259	0.158663911
ENSG00000155287	*SLC25A28*	4.407152903	0.118563931	0.051941042	0.066622889	0.999933887	0.024085349	-0.2291079	0.253193249	0.158800929
ENSG00000147905	*ZCCHC7*	2.947000803	-0.020170102	-0.116937054	0.096766952	0.999933887	-0.01045924	-0.211389527	0.200930287	0.158800929
ENSG00000167461	*RAB8A*	4.725745595	-0.02760791	-0.006220097	-0.021387813	0.999933887	-0.326066592	-0.502813538	0.176746945	0.159221423
ENSG00000027847	*B4GALT7*	2.702976481	0.007016999	0.230346861	-0.223329863	0.999933887	0.51968674	0.76583257	-0.24614583	0.159413832
ENSG00000050405	*LIMA1*	0.758146415	-0.012214196	0.068661015	-0.08087521	0.999933887	-0.13351771	0.249469871	-0.382987581	0.159466978
ENSG00000157551	*KCNJ15*	8.804238678	-0.048968082	-0.033371542	-0.01559654	0.999933887	-0.531881846	-0.369396346	-0.162485499	0.15967798
ENSG00000111711	*GOLT1B*	2.974906168	-0.126357353	-0.114335797	-0.012021557	0.999933887	-0.225687628	0.0362114	-0.261899028	0.159975042
ENSG00000184106	*TREML3P*	5.075255847	0.014772457	-0.043827023	0.05859948	0.999933887	0.558227194	0.75171268	-0.193485486	0.160066378
ENSG00000158480	*SPATA2*	4.85597875	0.066226163	0.00632606	0.059900103	0.999933887	0.370873223	0.544307026	-0.173433803	0.160171466
ENSG00000185477	*GPRIN3*	4.744285639	-0.024839705	-0.254675828	0.229836123	0.999933887	-0.333077954	0.07126583	-0.404343784	0.160171466
ENSG00000233276	*GPX1*	6.170466486	-0.054587908	-0.06788634	0.013298431	0.999933887	-0.122465676	-0.339259677	0.216794001	0.160182584
ENSG00000116205	*TCEANC2*	2.59467553	0.094413595	-0.064747868	0.159161464	0.999933887	-0.013410946	-0.20902158	0.195610635	0.16021872
ENSG00000128271	*ADORA2A*	4.754548031	0.31873947	0.182726043	0.136013427	0.999933887	1.989422872	2.381790948	-0.392368075	0.160318555
ENSG00000007520	*TSR3*	3.776125621	0.054410988	0.086634107	-0.032223119	0.999933887	-0.138099942	-0.376940164	0.238840222	0.160318555
ENSG00000108932	*SLC16A6*	5.137145824	-0.137726838	-0.040984748	-0.09674209	0.999933887	-0.148468827	0.054445931	-0.202914758	0.160477575
ENSG00000131263	*RLIM*	6.870254875	-0.012323865	-0.089125015	0.07680115	0.999933887	-0.169070457	0.069472452	-0.238542909	0.160523818
ENSG00000163882	*POLR2H*	1.921291022	0.012773263	-0.003293614	0.016066877	0.999933887	0.15472329	-0.112242159	0.266965449	0.160523818
ENSG00000223722	*IFITM3P2*	2.129389531	0.225343406	0.046343482	0.178999924	0.999933887	1.180059664	1.536758264	-0.356698599	0.160556796
ENSG00000253194	*AL137009.1*	-0.125747972	-0.063142635	-0.05449761	-0.008645024	0.999933887	-0.218889069	0.549101347	-0.767990416	0.160669596
ENSG00000165525	*NEMF*	4.406900757	0.002042069	-0.110488986	0.112531056	0.999933887	-0.335949137	0.045686408	-0.381635545	0.160678219
ENSG00000102100	*SLC35A2*	4.81232209	0.037279955	0.066296703	-0.029016748	0.999933887	1.519839992	1.826923554	-0.307083562	0.160737302
ENSG00000169499	*PLEKHA2*	7.738224032	-0.083798043	0.001466979	-0.085265023	0.999933887	-0.321345534	-0.158955767	-0.162389767	0.160904192
ENSG00000168685	*IL7R*	7.007448576	-0.005164961	-0.114242595	0.109077634	0.999933887	0.122487692	0.25519128	-0.132703588	0.161332093
ENSG00000072694	*FCGR2B*	3.02994491	0.022213818	-0.0869438	0.109157617	0.999933887	-0.159700258	-0.423540802	0.263840544	0.16160197
ENSG00000108465	*CDK5RAP3*	3.998276691	-0.005234761	0.035896686	-0.041131446	0.999933887	0.023887012	-0.157091508	0.18097852	0.161804543
ENSG00000162600	*OMA1*	2.002704132	-0.046527756	-0.042426549	-0.004101206	0.999933887	-0.062057924	-0.316733417	0.254675493	0.161805694
ENSG00000090273	*NUDC*	4.277730574	-0.002312824	-0.001061773	-0.001251052	0.999933887	-0.209061669	-0.411733398	0.202671729	0.161805694
ENSG00000211689	*TRGC1*	2.150069979	-0.108510016	-0.024596059	-0.083913957	0.999933887	0.029068701	0.301197817	-0.272129116	0.161823148
ENSG00000031698	*SARS1*	4.903998316	-0.017348021	-0.009075557	-0.008272463	0.999933887	-0.040821219	-0.189261121	0.148439902	0.162128337
ENSG00000103264	*FBXO31*	2.924085817	0.030657978	0.043677905	-0.013019928	0.999933887	0.071707593	-0.141231891	0.212939484	0.16216114
ENSG00000267680	*ZNF224*	2.633110261	0.027953805	-0.114639644	0.142593449	0.999933887	-0.239259784	-0.546661816	0.307402032	0.16216114
ENSG00000213337	*ANKRD39*	0.193415555	-0.125899301	-0.208969689	0.083070387	0.999933887	-0.122949757	-0.596021656	0.473071899	0.162592754
ENSG00000136147	*PHF11*	5.76386695	-0.029253179	-0.03378273	0.004529551	0.999933887	-0.175556312	-0.051316926	-0.124239386	0.162592754
ENSG00000095321	*CRAT*	1.42944817	-0.034750817	0.029965145	-0.064715961	0.999933887	-0.067193385	-0.39064172	0.323448335	0.162657362
ENSG00000225111	*AC083900.1*	-0.010255455	0.077713752	0.350702673	-0.272988921	0.999933887	1.704944152	2.122024712	-0.417080561	0.162692383
ENSG00000188735	*TMEM120B*	2.795989894	0.048348207	0.15185605	-0.103507843	0.999933887	1.517554251	1.859325196	-0.341770945	0.162829139
ENSG00000225963	*AC009950.1*	2.11078961	-0.052205041	-0.047297813	-0.004907228	0.999933887	-0.788741936	-1.153720805	0.364978869	0.163066226
ENSG00000179041	*RRS1*	1.249755979	-0.016569068	0.018191118	-0.034760187	0.999933887	0.206796793	0.54475004	-0.337953246	0.163080517
ENSG00000163597	*SNHG16*	4.075314831	-0.066184828	-0.087198183	0.021013355	0.999933887	-0.237785348	-0.453216213	0.215430865	0.163225906
ENSG00000105223	*PLD3*	4.513436463	0.118243013	-0.060783396	0.17902641	0.944094537	-0.058317632	-0.227992105	0.169674474	0.163285189
ENSG00000273117	*AC144652.1*	1.976465754	0.213623652	0.126133137	0.087490515	0.999933887	0.881895829	1.301334822	-0.419438993	0.163285189
ENSG00000248734	*AC008906.1*	1.126952167	-0.102212457	0.035871778	-0.138084235	0.999933887	-0.618662844	-0.104592072	-0.514070772	0.163285189
ENSG00000278017	*AC064801.1*	0.362519152	-0.067490024	0.030129872	-0.097619896	0.999933887	0.365873319	0.877452301	-0.511578981	0.163474065
ENSG00000254887	*AC010247.1*	0.428779697	-0.013879049	0.436844457	-0.450723506	0.999933887	0.385699636	0.719584332	-0.333884696	0.163563378
ENSG00000275074	*NUDT18*	2.058544928	0.027822096	-0.010033389	0.037855485	0.999933887	-0.159805067	-0.433535047	0.273729979	0.163638522
ENSG00000139496	*NUP58*	6.263129368	-0.023220127	-0.027773901	0.004553774	0.999933887	0.211090134	0.412721571	-0.201631437	0.163696317
ENSG00000235944	*ZNF815P*	1.062797218	0.152730718	0.290899657	-0.13816894	0.999933887	0.117319008	-0.275541913	0.392860921	0.163721183
ENSG00000068308	*OTUD5*	7.280536145	0.037633207	0.020561421	0.017071787	0.999933887	0.418434224	0.56630929	-0.147875066	0.163788969
ENSG00000185344	*ATP6V0A2*	3.645483132	-0.020376863	-0.018674745	-0.001702118	0.999933887	-0.093023699	-0.29740685	0.20438315	0.163788969
ENSG00000183696	*UPP1*	6.83658304	-0.038523464	-0.004891202	-0.033632262	0.999933887	0.739836551	0.910476463	-0.170639912	0.163950339
ENSG00000198668	*CALM1*	7.448914695	0.015479078	-0.070034931	0.085514009	0.999933887	-0.023892593	-0.154812084	0.13091949	0.163950339
ENSG00000100142	*POLR2F*	3.209193017	0.04670558	-0.011998846	0.058704426	0.999933887	-0.161836422	-0.398565675	0.236729253	0.16410975
ENSG00000106400	*ZNHIT1*	4.533475458	0.004056347	0.033424672	-0.029368325	0.999933887	-0.198774468	-0.372450382	0.173675914	0.16410975
ENSG00000143947	*RPS27A*	7.768833755	-0.038940419	-0.051562504	0.012622085	0.999933887	0.18207037	0.33799942	-0.15592905	0.164141745
ENSG00000215717	*TMEM167B*	6.812054554	-0.056870274	-0.044604698	-0.012265575	0.999933887	0.012750839	0.143777486	-0.131026647	0.164219168
ENSG00000198492	*YTHDF2*	4.307808443	-0.043852592	-0.1011873	0.057334708	0.999933887	-0.27784318	-0.445866177	0.168022997	0.164348382
ENSG00000067829	*IDH3G*	4.144783375	0.07618991	-0.003585585	0.079775495	0.999933887	-0.238005735	-0.404196692	0.166190958	0.164366048
ENSG00000204389	*HSPA1A*	5.551846764	0.038676375	0.032205551	0.006470824	0.999933887	0.69334642	1.030084026	-0.336737606	0.164852794
ENSG00000146834	*MEPCE*	6.745041824	0.096606901	0.117481429	-0.020874527	0.999933887	0.225277576	0.384794971	-0.159517395	0.165055845
ENSG00000034510	*TMSB10*	7.847556443	0.011431077	-0.064823381	0.076254458	0.999933887	0.032991443	-0.118218616	0.151210058	0.165055845
ENSG00000103507	*BCKDK*	5.157297172	-0.022511054	0.019167232	-0.041678287	0.999933887	-0.658758007	-0.849577846	0.190819839	0.165074964
ENSG00000198786	*MT-ND5*	8.288225626	0.009004829	-0.029462681	0.038467509	0.999933887	0.024484933	0.249308807	-0.224823873	0.165074964
ENSG00000108094	*CUL2*	3.817796161	-0.098255811	-0.127170332	0.028914522	0.999933887	-0.351059382	-0.126961511	-0.224097872	0.165308352
ENSG00000093010	*COMT*	2.026381423	-0.029922311	0.178344312	-0.208266623	0.999933887	0.02732074	-0.213060854	0.240381594	0.165308352
ENSG00000176953	*NFATC2IP*	5.187939059	-0.048496882	-0.002973182	-0.045523701	0.999933887	0.079692606	0.222299853	-0.142607247	0.165340798
ENSG00000181450	*ZNF678*	0.419191058	-0.056810807	-0.261145344	0.204334537	0.999933887	-0.290840793	0.250483627	-0.54132442	0.165519511
ENSG00000068903	*SIRT2*	6.316457954	0.025235606	0.00434272	0.020892886	0.999933887	0.266667438	0.394364459	-0.127697021	0.165540782
ENSG00000279133	*AC018628.1*	1.437137204	-0.048769566	0.114379111	-0.163148677	0.999933887	0.223689871	0.658319438	-0.434629567	0.165540782
ENSG00000004961	*HCCS*	2.58428543	0.021642651	0.058425461	-0.036782809	0.999933887	0.046484222	-0.170765942	0.217250164	0.165987102
ENSG00000140941	*MAP1LC3B*	8.590791287	0.037890441	0.002209295	0.035681146	0.999933887	0.032676359	0.154264305	-0.121587946	0.166040855
ENSG00000103245	*CIAO3*	2.161629858	0.052034934	0.084300988	-0.032266053	0.999933887	0.084435584	-0.17657361	0.261009194	0.166067792
ENSG00000170075	*GPR37L1*	1.033503484	-0.104099597	-0.00775678	-0.096342817	0.999933887	-0.437628156	-0.074294351	-0.363333806	0.166077014
ENSG00000123836	*PFKFB2*	5.324714618	-0.012049048	0.064652259	-0.076701307	0.999933887	0.040088355	-0.188818579	0.228906934	0.166132388
ENSG00000132434	*LANCL2*	1.985340944	-0.033471258	-0.110953879	0.077482621	0.999933887	0.368575482	0.639705936	-0.271130454	0.166374001
ENSG00000274528	*AC090970.2*	2.2101638	0.200183731	0.152018189	0.048165542	0.999933887	2.297669292	2.675088304	-0.377419012	0.166402414
ENSG00000008517	*IL32*	6.062189682	0.021446175	-0.011033686	0.032479862	0.999933887	0.260576276	0.413080643	-0.152504367	0.166402414
ENSG00000226312	*CFLAR-AS1*	4.0942492	-0.101022558	-0.083457962	-0.017564596	0.999933887	1.244516426	1.555397472	-0.310881046	0.166885271
ENSG00000111875	*ASF1A*	1.729746154	-0.095156135	-0.021912072	-0.073244063	0.999933887	-0.098907767	0.195542844	-0.294450611	0.166932868
ENSG00000196642	*RABL6*	4.340029741	0.062117072	0.088685019	-0.026567947	0.999933887	-0.029064837	-0.219044063	0.189979226	0.167195941
ENSG00000120594	*PLXDC2*	6.222109369	-0.036358907	-0.033128987	-0.00322992	0.999933887	-0.86866252	-1.100213449	0.231550929	0.167195941
ENSG00000099377	*HSD3B7*	1.737541196	0.048723223	0.085726093	-0.03700287	0.999933887	-0.840546821	-0.53739554	-0.30315128	0.167195941
ENSG00000150776	*NKAPD1*	2.469714522	-0.012349214	-0.038475023	0.026125809	0.999933887	-0.067016823	-0.286465476	0.219448653	0.167195941
ENSG00000168765	*GSTM4*	2.154164385	-0.004834312	-0.039964383	0.035130071	0.999933887	-0.813940895	-1.20902836	0.395087465	0.167195941
ENSG00000093167	*LRRFIP2*	5.327372322	-0.056337819	-0.047704098	-0.008633721	0.999933887	0.093721164	0.234241278	-0.140520114	0.167398057
ENSG00000122779	*TRIM24*	3.674631016	-0.005369875	0.005901876	-0.01127175	0.999933887	-0.774312419	-1.007790609	0.23347819	0.167591722
ENSG00000049769	*PPP1R3F*	0.357697655	0.170822157	0.081370888	0.089451269	0.999933887	-0.063797986	-0.466732223	0.402934237	0.167986553
ENSG00000109323	*MANBA*	6.528274168	-0.043159443	-0.077960817	0.034801374	0.999933887	-0.111028959	0.020815479	-0.131844438	0.167986553
ENSG00000105053	*VRK3*	5.283668129	0.01391266	0.005280814	0.008631845	0.999933887	-0.171080358	-0.028622065	-0.142458293	0.167986553
ENSG00000265972	*TXNIP*	10.512277695	-0.17702616	-0.197161798	0.020135638	0.999933887	-1.03258688	-1.354015515	0.321428635	0.168037676
ENSG00000254236	*AP003550.1*	0.024215259	0.300718149	0.780841915	-0.480123766	0.999933887	0.933927519	1.442062065	-0.508134546	0.168152777
ENSG00000170293	*CMTM8*	0.040564734	0.181903914	0.165601609	0.016302305	0.999933887	-0.309279019	-0.710702607	0.401423588	0.168184299
ENSG00000141560	*FN3KRP*	2.016283148	-0.03716632	-0.111219911	0.074053591	0.999933887	0.050005094	0.293095296	-0.243090201	0.168496568
ENSG00000234338	*AC073349.2*	-1.528467227	0.005325493	0.032345813	-0.02702032	0.999933887	-0.223166978	0.578394364	-0.801561342	0.168496568
ENSG00000125458	*NT5C*	2.025674963	0.103247971	0.074707996	0.028539975	0.999933887	-0.243042021	-0.536484573	0.293442552	0.168625766
ENSG00000132670	*PTPRA*	4.744921874	0.037856943	0.012997131	0.024859812	0.999933887	-0.113568224	-0.287171249	0.173603026	0.168632907
ENSG00000075223	*SEMA3C*	1.668887345	-0.075022802	-0.241659107	0.166636305	0.999933887	-1.002752159	-1.372392505	0.369640345	0.168632907
ENSG00000278231	*AL133342.1*	3.452509851	-0.033275498	0.004501708	-0.037777205	0.999933887	-0.235408509	-0.426307502	0.190898993	0.168632907
ENSG00000198586	*TLK1*	5.529394472	-0.031533926	-0.044791821	0.013257895	0.999933887	-0.505003718	-0.299012634	-0.205991085	0.168632907
ENSG00000139505	*MTMR6*	5.728584314	-0.066083066	-0.116541927	0.050458861	0.999933887	-0.332328988	-0.096863347	-0.235465641	0.168719835
ENSG00000185596	*WASH3P*	2.70463859	0.013962856	0.134912447	-0.120949591	0.999933887	-0.107250106	-0.333206423	0.225956316	0.168719835
ENSG00000203999	*LINC01270*	3.730721436	0.031994255	-0.005764908	0.037759164	0.999933887	0.5881969	0.795556364	-0.207359464	0.168853464
ENSG00000254901	*BORCS8*	4.001338622	-0.07176352	0.00453343	-0.07629695	0.999933887	-0.47241228	-0.646850246	0.174437966	0.168854627
ENSG00000153975	*ZUP1*	2.107948158	-0.068455187	-0.08978608	0.021330893	0.999933887	0.327366788	0.617116291	-0.289749503	0.168854627
ENSG00000132463	*GRSF1*	3.384127107	0.037791937	-0.040226614	0.078018551	0.999933887	0.061448839	-0.146799643	0.208248482	0.168854627

ENSG00000248483	*POU5F2*	0.356912665	0.230306749	0.54674455	-0.316437801	0.999933887	-0.078699941	0.641639865	-0.720339806	0.168886807
ENSG00000170348	*TMED10*	5.337252734	-0.029133123	0.022284368	-0.051417491	0.999933887	-0.420517258	-0.584433981	0.163916723	0.168958981
ENSG00000166794	*PPIB*	3.830802316	0.030919949	0.00158061	0.02933934	0.999933887	-0.108454279	-0.307186972	0.198732693	0.169125077
ENSG00000100023	*PPIL2*	4.033603191	0.065326025	0.08392385	-0.018597825	0.999933887	-0.095654637	-0.248887183	0.153232547	0.169224134
ENSG00000010017	*RANBP9*	5.268612133	0.014913242	0.002386254	0.012526988	0.999933887	-0.0140471	0.106525316	-0.120572416	0.169436978
ENSG00000170088	*TMEM192*	2.796648395	-0.02951041	-0.042797372	0.013286962	0.999933887	-0.344441077	-0.152393217	-0.19204786	0.169436978
ENSG00000181163	*NPM1*	6.95700912	-0.023035073	-0.049137826	0.026102753	0.999933887	0.249595944	0.416822562	-0.167226618	0.169622904
ENSG00000260236	*AC099778.1*	1.527451659	0.154982741	0.233368401	-0.07838566	0.999933887	0.456430201	-0.011213855	0.467644056	0.169807669
ENSG00000036828	*CASR*	-1.342220193	-0.215533354	0.406629513	-0.622162867	0.999933887	1.028319228	1.618891059	-0.59057183	0.170141057
ENSG00000203896	*LIME1*	0.645662941	0.062528037	-0.090966535	0.153494573	0.999933887	-0.199098589	-0.577995038	0.378896449	0.170141057
ENSG00000247199	*AC011346.1*	0.060381407	0.025574829	0.140457196	-0.114882367	0.999933887	0.714982754	1.332345427	-0.617362673	0.170229485
ENSG00000066926	*FECH*	1.716388638	-0.155409073	-0.106594245	-0.048814828	0.999933887	-0.349744626	-0.643193802	0.293449175	0.170258221
ENSG00000080986	*NDC80*	0.44428372	0.034606381	0.051448696	-0.016842315	0.999933887	-0.555508535	-1.074347997	0.518839462	0.170439923
ENSG00000113282	*CLINT1*	5.39119104	-0.020143585	-0.072537429	0.052393844	0.999933887	0.014451374	0.160941467	-0.146490093	0.17052161
ENSG00000198937	*CCDC167*	0.806206901	0.016065715	0.054657306	-0.03859159	0.999933887	0.032871558	0.430301868	-0.39743031	0.17052161
ENSG00000143537	*ADAM15*	3.27448644	0.005463239	-0.080241346	0.085704585	0.999933887	-0.309190624	-0.551397768	0.242207143	0.170616731
ENSG00000173208	*ABCD2*	0.911082131	-0.039312768	-0.274967718	0.23565495	0.999933887	-0.066297161	0.319940127	-0.386237289	0.170638557
ENSG00000153898	*MCOLN2*	2.914891338	0.010716467	-0.014456463	0.02517293	0.999933887	0.094107711	0.296303442	-0.202195731	0.170815074
ENSG00000062582	*MRPS24*	0.049457478	0.064656444	-0.071334418	0.135990862	0.999933887	2.340626823	2.961658755	-0.621031932	0.170890798
ENSG00000174915	*PTDSS2*	1.693374924	0.126475794	0.001449717	0.125026077	0.999933887	0.0459621	-0.271951332	0.317913432	0.171143429
ENSG00000168214	*RBPJ*	7.469689036	-0.07110556	-0.073711231	0.00260567	0.999933887	-0.649864043	-0.460056424	-0.189807619	0.171143429
ENSG00000119906	*SLF2*	3.196934159	-0.049966188	-0.062202703	0.012236515	0.999933887	-0.582287406	-0.920522646	0.33823524	0.171143429
ENSG00000260249	*AC007608.3*	0.32120543	-0.035538739	0.013123423	-0.048662161	0.999933887	0.20126053	0.718793218	-0.517532688	0.171143429
ENSG00000279605	*AC067930.5*	0.976794767	-0.00724727	0.240667007	-0.247914277	0.999933887	-0.802176632	-1.214709658	0.412533026	0.171189159
ENSG00000187953	*PMS2CL*	3.113592145	0.070645786	-0.008811135	0.079456921	0.999933887	-0.238582617	-0.451489159	0.212906542	0.171295225
ENSG00000284052	*AC006460.2*	2.051849237	0.233443224	0.014156732	0.219286491	0.999933887	0.656574402	0.906836537	-0.250262135	0.171446857
ENSG00000106348	*IMPDH1*	7.755210333	0.03649565	-0.003103892	0.039599542	0.999933887	0.724230139	0.913069374	-0.188839236	0.171467481
ENSG00000151883	*PARP8*	7.451058323	-0.044291597	-0.09330855	0.049016953	0.999933887	-0.521796953	-0.726671517	0.204874564	0.171788771
ENSG00000119616	*FCF1*	4.411126394	-0.028639696	0.068917348	-0.097557045	0.999933887	0.24635988	0.411596689	-0.165236809	0.171900575
ENSG00000100926	*TM9SF1*	1.292612121	0.082119156	0.159972264	-0.077853108	0.999933887	-0.023124866	-0.340235103	0.317110236	0.171909809
ENSG00000179454	*KLHL28*	4.112935731	-0.036942749	-0.119921707	0.082978958	0.999933887	-0.411624755	-0.11291862	-0.298706134	0.172017033
ENSG00000158019	*BABAM2*	6.001529588	0.167953621	0.205390773	-0.037437152	0.999933887	0.78001031	1.038456503	-0.258446193	0.172124277
ENSG00000197272	*IL27*	2.478647467	0.106489161	0.089435457	0.017053704	0.999933887	0.295262992	0.661335411	-0.366072419	0.172280103
ENSG00000108509	*CAMTA2*	5.505328765	0.043249368	0.004301525	0.038947842	0.999933887	0.289484589	0.416227794	-0.126743205	0.172280103
ENSG00000136867	*SLC31A2*	3.024695837	0.045827467	-0.061187925	0.107015392	0.999933887	0.420486322	0.658236247	-0.237749925	0.172280103
ENSG00000228804	*AC072022.1*	0.180303678	-0.023298689	0.232459456	-0.255758145	0.999933887	0.605994784	1.130258484	-0.5242637	0.172280103
ENSG00000116667	*C1orf21*	2.826400629	-0.01195602	-0.0077624	-0.00419362	0.999933887	0.042499081	0.246272326	-0.203773245	0.172420599
ENSG00000117242	*PINK1-AS*	0.234941419	0.117810822	-0.106219131	0.224029954	0.999933887	-0.268780995	-0.739538991	0.470757996	0.172639932
ENSG00000134371	*CDC73*	4.996110391	-0.006964085	-0.019121158	0.012157073	0.999933887	-0.29322606	-0.083344306	-0.209881754	0.172676238
ENSG00000120333	*MRPS14*	1.567961948	0.063780009	-0.171528171	0.235308179	0.999933887	-0.166672323	-0.450776648	0.284104325	0.172734468
ENSG00000144589	*STK11IP*	4.304915855	0.083258522	0.039559997	0.043698525	0.999933887	-0.001242584	-0.165486625	0.164244041	0.172756613
ENSG00000197021	*EOLA2*	3.404578414	-0.047108409	-0.021211366	-0.025897043	0.999933887	-0.021089453	-0.230462085	0.209372632	0.172756613
ENSG00000014123	*UFL1*	2.894594901	-0.09075108	-0.193359211	0.10260813	0.999933887	-0.363076156	0.065812645	-0.428888801	0.172756613
ENSG00000163743	*RCHY1*	4.022364394	-0.040498886	-0.149492242	0.108993356	0.999933887	0.043059143	0.230925369	-0.187866226	0.172756613
ENSG00000161835	*TAMALIN*	2.9853007	0.109652561	0.131222016	-0.021569455	0.999933887	0.383166713	0.659032441	-0.275865728	0.172779036
ENSG00000170325	*PRDM10*	3.790180812	-0.032203394	-0.114669045	0.08246565	0.999933887	-0.326609062	-0.513779238	0.187170176	0.172855447
ENSG00000176463	*SLCO3A1*	6.723549235	-0.000506741	0.012553812	-0.013060553	0.999933887	-0.026987406	-0.15369573	0.126708324	0.172855447
ENSG00000114544	*SLC41A3*	2.190289269	-0.012380377	0.038451771	-0.050832147	0.999933887	0.124080769	-0.103486938	0.227567707	0.173271278
ENSG00000241155	*ARHGAP31-A*	-0.514177727	0.174332697	-0.369114351	0.543447048	0.999933887	2.812653789	3.447450386	-0.634796596	0.17327716
ENSG00000198829	*SUCNR1*	5.154505779	-0.150894327	-0.165921656	0.015027329	0.999933887	-1.29408358	-0.977962588	-0.316120992	0.173323939
ENSG00000197020	*ZNF100*	4.29303886	-0.060771795	-0.071568933	0.010797138	0.999933887	-0.326822847	0.041583357	-0.368406204	0.173411624
ENSG00000079263	*SP140*	5.968425512	0.023366888	-0.027628544	0.050995431	0.999933887	-0.16952754	-0.015657986	-0.153869554	0.173519136
ENSG00000287011	*AC003077.1*	0.043917954	-0.10483928	0.43430625	-0.53914553	0.999933887	0.401170218	0.807658062	-0.406487844	0.173519136
ENSG00000197381	*ADARB1*	4.067770607	0.03143532	0.084894418	-0.053459098	0.999933887	-0.373976772	-0.530888711	0.156911939	0.17377709
ENSG00000164007	*CLDN19*	-0.567447497	0.207135868	0.574129394	-0.366993526	0.999933887	0.297923223	0.800340151	-0.502416928	0.173839322
ENSG00000028203	*VEZT*	2.441495877	-0.069666685	-0.411811341	0.342144656	0.944094537	0.049412403	0.328849526	-0.279437123	0.173958452
ENSG00000260367	*AC109460.1*	1.258986061	0.05133462	0.143138601	-0.091803981	0.999933887	0.333501512	0.66174683	-0.328245318	0.173958452
ENSG00000214331	*AC009053.1*	3.348365461	-0.016972667	-0.107425117	0.09045245	0.999933887	-0.062901453	0.131960907	-0.19486236	0.173958452
ENSG00000285921	*AP000779.1*	-0.971631252	0.061969587	0.684713904	-0.622744317	0.999933887	0.739292834	1.322243684	-0.58295085	0.173958452
ENSG00000152683	*SLC30A6*	2.760945817	0.009425923	0.002313608	0.007112315	0.999933887	-0.097852164	0.085506491	-0.183358655	0.173958452
ENSG00000147162	*OGT*	7.990133434	-0.044930006	-0.035775475	-0.009154531	0.999933887	-0.084490675	-0.210495376	0.126004701	0.173985245
ENSG00000167863	*ATP5PD*	3.729991456	-0.07639306	0.004449876	-0.080842936	0.999933887	-0.128435788	-0.31492804	0.186492252	0.174052063
ENSG00000139324	*TMTC3*	0.733129559	-0.103428292	-0.37430475	0.270876458	0.999933887	-0.334162641	0.179799768	-0.513962408	0.174302672
ENSG00000028310	*BRD9*	4.983004942	-0.021330102	-0.018055744	-0.003274358	0.999933887	-0.0487657	-0.190363242	0.141597543	0.174493198
ENSG00000082153	*BZW1*	6.533477806	-0.049794588	-0.131612089	0.081817501	0.999933887	0.078981155	0.222618591	-0.143637436	0.174525816
ENSG00000008735	*MAPK8IP2*	-0.578159324	0.056772892	-0.022434843	0.079207735	0.999933887	-0.999853997	-1.535703091	0.535849094	0.174581823
ENSG00000184988	*TMEM106A*	1.726811353	-0.107851915	0.357540199	-0.465392114	0.999933887	0.745843981	1.055738233	-0.309894252	0.174954733
ENSG00000197599	*CCDC154*	3.074701434	0.231553512	0.330611383	-0.099057871	0.999933887	0.56553175	0.83578364	-0.270251889	0.175348201
ENSG00000260078	*AC007342.3*	4.866318222	-0.107766711	-0.113456358	0.005689648	0.999933887	-0.600629756	-0.838264492	0.237634735	0.175434428
ENSG00000171174	*RBKS*	4.783055551	0.385312577	0.224536636	0.160775941	0.999933887	1.082647059	1.6014202	-0.518773142	0.175478339
ENSG00000183283	*DAZAP2*	9.956114324	-0.005797227	-0.015550024	0.009752797	0.999933887	0.127980123	0.249752436	-0.121772312	0.175478339
ENSG00000232774	*AL355916.1*	-0.477952261	-0.075459097	-0.14447892	0.069019823	0.999933887	0.613340653	1.264756338	-0.651415685	0.175783702
ENSG00000260271	*AL132996.1*	-0.28335596	0.46792952	0.482977756	-0.015048236	0.999933887	0.575230906	1.002322075	-0.427091169	0.175955849
ENSG00000240230	*COX19*	3.582877234	-0.061250618	0.031128451	-0.092379069	0.999933887	-0.233655969	-0.437751973	0.204096004	0.175955849
ENSG00000061987	*MON2*	5.651692099	0.027151842	0.035040409	-0.007888566	0.999933887	-0.199698772	0.023838576	-0.223537349	0.175998577
ENSG00000218739	*CEBPZOS*	2.030272082	-0.002057965	-0.064202898	0.062144933	0.999933887	-0.187919579	0.109344509	-0.297264089	0.176111578
ENSG00000135801	*TAF5L*	3.16434771	-0.068715205	0.028658634	-0.097373839	0.999933887	-0.019413885	-0.202330472	0.182916587	0.176140951
ENSG00000171101	*SIGLEC17P*	0.457197775	0.268359015	-0.136217052	0.404576067	0.944094537	-0.017558634	0.398210029	-0.415768663	0.176225663
ENSG00000260475	*AL353719.1*	-0.665367825	0.159465204	0.227009439	-0.067544235	0.999933887	0.016667696	0.915574683	-0.898906987	0.176225663
ENSG00000131016	*AKAP12*	0.882509179	0.171220403	-0.358395017	0.529615421	0.999933887	-1.186380897	-1.563695999	0.377315102	0.176225663
ENSG00000214174	*AMZ2P1*	4.392394319	0.11397349	0.036984458	0.076989032	0.999933887	0.503184188	0.706496026	-0.203311838	0.176374486
ENSG00000286302	*AC084724.1*	-1.069427725	-0.179634575	-0.028613899	-0.151020676	0.999933887	-0.176950554	0.7365537	-0.913504254	0.176584169
ENSG00000125952	*MAX*	6.352412625	-0.025014594	0.00492264	-0.029937234	0.999933887	-0.498430811	-0.641033806	0.142602995	0.176811193
ENSG00000274736	*CCL23*	-0.29898285	0.033078283	-0.169087896	0.202166179	0.999933887	0.600981679	1.317934389	-0.71695271	0.177067816
ENSG00000138756	*BMP2K*	4.453322627	-0.013311387	0.038087316	-0.051398703	0.999933887	-0.925891884	-1.135674927	0.209783043	0.177381562
ENSG00000229236	*TTTY10*	-3.83077962	0.128762019	0.312187821	-0.183425803	0.999933887	-0.501920466	0.184622592	-0.686543058	0.177534315
ENSG00000100294	*MCAT*	2.952646283	0.17410915	0.065690895	0.108418255	0.999933887	0.236531402	0.508024202	-0.2714928	0.177633924
ENSG00000131188	*PRR7*	5.972431432	0.197424939	0.139629399	0.057795541	0.999933887	1.002234727	1.220566619	-0.218331893	0.177873913
ENSG00000274070	*CASTOR2*	2.942883207	-0.016736406	-0.058884038	0.042147632	0.999933887	-0.463985802	-0.69153247	0.227546668	0.178056519
ENSG00000133119	*RFC3*	0.785796998	-0.305788025	-0.068667551	-0.237120475	0.999933887	-0.059768836	0.491134271	-0.550903107	0.178084379
ENSG00000143924	*EML4*	7.520737537	-0.06468975	-0.03664517	-0.02804458	0.999933887	-0.038784197	0.194243489	-0.233027686	0.178084379
ENSG00000228817	*BACH1-IT2*	2.551703061	0.103561397	0.054267863	0.049293534	0.999933887	0.059168396	0.296682275	-0.237513879	0.17810489
ENSG00000072786	*STK10*	9.616683992	0.002004767	-0.00939541	0.011400178	0.999933887	0.271797917	0.375703899	-0.103905982	0.17810489
ENSG00000156831	*NSMCE2*	3.978863249	0.053786466	0.06183083	-0.008044364	0.999933887	0.310171443	0.502409292	-0.192237848	0.178147436
ENSG00000110880	*CORO1C*	7.994146465	-0.02729961	-0.04555386	0.018254249	0.999933887	-0.832022994	-0.640749074	-0.19127392	0.178157581
ENSG00000185787	*MORF4L1*	7.42413206	-0.038498211	-0.080127794	0.041629584	0.999933887	-0.182306255	-0.069412187	-0.112894068	0.178313468
ENSG00000147050	*KDM6A*	6.740721469	0.019379124	0.010098599	0.009280525	0.999933887	0.031854371	0.205320746	-0.173466375	0.178313468
ENSG00000010318	*PHF7*	1.338482601	0.034922316	-0.087713419	0.122635735	0.999933887	-0.270884263	-0.597925835	0.327041572	0.178313468
ENSG00000239732	*TLR9*	-0.588572654	-0.127387464	0.143259345	-0.270646809	0.999933887	-0.28651211	-1.086737323	0.800225213	0.178389187
ENSG00000166783	*MARF1*	7.696292775	-0.04652181	-0.066429402	0.019907592	0.999933887	-0.129767773	0.020941915	-0.150709688	0.178440911
ENSG00000100319	*ZMAT5*	2.012507125	-0.064765619	0.120246758	-0.185012377	0.999933887	-0.462361023	-0.786407045	0.324046021	0.178609502
ENSG00000177854	*TMEM187*	1.400391199	-0.00888758	-0.260344847	0.251457267	0.999933887	0.686164432	0.981769603	-0.29560517	0.178739202
ENSG00000275791	*TRBV10-3*	-0.339504024	0.150381547	0.062801484	0.087580063	0.999933887	0.187296373	0.729165112	-0.541868738	0.178749941
ENSG00000204348	*DXO*	4.088379671	0.099298347	0.039995769	0.059302578	0.999933887	0.536879992	0.773482581	-0.236602589	0.17875345
ENSG00000109103	*UNC119*	7.046232332	0.079702333	0.024314978	0.055387355	0.999933887	1.479700126	1.687413959	-0.207713833	0.17875345

ENSG00000177989	ODF3B	6.376780037	0.172970771	0.178212189	-0.005241419	0.999933887	-0.077253213	-0.310512681	0.233259468	0.179062202
ENSG00000065989	PDE4A	2.633718399	-0.064717127	0.056881409	-0.121598536	0.999933887	0.124631033	0.390920239	-0.266289206	0.179100033
ENSG00000184220	CMSS1	0.315331842	-0.088557121	-0.083397911	-0.00515921	0.999933887	0.120868235	0.437883071	-0.317014836	0.179121649
ENSG00000134602	STK26	5.610555809	-0.013804754	-0.019278369	0.005473615	0.999933887	-0.14769771	0.073812206	-0.221509917	0.179277364
ENSG00000108055	SMC3	4.0600093	-0.10260886	-0.127839803	0.025230943	0.999933887	-0.843246817	-0.493752413	-0.349494404	0.179495832
ENSG00000163406	SLC15A2	0.719994677	-0.17470852	-0.01486629	-0.15984223	0.999933887	0.005618467	0.366931921	-0.361313454	0.179605983
ENSG00000251323	LINC02728	1.437673425	0.213977451	0.415740545	-0.201763094	0.999933887	2.470800605	2.964190381	-0.493389777	0.179859648
ENSG00000147548	NSD3	6.089558109	-0.018856483	-0.065964576	0.047108093	0.999933887	-0.470602279	-0.640048033	0.169445754	0.180205846
ENSG00000203865	ATP1A1-AS1	1.326045524	0.023314509	0.086193231	-0.062878722	0.999933887	0.312445028	0.716715036	-0.404270008	0.180285708
ENSG00000106479	ZNF862	3.249981881	-0.011677418	-0.014835292	0.003157874	0.999933887	-0.019694508	-0.226581116	0.206886608	0.180399105
ENSG00000107020	PLGRKT	1.662012881	0.051312019	-0.05708135	0.108393369	0.999933887	0.341490819	0.711678171	-0.370187353	0.180759907
ENSG00000133028	SCO1	2.89554024	-0.032504243	0.020841553	-0.053345796	0.999933887	-0.059900149	-0.283514351	0.223614202	0.180759907
ENSG00000172000	ZNF556	-2.853330483	-0.149840275	-0.529393997	0.379553721	0.999933887	0.543756038	1.301532825	-0.757776787	0.180759907
ENSG00000181827	RFX7	3.189730406	0.076862112	-0.190882673	0.267744785	0.999933887	-0.262447714	0.104889573	-0.367337287	0.180765703
ENSG00000144426	NBEAL1	1.559591248	0.059478303	0.066589472	-0.007111169	0.999933887	0.225582799	0.750997981	-0.525415182	0.180847507
ENSG00000103091	WDR59	3.66041074	0.012111577	-0.018608998	0.030720574	0.999933887	-0.050782698	-0.216313711	0.165531013	0.181059169
ENSG00000211753	TRBV28	0.6315717	0.054778486	0.232843492	-0.178065006	0.999933887	0.143803878	-0.201593825	0.345397704	0.181119605
ENSG00000228784	LINC00954	3.576443095	0.078943383	0.019344797	0.059598586	0.999933887	1.183571075	1.465631157	-0.282060082	0.181333979
ENSG00000184014	DENND5A	9.892119275	-0.037084846	0.009331009	-0.046415856	0.999933887	1.102781914	1.246631291	-0.143849376	0.181333979
ENSG00000119685	TTLL5	2.210058384	0.028988714	-0.096858809	0.125847523	0.999933887	-0.194080093	0.131814221	-0.325894314	0.181333979
ENSG00000258907	PSMB3P1	2.157245527	0.22740673	0.471004721	-0.243597991	0.999933887	1.196200287	1.483926363	-0.287726076	0.181382578
ENSG00000068323	TFE3	8.357767755	0.038739989	0.019775298	0.018964691	0.999933887	0.19955477	0.331040433	-0.131485663	0.181382578
ENSG00000172123	SLFN12	1.850002766	-0.033650061	0.07258509	-0.106235151	0.999933887	-0.448456192	-0.760520155	0.312063963	0.181447458
ENSG00000261737	CLCA4-AS1	0.450152391	0.005723942	-0.032793004	0.038516946	0.999933887	0.525675934	0.952077758	-0.426401824	0.181502439
ENSG00000108799	EZH1	6.195777161	-0.042332222	0.00830109	-0.050633312	0.999933887	-0.323206335	-0.442755608	0.119549273	0.181519669
ENSG00000161011	SQSTM1	10.502997176	0.034663942	0.026990082	0.007673861	0.999933887	0.540812299	0.681131759	-0.14031946	0.181519669
ENSG00000163145	C1QTNF7	-0.572732477	0.021730635	0.358173437	-0.336442803	0.999933887	0.047389989	0.716544501	-0.669154512	0.181519669
ENSG00000273559	CWC25	7.866754096	0.010694945	0.012899751	-0.002204806	0.999933887	0.598264228	0.739558054	-0.141293826	0.181587107
ENSG00000103145	HCFC1R1	1.688180614	-0.020210277	-0.043531855	0.023321578	0.999933887	-0.38181192	-0.665131793	0.283319873	0.181674881
ENSG00000086061	DNAJA1	7.245092205	-0.018454669	-0.11081259	0.092357921	0.999933887	0.700202767	0.866635772	-0.166433005	0.181764186
ENSG00000166225	FRS2	3.471800598	-0.062998065	-0.032102296	-0.030895769	0.999933887	0.01071239	0.233639053	-0.222926663	0.181929992
ENSG00000135870	RC3H1	6.511335337	-0.054885762	-0.017215	-0.037670762	0.999933887	-0.705683487	-0.476994429	-0.228689058	0.181929992
ENSG00000119431	HDHD3	1.682961839	-0.064772537	-0.049229026	-0.015543511	0.999933887	0.175687388	-0.081624736	0.257312124	0.181929992
ENSG00000243335	KCTD7	5.356766768	0.034468516	-0.020012559	0.054481075	0.999933887	0.189622812	0.340070113	-0.150447301	0.181929992
ENSG00000073111	MCM2	3.062284711	0.013787219	0.080591129	-0.066803911	0.999933887	0.704947055	0.935204194	-0.230257139	0.181929992
ENSG00000258704	SRP54-AS1	0.158428895	-0.019797523	0.516893711	-0.536691234	0.999933887	1.04864719	1.513869866	-0.465222676	0.181929992
ENSG00000198081	ZBTB14	2.229574643	-0.006892281	-0.145004316	0.138112034	0.999933887	0.031315933	-0.202230199	0.233546132	0.181929992
ENSG00000139697	SBNO1	6.285749271	-0.078688735	-0.132120018	0.053431283	0.999933887	-0.226252932	-0.00815448	-0.218098452	0.181959553
ENSG00000116120	FARSB	1.463143201	-0.142338568	-0.167944506	0.025605938	0.999933887	0.245353743	0.515775603	-0.270421859	0.181974864
ENSG00000101347	SAMHD1	5.47958537	-0.026503228	-0.07596972	0.049466492	0.999933887	-0.008359597	-0.157290263	0.148930666	0.182142553
ENSG00000253795	MTND1P5	-1.417281792	0.445227633	0.245394028	0.199833605	0.999933887	2.237472123	3.157872024	-0.920399901	0.182209603
ENSG00000105639	JAK3	6.86800901	0.038707133	0.038195905	0.000511228	0.999933887	0.24633491	0.106707047	0.139627863	0.182231713
ENSG00000104805	NUCB1	6.328768563	0.056676656	0.027923852	0.028752805	0.999933887	-0.176187656	-0.333230152	0.157042497	0.182320843
ENSG00000101236	RNF24	7.267335991	-0.059756647	-0.011366468	-0.048390179	0.999933887	-0.858286022	-1.114309146	0.256023125	0.182370404
ENSG00000135148	TRAFD1	5.651181026	-0.011862147	0.013639009	-0.025501156	0.999933887	-0.309831896	-0.504694507	0.19486261	0.182499692
ENSG00000275070	Metazoa_SRP	0.59090211	0.081978883	-0.046619567	0.12859845	0.999933887	-0.063742098	0.419967378	-0.483709476	0.182837464
ENSG00000161955	TNFSF13	1.553114066	-0.003977322	0.197711939	-0.201689261	0.999933887	0.071749371	-0.237158518	0.308907889	0.183399783
ENSG00000197771	MCMBP	4.597409762	-0.026879001	0.076874398	-0.103753399	0.999933887	0.032058858	-0.128015985	0.160074844	0.183523858
ENSG00000023191	RNH1	5.506951155	0.069697268	0.049175514	0.020521754	0.999933887	-0.132350582	-0.301597	0.169246418	0.183707113
ENSG00000091262	ABCC6	1.014959991	-0.099501249	-0.194903379	0.095402131	0.999933887	-0.41883731	0.013916256	-0.432753565	0.183760983
ENSG00000257743	MGAM2	2.956233852	0.03162847	-0.160740534	0.192369004	0.999933887	-0.168389083	0.202548205	-0.370937289	0.183760983
ENSG00000156345	CDK20	3.228940981	0.129646417	0.172892173	-0.043245755	0.999933887	-0.015582363	0.177223408	-0.192805771	0.183768477
ENSG00000131748	STARD3	6.266426256	0.012015929	0.038684408	-0.026668479	0.999933887	-0.128833308	-0.259352568	0.13051926	0.183768477
ENSG00000116260	QSOX1	6.832929673	0.013755866	0.008936274	0.004819592	0.999933887	0.06917529	0.222868721	-0.153693431	0.183768477
ENSG00000158874	APOA2	2.23070346	0.108328676	0.079806799	0.028521877	0.999933887	0.753790705	1.139090991	-0.385300286	0.18382937
ENSG00000107249	GLIS3	-0.04978007	-0.274934666	-0.00620992	-0.268724746	0.999933887	-0.227897518	-0.67281915	0.444921632	0.183837741
ENSG00000136244	IL6	0.016161984	0.760927628	1.458129661	-0.697202034	0.999933887	3.561017055	4.486722749	-0.925705694	0.183837741
ENSG00000230989	HSBP1	4.85004988	-0.02980246	-0.094846511	0.065044051	0.999933887	-0.433823913	-0.614048696	0.180224783	0.183837741
ENSG00000174175	SELP	0.51036522	0.038622809	-0.083318797	0.121941606	0.999933887	-0.385525445	-0.830535817	0.445010371	0.183837741
ENSG00000101849	TBL1X	6.808825556	-0.053802895	-0.026267423	-0.027535472	0.999933887	-0.220559954	-0.343961559	0.123401605	0.18384731
ENSG00000198131	ZNF544	1.118511069	-0.075647845	-0.045295677	-0.030352168	0.999933887	-0.060947652	-0.391835483	0.330887831	0.18384731
ENSG00000076555	ACACB	2.657004719	0.130113732	-0.067748508	0.19786224	0.999933887	-0.05927546	0.166342434	-0.225617894	0.183856506
ENSG00000138641	HERC3	1.114431943	0.107056633	0.137017608	-0.029960974	0.999933887	-0.279184413	-0.794280807	0.515096393	0.183856506
ENSG00000171219	CDC42BPG	2.705915289	0.076185742	0.027634174	0.048551568	0.999933887	0.5610029	0.888613609	-0.327610709	0.183856506
ENSG00000064545	TMEM161A	1.618741653	-0.061799436	-0.073951205	0.01215177	0.999933887	0.197904127	0.474138066	-0.276233938	0.183856506
ENSG00000125352	RNF113A	3.093779466	-0.026266869	-0.20761094	0.181344071	0.999933887	-0.373647016	-0.613771128	0.240124112	0.183856506
ENSG00000137842	TMEM62	2.143357386	-0.036083638	0.064151871	-0.100235509	0.999933887	0.076286121	-0.330536925	0.406823046	0.184455379
ENSG00000183150	GPR19	-0.303793775	-0.188357167	-0.246556474	0.058199307	0.999933887	-0.73252546	-1.275609811	0.543084351	0.184524022
ENSG00000197093	GAL3ST4	1.234189757	0.022836377	-0.048266327	0.071102704	0.999933887	-0.022353672	-0.454500853	0.432147182	0.184592676
ENSG00000186350	RXRA	8.10906012	-0.013262276	0.025937052	-0.039199328	0.999933887	-0.747633421	-0.936955816	0.189322395	0.184856796
ENSG00000156709	AIFM1	1.849471844	-0.092827915	-0.001646134	-0.091181781	0.999933887	-0.095197255	-0.359571303	0.264374048	0.184869261
ENSG00000113810	SMC4	3.904434	-0.050213273	-0.309237628	0.259024355	0.999933887	-0.44155308	0.110534358	-0.552087438	0.184869261
ENSG00000173264	GPR137	3.949200184	0.058983481	0.014374509	0.044608972	0.999933887	0.456570473	0.625022866	-0.168452394	0.185187362
ENSG00000083812	ZNF324	3.425178011	0.104493294	0.001587371	0.102905923	0.999933887	-0.042824057	-0.249200709	0.206376652	0.185717593
ENSG00000237938	AL450998.2	2.992869723	-0.08609283	-0.080403896	-0.005688934	0.999933887	0.286671208	0.467370181	-0.180698974	0.185771243
ENSG00000175105	ZNF654	4.01722546	-0.172873457	-0.195695789	0.022822332	0.999933887	-0.283222593	0.079789989	-0.363012581	0.186131254
ENSG00000243156	MICAL3	1.894903314	0.184980252	0.071267236	0.113713017	0.999933887	0.409564327	0.733487214	-0.323922887	0.186205687
ENSG00000111906	HDDC2	1.624229636	0.037773464	-0.05848795	0.096261414	0.999933887	-0.07602063	0.145569977	-0.221590607	0.186205687
ENSG00000196878	LAMB3	6.268445034	0.059899626	0.022208523	0.037691103	0.999933887	1.707740514	1.938203041	-0.230462528	0.186491974
ENSG00000163754	GYG1	4.059260335	-0.048561699	-0.092640957	0.044079259	0.999933887	-0.28369017	-0.459328847	0.175638678	0.186601883
ENSG00000198673	TAFA2	3.39827038	0.052295018	0.103622136	-0.051327118	0.999933887	0.265167859	0.508766141	-0.243598281	0.186601883
ENSG00000131381	RBSN	4.868285704	-0.00279801	-0.015774458	0.012976448	0.999933887	-0.086219011	0.055613028	-0.141832039	0.186601883
ENSG00000183878	UTY	-2.683781403	0.109669248	0.122254147	-0.012584899	0.999933887	-0.319837425	-0.035853184	-0.283984241	0.186604325
ENSG00000171456	ASXL1	6.113512006	-0.011798731	-0.015231033	0.003432302	0.999933887	0.173806906	0.287898762	-0.114091856	0.186606524
ENSG00000166126	AMN	-0.047544036	0.015052588	0.273533766	-0.258481178	0.999933887	1.190881919	1.672872595	-0.481990677	0.186606524
ENSG00000133275	CSNK1G2	8.072552564	0.070227231	0.063049572	0.007177659	0.999933887	-0.004053618	-0.152710171	0.148656553	0.18668151
ENSG00000163219	ARHGAP25	9.10721606	-0.086194202	-0.042192046	-0.044002156	0.999933887	-0.635029599	-0.449932322	-0.185097276	0.18672216
ENSG00000235724	AC009299.2	1.630510751	-0.17302697	0.083591172	-0.256618141	0.999933887	0.687258717	1.211104629	-0.523845912	0.18672216
ENSG00000107960	STN1	2.973232091	0.068541762	-0.029634923	0.098176685	0.999933887	0.133499262	-0.158864822	0.292364083	0.18672216
ENSG00000089053	ANAPC5	5.211005677	-0.013104439	0.005515646	-0.018620085	0.999933887	0.22036077	0.351764464	-0.131403694	0.186901129
ENSG00000101161	PRPF6	5.50687943	0.002453395	-0.023304683	0.025758078	0.999933887	-0.160361064	-0.308501138	0.148140073	0.187038286
ENSG00000081014	AP4E1	2.135478254	-0.060028727	-0.059775437	-0.00025329	0.999933887	0.071421914	0.347291856	-0.275869942	0.187082452
ENSG00000280832	GSEC	2.574466017	-0.045016529	0.201850723	-0.246867252	0.999933887	0.413684968	0.660188868	-0.2465039	0.187138536
ENSG00000234797	RPS3AP6	0.339134836	-0.032554825	-0.494045976	0.461491151	0.999933887	0.142522601	0.571973193	-0.429450591	0.187328357
ENSG00000236859	NIFK-AS1	0.66874756	-0.042607418	0.071223749	-0.113831167	0.999933887	-0.09771223	-0.444968017	0.347255787	0.187445303
ENSG00000138785	INTS12	2.846597745	-0.011913276	-0.13501573	0.123102454	0.999933887	-0.037847521	0.172528159	-0.210375681	0.187465582
ENSG00000173597	SULT1B1	3.694438434	0.003007335	0.023868754	-0.020861419	0.999933887	-0.226286322	-0.625228322	0.398942	0.187465582
ENSG00000238160	LINC02863	2.002964814	-0.024458266	0.025695178	-0.050153444	0.999933887	-0.126555955	-0.537167098	0.410611142	0.187654811
ENSG00000259600	AC066616.2	3.790440321	0.342918097	0.714833014	-0.371914918	0.999933887	2.000964737	2.609509898	-0.608545162	0.187825005
ENSG00000182199	SHMT2	2.788134499	0.027919774	0.14546985	-0.117550075	0.999933887	0.383853293	0.617659435	-0.233806143	0.187916883
ENSG00000039123	MTREX	3.296831769	-0.13098811	-0.076106268	-0.054881842	0.999933887	-0.17352027	0.088430194	-0.261950464	0.187959522
ENSG00000204856	FAM216A	-0.272856561	0.076993618	-0.317135764	0.394129382	0.999933887	-0.060718833	-0.510718526	0.449999692	0.187959522
ENSG00000069188	SDK2	-0.619057669	-0.105790004	0.104089719	-0.209879723	0.999933887	-0.491654756	0.225924329	-0.717579085	0.187971885
ENSG00000135074	ADAM19	5.764349831	0.000394853	0.035253069	-0.034858216	0.999933887	0.020390036	-0.104771849	0.125161884	0.188341703
ENSG00000240288	GHRLOS	1.424094059	0.115959691	-0.074641965	0.190601656	0.999933887	1.674244089	2.112100446	-0.437856357	0.188595294

ENSG00000261220	*AC103706.1*	2.175309903	0.021612376	0.03773597	-0.016123594	0.999933887	0.948810338	1.236702235	-0.287891897	0.188595294
ENSG00000136541	*ERMN*	3.484827207	-0.146350954	-0.041210901	-0.105140053	0.999933887	-0.541499769	-0.229627455	-0.311872314	0.188671181
ENSG00000152061	*RABGAP1L*	5.353477011	0.021144371	-0.0754234	0.096567771	0.999933887	-0.685023473	-0.926773227	0.241749754	0.188774881
ENSG00000127837	*AAMP*	4.044647571	0.074028624	0.102365564	-0.028336941	0.999933887	0.101306895	-0.065779031	0.167085926	0.189261209
ENSG00000110237	*ARHGEF17*	-0.366256938	-0.034094834	0.05683234	-0.090927174	0.999933887	-0.314754215	-0.946390525	0.631636309	0.18941553
ENSG00000148288	*GBGT1*	1.951347101	0.097538154	0.144111452	-0.046573298	0.999933887	-0.229407874	-0.507274806	0.277866933	0.189431182
ENSG00000108468	*CBX1*	3.160556198	0.018420545	-0.133008241	0.151428786	0.999933887	0.021531582	-0.233302428	0.25483401	0.189431182
ENSG00000110497	*AMBRA1*	4.143314763	-0.005449251	0.081931024	-0.087380275	0.999933887	-0.07802557	0.064197238	-0.142222809	0.189431182
ENSG00000211456	*SACM1L*	4.523775604	0.01280508	0.020113495	-0.007308415	0.999933887	-0.468486992	-0.703230887	0.234743896	0.189449711
ENSG00000053918	*KCNQ1*	4.076907808	0.080361664	0.029269947	0.051091718	0.999933887	-0.057708298	-0.279039552	0.221331253	0.189458212
ENSG00000125826	*RBCK1*	6.682452775	0.037828127	0.019101291	0.018726836	0.999933887	-0.287875829	-0.43171363	0.143837801	0.190033391
ENSG00000119698	*PPP4R4*	-0.898660693	-0.124956213	0.111116571	-0.236072784	0.999933887	2.359235975	2.997889973	-0.638653999	0.190161273
ENSG00000224187	*LINC01991*	0.853918925	0.226047171	0.378382195	-0.152335023	0.999933887	1.93712091	2.37482496	-0.43770405	0.190234321
ENSG00000166478	*ZNF143*	5.106911191	-0.022337575	-0.035236861	0.012899286	0.999933887	-0.234339057	-0.096941496	-0.137397562	0.190234321
ENSG00000163848	*ZNF148*	5.157257564	-0.070732334	-0.043974031	-0.026758304	0.999933887	-0.823483019	-0.557770957	-0.265712062	0.190527174
ENSG00000162734	*PEA15*	3.6410487	0.008910731	-0.171200736	0.180111467	0.999933887	0.329046625	0.505607387	-0.176560762	0.190527174
ENSG00000159899	*NPR2*	-1.028326909	-0.418416607	-0.211139006	-0.207277601	0.999933887	0.341155881	-0.190062925	0.531218806	0.190543255
ENSG00000134460	*IL2RA*	1.266715303	-0.079350256	0.191440499	-0.270790756	0.999933887	0.99675974	1.395325381	-0.398565642	0.190543255
ENSG00000117682	*DHDDS*	3.363444088	-0.025099119	-0.062466294	0.037367175	0.999933887	-0.178941654	-0.342490388	0.163548734	0.190543255
ENSG00000012223	*LTF*	3.300143326	-0.016795668	-0.076280847	0.059485179	0.999933887	0.580836344	0.780861145	-0.2000248	0.190543255
ENSG00000170027	*YWHAG*	5.551651615	0.002163772	0.027470149	-0.025306376	0.999933887	-0.480502151	-0.629824152	0.149322001	0.190543255
ENSG00000262001	*DLGAP1-AS2*	3.815569034	-0.083652627	0.171896539	-0.255549166	0.981460244	0.444911639	0.634146679	-0.18923504	0.190822664
ENSG00000005156	*LIG3*	3.942474639	0.012734445	-0.105599875	0.11833432	0.999933887	0.43398652	0.615513907	-0.181527387	0.190822664
ENSG00000011566	*MAP4K3*	1.234893629	-0.045955092	-0.007510109	-0.038444983	0.999933887	-0.536811686	-0.891870775	0.355059089	0.190843641
ENSG00000162775	*RBM15*	4.427260877	-0.003225333	0.060817775	-0.064043108	0.999933887	-0.123009001	-0.267406119	0.144397118	0.191053822
ENSG00000070882	*OSBPL3*	3.475278207	-0.043527461	-0.049756039	0.006228578	0.999933887	-0.026488024	0.13532325	-0.161811274	0.191177979
ENSG00000102760	*RGCC*	5.299659925	0.022817036	-0.019733055	0.042550091	0.999933887	-0.164095504	-0.323278382	0.159182878	0.191586116
ENSG00000100376	*FAM118A*	4.250697881	-0.051116807	-0.025209937	-0.02590687	0.999933887	0.109074707	-0.036977962	0.146052669	0.191591718
ENSG00000007968	*E2F2*	3.965358268	-0.081538495	-0.116235771	0.034697276	0.999933887	-0.401816509	-0.184860803	-0.216955707	0.191591718
ENSG00000153113	*CAST*	7.587309754	-0.002370379	0.010103365	-0.012473744	0.999933887	-0.375894998	-0.221295567	-0.154599431	0.191669166
ENSG00000185838	*GNB1L*	1.117320829	-0.100949466	0.162158108	-0.263107574	0.999933887	-0.063252996	-0.430132361	0.366879365	0.191675662
ENSG00000245149	*RNF139-AS1*	0.556662892	-0.049947918	-0.136335404	0.086387486	0.999933887	0.165543935	-0.303976501	0.469520436	0.191940765
ENSG00000166147	*FBN1*	0.25619395	-0.191256911	0.065625606	-0.256882517	0.999933887	0.550839619	1.045397753	-0.494558134	0.192033427
ENSG00000272384	*AC016405.3*	-0.957809547	0.259650856	0.532398624	-0.272747768	0.999933887	2.441712018	3.327446891	-0.885734873	0.192146618
ENSG00000196510	*ANAPC7*	2.984354176	-0.090904161	-0.025057741	-0.06584642	0.999933887	-0.180442228	-0.352577911	0.172135683	0.192219758
ENSG00000213588	*ZBTB9*	0.862028466	0.052019348	-0.018986191	0.071005539	0.999933887	0.10318981	0.416373788	-0.313183978	0.192365686
ENSG00000105429	*MEGF8*	2.128004162	0.077208586	0.090327659	-0.013119073	0.999933887	-0.020724978	0.235271919	-0.255996896	0.192414872
ENSG00000125746	*EML2*	4.214535944	0.013657983	0.111287683	-0.097629699	0.999933887	0.258250112	0.46790859	-0.209658478	0.192586484
ENSG00000267385	*AC011498.4*	-1.084610637	-0.122921618	0.104539333	-0.22746095	0.999933887	-0.142928175	0.82130449	-0.964232665	0.192920622
ENSG00000247556	*OIP5-AS1*	4.050477191	-0.087639361	-0.070016086	-0.017623274	0.999933887	-0.126744876	-0.297121986	0.170377109	0.193191833
ENSG00000185453	*ZSWIM9*	1.261518116	0.150398448	0.1165768	0.033821648	0.999933887	-0.025345089	-0.403046385	0.377701296	0.193191833
ENSG00000113240	*CLK4*	5.231118164	-0.09244776	-0.138386655	0.045938895	0.999933887	0.084288836	0.342820156	-0.258531319	0.193191833
ENSG00000002549	*LAP3*	5.473066924	-0.00245799	-0.062430155	0.059972165	0.999933887	-0.324098692	-0.518221474	0.194122781	0.19353446
ENSG00000164398	*ACSL6*	0.325665777	-0.039296087	-0.046801894	0.007505808	0.999933887	-0.283592449	0.133934545	-0.417526995	0.193726321
ENSG00000087274	*ADD1*	6.844693262	-0.043723531	-0.005072221	-0.03865131	0.999933887	-0.157270405	-0.277290318	0.120019913	0.193834652
ENSG00000258667	*HIF1A-AS3*	4.619712846	0.084588197	0.318560982	-0.233972785	0.999933887	1.009466909	1.35464718	-0.345180271	0.193842009
ENSG00000135253	*KCP*	0.633048877	0.14848317	0.332604508	-0.184121338	0.999933887	0.417728876	0.813013331	-0.395284455	0.193977764
ENSG00000197044	*ZNF441*	1.703447733	-0.291757411	-0.160433869	-0.131323542	0.999933887	-0.389009832	0.002180526	-0.391190358	0.194049511
ENSG00000196405	*EVL*	6.61292162	0.006332811	0.054608653	-0.048275843	0.999933887	0.00541217	-0.117671736	0.123083906	0.194118072
ENSG00000130479	*MAP1S*	4.779337765	-0.016429334	-0.033352991	0.016923657	0.999933887	-0.958231187	-1.167664742	0.209433555	0.194123952
ENSG00000196712	*NF1*	5.202762455	-0.04755642	-0.031973805	-0.015582616	0.999933887	-0.245134542	-0.017990105	-0.227144437	0.194266329
ENSG00000237772	*LINC02631*	1.570826029	-0.032032661	0.002736353	-0.034769014	0.999933887	0.174858092	0.59344807	-0.418589978	0.19428112
ENSG00000121417	*ZNF211*	3.355298123	-0.021694326	-0.035003705	0.013309378	0.999933887	-0.009735385	-0.170062405	0.160327021	0.194307756
ENSG00000197057	*DTHD1*	1.817580655	0.075977475	-0.031261739	0.107239214	0.999933887	-0.192900896	0.197235112	-0.390136008	0.194334688
ENSG00000160094	*ZNF362*	2.617646831	0.079214801	0.068826176	0.010388625	0.999933887	-0.160520723	-0.402229639	0.241708916	0.194382582
ENSG00000148481	*MINDY3*	3.012348109	-0.053416006	-0.190354362	0.136938356	0.999933887	0.512619442	0.828501888	-0.315882446	0.194382582
ENSG00000106948	*AKNA*	9.130536172	0.04273028	0.029888317	0.012841963	0.999933887	0.290729555	0.41434944	-0.123619885	0.194527043
ENSG00000171943	*SRGAP2C*	1.644519703	0.052347014	0.007924177	0.044422837	0.999933887	-0.023882282	0.339230427	-0.363112708	0.194560218
ENSG00000133142	*TCEAL4*	1.634519793	0.014748219	-0.186489155	0.201237374	0.999933887	0.142552486	0.432457517	-0.289905031	0.194560218
ENSG00000186432	*KPNA4*	6.708511875	-0.05863671	-0.017137682	-0.041499029	0.999933887	-0.391189462	-0.252762968	-0.138426495	0.194866424
ENSG00000130204	*TOMM40*	2.541539774	0.077354526	0.116132242	-0.038777715	0.999933887	0.080025794	0.307691941	-0.227666147	0.194866424
ENSG00000151366	*NDUFC2*	-0.256571517	0.057263968	0.289061898	-0.23179793	0.999933887	0.122874292	-0.31941586	0.442290153	0.194866424
ENSG00000117597	*UTP25*	2.346642729	-0.147359788	-0.18142258	0.034062792	0.999933887	-0.12385642	0.105259393	-0.229115813	0.195237847
ENSG00000223768	*LINC00205*	1.885491319	0.089831665	0.110472397	-0.020640732	0.999933887	-0.029454571	-0.299659601	0.27020503	0.195237847
ENSG00000197536	*IRF1-AS1*	4.609300455	0.125492589	0.109651049	0.01584154	0.999933887	-0.180651203	-0.363209111	0.182557908	0.195246429
ENSG00000232573	*RPL3P4*	1.110125329	0.006524843	-0.176833685	0.183358529	0.999933887	0.017955108	0.332677269	-0.314722161	0.195246429
ENSG00000273341	*AC004921.1*	-0.541623447	0.1803312	-0.011499198	0.191830397	0.999933887	1.687213486	2.0778867	-0.390673214	0.195361482
ENSG00000169764	*UGP2*	4.550540938	-0.060101867	-0.174809679	0.114707811	0.999933887	-0.213355123	-0.376658394	0.163303271	0.195369851
ENSG00000177302	*TOP3A*	5.406005787	-4.6527e-05	0.007852654	-0.007899181	0.999933887	-0.525634033	-0.693980742	0.168346709	0.195573101
ENSG00000185721	*DRG1*	2.769939668	-0.040790252	-0.083974003	0.043183751	0.999933887	-0.029732677	0.170228986	-0.199961663	0.195588445
ENSG00000259626	*MTND3P12*	1.705217849	0.445543901	0.762015135	-0.316471234	0.999933887	1.976246946	2.796163864	-0.819916918	0.195731689
ENSG00000229727	*AC013460.1*	-0.749103291	-0.280433634	-0.152657806	-0.127775828	0.999933887	-0.344490358	0.138385475	-0.482875833	0.195731689
ENSG00000115539	*PDCL3*	1.488956049	-0.025967974	-0.151315226	0.125347252	0.999933887	-0.05008093	-0.400688946	0.350608016	0.195807139
ENSG00000048707	*VPS13D*	6.0222617	-0.03660012	-0.055402942	0.018802822	0.999933887	0.200039527	0.350347941	-0.150308414	0.19598738
ENSG00000211653	*IGLV1-40*	-1.667620815	-0.197364011	-0.020067388	-0.177296623	0.999933887	0.213819735	-0.89632234	1.110142076	0.19598738
ENSG00000136319	*TTC5*	1.1479107	0.054156561	-0.267014133	0.321170694	0.999933887	0.060363163	-0.262394037	0.3227572	0.19598738
ENSG00000077147	*TM9SF3*	6.562663315	-0.038967465	-0.072734641	0.033767176	0.999933887	0.015589555	0.188278521	-0.172688966	0.196012299
ENSG00000169359	*SLC33A1*	0.943706444	-0.177112531	-0.120251181	-0.05686135	0.999933887	-0.405325602	-0.072478613	-0.332846989	0.19606161
ENSG00000188322	*SBK1*	2.776939717	0.052598175	0.026596282	0.026001893	0.999933887	0.042752602	-0.170910314	0.213662916	0.19606161
ENSG00000135931	*ARMC9*	0.09461823	-0.422467336	-0.518068188	0.095600853	0.999933887	0.50287414	0.836022259	-0.333148119	0.196073865
ENSG00000239306	*RBM14*	4.486638618	-0.033106411	-0.008327313	-0.024779098	0.999933887	0.008454831	-0.156459692	0.164914523	0.196073865
ENSG00000135426	*TESPA1*	3.775325896	-0.018113114	-0.126766377	0.108653264	0.999933887	0.116834539	0.25163555	-0.134801012	0.196073865
ENSG00000106785	*TRIM14*	4.126977412	0.012022918	-0.003247303	0.015272021	0.999933887	-0.124766351	-0.299431019	0.174664668	0.196123385
ENSG00000075292	*ZNF638*	5.459637749	-0.069003028	-0.102419136	0.033416108	0.999933887	-0.372395106	-0.064982194	-0.307412912	0.196180834
ENSG00000268705	*BNIP3P26*	-0.016659485	0.089236777	0.146766935	-0.057530158	0.999933887	-0.100067405	0.468031162	-0.568098567	0.196180834
ENSG00000105866	*SP4*	4.170566432	0.032658337	0.025126284	0.007532052	0.999933887	-0.263506152	0.04553576	-0.309041912	0.196320165
ENSG00000122965	*RBM19*	3.545715682	-3.7927e-05	0.054327618	-0.054365545	0.999933887	0.238399942	0.41669044	-0.178290498	0.196323964
ENSG00000083312	*TNPO1*	6.203737491	-0.041022639	-0.102217565	0.061194926	0.999933887	-0.585446824	-0.336614621	-0.248832203	0.196380423
ENSG00000275339	*Z99129.1*	0.595158023	0.052207492	-0.053636452	0.105843944	0.999933887	-0.240702069	0.228372186	-0.469074254	0.196539261
ENSG00000233175	*AC008105.1*	1.556217538	-0.070534545	-0.044114697	-0.026419847	0.999933887	-0.518084468	-0.190757148	-0.327327321	0.196574605
ENSG00000033627	*ATP6V0A1*	6.18618972	-0.100894582	-0.028980693	-0.071913889	0.999933887	-0.739423617	-0.917836329	0.178412712	0.196621915
ENSG00000167173	*C15orf39*	9.773225456	-0.0460779	-0.140970242	0.094892342	0.999933887	0.881874478	1.042122336	-0.160247858	0.196793496
ENSG00000164659	*ELAPOR2*	0.363163024	-0.040814858	-0.33849075	0.297675892	0.999933887	-0.145615425	0.230456424	-0.376071849	0.196793496
ENSG00000198892	*SHISA4*	-0.829418696	0.217601653	0.223562395	-0.005960742	0.999933887	-0.05902966	-0.713373111	0.654343451	0.197048193
ENSG00000129680	*MAP7D3*	0.731112094	-0.070674236	-0.223720891	0.153046654	0.999933887	-0.192813491	0.123237361	-0.316050852	0.197048193
ENSG00000218018	*RBM38-AS1*	0.8251089	-0.019044751	0.009599444	-0.028644195	0.999933887	-0.044967001	-0.436037947	0.391070946	0.197183763
ENSG00000169683	*LRRC45*	1.562848916	-0.000126332	0.217850756	-0.217977088	0.999933887	0.162189339	-0.102169134	0.264358473	0.197472695
ENSG00000065618	*COL17A1*	1.762367852	-0.048064942	0.090483846	-0.138548788	0.999933887	3.834598555	4.400063359	-0.565464803	0.197577067
ENSG00000118197	*DDX59*	4.313960224	-0.058789195	0.110094996	-0.168884191	0.999933887	-0.307080388	-0.155565911	-0.151514477	0.197607008
ENSG00000257621	*PSMA3-AS1*	6.300888875	-0.017886405	-0.107351892	0.089465487	0.999933887	-0.218388253	-0.008500697	-0.209887556	0.197607008
ENSG00000100393	*EP300*	9.093806431	-0.009834847	-0.016463801	0.006628955	0.999933887	-0.150932101	-0.024328685	-0.126603416	0.197607008
ENSG00000164209	*SLC25A46*	3.660844706	-0.012825063	-0.135959797	0.123134734	0.999933887	-0.22058637	0.050579804	-0.271166174	0.197956608
ENSG00000079999	*KEAP1*	4.785558611	-0.006959434	-0.132467313	0.125507879	0.999933887	0.343562363	0.511403883	-0.167841519	0.197984176
ENSG00000198695	*MT-ND6*	6.690793266	0.005390806	0.027065013	-0.021674208	0.999933887	-0.010477852	0.208377045	-0.218854897	0.198062285
ENSG00000227032	*RPS2P36*	1.270450085	0.116559889	0.256060715	-0.139500826	0.999933887	2.476949956	2.919852004	-0.442902048	0.198133737

ENSG00000226233	*Z82188.1*	-0.807766546	0.360578279	-0.175614105	0.536192384	0.999933887	0.76006762	1.249062805	-0.488995185	0.198341898
ENSG00000102119	*EMD*	7.531002598	0.106829157	0.113959646	-0.007130489	0.999933887	0.569594302	0.728938574	-0.159344273	0.198589712
ENSG00000141480	*ARRB2*	9.725086708	-0.001775364	0.016768644	-0.018544009	0.999933887	-0.400598251	-0.525489439	0.124891187	0.198660463
ENSG00000276718	*AC005840.4*	0.282184149	-0.155032269	0.062809704	-0.217841972	0.999933887	-0.125559455	-0.603876575	0.47831712	0.198693911
ENSG00000145734	*BDP1*	4.189067537	-0.103884877	-0.03939343	-0.064491447	0.999933887	-0.343500335	0.08298176	-0.426482096	0.198693911
ENSG00000102978	*POLR2C*	4.390580802	-0.039684002	0.030490702	-0.070174705	0.999933887	-0.092011955	-0.271254046	0.179242091	0.198693911
ENSG00000132405	*TBC1D14*	6.573201191	-0.023342315	-0.057941123	0.034598809	0.999933887	-0.592947046	-0.742202941	0.149255895	0.198693911
ENSG00000164180	*TMEM161B*	2.626080322	-0.040622389	-0.104829085	0.064206696	0.999933887	-0.347832067	-0.037982815	-0.309849251	0.198944725
ENSG00000251230	*MIR3945HG*	2.412585636	0.13672564	0.098264635	0.038461005	0.999933887	2.752940384	3.175819509	-0.422879124	0.199009695
ENSG00000196757	*ZNF700*	3.148859006	-0.107093259	-0.096980125	-0.010113134	0.999933887	-0.433877074	-0.707841539	0.273964465	0.199009695
ENSG00000173530	*TNFRSF10D*	1.116496837	-0.066993073	0.071476142	-0.138469215	0.999933887	-0.312465691	-0.718320416	0.405854725	0.199009695
ENSG00000259820	*AC083843.2*	2.386712739	-0.168164408	-0.212282161	0.044117753	0.999933887	0.059140819	0.537342828	-0.478202008	0.199266532
ENSG00000244625	*MIATNB*	3.206703678	0.045198423	-0.029994338	0.07519276	0.999933887	-0.369262675	-0.540518451	0.171255776	0.199266532
ENSG00000145012	*LPP*	5.084687729	0.005459727	-0.007768245	0.013227972	0.999933887	-0.1953495	0.052906044	-0.248255544	0.199266532
ENSG00000181038	*METTL23*	3.908292021	0.049242829	0.084602961	-0.035360132	0.999933887	0.612300411	0.79994858	-0.187648169	0.199306018
ENSG00000177150	*FAM210A*	3.516441912	0.017323055	0.028100849	-0.010777794	0.999933887	0.199819191	0.469008427	-0.269189236	0.199317422
ENSG00000155827	*RNF20*	4.217015335	-0.037477898	-0.123332817	0.085854919	0.999933887	-0.475985006	-0.658788982	0.182803975	0.199606608
ENSG00000091136	*LAMB1*	1.029658159	-0.234004299	-0.035599891	-0.198404408	0.999933887	0.073180579	0.397285416	-0.324104837	0.199628604
ENSG00000162604	*TM2D1*	2.722576197	0.043747427	0.043422334	0.000325094	0.999933887	-0.224610954	-0.044346674	-0.18026428	0.199628604
ENSG00000272047	*GTF2H5*	1.09055742	-0.00932243	-0.170927579	0.161605149	0.999933887	-0.193407541	0.145815724	-0.339223265	0.199628604
ENSG00000229473	*RGS17P1*	2.326719132	0.015999966	0.011538988	0.004460978	0.999933887	0.495219924	0.786402108	-0.291182184	0.19963107
ENSG00000163608	*NEPRO*	3.939331577	-0.114140842	-0.171605724	0.057464881	0.999933887	-0.052057972	0.108775218	-0.16083319	0.199657821
ENSG00000248996	*AC145098.1*	4.68856727	-0.02260588	-0.010343894	-0.012261986	0.999933887	-0.306881884	-0.120246008	-0.186635876	0.199657821
ENSG00000143612	*C1orf43*	7.658755053	-0.014442514	-0.053664418	0.039221903	0.999933887	0.05424652	0.198061844	-0.143815325	0.199722321
ENSG00000128606	*LRRC17*	-0.364921159	0.230409757	0.141596008	0.088813749	0.999933887	-0.303310868	0.197400323	-0.500711191	0.199782955
ENSG00000108784	*NAGLU*	3.749597044	-0.088685196	-0.202818764	0.114133568	0.999933887	-0.430951706	-0.623458461	0.192506755	0.199840367
ENSG00000254317	*AC022973.5*	1.63219855	0.034469463	0.19559467	-0.161125207	0.999933887	-0.237250295	0.194893115	-0.43214341	0.199840367
ENSG00000157181	*ODR4*	3.188717631	0.001801913	-0.123476739	0.125278652	0.999933887	-0.102645261	0.226904478	-0.329549739	0.199840367
ENSG00000196821	*ILRUN*	7.10877493	-0.065713416	-0.032588163	-0.033125254	0.999933887	0.014273187	0.140089949	-0.125816761	0.199964441
ENSG00000136689	*IL1RN*	9.211585529	0.063303932	0.054122856	0.009181076	0.999933887	2.197090949	2.646006395	-0.448915446	0.199994474
ENSG00000214922	*HLA-F-AS1*	2.151527292	0.100430986	0.013893842	0.086537144	0.999933887	0.730462896	1.036522546	-0.306059651	0.20013866
ENSG00000100365	*NCF4*	7.939531781	0.042184221	0.007992592	0.034191629	0.999933887	0.672031555	0.486279544	0.185752011	0.20013866
ENSG00000167981	*ZNF597*	2.499887945	-0.048754182	-0.066079831	0.017325649	0.999933887	0.459861006	0.776856786	-0.31699578	0.20013866
ENSG00000168785	*TSPAN5*	0.990364079	0.015717593	-0.016326627	0.03204422	0.999933887	0.065931856	0.40487113	-0.338939274	0.200219493
ENSG00000169062	*UPF3A*	3.510572986	-0.075901183	0.00804436	-0.083945543	0.999933887	-0.177423386	-0.368965285	0.191541898	0.200351112
ENSG00000146729	*NIPSNAP2*	2.735961631	0.006722523	0.035240306	-0.028517783	0.999933887	0.106088404	0.322173133	-0.216084729	0.200725708
ENSG00000141977	*CIB3*	-1.048114633	-0.046224654	-0.085701288	0.039476634	0.999933887	-0.124192741	-1.103842398	0.979649657	0.200747001
ENSG00000111011	*RSRC2*	6.563669984	-0.037611675	-0.054317997	0.016706322	0.999933887	-0.076577537	0.13032767	-0.206905207	0.201116779
ENSG00000168016	*TRANK1*	7.727611046	-0.027803953	-0.077883088	0.050079134	0.999933887	-0.105941056	-0.259809702	0.153868646	0.201193581
ENSG00000061794	*MRPS35*	1.407632782	-0.042204248	-0.107042319	0.064838071	0.999933887	0.109170097	0.40025914	-0.291089043	0.201193581
ENSG00000280213	*UCKL1-AS1*	2.117387605	0.031283425	0.139521061	-0.108237635	0.999933887	0.388247226	0.684263386	-0.296016161	0.201193581
ENSG00000143669	*LYST*	8.383569106	-0.030702861	-0.082215066	0.051512205	0.999933887	-0.126255382	0.118294154	-0.244549537	0.201193581
ENSG00000165312	*OTUD1*	5.45079299	-0.010137281	0.020168576	-0.030305858	0.999933887	-0.467875306	-0.637434167	0.169558861	0.201193581
ENSG00000236577	*SNRPGP14*	0.40251416	-0.093738928	0.449709806	-0.543448734	0.999933887	0.324383784	0.604296928	-0.279913144	0.201263205
ENSG00000163466	*ARPC2*	9.236149927	-0.020218877	-0.006702324	-0.013516553	0.999933887	-0.101397558	-0.19784244	0.096444882	0.201352469
ENSG00000106397	*PLOD3*	3.103260916	0.055235086	0.016414653	0.038820432	0.999933887	-0.175999675	-0.385713687	0.209714012	0.201418898
ENSG00000269352	*PTOV1-AS2*	1.966491718	0.033552975	0.099993956	-0.066440981	0.999933887	-0.212090778	-0.528982328	0.31689155	0.201418898
ENSG00000139793	*MBNL2*	2.324878572	0.035336488	-0.14231105	0.177647538	0.999933887	-0.157465169	0.275443703	-0.432908872	0.201418898
ENSG00000179958	*DCTPP1*	0.584673836	0.116707587	-0.13952088	0.256228467	0.999933887	0.191468466	0.578654503	-0.387186037	0.201419794
ENSG00000237854	*LINC00674*	2.271509983	0.109021298	-0.012672305	0.121693602	0.999933887	0.075342876	-0.125952115	0.201294991	0.201645777
ENSG00000109670	*FBXW7*	6.40244251	0.00478782	0.012113516	-0.007325697	0.999933887	0.141446988	0.324448611	-0.183001624	0.201702726
ENSG00000230844	*ZNF674-AS1*	4.787185561	0.086546227	0.126491847	-0.03994562	0.999933887	0.601114272	0.803205677	-0.202091404	0.201854762
ENSG00000166886	*NAB2*	4.512122108	0.026011743	-0.191806702	0.217818445	0.999933887	-1.287523721	-1.543178596	0.255654875	0.201854762
ENSG00000136504	*KAT7*	5.597170939	-0.021952605	-0.028679045	0.00672644	0.999933887	-0.300243885	-0.445148572	0.144904687	0.201862142
ENSG00000204388	*HSPA1B*	3.272113394	-0.048492296	-0.010540374	-0.037951922	0.999933887	0.513891948	0.870866323	-0.356974374	0.201862142
ENSG00000095574	*IKZF5*	4.930826966	-0.024359614	-0.033623883	0.009264269	0.999933887	-0.296402101	-0.12726751	-0.169134591	0.202003879
ENSG00000084623	*EIF3I*	5.30007952	0.050832502	-0.012553759	0.06338626	0.999933887	0.18855323	0.336333329	-0.147780098	0.202049585
ENSG00000117090	*SLAMF1*	3.243848839	-0.071535933	-0.118891589	0.047355656	0.999933887	0.276520365	0.473542327	-0.197021963	0.202063266
ENSG00000129535	*NRL*	-0.042732824	0.242788418	-0.18320434	0.425992758	0.999933887	-0.279541853	-0.837102057	0.557560204	0.202136416
ENSG00000081721	*DUSP12*	1.582771609	-0.063391629	-0.058504186	-0.004887444	0.999933887	0.149889532	-0.150819239	0.300708771	0.202136416
ENSG00000069869	*NEDD4*	0.803384494	0.029363073	-0.173705995	0.203069068	0.999933887	0.153520398	0.570956471	-0.417436073	0.202136416
ENSG00000054116	*TRAPPC3*	5.356175229	0.004036009	-0.0561287	0.060164708	0.999933887	-0.079508613	0.074172059	-0.153680671	0.202136416
ENSG00000015479	*MATR3*	2.492096148	0.033041235	0.052455374	-0.019414138	0.999933887	-0.152856033	0.239045541	-0.391901574	0.20218443
ENSG00000104894	*CD37*	8.285953673	0.026106531	0.012249807	0.013856724	0.999933887	-0.104258131	-0.238297366	0.134039235	0.202189559
ENSG00000170382	*LRRN2*	3.453589991	-0.118938856	0.101570418	-0.220509273	0.999933887	-0.522802242	-0.735522968	0.212720726	0.202368017
ENSG00000167995	*BEST1*	7.856505041	0.057066946	0.057535442	-0.000468496	0.999933887	0.327675267	0.456636583	-0.128961316	0.202368017
ENSG00000106052	*TAX1BP1*	6.726240257	-0.069927763	-0.136784673	0.06685691	0.999933887	-0.308348275	-0.124192848	-0.184155427	0.202368017
ENSG00000196507	*TCEAL3*	0.91557481	0.182549539	0.020297647	0.162251892	0.999933887	0.318757785	0.649030193	-0.330272408	0.202636302
ENSG00000196961	*AP2A1*	5.983901361	0.011533926	0.001449195	0.010084731	0.999933887	-0.409133012	-0.537915989	0.128782977	0.202738688
ENSG00000122644	*ARL4A*	3.726489523	0.077241871	0.039729404	0.037512467	0.999933887	0.600150178	0.817530383	-0.217380204	0.202749692
ENSG00000223705	*NSUN5P1*	2.7940126	0.032502671	0.071699098	-0.039196428	0.999933887	0.04411699	-0.157095007	0.201211997	0.202749692
ENSG00000066557	*LRRC40*	1.352237073	-0.151755507	-0.163822154	0.012066648	0.999933887	-0.082558811	0.337498548	-0.420057359	0.202883127
ENSG00000253556	*MTCO1P4*	-0.75750417	0.385872235	0.868876603	-0.483004368	0.999933887	2.081671533	2.749226772	-0.667555239	0.202923389
ENSG00000272316	*AL021368.2*	-1.064827532	-0.202180821	-0.351951912	0.149771092	0.999933887	-0.387763214	0.160222238	-0.547985452	0.20301073
ENSG00000129691	*ASH2L*	3.354853478	0.044375922	0.018145526	0.026230395	0.999933887	-0.392161747	-0.580768582	0.188606835	0.20301073
ENSG00000073331	*ALPK1*	6.667106283	-0.031224944	-0.046983066	0.015758122	0.999933887	0.305921357	0.448961308	-0.143039951	0.20301073
ENSG00000186575	*NF2*	2.921943542	0.008952619	0.066028217	-0.057075597	0.999933887	-0.079253392	-0.370065452	0.29081206	0.20301073
ENSG00000265656	*AC106037.3*	-0.858252135	-0.008476665	0.000417807	-0.008894472	0.999933887	-0.7082289	-1.280582098	0.572353198	0.20301073
ENSG00000116675	*DNAJC6*	-0.968898966	-0.324627537	-0.059544947	-0.26508259	0.999933887	0.056342816	0.530903836	-0.47456102	0.203103104
ENSG00000117281	*CD160*	0.766610422	-0.066612112	-0.268519929	0.201907817	0.999933887	0.065173211	0.434575055	-0.369401845	0.203337807
ENSG00000007392	*LUC7L*	4.659595495	-0.023394382	-0.000618629	-0.022775752	0.999933887	0.074543002	-0.04146528	0.116008282	0.204086508
ENSG00000143858	*SYT2*	0.417151308	-0.090129118	0.083548138	-0.173677255	0.999933887	-1.159539867	-1.649512506	0.489972639	0.204088963
ENSG00000169689	*CENPX*	1.490196508	0.140810654	-0.014421735	0.155232389	0.999933887	0.216359565	-0.136674436	0.353034001	0.204156746
ENSG00000168894	*RNF181*	4.180966032	-0.025494163	0.024462001	-0.049956164	0.999933887	-0.265890864	-0.43188433	0.165993465	0.204204694
ENSG00000184557	*SOCS3*	7.322245178	0.495545422	0.518487132	-0.02294171	0.999933887	0.563328158	0.873553519	-0.310225361	0.20422717
ENSG00000161714	*PLCD3*	1.391745208	0.024126822	-0.038271148	0.062397971	0.999933887	-0.544583231	-0.86484689	0.320263659	0.20422717
ENSG00000275385	*CCL18*	-0.184975449	0.386090249	0.219971606	0.166118643	0.999933887	1.364881081	1.779914993	-0.415033912	0.204461643
ENSG00000115207	*GTF3C2*	3.625354181	-0.048057783	0.008772985	-0.056830768	0.999933887	-0.506960311	-0.675867842	0.168907531	0.204461643
ENSG00000173598	*NUDT4*	4.397737858	-0.034592103	0.03112653	-0.065718632	0.999933887	-0.048864204	0.120426688	-0.169290892	0.204461643
ENSG00000119383	*PTPA*	3.49040645	0.013056298	0.040261412	-0.027205114	0.999933887	-0.027244822	-0.23797464	0.210729818	0.204461643
ENSG00000124193	*SRSF6*	6.27521514	-0.051711769	0.008733463	-0.060445232	0.999933887	-0.092857731	-0.203274228	0.110416497	0.204599989
ENSG00000198168	*SVIP*	3.142754333	-0.050260428	-0.128469556	0.078209128	0.999933887	0.008067223	0.242884535	-0.234817313	0.204599989
ENSG00000145391	*SETD7*	2.278996187	0.047393826	0.088261645	-0.040867819	0.999933887	-0.559984785	-0.881953456	0.321968671	0.204599989
ENSG00000123405	*NFE2*	4.850055256	0.019338608	0.050944177	-0.031605569	0.999933887	-0.041722227	-0.25215937	0.210437143	0.204701456
ENSG00000241399	*CD302*	2.284790487	-0.067786218	0.025983424	-0.093769642	0.999933887	-0.803588115	-1.189962526	0.386374411	0.204995443
ENSG00000278845	*MRPL45*	1.174916901	-0.039196795	0.00704524	-0.046242035	0.999933887	0.056899271	0.36185084	-0.304951569	0.205047192
ENSG00000146859	*TMEM140*	8.069630565	-0.014091124	-0.055108662	0.041017538	0.999933887	-0.300678593	-0.131622651	-0.169055942	0.205077737
ENSG00000225663	*MCRIP1*	5.01952148	0.052247814	-0.007988881	0.060236694	0.999933887	0.280878336	0.436202091	-0.155323755	0.205164696
ENSG00000128607	*KLHDC10*	3.366528759	-0.000833254	-0.055347602	0.054514347	0.999933887	-0.493586843	-0.688702553	0.195115709	0.205164696
ENSG00000150401	*DCUN1D2*	3.474580894	-0.06623733	-0.0314665	-0.03477083	0.999933887	-0.661202587	-0.855875783	0.194673196	0.20525403
ENSG00000261505	*AL031714.1*	2.890887975	-0.068300464	-0.013916871	-0.054383593	0.999933887	0.170134669	0.372783115	-0.202648446	0.20525403
ENSG00000122783	*CYREN*	7.294068143	-0.018928228	-0.002334754	-0.016593474	0.999933887	-0.229304676	-0.087210445	-0.142094231	0.20525403
ENSG00000075275	*CELSR1*	0.267663794	0.125547599	-0.030695447	0.156243047	0.999933887	-0.363137817	-0.007467006	-0.35567081	0.205493125
ENSG00000143614	*GATAD2B*	6.216365285	-0.082594755	-0.011011335	-0.07158342	0.999933887	-0.540780537	-0.687596906	0.146816369	0.205555199
ENSG00000103257	*SLC7A5*	6.350055892	0.076280809	0.041320739	0.034960069	0.999933887	1.595486851	1.985164352	-0.389677501	0.205555199

ENSG00000269303	*AC020907.3*	-0.442434602	0.121334496	0.439116347	-0.317781851	0.999933887	2.671951782	3.384398589	-0.712446808	0.205565536
ENSG00000138606	*SHF*	-0.086826206	0.010895211	-0.219973404	0.230868614	0.999933887	0.593847666	0.949743956	-0.355896291	0.205611947
ENSG00000109680	*TBC1D19*	-0.101250652	0.12233879	-0.342488575	0.464827365	0.999933887	-0.070777602	0.427773586	-0.498551188	0.205825341
ENSG00000176095	*IP6K1*	7.144338014	-0.065493552	-0.042016838	-0.023476713	0.999933887	-0.137985201	-0.011271896	-0.126713305	0.205884958
ENSG00000167978	*SRRM2*	10.056826482	0.007319141	-0.009240226	0.016559368	0.999933887	-0.09202052	0.004622242	-0.096642762	0.206476137
ENSG00000205423	*CNEP1R1*	4.11609446	0.001367387	0.043707798	-0.042340411	0.999933887	-0.120104111	0.070464239	-0.19056835	0.206569407
ENSG00000238105	*GOLGA2P5*	2.43671913	-0.004731366	-0.001595097	-0.003136268	0.999933887	0.060906066	-0.127837968	0.188744034	0.206642147
ENSG00000166971	*AKTIP*	2.700341696	0.0513076	0.104820075	-0.053512476	0.999933887	-0.220272459	-0.481933511	0.261661053	0.206644168
ENSG00000225855	*RUSC1-AS1*	0.506272167	-0.028943487	0.106016794	-0.134960282	0.999933887	0.091772509	-0.295081698	0.386854208	0.206717658
ENSG00000157796	*WDR19*	3.371354337	-0.067725669	-0.096307954	0.028582284	0.999933887	-0.248224438	-0.412700064	0.164475626	0.206864637
ENSG00000073350	*LLGL2*	3.538834269	0.010811172	0.04640992	-0.035598748	0.999933887	0.116375266	-0.070481798	0.186857064	0.207060691
ENSG00000204291	*COL15A1*	1.608881251	0.076596099	0.170978547	-0.094382448	0.999933887	1.727781734	2.182884049	-0.455102314	0.207132267
ENSG00000285693	*AP002381.2*	3.115356322	-0.095635711	-0.102574397	0.006938686	0.999933887	-0.81526546	-1.075397294	0.260131834	0.207299667
ENSG00000176014	*TUBB6*	1.101766193	-0.059163005	-0.247514014	0.188351009	0.999933887	-0.162242859	0.123465224	-0.285708083	0.208154186
ENSG00000069998	*HDHD5*	1.8194702	-0.020016365	0.215214076	-0.235230442	0.999933887	0.13062737	-0.114082501	0.244709871	0.208154186
ENSG00000260086	*AC007611.1*	1.918678797	0.277575951	0.442380211	-0.16480426	0.999933887	1.786853826	1.467816069	0.319037757	0.208292773
ENSG00000064115	*TM7SF3*	2.290603047	0.079971804	0.090971518	-0.010999714	0.999933887	-0.221576281	-0.494234199	0.272657918	0.208292773
ENSG00000178573	*MAF*	4.439105388	0.021225464	0.01607909	0.005146374	0.999933887	0.030233142	-0.124107863	0.154341004	0.208292773
ENSG00000235859	*AC006978.1*	2.541567731	0.00558867	-0.058101361	0.063690031	0.999933887	-0.444488557	-0.815249562	0.370761005	0.208715828
ENSG00000204020	*LIPN*	5.285532734	-0.005014143	-0.056353636	0.051339493	0.999933887	0.793226129	1.106231766	-0.313005637	0.208715828
ENSG00000270055	*AC127502.2*	0.680883054	-0.076298673	0.457444723	-0.533743396	0.999933887	-0.156858945	0.288345771	-0.445204716	0.209006869
ENSG00000183250	*LINC01547*	1.209043008	-0.12455397	0.009000159	-0.133554129	0.999933887	0.979373763	1.520919254	-0.54154549	0.209144882
ENSG00000286256	*AC084871.3*	2.627079471	0.035594952	0.053282833	-0.017687881	0.999933887	2.740514292	3.125407972	-0.38489368	0.209144882
ENSG00000170113	*NIPA1*	1.941682273	0.039773212	0.048021646	-0.008248435	0.999933887	0.069003487	0.303514019	-0.234510532	0.209255892
ENSG00000149781	*FERMT3*	8.984504227	0.060899751	0.04955454	0.01134521	0.999933887	0.197741755	0.350781472	-0.153039717	0.209260934
ENSG00000198574	*SH2D1B*	0.402245452	-0.044293042	-0.07840315	0.034110107	0.999933887	0.392322849	0.792800754	-0.400477905	0.209368001
ENSG00000174437	*ATP2A2*	5.819159113	-0.000634895	-0.010638821	0.010003926	0.999933887	-0.0967436	-0.202171797	0.105428197	0.209390997
ENSG00000104324	*CPQ*	6.264197659	-0.000358913	-0.049997071	0.049638159	0.999933887	-0.146540654	-0.280625179	0.134084525	0.209400863
ENSG00000114737	*CISH*	1.666091077	-0.090744791	-0.109440842	0.018696052	0.999933887	0.085516648	-0.303672002	0.38918865	0.209419523
ENSG00000140403	*DNAJA4*	2.596098751	-0.010496249	-0.026106309	0.01561006	0.999933887	0.127803172	0.342266126	-0.214462954	0.209534681
ENSG00000072121	*ZFYVE26*	6.089405533	-0.026366112	0.017072031	-0.043438143	0.999933887	0.006096137	0.162272033	-0.156175895	0.209624983
ENSG00000251442	*LINC01094*	1.799487076	-0.085141014	-0.138994408	0.053853395	0.999933887	-0.725790835	-1.10465184	0.378861005	0.209952292
ENSG00000257218	*GATC*	1.688325016	0.002659262	-0.043060416	0.045719677	0.999933887	0.011653568	-0.282633503	0.294287071	0.209952292
ENSG00000171786	*NHLH1*	0.012003794	-0.104185355	0.292937998	-0.397123353	0.944094537	-0.306013111	0.256739803	-0.562752914	0.210117899
ENSG00000007341	*ST7L*	3.525712695	0.094908949	0.081869098	0.013039852	0.999933887	0.403255346	0.586033032	-0.182777686	0.210140885
ENSG00000170832	*USP32*	7.525524438	-0.044594507	-0.08039628	0.035801773	0.999933887	-0.671339416	-0.826806027	0.155466611	0.210140885
ENSG00000225931	*AL139246.2*	4.188291812	0.337292302	0.451600417	-0.114308115	0.999933887	1.440095157	1.762292622	-0.322197465	0.210571279
ENSG00000086730	*LAT2*	8.05287523	0.02933135	0.002241776	0.027089574	0.999933887	-0.187268803	-0.352572457	0.165303653	0.210586225
ENSG00000008853	*RHOBTB2*	0.65904543	0.240464003	0.215967363	0.02449664	0.999933887	-0.164727922	-0.543469232	0.37874131	0.210626477
ENSG00000257315	*ZBED6*	0.96228462	0.093687088	0.063616462	0.030070627	0.999933887	-0.154892688	0.117697494	-0.272590181	0.210919769
ENSG00000105204	*DYRK1B*	3.826701816	0.034368679	0.045671983	-0.011303304	0.999933887	-0.422214081	-0.585613141	0.163399059	0.210919769
ENSG00000145428	*RNF175*	4.269331754	-0.138154662	0.032614693	-0.170769355	0.999933887	0.320838866	0.608071291	-0.287232425	0.210996174
ENSG00000140350	*ANP32A*	8.640934803	-0.058681862	-0.054468659	-0.004213204	0.999933887	0.850815782	0.990014258	-0.139198476	0.211136931
ENSG00000148180	*GSN*	7.41764167	-0.045468261	0.037858301	-0.083326562	0.999933887	-0.51153821	-0.736024376	0.224486166	0.211144111
ENSG00000257605	*MYG1-AS1*	0.671234136	-0.023107208	-0.118499072	0.095391864	0.999933887	-0.138755786	0.296191804	-0.434947589	0.211144111
ENSG00000275066	*SYNRG*	5.866459677	-0.023820579	-0.032730174	0.008909595	0.999933887	-0.239794442	-0.121464316	-0.118330127	0.211228175
ENSG00000162711	*NLRP3*	3.867420876	0.185260778	0.173198398	0.01206238	0.999933887	1.350080047	1.817186357	-0.46710631	0.211259098
ENSG00000170837	*GPR27*	3.568413941	0.020667335	0.060063798	-0.039396463	0.999933887	-0.587373755	-0.896701274	0.309327519	0.211259098
ENSG00000114423	*CBLB*	4.551189251	-0.050275496	0.106139407	-0.156414903	0.999933887	-0.037254859	0.16227091	-0.199525769	0.211505812
ENSG00000229638	*RPL4P4*	-0.756844348	0.070124822	0.163426257	-0.093301435	0.999933887	0.137799459	-0.387829076	0.525628535	0.211541619
ENSG00000175040	*CHST2*	3.376776869	0.057676396	-0.000643088	0.058319484	0.999933887	0.201689204	0.50143171	-0.299742506	0.211643255
ENSG00000178996	*SNX18*	6.402132813	-0.009469373	0.045844184	-0.055313557	0.999933887	-0.962579027	-1.243580746	0.281001718	0.211777011
ENSG00000235081	*AC245052.3*	-0.972438835	0.179126637	-0.028740258	0.207866895	0.999933887	-0.639670332	0.201425261	-0.841095592	0.211871914
ENSG00000110619	*CARS1*	5.676116628	-0.032781833	0.030580671	-0.063362504	0.999933887	0.14750271	0.255024896	-0.107522186	0.211871914
ENSG00000254996	*ANKHD1-EIF4*	2.015838751	0.083418265	0.041629245	0.041789021	0.999933887	0.044809066	0.43059895	-0.385789884	0.211871914
ENSG00000168038	*ULK4*	2.295705627	0.005656046	0.028215813	-0.022559767	0.999933887	-0.051478562	0.220782923	-0.272261485	0.211871914
ENSG00000105617	*LENG1*	2.194909898	0.097055371	0.124880889	-0.027825518	0.999933887	-0.343165415	-0.609022242	0.265856827	0.21195424
ENSG00000260711	*AL121839.2*	0.080759043	0.007386226	0.200942079	-0.193555853	0.999933887	-0.29519921	0.238994032	-0.534193242	0.212183806
ENSG00000147669	*POLR2K*	2.815064837	-0.065268549	-0.060985661	-0.004282888	0.999933887	0.354733445	0.611812597	-0.257079152	0.212198323
ENSG00000277595	*AC007546.1*	0.958502726	-0.048652221	-0.020552293	-0.028099928	0.999933887	0.692766341	1.163189451	-0.47042311	0.212198323
ENSG00000167968	*DNASE1L2*	-0.452044284	0.155399462	0.089300878	0.066098584	0.999933887	-0.194278573	-0.908783749	0.714505176	0.212441007
ENSG00000102081	*FMR1*	6.228552573	-0.048676588	-0.091233471	0.042556884	0.999933887	-0.517165932	-0.270755527	-0.246410405	0.21293742
ENSG00000004838	*ZMYND10*	-0.83884943	0.059107948	0.090988349	-0.031880401	0.999933887	-0.543178454	-1.224766792	0.681588338	0.21293742
ENSG00000213079	*SCAF8*	5.667227789	0.013325426	-0.020116408	0.033441834	0.999933887	-0.197124626	-0.075850601	-0.121274025	0.212946491
ENSG00000088970	*KIZ*	3.116210404	-0.021359196	-0.05331719	0.031957994	0.999933887	-0.199064511	-0.4182598	0.219195289	0.212955218
ENSG00000197323	*TRIM33*	6.942944657	-0.006806565	-0.083666555	0.07685999	0.999933887	-0.943658186	-0.731074055	-0.212584131	0.213453737
ENSG00000171425	*ZNF581*	2.610023595	0.005767643	-0.182954479	0.188722123	0.999933887	-0.235948262	-0.437174161	0.201225899	0.213539619
ENSG00000228918	*LINC01344*	-0.284919139	-0.009647847	-0.410803663	0.401155815	0.999933887	0.031615171	0.568863355	-0.537248184	0.213570014
ENSG00000275139	*AL133492.1*	-0.779206134	-0.286490956	-0.114161379	-0.172329577	0.999933887	-0.189400804	0.307294624	-0.496695428	0.213620424
ENSG00000164011	*ZNF691*	0.1213705	0.077304209	-0.26017885	0.337483059	0.999933887	-0.068710152	-0.396747974	0.328037822	0.213910589
ENSG00000228763	*LIMS1-AS1*	0.280335583	-0.082338207	0.184675086	-0.267013294	0.999933887	1.861182273	2.325862088	-0.464679815	0.213951323
ENSG00000126777	*KTN1*	4.567497489	-0.156362929	-0.223170808	0.06680788	0.999933887	-0.498145381	-0.148971653	-0.349173728	0.214115557
ENSG00000060339	*CCAR1*	4.394097191	-0.051006285	-0.139235423	0.088229138	0.999933887	-0.179910466	0.044501699	-0.224412166	0.214115557
ENSG00000111266	*DUSP16*	5.780068989	-0.03443027	-0.005407206	-0.029023064	0.999933887	1.103882965	1.409337949	-0.305454985	0.214115557
ENSG00000099725	*PRKY*	-3.049772494	-0.00748937	-0.026134675	0.018645306	0.999933887	-0.156217412	-0.466384162	0.31016675	0.214115557
ENSG00000130522	*JUND*	8.868936533	0.109273971	0.077879656	0.031394315	0.999933887	-0.037976032	-0.243779719	0.205803687	0.214183941
ENSG00000131844	*MCCC2*	1.942208631	-0.117405733	-0.005754171	-0.111651562	0.999933887	0.090095599	0.316181446	-0.226085846	0.214183941
ENSG00000198837	*DENND4B*	7.778338528	0.030372452	0.10840944	-0.078036988	0.999933887	0.06386089	-0.070919019	0.134779909	0.214183941
ENSG00000133101	*CCNA1*	-1.014024091	-0.140729538	-0.156247526	0.015517988	0.999933887	1.282509666	2.068293424	-0.785783758	0.214183941
ENSG00000074935	*TUBE1*	1.373084312	0.033264288	-0.15436234	0.187626628	0.999933887	0.037946123	0.291756862	-0.253810739	0.214183941
ENSG00000254477	*AP000640.1*	3.939046447	0.01311293	-0.06396819	0.077081121	0.999933887	0.521437428	0.666322725	-0.144885296	0.214183941
ENSG00000117676	*RPS6KA1*	7.737003776	0.004619033	0.002072498	0.002546535	0.999933887	0.044050523	-0.061697451	0.105747974	0.214183941
ENSG00000140471	*LINS1*	3.162477008	-0.006748917	-0.087616246	0.080867329	0.999933887	-0.149387756	0.021730359	-0.171118114	0.214183941
ENSG00000172671	*ZFAND4*	2.315879574	0.044433965	-0.104758984	0.149192949	0.999933887	-0.051764728	0.201285036	-0.253049763	0.214242746
ENSG00000211455	*STK38L*	5.491241288	-0.095480992	-0.076026544	-0.019454447	0.999933887	-0.825020279	-1.077573708	0.252553429	0.214306277
ENSG00000166887	*VPS39*	6.218411033	-0.042349128	-0.020163894	-0.022185234	0.999933887	-0.156299381	-0.261137596	0.104838214	0.214460145
ENSG00000123200	*ZC3H13*	5.668153139	-0.079028979	-0.15287557	0.073846592	0.999933887	-0.894975087	-0.58627232	-0.308702767	0.214460145
ENSG00000120837	*NFYB*	2.242723529	-0.015473238	0.051250566	-0.066723804	0.999933887	0.30618189	0.505476681	-0.199294791	0.215473197
ENSG00000140406	*TLNRD1*	4.950741705	0.146528726	0.186228626	-0.0396999	0.999933887	0.519311087	0.769690981	-0.250379894	0.215818278
ENSG00000064886	*CHI3L2*	2.023988906	0.134673263	0.338163612	-0.203490349	0.999933887	0.511289687	0.274671999	0.236617688	0.215818278
ENSG00000165806	*CASP7*	2.55063681	-0.05968786	-0.004001437	-0.055686423	0.999933887	0.243320107	0.454054684	-0.210734578	0.215952263
ENSG00000186184	*POLR1D*	4.727206984	0.036937852	0.080623938	-0.043686086	0.999933887	-0.162125582	-0.306240534	0.144114952	0.216011695
ENSG00000168434	*COG7*	1.801507013	-0.037606718	0.082663679	-0.120270397	0.999933887	-0.093464694	-0.360191285	0.266726591	0.216011695
ENSG00000163872	*YEATS2*	3.507959787	0.039799423	0.024722875	0.015076548	0.999933887	-0.181012651	-0.329573328	0.148560676	0.216304763
ENSG00000179526	*SHARPIN*	3.871693207	0.055558335	0.048393435	0.007164899	0.999933887	-0.004432705	-0.168987222	0.164554516	0.216573195
ENSG00000004455	*AK2*	4.621844254	-0.042792803	0.019096842	-0.061889645	0.999933887	0.516568956	0.69043917	-0.173870214	0.216604859
ENSG00000107651	*SEC23IP*	4.09591294	-0.075093133	0.011623537	-0.08671667	0.999933887	-0.180220855	-0.050321202	-0.129899653	0.217131584
ENSG00000109458	*GAB1*	3.682584599	-0.086647122	-0.023070551	-0.063576572	0.999933887	-1.242950848	-1.560140486	0.317189638	0.217232212
ENSG00000105810	*CDK6*	3.31171396	-0.061513593	-0.130043595	0.068530003	0.999933887	-0.142169388	0.112913341	-0.255082729	0.217393128
ENSG00000106077	*ABHD11*	1.207962003	0.211360766	-0.118810039	0.330170804	0.944094537	-0.194606276	-0.511600934	0.316994658	0.217404213
ENSG00000214212	*C19orf38*	4.563138394	0.095754921	0.056839902	0.03891502	0.999933887	-0.116609011	-0.340792179	0.224183168	0.217710111
ENSG00000143119	*CD53*	10.294225686	-0.008410999	-0.040890272	0.032479273	0.999933887	0.21786001	0.34193714	-0.12407713	0.218020636
ENSG00000185905	*C16orf54*	6.94920641	-0.012930814	-0.05721048	0.044279666	0.999933887	-0.808589552	-0.983587171	0.174997619	0.218059683
ENSG00000141034	*GID4*	1.808393201	-0.084619922	0.121688212	-0.206308133	0.999933887	-0.331638238	-0.608875784	0.277237545	0.218075888
ENSG00000116809	*ZBTB17*	5.898344568	0.05109796	0.041183177	0.009914783	0.999933887	0.447160681	0.621547143	-0.174386462	0.218144408

ENSG00000157654	*PALM2AKAP2*	3.109099923	-0.069137064	0.001035579	-0.070172644	0.999933887	0.534258521	0.747914319	-0.213655798	0.218733498
ENSG00000125148	*MT2A*	4.005395807	0.055040709	-0.050379884	0.105420593	0.999933887	0.577882953	0.848976627	-0.271093675	0.218733498
ENSG00000170873	*MTSS1*	3.463107085	-0.038958459	0.083832326	-0.122790785	0.999933887	0.02004673	0.196633022	-0.176586292	0.218788434
ENSG00000166226	*CCT2*	3.963734763	0.024624378	-0.071639377	0.096263755	0.999933887	0.25919549	0.447898392	-0.188702902	0.218819072
ENSG00000139112	*GABARAPL1*	8.693798455	0.010195445	-0.024680601	0.034876045	0.999933887	-0.190391045	-0.057955493	-0.132435552	0.218843024
ENSG00000188227	*ZNF793*	-0.431901112	-0.277631685	-0.41043191	0.132800225	0.999933887	-0.138740392	0.402294427	-0.541034819	0.219033369
ENSG00000118960	*HS1BP3*	6.10684051	-0.012639452	-0.03589455	0.023255098	0.999933887	-0.334946951	-0.186450964	-0.148495987	0.219176841
ENSG00000183160	*TMEM119*	2.528007058	-0.050709921	-0.019998965	-0.030710956	0.999933887	-0.386993153	-0.69751398	0.310520827	0.219266668
ENSG00000099364	*FBXL19*	2.860711554	0.055394576	0.171389328	-0.115994751	0.999933887	-0.242927836	-0.496601947	0.253674111	0.219266668
ENSG00000139974	*SLC38A6*	1.513385555	-0.378497153	0.048585706	-0.427082859	0.999933887	-0.051599452	0.187181034	-0.238780486	0.219292803
ENSG00000241343	*RPL36A*	1.612171075	0.146063249	0.148279368	-0.002216119	0.999933887	0.575431992	0.930330346	-0.354898354	0.219301185
ENSG00000198521	*ZNF43*	2.841322461	-0.132768317	-0.063333959	-0.069434358	0.999933887	-0.244375727	0.021543806	-0.265919533	0.219301185
ENSG00000173230	*GOLGB1*	6.452027756	-0.139425424	-0.124514692	-0.014910732	0.999933887	-0.642289706	-0.211206941	-0.431082765	0.219301185
ENSG00000168487	*BMP1*	1.169656541	0.11672113	0.074669042	0.042052088	0.999933887	-0.000380177	0.32440189	-0.324782066	0.219301185
ENSG00000107372	*ZFAND5*	8.6358189	0.027092702	0.060590872	-0.03349817	0.999933887	-0.216972869	-0.092491751	-0.124481118	0.219301185
ENSG00000173818	*ENDOV*	2.427955687	0.025302371	-0.002818619	0.028120991	0.999933887	-0.007252071	0.217262986	-0.224515058	0.219301185
ENSG00000169122	*FAM110B*	1.3558417	0.129690747	0.062831477	0.06685927	0.999933887	0.238189685	0.558923793	-0.320734109	0.219313346
ENSG00000145494	*NDUFS6*	2.96657084	0.052255963	-0.025205744	0.077461707	0.999933887	-0.038445742	-0.229693609	0.191247867	0.219424567
ENSG00000171155	*C1GALT1C1*	2.649284705	-0.055321511	0.063817238	-0.119138749	0.999933887	-0.558876449	-0.825216712	0.266340263	0.21948097
ENSG00000205476	*CCDC85C*	4.136672959	0.011853686	0.010667143	0.001186544	0.999933887	-0.047799753	0.130867012	-0.178666766	0.21948097
ENSG00000171365	*CLCN5*	2.052612287	-0.108392737	0.074469207	-0.182861944	0.999933887	-0.16161636	0.092120703	-0.253737063	0.219557035
ENSG00000099365	*STX1B*	1.607999604	0.029757482	-0.034819828	0.064577309	0.999933887	0.050875678	0.295926718	-0.24505104	0.219773345
ENSG00000119280	*C1orf198*	-0.184234353	-0.11296507	0.355546933	-0.468512002	0.999933887	0.024899543	0.458421453	-0.43352191	0.220018331
ENSG00000155100	*OTUD6B*	0.981910148	-0.024742037	-0.237860515	0.213118478	0.999933887	-0.404552508	0.065457012	-0.47000952	0.220018331
ENSG00000008118	*CAMK1G*	2.373980682	0.145629008	0.285781208	-0.1401522	0.999933887	2.765854393	3.188419514	-0.422565121	0.220022477
ENSG00000178977	*LINC00324*	1.611589587	0.01274244	-0.113380597	0.126123037	0.999933887	-0.324210623	-0.659238618	0.335027996	0.220051409
ENSG00000161265	*U2AF1L4*	2.545841849	0.015415359	-0.012430111	0.02784547	0.999933887	0.058012473	-0.165303307	0.22331578	0.220073447
ENSG00000177082	*WDR73*	1.263793017	-0.115629361	-0.042157457	-0.073471904	0.999933887	-0.107243627	-0.396071223	0.288827596	0.220288586
ENSG00000197535	*MYO5A*	5.156083749	-0.007214585	-0.07157093	0.064356345	0.999933887	-0.285524514	-0.525096345	0.239571831	0.220288586
ENSG00000161847	*RAVER1*	4.016369561	-0.013003472	-0.039521293	0.026517821	0.999933887	-0.109152435	-0.271336193	0.162183758	0.220441609
ENSG00000007080	*CCDC124*	3.232727401	0.1485641	0.077889765	0.070674336	0.999933887	0.226027854	0.446821072	-0.220793218	0.220482732
ENSG00000090470	*PDCD7*	2.341002788	0.047051847	0.093158728	-0.046106881	0.999933887	0.188310734	-0.00649032	0.194801054	0.220482732
ENSG00000180228	*PRKRA*	2.146003316	-0.045743441	0.063515301	-0.109258742	0.999933887	-0.196189361	-0.412827922	0.216638561	0.220482732
ENSG00000130695	*CEP85*	3.182553838	0.040821479	0.023380398	0.017441081	0.999933887	-0.418992758	-0.60193466	0.182941902	0.220482732
ENSG00000181852	*RNF41*	4.757459318	0.017006299	0.084222969	-0.06721667	0.999933887	0.002319079	0.146668359	-0.14434928	0.220482732
ENSG00000185414	*MRPL30*	1.729449186	0.019338843	-0.112618747	0.13195759	0.999933887	0.138649176	-0.091082321	0.229731497	0.220482732
ENSG00000101181	*MTG2*	4.262483288	-0.004487157	-0.026115748	0.021628591	0.999933887	-0.155734209	-0.301291236	0.145557027	0.220482732
ENSG00000154678	*PDE1C*	-1.054496793	-0.005228171	-0.260131481	0.25490331	0.999933887	0.00312977	0.693402008	-0.690272238	0.220482732
ENSG00000135535	*CD164*	7.848524822	-0.068870441	-0.079997399	0.011126958	0.999933887	-0.578831193	-0.444012973	-0.13481822	0.220715512
ENSG00000151136	*BTBD11*	2.400922165	0.00362963	0.002365555	0.001264075	0.999933887	0.257297012	0.003313701	0.253983311	0.22103979
ENSG00000181090	*EHMT1*	4.771278693	0.032633608	0.065956364	-0.033322755	0.999933887	-0.044486173	-0.191584553	0.14709838	0.221159679
ENSG00000166323	*C11orf65*	-0.898392946	0.229969765	0.283347478	-0.053377713	0.999933887	-0.439130946	0.275765411	-0.714896358	0.221322853
ENSG00000124733	*MEA1*	2.94904805	0.047074293	-0.080504961	0.127579254	0.999933887	-0.153740327	-0.384380704	0.230640377	0.221322853
ENSG00000186716	*BCR*	5.005331233	0.033083387	-0.011338339	0.044421726	0.999933887	0.008421193	-0.165095238	0.17351643	0.221322853
ENSG00000177853	*ZNF518A*	3.649872098	-0.076693307	-0.152036904	0.075343597	0.999933887	-0.683367956	-0.247392479	-0.435975476	0.221322853
ENSG00000108448	*TRIM16L*	-1.024620282	-0.141664773	0.096147505	-0.237812278	0.999933887	0.776067208	1.279388034	-0.503320826	0.221322853
ENSG00000178184	*PARD6G*	1.525128647	-0.014922128	0.261665245	-0.276587373	0.999933887	0.790762445	1.105410735	-0.314648289	0.221322853
ENSG00000134028	*ADAMDEC1*	-1.099869093	-0.409392558	0.05386199	-0.463254548	0.999933887	1.288292153	1.913079915	-0.624787763	0.221402888
ENSG00000187742	*SECISBP2*	6.014768217	-0.002497817	-0.022218344	0.019720526	0.999933887	0.023856405	0.13380553	-0.109949125	0.221402888
ENSG00000114026	*OGG1*	2.173236138	0.033942803	0.068087487	-0.034144683	0.999933887	-0.168311932	-0.369077789	0.200765857	0.221515353
ENSG00000185122	*HSF1*	5.180798245	0.05804897	0.073355963	-0.015306993	0.999933887	-0.020035561	-0.150460019	0.130424458	0.221743179
ENSG00000188211	*NCR3LG1*	5.742352781	-0.169230572	0.010260156	-0.179490728	0.999933887	3.024943994	3.369204968	-0.344260973	0.221743179
ENSG00000065457	*ADAT1*	5.465431561	-0.062347563	-0.03552577	-0.026821793	0.999933887	1.388782008	1.600947981	-0.212165973	0.221743179
ENSG00000105993	*DNAJB6*	7.346926465	0.012860838	0.026811587	-0.01395075	0.999933887	-0.106771024	0.024653756	-0.13142478	0.221743179
ENSG00000153250	*RBMS1*	6.627171754	-0.004808187	-0.017064906	0.012256719	0.999933887	-0.4684893	-0.321097795	-0.147391505	0.22201348
ENSG00000206337	*HCP5*	8.31806591	0.014256146	0.022352027	-0.00809588	0.999933887	0.825096506	0.960675971	-0.135579465	0.222049373
ENSG00000099840	*IZUMO4*	0.917372129	-0.010744935	0.080333817	-0.091078752	0.999933887	0.216915623	-0.11502772	0.331943343	0.222049373
ENSG00000183735	*TBK1*	6.226871708	-0.141926028	-0.182193537	0.040267509	0.999933887	1.11432196	1.393394434	-0.279072474	0.222239833
ENSG00000104983	*CCDC61*	2.03721637	0.071214201	0.070818525	0.000395675	0.999933887	-0.026838758	-0.296557509	0.269718751	0.222239833
ENSG00000215021	*PHB2*	4.664776247	0.027172958	0.036234553	-0.009061596	0.999933887	0.020383914	-0.123447119	0.143831033	0.222239833
ENSG00000205181	*LINC00654*	2.09744427	0.045164168	0.137433267	-0.0922691	0.999933887	-0.144327703	0.050199259	-0.194526962	0.222239833
ENSG00000128309	*MPST*	2.644843599	0.039104559	0.045298918	-0.006194359	0.999933887	-0.007276206	-0.265463675	0.258187469	0.222518788
ENSG00000134955	*SLC37A2*	3.675921609	-0.014598763	0.104088258	-0.118687022	0.999933887	-0.486539222	-0.329936205	-0.156603017	0.22268021
ENSG00000159720	*ATP6V0D1*	9.151414177	-0.048479645	-0.040657568	-0.007822077	0.999933887	0.307693712	0.432814828	-0.125121116	0.222918861
ENSG00000112425	*EPM2A*	-0.165599321	-0.030012239	-0.184341319	0.154329081	0.999933887	0.346958496	-0.151862747	0.498821244	0.222965489
ENSG00000230587	*LINC02580*	1.018189129	0.081902807	-0.104900223	0.18680303	0.999933887	-0.393105076	-0.744147624	0.351042548	0.223014107
ENSG00000162591	*MEGF6*	5.339239523	0.113503923	0.126254806	-0.012750883	0.999933887	0.026855508	-0.157794966	0.184650474	0.223035611
ENSG00000276231	*PIK3R6*	3.584416219	0.09191452	0.046736854	0.045177666	0.999933887	0.176438758	0.385687631	-0.209248873	0.223075582
ENSG00000173214	*MFSD4B*	2.79778972	-0.056264334	-0.045692516	-0.010571818	0.999933887	-0.026951807	0.201048941	-0.228000748	0.223075582
ENSG00000144485	*HES6*	-0.057939177	0.159410764	0.060025235	0.099385428	0.999933887	0.304479697	-0.160640918	0.465120615	0.22313598
ENSG00000138182	*KIF20B*	1.212244148	-0.244926658	-0.19082073	-0.054105928	0.999933887	-0.313199091	0.11349715	-0.42669624	0.223174775
ENSG00000164961	*WASHC5*	5.462542	0.035616632	-0.022533753	0.058150385	0.999933887	-0.145591689	0.047089283	-0.192680972	0.223181955
ENSG00000174718	*RESF1*	9.946383699	-0.152241814	-0.168438653	0.016196839	0.999933887	-0.563677955	-0.228087909	-0.335590046	0.223563887
ENSG00000213064	*SFT2D2*	4.994756033	-0.022136678	0.00672571	-0.028862388	0.999933887	-0.172161694	-0.374412447	0.202250754	0.223593925
ENSG00000229474	*PATL2*	5.356681531	-0.00528931	0.052147978	-0.057437288	0.999933887	0.33066486	0.46509762	-0.13443276	0.223712385
ENSG00000171953	*ATPAF2*	1.671884251	-0.052442406	0.089006137	-0.141448544	0.999933887	-0.054076195	-0.302704877	0.248628682	0.223865162
ENSG00000115540	*MOB4*	2.48366919	-0.057932389	-0.003560732	-0.054371657	0.999933887	-0.396454492	-0.115156867	-0.281297625	0.224375836
ENSG00000074054	*CLASP1*	5.300759236	0.032665885	-0.022889313	0.055555198	0.999933887	-0.20980091	-0.356457222	0.146656312	0.224470944
ENSG00000100490	*CDKL1*	-0.147604135	-0.038019386	0.013598476	-0.051617862	0.999933887	-0.360663501	0.170459231	-0.531122733	0.224470944
ENSG00000213551	*DNAJC9*	1.168748334	0.105279006	-0.160078174	0.265357179	0.999933887	0.10096818	-0.308767811	0.40973599	0.224607124
ENSG00000158402	*CDC25C*	0.333483579	0.193152826	-0.107060955	0.300213781	0.999933887	0.033594053	-0.50472449	0.538318543	0.224711306
ENSG00000137522	*RNF121*	2.904095567	0.07796524	0.046577727	0.031387513	0.999933887	0.299866152	0.459166291	-0.159300139	0.224711306
ENSG00000089737	*DDX24*	6.530452822	-0.048339668	-0.078301139	0.029961472	0.999933887	0.241995422	0.37586416	-0.133868738	0.224711306
ENSG00000128915	*ICE2*	2.348289092	-0.011975456	-0.219132608	0.207157152	0.999933887	-0.112230128	0.198181883	-0.310412012	0.224813007
ENSG00000088448	*ANKRD10*	5.670751453	-0.030538337	-0.071523607	0.040985269	0.999933887	0.207997185	0.412194602	-0.204197417	0.224952253
ENSG00000165156	*ZHX1*	2.154667194	-0.137658932	-0.141358557	0.003699624	0.999933887	-0.326715283	0.014302346	-0.341017629	0.225109902
ENSG00000136193	*SCRN1*	1.05427393	0.040524857	-0.143894586	0.184419443	0.999933887	-0.16898795	0.123469127	-0.292457077	0.225109902
ENSG00000152457	*DCLRE1C*	3.176222321	0.011928531	-0.076677746	0.088606277	0.999933887	-0.565344369	-0.791189484	0.225845116	0.225109902
ENSG00000231711	*LINC00899*	-0.473782536	-0.011490281	-0.087096627	0.075606346	0.999933887	-0.336727404	-0.889073013	0.552345609	0.225109902
ENSG00000105552	*BCAT2*	0.935793881	0.038183677	-0.088492577	0.126676254	0.999933887	0.116279885	0.423450397	-0.307170512	0.225272067
ENSG00000212694	*LINC01089*	2.728631247	0.037950059	-0.032577518	0.070527577	0.999933887	-0.072784135	-0.241262001	0.168477866	0.22530063
ENSG00000247708	*STX18-AS1*	-0.072302856	0.036795816	0.052621331	-0.015825515	0.999933887	0.197809322	0.659962696	-0.462153374	0.225439458
ENSG00000227543	*SPAG5-AS1*	1.511758829	0.120388781	0.043229454	0.077159327	0.999933887	0.733062069	0.991304447	-0.258242378	0.225454148
ENSG00000086712	*TXLNG*	3.118594467	0.041978525	-0.054547914	0.09652644	0.999933887	0.048491487	0.241541306	-0.193049819	0.225561735
ENSG00000107937	*GTPBP4*	3.7730535	-0.088817492	-0.065164387	-0.023653105	0.999933887	0.389394799	0.538763007	-0.149368209	0.225599785
ENSG00000273759	*AL117379.1*	-0.015752114	-0.399227266	-0.169997567	-0.229229699	0.999933887	-0.351828354	-0.727147712	0.375319359	0.225600188
ENSG00000273272	*U62317.3*	1.865572929	0.280263686	0.342820381	-0.062556695	0.999933887	0.486537141	0.100274746	0.386262394	0.225659388
ENSG00000109667	*SLC2A9*	0.560943725	0.078355827	0.076887487	0.00146834	0.999933887	0.258038829	-0.109138046	0.367176875	0.225659388
ENSG00000198169	*ZNF251*	1.70253195	-0.114742564	0.037059421	-0.151801985	0.999933887	0.155389361	-0.090830707	0.246220068	0.225754466
ENSG00000176092	*CRYBG2*	0.082246308	0.046311775	0.248643089	-0.202331314	0.999933887	-0.05683112	-0.489633103	0.432801983	0.225853944
ENSG00000152926	*ZNF117*	6.118179695	-0.127649219	-0.169317681	0.041668462	0.999933887	-0.70301143	-0.321026217	-0.381985213	0.225854087
ENSG00000103978	*TMEM87A*	3.79291642	-0.03783468	-0.033300524	-0.004534156	0.999933887	-0.174486039	-0.346812567	0.172326528	0.22595505
ENSG00000231006	*RPL7P32*	-0.281558741	0.391624452	0.485923326	-0.094298874	0.999933887	0.531128474	1.652164099	-1.121035624	0.22596074
ENSG00000104221	*BRF2*	1.018283542	-0.040088994	-0.10551778	0.065428785	0.999933887	-0.057818473	-0.458385922	0.400567449	0.22596074
ENSG00000135678	*CPM*	0.096639732	0.020315292	-0.026709821	0.047025113	0.999933887	0.326723277	0.762827087	-0.43610381	0.226034797

ENSG00000182107	*TMEM30B*	-0.164382455	-0.2569466	-0.142431619	-0.114514981	0.999933887	0.338136674	-0.18573591	0.523872584	0.226173351
ENSG00000198399	*ITSN2*	7.293194482	-0.005514488	-0.145804558	0.140290069	0.999933887	-0.327129046	0.002783969	-0.329913015	0.226173351
ENSG00000144320	*LNPK*	2.64495814	0.003511401	-0.152374184	0.155885585	0.999933887	-0.430672837	-0.123375109	-0.307297728	0.226233963
ENSG00000227507	*LTB*	7.187123742	0.146480226	0.164340986	-0.01786076	0.999933887	0.167601235	-0.149984697	0.317585933	0.226408617
ENSG00000185669	*SNAI3*	2.14294324	0.072416953	-0.133176499	0.205593452	0.999933887	0.217892824	0.479508268	-0.261615444	0.226409795
ENSG00000279453	*Z99129.4*	2.738305493	-0.036166205	-0.003344918	-0.032821287	0.999933887	-0.077628109	0.187623676	-0.265251784	0.226441642
ENSG00000204287	*HLA-DRA*	6.652120263	-0.022054515	-0.018891451	-0.003163065	0.999933887	1.083762593	1.424404829	-0.340642236	0.226441642
ENSG00000112473	*SLC39A7*	4.891833492	0.010608498	0.012384991	-0.001776493	0.999933887	-0.295345957	-0.464167003	0.168821046	0.226441642
ENSG00000170684	*ZNF296*	1.567223572	0.023018972	-0.006630024	0.029648996	0.999933887	-0.291560666	-0.619711459	0.328150794	0.226447435
ENSG00000222898	*RN7SKP97*	0.253630465	0.155818634	0.033533639	0.122284995	0.999933887	-0.388976847	0.078059654	-0.467036502	0.226583314
ENSG00000269951	*AC090181.2*	-0.059316191	0.246093137	0.061000384	0.185092752	0.999933887	0.880188454	1.221067293	-0.34087884	0.226820581
ENSG00000179021	*C3orf38*	3.11600442	-0.018491549	-0.076040574	0.057549025	0.999933887	-0.132158777	0.040716835	-0.172875613	0.226963976
ENSG00000198015	*MRPL42*	2.263410535	-0.059193001	-0.10545339	0.046260389	0.999933887	-0.152512145	0.071517228	-0.224029372	0.227144762
ENSG00000282988	*AL031777.2*	-0.466456386	0.035213569	0.11637193	-0.08115836	0.999933887	-0.809325282	-1.375589145	0.566263863	0.227144762
ENSG00000172322	*CLEC12A*	5.75470992	-0.006051057	-0.035833983	0.029782926	0.999933887	1.141349311	1.467431411	-0.3260821	0.227468597
ENSG00000271936	*AC012073.1*	-0.47320171	0.121768234	0.205706392	-0.083938157	0.999933887	1.027124602	1.50582053	-0.478695928	0.227618189
ENSG00000240905	*RN7SL798P*	2.030099349	0.107363361	0.228183524	-0.120820164	0.999933887	1.305626627	1.693381151	-0.387754524	0.227687311
ENSG00000167333	*TRIM68*	0.895260775	0.122055833	-0.032139286	0.154195119	0.999933887	0.190493857	-0.105566151	0.296060008	0.227687311
ENSG00000105656	*ELL*	7.78406305	0.032707058	0.051955889	-0.019248831	0.999933887	0.020251002	0.134217804	-0.113966803	0.227687311
ENSG00000079257	*LXN*	1.248850527	0.053696466	0.104823453	-0.051126988	0.999933887	-0.681160153	-1.003462296	0.322302143	0.22780962
ENSG00000067900	*ROCK1*	7.240742722	-0.055689751	-0.179554284	0.123864533	0.999933887	-0.733300883	-0.327721239	-0.405579644	0.228044187
ENSG00000249915	*PDCD6*	3.472842492	0.025185009	-0.088266479	0.113451488	0.999933887	-0.191288007	-0.343766506	0.152478499	0.228066994
ENSG00000157911	*PEX10*	0.71853649	0.036810086	-0.018654772	0.055464858	0.999933887	0.004445041	-0.301636023	0.306081064	0.228101209
ENSG00000130066	*SAT1*	12.281217993	0.067255902	0.02685472	0.040401182	0.999933887	0.53409719	0.689945315	-0.155848124	0.228130322
ENSG00000187010	*RHD*	2.368661331	-0.126036043	-0.010107571	-0.115928471	0.999933887	-0.162280507	-0.404512422	0.242231915	0.228272166
ENSG00000202538	*RNU4-2*	0.222315398	0.163489024	-0.815744824	0.979233848	0.999933887	2.079131716	0.05297284	2.026158876	0.22848546
ENSG00000067533	*RRP15*	1.480423379	-0.202943076	-0.252723532	0.049780456	0.999933887	-0.076986185	0.225558492	-0.302544677	0.228539045
ENSG00000229740	*U91324.1*	-1.869401856	0.392894657	0.728329068	-0.335434411	0.999933887	3.092115951	3.875106913	-0.782990962	0.22856947
ENSG00000239779	*WBP1*	2.015355923	0.058545306	0.086151354	-0.027606048	0.999933887	0.123829724	-0.127458263	0.251287987	0.22856947
ENSG00000114742	*WDR48*	5.914491883	-0.059180702	-0.059390061	0.00020936	0.999933887	0.272563401	0.39021344	-0.117650039	0.228674558
ENSG00000135929	*CYP27A1*	4.880354203	0.004667435	0.053115582	-0.048448147	0.999933887	0.119100784	0.340430629	-0.221329845	0.228824361
ENSG00000010810	*FYN*	6.258099936	-0.049281969	-0.038971053	-0.010310915	0.999933887	0.099551778	0.208096392	-0.108544614	0.228901802
ENSG00000104643	*MTMR9*	2.584875563	-0.141422539	-0.065074526	-0.076348013	0.999933887	-0.194361928	0.04144326	-0.235805188	0.229108597
ENSG00000258826	*LINC01147*	-0.850907659	-0.148595187	-0.026424421	-0.122170767	0.999933887	0.87486864	1.65495952	-0.78009088	0.229108597
ENSG00000107863	*ARHGAP21*	2.186466606	-0.032124006	-0.257919315	0.225795309	0.999933887	-0.063598875	0.232107115	-0.29570599	0.229108597
ENSG00000112561	*TFEB*	5.732300805	0.005871045	0.032717408	-0.026846363	0.999933887	-0.678875904	-0.889282612	0.210406707	0.229203194
ENSG00000167272	*POP5*	1.431549351	0.047108057	-0.032766953	0.07987501	0.999933887	0.204992011	-0.088703844	0.293695855	0.229240064
ENSG00000077514	*POLD3*	2.866833089	-0.010602233	-0.071020339	0.060418107	0.999933887	0.019165691	0.229906293	-0.210740602	0.229240064
ENSG00000140263	*SORD*	2.589723362	-0.105323724	0.086405487	-0.191729211	0.999933887	-0.055193884	-0.246817578	0.191623694	0.229270799
ENSG00000266993	*AL050343.2*	0.077393312	0.153586897	0.054666072	0.098920825	0.999933887	-0.296693372	0.218062357	-0.514755729	0.229270799
ENSG00000175711	*B3GNTL1*	4.589244896	-0.012302876	0.057403092	-0.069705968	0.999933887	-0.170401006	-0.301262619	0.130861613	0.229270799
ENSG00000187446	*CHP1*	6.352834598	-0.032703851	-0.027542574	-0.005161277	0.999933887	-0.021481588	0.109800901	-0.131282489	0.229330215
ENSG00000140526	*ABHD2*	7.87520672	-0.102122032	-0.01485443	-0.087267603	0.999933887	-0.838146637	-1.024109654	0.185963017	0.229389357
ENSG00000178038	*ALS2CL*	1.62731872	-0.025517268	-0.035374368	0.0098571	0.999933887	0.082047845	0.368101972	-0.286054127	0.229389357
ENSG00000164023	*SGMS2*	1.928334168	-0.007503145	-0.087869151	0.080366006	0.999933887	-0.121443146	0.231363892	-0.352807038	0.229389357
ENSG00000131788	*PIAS3*	2.816649411	0.052193569	0.054914333	-0.002720764	0.999933887	0.048079294	-0.109527138	0.157606433	0.229404431
ENSG00000114853	*ZBTB47*	1.023881459	-0.069285813	0.073151426	-0.142437239	0.999933887	-0.612342076	-1.145257178	0.532915102	0.229404431
ENSG00000152464	*RPP38*	1.034488639	0.04625275	-0.129293472	0.175546223	0.999933887	0.007588922	-0.315146576	0.322735497	0.229465646
ENSG00000244486	*SCARF2*	1.151170535	0.088799471	0.231402136	-0.142602665	0.999933887	-0.186919403	-0.568638275	0.381718872	0.229478989
ENSG00000244065	*MARK2P17*	-0.911515038	-0.026933071	0.173784024	-0.200717095	0.999933887	0.490280393	1.085211175	-0.594930782	0.229527074
ENSG00000266947	*AC022916.1*	0.351716169	-0.005571364	0.049193644	-0.054765008	0.999933887	0.370166752	0.801020648	-0.430853897	0.229533767
ENSG00000140265	*ZSCAN29*	3.489179969	-0.111082487	0.005730776	-0.116813263	0.999933887	-0.170883587	-0.341062859	0.170179272	0.229684474
ENSG00000287920	*AL022098.1*	5.095673133	0.064446594	0.278945792	-0.214499198	0.999933887	0.595471613	0.859178775	-0.263707162	0.229874403
ENSG00000203950	*RTL8A*	1.405693327	0.116636941	-0.171419507	0.288056448	0.999933887	-0.118471273	-0.467564597	0.349093324	0.230102336
ENSG00000174365	*SNHG11*	0.558933031	-0.036435688	0.316088751	-0.35252444	0.999933887	0.347123592	-0.029600412	0.376724004	0.230284359
ENSG00000196227	*FAM217B*	3.064147454	-0.026981742	-0.281538072	0.254556331	0.999933887	-0.291909943	-0.581161586	0.289251643	0.230354353
ENSG00000151806	*GUF1*	1.802883684	-0.002436255	-0.255503933	0.253067678	0.999933887	-0.165468401	0.170272969	-0.33574137	0.230354353
ENSG00000070610	*GBA2*	5.854182406	0.002431808	0.067688128	-0.06525632	0.999933887	-0.02930113	-0.140192171	0.110891042	0.230364837
ENSG00000100029	*PES1*	3.256686307	0.008060405	0.061400813	-0.053340409	0.999933887	0.175557733	0.340192861	-0.164635128	0.230603078
ENSG00000047365	*ARAP2*	5.077033113	-0.058171744	-0.099318937	0.041147193	0.999933887	-0.321946922	0.063132269	-0.385079191	0.230724162
ENSG00000172775	*PSME3IP1*	6.169439179	-0.040059851	0.001001916	-0.041061767	0.999933887	-0.078452877	0.014768762	-0.093221639	0.230769715
ENSG00000064666	*CNN2*	8.673683212	0.060937534	0.000570582	0.060366952	0.999933887	-0.738477452	-0.912839443	0.174361991	0.230769715
ENSG00000067606	*PRKCZ*	3.239140753	-0.015241137	0.002106964	-0.017348101	0.999933887	-0.136556656	-0.325891139	0.189334483	0.230769715
ENSG00000198356	*GET3*	4.966741491	0.027422913	-0.029189468	0.056612381	0.999933887	0.03989823	0.174290504	-0.134392273	0.230865264
ENSG00000139117	*CPNE8*	0.356884507	-0.205244405	0.007985884	-0.213230288	0.999933887	0.243470809	0.651467357	-0.407996548	0.231039069
ENSG00000235453	*SMIM27*	1.841990716	0.077833563	0.057247675	0.020585887	0.999933887	-0.150500204	-0.430661545	0.280161341	0.231039069
ENSG00000222020	*HDAC4-AS1*	1.491085034	-0.018362044	-0.184547682	0.166185638	0.999933887	0.405590329	0.157179364	0.248410965	0.231039069
ENSG00000196924	*FLNA*	11.249699033	0.073511841	0.076940231	-0.003428389	0.999933887	0.250546898	0.378246585	-0.127699688	0.231172185
ENSG00000197858	*GPAA1*	3.902035034	0.024728006	0.041122733	-0.016394727	0.999933887	0.176113858	0.015577676	0.160536182	0.231172185
ENSG00000102471	*NDFIP2*	0.711093038	-0.138628463	0.02465166	-0.163280123	0.999933887	-0.093668881	0.153777379	-0.24744626	0.231517491
ENSG00000141564	*RPTOR*	3.679292239	0.0521026	0.043929212	0.008173388	0.999933887	-0.120922606	0.04389581	-0.164818415	0.231636175
ENSG00000227392	*HPN-AS1*	-1.705640796	0.114898045	0.164746443	-0.049848398	0.999933887	3.252104291	4.382684418	-1.130580127	0.231880594
ENSG00000163704	*PRRT3*	1.898722445	0.108510968	0.121702951	-0.013191983	0.999933887	-0.58336795	-0.806537255	0.223169305	0.232186648
ENSG00000259005	*AC005479.1*	0.942356756	-0.030003559	-0.127310124	0.097306565	0.999933887	-0.018293265	0.221121157	-0.239414422	0.232307821
ENSG00000170827	*CELP*	-0.903828445	0.070795374	0.218920798	-0.148125424	0.999933887	0.682940741	1.200886745	-0.517946004	0.232435065
ENSG00000126464	*PRR12*	4.118526324	0.060782998	0.031793818	0.028989179	0.999933887	-0.111398173	-0.279646481	0.168248308	0.232722802
ENSG00000167515	*TRAPPC2L*	3.451785999	0.109624829	-0.106467931	0.21609276	0.944094537	-0.060515196	-0.219071847	0.158556651	0.232810188
ENSG00000163527	*STT3B*	6.136670694	-0.046686698	-0.049000183	0.002313484	0.999933887	-0.426245589	-0.559049064	0.132803475	0.232810188
ENSG00000091140	*DLD*	4.571971143	-0.043041772	-0.072795219	0.029753447	0.999933887	-0.086423341	0.065089336	-0.151512677	0.232810188
ENSG00000101624	*CEP76*	0.091226871	-0.075147062	0.084495309	-0.159642371	0.999933887	-0.036667348	-0.390917616	0.354250268	0.232810188
ENSG00000188612	*SUMO2*	5.118527681	-0.021546521	-0.01999022	-0.001556301	0.999933887	-0.051532315	-0.18432763	0.132795315	0.232810188
ENSG00000156384	*SFR1*	2.770379725	0.084879461	0.062519129	0.022360332	0.999933887	2.801710804	3.106833513	-0.305122709	0.232811354
ENSG00000147677	*EIF3H*	5.746595155	-0.044056737	-0.097950604	0.053893867	0.999933887	-0.013945632	0.108020514	-0.121966147	0.232926554
ENSG00000072849	*DERL2*	4.636364345	-0.043470498	-0.044194632	0.000724134	0.999933887	-0.199692674	-0.32013772	0.120445046	0.232926554
ENSG00000167986	*DDB1*	4.955767126	-0.035946253	0.060881515	-0.096827768	0.999933887	0.232846777	0.368825032	-0.135978254	0.232926554
ENSG00000055332	*EIF2AK2*	5.838943421	-0.038089871	-0.032042041	-0.00604783	0.999933887	-0.423260949	-0.576085789	0.15282484	0.232926554
ENSG00000099849	*RASSF7*	2.504387369	0.039896144	0.040723512	-0.000827368	0.999933887	0.067902669	-0.174997806	0.242900475	0.232926554
ENSG00000214783	*POLR2J4*	0.265265903	-0.008100897	0.103463887	-0.111564784	0.999933887	0.34319244	0.788061235	-0.444868795	0.232926554
ENSG00000196218	*RYR1*	0.029637081	-0.24779572	-0.068890044	-0.178905676	0.999933887	-0.356902831	0.169240075	-0.526142906	0.23306673
ENSG00000119953	*SMNDC1*	5.255781506	-0.066588391	-0.048654319	-0.017934072	0.999933887	-0.021077033	0.080934364	-0.102011397	0.23310471
ENSG00000177479	*ARIH2*	4.884075107	-0.06635151	0.016287295	-0.082638805	0.999933887	-0.088234543	-0.195991656	0.107757113	0.23310471
ENSG00000184857	*TMEM186*	0.328272484	0.05182955	0.009243175	0.042586375	0.999933887	-0.127471737	-0.542560993	0.415089256	0.23310471
ENSG00000075914	*EXOSC7*	1.636935552	0.029800074	-0.143416427	0.173216502	0.999933887	0.134504728	0.412630012	-0.278125284	0.23310471
ENSG00000123349	*PFDN5*	7.150197751	-0.0064528	-0.027227592	0.020774793	0.999933887	0.03954209	0.167158283	-0.127616193	0.23310471
ENSG00000102897	*LYRM1*	4.383406991	-0.010087368	-0.03722521	0.027137842	0.999933887	-0.25917862	-0.421989509	0.162810889	0.233316123
ENSG00000197448	*GSTK1*	6.256362161	0.005636178	0.001190253	0.004445925	0.999933887	0.077659453	-0.041588112	0.119247565	0.233484896
ENSG00000183762	*KREMEN1*	6.069600774	-0.026037986	0.039083619	-0.065121605	0.999933887	-0.242997786	-0.417754367	0.174756581	0.233661929
ENSG00000125505	*MBOAT7*	9.292205823	0.012953136	-0.001636437	0.014589573	0.999933887	-0.849609705	-1.032887916	0.183278211	0.233837867
ENSG00000173614	*NMNAT1*	1.368784744	0.000319952	-0.02626928	0.026589232	0.999933887	-0.550033457	-0.806821157	0.256787699	0.23440533
ENSG00000012124	*CD22*	4.234154691	-0.022990251	0.227345834	-0.250336086	0.999933887	0.081590448	0.226602228	-0.14501178	0.234685061
ENSG00000099937	*SERPIND1*	0.795835561	0.083746937	0.037426922	0.046320014	0.999933887	0.077802925	0.458521434	-0.380718509	0.234849077
ENSG00000019144	*PHLDB1*	2.274697057	0.042529265	-0.11828009	0.160809355	0.999933887	0.43332534	0.645113321	-0.211787981	0.23549024
ENSG00000260916	*CCPG1*	4.170548144	-0.102362653	-0.132485429	0.030122777	0.999933887	-1.052026088	-1.33878979	0.286763701	0.235866944
ENSG00000166311	*SMPD1*	5.034368474	0.008425964	-0.019699786	0.02812575	0.999933887	0.312412671	0.449323823	-0.136911151	0.235866944
ENSG00000143363	*PRUNE1*	3.42781289	-0.049791594	-0.044884714	-0.004906881	0.999933887	-0.320691311	-0.533222607	0.212531296	0.235901274

ENSG00000253352	*TUG1*	7.408519758	0.000384848	-0.00552474	0.005909588	0.999933887	-0.093525588	0.110936173	-0.204461761	0.236030409
ENSG00000184677	*ZBTB40*	4.665025644	-0.007362473	-0.018480394	0.011117921	0.999933887	-0.027790444	0.093788177	-0.121578622	0.236228429
ENSG00000253719	*ATXN7L3B*	4.43880817	-0.060975257	-0.050142027	-0.010833231	0.999933887	0.007659624	-0.123451738	0.131111363	0.236377475
ENSG00000205765	*C5orf51*	4.481604503	-0.113368046	-0.100996498	-0.012371547	0.999933887	-0.237263839	-0.021645451	-0.215618388	0.236377475
ENSG00000108344	*PSMD3*	4.489163519	0.029762289	-0.060645677	0.090407966	0.999933887	-0.154037334	-0.284425364	0.13038803	0.236377475
ENSG00000132570	*PCBD2*	0.763570968	0.029660983	-0.195645353	0.225306336	0.999933887	-0.138186639	-0.45621249	0.31802585	0.236377475
ENSG00000236320	*SLFN14*	0.403050343	0.010379396	-0.162990177	0.173369574	0.999933887	-0.054585572	0.368305038	-0.42289061	0.236377475
ENSG00000223496	*EXOSC6*	2.925002789	-0.001309624	0.034233627	-0.03554325	0.999933887	0.093375998	-0.147151415	0.240527413	0.236577008
ENSG00000204351	*SKIV2L*	6.383092959	0.018159399	0.045274156	-0.027114756	0.999933887	0.063741079	-0.062044001	0.125785081	0.236637658
ENSG00000163811	*WDR43*	3.219495365	-0.008796185	-0.045767191	0.036971006	0.999933887	0.124409235	0.311340539	-0.186931304	0.237059877
ENSG00000141699	*RETREG3*	4.923129365	-0.049817335	-0.059689449	0.009872115	0.999933887	-0.292448056	-0.433348642	0.140900585	0.237060737
ENSG00000158156	*XKR8*	4.870593798	-0.014186797	-0.066743208	0.052556411	0.999933887	-0.242372223	-0.50553625	0.263164026	0.237060737
ENSG00000137404	*NRM*	4.344457259	-0.018749505	-0.002635221	-0.016114284	0.999933887	0.29506072	0.4340982	-0.13903748	0.237458285
ENSG00000105953	*OGDH*	5.644058786	-0.023121622	0.023131137	-0.046252759	0.999933887	-0.220928933	-0.349446813	0.128517879	0.237553522
ENSG00000088682	*COQ9*	1.780307752	-0.04455333	0.058515855	-0.103069185	0.999933887	-0.012336896	-0.244351195	0.232014299	0.237831898
ENSG00000135521	*LTV1*	1.034580566	-0.114349139	-0.368536004	0.254186866	0.999933887	0.180128056	0.481429535	-0.301301478	0.237937629
ENSG00000105072	*C19orf44*	0.359366415	0.149668085	0.383473151	-0.233805066	0.999933887	0.041547545	0.4245472	-0.382999655	0.237937629
ENSG00000177191	*B3GNT8*	5.71269006	-0.048209634	-0.151759329	0.103549695	0.999933887	-0.689311651	-0.47636424	-0.212947411	0.237937629
ENSG00000266472	*MRPS21*	3.058220914	-0.015191694	-0.080477715	0.06528602	0.999933887	0.086131493	-0.095160567	0.181292059	0.237937629
ENSG00000168397	*ATG4B*	5.549164024	0.000194897	0.050382052	-0.050187155	0.999933887	0.109623753	-0.014931798	0.12455555	0.237937629
ENSG00000236255	*AC009404.1*	2.652970803	-0.007858723	0.001077597	-0.008936319	0.999933887	0.123932686	0.416773929	-0.292841242	0.238245525
ENSG00000175265	*GOLGA8A*	3.825676351	-0.02417841	0.001167823	-0.025346234	0.999933887	-0.031948647	0.149006903	-0.180955551	0.238309377
ENSG00000134202	*GSTM3*	0.46330991	-0.048874012	0.158530432	-0.207404445	0.999933887	0.025331032	0.473107759	-0.447776727	0.238383629
ENSG00000105341	*DMAC2*	3.324473972	-0.085586925	0.009145641	-0.094732565	0.999933887	-0.029841227	0.147670073	-0.177511301	0.238388961
ENSG00000234284	*ZNF879*	-0.532860116	-0.069863804	0.178299977	-0.248163781	0.999933887	-0.111321604	0.38620667	-0.497528274	0.238518916
ENSG00000134256	*CD101*	1.843885236	0.088482998	0.083994983	0.004488016	0.999933887	-0.121420544	-0.429953502	0.308532958	0.238736625
ENSG00000176124	*DLEU1*	2.112728246	0.136114317	0.205030598	-0.068916281	0.999933887	0.278873767	0.67724014	-0.398366373	0.238870669
ENSG00000135245	*HILPDA*	-0.83696805	-0.054936789	0.102626348	-0.157563137	0.999933887	1.096974689	1.584376035	-0.487401346	0.239064594
ENSG00000153037	*SRP19*	2.734547379	-0.096498063	-0.087554538	-0.008943524	0.999933887	-0.028015927	0.133169204	-0.161185131	0.2390863
ENSG00000135823	*STX6*	4.638912074	-0.097063378	-0.053201995	-0.043861383	0.999933887	-0.706811353	-0.890699033	0.18388768	0.239149012
ENSG00000149932	*TMEM219*	4.044276268	-0.028339737	-0.043188084	0.014848348	0.999933887	-0.085838307	-0.266016534	0.180178226	0.239189277
ENSG00000121350	*PYROXD1*	2.356191021	-0.153022757	-0.114619972	-0.038402785	0.999933887	-0.102545505	0.221777505	-0.32432301	0.239249136
ENSG00000181915	*ADO*	3.99604311	-0.085227999	-0.136027545	0.050799546	0.999933887	-0.254775787	-0.463856093	0.209080306	0.239423814
ENSG00000101916	*TLR8*	5.998082979	-0.085624102	-0.098206527	0.012582425	0.999933887	-1.03910086	-1.298435906	0.259335046	0.239423814
ENSG00000000457	*SCYL3*	3.004546847	-0.050082408	-0.090733992	0.040651584	0.999933887	-0.187289762	0.00384429	-0.191134053	0.239423814
ENSG00000140368	*PSTPIP1*	6.89343696	-0.016153737	0.046901102	-0.063054838	0.999933887	-0.106399293	-0.254598496	0.148199203	0.239423814
ENSG00000135698	*MPHOSPH6*	1.223636139	0.008265199	-0.011790179	0.020055378	0.999933887	0.002251992	0.325169833	-0.322917841	0.239423814
ENSG00000133858	*ZFC3H1*	6.749574552	0.057106725	0.004434054	0.052672671	0.999933887	-0.034992113	0.243485638	-0.27847775	0.239475486
ENSG00000148943	*LIN7C*	2.917222607	-0.08140238	-0.025295077	-0.056107303	0.999933887	-0.263270441	0.046025596	-0.309296037	0.239844443
ENSG00000256464	*YWHABP2*	0.079246289	0.045766631	0.099325028	-0.053558398	0.999933887	0.139601966	0.601709024	-0.462107058	0.239844443
ENSG00000197566	*ZNF624*	-0.225787531	-0.177428209	0.072328397	-0.249756605	0.999933887	-0.26546686	0.271277145	-0.536744005	0.240002719
ENSG00000145416	*MARCHF1*	0.736813788	-0.199806858	-0.024123211	-0.175683647	0.999933887	-0.459895873	-0.138540356	-0.321355518	0.240146555
ENSG00000166451	*CENPN*	0.232107243	-0.012645955	0.045531468	-0.058177424	0.999933887	0.076943659	-0.39934136	0.476285019	0.240594825
ENSG00000084463	*WBP11*	5.532383847	-0.030691204	-0.034519611	0.003828407	0.999933887	0.134407274	0.251284749	-0.116877475	0.240634991
ENSG00000130985	*UBA1*	7.822508439	0.008411879	0.028941499	-0.02052962	0.999933887	0.203052382	0.299367573	-0.096315191	0.240702678
ENSG00000167113	*COQ4*	2.520618958	-0.008307756	0.048755384	-0.05706314	0.999933887	0.169239207	-0.013266327	0.182505534	0.240702678
ENSG00000011451	*WIZ*	3.729189366	0.0749915	0.008728537	0.066262963	0.999933887	-0.029293426	-0.205071595	0.17577817	0.240782274
ENSG00000188158	*NHS*	1.792640401	-0.073775627	-0.192603549	0.118827922	0.999933887	-0.566484371	-1.153763759	0.587279389	0.240782274
ENSG00000203965	*EFCAB7*	0.770467027	-0.115847119	-0.066981159	-0.04886596	0.999933887	0.114900419	0.469108837	-0.354208418	0.240828802
ENSG00000076685	*NT5C2*	6.54435891	-0.046495017	-0.033808909	-0.012686108	0.999933887	-0.110259001	0.035943003	-0.146202004	0.240903748
ENSG00000116983	*HPCAL4*	2.130914257	-0.041545696	-0.080842019	0.039296323	0.999933887	0.123990785	-0.099253703	0.223244488	0.240903748
ENSG00000175387	*SMAD2*	5.475017775	-0.064944435	0.004933687	-0.069878121	0.999933887	-0.654231506	-0.788800533	0.134569027	0.241046966
ENSG00000008311	*AASS*	-0.830272901	-0.183742278	0.028053124	-0.211795402	0.999933887	-0.38559059	0.134484822	-0.520075412	0.241046966
ENSG00000139266	*MARCHF9*	2.791051643	0.058618607	0.021547178	0.037071429	0.999933887	0.129658414	-0.056263744	0.185922158	0.241046966
ENSG00000211584	*SLC48A1*	4.077025245	0.030395603	-0.028779596	0.059175198	0.999933887	-0.578486553	-0.807548486	0.229061933	0.241046966
ENSG00000163606	*CD200R1*	0.750599769	0.042814689	-0.057098499	0.099913188	0.999933887	-0.092893249	0.250614032	-0.343507281	0.241303506
ENSG00000133704	*IPO8*	3.698178608	0.001359667	-0.033072695	0.034432362	0.999933887	-0.061034157	-0.230922529	0.169888372	0.241514368
ENSG00000204304	*PBX2*	7.792067758	0.043833417	0.0304153	0.013418117	0.999933887	0.013766934	-0.097002741	0.110769675	0.241534568
ENSG00000171530	*TBCA*	3.568119575	0.021498453	-0.014610418	0.036108871	0.999933887	0.068294506	-0.101301459	0.169595965	0.241534568
ENSG00000136718	*IMP4*	3.33230214	0.017903807	-0.027022457	0.044926264	0.999933887	0.202189549	0.044439484	0.157750065	0.241915853
ENSG00000236936	*AL031005.1*	0.352891774	0.024016025	-0.013599924	0.037615949	0.999933887	2.226029064	3.002955135	-0.776926071	0.241915853
ENSG00000230753	*ZNF341-AS1*	-0.311424819	-0.019269615	0.39180285	-0.411072465	0.999933887	1.05831182	1.422180455	-0.363868635	0.241928367
ENSG00000169609	*C15orf40*	2.098415627	-0.047631332	-0.019161363	-0.028469968	0.999933887	0.017481124	-0.181892777	0.199373901	0.24221276
ENSG00000104957	*CCDC130*	4.894888945	0.000747652	-0.004668937	0.00541659	0.999933887	0.023687826	-0.102806648	0.126494474	0.24252744
ENSG00000255197	*AC090559.1*	6.816044574	0.012660709	0.034175601	-0.021514892	0.999933887	0.226132901	0.355527314	-0.129394412	0.242529234
ENSG00000119787	*ATL2*	3.589899871	0.002645331	-0.159516723	0.162162054	0.999933887	-0.005890869	0.157745501	-0.16363637	0.242529234
ENSG00000153786	*ZDHHC7*	5.976649211	-0.095270778	-0.070549342	-0.024721436	0.999933887	-0.797850452	-0.966336418	0.168485965	0.242708126
ENSG00000254858	*MPV17L2*	1.753044268	0.159745462	0.002246553	0.157498909	0.999933887	-0.112964845	-0.409046047	0.296081202	0.242708126
ENSG00000141858	*SAMD1*	2.517514438	-0.007368056	0.15093937	-0.158307425	0.999933887	0.175963117	0.401443949	-0.225480832	0.242708126
ENSG00000166595	*CIAO2B*	3.80360706	0.019229674	0.030586466	-0.011356793	0.999933887	-0.046280393	-0.221673702	0.175393308	0.242845729
ENSG00000136153	*LMO7*	1.185298851	-0.016675448	-0.168553405	0.151877957	0.999933887	0.019576114	0.332714365	-0.313138251	0.242845729
ENSG00000010072	*SPRTN*	3.385633671	-0.060705467	-0.047562388	-0.013143079	0.999933887	-0.164273364	0.01858525	-0.182858613	0.242949038
ENSG00000073008	*PVR*	0.509769701	0.078057942	0.102022119	-0.023964178	0.999933887	-0.104235066	-0.541326179	0.437091113	0.242949038
ENSG00000279862	*AC092964.1*	0.437437933	0.02529584	0.252106063	-0.226810223	0.999933887	1.477528183	2.150149928	-0.672621745	0.242949038
ENSG00000278818	*Metazoa_SRP*	-0.961860904	0.500092918	0.249979726	0.250113192	0.999933887	-0.242361147	0.503289005	-0.745650152	0.242969918
ENSG00000230013	*CT70*	1.470814998	0.358601241	0.024958046	0.333643195	0.999933887	0.018602639	-0.45932233	0.477924969	0.242969918
ENSG00000184979	*USP18*	0.140076273	0.114564165	0.087692105	0.02687206	0.999933887	-0.175950737	-0.582547646	0.406596909	0.242969918
ENSG00000058600	*POLR3E*	4.104433589	0.037387234	0.090179982	-0.052792748	0.999933887	0.10035538	0.244843306	-0.144487927	0.242969918
ENSG00000225889	*AC012368.1*	6.729594239	-0.025657068	-0.005226363	-0.020430705	0.999933887	0.107327508	-0.015035974	0.122363483	0.243256434
ENSG00000254470	*AP5B1*	4.884928763	-0.059619824	-0.039980373	-0.01963945	0.999933887	-0.621650509	-0.756385171	0.134734662	0.243426178
ENSG00000237094	*AL732372.2*	3.469099398	0.023430927	-0.008379638	0.031810564	0.999933887	0.195198065	0.364761133	-0.169563069	0.24348952
ENSG00000132881	*CPLANE2*	-0.563244951	0.495157856	0.17453552	0.320622335	0.999933887	0.227289833	-0.34630807	0.573597903	0.243662825
ENSG00000239569	*KMT2E-AS1*	4.247120025	0.155525762	0.17045568	-0.014929917	0.999933887	0.687251593	0.923001093	-0.2357495	0.24376341
ENSG00000089902	*RCOR1*	6.811626348	-0.076860812	0.03361649	-0.110477302	0.999933887	-0.79545424	-0.999030167	0.203575927	0.244044175
ENSG00000058262	*SEC61A1*	5.988535324	0.00159468	-0.021540285	0.023134966	0.999933887	0.267308981	0.374465399	-0.107156418	0.244148416
ENSG00000259438	*MAPK6-DT*	1.119025083	0.272428704	0.05327954	0.219149164	0.999933887	2.227924926	2.603875344	-0.375950419	0.244160852
ENSG00000107731	*UNC5B*	-1.682413883	0.264856407	-0.185468933	0.45032534	0.999933887	1.300456331	1.785182053	-0.484725722	0.24427361
ENSG00000188511	*C22orf34*	1.671987355	-0.011514428	0.089129361	-0.100643789	0.999933887	-0.112989186	-0.378244519	0.265255333	0.24427361
ENSG00000155850	*SLC26A2*	2.58236106	-0.026393989	-0.159507878	0.133113889	0.999933887	-0.075714273	0.154833879	-0.230548152	0.244520288
ENSG00000166167	*BTRC*	1.939052816	-0.061619624	-0.122866476	0.061246852	0.999933887	-0.032438156	0.200922171	-0.233360326	0.244764798
ENSG00000236449	*AC010894.2*	0.834365541	0.061477833	0.177518726	-0.116040893	0.999933887	-0.615474741	-0.990359129	0.374884388	0.244900579
ENSG00000172340	*SUCLG2*	2.235982191	0.038190219	0.028654733	0.009535486	0.999933887	-0.014476713	0.226463707	-0.24094042	0.244900579
ENSG00000273018	*FAM106A*	-0.012678446	0.050242387	-0.457662078	0.507904465	0.999933887	-0.403393438	0.029321353	-0.432714791	0.244900579
ENSG00000139880	*CDH24*	-0.29961568	-0.029222482	0.11085122	-0.140073702	0.999933887	0.230077335	0.723551318	-0.493473983	0.244900579
ENSG00000274020	*LINC01138*	2.060683803	0.035234915	0.06241416	-0.027179246	0.999933887	-0.173001002	-0.395473909	0.222472908	0.244921844
ENSG00000230224	*PHBP9*	1.718325976	-0.056069048	0.094018422	-0.15008747	0.999933887	-0.025140234	0.206903252	-0.232043485	0.244964071
ENSG00000223697	*AF230666.1*	0.053245605	-0.26333539	-0.067110974	-0.196224416	0.999933887	-0.676052261	-0.035006946	-0.641045314	0.245124187
ENSG00000102393	*GLA*	7.880531575	0.075756663	-0.01334327	0.089099932	0.999933887	1.145575496	1.37326686	-0.227691364	0.245124187
ENSG00000229833	*PET100*	1.978050118	-0.150150424	-0.142854252	-0.007296172	0.999933887	-0.131995013	-0.363576266	0.231581253	0.245229535
ENSG00000111669	*TPI1*	6.202601176	0.027186206	-0.035711687	0.062897892	0.999933887	0.010756465	-0.135845188	0.146601654	0.245229535
ENSG00000139714	*MORN3*	1.69916546	-0.006846427	-0.024533298	0.017686871	0.999933887	0.046395654	-0.187958128	0.234353782	0.245229535
ENSG00000215421	*ZNF407*	5.441222401	0.086025822	0.215247971	-0.129222149	0.999933887	0.06086461	0.220047524	-0.159182914	0.24526117
ENSG00000131779	*PEX11B*	2.087139105	-0.176539468	-0.097773584	-0.078765885	0.999933887	-0.514373766	-0.806193045	0.291819279	0.24526117
ENSG00000155755	*TMEM237*	-0.855642361	0.097898846	-0.129646618	0.227545464	0.999933887	-0.367599449	0.23304272	-0.600642169	0.24526117
ENSG00000115993	*TRAK2*	3.705444288	-0.039061074	-0.082133945	0.043072871	0.999933887	-0.116180135	-0.262363446	0.146183311	0.24530323

ENSG00000225190	*PLEKHM1*	7.722207243	-0.017256147	-0.005612173	-0.011643974	0.999933887	-0.06576827	0.037394221	-0.103162491	0.245319491
ENSG00000187556	*NANOS3*	0.751029633	0.209206345	-0.113555549	0.322761894	0.999933887	1.550770547	1.893689846	-0.342919299	0.245343423
ENSG00000269553	*U62631.1*	-1.011304884	0.161534324	-0.06192836	0.223462684	0.999933887	0.069487756	0.41025231	-0.340764554	0.246040207
ENSG00000152767	*FARP1*	2.016834661	0.132000625	0.199408041	-0.067407416	0.999933887	-0.724671588	-0.995458079	0.270786491	0.246149094
ENSG00000157077	*ZFYVE9*	-0.192620261	-0.088910727	0.078941414	-0.167852142	0.999933887	-0.008767952	-0.429242244	0.420474292	0.246149094
ENSG00000134262	*AP4B1*	3.086759807	-0.03072525	-0.060737675	0.030012424	0.999933887	0.112646294	-0.081362063	0.194008357	0.2461969
ENSG00000148358	*GPR107*	5.873480588	-0.050798024	-0.008903379	-0.041894645	0.999933887	0.149399277	0.328844474	-0.179445197	0.246349525
ENSG00000134686	*PHC2*	7.319659006	-0.035714047	-0.016810153	-0.018903894	0.999933887	-1.33820463	-1.553409951	0.215205321	0.246378869
ENSG00000124233	*SEMG1*	0.91179455	0.049706299	-0.035560611	0.08526691	0.999933887	-0.062955877	0.233398146	-0.296354023	0.246378869
ENSG00000114956	*DGUOK*	3.65983173	-0.060884169	0.049181468	-0.110065637	0.999933887	0.048764984	0.19527547	-0.146510487	0.246405044
ENSG00000138621	*PPCDC*	7.699834655	0.003416423	-0.030145619	0.033562042	0.999933887	1.157354369	1.31202137	-0.154667002	0.246405044
ENSG00000175376	*EIF1AD*	3.738886522	-0.097781088	0.011301945	-0.109083033	0.999933887	-0.323872171	-0.467337525	0.143465355	0.24647985
ENSG00000142409	*ZNF787*	5.344783316	0.095763212	0.127413519	-0.031650307	0.999933887	-0.213919882	-0.441308991	0.227389108	0.24647985
ENSG00000177337	*DLGAP1-AS1*	4.14766104	-0.03613244	-0.147085521	0.110953081	0.999933887	0.300624678	0.454311705	-0.153687028	0.24647985
ENSG00000175203	*DCTN2*	5.101488593	-0.016155044	-0.000192719	-0.015962325	0.999933887	-0.157873924	-0.045811982	-0.112061942	0.24647985
ENSG00000041353	*RAB27B*	-0.097657329	0.043641055	-0.065124188	0.108765243	0.999933887	-0.236715141	0.209055014	-0.445770155	0.24647985
ENSG00000146263	*MMS22L*	0.648843606	-0.019366206	-0.341077648	0.321711442	0.999933887	-0.094846655	0.380197541	-0.475044196	0.246526851
ENSG00000141084	*RANBP10*	3.472110808	-0.009837408	-0.029153534	0.019316127	0.999933887	0.044137214	-0.108372025	0.152509239	0.246674077
ENSG00000240211	*PPP1R35-AS1*	-0.724308689	0.09764095	0.341689248	-0.244048298	0.999933887	0.642283075	1.238561431	-0.596278356	0.246727302
ENSG00000118058	*KMT2A*	5.396438948	-0.026807472	-0.033192367	0.006384895	0.999933887	-0.070270894	0.078558828	-0.148829721	0.246792119
ENSG00000165661	*QSOX2*	3.690651122	-0.038565711	0.080067523	-0.118633233	0.999933887	0.019040241	-0.128772796	0.147813037	0.24741361
ENSG00000162236	*STX5*	5.479107703	0.018303768	-0.023777889	0.042081656	0.999933887	0.223242305	0.333979819	-0.110737515	0.24741361
ENSG00000170365	*SMAD1*	0.060878494	-0.033538832	-0.124460071	0.090921239	0.999933887	-0.017151479	0.385202834	-0.402354313	0.24741361
ENSG00000151292	*CSNK1G3*	2.915317178	-0.008103554	-0.267721522	0.259617968	0.999933887	-0.217069219	0.091553981	-0.3086232	0.247492535
ENSG00000172586	*CHCHD1*	2.146912597	0.04788579	-0.268823269	0.316709058	0.999933887	-0.397198174	-0.63612369	0.238925516	0.247554737
ENSG00000197647	*ZNF433*	1.399001056	-0.329442605	-0.089223928	-0.240218677	0.999933887	-0.747552931	-0.522736139	-0.224816792	0.247611707
ENSG00000091073	*DTX2*	5.50621606	0.009192467	0.011908107	-0.002715641	0.999933887	0.405567366	0.535104536	-0.12953717	0.247611707
ENSG00000180667	*YOD1*	4.140571976	-0.08109496	-0.212036673	0.130941713	0.999933887	-0.170200732	0.090405283	-0.260606015	0.247662633
ENSG00000180509	*KCNE1*	2.188964634	0.193130503	0.161120163	0.03201034	0.999933887	1.085912389	1.586353639	-0.50044125	0.247796476
ENSG00000177595	*PIDD1*	2.767705837	0.051205324	0.077143964	-0.025938639	0.999933887	0.099864429	-0.116161357	0.216025786	0.247796476
ENSG00000148842	*CNNM2*	3.194420715	-0.014939563	-0.046553815	0.031614252	0.999933887	-0.063543147	0.117591532	-0.181134679	0.247796476
ENSG00000214113	*LYRM4*	1.519198763	-0.02830405	-0.073241063	0.044937013	0.999933887	0.331676976	0.604713898	-0.273036922	0.247796476
ENSG00000052841	*TTC17*	4.82021902	0.000119973	-0.081576364	0.081696337	0.999933887	-0.210625477	-0.089410922	-0.121214555	0.247796476
ENSG00000182208	*MOB2*	4.490966879	0.022881306	-0.026406125	0.04928743	0.999933887	-0.080002726	-0.24034967	0.160346945	0.247923924
ENSG00000117500	*TMED5*	6.391549143	-0.034646373	-0.14466039	0.110014018	0.999933887	-0.354330081	-0.120218098	-0.234111983	0.248003555
ENSG00000163661	*PTX3*	3.84656883	0.25098728	0.254162448	-0.003175168	0.999933887	1.949971787	2.307487107	-0.35751532	0.248058229
ENSG00000253977	*MTCO2P4*	-2.564837008	0.393512783	-0.118511849	0.512024632	0.999933887	2.017615384	2.926771231	-0.909155846	0.248146609
ENSG00000012660	*ELOVL5*	8.022349656	-0.025189673	0.003667515	-0.028857188	0.999933887	-0.618920275	-0.740188011	0.121267736	0.248146609
ENSG00000286319	*AL135978.1*	1.676238942	-0.069710257	0.077345573	-0.14705583	0.999933887	0.004858537	0.393135676	-0.388277139	0.248147311
ENSG00000144746	*ARL6IP5*	6.016158462	-0.025606097	-0.092533171	0.066927074	0.999933887	-0.070665518	-0.239067165	0.168401648	0.248284267
ENSG00000113558	*SKP1*	5.993568678	-0.042618465	-0.024728223	-0.017890242	0.999933887	-0.002738077	0.098960401	-0.101698478	0.24838636
ENSG00000275665	*AC006449.3*	-0.435276806	-0.154658018	0.128105014	-0.282763032	0.999933887	0.965867141	1.319818076	-0.353950936	0.24838636
ENSG00000198715	*GLMP*	4.747025416	-0.06235213	-0.096888612	0.034536482	0.999933887	-0.406093515	-0.556830225	0.15073671	0.248454731
ENSG00000152818	*UTRN*	6.266542981	0.061169689	-0.01286474	0.074034428	0.999933887	-0.23046754	0.101151672	-0.331619212	0.248621208
ENSG00000156966	*B3GNT7*	1.282384716	0.158481402	0.128019553	0.030461849	0.999933887	2.791419378	3.181742118	-0.39032274	0.248675125
ENSG00000166986	*MARS1*	6.496905109	-0.019690911	0.001613629	-0.02130454	0.999933887	0.016218851	-0.08211038	0.098329231	0.248675125
ENSG00000124641	*MED20*	2.670817775	-0.037727947	-0.056886686	0.019158739	0.999933887	0.469583083	0.656300197	-0.186717114	0.248675125
ENSG00000144306	*SCRN3*	3.379326491	-0.020331626	-0.164642354	0.144310728	0.999933887	-0.1568738	0.028135671	-0.185009471	0.248675125
ENSG00000044446	*PHKA2*	4.389737492	-0.024981749	0.027156137	-0.052137886	0.999933887	-0.018807634	-0.164559678	0.145752045	0.248739366
ENSG00000102317	*RBM3*	6.611778508	-0.021482932	0.005222661	-0.026705593	0.999933887	0.113220314	0.231222536	-0.118002221	0.248770293
ENSG00000177885	*GRB2*	8.878790496	0.013781792	0.020350883	-0.006569091	0.999933887	0.3555361	0.467855701	-0.112319602	0.249042833
ENSG00000125089	*SH3TC1*	2.787310839	0.037391474	-0.001040641	0.038432115	0.999933887	0.005268531	-0.242652675	0.247921206	0.249079498
ENSG00000165685	*TMEM52B*	0.134572055	-0.041609167	-0.041449055	-0.000160112	0.999933887	1.099683559	1.710760906	-0.611077347	0.249085453
ENSG00000152234	*ATP5F1A*	5.101624655	-0.025870212	-0.127413009	0.101542797	0.999933887	0.052891598	-0.095889405	0.148781003	0.249094089
ENSG00000259834	*AL365361.1*	2.500830346	-0.122444223	-0.305397261	0.182953038	0.999933887	-0.323435345	0.130206819	-0.453642164	0.249171824
ENSG00000177426	*TGIF1*	7.702795341	0.1103923	0.114590751	-0.004198451	0.999933887	0.10006836	0.224530501	-0.124462142	0.249498429
ENSG00000183625	*CCR3*	4.582986747	-0.082975209	-0.000258152	-0.082717057	0.999933887	-0.288566409	-0.47139773	0.182831321	0.249645025
ENSG00000108443	*RPS6KB1*	3.614223383	-0.055761041	-0.123987025	0.068225984	0.999933887	-0.295688385	-0.049695703	-0.245992682	0.249645025
ENSG00000279141	*LINC01451*	1.249196372	0.031784845	-0.25110367	0.282888515	0.999933887	-1.600412472	-1.948848334	0.348435862	0.249645025
ENSG00000120800	*UTP20*	1.832178656	0.00110853	-0.150518759	0.151627289	0.999933887	-0.150002824	0.123869798	-0.273872623	0.249825094
ENSG00000227741	*AL121987.2*	-0.330361581	0.187715556	-0.05003202	0.237747576	0.999933887	0.551913973	0.976151378	-0.424237405	0.24987066
ENSG00000008405	*CRY1*	5.457394232	-0.02697344	-0.073320557	0.046347118	0.999933887	0.207796292	0.387284582	-0.17948829	0.24987066
ENSG00000130758	*MAP3K10*	2.963980012	0.027428206	0.156414095	-0.128985889	0.999933887	0.120283469	-0.101840496	0.222123965	0.24987066
ENSG00000138463	*SLC49A4*	5.755589162	-0.003206275	0.000311746	-0.00351802	0.999933887	0.043539889	0.148426816	-0.104886927	0.24990195
ENSG00000286696	*AC090826.3*	0.356419243	-0.080625146	0.160887921	-0.241513068	0.999933887	0.041856982	-0.400930136	0.442787118	0.25023928
ENSG00000135341	*MAP3K7*	4.572953458	-0.050411342	-0.122332302	0.07192096	0.999933887	-0.361185354	-0.208296429	-0.152888925	0.25023928
ENSG00000177406	*NINJ2-AS1*	4.33011703	-0.031569484	-0.023409732	-0.008159752	0.999933887	0.48679017	0.65906793	-0.172277761	0.25023928
ENSG00000182952	*HMGN4*	4.922276311	-0.009528358	-0.022734204	0.013205846	0.999933887	-0.142346834	-0.275190952	0.132844118	0.25023928
ENSG00000198824	*CHAMP1*	2.084685514	-0.010462797	-0.095715252	0.085252455	0.999933887	-0.392174089	-0.640620486	0.248446396	0.25023928
ENSG00000166272	*WBP1L*	6.237150294	-0.090115613	-0.018114862	-0.072000751	0.999933887	-0.113557711	0.045390577	-0.158948288	0.250250037
ENSG00000215012	*RTL10*	2.984830309	-0.114544149	-0.093212854	-0.021331294	0.999933887	-0.234359514	-0.42913729	0.194777777	0.250494937
ENSG00000122912	*SLC25A16*	3.745460569	-0.020062037	0.013561183	-0.03362322	0.999933887	-0.118206248	0.032864388	-0.151070636	0.250602347
ENSG00000061273	*HDAC7*	6.808276139	0.035323836	0.085539982	-0.050216147	0.999933887	0.062083095	-0.056968702	0.119051797	0.250613965
ENSG00000182944	*EWSR1*	7.869517459	-0.008735406	0.021602863	-0.030338269	0.999933887	-0.007979571	0.088312396	-0.096291966	0.250617913
ENSG00000156973	*PDE6D*	2.583119079	0.053131111	0.045293049	0.007838062	0.999933887	0.469212541	0.662193818	-0.192981277	0.250651003
ENSG00000124486	*USP9X*	8.097883148	-0.036900774	-0.067895308	0.030994534	0.999933887	-0.323734986	-0.119616701	-0.204118285	0.250651003
ENSG00000171827	*ZNF570*	0.765149552	0.10128834	-0.15459385	0.255882191	0.999933887	-0.160540907	0.235834434	-0.396375342	0.250870295
ENSG00000169139	*UBE2V2*	2.961566692	-0.007439205	-0.103318108	0.095878903	0.999933887	-0.243900521	-0.058480644	-0.185419876	0.250870295
ENSG00000100109	*TFIP11*	5.828148683	-0.037819891	-0.044496252	0.006676361	0.999933887	-0.011843719	0.092277851	-0.10412157	0.250878664
ENSG00000134077	*THUMPD3*	4.451441801	-0.075059691	-0.067860968	-0.007198724	0.999933887	-0.24039677	-0.080324957	-0.160071813	0.250982021
ENSG00000114023	*FAM162A*	1.689872131	-0.078191994	-0.075401416	-0.002790577	0.999933887	0.198561058	-0.03346191	0.232022968	0.251320452
ENSG00000122068	*FYTTD1*	4.204989577	-0.000751382	-0.059901281	0.059149899	0.999933887	-0.272844782	-0.101031119	-0.171813663	0.251320452
ENSG00000182150	*ERCC6L2*	2.699957663	-0.054925329	-0.258399837	0.203474508	0.999933887	-0.187870465	0.192019574	-0.379890039	0.251384568
ENSG00000183918	*SH2D1A*	3.591322342	-0.085851786	-0.204299322	0.118447536	0.999933887	0.098855046	0.274967115	-0.176112069	0.251384664
ENSG00000205808	*PLPP6*	1.176091444	0.027537195	-0.210366324	0.237903519	0.999933887	0.696639251	0.353814302	0.342824948	0.251384664
ENSG00000230569	*AC114763.2*	-1.296628132	-0.298683184	1.004887334	-1.303570518	0.999933887	0.969471554	1.443846138	-0.474374584	0.251400757
ENSG00000184277	*TM2D3*	6.078624464	0.028242606	0.029877685	-0.001635079	0.999933887	-0.051864169	0.067570433	-0.119434602	0.251400757
ENSG00000072506	*HSD17B10*	2.467805091	-0.027206296	-0.017922651	-0.009283644	0.999933887	0.137193799	-0.072629489	0.209823288	0.25140547
ENSG00000198105	*ZNF248*	0.327593888	-0.213865114	0.051571224	-0.265436337	0.999933887	-0.149688848	0.196373688	-0.346062536	0.251677643
ENSG00000171798	*KNDC1*	-0.543252342	0.134217114	-0.038872586	0.173089701	0.999933887	0.919984687	1.38389285	-0.463908164	0.251834907
ENSG00000100554	*ATP6V1D*	7.004702264	0.000598166	-0.025371805	0.025969971	0.999933887	0.102989255	0.263547742	-0.160558486	0.251834907
ENSG00000149499	*EML3*	5.635907405	0.04227735	0.095498661	-0.053221311	0.999933887	0.369070801	0.550906748	-0.181835947	0.251850199
ENSG00000285868	*AC008676.3*	0.85855022	0.041296481	-0.085550381	0.126846862	0.999933887	-0.399157555	-0.029822675	-0.36933488	0.252085977
ENSG00000198520	*ARMH1*	3.001798214	0.123871714	0.029108765	0.094762948	0.999933887	-0.003776788	-0.203674163	0.199897375	0.252094401
ENSG00000273356	*LINC02019*	1.271768408	-0.092185664	-0.049952954	-0.04223271	0.999933887	-0.652115843	-0.989877759	0.337761916	0.252094401
ENSG00000110719	*TCIRG1*	9.73619722	0.047133643	0.063762521	-0.016628878	0.999933887	-0.297326173	-0.427568564	0.130242391	0.252231725
ENSG00000012232	*EXTL3*	5.331098713	-0.061104431	-0.042682794	-0.018421637	0.999933887	-0.894873475	-1.096491802	0.201618327	0.252231725
ENSG00000182173	*TSEN54*	3.551921334	0.053559228	0.068962473	-0.015403245	0.999933887	0.17411875	0.012742345	0.161376405	0.252385262
ENSG00000228253	*MT-ATP8*	4.599706188	0.053139016	-0.068283522	0.121422538	0.999933887	-0.037410668	0.21142213	-0.248832798	0.252385262
ENSG00000198826	*ARHGAP11A*	-0.582986989	-0.014890586	0.2356783	-0.250568886	0.999933887	-0.195661615	0.380190531	-0.575852146	0.252385262
ENSG00000142168	*SOD1*	4.379956038	0.027789309	0.017669245	0.010120063	0.999933887	0.165477406	0.015865786	0.14961162	0.252541241
ENSG00000265354	*TIMM23*	2.306743655	-0.050490553	-0.076506774	0.02601622	0.999933887	0.071859447	0.255725687	-0.183866241	0.252860121
ENSG00000274173	*AL035661.1*	2.269508669	-0.051964107	-0.006775145	-0.045188962	0.999933887	0.351109685	0.651692871	-0.300583186	0.252860121
ENSG00000135378	*PRRG4*	3.734579038	0.032789539	0.053674704	-0.020885164	0.999933887	0.521501724	0.743845417	-0.222343693	0.252860121
ENSG00000099866	*MADCAM1*	0.473971354	-0.053575188	-0.133189792	0.079614604	0.999933887	-0.104712729	-0.474385393	0.369672664	0.252893771

ENSG00000271933	*AL603756.1*	1.999367955	0.146092939	0.174573751	-0.028480812	0.999933887	0.246400759	0.557231136	-0.310830377	0.252911027
ENSG00000173153	*ESRRA*	5.777376163	0.09962337	0.010341838	0.089281532	0.999933887	-0.102360375	0.083500781	-0.185861156	0.252911027
ENSG00000237310	*GS1-124K5.4*	-0.346742182	0.200355855	0.023368984	0.176986872	0.999933887	0.37374644	0.820410089	-0.446663649	0.252911027
ENSG00000224137	*LINC01857*	0.412392475	-0.180068652	-0.035316644	-0.144752009	0.999933887	1.012007809	1.261613453	-0.249605644	0.252911027
ENSG00000104142	*VPS18*	9.269916501	0.03207161	0.014038016	0.018033594	0.999933887	0.373583966	0.523179659	-0.149595693	0.252911027
ENSG00000278867	*AC090616.6*	0.403470944	0.066050697	-0.083189299	0.149239996	0.999933887	-0.481310603	-0.157512708	-0.323797895	0.252911027
ENSG00000115464	*USP34*	6.574384054	-0.048509621	-0.094718538	0.046208917	0.999933887	-0.437912712	-0.134291021	-0.303621692	0.252911027
ENSG00000107485	*GATA3*	3.561352223	-0.003391547	-0.06958019	0.066188643	0.999933887	0.132489575	0.290977629	-0.158488054	0.252911027
ENSG00000173402	*DAG1*	2.093903305	0.003322207	-0.100756776	0.104078983	0.999933887	-0.136569618	-0.38903472	0.252465102	0.252911027
ENSG00000214013	*GANC*	2.125418649	-0.000563826	-0.135758766	0.135194939	0.999933887	-0.022340878	-0.259185776	0.236844899	0.252911027
ENSG00000165071	*TMEM71*	6.322009841	-0.039291587	-0.060653434	0.021361847	0.999933887	-0.222872847	-0.363356701	0.140483854	0.25297253
ENSG00000224086	*PPM1F-AS1*	3.831650287	-0.216878773	-0.237286014	0.020407241	0.999933887	0.487056511	0.722969281	-0.23591277	0.252974786
ENSG00000271254	*AC240274.1*	1.400447512	-0.090671685	-0.013629908	-0.077041777	0.999933887	0.048642436	0.270164803	-0.221522367	0.252974786
ENSG00000196172	*ZNF681*	-0.795557699	-0.248442396	0.373413288	-0.621855684	0.999933887	-0.716546025	-0.221494932	-0.495051093	0.252974786
ENSG00000261008	*LINC01572*	-1.116776423	-0.376641589	-0.302387774	-0.074253815	0.999933887	1.040941667	1.554392934	-0.513451267	0.252974786
ENSG00000083099	*LYRM2*	4.102764474	-0.045050429	-0.000342397	-0.044708032	0.999933887	-0.170638839	-0.030926632	-0.139712208	0.252974786
ENSG00000153914	*SREK1*	6.044705626	-0.048083307	-0.194990954	0.146907647	0.999933887	-0.594882074	-0.200787514	-0.39409456	0.252974786
ENSG00000167874	*TMEM88*	4.483753913	0.227741287	0.140006291	0.087734996	0.999933887	1.143710578	1.345346634	-0.201636056	0.253118333
ENSG00000282100	*HSP90AB4P*	0.673818975	-0.010150828	0.08563397	-0.095784798	0.999933887	0.051410952	-0.38370093	0.435111882	0.253118333
ENSG00000121413	*ZSCAN18*	3.293309107	0.007917211	-0.046676507	0.054593718	0.999933887	-0.079010777	0.109029549	-0.188040326	0.253511706
ENSG00000067208	*EVI5*	2.258004137	-0.086471871	-0.10920256	0.022730689	0.999933887	-0.644835709	-1.01365439	0.368818681	0.253514194
ENSG00000225138	*SLC9A3-AS1*	1.832564671	-0.04119131	-0.009952778	-0.031238532	0.999933887	0.277273619	0.482713564	-0.205439945	0.253514194
ENSG00000237927	*AL078604.2*	0.436640649	-0.131581763	-0.087148092	-0.044433672	0.999933887	1.300691709	1.6215609	-0.320869191	0.25364839
ENSG00000113263	*ITK*	5.790654067	-0.026894444	-0.037737043	0.010842599	0.999933887	0.142850478	0.255337626	-0.112487148	0.253707741
ENSG00000277203	*F8A1*	3.024160019	-0.012536247	0.009760281	-0.022296528	0.999933887	1.047379327	1.34538165	-0.298002323	0.253789373
ENSG00000165688	*PMPCA*	4.347184567	-0.038815521	0.013391891	-0.052207412	0.999933887	-0.030811359	-0.164016349	0.13320499	0.253880076
ENSG00000224195	*AC022400.1*	-0.422073828	0.163455823	0.090148853	0.07330697	0.999933887	-0.589077524	-1.17538667	0.586309147	0.253897313
ENSG00000228606	*AL139011.1*	-0.14930991	0.142625218	-0.098421579	0.241046798	0.999933887	0.508791485	0.861819758	-0.353028273	0.253897313
ENSG00000237551	*AC096775.1*	-0.231139474	0.042910454	0.279365688	-0.236455234	0.999933887	0.516985942	1.05797841	-0.540992467	0.253897313
ENSG00000175087	*PDIK1L*	0.646293497	-0.14728428	0.106933848	-0.254218127	0.999933887	-0.278534923	0.088902304	-0.367437227	0.253955163
ENSG00000110092	*CCND1*	-0.599651607	0.032146734	0.016984916	0.015161818	0.999933887	0.215297946	0.665399498	-0.450101552	0.253955163
ENSG00000104299	*INTS9*	2.506559377	0.076633299	-0.020454017	0.097087316	0.999933887	0.434657708	0.680273167	-0.245615459	0.253957182
ENSG00000091127	*PUS7*	0.097733048	-0.135409772	0.014394894	-0.149804666	0.999933887	0.049325168	-0.363826908	0.413152076	0.253957182
ENSG00000188229	*TUBB4B*	6.589822627	0.045852824	-0.012437375	0.058290199	0.999933887	-0.45114869	-0.623966424	0.172817734	0.253957182
ENSG00000185009	*AP3M1*	3.664803598	-0.050937506	-0.013306218	-0.037631287	0.999933887	-0.643377174	-0.807645658	0.164268484	0.253957182
ENSG00000101290	*CDS2*	6.805055581	-0.026425047	0.00044391	-0.026868957	0.999933887	-0.990332694	-1.158893865	0.168561171	0.253957182
ENSG00000179915	*NRXN1*	-0.649661643	-0.046208231	0.172858417	-0.219066647	0.999933887	-0.647497643	0.192671935	-0.840169578	0.253957182
ENSG00000163755	*HPS3*	3.040218573	0.015167749	-0.081136875	0.096304624	0.999933887	-0.584048132	-0.761702429	0.177654297	0.253957182
ENSG00000081386	*ZNF510*	1.178393201	-0.027200327	-0.301846088	0.274645761	0.999933887	0.059307738	0.404975511	-0.345667773	0.253957182
ENSG00000144535	*DIS3L2*	2.493116584	0.006343652	-0.082078393	0.088422045	0.999933887	0.11990502	-0.056168855	0.176073874	0.253957182
ENSG00000164542	*KIAA0895*	-0.270054086	0.012461689	0.023556911	-0.011095221	0.999933887	0.289337623	0.759868262	-0.470530639	0.253957182
ENSG00000276649	*AL117335.1*	4.217305933	0.010517436	0.053791967	-0.04327453	0.999933887	0.814422839	0.967605899	-0.15318306	0.254050997
ENSG00000141384	*TAF4B*	0.972549261	0.011288433	-0.083006229	0.094294661	0.999933887	0.116430158	0.407650698	-0.29122054	0.254050997
ENSG00000081870	*HSPB11*	1.851977295	-0.03065164	0.033496521	-0.064148161	0.999933887	-0.013452565	-0.240596444	0.227143879	0.254119588
ENSG00000237169	*RPL12P27*	0.29105623	-0.108538703	0.020997143	-0.129535845	0.999933887	-1.20670675	-1.653390387	0.446683637	0.254177244
ENSG00000077458	*FAM76B*	4.940174824	-0.009708542	-0.026567469	0.016858927	0.999933887	-0.342561852	-0.105815706	-0.236746147	0.254401794
ENSG00000117983	*MUC5B*	1.639681115	-0.04475443	-0.033161302	-0.011593128	0.999933887	0.9254053	1.28768235	-0.36227705	0.254974199
ENSG00000146109	*ABT1*	4.087536376	-0.012982622	0.011710568	-0.02469319	0.999933887	-0.117038092	-0.285836419	0.168798326	0.255045423
ENSG00000286532	*PARTICL*	0.683301261	-0.069187199	-0.393829101	0.324641903	0.999933887	-0.962355207	-1.446483341	0.484128134	0.255059508
ENSG00000179195	*ZNF664*	3.111654615	-0.035199003	0.013574221	-0.048773224	0.999933887	-0.079651126	-0.234020544	0.154369418	0.255123147
ENSG00000169249	*ZRSR2*	3.448362971	-0.069015755	-0.091021181	0.022005426	0.999933887	-0.008750735	-0.179497335	0.1707466	0.25548312
ENSG00000082269	*FAM135A*	-0.029120792	0.108410134	-0.316211476	0.42462161	0.999933887	-0.732906432	-0.31346554	-0.419440892	0.25548312
ENSG00000199405	*SNORA1B*	-0.084590515	0.123108042	0.113365896	0.009742147	0.999933887	0.98305983	1.617940756	-0.634880926	0.255551747
ENSG00000159658	*EFCAB14*	5.490944822	0.000155085	-0.03885358	0.039008665	0.999933887	-0.147776535	-0.264408061	0.116631526	0.255666453
ENSG00000180815	*MAP3K15*	-0.639747961	-0.086062888	-0.043739772	-0.042323116	0.999933887	-0.63272825	0.086039447	-0.718767696	0.255911754
ENSG00000132436	*FIGNL1*	0.438022119	0.034835838	0.187034954	-0.152199116	0.999933887	-0.091358376	0.300909357	-0.392267733	0.256108687
ENSG00000134759	*ELP2*	3.964120659	-0.101362074	-0.010865306	-0.090496768	0.999933887	0.03513563	-0.090509997	0.125645627	0.256179533
ENSG00000204086	*RPA4*	-1.069204337	0.432798387	-0.009801182	0.44259957	0.999933887	1.136636744	1.605435028	-0.468798284	0.256194337
ENSG00000164284	*GRPEL2*	1.177533581	-0.085722277	-0.243685742	0.157963465	0.999933887	-0.234702501	-0.538324295	0.303621794	0.256194337
ENSG00000137054	*POLR1E*	1.747497516	-0.023080326	-0.073245238	0.050164912	0.999933887	0.147446517	0.377271143	-0.229824626	0.256389421
ENSG00000157036	*EXOG*	1.533717356	-0.015199736	-0.092354378	0.077154643	0.999933887	0.025401446	0.248456411	-0.223054966	0.256389421
ENSG00000103342	*GSPT1*	4.882631858	-0.025853426	-0.013108897	-0.012744528	0.999933887	0.091036183	0.205665602	-0.114629419	0.256739456
ENSG00000198178	*CLEC4C*	-1.958163524	0.09534865	-0.051802827	0.147151477	0.999933887	-0.114510619	-1.054563853	0.940053234	0.256874495
ENSG00000132485	*ZRANB2*	4.848247159	-0.118706974	-0.145596074	0.0268891	0.999933887	-0.228259948	0.089995673	-0.318255621	0.256893353
ENSG00000169221	*TBC1D10B*	5.930117421	0.079529952	0.061833176	0.017696776	0.999933887	-0.750046066	-0.949667602	0.199621536	0.257278905
ENSG00000146576	*C7orf26*	5.321807067	-0.037852917	-0.068238919	0.030386002	0.999933887	-0.422325334	-0.557625084	0.13529975	0.257306876
ENSG00000255139	*AP000442.1*	1.566324143	-0.130708038	0.122096746	-0.252784784	0.999933887	0.291178862	0.552369579	-0.261190717	0.25740934
ENSG00000130511	*SSBP4*	4.121803212	0.079086755	0.034057602	0.045029154	0.999933887	0.035289292	-0.134509701	0.169798993	0.257594788
ENSG00000279633	*AL137918.1*	-1.239683501	0.053919256	0.39110096	-0.337181703	0.999933887	0.44697574	0.994837014	-0.547861274	0.257594788
ENSG00000047578	*KATNIP*	5.213114173	0.003882951	0.015118793	-0.011235841	0.999933887	0.009598765	-0.102574533	0.112173298	0.257594788
ENSG00000175591	*P2RY2*	2.815205078	-0.023467127	0.006374251	-0.029841378	0.999933887	0.161608472	0.431756378	-0.270147905	0.257675536
ENSG00000228043	*AC114763.1*	0.587933041	0.181002104	0.083503737	0.097498367	0.999933887	1.334648678	1.608565854	-0.273917176	0.258045338
ENSG00000161048	*NAPEPLD*	0.632011712	-0.269763684	-0.216678879	-0.053084805	0.999933887	-0.29802822	0.046605547	-0.344633767	0.258110157
ENSG00000153443	*UBALD1*	5.862272353	0.031437805	0.032504439	-0.001066633	0.999933887	-0.428068825	-0.612728185	0.18465936	0.258310554
ENSG00000182310	*SPACA6*	4.150661681	0.043893188	0.163398433	-0.119505245	0.999933887	0.920980183	1.089979676	-0.168999494	0.258333701
ENSG00000086589	*RBM22*	6.071677097	0.00833963	0.008387402	-4.7772e-05	0.999933887	0.279866651	0.387217039	-0.107350388	0.258333701
ENSG00000078177	*N4BP2*	2.606986703	0.02164348	-0.163678793	0.185322273	0.999933887	-0.274885799	0.138516751	-0.41340255	0.258333701
ENSG00000095261	*PSMD5*	2.790970235	-0.013291001	0.044941675	-0.058232676	0.999933887	0.275325203	0.513035202	-0.237709998	0.258333701
ENSG00000196810	*CTBP1-DT*	2.452281391	0.139693141	0.109703045	0.029990096	0.999933887	0.664164299	0.913645747	-0.249481449	0.258451406
ENSG00000120910	*PPP3CC*	3.399779411	0.003221701	-0.004298496	0.007520197	0.999933887	0.292900657	0.447909852	-0.155009196	0.258451406
ENSG00000101473	*ACOT8*	2.645057399	0.075653723	0.021032039	0.054621684	0.999933887	-0.269590714	-0.491054003	0.221463289	0.258482332
ENSG00000277449	*CEBPB-AS1*	1.641977555	0.00087961	-0.047137307	0.048016917	0.999933887	0.179582265	0.470284038	-0.290701773	0.258482332
ENSG00000213983	*AP1G2*	4.598427449	0.054127073	0.093000442	-0.038873369	0.999933887	-0.024844031	-0.182425633	0.157581602	0.258667233
ENSG00000082701	*GSK3B*	6.122414554	-0.022952933	0.011429407	-0.034382339	0.999933887	-0.023736719	-0.142602207	0.118865488	0.258667233
ENSG00000154122	*ANKH*	4.221764854	-0.087561268	0.049015981	-0.136577249	0.999933887	-0.043187269	-0.163362957	0.120175688	0.258947004
ENSG00000180089	*TMEM86B*	1.119556436	0.117511545	-0.018431326	0.135942871	0.999933887	-0.336584067	-0.66652736	0.329943294	0.258947004
ENSG00000167555	*ZNF528*	0.760950837	-0.033350712	0.023945984	-0.057296697	0.999933887	-0.257016563	0.051685885	-0.308702449	0.258947004
ENSG00000165630	*PRPF18*	1.868416104	-0.061036111	-0.127156804	0.066120693	0.999933887	-0.336530238	-0.106899671	-0.229630567	0.259039694
ENSG00000010030	*ETV7*	1.806353915	0.053497685	0.027532594	0.025965091	0.999933887	-0.139114539	-0.436912277	0.297797738	0.259199401
ENSG00000114978	*MOB1A*	7.563499047	-0.043933284	0.005645596	-0.04957888	0.999933887	-0.11157583	0.004151712	-0.115727542	0.259310854
ENSG00000100427	*MLC1*	-0.490446854	-0.036103351	0.026671707	-0.062775058	0.999933887	0.20201217	-0.218443465	0.420455634	0.259396899
ENSG00000259956	*RBM15B*	4.722057338	0.031967815	0.004423438	0.027544377	0.999933887	-0.317813718	-0.467732311	0.149918593	0.259649996
ENSG00000132376	*INPP5K*	6.77252597	-0.012736646	0.013668376	-0.026405022	0.999933887	0.015753719	-0.094903154	0.110656873	0.259649996
ENSG00000136205	*TNS3*	0.874410538	0.315063994	0.21387588	0.101188114	0.999933887	-0.577441163	-0.231402462	-0.3460387	0.259887085
ENSG00000140564	*FURIN*	7.395672235	0.038750186	0.036975363	0.001774823	0.999933887	-0.811546362	-0.98891419	0.177367828	0.259890869
ENSG00000117620	*SLC35A3*	2.20490282	0.062618534	-0.158851936	0.22147047	0.999933887	-0.226508128	0.113006819	-0.339514947	0.259890869
ENSG00000007944	*MYLIP*	8.061033939	0.069556497	0.054874796	0.014681702	0.999933887	0.302546972	0.414450371	-0.111903398	0.259959364
ENSG00000139719	*VPS33A*	2.884196416	-0.02007701	-0.089880779	0.069803769	0.999933887	-0.185088533	-0.036825507	-0.148263026	0.259985751
ENSG00000091317	*CMTM6*	8.458886746	-0.051005394	-0.003439041	-0.047566353	0.999933887	0.169761975	0.278195719	-0.108433744	0.260025142
ENSG00000259924	*AC011939.1*	-0.063243474	0.201286469	0.489256975	-0.287970506	0.999933887	2.787512001	3.390908443	-0.603396442	0.260025142
ENSG00000180346	*TIGD2*	-0.590085163	-0.147578156	0.006558266	-0.154136422	0.999933887	0.122804949	-0.346425255	0.469230204	0.260025142
ENSG00000260152	*AC113208.2*	0.295514393	-0.151161789	0.037177481	-0.188339269	0.999933887	1.530539109	2.049714227	-0.519175119	0.260025142
ENSG00000162769	*FLVCR1*	3.184700582	-0.045501269	-0.145378358	0.099877088	0.999933887	-0.041090492	0.118051411	-0.159141902	0.260025142
ENSG00000076201	*PTPN23*	5.649348561	0.025395964	0.00366964	0.021726324	0.999933887	-0.605344628	-0.738162262	0.132817634	0.260025142
ENSG00000211598	*IGKV4-1*	-0.630195326	0.025110263	0.144683777	-0.119573514	0.999933887	0.405503139	-0.197822134	0.603325273	0.260088802

ENSG00000169738	*DCXR*	3.283672476	0.008667507	0.120186792	-0.111519286	0.999933887	0.173283639	-0.047397972	0.220681611	0.260178983
ENSG00000196912	*ANKRD36B*	1.119880309	-0.089908348	-0.248237332	0.158328985	0.999933887	-0.159986441	0.233402432	-0.393388873	0.260199691
ENSG00000260101	*AC008074.2*	0.917302412	-0.189246217	-0.17590234	-0.013343877	0.999933887	1.772645197	2.26760237	-0.494957173	0.260219204
ENSG00000287613	*AC069549.2*	-0.412947575	0.182957884	0.164305454	0.01865243	0.999933887	-0.765804779	-1.686387457	0.920582677	0.260322229
ENSG00000151657	*KIN*	3.457237686	-0.10209046	-0.24228726	0.1401968	0.999933887	-0.091818138	0.065869861	-0.157688	0.260530623
ENSG00000166394	*CYB5R2*	0.003486621	-0.062750777	-0.079459692	0.016708915	0.999933887	2.462213098	3.029348384	-0.567135286	0.260530623
ENSG00000237672	*KRR1P1*	0.328626845	0.021964676	0.124063923	-0.102099247	0.999933887	-0.310710649	0.08802341	-0.398734059	0.260530623
ENSG00000185261	*KIAA0825*	3.475406632	-0.001770767	0.024080113	-0.02585088	0.999933887	-0.374065181	-0.065513406	-0.308551775	0.260530623
ENSG00000273142	*LINC02604*	2.632973269	0.044862524	-0.065135691	0.109998215	0.999933887	0.410474986	0.62857956	-0.218104573	0.26061061
ENSG00000177272	*KCNA3*	3.203200357	0.03999609	-0.045973338	0.085969428	0.999933887	-0.052443159	0.201540478	-0.253983636	0.26061061
ENSG00000083444	*PLOD1*	3.553677997	0.012497438	0.02731341	-0.014815972	0.999933887	-0.243727486	-0.473145396	0.22941791	0.26082082
ENSG00000117525	*F3*	4.154552386	0.353855453	0.468323516	-0.114468063	0.999933887	1.668017472	2.103821078	-0.435803606	0.261247738
ENSG00000240219	*AL512306.2*	2.286377369	-0.010998622	0.104683931	-0.115682553	0.999933887	-0.545990345	-0.752124161	0.206133816	0.261247738
ENSG00000285016	*AC017002.3*	-2.353046832	0.896208329	0.671734573	0.224473756	0.999933887	1.950972295	2.540011812	-0.589039517	0.261691347
ENSG00000273141	*AP001269.4*	1.086167633	-0.086463481	-0.162890088	0.076426607	0.999933887	-0.594416293	-1.008873536	0.414457243	0.261887428
ENSG00000172500	*FIBP*	2.681735607	0.035757888	-0.053836048	0.089593936	0.999933887	-0.036214876	-0.246752578	0.210537701	0.261887428
ENSG00000136514	*RTP4*	0.506545224	-0.189038863	0.008205802	-0.197244665	0.999933887	-0.360569252	-0.732072586	0.371503333	0.261927904
ENSG00000166704	*ZNF606*	1.11304888	-0.188017091	-0.150525577	-0.037491513	0.999933887	-0.180653769	-0.439672396	0.259018628	0.262030463
ENSG00000176105	*YES1*	1.735184792	-0.091552049	-0.124417084	0.032865034	0.999933887	-0.226253128	0.149243968	-0.375497096	0.262030463
ENSG00000139675	*HNRNPA1L2*	-0.005043076	-0.134429847	0.060233836	-0.194663682	0.999933887	0.099325489	0.455834826	-0.356509337	0.262030463
ENSG00000236194	*AC099811.1*	3.13895677	0.056761025	0.034073387	0.022687638	0.999933887	1.230328765	1.553127242	-0.322798477	0.262030463
ENSG00000168268	*NT5DC2*	-0.726170414	-0.085687819	-0.256536506	0.170848687	0.999933887	0.022443872	-0.472245199	0.494689071	0.262030463
ENSG00000131013	*PPIL4*	3.949939115	-0.036665479	-0.014051643	-0.022613836	0.999933887	-0.343230067	-0.116575307	-0.22665476	0.262030463
ENSG00000048405	*ZNF800*	4.8001644	0.034751316	-0.106199605	0.140950921	0.999933887	-0.328524428	-0.027362819	-0.30116161	0.262030463
ENSG00000287742	*AC068282.2*	0.468227778	-0.010468459	-0.178859497	0.168391037	0.999933887	-0.980156923	-1.294998527	0.314841604	0.262030463
ENSG00000197712	*FAM114A1*	-0.123454588	-0.145336767	-0.217693086	0.07235632	0.999933887	-1.02116743	-1.533733306	0.512565876	0.262291183
ENSG00000167987	*VPS37C*	5.936455082	-0.032191484	-0.005226756	-0.026964728	0.999933887	-0.110242777	0.023691458	-0.133934235	0.262376517
ENSG00000134242	*PTPN22*	4.433539476	-0.054137706	-0.178497708	0.124360002	0.999933887	-0.209868021	0.079192389	-0.28906041	0.262377218
ENSG00000122126	*OCRL*	0.241464006	-0.020494114	-0.194621145	0.17412703	0.999933887	-0.178577148	0.125528006	-0.304105154	0.262377218
ENSG00000160007	*ARHGAP35*	4.397634074	0.022107831	0.036637646	-0.014529815	0.999933887	-0.242302816	-0.378086388	0.135783572	0.262400567
ENSG00000178741	*COX5A*	3.029674148	0.088779709	-0.091544854	0.180324563	0.999933887	-0.144520344	-0.355086115	0.210565771	0.262983286
ENSG00000174238	*PITPNA*	7.7874424	-0.020920233	-0.050699562	0.029779329	0.999933887	0.053070497	0.151269697	-0.0981992	0.263045661
ENSG00000162522	*KIAA1522*	-0.510385436	-0.057417376	-0.214045928	0.156628552	0.999933887	0.239343208	-0.143465528	0.382808735	0.26326015
ENSG00000167612	*ANKRD33*	2.403511691	0.049016121	0.145379164	-0.096363043	0.999933887	-1.159010675	-1.371366144	0.212355469	0.26326015
ENSG00000123159	*GIPC1*	2.1669082	0.148708349	0.009610241	0.139098108	0.999933887	0.116752103	-0.107228732	0.223980834	0.263273159
ENSG00000167202	*TBC1D2B*	5.802101253	-0.002622409	0.060946328	-0.063568737	0.999933887	0.300973614	0.483332797	-0.182359183	0.263526722
ENSG00000123975	*CKS2*	1.764067974	0.103191099	0.032863534	0.070327565	0.999933887	0.391381503	0.710966282	-0.319584778	0.263956459
ENSG00000285596	*AC017116.2*	-0.16853513	-0.067019435	-0.592778067	0.525758632	0.999933887	-0.082593897	0.307378715	-0.389972612	0.264310507
ENSG00000062650	*WAPL*	6.583474142	-0.066690407	-0.088105251	0.021414844	0.999933887	-0.260974366	-0.126432535	-0.134541831	0.264401685
ENSG00000181991	*MRPS11*	1.220247059	-0.07975607	0.063440602	-0.143196672	0.999933887	-0.045825385	-0.307554233	0.261728847	0.264401685
ENSG00000125652	*ALKBH7*	3.292294305	0.058882898	0.048511783	0.010371115	0.999933887	-0.147700477	-0.376546094	0.228845617	0.264401685
ENSG00000082293	*COL19A1*	-1.259340851	-0.149589426	0.136102238	-0.285691665	0.999933887	-0.361550152	0.126798768	-0.48834892	0.26444703
ENSG00000131697	*NPHP4*	3.377883411	0.007716379	0.037439466	-0.029723087	0.999933887	-0.172264105	-0.338616397	0.166352292	0.26444703
ENSG00000136688	*IL36G*	-2.242390043	0.399621435	-0.134800847	0.534422282	0.999933887	4.180979671	5.169417813	-0.988438143	0.264541549
ENSG00000143756	*FBXO28*	3.833173239	-0.058051747	0.042368855	-0.100420602	0.999933887	-0.212122014	-0.075639324	-0.13648269	0.264603837
ENSG00000143344	*RGL1*	2.180995805	-0.07973155	0.033055117	-0.112786666	0.999933887	0.28808934	0.509562827	-0.221473487	0.264603837
ENSG00000081059	*TCF7*	6.24035252	-0.004632485	0.042311812	-0.046944296	0.999933887	0.198983178	0.310384297	-0.111401118	0.264603837
ENSG00000150782	*IL18*	0.191901289	-0.069877065	-0.230869715	0.16099265	0.999933887	0.377276726	-0.129678029	0.506954756	0.264793082
ENSG00000255355	*AP000640.2*	1.500169793	0.285751188	0.327750599	-0.041999411	0.999933887	1.800760302	2.077339295	-0.276578993	0.264886827
ENSG00000131375	*CAPN7*	5.038191227	0.048746931	-0.008988565	0.057735496	0.999933887	-0.003965773	0.197202326	-0.201168099	0.264886827
ENSG00000161277	*THAP8*	1.01217966	-0.084703273	0.089547621	-0.174250894	0.999933887	-0.85202916	-1.106675889	0.254646729	0.264886827
ENSG00000079335	*CDC14A*	4.267827927	0.018489124	-0.035469436	0.05395856	0.999933887	-0.027416432	0.139106408	-0.16652284	0.264886827
ENSG00000248559	*AC109454.2*	0.105814626	0.157852543	-0.015934938	0.173787481	0.999933887	0.334139513	0.960268361	-0.626128848	0.264896198
ENSG00000134748	*PRPF38A*	5.545002454	-0.025257729	-0.066285897	0.041028168	0.999933887	0.075307032	0.180353778	-0.105046746	0.265367559
ENSG00000070214	*SLC44A1*	2.611425698	-0.042512784	0.064881482	-0.107394265	0.999933887	-0.526224802	-0.725447858	0.199223056	0.265367559
ENSG00000089248	*ERP29*	4.820199946	0.031873759	-0.032860435	0.064734195	0.999933887	-0.060378656	-0.21898419	0.158605534	0.265367559
ENSG00000260339	*HEXA-AS1*	1.093256482	0.141946221	0.018107749	0.123838472	0.999933887	0.559098198	0.869965305	-0.310867107	0.265537451
ENSG00000132475	*H3-3B*	11.92396565	0.070118697	0.059120028	0.010998669	0.999933887	0.081977881	0.205119105	-0.123141223	0.265933868
ENSG00000179409	*GEMIN4*	2.563314279	-0.020042855	0.050855434	-0.070898289	0.999933887	0.490787853	0.718228596	-0.227440743	0.265965265
ENSG00000126522	*ASL*	2.635529239	0.022998934	0.156798807	-0.133799873	0.999933887	0.046539501	-0.144506532	0.191046032	0.266011137
ENSG00000198585	*NUDT16*	3.238781882	0.012536927	-0.019476871	0.032013797	0.999933887	-0.177105854	-0.393803245	0.216697391	0.266049856
ENSG00000111142	*METAP2*	3.054187841	-0.081889837	-0.085422372	0.003532535	0.999933887	0.104469766	0.285469196	-0.18099943	0.266113226
ENSG00000252908	*RNU6-1003P*	0.447916348	0.164252499	0.539631012	-0.375378513	0.999933887	0.675428933	1.201290308	-0.525861374	0.266113226
ENSG00000101265	*RASSF2*	10.383930598	-0.077215854	-0.076571011	-0.000644843	0.999933887	0.022454586	0.126243591	-0.103789006	0.266122791
ENSG00000225492	*GBP1P1*	-0.850516783	-0.004789218	0.246181448	-0.250970666	0.999933887	0.780547924	1.575997159	-0.795449234	0.266122791
ENSG00000130177	*CDC16*	3.666213527	-0.099626911	-0.04217463	-0.057452281	0.999933887	-0.116491858	-0.245728009	0.129236152	0.26612303
ENSG00000215375	*MYL5*	2.176828348	0.022208269	0.144793134	-0.122584865	0.999933887	-0.163883802	-0.378432116	0.214548314	0.266638202
ENSG00000129351	*ILF3*	7.768365671	0.035138561	0.018375251	0.01676331	0.999933887	0.299585358	0.435700169	-0.136114811	0.266646789
ENSG00000152127	*MGAT5*	5.096230922	-0.025262967	0.024733028	-0.049995994	0.999933887	0.126432889	0.272758951	-0.146326062	0.266700056
ENSG00000230322	*AL136320.1*	0.16829944	-0.05228489	0.032737012	-0.085021901	0.999933887	-0.374928322	-1.256141405	0.881213083	0.266808489
ENSG00000151470	*C4orf33*	2.49198216	-0.037342219	-0.107684976	0.070342757	0.999933887	-0.25668978	0.057965341	-0.314655121	0.266861922
ENSG00000163626	*COX18*	1.678828098	-0.018962592	-0.066924731	0.047962138	0.999933887	0.151545371	-0.064256669	0.215802039	0.266910643
ENSG00000122641	*INHBA*	4.371739503	0.060288034	-0.104598568	0.164886602	0.999933887	0.543999168	0.775587668	-0.2315885	0.267009531
ENSG00000170364	*SETMAR*	0.783383388	0.066617319	-0.05071327	0.117330589	0.999933887	0.170365974	-0.159595832	0.329961806	0.267009531
ENSG00000162739	*SLAMF6*	3.514317629	0.010516399	-0.093027113	0.103543512	0.999933887	0.076174704	0.236084756	-0.159910053	0.267009531
ENSG00000158417	*EIF5B*	3.866023764	-0.178049404	-0.212462587	0.034413183	0.999933887	-0.139413884	0.135948881	-0.275362765	0.267012521
ENSG00000258044	*AC073569.1*	0.00063561	0.171428258	0.082255692	0.089172566	0.999933887	2.068062065	2.61513357	-0.547071504	0.267012521
ENSG00000143379	*SETDB1*	4.192328216	-0.033884233	-0.011752805	-0.022131429	0.999933887	-0.363851959	-0.497759121	0.133907162	0.267201757
ENSG00000133740	*E2F5*	0.012008668	-0.238732006	0.094694254	-0.333426261	0.999933887	-0.142080152	0.243723206	-0.385803358	0.267612074
ENSG00000229780	*UBE2Q1-AS1*	-0.396000412	-0.042150782	-0.230981613	0.188830831	0.999933887	-0.077112422	0.273369988	-0.35048241	0.267612074
ENSG00000136709	*WDR33*	4.848596613	-0.011921833	0.033959841	-0.045881674	0.999933887	-0.187258358	-0.289675569	0.102417211	0.267628799
ENSG00000260708	*AL118516.1*	4.200799781	0.212559423	0.178792217	0.033767206	0.999933887	1.643545716	1.85092108	-0.207375365	0.267809233
ENSG00000273654	*AC020904.2*	0.470619217	-0.103141834	-0.551704632	0.448562798	0.999933887	0.27801415	0.47878329	-0.200769139	0.267819057
ENSG00000254325	*AC018607.1*	2.270226053	-0.046158044	0.129135531	-0.175293575	0.999933887	1.134125007	1.540544256	-0.406419249	0.267819057
ENSG00000259225	*LINC02345*	-1.098934113	0.093938079	-0.23492956	0.32886764	0.999933887	0.593440873	1.134412906	-0.540972033	0.267819057
ENSG00000120156	*TEK*	-0.191252688	-0.175225714	-0.268650291	0.093424577	0.999933887	-0.496124553	-1.086173819	0.590049266	0.26784826
ENSG00000168903	*BTNL3*	0.643034476	-0.052059588	-0.02714119	-0.024918398	0.999933887	0.121153114	0.391537794	-0.27038468	0.268167537
ENSG00000143499	*SMYD2*	2.482022431	-0.053048334	-0.081721821	0.028673487	0.999933887	-0.028887351	0.164352803	-0.193240154	0.268440149
ENSG00000008441	*NFIX*	0.176900947	0.109543838	-0.012749991	0.122293829	0.999933887	-0.204469453	-0.648154845	0.443685393	0.268440149
ENSG00000204253	*HNRNPCP2*	-0.030632061	-0.133305889	-0.102951604	-0.030354286	0.999933887	1.216325794	1.695917665	-0.479591871	0.268440149
ENSG00000147439	*BIN3*	6.141072056	-0.021202434	-0.053318623	0.032116189	0.999933887	0.161629615	0.275702304	-0.114072688	0.268450304
ENSG00000156958	*GALK2*	1.354195946	-0.077402011	0.026791997	-0.104194008	0.999933887	-0.187059564	-0.452157401	0.265097837	0.26859582
ENSG00000088876	*ZNF343*	0.442584944	0.01752839	-0.281149078	0.298677468	0.999933887	0.305370145	0.00081848	0.304551664	0.268899096
ENSG00000184428	*TOP1MT*	2.221722967	0.098297474	-0.035708566	0.13400604	0.999933887	0.165159192	-0.04428328	0.209442472	0.269212325
ENSG00000136436	*CALCOCO2*	7.005696381	-0.061182693	-0.07999871	0.018816017	0.999933887	0.062299344	0.153276437	-0.090977094	0.269363502
ENSG00000069275	*NUCKS1*	4.40167743	-0.042571352	-0.037688462	-0.00488289	0.999933887	0.053848465	0.192191149	-0.138342685	0.269781635
ENSG00000083520	*DIS3*	3.933419187	-0.111691454	-0.196387006	0.084695552	0.999933887	-0.341189516	-0.09274106	-0.248448456	0.27005471
ENSG00000148090	*AUH*	2.103426415	0.068576128	-0.001408555	0.069984683	0.999933887	-0.145107648	-0.340807904	0.195700255	0.270123756
ENSG00000100888	*CHD8*	6.277800517	-0.04198681	-0.127902507	0.085915697	0.999933887	-0.34449646	-0.466031199	0.121534739	0.270123756
ENSG00000135083	*CCNJL*	3.3020445	0.037613609	0.013141895	0.024471713	0.999933887	0.282123689	0.005100131	0.277023558	0.270123756
ENSG00000279748	*AC008764.9*	-0.369872791	0.051280057	0.381456774	-0.330176717	0.999933887	0.0662154	0.561450928	-0.495235528	0.270123756
ENSG00000100532	*CGRRF1*	2.098702934	-0.019566337	-0.103786608	0.08422027	0.999933887	-0.094224945	0.126367038	-0.220591983	0.270323065
ENSG00000157500	*APPL1*	2.614839314	0.028556925	-0.061304375	0.0898613	0.999933887	-0.187435247	0.080195755	-0.267631001	0.270458519
ENSG00000198805	*PNP*	5.155111644	0.094812927	0.145300524	-0.050487597	0.999933887	-0.104320317	0.09233085	-0.196651167	0.270725658
ENSG00000167702	*KIFC2*	4.62170921	0.114160785	0.206307893	-0.092147107	0.999933887	0.778125362	1.038790752	-0.26066539	0.270725658

ENSG00000066455	*GOLGA5*	3.789396785	-0.045253677	-0.117032838	0.071779161	0.999933887	-0.266661683	-0.121675438	-0.144986245	0.270725658
ENSG00000196367	*TRRAP*	5.64019612	0.057914801	0.011690048	0.046224753	0.999933887	-0.026625089	-0.14584745	0.119222361	0.271145385
ENSG00000152749	*GPR180*	0.016872896	-0.085070818	-0.194629465	0.109558646	0.999933887	-0.205041037	0.190348059	-0.395389096	0.271146852
ENSG00000185262	*UBALD2*	8.699214568	0.035826867	0.025236953	0.010589914	0.999933887	0.014732661	0.155426119	-0.140693459	0.271210195
ENSG00000175866	*BAIAP2*	3.08536533	0.029116131	0.015175547	0.013940584	0.999933887	0.087755867	0.300374513	-0.212618646	0.27158916
ENSG00000259984	*AL928711.1*	-0.910191433	-0.271788034	-0.160186514	-0.11160152	0.999933887	0.320604028	-0.185630514	0.506234542	0.271989589
ENSG00000090776	*EFNB1*	0.727527035	-0.070537608	0.164713258	-0.235250865	0.999933887	0.158056249	0.510766181	-0.352709932	0.2720204
ENSG00000166432	*ZMAT1*	2.880367181	-0.085239278	-0.301149167	0.215909889	0.999933887	-0.420734249	0.073065601	-0.493799849	0.2720204
ENSG00000134107	*BHLHE40*	8.590299863	-0.006890664	-0.060909942	0.054019278	0.999933887	0.507483543	0.632269187	-0.124785644	0.2720204
ENSG00000135837	*CEP350*	5.548484521	-0.026149825	-0.103576947	0.077427121	0.999933887	-0.488940508	-0.129196217	-0.359744291	0.272043983
ENSG00000183018	*SPNS2*	1.873273131	-0.021788628	-0.088051063	0.066262435	0.999933887	-0.786076394	-1.089627831	0.303551437	0.272245967
ENSG00000267278	*MAP3K14-AS1*	1.832058079	0.02036937	0.102227125	-0.081857755	0.999933887	0.219743255	-0.095781164	0.31552442	0.272281334
ENSG00000135655	*USP15*	8.691994775	-0.079356461	-0.183078807	0.103722346	0.999933887	-0.538356505	-0.257457589	-0.280898916	0.272548391
ENSG00000116747	*RO60*	4.344272725	-0.046789909	-0.039433108	-0.007356801	0.999933887	-0.551956184	-0.265372863	-0.286583321	0.272548391
ENSG00000154222	*CC2D1B*	6.089022171	-0.006266615	0.055263456	-0.061530071	0.999933887	-0.115856939	-0.009418295	-0.106438644	0.272548391
ENSG00000168298	*H1-4*	0.148197138	0.000574479	-0.201356393	0.201930873	0.999933887	0.320062618	-0.182473621	0.502536239	0.272548391
ENSG00000116984	*MTR*	3.92563646	-0.027422821	-0.030945743	0.003522922	0.999933887	0.040535778	0.190520576	-0.149984799	0.272720982
ENSG00000181523	*SGSH*	7.562273086	-0.053122333	-0.076130755	0.023008422	0.999933887	0.115257367	0.226965805	-0.111708438	0.272787045
ENSG00000148690	*FRA10AC1*	1.916475916	0.094054542	-0.053808013	0.147862555	0.999933887	-0.21934168	0.063870346	-0.283212026	0.27338867
ENSG00000075151	*EIF4G3*	5.573682417	0.001884505	-0.064160019	0.066044524	0.999933887	-0.083788799	0.028467335	-0.112256134	0.273449153
ENSG00000246982	*Z84485.1*	0.268584496	0.185257449	0.225120089	-0.03986264	0.999933887	0.301845883	0.63096238	-0.329116497	0.273494603
ENSG00000162227	*TAF6L*	1.126526493	0.137366473	-0.073984886	0.211351359	0.999933887	0.082697898	-0.166500008	0.249197906	0.274390559
ENSG00000070371	*CLTCL1*	0.866214969	-0.026714941	-0.066892298	0.040177357	0.999933887	-0.465089275	-0.905311322	0.440222047	0.274390559
ENSG00000134470	*IL15RA*	1.440561588	-0.183521103	0.050617966	-0.23413907	0.999933887	0.865671962	1.203554711	-0.337882749	0.274734375
ENSG00000261442	*AC023830.1*	0.13140331	0.222889337	-0.062243485	0.285132822	0.999933887	-0.005129887	-0.50752498	0.502395093	0.27516543
ENSG00000233230	*AC079807.1*	0.035336511	-0.043819436	0.014883549	-0.058702985	0.999933887	0.344674688	-0.000355216	0.345029904	0.275212397
ENSG00000144290	*SLC4A10*	-0.695977701	-0.170928294	-0.315127071	0.144198776	0.999933887	-0.286215927	0.328251181	-0.614467109	0.275213185
ENSG00000162461	*SLC25A34*	2.754326534	-0.01490416	0.124407456	-0.139311616	0.999933887	0.615413471	0.845142408	-0.229728937	0.275697734
ENSG00000249898	*MCPH1-AS1*	0.104234291	0.108410545	-0.174194317	0.282604862	0.999933887	1.581000226	2.106359877	-0.525359651	0.27590668
ENSG00000227591	*HSD11B1-AS1*	2.938427847	0.173304641	0.060155752	0.113148888	0.999933887	1.11643837	1.315289153	-0.198850783	0.275995132
ENSG00000189266	*PNRC2*	5.726782622	-0.086564176	-0.072039587	-0.014524589	0.999933887	-0.031256292	0.080127982	-0.111384273	0.276132028
ENSG00000225831	*RPS18P1*	-0.639894608	0.104328317	0.351719415	-0.247391098	0.999933887	-0.448755518	0.069364582	-0.5181201	0.276132028
ENSG00000135486	*HNRNPA1*	7.598945592	-0.068140163	-0.138570109	0.070429945	0.999933887	0.076229263	0.179948687	-0.103719424	0.27639565
ENSG00000013441	*CLK1*	8.607286562	-0.055440299	-0.058946659	0.003506361	0.999933887	-0.021349205	0.136076268	-0.157425472	0.27639565
ENSG00000148334	*PTGES2*	2.896927484	0.031954781	0.133658806	-0.101704025	0.999933887	0.161153978	0.00410187	0.157052108	0.27639565
ENSG00000254789	*AC073172.1*	1.342482479	0.011451712	0.046436452	-0.03498474	0.999933887	-0.920157315	-1.294241532	0.374084217	0.27639565
ENSG00000114098	*ARMC8*	4.125817928	-0.030796984	-0.061957497	0.031160513	0.999933887	-0.154515829	-0.306430997	0.151915168	0.276656979
ENSG00000205622	*AP001042.1*	0.943034243	0.286378263	0.566788787	-0.280410524	0.999933887	1.975932007	2.511175118	-0.535243111	0.276722527
ENSG00000140950	*MEAK7*	1.364490072	-0.0442772	-0.105964806	0.061687606	0.999933887	0.170131246	0.446350768	-0.276219522	0.276789347
ENSG00000126746	*ZNF384*	4.460491468	-0.037034908	-0.029848892	-0.007186016	0.999933887	-0.360440306	-0.497399259	0.136958954	0.276824331
ENSG00000228037	*AL139246.3*	2.038037642	-0.093379546	0.015001069	-0.108380615	0.999933887	1.584395528	1.81257836	-0.228182833	0.276824331
ENSG00000183617	*MRPL54*	3.367115144	0.010694756	-0.011157104	0.02185186	0.999933887	0.201166368	0.413368736	-0.212202368	0.277026512
ENSG00000250069	*AC011379.1*	2.476347991	0.233957957	0.428158292	-0.194200335	0.999933887	0.527626614	0.898812058	-0.371185444	0.27720024
ENSG00000278259	*MYO19*	2.912830039	-0.12432286	0.104358342	-0.228681202	0.944094537	0.067031264	-0.081922339	0.148953602	0.27720024
ENSG00000221949	*LINC01465*	1.3591687	0.264758064	0.209442147	0.055315918	0.999933887	2.12002447	2.524159275	-0.404134805	0.27720024
ENSG00000128272	*ATF4*	8.426616977	0.054700143	0.100064516	-0.045364373	0.999933887	-0.002466212	-0.113192775	0.110726564	0.27720024
ENSG00000198663	*C6orf89*	6.044223312	-0.04626023	-0.022292366	-0.023967864	0.999933887	-0.171466408	-0.055348061	-0.116118348	0.27720024
ENSG00000227671	*AL390728.4*	4.645705531	-0.051957885	-0.121370062	0.069412177	0.999933887	0.127473867	0.42897958	-0.301505714	0.27720024
ENSG00000172748	*ZNF596*	1.14092899	0.027590543	0.047268036	-0.019677493	0.999933887	0.176719184	0.438053992	-0.261334808	0.27720024
ENSG00000187775	*DNAH17*	5.559287431	0.013982777	0.034100562	-0.020117785	0.999933887	0.653024369	0.904226113	-0.251201744	0.27720024
ENSG00000125445	*MRPS7*	2.018318304	0.000261868	-0.008610974	0.008872843	0.999933887	0.100865131	-0.090659789	0.191524921	0.27720024
ENSG00000100290	*BIK*	1.078509623	-0.086723958	-0.148917383	0.062193425	0.999933887	0.2227059	0.542871858	-0.320165958	0.27771342
ENSG00000144043	*TEX261*	4.120118334	-0.00021371	0.034466633	-0.034680342	0.999933887	-0.200684104	-0.340423514	0.13973941	0.27771342
ENSG00000099385	*BCL7C*	1.603636698	0.139731115	0.154665295	-0.01493418	0.999933887	0.130071657	-0.175878084	0.305949741	0.277788783
ENSG00000172270	*BSG*	7.559088295	0.049190588	0.00649081	0.042699778	0.999933887	-0.088898815	-0.217026819	0.128128004	0.277887968
ENSG00000143919	*CAMKMT*	0.229910135	-0.049278195	-0.242887427	0.193609231	0.999933887	-0.169359333	0.269964673	-0.439324006	0.277913063
ENSG00000239264	*TXNDC5*	0.059866445	0.061817282	0.054399411	0.00741787	0.999933887	0.085861404	-0.403864259	0.489725663	0.278200723
ENSG00000179406	*LINC00174*	3.299993653	0.007192623	-0.036292748	0.043485371	0.999933887	0.419267219	0.628465178	-0.209197958	0.278394724
ENSG00000171659	*GPR34*	0.428731112	-0.08590618	-0.332454825	0.246548645	0.999933887	-0.275170683	0.040921807	-0.316092491	0.278457362
ENSG00000274184	*AC011815.2*	-0.441257193	0.417561044	0.10414167	0.313419375	0.999933887	-1.137043692	-0.71645253	-0.420591161	0.278522778
ENSG00000182551	*ADI1*	3.058108024	-0.023581492	0.002149583	-0.025731075	0.999933887	-0.063020631	-0.270929808	0.207909177	0.278522778
ENSG00000197343	*ZNF655*	6.079793294	0.016353273	-0.026995355	0.043348628	0.999933887	-0.178403777	-0.070842291	-0.107561486	0.278651684
ENSG00000131408	*NR1H2*	7.519665253	0.073806263	0.038133428	0.035672835	0.999933887	0.312337947	0.428185545	-0.115847598	0.278760383
ENSG00000135723	*FHOD1*	5.48551065	-0.050962938	0.023781368	-0.074744306	0.999933887	0.050061608	-0.104322379	0.154383986	0.278896867
ENSG00000175356	*SCUBE2*	-0.51355272	-0.043597953	0.164466187	-0.208064141	0.999933887	0.899164984	1.334101099	-0.434936115	0.278901251
ENSG00000129083	*COPB1*	5.469658968	-0.085030792	-0.09539937	0.010368578	0.999933887	-0.328843271	-0.183548822	-0.14529445	0.278970443
ENSG00000221838	*AP4M1*	1.394602786	-0.030446055	0.139396294	-0.169842349	0.999933887	0.236871918	0.45981808	-0.222946162	0.27917681
ENSG00000133302	*SLF1*	3.193802257	0.109160182	-0.06239379	0.171553973	0.999933887	-0.439113323	-0.087319788	-0.351793535	0.279345469
ENSG00000240445	*FOXO3B*	0.057114933	-0.018959543	0.449071651	-0.468031194	0.999933887	-0.184094307	0.257978188	-0.442072495	0.280022919
ENSG00000172336	*POP7*	1.274768664	-0.036724121	0.092131721	-0.128855842	0.999933887	0.112434111	-0.193084166	0.305518277	0.280096223
ENSG00000143633	*C1orf131*	1.741368877	-0.018362033	-0.256056138	0.237694104	0.999933887	-0.177129682	-0.378203466	0.201073784	0.280096223
ENSG00000168004	*PLAAT5*	0.393607791	-0.12473551	0.215643925	-0.340379435	0.944094537	-0.132622261	0.307862149	-0.44048441	0.280207593
ENSG00000120727	*PAIP2*	6.031989062	-0.018777199	-0.12422656	0.105449361	0.999933887	-0.180295207	-0.057896168	-0.12239904	0.280222681
ENSG00000075884	*ARHGAP15*	5.957455887	-0.013598791	-0.073469106	0.059870315	0.999933887	0.00625548	0.148528925	-0.142273445	0.280288323
ENSG00000113580	*NR3C1*	8.041959291	-0.024632676	-0.039424375	0.014791699	0.999933887	0.075700525	0.204056908	-0.128356384	0.280650011
ENSG00000278133	*AC135050.6*	2.65553361	-0.01322511	-0.012364711	-0.000860399	0.999933887	-0.139242433	-0.35067596	0.211433527	0.280650011
ENSG00000182985	*CADM1*	-0.23796074	0.003485361	0.283178884	-0.279693523	0.999933887	-0.362140781	0.10752351	-0.469664291	0.280834459
ENSG00000130023	*ERMARD*	1.992167778	-0.001947077	0.208938992	-0.210886069	0.999933887	0.033380689	-0.146219167	0.179599856	0.280981326
ENSG00000113318	*MSH3*	1.791626264	-0.02459746	0.013178702	-0.037776162	0.999933887	-0.043091548	-0.290488884	0.247397337	0.281030444
ENSG00000115339	*GALNT3*	3.788402556	-0.104306341	-0.066493984	-0.037812358	0.999933887	-0.474357114	-0.679223368	0.204866254	0.28103047
ENSG00000035681	*NSMAF*	8.591189648	0.021735162	0.013338094	0.008397068	0.999933887	0.711157443	0.861195741	-0.150038298	0.281101419
ENSG00000278969	*AC026310.2*	-0.121546147	-0.000638088	0.079424147	-0.080062235	0.999933887	0.31723304	0.711217126	-0.393984086	0.281181739
ENSG00000106714	*CNTNAP3*	3.767438667	0.017113768	0.058708594	-0.041594826	0.999933887	-0.329434961	-0.484650558	0.155215597	0.281243516
ENSG00000163378	*EOGT*	1.890325282	0.041473844	-0.126268595	0.167742439	0.999933887	0.054742211	0.360402769	-0.305660558	0.281564565
ENSG00000168280	*KIF5C*	1.300661339	0.021178623	-0.249153457	0.27033208	0.999933887	-0.160539899	0.106139536	-0.266679435	0.281564565
ENSG00000162599	*NFIA*	0.458125347	-0.061469193	-0.217846625	0.156377432	0.999933887	-0.679747219	-0.097499677	-0.582247542	0.281916543
ENSG00000255538	*OR10V2P*	1.013450476	0.027362843	0.097012784	-0.069649941	0.999933887	0.340400843	0.601097515	-0.260696672	0.282358822
ENSG00000121807	*CCR2*	0.970973056	-0.004282144	-0.048714709	0.044432564	0.999933887	-0.134177339	0.095140157	-0.229317496	0.282358822
ENSG00000184162	*NR2C2AP*	1.021146402	0.057639535	0.220467997	-0.162828461	0.999933887	0.164351592	-0.092767946	0.257119537	0.282676006
ENSG00000110172	*CHORDC1*	4.264199685	-0.039865545	-0.051213071	0.011347526	0.999933887	-0.134154061	0.192069828	-0.326223889	0.282676006
ENSG00000100263	*RHBDD3*	1.863303365	-0.018044368	0.024531299	-0.042575667	0.999933887	-0.205472123	-0.405981332	0.200509209	0.282676006
ENSG00000273841	*TAF9*	4.655405699	0.013981518	-0.085322028	0.099303545	0.999933887	0.060910198	0.205818088	-0.144907889	0.282912232
ENSG00000187912	*CLEC17A*	3.036858135	-0.097720618	-0.066314182	-0.031406437	0.999933887	-0.575109586	-0.800035709	0.224926123	0.283055333
ENSG00000186001	*LRCH3*	4.162500204	-0.003070693	-0.009291751	0.006221057	0.999933887	0.177008898	0.295887531	-0.118878632	0.283055333
ENSG00000064995	*TAF11*	3.342228569	0.010020859	0.074284774	-0.064263915	0.999933887	-0.080517206	-0.231842046	0.15132484	0.283056818
ENSG00000046651	*OFD1*	4.88694655	-0.038934009	-0.116763702	0.077829693	0.999933887	0.191079487	0.397912407	-0.20683292	0.283209976
ENSG00000283355	*AC074194.2*	-2.258740839	-0.035678623	-0.159941971	0.124263348	0.999933887	-0.353083034	0.09233581	-0.445418844	0.283307596
ENSG00000165280	*VCP*	7.701638793	0.012238808	-0.021834002	0.03407281	0.999933887	-0.150118653	-0.032252298	-0.117866355	0.283328145
ENSG00000121274	*TENT4B*	3.749683542	0.065642284	0.067801155	-0.002158871	0.999933887	-0.281130956	-0.494825538	0.213694581	0.28370033
ENSG00000225313	*AL513327.1*	3.547219897	-0.170252248	-0.126183983	-0.044068265	0.999933887	-1.185318051	-1.412152483	0.226834432	0.283960695
ENSG00000055118	*KCNH2*	0.778055298	0.00441588	0.048252679	-0.043836799	0.999933887	-0.103529627	0.263982348	-0.367511975	0.284022567
ENSG00000262919	*CCNQ*	2.032299512	0.05054627	-0.068184037	0.118730307	0.999933887	0.083433718	-0.181511669	0.264945387	0.284495462
ENSG00000260260	*SNHG19*	0.195732722	0.006071682	0.081639096	-0.075567414	0.999933887	0.101301701	-0.193732044	0.295033745	0.28488271
ENSG00000119705	*SLIRP*	1.717603668	-0.057894492	-0.093933803	0.036039311	0.999933887	-0.057046054	0.188272124	-0.245318178	0.284914845
ENSG00000258738	*AL121603.2*	3.887913445	0.111283099	0.194711243	-0.083428145	0.999933887	1.747816878	1.930069065	-0.182252187	0.284932823

ENSG00000174442	*ZWILCH*	0.363244912	-0.093025416	-0.089761235	-0.00326418	0.999933887	-0.020382651	0.417954169	-0.43833682	0.284932823
ENSG00000174705	*SH3PXD2B*	-0.567571259	-0.053450565	-0.231440451	0.177989887	0.999933887	1.703877754	2.198589052	-0.494691298	0.284932823
ENSG00000256667	*KLRA1P*	1.083901385	0.138142872	-0.209513468	0.347656339	0.999933887	-0.064225821	0.267008478	-0.331234298	0.285468443
ENSG00000149489	*ROM1*	0.567511893	0.091708279	0.057376944	0.034331335	0.999933887	1.383089031	1.816578283	-0.433489252	0.285468443
ENSG00000003147	*ICA1*	0.212099652	-0.052893325	-0.117353748	0.064460423	0.999933887	0.113144476	0.495725685	-0.382581208	0.285468443
ENSG00000198498	*TMA16*	0.09839524	0.066210317	0.005155323	0.061054994	0.999933887	0.198359938	0.501365315	-0.303005377	0.285614516
ENSG00000136279	*DBNL*	7.61258067	-0.01857991	0.00905171	-0.027631621	0.999933887	-0.191500001	-0.296985684	0.105485683	0.285614516
ENSG00000241772	*AC092620.1*	0.305231002	-0.040820441	0.281571899	-0.32239234	0.999933887	-0.023677799	0.430917531	-0.45459533	0.285614516
ENSG00000187735	*TCEA1*	3.510029847	-0.019726973	-0.01143308	-0.008293893	0.999933887	-0.032912487	0.111254076	-0.144166563	0.285628632
ENSG00000166902	*MRPL16*	3.29524444	-0.12043595	0.049625367	-0.170061317	0.999933887	-0.009898612	0.151173184	-0.161071797	0.285799451
ENSG00000226029	*LINC01772*	0.917017397	-0.201702105	0.151697622	-0.353399727	0.999933887	0.227370939	0.495733328	-0.26836239	0.285799451
ENSG00000104388	*RAB2A*	6.707991462	-0.0289408	-0.020776189	-0.008164611	0.999933887	-0.560869406	-0.447639431	-0.113229975	0.285799451
ENSG00000130749	*ZC3H4*	5.711489037	0.013693819	0.04023933	-0.026545511	0.999933887	0.227949906	0.339780324	-0.111830418	0.285799451
ENSG00000123342	*MMP19*	2.492035941	-0.156082505	-0.281403004	0.125320499	0.999933887	-0.851402728	-1.074649341	0.223246613	0.285830904
ENSG00000215712	*TMEM242*	1.363236744	-0.171313089	-0.198617872	0.027304783	0.999933887	0.177955278	0.423088133	-0.245132855	0.286391497
ENSG00000177410	*ZFAS1*	4.907893026	-0.072562302	-0.060672571	-0.011889731	0.999933887	-0.060295072	-0.179663258	0.119368186	0.286391497
ENSG00000121966	*CXCR4*	12.034696704	0.071801421	0.110887076	-0.039085655	0.999933887	-0.394820122	-0.254847859	-0.139972263	0.286391497
ENSG00000196756	*SNHG17*	3.181348503	-0.053107209	-0.002610999	-0.05049621	0.999933887	0.077888133	-0.08611698	0.164005113	0.286391497
ENSG00000108691	*CCL2*	3.114443579	0.037710391	0.139692266	-0.101981874	0.999933887	-1.226499004	-1.554262295	0.327763291	0.286391497
ENSG00000124201	*ZNFX1*	8.578014237	-0.036434054	-0.05553424	0.019100187	0.999933887	0.47702346	0.589744832	-0.112721373	0.286606187
ENSG00000160271	*RALGDS*	8.679815513	0.095933701	0.119098	-0.023164298	0.999933887	1.032931589	1.199806249	-0.16687466	0.286701828
ENSG00000112335	*SNX3*	5.621069583	0.041321037	-0.06258451	0.103905547	0.999933887	-0.595358571	-0.736907923	0.141549352	0.286701828
ENSG00000135390	*ATP5MC2*	4.657020735	0.026261903	-0.013848223	0.040110126	0.999933887	-0.011888465	-0.151320983	0.139432517	0.286701828
ENSG00000140396	*NCOA2*	6.670166789	-0.022363531	-0.071170295	0.048806764	0.999933887	-0.713903122	-0.917927349	0.204024226	0.286701828
ENSG00000241743	*XACT*	-1.495236294	-1.219171742	-0.206594579	-1.012577163	0.999933887	-1.489208745	-0.635027316	-0.85418143	0.28675871
ENSG00000148218	*ALAD*	2.704261374	-0.022091543	0.034417386	-0.056508929	0.999933887	0.012522564	-0.184580755	0.197103318	0.28675871
ENSG00000144741	*SLC25A26*	2.14451982	-0.076814389	-0.148511197	0.071696809	0.999933887	0.168416951	0.369454293	-0.201037341	0.286821154
ENSG00000154114	*TBCEL*	0.768193556	0.122687335	0.120902389	0.001784947	0.999933887	-0.183026264	0.152001786	-0.33502805	0.286821154
ENSG00000270000	*AC005479.2*	0.014793036	-0.088787697	0.262806171	-0.351593868	0.999933887	-0.019107802	0.28801648	-0.307124282	0.286821154
ENSG00000150768	*DLAT*	1.36548977	0.003674588	0.058772536	-0.055097948	0.999933887	-0.008902176	0.232313739	-0.241215914	0.286926577
ENSG00000162585	*FAAP20*	3.839035338	0.078495375	0.000876057	0.077619319	0.999933887	-0.112355446	-0.274645282	0.162289835	0.286936361
ENSG00000166321	*NUDT13*	0.524521751	0.210111815	0.116313304	0.093798511	0.999933887	0.143311688	0.693282987	-0.549971299	0.286936361
ENSG00000110917	*MLEC*	3.368206233	-0.038764077	-0.044398683	0.005634606	0.999933887	-0.061463335	-0.209213746	0.147750411	0.286936361
ENSG00000138613	*APH1B*	3.842148338	-0.049064662	0.043598146	-0.092662808	0.999933887	-0.227443673	-0.364719785	0.137276113	0.286955831
ENSG00000102218	*RP2*	6.272979765	0.029390544	0.044545116	-0.015154572	0.999933887	-0.225834837	-0.069922977	-0.155911861	0.286955831
ENSG00000183955	*KMT5A*	4.426561532	0.067530442	0.058362026	0.009168415	0.999933887	0.511412363	0.659729376	-0.148317012	0.2870818
ENSG00000176490	*DIRAS1*	-1.336713655	0.250160347	-0.164013675	0.414174022	0.999933887	-0.750897801	-0.134538388	-0.616359413	0.2870818
ENSG00000205268	*PDE7A*	5.638469883	0.013626704	0.018079798	-0.004453094	0.999933887	0.085456348	0.226944344	-0.141487996	0.2870818
ENSG00000129749	*CHRNA10*	2.79740796	0.038122286	0.093995742	-0.055873457	0.999933887	0.59236709	0.823874162	-0.231507072	0.287168449
ENSG00000204439	*C6orf47*	3.516982796	0.083190708	0.06308054	0.020110168	0.999933887	-0.504483658	-0.696891087	0.192407428	0.287317707
ENSG00000125245	*GPR18*	1.687502256	-0.147079891	-0.056525507	-0.090554384	0.999933887	-0.002447234	0.266211395	-0.268658629	0.287507271
ENSG00000128708	*HAT1*	3.697193641	-0.088660734	-0.163597075	0.074936342	0.999933887	0.194264073	0.437340401	-0.243076328	0.287507271
ENSG00000276337	*AC105429.1*	-0.661304824	0.201543681	0.114581561	0.08696212	0.999933887	0.339659609	0.791544618	-0.451885009	0.287507271
ENSG00000075407	*ZNF37A*	1.610759869	-0.064157431	-0.024601356	-0.039556075	0.999933887	-0.268721456	0.118059805	-0.386781261	0.287507271
ENSG00000108953	*YWHAE*	6.090339472	-0.054708259	-0.031894439	-0.02281382	0.999933887	0.335366816	0.458651076	-0.12328426	0.287519859
ENSG00000092200	*RPGRIP1*	1.294972907	-0.084041221	-0.015489822	-0.068551399	0.999933887	-0.864851802	-1.347429697	0.482577896	0.287554317
ENSG00000154803	*FLCN*	7.881159032	-0.054300159	-0.010950737	-0.043349422	0.999933887	0.133940688	0.245229869	-0.111289181	0.287726654
ENSG00000281026	*N4BP2L2-IT2*	2.094553362	-0.242168803	0.011944637	-0.254113441	0.999933887	-0.008732291	0.32456412	-0.333296411	0.287731847
ENSG00000273112	*AL590385.2*	1.066949222	-0.1197242	-0.019848866	-0.099875315	0.999933887	0.915676962	1.265110193	-0.349433231	0.287898439
ENSG00000226067	*LINC00623*	2.500103182	-0.041894184	-0.093852685	0.051958501	0.999933887	0.003264587	-0.230313974	0.233578561	0.28795106
ENSG00000083223	*TUT7*	8.244547187	-0.07170838	-0.085719241	0.014010861	0.999933887	-0.550957142	-0.363665316	-0.187291826	0.288173242
ENSG00000111666	*CHPT1*	3.19915736	0.029692592	0.035113519	-0.005420927	0.999933887	-0.067768703	-0.239417579	0.171648876	0.288249213
ENSG00000151287	*TEX30*	0.602214968	0.031060316	-0.095556249	0.126616564	0.999933887	0.021026714	-0.320348403	0.341375117	0.288249213
ENSG00000053438	*NNAT*	0.196577742	0.224243643	-0.109467743	0.333711386	0.999933887	-0.439935357	-0.742185028	0.302249671	0.288372668
ENSG00000197579	*TOPORS*	4.666928309	-0.112473257	-0.198303193	0.085829936	0.999933887	-0.792218381	-1.004505695	0.212287315	0.288599766
ENSG00000215039	*CD27-AS1*	0.831826575	-0.069634947	-0.140048062	0.070413116	0.999933887	-0.124600345	-0.459233769	0.334633424	0.288599766
ENSG00000211696	*TRGV8*	-0.794009336	0.163498637	-0.172786444	0.336285081	0.999933887	0.181406876	0.641334993	-0.459928117	0.288665157
ENSG00000101557	*USP14*	3.175478047	-0.136637687	-0.038035067	-0.09860262	0.999933887	0.056465914	0.216455114	-0.1599892	0.28873381
ENSG00000168795	*ZBTB5*	3.097828943	-0.050693006	-0.082994461	0.032301455	0.999933887	0.378759968	0.568161292	-0.189401324	0.28873381
ENSG00000126259	*KIRREL2*	-0.757110984	0.171116006	0.00228962	0.168826386	0.999933887	2.733374514	3.371110113	-0.637735599	0.288948221
ENSG00000114125	*RNF7*	2.869750962	0.033516976	-0.045852529	0.079369505	0.999933887	-0.190547012	-0.391495613	0.200948602	0.289462553
ENSG00000131653	*TRAF7*	4.839495041	0.058000597	0.046555213	0.011445384	0.999933887	-0.211585728	-0.342093754	0.130508026	0.289905984
ENSG00000012983	*MAP4K5*	2.904763333	-0.070070807	-0.114341084	0.044270277	0.999933887	-0.126589724	0.159271211	-0.285860935	0.290165997
ENSG00000102781	*KATNAL1*	0.52264102	-0.076836812	-0.257092591	0.180255778	0.999933887	-0.142458306	0.251168008	-0.393626314	0.290171288
ENSG00000110851	*PRDM4*	3.891034862	0.015631648	0.007900282	0.007731367	0.999933887	-0.267829767	-0.41603025	0.148200483	0.290251101
ENSG00000089558	*KCNH4*	-0.790428772	0.062624738	-0.287384593	0.350009331	0.999933887	0.150671309	0.708721727	-0.558050418	0.290537006
ENSG00000273311	*DGCR11*	1.326019582	-0.005620673	-0.072643356	0.067022683	0.999933887	-0.185121157	-0.596402622	0.411281465	0.290560694
ENSG00000185432	*METTL7A*	1.77605804	-0.144827932	-0.016658044	-0.128169888	0.999933887	0.195244948	0.475883985	-0.280639037	0.290583249
ENSG00000091490	*SEL1L3*	4.248062843	-0.011354666	0.026950076	-0.038304743	0.999933887	0.13944928	0.267896534	-0.128447254	0.290600652
ENSG00000056277	*ZNF280C*	-0.153473481	-0.255647435	-0.000524517	-0.255122918	0.999933887	-0.021910694	0.4418342	-0.463744894	0.290992846
ENSG00000114062	*UBE3A*	3.763472716	0.033266204	-0.039762586	0.07302879	0.999933887	-0.120009901	0.023744057	-0.143753959	0.290992846
ENSG00000108924	*HLF*	-0.224693161	0.04962882	-0.341417543	0.391046363	0.999933887	0.138699779	0.549579372	-0.410879593	0.290992846
ENSG00000100335	*MIEF1*	3.576476048	-0.068078723	-0.174495492	0.106416769	0.999933887	-0.259877706	-0.416926968	0.157049263	0.291205353
ENSG00000029363	*BCLAF1*	7.142459828	-0.042293744	-0.190051239	0.147757495	0.999933887	-0.357748079	-0.07940069	-0.278347389	0.291317819
ENSG00000108960	*MMD*	0.564241552	0.101714683	0.160188903	-0.058474221	0.999933887	-0.131279937	0.207432173	-0.33871211	0.291323958
ENSG00000236914	*LINC01852*	-1.054550973	0.364275064	0.359449728	0.004825336	0.999933887	0.682072203	1.427988325	-0.745916121	0.291330202
ENSG00000156050	*FAM161B*	-0.079755105	-0.257388068	-0.150073411	-0.107314657	0.999933887	0.110961194	0.552580911	-0.441619716	0.291330202
ENSG00000185504	*FAAP100*	3.910613582	0.046640393	0.029269998	0.017370395	0.999933887	0.632074683	0.79390046	-0.161825777	0.291393238
ENSG00000196611	*MMP1*	0.997572015	-0.147930837	-0.550297153	0.402366316	0.999933887	-0.260670015	0.016609214	-0.277279228	0.291393238
ENSG00000154146	*NRGN*	1.693138415	0.039005853	-0.014109628	0.053115481	0.999933887	0.003971361	-0.264567459	0.26853882	0.291393238
ENSG00000163159	*VPS72*	2.939848858	-0.011816771	0.062178414	-0.073995185	0.999933887	-0.227573239	-0.403093537	0.175520298	0.291393238
ENSG00000158615	*PPP1R15B*	9.09924218	0.075659622	0.075967828	-0.000308206	0.999933887	0.325259246	0.419367871	-0.094108625	0.291590646
ENSG00000262155	*LINC02175*	0.174851936	0.094681973	-0.180664451	0.275346423	0.999933887	0.446435138	0.824895854	-0.378460716	0.291590646
ENSG00000228363	*AC015971.1*	3.916274292	-0.027761656	0.067267697	-0.095029353	0.999933887	0.180163723	0.376970445	-0.196806722	0.291590646
ENSG00000174579	*MSL2*	6.696183222	-0.060103682	-0.031771376	-0.028332306	0.999933887	-0.247913856	-0.350800552	0.102886696	0.291692367
ENSG00000175398	*OR10P1*	-0.092161883	0.268394403	0.321341281	-0.052946878	0.999933887	1.205384299	1.58001717	-0.374632871	0.291692367
ENSG00000070269	*TMEM260*	2.969673418	0.034734651	-0.034878297	0.069612948	0.999933887	-0.165133007	-0.374127802	0.208994795	0.291692367
ENSG00000140386	*SCAPER*	2.543093435	0.021868411	-0.151444649	0.173313061	0.999933887	-0.116548324	0.109474481	-0.226022805	0.291692367
ENSG00000142937	*RPS8*	7.623218405	0.00247836	-0.038071159	0.040549519	0.999933887	0.106590683	0.232167128	-0.125576445	0.291692367
ENSG00000130598	*TNNI2*	5.248733761	-0.054385007	-0.032832899	-0.021552108	0.999933887	-0.546070153	-0.734968841	0.188898688	0.291891071
ENSG00000171204	*TMEM126B*	3.100907118	-0.095206364	-0.201423985	0.106217621	0.999933887	-0.416045216	-0.57489722	0.158852005	0.291960637
ENSG00000184584	*STING1*	3.601090489	0.076292926	-0.000196021	0.076488948	0.999933887	0.129532186	-0.019049648	0.148581835	0.291978354
ENSG00000065183	*WDR3*	2.100249573	-0.062573995	-0.030551954	-0.032022041	0.999933887	0.084788372	0.339639472	-0.2548511	0.291978354
ENSG00000213096	*ZNF254*	3.860671196	-0.091693526	-0.16810895	0.076415424	0.999933887	-0.850135454	-0.606141863	-0.243993591	0.292203829
ENSG00000157303	*SUSD3*	2.305586584	0.029981674	-0.071228143	0.101209817	0.999933887	-0.256513053	-0.46717753	0.210664477	0.292290841
ENSG00000048140	*TSPAN17*	3.024361955	-0.000137055	-0.060149938	0.060012884	0.999933887	-0.067723327	-0.242572906	0.174849579	0.292345791
ENSG00000286242	*AC018797.3*	2.287423924	0.123978397	0.152570005	-0.028591608	0.999933887	-0.290358021	0.08053046	-0.370888482	0.292453834
ENSG00000183154	*AC138356.1*	0.967477779	0.130059958	0.314680409	-0.184620451	0.999933887	0.494499092	-0.098244728	0.59274382	0.292565604
ENSG00000151623	*NR3C2*	1.26950213	-0.116399115	-0.054358229	-0.062040887	0.999933887	0.032825084	0.309217761	-0.276392677	0.29259419
ENSG00000139410	*SDSL*	-0.442444938	0.29558083	0.403235983	-0.107655152	0.999933887	-0.637148551	-0.317839898	-0.319308653	0.29259419
ENSG00000141258	*SGSM2*	5.244204496	0.042796995	0.053793787	-0.010996792	0.999933887	0.058097865	-0.058310765	0.11640863	0.292600654
ENSG00000081307	*UBA5*	2.084171598	-0.027606984	-0.139185247	0.111578263	0.999933887	-0.161953067	0.104648391	-0.266601458	0.292629844
ENSG00000239305	*RNF103*	6.933818964	-0.088716365	-0.024692859	-0.064023507	0.999933887	-0.254248882	-0.119246661	-0.135002221	0.292721309
ENSG00000147231	*RADX*	0.703487816	-0.133206367	-0.174313729	0.041107361	0.999933887	-0.109914347	0.262442334	-0.37235668	0.292965261
ENSG00000146143	*PRIM2*	0.026832867	0.12312992	0.089862714	0.033267206	0.999933887	-0.234198124	0.168528183	-0.402726307	0.292989684

ENSG00000132792	*CTNNBL1*	4.139105815	0.025635455	-0.035599537	0.061234992	0.999933887	-0.173461751	-0.310257148	0.136795397	0.293119072
ENSG00000241852	*C8orf58*	2.23805294	0.047438042	0.041730881	0.005707161	0.999933887	-0.000113522	-0.272567446	0.272453923	0.293168643
ENSG00000173114	*LRRN3*	0.981323411	0.055880948	-0.023996891	0.07987784	0.999933887	-0.215110318	0.036173267	-0.251283585	0.293246365
ENSG00000129636	*ITFG1*	2.823090703	0.019966801	0.045433547	-0.025466746	0.999933887	-0.229380466	-0.414428174	0.185047708	0.293246365
ENSG00000219545	*UMAD1*	2.505501195	-0.010475053	0.022049882	-0.032524935	0.999933887	-0.177227004	0.028676855	-0.205903859	0.293246365
ENSG00000168876	*ANKRD49*	4.311356417	-0.158045204	-0.210528447	0.052483243	0.999933887	-0.116744061	0.053266781	-0.170010842	0.293425672
ENSG00000169251	*NMD3*	3.308864504	-0.056460667	-0.07617867	0.019718003	0.999933887	-0.091767006	0.132518844	-0.22428585	0.294205937
ENSG00000110446	*SLC15A3*	9.238425703	0.06182005	0.082405427	-0.020585377	0.999933887	0.997437639	1.110494007	-0.113056367	0.294276092
ENSG00000175213	*ZNF408*	2.620030124	-0.071134857	0.002478424	-0.073613282	0.999933887	-0.345399527	-0.532163229	0.186763702	0.294423464
ENSG00000111785	*RIC8B*	0.890560441	-0.133365811	-0.040418748	-0.092947063	0.999933887	-0.030563424	0.258736426	-0.289299849	0.294573791
ENSG00000172216	*CEBPB*	9.125505557	0.263699909	0.273323652	-0.009623743	0.999933887	0.112493981	0.329989708	-0.217495727	0.295061266
ENSG00000228446	*ANKRD49P1*	-0.078526098	0.222841789	0.241550657	-0.018708868	0.999933887	-0.299238542	0.222956293	-0.522194835	0.295250122
ENSG00000255145	*STX17-AS1*	-0.845624211	0.116130462	0.266442378	-0.150311916	0.999933887	0.232244644	1.007871669	-0.775627025	0.295250122
ENSG00000087152	*ATXN7L3*	7.112451034	0.031246822	0.005802008	0.025444813	0.999933887	-0.238008708	-0.359180535	0.121171827	0.295250122
ENSG00000234912	*SNHG20*	4.142675418	-0.090296072	-0.036672353	-0.05362372	0.999933887	0.270105473	0.127886452	0.142219021	0.29532307
ENSG00000134697	*GNL2*	4.76519878	-0.04035273	-0.120391542	0.080038812	0.999933887	0.322513685	0.490658901	-0.168145216	0.29545569
ENSG00000158169	*FANCC*	-0.165125259	-0.001210672	0.070869933	-0.072080605	0.999933887	0.060325161	0.470194823	-0.409869662	0.295511449
ENSG00000134152	*KATNBL1*	8.729749927	-0.00562564	0.079305922	-0.084931562	0.999933887	0.151255026	0.280868227	-0.129613201	0.295623254
ENSG00000259865	*AL390728.6*	2.19187607	-0.130442392	0.050590328	-0.18103272	0.999933887	0.055475	0.437527572	-0.382052572	0.295716464
ENSG00000234617	*SNRK-AS1*	0.744578604	0.056613205	-0.142440429	0.199053634	0.999933887	0.026233283	0.460243589	-0.434010306	0.295716464
ENSG00000184068	*SREBF2-AS1*	2.182230176	0.105326223	0.027437159	0.077889064	0.999933887	0.808835608	1.048115649	-0.239280041	0.295928053
ENSG00000172716	*SLFN11*	3.725787442	-0.020510638	-0.082420693	0.061910055	0.999933887	-0.108919111	-0.255051104	0.146131994	0.295928053
ENSG00000105750	*ZNF85*	-0.376300655	0.059650487	-0.094522106	0.154172593	0.999933887	-0.631736226	-0.051434888	-0.580301338	0.295928053
ENSG00000161960	*EIF4A1*	4.726107279	0.24296503	0.349228059	-0.106263029	0.999933887	0.205727887	0.393082105	-0.187354218	0.295984317
ENSG00000066379	*POLR1H*	2.550313701	0.042915536	0.023125606	0.01978993	0.999933887	0.01014449	-0.199783031	0.209927521	0.296073037
ENSG00000241878	*PISD*	7.25198499	-0.052347571	-0.071627587	0.019280016	0.999933887	-0.252117623	-0.368005739	0.115888116	0.296519192
ENSG00000135317	*SNX14*	4.010962043	-0.02218791	-0.040337816	0.018149906	0.999933887	-0.325884098	-0.523505465	0.197621368	0.296519192
ENSG00000262468	*LINC01569*	-0.863561475	-0.212957011	0.46936833	-0.68232534	0.999933887	-0.243576292	0.283596417	-0.527172709	0.296555117
ENSG00000054654	*SYNE2*	7.286963854	-0.0628977	-0.004760319	-0.058137381	0.999933887	-0.403876362	-0.156182484	-0.247693878	0.296555117
ENSG00000166707	*ZCCHC18*	-0.634455483	-0.228049702	-0.114559634	-0.113490068	0.999933887	0.129699211	0.545223682	-0.415524471	0.296751747
ENSG00000121454	*LHX4*	-0.414884686	0.388732541	-0.237318691	0.626051232	0.944094537	-0.126943928	0.286650533	-0.413594461	0.29731488
ENSG00000121542	*SEC22A*	0.730875523	0.154333543	0.014401968	0.139931575	0.999933887	0.404405906	0.709032856	-0.304626951	0.297399553
ENSG00000075399	*VPS9D1*	5.713361497	0.043475967	0.071681496	-0.028205529	0.999933887	0.111364787	-0.015711861	0.127076647	0.297399553
ENSG00000106477	*CEP41*	-0.421471578	-0.122262961	-0.020324652	-0.101938309	0.999933887	-0.087677604	0.317205187	-0.404882791	0.297399553
ENSG00000100345	*MYH9*	10.672429126	0.014336789	-0.016892051	0.03122884	0.999933887	0.381882232	0.510848429	-0.128966198	0.297399553
ENSG00000231160	*KLF3-AS1*	1.597034894	0.021063488	-0.033970516	0.055034004	0.999933887	-0.273522182	-0.522124091	0.248601909	0.297399553
ENSG00000080546	*SESN1*	3.039795166	-0.018567284	-0.099029464	0.08046218	0.999933887	0.087689868	0.278614654	-0.190924786	0.29748176
ENSG00000205578	*POM121B*	-0.711603033	0.080745797	-0.066569886	0.147315684	0.999933887	-0.235451241	0.306285055	-0.541736296	0.297749785
ENSG00000251381	*LINC00958*	2.072548651	0.166764308	0.370017036	-0.203252728	0.999933887	0.310821574	0.580932162	-0.270110587	0.297795667
ENSG00000279277	*AC012020.1*	2.296718958	0.035600603	0.091559217	-0.055958614	0.999933887	0.195738345	0.406374163	-0.210635818	0.297851446
ENSG00000115159	*GPD2*	4.405591045	-0.028288764	-0.099674742	0.071385978	0.999933887	-1.033741054	-0.815593106	-0.218147948	0.298226407
ENSG00000103061	*SLC7A6OS*	1.870887929	0.073703197	-0.057204708	0.130907905	0.999933887	-0.26866557	-0.482304829	0.213639259	0.298570377
ENSG00000142065	*ZFP14*	0.442191209	0.012369495	-0.080398432	0.092767927	0.999933887	-0.231910887	0.075548539	-0.307459426	0.298597094
ENSG00000105402	*NAPA*	8.100610235	0.057433165	0.073716705	-0.01628354	0.999933887	0.236702937	0.332184467	-0.095481529	0.299120989
ENSG00000228393	*LINC01004*	2.348519824	0.134469139	0.190484003	-0.056014864	0.999933887	0.960473814	1.180605779	-0.220131965	0.299120989
ENSG00000140691	*ARMC5*	3.608018511	0.092201453	0.15041461	-0.058213157	0.999933887	0.47513476	0.687759387	-0.212624626	0.299120989
ENSG00000198198	*SZT2*	5.691959201	0.036944639	0.049122965	-0.012178326	0.999933887	-0.068472026	-0.174460366	0.105988339	0.299120989
ENSG00000149212	*SESN3*	4.333366859	-0.044054292	-0.157117242	0.11306295	0.999933887	-0.366930486	-0.093996418	-0.272934068	0.299120989
ENSG00000104267	*CA2*	3.859937273	0.079300167	0.054361182	0.024938985	0.999933887	0.663338528	0.842110089	-0.178771561	0.299434453
ENSG00000132510	*KDM6B*	11.141986875	0.132918862	0.108933785	0.023985077	0.999933887	1.046044448	1.180106822	-0.134062374	0.299446701
ENSG00000112033	*PPARD*	4.91811744	-0.037381366	-0.061026301	0.023644935	0.999933887	0.657633698	0.808080194	-0.150446496	0.299919919
ENSG00000150907	*FOXO1*	4.311436619	0.032838413	-0.013408995	0.046247408	0.999933887	-0.055887989	-0.205130646	0.149242658	0.299927919
ENSG00000107625	*DDX50*	3.711575824	0.018735893	-0.132840537	0.151576429	0.999933887	0.147379318	0.287566376	-0.140187058	0.299962007
ENSG00000138594	*TMOD3*	5.182834405	0.026257524	0.0595571	-0.033299576	0.999933887	0.068870712	0.217663753	-0.14879304	0.299995528
ENSG00000055130	*CUL1*	3.830311108	0.02273919	-0.139112488	0.161851678	0.999933887	0.030680607	0.16769195	-0.137011343	0.300006892
ENSG00000140332	*TLE3*	9.097672469	0.015379966	0.046507567	-0.031127601	0.999933887	0.147189455	0.26755112	-0.120361666	0.30003225
ENSG00000023734	*STRAP*	5.262559601	-0.054336182	-0.076073134	0.021736952	0.999933887	0.058424922	0.160133636	-0.101708714	0.30012988
ENSG00000179632	*MAF1*	5.783214925	0.029040148	0.028996944	4.3204e-05	0.999933887	-0.164792322	-0.289057482	0.12426516	0.30012988
ENSG00000165283	*STOML2*	2.979053949	0.008540299	-0.066458456	0.074998754	0.999933887	-0.04444009	-0.191434583	0.146994493	0.30012988
ENSG00000118690	*ARMC2*	1.866484617	-0.055636171	-0.146732867	0.091096696	0.999933887	0.039779109	0.284362122	-0.244583014	0.300337604
ENSG00000131368	*MRPS25*	2.667310285	-0.007776974	0.065278113	-0.073055088	0.999933887	-0.048313983	0.106574919	-0.154888901	0.300337604
ENSG00000284948	*AC107959.4*	2.016307841	0.127581012	-0.024971831	0.152552842	0.999933887	-0.8825226	-1.290849758	0.408327158	0.3007583
ENSG00000153283	*CD96*	5.06080328	-0.083018212	-0.06543164	-0.017586572	0.999933887	-0.025762509	0.108603324	-0.134365833	0.300833622
ENSG00000181754	*AMIGO1*	1.343459702	-0.002448984	0.064899762	-0.067348746	0.999933887	0.176498002	-0.054012584	0.230510586	0.300833622
ENSG00000149658	*YTHDF1*	4.749880778	0.008601413	-0.023382179	0.031983593	0.999933887	-0.186941316	-0.296651765	0.109710449	0.301078407
ENSG00000114346	*ECT2*	0.058503358	-0.055427287	-0.240691217	0.18526393	0.999933887	0.334783519	0.920440506	-0.585656986	0.301125479
ENSG00000231721	*LINC-PINT*	5.69203179	0.020039829	0.153307422	-0.133267593	0.999933887	-0.320567287	-0.476299901	0.155732615	0.301125479
ENSG00000173611	*SCAI*	0.968833549	-0.022519251	-0.042433338	0.019914087	0.999933887	-0.339819576	0.016947155	-0.35676673	0.301125479
ENSG00000261716	*H2BC20P*	3.465730539	-0.130420067	-0.046074839	-0.084345229	0.999933887	-0.626532998	-0.823058986	0.196525988	0.301204438
ENSG00000156983	*BRPF1*	4.62300381	0.125711765	0.044725114	0.080986651	0.999933887	-0.449976468	-0.589840028	0.13986356	0.301254379
ENSG00000261888	*AC144831.1*	2.421604573	0.058996512	-0.019743314	0.078739825	0.999933887	-0.042306028	-0.29918108	0.256875052	0.301254379
ENSG00000146463	*ZMYM4*	2.280050578	-0.062940906	-0.14155207	0.078611164	0.999933887	-0.131409437	0.086583378	-0.217992815	0.301254379
ENSG00000185104	*FAF1*	2.475020716	-0.05734582	-0.108178295	0.050832475	0.999933887	0.01091249	0.184793769	-0.173881278	0.301403313
ENSG00000134882	*UBAC2*	4.4724491	0.008469569	-0.044871609	0.053341179	0.999933887	-0.167816078	-0.279788791	0.111972713	0.301421984
ENSG00000124767	*GLO1*	2.636047469	0.011314875	-0.179635155	0.19095003	0.999933887	-0.163111814	0.033645595	-0.196757409	0.301421984
ENSG00000185624	*P4HB*	7.279233212	0.053625423	0.017084536	0.036540887	0.999933887	0.584336626	0.723183551	-0.138846925	0.301823432
ENSG00000070501	*POLB*	4.215158525	0.008303231	0.024989122	-0.016685891	0.999933887	-0.199754125	-0.341059962	0.141305837	0.301987303
ENSG00000114554	*PLXNA1*	2.817673411	0.014697828	0.136887551	-0.122189723	0.999933887	0.38664588	0.567494789	-0.180848909	0.302177819
ENSG00000136824	*SMC2*	0.230267956	-0.007963233	0.051307107	-0.05927034	0.999933887	-0.277897636	0.160698477	-0.438596113	0.3026933
ENSG00000248544	*AC008676.1*	1.488920181	0.169483817	0.03065897	0.138824847	0.999933887	-0.489405011	-0.19173256	-0.297672451	0.302765744
ENSG00000155719	*OTOA*	0.949656282	-0.195970468	-0.404536662	0.208566194	0.999933887	-0.092271454	0.128364666	-0.220636119	0.302765744
ENSG00000135506	*OS9*	7.745248323	-0.001060663	-0.004570646	0.003509983	0.999933887	-0.284338694	-0.402250444	0.117911749	0.302765744
ENSG00000163281	*GNPDA2*	0.862751859	-0.235207006	-0.381802055	0.146595049	0.999933887	-0.141786485	0.175985099	-0.317771585	0.30278964
ENSG00000237719	*Z95152.1*	2.369565852	0.002186799	-0.078689057	0.080875856	0.999933887	-0.293091622	0.017924559	-0.311016181	0.30278964
ENSG00000154781	*CCDC174*	4.958155293	-0.082808071	-0.05603709	-0.026770981	0.999933887	0.104553388	0.198773597	-0.094220209	0.303157862
ENSG00000155366	*RHOC*	2.664640401	0.077961697	-0.141364879	0.219326576	0.999933887	-0.005062135	-0.200099545	0.195037411	0.303169253
ENSG00000159267	*HLCS*	1.328601503	-0.0680263	-0.093499361	0.025473061	0.999933887	-0.091991036	0.165307893	-0.257298928	0.303169253
ENSG00000147138	*GPR174*	3.656452366	-0.067081076	-0.094625296	0.02754422	0.999933887	-0.03722257	0.203809676	-0.241032247	0.303169253
ENSG00000136827	*TOR1A*	5.199715362	-0.046582221	-0.058032873	0.011450652	0.999933887	-0.972416651	-1.149734535	0.177317884	0.303169253
ENSG00000172175	*MALT1*	4.259424399	-0.044982726	-0.076583523	0.031600797	0.999933887	-0.185739609	0.009767475	-0.195507084	0.303169253
ENSG00000133619	*KRBA1*	2.05108466	-0.014086076	0.114343916	-0.128429992	0.999933887	0.230873855	0.425147699	-0.194273844	0.303169253
ENSG00000248719	*AC021127.1*	1.639830074	0.009992719	0.180335377	-0.170342658	0.999933887	0.261300921	-0.024771649	0.28607257	0.303169253
ENSG00000241553	*ARPC4*	6.962898247	-0.007972868	0.028057703	-0.036030571	0.999933887	-0.084400679	-0.190546208	0.106145529	0.303502598
ENSG00000231105	*ECE1-AS1*	2.44046279	0.191832627	0.19818664	-0.006354012	0.999933887	2.143996589	2.453247232	-0.309250643	0.303623595
ENSG00000145741	*BTF3*	6.42232234	-0.022261136	-0.110093746	0.08783261	0.999933887	0.105405819	0.212868952	-0.107463133	0.303623595
ENSG00000189195	*BTBD8*	0.418132501	-0.12184242	-0.201072769	0.07923035	0.999933887	-0.745582149	-0.355370009	-0.390212141	0.303636099
ENSG00000163577	*EIF5A2*	2.043030431	0.051441825	-0.130509603	0.181951428	0.999933887	-0.333031936	-0.137870122	-0.195161814	0.303693079
ENSG00000151690	*MFSD6*	3.345641168	-0.079141203	-0.022174521	-0.056966682	0.999933887	0.031640344	-0.123660391	0.155300734	0.303730857
ENSG00000134313	*KIDINS220*	6.460977747	-0.017763628	-0.053964371	0.036200743	0.999933887	-0.353983278	-0.21189641	-0.142086867	0.303730857
ENSG00000107951	*MTPAP*	1.835857406	-0.108566369	-0.083856124	-0.024710245	0.999933887	0.076538973	0.27719555	-0.200656577	0.303847995
ENSG00000279570	*AC099804.1*	1.0171755	-0.038021138	0.237305992	-0.27532713	0.999933887	0.600041658	0.947199993	-0.347158334	0.303993955
ENSG00000164930	*FZD6*	-0.923144898	-0.023513161	0.138494031	-0.162007192	0.999933887	0.81041168	1.338273525	-0.527861846	0.30404276
ENSG00000237604	*AP001056.1*	-2.50570992	-0.215322332	0.318097544	-0.533419876	0.999933887	2.662073994	3.288861089	-0.626787095	0.304062752
ENSG00000134709	*HOOK1*	0.399916819	-0.37113301	-0.095941673	-0.275191337	0.999933887	-0.185140502	0.265366482	-0.450506985	0.304104117
ENSG00000104853	*CLPTM1*	5.62816866	0.027777817	0.007120467	0.020657349	0.999933887	-0.072013458	-0.177675042	0.105661584	0.304104117

ENSG00000204209	*DAXX*	4.697949522	-0.002742045	-0.042562139	0.039820094	0.999933887	0.017792327	-0.099261626	0.117053953	0.304186757
ENSG00000147475	*ERLIN2*	5.414451358	0.033892709	-0.036687576	0.070580286	0.999933887	-0.715038345	-0.890564773	0.175526427	0.304281984
ENSG00000164244	*PRRC1*	2.924306808	0.014088865	-0.115493942	0.129582807	0.999933887	-0.186865666	-0.035841482	-0.151024184	0.304281984
ENSG00000155380	*SLC16A1*	1.544718511	0.00545963	0.043314007	-0.037854378	0.999933887	0.16200753	0.440333703	-0.278326173	0.304284326
ENSG00000250995	*TMEM30BP1*	-0.150542743	0.106910236	-0.026372507	0.133282744	0.999933887	0.674344052	0.174507983	0.49983607	0.30469462
ENSG00000242474	*AC093627.5*	0.255814313	-0.16438442	0.027985415	-0.192369835	0.999933887	-0.562921454	-0.878862943	0.315941489	0.304827282
ENSG00000136141	*LRCH1*	2.898471307	-0.066615127	-0.023955498	-0.042659629	0.999933887	0.08349576	0.257555777	-0.174060017	0.304827282
ENSG00000183665	*TRMT12*	2.569667405	-0.068834688	-0.142715213	0.073880525	0.999933887	-0.287767849	-0.490007978	0.202240129	0.304827282
ENSG00000029639	*TFB1M*	0.388641905	-0.051025959	0.194806616	-0.245832575	0.999933887	-0.067911158	0.212781986	-0.280693145	0.304827282
ENSG00000167553	*TUBA1C*	2.8826497	0.016218907	-0.071802254	0.088021161	0.999933887	-0.034056537	-0.220375642	0.186319105	0.305414123
ENSG00000174891	*RSRC1*	1.343213048	0.133681178	-0.070972272	0.204653451	0.999933887	-0.062104079	0.256307764	-0.318411842	0.30560508
ENSG00000166348	*USP54*	0.246657566	-0.316541804	0.071165711	-0.387707515	0.999933887	0.083870636	-0.310359586	0.394230221	0.305642982
ENSG00000244720	*NT5C3AP2*	0.277659503	-0.291367652	-0.111765385	-0.179602267	0.999933887	-0.299807849	0.148177145	-0.447984994	0.30587961
ENSG00000114395	*CYB561D2*	3.128862285	0.017013272	-0.003361052	0.020374323	0.999933887	-0.23875225	-0.404293484	0.165541234	0.306084574
ENSG00000272994	*AC012360.3*	0.383634503	0.143905003	0.172592141	-0.028687138	0.999933887	0.619917479	0.239530326	0.380387153	0.306112733
ENSG00000151611	*MMAA*	0.121301338	-0.047238819	-0.319333991	0.272095171	0.999933887	-0.080931091	0.25272399	-0.333655081	0.306138201
ENSG00000251405	*AC016571.1*	0.629497176	0.035198189	0.056064115	-0.020865925	0.999933887	-0.566068374	-0.223742047	-0.342326328	0.306639006
ENSG00000130175	*PRKCSH*	6.803855555	0.042696068	0.014601903	0.028094166	0.999933887	-0.033159892	-0.148374781	0.11521489	0.306707273
ENSG00000163001	*CFAP36*	2.385927174	-0.038012108	-0.061566447	0.023554339	0.999933887	-0.004036765	0.188537685	-0.19257445	0.306782565
ENSG00000084652	*TXLNA*	4.208744391	0.000342918	0.043574284	-0.043231366	0.999933887	0.060089899	-0.096509034	0.156598934	0.306782565
ENSG00000143761	*ARF1*	9.120348572	0.018616286	0.005714634	0.012901652	0.999933887	0.15214363	0.242258091	-0.090114462	0.30694257
ENSG00000132522	*GPS2*	2.346244887	0.012038361	0.039760252	-0.027721891	0.999933887	0.259196033	0.053539477	0.205656556	0.30694257
ENSG00000177058	*SLC38A9*	1.162577422	0.116023814	-0.002623963	0.118647778	0.999933887	-0.207293676	0.0652812	-0.272574877	0.307239947
ENSG00000101193	*GID8*	5.099465444	-0.072853542	-0.108084115	0.035230573	0.999933887	-0.43965668	-0.567514534	0.127857854	0.307244598
ENSG00000258890	*CEP95*	4.318976597	-0.032165899	-0.092141448	0.059975549	0.999933887	-0.085571281	0.054164313	-0.139735594	0.307291753
ENSG00000123983	*ACSL3*	4.575405319	-0.028375607	-0.02828332	-9.2287e-05	0.999933887	-0.55178247	-0.707489509	0.155707038	0.307291753
ENSG00000121579	*NAA50*	6.66932333	-0.094764234	-0.11111627	0.016352036	0.999933887	-0.276291925	-0.147673206	-0.128618719	0.307338154
ENSG00000256223	*ZNF10*	1.107354247	-0.173441253	-0.141474898	-0.031966355	0.999933887	-0.300825058	0.137071514	-0.437896572	0.307338154
ENSG00000100442	*FKBP3*	1.436327078	0.056219846	-0.026678973	0.082898819	0.999933887	-0.077057405	0.199942365	-0.276999769	0.307338154
ENSG00000136828	*RALGPS1*	2.502081137	0.026924371	0.025005775	0.001918596	0.999933887	-0.145005309	-0.356511137	0.211505827	0.307520293
ENSG00000237788	*AL162615.1*	1.525050745	0.135904608	0.051860579	0.084044028	0.999933887	1.243656773	1.55513896	-0.311482187	0.307729237
ENSG00000131018	*SYNE1*	6.849130562	-0.062119919	-0.020504698	-0.041615221	0.999933887	-0.076394577	0.087649485	-0.164044062	0.307729237
ENSG00000115524	*SF3B1*	9.367109048	-0.070950952	-0.132099917	0.061148965	0.999933887	-0.184419587	0.00753021	-0.191949797	0.307729237
ENSG00000106991	*ENG*	1.887165334	0.100474724	-0.050099732	0.150574456	0.999933887	-0.679864449	-0.913183607	0.233319158	0.307729237
ENSG00000259330	*INAFM2*	7.005262551	0.039092015	0.06499629	-0.025904275	0.999933887	0.404367209	0.565980864	-0.161613655	0.307729237
ENSG00000115425	*PECR*	0.690019222	0.008616621	-0.102934355	0.111550977	0.999933887	-0.063449823	-0.424308218	0.360858395	0.307730705
ENSG00000213066	*CEP43*	2.711726721	-0.020236222	-0.06860776	0.048371538	0.999933887	-0.366891655	-0.566617774	0.199726119	0.307731986
ENSG00000231113	*AL035587.1*	1.860144449	-0.119817724	-0.077978041	-0.041839683	0.999933887	-0.482946624	-0.789796033	0.306849409	0.307846515
ENSG00000156787	*TBC1D31*	0.502694386	-0.114872877	-0.138481767	0.02360889	0.999933887	-0.252351881	0.096627673	-0.348979554	0.307846515
ENSG00000125676	*THOC2*	4.05955616	-0.080054958	-0.145613685	0.065558728	0.999933887	-0.377148774	-0.03939318	-0.337755594	0.308012884
ENSG00000198258	*UBL5*	5.860487104	0.009213462	-0.026272727	0.03548619	0.999933887	0.021844571	-0.084118484	0.105963055	0.308077185
ENSG00000077800	*FKBP6*	0.160763901	0.156149286	-0.070613198	0.226762484	0.999933887	0.511137331	0.85651822	-0.345380889	0.30827461
ENSG00000147164	*SNX12*	4.431241421	-0.019101139	-0.057074825	0.037973686	0.999933887	-0.545576949	-0.40656078	-0.139016169	0.308353459
ENSG00000135913	*USP37*	2.141462302	-0.007779718	-0.017126645	0.009346927	0.999933887	-0.202945655	0.037535917	-0.240481572	0.308380557
ENSG00000276916	*AL442125.2*	-0.175994355	-0.177177351	-0.566133532	0.388956182	0.999933887	-1.03877656	-0.689466603	-0.349309957	0.308392663
ENSG00000081154	*PCNP*	5.147363878	-0.026873288	-0.140946679	0.114073391	0.999933887	-0.142178554	-0.00484876	-0.137329793	0.308602856
ENSG00000159140	*SON*	8.548814551	-0.052370256	-0.064666733	0.012296477	0.999933887	-0.080596659	0.005068313	-0.085664972	0.308641111
ENSG00000159409	*CELF3*	-0.169553046	-0.042520246	0.226936213	-0.26945646	0.999933887	-0.364719656	-0.040815252	-0.323904404	0.308704764
ENSG00000174697	*LEP*	0.549093674	-0.039720429	-0.022448804	-0.017271625	0.999933887	-0.0501095	0.172943114	-0.223052615	0.308704764
ENSG00000246596	*AC139795.1*	-0.381623755	-0.019556784	0.20280406	-0.222360845	0.999933887	-0.540122324	-0.076533869	-0.463588456	0.308774759
ENSG00000116678	*LEPR*	0.82446939	0.031112634	-0.158605481	0.189718114	0.999933887	-0.353550137	-0.094224773	-0.259325364	0.309169204
ENSG00000058799	*YIPF1*	3.561553404	0.015084475	-0.109374344	0.124458819	0.999933887	-0.086758659	-0.295771208	0.209012549	0.309589548
ENSG00000170946	*DNAJC24*	0.812221738	-0.260890385	0.006866574	-0.267756959	0.999933887	-0.100106205	0.178668995	-0.2787752	0.309958105
ENSG00000163993	*S100P*	7.542199024	0.029882556	-0.003934728	0.033817284	0.999933887	0.426726283	0.579443451	-0.152717167	0.310245231
ENSG00000251259	*AC004069.1*	1.291162419	0.029934679	0.017837356	0.012097324	0.999933887	-0.067898194	0.278960125	-0.346858319	0.310444697
ENSG00000279159	*AC003681.1*	5.377343344	0.049923435	0.066182327	-0.016258893	0.999933887	0.05110481	0.213108479	-0.162003669	0.310511668
ENSG00000279530	*AC092881.1*	1.50695491	0.090278841	0.01335659	0.076922251	0.999933887	0.136221403	0.624464355	-0.488242952	0.310511668
ENSG00000073737	*DHRS9*	4.080613686	-0.085341624	-0.07554033	-0.009801295	0.999933887	-1.908485059	-2.173582649	0.265097591	0.310653568
ENSG00000115241	*PPM1G*	4.960694502	-0.030564513	0.022242068	-0.052806581	0.999933887	-0.259154495	-0.383507332	0.124352836	0.311177273
ENSG00000060069	*CTDP1*	6.083198514	0.008364537	0.019895572	-0.011531035	0.999933887	-0.354297758	-0.490131665	0.135833907	0.311193637
ENSG00000235173	*HGH1*	0.957240393	0.160817859	-0.160080712	0.320898571	0.999933887	0.208370604	-0.032167498	0.240538103	0.31154297
ENSG00000254760	*AC008750.1*	-0.455885203	0.017172301	-0.183682266	0.200854567	0.999933887	0.964833286	1.358536334	-0.393703049	0.311622642
ENSG00000177888	*ZBTB41*	1.228203412	-0.220399202	-0.208120415	-0.012278787	0.999933887	-0.288581143	0.144003475	-0.432584618	0.311777209
ENSG00000128298	*BAIAP2L2*	-0.66744515	0.218819177	0.111901112	0.106918065	0.999933887	0.32085029	0.747724815	-0.426874525	0.311798425
ENSG00000110107	*PRPF19*	3.672844477	0.027904033	-0.017166866	0.045070899	0.999933887	0.156641802	0.0168636	0.139778202	0.311798425
ENSG00000173273	*TNKS*	4.805486476	-0.019164668	-0.070383588	0.05121892	0.999933887	-0.07397056	0.057050994	-0.131021554	0.311798425
ENSG00000162676	*GFI1*	3.129846514	-0.012029652	-0.084939908	0.072910256	0.999933887	0.19944382	0.06069719	0.13874663	0.311866112
ENSG00000251301	*LINC02384*	1.91249618	0.012471212	0.013382112	-0.0009109	0.999933887	0.140768413	0.335174	-0.194405587	0.311866112
ENSG00000129518	*EAPP*	4.758368845	-0.058252944	-0.112849951	0.054597007	0.999933887	-0.38218241	-0.233002333	-0.149180077	0.311977901
ENSG00000232284	*GNG12-AS1*	0.097287676	0.089314473	-0.057029953	0.146344426	0.999933887	-1.246172204	-1.682232757	0.436060553	0.311980954
ENSG00000257557	*PPP1R12A-AS*	-1.044490286	0.212161123	0.038884932	0.173276191	0.999933887	1.850374317	2.292936287	-0.442561969	0.311980954
ENSG00000227372	*TP73-AS1*	2.09133804	0.051075118	-0.054447854	0.105522972	0.999933887	0.01366042	-0.180057806	0.193718227	0.311980954
ENSG00000144130	*NT5DC4*	2.228686899	-0.015983011	0.101809344	-0.117792355	0.999933887	-0.222798441	-0.391918029	0.169119588	0.311980954
ENSG00000100889	*PCK2*	2.439267763	0.003432607	-0.019629498	0.023062105	0.999933887	-0.349878408	-0.530308152	0.180429744	0.311980954
ENSG00000198945	*L3MBTL3*	2.814041948	-0.026471529	-0.136799382	0.110327854	0.999933887	-0.241547255	-0.453952667	0.212405412	0.311984172
ENSG00000170653	*ATF7*	2.57823457	0.090755162	0.022151102	0.06860406	0.999933887	-0.273931875	-0.455671284	0.181739409	0.312116754
ENSG00000196313	*POM121*	5.124712745	0.041426782	0.069549341	-0.028122559	0.999933887	0.303290806	0.436554788	-0.133263983	0.312239711
ENSG00000147459	*DOCK5*	9.048850269	-0.013255508	-0.034304568	0.02104906	0.999933887	-0.442351862	-0.563222896	0.120871034	0.312239711
ENSG00000160961	*ZNF333*	5.280986404	-0.037820542	-0.001182376	-0.036638166	0.999933887	-0.15867918	-0.05891901	-0.09976017	0.31224018
ENSG00000087088	*BAX*	6.205475265	0.020330051	0.07730465	-0.0569746	0.999933887	-0.092978165	-0.196106573	0.103128407	0.312484924
ENSG00000134910	*STT3A*	4.239798083	-0.019407734	-0.011978656	-0.007429078	0.999933887	0.085712913	0.205708312	-0.119995398	0.312771408
ENSG00000140521	*POLG*	5.829067756	0.030287131	0.068335887	-0.038048756	0.999933887	0.164412873	0.263149739	-0.098736866	0.312957702
ENSG00000101278	*RPS10P5*	0.39715961	-0.215706931	-0.080208832	-0.135498099	0.999933887	0.579938509	0.88491379	-0.304975281	0.313030214
ENSG00000186866	*POFUT2*	6.293069613	0.028584043	0.00488577	0.023698273	0.999933887	0.013771293	-0.092615669	0.106386962	0.313030214
ENSG00000222389	*RNU2-28P*	-1.801022039	0.078990957	0.137055685	-0.058064728	0.999933887	1.0849111	1.622689247	-0.537778148	0.313030214
ENSG00000126254	*RBM42*	5.493023705	0.050187707	0.023029346	0.027158361	0.999933887	0.231768717	0.357197841	-0.125429125	0.313191136
ENSG00000228486	*C2orf92*	0.169517226	0.018819047	0.296644002	-0.277824955	0.999933887	0.911209575	1.239587423	-0.328377848	0.313191136
ENSG00000138081	*FBXO11*	5.545937931	-0.068638721	-0.049204466	-0.019434255	0.999933887	-0.180034353	-0.06581612	-0.114218233	0.313360992
ENSG00000143324	*XPR1*	4.140348866	-0.043071937	-0.003968983	-0.039102954	0.999933887	-0.045547427	0.104029266	-0.149576693	0.313360992
ENSG00000068971	*PPP2R5B*	3.076161509	0.038178563	0.010866755	0.027311808	0.999933887	0.007298325	-0.189819223	0.197117548	0.313360992
ENSG00000204257	*HLA-DMA*	3.150404487	0.035190823	0.046506901	-0.011316078	0.999933887	-0.055606881	-0.265026754	0.209419873	0.313360992
ENSG00000106066	*CPVL*	0.349806768	-0.052075838	-0.081731165	0.029655327	0.999933887	0.051573954	-0.46554337	0.517117324	0.313493662
ENSG00000163312	*HELQ*	3.545291612	-0.045438225	-0.041783805	-0.003654419	0.999933887	0.106183909	0.246313376	-0.140129467	0.313707922
ENSG00000139946	*PELI2*	6.173464434	-0.093309815	-0.012167438	-0.081142377	0.999933887	-0.819190161	-0.974744104	0.155553943	0.314063353
ENSG00000151131	*C12orf45*	0.35521721	-0.185940782	0.073928512	-0.259869294	0.999933887	-0.294698681	0.031143752	-0.325842433	0.314063353
ENSG00000128203	*ASPHD2*	1.75775837	0.032288219	-0.10500746	0.137295679	0.999933887	-0.36111131	-0.630618754	0.269507443	0.314063353
ENSG00000135821	*GLUL*	12.329701529	0.058900992	0.053039173	0.005861819	0.999933887	-0.519119779	-0.390031047	-0.129088732	0.314073325
ENSG00000107789	*MINPP1*	0.021978669	-0.086095657	-0.063700525	-0.022395132	0.999933887	-0.01552326	-0.36941065	0.35388739	0.314073325
ENSG00000226419	*SLC16A1-AS1*	0.012850316	-0.145332867	-0.047264903	-0.098067964	0.999933887	-0.168445011	0.246679649	-0.41512466	0.314791627
ENSG00000140961	*OSGIN1*	5.232075232	0.098195029	0.00239486	0.095800169	0.999933887	0.372694657	0.546516899	-0.173822241	0.314922028
ENSG00000273899	*NOL12*	2.068090282	-0.092068779	-0.04660765	-0.04546113	0.999933887	-0.071855119	-0.248289535	0.176434416	0.314922028
ENSG00000100219	*XBP1*	5.202833331	0.014796002	0.073355211	-0.05855921	0.999933887	1.387549779	1.659090153	-0.271540373	0.315005272
ENSG00000127530	*OR7C1*	-0.533981898	-0.301493618	-0.035265211	-0.266228406	0.999933887	-0.85068449	-0.299265318	-0.551419172	0.315166309
ENSG00000174130	*TLR6*	5.960527456	-0.094863833	-0.108931511	0.014067678	0.999933887	-1.458260221	-1.687946372	0.229686151	0.315440179
ENSG00000246089	*AC016065.1*	1.18373491	0.010065256	0.470724452	-0.460659196	0.944094537	0.606788533	0.334986262	0.271802271	0.315513034

ENSG00000197614	*MFAP5*	-2.531273955	0.45167895	0.221284131	0.230394819	0.999933887	1.887870258	2.428091473	-0.540221216	0.315550802
ENSG00000256060	*TRAPPC2B*	0.893416259	0.006049363	0.223416138	-0.217366776	0.999933887	0.320668367	0.666825583	-0.346157216	0.315550802
ENSG00000130202	*NECTIN2*	2.994084766	-0.061957528	-0.059986924	-0.001970604	0.999933887	0.84209734	1.150666588	-0.308569248	0.315572544
ENSG00000102755	*FLT1*	3.84496603	0.035794004	-0.005257617	0.04105162	0.999933887	1.00263634	1.240115818	-0.237479478	0.315572544
ENSG00000287586	*Z98886.1*	-0.960807839	0.186893901	0.057483039	0.129410862	0.999933887	-0.162838987	-0.653093634	0.490254647	0.315779991
ENSG00000276842	*AC023510.2*	2.219098589	-0.071066552	-0.11089133	0.039824778	0.999933887	-0.685900778	-1.110559419	0.424658641	0.315831381
ENSG00000109321	*AREG*	2.480506121	0.140583578	0.097568925	0.043014653	0.999933887	-0.077572977	-0.398926085	0.321353108	0.316065705
ENSG00000185716	*MOSMO*	1.821184226	-0.13436881	-0.048338011	-0.086030799	0.999933887	-0.589104231	-0.395840068	-0.193264163	0.316065705
ENSG00000226091	*LINC00937*	1.776595121	0.071610952	-0.104175794	0.175786746	0.999933887	1.150675511	1.452156422	-0.301480912	0.316113324
ENSG00000076554	*TPD52*	0.495997839	-0.138438749	0.058109005	-0.196547754	0.999933887	-0.072588865	0.263135561	-0.335724426	0.316156598
ENSG00000142330	*CAPN10*	3.905131582	0.059719053	0.127216547	-0.067497494	0.999933887	-0.04630784	-0.186460559	0.14015272	0.316156598
ENSG00000183258	*DDX41*	5.32767766	-0.022562558	-0.021430116	-0.001132442	0.999933887	-0.27333029	-0.385069947	0.111739657	0.316156598
ENSG00000188878	*FBF1*	0.281006165	-0.056588277	0.135719533	-0.192307809	0.999933887	0.132930339	-0.167547152	0.300477492	0.316156598
ENSG00000177189	*RPS6KA3*	6.237008822	-0.028529832	-0.090209845	0.061680014	0.999933887	-0.008353852	0.168384985	-0.176738837	0.316156598
ENSG00000233913	*RPL10P9*	4.003630998	-0.020745083	0.060347072	-0.081092155	0.999933887	0.203795382	0.351983515	-0.148188133	0.316156598
ENSG00000113658	*SMAD5*	2.049347664	0.055134932	-0.012782724	0.067917656	0.999933887	-0.124489246	0.069846399	-0.194335645	0.316188355
ENSG00000102898	*NUTF2*	3.018569163	0.034643372	-0.009393905	0.044037277	0.999933887	-0.021520316	-0.186879406	0.165359091	0.316188355
ENSG00000215493	*AC007731.1*	-1.01702913	-0.217680745	0.255399649	-0.473080394	0.999933887	-0.037802604	-0.430021778	0.392219174	0.316205877
ENSG00000136840	*ST6GALNAC4*	1.543747113	0.025843821	-0.099638499	0.12548232	0.999933887	-0.125605599	-0.337455444	0.211849845	0.316205877
ENSG00000264757	*MIR3198-1*	0.585288127	-0.034837966	0.082991599	-0.117829565	0.999933887	1.573335993	2.003661236	-0.430325243	0.316205877
ENSG00000100147	*CCDC134*	1.469595584	0.018342968	0.003491228	0.01485174	0.999933887	0.078062831	0.382021014	-0.303958183	0.316205877
ENSG00000106462	*EZH2*	2.806555543	-0.013260537	-0.004498836	-0.008761701	0.999933887	0.6203832	0.861193953	-0.240810753	0.316205877
ENSG00000168282	*MGAT2*	-0.375244806	-0.07182599	0.205482843	-0.277308834	0.999933887	0.011802332	-0.478687347	0.490489679	0.316223992
ENSG00000268403	*AC132192.2*	1.175899228	0.164531675	0.034789917	0.129741758	0.999933887	0.665740968	0.388620784	0.277120184	0.31629929
ENSG00000090432	*MUL1*	4.846969661	-0.030194846	-0.021006194	-0.009188653	0.999933887	-0.129757213	-0.007891659	-0.121865553	0.316905311
ENSG00000029725	*RABEP1*	5.01386457	-0.101395431	-0.068730601	-0.03266483	0.999933887	-0.20168234	-0.073874651	-0.127807689	0.317017701
ENSG00000228686	*AL590723.1*	1.082165657	-0.082233108	0.053671223	-0.135904332	0.999933887	-0.633653112	-1.052117466	0.418464354	0.317017701
ENSG00000067840	*PDZD4*	3.271059213	0.023611264	-0.008278507	0.031889771	0.999933887	0.197919204	0.002026209	0.195892995	0.317017701
ENSG00000013588	*GPRC5A*	-1.199766287	-0.095125877	-0.100965553	0.005839676	0.999933887	-0.247351036	0.196055326	-0.443406362	0.317022265
ENSG00000254986	*DPP3*	2.283184117	0.062779734	0.017881474	0.04489826	0.999933887	-0.010172505	0.179040858	-0.189213363	0.317167363
ENSG00000223478	*AL441992.1*	0.061869542	0.106509323	0.081991927	0.024517395	0.999933887	0.048562306	-0.328648887	0.377211193	0.317205763
ENSG00000116786	*PLEKHM2*	8.438795292	0.024353874	0.030385795	-0.006031921	0.999933887	0.303357459	0.411067143	-0.107709684	0.317205763
ENSG00000181652	*ATG9B*	-0.436566434	0.020422538	-0.157635745	0.178058283	0.999933887	-0.449731487	-0.053887683	-0.395843804	0.317205763
ENSG00000134453	*RBM17*	4.827094474	-0.032012886	0.007990437	-0.040003322	0.999933887	0.324312468	0.448422987	-0.124110519	0.31743379
ENSG00000143630	*HCN3*	-0.568637064	-0.034746067	-0.189686482	0.154940415	0.999933887	-0.059180968	0.3499049	-0.409085868	0.317455784
ENSG00000163104	*SMARCAD1*	1.792722543	-0.008076391	-0.193758977	0.185682585	0.999933887	-0.267081228	0.031074552	-0.29815578	0.318093067
ENSG00000122678	*POLM*	4.750514404	0.030848354	0.021630205	0.009218149	0.999933887	-0.012728893	-0.125746662	0.113017769	0.318200868
ENSG00000185697	*MYBL1*	3.46860944	-0.053311245	-0.11802412	0.064712875	0.999933887	-0.204695077	0.072552349	-0.277247426	0.318229926
ENSG00000185897	*FFAR3*	0.341051862	0.1194629	0.057977947	0.061484953	0.999933887	0.691469715	1.124002958	-0.432533243	0.318241158
ENSG00000162377	*COA7*	1.621146103	0.016258048	-0.009906044	0.026164093	0.999933887	0.01029768	0.233375356	-0.223077676	0.318567601
ENSG00000078053	*AMPH*	1.185701051	-0.095465691	0.167230768	-0.26269646	0.999933887	1.081078891	1.367330001	-0.28625111	0.318759485
ENSG00000181744	*DIPK2A*	3.134058802	0.053559571	0.066168187	-0.012608616	0.999933887	-0.465203825	-0.646633402	0.181429577	0.318759485
ENSG00000020922	*MRE11*	3.06868973	0.013620547	-0.113834225	0.127454772	0.999933887	-0.291562846	-0.039414741	-0.252148106	0.318759485
ENSG00000132199	*ENOSF1*	3.030429243	-0.051813347	-0.032113288	-0.019700059	0.999933887	0.03469413	0.17848536	-0.14379123	0.319088173
ENSG00000145833	*DDX46*	4.619552216	-0.001304432	-0.183732935	0.182428502	0.999933887	-0.234369926	-0.414732036	0.180362109	0.319242184
ENSG00000167186	*COQ7*	2.942590032	0.097156195	0.032732589	0.064423606	0.999933887	0.332449473	0.140192465	0.192257008	0.319334151
ENSG00000159147	*DONSON*	1.768806839	-0.002530949	0.044229353	-0.046760301	0.999933887	-0.01497624	0.24205933	-0.25703557	0.319419905
ENSG00000229873	*OGFR-AS1*	0.840389528	0.321376234	0.257432666	0.063943568	0.999933887	0.455625398	0.716935766	-0.261310368	0.320068416
ENSG00000138639	*ARHGAP24*	1.393098365	0.035955263	-0.051120128	0.087075391	0.999933887	0.425022775	0.75428356	-0.329260784	0.320123854
ENSG00000015532	*XYLT2*	2.590780884	-0.00837841	-0.018593849	0.010215439	0.999933887	-0.016531027	-0.199414629	0.182883602	0.320190447
ENSG00000112667	*DNPH1*	1.721086764	0.129122016	0.067768123	0.061353894	0.999933887	0.386024019	0.589485062	-0.203461043	0.320316312
ENSG00000285280	*AL390957.1*	1.604308981	-0.053101294	0.15090771	-0.204009004	0.999933887	-0.236776589	-0.584737966	0.347961377	0.320316312
ENSG00000130304	*SLC27A1*	0.4730001	-0.025930818	0.318297865	-0.344228684	0.999933887	0.016347701	-0.297042553	0.313390254	0.320316312
ENSG00000137309	*HMGA1*	7.262404805	0.073506697	0.009481766	0.06402493	0.999933887	-0.027205561	0.086286892	-0.113492454	0.320341169
ENSG00000234684	*SDCBP2-AS1*	1.326569086	-0.084046231	0.014400322	-0.098446552	0.999933887	-0.114356611	-0.376091709	0.261735098	0.320341169
ENSG00000246250	*AC087521.2*	-0.129366152	-0.113149787	-0.28520043	0.172050643	0.999933887	0.896150817	1.50084013	-0.604689313	0.320341169
ENSG00000116691	*MIIP*	3.954969578	0.091326068	0.081044308	0.01028176	0.999933887	0.219905849	0.057769008	0.162136841	0.32057648
ENSG00000156876	*SASS6*	0.839134312	0.140934286	-0.13596087	0.276895157	0.999933887	-0.565825265	-0.152742349	-0.413082917	0.32057648
ENSG00000166046	*TCP11L2*	5.789160153	-0.078955695	-0.026303924	-0.052651771	0.999933887	-0.443475556	-0.552773626	0.10929807	0.321017956
ENSG00000114120	*SLC25A36*	4.322755349	-0.084216479	-0.14440987	0.060193391	0.999933887	-0.25477767	0.01718194	-0.27195961	0.321017956
ENSG00000070785	*EIF2B3*	0.171390491	-0.12198664	-0.008307807	-0.113678834	0.999933887	-0.079262903	0.278100509	-0.357363413	0.321017956
ENSG00000182899	*RPL35A*	6.935212694	-0.001225001	-0.033557039	0.032332038	0.999933887	0.102489924	0.223025486	-0.120535562	0.321017956
ENSG00000242244	*ATP5MC1P3*	0.470163226	0.4555511	0.476527276	-0.020976176	0.999933887	1.051778485	1.587393574	-0.535615089	0.321077014
ENSG00000166881	*NEMP1*	3.882219585	-0.076696533	-0.001131943	-0.07556459	0.999933887	-0.411944924	-0.291170247	-0.120774677	0.321130712
ENSG00000112299	*VNN1*	5.380943966	-0.07081812	-0.048397517	-0.022420602	0.999933887	-0.315531099	-0.139405645	-0.176125454	0.321130712
ENSG00000286122	*AC016074.2*	-0.017459404	-0.109587608	0.381740104	-0.491327712	0.999933887	-0.265336622	0.140380179	-0.405716801	0.321130712
ENSG00000176209	*SMIM19*	1.859241559	-0.023429706	-0.087900392	0.064470686	0.999933887	0.149770835	0.349056434	-0.199285599	0.321130712
ENSG00000159593	*NAE1*	2.368766339	-0.017094925	-0.079325701	0.062230775	0.999933887	-0.068028308	0.113242672	-0.181270981	0.32119649
ENSG00000125149	*C16orf70*	3.405066141	-0.06126666	-0.076792207	0.015525547	0.999933887	-0.798704699	-0.983945101	0.185240402	0.321339327
ENSG00000143819	*EPHX1*	2.279781481	0.066233584	0.092761759	-0.026528175	0.999933887	0.035547487	-0.149435568	0.184983054	0.321469242
ENSG00000185278	*ZBTB37*	5.415973502	-0.004626042	-0.108804772	0.104178729	0.999933887	-0.59034781	-0.329929101	-0.260418709	0.321469242
ENSG00000168538	*TRAPPC11*	3.61984693	-0.062688083	-0.080385383	0.0176973	0.999933887	-0.17201934	-0.331714815	0.159695475	0.321528884
ENSG00000109390	*NDUFC1*	2.213288745	-0.023647757	-0.101710953	0.078063196	0.999933887	-0.052099703	-0.244041045	0.191941342	0.321528884
ENSG00000213316	*LTC4S*	1.419442019	0.02177887	0.078670034	-0.056891164	0.999933887	-0.080964803	-0.312980385	0.232015582	0.321528884
ENSG00000159247	*TUBBP5*	0.118858898	0.000132537	0.159597614	-0.159465077	0.999933887	-1.292194742	-1.619664338	0.327469596	0.321528884
ENSG00000273253	*AL022328.4*	2.40222602	0.104517987	-0.017406863	0.12192485	0.999933887	-0.296449906	-0.486129607	0.189679701	0.32231521
ENSG00000118242	*MREG*	0.289280248	-0.136918151	-0.075156315	-0.061761836	0.999933887	0.455105436	0.7759927	-0.320887264	0.322660399
ENSG00000112062	*MAPK14*	6.818665062	-0.015573465	-0.024910652	0.009337186	0.999933887	-0.33401568	-0.453858035	0.119842355	0.323279582
ENSG00000164715	*LMTK2*	6.044285205	-0.059252457	-0.038755345	-0.020497112	0.999933887	-0.339017286	-0.445745814	0.106728528	0.323289144
ENSG00000213373	*LINC00671*	0.278954297	0.024520681	0.089822328	-0.065301648	0.999933887	-0.463362217	-0.948005734	0.484643518	0.323289144
ENSG00000049618	*ARID1B*	5.260100077	0.058776733	0.015349255	0.043427478	0.999933887	-0.011007536	-0.115919144	0.104911608	0.323350149
ENSG00000101361	*NOP56*	4.50838189	-0.013379461	0.033550061	-0.046929522	0.999933887	0.189024204	0.064644829	0.124379375	0.323378096
ENSG00000119397	*CNTRL*	4.564812054	-0.090919348	-0.125395311	0.034475963	0.999933887	-0.362324383	-0.048541817	-0.313782566	0.323399599
ENSG00000125870	*SNRPB2*	3.60990147	-0.065664488	-0.074948158	0.00928367	0.999933887	0.132437708	0.26052358	-0.128085872	0.323616652
ENSG00000107130	*NCS1*	-0.643266119	0.054520817	-0.459026848	0.513547666	0.999933887	-0.152879574	-0.594729397	0.441849823	0.323616652
ENSG00000212176	*RNA5SP207*	1.260514966	0.14373361	0.213218028	-0.069484418	0.999933887	0.48121928	0.860112148	-0.378892868	0.323849544
ENSG00000103024	*NME3*	2.626580002	0.145168604	0.002862344	0.14230626	0.999933887	0.25507087	0.039427432	0.215643438	0.324028357
ENSG00000286974	*AL662884.5*	1.327300235	0.086624008	0.091935841	-0.005311833	0.999933887	-0.103233991	-0.441389868	0.338155877	0.324302843
ENSG00000109906	*ZBTB16*	1.338424449	0.071266721	0.085791277	-0.014524556	0.999933887	-0.386909012	-0.661900496	0.274991484	0.324302843
ENSG00000064547	*LPAR2*	6.749247419	0.046358728	0.039403555	0.006955173	0.999933887	-0.152834915	-0.253683218	0.100848303	0.324345989
ENSG00000073921	*PICALM*	8.605419223	0.014372038	0.00784498	0.006527059	0.999933887	-0.30172911	-0.171840975	-0.129888135	0.324569808
ENSG00000091732	*ZC3HC1*	1.787541095	0.00379838	-0.287153927	0.290952307	0.999933887	0.145893254	0.31712001	-0.171226756	0.324569808
ENSG00000171823	*FBXL14*	2.947175781	0.184355371	-0.005353793	0.189709164	0.999933887	0.174187415	0.417299073	-0.243111658	0.324588255
ENSG00000160299	*PCNT*	4.393851639	-0.041843184	0.022493416	-0.0643366	0.999933887	0.056292925	0.182813605	-0.12652068	0.324683501
ENSG00000160999	*SH2B2*	6.644102117	0.030503595	-0.026548655	0.057052251	0.999933887	0.654474805	0.795853208	-0.141378403	0.324683501
ENSG00000288596	*C8orf44*	0.235659949	-0.049960867	0.11437881	-0.164339677	0.999933887	-0.46907504	-0.147548558	-0.321526482	0.324683501
ENSG00000131148	*EMC8*	2.669937797	-0.0146378	0.059383269	-0.074021069	0.999933887	-0.136265575	-0.299205231	0.162939656	0.324890582
ENSG00000130818	*ZNF426*	2.540393902	-0.005852177	0.032233356	-0.038085534	0.999933887	0.053474779	0.237011328	-0.183536549	0.325242621
ENSG00000163492	*CCDC141*	-0.384386241	-0.008287615	0.453673123	-0.461960738	0.999933887	-0.388385177	0.174302922	-0.562688099	0.325560066
ENSG00000167657	*DAPK3*	5.172863057	0.060644981	0.070165712	-0.009520731	0.999933887	-0.292153939	-0.461663693	0.169509754	0.325586722
ENSG00000023902	*PLEKHO1*	6.470145705	0.000795257	0.023018015	-0.022222758	0.999933887	-0.079508916	-0.262867184	0.183358268	0.325586722
ENSG00000100592	*DAAM1*	1.342170834	-0.126605285	-0.007154391	-0.119450894	0.999933887	-0.241241215	0.027528383	-0.268769598	0.325625014
ENSG00000117602	*RCAN3*	3.667111219	-0.012436933	0.082682704	-0.095119638	0.999933887	0.086492359	-0.030503195	0.116995554	0.325656682
ENSG00000272086	*AC025181.2*	-0.322528396	-0.067771031	-0.302816786	0.235045756	0.999933887	-0.288685012	-0.760684959	0.471999947	0.325810121
ENSG00000220378	*KRT8P42*	3.375161882	0.167662648	0.092347637	0.075315011	0.999933887	1.164363045	1.431888635	-0.267525591	0.326000169

ENSG00000086666	*ZFAND6*	6.414275374	-0.068349342	-0.076903733	0.008554391	0.999933887	-0.207869145	-0.109585552	-0.098283593	0.326000169
ENSG00000215252	*GOLGA8B*	3.944716283	-0.05476358	-0.047618727	-0.007144853	0.999933887	-0.045585388	0.112092763	-0.157678151	0.326000169
ENSG00000177051	*FBXO46*	4.531706561	0.03122424	0.047864455	-0.016640215	0.999933887	0.357777288	0.474956609	-0.117179321	0.326000169
ENSG00000272768	*AC004854.2*	1.040485724	0.18533914	0.294285609	-0.108946469	0.999933887	1.327933442	0.934200372	0.393733069	0.326008705
ENSG00000233214	*AC002511.2*	0.743522741	-0.122462289	-0.00879024	-0.113672049	0.999933887	2.10967082	2.508495557	-0.398824737	0.326151457
ENSG00000125037	*EMC3*	5.694896567	-0.040184979	-0.031651144	-0.008533835	0.999933887	-0.616049974	-0.46610908	-0.149940894	0.326151457
ENSG00000213995	*NAXD*	3.096913304	-0.001705899	0.038309586	-0.040015485	0.999933887	0.318743723	0.475999267	-0.157255544	0.326151457
ENSG00000174749	*FAM241A*	1.969902787	0.00300896	0.1360456	-0.13303664	0.999933887	0.005659441	0.209552273	-0.203892832	0.326153621
ENSG00000147955	*SIGMAR1*	2.58637279	0.097993904	-0.038991691	0.136985595	0.999933887	0.010431197	-0.182348453	0.19277965	0.326196313
ENSG00000106635	*BCL7B*	5.534462122	0.046343345	0.028735691	0.017607654	0.999933887	0.356331373	0.475631016	-0.119299643	0.326201209
ENSG00000101464	*PIGU*	1.349514124	-0.018914786	0.10268128	-0.121596066	0.999933887	0.258425294	0.498058183	-0.23963289	0.326330433
ENSG00000159459	*UBR1*	4.134332112	0.031196755	-0.109043461	0.140240217	0.999933887	-0.023350584	0.174975525	-0.198326109	0.32635824
ENSG00000175054	*ATR*	3.331212195	-0.035160875	-0.07365392	0.038493045	0.999933887	-0.215144755	0.091113523	-0.306258279	0.32635971
ENSG00000197937	*ZNF347*	0.150419608	-0.197340626	0.052756447	-0.250097073	0.999933887	-0.231885199	-0.532409124	0.300523925	0.326360802
ENSG00000243716	*NPIPB5*	3.508951875	-0.006643569	0.004011819	-0.010655388	0.999933887	0.091697265	0.254358092	-0.162660827	0.326360802
ENSG00000205786	*LINC01531*	-0.067205137	-0.081604369	-0.112978526	0.031374157	0.999933887	1.247896303	1.821657267	-0.573760963	0.326595396
ENSG00000086544	*ITPKC*	4.668808193	0.067281508	0.024142737	0.043138771	0.999933887	0.766145088	0.903458288	-0.1373132	0.326662086
ENSG00000165792	*METTL17*	2.590535626	0.039765608	0.059287159	-0.019521552	0.999933887	0.119038116	-0.062732516	0.181770633	0.326662086
ENSG00000129255	*MPDU1*	3.529494834	0.107218533	0.124006863	-0.01678833	0.999933887	0.495785235	0.678907598	-0.183122363	0.327082789
ENSG00000134874	*DZIP1*	-0.332857878	-0.1358559	0.088989453	-0.224845353	0.999933887	0.964715794	1.196949433	-0.232233638	0.327096352
ENSG00000244134	*RPS12P20*	1.723726924	-0.011608084	-0.14692705	0.135318967	0.999933887	0.027290575	0.326376496	-0.299085921	0.327112185
ENSG00000267523	*AC008735.2*	1.112379431	0.162400266	-0.018501371	0.180901638	0.999933887	-0.4073164	-0.654632514	0.247316114	0.327442707
ENSG00000162496	*DHRS3*	1.848360274	-0.098488813	0.034948181	-0.133436994	0.999933887	0.264434256	0.455420465	-0.190986209	0.327480333
ENSG00000180035	*ZNF48*	0.40379677	0.123316454	0.046182901	0.077133553	0.999933887	0.172388935	-0.101309083	0.273698018	0.327480333
ENSG00000122877	*EGR2*	7.617477208	0.288349865	0.26118325	0.027166615	0.999933887	0.321429183	0.495920018	-0.174490835	0.327527987
ENSG00000250116	*RHOQ-AS1*	2.556392339	0.008662117	0.083045402	-0.074383284	0.999933887	0.377409378	0.610924993	-0.233515614	0.327527987
ENSG00000125447	*GGA3*	6.051841058	-0.03649329	0.05461188	-0.09110517	0.999933887	-0.197116958	-0.277568917	0.080451959	0.327812421
ENSG00000111647	*UHRF1BP1L*	5.737186018	-0.040256812	-0.113974512	0.073717699	0.999933887	-0.595757007	-0.402471853	-0.193285154	0.327827494
ENSG00000167792	*NDUFV1*	4.51733889	0.025414527	0.00407619	0.021338337	0.999933887	0.147722195	0.002642638	0.145079558	0.327964052
ENSG00000166676	*TVP23A*	-0.571712128	0.09806023	-0.009164373	0.107224603	0.999933887	-0.212667776	0.225859781	-0.438527558	0.327969975
ENSG00000120896	*SORBS3*	2.356590021	0.004976837	0.085385574	-0.080408737	0.999933887	0.099622034	0.274296483	-0.174674449	0.327969975
ENSG00000142207	*URB1*	2.47969446	0.047717991	-0.002524416	0.050242407	0.999933887	0.227987686	0.406536366	-0.17854868	0.328052327
ENSG00000160695	*VPS11*	5.329431364	0.004196278	-0.043647619	0.047843897	0.999933887	-0.009989634	-0.127837668	0.117848034	0.328060635
ENSG00000075391	*RASAL2*	-0.73540456	0.035757013	0.219182891	-0.183425878	0.999933887	-0.919816443	-0.492871254	-0.426945189	0.32815189
ENSG00000169519	*METTL15*	0.288703078	-0.024683504	0.172786751	-0.197470255	0.999933887	-0.246248007	0.211856197	-0.458104204	0.328226075
ENSG00000170100	*ZNF778*	2.169938079	-0.194124927	0.075404454	-0.269529382	0.999933887	-0.119036579	-0.299523598	0.180487019	0.328356945
ENSG00000272444	*AL118558.4*	-0.6967068	-0.042721108	-0.119233267	0.076512159	0.999933887	0.229658779	0.607618898	-0.377960119	0.328402506
ENSG00000171791	*BCL2*	4.345427458	0.035730265	-0.027656166	0.063386431	0.999933887	0.187540027	0.314686391	-0.127146364	0.328491483
ENSG00000179476	*C14orf28*	1.315935026	-0.024826754	-0.161930799	0.137104045	0.999933887	-0.392447136	-0.646701311	0.254254175	0.328566694
ENSG00000160185	*UBASH3A*	3.066755244	-0.06408002	0.101775642	-0.165855662	0.999933887	0.351413885	0.517039385	-0.165625501	0.328936628
ENSG00000118518	*RNF146*	4.912574883	-0.068571327	-0.096843846	0.028272519	0.999933887	-0.29573261	-0.166422906	-0.129309704	0.32928822
ENSG00000179918	*SEPHS2*	5.481982601	0.031398626	-0.102784306	0.134182932	0.999933887	1.12987129	0.881525022	0.248346269	0.329307816
ENSG00000135625	*EGR4*	-0.422727745	0.205971308	0.130865547	0.07510576	0.999933887	1.050657597	1.6491997	-0.598542103	0.329358883
ENSG00000283286	*AC093277.1*	-0.622042305	-0.137520277	-0.34281803	0.205297753	0.999933887	1.669480342	2.079803985	-0.410323643	0.329358883
ENSG00000138430	*OLA1*	2.322737305	-0.002692989	-0.24095434	0.238261351	0.999933887	-0.064431161	0.141331719	-0.20576288	0.329358883
ENSG00000136830	*NIBAN2*	2.08262265	0.005413675	-0.073880086	0.079293761	0.999933887	-0.770063284	-0.991061394	0.22099811	0.329401125
ENSG00000179840	*PIK3CD-AS1*	1.045120834	-0.005686569	0.14286734	-0.148553909	0.999933887	0.946774566	1.336497164	-0.389722598	0.329498164
ENSG00000265205	*AC010761.3*	1.143839332	-0.012705358	-0.095004283	0.082298925	0.999933887	0.044149481	0.441774299	-0.397624818	0.329515158
ENSG00000165416	*SUGT1*	2.377911991	-0.110443093	0.068074105	-0.178517198	0.999933887	-0.045229392	0.163958127	-0.209187519	0.329524782
ENSG00000172534	*HCFC1*	5.516458494	0.031615106	0.152813759	-0.121198652	0.999933887	0.225191098	0.331871736	-0.106680637	0.32953575
ENSG00000163798	*SLC4A1AP*	3.300643928	0.007666096	0.0404819	-0.032815803	0.999933887	-0.094729208	0.059152222	-0.15388143	0.32953575
ENSG00000163635	*ATXN7*	6.401520444	0.043113072	0.010861063	0.032252009	0.999933887	-0.249041605	-0.393464001	0.144422396	0.329689054
ENSG00000165476	*REEP3*	2.558756503	0.025615913	-0.005225485	0.030841398	0.999933887	-0.587622434	-0.823708556	0.236086123	0.329945955
ENSG00000115145	*STAM2*	4.045496155	-0.013983925	0.017813059	-0.031796984	0.999933887	-0.581931391	-0.41945601	-0.162475381	0.329965408
ENSG00000163453	*IGFBP7*	-0.065372688	-0.00106157	0.182341326	-0.183402896	0.999933887	0.096055705	-0.343904907	0.439960612	0.329965408
ENSG00000288093	*AL592295.5*	-0.232262627	-0.144421535	0.19935466	-0.343776195	0.999933887	0.094806526	0.639207413	-0.544400886	0.330059208
ENSG00000139116	*KIF21A*	1.916517712	-0.11802535	-0.099310397	-0.018714953	0.999933887	-0.102221509	0.190446059	-0.292667568	0.330378616
ENSG00000256294	*ZNF225*	-0.25576226	-0.010681114	0.12277857	-0.133459685	0.999933887	0.055215208	-0.249887311	0.305102519	0.330558523
ENSG00000164053	*ATRIP*	2.161632629	0.038801222	0.042014948	-0.003213726	0.999933887	0.043982086	-0.150854442	0.194836529	0.330770747
ENSG00000171735	*CAMTA1*	2.740539043	-0.045277409	0.131424861	-0.17670227	0.999933887	0.168549155	0.334537377	-0.165988222	0.331003492
ENSG00000235919	*ASH1L-AS1*	1.232310373	-0.022832763	-0.009513327	-0.013319436	0.999933887	0.834915472	1.056114039	-0.221198567	0.331003492
ENSG00000106682	*EIF4H*	7.406738012	0.008904878	0.017272234	-0.008367356	0.999933887	0.077973868	0.170436606	-0.092462737	0.331114758
ENSG00000232671	*ZNF687-AS1*	0.540368078	0.063102256	0.01326526	0.049836996	0.999933887	0.942393321	0.598613233	0.343780088	0.331321536
ENSG00000116586	*LAMTOR2*	2.657155404	0.050732987	0.034308173	0.016424815	0.999933887	0.006041911	-0.230290648	0.236332559	0.331389868
ENSG00000197013	*ZNF429*	5.639091939	0.044182741	0.008019518	0.036163223	0.999933887	0.410808898	0.635260253	-0.224451355	0.331389868
ENSG00000148110	*MFSD14B*	4.553773774	-0.007295718	-0.007411172	0.000115454	0.999933887	-0.2696697	-0.402803001	0.133133301	0.331742375
ENSG00000232434	*AJM1*	1.665258457	0.176687805	0.142843111	0.033844694	0.999933887	0.130245807	0.385311885	-0.255066078	0.33183483
ENSG00000162063	*CCNF*	0.206870723	-0.080432794	-0.114249676	0.033816882	0.999933887	0.309800826	0.602187961	-0.292387135	0.33183483
ENSG00000119541	*VPS4B*	7.157127537	-0.032459164	-0.025341047	-0.007118117	0.999933887	-0.03392053	0.07263134	-0.10655187	0.331938995
ENSG00000254634	*SMG1P6*	-0.701746982	-0.217820057	0.022746364	-0.240566422	0.999933887	-0.122034599	0.266548581	-0.388583181	0.331940938
ENSG00000198453	*ZNF568*	0.250893502	-0.02714334	-0.072249838	0.045106498	0.999933887	-0.03522239	0.297546683	-0.332769072	0.331940938
ENSG00000126264	*HCST*	2.842524596	0.130523928	0.153497007	-0.022973079	0.999933887	0.69012643	0.887795673	-0.197669242	0.331951559
ENSG00000230492	*AL049651.1*	-0.53722594	-0.058694153	-0.004823895	-0.053870258	0.999933887	-0.066261598	0.456847718	-0.523109316	0.332111088
ENSG00000131467	*PSME3*	6.62156325	0.015672853	0.004927203	0.01074565	0.999933887	0.512930756	0.34677779	0.166152966	0.332646496
ENSG00000007516	*BAIAP3*	4.666910992	0.058838732	0.068744042	-0.00990531	0.999933887	0.10608473	-0.033097684	0.139182414	0.333357518
ENSG00000182463	*TSHZ2*	1.152566889	-0.098726289	0.028042104	-0.126768392	0.999933887	-0.1623195	0.108050729	-0.270370229	0.333494241
ENSG00000143179	*UCK2*	-0.290540822	-0.009386875	0.028674811	-0.038061686	0.999933887	-0.021340494	0.388826903	-0.410167397	0.333494241
ENSG00000181751	*MACIR*	0.695575895	-0.193356008	-0.068878698	-0.12447731	0.999933887	-0.48146526	-0.762157141	0.28069188	0.333675355
ENSG00000138399	*FASTKD1*	0.878042952	-0.141322251	-0.156598548	0.015276297	0.999933887	-0.062696919	0.175043215	-0.237740134	0.333823078
ENSG00000163956	*LRPAP1*	6.701964154	0.025720597	0.020369936	0.005350661	0.999933887	-0.209738741	-0.341684429	0.131945688	0.333823442
ENSG00000116212	*LRRC42*	2.225469	-0.001619942	0.06186898	-0.063488922	0.999933887	-0.504422595	-0.732509567	0.228086972	0.33399772
ENSG00000163877	*SNIP1*	5.410829119	0.02013787	-0.000294599	0.020432468	0.999933887	0.208367173	0.309531822	-0.101164649	0.334069453
ENSG00000110318	*CEP126*	-0.834772158	-0.228238464	0.04080991	-0.269048374	0.999933887	-0.414992292	0.129844869	-0.544837161	0.334097617
ENSG00000114480	*GBE1*	6.611067153	0.155799498	0.236605344	-0.080805846	0.999933887	1.555062256	1.770500338	-0.215438082	0.334107454
ENSG00000260852	*FBXL19-AS1*	0.176039928	0.004049564	-0.012765259	0.016814823	0.999933887	-0.253176058	0.045368136	-0.298544194	0.334144004
ENSG00000164120	*HPGD*	1.26208231	-0.144579932	-0.090935145	-0.053644787	0.999933887	-0.279394879	-0.044528464	-0.234866415	0.334150831
ENSG00000185129	*PURA*	3.06568979	0.065789138	-0.039467421	0.105256559	0.999933887	-0.270893473	-0.450184656	0.179291183	0.334150831
ENSG00000269843	*AC008537.2*	0.513423803	-0.052675884	-0.351930461	0.299254578	0.999933887	-0.151017048	0.150671819	-0.301688867	0.334456217
ENSG00000197134	*ZNF257*	-1.029678156	0.110970157	0.185732551	-0.074762394	0.999933887	-0.27245064	0.223322414	-0.495773054	0.334480637
ENSG00000239351	*NPM1P29*	1.146700382	-0.152802979	-0.129925662	-0.022877317	0.999933887	-0.14485623	0.20004902	-0.34490525	0.33454561
ENSG00000058063	*ATP11B*	7.164121083	-0.042404632	-0.094060944	0.051656312	0.999933887	-0.557538707	-0.364688151	-0.192850557	0.33454561
ENSG00000267293	*AC012569.1*	0.821624879	-0.065139256	0.019131682	-0.084270938	0.999933887	1.872836816	2.353302908	-0.480466092	0.33454561
ENSG00000265148	*TSPOAP1-AS1*	2.540770673	0.159361398	0.049247114	0.110114284	0.999933887	0.225070028	0.027137973	0.197932056	0.334616545
ENSG00000167881	*SRP68*	3.829878648	-0.017396799	-0.109916217	0.092519418	0.999933887	0.077505346	0.201278921	-0.123773575	0.334796045
ENSG00000116688	*MFN2*	6.541925563	-0.014600026	0.059632409	-0.074232436	0.999933887	-0.039242076	0.064915739	-0.104157816	0.335108565
ENSG00000231346	*LINC01160*	-0.208747872	-0.042476034	-0.066066108	0.023590073	0.999933887	0.112600427	-0.222902291	0.335502719	0.33517383
ENSG00000148019	*CEP78*	3.093383579	-0.053109807	-0.070913775	0.017803967	0.999933887	-0.207825899	0.029888808	-0.237714707	0.335206062
ENSG00000145495	*MARCHF6*	7.992892481	-0.047627554	-0.027007515	-0.020620039	0.999933887	0.112445996	0.197621705	-0.085175709	0.335323566
ENSG00000172301	*COPRS*	1.622790807	0.232347266	0.130884115	0.101463152	0.999933887	0.008499927	-0.203125499	0.211625426	0.335565302
ENSG00000116824	*CD2*	4.766589055	-0.021516244	-0.106638964	0.08512272	0.999933887	0.16161441	0.288649054	-0.127034644	0.335565302
ENSG00000087460	*GNAS*	8.745986019	0.015346163	-0.00490456	0.020250723	0.999933887	-0.181803349	-0.283847693	0.102044343	0.335565302
ENSG00000145439	*CBR4*	1.850625501	-0.075898981	-0.125814849	0.049915869	0.999933887	-0.571725738	-0.821913703	0.250187965	0.335940346
ENSG00000274270	*AL137060.3*	1.051810949	0.176014928	0.130837993	0.045176935	0.999933887	0.88388121	1.165171149	-0.281289938	0.336111577
ENSG00000261098	*AP000766.1*	-0.439203465	0.221042635	0.33190195	-0.110859315	0.999933887	-0.311969001	0.267625003	-0.579594004	0.336111577
ENSG00000172172	*MRPL13*	0.517810536	-0.147635917	-0.518169642	0.370533725	0.999933887	0.072833434	0.347755632	-0.274922198	0.336116428

ENSG00000137145	*DENND4C*	3.992415389	-0.046243294	-0.000902677	-0.045340617	0.999933887	-0.305167344	-0.108073631	-0.197093713	0.336218604
ENSG00000124217	*MOCS3*	1.351067341	-0.125265921	-0.145870379	0.020604457	0.999933887	-0.127016951	-0.346073905	0.219056954	0.336306623
ENSG00000070367	*EXOC5*	3.753649425	0.035428455	-0.19777316	0.233201616	0.999933887	-0.72056318	-0.45616271	-0.26440047	0.336332624
ENSG00000154727	*GABPA*	3.610443625	-0.083678824	-0.123204146	0.039525322	0.999933887	-0.607039453	-0.356208632	-0.250830822	0.336572944
ENSG00000065600	*PACC1*	0.983835811	-0.049067862	-0.074286497	0.025218635	0.999933887	-0.197668611	0.03304707	-0.230715682	0.336605497
ENSG00000113552	*GNPDA1*	7.859820952	-0.056766586	-0.014207251	-0.042559336	0.999933887	0.177491571	0.304608209	-0.127116638	0.336613287
ENSG00000235092	*ID2-AS1*	-0.334901709	-0.130011504	-0.286789116	0.156777611	0.999933887	0.279825047	0.656044094	-0.376219046	0.336613287
ENSG00000111674	*ENO2*	3.421895049	-0.030051541	-0.139063734	0.109012193	0.999933887	0.159157564	0.003136394	0.156021169	0.336613287
ENSG00000253669	*GASAL1*	1.38173175	0.288892857	0.346664076	-0.05777122	0.999933887	1.347924838	1.653480688	-0.30555585	0.336677763
ENSG00000172115	*CYCS*	4.294115147	-0.028971137	-0.047313716	0.018342579	0.999933887	0.352310048	0.506063299	-0.153753251	0.336998633
ENSG00000270108	*AL049840.6*	0.405379445	-0.182205196	0.054762874	-0.236968071	0.999933887	-0.191741468	-0.555457767	0.3637163	0.337448109
ENSG00000127452	*FBXL12*	4.221584186	-0.023233148	-0.065033763	0.041800615	0.999933887	-0.29584045	-0.422126244	0.126285794	0.337599203
ENSG00000189077	*TMEM120A*	5.597779666	0.065181358	0.020607748	0.04457361	0.999933887	0.505806895	0.683225275	-0.17741838	0.337667766
ENSG00000246790	*AP000977.1*	0.021576329	-0.084597925	-0.377501764	0.292903839	0.999933887	-0.023526768	-0.40727344	0.383746672	0.337667766
ENSG00000092010	*PSME1*	7.443098474	-0.025257916	-0.094225464	0.068967549	0.999933887	0.045011491	-0.055443008	0.100454499	0.337667766
ENSG00000272888	*CHASERR*	6.909805833	-0.039266925	-0.018982219	-0.020284706	0.999933887	0.311110978	0.412748716	-0.101637738	0.337894538
ENSG00000255587	*RAB44*	4.155131005	-0.052030984	0.07986985	-0.131900834	0.999933887	0.032986562	0.18282858	-0.149842019	0.337894538
ENSG00000106346	*USP42*	4.606552461	0.079856055	0.109336663	-0.029480608	0.999933887	-0.100755762	-0.2398888	0.139133038	0.338054044
ENSG00000152207	*CYSLTR2*	2.178767249	-0.034688354	-0.087998788	0.053310434	0.999933887	-0.072427942	0.137069687	-0.209497629	0.338070391
ENSG00000170860	*LSM3*	3.385833706	0.033705232	-0.060903133	0.094608365	0.999933887	0.045512497	0.221947261	-0.176434765	0.338070391
ENSG00000105677	*TMEM147*	2.081580558	0.012247792	0.114734591	-0.102486799	0.999933887	0.099411489	0.281296859	-0.18188537	0.338070391
ENSG00000211776	*TRAV2*	-0.060856339	0.096579334	0.175265582	-0.078686247	0.999933887	0.004382928	-0.404904928	0.409287857	0.338111537
ENSG00000114573	*ATP6V1A*	7.182042024	-0.112604952	-0.011249441	-0.10135551	0.999933887	-0.228964142	-0.112101824	-0.116862318	0.338295593
ENSG00000154719	*MRPL39*	0.785505827	-0.062726287	-0.095320401	0.032594114	0.999933887	0.174416238	0.421290421	-0.246874183	0.338295593
ENSG00000104047	*DTWD1*	0.33387022	-0.187755709	-0.021580481	-0.166175228	0.999933887	0.006195369	0.327235885	-0.321040516	0.338307457
ENSG00000100227	*POLDIP3*	5.901445744	-0.034669296	-0.017544208	-0.017125087	0.999933887	-0.186079085	-0.282378098	0.096299013	0.338307457
ENSG00000267394	*AC004596.1*	0.16108696	0.058433784	0.281376007	-0.222942223	0.999933887	0.146851072	-0.24599524	0.392846312	0.338307457
ENSG00000100505	*TRIM9*	0.813847796	-0.034013112	0.009399311	-0.043412422	0.999933887	-0.069912485	-0.458655597	0.388743113	0.338307457
ENSG00000136527	*TRA2B*	6.640017976	0.012480947	0.015701372	-0.003220425	0.999933887	0.11978608	0.207642042	-0.087855962	0.338307457
ENSG00000184983	*NDUFA6*	3.8355635	-0.009613723	-0.036807128	0.027193404	0.999933887	-0.194620063	-0.33679429	0.142174226	0.338307457
ENSG00000167693	*NXN*	-0.995940045	0.044605549	-0.077619881	0.12222543	0.999933887	1.843565773	2.402838885	-0.559273111	0.338398211
ENSG00000100003	*SEC14L2*	0.974425595	0.062945333	-0.123596606	0.186541938	0.999933887	0.188224354	-0.099891056	0.28811541	0.338512359
ENSG00000178105	*DDX10*	1.203369418	-0.074061077	-0.111941965	0.037880888	0.999933887	0.103371458	0.349512922	-0.246141464	0.338512359
ENSG00000211795	*TRAV8-6*	-0.319719669	0.002181678	-0.287413887	0.289595565	0.999933887	0.186516854	-0.156596627	0.343113481	0.338512359
ENSG00000123360	*PDE1B*	2.53300511	-0.033653039	0.094673303	-0.128326342	0.999933887	-0.005620006	-0.213382838	0.207762831	0.338542907
ENSG00000205777	*SAMD4A*	0.007235702	0.175064088	0.502813341	-0.327749254	0.999933887	0.166306546	0.52702844	-0.360721895	0.338795787
ENSG00000187554	*TLR5*	2.2157949	0.003542624	-0.055867017	0.059409641	0.999933887	0.263373157	0.570388296	-0.307015139	0.338795787
ENSG00000125450	*NUP85*	3.935195736	-0.044292447	0.036901368	-0.081193814	0.999933887	0.042173346	-0.073899561	0.116072907	0.338849688
ENSG00000283597	*FAM169B*	-0.092872869	0.2234248	-0.024831379	0.248256179	0.999933887	-0.20792278	-0.604616597	0.396693817	0.339069522
ENSG00000277117	*FP565260.3*	-1.137130057	-0.035507573	0.245948512	-0.281456085	0.999933887	1.226995509	1.677375514	-0.450380005	0.339190279
ENSG00000112182	*BACH2*	4.11905326	-0.0176433	0.082686965	-0.100330265	0.999933887	-0.319138018	-0.458659403	0.139521385	0.339320608
ENSG00000143570	*SLC39A1*	-0.010207982	0.244509777	0.169379796	0.075129981	0.999933887	1.095975168	1.44020381	-0.344228642	0.339331085
ENSG00000126895	*AVPR2*	1.058746855	0.037253503	0.143787554	-0.106534051	0.999933887	0.030748282	0.289442402	-0.25869412	0.339829412
ENSG00000122435	*TRMT13*	1.404726832	-0.163714788	-0.17226943	0.008554642	0.999933887	-0.285583906	0.01774919	-0.303333097	0.339863653
ENSG00000101365	*IDH3B*	4.783918958	-0.03089855	0.014611284	-0.045509834	0.999933887	-0.136570168	-0.24057835	0.104008182	0.340222924
ENSG00000136932	*TRMO*	4.99045778	-0.044892826	-0.035977143	-0.008915683	0.999933887	0.17174632	0.277159563	-0.105413243	0.340230285
ENSG00000131462	*TUBG1*	0.021113652	0.039920324	0.086677879	-0.046757556	0.999933887	0.132926448	-0.201384066	0.334310514	0.340230285
ENSG00000232043	*AL133230.1*	1.029198323	-0.031839942	-0.098163715	0.066323773	0.999933887	0.456769287	0.782671771	-0.325902484	0.340280792
ENSG00000159592	*GPBP1L1*	6.267673797	-0.039352402	-0.001555851	-0.037796552	0.999933887	-0.360319277	-0.455870901	0.095551624	0.340584677
ENSG00000065923	*SLC9A7*	4.338047056	0.009144098	0.010121753	-0.000977655	0.999933887	0.173158712	0.331204317	-0.158045605	0.340713137
ENSG00000131127	*ZNF141*	2.089467524	0.078584234	-0.122450484	0.201034717	0.999933887	-0.508430365	-0.191919217	-0.316511148	0.340796842
ENSG00000129315	*CCNT1*	6.020839651	-0.057525117	-0.044159745	-0.013365372	0.999933887	0.099340128	0.208793322	-0.109453194	0.340949609
ENSG00000067082	*KLF6*	10.320545264	0.030150795	0.029254187	0.000896608	0.999933887	0.077157205	0.181288405	-0.1041312	0.340949609
ENSG00000135299	*ANKRD6*	1.815867557	-0.060364203	-0.017406719	-0.042957484	0.999933887	-0.546275403	-0.73070691	0.184431507	0.340949609
ENSG00000131389	*SLC6A6*	10.256554915	-0.012716973	-0.012087158	-0.000629816	0.999933887	0.142605135	0.234625675	-0.09202054	0.340949609
ENSG00000181826	*RELL1*	4.671257364	-0.106261425	-0.011334593	-0.094926831	0.999933887	-0.312484568	-0.45443083	0.141946262	0.341262589
ENSG00000235706	*DICER1-AS1*	0.528653179	0.035047563	-0.350708657	0.38575622	0.999933887	-0.181319796	-0.486402637	0.305082842	0.341262589
ENSG00000112312	*GMNN*	1.528535017	0.015594277	-0.012343577	0.027937854	0.999933887	0.076137655	0.275141275	-0.19900362	0.341262589
ENSG00000170581	*STAT2*	6.897219487	0.003386041	0.012423465	-0.009037424	0.999933887	-0.256231239	-0.377783515	0.121552276	0.341262589
ENSG00000244045	*TMEM199*	3.266625078	0.035713683	0.001313175	0.034400509	0.999933887	-0.014440495	0.122981566	-0.137422061	0.341714729
ENSG00000103226	*NOMO3*	0.652521392	-0.087404141	-0.000279697	-0.087124445	0.999933887	-0.025229746	-0.426268217	0.401038471	0.341963187
ENSG00000176853	*FAM91A1*	6.748810771	-0.017359551	-0.099749199	0.082389648	0.999933887	-0.247662667	-0.071938737	-0.17572393	0.341997012
ENSG00000213722	*DDAH2*	5.292440434	0.098061807	0.120730513	-0.022668706	0.999933887	-0.766053829	-0.957563298	0.191509469	0.342062328
ENSG00000084764	*MAPRE3*	2.696213156	0.058167189	0.088162069	-0.02999488	0.999933887	-0.622943416	-0.780282413	0.157338997	0.342062328
ENSG00000127337	*YEATS4*	1.203956456	-0.05395606	-0.182763492	0.128807432	0.999933887	0.264776933	0.557961485	-0.293184552	0.342098864
ENSG00000251161	*AC020661.1*	3.518126309	0.155736862	0.057841769	0.097895093	0.999933887	0.90931874	1.118625381	-0.209306641	0.342109117
ENSG00000140374	*ETFA*	2.698510615	-0.0360116	-0.17355999	0.137548391	0.999933887	-0.157652054	-0.352029787	0.194377733	0.342162645
ENSG00000112514	*CUTA*	4.303739466	0.01921681	0.044932547	-0.025715737	0.999933887	0.01981866	-0.112380517	0.132199178	0.342162645
ENSG00000285799	*AL645929.2*	-0.197604522	0.053140033	0.05411942	-0.000979387	0.999933887	0.60042733	0.176195155	0.424232175	0.342162645
ENSG00000101216	*GMEB2*	5.389642328	-0.061396735	0.002138106	-0.063534841	0.999933887	-0.254089006	-0.362215889	0.108126882	0.342240513
ENSG00000116299	*ELAPOR1*	5.159066123	0.035171633	-0.030120606	0.065292239	0.999933887	-0.247803715	-0.39304562	0.145241905	0.342347242
ENSG00000167985	*SDHAF2*	2.987390091	-0.016340384	-0.08232698	0.065986596	0.999933887	-0.02690015	0.131159115	-0.158059265	0.342623362
ENSG00000274051	*AC005837.3*	0.514605023	0.182055679	0.351958456	-0.169902777	0.999933887	0.576897425	0.945866346	-0.368968922	0.342853848
ENSG00000164631	*ZNF12*	5.160034232	-0.048788328	-0.113486199	0.06469787	0.999933887	-0.297811351	-0.102610488	-0.195200863	0.342853848
ENSG00000180549	*FUT7*	2.15704491	0.031982102	0.088322985	-0.056340883	0.999933887	-0.481167782	-0.851914407	0.370746625	0.342853848
ENSG00000126262	*FFAR2*	8.82550716	0.026354543	-0.001260145	0.027614687	0.999933887	2.082068701	2.303909946	-0.221841245	0.342853848
ENSG00000237637	*FRY-AS1*	-0.091096072	-0.016279703	0.083497151	-0.099776855	0.999933887	0.222261804	0.678265361	-0.456003557	0.342853848
ENSG00000263335	*AF001548.2*	1.170760767	0.076041625	-0.044367072	0.120408697	0.999933887	0.212422664	0.487900745	-0.275478081	0.342964912
ENSG00000183172	*SMDT1*	3.157052421	0.03951473	0.04539815	-0.00588342	0.999933887	-0.154611603	-0.311321434	0.15670983	0.342964912
ENSG00000285737	*LINC02680*	0.393253368	0.081601168	0.196961401	-0.115360233	0.999933887	0.120379878	-0.234370408	0.354750286	0.342964912
ENSG00000148908	*RGS10*	3.336659768	-0.022591168	-0.120541889	0.097950721	0.999933887	0.034901364	0.177349619	-0.142448255	0.342964912
ENSG00000198246	*SLC29A3*	0.438144173	0.007629916	0.191278152	-0.183648236	0.999933887	-0.031997995	-0.328784345	0.296786351	0.342964912
ENSG00000067560	*RHOA*	9.266940396	-0.022324988	-0.055628891	0.033303903	0.999933887	0.054949554	0.153064642	-0.098115088	0.343059112
ENSG00000020129	*NCDN*	2.37443561	0.032496462	0.123563283	-0.091066821	0.999933887	0.417141507	0.601629485	-0.184487978	0.343059112
ENSG00000110395	*CBL*	7.129070292	-0.060662064	0.027163119	-0.087825183	0.999933887	-0.937214767	-1.112631981	0.175417214	0.343088828
ENSG00000115808	*STRN*	4.985957516	-0.003678852	-0.06331348	0.059634628	0.999933887	-0.806140507	-0.997674043	0.191533536	0.343088828
ENSG00000235910	*APOA1-AS*	-0.354774261	-0.013064512	-0.00937102	-0.003693493	0.999933887	0.285522544	0.771272098	-0.485749554	0.343301716
ENSG00000197857	*ZNF44*	3.877039267	-0.085321407	-0.008719693	-0.076601714	0.999933887	0.170613468	0.333167249	-0.162553781	0.343360246
ENSG00000229848	*AC139149.1*	-0.648685394	0.237955212	0.206543097	0.031412115	0.999933887	1.04107486	0.474606216	0.566468643	0.343388302
ENSG00000005194	*CIAPIN1*	2.363782848	-0.054827404	-0.135163615	0.080336211	0.999933887	0.060023208	-0.104500094	0.164523302	0.343427592
ENSG00000174501	*ANKRD36C*	2.105908151	-0.049952603	0.059582951	-0.109535554	0.999933887	-0.174235118	0.133052678	-0.307287796	0.343427592
ENSG00000123066	*MED13L*	9.125799283	-0.030732701	-0.029845496	-0.000887205	0.999933887	-0.182576621	-0.091379847	-0.091196774	0.343559288
ENSG00000196329	*GIMAP5*	2.250181759	-0.138447039	-0.009199392	-0.129247648	0.999933887	0.271391261	0.082093966	0.189297295	0.343892865
ENSG00000258769	*RAP1AP*	1.184157771	0.140362019	-0.112817852	0.25317987	0.999933887	-0.189980433	0.054269344	-0.244249778	0.343939212
ENSG00000100372	*SLC25A17*	0.93200595	0.086863499	0.00546912	0.081394379	0.999933887	-0.086074531	-0.297258473	0.211183941	0.344006796
ENSG00000125124	*BBS2*	3.138906874	-0.046696557	0.078193467	-0.124890024	0.999933887	0.144299608	0.012244629	0.132054979	0.34409437
ENSG00000160714	*UBE2Q1*	5.974629053	0.01618447	0.022801035	-0.006616565	0.999933887	-0.069391612	0.021917651	-0.091309263	0.34409437
ENSG00000171049	*FPR2*	7.843899173	0.035753563	-0.001268938	0.037022501	0.999933887	0.692962334	0.817828462	-0.124866128	0.344113797
ENSG00000174842	*GLMN*	0.719702315	0.018460421	-0.242015761	0.260476182	0.999933887	-0.133840873	0.16963605	-0.303476923	0.344290012
ENSG00000184402	*SS18L1*	4.249788199	0.024752875	-9.7332e-05	0.024850207	0.999933887	-0.008021596	-0.132714295	0.124692699	0.344394113
ENSG00000198780	*FAM169A*	0.579030145	-0.21668549	-0.151341663	-0.065343827	0.999933887	-0.214164167	0.145148152	-0.359312318	0.344483074
ENSG00000083635	*NUFIP1*	2.052615656	0.010152165	-0.117901928	0.128054093	0.999933887	0.547331589	0.834867778	-0.287536189	0.344483074
ENSG00000275022	*MIR6753*	1.509318352	0.045278996	0.039045387	0.006233609	0.999933887	-0.40546831	-0.153553763	-0.251914547	0.344492098
ENSG00000163686	*ABHD6*	0.672135939	-0.013674361	-0.049300938	0.035626576	0.999933887	0.114303951	0.342703054	-0.228399102	0.344492098
ENSG00000173578	*XCR1*	-0.311360443	-0.022115598	-0.017026215	-0.005089383	0.999933887	-1.08787244	-1.390447009	0.302574569	0.344492098

ENSG00000179818	*PCBP1-AS1*	6.979802649	0.017883693	0.107584674	-0.089700981	0.999933887	0.489072048	0.602003142	-0.112931094	0.34460163
ENSG00000242113	*RN7SL124P*	0.629289823	0.263977351	0.418946373	-0.154969023	0.999933887	3.492248646	4.054980505	-0.562731858	0.344636012
ENSG00000130227	*XPO7*	4.143394428	-0.047765028	0.00424709	-0.052012117	0.999933887	-0.169875096	-0.298661183	0.128786087	0.344636012
ENSG00000139318	*DUSP6*	7.122415804	-0.048689708	-0.015388571	-0.033301138	0.999933887	0.075470905	0.263522613	-0.188051708	0.344636012
ENSG00000286599	*AC110611.1*	-0.186521137	-0.040706726	-0.00994367	-0.030763056	0.999933887	-0.122285012	0.353922216	-0.476207228	0.344636012
ENSG00000272374	*Z97832.2*	-0.220182229	0.082210207	-0.187519715	0.269729922	0.999933887	0.188172801	0.619913653	-0.431740852	0.344777713
ENSG00000156136	*DCK*	3.370513322	-0.107672052	0.033822805	-0.141494857	0.999933887	-0.388722812	-0.543427408	0.154704596	0.344882174
ENSG00000184967	*NOC4L*	3.109908244	0.064654293	0.036389308	0.028264985	0.999933887	0.263357425	0.433777734	-0.17042031	0.344884631
ENSG00000179820	*MYADM*	8.417082576	0.045772684	0.068356639	-0.022583955	0.999933887	-0.033169935	-0.162522147	0.129352212	0.345108809
ENSG00000080824	*HSP90AA1*	8.093110987	-0.147444109	-0.20766965	0.060225541	0.999933887	0.348555321	0.559116428	-0.210561107	0.345193997
ENSG00000102984	*ZNF821*	1.520209715	0.080125481	-0.003036118	0.083161599	0.999933887	0.063004475	0.284826053	-0.221821578	0.34522745
ENSG00000163541	*SUCLG1*	3.310226978	0.022724045	-0.146500142	0.169224187	0.999933887	0.033109798	0.172478009	-0.139368211	0.34525022
ENSG00000276728	*AC142472.1*	-0.195812751	0.187689674	-0.023884237	0.211573911	0.999933887	-0.179864834	0.141082524	-0.320947358	0.345318873
ENSG00000156802	*ATAD2*	2.433923508	-0.101611118	0.009184827	-0.110795945	0.999933887	-0.24196467	0.111798632	-0.353763302	0.345318873
ENSG00000120992	*LYPLA1*	4.235766183	-0.072829228	-0.0765628	0.003733572	0.999933887	-0.225355188	-0.100737779	-0.124617409	0.345661848
ENSG00000160229	*ZNF66*	1.758874479	-0.012717288	-0.048495015	0.035777727	0.999933887	-0.575063069	-0.980470823	0.405407754	0.345661848
ENSG00000261468	*AC096921.2*	1.238622465	0.047788863	0.001363283	0.046425581	0.999933887	-0.118496848	-0.482521695	0.364024847	0.345800449
ENSG00000176225	*RTTN*	1.87093078	0.039104267	-0.11163721	0.150741477	0.999933887	-0.107337205	0.131458405	-0.23879561	0.345813316
ENSG00000125814	*NAPB*	2.750062861	-0.09365231	0.013576055	-0.107228365	0.999933887	-0.209024122	-0.394822623	0.185798501	0.34640681
ENSG00000157152	*SYN2*	-0.381303369	0.126828111	-0.014272431	0.141100542	0.999933887	-0.081645303	-0.576387003	0.4947417	0.34640681
ENSG00000235958	*UBOX5-AS1*	-0.262089249	-0.143420306	-0.316313761	0.172893455	0.999933887	0.090010896	0.522909723	-0.432898827	0.34640681
ENSG00000160221	*GATD3A*	-0.082545364	-0.14809618	-0.230256222	0.082160042	0.999933887	0.248629015	-0.041149382	0.289778397	0.347091517
ENSG00000141562	*NARF*	6.349602605	-0.009726325	0.034301292	-0.044027617	0.999933887	-0.041296149	-0.161730546	0.120434397	0.347241815
ENSG00000125648	*SLC25A23*	0.979330555	0.128554744	0.05236438	0.076190363	0.999933887	0.082667149	-0.216393897	0.299061045	0.347357337
ENSG00000005700	*IBTK*	2.286625158	-0.053381364	-0.068147837	0.014766474	0.999933887	-0.128375222	0.141089327	-0.269464549	0.347357337
ENSG00000172086	*KRCC1*	3.453247984	0.028156318	-0.115771601	0.143927919	0.999933887	-0.138137496	0.015509472	-0.153646967	0.347357337
ENSG00000260302	*LINC01882*	-0.860276536	0.024222019	-0.45384835	0.478070369	0.999933887	0.015923744	0.498475574	-0.482551831	0.347357337
ENSG00000172239	*PAIP1*	2.45648233	-0.071584469	-0.024307606	-0.047276863	0.999933887	0.006254548	-0.163025621	0.169280169	0.34740548
ENSG00000165916	*PSMC3*	3.804741076	-0.043173784	0.005157019	-0.048330803	0.999933887	0.078580484	0.206055508	-0.127475024	0.34740548
ENSG00000145945	*FAM50B*	-0.616955929	0.131641183	0.024392557	0.107248626	0.999933887	0.174090818	-0.248762041	0.422852859	0.34740548
ENSG00000226696	*LENG8-AS1*	0.823062216	-0.045303368	-0.036831456	-0.008471911	0.999933887	0.004648604	-0.209196671	0.213845275	0.34740548
ENSG00000137504	*CREBZF*	5.276315989	-0.012179737	0.049805142	-0.061984879	0.999933887	-0.193860945	-0.30716143	0.113300485	0.347467454
ENSG00000078668	*VDAC3*	4.363814656	-0.069357172	-0.162682519	0.093325347	0.999933887	0.098512295	-0.023314012	0.121826307	0.347495649
ENSG00000170522	*ELOVL6*	-0.4540306	-0.081610931	-0.107978049	0.026367118	0.999933887	-0.268356924	0.1712062	-0.439563124	0.347495649
ENSG00000181830	*SLC35C1*	2.224583919	0.075510383	-0.144748441	0.220258824	0.999933887	-0.206134537	-0.437081948	0.230947411	0.347630955
ENSG00000258682	*AL132989.1*	1.356083052	0.085619033	-0.314856752	0.400475785	0.999933887	-0.248155389	-0.010803868	-0.237351522	0.347630955
ENSG00000267731	*AC005332.2*	-0.639571254	-0.260222432	0.238731446	-0.498953877	0.999933887	-0.180929051	0.276307035	-0.457236086	0.347678901
ENSG00000101146	*RAE1*	3.935619718	-0.126854067	-0.156549961	0.029695894	0.999933887	-0.257657791	-0.37179342	0.114135629	0.347776948
ENSG00000057757	*PITHD1*	4.322216725	0.053275979	-0.014336983	0.067612962	0.999933887	-0.302081945	-0.442281731	0.140199786	0.348765561
ENSG00000125356	*NDUFA1*	4.686651911	-0.001428745	-0.020187553	0.018758808	0.999933887	0.09557118	-0.032253741	0.127824921	0.348765561
ENSG00000183638	*RP1L1*	-2.659235282	0.146377212	0.351148653	-0.204771441	0.999933887	-0.158854446	-1.076324898	0.917470453	0.349179103
ENSG00000178458	*H3P16*	0.125337931	-0.068737433	0.01094287	-0.079680303	0.999933887	-0.451217877	-0.058542516	-0.392675362	0.349307626
ENSG00000177169	*ULK1*	7.961077405	-0.012827991	0.019976819	-0.03280481	0.999933887	-0.728154058	-0.858173506	0.130019448	0.349307626
ENSG00000226210	*WASH8P*	3.527076066	0.010175101	0.056778261	-0.046603161	0.999933887	-0.207325486	-0.337800138	0.130474652	0.349373608
ENSG00000137871	*ZNF280D*	0.756016469	-0.072761412	-0.040314538	-0.032446875	0.999933887	-0.309261145	0.051440082	-0.360701227	0.349655685
ENSG00000186364	*NUDT17*	-0.454660185	0.079556104	-0.207063654	0.286619758	0.999933887	0.267979965	0.600416658	-0.332436693	0.349808807
ENSG00000122085	*MTERF4*	2.899105092	-0.028470245	-0.068642515	0.04017227	0.999933887	0.051424166	-0.094304714	0.145728881	0.349831648
ENSG00000143162	*CREG1*	6.94431823	-0.076642703	-0.115982394	0.03933969	0.999933887	-0.322582368	-0.192559833	-0.130022536	0.349887003
ENSG00000198000	*NOL8*	3.746456039	-0.079163672	-0.199083556	0.119919885	0.999933887	-0.375776917	-0.135436004	-0.240340913	0.349887003
ENSG00000151893	*CACUL1*	6.939697518	-0.028234355	0.018394951	-0.046629307	0.999933887	-0.642884789	-0.755531404	0.112646615	0.349887003
ENSG00000141933	*TPGS1*	1.359644594	0.1892375	-0.022398036	0.211635536	0.999933887	-0.209424394	0.039879895	-0.249304289	0.350042714
ENSG00000135930	*EIF4E2*	4.618245173	-0.025962256	-0.099892489	0.073930233	0.999933887	-0.221921749	-0.329410664	0.107488915	0.350042714
ENSG00000125775	*SDCBP2*	-0.183952476	0.073631809	0.159568954	-0.085937144	0.999933887	-0.102263268	-0.567234132	0.464970863	0.350147959
ENSG00000117528	*ABCD3*	2.073559942	-0.146976691	0.016199621	-0.163176312	0.999933887	-0.2769444	-0.529872156	0.252927756	0.350343958
ENSG00000287237	*AC073655.3*	1.717267772	-0.141142613	0.179825231	-0.320967844	0.999933887	0.970286859	1.223250633	-0.252963774	0.350377122
ENSG00000095303	*PTGS1*	2.978624938	0.035338059	-0.026158939	0.061496998	0.999933887	-0.103537713	0.065361845	-0.168899558	0.350377122
ENSG00000005206	*SPPL2B*	4.014336128	0.025578015	0.059539998	-0.033961983	0.999933887	0.027833067	-0.132575454	0.160408521	0.350377122
ENSG00000106733	*NMRK1*	3.585486124	-0.006898455	0.028756068	-0.035654522	0.999933887	-0.266654168	-0.407671187	0.141017019	0.350377122
ENSG00000243704	*RN7SL105P*	-0.106888984	0.00873719	-0.140248059	0.14898525	0.999933887	0.263774124	0.667268215	-0.403494091	0.350377122
ENSG00000261101	*AC234775.2*	0.263115654	0.058476462	-0.066254827	0.124731289	0.999933887	0.555904374	0.953980806	-0.398076432	0.350445309
ENSG00000160352	*ZNF714*	1.689309793	-0.031574057	-0.017876203	-0.013697854	0.999933887	-0.126672785	0.128818798	-0.255491583	0.35045011
ENSG00000064651	*SLC12A2*	2.275467716	0.019137415	-0.034859711	0.053997125	0.999933887	-0.190430931	0.062884062	-0.253314993	0.350639373
ENSG00000168003	*SLC3A2*	9.393148447	-0.008991904	0.005865828	-0.014857732	0.999933887	-0.023399128	0.088505146	-0.111904274	0.350847136
ENSG00000160408	*ST6GALNAC6*	-0.786098414	0.148956922	0.536166367	-0.387209445	0.999933887	0.041941792	-0.408155161	0.450096953	0.350875564
ENSG00000111707	*SUDS3*	5.63520046	-0.030389697	0.050239654	-0.080629351	0.999933887	-0.403499263	-0.509188284	0.105689021	0.350875564
ENSG00000135315	*CEP162*	1.809359156	-0.227113178	-0.347464064	0.120350886	0.999933887	-0.448611842	-0.156379648	-0.292232195	0.35124101
ENSG00000099904	*ZDHHC8*	2.913592741	0.066427171	0.024401642	0.042025529	0.999933887	0.073590536	-0.080896951	0.154487487	0.351242345
ENSG00000071655	*MBD3*	2.670761763	0.045699563	0.057109798	-0.011410235	0.999933887	-0.032433243	-0.193906087	0.161472844	0.351242345
ENSG00000004975	*DVL2*	4.971026376	0.042550274	0.12617549	-0.083625215	0.999933887	-0.500664721	-0.641593416	0.140928696	0.351242345
ENSG00000122257	*RBBP6*	6.545793942	0.010495939	-0.070558632	0.081054571	0.999933887	-0.160627216	-0.011363072	-0.149264143	0.351242345
ENSG00000164684	*ZNF704*	-1.5133409	0.009050141	-0.423629294	0.432679434	0.999933887	-0.316252831	0.07513073	-0.391383561	0.351242345
ENSG00000108599	*AKAP10*	5.086703254	-0.053214154	-0.044435827	-0.008778327	0.999933887	-0.41118291	-0.507733803	0.096550893	0.351256165
ENSG00000148429	*USP6NL*	1.944864469	-0.023325319	0.006713567	-0.030038886	0.999933887	-0.332221962	-0.56917474	0.236952777	0.351378471
ENSG00000162430	*SELENON*	2.550944076	0.11673335	0.103784468	0.012948881	0.999933887	-0.099520564	-0.300424373	0.200903809	0.351408501
ENSG00000161091	*MFSD12*	6.911023415	0.063799752	0.068187767	-0.004388015	0.999933887	0.318822555	0.444594779	-0.125772223	0.351408501
ENSG00000287091	*AC009961.3*	0.556507958	0.037167824	0.116305506	-0.079137682	0.999933887	-0.896046827	-1.197591956	0.30154513	0.351444194
ENSG00000228261	*ITPRIP-AS1*	3.279307051	0.213578328	0.246144124	-0.032565796	0.999933887	1.287922097	1.589997711	-0.302075614	0.35148982
ENSG00000135049	*AGTPBP1*	6.708864306	-0.046787549	-0.107681515	0.060893966	0.999933887	0.054523822	0.249396062	-0.19487224	0.351557808
ENSG00000262903	*AC027796.4*	0.1780978	0.050860698	-0.026352509	0.077213207	0.999933887	0.07181965	-0.247946006	0.319765657	0.351557808
ENSG00000148484	*RSU1*	3.990600694	0.042714604	0.007297013	0.035417591	0.999933887	-0.025671257	0.115008344	-0.140679601	0.351608515
ENSG00000137802	*MAPKBP1*	3.743560151	-0.018697367	0.093248075	-0.111945442	0.999933887	-0.082637113	-0.18356716	0.100930047	0.351608515
ENSG00000249379	*AL033397.2*	-1.113143044	0.075993458	-0.071816346	0.147809804	0.999933887	1.207746869	1.602089278	-0.394342409	0.351608515
ENSG00000254632	*AP003119.1*	-0.569905368	0.379390363	0.777838473	-0.39844811	0.999933887	1.029968865	1.325867191	-0.295898325	0.351799078
ENSG00000224078	*SNHG14*	3.234431898	-0.057026138	0.027227155	-0.084253294	0.999933887	-0.233886852	-0.03320948	-0.200677371	0.351799078
ENSG00000120254	*MTHFD1L*	1.239075089	-0.033824557	0.008777977	-0.042602534	0.999933887	0.109913197	0.317095879	-0.207182682	0.352110272
ENSG00000255097	*AP006259.1*	0.047991689	-0.066831876	0.050322612	-0.117154487	0.999933887	0.902021586	1.342963678	-0.440942092	0.352161969
ENSG00000166454	*ATMIN*	3.415805902	0.071020042	-0.15756811	0.228588152	0.944094537	-0.05423138	-0.201254519	0.147023139	0.352521291
ENSG00000142192	*APP*	4.131568789	0.032813067	-0.018491544	0.051304611	0.999933887	-0.121088453	-0.249862764	0.12877431	0.352720904
ENSG00000185864	*NPIPB4*	4.313147683	0.034776182	-0.016644389	0.051420571	0.999933887	0.058381884	0.18115887	-0.122776986	0.352772147
ENSG00000152380	*FAM151B*	1.079325306	-0.014868752	-0.184768377	0.169899626	0.999933887	0.005557112	0.213545132	-0.20798802	0.352772147
ENSG00000078319	*PMS2P1*	0.764239792	-0.12340407	-0.105639572	-0.017764498	0.999933887	0.095592231	0.378384183	-0.282791952	0.35290127
ENSG00000279078	*SND1-IT1*	-0.409351536	-0.016753824	-0.062887261	0.046133437	0.999933887	-0.751224253	-1.272104518	0.520880264	0.352913778
ENSG00000143889	*HNRNPLL*	4.192811192	-0.008833143	0.01561078	-0.024443923	0.999933887	-0.027489692	-0.149198741	0.121709049	0.352925843
ENSG00000250696	*AC111000.4*	-0.203645374	-0.211005101	-0.144125114	-0.066879987	0.999933887	-0.502791383	-0.079245604	-0.423545779	0.352936352
ENSG00000140199	*SLC12A6*	9.438144699	-0.008477561	-0.001646773	-0.006830788	0.999933887	-0.151171368	-0.063795286	-0.087376082	0.353061853
ENSG00000137713	*PPP2R1B*	2.976928779	-0.015529356	0.011112822	-0.026642178	0.999933887	0.102264042	0.274046706	-0.171782664	0.353123988
ENSG00000164040	*PGRMC2*	3.138218539	0.054345289	0.000671071	0.053674218	0.999933887	-0.227795854	-0.394330958	0.166535103	0.353939412
ENSG00000121067	*SPOP*	5.269632138	-0.096360545	-0.092702938	-0.003657607	0.999933887	-0.445831988	-0.565463109	0.119631121	0.353975889
ENSG00000172757	*CFL1*	8.701399177	0.014597035	-0.008805603	0.023402638	0.999933887	-0.139559525	-0.234750452	0.095190927	0.354142356
ENSG00000152582	*SPEF2*	0.367782365	-0.191341122	-0.497923184	0.306582062	0.999933887	-0.124293906	0.208560955	-0.332854861	0.35421251
ENSG00000132002	*DNAJB1*	7.066580199	0.060968987	0.005955945	0.055013042	0.999933887	0.153670655	0.340548631	-0.186877975	0.354216864
ENSG00000172057	*ORMDL3*	4.221535806	0.04159434	0.104647967	-0.063053627	0.999933887	0.064777248	-0.079590974	0.144368222	0.354216864
ENSG00000114812	*VIPR1*	1.294519302	0.024252319	0.049598838	-0.025346519	0.999933887	-0.061106001	0.20481783	-0.265923831	0.354216864
ENSG00000022556	*NLRP2*	0.721392727	-0.128956001	0.13798825	-0.266944251	0.999933887	-0.042906582	0.217242998	-0.26014958	0.35451793
ENSG00000198208	*RPS6KL1*	-0.70218667	0.129872237	-0.272542504	0.40241474	0.999933887	-0.179580976	0.291210756	-0.470791732	0.35451793

ENSG00000161618	*ALDH16A1*	2.812605969	0.022277659	0.1006259	-0.078348241	0.999933887	0.005828021	-0.204236424	0.210064445	0.35451793
ENSG00000152348	*ATG10*	0.099883273	-0.024490114	0.022737343	-0.047227457	0.999933887	0.010635194	-0.408065193	0.418700388	0.35451793
ENSG00000109911	*ELP4*	0.681165284	0.233802019	0.010274759	0.223527261	0.999933887	-0.001206608	-0.338271795	0.337065187	0.354566477
ENSG00000280254	*AC233723.2*	0.760334952	-0.199707381	-0.205908431	0.006201049	0.999933887	0.035804938	0.40290067	-0.367095732	0.354970622
ENSG00000285850	*AC009152.3*	-0.184502454	-0.107053254	0.097352373	-0.204405627	0.999933887	-0.090340091	-0.585827757	0.495487665	0.354998195
ENSG00000267898	*AC026803.2*	1.789180905	0.126597921	-0.039399003	0.165996924	0.999933887	0.811521225	1.063667931	-0.252146706	0.355079573
ENSG00000171055	*FEZ2*	2.815488667	-0.046845014	-0.081922563	0.035077549	0.999933887	-0.29374194	-0.475995258	0.182253318	0.355079573
ENSG00000127914	*AKAP9*	4.987613431	-0.124039896	-0.144475465	0.020435569	0.999933887	-0.62351261	-0.232415137	-0.391097473	0.355317641
ENSG00000090674	*MCOLN1*	8.106500044	0.007366072	0.010157061	-0.002790989	0.999933887	0.201300657	0.307622492	-0.106321835	0.355323826
ENSG00000101782	*RIOK3*	6.543352794	-0.078801406	-0.082030565	0.003229159	0.999933887	-0.275937254	-0.18684425	-0.089093005	0.355346346
ENSG00000149531	*FRG1BP*	0.333922598	-0.221310347	0.032213041	-0.253523388	0.999933887	-0.070386486	0.220856251	-0.291242737	0.355346346
ENSG00000283573	*AL157371.2*	2.02113488	0.122541947	0.172474207	-0.049932261	0.999933887	0.400816371	0.613886998	-0.213070627	0.355346346
ENSG00000171813	*PWWP2B*	3.666612294	0.023350744	-0.01202148	0.035372224	0.999933887	-0.231895667	-0.407841638	0.175945972	0.355346346
ENSG00000232545	*AC253536.3*	-0.698536236	-0.001136879	0.059735856	-0.060872735	0.999933887	-0.023701704	0.416296277	-0.43999798	0.355387896
ENSG00000175787	*ZNF169*	1.368350021	-0.054719128	-0.083509637	0.028790509	0.999933887	-0.055271926	-0.263687431	0.208415505	0.355977015
ENSG00000226742	*HSBP1L1*	1.548288812	-0.028847669	-0.114471769	0.0856241	0.999933887	-0.605510601	-0.820801394	0.215290792	0.355977015
ENSG00000227218	*AL157935.1*	-0.560209144	0.019605321	0.143325607	-0.123720286	0.999933887	-0.109391653	-0.52027171	0.410880057	0.355977015
ENSG00000103707	*MTFMT*	1.875024486	0.035969944	0.223492924	-0.187522979	0.999933887	-0.393715934	-0.594404943	0.200689009	0.356041685
ENSG00000068438	*FTSJ1*	3.195099048	-0.015461418	0.05703249	-0.072493908	0.999933887	0.14868579	0.294829944	-0.146144153	0.356041685
ENSG00000103005	*USB1*	6.165794939	0.045070264	0.0269466	0.018123663	0.999933887	-0.070924893	-0.187725662	0.116800769	0.356378959
ENSG00000161671	*EMC10*	3.697993462	0.022698738	0.060372044	-0.037673306	0.999933887	0.031315625	-0.112421964	0.14373759	0.356378959
ENSG00000072501	*SMC1A*	4.844964679	-9.2528e-05	-0.077804213	0.077711685	0.999933887	-0.014743461	-0.116655983	0.101912522	0.356378959
ENSG00000063245	*EPN1*	6.241490577	0.037017346	0.005527575	0.031489771	0.999933887	-0.430328304	-0.540582635	0.110254331	0.35652937
ENSG00000090054	*SPTLC1*	4.510396311	-0.06780571	-0.055823863	-0.011981847	0.999933887	-0.119922231	-0.010489238	-0.109432993	0.356880292
ENSG00000184221	*OLIG1*	5.638544008	-0.258780094	-0.306452096	0.047672002	0.999933887	-0.60374426	-0.328075446	-0.275668814	0.35694399
ENSG00000076513	*ANKRD13A*	7.018866299	-0.026798023	-0.025715512	-0.001082512	0.999933887	0.263277896	0.382618525	-0.119340629	0.357025064
ENSG00000131061	*ZNF341*	2.112106855	0.031154473	-0.031282152	0.062436625	0.999933887	-0.133438198	-0.38574931	0.252311113	0.357025064
ENSG00000228923	*AP000355.1*	1.765231273	-0.096498151	0.103294003	-0.199792154	0.999933887	1.968624991	2.249900755	-0.281275764	0.357174819
ENSG00000140848	*CPNE2*	4.023965172	0.024705316	0.00972188	0.014983437	0.999933887	-0.690544489	-0.838503031	0.147958542	0.357174819
ENSG00000151150	*ANK3*	1.923752449	0.015036146	-0.008849466	0.023885612	0.999933887	-0.045169135	0.180316065	-0.2254852	0.357296442
ENSG00000163682	*RPL9*	7.4794904	-0.005732044	-0.036273427	0.030541383	0.999933887	0.1380829	0.249871946	-0.111789046	0.357504145
ENSG00000198734	*F5*	5.494285285	-0.049137982	-0.119938299	0.070800317	0.999933887	-0.420356974	-0.565716671	0.145359697	0.357509568
ENSG00000145241	*CENPC*	3.529254886	-0.017327101	-0.073479436	0.056152335	0.999933887	-0.20254082	0.157502246	-0.360043066	0.35774515
ENSG00000177494	*ZBED2*	-0.020405615	0.116259734	0.163319303	-0.047059569	0.999933887	-0.101493386	0.320941056	-0.422434442	0.357790074
ENSG00000116128	*BCL9*	4.088692084	-0.002239492	0.024705664	-0.026945156	0.999933887	-0.321257685	-0.184073033	-0.137184653	0.357806613
ENSG00000144136	*SLC20A1*	8.433244065	-0.047789401	0.032636562	-0.080425663	0.999933887	-0.453723701	-0.585037194	0.131313492	0.35820432
ENSG00000172071	*EIF2AK3*	5.033233361	0.009211509	-0.131237738	0.140449247	0.999933887	0.163493722	0.38559389	-0.222100168	0.358472074
ENSG00000143153	*ATP1B1*	-0.258498293	0.218374714	-0.089295073	0.307669787	0.999933887	-0.004198694	0.480391954	-0.484590648	0.358484809
ENSG00000103274	*NUBP1*	1.269119027	0.056236631	-0.130287945	0.186524576	0.999933887	-0.108001496	0.138663979	-0.246665474	0.358484809
ENSG00000120868	*APAF1*	5.255846987	-0.064928051	0.028652483	-0.093580534	0.999933887	-0.423471673	-0.569511886	0.146040213	0.358549544
ENSG00000116455	*WDR77*	1.305458704	-0.106922209	0.109383712	-0.21630592	0.999933887	0.101801431	-0.117352415	0.219153846	0.358549544
ENSG00000153574	*RPIA*	2.581139018	-0.009964451	0.038503585	-0.048468036	0.999933887	0.157780371	-0.012423241	0.170203612	0.358549544
ENSG00000171148	*TADA3*	5.323937642	0.02251014	0.014110001	0.008400139	0.999933887	-0.279752331	-0.39390943	0.114157099	0.358549932
ENSG00000105875	*WDR91*	7.082617896	0.01728914	0.077849118	-0.060559977	0.999933887	-0.239554751	-0.144830353	-0.094724398	0.358549932
ENSG00000083454	*P2RX5*	0.706745193	0.053110915	-0.141534896	0.194645811	0.999933887	0.129661833	-0.146842368	0.276504201	0.358839724
ENSG00000196781	*TLE1*	1.902813071	0.000907916	0.06679776	-0.065889843	0.999933887	0.588658092	0.817375565	-0.228717473	0.358839724
ENSG00000267493	*CIRBP-AS1*	1.281881005	-0.1052197	-0.062682495	-0.042537205	0.999933887	-0.275979699	-0.518016512	0.242036813	0.358843136
ENSG00000049247	*UTS2*	-2.806000659	0.149347421	-0.565476276	0.714823697	0.999933887	-0.144771686	0.392184635	-0.536956322	0.358904523
ENSG00000286786	*AC116158.3*	1.340154862	-0.199388199	0.055667184	-0.255055383	0.999933887	-0.194449322	0.16084007	-0.355289392	0.360594336
ENSG00000186187	*ZNRF1*	1.321117236	-0.125816528	0.218670863	-0.344487391	0.999933887	-0.156465008	-0.43664758	0.280182573	0.360594336
ENSG00000163617	*CCDC191*	1.141949104	0.014744216	-0.17229402	0.187038236	0.999933887	-0.192964846	0.12831462	-0.321279466	0.360594336
ENSG00000213585	*VDAC1*	3.509860399	0.036712655	-0.089529155	0.126241809	0.999933887	-0.008701901	0.128590862	-0.137292763	0.360607712
ENSG00000081791	*DELE1*	5.398371039	-0.02172351	-0.00616273	-0.01556078	0.999933887	-0.082535393	-0.16659284	0.084057447	0.360607712
ENSG00000163659	*TIPARP*	6.112379112	-0.028103057	-0.042090203	0.013987147	0.999933887	0.071715333	0.166491387	-0.094776054	0.36064981
ENSG00000128567	*PODXL*	0.054050208	0.125775057	-0.037664373	0.16343943	0.999933887	-0.212051343	-0.615713617	0.403662274	0.360686344
ENSG00000161912	*ADCY10P1*	3.051880221	-0.019639035	-0.099811001	0.080171966	0.999933887	0.090652065	0.289476314	-0.198824249	0.360686344
ENSG00000282826	*FRG1CP*	1.375804985	-0.026195596	-0.019268751	-0.006926845	0.999933887	0.091364884	-0.07759564	0.168960524	0.360687317
ENSG00000094975	*SUCO*	5.585018969	-0.004673814	-0.120641431	0.115967617	0.999933887	-0.068809011	0.189914488	-0.258723499	0.360687317
ENSG00000170004	*CHD3*	7.248221749	0.031350621	0.042607206	-0.011256585	0.999933887	0.027104235	-0.050198964	0.077303198	0.36072979
ENSG00000177200	*CHD9*	3.696866315	-0.082049359	-0.154350548	0.072301189	0.999933887	-0.432254558	-0.162560459	-0.269694099	0.36072979
ENSG00000049245	*VAMP3*	5.78958427	-0.032237109	-0.000358051	-0.031879058	0.999933887	-0.709990168	-0.84001985	0.130029682	0.36072979
ENSG00000107890	*ANKRD26*	0.545235764	-0.088757886	-0.176348997	0.087591111	0.999933887	-0.266654613	0.182068861	-0.448723473	0.36072979
ENSG00000281990	*IGHV1-69-2*	-1.878267035	0.167143795	0.268361777	-0.101217983	0.999933887	0.217371417	-0.311811257	0.529182673	0.360797226
ENSG00000258034	*AC012157.2*	-0.351626018	-0.063381374	-0.02081815	-0.042563224	0.999933887	0.161262507	0.670891176	-0.509628669	0.360797226
ENSG00000176386	*CDC26*	3.306479031	0.013597292	-0.100143875	0.113741167	0.999933887	-0.062845636	0.079908313	-0.142753949	0.360797226
ENSG00000102109	*PCSK1N*	0.23750683	0.046573191	0.092319779	-0.045746588	0.999933887	0.23530347	0.574932345	-0.339628875	0.36080581
ENSG00000025293	*PHF20*	6.326554385	-0.061903132	-0.042342038	-0.019561094	0.999933887	-0.235867138	-0.334220568	0.09835343	0.360878293
ENSG00000233431	*LINC02596*	0.363072893	-0.01036763	0.133133513	-0.143501142	0.999933887	0.341349071	-0.144145512	0.485494583	0.360878293
ENSG00000106683	*LIMK1*	4.207537664	0.002143857	0.043842888	-0.04169903	0.999933887	-0.053186958	-0.190130075	0.136916117	0.360878293
ENSG00000155846	*PPARGC1B*	0.083817235	0.013335446	-0.180845469	0.194180915	0.999933887	-0.274624438	0.069454132	-0.34407857	0.36092614
ENSG00000159210	*SNF8*	3.953925884	-0.030632071	-0.025485385	-0.005146686	0.999933887	0.322267876	0.431570044	-0.109302168	0.36101882
ENSG00000197299	*BLM*	1.066485812	-0.233347064	-0.072165999	-0.161181065	0.999933887	-0.180214539	0.040006558	-0.220221098	0.361105283
ENSG00000259962	*AC007342.2*	-0.931597219	-0.175998165	-0.780959706	0.604961541	0.999933887	-1.011621916	-1.544172633	0.532550717	0.361199691
ENSG00000259671	*MTCYBP23*	3.724417097	0.331451237	0.611899405	-0.280448168	0.999933887	2.06420905	2.524689447	-0.460480397	0.361352137
ENSG00000204178	*MACO1*	3.710330426	-0.013506816	-0.115331309	0.101824493	0.999933887	-0.102067934	0.011509361	-0.113577295	0.361352137
ENSG00000232888	*RPS11P5*	-0.555938203	-0.049398236	0.166949599	-0.216347835	0.999933887	-0.415627137	-0.009054701	-0.406572435	0.361352137
ENSG00000268061	*NAPA-AS1*	2.522260848	0.131019405	0.267772895	-0.13675349	0.999933887	0.309770388	0.562596305	-0.252825917	0.361394836
ENSG00000136367	*ZFHX2*	1.10371479	-0.10452435	0.148625165	-0.253149515	0.999933887	2.01505463	2.358230583	-0.343175953	0.361472733
ENSG00000073584	*SMARCE1*	1.448085843	-0.165917921	-0.034862923	-0.131054998	0.999933887	-0.384239118	-0.120389413	-0.263849705	0.36152472
ENSG00000175455	*CCDC14*	2.477902514	-0.12289013	-0.306324955	0.183434825	0.999933887	-0.309640865	0.040196457	-0.349837322	0.36152472
ENSG00000280205	*AC009716.2*	0.442062769	0.073263455	0.296857202	-0.223593747	0.999933887	-0.051700632	-0.446110318	0.394409687	0.36152472
ENSG00000186462	*NAP1L2*	0.03076592	-0.068307155	0.244674532	-0.312981688	0.999933887	0.075999885	0.45435371	-0.378353826	0.36152472
ENSG00000141577	*CEP131*	2.891563382	0.087747764	0.188092675	-0.100344911	0.999933887	0.075702025	0.220146674	-0.144444649	0.361781234
ENSG00000103194	*USP10*	6.14833005	-0.053305307	-0.07203572	0.018730413	0.999933887	-0.045026335	0.062604837	-0.107631172	0.361781234
ENSG00000010278	*CD9*	5.645110563	-0.109933115	0.00920388	-0.119136995	0.999933887	-1.576421192	-1.829326259	0.252905067	0.361892865
ENSG00000169045	*HNRNPH1*	8.358918157	-0.025019441	-0.028016341	0.002996901	0.999933887	0.00357076	0.132026616	-0.128455856	0.361986766
ENSG00000081913	*PHLPP1*	3.940471875	0.037823268	0.028865847	0.008957421	0.999933887	0.498110556	0.652582183	-0.154471628	0.362101545
ENSG00000162777	*DENND2D*	5.917967184	0.033126259	-0.007676245	0.040802503	0.999933887	0.304542099	0.421588157	-0.117046058	0.362111968
ENSG00000007264	*MATK*	4.193213542	0.025981775	0.076087899	-0.050106125	0.999933887	-0.034366065	-0.162608294	0.128242229	0.36216305
ENSG00000108839	*ALOX12*	4.902523687	0.110150747	0.118316098	-0.008165351	0.999933887	0.227351133	0.336883461	-0.109532328	0.362181082
ENSG00000270164	*LINC01480*	0.427695897	0.13979113	0.090534609	0.049256521	0.999933887	-0.497730146	-0.016710764	-0.481019382	0.362181082
ENSG00000137414	*FAM8A1*	6.950513556	-0.031488255	-0.004570588	-0.026917668	0.999933887	-0.373747307	-0.49503959	0.121292283	0.362181082
ENSG00000184956	*MUC6*	-0.742070872	0.028893865	0.072484536	-0.043590671	0.999933887	-0.111723646	-0.715737002	0.604013356	0.362265116
ENSG00000253616	*AC107959.3*	2.031289071	0.017447956	-0.14949837	0.166946326	0.999933887	-1.481906547	-1.806813841	0.324907294	0.362265116
ENSG00000268471	*MIR4453HG*	1.754664051	0.008148223	0.047392107	-0.039243884	0.999933887	0.251615168	0.469386426	-0.217771257	0.362265189
ENSG00000213523	*SRA1*	6.742021569	0.067603516	0.04177951	0.025824006	0.999933887	0.099474753	0.205980213	-0.10650546	0.362349628
ENSG00000149577	*SIDT2*	4.441469327	0.057514336	0.007112512	0.050401824	0.999933887	0.042319958	-0.049900539	0.092220497	0.362529395
ENSG00000245156	*AP001107.1*	0.69864762	-0.158672699	0.049826859	-0.208499558	0.999933887	-0.234658453	0.034422707	-0.26908116	0.362867594
ENSG00000101247	*NDUFAF5*	1.706433352	-0.023735327	0.063145076	-0.086880403	0.999933887	-0.021586654	-0.243762442	0.222175787	0.362867594
ENSG00000155849	*ELMO1*	5.404428118	0.031447949	0.008523961	0.022923987	0.999933887	-0.034247043	-0.116973728	0.082726685	0.362919482
ENSG00000112081	*SRSF3*	7.863614024	0.057426822	0.080143911	-0.022717089	0.999933887	0.536205132	0.65259501	-0.116389877	0.363074282
ENSG00000253552	*HOXA-AS2*	-1.088530908	-0.060783543	-0.066613386	0.005829843	0.999933887	-0.803093361	-1.429317111	0.626223751	0.363337606
ENSG00000106636	*YKT6*	6.235661808	-0.003957204	-0.019258547	0.015301342	0.999933887	-0.050241971	0.045769701	-0.096011672	0.363860597
ENSG00000143252	*SDHC*	3.469185499	-0.046284174	0.015198875	-0.061483049	0.999933887	0.074956127	0.21791288	-0.142956753	0.363904868
ENSG00000109111	*SUPT6H*	8.047464078	-0.001374843	-0.023363213	0.021988369	0.999933887	0.236642383	0.324009792	-0.087367409	0.363904868

ENSG00000159256	*MORC3*	5.469482085	-0.140518157	-0.148689955	0.008171798	0.999933887	-0.603085655	-0.417152086	-0.185933569	0.363906253
ENSG00000231365	*WARS2-AS1*	0.213713671	0.019972425	0.143490882	-0.123518457	0.999933887	-0.109776491	0.200818077	-0.310594568	0.363906253
ENSG00000162139	*NEU3*	4.016449184	-0.113104665	-0.067964766	-0.045139899	0.999933887	-0.454023327	-0.586036948	0.132013622	0.363948648
ENSG00000166484	*MAPK7*	3.752722877	0.061723175	0.068301171	-0.006577996	0.999933887	-0.09518382	-0.244286157	0.149102337	0.363948648
ENSG00000100664	*EIF5*	8.535056685	-0.014393258	-0.035378461	0.020985203	0.999933887	0.382525519	0.486357975	-0.103832456	0.364242949
ENSG00000101343	*CRNKL1*	5.152064679	-0.108995097	-0.1140649	0.005069803	0.999933887	-0.201440496	-0.083225443	-0.118215053	0.364459845
ENSG00000179148	*ALOXE3*	-0.978657269	-0.129851249	0.376594288	-0.506445537	0.999933887	0.442018914	0.778390463	-0.336371548	0.364459845
ENSG00000176476	*SGF29*	3.117667057	-0.065579328	-0.020119913	-0.045459415	0.999933887	-0.276550574	-0.425380487	0.148829914	0.364527732
ENSG00000103249	*CLCN7*	8.368751915	0.050797438	0.066755248	-0.01595781	0.999933887	0.254225687	0.382386081	-0.128160394	0.364710384
ENSG00000116560	*SFPQ*	7.668325219	-0.023515009	-0.010376533	-0.013138475	0.999933887	0.110047322	0.185558612	-0.07551129	0.364710384
ENSG00000127463	*EMC1*	3.106484377	-0.010414883	0.025706233	-0.036121116	0.999933887	0.153439934	0.31503612	-0.161596187	0.364710384
ENSG00000211460	*TSN*	3.739625819	-0.063760145	-0.048572056	-0.015188089	0.999933887	-0.109093596	-0.232580928	0.123487331	0.364762608
ENSG00000120093	*HOXB3*	-0.559439483	-0.169950739	0.032544071	-0.20249481	0.999933887	-0.415859473	-0.014503392	-0.401356081	0.364845737
ENSG00000235192	*AC009495.3*	0.198212184	0.074141457	-0.115098297	0.189239754	0.999933887	0.103259745	-0.288857429	0.392117174	0.364845737
ENSG00000253720	*AC022973.3*	3.639832241	-0.060712192	0.101844457	-0.162556648	0.999933887	-0.085352347	0.198098505	-0.283450853	0.364845737
ENSG00000249884	*RNF103-CHMI*	2.211071345	-0.014000303	-0.065259124	0.05125882	0.999933887	0.085364715	-0.096678012	0.182042727	0.364845737
ENSG00000148399	*DPH7*	2.725876704	0.000287412	0.008703637	-0.008416224	0.999933887	0.083681087	-0.07679434	0.160475427	0.364845737
ENSG00000068796	*KIF2A*	3.987042588	-0.106116571	-0.083486306	-0.022630265	0.999933887	-0.388965726	-0.229509885	-0.15945584	0.364940828
ENSG00000245748	*AC097382.2*	0.173711045	-0.083544646	-0.208863289	0.125318643	0.999933887	-0.583300721	-1.02254509	0.439244369	0.364940828
ENSG00000076356	*PLXNA2*	2.483909402	0.03779273	0.005074516	0.032718215	0.999933887	0.047305782	-0.136554563	0.183860345	0.365004335
ENSG00000138119	*MYOF*	1.365054692	-0.021010531	0.068911495	-0.089922026	0.999933887	-0.260181515	-0.026488028	-0.233693487	0.365292603
ENSG00000143751	*SDE2*	6.353893466	0.089687904	0.126668786	-0.036980882	0.999933887	-0.71506658	-0.598336615	-0.116729965	0.365540825
ENSG00000162368	*CMPK1*	4.553337222	-0.062609556	0.065267378	-0.127876934	0.999933887	-0.071521989	0.077092512	-0.148614502	0.365694385
ENSG00000166233	*ARIH1*	7.192679767	-0.039530419	-0.066059066	0.026528647	0.999933887	0.148224614	0.247596278	-0.099371663	0.365694385
ENSG00000139637	*MYG1*	2.7115491	0.035234375	-0.064912087	0.100146462	0.999933887	-0.328841253	-0.496511606	0.167670353	0.365694385
ENSG00000239300	*AC080162.1*	0.761365287	-0.025599717	0.093012998	-0.118612715	0.999933887	0.922383532	1.186393154	-0.264009622	0.365694385
ENSG00000198919	*DZIP3*	1.711805516	-0.066459902	-0.268267714	0.201807812	0.999933887	0.015397191	0.347619789	-0.332222598	0.365718922
ENSG00000147144	*CCDC120*	1.858412269	-0.059768726	0.039640343	-0.099409069	0.999933887	0.009542925	-0.174637104	0.18418003	0.36581505
ENSG00000237037	*NDUFA6-DT*	0.226532146	0.0927844	-0.042885067	0.135669467	0.999933887	-0.010335361	0.315928327	-0.326263687	0.36581505
ENSG00000148346	*LCN2*	0.940134419	-0.111972622	0.070117283	-0.182089904	0.999933887	0.826796766	1.088856904	-0.262060139	0.366828627
ENSG00000136710	*CCDC115*	3.512716487	0.05167643	-0.020261004	0.071937435	0.999933887	0.073430284	-0.067109691	0.140539975	0.366947919
ENSG00000274561	*AC005332.3*	0.498350864	0.039722599	-0.310926676	0.350649276	0.999933887	-0.302365778	-0.583853277	0.281487499	0.367068542
ENSG00000260908	*AC009093.3*	-0.267204477	0.092876251	-0.068486257	0.161362508	0.999933887	-0.098332008	0.252760043	-0.351092051	0.367615392
ENSG00000105501	*SIGLEC5*	3.903317228	0.064358789	0.024318833	0.040039956	0.999933887	0.726796412	0.579532615	0.147263797	0.367993007
ENSG00000051825	*MPHOSPH9*	2.563549138	0.011476115	-0.142735348	0.154211463	0.999933887	-0.272802041	-0.016373845	-0.256428196	0.367993007
ENSG00000101150	*TPD52L2*	9.48797349	-0.034782345	-0.050756454	0.015974109	0.999933887	-0.105595922	-0.016792402	-0.08880352	0.368118952
ENSG00000100201	*DDX17*	9.787003508	-0.060647175	-0.054393844	-0.006253331	0.999933887	-0.142450876	-0.080764009	-0.061686867	0.368381894
ENSG00000277969	*AC006449.6*	4.368803151	0.157386804	0.110212489	0.047174314	0.999933887	2.153858215	2.340643443	-0.186785228	0.368381894
ENSG00000123552	*USP45*	0.683454735	-0.210419605	-0.345099133	0.134679528	0.999933887	-0.210905656	0.193636305	-0.404541961	0.368381894
ENSG00000185798	*WDR53*	1.491238655	0.0452173	-0.003384555	0.048601855	0.999933887	-0.02214203	-0.226057035	0.203915005	0.368381894
ENSG00000174007	*CEP19*	3.910493899	-0.025570049	0.045239313	-0.070809362	0.999933887	-0.714084537	-0.899884345	0.185799807	0.368381894
ENSG00000262777	*AC032044.1*	1.023150279	-0.001175054	0.121028448	-0.122203502	0.999933887	-0.201966822	0.158766051	-0.360732873	0.368381894
ENSG00000064313	*TAF2*	4.213699883	-4.4986e-05	-0.069304894	0.069259907	0.999933887	-0.05739552	0.122088246	-0.179483766	0.368381894
ENSG00000125449	*ARMC7*	4.170606945	-0.005634587	-0.131602068	0.125967481	0.999933887	-0.437372958	-0.570575973	0.133203015	0.368426058
ENSG00000152223	*EPG5*	7.057133794	-0.055738766	-0.011752175	-0.043986591	0.999933887	0.438320252	0.556378727	-0.118058475	0.368450453
ENSG00000283959	*AP002851.1*	-0.55527548	-0.21857555	-0.846347385	0.627771835	0.999933887	2.11624527	1.762232205	0.354013065	0.368450453
ENSG00000108840	*HDAC5*	7.823910508	-0.001456307	0.021145381	-0.022601688	0.999933887	-0.340331396	-0.436009098	0.095677702	0.368591693
ENSG00000137055	*PLAA*	4.512938918	-0.035769743	-0.067457669	0.031687926	0.999933887	-0.063226786	0.036252049	-0.099478836	0.369028409
ENSG00000197180	*CH17-340M24*	0.513830333	-0.340069567	-0.240384903	-0.099684664	0.999933887	0.389753286	0.628812001	-0.239058715	0.369099729
ENSG00000182473	*EXOC7*	5.253924349	0.072005518	-0.042313964	0.114319482	0.999933887	-0.091875887	-0.192249754	0.100373867	0.369795094
ENSG00000101460	*MAP1LC3A*	6.36211131	0.074233739	0.095400031	-0.021166292	0.999933887	1.127306007	1.268703512	-0.141397505	0.369877318
ENSG00000268154	*Metazoa_SRP*	-0.239224878	-0.063048655	-0.124337609	0.061288954	0.999933887	-0.51833434	-0.129489609	-0.388844732	0.369877318
ENSG00000167549	*CORO6*	0.551345291	-0.066817596	0.255092691	-0.321910287	0.999933887	0.011170746	-0.28035401	0.291524755	0.369877318
ENSG00000148335	*NTMT1*	2.188645246	-0.021595686	0.055407221	-0.077002906	0.999933887	0.213414705	-0.005990317	0.219405022	0.369877318
ENSG00000100104	*SRRD*	2.176876129	-0.012643655	-0.054296939	0.041653284	0.999933887	-0.017064563	0.187500031	-0.204564593	0.369877318
ENSG00000244165	*P2RY11*	0.593884999	0.022022967	0.010365695	0.011657271	0.999933887	0.125941369	-0.200135017	0.326076386	0.370040162
ENSG00000145545	*SRD5A1*	0.658931541	-0.080663703	0.158552316	-0.239216019	0.999933887	-0.162411692	0.15100481	-0.313416501	0.370143087
ENSG00000287974	*Z84486.1*	-0.539504172	-0.19717829	-0.336185462	0.139007171	0.999933887	-0.018823516	0.306324406	-0.325147921	0.37021375
ENSG00000141469	*SLC14A1*	0.677726222	-0.147398368	0.020686048	-0.168084416	0.999933887	-0.008353537	0.245181413	-0.25353495	0.370595106
ENSG00000272282	*LINC02084*	0.840063038	-0.273219037	-0.09486794	-0.178351097	0.999933887	0.082981139	0.325189508	-0.242208369	0.370734274
ENSG00000124091	*GCNT7*	2.993760338	0.124773369	0.148893572	-0.024120204	0.999933887	-0.030070699	0.183648944	-0.213719643	0.370734274
ENSG00000137822	*TUBGCP4*	1.928927164	0.077248286	0.154569101	-0.077320815	0.999933887	-0.006788255	0.178892808	-0.185681063	0.370734274
ENSG00000285967	*NIPBL-DT*	3.46345297	-0.072732672	-0.061291506	-0.011441166	0.999933887	-0.405536175	-0.556062197	0.150526022	0.370734274
ENSG00000132963	*POMP*	4.900097725	-0.018915761	-0.058641937	0.039726176	0.999933887	0.12937169	0.227908959	-0.098537269	0.370734274
ENSG00000179832	*MROH1*	4.919609217	0.006657	0.013948916	-0.007291916	0.999933887	-0.154736145	-0.261224788	0.106488643	0.370734274
ENSG00000196338	*NLGN3*	4.140438326	0.094009698	-0.091310927	0.185320625	0.981460244	0.499772189	0.630152827	-0.130380638	0.370794933
ENSG00000177646	*ACAD9*	2.859261656	-0.042693046	0.136619445	-0.179312492	0.999933887	-0.097590179	-0.226689548	0.12909937	0.371267928
ENSG00000107554	*DNMBP*	4.198786706	-0.107302706	-0.012397307	-0.094905399	0.999933887	-0.824112892	-0.971348856	0.147235964	0.37164658
ENSG00000249138	*SLED1*	2.257430437	0.125731172	0.128731127	-0.002999955	0.999933887	2.235949435	1.810525522	0.425423913	0.371850248
ENSG00000172922	*RNASEH2C*	4.023713011	0.091356528	0.059511046	0.031845482	0.999933887	0.173252283	0.296157768	-0.122905485	0.371956121
ENSG00000095059	*DHPS*	1.131322036	-0.061242104	0.16105992	-0.222302024	0.999933887	-0.088422655	-0.351667664	0.263245009	0.371956121
ENSG00000171476	*HOPX*	2.513463727	0.055587764	0.098111151	-0.042523387	0.999933887	0.041300112	0.205162415	-0.163862303	0.372215488
ENSG00000067836	*ROGDI*	0.553130624	0.323886909	0.05244132	0.271445588	0.999933887	0.047845459	-0.343599546	0.391445005	0.372407784
ENSG00000173077	*DELEC1*	1.215529739	0.299355582	0.602792516	-0.303436935	0.999933887	0.447940986	0.708464918	-0.260523933	0.372407784
ENSG00000065057	*NTHL1*	-0.424926111	0.189827882	-0.00982622	0.199654101	0.999933887	0.235045573	-0.205709034	0.440754607	0.372407784
ENSG00000107949	*BCCIP*	2.37646899	-0.160315022	-0.081095042	-0.07921998	0.999933887	0.005301557	0.19992387	-0.194622312	0.372798759
ENSG00000273340	*MICE*	-0.816529719	0.271688668	-0.126233249	0.397921916	0.999933887	-0.337013465	0.104942662	-0.441956127	0.373125755
ENSG00000106113	*CRHR2*	-0.664702178	0.125723365	0.21793021	-0.092206845	0.999933887	0.496334828	0.076646406	0.419688422	0.373125755
ENSG00000123213	*NLN*	4.576596684	-0.034205582	0.015164003	-0.049369586	0.999933887	-0.10303822	0.027005987	-0.130044207	0.373379262
ENSG00000160445	*ZER1*	5.588116733	-0.029708047	-0.025833001	-0.003875046	0.999933887	-0.53300639	-0.643976473	0.110970083	0.373440892
ENSG00000114942	*EEF1B2*	6.160099324	-0.00578463	-0.056269301	0.050484671	0.999933887	0.109229487	0.223757939	-0.114528453	0.373700145
ENSG00000166529	*ZSCAN21*	1.033910891	0.035977367	-0.283677702	0.319655069	0.999933887	0.387408259	0.698929544	-0.311521286	0.373982971
ENSG00000116489	*CAPZA1*	8.246678056	-0.090879542	-0.062448548	-0.028430994	0.999933887	-0.194895944	-0.088140422	-0.106755522	0.374119562
ENSG00000196605	*ZNF846*	0.059893101	-0.172702522	-0.200228491	0.027525969	0.999933887	-0.144729239	0.143423449	-0.288152688	0.374119562
ENSG00000160218	*TRAPPC10*	6.174930517	-0.027556088	0.02658477	-0.054140858	0.999933887	-0.08096723	-0.004748724	-0.076218505	0.374172342
ENSG00000183340	*JRKL*	2.77374686	-0.045845437	0.068541334	-0.114386771	0.999933887	1.000544914	1.218124993	-0.217580079	0.374172342
ENSG00000223534	*HLA-DQB1-AS*	-0.331917848	-0.002466037	0.417829963	-0.420296	0.999933887	1.497090189	1.963914676	-0.466824487	0.374269278
ENSG00000269343	*ZNF587B*	2.824033083	0.021292177	0.009806596	0.011485581	0.999933887	0.024718117	-0.148884353	0.17360247	0.374428041
ENSG00000204580	*DDR1*	0.361503295	0.056090212	-0.274976378	0.33106659	0.999933887	0.280931732	0.541579172	-0.26064744	0.374779213
ENSG00000236349	*SUCLG2P2*	-0.909910089	0.156993399	0.268678448	-0.11168505	0.999933887	-0.701486857	0.025989134	-0.727475991	0.374889026
ENSG00000140320	*BAHD1*	3.940762052	0.039642831	-0.011727742	0.051370573	0.999933887	-0.057894523	-0.177705304	0.11981078	0.374889026
ENSG00000143198	*MGST3*	2.188262771	0.065338861	-0.009134744	0.074473605	0.999933887	0.097719882	-0.112912066	0.210631948	0.375021046
ENSG00000138735	*PDE5A*	0.572978855	0.006078248	-0.020340539	0.026418787	0.999933887	-0.44385956	0.040903341	-0.4847629	0.375298107
ENSG00000234975	*FTH1P2*	4.100635736	-0.029650447	0.010966521	-0.040616969	0.999933887	0.5418545	0.702328298	-0.160473797	0.375485899
ENSG00000014257	*ACP3*	3.466879421	-0.082559805	0.049077123	-0.131636928	0.999933887	-0.36456193	-0.646071522	0.281509592	0.375586193
ENSG00000163534	*FCRL1*	1.076574914	0.011578103	0.067886773	-0.056308669	0.999933887	-0.12209811	-0.374882941	0.252784831	0.375749598
ENSG00000005884	*ITGA3*	0.610749712	-0.014233097	0.040380178	-0.054613275	0.999933887	0.054029087	0.369354742	-0.315325655	0.376195433
ENSG00000109113	*RAB34*	0.383627074	0.161946189	-0.082716578	0.244662768	0.999933887	0.294809401	-0.018154609	0.31296401	0.376276728
ENSG00000073711	*PPP2R3A*	-0.045484615	-0.183941562	-0.120758963	-0.063182599	0.999933887	-0.56574603	-0.119582715	-0.446163315	0.376899573
ENSG00000152332	*UHMK1*	5.389817851	0.05161173	-0.009231916	0.060843645	0.999933887	-0.329155875	-0.181668707	-0.147487168	0.376899573
ENSG00000150938	*CRIM1*	2.37914164	0.050996413	0.150095153	-0.09909874	0.999933887	0.157358102	0.31913981	-0.161781708	0.376899573
ENSG00000147443	*DOK2*	4.713165953	0.060239557	0.000953122	0.059286436	0.999933887	-0.065822539	-0.189935341	0.124112802	0.376972341
ENSG00000113732	*ATP6V0E1*	8.090158561	-0.003658517	-0.060471099	0.056812582	0.999933887	0.066179434	0.163979936	-0.097800502	0.376974037
ENSG00000166839	*ANKDD1A*	4.454321501	-0.047218641	-0.109410041	0.0621914	0.999933887	0.380969387	0.552529139	-0.171559752	0.377198657
ENSG00000179344	*HLA-DQB1*	5.351165141	-0.0738818	0.046361135	-0.120242936	0.999933887	1.182614356	1.466902111	-0.284287755	0.377536172

ENSG00000114503	*NCBP2*	3.6993436	-0.060041361	0.000490683	-0.060532044	0.999933887	0.051908714	0.161987953	-0.110079239	0.377795701
ENSG00000171291	*ZNF439*	0.597094643	-0.079471236	0.12796122	-0.207432456	0.999933887	-0.283591006	-0.605233802	0.321642795	0.377795701
ENSG00000124275	*MTRR*	3.158104122	-0.129551424	-0.165464664	0.035913241	0.999933887	0.015063765	0.147233642	-0.132169877	0.378035441
ENSG00000104812	*GYS1*	3.41949693	0.05499024	-0.031636326	0.086626566	0.999933887	0.11109698	-0.011481421	0.122578401	0.378035441
ENSG00000272419	*LINC01145*	1.418442993	-0.000690544	-0.170704014	0.17001347	0.999933887	-0.134930093	-0.391558607	0.256628514	0.378035441
ENSG00000267796	*LIN37*	1.039713059	-0.116709479	0.126473452	-0.243182931	0.999933887	-0.506719416	-0.759563961	0.252844544	0.378269623
ENSG00000116584	*ARHGEF2*	7.724243173	0.002873438	0.018783891	-0.015910454	0.999933887	0.302560114	0.395795056	-0.093234942	0.378269623
ENSG00000134294	*SLC38A2*	7.394747794	-0.035171989	-0.085637359	0.05046537	0.999933887	-0.480473853	-0.298212752	-0.182261102	0.37854352
ENSG00000006327	*TNFRSF12A*	3.225359528	-0.102892072	-0.161232949	0.058340877	0.999933887	-1.643643547	-1.954847409	0.311203862	0.378550461
ENSG00000178980	*SELENOW*	3.292866181	0.056629476	0.014943391	0.041686085	0.999933887	0.216872977	0.052256419	0.164616558	0.378699312
ENSG00000166908	*PIP4K2C*	5.295106153	-0.085580772	0.022063155	-0.107643927	0.999933887	-0.516446857	-0.616997797	0.100550941	0.378853753
ENSG00000005893	*LAMP2*	8.665768927	-0.004552788	-0.053196975	0.048644187	0.999933887	0.011844566	0.096331836	-0.08448727	0.378917794
ENSG00000107816	*LZTS2*	1.705599241	0.144016989	0.040788503	0.103228487	0.999933887	-0.007944771	-0.215950776	0.208006005	0.379294838
ENSG00000197081	*IGF2R*	11.01422367	-0.028434258	-0.003597299	-0.024836959	0.999933887	-0.197073138	-0.11142838	-0.085644757	0.379294838
ENSG00000083828	*ZNF586*	4.437113287	-0.079517113	-0.068225191	-0.011291922	0.999933887	-0.280968216	-0.392004919	0.111036702	0.379345946
ENSG00000125454	*SLC25A19*	0.736690482	0.189057027	-0.024225594	0.213282621	0.999933887	0.09104383	-0.174620628	0.265664458	0.379345946
ENSG00000124104	*SNX21*	-0.270859692	0.168447846	-0.043179416	0.211627262	0.999933887	-0.215653991	-0.575287331	0.359633341	0.37935159
ENSG00000100528	*CNIH1*	2.53865695	-0.056750867	-0.065716808	0.008965941	0.999933887	-0.098140826	0.062936626	-0.161077451	0.37935159
ENSG00000247925	*AL139807.1*	0.858850205	-0.091848658	0.40265906	-0.494507718	0.944094537	-0.104917666	0.271795859	-0.376713526	0.37935159
ENSG00000104885	*DOT1L*	6.940421428	0.036643413	-0.009324892	0.045968305	0.999933887	1.56724835	1.784352272	-0.217103922	0.37935159
ENSG00000103313	*MEFV*	7.446818147	-0.038381309	0.017267778	-0.055649087	0.999933887	-0.029295517	-0.180725125	0.151429608	0.379402196
ENSG00000204386	*NEU1*	7.767864153	0.096946745	0.049268347	0.047678398	0.999933887	0.298189931	0.406268465	-0.108078534	0.379531709
ENSG00000078098	*FAP*	-1.174151871	-0.493735099	-0.596786423	0.103051325	0.999933887	0.800126832	1.201035813	-0.400908981	0.379590599
ENSG00000177830	*CHID1*	1.720491345	-0.001151132	-0.147368363	0.14621723	0.999933887	0.056848607	-0.160532186	0.217380793	0.379802765
ENSG00000129292	*PHF20L1*	6.857645857	-0.03787017	-0.035121688	-0.002748481	0.999933887	-0.241184373	-0.097141385	-0.144042988	0.379894157
ENSG00000262714	*AC007342.5*	1.316421687	-0.038689849	0.028026603	-0.066716453	0.999933887	-1.272340439	-1.73047583	0.458135391	0.379972053
ENSG00000259398	*AC090970.1*	-0.372334467	-0.038359207	0.349365917	-0.387725124	0.999933887	1.038287797	1.526746164	-0.488458367	0.380188829
ENSG00000143164	*DCAF6*	3.411384722	0.008196	-0.213332063	0.221528063	0.999291887	-0.446506252	-0.615785179	0.169278928	0.380347662
ENSG00000157890	*MEGF11*	0.719603768	-0.155368909	-0.032936656	-0.122432254	0.999933887	0.680135512	0.908600627	-0.228465115	0.38044301
ENSG00000197386	*HTT*	4.879418027	0.029504681	0.052791643	-0.023286962	0.999933887	0.057398561	-0.041129222	0.098527784	0.38044301
ENSG00000158717	*RNF166*	6.90614422	0.053435582	0.032354283	0.021081299	0.999933887	-0.084959978	-0.190769752	0.105809774	0.38060018
ENSG00000135473	*PAN2*	3.38072249	-0.03421153	0.030811634	-0.065023164	0.999933887	0.26736312	0.42044701	-0.15308389	0.380704108
ENSG00000189420	*ZFP92*	2.417525829	-0.050216324	-0.201946491	0.151730167	0.999933887	-0.971901644	-1.168368905	0.196467261	0.380718642
ENSG00000103540	*CCP110*	2.142730556	-0.044484602	-0.061049882	0.01656528	0.999933887	-0.416757767	-0.058710436	-0.358047331	0.380718642
ENSG00000151239	*TWF1*	3.464165263	-0.034072646	-0.186138454	0.152065808	0.999933887	-0.536807069	-0.324442723	-0.212364347	0.380777679
ENSG00000105607	*GCDH*	1.983243793	0.030672509	-0.045373673	0.076046182	0.999933887	0.094715785	-0.09568521	0.190400995	0.380777679
ENSG00000122565	*CBX3*	5.787085993	-0.046973836	-0.114375841	0.067402005	0.999933887	-0.479959138	-0.584585931	0.104626793	0.38096682
ENSG00000072778	*ACADVL*	6.799847763	0.071405485	0.053135373	0.018270113	0.999933887	-0.171372161	-0.280579495	0.109207334	0.381027968
ENSG00000269743	*SLC25A53*	2.70028341	-0.073792198	0.122008587	-0.195800785	0.999933887	-0.075023246	0.052541451	-0.127564697	0.381071298
ENSG00000242960	*FTH1P23*	0.262732854	-0.059272354	-0.341528149	0.282255795	0.999933887	0.396849768	0.670544485	-0.273694716	0.381071298
ENSG00000104763	*ASAH1*	10.305378173	-0.076675857	-0.079284477	0.00260862	0.999933887	-0.24100979	-0.155568831	-0.085440959	0.38115011
ENSG00000149016	*TUT1*	1.110456634	-0.188367653	0.140112044	-0.328479697	0.999933887	0.019790605	-0.215492774	0.235283378	0.381207458
ENSG00000092068	*SLC7A8*	-0.698943028	-0.094403739	0.168889709	-0.263293448	0.999933887	-0.604992088	-0.111010749	-0.493981339	0.381207458
ENSG00000058091	*CDK14*	4.231312167	-0.000722575	0.007499272	-0.008221846	0.999933887	0.419949057	0.269052102	0.150896955	0.381207458
ENSG00000211810	*TRAV29DV5*	-0.706489094	0.063688157	0.179116063	-0.115427906	0.999933887	-0.124262899	0.29649444	-0.420757339	0.381234535
ENSG00000231794	*AC009542.1*	2.026779168	0.076929308	0.108675957	-0.031746649	0.999933887	-0.190898678	0.081445804	-0.272344482	0.381868101
ENSG00000173542	*MOB1B*	1.559615076	-0.058834734	-0.150967069	0.092132335	0.999933887	-0.331049311	-0.025499353	-0.305549958	0.381983074
ENSG00000273148	*LINC00653*	0.313817549	-0.223799798	-0.079027547	-0.144772251	0.999933887	0.072902415	0.406435334	-0.333532919	0.382091206
ENSG00000127951	*FGL2*	6.29646366	-0.035509321	-0.156626057	0.121116736	0.999933887	-0.494042089	-0.717069181	0.223027093	0.382123807
ENSG00000171865	*RNASEH1*	2.508943219	-0.030233294	-0.146159446	0.115926152	0.999933887	-0.071789547	0.089620742	-0.161410289	0.382131174
ENSG00000102181	*CD99L2*	2.445666831	0.05451297	0.014951716	0.039561254	0.999933887	-0.028331154	-0.231388497	0.203057343	0.382171697
ENSG00000184009	*ACTG1*	10.349632366	0.044226033	0.022810069	0.021415964	0.999933887	-0.182765079	-0.281125807	0.098360728	0.382220866
ENSG00000146592	*CREB5*	5.816777582	-0.029019984	-0.025523401	-0.003496582	0.999933887	-0.935602654	-1.13845047	0.202847816	0.382258854
ENSG00000102245	*CD40LG*	1.983078327	-0.072930201	-0.208885991	0.13595579	0.999933887	0.208433518	0.412913192	-0.204479674	0.382414632
ENSG00000180357	*ZNF609*	4.431092604	0.021062097	0.035639478	-0.01457738	0.999933887	-0.029529789	0.080777304	-0.110307093	0.382414632
ENSG00000167766	*ZNF83*	3.345115819	-0.090776945	-0.049175308	-0.041601638	0.999933887	-0.142667302	0.066896525	-0.209563827	0.382441297
ENSG00000155506	*LARP1*	6.134940474	0.005278305	0.086688688	-0.081410383	0.999933887	0.001813239	0.116595338	-0.1147821	0.382441297
ENSG00000122223	*CD244*	5.133626918	-0.033470538	-0.063812895	0.030342357	0.999933887	-0.810754711	-0.652614323	-0.158140388	0.382862442
ENSG00000103932	*RPAP1*	1.986674762	0.017144659	0.034813466	-0.017668807	0.999933887	0.124258882	0.290649414	-0.166390532	0.382992475
ENSG00000243806	*RPL7P18*	0.030434381	-0.257494505	-0.035932123	-0.221562382	0.999933887	1.500037705	1.875527737	-0.375490031	0.383647413
ENSG00000259635	*AC100830.1*	0.057180124	-0.143930877	-0.063550126	-0.080380752	0.999933887	0.281295666	0.602183371	-0.320887705	0.383789656
ENSG00000139370	*SLC15A4*	8.931341969	-0.073241718	-0.035787602	-0.037454116	0.999933887	-0.265937564	-0.169097999	-0.096839565	0.384213048
ENSG00000250318	*AC003072.1*	1.117551135	-0.125125658	0.033147813	-0.158273471	0.999933887	0.107523897	-0.144579226	0.252103124	0.384213048
ENSG00000108774	*RAB5C*	6.164366881	0.011036165	-0.032953513	0.043989678	0.999933887	0.058621529	0.154876856	-0.096255327	0.384213048
ENSG00000123600	*METTL8*	0.849570795	-0.016807612	0.068966362	-0.085773974	0.999933887	-0.013922002	0.235299458	-0.249221461	0.384213048
ENSG00000123179	*EBPL*	1.460654345	0.030780455	-0.153200705	0.18398116	0.999933887	-0.331557233	-0.619568975	0.288011742	0.384282822
ENSG00000175606	*TMEM70*	4.321381871	-0.094288504	-0.067461848	-0.026826656	0.999933887	-0.867357386	-1.019890483	0.152533097	0.384393035
ENSG00000181690	*PLAG1*	-0.010813796	-0.170554963	-0.522813352	0.352258388	0.999933887	-0.158015518	0.144684193	-0.302699711	0.384393035
ENSG00000196597	*ZNF782*	0.42847924	0.083537772	0.229350018	-0.145812245	0.999933887	-0.116294678	0.128565438	-0.244860117	0.384438975
ENSG00000040341	*STAU2*	2.637115849	0.0549952	-0.172174649	0.22716985	0.999933887	-0.397340349	-0.603685985	0.206345636	0.384438975
ENSG00000129467	*ADCY4*	3.625529153	0.09024456	0.017725855	0.072518706	0.999933887	-0.14471757	-0.290951509	0.146233939	0.384562469
ENSG00000178449	*COX14*	2.509742067	-0.007312025	0.028241081	-0.035553106	0.999933887	-0.086152284	-0.267602304	0.18145002	0.384897548
ENSG00000121073	*SLC35B1*	3.69738859	0.012154708	-0.018710539	0.030865247	0.999933887	-0.032994862	-0.163339792	0.13034493	0.38502062
ENSG00000162704	*ARPC5*	8.968147536	-0.04299571	-0.05773258	0.01473687	0.999933887	-0.375744783	-0.278778147	-0.096966635	0.385170301
ENSG00000187105	*HEATR4*	0.289127706	-0.26573577	0.02318411	-0.28891988	0.999933887	0.996959231	1.260218363	-0.263259132	0.385270104
ENSG00000266498	*AC055811.3*	1.428255905	0.079925292	0.145818998	-0.065893706	0.999933887	0.538656381	0.810260979	-0.271604598	0.385270104
ENSG00000110074	*FOXRED1*	2.080912045	0.013958393	-0.070302674	0.084261067	0.999933887	0.150768713	0.335575454	-0.184806741	0.385270104
ENSG00000119917	*IFIT3*	7.926532732	-0.137513989	-0.174656998	0.037143009	0.999933887	-0.969163602	-1.133014233	0.163850631	0.38536829
ENSG00000235863	*B3GALT4*	2.839378644	-0.104223627	0.107312014	-0.211535641	0.999933887	-0.504923404	-0.660529051	0.155605648	0.38536829
ENSG00000187860	*CCDC157*	1.348021218	0.131061632	0.176967592	-0.04590596	0.999933887	0.238475609	0.505052684	-0.266577075	0.385561603
ENSG00000166228	*PCBD1*	-0.175392578	0.015991759	0.116960238	-0.100968479	0.999933887	0.219362718	-0.149084787	0.368447505	0.385561603
ENSG00000102931	*ARL2BP*	0.020919388	0.083987953	-0.277083261	0.361071214	0.999933887	0.230535478	-0.140503636	0.371039113	0.385899824
ENSG00000125347	*IRF1*	8.546380233	0.053334309	0.033129351	0.020204957	0.999933887	0.244692815	0.128179624	0.116513191	0.386006309
ENSG00000164414	*SLC35A1*	0.564880149	0.168326359	0.085050699	0.08327566	0.999933887	-0.66064849	-1.001141665	0.340493176	0.386069564
ENSG00000229331	*GK-IT1*	3.344537548	0.041777205	0.06539262	-0.023615416	0.999933887	0.284021784	0.437929307	-0.153907523	0.386186424
ENSG00000245532	*NEAT1*	11.836750613	0.127632586	0.114400182	0.013232404	0.999933887	0.345530746	0.459743591	-0.114212846	0.386532364
ENSG00000262312	*AC004494.1*	0.21082752	-0.150338181	0.127015409	-0.27735359	0.999933887	0.033029438	0.344159189	-0.311129751	0.386778177
ENSG00000213903	*LTB4R*	5.199285646	0.011132074	-0.069097883	0.080229958	0.999933887	-0.835372835	-1.021004904	0.18563207	0.386848078
ENSG00000109445	*ZNF330*	3.663224061	-0.060125798	-0.098613032	0.038487234	0.999933887	0.043059985	0.150894474	-0.107834488	0.386952481
ENSG00000142444	*TIMM29*	1.511037666	0.210066924	0.130959725	0.079107199	0.999933887	-0.019280726	-0.217375498	0.198094772	0.387049776
ENSG00000179922	*ZNF784*	1.318016984	0.137894286	0.172441289	-0.034547003	0.999933887	-0.410622048	-0.65820786	0.247585812	0.38715418
ENSG00000154589	*LY96*	5.811579667	0.002361747	0.003926606	-0.001564859	0.999933887	-0.212284051	-0.340673985	0.128389934	0.387366564
ENSG00000080561	*MID2*	-0.481097287	0.085616193	-0.10349546	0.189111653	0.999933887	-0.114376148	0.218759613	-0.333135762	0.387459659
ENSG00000204160	*ZDHHC18*	9.896033081	0.027706456	0.031117797	-0.00341134	0.999933887	0.317288395	0.402784677	-0.085496282	0.387486535
ENSG00000117335	*CD46*	8.160643245	-0.048233708	-0.106058229	0.057824521	0.999933887	-0.289415597	-0.394186582	0.104770985	0.387619602
ENSG00000105643	*ARRDC2*	7.729696806	-0.067785512	-0.055570671	-0.012214841	0.999933887	-0.201189279	-0.064140119	-0.13704916	0.387619602
ENSG00000183605	*SFXN4*	-0.177161177	-0.129556404	0.073282302	-0.202838706	0.999933887	0.285125359	-0.098529648	0.383655007	0.387619602
ENSG00000100056	*ESS2*	4.05576622	0.02637396	0.033694617	-0.007320657	0.999933887	-0.072886711	-0.211706227	0.138819516	0.387619602
ENSG00000140854	*KATNB1*	3.830718976	0.040714578	0.081858286	-0.041143708	0.999933887	-0.068826467	-0.222646021	0.153819554	0.387648165
ENSG00000263934	*SNORD3A*	0.357973119	-0.314656544	-0.769332566	0.454676022	0.999933887	0.686300218	0.110236114	0.576064104	0.387707388
ENSG00000214135	*AC132008.2*	2.181430449	-0.010984589	-0.003631624	-0.007352966	0.999933887	-0.221263167	-0.401445358	0.180182191	0.387789111
ENSG00000272636	*DOC2B*	-0.351155701	0.160026136	0.265851097	-0.105824961	0.999933887	1.175065644	1.718324656	-0.543259012	0.387934232
ENSG00000109929	*SC5D*	2.54086778	-0.11238772	-0.067479462	-0.044908259	0.999933887	-0.187087627	-0.004039483	-0.183048145	0.388056535
ENSG00000236383	*CCDC200*	0.336525355	-0.050809337	0.078184593	-0.128993929	0.999933887	0.433410686	0.779285135	-0.345874449	0.388056535
ENSG00000163605	*PPP4R2*	6.996170102	-0.08816864	-0.057767775	-0.030400865	0.999933887	0.404226805	0.571391186	-0.167164381	0.388211862

ENSG00000012048	*BRCA1*	2.110855657	-0.045712183	-0.110438776	0.064726592	0.999933887	-0.301000293	-0.04048232	-0.260517973	0.388211862
ENSG00000100379	*KCTD17*	1.498883114	0.006799007	-0.041877879	0.048676886	0.999933887	0.28700348	0.090132607	0.196870873	0.388211862
ENSG00000110435	*PDHX*	1.454710354	-0.146791474	-0.214700103	0.067908629	0.999933887	0.13581347	0.329082951	-0.19326948	0.388818478
ENSG00000104687	*GSR*	6.106962076	-0.063798355	0.022360049	-0.086158404	0.999933887	-0.304930553	-0.422028088	0.117097535	0.388931669
ENSG00000133606	*MKRN1*	7.203557322	-0.073188351	-0.082871284	0.009682933	0.999933887	-0.568590404	-0.679148043	0.110557639	0.388944861
ENSG00000246082	*NUDT16P1*	-1.619843875	0.155818744	0.117833122	0.037985622	0.999933887	-0.726014904	-1.411398496	0.685383592	0.38898874
ENSG00000267141	*AC012615.4*	-1.336924417	-0.113774703	-0.168666608	0.054891905	0.999933887	-0.485661358	-1.17214686	0.686485502	0.38898874
ENSG00000258439	*AC007956.2*	0.616789807	-0.111149822	0.161960032	-0.273109854	0.999933887	0.098281286	0.421732928	-0.323451641	0.38907558
ENSG00000139970	*RTN1*	1.005769503	0.101716472	0.191595159	-0.089878687	0.999933887	0.071653285	-0.285400587	0.357053872	0.38907558
ENSG00000228594	*FNDC10*	0.41275377	0.156168373	-0.213161058	0.369329432	0.999933887	-0.345421309	-0.628408976	0.282987666	0.389083283
ENSG00000150477	*KIAA1328*	0.400992093	-0.028898228	-0.133967492	0.105069263	0.999933887	-0.296941273	0.034943479	-0.331884752	0.389445218
ENSG00000173692	*PSMD1*	4.674412882	-0.082197744	-0.129264585	0.047066841	0.999933887	0.015970865	0.122462195	-0.106491329	0.389534719
ENSG00000153207	*AHCTF1*	6.574048305	-0.100359474	-0.209935049	0.109575575	0.999933887	-1.582010737	-1.869997823	0.287987086	0.389534719
ENSG00000107614	*TRDMT1*	2.02686489	0.039161921	-0.249113057	0.288274978	0.944094537	-0.257236649	-0.05521781	-0.202018839	0.389534719
ENSG00000122566	*HNRNPA2B1*	9.014550548	-0.02276689	-0.021833354	-0.000933536	0.999933887	0.154671553	0.258558435	-0.103886882	0.389534719
ENSG00000036672	*USP2*	0.943335283	0.037678269	0.112105281	-0.074427012	0.999933887	-0.049176099	0.320480035	-0.369656134	0.389534719
ENSG00000092208	*GEMIN2*	-0.417369299	0.059106751	0.267024757	-0.207918007	0.999933887	-0.058453925	0.318741362	-0.377195287	0.389534719
ENSG00000123700	*KCNJ2*	5.653375357	0.000933634	0.102163454	-0.10122982	0.999933887	3.305401548	3.643217397	-0.337815849	0.389534719
ENSG00000069849	*ATP1B3*	4.421520609	-0.042015723	-0.08437172	0.042355997	0.999933887	0.096904678	0.21507568	-0.118171003	0.389856611
ENSG00000189046	*ALKBH2*	1.928903219	0.007232239	0.027569	-0.020336761	0.999933887	0.442640279	0.159025518	0.283614761	0.390077135
ENSG00000211893	*IGHG2*	0.750101257	-0.102772025	-0.109759124	0.006987099	0.999933887	0.248781616	0.714277099	-0.465495483	0.390097339
ENSG00000214193	*SH3D21*	7.00155958	0.021569494	0.02093194	0.000637553	0.999933887	0.265279173	0.376378357	-0.111099184	0.390097339
ENSG00000077348	*EXOSC5*	0.61377402	-0.113577917	0.049054383	-0.1626323	0.999933887	0.136468462	-0.108633728	0.24510219	0.390327519
ENSG00000198700	*IPO9*	3.836378056	-0.059692258	-0.009479567	-0.050212691	0.999933887	-0.233314985	-0.340390401	0.107075416	0.390611284
ENSG00000183520	*UTP11*	1.846018203	-0.1126591	-0.161316111	0.048657011	0.999933887	-0.198024364	-0.058166123	-0.139858241	0.390629413
ENSG00000119862	*LGALSL*	3.962119735	-0.063794913	0.016379819	-0.080174732	0.999933887	-0.850068832	-1.00025845	0.150189617	0.390629413
ENSG00000135077	*HAVCR2*	2.659146536	-0.162034292	-0.098418376	-0.063615916	0.999933887	-0.499253063	-0.670843064	0.171590001	0.390975964
ENSG00000111727	*HCFC2*	1.847466941	-0.070314875	-0.272491675	0.2021768	0.999933887	0.452631822	0.702481	-0.249849178	0.39103307
ENSG00000110700	*RPS13*	6.587906868	-0.015936597	-0.032311214	0.016374617	0.999933887	0.143146404	0.249436602	-0.106290199	0.39103307
ENSG00000110203	*FOLR3*	4.947869815	0.059947874	0.017069637	0.042878237	0.999933887	0.0013743	-0.161004474	0.162378774	0.391159837
ENSG00000111684	*LPCAT3*	2.401571196	0.06306674	-0.015549925	0.078616665	0.999933887	-0.033804458	0.195471661	-0.229276119	0.391159837
ENSG00000165943	*MOAP1*	5.492832066	0.046880505	0.047080559	-0.000200054	0.999933887	0.294695347	0.167545382	0.127149965	0.391159837
ENSG00000279789	*AC120114.3*	1.23718092	-0.103798986	-0.337239899	0.233440913	0.999933887	0.019696467	0.27338858	-0.253692113	0.391159837
ENSG00000204577	*LILRB3*	8.720109075	0.022450544	0.020608645	0.001841899	0.999933887	-0.398173778	-0.31167063	-0.086503148	0.391159837
ENSG00000103275	*UBE2I*	5.539406869	-0.018140513	-0.030304038	0.012163525	0.999933887	0.144520726	0.221280315	-0.076759588	0.391159837
ENSG00000132635	*PCED1A*	3.495743674	0.025661447	0.036747806	-0.011086359	0.999933887	0.100426171	-0.013058557	0.113484728	0.391159837
ENSG00000237356	*AL365295.1*	-0.446897804	-0.134093372	0.030133106	-0.164226479	0.999933887	0.201750216	0.549873922	-0.348123706	0.391159837
ENSG00000118997	*DNAH7*	-0.296413828	-0.058857622	-0.073616051	0.014758429	0.999933887	-0.4766272	0.05627566	-0.53290286	0.391159837
ENSG00000177688	*SUMO4*	1.239798118	0.034337623	0.148804237	-0.114466613	0.999933887	0.728157926	1.008989347	-0.280831421	0.391159837
ENSG00000096093	*EFHC1*	2.289571592	0.023288904	-0.037246292	0.060535196	0.999933887	0.110030547	0.327504394	-0.217473847	0.391159837
ENSG00000133317	*LGALS12*	2.956018672	0.008784368	0.049287394	-0.040503026	0.999933887	0.028017773	0.22528436	-0.197266588	0.391159837
ENSG00000141002	*TCF25*	7.172767071	0.025235507	0.012459796	0.01277571	0.999933887	0.026426246	0.105181545	-0.078755299	0.391172484
ENSG00000266378	*AC005224.2*	-1.020695862	0.112786487	0.246337444	-0.133550957	0.999933887	1.423820742	2.108928279	-0.685107536	0.391252784
ENSG00000236409	*NRADDP*	-0.067692715	-0.042511576	0.059091335	-0.101602911	0.999933887	0.408305703	0.794929393	-0.386623689	0.391252784
ENSG00000142188	*TMEM50B*	3.317359979	-0.064078722	-0.059384196	-0.004694526	0.999933887	0.123783912	0.250431739	-0.126647827	0.391375525
ENSG00000108733	*PEX12*	1.1468614	-0.243115924	-0.088336592	-0.154779332	0.999933887	-0.238433815	-0.442328821	0.203895006	0.391496613
ENSG00000214279	*SCART1*	0.768384304	0.092754988	0.268861387	-0.176106399	0.999933887	0.015436483	0.262755344	-0.247318861	0.391496613
ENSG00000224080	*UBE2FP1*	0.065950991	-0.075780104	0.10596344	-0.181743544	0.999933887	0.172395929	0.632556249	-0.460160319	0.391635957
ENSG00000248712	*CCDC153*	0.726207416	-0.114978144	0.05523489	-0.170213034	0.999933887	-0.229560079	-0.649220857	0.419660779	0.391838052
ENSG00000280721	*LINC01943*	-0.653900346	-0.350052952	0.056206481	-0.406259433	0.999933887	-0.081418987	-0.48959587	0.408176883	0.39184136
ENSG00000196693	*ZNF33B*	1.821640278	-0.088149164	-0.269205357	0.181056193	0.999933887	-0.250928357	0.035678241	-0.286606598	0.391883245
ENSG00000103253	*HAGHL*	1.329284266	0.117667742	0.261362388	-0.143694646	0.999933887	0.103468172	-0.124180521	0.227648693	0.392333752
ENSG00000088538	*DOCK3*	-0.044260941	0.009281791	-0.141218214	0.150500006	0.999933887	0.279290028	-0.048460782	0.32775081	0.392358837
ENSG00000117859	*OSBPL9*	5.622956163	-0.050918442	-0.112540385	0.061621943	0.999933887	-0.124557435	0.024908569	-0.149466004	0.392463399
ENSG00000268027	*AC243960.1*	1.324412473	-0.044741428	0.027147227	-0.071888655	0.999933887	0.089554389	-0.131540663	0.221095052	0.3926314
ENSG00000126524	*SBDS*	4.384686062	-0.001089319	-0.003199505	0.002110187	0.999933887	0.123271146	0.241394521	-0.118123376	0.393025405
ENSG00000175567	*UCP2*	5.593533885	-0.015925933	-0.047902122	0.03197619	0.999933887	0.065457333	-0.042959627	0.108416959	0.393149761
ENSG00000099326	*MZF1*	4.028085084	0.059197442	0.031607176	0.027590266	0.999933887	-0.294085563	-0.403702063	0.1096165	0.393191988
ENSG00000145649	*GZMA*	3.707372663	0.025229851	-0.089389501	0.114619353	0.999933887	0.0602235	0.208178546	-0.147955046	0.393210415
ENSG00000119402	*FBXW2*	4.737839111	-0.084764374	-0.031422361	-0.053342013	0.999933887	0.080744108	0.190924252	-0.110180143	0.393387483
ENSG00000167635	*ZNF146*	3.058040887	-0.080398616	-0.236178459	0.155779842	0.999933887	-0.178607088	0.026869782	-0.20547687	0.393461717
ENSG00000244071	*RPL9P33*	1.859426316	-0.069491814	0.035449066	-0.10494088	0.999933887	0.750669916	0.526137251	0.224532665	0.393719049
ENSG00000242622	*AC092910.3*	1.232977065	-0.11456175	-0.247674262	0.133112512	0.999933887	0.763513914	0.506011614	0.2575023	0.393719049
ENSG00000160917	*CPSF4*	2.892726495	0.030444733	0.030661003	-0.000216271	0.999933887	0.042393891	-0.101091167	0.143485058	0.393844161
ENSG00000110200	*ANAPC15*	1.460063691	0.127661011	0.047976385	0.079684626	0.999933887	0.175238034	-0.105026418	0.280264451	0.394165866
ENSG00000279469	*AC109454.4*	-0.083020404	-0.160953674	-0.071195037	-0.089758638	0.999933887	0.052965709	0.480085329	-0.427119621	0.394304437
ENSG00000143971	*ETAA1*	0.628386502	-0.239033303	-0.179859767	-0.059173536	0.999933887	-0.403826605	-0.039093148	-0.364733457	0.394561425
ENSG00000256720	*BTG1P1*	-0.504513304	-0.187254556	-0.137103779	-0.050150777	0.999933887	-0.295442692	0.120348991	-0.415791683	0.394614093
ENSG00000138867	*GUCD1*	7.172487083	-0.053597676	-0.041589524	-0.012008152	0.999933887	-0.110036457	-0.013796556	-0.0962399	0.394614093
ENSG00000155660	*PDIA4*	3.526778358	-0.007096106	-0.036393149	0.029297043	0.999933887	0.013879424	0.133316508	-0.119437084	0.394614093
ENSG00000158987	*RAPGEF6*	3.537465745	-0.075979594	-0.104125698	0.028146104	0.999933887	-0.221849631	0.049685934	-0.271535565	0.395010806
ENSG00000169607	*CKAP2L*	0.221398075	-0.196901804	0.00905695	-0.205958754	0.999933887	-0.424402809	-0.719881894	0.295479084	0.395393345
ENSG00000131732	*ZCCHC9*	2.518331936	-0.029471522	-0.111607145	0.082135624	0.999933887	-0.240487442	-0.09100911	-0.149478332	0.395393345
ENSG00000055609	*KMT2C*	8.159105151	-0.006269076	-0.05280542	0.046536345	0.999933887	-0.431777231	-0.258610701	-0.173166529	0.395393345
ENSG00000243438	*LARP1BP2*	-0.638401342	0.004354543	0.108805738	-0.104451195	0.999933887	1.018943663	1.541412381	-0.522468718	0.395412553
ENSG00000141576	*RNF157*	1.469160622	0.221260569	0.098795442	0.122465127	0.999933887	0.093895008	-0.122121984	0.216016992	0.395415748
ENSG00000156475	*PPP2R2B*	1.599462206	-0.09717396	-0.020199887	-0.076974073	0.999933887	-0.047470336	0.136695938	-0.184166275	0.395915564
ENSG00000204256	*BRD2*	8.953686016	0.047686198	0.043994434	0.003691765	0.999933887	0.723936155	0.826909918	-0.102973763	0.396224467
ENSG00000032742	*IFT88*	2.360635531	0.038480307	-0.001212273	0.03969258	0.999933887	-0.462942584	-0.233364313	-0.229578271	0.396473386
ENSG00000225093	*RPL3P7*	-1.42783911	-0.521270934	0.143845114	-0.665116048	0.999933887	0.021087702	0.561826048	-0.540738346	0.396555812
ENSG00000163960	*UBXN7*	5.867010693	-0.083587526	-0.075557514	-0.008030012	0.999933887	-0.3967221	-0.240554919	-0.156167181	0.396555812
ENSG00000100300	*TSPO*	6.584629101	0.057475807	0.026724835	0.030750972	0.999933887	0.019416782	-0.109135274	0.128552056	0.396555812
ENSG00000172845	*SP3*	7.719800701	-0.031573799	-0.044331811	0.012758012	0.999933887	-0.895578189	-0.75428141	-0.141296779	0.396555812
ENSG00000100629	*CEP128*	0.913078363	0.022879696	-0.305000905	0.327880601	0.999933887	0.263355356	0.539428603	-0.276073247	0.396555812
ENSG00000087269	*NOP14*	2.920171252	-0.005501932	-0.037837727	0.032335795	0.999933887	0.203144783	0.354346569	-0.151201786	0.396555812
ENSG00000242553	*AP001432.1*	-0.605803205	0.037293006	0.183199923	-0.145906916	0.999933887	-0.521625087	-0.948529421	0.426904335	0.396587616
ENSG00000130717	*UCK1*	3.774897772	-0.034424042	0.015217512	-0.049641553	0.999933887	-0.276970198	-0.391336556	0.114366357	0.396706932
ENSG00000254910	*AC136475.2*	0.877679636	-0.124452194	-0.320298961	0.195846767	0.999933887	-0.400226841	-0.700080827	0.299853986	0.397036466
ENSG00000278831	*AL513190.1*	-0.787843835	0.06496948	-0.132476421	0.197445901	0.999933887	-0.196618001	-0.794553753	0.597935753	0.397036466
ENSG00000241468	*ATP5MF*	2.732361634	0.007031267	0.027568971	-0.020537704	0.999933887	-0.033072507	-0.213951469	0.180878962	0.397036466
ENSG00000196220	*SRGAP3*	-0.193091804	0.059872803	0.114420096	-0.054547293	0.999933887	-0.378810506	0.16859726	-0.547407767	0.397080112
ENSG00000145332	*KLHL8*	2.766749282	0.029027653	0.002785399	0.026242254	0.999933887	-0.347336709	-0.537569888	0.190233179	0.397080112
ENSG00000100298	*APOBEC3H*	-0.308514383	0.137386953	0.031262994	0.106123959	0.999933887	-0.013422131	-0.302240461	0.28881833	0.397234128
ENSG00000149571	*KIRREL3*	-0.182646159	0.028295375	0.008581247	0.019714128	0.999933887	0.417869837	0.900189059	-0.482319222	0.397287083
ENSG00000239911	*PRKAG2-AS1*	-0.453810935	-0.144955085	0.01680864	-0.161763725	0.999933887	1.003039427	1.338185607	-0.33514618	0.397324266
ENSG00000104825	*NFKBIB*	6.598130406	0.120450899	0.093361381	0.027089518	0.999933887	1.084692444	1.2361224	-0.151429956	0.397396787
ENSG00000116521	*SCAMP3*	3.482909549	-0.042482031	-0.027230839	-0.015251192	0.999933887	0.049422563	-0.063364204	0.112786767	0.397396787
ENSG00000048544	*MRPS10*	2.893110927	-0.043051471	-0.101968425	0.058916955	0.999933887	-0.151527953	-0.019512155	-0.132015798	0.397396787
ENSG00000100181	*TPTEP1*	0.632734792	0.070883161	0.045627002	0.025256159	0.999933887	-0.834181195	-0.585249497	-0.248931698	0.397396787
ENSG00000135898	*GPR55*	-0.097321374	-0.061982196	-0.010829024	-0.051153172	0.999933887	0.387515112	0.721333668	-0.333818556	0.397396787
ENSG00000118508	*RAB32*	6.168489882	0.014657905	-0.062085388	0.076743293	0.999933887	0.083437213	0.193022108	-0.109584895	0.397396787
ENSG00000128524	*ATP6V1F*	6.470606173	0.0125219	-0.040293636	0.052815536	0.999933887	0.268995404	0.388636302	-0.119640897	0.397396787
ENSG00000003393	*ALS2*	3.610006962	0.013023046	-0.033837451	0.046860496	0.999933887	-0.236738146	-0.108133593	-0.128604553	0.397396787
ENSG00000112406	*HECA*	7.948795342	-0.006424647	0.031224813	-0.03764946	0.999933887	-0.278707147	-0.175891942	-0.102815205	0.397396787
ENSG00000108592	*FTSJ3*	3.727299649	-0.005497833	0.048575704	-0.054073536	0.999933887	0.009144473	-0.117268131	0.126412604	0.397396787

ENSG00000169918	*OTUD7A*	-0.090235338	0.153445531	0.035151806	0.118293725	0.999933887	0.097307314	0.421016507	-0.323709193	0.397440292
ENSG00000127080	*IPPK*	1.990337457	-0.046528104	0.036161318	-0.082689423	0.999933887	0.628090176	0.852981523	-0.224891347	0.397440292
ENSG00000166710	*B2M*	13.388422422	-0.002253769	-0.043354406	0.041100637	0.999933887	0.299354463	0.384217641	-0.084863178	0.39744833
ENSG00000164919	*COX6C*	2.665202494	-0.091752996	-0.062260824	-0.029492172	0.999933887	0.150609975	0.312432017	-0.161822042	0.397548969
ENSG00000078687	*TNRC6C*	5.170909987	0.025105311	0.044702883	-0.019597572	0.999933887	-0.209506471	-0.099791895	-0.109714575	0.397632222
ENSG00000106610	*STAG3L4*	2.901376332	0.000272935	0.036124467	-0.035851532	0.999933887	-0.071375553	0.090213343	-0.161588896	0.397632222
ENSG00000243646	*IL10RB*	2.687894188	-0.021501198	-0.001003203	-0.020497994	0.999933887	0.424486497	0.251805556	0.172680941	0.397857484
ENSG00000148572	*NRBF2*	6.949409664	0.024480375	-0.012317669	0.036798045	0.999933887	-0.340488325	-0.240212548	-0.100275777	0.398258937
ENSG00000231249	*ITPR1-DT*	0.14059268	0.108641578	-0.068640559	0.177282137	0.999933887	0.332385071	0.562435578	-0.230050507	0.398258937
ENSG00000175662	*TOM1L2*	4.344456875	0.010103806	0.059812204	-0.049708398	0.999933887	-0.18128889	-0.301799769	0.120510879	0.398258937
ENSG00000092140	*G2E3*	2.79792797	-0.000968522	-0.052287305	0.051318783	0.999933887	-0.406382423	-0.121309712	-0.285072712	0.398897839
ENSG00000176731	*RBIS*	1.77093704	-0.043747888	0.000173132	-0.04392102	0.999933887	0.038247041	-0.153956583	0.192203625	0.399276838
ENSG00000257433	*AC004241.1*	1.084222433	0.044622779	-0.188166658	0.232789437	0.999933887	-0.06172666	-0.339228832	0.277502173	0.399276838
ENSG00000211799	*TRAV19*	0.17272202	0.086912959	-0.302600238	0.389513196	0.999933887	0.215514955	0.545158107	-0.329643152	0.399287442
ENSG00000256039	*LINC02446*	1.354923085	0.065508738	-0.029005337	0.094514075	0.999933887	0.005999436	0.212985404	-0.206985968	0.399316381
ENSG00000042429	*MED17*	1.823208548	-0.094600179	0.004539506	-0.099139685	0.999933887	-0.273324576	-0.462211988	0.188887412	0.399512499
ENSG00000118620	*ZNF430*	3.243626897	0.08899169	-0.055217307	0.144208997	0.999933887	-0.385425931	-0.165795812	-0.219630119	0.399512499
ENSG00000172164	*SNTB1*	3.402021299	0.060080802	0.16923701	-0.109156209	0.999933887	-0.304770033	-0.485067797	0.180297764	0.399512499
ENSG00000213366	*GSTM2*	1.936883833	0.042118097	0.001654656	0.040463441	0.999933887	-0.333820597	-0.572265112	0.238444515	0.399512499
ENSG00000277791	*PSMB3*	6.052291836	-0.008872347	-0.034414437	0.02554209	0.999933887	-0.21655787	-0.329027684	0.112469814	0.399512499
ENSG00000288398	*AL109627.1*	-0.226950565	0.149243344	-0.107410017	0.256653361	0.999933887	0.09943571	0.507360409	-0.407924699	0.399678327
ENSG00000103248	*MTHFSD*	2.703088704	-0.151246429	0.014853076	-0.166099506	0.999933887	0.196913668	0.334806796	-0.137893128	0.399695599
ENSG00000162385	*MAGOH*	4.389513208	0.0271235	-0.017785212	0.044908712	0.999933887	0.190580979	0.310224769	-0.11964379	0.399695599
ENSG00000272277	*AL031963.3*	0.490012388	0.068228385	0.33195912	-0.263730736	0.999933887	0.053113085	-0.293136855	0.34624994	0.399710615
ENSG00000174099	*MSRB3*	1.470858717	-0.148628988	0.028385481	-0.177014469	0.999933887	-0.071335819	0.180347084	-0.251682903	0.399766473
ENSG00000113328	*CCNG1*	3.563885982	-0.118694148	-0.243686817	0.12499267	0.999933887	-0.114757341	0.03554317	-0.150300511	0.399766473
ENSG00000122705	*CLTA*	5.643209226	-0.077653152	-0.152668822	0.07501567	0.999933887	-0.357839039	-0.243118383	-0.114720655	0.399766473
ENSG00000189269	*DRICH1*	-0.410207254	-0.175473276	0.067797456	-0.243270732	0.999933887	0.357943208	-0.071450916	0.429394125	0.399766473
ENSG00000167302	*TEPSIN*	4.715338638	-0.035228	0.027726351	-0.06295435	0.999933887	-0.113054948	-0.209500053	0.096445104	0.399766473
ENSG00000142235	*LMTK3*	0.972874861	0.063601315	-0.051154336	0.11475565	0.999933887	-0.006551099	0.281390283	-0.287941382	0.399766473
ENSG00000131023	*LATS1*	3.722821082	-0.009979104	-0.005306178	-0.004672926	0.999933887	-0.373659175	-0.218515603	-0.155143572	0.399766473
ENSG00000246548	*LINC02288*	-0.941253711	-0.126259992	0.104138114	-0.230398106	0.999933887	-0.430871466	-1.100685406	0.66981394	0.399953371
ENSG00000240710	*AL512306.3*	1.043820177	-0.12536514	-0.107605114	-0.017760026	0.999933887	-0.378219646	-0.610629127	0.232409482	0.399953371
ENSG00000172893	*DHCR7*	2.858806845	-0.005372731	-0.043678385	0.038305655	0.999933887	0.103045828	0.298558981	-0.195513153	0.399953371
ENSG00000105767	*CADM4*	2.573649613	0.079173958	-0.044158184	0.123332141	0.999933887	0.683201461	0.856971183	-0.173769722	0.400174272
ENSG00000101191	*DIDO1*	6.124748458	-0.019913391	-0.004269954	-0.015643437	0.999933887	-0.143441781	-0.217299124	0.073857342	0.400174272
ENSG00000152778	*IFIT5*	5.333502424	-0.150263423	-0.139000687	-0.011262736	0.999933887	-0.616616447	-0.799281635	0.182665189	0.400427698
ENSG00000168813	*ZNF507*	3.789374925	-0.145707507	-0.164111186	0.018403679	0.999933887	-0.484018955	-0.295754475	-0.18826448	0.400560758
ENSG00000103056	*SMPD3*	3.383682438	0.005166978	0.087951389	-0.082784411	0.999933887	-0.17948226	-0.372082649	0.192600389	0.400560758
ENSG00000261373	*VPS9D1-AS1*	1.907983872	0.125312897	0.236987241	-0.111674344	0.999933887	0.117444821	-0.18714622	0.304591041	0.40075414
ENSG00000143106	*PSMA5*	4.617754995	-0.018851234	-0.066798694	0.04794746	0.999933887	0.264722068	0.363802162	-0.099080094	0.40075414
ENSG00000173145	*NOC3L*	1.006330962	-0.155742638	-0.131040485	-0.024702153	0.999933887	0.106461173	0.319239439	-0.212778265	0.401104949
ENSG00000142230	*SAE1*	3.873891848	-0.025796936	0.035961247	-0.061758184	0.999933887	-0.196094356	-0.316364777	0.120270421	0.401104949
ENSG00000267980	*AC007292.1*	-1.043094908	0.277437042	0.144495997	0.132941045	0.999933887	0.04179712	0.67201139	-0.63021427	0.401116965
ENSG00000144749	*LRIG1*	3.532729755	-0.025691991	0.106339323	-0.132031314	0.999933887	0.119245498	0.234961967	-0.115716469	0.401177861
ENSG00000204138	*PHACTR4*	2.695670901	-0.002875965	-0.026244765	0.0233688	0.999933887	-0.152253386	0.003143883	-0.155397269	0.401177861
ENSG00000147601	*TERF1*	2.583848905	0.030564784	-0.091870589	0.122435373	0.999933887	-0.219955753	-0.043181652	-0.176774101	0.401197865
ENSG00000139289	*PHLDA1*	4.818905915	0.122235114	0.296860184	-0.174625069	0.999933887	0.395630625	0.541806366	-0.146175741	0.401302165
ENSG00000128917	*DLL4*	-0.003403981	0.125467698	0.292287892	-0.166820194	0.999933887	0.485692093	0.866978158	-0.381286065	0.401462955
ENSG00000189298	*ZKSCAN3*	0.003052783	-0.036289626	-0.093947736	0.057658111	0.999933887	0.031448693	-0.260105863	0.291554555	0.401462955
ENSG00000221988	*PPT2*	-0.773885672	-0.037042998	0.442966707	-0.480009704	0.999933887	0.045798306	0.526721672	-0.480923366	0.401462955
ENSG00000143226	*FCGR2A*	8.9676917	0.007650373	-0.04131054	0.04960389	0.999933887	0.586130751	0.697865016	-0.111734265	0.401462955
ENSG00000278829	*AC099811.5*	2.621825764	0.200732083	0.12356717	0.077164913	0.999933887	1.398212828	1.663970529	-0.265757701	0.401516707
ENSG00000239857	*GET4*	2.613697729	0.163313222	0.145535196	0.017778026	0.999933887	0.125374439	-0.064337382	0.189711821	0.401516707
ENSG00000070087	*PFN2*	1.187062548	-0.036632579	0.03520318	-0.071835759	0.999933887	-0.627816783	-0.857629109	0.229812326	0.401728457
ENSG00000116106	*EPHA4*	3.045250227	-0.062317902	-0.117463462	0.055145559	0.999933887	-0.03045507	0.110309344	-0.140764414	0.402000906
ENSG00000172765	*TMCC1*	6.309514582	-0.003885475	0.002429109	-0.006314584	0.999933887	-0.221526619	-0.120673912	-0.100852707	0.402755934
ENSG00000198612	*COPS8*	2.620218192	-0.010067376	-0.087743297	0.077675921	0.999933887	0.013969247	0.181253344	-0.167284098	0.402848813
ENSG00000170291	*ELP5*	2.526642888	-0.070723327	0.076550903	-0.147274229	0.999933887	0.293530614	0.129765214	0.1637654	0.402939058
ENSG00000103043	*VAC14*	4.51144786	-0.012297516	0.185647178	-0.197944694	0.944094537	-0.069199996	-0.156075581	0.086875585	0.402939058
ENSG00000221944	*TIGD1*	1.396362281	-0.032614927	-0.120076554	0.087461627	0.999933887	0.134346968	-0.074154539	0.208501507	0.40304495
ENSG00000249936	*RAC1P2*	-0.290281765	0.017877224	0.150153571	-0.132276347	0.999933887	0.370032133	0.071023435	0.299008698	0.40306369
ENSG00000005812	*FBXL3*	5.077230146	-0.142293296	-0.213010354	0.070717057	0.999933887	-0.66125204	-0.532550747	-0.128701294	0.403081496
ENSG00000165113	*GKAP1*	1.13994411	0.095798443	-0.190659591	0.286458034	0.999933887	-0.525088367	-0.721674993	0.196586626	0.403081496
ENSG00000134982	*APC*	5.131834449	-0.044782621	-0.111142474	0.066359852	0.999933887	-0.912165798	-0.627890917	-0.284274882	0.403081496
ENSG00000111728	*ST8SIA1*	1.088018715	0.24284265	0.272506655	-0.029664005	0.999933887	0.041291757	0.227754324	-0.186462567	0.403134551
ENSG00000252904	*AC010359.1*	-0.730965894	-0.180873554	0.239314112	-0.420187667	0.999933887	0.997102479	1.463976452	-0.466873973	0.403134551
ENSG00000038219	*BOD1L1*	7.834311808	-0.111186731	-0.186869777	0.075683046	0.999933887	-0.905319985	-0.651305063	-0.254014922	0.403276497
ENSG00000130826	*DKC1*	5.084719847	-0.02608809	-0.081437251	0.055349161	0.999933887	-0.070276868	0.013229809	-0.083506677	0.403276497
ENSG00000144048	*DUSP11*	3.782190131	-0.012308932	0.005801709	-0.018110641	0.999933887	-0.323563137	-0.449041617	0.125478479	0.403276497
ENSG00000132773	*TOE1*	2.574086065	0.040396531	0.020272375	0.020124155	0.999933887	0.190712764	0.041058037	0.149654727	0.403394952
ENSG00000118513	*MYB*	1.152967588	-0.140152769	-0.267858216	0.127705447	0.999933887	0.086366936	0.243779896	-0.15741296	0.403633689
ENSG00000133687	*TMTC1*	-0.191527272	-0.076106793	0.082333173	-0.158439965	0.999933887	-0.623005186	-1.024432444	0.401427258	0.403633689
ENSG00000174946	*GPR171*	2.771047324	-0.076285001	0.051186777	-0.127471778	0.999933887	0.009738724	0.175541331	-0.165802607	0.403722661
ENSG00000006576	*PHTF2*	3.691842123	-0.047810251	-0.092403821	0.04459357	0.999933887	-0.396548346	-0.222508911	-0.174039436	0.403722661
ENSG00000001036	*FUCA2*	1.509733165	0.142941842	-0.022110637	0.165052479	0.999933887	-0.163883169	-0.361586405	0.197703236	0.403794834
ENSG00000163297	*ANTXR2*	6.703502957	0.024009137	0.048259469	-0.024250332	0.999933887	-0.467283313	-0.570686532	0.103403219	0.403794834
ENSG00000132906	*CASP9*	3.17417455	-0.001135785	0.071747037	-0.072882821	0.999933887	-0.066873452	-0.216953836	0.150080383	0.403803404
ENSG00000141552	*ANAPC11*	3.507290795	0.081808333	0.093772407	-0.011964074	0.999933887	0.131317972	-0.03772662	0.169044592	0.403862683
ENSG00000243302	*AC018638.4*	4.043281015	0.082707873	0.059794373	0.0229135	0.999933887	-0.121496911	0.037479819	-0.15897673	0.404555234
ENSG00000113119	*TMCO6*	3.242960492	-0.013630448	0.10058194	-0.114212388	0.999933887	-0.313480617	-0.437828346	0.124347729	0.404857881
ENSG00000148824	*MTG1*	0.309089123	0.108582515	-0.136169033	0.244751548	0.999933887	0.135792517	-0.119124529	0.254917045	0.404995395
ENSG00000154710	*RABGEF1*	4.386371929	0.117662046	0.067585283	0.050076764	0.999933887	0.871281311	1.013492641	-0.14221133	0.405002796
ENSG00000167528	*ZNF641*	5.496147512	-0.099704136	0.052902455	-0.152606591	0.999933887	-0.34220898	-0.456853025	0.114644046	0.405002796
ENSG00000152413	*HOMER1*	1.578780595	0.093760384	0.075618748	0.018141636	0.999933887	0.474006113	0.725992457	-0.251986344	0.405002796
ENSG00000009694	*TENM1*	2.282727761	-0.112996544	-0.149262289	0.036265746	0.999933887	-0.192172619	0.145083632	-0.337256251	0.405002796
ENSG00000130021	*PUDP*	0.797878703	0.081116635	-0.142869493	0.223986128	0.999933887	-0.021214924	-0.276379734	0.25516481	0.405002796
ENSG00000108395	*TRIM37*	3.017075323	-0.046338719	-0.095876042	0.049537323	0.999933887	-0.344468848	-0.494518221	0.150049373	0.405002796
ENSG00000151532	*VTI1A*	4.84901986	0.025962897	-0.011167961	0.037130858	0.999933887	0.464540411	0.564689193	-0.100148783	0.405002796
ENSG00000116237	*ICMT*	1.716190552	0.048210289	-0.184748174	0.232958463	0.999933887	0.094880972	-0.10698198	0.201862952	0.405002796
ENSG00000186448	*ZNF197*	0.848657842	0.037850269	-0.132282722	0.170132991	0.999933887	-0.270257615	-0.042828397	-0.227429218	0.405002796
ENSG00000068305	*MEF2A*	5.222027478	-0.006769427	0.016673348	-0.023442775	0.999933887	-0.284647946	-0.405879122	0.121231177	0.405002796
ENSG00000165527	*ARF6*	6.395168552	0.079179509	0.011170908	0.068008602	0.999933887	-0.392253558	-0.493631337	0.101377779	0.405165086
ENSG00000107736	*CDH23*	4.983649706	0.048224089	0.054673901	-0.006449812	0.999933887	0.471512241	0.579258977	-0.107746736	0.405165086
ENSG00000165572	*KBTBD6*	1.332619251	0.028370549	-0.205933765	0.234304314	0.999933887	0.344173972	0.580843299	-0.236669327	0.405165086
ENSG00000197056	*ZMYM1*	0.450595275	0.006479466	-0.173797696	0.180277162	0.999933887	-0.244136071	0.043151873	-0.287287944	0.405294489
ENSG00000126088	*UROD*	3.076676974	0.017973466	0.164346587	-0.146373121	0.999933887	0.184576811	0.295422151	-0.11084534	0.405520726
ENSG00000137507	*LRRC32*	-0.997003014	0.081721862	-0.18124692	0.262968782	0.999933887	1.665095594	2.231595654	-0.56650006	0.405520726
ENSG00000135108	*FBXO21*	2.778526475	-0.114425561	0.105890173	-0.220315734	0.944094537	0.018257807	0.138036051	-0.119778245	0.4058403
ENSG00000102786	*INTS6*	5.39869072	0.024045129	0.032118244	-0.008073116	0.999933887	0.267274551	0.415926871	-0.14865232	0.4058403
ENSG00000106266	*SNX8*	5.164972794	-0.006954264	0.055044997	-0.061999261	0.999933887	0.241273259	0.395932529	-0.154659271	0.4058403
ENSG00000185803	*SLC52A2*	2.337949113	0.005228902	-0.033183468	0.03841237	0.999933887	0.009573195	-0.160385929	0.169959124	0.4058403
ENSG00000168060	*NAALADL1*	-0.251660168	-0.003695955	0.060463973	-0.064159928	0.999933887	0.258636356	-0.077302813	0.33593917	0.405849431
ENSG00000181031	*RPH3AL*	-0.182124615	-0.099285106	0.022557421	-0.121842527	0.999933887	-0.194666568	0.185899231	-0.380565799	0.405923021
ENSG00000286071	*AL136171.2*	0.609490487	0.035162385	0.28918546	-0.254023074	0.999933887	0.336907922	0.92319135	-0.586283428	0.405973076

ENSG00000224383	*PRR29*	1.451728328	0.076657012	-0.011238787	0.087895799	0.999933887	0.416646148	0.670277126	-0.253630978	0.406071602
ENSG00000143442	*POGZ*	6.368361254	0.00249648	-0.020423819	0.0229203	0.999933887	0.013726809	0.082403338	-0.068676529	0.406166607
ENSG00000168995	*SIGLEC7*	2.500241701	-0.037640894	-0.015128424	-0.02251247	0.999933887	-0.299483373	-0.534135448	0.234652076	0.406529282
ENSG00000180957	*PITPNB*	4.587425384	-0.040057911	-0.098002526	0.057944616	0.999933887	0.046384735	0.141965243	-0.095580508	0.406697903
ENSG00000143314	*MRPL24*	1.262324234	0.066257984	0.087863256	-0.021605272	0.999933887	0.380723557	0.595969368	-0.21524581	0.406700916
ENSG00000089289	*IGBP1*	4.229233572	-0.01824403	-0.166494288	0.148250258	0.999933887	-0.053907294	0.053448435	-0.107355729	0.406700916
ENSG00000251192	*ZNF674*	3.922627422	-0.00761222	0.102082276	-0.109694497	0.999933887	-0.025637191	0.115915303	-0.141552494	0.406700916
ENSG00000160180	*TFF3*	-1.482278117	0.12822988	-0.11654847	0.24477835	0.999933887	0.216005785	-0.514553981	0.730559766	0.406812231
ENSG00000143643	*TTC13*	3.242153249	-0.051758485	0.021567162	-0.073325647	0.999933887	-0.18680699	-0.334526301	0.147719311	0.407089928
ENSG00000092529	*CAPN3*	0.741318363	0.051451699	-0.008505533	0.059957231	0.999933887	0.07103573	-0.211642676	0.282678407	0.407089928
ENSG00000189079	*ARID2*	4.589634707	-0.072001961	-0.079674385	0.007672425	0.999933887	-0.053790946	0.112635273	-0.166426219	0.407227055
ENSG00000183647	*ZNF530*	-0.155777656	-0.162969146	0.124515812	-0.287484958	0.999933887	-0.036778429	0.262838903	-0.299617333	0.407267848
ENSG00000179091	*CYC1*	3.535948725	-0.013394368	-0.011471595	-0.001922773	0.999933887	0.231526589	0.348937477	-0.117410888	0.407267848
ENSG00000258733	*LINC02328*	-0.012553141	-0.017187881	0.18361659	-0.200804471	0.999933887	-0.007199686	0.341969086	-0.349168772	0.407286368
ENSG00000230061	*TRPM2-AS*	0.460067722	-0.076587192	-0.158359611	0.081772418	0.999933887	0.976993768	1.207874519	-0.230880751	0.407348886
ENSG00000105298	*CACTIN*	3.514874457	0.045432772	0.16871533	-0.123282558	0.999933887	0.005890698	-0.157749681	0.16364038	0.407381375
ENSG00000000419	*DPM1*	4.307588162	-0.005036904	-0.032891046	0.027854143	0.999933887	0.05269906	0.136969464	-0.084270404	0.407403629
ENSG00000162852	*CNST*	4.838249709	-0.025383907	-0.013069283	-0.012314624	0.999933887	-0.04417601	0.050662114	-0.094838124	0.407826095
ENSG00000130779	*CLIP1*	5.932587926	0.008925573	-0.085231716	0.09415729	0.999933887	-0.584026037	-0.438664078	-0.145361959	0.407826095
ENSG00000054282	*SDCCAG8*	2.487326637	0.055826314	0.036875925	0.01895039	0.999933887	-0.130825124	-0.296167756	0.165342632	0.407877632
ENSG00000086619	*ERO1B*	3.707606754	0.064656398	0.031958806	0.032697593	0.999933887	0.010916038	-0.155329436	0.166245474	0.40792702
ENSG00000202019	*Y_RNA*	2.795004321	0.309277177	0.444832195	-0.135555019	0.999933887	1.730074188	2.049387531	-0.319313343	0.408303917
ENSG00000188157	*AGRN*	3.320918258	0.06711897	0.090892331	-0.023773362	0.999933887	0.494634431	0.677436252	-0.182801821	0.408493697
ENSG00000135914	*HTR2B*	-1.360124062	-0.138856324	-0.425562392	0.286706068	0.999933887	0.628176493	0.976173906	-0.347997413	0.408493697
ENSG00000111196	*MAGOHB*	0.646761501	-0.004168294	0.064278274	-0.068446567	0.999933887	0.024453416	0.291674838	-0.267221422	0.408493697
ENSG00000150457	*LATS2*	6.460778632	0.019391805	0.066529576	-0.047137771	0.999933887	-0.082491073	0.000318551	-0.082809624	0.408983092
ENSG00000281404	*LINC01176*	1.19392998	-0.070837145	-0.013109696	-0.057727449	0.999933887	-0.521680185	-0.817196785	0.2955166	0.409025303
ENSG00000271734	*Z98200.1*	0.469941728	0.029559067	0.020268155	0.009290912	0.999933887	0.231663275	0.438181321	-0.206518045	0.409097912
ENSG00000228477	*AL663070.1*	-0.137149269	-0.293636698	0.39237074	-0.686007438	0.999933887	2.41987694	2.762587369	-0.34271043	0.409210928
ENSG00000102158	*MAGT1*	5.013329613	-0.023095916	-0.037227032	0.014131116	0.999933887	0.229692795	0.333844543	-0.104151749	0.409210928
ENSG00000238005	*AL391832.2*	2.905476904	-0.143660044	0.041518139	-0.185178183	0.999933887	1.426926015	1.636532423	-0.209606407	0.409737508
ENSG00000197372	*ZNF675*	1.977053414	-0.000370279	-0.09494412	0.09457384	0.999933887	-0.67507888	-0.420556895	-0.254521985	0.409737508
ENSG00000128609	*NDUFA5*	2.958492507	-0.118983395	0.015602675	-0.134586071	0.999933887	-0.00888639	0.1819985	-0.19088489	0.409903451
ENSG00000078902	*TOLLIP*	6.492903424	0.026783487	-0.034052015	0.060835503	0.999933887	-0.063766984	0.02824578	-0.092012765	0.409920144
ENSG00000135018	*UBQLN1*	6.601045733	-0.047535143	-0.030348041	-0.017187102	0.999933887	-0.334055735	-0.26439557	-0.069660165	0.40992564
ENSG00000198894	*CIPC*	2.702830118	-0.080440744	0.183576079	-0.264016823	0.981460244	-0.61625711	-0.770234687	0.153977577	0.40992564
ENSG00000086015	*MAST2*	2.43237346	0.016189255	0.000760649	0.015428606	0.999933887	-0.084337815	0.047706074	-0.132043889	0.40992564
ENSG00000109618	*SEPSECS*	2.482630723	0.00124292	-0.262082369	0.263325289	0.999933887	-0.006637217	0.191115558	-0.197752776	0.4099505
ENSG00000165115	*KIF27*	2.811714797	-0.028786244	-0.21479548	0.186009236	0.999933887	-0.629549155	-0.938865846	0.30931669	0.410231666
ENSG00000169495	*HTRA4*	1.988433457	-0.013791851	0.165043477	-0.178835328	0.999933887	0.172244319	0.311738818	-0.139494499	0.410231666
ENSG00000102144	*PGK1*	8.118777071	-0.005844116	-0.013890239	0.008046123	0.999933887	0.053208431	-0.056269611	0.109478042	0.410243942
ENSG00000178163	*ZNF518B*	3.821275254	-0.051019397	-0.049298742	-0.001720655	0.999933887	-0.509918077	-0.374179275	-0.135738802	0.410672271
ENSG00000135722	*FBXL8*	0.996340956	0.095735811	-0.04577307	0.141508881	0.999933887	-0.046992112	-0.314347455	0.267355343	0.411062093
ENSG00000079337	*RAPGEF3*	1.053581794	-0.048031529	-0.11806063	0.070029101	0.999933887	-0.321030208	-0.539776028	0.21874582	0.411605426
ENSG00000186312	*CA5BP1*	2.221195622	0.026197858	-0.056930231	0.083128089	0.999933887	0.112983944	0.308054594	-0.19507065	0.411605426
ENSG00000287104	*AC097382.3*	1.589727941	-0.076251003	-0.021394667	-0.054856335	0.999933887	0.259196053	0.083909846	0.175286206	0.411874053
ENSG00000133393	*CEP20*	2.421968347	-0.031900961	0.111876738	-0.143777698	0.999933887	-0.053741194	0.079478878	-0.133220072	0.411964078
ENSG00000162923	*WDR26*	8.752050268	-0.010469804	-0.000886666	-0.009583138	0.999933887	-0.392642775	-0.312644722	-0.079998054	0.412015143
ENSG00000173653	*RCE1*	3.306570569	-0.028832004	0.008336833	-0.037168837	0.999933887	-0.065317954	-0.190560864	0.125242909	0.412167504
ENSG00000119812	*FAM98A*	1.570634132	-0.033899527	-0.089084735	0.055185208	0.999933887	0.123497804	0.338968487	-0.215470683	0.412167504
ENSG00000267074	*AC015911.3*	0.920914463	-0.01716385	0.042419252	-0.059583103	0.999933887	-0.194406516	0.044243686	-0.238650203	0.41216789
ENSG00000133627	*ACTR3B*	0.606441812	0.075330086	-0.21887985	0.294209936	0.999933887	-0.048884503	-0.39778451	0.348900007	0.412268408
ENSG00000229180	*AC006001.3*	4.069638834	0.064571614	0.06436161	0.000210004	0.999933887	-0.158525705	-0.258189568	0.099663864	0.412306201
ENSG00000176485	*PLAAT3*	0.343370809	-0.182915082	-0.204899212	0.021984129	0.999933887	0.034577394	0.331188308	-0.296610914	0.412509598
ENSG00000160818	*GPATCH4*	2.122919449	-0.13155037	-0.11424808	-0.01730229	0.999933887	0.25870346	0.441353271	-0.182649811	0.412509598
ENSG00000166535	*A2ML1*	0.802490654	-0.398720766	-0.270623955	-0.128096811	0.999933887	-0.564783254	-0.383792775	-0.18099048	0.412509598
ENSG00000130713	*EXOSC2*	2.31804448	-0.075726576	-0.076439314	0.000712738	0.999933887	0.227958894	0.078482178	0.149476716	0.412509598
ENSG00000244436	*AC023050.1*	1.354287492	0.140255941	0.044458461	0.09579748	0.999933887	0.495885572	0.783249547	-0.287363975	0.412509598
ENSG00000284976	*BX255925.3*	3.155221776	-0.02026483	0.22990538	-0.25017021	0.594252256	0.006284006	-0.128121998	0.134406004	0.412509598
ENSG00000176531	*PHLDB3*	1.349439004	0.005947172	0.158243447	-0.152296275	0.999933887	-0.063691104	0.175416907	-0.239108011	0.412509598
ENSG00000160051	*IQCC*	0.680050692	0.025875266	0.038185482	-0.012310216	0.999933887	0.135384392	-0.143918277	0.279302669	0.412689421
ENSG00000179335	*CLK3*	7.419658153	0.030312763	0.047835453	-0.01752269	0.999933887	0.202035454	0.276643134	-0.07460768	0.412933188
ENSG00000266983	*AC011444.1*	-0.905412683	-0.022949819	0.294803307	-0.317753125	0.999933887	-0.413747749	-1.039761038	0.626013289	0.412941082
ENSG00000285909	*AP002762.2*	0.295228929	0.0166776	0.153475619	-0.136798018	0.999933887	-0.226246371	0.110052866	-0.336299236	0.413116752
ENSG00000140006	*WDR89*	0.828639565	0.109967756	0.026531638	0.083436118	0.999933887	0.097827706	0.355699804	-0.257872099	0.413444721
ENSG00000186908	*ZDHHC17*	5.228723557	-0.053004019	-0.118478455	0.065474436	0.999933887	-0.516273527	-0.300102949	-0.216170579	0.413475831
ENSG00000277715	*AC079174.2*	0.699623789	-0.136961136	-0.099932953	-0.037028183	0.999933887	1.349020399	1.6795804	-0.330560002	0.414034662
ENSG00000273032	*DGCR5*	-0.967128788	-0.181531137	0.333307411	-0.514838548	0.594252256	0.18947005	-0.454480861	0.643950911	0.414569741
ENSG00000182022	*CHST15*	8.44162772	-0.020389029	-0.008768116	-0.011620912	0.999933887	-0.414061915	-0.329185378	-0.084876537	0.414741309
ENSG00000137216	*TMEM63B*	3.61737624	0.041265236	0.129268688	-0.088003452	0.999933887	0.72460454	0.866986793	-0.142382253	0.414752306
ENSG00000267212	*AC018761.3*	0.685132907	0.205599436	0.258298998	-0.052699562	0.999933887	0.585462798	0.836439317	-0.250976519	0.415034046
ENSG00000261114	*AC012181.1*	0.123975933	0.142486141	0.112490277	0.029995864	0.999933887	0.3438285	-0.149735857	0.493564357	0.415034046
ENSG00000105339	*DENND3*	9.548978313	0.0512974	0.030996987	0.020300413	0.999933887	-1.145216073	-1.282281984	0.137065911	0.415034046
ENSG00000147649	*MTDH*	5.103871151	-0.024553603	-0.025636097	0.001082494	0.999933887	-0.305403832	-0.416228811	0.110824979	0.415034046
ENSG00000049860	*HEXB*	6.874466615	0.000677249	-0.00055155	0.001228798	0.999933887	-0.593677043	-0.689600256	0.095923213	0.415034046
ENSG00000125651	*GTF2F1*	5.517300392	0.076413477	0.052374746	0.024038731	0.999933887	-0.082103524	0.044649321	-0.126752845	0.415186187
ENSG00000249992	*TMEM158*	2.669153639	0.056724758	0.073357749	-0.016632991	0.999933887	-0.634858518	-0.869543962	0.234685444	0.415186187
ENSG00000121060	*TRIM25*	8.730081719	-0.139767048	-0.066140581	-0.073626467	0.999933887	-0.746475382	-0.86826571	0.121790328	0.415413212
ENSG00000246889	*AP000487.1*	-0.144981414	0.212350405	-0.053275187	0.265625593	0.999933887	0.117269806	0.432636271	-0.315366465	0.415413212
ENSG00000196743	*GM2A*	4.250096449	-0.058144418	-0.069100124	0.010955706	0.999933887	1.016231142	1.260028749	-0.243797607	0.415413212
ENSG00000274092	*AC106739.1*	-1.150466273	0.23865879	-0.096539725	0.335198515	0.999933887	0.599323284	1.203196687	-0.603873403	0.415439214
ENSG00000285852	*AL353147.1*	-0.150880706	0.048853132	0.003359237	0.045493895	0.999933887	-0.386744727	-0.848148168	0.461403441	0.415439214
ENSG00000038945	*MSR1*	0.183222322	-0.270353759	0.025140706	-0.295494465	0.999933887	-1.502986528	-1.896344837	0.393358309	0.415480262
ENSG00000131845	*ZNF304*	1.291972821	-0.105364402	0.013935627	-0.119300028	0.999933887	-0.033204117	0.210270118	-0.243474235	0.415480262
ENSG00000075711	*DLG1*	3.615064667	0.021213885	-0.091956936	0.113170822	0.999933887	-0.153582126	0.02421803	-0.177800156	0.415492353
ENSG00000110777	*POU2AF1*	0.288563124	-0.127885196	-0.063288628	-0.064596568	0.999933887	-0.14411546	0.085829802	-0.229945263	0.415620968
ENSG00000168405	*CMAHP*	2.706711876	0.056180129	-0.158488888	0.214669017	0.999933887	0.016041029	0.205548693	-0.189507664	0.415620968
ENSG00000125354	*SEPTIN6*	5.72570065	0.011029652	-0.02285082	0.033880472	0.999933887	-0.010217528	-0.105818579	0.095601051	0.415620968
ENSG00000286681	*AF235103.3*	0.044516336	-0.029604217	0.08431322	-0.113917436	0.999933887	0.553097272	0.913544498	-0.360447225	0.415620968
ENSG00000282022	*AC006581.2*	-0.780803793	0.142181145	0.04857165	0.093609495	0.999933887	-0.000799944	0.21712187	-0.217921814	0.415699209
ENSG00000169976	*SF3B5*	5.149371463	0.045451397	0.010553491	0.034897906	0.999933887	0.100443338	-0.009877984	0.110321323	0.41610153
ENSG00000202502	*RNA5SP151*	-0.005886161	0.467361403	0.393619523	0.07374188	0.999933887	0.542816722	1.252066359	-0.709249637	0.416273928
ENSG00000154814	*OXNAD1*	3.027337099	0.016844458	-0.105280211	0.12212467	0.999933887	-0.033974089	0.092279426	-0.126253515	0.416380505
ENSG00000116663	*FBXO6*	3.066402786	0.035188072	0.021263177	0.013924895	0.999933887	0.302716089	0.458468491	-0.155752402	0.416474837
ENSG00000278970	*HEIH*	3.634041092	0.028467112	0.046593716	-0.018126603	0.999933887	0.560119577	0.696946931	-0.136827354	0.416474837
ENSG00000156875	*MFSD14A*	2.677517288	0.029229574	-0.085219248	0.114448823	0.999933887	0.233706913	0.377263565	-0.143556652	0.416698044
ENSG00000129473	*BCL2L2*	0.557361681	0.060161806	0.371217466	-0.31105566	0.999933887	-0.213309734	-0.428174684	0.214864951	0.417089965
ENSG00000248049	*UBA6-AS1*	-0.155197092	-0.006943757	0.17390522	-0.180848977	0.999933887	0.027074543	-0.289032237	0.316106779	0.417607823
ENSG00000066136	*NFYC*	6.076308337	-0.00857586	-0.022931517	0.014355656	0.999933887	-0.21505254	-0.134138695	-0.080913844	0.417632855
ENSG00000132879	*FBXO44*	2.691769682	0.013679588	0.09048903	-0.076809442	0.999933887	0.152369049	0.018002025	0.134367024	0.417632855
ENSG00000198799	*LRIG2*	2.607887233	-0.015993223	-0.042612474	0.026619252	0.999933887	-0.046307075	-0.183753405	0.13744633	0.417953139
ENSG00000102934	*PLLP*	-0.746990128	-0.243233626	0.061795978	-0.305029604	0.999933887	-0.398457238	0.012697458	-0.411154697	0.417957921
ENSG00000169258	*GPRIN1*	-0.193361395	0.152388843	-0.233712563	0.386101407	0.999933887	-0.253172265	-0.688731831	0.435559566	0.418190779
ENSG00000166140	*ZFYVE19*	2.545344709	0.019355435	0.109463692	-0.090108257	0.999933887	0.054845367	-0.104389934	0.159235301	0.418190779
ENSG00000129534	*MIS18BP1*	2.968922918	-0.038181972	-0.178696427	0.140514455	0.999933887	-0.818810034	-1.081002002	0.262191968	0.418355377

ENSG00000129354	*AP1M2*	0.271288919	-0.078522361	0.082568256	-0.161090617	0.999933887	-1.598241514	-2.031655821	0.433414306	0.41854785
ENSG00000233622	*CYP2T1P*	2.119380496	0.058063769	0.164661273	-0.106597504	0.999933887	0.024867963	0.254814041	-0.229946078	0.41854785
ENSG00000133069	*TMCC2*	0.179546413	-0.106487903	-0.254017999	0.147530095	0.999933887	0.123739233	-0.186459832	0.310199065	0.418814195
ENSG00000143167	*GPA33*	-0.052955639	-0.085343692	0.05444446	-0.139788151	0.999933887	0.076799645	0.41012975	-0.333330105	0.418814195
ENSG00000090971	*NAT14*	0.750286031	0.326205161	0.180145684	0.146059477	0.999933887	-0.203456174	-0.536281971	0.332825796	0.418954831
ENSG00000169902	*TPST1*	2.914284028	0.110399706	0.128102487	-0.017702782	0.999933887	-0.159507979	0.000629443	-0.160137422	0.418954831
ENSG00000228863	*AL121985.1*	3.617478287	0.031464634	0.198234542	-0.166769908	0.999933887	0.603845036	0.80586809	-0.202023054	0.418954831
ENSG00000177738	*AC025171.1*	1.582199328	-0.009302552	0.151879872	-0.161182424	0.999933887	-0.036449779	-0.249648564	0.213198785	0.418954831
ENSG00000116604	*MEF2D*	8.385059644	0.051081732	0.097275799	-0.046194067	0.999933887	-0.059206294	0.020374622	-0.079580915	0.418956694
ENSG00000174851	*YIF1A*	2.321080261	-0.023678411	0.049000767	-0.072679178	0.999933887	0.139694339	0.008702453	0.130991886	0.418970147
ENSG00000279942	*AL353597.3*	-1.234055546	0.04701667	-0.075800834	0.122817504	0.999933887	0.426243913	0.017317941	0.408925972	0.418970147
ENSG00000253593	*AC110741.1*	-0.83551364	0.334109778	0.483945349	-0.149835572	0.999933887	1.617542099	2.056572174	-0.439030075	0.419620288
ENSG00000118816	*CCNI*	8.232649341	-0.005443128	-0.016543681	0.011100552	0.999933887	0.042120365	0.112076192	-0.069955827	0.419726937
ENSG00000128585	*MKLN1*	5.316187685	-0.058772266	-0.058042783	-0.000729483	0.999933887	-0.724512168	-0.567171917	-0.157340252	0.420185791
ENSG00000282458	*WASH5P*	0.846667497	0.043936696	0.203946222	-0.160009526	0.999933887	-0.046190164	-0.275448822	0.229258658	0.420185791
ENSG00000160888	*IER2*	6.611099344	0.346861873	0.456306884	-0.109445011	0.999933887	0.197634057	-0.034348464	0.231982521	0.420478379
ENSG00000131981	*LGALS3*	8.9632194	-0.058003876	-0.024361024	-0.033642852	0.999933887	0.133034525	0.231568883	-0.098534359	0.420669487
ENSG00000136628	*EPRS1*	3.515730537	-0.067776218	-0.141097645	0.073321426	0.999933887	0.024759356	0.180257948	-0.155498591	0.420669487
ENSG00000008277	*ADAM22*	0.214776548	-0.066311029	-0.195940572	0.129629543	0.999933887	-0.321684677	0.063779116	-0.385463792	0.420868096
ENSG00000228315	*GUSBP11*	4.189257718	-0.00677704	0.015145982	-0.021923022	0.999933887	0.123036225	0.236966618	-0.113930393	0.420953378
ENSG00000138738	*PRDM5*	-0.010923751	-0.031647026	-0.055751219	0.024104194	0.999933887	-0.378148996	-0.764470679	0.386321683	0.420973309
ENSG00000180008	*SOCS4*	3.872633265	0.019696192	-0.180971107	0.200667299	0.999933887	-0.429040756	-0.21037787	-0.218662885	0.421370576
ENSG00000107862	*GBF1*	5.895502546	-0.015807412	0.03185658	-0.047663992	0.999933887	0.094258008	0.171121622	-0.076863614	0.421591836
ENSG00000280035	*AC011676.5*	4.829007373	0.027245958	0.021566329	0.005679629	0.999933887	0.146700017	0.305226641	-0.158526623	0.421593064
ENSG00000181873	*IBA57*	3.299007863	0.047851328	0.127723351	-0.079872023	0.999933887	0.091209935	0.223910377	-0.132700442	0.421805168
ENSG00000135365	*PHF21A*	6.986451057	0.013635355	-0.029650474	0.043285828	0.999933887	0.013423959	-0.062157662	0.07558162	0.421805168
ENSG00000160075	*SSU72*	7.210266976	-0.010754156	-0.030512568	0.019758412	0.999933887	-0.088415569	-0.015919419	-0.072496151	0.421913089
ENSG00000129932	*DOHH*	1.332178817	0.078651897	0.147228035	-0.068576137	0.999933887	0.118611734	0.357529613	-0.238917879	0.422246503
ENSG00000167074	*TEF*	0.49015101	0.0524783	-0.127397796	0.179876096	0.999933887	0.05558538	-0.248796107	0.304381487	0.422601349
ENSG00000160685	*ZBTB7B*	7.57600997	0.073884565	0.091360272	-0.017475706	0.999933887	-0.309764872	-0.172693291	-0.137071582	0.422697224
ENSG00000163564	*PYHIN1*	3.224363586	-0.080233605	-0.124740019	0.044506414	0.999933887	-0.125564897	0.068529417	-0.194094313	0.422697224
ENSG00000151923	*TIAL1*	5.231575354	-0.015752838	0.047287246	-0.063040084	0.999933887	-0.272486575	-0.37030392	0.097817345	0.422697224
ENSG00000146963	*LUC7L2*	4.900580517	-0.029524004	-0.038859175	0.009335171	0.999933887	-0.149941571	-0.029424094	-0.120517476	0.422846143
ENSG00000101082	*SLA2*	3.359858945	-0.002517885	-0.017592704	0.015074819	0.999933887	0.053378942	0.186405378	-0.133026436	0.423209564
ENSG00000116209	*TMEM59*	6.748710914	-0.023759276	-0.067329515	0.043570239	0.999933887	-0.167096221	-0.261118145	0.094021924	0.423390636
ENSG00000233896	*PDYN-AS1*	0.064685822	0.2007075	0.197169053	0.003538447	0.999933887	2.975996146	3.510385635	-0.534389488	0.42416159
ENSG00000144579	*CTDSP1*	6.926588914	0.030298605	0.029846699	0.000451906	0.999933887	-0.201332205	-0.295568927	0.094236722	0.42416159
ENSG00000243679	*AC018638.5*	4.186724631	0.0623405	-0.044632618	0.106973118	0.999933887	-0.169927337	-0.052537766	-0.117389571	0.424269749
ENSG00000220323	*H2BC19P*	1.968216404	0.056034568	-0.086641659	0.142676227	0.999933887	-0.692409566	-0.874937387	0.18252782	0.424269749
ENSG00000279170	*TSTD3*	-0.287056488	-0.095548836	-0.052494026	-0.04305481	0.999933887	-0.128494654	0.288363671	-0.416858325	0.424269749
ENSG00000188070	*C11orf95*	0.270735584	0.068923826	0.294730991	-0.225807165	0.999933887	0.215617485	-0.076640998	0.292258483	0.424269749
ENSG00000268081	*AC123912.1*	0.490695371	0.283311819	0.362521644	-0.079209825	0.999933887	0.69242113	0.373675242	0.318745888	0.424352893
ENSG00000173715	*C11orf80*	0.510039215	-0.028119193	-0.015214227	-0.012904966	0.999933887	-0.048602558	-0.342114526	0.293511968	0.424352893
ENSG00000157625	*TAB3*	4.954494624	-0.003378347	-0.079211218	0.075832871	0.999933887	-0.371312478	-0.199123448	-0.17218903	0.42462857
ENSG00000155368	*DBI*	4.54317514	0.017655599	-0.059613908	0.077269508	0.999933887	0.436728861	0.306072931	0.13065593	0.424953729
ENSG00000258623	*AL121820.1*	1.821148952	0.386309678	0.217304786	0.169004892	0.999933887	0.375139479	0.57238856	-0.197249081	0.42495488
ENSG00000223552	*CCR5AS*	3.365449631	0.074023746	0.244005478	-0.169981732	0.999933887	0.462883168	0.596268795	-0.133385627	0.42495488
ENSG00000268583	*AC011466.1*	0.608531257	0.101077916	0.013025856	0.088052059	0.999933887	-0.776665438	-1.050496703	0.273831264	0.425197172
ENSG00000144848	*ATG3*	6.773609491	-0.022399463	-0.014019876	-0.008379587	0.999933887	0.193786243	0.26441469	-0.070628447	0.425605006
ENSG00000100353	*EIF3D*	5.95011214	-0.044244959	-0.073115102	0.028870143	0.999933887	-0.068356999	-0.141930787	0.073573788	0.425708339
ENSG00000283525	*AC005622.1*	2.06892188	0.058823374	0.052876457	0.005946917	0.999933887	1.340126099	1.522675858	-0.182549759	0.425708339
ENSG00000111321	*LTBR*	5.733837862	0.057456294	0.0281742	0.029282094	0.999933887	-0.913476688	-1.02518364	0.111706952	0.4257142
ENSG00000137814	*HAUS2*	1.75411252	0.090050554	-0.151440581	0.241491135	0.999933887	0.103658563	0.277021084	-0.173362521	0.425860856
ENSG00000180953	*ST20*	1.414192712	-0.022552742	-0.08005169	0.057498948	0.999933887	-0.277194746	-0.014609559	-0.262585187	0.425920399
ENSG00000162688	*AGL*	2.542003888	-0.179454606	-0.413632585	0.234177978	0.999933887	-0.336372026	-0.098442961	-0.237929065	0.426062347
ENSG00000142687	*KIAA0319L*	5.629426713	-0.076103462	-0.047764431	-0.028339031	0.999933887	1.145471753	1.367551934	-0.222080181	0.426062347
ENSG00000269220	*LINC00528*	6.643813863	0.158851478	0.120681005	0.038170473	0.999933887	1.704264381	1.824591788	-0.120327407	0.426144503
ENSG00000112695	*COX7A2*	5.06321995	-0.06029965	-0.0403601	-0.01993955	0.999933887	0.18048489	0.27880925	-0.098324361	0.426144503
ENSG00000225973	*PIGBOS1*	1.507702554	-0.046725883	0.066407215	-0.113133097	0.999933887	-0.209400033	-0.398030155	0.188630122	0.426144503
ENSG00000112144	*CILK1*	1.400294271	0.019099044	-0.171199251	0.190298295	0.999933887	-0.167669512	0.061834382	-0.229503894	0.426144503
ENSG00000133226	*SRRM1*	6.227440609	-0.044718536	-0.063711146	0.01899261	0.999933887	-0.214840538	-0.129855261	-0.084985277	0.42663615
ENSG00000204116	*CHIC1*	1.390942029	-0.121754798	-0.252991518	0.13123672	0.999933887	-0.105075494	0.236045635	-0.341121129	0.42663615
ENSG00000161920	*MED11*	4.063326664	0.01461198	-0.038482206	0.053094186	0.999933887	-0.369038281	-0.250767499	-0.118270782	0.42663615
ENSG00000163357	*DCST1*	-0.154351611	-0.027879166	-0.011149567	-0.016729598	0.999933887	0.049974114	0.423040877	-0.373066763	0.42663615
ENSG00000131374	*TBC1D5*	3.927259611	-0.011150083	-0.030518663	0.019368581	0.999933887	-0.100774035	-0.207313429	0.106539394	0.426685335
ENSG00000204564	*C6orf136*	2.208911413	0.009693744	-0.018807741	0.028501485	0.999933887	-0.152667562	-0.311998302	0.15933074	0.426703809
ENSG00000007545	*CRAMP1*	3.162463371	-0.106339986	0.029876778	-0.136216764	0.999933887	-0.184020037	-0.298189358	0.114169321	0.426705257
ENSG00000068745	*IP6K2*	5.591509663	0.040243877	0.00093994	0.039303937	0.999933887	0.046695492	0.136822631	-0.090127139	0.426705257
ENSG00000164440	*TXLNB*	-0.140328828	0.02082027	0.017732743	0.003087528	0.999933887	-0.084811325	0.1904113	-0.275222625	0.426705257
ENSG00000119718	*EIF2B2*	1.549177946	-0.155819295	-0.034497997	-0.121321298	0.999933887	0.025136049	0.200195722	-0.175059674	0.427169065
ENSG00000125817	*CENPB*	3.581404182	0.06396701	0.064280325	-0.000313315	0.999933887	-0.018772143	-0.156805707	0.138033564	0.427169065
ENSG00000157778	*PSMG3*	1.7535503	-0.055681614	-0.060664654	0.004983041	0.999933887	0.176194704	0.010693459	0.165501245	0.427169065
ENSG00000254827	*SLC22A18AS*	3.182130046	0.041618845	-0.042961676	0.084580521	0.999933887	0.20207969	0.341935981	-0.139856291	0.427169065
ENSG00000269514	*AC024257.3*	-0.449025688	-0.059995969	0.032182637	-0.092178606	0.999933887	-0.376760229	0.098060921	-0.47482115	0.427293039
ENSG00000178789	*CD300LB*	5.600773302	-0.024296698	-0.004796358	-0.019500341	0.999933887	0.05394884	-0.108766749	0.16271559	0.42747245
ENSG00000231177	*LINC00852*	1.273579983	0.061003199	0.039810509	0.02119269	0.999933887	1.170791659	1.433466897	-0.262675238	0.427619642
ENSG00000139668	*WDFY2*	4.211911343	0.002226946	-0.030894676	0.033121623	0.999933887	0.113768487	-0.00769553	0.121464017	0.428414644
ENSG00000167447	*SMG8*	1.571427471	-0.116254736	-0.13196289	0.015708155	0.999933887	-0.035107916	-0.225818278	0.190710363	0.428822948
ENSG00000117262	*GPR89A*	-0.15153103	-0.265394154	-0.047250561	-0.218143593	0.999933887	0.071794228	-0.206543184	0.278337412	0.428933957
ENSG00000168826	*ZBTB49*	2.987688522	0.09016085	-0.085792511	0.175953361	0.999933887	-0.150795672	-0.264354556	0.113558884	0.428933957
ENSG00000185324	*CDK10*	3.850130858	0.026443555	0.139091557	-0.112648002	0.999933887	0.112246387	0.009848721	0.102397666	0.428933957
ENSG00000166762	*CATSPER2*	-0.988747999	-0.310270014	0.253412425	-0.563682438	0.999933887	-0.16637568	0.242008694	-0.408384374	0.428996813
ENSG00000146909	*NOM1*	2.625678984	-0.058280889	0.048168244	-0.106449133	0.999933887	0.200850054	0.049649449	0.151200605	0.428996813
ENSG00000161010	*MRNIP*	6.938306133	0.050560767	0.11103131	-0.060470542	0.999933887	0.673772381	0.79807475	-0.124302368	0.428996813
ENSG00000138496	*PARP9*	7.250570676	-0.017880747	-0.058296606	0.04041586	0.999933887	-0.302174136	-0.393465491	0.091291355	0.42918102
ENSG00000023516	*AKAP11*	3.443371836	-0.053286058	-0.151087278	0.09780122	0.999933887	-0.360658966	-0.088168729	-0.272490237	0.42918102
ENSG00000156642	*NPTN*	6.189859953	-0.003125117	-0.043254546	0.04012943	0.999933887	-0.209741039	-0.309188575	0.099447537	0.429236143
ENSG00000183474	*GTF2H2C*	0.742508929	-0.18971304	-0.010151119	-0.179561921	0.999933887	-0.742741003	-0.97847971	0.235738706	0.429445255
ENSG00000232931	*LINC00342*	2.640958016	-0.172293536	-0.157399159	-0.014894376	0.999933887	-0.263344982	0.040565307	-0.303910289	0.429624632
ENSG00000111237	*VPS29*	4.462255849	-0.017962493	-0.014147863	-0.00381463	0.999933887	-0.244600324	-0.339012948	0.094412624	0.429624632
ENSG00000180769	*WDFY3-AS2*	2.352965681	0.121940374	-0.000232431	0.122172805	0.999933887	0.766567124	0.988422107	-0.221854983	0.42973878
ENSG00000102870	*ZNF629*	0.626188144	0.00340058	-0.088366949	0.091767529	0.999933887	-0.00969915	0.213958888	-0.223658038	0.429943373
ENSG00000149823	*VPS51*	4.987548148	0.114615806	0.041489603	0.073126203	0.999933887	0.049701789	-0.066066824	0.115768613	0.429948697
ENSG00000159588	*CCDC17*	2.542881098	0.089934873	0.177504431	-0.087569558	0.999933887	-0.203269263	-0.041314965	-0.161954298	0.430064782
ENSG00000232578	*AC093311.1*	0.871812761	-0.024044864	0.000598616	-0.024643481	0.999933887	0.06919742	0.394618745	-0.325421325	0.430064782
ENSG00000197582	*GPX1P1*	-0.404353151	0.03823474	-0.0057293	0.043964041	0.999933887	-0.210755496	-0.502000147	0.291244651	0.430246267
ENSG00000182979	*MTA1*	3.672397577	0.071465667	-0.033028465	0.104494131	0.999933887	0.071861823	-0.053988186	0.125850009	0.430407231
ENSG00000167136	*ENDOG*	-0.674432157	0.077261675	-0.199861091	0.277122766	0.999933887	0.307993587	-0.066337723	0.374331309	0.430407231
ENSG00000116288	*PARK7*	4.483632066	0.007340447	-0.121836697	0.129177144	0.999933887	0.085880363	-0.030258477	0.11613884	0.430407231
ENSG00000280128	*AL662795.2*	0.985841949	-0.038360996	-0.059023698	0.020662702	0.999933887	-0.102729587	0.330151255	-0.432880842	0.430475
ENSG00000152620	*NADK2*	1.707930852	0.039783537	-0.046298278	0.086081815	0.999933887	-0.249182755	-0.438396013	0.189213258	0.430674832
ENSG00000229325	*ACAP2-IT1*	0.981893615	0.115000141	0.059414739	0.055585403	0.999933887	-0.481002563	-0.154333328	-0.326669235	0.430728967
ENSG00000211788	*TRAV13-1*	0.398312715	-0.021061019	-0.05830531	0.037244291	0.999933887	0.024720857	0.386291538	-0.361570681	0.430746482
ENSG00000100578	*KIAA0586*	2.43998676	0.096833677	-0.02326524	0.120098917	0.999933887	-0.31516747	-0.030998012	-0.284169458	0.430783419
ENSG00000110046	*ATG2A*	9.752182062	0.036809283	0.058095447	-0.021286164	0.999933887	0.132757512	0.221322704	-0.088565192	0.431044284

ENSG00000126461	*SCAF1*	6.024860416	-0.005118846	0.105366028	-0.110484874	0.999933887	-0.160895246	-0.278261071	0.117365826	0.431044284
ENSG00000168389	*MFSD2A*	4.15236092	-0.020759502	0.056733464	-0.077492966	0.999933887	2.532932297	2.767768622	-0.234836325	0.431083742
ENSG00000087076	*HSD17B14*	-0.298918243	-0.897071737	0.062270514	-0.959342251	0.999933887	0.369688376	0.563404678	-0.193716302	0.43124129
ENSG00000092295	*TGM1*	2.593388048	0.116981535	0.120738237	-0.003756702	0.999933887	1.067649911	1.203067188	-0.135417277	0.43124129
ENSG00000083720	*OXCT1*	2.008405594	0.042066641	-0.0287332	0.070799841	0.999933887	0.075911071	0.26301402	-0.187102949	0.43124129
ENSG00000167711	*SERPINF2*	-0.099592343	0.070693144	0.064516185	0.006176958	0.999933887	0.135300289	-0.145660142	0.280960431	0.43124129
ENSG00000086570	*FAT2*	-0.512992995	-0.273079214	0.226158329	-0.499237543	0.999933887	-0.60982218	-0.936742206	0.326920027	0.431302161
ENSG00000133392	*MYH11*	3.453045942	-0.007462881	-0.025561977	0.018099097	0.999933887	0.366601504	0.530185778	-0.163584273	0.431302161
ENSG00000147889	*CDKN2A*	-0.670272726	0.009395607	-0.033568674	0.042964281	0.999933887	0.227177897	-0.145524079	0.372701977	0.431302161
ENSG00000133193	*FAM104A*	3.538605263	-0.049216949	-0.023554733	-0.025662216	0.999933887	-0.599519814	-0.744345155	0.144825341	0.431439919
ENSG00000279086	*AC073130.3*	0.396886098	0.032957087	0.214570182	-0.181613094	0.999933887	0.196754823	0.545073393	-0.34831857	0.431439919
ENSG00000189180	*ZNF33A*	4.853511799	-0.020301269	-0.098033561	0.077732293	0.999933887	-0.553006864	-0.324019732	-0.228987132	0.431439919
ENSG00000104863	*LIN7B*	-0.667760445	0.01727897	-0.272938063	0.290217033	0.999933887	-0.038216263	-0.467878103	0.42966184	0.431586224
ENSG00000261609	*GAN*	0.585549427	0.142774302	0.103314095	0.039460207	0.999933887	-0.153506189	-0.482166958	0.328660769	0.431594896
ENSG00000198933	*TBKBP1*	5.058395722	0.061585883	0.048747951	0.012837933	0.999933887	0.098212048	-0.0222291	0.120441148	0.431594896
ENSG00000166224	*SGPL1*	4.222733729	-0.053010905	-0.048709837	-0.004301068	0.999933887	-0.318711323	-0.433750075	0.115038753	0.431594896
ENSG00000181029	*TRAPPC5*	0.619378992	-0.04207379	-0.124815049	0.082741259	0.999933887	0.124708707	0.374428951	-0.249720243	0.431594896
ENSG00000242372	*EIF6*	3.708092431	-0.019669153	-0.031142406	0.011473253	0.999933887	-0.09941686	-0.227420698	0.128003838	0.431594896
ENSG00000143553	*SNAPIN*	2.087135563	0.050388227	-0.16852966	0.218917887	0.999933887	-0.017880016	-0.188922529	0.171042513	0.431744863
ENSG00000171658	*NMRAL2P*	-0.058632408	-0.237998758	-0.119606824	-0.118391934	0.999933887	-0.02727659	0.169178553	-0.196455143	0.431780438
ENSG00000239521	*CASTOR3*	2.769677378	0.069258218	-0.017629918	0.086888135	0.999933887	-0.046372396	-0.220017365	0.173644969	0.431785621
ENSG00000214517	*PPME1*	2.395906709	0.034858404	0.018607563	0.016250841	0.999933887	-0.09248481	-0.254287779	0.161802969	0.431785621
ENSG00000122490	*SLC66A2*	5.995223692	0.07761638	0.040137137	0.037479244	0.999933887	0.117066567	0.005680056	0.111386511	0.431825041
ENSG00000148200	*NR6A1*	3.687621744	-0.033308333	-0.053685935	0.020377602	0.999933887	0.910766174	1.060540044	-0.14977387	0.431825041
ENSG00000006015	*REX1BD*	3.570798203	0.068119502	-0.007228382	0.075347885	0.999933887	0.114223023	-0.0542503	0.168473323	0.431835306
ENSG00000269019	*HOMER3-AS1*	-0.050529624	0.149197572	0.065011261	0.084186311	0.999933887	0.423772947	0.666669359	-0.242896412	0.431976865
ENSG00000131828	*PDHA1*	3.74148977	0.007047131	-0.042975273	0.050022404	0.999933887	0.257744551	0.376497188	-0.118752637	0.431976865
ENSG00000143493	*INTS7*	1.009352564	-0.095305316	-0.325585821	0.230280505	0.999933887	0.010127055	0.235525421	-0.225398366	0.432098567
ENSG00000186416	*NKRF*	1.394334756	-0.063537202	-0.152535145	0.088997943	0.999933887	0.154080905	-0.058426675	0.21250758	0.432098567
ENSG00000177721	*ANXA2R*	2.277709618	0.045266438	0.091053242	-0.045786804	0.999933887	0.173178459	0.005463549	0.167714911	0.432098567
ENSG00000180964	*TCEAL8*	0.446541358	0.062161851	0.053876952	0.008284899	0.999933887	0.161265298	0.386966021	-0.225700723	0.432521845
ENSG00000173137	*ADCK5*	1.701906796	-0.071289334	-0.063842857	-0.007446477	0.999933887	0.329634802	0.55304576	-0.223410958	0.432521845
ENSG00000151348	*EXT2*	3.073678434	0.01798516	-0.067859388	0.085844548	0.999933887	-0.002115688	0.135971166	-0.138086854	0.432521845
ENSG00000063322	*MED29*	5.398694973	0.060716837	0.002184282	0.058532555	0.999933887	0.206233162	0.098797551	0.10743561	0.432863249
ENSG00000186132	*C2orf76*	1.072065393	-0.045522749	0.143635466	-0.189158215	0.999933887	-0.104244088	-0.302894436	0.198650348	0.432902934
ENSG00000163463	*KRTCAP2*	0.223615614	0.032740779	0.131330773	-0.098589994	0.999933887	-0.082237074	-0.368038309	0.285801235	0.432902934
ENSG00000280069	*AC127024.8*	-0.555486244	-0.126676753	0.280948579	-0.407625332	0.999933887	-0.255830142	0.06254714	-0.318377281	0.433065515
ENSG00000156990	*RPUSD3*	1.947743751	-0.029789756	-0.020805687	-0.008984069	0.999933887	0.022707637	-0.130202068	0.152909705	0.433324174
ENSG00000112305	*SMAP1*	2.145695966	0.09509895	-0.028874593	0.123973543	0.999933887	-0.096210631	-0.282653545	0.186442914	0.433428515
ENSG00000135372	*NAT10*	3.264762771	-0.000898418	0.011804485	-0.012702903	0.999933887	0.1984345	0.305162221	-0.106727721	0.433537846
ENSG00000230202	*AL450405.1*	2.56181285	0.115050791	0.070300963	0.044749828	0.999933887	0.018377671	0.200703113	-0.182325442	0.433766885
ENSG00000103811	*CTSH*	3.732284528	0.05607543	-0.021935968	0.078011398	0.999933887	0.396921485	0.534422623	-0.137501138	0.433766885
ENSG00000131584	*ACAP3*	4.784696125	0.032005749	0.120583718	-0.088577969	0.999933887	0.109024541	-0.003056387	0.112080928	0.433771792
ENSG00000121577	*POPDC2*	0.553731669	0.035906078	0.008312678	0.0275934	0.999933887	0.443046243	0.711758328	-0.268712086	0.433781087
ENSG00000138686	*BBS7*	0.458879486	-0.179913522	0.065747691	-0.245661213	0.999933887	-0.028958016	0.250321983	-0.279279999	0.433964539
ENSG00000223891	*OSER1-DT*	4.285269325	-0.023483179	-0.132882142	0.109398963	0.999933887	-0.594706797	-0.456408931	-0.138297866	0.433964539
ENSG00000254554	*AC080023.1*	2.922314809	0.118762643	0.201541866	-0.082779223	0.999933887	0.69138961	0.828785638	-0.137396029	0.43402369
ENSG00000012061	*ERCC1*	3.190817816	0.034489945	0.027718342	0.006771604	0.999933887	0.106302507	0.247531105	-0.141228598	0.43402369
ENSG00000185608	*MRPL40*	0.879777607	-0.017652436	0.176537911	-0.194190347	0.999933887	-0.179294848	-0.485594693	0.306299845	0.434236608
ENSG00000158796	*DEDD*	4.724273216	-0.040638418	-0.039459345	-0.001179072	0.999933887	-0.194266778	-0.105553192	-0.088713587	0.43432134
ENSG00000103254	*ANTKMT*	1.093415595	0.067010931	-0.004807739	0.07181867	0.999933887	0.021181656	-0.188363551	0.209545206	0.43432134
ENSG00000162804	*SNED1*	1.599756314	0.025112743	0.151471266	-0.126358523	0.999933887	-0.009786263	0.159334345	-0.169120607	0.434474819
ENSG00000242588	*AC108010.1*	2.222219325	0.020319945	0.005655432	0.014664513	0.999933887	-0.172865111	-0.009837017	-0.163028094	0.434571369
ENSG00000149970	*CNKSR2*	-0.045733064	-0.254161059	0.10498724	-0.359148299	0.999933887	-0.267280885	0.066749394	-0.334030279	0.434812779
ENSG00000185246	*PRPF39*	3.580097755	-0.088020499	-0.171003559	0.08298306	0.999933887	-0.030534481	0.158489349	-0.189023829	0.434812779
ENSG00000110514	*MADD*	5.430996417	0.001181764	-0.035091648	0.036273412	0.999933887	-0.037065724	-0.114932853	0.077867129	0.434812779
ENSG00000228716	*DHFR*	0.456068669	0.101563665	-0.120762084	0.222325749	0.999933887	-0.387158666	-0.083909752	-0.303248915	0.434829791
ENSG00000215067	*ALOX12-AS1*	4.864984605	0.11028519	0.086713978	0.023571213	0.999933887	0.392774434	0.486174481	-0.093400047	0.435059528
ENSG00000251369	*ZNF550*	1.168804741	-0.064620807	-0.163509287	0.098888479	0.999933887	0.097904691	-0.076494309	0.174399	0.435119114
ENSG00000112699	*GMDS*	2.068591359	0.102342992	0.122799026	-0.020456034	0.999933887	-0.042150359	0.186578603	-0.228728962	0.435419491
ENSG00000102977	*ACD*	3.405384687	0.067515496	0.047860838	0.019654658	0.999933887	0.231873841	0.355583092	-0.123709251	0.435432729
ENSG00000065518	*NDUFB4*	3.74405201	-0.037262911	0.009650247	-0.046913158	0.999933887	0.043807526	0.155816803	-0.112009276	0.435432729
ENSG00000213462	*ERV3-1*	6.981770791	-0.027264433	-0.04377259	0.016508157	0.999933887	-0.258687142	-0.144166307	-0.114520835	0.435432729
ENSG00000205937	*RNPS1*	5.839797492	-0.011674181	0.001948983	-0.013623163	0.999933887	-0.068065896	-0.141431915	0.073366019	0.435432729
ENSG00000139211	*AMIGO2*	2.496296722	0.010087121	6.234e-05	0.010024781	0.999933887	0.327937655	0.459715858	-0.131778202	0.435432729
ENSG00000104972	*LILRB1*	3.966999872	0.04092912	-0.004767878	0.045696998	0.999933887	-0.083693501	-0.214135606	0.130442106	0.435549788
ENSG00000112237	*CCNC*	2.072213805	-0.008305332	-0.280104046	0.271798714	0.999933887	-0.001980273	0.197192837	-0.19917311	0.435549788
ENSG00000167550	*RHEBL1*	-0.168095024	0.021319641	-0.389544931	0.410864573	0.999933887	0.017410633	-0.428136233	0.445546866	0.435691914
ENSG00000073150	*PANX2*	5.729818791	0.001210012	0.069950211	-0.068740199	0.999933887	-0.344736376	-0.45686494	0.112128564	0.435691914
ENSG00000139908	*TSSK4*	2.26904951	-0.090888997	-0.057090881	-0.033798116	0.999933887	0.09147737	0.237858116	-0.146380745	0.435820145
ENSG00000198373	*WWP2*	7.44136283	-0.027706055	-0.009280708	-0.018425347	0.999933887	-0.417081142	-0.507742301	0.090661159	0.435820145
ENSG00000198624	*CCDC69*	6.498340988	0.003196142	-0.01927311	0.022469252	0.999933887	-0.064529538	-0.147528735	0.082999196	0.435950156
ENSG00000086848	*ALG9*	-0.240451794	-0.366072647	0.031240157	-0.397312804	0.999933887	-0.105303223	0.231514306	-0.336817529	0.436558769
ENSG00000182325	*FBXL6*	2.619724797	0.124138171	0.087008538	0.037129633	0.999933887	0.276797037	0.097411876	0.179385161	0.436558769
ENSG00000117009	*KMO*	-0.230947534	-0.101394682	0.290768444	-0.392163127	0.999933887	1.128851879	1.450699863	-0.321847984	0.436558769
ENSG00000111145	*ELK3*	3.216217959	0.008389108	0.145369927	-0.136980819	0.999933887	-0.193276608	-0.340607463	0.147330855	0.436558769
ENSG00000233365	*AL121956.1*	1.863923288	-0.033631623	-0.131923451	0.098291829	0.999933887	0.507288153	0.706214842	-0.198926688	0.436569498
ENSG00000143368	*SF3B4*	5.596400786	-0.003306414	0.007722881	-0.011029295	0.999933887	-0.163105527	-0.071789756	-0.091315771	0.436571338
ENSG00000196267	*ZNF836*	0.846893153	-0.114280709	-0.080676569	-0.03360414	0.999933887	-0.285841288	-0.518657099	0.232815811	0.436586748
ENSG00000198677	*TTC37*	3.342249665	-0.111132484	-0.161231272	0.050098788	0.999933887	-0.324467717	-0.102637266	-0.221830451	0.436801046
ENSG00000011485	*PPP5C*	2.44089097	-0.035605397	0.002378864	-0.037984261	0.999933887	0.109402097	0.259826699	-0.150424603	0.436801046
ENSG00000160201	*U2AF1*	3.274935997	0.070297619	0.090209784	-0.019912165	0.999933887	-0.029785092	0.120574676	-0.150359768	0.436834436
ENSG00000119707	*RBM25*	7.563893181	-0.068129443	-0.179841558	0.111712114	0.999933887	-0.504659153	-0.238864594	-0.265794559	0.436899904
ENSG00000011009	*LYPLA2*	5.261349308	0.016054062	-0.060345601	0.076399663	0.999933887	-0.249565941	-0.340488004	0.090922063	0.436899904
ENSG00000182885	*ADGRG3*	8.39324188	0.008768714	0.015618478	-0.006849764	0.999933887	0.02220641	0.105549116	-0.083342706	0.436899904
ENSG00000114491	*UMPS*	1.174000026	-0.004914518	0.246548056	-0.251462574	0.999933887	0.191633114	0.365108006	-0.173474892	0.437101227
ENSG00000112739	*PRPF4B*	5.450741692	-0.083622335	-0.154683846	0.071061511	0.999933887	-0.435128086	-0.224866253	-0.210261833	0.437463803
ENSG00000102901	*CENPT*	3.550958174	-0.034156514	0.059928222	-0.094084735	0.999933887	0.119713638	-0.001636166	0.121349804	0.437466102
ENSG00000156232	*WHAMM*	4.749004228	-0.006946786	-0.000366977	-0.00657981	0.999933887	-0.006464136	-0.09932676	0.092862625	0.437562302
ENSG00000140464	*PML*	6.044295949	0.031620963	-0.007003956	0.038624918	0.999933887	-0.037517538	-0.128685502	0.091167964	0.437675341
ENSG00000236008	*LINC01814*	0.36711483	0.078303595	-0.014000572	0.092304167	0.999933887	0.41733737	0.746520091	-0.329182721	0.437675341
ENSG00000173212	*MAB21L3*	-1.142792871	-0.058342271	-0.139706829	0.081364559	0.999933887	-0.700969494	-0.114654794	-0.5863147	0.437675341
ENSG00000099974	*DDTL*	0.546013157	-0.069545003	0.108703573	-0.178248575	0.999933887	0.078532342	-0.159146292	0.237678634	0.437720445
ENSG00000148362	*PAXX*	3.772858774	0.017840068	0.087249268	-0.069409201	0.999933887	0.167563529	0.048401446	0.119162083	0.437720445
ENSG00000268758	*ADGRE4P*	4.232281309	-0.062462862	0.016967653	-0.079430515	0.999933887	-0.045548426	0.08083675	-0.126385176	0.437724391
ENSG00000160953	*PWWP3A*	2.213158326	0.034845974	-0.009211201	0.044057176	0.999933887	-0.081331195	-0.258723634	0.177392439	0.437724391
ENSG00000126756	*UXT*	3.581309839	-0.006724188	0.043729119	-0.050453307	0.999933887	0.256371984	0.373562988	-0.117191004	0.437724391
ENSG00000280295	*AC099811.6*	-0.035378373	0.063368722	0.13722879	-0.073860067	0.999933887	0.619223973	0.993006821	-0.373782847	0.437789686
ENSG00000165819	*METTL3*	3.995450214	-0.031520137	-0.033534363	0.002014227	0.999933887	-0.094940169	-0.190152145	0.095211977	0.437872014
ENSG00000143443	*C1orf56*	1.420310101	0.009884985	0.187076727	-0.177191742	0.999933887	0.473442859	0.275570714	0.197872144	0.437872014
ENSG00000078304	*PPP2R5C*	8.028982969	-0.027839104	-0.053009239	0.025170135	0.999933887	0.217218716	0.290028737	-0.072810021	0.437928367
ENSG00000173889	*PHC3*	5.623971371	-0.035714446	-0.037164489	0.001450043	0.999933887	-0.512493102	-0.342512498	-0.169980604	0.438153334
ENSG00000120688	*WBP4*	2.797753998	-0.031268603	-0.119950023	0.088681419	0.999933887	-0.251784251	-0.114230171	-0.13755408	0.438153334
ENSG00000132334	*PTPRE*	10.027251754	0.067403965	0.056463368	0.010940597	0.999933887	0.086964951	0.161961363	-0.074996412	0.4385266
ENSG00000161813	*LARP4*	2.037500059	-0.180725191	-0.136584462	-0.044140728	0.999933887	-0.229607805	-0.052856444	-0.176751361	0.4385266

ENSG00000197903	*H2BC12*	4.321325703	0.066556603	0.014083527	0.052473076	0.999933887	-0.128888175	-0.26883151	0.139943334	0.438670247
ENSG00000150637	*CD226*	3.421098679	-0.053997606	-0.074635011	0.020637404	0.999933887	-0.163721456	0.0231733	-0.186894756	0.438862344
ENSG00000071537	*SEL1L*	6.496471917	-0.100204531	-0.107602806	0.007398275	0.999933887	-0.505733071	-0.375853574	-0.129879497	0.438873136
ENSG00000105821	*DNAJC2*	3.279690952	-0.091854987	-0.195104426	0.103249439	0.999933887	-0.092633412	0.027836579	-0.120469991	0.43899152
ENSG00000125898	*FAM110A*	5.254600692	-0.076128325	-0.097167892	0.021039567	0.999933887	0.51829197	0.646057121	-0.12776515	0.43899704
ENSG00000124786	*SLC35B3*	2.485006796	0.030357023	0.170677669	-0.140320646	0.999933887	-0.097287519	0.038901475	-0.136188994	0.43899704
ENSG00000153071	*DAB2*	-0.041959497	-0.00903913	-0.001992562	-0.007046567	0.999933887	-1.279884245	-1.738966658	0.459082412	0.43899704
ENSG00000152904	*GGPS1*	1.571603343	0.009951752	-0.029828937	0.039780689	0.999933887	-0.300800547	-0.134080819	-0.166719728	0.439377757
ENSG00000024048	*UBR2*	7.585991983	-0.056403891	-0.07232285	0.015918959	0.999933887	-0.967836603	-1.111018327	0.143181724	0.43969078
ENSG00000145388	*METTL14*	3.170035678	-0.10923158	-0.076637051	-0.032594529	0.999933887	-0.226829109	-0.055380181	-0.171448928	0.439701842
ENSG00000111361	*EIF2B1*	3.407088655	-0.056185891	-0.156133837	0.099947945	0.999933887	-0.056994993	-0.180936777	0.123941784	0.439701842
ENSG00000130311	*DDA1*	4.707631515	0.01215372	0.023842227	-0.011688507	0.999933887	0.028099819	0.119956174	-0.091856355	0.439701842
ENSG00000139197	*PEX5*	2.921950741	0.043261548	-0.081605928	0.124867476	0.999933887	-0.085654014	-0.220469555	0.134815541	0.439705448
ENSG00000182183	*SHISAL2A*	2.104167001	-0.05768774	-0.010604293	-0.047083447	0.999933887	-0.158538714	-0.305254501	0.146715787	0.439789632
ENSG00000084234	*APLP2*	8.566714692	-0.017130273	-0.043603804	0.026473531	0.999933887	0.162093699	0.2535233	-0.091429601	0.439789632
ENSG00000278535	*DHRS11*	-0.576507638	-0.141311339	-0.284617693	0.143306354	0.999933887	0.097253656	-0.286119028	0.383372685	0.439941655
ENSG00000164548	*TRA2A*	6.415977773	-0.030324581	-0.000143784	-0.030180797	0.999933887	0.19041918	0.280061738	-0.089642558	0.440084406
ENSG00000107560	*RAB11FIP2*	3.213050337	0.025298661	-0.15695177	0.182250431	0.999933887	-0.839296403	-0.602108958	-0.237187445	0.440084406
ENSG00000136478	*TEX2*	1.889779599	0.118794327	0.071312235	0.047482092	0.999933887	-0.267086782	-0.458201835	0.191115053	0.440138423
ENSG00000102893	*PHKB*	4.048204041	-0.052634873	-0.086669966	0.034035093	0.999933887	-0.342897817	-0.472746441	0.129848624	0.440138423
ENSG00000259657	*PIGHP1*	0.71230326	0.080741815	0.113500759	-0.032758945	0.999933887	-0.025814998	0.270032807	-0.295847804	0.440138423
ENSG00000225231	*LINC02470*	3.041073108	0.018609911	-0.150005667	0.168615578	0.999933887	1.768593512	2.131737885	-0.363144373	0.440138423
ENSG00000279719	*AC015871.5*	1.268849667	0.359649133	0.238294432	0.121354702	0.999933887	1.647140741	1.282446425	0.364694316	0.440175391
ENSG00000013016	*EHD3*	0.063343782	-0.202736928	0.006330933	-0.209067861	0.999933887	-0.035827878	-0.362951351	0.327123473	0.440175391
ENSG00000125843	*AP5S1*	1.273977799	0.008694246	-0.038416716	0.047110963	0.999933887	-0.128060821	0.091254565	-0.219315386	0.440227371
ENSG00000088387	*DOCK9*	3.700928559	-0.004277145	-0.086743911	0.082466767	0.999933887	-0.01069384	0.109490868	-0.120184709	0.440227371
ENSG00000134480	*CCNH*	4.920627687	-0.002483947	-0.015820939	0.013336991	0.999933887	-0.021357976	0.156603407	-0.177961384	0.440227371
ENSG00000142166	*IFNAR1*	7.131455605	-0.021095376	-0.010061476	-0.011033901	0.999933887	0.272211891	0.387135456	-0.114923564	0.440575844
ENSG00000184005	*ST6GALNAC3*	1.140718969	-0.019210071	0.003440244	-0.022650316	0.999933887	-0.266581105	-0.507766284	0.241185179	0.440680863
ENSG00000146426	*TIAM2*	3.283728657	-0.078692599	-0.052481507	-0.026211092	0.999933887	-0.146190232	-0.018446455	-0.127743777	0.440710789
ENSG00000197992	*CLEC9A*	1.916746514	0.117869953	-0.012811698	0.130681652	0.999933887	-0.93962745	-1.216667785	0.277040335	0.440906742
ENSG00000144218	*AFF3*	-0.086264048	-0.059531549	0.035328291	-0.09485984	0.999933887	-0.086396857	0.193237846	-0.279634703	0.441063494
ENSG00000196235	*SUPT5H*	7.313314958	-0.013079047	0.022022513	-0.03510156	0.999933887	-0.00863792	-0.08546549	0.07682757	0.441281543
ENSG00000163684	*RPP14*	2.348122377	-0.075516848	0.042921698	-0.118438547	0.999933887	0.042438779	-0.131260141	0.17369892	0.441557181
ENSG00000183779	*ZNF703*	-0.139892158	0.195716195	0.224177981	-0.028461786	0.999933887	-0.18942488	-0.593699968	0.404275087	0.441855448
ENSG00000177106	*EPS8L2*	0.111668528	-0.226312471	-0.251238771	0.024926299	0.999933887	0.040648096	-0.301999415	0.34264751	0.441869754
ENSG00000164117	*FBXO8*	2.269922727	-0.038378122	0.038290698	-0.07666882	0.999933887	-0.039884371	0.095100182	-0.134984553	0.441869754
ENSG00000136986	*DERL1*	5.467730424	0.014732417	0.020963407	-0.00623099	0.999933887	-0.11129107	-0.187490723	0.076199654	0.441869754
ENSG00000181788	*SIAH2*	5.434428349	0.001281699	0.063116332	-0.061834633	0.999933887	0.717475211	0.854430582	-0.13695537	0.441869754
ENSG00000182628	*SKA2*	0.793085433	0.061505105	-0.077564361	0.139069466	0.999933887	0.006319872	0.252726216	-0.246406344	0.442196293
ENSG00000035928	*RFC1*	3.655478145	0.005787071	-0.109433599	0.11522067	0.999933887	-0.230429476	-0.051564537	-0.17886494	0.442238367
ENSG00000197054	*ZNF763*	0.909294147	-0.134940467	0.037929203	-0.17286967	0.999933887	-0.089662987	-0.299675136	0.210012148	0.442351452
ENSG00000143479	*DYRK3*	1.208346585	0.38466585	0.077215565	0.307450285	0.999933887	2.299599505	2.631547826	-0.331948322	0.44241127
ENSG00000168710	*AHCYL1*	5.791958324	-0.040077521	-0.055962465	0.015884944	0.999933887	-0.249554002	-0.171764414	-0.077789588	0.44241127
ENSG00000248905	*FMN1*	1.056137661	-0.091018972	-0.066402354	-0.024616618	0.999933887	-0.128480578	0.158018214	-0.286498791	0.44241127
ENSG00000240053	*LY6G5B*	2.409099014	-0.041526079	-7.5884e-05	-0.041450195	0.999933887	-0.476825459	-0.317021607	-0.159803852	0.44241127
ENSG00000175602	*CCDC85B*	3.136530432	0.140649833	0.188189884	-0.04754005	0.999933887	0.083323925	-0.137727672	0.221051598	0.442411622
ENSG00000235652	*FBXO30-DT*	-0.797670267	-0.242684652	-0.034389768	-0.208294885	0.999933887	-0.054680096	0.379692236	-0.434372331	0.44242094
ENSG00000118418	*HMGN3*	1.810120894	-0.178733207	0.093702863	-0.27243607	0.999933887	0.134714708	-0.064446409	0.199161117	0.442425842
ENSG00000139180	*NDUFA9*	0.775407141	0.133109119	-0.013965134	0.147074253	0.999933887	0.132393169	-0.158036365	0.290429534	0.442537988
ENSG00000151413	*NUBPL*	-0.581571951	0.082521508	-0.215338819	0.297860327	0.999933887	-0.3910358	-0.019573629	-0.371462171	0.442566292
ENSG00000168701	*TMEM208*	3.582315958	-0.019022309	-0.12767955	0.108657241	0.999933887	0.237259223	0.116935054	0.120324169	0.442566292
ENSG00000272288	*AL451165.2*	2.782993941	0.032238519	0.256500102	-0.224261583	0.999933887	1.005171095	0.862136864	0.143034232	0.442648341
ENSG00000040633	*PHF23*	5.169385821	-0.041154204	-0.024108555	-0.017045649	0.999933887	-0.936054542	-1.095682937	0.159628395	0.442727765
ENSG00000175806	*MSRA*	3.202104911	-0.014184922	-0.024109655	0.009924733	0.999933887	-0.112537885	-0.25418542	0.141647535	0.442727765
ENSG00000096746	*HNRNPH3*	7.784271865	-0.090070152	-0.096298913	0.006228761	0.999933887	-0.27444416	-0.166747308	-0.107696852	0.442787425
ENSG00000113712	*CSNK1A1*	6.708315653	0.014450558	-0.025205135	0.039655693	0.999933887	0.070145097	0.132408439	-0.062263342	0.44290307
ENSG00000263753	*LINC00667*	2.357902607	-0.080473448	0.075553287	-0.156026736	0.999933887	0.022621482	0.151622173	-0.129000691	0.443097506
ENSG00000123384	*LRP1*	2.955063883	0.083590078	0.034740313	0.048849765	0.999933887	-0.485467528	-0.721958086	0.236490558	0.443108249
ENSG00000125846	*ZNF133*	0.675771565	-0.118376963	-0.145781443	0.027404481	0.999933887	0.120574785	-0.090338254	0.210913039	0.443474177
ENSG00000101220	*C20orf27*	1.129446902	-0.074091872	0.114151452	-0.188243324	0.999933887	0.16116641	-0.104631957	0.265798366	0.443474177
ENSG00000215867	*KRT18P57*	-0.163931597	0.085896601	0.221461583	-0.135564982	0.999933887	0.014482508	-0.417820619	0.432303127	0.443568213
ENSG00000224870	*MRPL20-AS1*	2.916357001	0.001643708	0.079425774	-0.077782066	0.999933887	-0.236586291	-0.374832627	0.138246336	0.443568213
ENSG00000053501	*USE1*	2.802086088	0.044602166	0.100404655	-0.055802489	0.999933887	0.271776644	0.421411758	-0.149635114	0.443685319
ENSG00000196586	*MYO6*	0.915469244	-0.058195836	-0.240760292	0.182564456	0.999933887	-0.085270534	0.213644252	-0.298914786	0.44390599
ENSG00000274008	*Metazoa_SRP*	2.182488175	0.454227552	0.430739685	0.023487867	0.999933887	0.974457007	1.355338204	-0.380881197	0.443911643
ENSG00000078808	*SDF4*	6.719146091	0.047296016	-0.023048541	0.070344557	0.999933887	0.176823089	0.264019656	-0.087196567	0.443911643
ENSG00000257913	*DDN-AS1*	-0.319669527	0.106785734	0.131797762	-0.025012028	0.999933887	-0.058576815	0.373513671	-0.432090486	0.443911643
ENSG00000137720	*C11orf1*	-0.344569991	-0.112877112	-0.049286567	-0.063590545	0.999933887	-0.078660422	0.297781341	-0.376441763	0.443911643
ENSG00000273749	*CYFIP1*	1.640941994	-0.062952998	-0.143996865	0.081043867	0.999933887	0.022729559	0.190016275	-0.167286716	0.443911643
ENSG00000140287	*HDC*	0.911800381	-0.007061402	0.110689268	-0.11775067	0.999933887	0.206152207	0.433266384	-0.227114177	0.443911643
ENSG00000163918	*RFC4*	0.702251066	-0.001112711	0.197431851	-0.198544562	0.999933887	0.033425324	0.274493731	-0.241068406	0.444055938
ENSG00000276386	*CNTNAP3P2*	0.025359603	0.156116047	0.032577677	0.12353837	0.999933887	-1.026371322	-1.32008656	0.293715238	0.444057477
ENSG00000187091	*PLCD1*	3.98124933	0.092409003	0.087894076	0.004514928	0.999933887	0.396567662	0.545727825	-0.149160163	0.444068658
ENSG00000280138	*AC027290.2*	6.39685346	0.076060297	0.121491213	-0.045430916	0.999933887	0.077039655	0.222848179	-0.145808524	0.444584447
ENSG00000171246	*NPTX1*	-1.034834137	0.04725763	-0.108937931	0.156195561	0.999933887	-0.347871211	-0.019974441	-0.32789677	0.444830171
ENSG00000257337	*AC068888.1*	1.311528151	0.005724096	0.05434279	-0.048618695	0.999933887	-0.042342612	0.156066635	-0.198409247	0.445051338
ENSG00000105136	*ZNF419*	0.713211923	0.003185941	0.239341259	-0.236155318	0.999933887	-0.117820387	-0.362675677	0.244855289	0.445506896
ENSG00000179583	*CIITA*	3.414458531	0.063678545	0.076546317	-0.012867771	0.999933887	-0.013517358	-0.182448581	0.168931223	0.445560785
ENSG00000134539	*KLRD1*	4.377350822	-0.019266915	-0.016895036	-0.002371879	0.999933887	-0.039987544	0.050350075	-0.090337618	0.44561203
ENSG00000010539	*ZNF200*	4.305425591	-0.081456257	-0.021781423	-0.059674834	0.999933887	-0.728711638	-0.856568661	0.127857023	0.445952194
ENSG00000225886	*AL445490.1*	0.323223597	-0.152438368	-0.18205862	0.029620252	0.999933887	-0.357651573	-0.693979762	0.33632819	0.445952194
ENSG00000122696	*SLC25A51*	2.035821308	0.071935781	0.016359025	0.055576756	0.999933887	-0.015844249	-0.180170627	0.164326378	0.445952194
ENSG00000091831	*ESR1*	0.579519491	-0.081950647	0.135006459	-0.216957106	0.999933887	-0.204747562	0.102435045	-0.307182606	0.446355635
ENSG00000143437	*ARNT*	4.906049245	-0.085055785	0.021573097	-0.106628881	0.999933887	-0.447759186	-0.549529217	0.101770031	0.446623553
ENSG00000118363	*SPCS2*	3.789917313	-0.046598663	-0.11511277	0.068514107	0.999933887	0.000398947	0.121433033	-0.121034086	0.446623553
ENSG00000088179	*PTPN4*	3.815375606	-0.017330594	-0.072917151	0.055586557	0.999933887	-0.192792336	0.00742221	-0.200214547	0.446623553
ENSG00000283646	*LINC02009*	3.463274605	0.039785994	0.266991742	-0.227205747	0.999933887	0.723118726	0.864839388	-0.141720662	0.446879949
ENSG00000230882	*AC005077.4*	-1.721416287	0.259957031	0.122293093	0.137663938	0.999933887	1.017496176	1.407771499	-0.390275323	0.447118021
ENSG00000033050	*ABCF2*	1.179796829	-0.019844323	0.105389783	-0.125234106	0.999933887	0.038152138	0.209904938	-0.1717528	0.447244235
ENSG00000136111	*TBC1D4*	3.687116683	-0.06360019	-0.102340099	0.038739909	0.999933887	-0.082408996	0.042288178	-0.124697175	0.447343517
ENSG00000117228	*GBP1*	7.878296784	0.042753646	-0.048235638	0.090989284	0.999933887	0.727488079	0.863731728	-0.136243648	0.447343517
ENSG00000117480	*FAAH*	1.339697287	0.037990628	0.07180691	-0.033816282	0.999933887	0.316981301	0.52100356	-0.204022259	0.447352545
ENSG00000235576	*LINC01871*	1.141566749	-0.062316901	-0.166070806	0.103753905	0.999933887	0.083221668	0.300737891	-0.217516222	0.447471547
ENSG00000102780	*DGKH*	2.008673048	-0.047814531	-0.058066502	0.01025197	0.999933887	-0.30566228	-0.017476602	-0.288185678	0.447527534
ENSG00000169994	*MYO7B*	1.909069486	0.074775077	0.045226364	0.029548713	0.999933887	-0.337892309	-0.666232689	0.32834038	0.44766618
ENSG00000124920	*MYRF*	-0.284612911	-0.06057879	0.04628862	-0.10686741	0.999933887	0.00136827	-0.359476493	0.360844763	0.447698848
ENSG00000124155	*PIGT*	4.788859712	-0.055640342	-0.055516893	-0.00012345	0.999933887	-0.128482781	-0.214696668	0.086213886	0.447712705
ENSG00000259712	*AC023906.5*	1.297760944	0.000261069	0.054798205	-0.054537137	0.999933887	1.275646307	1.532117199	-0.256470892	0.447712705
ENSG00000242473	*KIR2DP1*	-1.290520394	-0.085128452	0.23170371	-0.316832163	0.999933887	1.40318492	1.731460744	-0.328275824	0.447763788
ENSG00000270629	*NBPF14*	4.690473129	-0.056233136	-0.032547087	-0.023686049	0.999933887	0.216249465	0.326077242	-0.109827777	0.447806276
ENSG00000105372	*RPS19*	7.6322358	0.027826255	-0.027437818	0.055264073	0.999933887	0.197865623	0.298069517	-0.100203894	0.447980062
ENSG00000109854	*HTATIP2*	5.433476625	-0.023375435	-0.031985164	0.008609729	0.999933887	-0.237920894	-0.334002554	0.096081661	0.447980062
ENSG00000111252	*SH2B3*	6.121529759	-0.021325473	0.015734855	-0.037060328	0.999933887	0.071125072	-0.065282645	0.136407717	0.447980062
ENSG00000140365	*COMMD4*	4.085938225	0.038367657	0.030618777	0.007748881	0.999933887	0.42526471	0.528807007	-0.103542297	0.448372355

ENSG00000268279	*AC090004.1*	0.782960789	-0.028137983	-0.25440607	0.226268087	0.999933887	-0.375392636	-0.585454551	0.210061915	0.448372355
ENSG00000122971	*ACADS*	1.178924913	0.091400146	-0.152786619	0.244186765	0.999933887	0.050345966	-0.177660762	0.228006728	0.448488656
ENSG00000093144	*ECHDC1*	3.363186262	0.026514823	-0.041957763	0.068472585	0.999933887	-0.167523993	-0.010459106	-0.157064887	0.448488656
ENSG00000175489	*LRRC25*	8.11325279	-0.089675163	-0.0670848	-0.022590363	0.999933887	-0.827450314	-0.695647354	-0.13180296	0.448867353
ENSG00000229391	*HLA-DRB6*	0.557158333	0.130122123	0.171690562	-0.041568438	0.999933887	0.993698212	1.322174851	-0.328476639	0.448867353
ENSG00000090061	*CCNK*	4.589688137	0.016054399	0.025218079	-0.00916368	0.999933887	0.119456473	0.215088895	-0.095632422	0.448931517
ENSG00000003436	*TFPI*	-1.597029895	-0.124357056	-0.475519784	0.351162728	0.999933887	-0.422551505	-0.773613104	0.351061599	0.449065114
ENSG00000145191	*EIF2B5*	4.080412937	-0.089661707	0.048126726	-0.137788433	0.999933887	0.055233347	-0.042638589	0.097871936	0.449125714
ENSG00000130731	*METTL26*	2.600163822	0.103917331	-0.041041813	0.144959144	0.999933887	0.148921235	0.00381321	0.145108025	0.449708799
ENSG00000105926	*MPP6*	0.636394756	0.13367788	0.042239234	0.091438646	0.999933887	-0.097176529	0.205388766	-0.302565295	0.449708799
ENSG00000158467	*AHCYL2*	2.061444824	0.032437518	0.019072802	0.013364716	0.999933887	-0.331861921	-0.509804062	0.177942141	0.449708799
ENSG00000160753	*RUSC1*	1.910294837	0.01631173	-0.022092215	0.038403945	0.999933887	0.178702367	-0.022858811	0.201561178	0.449708799
ENSG00000105321	*CCDC9*	6.56064689	0.089815633	0.098757345	-0.008941712	0.999933887	0.285699703	0.425546457	-0.139846754	0.449752785
ENSG00000134905	*CARS2*	5.186100015	0.024515956	0.053098379	-0.028582423	0.999933887	-0.129268838	-0.21940898	0.090140141	0.449982046
ENSG00000085231	*AK6*	0.99473922	-0.022546264	-0.059649463	0.037103199	0.999933887	-0.162884043	0.025961736	-0.188845779	0.449982046
ENSG00000185304	*RGPD2*	0.723880921	-0.231312672	-0.217912562	-0.013400111	0.999933887	0.511012325	0.690567757	-0.179555431	0.450238208
ENSG00000149809	*TM7SF2*	0.683909688	0.116561948	0.171253835	-0.054691887	0.999933887	0.031449053	-0.197844937	0.229293989	0.450238208
ENSG00000153147	*SMARCA5*	5.769495029	-0.028347837	-0.137623605	0.109275768	0.999933887	-0.201723282	-0.019459281	-0.182264	0.450238208
ENSG00000146416	*AIG1*	3.807493733	-0.018208615	0.070785663	-0.088994278	0.999933887	0.825293026	0.948636142	-0.123343115	0.450238208
ENSG00000179750	*APOBEC3B*	2.492388996	0.020597906	0.048596824	-0.027998919	0.999933887	0.02654241	0.208133005	-0.181590595	0.450238208
ENSG00000074201	*CLNS1A*	2.533499582	-0.031521316	-0.062049989	0.030528673	0.999933887	0.052683943	0.200915935	-0.148231993	0.450338827
ENSG00000173327	*MAP3K11*	8.276404791	0.076549113	0.05376662	0.022782493	0.999933887	0.490434617	0.59524132	-0.104806703	0.450695741
ENSG00000180902	*D2HGDH*	2.48769711	0.073678873	0.084723981	-0.011045108	0.999933887	0.210170984	0.371660863	-0.161489878	0.450819875
ENSG00000113140	*SPARC*	0.689761376	0.111405796	0.263892302	-0.152486506	0.999933887	0.010736278	0.282887753	-0.272151475	0.450819875
ENSG00000189376	*C8orf76*	-0.076352423	-0.092826996	0.156499491	-0.249326488	0.999933887	-0.278483259	-0.005205938	-0.273277321	0.450819875
ENSG00000108352	*RAPGEFL1*	3.387900882	-0.005561729	0.088604174	-0.094165903	0.999933887	-0.215603898	-0.337121509	0.121517612	0.450819875
ENSG00000196126	*HLA-DRB1*	5.750035095	0.002821558	0.022727309	-0.019905751	0.999933887	0.610118255	0.817355259	-0.207237005	0.450819875
ENSG00000186056	*MATN1-AS1*	0.950307025	0.135514348	0.100196421	0.035317927	0.999933887	0.202675842	0.490266256	-0.287590415	0.451038059
ENSG00000138134	*STAMBPL1*	1.871369996	-0.020212585	-0.026244457	0.006031871	0.999933887	-0.009521007	0.146526336	-0.156047343	0.451038059
ENSG00000176473	*WDR25*	1.806532398	0.003187325	-0.046171401	0.049358726	0.999933887	-0.308208614	-0.468774663	0.160566049	0.451038059
ENSG00000116954	*RRAGC*	6.593941773	-0.075778759	-0.053584494	-0.022194265	0.999933887	0.254981182	0.352964429	-0.097983247	0.451109072
ENSG00000116198	*CEP104*	3.249894459	0.065747317	-0.006948767	0.072696084	0.999933887	-0.135234746	-0.269089389	0.133854644	0.451109072
ENSG00000259379	*MTND5P32*	4.410233307	0.366135632	0.695346795	-0.329211163	0.999933887	2.043678727	2.424605132	-0.380926405	0.451591653
ENSG00000144893	*MED12L*	0.080809264	-0.232976854	-0.140429734	-0.092547119	0.999933887	-0.421090303	-0.080899714	-0.340190589	0.451591653
ENSG00000084112	*SSH1*	6.297950829	-0.063407954	0.021289506	-0.08469746	0.999933887	-0.455973158	-0.558783187	0.102810029	0.451591653
ENSG00000160710	*ADAR*	9.51326002	-0.034545001	-0.057393329	0.022848328	0.999933887	0.211173044	0.28190391	-0.070730866	0.451792869
ENSG00000170160	*CCDC144A*	-2.872688351	-0.060693446	0.35520759	-0.415901036	0.999933887	-0.460881934	-0.010600243	-0.450281691	0.45194673
ENSG00000119004	*CYP20A1*	3.054528252	-0.055491527	0.041076275	-0.096567802	0.999933887	-0.103002922	0.015906275	-0.118909197	0.452027914
ENSG00000095370	*SH2D3C*	6.829291587	0.000822902	0.022875963	-0.022053061	0.999933887	-0.408993858	-0.513911032	0.104917174	0.452027914
ENSG00000140859	*KIFC3*	2.930068825	0.009740369	0.171212362	-0.161471993	0.999933887	0.023069884	0.149831196	-0.126761311	0.452426215
ENSG00000173013	*CCDC96*	0.167547791	0.009134862	0.088685693	-0.079550831	0.999933887	-0.439619045	-0.769579852	0.329960808	0.452426215
ENSG00000122484	*RPAP2*	1.838091447	0.065802184	-0.076685528	0.142487712	0.999933887	-0.226581784	0.002987059	-0.229568843	0.452453768
ENSG00000101255	*TRIB3*	5.94181048	-0.086598101	-0.070192349	-0.016405753	0.999933887	0.133113827	0.24199159	-0.108877763	0.452530869
ENSG00000144736	*SHQ1*	0.904197099	0.075944435	-0.233426569	0.309371003	0.999933887	-0.022898947	0.203112297	-0.226011245	0.452530869
ENSG00000184432	*COPB2*	5.53560194	-0.079364795	-0.227167765	0.14780297	0.999933887	-0.017130532	0.078137114	-0.095267646	0.452716301
ENSG00000103187	*COTL1*	9.67635487	-0.000888757	-0.017694072	0.016805315	0.999933887	-0.051991253	-0.14978335	0.097792097	0.452908843
ENSG00000273783	*AL136040.1*	-0.201222567	0.240958355	0.173535286	0.067423069	0.999933887	1.195747079	0.834129546	0.361617533	0.452950105
ENSG00000100802	*C14orf93*	1.734512719	0.084661334	-0.099440755	0.184102089	0.999933887	-0.434439049	-0.650660931	0.216221882	0.452950105
ENSG00000100243	*CYB5R3*	5.399114331	0.072912972	0.070165923	0.002747049	0.999933887	0.027663539	0.11121577	-0.083552231	0.453198035
ENSG00000282164	*PEG13*	1.034498208	0.099512022	0.195294323	-0.0957823	0.999933887	0.121585271	-0.160607228	0.282192499	0.453198035
ENSG00000143185	*XCL2*	0.911381038	-0.088447454	0.014874897	-0.103322351	0.999933887	0.028238227	0.234379525	-0.206141298	0.453198035
ENSG00000074966	*TXK*	1.796382369	-0.068433026	-0.088645531	0.020212505	0.999933887	-0.018207016	0.161133407	-0.179340423	0.453198035
ENSG00000196116	*TDRD7*	4.628306984	0.005590101	-0.010155377	0.015745478	0.999933887	-0.176958894	-0.264938987	0.087980092	0.453198035
ENSG00000196704	*AMZ2*	4.590969203	-0.000950959	-0.012720933	0.011769974	0.999933887	-0.088092664	-0.191205687	0.103113023	0.453198035
ENSG00000252135	*RNU1-155P*	-2.466719317	-0.105209764	0.041669813	-0.146879577	0.999933887	-0.23294869	0.236173668	-0.469122358	0.453219436
ENSG00000175066	*GK5*	3.33522643	-0.077910689	0.108292178	-0.186202867	0.999933887	-0.137312338	0.005250196	-0.142562534	0.45322448
ENSG00000267554	*AC015911.7*	0.983529684	-0.110139625	-0.414076216	0.303936591	0.999933887	-0.388311422	-0.11602093	-0.272290492	0.45322448
ENSG00000166669	*ATF7IP2*	1.460740894	-0.069914407	-0.185818227	0.11590382	0.999933887	0.241425941	0.462831943	-0.221406003	0.45322448
ENSG00000140319	*SRP14*	6.221441416	0.007237449	-0.106355881	0.113593329	0.999933887	0.163808495	0.252736635	-0.08892814	0.453604357
ENSG00000288066	*AC097448.1*	-0.375706354	0.1126811	-0.07454065	0.187221749	0.999933887	-0.24419793	0.15834724	-0.40254517	0.453619896
ENSG00000232528	*AL109809.1*	2.622147491	0.018462587	0.073974702	-0.055512115	0.999933887	0.007428389	0.140356401	-0.132928012	0.453619896
ENSG00000137288	*UQCC2*	0.908761419	0.023452109	-0.11346651	0.136918619	0.999933887	0.168097969	-0.044810081	0.212908051	0.453654798
ENSG00000095066	*HOOK2*	2.848804052	-0.007307326	-0.086175311	0.078867985	0.999933887	0.375366344	0.523710684	-0.148344341	0.453795926
ENSG00000114391	*RPL24*	6.932504385	0.018832904	0.002430861	0.016402043	0.999933887	0.158964821	0.257627651	-0.098662831	0.453958048
ENSG00000139055	*ERP27*	0.998181778	0.00982842	-0.009070363	0.018898783	0.999933887	-0.171248849	-0.517258189	0.34600934	0.454270851
ENSG00000132530	*XAF1*	4.157199497	-0.018725993	-0.12273813	0.104012137	0.999933887	-0.306294194	-0.457323482	0.151029288	0.454504022
ENSG00000160867	*FGFR4*	-0.384636376	0.161575545	0.057234206	0.104341339	0.999933887	-0.145790395	-0.560289059	0.414498664	0.454816926
ENSG00000273319	*AC058791.1*	2.489014556	0.118558156	0.219872156	-0.101313999	0.999933887	0.017587782	-0.323269912	0.340857695	0.454894671
ENSG00000111450	*STX2*	2.026306843	-0.050195607	0.058809697	-0.109005304	0.999933887	0.012726037	-0.14194585	0.154671886	0.454894671
ENSG00000107175	*CREB3*	3.748857259	-0.013818943	-0.02737638	0.013557437	0.999933887	0.666556048	0.799879377	-0.133323329	0.454944507
ENSG00000182117	*NOP10*	7.712924609	0.045884954	-0.03640987	0.082294824	0.999933887	0.065353771	0.15647167	-0.091117899	0.455095228
ENSG00000285492	*AL356417.3*	3.720341545	-0.089920033	0.208975587	-0.29889562	0.999933887	3.861821037	4.163468757	-0.30164772	0.45510873
ENSG00000125375	*DMAC2L*	1.349309127	-0.044497481	0.191497112	-0.235994593	0.999933887	-0.183633523	0.028568934	-0.212202457	0.455172773
ENSG00000259374	*NDUFB4P11*	-0.521235615	0.265378601	0.452790101	-0.1874115	0.999933887	0.973855778	1.461321224	-0.487465446	0.45525749
ENSG00000176978	*DPP7*	5.979710445	0.05919994	0.059131198	6.8741e-05	0.999933887	-0.030874676	-0.163291579	0.132416903	0.45525749
ENSG00000074590	*NUAK1*	-0.920192729	-0.102501219	-0.054020524	-0.048480696	0.999933887	0.041830817	-0.308556231	0.350387047	0.45525749
ENSG00000168497	*CAVIN2*	-0.601358929	0.054512437	0.110755237	-0.0562428	0.999933887	-0.038608348	0.386905435	-0.425513783	0.45525749
ENSG00000145687	*SSBP2*	2.427069438	-0.004834485	0.044182782	-0.049017267	0.999933887	0.05729132	-0.093404639	0.150695959	0.45525749
ENSG00000280228	*AC079753.1*	-0.24812537	0.585328041	0.644199944	-0.058871903	0.999933887	2.361276373	2.76688332	-0.405606947	0.455363178
ENSG00000182149	*IST1*	7.821176376	-0.053866909	-0.053559931	-0.000306979	0.999933887	0.058000851	0.128724106	-0.070723255	0.455363178
ENSG00000136695	*IL36RN*	-2.369888072	-0.200623561	-0.513784362	0.313160801	0.999933887	0.994262985	1.301260667	-0.306997682	0.455363178
ENSG00000022840	*RNF10*	7.5623103	0.007110274	-0.004623455	0.011733729	0.999933887	-0.155756767	-0.071609799	-0.084146969	0.455363178
ENSG00000152520	*PAN3*	6.861187645	-0.064493618	-0.000944844	-0.063548774	0.999933887	-0.395414942	-0.28050994	-0.114905003	0.455678816
ENSG00000224442	*RPL4P7*	-0.558513495	0.356361529	0.240385337	0.115976192	0.999933887	-0.274969293	-0.7785015	0.503532207	0.455720288
ENSG00000179914	*ITLN1*	-0.900894213	-0.412434416	0.014781232	-0.427215647	0.999933887	-0.033302806	0.216279316	-0.249582121	0.455756536
ENSG00000100938	*GMPR2*	6.398750329	0.049408927	-0.023805531	0.073214458	0.999933887	0.227936245	0.152771167	0.075165079	0.455756536
ENSG00000166681	*BEX3*	-0.13179427	-0.054478666	0.019549226	-0.074027892	0.999933887	0.357262195	0.006780636	0.350481559	0.455756536
ENSG00000240666	*MME-AS1*	-0.620020761	-0.023035884	-0.134022106	0.110986222	0.999933887	-1.591098759	-0.92678675	-0.664312009	0.455756536
ENSG00000175691	*ZNF77*	0.62079604	-0.012742929	-0.126259802	0.113516873	0.999933887	0.285612011	0.026712284	0.258899727	0.455756536
ENSG00000132383	*RPA1*	3.604876407	-0.000145478	-0.046080272	0.045934794	0.999933887	-0.012094837	-0.119517332	0.107422495	0.455756536
ENSG00000204792	*LINC01291*	-2.799989166	0.260775074	0.294098269	-0.033323195	0.999933887	-0.357048498	0.19072187	-0.547770368	0.455932147
ENSG00000125630	*POLR1B*	2.527953407	-0.117946352	0.000695053	-0.118641405	0.999933887	0.202198416	0.33823272	-0.136034304	0.456131957
ENSG00000228302	*AL512770.1*	0.783247697	-0.086090235	-0.046353781	-0.039736454	0.999933887	-0.144417084	-0.473148226	0.328731142	0.456347758
ENSG00000105287	*PRKD2*	7.318006007	0.039327669	0.019297094	0.020030575	0.999933887	-0.31197417	-0.402614318	0.090640148	0.456361743
ENSG00000115507	*OTX1*	-1.418469508	-0.080891519	0.063160442	-0.144051961	0.999933887	-1.162854062	-1.857488724	0.694634661	0.456372574
ENSG00000117592	*PRDX6*	4.469038489	-0.002699308	-0.009008572	0.006309264	0.999933887	0.286264432	0.177919846	0.108344586	0.456559861
ENSG00000149925	*ALDOA*	4.466751056	0.076877874	0.042521306	0.034356568	0.999933887	-0.194239601	-0.311907321	0.11766772	0.456603958
ENSG00000154845	*PPP4R1*	7.545833013	-0.053125992	-0.058907735	0.005781743	0.999933887	-1.109714589	-1.229028025	0.119313435	0.456603958
ENSG00000167081	*PBX3*	2.709381308	-0.015357101	-0.032146342	0.016789241	0.999933887	-0.379859565	-0.516519044	0.136659479	0.456603958
ENSG00000238045	*AC009133.1*	1.220292678	0.020814568	-0.021951007	0.042765576	0.999933887	0.165737595	-0.045731839	0.211469434	0.456603958
ENSG00000108342	*CSF3*	-1.916670986	-0.161945523	0.39691269	-0.558858213	0.999933887	6.277553593	7.093363477	-0.815809884	0.4566215
ENSG00000147118	*ZNF182*	2.77350099	-0.135626258	-0.243112786	0.107486529	0.999933887	0.066315034	0.228305216	-0.161990182	0.456764138
ENSG00000197670	*AL157838.1*	1.876109995	-0.233276713	-0.381725796	0.148449083	0.999933887	-0.062596612	-0.230868419	0.168271807	0.456901055
ENSG00000197345	*MRPL21*	2.181252821	0.017779413	0.108628716	-0.090849303	0.999933887	0.411399883	0.604272888	-0.192873005	0.456999593
ENSG00000204356	*NELFE*	5.316793848	0.04667766	0.046960927	-0.000283268	0.999933887	0.105291706	0.189338113	-0.084046407	0.45702608

ENSG00000148450	*MSRB2*	2.139587578	0.064408819	-0.053886697	0.118295516	0.999933887	-0.126521779	-0.358084931	0.231563153	0.457097987
ENSG00000117010	*ZNF684*	-0.209233911	-0.080377744	-0.111307551	0.030929807	0.999933887	0.200874452	-0.186128784	0.387003236	0.457097987
ENSG00000168890	*TMEM150A*	1.206889977	0.060443285	0.048494644	0.011948641	0.999933887	0.05880626	-0.141987437	0.200793697	0.457140852
ENSG00000086548	*CEACAM6*	-0.276148934	0.130837782	0.190288887	-0.059451105	0.999933887	-0.357102876	0.015961645	-0.37306452	0.457252813
ENSG00000184281	*TSSC4*	4.864818007	0.01766396	0.063007558	-0.045343598	0.999933887	-0.134741014	-0.266974979	0.132233965	0.457364638
ENSG00000170802	*FOXN2*	4.6722822	-0.057227533	-0.160109054	0.102881521	0.999933887	-0.686169471	-0.473239005	-0.212930467	0.457700483
ENSG00000035141	*FAM136A*	2.557850445	-0.111570682	0.074965218	-0.1865359	0.999933887	0.089838364	0.222581141	-0.132742777	0.457763804
ENSG00000111364	*DDX55*	2.460875972	-0.0533059	-0.12136755	0.06806165	0.999933887	0.090701596	-0.054977546	0.145679142	0.457763804
ENSG00000168300	*PCMTD1*	5.089186466	-0.025628944	-0.005654811	-0.019974134	0.999933887	-0.648885512	-0.517962725	-0.130922786	0.457763804
ENSG00000178381	*ZFAND2A*	4.76363391	0.046564306	0.026871265	0.019693042	0.999933887	0.334239102	0.427681278	-0.093442177	0.458063003
ENSG00000271870	*AC024060.2*	-0.083424042	-0.134606345	-0.157615777	0.023009432	0.999933887	0.064982941	-0.215783842	0.280766782	0.458063003
ENSG00000280027	*AC007342.9*	0.358524997	-0.140605191	0.020260957	-0.160866148	0.999933887	-1.374225722	-1.876149659	0.501923937	0.458099675
ENSG00000113845	*TIMMDC1*	2.715641318	-0.015436853	0.131573462	-0.147010315	0.999933887	0.072321724	0.197286207	-0.124964483	0.458099675
ENSG00000196369	*SRGAP2B*	0.945063625	0.011156088	0.187563704	-0.176407616	0.999933887	-0.212328569	0.059229737	-0.271558305	0.458099675
ENSG00000062485	*CS*	5.647696947	-0.042637804	-0.023865415	-0.01877239	0.999933887	0.186337175	0.271204669	-0.084867494	0.458143699
ENSG00000103353	*UBFD1*	2.459409847	-0.02071771	0.095172955	-0.115890665	0.999933887	0.046849242	-0.077361171	0.124210414	0.458197163
ENSG00000026036	*RTEL1-TNFRS*	0.183212274	-0.124050448	0.101458638	-0.225509086	0.999933887	0.164313398	-0.150236477	0.314549875	0.458366285
ENSG00000173960	*UBXN2A*	1.768116836	-0.047214514	-0.057444286	0.010229772	0.999933887	0.146943188	0.351023576	-0.204080388	0.458366285
ENSG00000101004	*NINL*	-1.253962586	-0.25774707	0.337691799	-0.595438869	0.999933887	-0.044500519	0.326834262	-0.371334781	0.458863068
ENSG00000234494	*SP2-AS1*	0.364574442	0.193489232	0.26763518	-0.074145949	0.999933887	0.166274087	0.444025804	-0.277751717	0.45887709
ENSG00000241983	*RN7SL566P*	-0.280576526	0.056645112	-0.173053022	0.229698134	0.999933887	0.005841931	0.441254068	-0.435412137	0.459176637
ENSG00000086200	*IPO11*	3.763767833	-0.022556759	-0.002082918	-0.02047384	0.999933887	-0.180709515	-0.061287849	-0.119421666	0.459176637
ENSG00000146701	*MDH2*	4.17852923	-0.028545504	-0.071808451	0.043262947	0.999933887	-0.112922606	-0.220882763	0.107960156	0.459246486
ENSG00000173213	*TUBB8B*	-1.762197812	0.054332579	-0.193409847	0.247742426	0.999933887	-1.700996457	-1.367573253	-0.333423204	0.459304632
ENSG00000129450	*SIGLEC9*	4.630073569	-0.012873886	-0.076118634	0.063244748	0.999933887	0.407992847	0.577630041	-0.169637193	0.45934096
ENSG00000055163	*CYFIP2*	8.363060834	0.021355631	0.019318027	0.002037604	0.999933887	-0.300165547	-0.219373989	-0.080791558	0.459406799
ENSG00000006194	*ZNF263*	5.365548633	0.000431395	0.03067381	-0.030242414	0.999933887	-0.060534878	-0.169379355	0.108844478	0.459446352
ENSG00000119820	*YIPF4*	5.102710299	-0.062140496	0.043635346	-0.105775842	0.999933887	-0.320830577	-0.238284376	-0.082546201	0.459485106
ENSG00000103522	*IL21R*	3.14566029	-0.065617468	0.054535705	-0.120153173	0.999933887	0.341580257	0.48674529	-0.145165033	0.459485106
ENSG00000169967	*MAP3K2*	8.747622876	-0.055655111	-0.104929056	0.049273944	0.999933887	-0.993838437	-0.806680827	-0.18715761	0.459485106
ENSG00000118655	*DCLRE1B*	0.478435483	0.068294425	0.101605236	-0.03331081	0.999933887	0.113595498	-0.231178492	0.34477399	0.459485106
ENSG00000136878	*USP20*	6.312013666	0.016125402	0.030863288	-0.014737886	0.999933887	-0.210950678	-0.310074018	0.09912334	0.459624159
ENSG00000259475	*AC036108.1*	-0.298813687	0.006221405	0.289387783	-0.283166379	0.999933887	-0.807817334	-1.22798862	0.420171286	0.459761284
ENSG00000279207	*AC015813.6*	1.554607278	0.026579926	-0.06766512	0.094245047	0.999933887	-0.144614982	-0.391973621	0.247358639	0.459879271
ENSG00000124608	*AARS2*	1.824036607	-0.117264251	0.179853623	-0.297117874	0.962755146	0.147955247	-0.002829541	0.150784789	0.459896559
ENSG00000106404	*CLDN15*	3.524631817	0.077937567	-0.012853523	0.09079109	0.999933887	-0.01762021	-0.13017328	0.11255307	0.459896559
ENSG00000267369	*TAF5LP1*	1.87493884	-0.036488869	0.188627074	-0.225115943	0.999933887	-0.070488045	-0.243491815	0.17300377	0.459896559
ENSG00000160563	*MED27*	1.975244553	0.025465012	0.010383327	0.015081685	0.999933887	0.217684264	0.393578866	-0.175894602	0.459896559
ENSG00000065621	*GSTO2*	2.807777042	0.201662482	0.210581652	-0.00891917	0.999933887	0.592881836	0.808952202	-0.216070365	0.459968799
ENSG00000149527	*PLCH2*	2.858316697	0.102082098	0.259411736	-0.157329638	0.999933887	0.067545092	0.209394165	-0.141849073	0.459968799
ENSG00000101558	*VAPA*	7.011922619	-0.028884256	0.014639969	-0.043524224	0.999933887	-0.109340197	-0.037902883	-0.071437315	0.459968799
ENSG00000246130	*AC107959.2*	1.304157169	-0.092846159	0.049651953	-0.142498112	0.999933887	-1.208616782	-1.480333426	0.271716645	0.459968799
ENSG00000025770	*NCAPH2*	5.035411403	0.060379654	0.095780171	-0.035400517	0.999933887	0.280894341	0.380356566	-0.099462225	0.459999388
ENSG00000160223	*ICOSLG*	-0.444391748	0.102366218	0.212657164	-0.110290946	0.999933887	1.207683667	1.501067782	-0.293384115	0.460184206
ENSG00000140107	*SLC25A47*	-1.398445758	0.054142527	-0.078678457	0.132820985	0.999933887	1.149192067	0.413286177	0.73590589	0.460184206
ENSG00000123104	*ITPR2*	5.000225622	-0.004152388	-0.044237359	0.040084971	0.999933887	-0.084743117	0.103915889	-0.188659005	0.460184206
ENSG00000167085	*PHB*	1.879164641	0.046458063	-0.020119115	0.066577178	0.999933887	0.284059567	0.439603155	-0.155543589	0.460484496
ENSG00000132964	*CDK8*	1.420214949	-0.032295421	-0.054797127	0.022501707	0.999933887	-0.078772709	-0.278595156	0.199822447	0.460484496
ENSG00000133641	*C12orf29*	0.960382291	-0.120610803	-0.076451706	-0.044159097	0.999933887	-0.052725661	0.217986642	-0.270712303	0.460731085
ENSG00000149257	*SERPINH1*	0.010010658	0.196946924	0.043200512	0.153746412	0.999933887	0.125203247	0.352361371	-0.227158124	0.461117044
ENSG00000268087	*AC008764.2*	-0.511119937	-0.151645046	-0.266204333	0.114559287	0.999933887	-0.225938747	-0.52886084	0.302922093	0.461117044
ENSG00000124177	*CHD6*	4.205259807	0.047068829	-0.032893545	0.079962374	0.999933887	-0.000845048	0.13501273	-0.135857778	0.461117044
ENSG00000164399	*IL3*	-2.830725274	0.115218025	-0.003591772	0.118809797	0.999933887	0.594638792	-0.247585094	0.842223885	0.461117044
ENSG00000257335	*MGAM*	8.356101024	-0.014752025	-0.046738137	0.031986112	0.999933887	0.281101304	0.367905586	-0.086804282	0.461117044
ENSG00000275764	*AC092747.4*	1.524384534	-0.027402629	-0.075543224	0.048140595	0.999933887	-0.411762423	-0.701154684	0.28939226	0.461117044
ENSG00000132182	*NUP210*	5.558907176	0.026287807	0.032489603	-0.006201796	0.999933887	0.072466174	-0.002652512	0.075118686	0.461409024
ENSG00000148488	*ST8SIA6*	-0.223669413	0.283755447	0.143043534	0.140711913	0.999933887	-0.16811632	0.080753263	-0.248869582	0.461530768
ENSG00000259883	*EHD4-AS1*	-0.850926224	0.149320659	0.296458876	-0.147138216	0.999933887	0.206492704	0.764412163	-0.55791946	0.461530768
ENSG00000256628	*ZBTB11-AS1*	-0.022823308	0.150574049	0.121651905	0.028922145	0.999933887	0.184658927	0.503257168	-0.318598241	0.461530768
ENSG00000135617	*PRADC1*	-0.140150125	0.125802852	-0.06648045	0.192283302	0.999933887	0.124076363	-0.263445337	0.3875217	0.461530768
ENSG00000109452	*INPP4B*	3.188704336	-0.065432917	-0.153755847	0.088322929	0.999933887	-0.194298841	0.01316255	-0.20746139	0.461530768
ENSG00000286864	*AC012076.1*	0.829171587	0.073437506	0.033450001	0.039987505	0.999933887	-0.648881985	-1.138055442	0.489173456	0.461530768
ENSG00000126838	*PZP*	1.034177133	0.094974222	0.337693754	-0.242719532	0.999933887	-0.108511987	0.218125361	-0.326637348	0.461530768
ENSG00000211667	*IGLV3-12*	-2.252569817	0.124448791	-0.181346057	0.305794848	0.999933887	-0.206105545	-0.557028196	0.35092265	0.461530768
ENSG00000197265	*GTF2E2*	3.287779772	0.035670795	0.102661425	-0.06699063	0.999933887	-0.155771678	-0.279793118	0.12402144	0.461530768
ENSG00000102225	*CDK16*	5.92940624	0.019688255	0.084629277	-0.064941022	0.999933887	0.217673934	0.304386732	-0.086712798	0.461530768
ENSG00000105707	*HPN*	-1.040715543	0.074934606	0.107194185	-0.032259579	0.999933887	3.597086467	4.158541478	-0.561455012	0.461530768
ENSG00000186577	*SMIM29*	6.452903048	0.00041874	-0.070393209	0.070811949	0.999933887	0.282169226	0.175042883	0.107126342	0.461559664
ENSG00000173915	*ATP5MD*	3.533595555	0.019827402	0.034091548	-0.014264146	0.999933887	-0.027071222	0.117813226	-0.144884448	0.462447268
ENSG00000115317	*HTRA2*	3.93742306	0.098671429	0.118827559	-0.02015613	0.999933887	0.440099643	0.308241364	0.131858279	0.462572513
ENSG00000259366	*AC108449.2*	0.989874234	0.023006749	-0.206074607	0.229081356	0.999933887	0.231931492	0.452488996	-0.220557504	0.462654333
ENSG00000237232	*ZNF295-AS1*	0.035841628	0.245975868	0.192471381	0.053504487	0.999933887	0.636913759	0.904550262	-0.267636503	0.46270627
ENSG00000211710	*TRBV4-1*	0.174289754	-0.124236464	-0.116282196	-0.007954268	0.999933887	0.235481408	-0.011438313	0.246919721	0.46270627
ENSG00000163599	*CTLA4*	1.919326338	-0.089714251	0.236772546	-0.326486797	0.944094537	-0.000425823	-0.163138763	0.162712941	0.462722449
ENSG00000286196	*AL162586.2*	2.39579799	-0.083084978	0.01272679	-0.095811768	0.999933887	-0.250103315	-0.411266292	0.161162977	0.462722449
ENSG00000239382	*ALKBH6*	0.448631629	0.180813593	-0.229663447	0.41047704	0.999933887	-0.012402106	-0.288274737	0.275872631	0.462960334
ENSG00000141499	*WRAP53*	2.113443503	0.101049674	0.027063556	0.073986118	0.999933887	0.271380136	0.096337469	0.175042668	0.462990667
ENSG00000189068	*VSTM1*	2.095341738	0.02164532	-0.033628343	0.055273663	0.999933887	-0.114904857	-0.297172198	0.182267341	0.462997864
ENSG00000149115	*TNKS1BP1*	1.730348951	0.066417462	0.023411297	0.043006165	0.999933887	0.395527491	0.573693603	-0.178166112	0.463274892
ENSG00000183060	*LYSMD4*	1.461292441	-0.042374078	-0.171049617	0.128675539	0.999933887	-0.001731351	-0.169516014	0.167784662	0.463274892
ENSG00000114166	*KAT2B*	5.891632166	-0.000866086	0.017230816	-0.018096902	0.999933887	-0.541615853	-0.63947291	0.097857057	0.46371367
ENSG00000140575	*IQGAP1*	9.388004539	-0.058742886	-0.108559925	0.049817039	0.999933887	-0.440561936	-0.326639234	-0.113922702	0.463963609
ENSG00000261423	*TMEM202-AS1*	1.370169746	0.114192359	0.194265502	-0.080073143	0.999933887	0.537074216	0.291006124	0.246068092	0.464170965
ENSG00000230076	*RPL10P6*	0.56956171	0.074077821	0.317222614	-0.243144793	0.999933887	0.181334713	-0.126342685	0.307677397	0.464264373
ENSG00000119977	*TCTN3*	1.487564917	-0.032991918	-0.107686451	0.074694534	0.999933887	0.152279949	-0.039198984	0.191478933	0.464264373
ENSG00000246451	*AL049840.2*	2.928196324	0.101002404	0.109496458	-0.008494054	0.999933887	-0.283860769	-0.167947885	-0.115912884	0.464265661
ENSG00000184719	*RNLS*	0.289763574	0.087119813	0.113568608	-0.026448794	0.999933887	0.567989831	0.205927732	0.362062099	0.464265661
ENSG00000168724	*DNAJC21*	3.699932361	-0.036783683	-0.103121881	0.066338199	0.999933887	0.011775314	0.135476729	-0.123701415	0.464265661
ENSG00000182568	*SATB1*	6.101339608	-0.013109992	0.002227251	-0.015337243	0.999933887	-0.211449542	-0.307970975	0.096521432	0.464265661
ENSG00000143952	*VPS54*	2.771852296	-0.004738225	-0.136495415	0.13175719	0.999933887	-0.417034255	-0.231669993	-0.185364262	0.464265661
ENSG00000140688	*RUSF1*	2.561503756	0.037760006	0.144406768	-0.106646762	0.999933887	0.106029233	-0.044744714	0.150773947	0.46441848
ENSG00000165672	*PRDX3*	3.140595607	0.035533404	-0.087565324	0.123098728	0.999933887	-0.188985493	-0.329796719	0.140811226	0.464546956
ENSG00000152382	*TADA1*	1.372297196	-0.050191096	0.035180737	-0.085371833	0.999933887	0.18700778	0.376514577	-0.189506797	0.464546956
ENSG00000169446	*MMGT1*	3.878444287	-0.03351291	-0.011597394	-0.021915516	0.999933887	-0.25813618	-0.377887351	0.11975117	0.464549387
ENSG00000187325	*TAF9B*	2.146648759	0.101613086	0.066022491	0.035590595	0.999933887	-0.133476273	0.112018319	-0.245494592	0.464576264
ENSG00000211809	*TRAV27*	-1.272395362	-0.222179061	0.011457362	-0.233636423	0.999933887	0.113387925	0.519250063	-0.405862138	0.464981963
ENSG00000259494	*MRPL46*	-0.529526429	-0.054019507	0.162357371	-0.216376878	0.999933887	-0.020199617	-0.387360146	0.367160529	0.465140393
ENSG00000143811	*PYCR2*	2.805808474	-0.044548051	0.07297831	-0.117526361	0.999933887	-0.132790978	-0.25441288	0.121621903	0.465174859
ENSG00000158985	*CDC42SE2*	7.773267026	-0.080614366	-0.040352091	-0.040262275	0.999933887	-0.084047027	0.020956146	-0.105003172	0.46524233
ENSG00000125691	*RPL23*	7.330418645	0.028831076	-0.016856999	0.045688075	0.999933887	0.107058167	0.202765207	-0.09570704	0.465891704
ENSG00000091157	*WDR7*	3.163336681	0.092960503	-0.02635695	0.119317453	0.999933887	-0.03692941	-0.168266006	0.131336596	0.466303132
ENSG00000122692	*SMU1*	5.280557586	-0.085361739	-0.039966251	-0.045395488	0.999933887	0.130942701	0.206407668	-0.075464968	0.466352576
ENSG00000189127	*ANKRD34B*	0.469462176	-0.063504775	-0.144661156	0.081156381	0.999933887	-0.778317974	-0.474611511	-0.303706463	0.466387528
ENSG00000139626	*ITGB7*	4.739169149	0.024242698	0.02174127	0.002501428	0.999933887	0.087961763	-0.014595341	0.102557103	0.466508069
ENSG00000263393	*AC011825.2*	-1.454821508	0.020233893	0.03131654	-0.011082647	0.999933887	-0.982489432	-0.22025059	-0.762238842	0.467094542

ENSG00000138095	*LRPPRC*	3.381043468	-0.166607231	-0.095207644	-0.071399587	0.999933887	-0.025506686	0.110945921	-0.136452607	0.467328729
ENSG00000268433	*MTDHP3*	3.64880396	0.121279004	0.0628522	0.058426804	0.999933887	-0.102628752	0.088654651	-0.191283403	0.467328729
ENSG00000121766	*ZCCHC17*	1.851118845	0.059575771	0.063241253	-0.003665481	0.999933887	0.201373737	-0.007358661	0.208732398	0.467896568
ENSG00000120159	*CAAP1*	1.456777178	-0.128030014	0.179832483	-0.307862497	0.999933887	0.175980691	0.368098911	-0.19211822	0.468049394
ENSG00000198060	*MARCHF5*	4.275703093	0.016125628	-0.019592742	0.035718371	0.999933887	-0.27326115	-0.376112153	0.102851004	0.468090424
ENSG00000229809	*ZNF688*	2.07964784	0.13246773	0.082736947	0.049730783	0.999933887	-0.054965049	-0.261398635	0.206433586	0.468443292
ENSG00000117481	*NSUN4*	3.760142164	-0.081076315	0.033042651	-0.114118966	0.999933887	0.954854101	1.094958165	-0.140104064	0.468563942
ENSG00000280239	*AC011498.7*	0.319979454	0.101116437	0.096025647	0.00509079	0.999933887	0.170595743	-0.071562262	0.242158004	0.468563942
ENSG00000118298	*CA14*	0.11068671	0.048166744	-0.082627396	0.130794141	0.999933887	0.090952793	-0.288383288	0.379336081	0.468563942
ENSG00000103174	*NAGPA*	2.101291041	-0.056261975	0.010401577	-0.066663553	0.999933887	-0.077600947	-0.226068632	0.148467685	0.468828551
ENSG00000163376	*KBTBD8*	2.426154434	-0.152284047	-0.217823306	0.06553926	0.999933887	-0.157192762	0.038035699	-0.19522846	0.468928526
ENSG00000202272	*Y_RNA*	-0.774025372	0.331282114	0.482420904	-0.15113879	0.999933887	1.271793548	0.563580603	0.708212945	0.469329816
ENSG00000278745	*AL391903.3*	1.659887519	0.146925734	0.181744073	-0.034818339	0.999933887	0.68808814	0.989215849	-0.301127709	0.469361593
ENSG00000258920	*FOXN3-AS1*	0.990765986	0.06872887	0.206169609	-0.137440739	0.999933887	-0.549262476	-0.759427121	0.210164645	0.469361593
ENSG00000112303	*VNN2*	8.436156051	-0.002695991	-0.090915869	0.088219878	0.999933887	-0.413650564	-0.281713272	-0.131937292	0.469865531
ENSG00000270959	*LPP-AS2*	2.719815477	0.047130406	-0.051824594	0.098954999	0.999933887	0.671397699	0.814083473	-0.142685775	0.470135436
ENSG00000103196	*CRISPLD2*	5.006638679	-0.056416031	0.016645247	-0.073061278	0.999933887	-0.494427627	-0.633907162	0.139479535	0.470191192
ENSG00000205702	*CYP2D7*	2.545348161	0.05674483	0.171475661	-0.114730831	0.999933887	-0.000633514	-0.12820157	0.127568056	0.470191192
ENSG00000163959	*SLC51A*	2.97583014	0.00970471	-0.002926381	0.012631091	0.999933887	-0.093199009	0.041877742	-0.135076751	0.470191192
ENSG00000183977	*PP2D1*	1.581762832	-0.046601597	-0.016518056	-0.030083541	0.999933887	0.231475978	0.437296452	-0.205820474	0.470321454
ENSG00000072952	*IRAG1*	3.310928901	-0.091084216	-0.010041381	-0.081042835	0.999933887	-0.026942454	-0.261313545	0.234371091	0.470515429
ENSG00000196083	*IL1RAP*	7.339034021	-0.023707998	-0.016892154	-0.006815844	0.999933887	-0.242144854	-0.123057944	-0.11908691	0.470630454
ENSG00000172009	*THOP1*	2.724440385	0.011949566	0.05439937	-0.042449805	0.999933887	0.199762787	0.069411165	0.130351622	0.470630454
ENSG00000272256	*AC044849.1*	1.103425911	0.162647601	0.192926788	-0.030279187	0.999933887	0.25759176	-0.042391803	0.299983563	0.470631792
ENSG00000189144	*ZNF573*	-0.692664508	-0.188233164	-0.32665053	0.138417366	0.999933887	-0.246052189	0.133901818	-0.379954007	0.470631792
ENSG00000070366	*SMG6*	3.31720326	-0.021663001	-0.060768134	0.039105133	0.999933887	0.084528706	-0.035400033	0.119928739	0.470631792
ENSG00000145335	*SNCA*	-1.630215482	-0.454202074	-0.015197755	-0.439004319	0.999933887	-0.191461889	0.353721395	-0.545183284	0.470722072
ENSG00000235328	*AC006946.1*	-0.93156693	-0.257621015	-0.289300017	0.031679002	0.999933887	-0.293156632	-0.791563275	0.498406643	0.470722072
ENSG00000166598	*HSP90B1*	5.766556572	-0.074771157	-0.208894468	0.134123311	0.999933887	-0.008297629	0.11662528	-0.124922909	0.470722072
ENSG00000162636	*FAM102B*	3.113681437	-0.093794681	-0.159328881	0.0655342	0.999933887	-0.32268717	-0.128383444	-0.194303727	0.470722072
ENSG00000101997	*CCDC22*	2.588884707	-0.021310027	0.117560825	-0.138870851	0.999933887	0.170767769	0.030029524	0.140738245	0.470722072
ENSG00000182934	*SRPRA*	6.83143201	0.007682616	0.006351664	0.001330952	0.999933887	0.116061867	0.188879877	-0.07281801	0.470722072
ENSG00000231964	*AL731567.1*	1.308840382	0.000887515	-0.094883928	0.095771442	0.999933887	0.36819597	0.640404239	-0.272208269	0.470722072
ENSG00000104738	*MCM4*	1.240305118	-0.063198129	-0.021981199	-0.04121693	0.999933887	0.058956693	0.27988019	-0.220923497	0.470737258
ENSG00000154451	*GBP5*	8.720262885	-0.010787355	-0.100984373	0.090197017	0.999933887	0.304721213	0.428078281	-0.123357067	0.470737258
ENSG00000286809	*AC008964.1*	0.524170022	-0.006623538	-0.073705205	0.067081668	0.999933887	-0.76000402	-0.518269265	-0.241734755	0.470969519
ENSG00000164080	*RAD54L2*	3.633713039	0.059081927	-0.026731791	0.085813718	0.999933887	0.025305872	-0.072823483	0.098129355	0.470998285
ENSG00000223501	*VPS52*	3.304547717	0.002486849	0.165820643	-0.163333794	0.999933887	0.032489904	-0.088034331	0.120524234	0.470998285
ENSG00000115504	*EHBP1*	0.919266448	0.120403158	0.037799402	0.082603756	0.999933887	-0.073127115	0.172670895	-0.245798011	0.471355465
ENSG00000185811	*IKZF1*	6.782199886	0.030733564	0.034711613	-0.003978049	0.999933887	0.032998847	0.119048582	-0.086049735	0.471355465
ENSG00000141753	*IGFBP4*	1.121804506	-0.064573842	0.047835978	-0.11240982	0.999933887	0.008429353	0.268956952	-0.260527599	0.471498252
ENSG00000135720	*DYNC1LI2*	5.170556928	-0.057768446	-0.057263642	-0.000504804	0.999933887	-0.402460453	-0.495023656	0.092563203	0.471845901
ENSG00000089050	*RBBP9*	0.742244505	0.108047045	0.095834682	0.012212363	0.999933887	0.483931496	0.737385424	-0.253453929	0.471931274
ENSG00000165240	*ATP7A*	4.526816848	-0.006619617	-0.02529466	0.018675043	0.999933887	-0.721899597	-0.867706168	0.145806571	0.472126748
ENSG00000227782	*AC002553.1*	-0.150592801	0.041822286	0.130229802	-0.088407516	0.999933887	-0.141800584	0.235565951	-0.377366535	0.472283787
ENSG00000099822	*HCN2*	-0.638268358	0.105400449	0.182232541	-0.076832093	0.999933887	0.293589979	0.704924312	-0.411334332	0.472348063
ENSG00000161677	*JOSD2*	2.041683987	0.135886775	0.203787663	-0.067900888	0.999933887	0.111285788	-0.062256677	0.173542465	0.47247283
ENSG00000154874	*CCDC144B*	1.112433042	0.07594099	-0.00453889	0.08047988	0.999933887	-0.137375773	0.129951996	-0.267327769	0.47247283
ENSG00000161654	*LSM12*	2.089489356	-0.016988939	-0.049844379	0.03285544	0.999933887	-0.044013819	-0.200577004	0.156563185	0.47247283
ENSG00000185946	*RNPC3*	4.058616713	0.017955916	-0.100791535	0.118747451	0.999933887	-0.387539517	-0.200806654	-0.186732863	0.47247283
ENSG00000186815	*TPCN1*	2.837523623	0.009683284	0.16349306	-0.153809776	0.999933887	0.0575854	0.184621303	-0.127035903	0.47247283
ENSG00000260661	*AC116903.2*	0.840569109	0.061137298	0.159372792	-0.098235494	0.999933887	0.057473297	-0.25410827	0.311581567	0.472609862
ENSG00000102309	*PIN4*	1.022101426	-0.063316964	-0.007648348	-0.055668616	0.999933887	-0.202343314	0.058629207	-0.260972521	0.472609862
ENSG00000197766	*CFD*	6.871300449	0.10056605	0.040948846	0.059617204	0.999933887	-0.088366917	-0.236321047	0.14795413	0.472612194
ENSG00000182504	*CEP97*	1.636472921	-0.003539633	-0.176646664	0.173107031	0.999933887	-0.486598132	-0.225992445	-0.260605687	0.472665395
ENSG00000164808	*SPIDR*	3.840121555	0.022237612	0.007567287	0.014670325	0.999933887	-0.156418184	-0.267817245	0.111399061	0.472741983
ENSG00000074755	*ZZEF1*	7.418008757	-0.035740283	-0.027460297	-0.008279986	0.999933887	-0.113816363	-0.183010342	0.069193979	0.4729293
ENSG00000223956	*LINC01767*	-1.655939125	0.201691409	-0.001519888	0.203211297	0.999933887	-0.297578893	-0.485234279	0.187655387	0.4729293
ENSG00000099995	*SF3A1*	7.565178962	-0.00808707	0.022238488	-0.030325558	0.999933887	0.056155311	0.11988605	-0.063730739	0.472953951
ENSG00000160803	*UBQLN4*	2.729181066	0.090722952	0.017559993	0.073162959	0.999933887	0.141048946	0.283711327	-0.142662382	0.473118703
ENSG00000214900	*LINC01588*	2.253878743	-0.030760564	0.039982586	-0.070743149	0.999933887	0.086408079	0.276454519	-0.190046439	0.473118703
ENSG00000152242	*C18orf25*	5.365688142	0.003909723	0.062835577	-0.058925854	0.999933887	-0.300027337	-0.380590999	0.080563662	0.473118703
ENSG00000224877	*NDUFAF8*	1.835262983	-0.099736805	0.077907634	-0.177644439	0.999933887	0.172259612	0.017388322	0.15487129	0.473586239
ENSG00000150681	*RGS18*	4.648263834	-0.076363166	-0.10353557	0.027172404	0.999933887	-0.784321384	-0.97326602	0.188944636	0.473586239
ENSG00000152439	*ZNF773*	0.104847979	-0.044316038	-0.419682394	0.375366356	0.999933887	-0.109484628	-0.361974715	0.252490087	0.473586239
ENSG00000164308	*ERAP2*	4.41564466	-0.070638846	-0.105186094	0.034547248	0.999933887	-0.156665608	-0.278466849	0.121801241	0.473894945
ENSG00000171103	*TRMT61B*	0.358017567	-0.123471934	-0.029013537	-0.094458397	0.999933887	0.141597736	0.382435011	-0.240837274	0.473952647
ENSG00000225342	*LRRK2-DT*	3.752065033	-0.210671311	-0.29164122	0.080969909	0.999933887	-0.394818193	-0.556758648	0.161940455	0.474003006
ENSG00000188234	*AGAP4*	1.256779868	-0.076868186	-0.047757576	-0.02911061	0.999933887	0.406083855	0.18688547	0.219198385	0.474003006
ENSG00000187566	*NHLRC1*	1.122232591	0.008138359	0.032105108	-0.023966749	0.999933887	1.104148084	1.392749087	-0.288601003	0.474003006
ENSG00000280077	*AL353763.2*	0.328491531	-0.208413538	-0.379535758	0.171122219	0.999933887	-0.570917065	-0.306164026	-0.264753039	0.474027257
ENSG00000236829	*Z97634.1*	0.839371903	-0.013464718	-0.377382869	0.363918151	0.999933887	0.304382453	0.11797781	0.186404643	0.474051429
ENSG00000198075	*SULT1C4*	-0.969771105	0.347089658	-0.135246395	0.482336053	0.999933887	1.637514486	1.945498999	-0.307984513	0.474110391
ENSG00000223522	*AC093690.1*	0.46601962	0.033484583	-0.256317828	0.289802411	0.999933887	0.04141753	0.368995755	-0.327578225	0.474110391
ENSG00000240143	*AL023653.1*	1.887986915	-0.027052475	-0.008995701	-0.018056774	0.999933887	0.191086021	0.377108836	-0.186022816	0.474291987
ENSG00000179950	*PUF60*	5.438681011	0.027182461	-0.011711392	0.038893853	0.999933887	-0.090975671	-0.184240787	0.093265116	0.47459318
ENSG00000162194	*LBHD1*	0.560380611	0.011165906	0.092116215	-0.08095031	0.999933887	0.54339109	0.806175792	-0.262784701	0.474861381
ENSG00000182180	*MRPS16*	3.248751142	-0.142173499	-0.067565636	-0.074607863	0.999933887	-0.130838012	-0.250807197	0.119969185	0.474925826
ENSG00000214353	*VAC14-AS1*	1.89062497	-0.072611959	0.04975995	-0.122371909	0.999933887	0.137196233	0.309831394	-0.172635162	0.475071905
ENSG00000182400	*TRAPPC6B*	1.819280423	-0.007955902	-0.023692483	0.015736581	0.999933887	-0.186406895	-0.344878774	0.158471879	0.475076722
ENSG00000154479	*CCDC173*	-0.523515933	-0.068924344	0.025218337	-0.094142681	0.999933887	0.025227086	-0.371912984	0.39714007	0.475254195
ENSG00000134072	*CAMK1*	-0.017200116	0.15012721	-0.031847247	0.181974456	0.999933887	-0.022360195	-0.308231284	0.285871089	0.475573634
ENSG00000169962	*TAS1R3*	0.650682678	0.105167989	-0.016183106	0.121351095	0.999933887	0.132715243	-0.162696414	0.295411657	0.475573634
ENSG00000226688	*ENTPD1-AS1*	2.090729564	-0.005164277	0.13184617	-0.137010447	0.999933887	-0.69084052	-0.930049377	0.239208856	0.475573634
ENSG00000167046	*AL357033.1*	1.488857872	0.059360354	0.057504075	0.001856279	0.999933887	0.517449443	0.664642043	-0.147192601	0.475653655
ENSG00000101605	*MYOM1*	0.586163713	-0.000454894	-0.13388115	0.133426256	0.999933887	-0.249199568	-9.7692e-05	-0.249101876	0.475789851
ENSG00000148343	*MIGA2*	4.007774003	0.07347642	0.097866479	-0.024390059	0.999933887	0.021922839	-0.087394853	0.109317693	0.475918936
ENSG00000105649	*RAB3A*	0.127948572	-0.032619928	-0.065068809	0.032448881	0.999933887	-0.44344752	-0.778192083	0.334744563	0.475929487
ENSG00000071859	*FAM50A*	6.847880209	0.041642472	0.107222799	-0.065580327	0.999933887	0.587311587	0.69086091	-0.103549323	0.476251077
ENSG00000160293	*VAV2*	0.50907056	0.21328414	0.081272344	0.132011797	0.999933887	0.45733761	0.698063759	-0.240726149	0.47632275
ENSG00000187626	*ZKSCAN4*	0.973148465	-0.143557621	-0.035181	-0.10837662	0.999933887	-0.449984051	-0.622206366	0.172222315	0.47632275
ENSG00000225032	*AL162586.1*	-0.227594982	0.136319471	-0.001322337	0.137641808	0.999933887	0.084053263	-0.157659837	0.2417131	0.47632275
ENSG00000173198	*CYSLTR1*	3.187826717	-0.052924378	-0.204690942	0.151766564	0.999933887	-0.240653005	-0.437967608	0.197314603	0.47632275
ENSG00000227057	*WDR46*	3.189689331	-0.031021485	-0.027820659	-0.003200826	0.999933887	0.024614889	-0.074716302	0.099331191	0.47632275
ENSG00000118579	*MED28*	6.961513881	-0.087767969	-0.087555811	-0.000212158	0.999933887	-0.09258054	-0.017415753	-0.075164786	0.476341888
ENSG00000198355	*PIM3*	9.245477955	0.20396058	0.216364814	-0.012404234	0.999933887	1.117685132	1.236277269	-0.118592137	0.476445143
ENSG00000051382	*PIK3CB*	4.96674403	-0.045231161	-0.047468129	0.002236968	0.999933887	-0.637202041	-0.741126245	0.103924204	0.476445143
ENSG00000287351	*AC092120.3*	-0.599280848	0.124773431	0.288171847	-0.163398416	0.999933887	0.048562314	-0.478699221	0.527261535	0.476445143
ENSG00000108556	*CHRNE*	2.184074368	0.023830333	0.058537841	-0.034707508	0.999933887	0.297543229	0.477458502	-0.179915272	0.476549218
ENSG00000182057	*OGFRP1*	-0.112183398	0.129085057	0.094733009	0.034352048	0.999933887	-0.012901257	-0.2719158	0.259014542	0.476701653
ENSG00000279382	*AC018665.1*	1.285028108	-0.100398784	-0.099917292	-0.000481493	0.999933887	-0.050386943	-0.238593402	0.188206459	0.476872579
ENSG00000158006	*PAFAH2*	3.636137771	-0.015470379	-0.001538699	-0.01393168	0.999933887	-0.024955146	-0.11274261	0.087787464	0.476872579
ENSG00000155229	*MMS19*	3.925150531	0.010714895	0.019379903	-0.008665008	0.999933887	-0.024512364	-0.106298511	0.081786147	0.476872579
ENSG00000156521	*TYSND1*	2.624080327	-0.014986626	-0.019550828	0.004564202	0.999933887	0.124891852	-0.00851517	0.133407022	0.476955649
ENSG00000165959	*CLMN*	4.751743447	-0.037588219	-0.043678634	0.006090415	0.999933887	0.223065202	0.333283274	-0.110218073	0.477189009

ENSG00000169228	*RAB24*	4.325064793	0.145844234	0.199720401	-0.053876167	0.999933887	0.493028736	0.292720164	0.200308572	0.477274191
ENSG00000285979	*AC009090.6*	3.43377209	-0.0021321	0.008527244	-0.010659344	0.999933887	-0.1008222	0.053203279	-0.15402548	0.477274191
ENSG00000107077	*KDM4C*	4.281932147	-0.042114866	-0.080577923	0.038463058	0.999933887	0.003243422	-0.077480369	0.08072379	0.477382978
ENSG00000090612	*ZNF268*	1.418979826	-0.108605603	-0.078559938	-0.030045665	0.999933887	-0.216593894	0.009559708	-0.226153602	0.477382978
ENSG00000237135	*DDX10P1*	1.718171581	-0.104547798	0.072558567	-0.177106365	0.999933887	-0.069042436	0.192895735	-0.261938171	0.477382978
ENSG00000229124	*VIM-AS1*	4.811074548	0.012240853	-0.041002856	0.053243709	0.999933887	0.276838998	0.389590667	-0.112751669	0.477382978
ENSG00000145781	*COMMD10*	0.928202231	0.00141909	-0.099663235	0.101082324	0.999933887	0.036337778	0.275547002	-0.239209223	0.477382978
ENSG00000137965	*IFI44*	4.707453048	-0.14672915	-0.162387577	0.015658427	0.999933887	-0.420291157	-0.608741463	0.188450306	0.477707339
ENSG00000176681	*LRRC37A*	-0.875405782	-0.207542051	-0.051755773	-0.155786279	0.999933887	-0.159758007	0.201648036	-0.361406043	0.478107565
ENSG00000143013	*LMO4*	2.806167422	-0.069102824	0.111764198	-0.180867022	0.999933887	-0.195749995	-0.349877052	0.154127057	0.478278704
ENSG00000171202	*TMEM126A*	1.3918651	-0.028595969	0.056493921	-0.08508989	0.999933887	0.128156835	0.310870605	-0.18271377	0.478330976
ENSG00000118181	*RPS25*	7.415819355	-0.007427569	-0.072725785	0.065298216	0.999933887	0.09645249	0.182400712	-0.085948222	0.478330976
ENSG00000105996	*HOXA2*	-0.805321252	-0.17759959	-0.453424625	0.275825035	0.999933887	-0.81884345	-1.224684274	0.405840824	0.479035825
ENSG00000134954	*ETS1*	7.35818214	-0.026871617	-0.024261793	-0.002609823	0.999933887	0.124792021	0.205826679	-0.081034658	0.479035825
ENSG00000187037	*GPR141*	1.446123349	0.116251068	-0.008148383	0.124399451	0.999933887	0.980814006	1.208597036	-0.22778303	0.4791631
ENSG00000119899	*SLC17A5*	3.540676111	-0.075266391	0.001429809	-0.0766962	0.999933887	-0.717846358	-0.569044789	-0.148801569	0.4791631
ENSG00000166387	*PPFIBP2*	3.149858166	0.037167356	-0.016202408	0.053369765	0.999933887	-0.214849256	-0.123899124	-0.090950133	0.4791631
ENSG00000161905	*ALOX15*	3.750265002	-0.00493622	-0.006113415	0.001177195	0.999933887	-0.060675448	0.056430729	-0.117106177	0.4791631
ENSG00000174171	*AC020659.1*	2.130098231	-0.055494431	0.041815119	-0.097309549	0.999933887	-0.026560727	0.124395316	-0.150956042	0.479218613
ENSG00000101246	*ARFRP1*	3.754968628	0.04106195	0.058233893	-0.017171943	0.999933887	0.059384379	-0.050911515	0.110295894	0.479258173
ENSG00000019995	*ZRANB1*	4.46643475	0.009701905	-0.082945756	0.092647661	0.999933887	-0.169933801	-0.07395113	-0.095982671	0.479267642
ENSG00000094841	*UPRT*	1.627542784	-0.079216994	0.026899084	-0.106116078	0.999933887	0.244656418	0.439799384	-0.195142965	0.479447185
ENSG00000187097	*ENTPD5*	1.10282196	-0.050629113	-0.179148654	0.128519541	0.999933887	-0.102414713	0.098717624	-0.201132337	0.479447207
ENSG00000206077	*ZDHHC11B*	-0.207057972	-0.072606762	0.20538836	-0.277995123	0.999933887	0.689983298	1.149099114	-0.459115816	0.479447207
ENSG00000162174	*ASRGL1*	0.555838537	-0.063393584	-0.157669136	0.094275553	0.999933887	-0.656505535	-0.883596124	0.227090589	0.479463312
ENSG00000128699	*ORMDL1*	4.232348478	0.011912691	-0.065285811	0.077198502	0.999933887	-0.04525428	-0.14659541	0.101341129	0.479463312
ENSG00000142303	*ADAMTS10*	1.867277188	0.012886195	0.061655183	-0.048768988	0.999933887	0.084561325	-0.068447203	0.153008528	0.479463312
ENSG00000244187	*TMEM141*	0.168008591	0.004010896	0.364378767	-0.360367871	0.999933887	0.13458113	-0.134207937	0.268789068	0.479463312
ENSG00000232593	*KANTR*	0.47777646	-0.121804069	-0.374739548	0.252935478	0.999933887	-0.276455019	0.026401947	-0.302856966	0.479521668
ENSG00000106459	*NRF1*	3.870453694	-0.032413207	0.076316848	-0.108730055	0.999933887	-0.312368892	-0.413443749	0.101074857	0.479521668
ENSG00000284977	*AL160272.1*	1.008078106	-0.008434509	-0.171624823	0.163190314	0.999933887	0.274794342	0.614951523	-0.340157181	0.479521668
ENSG00000253736	*AC022217.3*	2.589277696	0.157427889	0.114583689	0.0428442	0.999933887	0.980872983	1.167167038	-0.186294054	0.479739403
ENSG00000107164	*FUBP3*	3.334255093	0.006165225	0.018908023	-0.012742798	0.999933887	-0.080664056	-0.179753733	0.099089677	0.479739403
ENSG00000185305	*ARL15*	1.789892201	-0.005004898	-0.022961868	0.01795697	0.999933887	-0.198070579	-0.018108909	-0.17996167	0.479821459
ENSG00000155256	*ZFYVE27*	5.659879187	0.001487711	0.019652677	-0.018164967	0.999933887	-0.009488735	-0.078637482	0.069148748	0.479821459
ENSG00000113070	*HBEGF*	2.372560197	0.245795869	0.408750182	-0.162954313	0.999933887	0.241084335	0.48187588	-0.240791546	0.480069154
ENSG00000085365	*SCAMP1*	2.681230478	0.026974981	-0.123662934	0.150637914	0.999933887	-0.315769698	-0.478911741	0.163142043	0.480213414
ENSG00000158793	*NIT1*	3.697878195	-0.015164301	-0.018315218	0.003150917	0.999933887	-0.019554938	-0.139498428	0.11994349	0.480545805
ENSG00000204264	*PSMB8*	7.051329716	-0.021872116	-0.035338661	0.013466545	0.999933887	0.067060276	-0.009093392	0.076153668	0.480558054
ENSG00000078747	*ITCH*	6.282909992	-0.06174908	-0.053425615	-0.008323465	0.999933887	-0.323386009	-0.235421238	-0.087964771	0.480626287
ENSG00000125657	*TNFSF9*	1.615977089	0.023517385	-0.075788285	0.09930567	0.999933887	0.196092694	0.365800956	-0.169708262	0.481059292
ENSG00000250303	*LINC02762*	1.661459635	0.15547294	0.087687748	0.067785192	0.999933887	0.602809067	0.831329786	-0.228520719	0.481154214
ENSG00000261471	*AC092145.1*	2.531419934	-0.110180096	-0.053035429	-0.057144666	0.999933887	-0.612867133	-0.814665147	0.201798014	0.481482692
ENSG00000167664	*TMIGD2*	-0.50936403	0.212546329	0.085517223	0.127029106	0.999933887	0.038229463	-0.306498766	0.344728229	0.481482692
ENSG00000164611	*PTTG1*	0.160537794	0.027715293	-0.378523461	0.406238753	0.999933887	0.294019747	0.600678294	-0.306658547	0.481482692
ENSG00000274290	*H2BC6*	-0.872413457	0.021881778	-0.085925938	0.107807716	0.999933887	-0.871426129	-1.297763105	0.426336977	0.481482692
ENSG00000250539	*KRT8P33*	-0.122204846	-0.133183855	-0.248531355	0.1153475	0.999933887	0.071942441	0.358916837	-0.286974396	0.481790667
ENSG00000272145	*NFYC-AS1*	0.972011344	0.068325228	0.090225643	-0.021900415	0.999933887	0.076951796	0.288107263	-0.211155468	0.481881812
ENSG00000126247	*CAPNS1*	5.780929449	0.083884692	-0.036047352	0.119932044	0.999933887	-0.143337403	-0.24273886	0.099401457	0.481884062
ENSG00000172262	*ZNF131*	4.170017177	-0.043501368	-0.105998134	0.062496766	0.999933887	0.026756644	0.141142935	-0.11438629	0.482004444
ENSG00000231731	*AC010976.1*	0.968418578	-0.12652104	0.114033388	-0.240554428	0.999933887	-0.487895684	-0.269651279	-0.218244405	0.482038537
ENSG00000170248	*PDCD6IP*	5.898888627	0.015729298	-0.04894054	0.064669838	0.999933887	-0.125067511	-0.026398872	-0.098668639	0.482038537
ENSG00000087995	*METTL2A*	0.452093854	-0.27778181	-0.389717265	0.111935454	0.999933887	0.072527802	0.314415556	-0.241887754	0.482074203
ENSG00000275180	*AC048341.2*	0.975094022	0.025601334	-0.182272596	0.20787393	0.999933887	1.572954004	1.797337623	-0.224383619	0.482074203
ENSG00000148308	*GTF3C5*	4.34979168	-0.004587494	0.06363482	-0.068222314	0.999933887	-0.093049671	-0.18566342	0.092613749	0.482074203
ENSG00000196689	*TRPV1*	-0.210764798	-0.07179477	0.135766848	-0.207561618	0.999933887	-0.343734963	-0.673244185	0.329509222	0.482306408
ENSG00000271643	*AC112220.2*	0.210415421	-0.222748736	0.206484192	-0.429232929	0.999933887	0.106106398	0.33274729	-0.226640892	0.482488961
ENSG00000124602	*UNC5CL*	0.68420246	-0.109337013	0.043183268	-0.152520281	0.999933887	-0.057481105	-0.325360493	0.267879388	0.482514203
ENSG00000143158	*MPC2*	3.00700804	-0.067588556	0.133049269	-0.200637825	0.944094537	-0.262849969	-0.411440634	0.148590665	0.482572699
ENSG00000163328	*GPR155*	4.415159079	0.041465203	0.120038632	-0.078573429	0.999933887	-0.286243963	-0.453956744	0.167712781	0.482572699
ENSG00000233785	*AC131011.1*	0.675699703	0.250943233	0.274356951	-0.023413718	0.999933887	0.713251324	0.998835399	-0.285584074	0.482584377
ENSG00000090661	*CERS4*	3.203895319	0.145055059	0.070795425	0.074259635	0.999933887	0.709410422	0.853686015	-0.144275593	0.482584377
ENSG00000280003	*AC097637.2*	-0.495395676	-0.145916005	-0.010158866	-0.135757139	0.999933887	-0.225199343	-0.667572934	0.442373591	0.482584377
ENSG00000287597	*AC108105.1*	-1.575348332	-0.387803118	0.268506804	-0.656309922	0.999933887	1.11382882	1.474386309	-0.360557488	0.482584377
ENSG00000064607	*SUGP2*	5.393655109	-0.021888753	0.018186299	-0.040075052	0.999933887	-0.02718565	-0.10400605	0.0768204	0.482584377
ENSG00000067113	*PLPP1*	-0.519834658	-0.054821636	-0.347348472	0.292526836	0.999933887	0.315690554	0.596911485	-0.281220931	0.482584377
ENSG00000011260	*UTP18*	4.021356721	-0.007224424	-0.060264605	0.053040181	0.999933887	-0.164080009	-0.255974331	0.091894321	0.482584377
ENSG00000167378	*IRGQ*	5.142831131	0.006509981	0.032869624	-0.026359644	0.999933887	0.496514275	0.631132588	-0.134618313	0.482584377
ENSG00000002834	*LASP1*	8.612672732	-0.00450021	-0.009942513	0.005442304	0.999933887	-0.624184812	-0.749459998	0.125275186	0.482584377
ENSG00000134278	*SPIRE1*	1.610019313	0.003694471	-0.295050176	0.298744647	0.999933887	-0.40947281	-0.283584756	-0.125888054	0.482584377
ENSG00000272556	*GTF2IP13*	1.291165599	0.113386387	0.133365392	-0.019979005	0.999933887	0.305105685	0.518462698	-0.213357013	0.482631078
ENSG00000204590	*GNL1*	5.870978267	0.027263962	0.048744187	-0.021480224	0.999933887	0.128096293	0.216072256	-0.087975962	0.482631078
ENSG00000127870	*RNF6*	3.69786672	-0.013332832	-0.176611312	0.16327848	0.999933887	-0.528684747	-0.369770102	-0.158914645	0.482631078
ENSG00000107643	*MAPK8*	4.385857191	-0.002086609	0.007531384	-0.009617993	0.999933887	-0.125378021	-0.001285704	-0.124092317	0.482631078
ENSG00000132694	*ARHGEF11*	6.171229184	-0.020642458	0.038012295	-0.058654753	0.999933887	-0.442521592	-0.535509877	0.092988285	0.482671786
ENSG00000104969	*SGTA*	6.409717385	-0.026145079	-0.041075886	0.014930807	0.999933887	0.153126087	0.053063433	0.100062654	0.482681123
ENSG00000101882	*NKAP*	3.121065911	-0.076909661	-0.024847939	-0.052061722	0.999933887	-0.045256777	0.065980461	-0.111237238	0.482820773
ENSG00000230482	*ATP5MC2P3*	-0.491805444	0.028332125	0.066099242	-0.037767118	0.999933887	-0.252911518	-0.71717043	0.464258912	0.482820773
ENSG00000213928	*IRF9*	5.482750824	-0.01401815	-0.062030032	0.048011882	0.999933887	0.091574646	0.012350682	0.079223964	0.482975631
ENSG00000174748	*RPL15*	7.338046805	0.008441578	-0.057399327	0.065840906	0.999933887	0.179275234	0.267642983	-0.088367749	0.483031309
ENSG00000111725	*PRKAB1*	3.86052872	-0.076388492	0.029587164	-0.105975656	0.999933887	-0.253024349	-0.355500679	0.10247633	0.483143522
ENSG00000168495	*POLR3D*	2.340010294	0.049735312	-0.107764356	0.157499668	0.999933887	0.160655526	0.290647778	-0.129992252	0.483143522
ENSG00000011198	*ABHD5*	7.256276311	-0.014656754	-0.017836225	0.003179471	0.999933887	-1.411506436	-1.256914961	-0.154591476	0.483240025
ENSG00000131943	*C19orf12*	2.399278456	0.124485518	-0.079939925	0.204425443	0.999933887	-0.169677988	-0.337006964	0.167328975	0.483272017
ENSG00000183010	*PYCR1*	-1.650582791	0.118839251	-0.305322862	0.424162113	0.999933887	0.42165395	0.746291572	-0.324637623	0.483541501
ENSG00000271806	*AL590822.2*	1.091941801	0.142786928	0.090019782	0.052767146	0.999933887	-0.491714369	-0.191116858	-0.300597512	0.483593455
ENSG00000215068	*AC025171.2*	1.717404912	0.075073242	-0.075453976	0.150527218	0.999933887	-0.175168829	-0.370635557	0.195466728	0.483593455
ENSG00000119121	*TRPM6*	3.964642921	-0.012452077	-0.054519003	0.042066926	0.999933887	-0.442408576	-0.305756678	-0.136651899	0.484009755
ENSG00000251139	*AC084871.1*	2.127712305	0.28628208	0.255659422	0.030622658	0.999933887	1.79596637	2.136227155	-0.340260785	0.484068561
ENSG00000235945	*AC002543.1*	0.444026856	0.274304519	0.409103922	-0.134799403	0.999933887	-0.023785422	0.482063794	-0.505849216	0.484189846
ENSG00000119421	*NDUFA8*	0.714966948	0.29431049	0.048424708	0.245885782	0.999933887	0.186511859	-0.063357496	0.249869355	0.484260369
ENSG00000105397	*TYK2*	8.04486526	0.047395706	0.077076272	-0.029680566	0.999933887	0.16358773	0.078092952	0.085494778	0.485126773
ENSG00000169018	*FEM1B*	3.911991037	-0.071922905	-0.136135305	0.064212399	0.999933887	-0.461381467	-0.575867995	0.114486528	0.485206628
ENSG00000205784	*ARRDC5*	-0.080553664	-0.301848661	0.0365727	-0.338421361	0.999933887	0.023479942	-0.264395418	0.287875361	0.485383383
ENSG00000069345	*DNAJA2*	5.470724255	-0.055382564	-0.144281718	0.088899155	0.999933887	-0.339707589	-0.432385091	0.092677502	0.485738849
ENSG00000269335	*IKBKG*	4.021829883	0.069194886	0.024166796	0.04502809	0.999933887	0.137853618	0.231209754	-0.093356136	0.485876009
ENSG00000164211	*STARD4*	2.86443814	-0.131754934	-0.202780286	0.071025352	0.999933887	-0.184781042	0.029968707	-0.214749749	0.485876009
ENSG00000140367	*UBE2Q2*	2.910236043	-0.137375611	-0.09551727	-0.041858341	0.999933887	-0.106257339	0.010093714	-0.116351053	0.485900676
ENSG00000099949	*LZTR1*	2.395155387	0.082124802	-0.02548544	0.107610243	0.999933887	0.141772163	-0.017084956	0.158857118	0.485928762
ENSG00000160799	*CCDC12*	3.212216457	-0.036232997	0.048285974	-0.08451897	0.999933887	-0.030833037	-0.148456666	0.117623629	0.485928762
ENSG00000283994	*AC092652.2*	2.183274842	0.00864839	0.120910667	-0.112262277	0.999933887	0.222003726	0.475580898	-0.253577172	0.485928762
ENSG00000090686	*USP48*	4.509445263	0.013935094	-0.106528928	0.120464022	0.999933887	-0.126438626	-0.260058226	0.133619599	0.486839489
ENSG00000164118	*CEP44*	1.739493239	-0.014778265	-0.095188727	0.080410461	0.999933887	-0.417169814	-0.172428235	-0.24474158	0.487085013
ENSG00000239665	*AL157392.3*	2.823865843	0.015574936	0.0169541	-0.001379164	0.999933887	-0.217519212	-0.339257756	0.121738544	0.487107145
ENSG00000146830	*GIGYF1*	7.309671171	0.023890028	0.12530061	-0.101410582	0.999933887	-0.185422402	-0.275154288	0.089731886	0.487181244

ENSG00000197006	*METTL9*	6.042213068	0.051498151	0.007569264	0.043928887	0.999933887	-0.018334304	-0.102863318	0.084529015	0.48719309
ENSG00000127561	*SYNGR3*	-0.266257498	0.215052476	-0.012710847	0.227763324	0.999933887	0.582789516	0.875184328	-0.292394812	0.487244196
ENSG00000131437	*KIF3A*	1.539058633	-0.125029746	-0.121203367	-0.003826379	0.999933887	-0.1614501	0.065770008	-0.227220109	0.487511981
ENSG00000085662	*AKR1B1*	2.929073051	-0.030914638	0.141376917	-0.172291555	0.999933887	0.231949925	0.122129799	0.109820126	0.487511981
ENSG00000126261	*UBA2*	3.696192674	-0.09171401	-0.18354404	0.09183003	0.999933887	-0.043736675	0.053520871	-0.097257546	0.487557777
ENSG00000141556	*TBCD*	4.526703661	0.010005536	0.017048806	-0.007043271	0.999933887	0.027267202	-0.061961169	0.089228371	0.487557777
ENSG00000177548	*RABEP2*	2.983871712	0.076259719	0.033015616	0.043244104	0.999933887	0.015143903	-0.126833272	0.141977175	0.48789841
ENSG00000260276	*AC022167.2*	-0.518683507	0.16290974	-0.130366644	0.293276384	0.999933887	0.085967053	-0.283698619	0.369665672	0.48789841
ENSG00000115233	*PSMD14*	1.928097664	-0.089444345	-0.082294288	-0.007150057	0.999933887	0.293454706	0.436870001	-0.143415295	0.487938807
ENSG00000145022	*TCTA*	3.202527957	0.006643459	0.036243581	-0.029600122	0.999933887	-0.036911791	-0.143135569	0.106223778	0.487938807
ENSG00000268030	*AC005253.1*	1.48020081	-0.045001216	0.023591516	-0.068592733	0.999933887	-0.098455147	0.07332316	-0.171778306	0.488105217
ENSG00000009844	*VTA1*	3.101274328	-0.051837312	-0.150079348	0.098242035	0.999933887	-0.339584881	-0.468767863	0.129182982	0.488227334
ENSG00000159346	*ADIPOR1*	8.232347514	0.070462483	0.040622603	0.029839881	0.999933887	-0.098875667	-0.017923068	-0.080952599	0.488539347
ENSG00000225756	*DBH-AS1*	-1.328593185	-0.034905802	-0.046911941	0.012006139	0.999933887	0.064142221	-0.335730382	0.399872603	0.488539347
ENSG00000188215	*DCUN1D3*	4.409046874	-0.028728299	-0.015572139	-0.01315616	0.999933887	0.160211445	0.28636134	-0.126149896	0.488666322
ENSG00000231758	*ARHGAP15-A*	0.522444531	0.386812278	0.218335802	0.168476476	0.999933887	-0.106492099	0.466069054	-0.572561153	0.488737542
ENSG00000100722	*ZC3H14*	2.377089804	-0.118277647	-0.128016478	0.009738831	0.999933887	-0.072263593	0.066548687	-0.13881228	0.488774829
ENSG00000237702	*TRBV3-1*	-0.049355263	-0.018291216	-0.238105303	0.219814087	0.999933887	-0.104525745	0.151037171	-0.255562917	0.488774829
ENSG00000089486	*CDIP1*	4.675514441	0.006369059	0.020277457	-0.013908398	0.999933887	0.248410673	0.343417459	-0.095006785	0.488774829
ENSG00000156467	*UQCRB*	4.930959677	-0.046659172	-0.084889105	0.038229933	0.999933887	-0.008200207	0.080265958	-0.088466165	0.488810248
ENSG00000132196	*HSD17B7*	2.007790488	0.098549951	-0.059775832	0.158325784	0.999933887	-0.077217104	-0.215420528	0.138203423	0.48910632
ENSG00000121022	*COPS5*	4.085644608	-0.118750417	-0.113097259	-0.005653158	0.999933887	-0.2555307	-0.175431832	-0.080098868	0.489145362
ENSG00000166822	*TMEM170A*	4.876613202	0.01239322	0.005118549	0.00727467	0.999933887	-0.109085638	-0.022230873	-0.086854766	0.489386579
ENSG00000272008	*AL139274.2*	0.773867483	0.065398221	-0.315161219	0.38055944	0.999933887	0.766057059	1.014271104	-0.248214045	0.489454857
ENSG00000211445	*GPX3*	3.272076304	0.085043638	0.096207502	-0.011163864	0.999933887	1.471413697	1.662939044	-0.191525347	0.489627859
ENSG00000111775	*COX6A1*	2.887997002	-0.046069886	0.012213037	-0.058282923	0.999933887	0.023310264	-0.10345734	0.126767605	0.490176223
ENSG00000173875	*ZNF791*	3.732478539	0.022379093	-0.140500802	0.162879896	0.999933887	-0.216176232	-0.322976094	0.106799862	0.490176223
ENSG00000235016	*SEMA3F-AS1*	2.797831771	-0.031977816	0.139409399	-0.171387214	0.962755146	0.028137832	0.148214282	-0.12007645	0.490222102
ENSG00000254263	*AC022973.4*	1.237041793	0.036764013	0.1939866	-0.157222588	0.999933887	0.038669458	0.342108764	-0.303439306	0.490291187
ENSG00000166435	*XRRA1*	2.555580829	0.114607235	0.087349965	0.02725727	0.999933887	-0.095504277	-0.258871658	0.163367381	0.490315298
ENSG00000152133	*GPATCH11*	1.530585284	-0.067159316	-0.139322935	0.072163619	0.999933887	-0.136841773	0.059935746	-0.196777519	0.490315298
ENSG00000163734	*CXCL3*	4.834718089	0.716158847	0.966262516	-0.250103669	0.999933887	1.961179756	2.237087677	-0.275907921	0.490315896
ENSG00000125834	*STK35*	5.676223077	0.009608126	-0.030247342	0.039855468	0.999933887	-0.040482867	-0.146814768	0.106331901	0.490315896
ENSG00000156671	*SAMD8*	5.85173168	-0.003418768	0.02403165	-0.027450418	0.999933887	-0.55398796	-0.429128317	-0.124859643	0.490315896
ENSG00000001461	*NIPAL3*	2.714879197	-0.016042288	0.077225483	-0.093267771	0.999933887	0.026056268	-0.107333005	0.133389272	0.49056007
ENSG00000172354	*GNB2*	8.592488114	0.111221355	0.093622314	0.017599041	0.999933887	-0.269351143	-0.3729087	0.103557557	0.490675617
ENSG00000135956	*TMEM127*	8.184712541	-0.052529943	-0.029045902	-0.023484041	0.999933887	-0.241919064	-0.159371088	-0.082547975	0.490675617
ENSG00000130559	*CAMSAP1*	3.328732189	-0.033450242	-0.002640318	-0.030809924	0.999933887	-0.062061561	-0.17061127	0.108549709	0.491123168
ENSG00000198331	*HYLS1*	-0.652500195	-0.158746702	0.411012186	-0.569758889	0.999933887	0.123326644	0.44679334	-0.323466696	0.49113718
ENSG00000108590	*MED31*	1.480899419	-0.076382862	0.173729695	-0.250112557	0.999933887	-0.364521638	-0.523507127	0.158985489	0.49113718
ENSG00000111181	*SLC6A12*	1.102400584	-0.09566546	-0.084034826	-0.011630634	0.999933887	2.27358502	2.50392784	-0.23034282	0.491252624
ENSG00000116032	*GRIN3B*	0.511329102	0.079725567	0.103053423	-0.023327856	0.999933887	-0.158992792	-0.444080631	0.285087839	0.491618442
ENSG00000286408	*AC113410.4*	0.139566356	0.289875745	0.187649013	0.102226732	0.999933887	0.499481987	0.234734141	0.264747847	0.491827032
ENSG00000233038	*PTPRN2-AS1*	-0.98643194	0.01243753	0.098525934	-0.086088403	0.999933887	-0.229405161	-0.718194716	0.488789555	0.491890247
ENSG00000103942	*HOMER2*	-0.343608626	-0.2116465	-0.297881692	0.086235192	0.999933887	-0.32317873	-0.645919833	0.322741103	0.492077219
ENSG00000174695	*TMEM167A*	5.12159276	-0.060993384	-0.08867816	0.027684776	0.999933887	-0.50207887	-0.393547881	-0.108530989	0.492077219
ENSG00000213613	*RPL11P3*	1.58977303	-0.100397463	-0.004372364	-0.096025099	0.999933887	-0.463157209	-0.809080591	0.345923381	0.492427991
ENSG00000129696	*TTI2*	1.497238602	-0.065354506	-0.118515443	0.053160937	0.999933887	-0.201921264	-0.378585097	0.176663833	0.492427991
ENSG00000138326	*RPS24*	6.786519567	-0.025027271	-0.100529436	0.075502165	0.999933887	0.05985224	0.148390964	-0.088538724	0.492588954
ENSG00000137038	*DMAC1*	1.55686242	-0.009201237	-0.175961083	0.166759845	0.999933887	0.082889	-0.07214346	0.15503246	0.492588954
ENSG00000167283	*ATP5MG*	2.925260623	-0.116316734	-0.042001753	-0.074314981	0.999933887	0.019129376	-0.127253192	0.146382567	0.492613105
ENSG00000108423	*TUBD1*	0.973579851	0.100085567	0.091071468	0.009014099	0.999933887	-0.07355337	0.153226215	-0.226779585	0.492686586
ENSG00000280927	*CTBP1-AS*	1.740942223	0.152589793	0.017888835	0.134700957	0.999933887	-0.248984007	-0.075354768	-0.173629239	0.492978019
ENSG00000165949	*IFI27*	-1.390444154	-0.102594871	-0.200447397	0.097852526	0.999933887	0.223651927	-0.248760297	0.472412224	0.493099381
ENSG00000066855	*MTFR1*	1.588290172	-0.021982988	-0.089438626	0.067455637	0.999933887	-0.012490414	0.18223125	-0.194721665	0.493134419
ENSG00000262420	*AC007613.1*	1.30278892	0.174944631	0.23263579	-0.057691159	0.999933887	0.623103729	0.902257224	-0.279153495	0.493520863
ENSG00000121957	*GPSM2*	0.616373922	-0.143550897	0.018154147	-0.161705043	0.999933887	-0.410923426	-0.67700111	0.266077683	0.493521035
ENSG00000250462	*LRRC37BP1*	-0.268550867	0.280964939	-0.269677044	0.550641983	0.999933887	-0.145863353	-0.482125947	0.336262594	0.493676933
ENSG00000075142	*SRI*	3.654573091	-0.098943724	-0.098999567	5.5843e-05	0.999933887	-0.149193419	-0.052806474	-0.096386945	0.494021875
ENSG00000029993	*HMGB3*	-0.505209289	-0.078205216	0.252520737	-0.330725952	0.999933887	-0.044032443	-0.441891021	0.397858577	0.494021875
ENSG00000243477	*NAA80*	1.433725012	0.073617353	0.047258743	0.02635861	0.999933887	0.264214791	0.089784889	0.174429901	0.494048467
ENSG00000132704	*FCRL2*	-0.75856619	0.016962047	-0.167113771	0.184075818	0.999933887	-0.223596841	0.063154007	-0.286750848	0.494108737
ENSG00000233334	*FAM53B-AS1*	0.269067632	0.156592935	0.204383011	-0.047790075	0.999933887	-0.203657024	-0.724776567	0.521119543	0.494145005
ENSG00000096006	*CRISP3*	-0.528841823	-0.1276447	-0.117418178	-0.010226522	0.999933887	-0.28814158	0.065224303	-0.353365884	0.494145005
ENSG00000186918	*ZNF395*	2.800098595	-0.076957621	-0.094959708	0.018002087	0.999933887	-0.011761315	-0.117058954	0.105297639	0.49418814
ENSG00000136518	*ACTL6A*	1.523052618	-0.154925892	-0.159973756	0.005047864	0.999933887	0.037932543	0.186161561	-0.148229018	0.494230283
ENSG00000104218	*CSPP1*	1.891387306	-0.092078201	0.000420354	-0.092498556	0.999933887	-0.085341497	0.091386589	-0.176728086	0.494325254
ENSG00000169857	*AVEN*	0.25704978	0.069208644	0.117660987	-0.048452343	0.999933887	0.217940728	-0.033247704	0.251188432	0.494325254
ENSG00000276517	*AL133243.3*	1.15160527	-0.039284901	0.136350862	-0.175635763	0.999933887	0.01252396	-0.255225434	0.267749395	0.494325254
ENSG00000276293	*PIP4K2B*	3.853274934	0.025150745	0.011221717	0.013929028	0.999933887	-0.077907549	-0.171172857	0.093265308	0.494476605
ENSG00000117118	*SDHB*	4.842825092	0.041539863	-0.034621775	0.076161638	0.999933887	-0.091912733	-0.190152903	0.09824017	0.494507368
ENSG00000179115	*FARSA*	2.491384637	0.083626744	0.142470642	-0.058843898	0.999933887	-0.002079268	-0.132902825	0.130823557	0.494638563
ENSG00000140398	*NEIL1*	2.295230662	0.091333146	0.133853552	-0.042520406	0.999933887	-0.094722383	0.037439252	-0.132161635	0.494879998
ENSG00000164074	*ABHD18*	2.713659784	0.060307766	0.027159163	0.033148604	0.999933887	-0.394391333	-0.563691938	0.169300606	0.494879998
ENSG00000182195	*LDOC1*	-0.673621911	0.116330584	0.106314809	0.010015775	0.999933887	0.630165622	0.270709648	0.359455974	0.494879998
ENSG00000243279	*PRAF2*	2.460670722	0.075267665	0.119374376	-0.044106711	0.999933887	-0.206857723	-0.357201994	0.150344272	0.495046916
ENSG00000215769	*ARHGAP27P1*	1.117126807	-0.067806321	-0.116580705	0.048774384	0.999933887	-0.093090623	-0.333294654	0.240204032	0.495046916
ENSG00000246477	*AF131216.1*	-0.25286279	0.030293276	0.171799204	-0.141505928	0.999933887	-0.052105223	0.226357016	-0.278462239	0.495046916
ENSG00000170296	*GABARAP*	3.130871657	-0.012790772	-0.014695905	0.001905133	0.999933887	0.365780165	0.492056113	-0.126275948	0.495046916
ENSG00000162971	*TYW5*	1.887446826	-0.01751339	-0.173379653	0.155866263	0.999933887	-0.06718116	0.137544941	-0.204726101	0.495046916
ENSG00000260618	*AC025917.1*	-0.039393207	-0.011517189	0.089631429	-0.101148619	0.999933887	-0.511753194	-0.924140913	0.412387719	0.495046916
ENSG00000163833	*FBXO40*	0.395831396	-0.000641104	0.378439245	-0.379080349	0.944094537	-0.263195513	0.01601197	-0.279207483	0.495046916
ENSG00000186166	*CENATAC*	4.19682081	-0.074507669	0.082367303	-0.156874972	0.944094537	-0.115491244	-0.189983739	0.074492495	0.495047347
ENSG00000132155	*RAF1*	9.14395931	-0.038894822	-0.008778919	-0.030115903	0.999933887	-0.413174129	-0.474653021	0.061478891	0.495075506
ENSG00000258512	*LINC00239*	3.891267104	0.155631931	0.247224314	-0.091592383	0.999933887	2.577695291	2.786826832	-0.209131541	0.495255332
ENSG00000109572	*CLCN3*	3.861001803	-0.067715724	-0.064041862	-0.003673862	0.999933887	-0.088344218	0.010920786	-0.099265004	0.495255332
ENSG00000113356	*POLR3G*	-0.475273694	0.173394867	-0.199730344	0.373125211	0.999933887	0.209045454	-0.242741738	0.451787192	0.495255332
ENSG00000091106	*NLRC4*	2.562664026	-0.072358931	-0.1527251	0.08036617	0.999933887	-0.373688721	-0.165290249	-0.208398472	0.495255332
ENSG00000205022	*PABPN1L*	-0.979480997	-0.06431504	0.191389857	-0.255704897	0.999933887	0.054827379	-0.384302197	0.439129577	0.495255332
ENSG00000267412	*AC092068.2*	-0.731925296	-0.00626347	0.616983827	-0.623247297	0.999933887	0.397082856	-0.022059089	0.419141945	0.495255332
ENSG00000196236	*XPNPEP3*	2.114669819	-0.066314146	-0.071648991	0.005334845	0.999933887	-0.224241661	-0.385128584	0.160886923	0.495316935
ENSG00000275714	*H3C1*	-0.584902775	0.179704442	-0.167138675	0.346843117	0.999933887	0.070414151	0.512161981	-0.441747829	0.495387186
ENSG00000100036	*SLC35E4*	1.283182174	0.076234707	-0.075552331	0.151787037	0.999933887	0.748345644	1.000653692	-0.252308048	0.495782843
ENSG00000163875	*MEAF6*	3.547492338	0.042328804	0.006934702	0.035394102	0.999933887	-0.100006709	-0.197109116	0.097102408	0.495855098
ENSG00000204611	*ZNF616*	0.51439801	-0.065653937	-0.246108045	0.180454108	0.999933887	-0.568987365	-0.343155376	-0.225831989	0.495855098
ENSG00000114784	*EIF1B*	5.39850803	0.01410243	-0.011640832	0.025743262	0.999933887	0.392807224	0.488870623	-0.096063399	0.495855098
ENSG00000259768	*AC004943.2*	0.379053865	-0.032409784	0.002391006	-0.03480079	0.999933887	0.090103811	0.289610598	-0.199506787	0.495855098
ENSG00000104375	*STK3*	2.165146105	0.063859762	0.068534328	-0.004674566	0.999933887	0.137224161	0.326824983	-0.189600822	0.496008676
ENSG00000104490	*NCALD*	1.363678786	0.080015462	-0.150150238	0.230165701	0.999933887	0.029216205	0.24518852	-0.215972315	0.496008676
ENSG00000145936	*KCNMB1*	-0.493313337	0.096025323	0.035861561	0.060163762	0.999933887	0.735993271	1.085796633	-0.349803363	0.496008676
ENSG00000163428	*LRRC58*	3.654352226	0.045208354	-0.155237222	0.200445576	0.999933887	-0.509990627	-0.296073219	-0.213917408	0.496172135
ENSG00000126215	*XRCC3*	2.899974364	0.04299559	-0.044559127	0.087554717	0.999933887	-0.198390063	-0.320809621	0.122419557	0.496185202
ENSG00000080845	*DLGAP4*	5.233023584	0.028159992	-0.009823872	0.037983864	0.999933887	0.16511521	0.258768344	-0.093653134	0.496501038
ENSG00000138688	*KIAA1109*	6.700355284	-0.07092154	-0.079807926	0.008886386	0.999933887	-0.499640133	-0.227682282	-0.271957851	0.496501038
ENSG00000088986	*DYNLL1*	3.469689724	0.003386643	0.020801361	-0.017414718	0.999933887	-0.014421419	0.103638709	-0.118060128	0.496539336

ENSG00000167397	*VKORC1*	0.375127244	-0.032781773	-0.146267985	0.113486211	0.999933887	-0.210391297	0.046988402	-0.257379699	0.496611333
ENSG00000184990	*SIVA1*	4.524948434	0.075304556	0.01707589	0.058228666	0.999933887	0.079383473	-0.02652415	0.105907623	0.496760799
ENSG00000154723	*ATP5PF*	2.617105616	0.037273836	0.080839791	-0.043565955	0.999933887	-0.163671204	-0.315997786	0.152326582	0.496760799
ENSG00000171044	*XKR6*	1.17168196	0.089270328	0.103361433	-0.014091104	0.999933887	0.322848869	0.52067812	-0.197829251	0.496773428
ENSG00000171916	*LGALS9C*	-0.421263874	-0.030058369	0.078863456	-0.108921825	0.999933887	-1.24413375	-1.667594176	0.423460427	0.496773428
ENSG00000011201	*ANOS1*	-1.289336818	-0.094015344	0.532583345	-0.626598689	0.999933887	-0.878265414	-1.302971291	0.424705877	0.496926622
ENSG00000125877	*ITPA*	2.322785031	0.091247835	0.054959845	0.03628799	0.999933887	0.101691103	-0.038364083	0.140055186	0.497018518
ENSG00000185800	*DMWD*	2.987900865	0.085523967	-0.079603935	0.165127903	0.999933887	0.390764148	0.585323009	-0.194558861	0.497089245
ENSG00000102409	*BEX4*	2.389766013	0.001121019	0.124712316	-0.123591296	0.999933887	0.183116947	0.054337722	0.128779225	0.497107045
ENSG00000158747	*NBL1*	0.486250309	0.133766614	-0.123193628	0.256960242	0.999933887	0.338502044	0.021617574	0.31688447	0.497115576
ENSG00000186174	*BCL9L*	5.659977137	0.06029591	0.096197071	-0.03590116	0.999933887	0.755966629	0.899417259	-0.143450631	0.497116738
ENSG00000132005	*RFX1*	6.173403948	0.01878893	0.054468383	-0.035679453	0.999933887	-0.039034525	0.047381458	-0.086415983	0.497116738
ENSG00000198890	*PRMT6*	0.830754139	-0.039846947	0.191889836	-0.231736783	0.999933887	0.156855507	0.370306018	-0.213450511	0.497116738
ENSG00000067167	*TRAM1*	5.415816395	0.006927656	0.001278743	0.005648912	0.999933887	-0.261568148	-0.356370598	0.09480245	0.497116738
ENSG00000260060	*AC009088.1*	-0.228318228	0.188023921	0.198678724	-0.010654803	0.999933887	-0.185713994	0.219941931	-0.405655925	0.497781991
ENSG00000156030	*MIDEAS*	7.358916264	0.031687955	0.079575908	-0.047887953	0.999933887	-0.377538258	-0.469590973	0.092052715	0.497781991
ENSG00000159445	*THEM4*	2.214424704	-0.013240848	0.052751224	-0.065992072	0.999933887	-0.016095436	0.113495869	-0.129591306	0.497781991
ENSG00000182389	*CACNB4*	0.883132462	-0.051187623	-0.172272743	0.12108512	0.999933887	-0.622039038	-0.247141426	-0.374897612	0.497966359
ENSG00000166266	*CUL5*	2.508524128	-0.217926581	-0.05016653	-0.167760051	0.999933887	-0.376097194	-0.193472022	-0.182625172	0.498212765
ENSG00000116473	*RAP1A*	7.22166791	-0.031081581	-0.049605223	0.018523642	0.999933887	-0.292811754	-0.372801448	0.079989694	0.498212765
ENSG00000078596	*ITM2A*	4.206092762	-0.021577416	-0.123787222	0.102209807	0.999933887	0.114277217	0.223063407	-0.108786191	0.498212765
ENSG00000070814	*TCOF1*	4.451965473	0.040417461	0.094559405	-0.054141944	0.999933887	0.041048973	-0.056449981	0.097498954	0.498892697
ENSG00000099985	*OSM*	8.586899258	0.7293827	0.626963386	0.102419314	0.999933887	2.530232093	2.798648284	-0.268416191	0.499148432
ENSG00000167996	*FTH1*	12.864689448	0.150606785	0.102868728	0.047738058	0.999933887	0.596843273	0.705317432	-0.108474159	0.499148432
ENSG00000105982	*RNF32*	-0.320509166	-0.214509324	0.0243237	-0.238833024	0.999933887	-0.463831471	-0.154579428	-0.309252043	0.499148432
ENSG00000152404	*CWF19L2*	2.784663145	-0.12929782	-0.195088465	0.065790645	0.999933887	-0.096593283	0.080953247	-0.17754653	0.499148432
ENSG00000164114	*MAP9*	0.089083895	-0.130071256	-0.195243959	0.065172703	0.999933887	-0.140067014	0.213510363	-0.353577377	0.499148432
ENSG00000153046	*CDYL*	3.702420419	0.05640554	0.034956668	0.021448872	0.999933887	-0.284000844	-0.189366082	-0.094634762	0.499148432
ENSG00000233483	*AC008105.2*	0.512721484	0.042548112	-0.063838991	0.106387103	0.999933887	-0.289584538	-0.015943236	-0.273641302	0.499148432
ENSG00000167680	*SEMA6B*	5.1475801	0.041203893	0.071983518	-0.030779625	0.999933887	0.421938657	0.569708605	-0.147769948	0.499329262
ENSG00000258539	*AC068896.1*	-0.947833477	0.137614655	0.117452094	0.020162561	0.999933887	0.153291117	-0.283996079	0.437287196	0.499366246
ENSG00000196345	*ZKSCAN7*	-0.475264771	0.110223912	-0.264002036	0.374225948	0.999933887	-0.576877699	-0.897106617	0.320228918	0.499366246
ENSG00000211895	*IGHA1*	2.63314422	0.025335584	-0.023950346	0.04928593	0.999933887	0.333993823	0.08699137	0.247002454	0.499366246
ENSG00000181904	*C5orf24*	2.347219045	0.046487848	0.051533873	-0.005046026	0.999933887	-0.279502048	-0.428971481	0.149469433	0.499366954
ENSG00000071575	*TRIB2*	4.393174739	-0.035987414	0.057358845	-0.093346259	0.999933887	-0.005585449	-0.0896287	0.084043252	0.499416677
ENSG00000187122	*SLIT1*	0.75960102	-0.076713981	0.049027152	-0.125741133	0.999933887	0.069252518	0.387825103	-0.318572585	0.499662876
ENSG00000069943	*PIGB*	4.224673673	-0.060508425	-0.028459004	-0.032049421	0.999933887	-0.180378211	-0.286778572	0.106400362	0.499885043
ENSG00000268355	*AC243960.3*	2.186389891	0.031959107	-0.004502676	0.036461783	0.999933887	-0.01269733	0.18922745	-0.20192478	0.499885043
ENSG00000141428	*C18orf21*	1.681579965	-0.089213667	-0.073440353	-0.015773314	0.999933887	-0.446438627	-0.613089151	0.166650524	0.500033797
ENSG00000198435	*NRARP*	2.217924339	0.072767974	0.056970346	0.015797628	0.999933887	-0.255634459	-0.062079082	-0.193555377	0.500033797
ENSG00000155561	*NUP205*	3.557896121	-0.041101546	-0.055417112	0.014315566	0.999933887	0.069813671	0.175389907	-0.105576236	0.500033797
ENSG00000204576	*PRR3*	1.915734987	0.093342979	0.099233454	-0.005890475	0.999933887	-0.050901733	-0.207316122	0.156414389	0.500074033
ENSG00000013364	*MVP*	8.005463255	0.051745676	0.051942453	-0.000196777	0.999933887	0.178937606	0.087324894	0.091612712	0.500074033
ENSG00000165637	*VDAC2*	4.199860154	0.03356231	-0.00795988	0.04152219	0.999933887	-0.088399101	-0.175621217	0.087222116	0.500074033
ENSG00000270589	*AL158163.1*	1.253680729	-0.175244681	0.019426371	-0.194671052	0.999933887	0.334777141	0.608182015	-0.273404873	0.500279595
ENSG00000104936	*DMPK*	2.548901876	0.031237436	0.002857099	0.028380337	0.999933887	0.356248873	0.533253001	-0.177004128	0.500286697
ENSG00000041880	*PARP3*	0.890282165	0.197923553	-0.023263229	0.221186781	0.999933887	0.128911841	-0.077890356	0.206802198	0.500460324
ENSG00000018280	*SLC11A1*	10.21375508	0.025723341	0.068278905	-0.042555564	0.999933887	-0.118529478	-0.190766473	0.072236995	0.500460324
ENSG00000268670	*AC016586.1*	0.40291897	-0.061288381	0.097454953	-0.158743334	0.999933887	-0.136510469	0.111363025	-0.247873493	0.500539945
ENSG00000178935	*ZNF552*	2.784098071	-0.147570449	-0.117413715	-0.030156734	0.999933887	-0.469062551	-0.611840815	0.142778264	0.500639794
ENSG00000130589	*HELZ2*	8.297198147	0.058524535	0.084023088	-0.025498554	0.999933887	0.128717816	0.031232979	0.097484837	0.500639794
ENSG00000070770	*CSNK2A2*	2.760923538	0.050367372	0.078046643	-0.027679271	0.999933887	0.002175338	0.122118973	-0.119943634	0.500659053
ENSG00000205413	*SAMD9*	6.228931383	-0.135162393	-0.191000468	0.055838075	0.999933887	-0.812341467	-0.574957589	-0.237383877	0.500952583
ENSG00000228696	*ARL17B*	-0.622381786	0.168137057	0.01790659	0.150230466	0.999933887	-0.341533183	0.035273736	-0.376806919	0.501004495
ENSG00000182247	*UBE2E2*	-0.840533112	0.142782793	-0.006380406	0.149163198	0.999933887	0.526588951	0.819387648	-0.292798697	0.501004495
ENSG00000111412	*SPRING1*	4.5520569	-0.015187886	-0.065668118	0.050480232	0.999933887	-0.284297678	-0.182475748	-0.10182193	0.501004495
ENSG00000138794	*CASP6*	0.130223364	-0.105708593	-0.092963441	-0.012745152	0.999933887	-0.127870715	0.154224712	-0.282095427	0.50101113
ENSG00000089022	*MAPKAPK5*	2.934497142	-0.09433718	-0.039192483	-0.055144697	0.999933887	0.054831822	-0.040548913	0.095380735	0.501136654
ENSG00000245910	*SNHG6*	3.769339957	0.035483044	-0.06541128	0.100894323	0.999933887	0.075222815	-0.024640948	0.099863762	0.501136654
ENSG00000122642	*FKBP9*	-0.569512472	-0.171478982	0.036935888	-0.20841487	0.999933887	-0.263803245	-0.733541827	0.469738583	0.501164309
ENSG00000055957	*ITIH1*	-1.668595504	-0.312694584	-0.081337061	-0.231357523	0.999933887	2.290973827	2.678428974	-0.387455147	0.501164309
ENSG00000232818	*RPS2P32*	-0.097719879	0.129630665	-0.042549585	0.17218025	0.999933887	-0.032050989	0.338897821	-0.37094881	0.501164309
ENSG00000258476	*LINC02207*	2.288696801	0.012139222	-0.08851907	0.100658292	0.999933887	0.466046116	0.307793784	0.158252332	0.501164309
ENSG00000132256	*TRIM5*	3.441777789	0.027141906	-0.012432701	0.039574607	0.999933887	0.103509874	-0.000503179	0.104013053	0.501302794
ENSG00000233231	*HNRNPA1P49*	1.289217877	0.105659883	-0.026360337	0.13202022	0.999933887	0.248704047	0.467595556	-0.218891508	0.501431076
ENSG00000105948	*TTC26*	-0.799396049	0.181199921	-0.096234999	0.27743492	0.999933887	0.202099661	-0.186007772	0.388107433	0.501520562
ENSG00000233261	*FAM238A*	-0.268747849	0.057260909	-0.164759084	0.222019993	0.999933887	0.107578829	-0.31019992	0.41777875	0.501597927
ENSG00000273742	*MIR6075*	1.358474719	0.157949533	-0.042500961	0.200450494	0.999933887	0.314932241	0.112924494	0.202007747	0.501701176
ENSG00000105254	*TBCB*	4.129944754	0.075798953	0.022732476	0.053066477	0.999933887	0.022045339	-0.087113781	0.10915912	0.501874175
ENSG00000286966	*AL137800.1*	-0.278513819	-0.130084628	-0.129739778	-0.000344851	0.999933887	-0.709941709	-0.931730348	0.221788639	0.501874175
ENSG00000111788	*AC009533.1*	-0.456125656	0.11098933	-0.106482577	0.217471907	0.999933887	-0.120943637	0.185739363	-0.306683	0.501874175
ENSG00000189241	*TSPYL1*	5.465299011	-0.021522668	0.021111253	-0.042633922	0.999933887	0.105556648	0.026003013	0.079553635	0.501874175
ENSG00000145743	*FBXL17*	2.486049781	0.036875853	0.046799157	-0.009923304	0.999933887	-0.138242474	0.051365901	-0.189608375	0.501874175
ENSG00000083097	*DOP1A*	2.670721932	0.014221744	-0.208613496	0.22283524	0.999933887	-0.222185138	-0.000517869	-0.22166727	0.501874175
ENSG00000179933	*C14orf119*	3.494208778	-0.034845432	0.015003986	-0.049849418	0.999933887	0.006827304	0.105629195	-0.098801891	0.501937492
ENSG00000148672	*GLUD1*	4.230430496	-0.043447566	-0.081391555	0.037943989	0.999933887	-0.065243506	-0.156774131	0.091530625	0.501983422
ENSG00000232539	*LINC00945*	-0.228483636	-0.441307239	-0.679726165	0.238418926	0.999933887	-0.172004144	0.071937286	-0.243941431	0.502030367
ENSG00000198648	*STK39*	2.454658503	-0.05418664	0.113936371	-0.168123011	0.999933887	0.065360496	0.211909921	-0.146549424	0.502030367
ENSG00000259881	*AC092384.2*	-0.119615063	-0.109357592	-0.004929675	-0.104427918	0.999933887	-0.804153892	-1.11713205	0.312978158	0.502030367
ENSG00000267940	*AC022762.2*	-0.253744399	0.257767504	-0.165102534	0.422870038	0.999933887	1.659224872	1.231106823	0.428118048	0.502194492
ENSG00000158042	*MRPL17*	0.667292656	-0.137079535	-0.132818673	-0.004260863	0.999933887	-0.088152535	0.121830677	-0.209983212	0.502194492
ENSG00000143322	*ABL2*	4.085360612	0.046991794	0.069907595	-0.022915801	0.999933887	0.468889417	0.596419145	-0.127529728	0.502194492
ENSG00000197815	*AC122129.1*	0.612033319	0.039178922	0.094952982	-0.055774059	0.999933887	-0.22657646	0.046066952	-0.272643412	0.502194492
ENSG00000139899	*CBLN3*	-0.453677513	-0.024361382	0.219604198	-0.243965581	0.999933887	0.167463691	0.457645555	-0.290181863	0.502194492
ENSG00000172888	*ZNF621*	2.783797037	-0.074964636	-0.033503915	-0.041460721	0.999933887	0.022942732	-0.104913415	0.127856147	0.50221915
ENSG00000170190	*SLC16A5*	6.315908043	0.047103051	-0.05768531	0.104788361	0.999933887	0.006111598	0.081168246	-0.075056648	0.50221915
ENSG00000130653	*PNPLA7*	0.660678052	0.046978757	0.022977352	0.024001404	0.999933887	0.02178335	0.247603715	-0.225820364	0.50221915
ENSG00000261351	*AC116913.1*	0.313834857	-0.112833029	0.082347406	-0.195180435	0.999933887	0.595378466	0.859235896	-0.26385743	0.50238545
ENSG00000196505	*GDAP2*	3.775532973	0.007891685	-0.059065087	0.066956773	0.999933887	-0.615134384	-0.763362147	0.148227763	0.502614349
ENSG00000134690	*CDCA8*	0.505421779	-0.180198869	-0.27557952	0.095380651	0.999933887	-0.158001571	-0.365435087	0.207433515	0.502645694
ENSG00000167005	*NUDT21*	3.93159098	0.022956195	-0.06846096	0.091417156	0.999933887	-0.176941498	-0.280950539	0.104009041	0.502646978
ENSG00000136122	*BORA*	-0.364091141	0.283896023	-0.399479988	0.683376011	0.944094537	-0.060024536	0.238716857	-0.298741393	0.502674747
ENSG00000143569	*UBAP2L*	8.153463981	-0.01416755	0.006154177	-0.020321726	0.999933887	0.206274386	0.294756082	-0.088481696	0.502674747
ENSG00000163660	*CCNL1*	10.086549896	0.050855549	0.001363187	0.049492362	0.999933887	0.411324539	0.538185326	-0.126860787	0.502708338
ENSG00000253314	*LINC00293*	-0.417888911	0.004186705	0.41710968	-0.412922975	0.999933887	0.109556869	0.401121203	-0.291564334	0.502708338
ENSG00000102057	*KCND1*	2.122656242	0.058474799	-0.033013839	0.091488638	0.999933887	0.285034733	0.449814927	-0.164780194	0.502809692
ENSG00000196470	*SIAH1*	4.06645623	-0.047927757	0.031855209	-0.079782966	0.999933887	0.421632589	0.529713043	-0.108080454	0.502879388
ENSG00000180611	*MB21D2*	-0.609371813	0.040451591	0.049200053	-0.008748461	0.999933887	0.359289504	0.820144468	-0.460854965	0.502954405
ENSG00000277147	*LINC00869*	2.525183477	0.064082978	0.135149851	-0.071066873	0.999933887	0.18462014	0.323601042	-0.138980902	0.503202641
ENSG00000136874	*STX17*	3.766230647	-0.018564572	-0.051211333	0.032646761	0.999933887	-0.228942671	-0.0869694	-0.141973271	0.503202641
ENSG00000198171	*DDRGK1*	2.971070445	0.089434137	0.122507193	-0.033073056	0.999933887	0.093276285	-0.070755103	0.164031388	0.503206377
ENSG00000178921	*PFAS*	2.432847631	0.065072265	0.033365714	0.031706551	0.999933887	0.1734279	0.310917104	-0.137489204	0.503206377
ENSG00000140992	*PDPK1*	6.406993182	-0.017696553	-0.03600259	0.018306036	0.999933887	-0.676384386	-0.764611419	0.088227033	0.503206377
ENSG00000165704	*HPRT1*	2.092083839	-0.004469075	0.072200338	-0.076669413	0.999933887	0.055156313	0.203272186	-0.148115874	0.5035824
ENSG00000255040	*MORF4L1P3*	-0.863782885	0.258400673	0.084508928	0.173891745	0.999933887	-0.479079165	-0.960945875	0.48186671	0.50362464

ENSG00000286808	*AC092338.3*	-0.332939123	-0.106418166	0.170960801	-0.277378967	0.999933887	0.098084983	-0.211187302	0.309272286	0.50362464
ENSG00000157017	*GHRL*	2.76140388	-0.070915411	0.046824961	-0.117740372	0.999933887	1.138796146	1.300394894	-0.161598749	0.503930217
ENSG00000286076	*AC005050.3*	3.173407327	0.370895078	0.283893771	0.087001307	0.999933887	1.492894687	1.69104823	-0.198153542	0.50411223
ENSG00000166166	*TRMT61A*	1.600141299	0.120753933	0.234331202	-0.113577269	0.999933887	0.183289037	0.332958235	-0.149669198	0.504129248
ENSG00000165521	*EML5*	-0.919933931	0.167789511	-0.179191522	0.346981033	0.999933887	-0.171217069	0.19236762	-0.363584689	0.504129248
ENSG00000279253	*AL121753.2*	1.574197413	0.02050693	0.091768089	-0.071261159	0.999933887	-0.24708439	-0.466433566	0.219349176	0.504129248
ENSG00000108557	*RAI1*	3.159884442	0.070746453	0.163766418	-0.093019965	0.999933887	-0.22341199	-0.323250575	0.099838585	0.504268111
ENSG00000260452	*TPRKBP2*	0.01973934	0.152115923	0.027919974	0.124195949	0.999933887	-1.570981851	-1.11894572	-0.452036131	0.504314855
ENSG00000055950	*MRPL43*	2.317172638	0.068827907	0.0254041	0.043423807	0.999933887	-0.017884622	-0.168256033	0.150371411	0.504398234
ENSG00000274256	*AL391903.2*	1.691911647	0.132796437	0.044621088	0.088175349	0.999933887	0.485316716	0.718383216	-0.2330665	0.504537766
ENSG00000111058	*ACSS3*	1.256757361	-0.062908298	-0.028041386	-0.034866912	0.999933887	-0.923870578	-1.152103155	0.228232577	0.504823413
ENSG00000259651	*MTCO3P23*	2.208241005	0.50935204	0.704501292	-0.195149251	0.999933887	2.052939578	2.427598508	-0.37465893	0.504854029
ENSG00000164306	*PRIMPOL*	0.501255242	-0.129059505	0.114422303	-0.243481809	0.999933887	-0.171115563	0.025336189	-0.196451752	0.504854029
ENSG00000101335	*MYL9*	-0.125018008	-0.14781952	0.321891535	-0.469711055	0.999933887	0.195666745	0.466985981	-0.271319235	0.504854029
ENSG00000165359	*INTS6L*	4.443441401	0.019876058	-0.090998289	0.110874347	0.999933887	-0.030875129	0.088182009	-0.119057139	0.504902368
ENSG00000145703	*IQGAP2*	5.786352283	-0.007726209	-0.110808094	0.103081885	0.999933887	-0.330579365	-0.150166609	-0.180412756	0.504902368
ENSG00000136875	*PRPF4*	3.996880943	-0.038690504	-0.032120231	-0.006570273	0.999933887	0.075760328	0.174541761	-0.098781433	0.505128172
ENSG00000101224	*CDC25B*	5.33204401	0.014409805	-0.000616039	0.015025844	0.999933887	0.114163057	0.037516209	0.076646848	0.505256273
ENSG00000105497	*ZNF175*	0.019764992	-0.201534279	0.230675399	-0.432209678	0.999933887	0.05046633	-0.184312591	0.234778922	0.505513518
ENSG00000112893	*MAN2A1*	4.886434227	-0.005692681	-0.104954097	0.099261416	0.999933887	-0.340278684	-0.508422136	0.168143451	0.505513518
ENSG00000251453	*HAUS1P1*	-0.58469624	-0.5089487	-0.093859473	-0.415089227	0.999933887	-0.966047884	-0.574703854	-0.39134403	0.505540972
ENSG00000147896	*IFNK*	-1.148216675	-0.455081338	0.17426143	-0.629342767	0.999933887	0.263924461	0.654783611	-0.39085915	0.506157036
ENSG00000204305	*AGER*	4.93240099	-0.012713476	0.011948547	-0.024662023	0.999933887	0.002722968	-0.094732977	0.097455945	0.506157036
ENSG00000121892	*PDS5A*	4.978110916	-0.0266152	-0.052658129	0.026042929	0.999933887	-0.369761614	-0.254561174	-0.115200439	0.5064395
ENSG00000174744	*BRMS1*	5.250638831	-0.017097744	0.028756104	-0.045853848	0.999933887	-0.145268154	-0.226737151	0.081468997	0.506476341
ENSG00000153179	*RASSF3*	7.965029976	-0.054801888	0.007937497	-0.062739385	0.999933887	-0.244381767	-0.163257236	-0.081124531	0.506643944
ENSG00000134684	*YARS1*	5.461487815	-0.041494681	-0.108524517	0.067029836	0.999933887	0.011436534	0.086608178	-0.075171644	0.506643944
ENSG00000106609	*TMEM248*	5.532386866	-0.02661097	-0.044707294	0.018096323	0.999933887	-0.210583549	-0.282955221	0.072371671	0.506643944
ENSG00000159131	*GART*	2.9952691	-0.033436938	0.087870564	-0.121307502	0.999933887	0.062139331	0.167965827	-0.105826495	0.506643944
ENSG00000230124	*ACBD6*	2.175084836	0.044286272	0.155127787	-0.110841515	0.999933887	0.003976548	0.135060564	-0.131084015	0.506706448
ENSG00000141449	*GREB1L*	0.713664307	-0.057646514	0.401284973	-0.458931487	0.944094537	-0.232721526	0.14127535	-0.373996876	0.506706448
ENSG00000112167	*SAYSD1*	1.379364961	-0.056410431	-0.119379817	0.062969386	0.999933887	-0.070030887	0.082342481	-0.152373367	0.506710645
ENSG00000185900	*POMK*	0.293829071	0.049070521	-0.011927351	0.060997872	0.999933887	-0.172182668	-0.426028348	0.253845681	0.506710645
ENSG00000164543	*STK17A*	5.410886767	-0.024541758	0.078223119	-0.102764877	0.999933887	0.012474644	-0.070792061	0.083266705	0.506754336
ENSG00000115368	*WDR75*	2.703598638	-0.054369066	-0.11828663	0.063917565	0.999933887	0.112118001	0.270283449	-0.158165448	0.506787232
ENSG00000232388	*SMIM26*	1.528394389	-0.186619782	-0.087187068	-0.099432713	0.999933887	-0.154739114	-0.347710262	0.192971148	0.506983094
ENSG00000239280	*AC108693.1*	-0.465735188	-0.275941744	0.313533173	-0.589474916	0.944094537	-0.179349083	0.153271473	-0.332620556	0.506999166
ENSG00000138660	*AP1AR*	4.251953876	-0.139060134	-0.14160859	0.002548456	0.999933887	-0.729832232	-0.566208209	-0.163624022	0.507088707
ENSG00000011376	*LARS2*	1.950179727	0.045391713	0.22987923	-0.184487518	0.999933887	0.057607587	-0.100782747	0.158390334	0.507088707
ENSG00000270344	*POC1B-AS1*	1.745343045	0.151386442	0.028969767	0.122416676	0.999933887	0.975054614	1.195476594	-0.22042198	0.507161352
ENSG00000179627	*ZBTB42*	0.856627726	-0.011440181	0.112944856	-0.124385037	0.999933887	-0.182101714	-0.375182856	0.193081143	0.50720444
ENSG00000277687	*AL139407.1*	2.536732391	-0.037815344	0.00016985	-0.037985194	0.999933887	-0.698444829	-0.527610287	-0.170834542	0.507868255
ENSG00000164405	*UQCRQ*	2.803058354	0.055266285	-0.057490683	0.112756968	0.999933887	0.069184931	-0.060906799	0.13009173	0.507878496
ENSG00000244274	*DBNDD2*	-0.238310246	0.007875736	-0.017148016	0.025023752	0.999933887	1.209114231	0.824956327	0.384157904	0.507878496
ENSG00000267519	*AC020916.1*	9.498186257	0.315787628	0.153963974	0.161823654	0.999933887	1.095978776	1.223774549	-0.127795772	0.50794983
ENSG00000167842	*MIS12*	2.772859467	0.004952636	0.063205044	-0.058252408	0.999933887	0.029911345	0.125023982	-0.095112637	0.50794983
ENSG00000155393	*HEATR3*	1.197282256	-0.018902423	0.011717126	-0.03061955	0.999933887	-0.199685719	-0.429351813	0.229666094	0.508007543
ENSG00000087266	*SH3BP2*	8.482673546	-0.007285988	0.0196591	-0.026945088	0.999933887	0.091842978	0.166035939	-0.074192961	0.508007543
ENSG00000159479	*MED8*	3.244115759	-0.114656087	-0.128400487	0.013744401	0.999933887	-0.44285124	-0.555660335	0.112809095	0.508146372
ENSG00000168411	*RFWD3*	3.783730631	-0.025575916	0.08023122	-0.105807137	0.999933887	0.055521205	-0.058113966	0.113635171	0.508146372
ENSG00000261267	*AC026470.2*	2.19449542	0.055855552	0.023334239	0.032521314	0.999933887	1.400253809	1.594238324	-0.193984515	0.508522275
ENSG00000133561	*GIMAP6*	3.762843219	0.034137734	-0.021212451	0.055350185	0.999933887	0.158308045	0.256692791	-0.098384746	0.508522275
ENSG00000134014	*ELP3*	2.192043767	0.009656899	-0.08002563	0.08968253	0.999933887	0.081127266	0.223609946	-0.14248268	0.508882264
ENSG00000143771	*CNIH4*	2.734669377	-0.017574228	-0.021265624	0.003691395	0.999933887	0.075930294	-0.071988722	0.147919016	0.508931788
ENSG00000171960	*PPIH*	0.908307613	-0.110137124	-0.122243261	0.012106137	0.999933887	-0.075129659	0.113180791	-0.18831045	0.508943212
ENSG00000117543	*DPH5*	1.501390089	-0.06909131	-0.161885364	0.092794053	0.999933887	0.102778872	0.273891889	-0.171113017	0.508943212
ENSG00000278000	*AC139100.2*	-0.243282239	0.090470475	-0.022761012	0.113231487	0.999933887	0.956776296	1.200255667	-0.243479372	0.508998881
ENSG00000167384	*ZNF180*	1.608809122	-0.207813316	0.083520689	-0.291334005	0.999933887	-0.291732148	-0.477617698	0.185885551	0.509001758
ENSG00000225470	*JPX*	0.597668589	0.209143995	0.10970273	0.099441265	0.999933887	-0.36067166	-0.679099844	0.318428183	0.509010248
ENSG00000145817	*YIPF5*	3.459042963	-0.013201822	-0.033982317	0.020780495	0.999933887	-0.108457551	-0.010271652	-0.098185899	0.509247988
ENSG00000240356	*RPL23AP7*	0.422359332	-0.012048552	-0.12289732	0.110848768	0.999933887	-0.499748895	-0.682787967	0.183039072	0.509272311
ENSG00000275888	*AC132872.3*	4.195444893	0.086884807	0.115896905	-0.029012098	0.999933887	-0.303192809	-0.198691457	-0.104501352	0.509310365
ENSG00000167565	*SERTAD3*	4.560026859	0.120506312	0.090527539	0.029978772	0.999933887	-0.801736138	-0.935197448	0.13346131	0.509310365
ENSG00000180817	*PPA1*	2.716345997	-0.069149896	-0.120715099	0.051565203	0.999933887	0.114210162	0.234112519	-0.119902357	0.509310365
ENSG00000117640	*MTFR1L*	2.700835995	0.043660165	0.038618998	0.005041167	0.999933887	-0.176979458	-0.302294849	0.125315391	0.509310365
ENSG00000162300	*ZFPL1*	1.790846319	-0.059307891	0.039798038	-0.099105929	0.999933887	0.027426025	-0.16509972	0.192525745	0.509310365
ENSG00000137441	*FGFBP2*	4.030411868	0.03289071	-0.110459656	0.143350367	0.999933887	0.038381162	0.14567296	-0.107291798	0.509310365
ENSG00000188707	*ZBED6CL*	2.190635261	-0.045513594	-0.000469477	-0.045044117	0.999933887	0.698163568	0.535532866	0.162630702	0.509555954
ENSG00000222808	*RNU4-47P*	0.902028444	-0.20367981	0.078878586	-0.282558395	0.999933887	-0.710180076	-0.472748693	-0.237431383	0.510179851
ENSG00000143222	*UFC1*	3.665744337	-0.040920455	0.023311519	-0.064231974	0.999933887	-0.11065261	-0.213268891	0.102616281	0.510213989
ENSG00000198843	*SELENOT*	6.149118056	-0.054094949	-0.028866643	-0.025228306	0.999933887	-0.044937275	0.032011328	-0.076948604	0.510732453
ENSG00000251002	*AC244502.1*	-0.345887607	0.159436418	-0.094986965	0.254423382	0.999933887	-0.27654248	0.055899462	-0.332441942	0.510732453
ENSG00000072858	*SIDT1*	3.375499841	-0.036140274	-0.019500887	-0.016639387	0.999933887	0.047435695	0.142374212	-0.094938517	0.510732453
ENSG00000261308	*FIGNL2*	0.162602871	0.191808832	0.26682414	-0.075015308	0.999933887	-1.013206369	-0.815772343	-0.197434025	0.510745419
ENSG00000138642	*HERC6*	3.219500971	0.06129968	0.025138432	0.036161248	0.999933887	-0.160871986	-0.300454198	0.139582211	0.510745419
ENSG00000235602	*POU5F1P3*	3.115381747	-0.111854515	-0.142622652	0.030768137	0.999933887	0.128198709	0.265826657	-0.137627948	0.510790676
ENSG00000186517	*ARHGAP30*	9.109167637	-0.035313949	-0.018642192	-0.016671757	0.999933887	-0.640151621	-0.745879811	0.105728189	0.510790676
ENSG00000137310	*TCF19*	0.718824093	-0.022104689	-0.052418702	0.030314013	0.999933887	0.024830768	-0.214951187	0.239781956	0.510790676
ENSG00000143753	*DEGS1*	4.686225975	0.051397343	-0.045084663	0.096482006	0.999933887	0.255403395	0.373001596	-0.117598201	0.510834858
ENSG00000279394	*AC015871.4*	-1.001971841	0.043485049	-0.046402317	0.089887365	0.999933887	-0.21954978	0.381020481	-0.600570261	0.510834858
ENSG00000106665	*CLIP2*	1.761076605	0.113843701	-0.080070075	0.193913776	0.999933887	-0.061526372	-0.250160693	0.188634321	0.510846206
ENSG00000055211	*GINM1*	4.023576516	-0.056469423	-0.003983203	-0.05248622	0.999933887	-0.306093593	-0.403261551	0.097167958	0.51088005
ENSG00000129187	*DCTD*	2.900394579	0.079768707	0.004034126	0.075734581	0.999933887	0.11918861	0.231888486	-0.112699876	0.51130893
ENSG00000165409	*TSHR*	-1.288982725	-0.193176624	-0.438908344	0.24573172	0.999933887	-0.332271976	0.007235577	-0.339507552	0.51130893
ENSG00000081026	*MAGI3*	-0.00185426	-0.180979303	-0.548009787	0.367030484	0.999933887	-0.146226836	-0.512024235	0.365797399	0.511359009
ENSG00000182986	*ZNF320*	1.245571151	-0.077169131	-0.042658877	-0.034510254	0.999933887	-0.075334147	-0.243766325	0.168432178	0.511359009
ENSG00000182670	*TTC3*	4.670689662	-0.056856534	-0.141610674	0.084754141	0.999933887	-0.214683936	-0.047120895	-0.167563041	0.511359009
ENSG00000164077	*MON1A*	0.513078854	-0.104456407	0.049872445	-0.154328852	0.999933887	0.145516197	-0.060651589	0.206167786	0.51146359
ENSG00000166471	*TMEM41B*	4.67055504	0.021333489	0.011292698	0.010040792	0.999933887	0.598500252	0.710509702	-0.11200945	0.51152398
ENSG00000248208	*WDR45P1*	0.314838554	0.151802423	-0.09311396	0.244916383	0.999933887	0.813339969	1.103724607	-0.290384638	0.511590746
ENSG00000167658	*EEF2*	9.240823827	0.045158856	0.022453	0.022705856	0.999933887	0.118957241	0.197761537	-0.078804296	0.512173901
ENSG00000197785	*ATAD3A*	1.671645483	0.042784245	0.069829388	-0.027045142	0.999933887	0.217949246	0.065813213	0.152136034	0.512173901
ENSG00000213553	*RPLP0P6*	-0.250583877	0.056269168	0.098762827	-0.042493659	0.999933887	0.081850502	0.355376887	-0.273526385	0.512279791
ENSG00000100348	*TXN2*	3.64653746	0.01222829	0.129006833	-0.116778543	0.999933887	-0.030146026	-0.145136551	0.114990525	0.512279791
ENSG00000198440	*ZNF583*	-0.850562707	-0.423678455	-0.006886913	-0.416791542	0.999933887	-0.153573881	0.206850135	-0.360424016	0.512436179
ENSG00000188554	*NBR1*	6.603740022	-0.066261429	0.011297348	-0.077558777	0.999933887	0.19816726	0.29875267	-0.100585409	0.512436179
ENSG00000092841	*MYL6*	8.068841889	-0.010801483	-0.01878176	0.007980276	0.999933887	-0.075215985	-0.151708868	0.076492882	0.512436179
ENSG00000167524	*RSKR*	2.265014345	-0.052759972	0.041794266	-0.094554238	0.999933887	0.292366834	0.464492235	-0.172125401	0.512493302
ENSG00000155592	*ZKSCAN2*	0.913452348	0.109716215	-0.036707164	0.146423378	0.999933887	0.08570035	0.310726206	-0.225025856	0.512493458
ENSG00000232956	*SNHG15*	3.561900119	-0.041649702	0.075115192	-0.116764894	0.999933887	0.326821793	0.199355671	0.127466122	0.512591627
ENSG00000258413	*AL158801.2*	0.361149127	0.274488028	0.248704779	0.025783249	0.999933887	0.74389885	0.399267177	0.344631673	0.512937013
ENSG00000180901	*KCTD2*	4.690731421	-0.059820993	-0.084351712	0.02453072	0.999933887	-0.411106269	-0.497615808	0.086509539	0.512937013
ENSG00000180479	*ZNF571*	0.178385312	-0.12101242	-0.255121551	0.134109131	0.999933887	0.046719337	0.345382497	-0.298663161	0.512937013
ENSG00000204301	*NOTCH4*	1.038121046	0.078118402	0.056564992	0.02155341	0.999933887	0.387112001	0.166340783	0.220771218	0.512937013
ENSG00000196670	*ZFP62*	2.407381446	0.023674346	-0.130938762	0.154613107	0.999933887	-0.31834692	-0.435705199	0.117358279	0.512937013

ENSG00000068489	*PRR11*	0.55559687	-0.019489327	-0.114055484	0.094566156	0.999933887	-0.3325105	-0.5876702	0.2551597	0.512937013
ENSG00000164877	*MICALL2*	0.81430297	-0.001846567	-0.127396364	0.125549796	0.999933887	0.321500696	0.555450595	-0.233949898	0.512937013
ENSG00000143321	*HDGF*	6.211327598	0.035512352	-0.013468113	0.048980466	0.999933887	1.17756007	1.341110353	-0.163550282	0.512980934
ENSG00000147454	*SLC25A37*	9.694263637	-0.003255197	0.012127884	-0.015383081	0.999933887	0.162557328	0.103076404	0.059480924	0.512980934
ENSG00000259781	*HMGB1P6*	1.377975969	-0.129194048	0.103435516	-0.232629563	0.999933887	-0.105957213	0.076109644	-0.182066857	0.513210581
ENSG00000120656	*TAF12*	4.252983977	0.005552893	0.066502663	-0.06094977	0.999933887	-0.317887001	-0.420394814	0.102507814	0.513443782
ENSG00000228903	*RASA4CP*	0.633193971	0.064259314	0.099839678	-0.035580364	0.999933887	0.077736454	-0.210551062	0.288287516	0.513478038
ENSG00000106443	*PHF14*	2.018816118	0.038396737	-0.036317162	0.074713899	0.999933887	-0.131534746	0.013935736	-0.145470483	0.513478038
ENSG00000156886	*ITGAD*	2.093256388	0.041706141	-0.074313651	0.116019792	0.999933887	-0.102054881	0.060235194	-0.162290075	0.513478038
ENSG00000143376	*SNX27*	6.825024233	0.013491037	0.00317455	0.010316487	0.999933887	-0.472451668	-0.382666684	-0.089784984	0.513478038
ENSG00000106546	*AHR*	3.290194931	-0.043329799	0.08219843	-0.125528228	0.999933887	0.127900219	0.259955512	-0.132055293	0.513546133
ENSG00000002726	*AOC1*	-1.470738634	0.08725452	0.243031608	-0.155777088	0.999933887	0.68063956	1.233063531	-0.552423971	0.513546133
ENSG00000155744	*FAM126B*	5.888127794	-0.077906905	-0.092200656	0.014293751	0.999933887	-0.191323359	-0.036504784	-0.154818575	0.513598393
ENSG00000188026	*RILPL1*	1.196253866	0.011032826	0.071358116	-0.06032529	0.999933887	0.072675546	0.286330858	-0.213655312	0.513746814
ENSG00000186063	*AIDA*	2.393297691	-0.105780458	0.022719494	-0.128499952	0.999933887	-0.25655031	-0.390278031	0.133727721	0.513785977
ENSG00000115661	*STK16*	3.524729775	0.068801081	-0.01291434	0.081715421	0.999933887	-0.247968077	-0.356886593	0.108918516	0.513785977
ENSG00000233144	*AL512656.1*	-0.812287197	-0.255164246	0.074828158	-0.329992404	0.999933887	1.291992925	1.599134454	-0.307141528	0.513788437
ENSG00000166261	*ZNF202*	1.417234599	-0.017864811	-0.129876258	0.112011447	0.999933887	0.232680397	0.41113909	-0.178458693	0.513788437
ENSG00000148730	*EIF4EBP2*	7.046645585	0.004715846	-0.044509011	0.049224857	0.999933887	-0.268091311	-0.343481695	0.075390384	0.513788437
ENSG00000137106	*GRHPR*	2.891721563	0.082480758	0.014284971	0.068195786	0.999933887	0.047322598	-0.074845349	0.122167947	0.514214182
ENSG00000013503	*POLR3B*	0.736097835	-0.253401733	0.00170655	-0.255108283	0.999933887	-0.082630134	0.100403905	-0.18303404	0.514293914
ENSG00000164924	*YWHAZ*	9.604324744	-0.040227154	-0.052977796	0.012750641	0.999933887	-0.095825214	-0.031740114	-0.0640851	0.514293914
ENSG00000266405	*CBX3P2*	1.167298018	-0.084573054	-0.156442418	0.071869364	0.999933887	-0.632789141	-0.869493585	0.236704445	0.514293914
ENSG00000165046	*LETM2*	2.217406306	0.126975569	-0.068865388	0.195840957	0.999933887	-0.712671612	-0.898490618	0.185819006	0.514406316
ENSG00000140307	*GTF2A2*	2.89288741	0.116651746	0.120870261	-0.004218516	0.999933887	-0.126289852	-0.244515768	0.118225916	0.514877489
ENSG00000163938	*GNL3*	3.405346533	-0.116764225	-0.096596915	-0.02016731	0.999933887	0.150876359	0.266318941	-0.115442582	0.514877489
ENSG00000273004	*AL078644.1*	2.925482074	0.115962397	0.318006362	-0.202043965	0.999933887	0.414417988	0.571304049	-0.156886062	0.514877489
ENSG00000213714	*FAM209B*	1.417339728	-0.15223989	-0.031660042	-0.120579848	0.999933887	0.17340376	-0.033892701	0.207296461	0.514885165
ENSG00000068354	*TBC1D25*	4.633904325	-0.026938771	-0.018015473	-0.008923299	0.999933887	-0.120374831	-0.192832139	0.072457308	0.514885165
ENSG00000272941	*AC083862.1*	3.860077754	-0.023895263	0.023185004	-0.047080267	0.999933887	-0.255111115	-0.139277375	-0.11583374	0.514885165
ENSG00000143178	*TBX19*	0.982533868	0.023735512	0.295110925	-0.271375414	0.999933887	-0.115818601	-0.297367906	0.181549305	0.514885165
ENSG00000137942	*FNBP1L*	-0.196537636	-0.029471601	-0.185090872	0.155619271	0.999933887	-0.090529301	0.130531022	-0.221060323	0.514976828
ENSG00000286634	*AC146507.3*	-0.668637682	-0.103092528	0.126063487	-0.229156015	0.999933887	-0.585386215	-0.134084441	-0.451301774	0.514985321
ENSG00000227376	*FTH1P16*	1.030847865	-0.063020824	0.102069033	-0.165089857	0.999933887	0.382036952	0.573568935	-0.191531983	0.515263131
ENSG00000109920	*FNBP4*	5.740264125	-0.024852214	-0.053007597	0.028155384	0.999933887	-0.180400137	-0.261526302	0.081126165	0.515530462
ENSG00000261338	*AC021016.1*	0.774583027	-0.025303284	-0.034987876	0.009684593	0.999933887	-0.206855856	-0.495415451	0.288559594	0.515530462
ENSG00000157823	*AP3S2*	3.097977985	-0.140309137	-0.11628182	-0.024027318	0.999933887	0.039427075	-0.064737206	0.104164281	0.515762514
ENSG00000274272	*AC069281.2*	2.947991946	0.066227825	0.046464818	0.019763008	0.999933887	-0.157216839	-0.290309018	0.133092179	0.515762514
ENSG00000101452	*DHX35*	1.588771142	-0.021628675	-0.193657716	0.172029041	0.999933887	-0.021784794	-0.187885218	0.166100424	0.515762514
ENSG00000143977	*SNRPG*	3.579379471	0.036155325	-0.018102787	0.054258113	0.999933887	0.210896081	0.116671662	0.094224419	0.51589104
ENSG00000100580	*TMED8*	3.369183181	0.018083776	0.106598719	-0.088514943	0.999933887	-0.076938586	-0.196451106	0.11951252	0.516288959
ENSG00000280091	*AC020895.2*	0.791522309	-0.077304983	0.143487845	-0.220792829	0.999933887	1.677365391	1.933058657	-0.255693266	0.516639006
ENSG00000196407	*THEM5*	-0.594879431	-0.02148429	-0.063706673	0.042222382	0.999933887	0.045433926	0.36065908	-0.315225154	0.516639006
ENSG00000131475	*VPS25*	2.815940499	-0.009500124	0.12451168	-0.134011804	0.999933887	0.016568642	-0.097004436	0.113573077	0.516639006
ENSG00000112308	*C6orf62*	7.729954567	-0.06367134	-0.033996915	-0.029674425	0.999933887	0.296167325	0.362188172	-0.066020847	0.516702268
ENSG00000013392	*RWDD2A*	-0.658574075	0.193383065	0.043846931	0.149536134	0.999933887	0.143213015	0.451428098	-0.308215082	0.516877933
ENSG00000196092	*PAX5*	1.090047118	0.210612678	0.160667388	0.049945289	0.999933887	0.072342075	0.231426653	-0.159084578	0.517090455
ENSG00000214846	*AC114744.1*	1.625590695	-0.156926085	-0.040935736	-0.11599035	0.999933887	-0.684191109	-0.490672494	-0.193518614	0.517090455
ENSG00000167395	*ZNF646*	7.049062162	0.053564729	0.11858511	-0.065020382	0.999933887	-0.071280498	0.003705885	-0.074986383	0.517090455
ENSG00000203446	*SUGCT-AS1*	-0.508760758	0.025755425	-0.377170649	0.402926073	0.999933887	-0.030636247	0.158007886	-0.188644133	0.517090455
ENSG00000167862	*MRPL58*	0.641937738	0.0827475	-0.141404147	0.224151648	0.999933887	0.094108606	0.286260102	-0.192151497	0.517238052
ENSG00000245281	*AC124242.1*	0.016264256	-0.038058444	0.138212455	-0.176270898	0.999933887	0.089761829	0.306464893	-0.216703064	0.517238052
ENSG00000141526	*SLC16A3*	9.20323401	0.077869059	0.079399227	-0.001530168	0.999933887	-0.06605692	0.041213309	-0.107270229	0.517288093
ENSG00000263482	*ANTXRLP1*	-1.030583737	0.00286554	-0.311862981	0.31472852	0.999933887	0.170649495	-0.21960273	0.390252225	0.517316499
ENSG00000232273	*FTH1P1*	-0.693420105	0.149042698	0.419565264	-0.270522566	0.999933887	0.188307904	0.681599139	-0.493291235	0.517411572
ENSG00000115459	*ELMOD3*	3.370380456	0.022636481	0.057183239	-0.034546758	0.999933887	0.167869272	0.268501698	-0.100632426	0.517435774
ENSG00000134755	*DSC2*	2.620189431	-0.04955581	0.017133487	-0.066689296	0.999933887	-0.350603137	-0.569351599	0.218748461	0.517462606
ENSG00000273329	*AC078846.1*	1.381678041	-0.03950827	0.105766976	-0.145275246	0.999933887	-0.391358132	-0.230244773	-0.161113359	0.517462606
ENSG00000131931	*THAP1*	1.926304329	-0.035423568	0.037632525	-0.073056094	0.999933887	-0.610282902	-0.766224259	0.155941356	0.517462606
ENSG00000115446	*UNC50*	4.637629868	-0.01102491	-0.088042612	0.077017702	0.999933887	-0.018630442	0.054479106	-0.073109548	0.517462606
ENSG00000005249	*PRKAR2B*	-0.879616342	-0.008277733	0.035451112	-0.043728845	0.999933887	-0.071636314	-0.422793371	0.351157057	0.51756896
ENSG00000128342	*LIF*	4.154893208	0.301777365	0.280389673	0.021387692	0.999933887	0.87267222	1.0539358	-0.18126358	0.517608679
ENSG00000162654	*GBP4*	5.285727403	-0.001252464	0.027731918	-0.028984382	0.999933887	0.090831341	-0.00746637	0.09829771	0.517633077
ENSG00000126768	*TIMM17B*	4.461292077	0.089452273	0.018730724	0.070721549	0.999933887	0.837956334	0.942806677	-0.104850342	0.518235287
ENSG00000090905	*TNRC6A*	4.497348158	-0.06525951	-0.031487284	-0.033772226	0.999933887	-0.250623093	-0.336490585	0.085867492	0.518235287
ENSG00000186666	*BCDIN3D*	0.082334301	-0.133167569	-0.403745053	0.270577484	0.999933887	-0.136311453	0.104646607	-0.240958061	0.518427236
ENSG00000169570	*DTWD2*	-0.069187584	-0.11339753	-0.132958359	0.019560829	0.999933887	-0.467201241	-0.710269958	0.243068716	0.518579834
ENSG00000242314	*RPL12P32*	0.429641676	-0.153148283	0.006170157	-0.15931844	0.999933887	-0.141856945	0.167705636	-0.309562581	0.518724301
ENSG00000276557	*TRBV18*	-0.02456065	-0.184413369	-0.022350878	-0.162062491	0.999933887	0.014282439	0.290375834	-0.276093395	0.51883721
ENSG00000120708	*TGFBI*	1.285252489	-0.137201194	0.034419538	-0.171620732	0.999933887	-0.578872786	-0.849655431	0.270782645	0.51883721
ENSG00000095739	*BAMBI*	0.430314919	0.082270864	0.204545866	-0.122275003	0.999933887	0.322482259	0.566235875	-0.243753615	0.51883721
ENSG00000119943	*PYROXD2*	2.039845428	0.115144754	-0.112645532	0.227790286	0.999933887	0.139206568	-0.007051277	0.146257845	0.519232177
ENSG00000175548	*ALG10B*	0.59375082	-0.159818361	-0.326030498	0.166212137	0.999933887	-0.321867931	-0.02567019	-0.296197741	0.519232177
ENSG00000158373	*H2BC5*	2.576512681	0.062414259	0.070544338	-0.008130079	0.999933887	-0.30738416	-0.49695046	0.189566299	0.519232177
ENSG00000166734	*GOLM2*	5.553309955	0.011812471	-0.059300644	0.071113115	0.999933887	-0.307599116	-0.39769651	0.090097394	0.519232177
ENSG00000066777	*ARFGEF1*	6.056366135	-0.01151225	-0.009288648	-0.002223601	0.999933887	-0.302642151	-0.164315348	-0.138326803	0.519232177
ENSG00000137869	*CYP19A1*	-2.654635758	0.049625241	-0.6934194	0.743044641	0.999933887	3.796384535	4.392510181	-0.596125646	0.519232177
ENSG00000202533	*Y_RNA*	-0.552149792	0.159058365	-0.134026124	0.293084489	0.999933887	-0.026610515	-0.412527707	0.385917192	0.519451568
ENSG00000261596	*AC005632.3*	2.411404845	0.037061036	-0.014758709	0.051819745	0.999933887	0.313466441	0.166672675	0.146793766	0.519451568
ENSG00000271581	*AL671883.2*	0.081636829	0.077011981	0.160866372	-0.083854391	0.999933887	1.18582985	0.916343326	0.269486524	0.519464916
ENSG00000189337	*KAZN*	1.049398521	0.065184207	0.066555433	-0.001371227	0.999933887	-0.395291971	-0.121177867	-0.274114104	0.519465888
ENSG00000122378	*PRXL2A*	-0.698160463	-0.144739626	-0.225620197	0.080880571	0.999933887	-0.141490558	0.275595091	-0.417085649	0.519610575
ENSG00000284858	*AC091185.2*	-0.444120143	-0.470452736	0.105359036	-0.575811772	0.944094537	-1.17063324	-0.798222122	-0.372411118	0.519636528
ENSG00000171643	*S100Z*	-0.67713666	-0.022890783	0.029935774	-0.052826557	0.999933887	-0.742937469	-1.213015788	0.47007832	0.519872299
ENSG00000169715	*MT1E*	-0.736913977	0.176410452	-0.292896353	0.469306806	0.999933887	0.583095307	0.93082762	-0.347732312	0.519958527
ENSG00000261799	*AC007406.4*	1.404917026	-0.025957506	0.029119091	-0.055076597	0.999933887	-0.261228932	-0.014556213	-0.246672719	0.520037971
ENSG00000143452	*HORMAD1*	-0.002487571	-0.055283926	-0.167038181	0.111754254	0.999933887	-0.212454496	0.221799721	-0.434254217	0.520129029
ENSG00000196418	*ZNF124*	0.967355995	0.126431833	0.032169506	0.094262327	0.999933887	0.532797753	0.756771006	-0.223973253	0.520276062
ENSG00000068784	*SRBD1*	3.050167144	-0.045412025	-0.131747058	0.086335032	0.999933887	-0.234083805	-0.103924491	-0.130159315	0.520276062
ENSG00000070729	*CNGB1*	-0.584960153	-0.135531796	-0.126362464	-0.009169332	0.999933887	0.212774337	0.606762959	-0.393988622	0.520330651
ENSG00000136940	*PDCL*	2.317170979	-0.128630552	0.007868858	-0.13649941	0.999933887	-0.132841917	-0.000436491	-0.132405425	0.520463281
ENSG00000162630	*B3GALT2*	-0.528237173	0.357485255	0.701071685	-0.34358643	0.999933887	-0.252447088	0.284021483	-0.536468571	0.520463281
ENSG00000155966	*AFF2*	0.019430727	-0.078414406	0.078214131	-0.156628537	0.999933887	0.094357599	-0.251742714	0.346100312	0.520463281
ENSG00000224975	*INE1*	2.002412116	-0.028565096	0.158027223	-0.186592319	0.999933887	0.17062321	0.349416412	-0.178793202	0.520463281
ENSG00000138085	*ATRAID*	2.936869153	0.01360891	-0.036293632	0.049902542	0.999933887	-0.046990548	-0.163854795	0.116864248	0.520463281
ENSG00000115289	*PCGF1*	1.960344023	-0.139091882	-0.042360731	-0.096731151	0.999933887	-0.128824856	-0.242269971	0.113445115	0.520718835
ENSG00000005882	*PDK2*	0.743316265	0.072187837	0.002159037	0.0700288	0.999933887	0.168199114	-0.064009209	0.232208323	0.520828673
ENSG00000156738	*MS4A1*	2.256918192	0.014609727	-0.042447899	0.057057626	0.999933887	0.052199551	0.189530679	-0.137331127	0.520828673
ENSG00000226608	*FTLP3*	2.509552225	-0.032326312	-0.092680836	0.060354524	0.999933887	0.327560907	0.190302716	0.137258192	0.520837974
ENSG00000235568	*NFAM1*	8.579367426	-0.001326359	0.001046156	-0.002372515	0.999933887	-0.018466471	-0.11803346	0.099566989	0.520837974
ENSG00000182481	*KPNA2*	2.925843198	-0.030645264	-0.197337126	0.166691862	0.999933887	0.104158673	0.223478824	-0.119320151	0.520935763
ENSG00000286307	*AC017028.1*	-0.051121492	0.220613126	0.477144842	-0.256531716	0.999933887	-0.545804115	-0.206164687	-0.339639428	0.521003563
ENSG00000167112	*TRUB2*	0.920297618	0.136517132	-0.126154659	0.262671791	0.999933887	0.038926815	0.22270836	-0.183781544	0.521003563
ENSG00000165591	*FAAH2*	-0.525435924	-0.184657912	0.127260712	-0.311918623	0.999933887	0.291903863	0.594787925	-0.302884063	0.521003563
ENSG00000154518	*ATP5MC3*	3.324031578	0.051489987	-0.049198002	0.100687989	0.999933887	0.065031319	-0.055879089	0.120910408	0.521416244

ENSG00000226450	*CYP2D8P*	1.576434409	0.06455335	0.211299498	-0.146746148	0.999933887	0.00344349	-0.16233545	0.165778941	0.521419936
ENSG00000116985	*BMP8B*	-0.4210712	0.109781162	-0.003851162	0.113632324	0.999933887	-0.123428597	0.134396398	-0.257824995	0.521419936
ENSG00000267546	*AC015802.4*	-0.426383622	-0.229071231	-0.288471378	0.059400147	0.999933887	0.543147367	0.338717161	0.204430206	0.521771203
ENSG00000204936	*CD177*	2.219089988	0.010012207	-0.108349122	0.118361329	0.999933887	-0.282822945	-0.443681038	0.160858094	0.52219631
ENSG00000101188	*NTSR1*	2.032007362	0.091967566	-0.010853468	0.102821033	0.999933887	0.150252622	0.288088303	-0.13783568	0.522274367
ENSG00000146676	*PURB*	7.301351489	0.109393199	0.126758876	-0.017365677	0.999933887	0.150973686	0.247502553	-0.096528867	0.522294676
ENSG00000100865	*CINP*	3.552501697	-0.027492474	0.037239104	-0.064731578	0.999933887	0.036101978	0.156446661	-0.120344682	0.522294676
ENSG00000197077	*KIAA1671*	2.077985375	-0.053822468	0.08404627	-0.137868739	0.999933887	0.006328612	-0.122325985	0.128654597	0.522328735
ENSG00000257226	*AC079584.2*	-0.856488787	-0.076291296	-0.045877576	-0.03041372	0.999933887	-0.398792055	0.042151439	-0.440943493	0.522503453
ENSG00000275210	*AC245884.11*	0.917741162	0.063973948	0.112926524	-0.048952576	0.999933887	0.306255708	-0.006390301	0.312646008	0.52264761
ENSG00000131558	*EXOC4*	3.090338134	-0.030524837	0.012135934	-0.04266077	0.999933887	-0.002430798	0.119664231	-0.122095029	0.523186559
ENSG00000007255	*TRAPPC6A*	2.113439406	0.116749473	-0.059228458	0.175977931	0.999933887	0.001859759	-0.136323617	0.138183376	0.52338398
ENSG00000224470	*ATXN1L*	6.584887786	-0.001325838	-0.035568192	0.034242355	0.999933887	-0.12826395	-0.042334967	-0.085928983	0.52338398
ENSG00000003756	*RBM5*	7.206799642	-0.054905066	-0.022674585	-0.032230481	0.999933887	-0.027024458	0.037797918	-0.064822376	0.523566513
ENSG00000108179	*PPIF*	11.495385398	0.121742201	0.116142683	0.005599518	0.999933887	0.514381502	0.597109632	-0.08272813	0.523709647
ENSG00000132623	*ANKEF1*	-0.784195126	-0.032740003	-0.016916427	-0.015823577	0.999933887	0.130194636	0.422449602	-0.292254965	0.524149653
ENSG00000286629	*AC005803.1*	1.437364094	-0.083548782	-0.090420201	0.006871419	0.999933887	-0.20799695	0.037140298	-0.245137247	0.524420004
ENSG00000238825	*RNVU1-2*	-1.792845644	0.128970132	0.256220258	-0.127250126	0.999933887	0.20927309	-0.245636618	0.454909709	0.524420004
ENSG00000164597	*COG5*	3.717621483	0.018618007	-0.012290409	0.030908417	0.999933887	0.121188181	0.225089911	-0.10390173	0.524504884
ENSG00000130703	*OSBPL2*	7.507235436	-0.023128541	0.024481331	-0.047609872	0.999933887	-0.582063653	-0.651605787	0.069542134	0.524705969
ENSG00000225092	*AL355336.1*	-0.655554202	-0.058218433	0.273269145	-0.331487577	0.999933887	-0.739324166	-0.434313707	-0.305010459	0.524705969
ENSG00000128973	*CLN6*	2.885115118	-0.010618903	0.052768782	-0.063387685	0.999933887	-0.420146947	-0.572453917	0.152306969	0.524705969
ENSG00000213438	*YBX2P1*	-0.072125165	0.03388275	-0.082579042	0.116461792	0.999933887	-0.284159638	-0.739128383	0.454968745	0.524710976
ENSG00000238164	*TNFRSF14-AS*	5.705140576	0.165409869	0.209109628	-0.043699759	0.999933887	0.831424253	0.949725995	-0.118301742	0.524798409
ENSG00000198205	*ZXDA*	0.690098567	0.124660747	0.147962391	-0.023301644	0.999933887	0.150762782	-0.102678118	0.253440899	0.52484931
ENSG00000123352	*SPATS2*	-0.267198491	0.104964921	0.144396839	-0.039431918	0.999933887	-0.1020803	0.214344683	-0.316424984	0.52484931
ENSG00000079616	*KIF22*	3.651077799	-0.019475571	-0.018807914	-0.000667658	0.999933887	0.135630428	0.033309107	0.102321322	0.52484931
ENSG00000072134	*EPN2*	1.544748954	-0.058537703	0.151378866	-0.209916569	0.999933887	0.017932594	-0.149144258	0.167076853	0.525235031
ENSG00000273129	*PACERR*	0.982637913	0.355383502	0.356030625	-0.000647123	0.999933887	0.708379922	0.380639197	0.327740725	0.525405887
ENSG00000253328	*SUMO2P19*	2.280296175	0.001457641	-0.010890989	0.01234863	0.999933887	-0.48161379	-0.350838583	-0.130775206	0.525493786
ENSG00000068912	*ERLEC1*	2.714257187	-0.056870086	0.095834959	-0.152705045	0.999933887	-0.107646672	0.014600614	-0.122247286	0.525671507
ENSG00000264829	*AC087749.2*	-0.443560561	0.118394343	0.332700841	-0.214306498	0.999933887	0.34680311	0.826668008	-0.479864898	0.525671507
ENSG00000260943	*LINC02555*	0.476038689	0.067037424	-0.21955332	0.286590745	0.999933887	0.194111234	0.476847562	-0.282736329	0.525671507
ENSG00000165181	*SHOC1*	0.790006281	0.078382505	0.266172418	-0.187789913	0.999933887	0.100991291	0.500232472	-0.399241181	0.525671507
ENSG00000167325	*RRM1*	2.137536976	0.035103504	-0.091777422	0.126880926	0.999933887	0.052627153	0.185225795	-0.132598642	0.525671507
ENSG00000102054	*RBBP7*	4.48347253	0.019778299	-0.028548347	0.048326645	0.999933887	0.048073005	0.123867493	-0.075794488	0.525671507
ENSG00000100796	*PPP4R3A*	6.660323345	-0.020559032	-0.017739362	-0.00281967	0.999933887	-0.499433101	-0.577843791	0.07841069	0.525671507
ENSG00000177352	*CCDC71*	4.020096934	-0.132521146	-0.174895937	0.042374792	0.999933887	-0.368011887	-0.260072366	-0.107939521	0.525718495
ENSG00000267904	*AC024075.1*	-0.105656223	-0.177630677	-0.062202159	-0.115428518	0.999933887	-0.429817053	-0.132460136	-0.297356917	0.52588913
ENSG00000119699	*TGFB3*	-0.066775194	0.109020437	-0.054412302	0.163432739	0.999933887	-0.390767219	-0.116929761	-0.273837458	0.52588913
ENSG00000103423	*DNAJA3*	2.785416833	-0.035389599	-0.033984698	-0.001404901	0.999933887	0.137461782	0.016804768	0.120657013	0.52588913
ENSG00000269086	*AC008555.2*	-0.492370636	0.164395314	0.433196319	-0.268801005	0.999933887	-0.027089317	0.240787221	-0.267876538	0.52595401
ENSG00000085563	*ABCB1*	1.949157371	-0.174684385	-0.088726438	-0.085957947	0.999933887	-0.119820402	0.0122417	-0.132062102	0.52641766
ENSG00000196998	*WDR45*	5.469781368	-0.073708535	0.021145832	-0.094854366	0.999933887	-0.237687065	-0.313694428	0.076007363	0.52641766
ENSG00000126773	*PCNX4*	5.579718342	-0.091096486	-0.06665374	-0.024442745	0.999933887	-0.798268674	-0.903668187	0.105399514	0.52641766
ENSG00000105058	*FAM32A*	5.488111565	-0.059400488	-0.044581405	-0.014819083	0.999933887	-0.312473883	-0.397377139	0.084903255	0.52641766
ENSG00000277589	*AC244093.4*	-0.387391306	0.084179312	0.003358811	0.0808205	0.999933887	0.20718077	0.552415232	-0.345234462	0.52641766
ENSG00000166532	*RIMKLB*	4.151523451	-0.049750849	-0.080799787	0.031048938	0.999933887	-0.261420632	-0.153877838	-0.107542794	0.52641766
ENSG00000100897	*DCAF11*	6.883631996	0.010500099	0.005673959	0.00482614	0.999933887	-0.12352022	-0.190943132	0.067422911	0.52641766
ENSG00000127081	*ZNF484*	1.7926128	-0.026718232	-0.318646677	0.291928445	0.999933887	-0.148645962	0.061643759	-0.210289721	0.52641766
ENSG00000237441	*RGL2*	7.373571088	0.015012634	0.082906303	-0.067893669	0.999933887	0.207932656	0.146476636	0.06145602	0.526642699
ENSG00000084754	*HADHA*	5.434318676	-0.007304635	-0.05108829	0.043783655	0.999933887	-0.244824214	-0.315763608	0.070939394	0.526642699
ENSG00000073060	*SCARB1*	2.055533477	0.062914441	0.064610124	-0.001695684	0.999933887	-0.183944315	-0.035144583	-0.148799732	0.526756223
ENSG00000146918	*NCAPG2*	0.990760038	0.033319898	-0.237657953	0.270977851	0.999933887	-0.031745134	0.250456183	-0.282201317	0.526756223
ENSG00000033327	*GAB2*	8.92749996	0.025200145	0.054532895	-0.029332749	0.999933887	-0.140508202	-0.069360009	-0.071148193	0.526830821
ENSG00000150593	*PDCD4*	6.931190992	-0.092834169	-0.064180401	-0.028653767	0.999933887	0.245178012	0.378292012	-0.133114	0.526867567
ENSG00000119446	*RBM18*	3.841801211	-0.026187639	0.010903351	-0.037090989	0.999933887	-0.245466581	-0.151921177	-0.093545404	0.526867567
ENSG00000138802	*SEC24B*	5.63878536	-0.030694225	-0.0434848	0.012790576	0.999933887	-0.393183785	-0.283898389	-0.109285396	0.526960907
ENSG00000124181	*PLCG1*	4.999744451	0.038505685	0.046220906	-0.007715221	0.999933887	0.149054303	0.071522783	0.07753152	0.527056107
ENSG00000105393	*BABAM1*	1.858601874	-0.015073598	0.194578352	-0.20965195	0.999933887	0.153112499	-0.004333178	0.157445677	0.527081647
ENSG00000120903	*CHRNA2*	-0.283870347	0.173827766	-0.210069536	0.383897302	0.999933887	1.44080393	1.774342429	-0.333538499	0.527296524
ENSG00000166575	*TMEM135*	1.04832297	0.003489545	-0.114656443	0.118145989	0.999933887	-0.16013792	0.079973909	-0.240111828	0.527390324
ENSG00000167721	*TSR1*	2.338425007	0.02506831	-0.020853517	0.045921827	0.999933887	0.203560126	0.329070236	-0.125510111	0.527425057
ENSG00000276058	*STMN1P1*	-0.108710172	0.059379152	-0.264903113	0.324282264	0.999933887	0.231102299	0.522131088	-0.291028789	0.527425057
ENSG00000100604	*CHGA*	-0.031030736	0.120749917	0.297114748	-0.176364831	0.999933887	0.14193138	-0.286259784	0.428191164	0.527580499
ENSG00000151704	*KCNJ1*	-1.27882575	0.074896804	-0.07225117	0.147147974	0.999933887	0.134802064	-0.387714185	0.522516249	0.527627732
ENSG00000273320	*AC007032.1*	0.43468853	0.12953755	0.03037223	0.09916532	0.999933887	0.794817271	1.07348525	-0.278667979	0.527641868
ENSG00000128165	*ADM2*	0.287758646	-0.134319104	0.019415243	-0.153734347	0.999933887	-0.323665034	-0.086817255	-0.236847779	0.527641868
ENSG00000121594	*CD80*	-1.378958052	0.224733691	-0.619501282	0.844234974	0.999933887	1.941214945	2.368640659	-0.427425714	0.527641868
ENSG00000167232	*ZNF91*	2.731646223	-0.014974461	0.043135089	-0.058109551	0.999933887	-0.180502086	-0.016321731	-0.164180355	0.527641868
ENSG00000224003	*YES1P1*	-1.155384774	0.017511966	0.545587437	-0.528075472	0.999933887	0.77866637	1.099423403	-0.320757034	0.527641868
ENSG00000205581	*HMGN1*	6.289628042	-0.021495323	-0.012343084	-0.009152239	0.999933887	-0.225454167	-0.290840545	0.065386378	0.527645099
ENSG00000115904	*SOS1*	4.237268658	0.022419293	-0.009063099	0.031482392	0.999933887	-0.404192027	-0.263772189	-0.140419838	0.527686327
ENSG00000163820	*FYCO1*	4.328821542	-0.037691964	0.013504534	-0.051196498	0.999933887	-0.058247771	0.022063936	-0.080311707	0.52781068
ENSG00000196295	*GARS1-DT*	5.870335092	0.006607495	-0.012578803	0.019186298	0.999933887	-0.13142925	-0.016820479	-0.114608771	0.527849588
ENSG00000164338	*UTP15*	2.24756614	-0.076820872	-0.122716128	0.045895256	0.999933887	0.134868338	0.300804177	-0.16593584	0.528220325
ENSG00000114541	*FRMD4B*	3.762064714	0.030647312	-0.042362447	0.073009759	0.999933887	0.162368568	0.349131768	-0.1867632	0.528220325
ENSG00000147121	*KRBOX4*	2.536135653	0.011807795	0.008609401	0.003198394	0.999933887	-0.014741525	0.088932327	-0.103673852	0.528220325
ENSG00000275895	*U2AF1L5*	2.880162162	0.008227293	-0.014885716	0.023113009	0.999933887	0.003088665	0.13716794	-0.134079275	0.528220325
ENSG00000142185	*TRPM2*	6.222760427	0.081667812	0.030700568	0.050967245	0.999933887	0.873522556	0.97922543	-0.105702874	0.5282805
ENSG00000241889	*AC079944.2*	-0.56807151	-0.050254887	-0.315004614	0.264749727	0.999933887	-1.221039972	-1.63031528	0.409275307	0.528509807
ENSG00000182621	*PLCB1*	0.928622904	-0.080961988	-0.158095404	0.077133416	0.999933887	-0.213969232	-0.003524455	-0.210444777	0.528534852
ENSG00000259687	*LINC01220*	0.937812386	0.075417286	0.280869093	-0.205451807	0.999933887	0.183215565	-0.088363832	0.271579397	0.528551628
ENSG00000106537	*TSPAN13*	0.733358736	0.096343935	-0.130781319	0.227125254	0.999933887	-0.050371477	-0.316612169	0.266240692	0.529313547
ENSG00000143641	*GALNT2*	4.187675405	-0.028318264	-0.035687575	0.007369311	0.999933887	-0.292989415	-0.372887656	0.079898241	0.529313547
ENSG00000167685	*ZNF444*	2.453731437	-0.005939242	0.080003839	-0.08594308	0.999933887	0.109715817	-0.032927982	0.142643799	0.529542928
ENSG00000282936	*AC004706.3*	-0.74460185	0.283624842	0.362926719	-0.079301877	0.999933887	-0.515405343	-0.997145663	0.481740321	0.529594073
ENSG00000164082	*GRM2*	-0.280262436	0.105166913	0.206969074	-0.101802162	0.999933887	-0.171678879	0.157608523	-0.329287403	0.529594073
ENSG00000131043	*AAR2*	2.519289956	-0.118193409	-0.164766858	0.046573449	0.999933887	0.086825552	0.210746797	-0.123921245	0.529647362
ENSG00000173226	*IQCB1*	2.56585651	-0.099574527	-0.083853847	-0.015720679	0.999933887	-0.021334574	0.12166698	-0.143001554	0.529647362
ENSG00000155542	*SETD9*	0.695592039	0.113938559	0.086272881	0.027665679	0.999933887	0.055415241	0.248197235	-0.192781994	0.529647362
ENSG00000143797	*MBOAT2*	5.127699501	-0.035894687	-0.045035921	0.009141234	0.999933887	-0.293142746	-0.40272667	0.109583925	0.529770572
ENSG00000020426	*MNAT1*	-0.217018712	-0.083623956	-0.019947935	-0.063676021	0.999933887	-0.056106395	0.239049547	-0.295155942	0.529770572
ENSG00000175390	*EIF3F*	5.278059362	-0.015170129	-0.015243325	7.3195e-05	0.999933887	0.030542851	0.104645864	-0.074103013	0.529770572
ENSG00000106100	*NOD1*	2.501778758	0.030170353	-0.015517221	0.045687574	0.999933887	-0.123333303	-0.285987643	0.16265434	0.529815983
ENSG00000170425	*ADORA2B*	1.949467319	0.020044846	0.048682709	-0.028637862	0.999933887	-0.012379809	-0.166044802	0.153664993	0.529815983
ENSG00000273669	*AC015819.1*	3.065547757	0.013156205	0.039840887	-0.026684681	0.999933887	-0.154352216	-0.030951777	-0.123400439	0.529847546
ENSG00000004777	*ARHGAP33*	1.964520087	0.026724544	0.160019587	-0.133295043	0.999933887	0.053748117	0.194122289	-0.140374172	0.529891715
ENSG00000177119	*ANO6*	3.136358684	0.082704809	-0.077982965	0.160687774	0.999933887	-0.056192013	0.063818577	-0.12001059	0.530037343
ENSG00000029534	*ANK1*	0.725101593	0.167022772	0.089958439	0.077064333	0.999933887	-0.174563715	0.03975099	-0.214314705	0.530304342
ENSG00000226833	*AC092164.1*	0.483488014	0.077365084	0.203718179	-0.126353095	0.999933887	0.00029668	0.226948219	-0.226651539	0.530369919
ENSG00000167088	*SNRPD1*	1.995275691	-0.026254064	0.009674258	-0.035928323	0.999933887	0.106652226	0.240522129	-0.133869904	0.530369919
ENSG00000132326	*PER2*	3.225517594	0.002504984	0.032520646	-0.030015663	0.999933887	0.219922746	0.333766455	-0.113843709	0.530369919
ENSG00000265625	*AC104564.5*	1.816965525	-0.051167519	-0.058767664	0.007600145	0.999933887	-0.288793559	-0.072318954	-0.216474606	0.530667155
ENSG00000271503	*CCL5*	7.24993542	0.015555383	0.006189691	0.009365692	0.999933887	0.112321512	0.206324559	-0.094003047	0.530851357

ENSG00000167130	*DOLPP1*	0.2653315	0.081398485	-0.13692618	0.218324665	0.999933887	0.055635826	-0.160292462	0.215928289	0.530893984
ENSG00000267534	*S1PR2*	1.266126991	-0.060040178	-0.068358788	0.00831861	0.999933887	0.280558353	0.489241546	-0.208683193	0.530893984
ENSG00000168610	*STAT3*	8.605660758	-0.017703215	-0.007342673	-0.010360542	0.999933887	0.130315869	0.196171776	-0.065855906	0.530893984
ENSG00000100360	*IFT27*	2.120285953	0.031509948	0.166197522	-0.134687574	0.999933887	0.00652816	-0.121390132	0.127918292	0.530893984
ENSG00000240970	*RPL23AP64*	0.90431075	-0.036565062	-0.110663383	0.074098322	0.999933887	0.006432349	-0.174527263	0.180959612	0.530893984
ENSG00000206149	*HERC2P9*	0.516302662	0.048483977	0.087572032	-0.039088054	0.999933887	-0.017653825	0.207169442	-0.224823266	0.530893984
ENSG00000166825	*ANPEP*	8.844843386	-0.010805009	-0.031765328	0.020960319	0.999933887	-0.28967422	-0.381946004	0.092271784	0.530893984
ENSG00000130638	*ATXN10*	2.260880034	-0.01332005	-0.024848279	0.011528229	0.999933887	-0.013994129	0.128954455	-0.142948584	0.530893984
ENSG00000119314	*PTBP3*	7.286209082	-0.008522968	-0.017496055	0.008973087	0.999933887	-0.699632984	-0.591857487	-0.107775498	0.530893984
ENSG00000205643	*CDPF1*	-0.259712139	-0.288670671	0.164808386	-0.453479057	0.999933887	0.010305744	-0.206652567	0.216958311	0.531033933
ENSG00000127419	*TMEM175*	4.54628663	0.063993646	0.033258945	0.030734701	0.999933887	0.092132334	0.008907343	0.083224991	0.531033933
ENSG00000164051	*CCDC51*	0.164885861	-0.129947063	-0.069626272	-0.06032079	0.999933887	0.218278164	-0.037850861	0.256129025	0.531033933
ENSG00000006704	*GTF2IRD1*	-1.088717141	-0.386505105	0.18948914	-0.575994245	0.999933887	-0.497344336	-0.216901621	-0.280442714	0.531033933
ENSG00000125482	*TTF1*	3.462057508	-0.06293226	-0.055330167	-0.007602093	0.999933887	-0.308497895	-0.416415199	0.107917304	0.531033933
ENSG00000153140	*CETN3*	-0.893021359	0.141003527	-0.327794279	0.468797806	0.999933887	-0.249443317	0.061053088	-0.310496405	0.531033933
ENSG00000166446	*CDYL2*	2.560803581	-0.020014626	0.040138831	-0.060153457	0.999933887	-0.058328083	0.067460396	-0.12578848	0.531033933
ENSG00000170584	*NUDCD2*	1.968292479	0.010916413	-0.118791638	0.129708051	0.999933887	0.074951701	0.23136963	-0.156417929	0.531033933
ENSG00000282939	*TRBV7-2*	0.732973618	-0.076686627	-0.073128208	-0.003558419	0.999933887	0.261595592	0.450530562	-0.18893497	0.531139215
ENSG00000165434	*PGM2L1*	1.668398019	0.064740572	0.011457771	0.053282802	0.999933887	-0.292022545	-0.083487449	-0.208535096	0.531139215
ENSG00000048342	*CC2D2A*	3.934603125	0.063826187	0.316123731	-0.252297544	0.999933887	-0.088125757	0.129298134	-0.217423891	0.531171111
ENSG00000104946	*TBC1D17*	5.48158862	-0.004474926	0.080040291	-0.084515217	0.999933887	0.058575115	0.122757796	-0.06418268	0.531347555
ENSG00000249042	*AC008771.1*	3.599384004	0.165277373	0.345496753	-0.18021938	0.999933887	0.569901743	0.722926117	-0.153024375	0.531435371
ENSG00000187210	*GCNT1*	1.512289763	-0.094605465	0.026368207	-0.120973672	0.999933887	-0.231179242	-0.097198118	-0.133981125	0.531487711
ENSG00000179104	*TMTC2*	0.564132512	0.055993532	0.127259659	-0.071266127	0.999933887	0.256865198	-0.0420699	0.298935098	0.531670575
ENSG00000109519	*GRPEL1*	3.985915678	0.01439431	0.115181059	-0.100786749	0.999933887	0.121589928	0.235882546	-0.114292618	0.531670575
ENSG00000187624	*C17orf97*	-0.403503818	-0.172765992	-0.238919607	0.066153615	0.999933887	-0.134992761	-0.415893693	0.280900932	0.531754369
ENSG00000238024	*DDX39BP2*	-2.151899277	-0.069208684	0.498892258	-0.568100941	0.999933887	-0.763378992	-0.441557321	-0.321821671	0.532188661
ENSG00000244313	*AC024293.1*	-0.405192716	-0.106062505	0.074566616	-0.180629121	0.999933887	-0.034456217	-0.361214038	0.326757822	0.532358981
ENSG00000210196	*MT-TP*	1.822861493	-0.057939546	-0.076978664	0.019039118	0.999933887	0.112177001	0.314318347	-0.202141346	0.532358981
ENSG00000130024	*PHF10*	3.879728014	-0.024609033	-0.035188955	0.010579922	0.999933887	-0.017647787	0.088185967	-0.105833754	0.532358981
ENSG00000008256	*CYTH3*	3.49812401	-0.008057465	-0.027269247	0.019211782	0.999933887	0.246569269	0.36682961	-0.120260341	0.532358981
ENSG00000119326	*CTNNAL1*	1.788908255	-0.013432183	-0.054622934	0.041190751	0.999933887	-0.055365748	-0.214776552	0.159410804	0.532396934
ENSG00000174775	*HRAS*	1.791852163	0.012214967	0.072915046	-0.06070008	0.999933887	0.047647702	-0.125988867	0.173636569	0.532396934
ENSG00000178338	*ZNF354B*	0.548461419	0.130238231	-0.115770077	0.246008307	0.999933887	-0.088196284	0.117997856	-0.206194139	0.532422682
ENSG00000229097	*CALM2P2*	1.845753031	0.120897868	0.084470829	0.036427038	0.999933887	0.076393138	0.261806118	-0.185412979	0.532602686
ENSG00000163354	*DCST2*	0.640340024	-0.075618971	0.16180858	-0.237427551	0.999933887	0.107604899	-0.144698073	0.252302972	0.532624457
ENSG00000138166	*DUSP5*	5.69178993	0.111809839	0.156212762	-0.044402923	0.999933887	0.81414945	0.941772838	-0.127623389	0.53265516
ENSG00000278997	*AL662907.1*	-0.345183681	0.150014476	0.071818294	0.078196182	0.999933887	0.032610367	-0.280033497	0.312643864	0.533008566
ENSG00000211734	*TRBV5-1*	1.294477314	0.045527798	-0.021436873	0.066964671	0.999933887	0.269994925	0.101205688	0.168789238	0.533008566
ENSG00000239713	*APOBEC3G*	4.339547593	-0.03610546	-0.060817772	0.024712312	0.999933887	-0.019283738	-0.116538795	0.097255058	0.533409358
ENSG00000159086	*PAXBP1*	4.802967737	-0.078017006	-0.161397459	0.083380453	0.999933887	-0.338053717	-0.171990116	-0.166063601	0.533733195
ENSG00000213199	*ASIC3*	0.131949904	-0.033821262	0.468832561	-0.502653823	0.999933887	0.161581807	-0.049436664	0.211018471	0.533733195
ENSG00000138385	*SSB*	3.032476077	-0.083470771	-0.107271481	0.023800709	0.999933887	-0.037724919	0.08725275	-0.124977669	0.533757682
ENSG00000272578	*AP000347.1*	2.456843523	-0.067469739	-0.089361807	0.021892068	0.999933887	-0.055993528	0.088916004	-0.144909533	0.533757682
ENSG00000140104	*CLBA1*	0.548193022	0.11004085	-0.031677396	0.141718245	0.999933887	-0.250962185	-0.47560561	0.224643426	0.533757682
ENSG00000144580	*CNOT9*	3.992700483	-0.023962588	0.042805668	-0.066768256	0.999933887	0.031667396	0.105438153	-0.073770757	0.533757682
ENSG00000110717	*NDUFS8*	3.545439137	-0.007691415	0.042814533	-0.050505948	0.999933887	0.113054953	0.027745441	0.085309512	0.533757682
ENSG00000132640	*BTBD3*	1.176331402	-0.114134577	-0.192714628	0.078580051	0.999933887	-0.917005214	-0.735968916	-0.181036298	0.533776035
ENSG00000128059	*PPAT*	0.530907054	-0.136510414	-0.499871315	0.3633609	0.999933887	-0.088468964	0.136553931	-0.225022894	0.533776035
ENSG00000109466	*KLHL2*	5.559220556	-0.034091939	-0.038224958	0.004133019	0.999933887	-0.427439069	-0.340804538	-0.086634531	0.533776035
ENSG00000234663	*LINC01934*	0.427583759	-0.07578827	-0.17695232	0.101164049	0.999933887	-0.280167776	-0.059427235	-0.220740541	0.533776035
ENSG00000226396	*AL031727.1*	-0.280887528	0.035925194	-0.041391106	0.0773163	0.999933887	0.319077794	0.007846448	0.311231346	0.533776035
ENSG00000107833	*NPM3*	0.963671625	0.010849404	-0.016982233	0.027831637	0.999933887	0.041252923	-0.155502809	0.196755732	0.533776035
ENSG00000144134	*RABL2A*	1.125839343	-0.020760852	-0.241960699	0.221199847	0.999933887	-0.102552351	-0.244942568	0.142390216	0.534101185
ENSG00000256433	*AC005840.2*	-0.516444203	0.312015496	-0.012331583	0.324347079	0.999933887	-0.423892916	-0.864876154	0.440983238	0.534188357
ENSG00000125434	*SLC25A35*	-0.342963537	0.165914013	-0.020926159	0.186840172	0.999933887	0.065114447	-0.237001292	0.302115739	0.534188357
ENSG00000073614	*KDM5A*	7.205639989	-0.071833111	-0.131001544	0.059168433	0.999933887	-0.180492117	-0.103686265	-0.076805852	0.534355158
ENSG00000147044	*CASK*	2.564667449	-0.05526224	-0.084572546	0.029310306	0.999933887	-0.113525612	-0.002200199	-0.111325413	0.534355158
ENSG00000132591	*ERAL1*	1.301072641	-0.064658945	0.094018549	-0.158677494	0.999933887	0.502537412	0.704572371	-0.202034959	0.534355158
ENSG00000182903	*ZNF721*	2.344603138	-0.188797089	-0.172291464	-0.016505625	0.999933887	-0.564845073	-0.348594128	-0.216250945	0.534684219
ENSG00000088256	*GNA11*	0.374167166	-0.031113132	0.089253074	-0.120366206	0.999933887	-0.067939849	0.145766205	-0.213706054	0.534684219
ENSG00000165490	*DDIAS*	0.020042625	-0.029013104	0.088950653	-0.117963756	0.999933887	-0.153186677	-0.487315445	0.334128768	0.534856324
ENSG00000173914	*RBM4B*	1.722696629	0.031633687	-0.052960879	0.084594566	0.999933887	-0.165542507	-0.307399698	0.141857191	0.535307366
ENSG00000085274	*MYNN*	3.679909094	-0.020165227	-0.076879192	0.056713966	0.999933887	0.011242136	0.140344219	-0.129102083	0.53553461
ENSG00000240403	*KIR3DL2*	0.220425383	0.013513557	0.084976325	-0.071462768	0.999933887	0.036253212	0.272993193	-0.236739981	0.535568668
ENSG00000279491	*AP003733.3*	5.060934196	0.285879348	0.199069232	0.086810115	0.999933887	1.094407467	1.22816112	-0.133753653	0.535704774
ENSG00000072071	*ADGRL1*	2.876446002	0.076736846	0.077791534	-0.001054687	0.999933887	0.131339136	0.24236253	-0.111023395	0.535704774
ENSG00000111224	*PARP11*	2.598133796	0.06403045	-0.026265074	0.090295524	0.999933887	-0.291817552	-0.16540186	-0.126415692	0.535704774
ENSG00000137513	*NARS2*	0.423226908	-0.099813818	-0.070735082	-0.029078736	0.999933887	0.014943136	0.230878972	-0.215935837	0.535704774
ENSG00000126005	*MMP24OS*	5.072216257	0.018625863	-0.002829662	0.021455525	0.999933887	-0.2118434	-0.295519628	0.083676228	0.535704774
ENSG00000163565	*IFI16*	7.389644226	-0.118408244	-0.160978551	0.042570307	0.999933887	-0.817587748	-0.929144316	0.111556567	0.535722959
ENSG00000266913	*LINC01841*	-0.052613722	0.045551803	0.220543902	-0.1749921	0.999933887	1.060520024	1.367239445	-0.306719421	0.535799493
ENSG00000118507	*AKAP7*	-0.527380874	-0.067897627	0.130343514	-0.198241141	0.999933887	-0.145205426	0.276675181	-0.421880608	0.535821496
ENSG00000229598	*PRDX3P1*	2.391150233	0.105031531	0.071888328	0.033143202	0.999933887	0.114794836	0.266842575	-0.152047739	0.535854823
ENSG00000203667	*COX20*	0.137768051	-0.067650597	0.158758317	-0.226408914	0.999933887	0.15597497	-0.109565472	0.265540442	0.535897078
ENSG00000180846	*CSNK1G2-AS*	2.394691461	-0.101666392	-0.008342341	-0.093324051	0.999933887	0.050749368	-0.155639338	0.206388706	0.535915083
ENSG00000164902	*PHAX*	2.048460012	-0.063999076	-0.111895371	0.047896296	0.999933887	-0.10477416	0.031009097	-0.135783257	0.535915083
ENSG00000236552	*RPL13AP5*	1.330227331	-0.049211092	-0.137871194	0.088660101	0.999933887	0.221429186	0.012877389	0.208551797	0.535915083
ENSG00000188542	*DUSP28*	2.816701907	-0.134481769	-0.059170809	-0.07531096	0.999933887	-0.4705816	-0.604140233	0.133558633	0.536045778
ENSG00000185085	*INTS5*	2.732180864	0.049739661	0.009357535	0.040382126	0.999933887	0.242022374	0.117763737	0.124258637	0.536045778
ENSG00000123146	*ADGRE5*	9.546762568	0.017721455	-0.010283431	0.028004886	0.999933887	0.213080969	0.283749068	-0.0706681	0.536045778
ENSG00000225808	*DNAJC19P5*	2.232123786	-0.079568172	0.145068028	-0.2246362	0.999933887	0.487505058	0.696488327	-0.208983269	0.536085289
ENSG00000130821	*SLC6A8*	3.148749709	0.121177204	0.107820926	0.013356278	0.999933887	-0.05211642	-0.156627832	0.104511412	0.536706761
ENSG00000164169	*PRMT9*	2.674291257	-0.038440851	-0.107585604	0.069144753	0.999933887	-0.037452097	-0.193316448	0.155864351	0.537026953
ENSG00000164258	*NDUFS4*	0.578814243	0.013349053	-0.099503232	0.112852286	0.999933887	0.001074302	0.20322965	-0.202155348	0.537155668
ENSG00000149554	*CHEK1*	-0.419888025	0.125877784	0.024268885	0.1016089	0.999933887	0.296048758	0.627157684	-0.331108927	0.537185553
ENSG00000196850	*PPTC7*	6.643551414	-0.020200118	0.018860244	-0.039060362	0.999933887	-0.248156412	-0.342139239	0.093982827	0.537588977
ENSG00000116001	*TIA1*	4.081241626	0.013324614	-0.027053658	0.040378272	0.999933887	-0.29908621	-0.158204079	-0.140882131	0.537588977
ENSG00000173141	*MRPL57*	1.889758573	0.124254176	-0.100581997	0.224836173	0.999933887	0.150214198	0.00682574	0.143388457	0.537601961
ENSG00000173451	*THAP2*	1.713551038	0.098738844	0.157869966	-0.059131122	0.999933887	0.595771562	0.84228784	-0.246516278	0.537601961
ENSG00000158473	*CD1D*	-1.493756734	0.037502853	-0.198997694	0.236500547	0.999933887	-0.886856215	-0.276318276	-0.61053794	0.53785075
ENSG00000197563	*PIGN*	1.562338307	-0.028511256	-0.096641158	0.068129903	0.999933887	-0.172944975	-0.365525635	0.19258066	0.538622202
ENSG00000179085	*DPM3*	1.879267666	-0.009413838	-0.024684177	0.015270339	0.999933887	-0.033491768	-0.217109054	0.183617286	0.538892891
ENSG00000122203	*KIAA1191*	3.587650263	0.025391738	0.038738761	-0.013347023	0.999933887	-0.042422089	-0.143013026	0.100590937	0.539048536
ENSG00000214029	*ZNF891*	1.363988543	0.012333443	0.007617729	0.004715714	0.999933887	-0.223875663	0.012628199	-0.236503862	0.539095215
ENSG00000162642	*C1orf52*	4.262633368	0.006648666	-0.020178831	0.026827497	0.999933887	-0.161415028	-0.090226356	-0.071188672	0.539476704
ENSG00000113300	*CNOT6*	3.472592269	-0.119882788	-0.062167173	-0.057715616	0.999933887	-0.20152934	-0.094132848	-0.107396492	0.539492111
ENSG00000171311	*EXOSC1*	2.273888469	-0.032264688	-0.001024631	-0.031240056	0.999933887	-0.032975542	-0.176658621	0.143683079	0.539492111
ENSG00000174255	*ZNF80*	-0.04344884	0.021367428	-0.557490133	0.578857561	0.999933887	-0.077448837	0.204151837	-0.281600674	0.539612046
ENSG00000227124	*ZNF717*	-0.635826939	-0.002012854	0.106390296	-0.10840315	0.999933887	-0.041845743	-0.330160761	0.288315017	0.539627511
ENSG00000205485	*AC004980.1*	2.474163142	0.043682804	0.074346653	-0.03066385	0.999933887	-0.021528885	-0.184069568	0.162540683	0.539657763
ENSG00000066654	*THUMPD1*	4.603188849	0.018050039	-0.085581826	0.103631865	0.999933887	-0.444869629	-0.580998838	0.136129208	0.539657763
ENSG00000128011	*LRFN1*	4.856870022	0.024007847	0.056656473	-0.032648626	0.999933887	-0.787069216	-0.898068001	0.110998784	0.539807923
ENSG00000225690	*TREML5P*	2.669277858	0.116163737	0.247993895	-0.131830157	0.999933887	0.652638328	0.839096406	-0.186458078	0.540298336
ENSG00000231799	*PA2G4P6*	0.109307642	0.135017138	0.251545954	-0.116528816	0.999933887	0.108341783	-0.221222262	0.329564045	0.540298336

ENSG00000141510	*TP53*	3.054390174	-0.047137704	0.125072478	-0.172210182	0.999933887	0.19323591	0.097831953	0.095403957	0.540323893
ENSG00000162783	*IER5*	7.907271112	0.165141407	0.203442677	-0.03830127	0.999933887	2.24803212	2.414453499	-0.166421378	0.540471919
ENSG00000286608	*AC010201.3*	0.106943209	-0.089798326	-0.006351234	-0.083447092	0.999933887	0.186171989	0.515155772	-0.328983783	0.540471919
ENSG00000151414	*NEK7*	5.692236488	-0.053701332	-0.071039844	0.017338512	0.999933887	-0.62702446	-0.487722992	-0.139301468	0.540471919
ENSG00000101203	*COL20A1*	-0.646116046	0.296084602	0.18346942	0.112615182	0.999933887	1.529033025	1.765254048	-0.236221023	0.540699403
ENSG00000121851	*POLR3GL*	2.064554999	-0.016406729	-0.07683524	0.060428512	0.999933887	-0.09847978	-0.278528854	0.180049074	0.540813991
ENSG00000174013	*FBXO45*	1.272020183	0.057295409	0.067710678	-0.010415269	0.999933887	-0.1491836	-0.310059125	0.160875524	0.541150586
ENSG00000026025	*VIM*	8.12292753	0.034022577	0.003419783	0.030602794	0.999933887	0.033415255	0.103819402	-0.070404147	0.541212124
ENSG00000224116	*INHBA-AS1*	-0.611759192	-0.181845136	-0.287324046	0.10547891	0.999933887	0.824076409	1.124652831	-0.300576422	0.541269997
ENSG00000186230	*ZNF749*	-0.233590695	-0.01074155	0.139973919	-0.150715469	0.999933887	0.237797191	-0.012418684	0.250215876	0.541539986
ENSG00000069696	*DRD4*	-0.684047891	0.157738534	-0.275933364	0.433671898	0.999933887	-0.098862153	0.308087167	-0.40694932	0.541645101
ENSG00000237118	*CYP2F2P*	0.812755458	-0.02090563	-0.092100411	0.071194781	0.999933887	0.044271914	0.275393084	-0.23112117	0.541645101
ENSG00000196437	*ZNF569*	0.047573064	0.098300992	-0.046268485	0.144569476	0.999933887	-0.104825587	0.208344473	-0.31317006	0.541680694
ENSG00000196526	*AFAP1*	-0.57916891	-0.234871461	-0.176371961	-0.0584995	0.999933887	-0.075250185	0.265942748	-0.341192933	0.541726018
ENSG00000185495	*AC138393.1*	1.008449191	0.086061092	0.154479038	-0.068417946	0.999933887	0.03189945	0.272534942	-0.240635492	0.541726018
ENSG00000165121	*AL353743.1*	0.547576277	-0.216933441	-0.015666137	-0.201267305	0.999933887	-0.20564462	0.038207752	-0.243852372	0.541778929
ENSG00000119408	*NEK6*	5.874339181	-0.05467874	0.037423968	-0.092102708	0.999933887	-1.023806988	-1.131766521	0.107959533	0.541778929
ENSG00000259079	*AC005476.1*	-0.809202671	0.315421316	0.364293959	-0.048872643	0.999933887	0.922412758	0.403500635	0.518912122	0.541841106
ENSG00000280828	*AC090114.3*	0.645753984	0.215129554	-0.013588508	0.228718062	0.999933887	-0.125484223	-0.328654557	0.203170334	0.541880849
ENSG00000005379	*TSPOAP1*	4.768286396	0.07234599	0.10371541	-0.03136942	0.999933887	0.155073369	0.057245682	0.097827686	0.542053667
ENSG00000013619	*MAMLD1*	1.040982749	0.248368754	0.265693595	-0.017324841	0.999933887	-0.005522222	0.157358344	-0.162880567	0.542053667
ENSG00000173442	*EHBP1L1*	8.268678397	0.092643641	0.10457272	-0.011929079	0.999933887	0.218118489	0.346623295	-0.128504806	0.542053667
ENSG00000019582	*CD74*	8.665207372	0.042609252	0.00133684	0.041272411	0.999933887	0.064301521	0.137912233	-0.073610713	0.542053667
ENSG00000100225	*FBXO7*	5.146802319	-0.033758471	-0.071692405	0.037933934	0.999933887	-0.102817459	-0.167264999	0.06444754	0.542053667
ENSG00000198142	*SOWAHC*	-0.27222738	-0.276285213	0.12008533	-0.396370543	0.999933887	-0.764618443	-0.98492049	0.220302048	0.542053667
ENSG00000163221	*S100A12*	6.417815678	0.026173551	0.036973263	-0.010799712	0.999933887	0.132066102	0.243409006	-0.111342904	0.542053667
ENSG00000085433	*WDR47*	3.840836273	-0.014925618	0.030007146	-0.044932764	0.999933887	-0.138514328	-0.240653198	0.10213887	0.542053667
ENSG00000116127	*ALMS1*	2.638923065	-0.020417677	0.074660603	-0.09507828	0.999933887	-0.164530724	0.054131052	-0.218661776	0.542053667
ENSG00000075539	*FRYL*	5.452431705	0.000875102	-0.116104577	0.116979678	0.999933887	-0.462970315	-0.249130404	-0.213839911	0.542053667
ENSG00000038427	*VCAN*	1.693787103	-0.000240796	-0.036872733	0.036631937	0.999933887	-2.688808249	-3.161240944	0.472432695	0.542053667
ENSG00000134597	*RBMX2*	2.113052495	-0.079114827	-0.055531129	-0.023583698	0.999933887	-0.080000422	-0.230498793	0.150498371	0.542096648
ENSG00000151576	*QTRT2*	3.021999995	-0.020910386	0.035927049	-0.056837435	0.999933887	0.002757992	0.105541802	-0.10278381	0.542186471
ENSG00000268173	*AC007192.1*	0.081540838	0.328191365	0.465941024	-0.137749659	0.999933887	1.386249772	1.721688393	-0.335438621	0.542331724
ENSG00000119778	*ATAD2B*	4.152854199	0.060964782	-0.036747985	0.097712767	0.999933887	-0.182757413	-0.022063378	-0.160694035	0.542331724
ENSG00000177873	*ZNF619*	0.682368356	0.187025448	0.054859943	0.132165505	0.999933887	0.0070228	-0.195230782	0.202253582	0.542339935
ENSG00000279873	*LINC01126*	1.469878928	-0.198778598	0.206803081	-0.405581679	0.944094537	0.255232566	0.40932804	-0.154095474	0.542491694
ENSG00000132825	*PPP1R3D*	4.121401098	-0.064397165	-0.086323796	0.021926631	0.999933887	-0.360150968	-0.477601893	0.117450925	0.543100022
ENSG00000100034	*PPM1F*	7.233340241	-0.018237575	-0.027816567	0.009578992	0.999933887	0.026215605	0.132162141	-0.105946536	0.543115253
ENSG00000205537	*AC121338.1*	0.065000413	0.362993252	-0.014245926	0.377239178	0.999933887	0.373860102	0.087607239	0.286252863	0.543281795
ENSG00000267270	*PARD6G-AS1*	0.988314147	0.076474205	0.189678311	-0.113204106	0.999933887	0.628327397	0.803440581	-0.175113184	0.543281795
ENSG00000168569	*TMEM223*	0.833390843	0.137008188	-0.187706404	0.324714592	0.999933887	-0.058475763	-0.232830132	0.174354369	0.543338737
ENSG00000132341	*RAN*	5.047252237	-0.045102616	-0.073366416	0.0282638	0.999933887	0.368771805	0.458602925	-0.08983112	0.543394766
ENSG00000156162	*DPY19L4*	0.252363398	-0.041010183	-0.234064703	0.193054521	0.999933887	-0.10671343	0.153731856	-0.260445286	0.543404325
ENSG00000024862	*CCDC28A*	4.266219485	0.00342688	-0.036827066	0.040253946	0.999933887	-0.554080909	-0.649762743	0.095681833	0.543404325
ENSG00000106344	*RBM28*	4.503215832	-0.040364408	-0.078900452	0.038536044	0.999933887	-0.239923891	-0.164171084	-0.075752808	0.54373604
ENSG00000184205	*TSPYL2*	6.26076315	0.018967059	0.032813232	-0.013846173	0.999933887	0.163296802	0.239697214	-0.076400411	0.54373604
ENSG00000136521	*NDUFB5*	2.631213226	-0.085143926	-0.031492691	-0.053651235	0.999933887	-0.126233198	-0.011260373	-0.114972826	0.543755553
ENSG00000187607	*ZNF286A*	-1.044222905	-0.446323718	0.229495723	-0.675819441	0.999933887	-0.288959168	0.029351113	-0.318310281	0.543760404
ENSG00000279602	*AC109326.1*	2.121592383	0.260112154	0.215477324	0.044634831	0.999933887	0.519202615	0.754463141	-0.235260526	0.543915769
ENSG00000271303	*SRXN1*	2.946935863	0.262288956	0.034525614	0.227763342	0.999933887	0.243918338	0.431979799	-0.188061462	0.543915769
ENSG00000102805	*CLN5*	2.941537676	-0.081525197	-0.123812127	0.042286931	0.999933887	-0.504305366	-0.367457494	-0.136847872	0.543915769
ENSG00000169599	*NFU1*	1.415734487	-0.060837199	-0.164172551	0.103335352	0.999933887	0.001570491	0.221176578	-0.219606087	0.543915769
ENSG00000076248	*UNG*	1.148934673	0.034898186	-0.027859724	0.06275791	0.999933887	-0.033067173	0.151394357	-0.18446153	0.543915769
ENSG00000147274	*RBMX*	5.714978205	-0.064278587	-0.094770325	0.030491738	0.999933887	0.026171896	0.085750039	-0.059578143	0.544261187
ENSG00000207042	*RNU6-196P*	0.190222964	0.209110812	0.098827146	0.110283666	0.999933887	0.753984756	1.05677798	-0.302793224	0.544261187
ENSG00000175792	*RUVBL1*	2.361398223	-0.042010246	-0.025504191	-0.016506055	0.999933887	0.159472337	0.303185899	-0.143713562	0.544261187
ENSG00000171634	*BPTF*	5.931487063	0.009994805	-0.03910588	0.049100685	0.999933887	-0.468066151	-0.33311412	-0.134952031	0.544261187
ENSG00000167705	*RILP*	4.729897247	0.074774219	0.019465593	0.055308626	0.999933887	0.249462088	0.156036086	0.093426001	0.544321946
ENSG00000182220	*ATP6AP2*	6.293448911	-0.021795087	-0.052079974	0.030284886	0.999933887	0.011589831	0.074031602	-0.062441771	0.544321946
ENSG00000111877	*MCM9*	3.307635811	-0.042625635	-0.045721369	0.003095734	0.999933887	-0.287657631	-0.392654077	0.104996445	0.544443677
ENSG00000105671	*DDX49*	4.32165123	-0.008274322	0.055441946	-0.063716268	0.999933887	-0.08830623	-0.167101306	0.078795076	0.544572183
ENSG00000135838	*NPL*	8.289062843	-0.073437738	-0.050658277	-0.022779461	0.999933887	-0.462921504	-0.392191683	-0.070729821	0.544728465
ENSG00000236345	*SCAT8*	-0.067123585	-0.113429319	-0.164707298	0.051277979	0.999933887	-0.323983222	-0.63654517	0.312561948	0.544762498
ENSG00000245848	*CEBPA*	1.692038345	0.097505983	-0.060517928	0.158023912	0.999933887	-0.204374872	-0.042130305	-0.162244567	0.544766015
ENSG00000004487	*KDM1A*	3.204282428	-0.054818028	-0.030553528	-0.024264499	0.999933887	0.116750414	0.206757343	-0.090006929	0.544826184
ENSG00000178209	*PLEC*	8.249604755	0.056554025	0.073095302	-0.016541277	0.999933887	0.155994673	0.245209341	-0.089214668	0.544977277
ENSG00000136933	*RABEPK*	1.647820918	0.135024848	0.099093731	0.035931118	0.999933887	0.101024045	-0.077217435	0.17824148	0.545295672
ENSG00000286379	*AL591845.1*	0.285468804	0.192843213	0.076602363	0.116240851	0.999933887	1.227081538	1.000936221	0.226145317	0.545313782
ENSG00000168071	*CCDC88B*	8.762006199	0.122964867	0.141860914	-0.018896047	0.999933887	0.328668061	0.46146752	-0.132799459	0.54577016
ENSG00000245680	*ZNF585B*	0.712409395	-0.109319611	-0.092871542	-0.016448069	0.999933887	-0.081696854	0.084314265	-0.16601112	0.54577016
ENSG00000104883	*PEX11G*	1.800097129	0.016149766	0.00729864	0.008851126	0.999933887	0.097671879	0.274184509	-0.17651263	0.54577016
ENSG00000105516	*DBP*	1.651137158	-0.021058633	-0.094398877	0.073340244	0.999933887	-0.307257447	-0.482930313	0.175672867	0.54577016
ENSG00000255092	*AC010768.2*	2.312204289	-0.123410211	-0.093610681	-0.029799531	0.999933887	1.911108564	2.078023706	-0.166915142	0.545828559
ENSG00000145685	*LHFPL2*	3.206666047	-0.110756054	-0.01487772	-0.095878334	0.999933887	0.321341371	0.493769579	-0.172428207	0.545828559
ENSG00000168653	*NDUFS5*	3.223419938	0.051062313	-0.123885346	0.174947659	0.999933887	0.141259174	0.252690867	-0.111431693	0.545828559
ENSG00000135587	*SMPD2*	4.142869453	-0.051261963	0.005828062	-0.057090025	0.999933887	1.031536069	1.134953317	-0.103417247	0.545828559
ENSG00000188566	*NDOR1*	4.263235836	0.024798704	0.068430426	-0.043631722	0.999933887	-0.071450044	-0.159038849	0.087588805	0.545828559
ENSG00000146386	*ABRACL*	3.991811596	-0.045856087	-0.061742405	0.015886318	0.999933887	-0.235852917	-0.346174934	0.110322017	0.545882384
ENSG00000144152	*FBLN7*	0.585594101	0.05340189	0.016619808	0.036782081	0.999933887	0.106234867	-0.100302191	0.206537058	0.545882384
ENSG00000286531	*AC097015.1*	0.745503338	-0.017719882	0.109078755	-0.126798637	0.999933887	0.850912788	1.117890723	-0.266977935	0.545882384
ENSG00000123575	*FAM199X*	4.33692854	-0.009582425	-0.026061367	0.016478942	0.999933887	-0.679833279	-0.79709143	0.117258151	0.545882384
ENSG00000101017	*CD40*	0.539220589	0.217977839	-0.147609909	0.365587749	0.999933887	1.79243038	2.052459997	-0.260029617	0.546037982
ENSG00000135407	*AVIL*	2.401550918	-0.003290126	-0.051091642	0.047801516	0.999933887	-0.156523187	-0.285646534	0.129123346	0.546320641
ENSG00000115020	*PIKFYVE*	5.967390029	-0.081372521	-0.12364609	0.042273569	0.999933887	-0.555377863	-0.402306595	-0.153071269	0.546406826
ENSG00000177700	*POLR2L*	3.509947545	-0.021582965	0.045647144	-0.067230109	0.999933887	-0.166856372	-0.280833872	0.1139775	0.546968998
ENSG00000214026	*MRPL23*	0.758426503	0.114150543	0.059064986	0.055085556	0.999933887	0.020346933	-0.165903419	0.186250352	0.547019002
ENSG00000256705	*AL137779.1*	0.26188245	-0.09154982	0.112334402	-0.203884223	0.999933887	0.078644601	-0.182661205	0.261305806	0.547019002
ENSG00000108819	*PPP1R9B*	7.976307595	0.037042341	0.083225475	-0.046183134	0.999933887	0.06452098	-0.056569199	0.121090179	0.547019002
ENSG00000179912	*R3HDM2*	4.753151112	0.010513217	0.111365112	-0.100851895	0.999933887	0.019559461	-0.049627918	0.069187379	0.547019002
ENSG00000147119	*CHST7*	7.356671599	-0.005339856	-0.049825299	0.044485443	0.999933887	-0.013990726	0.088214863	-0.102205588	0.547019002
ENSG00000138757	*G3BP2*	6.600215397	-0.056070549	-0.082997683	0.026927134	0.999933887	-0.270610041	-0.334039285	0.063429244	0.547059143
ENSG00000169896	*ITGAM*	7.850145009	0.015680726	0.009922941	0.005757785	0.999933887	0.167635645	0.089941716	0.077693929	0.547059143
ENSG00000161040	*FBXL13*	3.502090469	0.002709912	-0.081242442	0.083952354	0.999933887	-0.482971652	-0.375300969	-0.107670682	0.547320026
ENSG00000211791	*TRAV13-2*	-0.49445346	0.004066667	-0.241391	0.245457667	0.999933887	0.017998146	0.352029074	-0.334030928	0.547320026
ENSG00000253829	*AC067817.2*	-0.460815977	0.268037792	-0.086444592	0.354482384	0.999933887	0.249235386	-0.091837541	0.341072927	0.547467681
ENSG00000214851	*LINC00612*	-0.507473646	0.170109724	0.290595559	-0.120485834	0.999933887	-0.078323077	0.30102396	-0.379347037	0.547486437
ENSG00000111445	*RFC5*	1.409294594	-0.035963119	-0.192147092	0.156183973	0.999933887	-0.189576561	-0.339402294	0.149825733	0.547486437
ENSG00000259673	*IQCH-AS1*	-0.71382007	-0.044796055	-0.583510267	0.538714212	0.999933887	-0.32554702	0.007991899	-0.333538919	0.547486437
ENSG00000116771	*AGMAT*	-0.530518472	0.056348854	-0.339131246	0.3954801	0.999933887	0.016494284	0.299056534	-0.28256225	0.547557778
ENSG00000280063	*AC012676.5*	0.673866687	-0.072856829	-0.234022973	0.161166144	0.999933887	-0.2807379	-0.482369944	0.201632044	0.547728654
ENSG00000126016	*AMOT*	-0.690043789	-0.011424923	-0.129440904	0.118015981	0.999933887	0.074431286	-0.255021832	0.329453118	0.547865268
ENSG00000271964	*AC090948.1*	-0.804183973	-0.420451312	0.03313048	-0.453581792	0.999933887	-0.155566515	0.254583357	-0.410149871	0.547946361
ENSG00000171492	*LRRC8D*	2.943481617	-0.055102446	0.057830598	-0.112933044	0.999933887	0.342658832	0.505849844	-0.163191013	0.547946361
ENSG00000267364	*AC022706.1*	-0.692004903	-0.05270851	0.044386675	-0.097095185	0.999933887	-0.141149507	0.204669699	-0.345819207	0.547946361
ENSG00000279568	*AC093525.9*	1.046162873	-0.020036649	-0.07089867	0.050862021	0.999933887	-0.620945974	-0.792669874	0.1717239	0.547946361

ENSG00000130803	*ZNF317*	4.811995925	-0.029744006	-0.029916446	0.000172439	0.999933887	-0.174655371	-0.095264481	-0.07939089	0.548088663
ENSG00000198400	*NTRK1*	-0.558118015	0.146077042	-0.235728731	0.381805773	0.999933887	-0.119003016	-0.467313248	0.348310233	0.548185439
ENSG00000145604	*SKP2*	1.458926358	-0.040655231	-0.001157921	-0.03949731	0.999933887	0.011193575	-0.134079948	0.145273523	0.54845709
ENSG00000187118	*CMC1*	2.770326881	-0.06429184	-0.043208046	-0.021083795	0.999933887	0.029252779	0.137341131	-0.108088352	0.548550934
ENSG00000182318	*ZSCAN22*	0.606362228	0.054392058	-0.010434543	0.064826602	0.999933887	-0.012840543	-0.241171449	0.228330906	0.548550934
ENSG00000175334	*BANF1*	2.62667887	0.002335044	-0.035473796	0.03780884	0.999933887	0.067076911	-0.055948607	0.123025518	0.548665769
ENSG00000261512	*AC092368.3*	1.357784997	0.130726165	0.090997901	0.039728264	0.999933887	-0.767942939	-0.989362476	0.221419536	0.548762017
ENSG00000116750	*UCHL5*	2.354959647	0.005616725	-0.205144985	0.21076171	0.999933887	-0.273784449	-0.088455275	-0.185329174	0.548762017
ENSG00000182774	*RPS17*	1.693037138	-0.024396525	-0.051582341	0.027185816	0.999933887	0.255563894	0.080603033	0.174960861	0.548834499
ENSG00000178057	*NDUFAF3*	2.796321203	-0.093220929	0.032261342	-0.125482272	0.999933887	-0.22449811	-0.327747894	0.103249784	0.54888921
ENSG00000197696	*NMB*	0.621597397	0.171636942	0.141349041	0.030287901	0.999933887	1.260241822	1.463507885	-0.203266063	0.549333055
ENSG00000228079	*AC012368.2*	0.641945106	0.021898311	0.241605308	-0.219706997	0.999933887	0.00922495	0.253814888	-0.244589938	0.549576916
ENSG00000163319	*MRPS18C*	1.980122383	0.162238997	0.158719659	0.003519338	0.999933887	-0.22304661	-0.102276036	-0.120770573	0.549727967
ENSG00000183742	*MACC1*	-0.884604311	-0.191128886	0.623952439	-0.815081325	0.999933887	1.284746847	1.593157894	-0.308411047	0.549727967
ENSG00000072864	*NDE1*	6.288428402	0.019367551	0.024795072	-0.005427521	0.999933887	0.536846968	0.647357171	-0.110510203	0.549727967
ENSG00000236337	*FMR1-IT1*	0.001067633	-0.036138316	0.093078375	-0.129216692	0.999933887	-0.365674024	-0.075300704	-0.290373319	0.549738433
ENSG00000279641	*AC120057.3*	-0.003407353	-0.019951625	-0.216612667	0.196661042	0.999933887	0.406484989	0.185376796	0.221108193	0.549767305
ENSG00000048052	*HDAC9*	1.594252982	0.055460381	-0.04260755	0.098067931	0.999933887	0.093951584	0.246084535	-0.152132951	0.549857332
ENSG00000105383	*CD33*	0.37312468	-0.107166661	0.071040471	-0.178207132	0.999933887	-0.702405346	-1.075335791	0.372930445	0.549987849
ENSG00000253251	*SHLD3*	0.737209784	-0.213879422	-0.019782585	-0.194096836	0.999933887	0.214167328	0.380142146	-0.165974818	0.550200364
ENSG00000203799	*CCDC162P*	0.560766294	-0.209329156	0.187614489	-0.396943645	0.999933887	-0.684826508	-0.477995374	-0.206831134	0.550200364
ENSG00000103227	*LMF1*	1.817335061	0.051922094	0.076524015	-0.024601921	0.999933887	0.087043429	0.231908222	-0.144864792	0.550200364
ENSG00000181026	*AEN*	3.332877827	-0.010727109	-0.006554314	-0.004172795	0.999933887	0.581922272	0.697820399	-0.115898127	0.550200364
ENSG00000132718	*SYT11*	3.437430778	-0.003436222	0.002070219	-0.005506441	0.999933887	0.005398046	-0.093990085	0.099388132	0.550200364
ENSG00000090263	*MRPS33*	0.805658856	-0.005126397	0.221923821	-0.227050219	0.999933887	0.056219583	0.245892031	-0.189672449	0.550200364
ENSG00000100814	*CCNB1IP1*	1.516244477	0.074729534	0.001213044	0.07351649	0.999933887	0.014398821	0.153869197	-0.139470376	0.550331637
ENSG00000173258	*ZNF483*	0.696587916	-0.110151542	-0.048433173	-0.061718369	0.999933887	-0.16916075	0.082714552	-0.251875302	0.550331637
ENSG00000085871	*MGST2*	2.312690504	-0.126410039	0.016760343	-0.143170383	0.999933887	-1.838554526	-2.036661585	0.198107059	0.550374606
ENSG00000125944	*HNRNPR*	4.866964669	-0.033703714	-0.005818519	-0.027885195	0.999933887	-0.03191595	-0.10393354	0.07201759	0.550374606
ENSG00000278376	*AP004609.3*	0.200206377	0.009435907	-0.203569729	0.213005636	0.999933887	0.037566037	-0.156001004	0.19356704	0.550374606
ENSG00000279957	*AC110769.3*	0.576592036	0.464220868	0.699350664	-0.235129796	0.999933887	1.322383807	0.855667952	0.466715855	0.550408644
ENSG00000173992	*CCS*	2.869165631	0.017119068	0.135295723	-0.118176656	0.999933887	-0.048865849	-0.20630713	0.157441281	0.550408644
ENSG00000169919	*GUSB*	4.652915197	-0.0027474	0.010602374	-0.013349774	0.999933887	-0.176556116	-0.255128974	0.078572858	0.550408644
ENSG00000066044	*ELAVL1*	3.481483633	-0.028063274	-0.112303144	0.084239871	0.999933887	-0.03369736	-0.135158418	0.101461058	0.550439571
ENSG00000125962	*ARMCX5*	0.671938415	0.011453732	0.099762919	-0.088309188	0.999933887	-0.109751046	0.072505718	-0.182256764	0.550439571
ENSG00000122591	*FAM126A*	2.559225268	0.004380038	-0.030372431	0.034752469	0.999933887	-0.072726809	0.095902371	-0.16862918	0.550439571
ENSG00000168899	*VAMP5*	2.712897497	0.043320633	0.000280619	0.043040014	0.999933887	0.059757048	-0.089961839	0.149718887	0.550444924
ENSG00000159884	*CCDC107*	-0.016548178	-0.051605825	-0.269090526	0.217484701	0.999933887	0.329434095	0.064469469	0.264964626	0.550444924
ENSG00000168404	*MLKL*	5.4089848	0.001393006	0.012781036	-0.01138803	0.999933887	0.05655747	-0.029476772	0.086034241	0.550444924
ENSG00000180336	*MEIOC*	-0.073051033	-0.172188244	0.056305236	-0.22849348	0.999933887	-0.434231699	-0.15610045	-0.278131249	0.550570499
ENSG00000234383	*CTBP2P8*	-0.880367407	-0.00089941	0.00909455	-0.00999396	0.999933887	-0.336391963	-0.724498957	0.388106995	0.550752691
ENSG00000143412	*ANXA9*	2.12882527	-0.094702307	0.045629101	-0.140331408	0.999933887	-0.635746188	-0.815655531	0.179909343	0.550936709
ENSG00000108688	*CCL7*	1.165890026	-0.049843003	-0.593314569	0.543471566	0.999933887	-1.710813698	-1.927837059	0.217023361	0.550936709
ENSG00000127528	*KLF2*	8.610655954	0.206388959	0.147586671	0.058802288	0.999933887	0.045840884	-0.055523998	0.101364882	0.551062964
ENSG00000179010	*MRFAP1*	6.634400531	-0.054964773	-0.111608332	0.056643558	0.999933887	-0.220797851	-0.149334325	-0.071463525	0.551062964
ENSG00000179152	*TCAIM*	1.3272632	0.082832888	0.10732076	-0.024487872	0.999933887	-0.1477452	0.099369329	-0.247114529	0.551062964
ENSG00000211899	*IGHM*	3.931355084	0.047612318	0.096164343	-0.048552025	0.999933887	0.098061629	-0.022212379	0.120274008	0.551062964
ENSG00000184110	*EIF3C*	-0.258930118	-0.086009596	-0.159188339	0.073178743	0.999933887	0.339783355	0.577287441	-0.237504086	0.551062964
ENSG00000141385	*AFG3L2*	2.998511863	-0.014966569	-0.132693881	0.117727311	0.999933887	-0.193090547	-0.081486104	-0.111604443	0.551062964
ENSG00000197841	*ZNF181*	0.458737868	0.028500017	-0.135937163	0.16443718	0.999933887	-0.064552218	0.129534654	-0.194086872	0.551062964
ENSG00000224852	*MTND5P24*	-0.848362444	0.14344575	-0.02738297	0.170828721	0.999933887	0.582379655	0.091669537	0.490710119	0.551144067
ENSG00000183397	*C19orf71*	3.009391955	0.208954328	0.179119579	0.029834749	0.999933887	0.466238173	0.642957035	-0.176718862	0.551959014
ENSG00000101109	*STK4*	9.043917462	-0.095005542	-0.013376251	-0.081629291	0.999933887	-0.358811598	-0.430809391	0.071997793	0.552217735
ENSG00000143374	*TARS2*	2.102360134	-0.063174269	-0.050098705	-0.013075564	0.999933887	0.097228783	-0.040544817	0.1377736	0.552421457
ENSG00000258928	*AL358333.2*	1.027476779	0.085247458	0.275670823	-0.190423366	0.999933887	2.398587058	2.748086012	-0.349498954	0.552421457
ENSG00000089876	*DHX32*	-0.412694554	-0.101541467	-0.048728948	-0.052812519	0.999933887	-0.145469792	0.064627958	-0.21009775	0.552421457
ENSG00000100138	*SNU13*	4.798014613	0.011440943	-0.048616429	0.060057371	0.999933887	-0.030136019	-0.111574212	0.081438193	0.552421457
ENSG00000188811	*NHLRC3*	1.827701535	-0.0080576	-0.01054958	0.002491981	0.999933887	-0.158142618	0.02059114	-0.178733758	0.552421457
ENSG00000170234	*PWWP2A*	3.821978021	0.003154575	0.043084456	-0.03992988	0.999933887	0.646030174	0.761374849	-0.115344675	0.552421457
ENSG00000167105	*TMEM92*	1.073125459	-0.163674499	-0.154361621	-0.009312877	0.999933887	-0.403519424	-0.678041088	0.274521664	0.552601863
ENSG00000147437	*GNRH1*	1.192125807	-0.132592585	0.137376236	-0.269968822	0.999933887	-0.620509379	-0.460707699	-0.159801681	0.552689624
ENSG00000101945	*SUV39H1*	0.880607496	-0.063882845	-0.210143201	0.146260356	0.999933887	0.019669803	-0.189201311	0.208871114	0.552689624
ENSG00000115350	*POLE4*	2.20075286	-0.007140991	-0.041270819	0.034129827	0.999933887	0.014693687	-0.128527279	0.143220966	0.552689624
ENSG00000138411	*HECW2*	1.890957976	0.003711738	-0.066848389	0.070560127	0.999933887	-0.436143392	-0.651180161	0.215036769	0.552689624
ENSG00000136104	*RNASEH2B*	3.627838867	-0.054267218	-0.110251613	0.055984395	0.999933887	-0.074980307	-0.164953394	0.089973087	0.552890884
ENSG00000027001	*MIPEP*	-0.571017394	0.068981119	-0.030070474	0.099051593	0.999933887	-0.280011629	-0.000114468	-0.279897161	0.55350745
ENSG00000196792	*STRN3*	3.651360401	0.03218137	0.008750238	0.023431131	0.999933887	-0.216731301	-0.102835657	-0.113895644	0.553544691
ENSG00000130255	*RPL36*	6.292475359	0.056428511	-0.028263066	0.084691577	0.999933887	0.103227778	0.18772637	-0.084498592	0.553873817
ENSG00000047662	*FAM184B*	1.167635321	0.117330202	0.11438308	0.002947122	0.999933887	0.133182097	0.333680623	-0.200498526	0.553918887
ENSG00000141040	*ZNF287*	-0.461815712	0.148207335	-0.008149064	0.156356399	0.999933887	0.170614839	-0.054672298	0.225287137	0.553938233
ENSG00000125875	*TBC1D20*	5.203629047	-0.019448613	-0.021751766	0.002303153	0.999933887	-0.199529398	-0.275802855	0.076273457	0.553938233
ENSG00000247679	*AC139795.2*	0.550771274	-0.035207516	0.123465133	-0.158672649	0.999933887	0.128109128	0.335810075	-0.207700946	0.553938233
ENSG00000204920	*ZNF155*	1.291156242	-0.139602141	0.123290403	-0.262892544	0.999933887	-0.29229379	-0.134564781	-0.15772901	0.554038187
ENSG00000110921	*MVK*	1.886712409	0.049517818	-0.087217605	0.136735423	0.999933887	0.518523931	0.687301402	-0.16877747	0.554038187
ENSG00000132849	*PATJ*	2.673548804	-0.048703705	-0.036924816	-0.011778889	0.999933887	-0.003255177	0.12265642	-0.125911596	0.554257068
ENSG00000223799	*IL10RB-DT*	2.677024702	0.074232611	-0.015146141	0.089378752	0.999933887	1.210192067	1.054840062	0.155352006	0.554311895
ENSG00000173110	*HSPA6*	6.353156523	0.049090843	0.043808052	0.005282791	0.999933887	0.683010358	0.576686551	0.106323807	0.554311895
ENSG00000034677	*RNF19A*	6.082764316	-0.025479051	-0.021044122	-0.004434929	0.999933887	-0.300478066	-0.204615114	-0.095862952	0.554311895
ENSG00000105327	*BBC3*	5.485617014	-0.018519678	0.062004671	-0.08052435	0.999933887	-0.970113763	-1.122746119	0.152632357	0.554311895
ENSG00000253269	*AC008680.1*	-0.161419635	0.171229146	0.226115303	-0.054886157	0.999933887	0.104637911	0.398972599	-0.294334688	0.554413726
ENSG00000100139	*MICALL1*	3.773265014	0.035646375	-0.020569484	0.056215859	0.999933887	0.038864951	-0.071774136	0.110639087	0.55474832
ENSG00000227232	*WASH7P*	0.822239974	0.073769026	0.001177949	0.072591077	0.999933887	-0.131976613	-0.334243021	0.202266407	0.55474832
ENSG00000161526	*SAP30BP*	7.00530837	-0.02804413	0.002762328	-0.030806457	0.999933887	0.358506007	0.451500462	-0.092994455	0.55474832
ENSG00000268521	*VN1R83P*	-0.570663874	-0.037041063	-0.525227812	0.488186748	0.999933887	-0.354971291	0.000402265	-0.355373556	0.55474832
ENSG00000125520	*SLC2A4RG*	4.257880684	0.109957598	0.050358144	0.059599455	0.999933887	0.136898727	0.03172016	0.105178567	0.554799118
ENSG00000136522	*MRPL47*	2.361083506	-0.110934447	-0.067408218	-0.043526229	0.999933887	0.127473034	0.255394342	-0.127921309	0.554799118
ENSG00000179889	*PDXDC1*	4.731513202	-0.0477826	-0.01953769	-0.02824491	0.999933887	0.054066989	0.114280266	-0.060213278	0.554799118
ENSG00000144161	*ZC3H8*	1.704347069	-0.04013328	-0.102863881	0.062730602	0.999933887	-0.101276499	0.048258778	-0.149535276	0.554799118
ENSG00000228013	*IL6R-AS1*	1.61147445	0.193800011	0.109636001	0.08416401	0.999933887	1.222897226	1.454068907	-0.231171681	0.554836677
ENSG00000110108	*TMEM109*	3.616118179	-0.029578477	-0.035989031	0.006410553	0.999933887	0.154173608	0.054140014	0.100033594	0.555247782
ENSG00000138758	*SEPTIN11*	3.035697715	-0.052697973	0.032372852	-0.085070825	0.999933887	0.040718327	0.161293705	-0.120575378	0.555276441
ENSG00000174871	*CNIH2*	0.696646442	0.088573539	-0.0536513	0.142224839	0.999933887	-0.026608237	0.242354093	-0.26896233	0.555276441
ENSG00000030582	*GRN*	9.14805352	-0.024575855	-0.016003435	-0.00857242	0.999933887	-0.275899319	-0.200959606	-0.074939712	0.555276441
ENSG00000014914	*MTMR11*	-0.510298127	0.019124143	-0.124473943	0.143598086	0.999933887	0.147172846	0.415741984	-0.268569138	0.555276441
ENSG00000240869	*RN7SL128P*	-0.218518979	0.080077439	0.2679498	-0.187872361	0.999933887	0.574223427	0.319388828	0.254834599	0.555357078
ENSG00000240344	*PPIL3*	0.981956615	-0.173115003	0.161267966	-0.334382969	0.999933887	0.1125251	-0.069267539	0.181792639	0.555693404
ENSG00000035115	*SH3YL1*	2.264465072	-0.094443148	-0.008688899	-0.085754249	0.999933887	0.020243116	0.143611084	-0.123367967	0.555693404
ENSG00000114739	*ACVR2B*	0.539244595	-0.064022124	-0.110346195	0.046324072	0.999933887	-0.010934604	0.141893724	-0.152828329	0.555693404
ENSG00000112304	*ACOT13*	3.931502638	-0.024667996	-0.019929888	-0.004738108	0.999933887	-0.192734538	-0.101857193	-0.090877345	0.555693404
ENSG00000065802	*ASB1*	2.845696126	-0.016763603	0.02198041	-0.038744013	0.999933887	0.025742642	0.1247424	-0.098999759	0.555693404
ENSG00000205710	*C17orf107*	2.983573055	0.224636694	0.177817802	0.046818892	0.999933887	0.864281913	1.073098585	-0.208816672	0.55578732
ENSG00000126775	*ATG14*	3.714010499	-0.091158575	0.03219195	-0.123350525	0.999933887	-0.157077823	-0.238933769	0.081855945	0.55578732
ENSG00000196312	*MFSD14C*	3.23661098	0.005664013	0.070380026	-0.064716012	0.999933887	-0.019274062	-0.140227293	0.120953231	0.55578732
ENSG00000157741	*UBN2*	3.670815405	-0.006671274	-0.055658361	0.048987087	0.999933887	-0.264755009	-0.135035634	-0.129719375	0.555856619
ENSG00000166313	*APBB1*	3.051673425	0.030650228	0.074835009	-0.044184781	0.999933887	0.158223579	0.250664538	-0.092440959	0.555889505

ENSG00000245750	*DRAIC*	2.428844363	-0.004787893	0.080590039	-0.085377932	0.999933887	0.31943968	0.471187679	-0.151747999	0.555889505
ENSG00000176714	*CCDC121*	-0.789481869	-0.104701235	0.012006416	-0.116707651	0.999933887	-0.536004418	-0.989023856	0.453019437	0.556045274
ENSG00000065243	*PKN2*	5.862065642	-0.041786233	-0.157595634	0.1158094	0.999933887	-0.82920777	-1.040962995	0.211755225	0.556131493
ENSG00000140968	*IRF8*	2.802145049	-0.004474411	0.08990174	-0.094376151	0.999933887	-0.006266283	-0.110674534	0.104408251	0.556131493
ENSG00000116350	*SRSF4*	6.352661212	-0.02079234	-0.034998026	0.014205686	0.999933887	-0.309711523	-0.372929614	0.063218091	0.556215758
ENSG00000278869	*BX539320.1*	2.309922343	0.179993278	0.156548124	0.023445153	0.999933887	1.432537111	1.569589688	-0.137052576	0.55628064
ENSG00000135218	*CD36*	1.294920756	0.171103475	-0.096001602	0.267105077	0.999933887	-1.690425609	-2.044614762	0.354189153	0.556600012
ENSG00000184445	*KNTC1*	2.509557452	0.05579458	-0.023927219	0.079721799	0.999933887	-0.214911858	-0.042232984	-0.172678874	0.556842885
ENSG00000114982	*KANSL3*	5.02380933	-0.050842437	-0.022807951	-0.028034486	0.999933887	-0.069285159	-0.133323546	0.064038387	0.556968756
ENSG00000165795	*NDRG2*	0.490832754	-0.099237873	-0.084985738	-0.014252135	0.999933887	-0.049415471	0.176991681	-0.226407152	0.557544056
ENSG00000058804	*NDC1*	0.950553552	-0.265480064	0.239179381	-0.504659445	0.944094537	0.112262571	0.306166344	-0.193903774	0.557667305
ENSG00000188529	*SRSF10*	5.732669717	-0.053164387	-0.08766491	0.034500523	0.999933887	-0.052423072	0.032890247	-0.08531332	0.557667305
ENSG00000198838	*RYR3*	-0.747492495	-0.162341056	-0.083604729	-0.078736327	0.999933887	-0.526927308	-0.182596685	-0.344330623	0.557667305
ENSG00000023572	*GLRX2*	1.850950629	0.057916882	0.143921808	-0.086004927	0.999933887	-0.284931028	-0.123028681	-0.161902348	0.557667305
ENSG00000071073	*MGAT4A*	5.148248286	0.041046242	-0.052621664	0.093667906	0.999933887	-0.62502322	-0.760119151	0.135095931	0.557667305
ENSG00000068654	*POLR1A*	2.999355547	0.011400543	0.010059461	0.001341081	0.999933887	0.068242384	-0.033104651	0.101347035	0.557667305
ENSG00000198711	*SSBP3-AS1*	-0.792182168	-0.000833102	0.315730798	-0.3165639	0.999933887	0.157351218	0.441720301	-0.284369083	0.557667305
ENSG00000092108	*SCFD1*	3.430419596	3.217e-05	-0.044436069	0.04446824	0.999933887	-0.317747973	-0.158052361	-0.159695612	0.557729014
ENSG00000278879	*AP000560.1*	-0.602549006	0.114869344	-0.040358653	0.155227998	0.999933887	-0.830393784	-0.430931069	-0.399462715	0.557778362
ENSG00000167110	*GOLGA2*	6.02091432	-0.00045669	0.079737614	-0.080194303	0.999933887	-0.322649496	-0.398506798	0.075857302	0.55810151
ENSG00000162511	*LAPTM5*	11.121983734	0.054675589	0.006405407	0.048270182	0.999933887	-0.019334076	0.046201286	-0.065535362	0.558505899
ENSG00000213015	*ZNF580*	3.561976913	0.04439293	0.072040791	-0.027647861	0.999933887	-0.149180419	-0.237997281	0.088816862	0.558505899
ENSG00000113734	*BNIP1*	0.769057077	-0.067854759	-0.048199423	-0.019655336	0.999933887	0.191497758	0.397467645	-0.205969887	0.558505899
ENSG00000214837	*LINC01347*	0.158398658	0.06669006	-0.17177509	0.23846515	0.999933887	0.054193356	0.278617933	-0.224424577	0.558505899
ENSG00000162664	*ZNF326*	3.480182584	-0.02665662	-0.08314959	0.05649297	0.999933887	-0.213912311	-0.028645893	-0.185266417	0.558505899
ENSG00000116754	*SRSF11*	6.492198778	-0.036678586	-0.098537448	0.061858861	0.999933887	-0.274345359	-0.126812436	-0.147532923	0.558801535
ENSG00000124491	*F13A1*	1.003918204	-0.151720319	-0.085689096	-0.066031222	0.999933887	-0.243254804	-0.023909163	-0.219345641	0.558905573
ENSG00000205726	*ITSN1*	0.34173651	-0.011121833	-0.067228856	0.056107023	0.999933887	-0.216472177	-0.011044784	-0.205427393	0.559105855
ENSG00000101152	*DNAJC5*	7.95568386	-0.103382201	-0.128940156	0.025557955	0.999933887	-0.089207092	-0.011932898	-0.077274195	0.559179847
ENSG00000230487	*PSMG3-AS1*	0.269669489	-0.153161488	-0.165419057	0.012257569	0.999933887	-0.020851488	-0.19276055	0.171909062	0.559179847
ENSG00000287680	*AC093330.3*	3.760459598	0.10707608	0.113827525	-0.006751445	0.999933887	-0.381540719	-0.202890093	-0.178650627	0.559179847
ENSG00000111832	*RWDD1*	2.916249191	-0.033188031	0.002129626	-0.035317656	0.999933887	0.046756232	0.152551779	-0.105795547	0.559179847
ENSG00000215158	*AC138409.2*	0.096483482	-0.003185633	-0.028749945	0.025564312	0.999933887	-0.347019206	-0.020794764	-0.326224442	0.559198761
ENSG00000143702	*CEP170*	4.889921927	0.042321134	0.01970286	0.022618274	0.999933887	-1.156579617	-0.97410611	-0.182473508	0.559256992
ENSG00000238025	*ZDHHC4P1*	-0.869219631	-0.061595103	0.241350714	-0.302945816	0.999933887	-0.215401473	0.221062304	-0.436463778	0.559589295
ENSG00000205531	*NAP1L4*	5.464116904	-0.009531436	-0.069943141	0.060411704	0.999933887	0.064918893	0.011601319	0.053317574	0.559591276
ENSG00000142396	*ERVK3-1*	3.28824721	0.108774603	0.077125988	0.031648615	0.999933887	0.003473881	-0.111576655	0.115050537	0.559895077
ENSG00000159352	*PSMD4*	5.985784817	-0.031057358	-0.070371443	0.039314085	0.999933887	0.382992872	0.459806453	-0.076813581	0.559895077
ENSG00000108654	*DDX5*	10.241222829	0.006995686	-0.015116012	0.022111698	0.999933887	0.265859254	0.336796282	-0.070937028	0.559966255
ENSG00000146842	*TMEM209*	1.06037889	-0.078277112	0.078651368	-0.15692848	0.999933887	-0.084883498	0.086871726	-0.171755224	0.56011233
ENSG00000115946	*PNO1*	1.623566699	-0.074794719	0.116376225	-0.191170944	0.999933887	0.291516105	0.448527937	-0.157011833	0.560149373
ENSG00000204954	*C12orf73*	-0.088082094	-0.125230717	0.006973612	-0.132204329	0.999933887	0.125661662	-0.143483298	0.26914496	0.560149373
ENSG00000134013	*LOXL2*	0.264916915	0.027544391	-0.138987381	0.166531772	0.999933887	0.194334253	-0.057974241	0.252308493	0.560149373
ENSG00000245025	*AC107959.1*	0.100768728	-0.142046375	-0.065842165	-0.07620421	0.999933887	0.106726033	-0.147403353	0.254129387	0.560374615
ENSG00000137331	*IER3*	7.493463611	0.773085391	0.913163131	-0.14007774	0.999933887	2.391680628	2.613741327	-0.222060698	0.560429408
ENSG00000092096	*SLC22A17*	-1.129891652	0.095135722	-0.025547645	0.120683367	0.999933887	-0.005341649	0.315232436	-0.320574084	0.560429408
ENSG00000245060	*LINC00847*	3.974991289	-0.02681225	0.013666495	-0.040478745	0.999933887	-0.027365698	0.055381836	-0.082747534	0.560429408
ENSG00000151651	*ADAM8*	10.414785529	0.087925181	0.069652016	0.018273165	0.999933887	0.524343495	0.591971293	-0.067627799	0.560537649
ENSG00000106617	*PRKAG2*	4.806693018	0.035006693	0.011876542	0.023130151	0.999933887	0.018372081	0.101485277	-0.083113196	0.560537649
ENSG00000184786	*TCTE3*	0.427239996	-0.02179815	0.263501672	-0.285299822	0.999933887	-0.106713795	0.08244792	-0.189161715	0.560537649
ENSG00000126581	*BECN1*	5.414187203	-0.006599936	0.020971823	-0.027571759	0.999933887	0.055481676	0.12139826	-0.065916584	0.560537649
ENSG00000013563	*DNASE1L1*	3.61324005	0.005799914	0.081829809	-0.076029895	0.999933887	-0.169776277	-0.068975968	-0.100800309	0.560537649
ENSG00000115009	*CCL20*	2.497830016	0.49179783	0.744759038	-0.252961208	0.999933887	4.006886721	4.548872928	-0.541986207	0.560983125
ENSG00000188493	*C19orf54*	2.676965487	0.069886843	-0.088841743	0.158728586	0.999933887	0.317362635	0.425168343	-0.107805708	0.560983125
ENSG00000169385	*RNASE2*	2.478254897	0.069556699	-0.006768405	0.076325104	0.999933887	-0.028063881	0.145032632	-0.173096513	0.560983125
ENSG00000100211	*CBY1*	-0.403803878	0.053383474	0.211727187	-0.158343713	0.999933887	-0.215700061	0.063346341	-0.279046402	0.560983125
ENSG00000236254	*MTND4P14*	-1.454007878	-0.10879382	0.029724192	-0.138518011	0.999933887	0.136508975	0.784816787	-0.648307812	0.561014404
ENSG00000162892	*IL24*	1.011010808	0.149527529	-0.074854717	0.224382245	0.999933887	-0.249032475	-0.102931932	-0.146100543	0.56108307
ENSG00000176928	*GCNT4*	1.24311599	-0.086127512	-0.105744924	0.019617411	0.999933887	-0.120456439	0.064046209	-0.184502648	0.56108307
ENSG00000286656	*AC008875.2*	1.652014745	0.046398032	0.08356288	-0.037164849	0.999933887	-0.02563128	-0.231642836	0.206011556	0.56108307
ENSG00000204070	*SYS1*	5.120783734	-0.047037644	0.000819954	-0.047857598	0.999933887	0.404289566	0.47868	-0.074390434	0.561430113
ENSG00000072609	*CHFR*	6.890191721	0.010623558	0.004526763	0.006096795	0.999933887	-0.169833855	-0.115386711	-0.054447145	0.561430113
ENSG00000197046	*SIGLEC15*	-0.09450986	-0.06501445	0.116125117	-0.181139567	0.999933887	-0.414722674	-0.688706596	0.273983923	0.561656425
ENSG00000260898	*ADPGK-AS1*	1.744660548	0.137241875	0.036884588	0.100357287	0.999933887	0.817977779	1.091469275	-0.273491497	0.561798572
ENSG00000115844	*DLX2*	-0.672825282	0.570187503	-0.28019723	0.850384733	0.144167088	1.400216405	1.162608319	0.237608086	0.562196353
ENSG00000099290	*WASHC2A*	6.371186319	-0.026524176	-0.058708276	0.0321841	0.999933887	0.05660835	0.126804816	-0.070196466	0.562196353
ENSG00000184436	*THAP7*	3.883466006	0.013951083	-0.119915044	0.133866127	0.999933887	0.256437418	0.135334235	0.121103183	0.562196353
ENSG00000169641	*LUZP1*	3.651598424	-0.026333846	-0.027179633	0.000845787	0.999933887	0.010775754	0.12087173	-0.110095976	0.56232508
ENSG00000177432	*NAP1L5*	1.368789095	0.078052435	0.150366888	-0.072314453	0.999933887	0.097819098	-0.094851543	0.192670642	0.562549552
ENSG00000108039	*XPNPEP1*	3.593888584	-0.044655082	0.084881085	-0.129536167	0.999933887	0.196087621	0.293526649	-0.097439028	0.562549552
ENSG00000160058	*BSDC1*	5.918952349	-0.025432691	0.020355391	-0.045788082	0.999933887	-0.122472463	-0.066337863	-0.0561346	0.562549552
ENSG00000100600	*LGMN*	0.686015018	-0.018780935	-0.067534202	0.048753267	0.999933887	-0.193774456	0.080770709	-0.274545164	0.562549552
ENSG00000146555	*SDK1*	-1.230384432	0.231691227	-0.01168859	0.243379817	0.999933887	-0.338166849	0.019175245	-0.357342094	0.562980648
ENSG00000118898	*PPL*	1.465695925	-0.037421156	-0.03496464	-0.002456516	0.999933887	-0.484591591	-0.287648248	-0.196943344	0.562980648
ENSG00000132603	*NIP7*	3.171340591	-0.082144406	0.020815338	-0.102959745	0.999933887	-0.034541102	-0.137437393	0.102896291	0.563050611
ENSG00000161055	*SCGB3A1*	-2.141746029	0.235333214	-0.3863466	0.621679814	0.999933887	0.375854007	0.018680562	0.357173445	0.563077771
ENSG00000165487	*MICU2*	4.099271165	0.025503684	-0.006913475	0.032417158	0.999933887	-0.442047259	-0.319054871	-0.122992388	0.563077771
ENSG00000073578	*SDHA*	1.621497085	0.034713254	-0.057278818	0.091992071	0.999933887	0.05839533	-0.093660881	0.152056211	0.563204084
ENSG00000167608	*TMC4*	1.142634172	0.194414579	0.049811164	0.144603414	0.999933887	-0.054184892	-0.274756946	0.220572054	0.563261988
ENSG00000117360	*PRPF3*	5.349143092	-0.027066206	0.017191282	-0.044257489	0.999933887	0.347442826	0.265463431	0.081979395	0.563261988
ENSG00000060140	*STYK1*	-0.678735529	0.068786163	-0.702839325	0.771625488	0.944094537	-0.069133506	0.222859042	-0.291992548	0.563261988
ENSG00000136870	*ZNF189*	3.287560637	-0.048218714	-0.1245654	0.076346686	0.999933887	0.029250013	0.126978325	-0.097728312	0.563352441
ENSG00000011295	*TTC19*	3.547705584	-0.069208134	-0.040057075	-0.029151059	0.999933887	0.032736675	-0.04184269	0.074579365	0.563591477
ENSG00000248593	*DSTNP2*	-0.116594005	-0.056770003	0.006240994	-0.063010997	0.999933887	0.135510231	-0.14860467	0.284114901	0.5636489
ENSG00000167770	*OTUB1*	5.603109796	0.062922002	-0.02884781	0.091769811	0.999933887	-0.026376395	-0.089507752	0.063131357	0.56373233
ENSG00000287771	*AC007271.1*	2.184513023	0.042521808	0.104080067	-0.061558259	0.999933887	0.689457369	0.856810116	-0.167352747	0.563852117
ENSG00000159784	*FAM131B*	-0.543843757	0.243727378	-0.244088364	0.487815742	0.999933887	-0.335640437	-0.731397041	0.395756604	0.564004956
ENSG00000115084	*SLC35F5*	4.74312492	-0.017386139	-0.011262184	-0.006123955	0.999933887	-0.465902552	-0.35879717	-0.107105383	0.564005472
ENSG00000167543	*TP53I13*	3.511157858	0.008221598	0.21959445	-0.211372852	0.999933887	0.094642748	-0.006215231	0.100857979	0.564354962
ENSG00000169184	*MN1*	-0.465442883	0.1655865	0.168639124	-0.003052624	0.999933887	0.19260331	0.498230978	-0.305627669	0.56442543
ENSG00000011258	*MBTD1*	4.132854868	-0.039222544	-0.092290091	0.053067547	0.999933887	-0.629480758	-0.748202908	0.118722151	0.564455386
ENSG00000154473	*BUB3*	4.504072939	-0.009660734	-0.072479614	0.06281888	0.999933887	-0.027971061	-0.111324608	0.083353546	0.564629376
ENSG00000168092	*PAFAH1B2*	5.632676947	-0.113612196	-0.027737218	-0.085874978	0.999933887	-0.59410867	-0.6863414	0.09223273	0.56469706
ENSG00000085733	*CTTN*	-0.680426681	-0.394103261	0.065304689	-0.45940795	0.999933887	-0.256598635	0.040882918	-0.297481553	0.56469706
ENSG00000248124	*RRN3P1*	5.060273886	-0.029248388	-0.059616042	0.030367653	0.999933887	-0.020527216	0.067914934	-0.088442151	0.56469706
ENSG00000164331	*ANKRA2*	3.634290374	-0.001805273	0.034185382	-0.035990655	0.999933887	-0.047397474	0.034128365	-0.08152584	0.56469706
ENSG00000167566	*NCKAP5L*	3.777376706	0.069145544	-0.019397518	0.088543062	0.999933887	0.020672165	-0.099307592	0.119979757	0.564737629
ENSG00000147592	*LACTB2*	1.396755509	0.179434457	0.008653489	0.170780968	0.999933887	0.056749784	-0.069138032	0.125887816	0.564744323
ENSG00000272899	*ATP6V1FNB*	3.243042497	0.084925921	0.355019606	-0.270093684	0.944094537	0.798262131	0.901234706	-0.102972575	0.564756864
ENSG00000171806	*METTL18*	-0.041691795	-0.339884261	0.140600393	-0.480484654	0.999933887	-0.072290719	0.141998887	-0.214289606	0.565095154
ENSG00000222043	*AC079305.1*	3.130071861	0.162885249	0.153406596	0.009478653	0.999933887	1.761551987	1.961331431	-0.199779445	0.565242069
ENSG00000237914	*SIRPG-AS1*	-1.320040626	0.058964257	-0.50777597	0.566740227	0.999933887	-0.182793119	0.160165868	-0.342958987	0.565383058
ENSG00000124126	*PREX1*	10.485574979	0.024324251	0.043091359	-0.018767107	0.999933887	0.009914935	-0.040438008	0.050352943	0.565929937
ENSG00000128694	*OSGEPL1*	-0.594480308	0.052520571	-0.072120336	0.124640907	0.999933887	-0.145660736	0.14282931	-0.288490046	0.565971592
ENSG00000179715	*PCED1B*	3.107635063	-0.019225145	0.005837986	-0.025063132	0.999933887	0.099033785	0.011273526	0.087760259	0.565974227

ENSG00000156170	*NDUFAF6*	1.049500426	-0.015188397	0.185036313	-0.20022471	0.999933887	0.19421301	0.396704575	-0.202491564	0.566199774
ENSG00000130544	*ZNF557*	3.162681662	-0.118877583	-0.114844425	-0.004033158	0.999933887	-0.245711982	-0.153565107	-0.092146875	0.566261905
ENSG00000263982	*AC009716.1*	-0.145245261	0.218404327	0.005943362	0.212460965	0.999933887	-0.158172264	-0.433651331	0.275479067	0.566261905
ENSG00000026950	*BTN3A1*	7.167291459	0.012539148	0.014593349	-0.002054201	0.999933887	-0.117092316	-0.180782805	0.063690489	0.566261905
ENSG00000182759	*MAFA*	-0.78951907	0.093340896	-0.487277866	0.580618763	0.999933887	-0.508960043	-0.854248017	0.345287974	0.566442075
ENSG00000175756	*AURKAIP1*	6.228130577	0.094475153	0.141885575	-0.047410422	0.999933887	0.02277609	-0.099457076	0.122233166	0.566500352
ENSG00000281398	*SNHG4*	0.35926767	-0.209164583	-0.264475003	0.05531042	0.999933887	0.733194882	0.941474405	-0.208279523	0.566738862
ENSG00000121058	*COIL*	2.90296333	-0.078288046	-0.061276059	-0.017011987	0.999933887	-0.395828388	-0.28697147	-0.108856918	0.566738862
ENSG00000198792	*TMEM184B*	7.250241482	-0.003979249	0.034295078	-0.038274327	0.999933887	-0.010053993	0.06112982	-0.071183812	0.566738862
ENSG00000087299	*L2HGDH*	-0.704774498	-0.023097658	-0.135398031	0.112300374	0.999933887	-0.24783561	0.132403263	-0.380238872	0.566809675
ENSG00000188042	*ARL4C*	7.468484542	0.022025465	0.007168945	0.01485652	0.999933887	0.09782645	0.169239143	-0.071412693	0.566921082
ENSG00000072954	*TMEM38A*	-0.480096911	0.098816845	0.124502999	-0.025686154	0.999933887	-0.585298441	-0.216149772	-0.36914867	0.566984193
ENSG00000285533	*RELA-DT*	1.285348957	-0.04090477	-0.048245266	0.007340497	0.999933887	-0.138075633	-0.317838511	0.179762878	0.567244328
ENSG00000151491	*EPS8*	-0.181424463	-0.22741609	-0.067314591	-0.160101499	0.999933887	-0.374870014	-0.174070098	-0.200799916	0.567380433
ENSG00000157379	*DHRS1*	4.319071365	0.127229541	0.050273499	0.076956042	0.999933887	0.901197471	1.008921359	-0.107723889	0.567584099
ENSG00000166436	*TRIM66*	2.874796416	0.050406775	-0.068004991	0.118411766	0.999933887	-0.16324769	-0.272630014	0.109382325	0.567673512
ENSG00000094916	*CBX5*	3.142790172	-0.085180904	-0.072719262	-0.012461642	0.999933887	0.003383866	0.112642055	-0.109258189	0.567871478
ENSG00000115944	*COX7A2L*	4.320247985	-0.010323597	-0.116150578	0.105826981	0.999933887	-0.237346384	-0.326041198	0.088694814	0.567873151
ENSG00000168461	*RAB31*	7.009263517	-0.064364411	-0.041108951	-0.02325546	0.999933887	-0.782864412	-0.682857203	-0.100007209	0.567876769
ENSG00000248571	*AC106882.1*	0.513814192	-0.242624496	-0.080495518	-0.162128978	0.999933887	-0.006521133	-0.311401466	0.304880334	0.56791542
ENSG00000205085	*FAM71F2*	1.624470165	-0.06824608	-0.05495993	-0.013286151	0.999933887	0.068039911	0.258473831	-0.19043392	0.56791542
ENSG00000085832	*EPS15*	5.203554586	0.0117669	-0.012983621	0.024750521	0.999933887	-0.415927741	-0.526074616	0.110146875	0.56791542
ENSG00000117016	*RIMS3*	-0.675897421	0.118567001	0.525261487	-0.406694485	0.999933887	0.016306264	0.344826161	-0.328519897	0.567968947
ENSG00000162825	*NBPF20*	-0.514150601	-0.033022357	0.025513001	-0.058535357	0.999933887	0.114616599	0.426381114	-0.311764515	0.567968947
ENSG00000100485	*SOS2*	6.29809357	0.032232705	-0.081751156	0.113983861	0.999933887	-0.621754711	-0.488774328	-0.132980383	0.568125869
ENSG00000157827	*FMNL2*	1.067129355	-0.01131511	-0.007530901	-0.003784209	0.999933887	-0.579745901	-0.772595699	0.192849798	0.568125869
ENSG00000260306	*AC092375.2*	0.393527898	0.364225668	0.18712618	0.177099488	0.999933887	0.363721963	0.602352456	-0.238630493	0.568239223
ENSG00000177294	*FBXO39*	-1.595489311	-0.209375686	-0.056705231	-0.152670455	0.999933887	0.059479545	-0.477721856	0.537201401	0.568239223
ENSG00000259436	*AC010247.2*	0.689003971	-0.290997953	0.132170371	-0.423168324	0.999933887	0.362150335	0.528883075	-0.166732739	0.568523334
ENSG00000237973	*MTCO1P12*	2.657220577	-0.046707539	-0.040517014	-0.006190524	0.999933887	0.027623083	0.159631642	-0.132008559	0.568523334
ENSG00000143365	*RORC*	0.233725422	0.189227042	0.035077402	0.154149639	0.999933887	0.133237238	-0.114687005	0.247924243	0.568913638
ENSG00000198538	*ZNF28*	1.103745098	-0.00662194	-0.246118782	0.239496842	0.999933887	-0.32249473	-0.148000928	-0.174493802	0.568913638
ENSG00000144596	*GRIP2*	0.349321166	-0.177019376	-0.018969974	-0.158049401	0.999933887	0.204105688	0.454131521	-0.250025833	0.568961365
ENSG00000211459	*MT-RNR1*	9.717257386	0.116654584	0.177056378	-0.060401794	0.999933887	0.314043947	0.436076916	-0.122032969	0.568976133
ENSG00000127603	*MACF1*	7.33027726	-0.059855088	-0.024873389	-0.034981699	0.999933887	-0.343855365	-0.448512556	0.104657191	0.568976133
ENSG00000274280	*MIR6069*	1.611574008	0.031480805	0.052895359	-0.021414554	0.999933887	0.702087855	0.889806888	-0.187719032	0.568987666
ENSG00000237298	*TTN-AS1*	2.075569751	-0.019097172	0.006072755	-0.025169927	0.999933887	-0.231786441	-0.390047993	0.158261553	0.569247989
ENSG00000281005	*LINC00921*	4.827250191	-0.016300833	0.06770834	-0.084009173	0.999933887	-0.023861469	0.069209316	-0.093070785	0.569350807
ENSG00000198887	*SMC5*	3.802907565	-0.022202388	-0.130556307	0.108353919	0.999933887	-0.378416717	-0.167018298	-0.211398419	0.569350807
ENSG00000141448	*GATA6*	-0.833963785	0.174853724	0.622827282	-0.447973558	0.999933887	0.059068317	-0.239712762	0.298781079	0.569381478
ENSG00000273345	*AC104109.4*	-0.268471656	0.026874342	-0.076623833	0.103498175	0.999933887	0.018028553	0.29488567	-0.276857118	0.569381478
ENSG00000112977	*DAP*	5.750891097	-0.002937211	-0.034066374	0.031129163	0.999933887	-0.036856219	-0.11624957	0.07939335	0.569415654
ENSG00000063015	*SEZ6*	2.862132983	0.101573359	0.080276001	0.021297359	0.999933887	0.707590826	0.851986354	-0.144395527	0.569527472
ENSG00000137818	*RPLP1*	8.222601129	0.009874096	-0.035113358	0.044987454	0.999933887	0.174441765	0.249719871	-0.075278105	0.569527472
ENSG00000123453	*SARDH*	-0.868969998	0.025141513	0.129834543	-0.10469303	0.999933887	-0.187946059	0.147008496	-0.334954555	0.569839305
ENSG00000149292	*TTC12*	1.540736335	0.108249474	0.038962472	0.069287003	0.999933887	0.001875971	-0.133588851	0.135464822	0.569956359
ENSG00000135045	*C9orf40*	3.306364165	-0.083408419	-0.144054399	0.06064598	0.999933887	-0.662247043	-0.568647178	-0.093599865	0.569956359
ENSG00000153339	*TRAPPC8*	5.714015199	-0.079886276	-0.111995125	0.03210885	0.999933887	-0.653926713	-0.531682471	-0.122244243	0.570240065
ENSG00000260033	*AC023813.2*	0.621122527	0.366792758	0.38891981	-0.022127052	0.999933887	1.530694765	1.899030381	-0.368335617	0.570460983
ENSG00000284526	*AC015802.6*	1.249286283	-0.112649066	-0.017341437	-0.095307629	0.999933887	-0.810809438	-0.961391426	0.150581988	0.570460983
ENSG00000109536	*FRG1*	3.73603053	-0.045490619	-0.042073456	-0.003417163	0.999933887	-0.199476051	-0.100301137	-0.099174914	0.570460983
ENSG00000167118	*URM1*	3.642712556	-0.022919832	-0.00696293	-0.015956902	0.999933887	0.026658888	-0.057642575	0.084301463	0.570460983
ENSG00000113319	*RASGRF2*	1.606123854	0.023752786	0.101034246	-0.07728146	0.999933887	-0.004029564	0.137703983	-0.141733547	0.570460983
ENSG00000174444	*RPL4*	8.111058384	-0.032523424	-0.13617366	0.103650236	0.999933887	0.107289747	0.187437491	-0.080147743	0.570571834
ENSG00000008988	*RPS20*	7.611378184	-0.00356769	-0.062873249	0.059305559	0.999933887	0.166057133	0.244329051	-0.078271918	0.570713792
ENSG00000179029	*TMEM107*	1.421260505	0.032040688	0.151962667	-0.119921979	0.999933887	0.107113138	-0.106861262	0.2139744	0.570921711
ENSG00000242324	*AL049634.1*	0.096602709	-0.081898838	-0.135633197	0.053734359	0.999933887	-0.223224515	-0.451664277	0.228439762	0.571222139
ENSG00000160767	*FAM189B*	1.933311028	0.115452943	-0.00305761	0.118510552	0.999933887	-0.073017042	-0.216065176	0.143048134	0.571247958
ENSG00000172613	*RAD9A*	4.9856506	0.07798223	0.068320369	0.009661861	0.999933887	0.103231077	0.011511778	0.091719299	0.571405204
ENSG00000138668	*HNRNPD*	6.902225375	0.005578614	0.018292616	-0.012714002	0.999933887	-0.187682034	-0.249232374	0.061550341	0.571448182
ENSG00000241975	*ELOCP19*	0.407720553	0.028693233	-0.094422603	0.123115836	0.999933887	-0.724622906	-1.214032822	0.489409916	0.571674884
ENSG00000285793	*ANAPC1P2*	1.670963229	0.145414104	0.125779006	0.019635099	0.999933887	-0.752863634	-0.98240473	0.229541096	0.57172616
ENSG00000255320	*AP000759.1*	1.002134254	-0.052261049	-0.096002449	0.043741401	0.999933887	-0.338081837	-0.576678213	0.238596376	0.57172616
ENSG00000170906	*NDUFA3*	3.085555927	0.036216905	-0.025661408	0.061878314	0.999933887	0.031206228	-0.077918865	0.109125093	0.57172616
ENSG00000114107	*CEP70*	-0.550199204	-0.219825528	-0.264725453	0.044899926	0.999933887	-0.06755548	0.264278086	-0.331833566	0.571796712
ENSG00000062725	*APPBP2*	4.094963494	-0.090424612	-0.049678042	-0.04074657	0.999933887	-0.312526505	-0.22538412	-0.087142385	0.571861734
ENSG00000137265	*IRF4*	4.090161946	-0.086991433	0.045481364	-0.132472797	0.999933887	0.326328054	0.415450534	-0.089122479	0.572037927
ENSG00000158528	*PPP1R9A*	-1.684315186	-0.413424794	-0.42415531	0.010730516	0.999933887	-1.22254922	-1.477672156	0.255122936	0.572037927
ENSG00000284697	*AC006511.5*	0.38237926	-0.187467385	0.001150065	-0.18861745	0.999933887	0.808403545	1.035329976	-0.226926431	0.572037927
ENSG00000166965	*RCCD1*	0.300824272	0.078434699	-0.004275569	0.082710268	0.999933887	0.229069902	0.024130882	0.20493902	0.572037927
ENSG00000274210	*RNVU1-27*	-0.851814116	-0.021701088	0.271909587	-0.293610675	0.999933887	0.377132021	0.055948215	0.321183807	0.572037927
ENSG00000118971	*CCND2*	4.828980693	-0.062519831	0.028729427	-0.091249258	0.999933887	-0.100293601	-0.180186288	0.079892687	0.572437936
ENSG00000105369	*CD79A*	2.035844489	0.132244675	-0.04526385	0.177508525	0.999933887	-0.009073698	0.122519699	-0.131593398	0.572470886
ENSG00000162419	*GMEB1*	4.415546727	-0.020564404	0.006130674	-0.026695078	0.999933887	-0.105227385	-0.034348071	-0.070879314	0.572621611
ENSG00000196214	*ZNF766*	3.725470031	-0.176581528	-0.187988593	0.011407064	0.999933887	-0.52860221	-0.639599569	0.110997359	0.572782162
ENSG00000171135	*JAGN1*	2.465831264	-0.139757808	-0.10305142	-0.036706388	0.999933887	0.030443119	-0.075757369	0.106200489	0.572782162
ENSG00000265399	*AP005329.2*	-0.168606598	0.213797549	0.319683607	-0.105886059	0.999933887	0.045808411	0.270819608	-0.225011196	0.572782162
ENSG00000135205	*CCDC146*	1.945493296	-0.121167015	-0.191521885	0.07035487	0.999933887	-0.196940826	0.042190876	-0.239131702	0.572782162
ENSG00000229515	*FLT1P1*	0.589264672	0.161414331	-0.08405722	0.245471552	0.999933887	-0.886886556	-0.550362738	-0.336523817	0.572782162
ENSG00000269959	*SPACA6P-AS*	-0.696989505	0.19563065	0.112193814	0.083436836	0.999933887	1.43728859	1.7541221	-0.31683351	0.572782162
ENSG00000119632	*IFI27L2*	1.881202022	0.067443119	0.008682299	0.05876082	0.999933887	0.570757338	0.752978685	-0.182221347	0.572782162
ENSG00000171777	*RASGRP4*	7.157490517	0.024372648	0.074659153	-0.050286505	0.999933887	-0.103254122	-0.181129788	0.077875666	0.572782162
ENSG00000249437	*NAIP*	4.0090528	-0.028012262	-0.031673497	0.003661235	0.999933887	-0.953479878	-1.066484051	0.113004174	0.572782162
ENSG00000119686	*FLVCR2*	0.729425534	-0.048241847	-0.109747446	0.061505598	0.999933887	0.194753804	0.43452954	-0.239775736	0.572782162
ENSG00000288056	*AL023806.4*	-1.569212785	-0.026753742	-0.293186965	0.266433222	0.999933887	0.090228524	0.304845074	-0.21461655	0.57295526
ENSG00000108021	*TASOR2*	4.380541027	-0.02915051	-0.167522627	0.138372117	0.999933887	-0.335818269	-0.168588926	-0.167229343	0.573151185
ENSG00000134490	*TMEM241*	-0.68124367	-0.389663105	0.236918908	-0.626582013	0.999933887	-0.288043928	-0.018524872	-0.269519056	0.573171562
ENSG00000139537	*CCDC65*	1.103340177	-0.049364968	-0.02157774	-0.027787228	0.999933887	-0.072999729	0.079171982	-0.152171711	0.573398829
ENSG00000123080	*CDKN2C*	0.660342819	0.17815334	0.009796097	0.168357243	0.999933887	0.781413561	0.989070049	-0.207656489	0.573518953
ENSG00000260005	*AC027601.1*	1.975271982	-0.009551325	0.144248773	-0.153800097	0.999933887	-0.164472365	-0.003366604	-0.161105761	0.574074781
ENSG00000236088	*COX10-AS1*	0.85943727	0.091220918	-0.224887104	0.316108022	0.999933887	-0.244932702	-0.062474586	-0.182458116	0.574146362
ENSG00000222046	*DCDC2B*	-0.014090983	0.08206942	-0.181778805	0.263848225	0.999933887	0.177771972	-0.098740574	0.276512546	0.574146452
ENSG00000131773	*KHDRBS3*	-0.544480653	-0.060943747	0.29891831	-0.359862057	0.999933887	1.396932238	1.619357035	-0.222424798	0.574149064
ENSG00000148773	*MKI67*	0.124703761	0.020824774	0.094576485	-0.073751711	0.999933887	0.221261258	0.499067255	-0.277805997	0.574468447
ENSG00000069329	*VPS35*	5.720197424	-0.120036099	-0.146946194	0.026910095	0.999933887	-0.571128177	-0.632317459	0.061189282	0.575286473
ENSG00000141570	*CBX8*	1.001800413	0.092678191	0.12948333	-0.036805139	0.999933887	-0.11884108	-0.348801853	0.229960773	0.575286473
ENSG00000213600	*U73169.1*	-0.52802878	-0.061768676	-0.243208409	0.181439733	0.999933887	-0.585814708	-0.904121249	0.318306541	0.575286473
ENSG00000144036	*EXOC6B*	0.693264892	0.032119215	-0.163385923	0.195505138	0.999933887	-0.267713929	-0.089535111	-0.178178818	0.575286473
ENSG00000004866	*ST7*	-0.109380883	-0.212852054	0.038405978	-0.251258032	0.999933887	-0.159183086	0.086377084	-0.24556017	0.575391422
ENSG00000171612	*SLC25A33*	4.149162284	0.006688122	-0.027103935	0.033792057	0.999933887	0.456512015	0.572415645	-0.11590363	0.575391422
ENSG00000074582	*BCS1L*	1.505007344	-0.013574233	-0.17122967	0.157655436	0.999933887	0.411256919	0.544831726	-0.133574808	0.575588161
ENSG00000146707	*POMZP3*	1.926839433	-0.119104449	-0.014760596	-0.104343852	0.999933887	0.751879496	0.933945865	-0.182066369	0.575588347
ENSG00000085788	*DDHD2*	2.926994666	-0.078253993	-0.20026648	0.122012487	0.999933887	-0.009948052	0.096556769	-0.10650482	0.575730251
ENSG00000157350	*ST3GAL2*	7.213195815	0.050098852	0.064258677	-0.014159825	0.999933887	0.28445554	0.377811552	-0.093356012	0.575999234
ENSG00000213965	*NUDT19*	1.307102304	-0.049246314	0.122326299	-0.171572614	0.999933887	-0.041253694	0.140134509	-0.181388203	0.575999234

ENSG00000282133	*TRBJ1-3*	-0.481853579	0.161739052	0.058981965	0.102757087	0.999933887	-0.019033068	0.220868068	-0.239901136	0.576072414
ENSG00000180694	*TMEM64*	1.664996866	-0.084662256	0.029156756	-0.113819012	0.999933887	0.095996276	0.27031783	-0.174321554	0.576072414
ENSG00000124383	*MPHOSPH10*	2.71920241	-0.161404682	-0.237357648	0.075952966	0.999933887	-0.096468241	0.037521762	-0.133990004	0.576200875
ENSG00000147679	*UTP23*	2.29151121	-0.077486663	-0.134127942	0.056641279	0.999933887	-0.303977771	-0.137959896	-0.166017875	0.576386738
ENSG00000254676	*AP000873.4*	0.45053472	0.184420088	0.237072852	-0.052652764	0.999933887	0.17026258	0.390425055	-0.220162475	0.576469021
ENSG00000158158	*CNNM4*	3.312896499	0.092660922	0.078845629	0.013815293	0.999933887	-0.134732383	-0.029099999	-0.105632384	0.576469021
ENSG00000282980	*AC055839.1*	-0.451993646	-0.229831507	0.040760015	-0.270591522	0.999933887	-0.070679658	-0.488922132	0.418242474	0.576469021
ENSG00000259719	*LINC02284*	-0.489747442	0.110204588	0.114197654	-0.003993065	0.999933887	-0.588591003	-0.148413667	-0.440177336	0.576469021
ENSG00000134215	*VAV3*	4.708047913	0.029766667	-0.065686977	0.095453644	0.999933887	-0.212800754	-0.300995367	0.088194613	0.576469021
ENSG00000181036	*FCRL6*	3.134879942	-0.016402187	-0.098894352	0.082492165	0.999933887	0.092620727	-0.008938367	0.101559094	0.576469021
ENSG00000080007	*DDX43*	-0.021204931	-0.038851303	0.259374951	-0.298226254	0.999933887	0.47249668	0.712060319	-0.23956364	0.576469021
ENSG00000152454	*ZNF256*	0.129039501	0.025471363	0.437225088	-0.411753725	0.999933887	0.337173242	0.119907253	0.21726599	0.576469021
ENSG00000108506	*INTS2*	1.593706942	-0.007139328	-0.203336448	0.196197121	0.999933887	-0.076852831	0.075342359	-0.15219519	0.576469021
ENSG00000204392	*LSM2*	1.016253269	0.001666583	-0.111926764	0.113593347	0.999933887	-0.045119138	0.151704144	-0.196823282	0.576469021
ENSG00000185024	*BRF1*	3.410992425	0.028497249	0.111494163	-0.082996913	0.999933887	0.068178797	-0.028883716	0.097062513	0.576992395
ENSG00000163026	*WDCP*	0.086642408	0.205770626	0.190919188	0.014851438	0.999933887	0.204751012	-0.002487477	0.207238489	0.577159371
ENSG00000052126	*PLEKHA5*	0.376338376	-0.153594911	-0.093633138	-0.059961773	0.999933887	-0.334685286	-0.074852229	-0.259833057	0.577349983
ENSG00000076944	*STXBP2*	7.792165593	0.045435686	0.074497757	-0.029062071	0.999933887	0.276699033	0.347614945	-0.070915912	0.577786042
ENSG00000157216	*SSBP3*	4.060045569	0.049669663	0.027585121	0.022084542	0.999933887	0.12872812	0.222899442	-0.094171322	0.577786042
ENSG00000181778	*TMEM252*	0.033309511	0.088704952	0.064449635	0.024255318	0.999933887	0.639669056	0.862996507	-0.223327451	0.57779728
ENSG00000137078	*SIT1*	1.899152849	0.012650036	0.082866233	-0.070216197	0.999933887	0.24519568	0.092887493	0.152308187	0.577811162
ENSG00000276698	*AL136295.6*	0.138622483	0.021198626	-0.157485724	0.17868435	0.999933887	0.203321413	0.013121042	0.190200371	0.577811796
ENSG00000131351	*HAUS8*	2.417225333	0.050448664	0.058186296	-0.007737632	0.999933887	-0.164409686	-0.062522268	-0.101887418	0.577905855
ENSG00000107341	*UBE2R2*	8.307491259	0.017570836	0.002886393	0.014684442	0.999933887	-0.661662777	-0.584282974	-0.077379804	0.577953129
ENSG00000131196	*NFATC1*	5.031377923	-0.007964305	0.056409768	-0.064374073	0.999933887	0.187214515	0.258632036	-0.071417521	0.577966497
ENSG00000120675	*DNAJC15*	1.814093381	0.007766383	-0.081321931	0.089088314	0.999933887	-0.128350768	0.041917167	-0.170267935	0.577966497
ENSG00000100151	*PICK1*	2.812322116	0.113579231	0.062760555	0.050818676	0.999933887	0.16340588	0.263092949	-0.099687068	0.577984339
ENSG00000256087	*ZNF432*	2.127716384	-0.103868577	-0.140687994	0.036819417	0.999933887	-0.044936929	0.114011012	-0.158947941	0.577984339
ENSG00000130669	*PAK4*	2.044980785	0.03515011	0.323248743	-0.288098632	0.999933887	0.224100291	0.347592431	-0.12349214	0.577984339
ENSG00000149196	*HIKESHI*	1.410460199	0.050876895	0.064029978	-0.013153082	0.999933887	0.115177096	0.240718705	-0.125541609	0.578070644
ENSG00000122707	*RECK*	0.747465722	0.08656855	-0.276930267	0.363498817	0.999933887	-0.150220456	0.04667756	-0.196898017	0.578470193
ENSG00000163818	*LZTFL1*	2.737564358	-0.012699885	0.002882247	-0.015582131	0.999933887	-0.721264865	-0.573567153	-0.147697712	0.578470193
ENSG00000102312	*PORCN*	1.891602943	0.041769883	0.099724973	-0.05795509	0.999933887	0.096665649	0.23811286	-0.141447211	0.578472056
ENSG00000242574	*HLA-DMB*	2.287137769	0.003720641	-0.110022925	0.113743566	0.999933887	-0.362319633	-0.504868343	0.142548711	0.578472056
ENSG00000147124	*ZNF41*	1.155210941	-0.102309832	0.064482107	-0.166791939	0.999933887	0.052128244	-0.119652142	0.171780387	0.578500016
ENSG00000198858	*R3HDM4*	9.260326339	0.038209896	0.01843845	0.019771447	0.999933887	-0.305738606	-0.377960101	0.072221495	0.578500016
ENSG00000135845	*PIGC*	2.771170838	-0.008949543	-0.131887303	0.122937761	0.999933887	-0.136342239	-0.250137498	0.113795259	0.578500016
ENSG00000213994	*AL157395.1*	-1.306686269	-0.015343761	0.243573739	-0.2589175	0.999933887	-0.211552796	0.357746436	-0.569299233	0.578500016
ENSG00000123612	*ACVR1C*	-1.0242719	-0.103262039	0.090616956	-0.193878995	0.999933887	-0.423098532	-0.056563645	-0.366534887	0.578959057
ENSG00000211797	*TRAV17*	-0.382161841	-0.027415506	0.202107158	-0.229522665	0.999933887	0.133399723	0.360356066	-0.226956343	0.578959057
ENSG00000187764	*SEMA4D*	8.215544589	-0.020195574	-0.003840227	-0.016355347	0.999933887	0.236049994	0.143231487	0.092818506	0.579198648
ENSG00000283674	*AC068587.4*	-0.157712047	-0.066239792	0.187008323	-0.253248114	0.999933887	-0.215592199	0.11212591	-0.327718108	0.579273169
ENSG00000236426	*AL583824.1*	-0.279236554	0.2068294	0.03714299	0.16968641	0.999933887	0.69761883	0.933211291	-0.235592461	0.579395657
ENSG00000184752	*NDUFA12*	2.496362883	-0.071852537	0.019471603	-0.091324141	0.999933887	0.025346225	-0.086941271	0.112287495	0.579395657
ENSG00000256581	*NLRP9P1*	1.525390939	-0.070396717	-0.115353096	0.044956379	0.999933887	-0.381547327	-0.17506141	-0.206485917	0.579395657
ENSG00000134744	*TUT4*	4.800696863	-0.042218442	-0.076888933	0.034670491	0.999933887	-0.292503046	-0.166990797	-0.12551225	0.57974535
ENSG00000176387	*HSD11B2*	1.881682979	0.132057167	0.429012373	-0.296955206	0.999933887	1.063340327	0.826322892	0.237017435	0.579845605
ENSG00000254635	*WAC-AS1*	3.798699484	-0.016666839	0.076778265	-0.093445105	0.999933887	-0.29077642	-0.195238965	-0.095537455	0.579868853
ENSG00000167525	*PROCA1*	2.084636866	0.159545387	0.172545776	-0.013000389	0.999933887	0.059069687	-0.100875779	0.159945465	0.579977509
ENSG00000080854	*IGSF9B*	2.832370444	-0.090331349	0.03022567	-0.120557019	0.999933887	0.020071677	0.120730314	-0.100658637	0.579977509
ENSG00000165861	*ZFYVE1*	5.710547918	-0.082234491	0.052512152	-0.134746643	0.999933887	-0.056001129	0.014530075	-0.070531204	0.580066163
ENSG00000114316	*USP4*	7.546363848	-0.046588929	-0.022873778	-0.023715152	0.999933887	-0.175989828	-0.222147319	0.04615749	0.580066163
ENSG00000260467	*AC018552.2*	1.916789557	-0.045356567	0.061036622	-0.106393189	0.999933887	-0.239389514	0.002643161	-0.242032674	0.580066163
ENSG00000107242	*PIP5K1B*	1.397796968	-0.045737861	-0.032798895	-0.012938967	0.999933887	-0.120548344	-0.287101658	0.166553314	0.580160851
ENSG00000180385	*EMC3-AS1*	0.999522829	-0.004320186	-0.27450382	0.270183634	0.999933887	-0.603822884	-0.454120104	-0.14970278	0.580160851
ENSG00000131876	*SNRPA1*	3.987077611	-0.08894675	-0.065045343	-0.023901407	0.999933887	0.068295863	0.135747767	-0.067451905	0.580344372
ENSG00000106789	*CORO2A*	3.56094914	0.104913867	0.150433284	-0.045519417	0.999933887	-0.031288644	0.071793137	-0.103081781	0.580344372
ENSG00000163655	*GMPS*	2.61657835	-0.044856486	-0.137159043	0.092302557	0.999933887	0.049419745	-0.049703504	0.099123249	0.580344372
ENSG00000160972	*PPP1R16A*	2.041228888	0.053865432	0.142503863	-0.088638431	0.999933887	0.291483539	0.13624049	0.15524305	0.580344372
ENSG00000183935	*HTR7P1*	1.354285353	0.012880411	0.174008467	-0.161128057	0.999933887	-0.063290111	0.089928738	-0.153218849	0.580507367
ENSG00000106003	*LFNG*	5.698483983	0.00257957	-0.025676923	0.028256493	0.999933887	0.281804196	0.380683661	-0.098879465	0.580579222
ENSG00000172508	*CARNS1*	1.30735398	0.094924361	0.109322327	-0.014397966	0.999933887	0.042237746	-0.124451689	0.166689435	0.580766234
ENSG00000050344	*NFE2L3*	2.952940707	-0.016937178	0.134612252	-0.151549431	0.999933887	-0.27432252	-0.14527847	-0.129044051	0.580766234
ENSG00000161509	*GRIN2C*	0.747529848	-0.105449921	-0.05287363	-0.052576291	0.999933887	-0.196044239	0.034682821	-0.23072706	0.580830142
ENSG00000172171	*TEFM*	-0.331971938	-0.087228573	0.195978919	-0.283207492	0.999933887	-0.1059559	0.147384581	-0.253340481	0.580890445
ENSG00000167535	*CACNB3*	0.350667633	0.013053738	0.223520052	-0.210466314	0.999933887	0.199379951	0.015870559	0.183509392	0.580890445
ENSG00000260911	*AC135050.3*	2.218941211	0.091972127	0.223854821	-0.131882694	0.999933887	0.518036966	0.634968711	-0.116931745	0.581226211
ENSG00000163291	*PAQR3*	2.068594268	0.020008139	-0.045544692	0.065552831	0.999933887	-0.257024284	-0.051581121	-0.205443163	0.581226211
ENSG00000042832	*TG*	2.735796233	-0.005430906	-0.039945089	0.034514183	0.999933887	0.096727715	-0.043735206	0.140462921	0.581350246
ENSG00000283538	*AC005972.3*	0.295131453	0.283281715	0.588669515	-0.3053878	0.999933887	-0.519549729	-0.78001054	0.260460811	0.581451905
ENSG00000181409	*AATK*	8.681984464	0.060003583	0.037500079	0.022503505	0.999933887	0.100715579	0.206615084	-0.105899505	0.581451905
ENSG00000228492	*RAB11FIP1P1*	-0.511414621	-0.34383886	-0.403421199	0.059582339	0.999933887	-0.42993137	-0.142311673	-0.287619697	0.581497419
ENSG00000255221	*CARD17*	4.793231737	0.107855359	-0.049465497	0.157320856	0.999933887	1.131596407	1.272570165	-0.140973758	0.581723793
ENSG00000228113	*AC003991.1*	0.699196985	-0.072666482	-0.125848392	0.05318191	0.999933887	-0.880002109	-0.538673373	-0.341328736	0.581723793
ENSG00000157404	*KIT*	0.952708911	-0.020397354	-0.08871247	0.068315116	0.999933887	-0.221133492	-0.08620873	-0.134924762	0.581723793
ENSG00000144233	*AMMECR1L*	4.145466458	-0.052899175	0.000621928	-0.053521103	0.999933887	-0.299822738	-0.371012566	0.071189828	0.581737478
ENSG00000165424	*ZCCHC24*	2.692287159	-0.040749985	0.097778839	-0.138528824	0.999933887	-0.498027937	-0.379186459	-0.118841478	0.581737478
ENSG00000286886	*AJ011931.2*	-0.769281762	-0.179154372	0.020310426	-0.199464798	0.999933887	0.432961427	0.21592783	0.217033597	0.582205778
ENSG00000174500	*GCSAM*	0.830096548	0.073185812	-0.121133045	0.194318857	0.999933887	0.258878445	0.459151563	-0.200273117	0.582302311
ENSG00000162735	*PEX19*	3.183544555	-0.019846951	0.095190021	-0.115036972	0.999933887	0.111558133	0.21823533	-0.106677197	0.582302311
ENSG00000203668	*CHML*	1.262163461	-0.020508531	-0.238906117	0.218397586	0.999933887	0.182461908	0.409440529	-0.22697862	0.582302311
ENSG00000198522	*GPN1*	2.309473412	-0.002403668	-0.006363652	0.003959984	0.999933887	0.117642731	0.003340824	0.114301908	0.582302311
ENSG00000164252	*AGGF1*	1.712603369	-0.089300975	-0.017221062	-0.072079913	0.999933887	-0.449277669	-0.584609217	0.135331548	0.582477354
ENSG00000281106	*TMEM272*	1.046113844	-0.029538063	0.020843346	-0.050381409	0.999933887	0.401445231	0.657196164	-0.255750933	0.582737047
ENSG00000181472	*ZBTB2*	4.581132961	-0.056450619	-0.028724372	-0.027726247	0.999933887	-0.219315136	-0.290875628	0.071560492	0.583129267
ENSG00000189043	*NDUFA4*	3.657612803	0.008476453	-0.098660807	0.10713726	0.999933887	0.021186143	-0.073528666	0.094714809	0.58317721
ENSG00000131591	*C1orf159*	2.14376013	0.056750316	0.086240615	-0.0294903	0.999933887	0.157351262	0.035077644	0.122273618	0.583228584
ENSG00000231439	*WASIR2*	-1.257444036	-0.322669344	-0.334173768	0.011504424	0.999933887	-1.092774771	-1.340617095	0.247842323	0.583420478
ENSG00000175003	*SLC22A1*	3.021901341	-0.080480741	-0.00505635	-0.07542439	0.999933887	0.483918254	0.600263679	-0.116345425	0.583608435
ENSG00000148339	*SLC25A25*	3.542883019	0.050562273	0.126403737	-0.075841464	0.999933887	-0.356606453	-0.467653182	0.111046729	0.583608435
ENSG00000211706	*TRBV6-1*	-0.375496737	-0.0874024	-0.052602721	-0.034799679	0.999933887	0.240894666	-0.020909398	0.261804065	0.583688625
ENSG00000073712	*FERMT2*	-1.694810052	-0.457480077	0.433840676	-0.891320754	0.999933887	0.424628841	0.642371742	-0.217742901	0.583758949
ENSG00000164707	*SLC13A4*	-0.816564434	-0.122927851	0.085055311	-0.207983162	0.999933887	-0.108612644	-0.354272119	0.245659476	0.583758949
ENSG00000268658	*LINC00664*	5.631655716	0.028169109	0.104718513	-0.076549405	0.999933887	0.999573339	1.139545157	-0.139971819	0.583941304
ENSG00000105270	*CLIP3*	-0.386028938	-0.025768939	0.185093391	-0.210862331	0.999933887	-0.081936613	-0.371751036	0.289814424	0.58471721
ENSG00000277161	*PIGW*	0.028948096	-0.243080904	-0.133481236	-0.109599668	0.999933887	0.145790892	0.386902963	-0.241112072	0.584794259
ENSG00000219481	*NBPF1*	1.163070071	0.09923961	-0.064895858	0.164135467	0.999933887	0.031089906	-0.138652954	0.16974286	0.584794259
ENSG00000120162	*MOB3B*	-0.282313615	0.230072496	-0.296893429	0.526965924	0.944094537	-0.0189062	0.180290419	-0.199196618	0.584941044
ENSG00000204252	*HLA-DOA*	1.296922041	0.067684901	0.023153677	0.044531224	0.999933887	0.109177636	0.271596327	-0.162418691	0.584971936
ENSG00000188846	*RPL14*	7.395381656	-0.002659006	-0.109130691	0.106471685	0.999933887	0.080127264	0.158984976	-0.078857713	0.585026451
ENSG00000256751	*PLBD1-AS1*	0.265265136	0.145866942	-0.061491643	0.207358585	0.999933887	-0.443520962	-0.786175481	0.342654519	0.585028334
ENSG00000062282	*DGAT2*	7.329642842	-0.000989285	0.031390898	-0.032380184	0.999933887	-0.081018311	-0.155491136	0.074472826	0.585433792
ENSG00000152795	*HNRNPDL*	6.835844828	-0.063799369	-0.053094038	-0.010705331	0.999933887	-0.108952963	-0.166592915	0.057639952	0.585523609
ENSG00000167785	*ZNF558*	1.845618071	-0.074462666	-0.149585167	0.075122501	0.999933887	-0.115471247	0.009891112	-0.125362359	0.585523609
ENSG00000139624	*CERS5*	4.839184203	-0.028952093	-0.004817467	-0.024134626	0.999933887	-0.226760785	-0.160361874	-0.066398911	0.585523609

ENSG00000168297	*PXK*	5.203125648	-0.031693283	-0.042517288	0.010824005	0.999933887	-0.469030635	-0.549398385	0.08036775	0.585523609
ENSG00000241769	*LINC00893*	0.067736479	-0.031279002	0.085434652	-0.116713654	0.999933887	-0.359809738	-0.597507761	0.237698024	0.585651699
ENSG00000168140	*VASN*	2.333277998	0.036424287	0.035074737	0.00134955	0.999933887	-0.118943408	-0.265769232	0.146825824	0.585923954
ENSG00000105609	*LILRB5*	2.28336474	0.073882627	0.047284173	0.026598454	0.999933887	-0.233738728	-0.337872046	0.104133318	0.586124687
ENSG00000176700	*SCAND2P*	0.051291707	-0.094600837	0.104427022	-0.199027859	0.999933887	-0.191326009	-0.393073907	0.201747897	0.586124687
ENSG00000139835	*GRTP1*	2.071732103	0.143515411	0.297607947	-0.154092536	0.999933887	0.504864234	0.647311712	-0.142447478	0.586511758
ENSG00000104804	*TULP2*	2.250652269	0.427318646	0.509005096	-0.08168645	0.999933887	1.938339188	2.213893793	-0.275554604	0.586782905
ENSG00000120949	*TNFRSF8*	4.79601977	0.12147477	0.110656753	0.010818016	0.999933887	0.252100569	0.346219343	-0.094118774	0.586782905
ENSG00000131171	*SH3BGRL*	5.673229765	-0.053193666	-0.164076285	0.110882619	0.999933887	-0.086251647	0.004477097	-0.090728744	0.586782905
ENSG00000218713	*AL512378.1*	0.768009052	-0.041378701	-0.082869977	0.041491276	0.999933887	-1.00386846	-0.808352959	-0.1955155	0.586841369
ENSG00000143457	*GOLPH3L*	1.693722613	-0.080366315	-0.136607395	0.05624108	0.999933887	-0.042577614	0.149721494	-0.192299108	0.586914344
ENSG00000254681	*PKD1P5*	-2.252094938	0.034287296	-0.01871349	0.053000786	0.999933887	0.122819264	-0.092088077	0.214907341	0.586914344
ENSG00000257698	*GIHCG*	1.286227457	-0.016980309	0.022733299	-0.039713608	0.999933887	-0.30529638	-0.454111153	0.148814774	0.586914344
ENSG00000245275	*SAP30L-AS1*	1.750347536	-0.037049313	-0.007322525	-0.029726787	0.999933887	-0.230130831	-0.381007136	0.150876306	0.587173032
ENSG00000286760	*AL022069.3*	0.785373048	0.264758856	0.238103147	0.026655709	0.999933887	0.937685493	1.130139786	-0.192454293	0.587228803
ENSG00000010165	*EEF1AKNMT*	2.480061682	0.055875221	0.11002263	-0.054147409	0.999933887	-0.149995148	-0.243837612	0.093842464	0.587228803
ENSG00000176087	*SLC35A4*	5.295151186	-0.001514362	-0.050441419	0.048927058	0.999933887	-0.04287281	0.027029486	-0.069902295	0.587228803
ENSG00000130748	*TMEM160*	1.591836276	0.001861986	0.16794135	-0.166079364	0.999933887	0.116835372	-0.065493359	0.182328731	0.587228803
ENSG00000131187	*F12*	2.925481772	0.168569482	-0.03346113	0.202030612	0.999933887	0.691088124	0.817178143	-0.126090019	0.587636607
ENSG00000114331	*ACAP2*	7.110820453	-0.074157619	-0.108538461	0.034380842	0.999933887	-0.603103606	-0.49201592	-0.111087685	0.587636607
ENSG00000083093	*PALB2*	0.371526063	-0.126636877	-0.028196621	-0.098440256	0.999933887	0.081549914	0.250869717	-0.169319803	0.587636607
ENSG00000163320	*CGGBP1*	5.870224576	-0.034054914	-0.028779182	-0.005275732	0.999933887	-0.249581088	-0.16992987	-0.079651218	0.587636607
ENSG00000122417	*ODF2L*	3.298392178	-0.009966907	-0.156269743	0.146302836	0.999933887	-0.271393287	-0.045466573	-0.225926714	0.587693529
ENSG00000271216	*LINC01050*	-0.569471066	-0.11660224	0.609217811	-0.72582005	0.999933887	1.04220912	1.216457886	-0.174248766	0.587781173
ENSG00000111879	*FAM184A*	-1.14782415	-0.123646796	-0.278474707	0.154827912	0.999933887	-0.348083034	0.01465724	-0.362740274	0.587796196
ENSG00000104517	*UBR5*	7.316523891	-0.057725401	-0.089875726	0.032150325	0.999933887	-0.097679566	-0.006201082	-0.091478484	0.587962552
ENSG00000169241	*SLC50A1*	2.811719373	-0.028872446	0.013229062	-0.042101508	0.999933887	0.728518027	0.852883194	-0.124365168	0.587985787
ENSG00000137218	*FRS3*	-0.086234459	0.123798318	-0.074247398	0.198045717	0.999933887	0.093923131	-0.114041365	0.207964496	0.587999825
ENSG00000204463	*BAG6*	8.100092869	0.057828936	0.039383275	0.01844566	0.999933887	0.260803933	0.316964708	-0.056160775	0.588257129
ENSG00000107902	*LHPP*	1.880383592	-0.07198457	0.05081658	-0.12280115	0.999933887	0.003951155	-0.170325269	0.174276424	0.588257129
ENSG00000108578	*BLMH*	2.535739642	0.007644752	0.090137897	-0.082493146	0.999933887	0.088555329	0.180180314	-0.091624986	0.588257129
ENSG00000279500	*AC108704.2*	4.566801656	-0.075415622	-0.034504179	-0.040911443	0.999933887	-0.257304993	-0.161612408	-0.095692584	0.588270813
ENSG00000161914	*ZNF653*	0.990237078	-0.056527928	0.041853499	-0.098381427	0.999933887	-0.020400896	-0.189263549	0.168862653	0.588304796
ENSG00000177917	*ARL6IP6*	2.831455889	0.115367867	0.062044309	0.053323558	0.999933887	-0.184904485	-0.314121724	0.129217238	0.588415871
ENSG00000164687	*FABP5*	0.810708396	-0.033549759	-0.122861324	0.089311565	0.999933887	-0.250962374	-0.453858737	0.202896363	0.588415871
ENSG00000100412	*ACO2*	4.076218217	-0.001026561	-0.009304162	0.0082776	0.999933887	0.006751188	-0.069586975	0.076338163	0.588785757
ENSG00000272914	*AL359532.1*	1.409280284	-0.058881979	-0.068098193	0.009216214	0.999933887	-0.154349681	-0.025297472	-0.129052209	0.588943882
ENSG00000286018	*AF129075.3*	-0.425452151	0.221040949	0.105436481	0.115604468	0.999933887	-0.090430808	0.161946121	-0.252376929	0.588996124
ENSG00000225518	*LINC01703*	-0.00757587	0.039251436	0.26415204	-0.224900604	0.999933887	0.441738866	0.029029033	0.412709833	0.589340504
ENSG00000173209	*AHSA2P*	5.334986223	0.000940589	-0.033032202	0.03397279	0.999933887	-0.065876336	-0.132869482	0.066993145	0.589408603
ENSG00000204054	*LINC00963*	7.156335847	-0.008836231	-0.002407821	-0.006428411	0.999933887	0.114038269	0.196539094	-0.082500825	0.589494495
ENSG00000177733	*HNRNPA0*	6.854885657	0.085149871	0.061948172	0.0232017	0.999933887	-0.019512575	-0.112502639	0.092990064	0.589584797
ENSG00000103037	*SETD6*	1.681384603	0.100872176	0.046163015	0.054709161	0.999933887	0.2606662	0.138716571	0.121949629	0.589584797
ENSG00000117322	*CR2*	-0.37873525	-0.180329347	0.159711397	-0.340040744	0.999933887	0.333235273	0.154460131	0.178775142	0.589584797
ENSG00000109220	*CHIC2*	5.422643243	0.041563828	0.098192599	-0.056628771	0.999933887	-0.127485095	-0.054545829	-0.072939266	0.589584797
ENSG00000180370	*PAK2*	7.744100962	-0.045104731	0.019499266	-0.064603997	0.999933887	-0.692140588	-0.778601602	0.086461014	0.589584797
ENSG00000118096	*IFT46*	0.547786777	0.051873045	0.057410158	-0.005537113	0.999933887	0.14057121	0.311709667	-0.171138457	0.589584797
ENSG00000114520	*SNX4*	1.844105378	-0.059435322	-0.119958139	0.060522817	0.999933887	-0.142331137	-0.291991641	0.149660504	0.589642963
ENSG00000135749	*PCNX2*	3.312794618	-0.019148771	0.004789492	-0.023938264	0.999933887	-0.068366427	0.02420987	-0.092576297	0.590311091
ENSG00000101695	*RNF125*	3.841141886	-0.072149315	-0.109595083	0.037445769	0.999933887	-0.088085664	-0.202839448	0.114753784	0.590441122
ENSG00000270972	*AC136475.9*	-1.293120329	-0.060552879	-0.182477596	0.121924718	0.999933887	0.040703095	-0.392930752	0.433633847	0.590523733
ENSG00000106089	*STX1A*	1.727659734	-0.105547602	0.092821962	-0.198369564	0.999933887	0.006187366	-0.161152043	0.167339409	0.59056313
ENSG00000138468	*SENP7*	4.473055003	-0.022899996	-0.133105182	0.110205186	0.999933887	-0.565786394	-0.313725253	-0.252061141	0.59056313
ENSG00000198039	*ZNF273*	0.759727734	0.016035755	0.039694641	-0.023658886	0.999933887	-0.564188491	-0.352675955	-0.211512536	0.59056313
ENSG00000121310	*ECHDC2*	3.072683862	-0.081576529	0.085947911	-0.167524441	0.999933887	-0.002947087	0.101553587	-0.104500674	0.590620513
ENSG00000119013	*NDUFB3*	3.595173947	-0.053640245	-0.028258111	-0.025382134	0.999933887	0.056953501	-0.042786639	0.099740141	0.590872516
ENSG00000119725	*ZNF410*	0.512364498	0.045136307	0.116875433	-0.071739126	0.999933887	0.320723368	0.520373938	-0.19965057	0.591015107
ENSG00000274213	*AC015912.3*	5.296030542	0.125879503	0.073716339	0.052163164	0.999933887	0.191392539	0.065379662	0.126012877	0.591056053
ENSG00000211829	*TRDC*	0.314651199	-0.090186051	-0.053978429	-0.036207622	0.999933887	0.009181652	0.179311007	-0.170129355	0.591056053
ENSG00000116016	*EPAS1*	2.806557642	-0.057215913	0.045636785	-0.102852698	0.999933887	-0.442206836	-0.345677186	-0.09652965	0.591357946
ENSG00000104356	*POP1*	-0.062606557	0.093939994	0.292240909	-0.198300914	0.999933887	0.346930289	0.137977522	0.208952767	0.591357946
ENSG00000114857	*NKTR*	6.447268965	-0.048310218	-0.195455003	0.147144784	0.999933887	-0.516747945	-0.272162579	-0.244585366	0.591357946
ENSG00000136732	*GYPC*	4.490347692	0.035219347	-0.033578962	0.068798309	0.999933887	0.085637702	-0.001449	0.087086702	0.591399284
ENSG00000187792	*ZNF70*	0.005227895	-0.003551289	-0.055435993	0.051884705	0.999933887	-0.010534145	0.22372274	-0.234256885	0.591453868
ENSG00000044574	*HSPA5*	8.280148799	0.0406526	-0.033691021	0.07434362	0.999933887	0.31174396	0.384099626	-0.072355666	0.59184642
ENSG00000214688	*C10orf105*	0.233847568	0.055598936	0.051587122	0.004011814	0.999933887	-0.292118065	-0.661184665	0.3690666	0.592016903
ENSG00000162836	*ACP6*	6.307476007	0.035940388	0.120375049	-0.084434661	0.999933887	-0.401535682	-0.494480869	0.092945186	0.592085039
ENSG00000124785	*NRN1*	-0.292426849	-0.161720656	0.186110925	-0.34783158	0.958987651	-0.442136182	-0.142800842	-0.29933534	0.592431423
ENSG00000173262	*SLC2A14*	0.096918458	-0.018947859	0.096010148	-0.114958008	0.999933887	-0.619303335	-0.387368752	-0.231934583	0.592431423
ENSG00000272033	*AL136984.1*	0.166785766	-0.040110695	0.089540153	-0.129650847	0.999933887	-0.458049355	-0.174708854	-0.283340501	0.59243454
ENSG00000153107	*ANAPC1*	2.201053379	-0.088509333	-0.110020686	0.021511353	0.999933887	0.134973762	0.242106849	-0.107133087	0.592936235
ENSG00000055732	*MCOLN3*	-0.838114487	0.143154537	-0.104941647	0.248096184	0.999933887	-0.163938935	0.127863802	-0.291802736	0.592949119
ENSG00000163935	*SFMBT1*	2.16812168	-0.052493864	0.075224458	-0.127718321	0.999933887	0.090560247	0.203629259	-0.113069012	0.592949119
ENSG00000227097	*RPS28P7*	-0.100819316	0.11499697	0.152160646	-0.037163676	0.999933887	-0.031701388	0.252102468	-0.283803856	0.592949119
ENSG00000251648	*AC008958.1*	0.138769411	0.125610813	0.287472086	-0.161861273	0.999933887	1.783615489	1.554643589	0.2289719	0.592949119
ENSG00000123636	*BAZ2B*	6.110643139	-0.039578565	-0.127994357	0.088415791	0.999933887	-0.749256221	-0.563002755	-0.186253466	0.592949119
ENSG00000177932	*ZNF354C*	0.194897448	-0.04611046	0.152143299	-0.198253759	0.999933887	0.02926761	0.238855042	-0.209587432	0.592949119
ENSG00000178878	*APOLD1*	0.686164632	0.021690232	0.023295564	-0.001605332	0.999933887	-0.418161026	-0.620124135	0.201963109	0.592949119
ENSG00000232587	*EEF1A1P3*	0.410023159	0.138281386	0.312232674	-0.173951288	0.999933887	-0.29507925	-0.034624735	-0.260454516	0.59311888
ENSG00000104814	*MAP4K1*	4.13084555	-0.012483753	0.024446023	-0.036929776	0.999933887	0.112175465	0.035419038	0.076756427	0.59311888
ENSG00000203709	*MIR29B2CHG*	7.947146887	0.047073287	0.001436171	0.045637116	0.999933887	-0.139553552	-0.063742218	-0.075811334	0.593190538
ENSG00000184347	*SLIT3*	-1.069008382	-0.224645969	-0.17100729	-0.053638678	0.999933887	-0.012748648	0.186280297	-0.199028945	0.593190538
ENSG00000272368	*AC074032.1*	-0.199040802	0.087102875	0.047632034	0.039470841	0.999933887	-0.052987695	-0.333246919	0.280259224	0.593577758
ENSG00000015171	*ZMYND11*	3.402874397	-0.014131743	0.045967872	-0.060099615	0.999933887	-0.01611835	0.074160898	-0.090279252	0.593727964
ENSG00000105185	*PDCD5*	2.09891753	-0.016424347	-0.103675096	0.087250748	0.999933887	0.165463276	0.281171401	-0.115708125	0.593727964
ENSG00000136158	*SPRY2*	2.706876355	-0.168094838	-0.04250072	-0.125594118	0.999933887	-0.545437306	-0.42396307	-0.121474236	0.594184951
ENSG00000273820	*USP27X*	0.504854671	-0.119839756	-0.002156504	-0.117683252	0.999933887	0.130736178	-0.026221857	0.156958036	0.594317934
ENSG00000275395	*FCGBP*	2.300204805	0.017312541	0.126003384	-0.108690843	0.999933887	0.312901377	0.412217114	-0.099315737	0.594647693
ENSG00000057663	*ATG5*	3.464965841	-0.148144896	0.012768063	-0.160912959	0.999933887	-0.020214274	0.059820897	-0.080035171	0.59484237
ENSG00000130167	*TSPAN16*	-0.084171647	-0.078820877	0.153199288	-0.232020164	0.999933887	-0.706098363	-1.047181713	0.34108335	0.59484237
ENSG00000101843	*PSMD10*	2.34696848	0.043383482	-0.118717665	0.162101147	0.999933887	-0.138430582	-0.024141037	-0.114289545	0.59484237
ENSG00000109046	*WSB1*	8.364977874	-0.028240345	0.025202232	-0.053442577	0.999933887	0.293482869	0.374138775	-0.080655906	0.594916201
ENSG00000221866	*PLXNA4*	-0.74653649	-0.017771792	0.073329518	-0.09110131	0.999933887	-0.132462669	0.190707118	-0.323169787	0.594916201
ENSG00000218809	*AL391903.1*	4.181776126	0.172248842	0.154230695	0.018018147	0.999933887	0.608705002	0.752458078	-0.143753076	0.594927061
ENSG00000164967	*RPP25L*	2.319663676	-0.000493772	-0.146871397	0.146377626	0.999933887	-0.131275062	-0.228506604	0.097231542	0.595321328
ENSG00000137154	*RPS6*	8.403367959	-0.028402557	-0.133598216	0.105195658	0.999933887	0.066141479	0.137777615	-0.071636137	0.595436617
ENSG00000186106	*ANKRD46*	0.656276162	0.009264209	0.079068121	-0.069803912	0.999933887	-0.12792576	0.037296279	-0.165222039	0.59587901
ENSG00000145217	*SLC26A1*	-0.204584828	-0.156188204	0.017158844	-0.173347047	0.999933887	0.099120295	-0.103351436	0.202471731	0.596214916
ENSG00000126803	*HSPA2*	1.07272921	-0.030409632	0.113485293	-0.143894925	0.999933887	0.524054442	0.334300411	0.189754031	0.596214916
ENSG00000167315	*ACAA2*	1.703365005	-0.021122462	-0.024819862	0.0036974	0.999933887	-0.241424845	-0.10312887	-0.138295975	0.596967301
ENSG00000130338	*TULP4*	3.142723608	-0.049536167	0.116379523	-0.16591569	0.999933887	-0.245845611	-0.328362798	0.082517186	0.597050859
ENSG00000186635	*ARAP1*	10.204659464	0.014293234	0.037300342	-0.023007108	0.999933887	0.025905817	-0.034327447	0.060233264	0.597171018
ENSG00000260805	*AC092803.1*	2.8315967	0.208728217	0.144377718	0.0643505	0.999933887	0.973949157	1.113255504	-0.139306347	0.597588826
ENSG00000264931	*MIR3138*	-1.630930885	0.31626762	0.629127595	-0.312859975	0.999933887	-0.07050854	0.464903379	-0.535411919	0.597662867
ENSG00000279811	*AC093330.2*	1.642152321	0.144816457	-0.041987395	0.186803852	0.999933887	-0.193086795	-0.038601804	-0.154484991	0.597662867

ENSG00000229619	*MBNL1-AS1*	0.584555836	-0.103872184	0.007375741	-0.111247924	0.999933887	0.111504863	-0.16220289	0.273707753	0.597662867
ENSG00000132763	*MMACHC*	-0.389583511	-0.408938396	-0.223641489	-0.185296906	0.999933887	0.329281236	0.551637746	-0.22235651	0.597806778
ENSG00000206013	*IFITM5*	0.043673938	0.278066364	0.066646844	0.21141952	0.999933887	0.171967391	-0.082208586	0.254175977	0.597827103
ENSG00000113360	*DROSHA*	2.468498638	0.058020862	-0.025639872	0.083660733	0.999933887	-0.082108017	0.013950572	-0.09605859	0.597827103
ENSG00000162714	*ZNF496*	2.499821597	-0.081649589	0.104575724	-0.186225313	0.999933887	0.095767652	-0.015781615	0.111549267	0.597989847
ENSG00000114473	*IQCG*	1.078045364	0.090785263	0.007935709	0.082849554	0.999933887	0.391312986	0.540686341	-0.149373355	0.597989847
ENSG00000239213	*NCK1-DT*	-0.175374478	-0.056546771	0.041723213	-0.098269984	0.999933887	-0.209136733	-0.436061734	0.226925001	0.597989847
ENSG00000129682	*FGF13*	2.542207022	0.168967604	0.306507753	-0.137540149	0.999933887	0.323628768	0.158444728	0.16518404	0.598123738
ENSG00000122406	*RPL5*	7.118425146	-0.032220479	-0.147972158	0.115751679	0.999933887	0.091654549	0.162810881	-0.071156332	0.598123738
ENSG00000269858	*EGLN2*	5.671420359	0.01197041	0.012126129	-0.000155719	0.999933887	0.011996593	0.083787962	-0.071791368	0.598123738
ENSG00000260942	*CAPN10-DT*	1.499479003	-0.259081527	-0.18990305	-0.069178477	0.999933887	-0.007918699	-0.192521202	0.184602503	0.598163741
ENSG00000182872	*RBM10*	5.336057943	0.067196224	0.041781505	0.025414719	0.999933887	-0.001710483	-0.060733438	0.059022955	0.598163741
ENSG00000091947	*TMEM101*	1.717714665	-0.110406169	-0.18597119	0.075565021	0.999933887	-0.300990718	-0.444288046	0.143297328	0.598163741
ENSG00000131634	*TMEM204*	1.941605226	0.028855758	0.011090311	0.017765447	0.999933887	0.07163282	-0.048603564	0.120236384	0.598163741
ENSG00000227920	*AL353597.1*	-1.41249047	0.040217228	0.506697488	-0.466480261	0.999933887	0.232006627	-0.063658908	0.295665536	0.598163741
ENSG00000137944	*KYAT3*	3.426163716	-0.134234221	-0.027957018	-0.106277203	0.999933887	-0.021432578	0.057504106	-0.078936684	0.5983948
ENSG00000267174	*AC011472.2*	0.869705872	0.126039404	0.092788218	0.033251186	0.999933887	-0.220881804	-0.46073367	0.239851866	0.598812188
ENSG00000113391	*FAM172A*	3.511696195	-0.015315673	0.034713051	-0.050028724	0.999933887	-0.092167228	0.000988526	-0.093155754	0.598812188
ENSG00000182544	*MFSD5*	4.138942983	-0.01115666	-0.080593636	0.069436976	0.999933887	-0.142336027	-0.052662423	-0.089673604	0.598812188
ENSG00000100614	*PPM1A*	6.764061706	-0.044798165	-0.043612591	-0.001185574	0.999933887	-0.518499842	-0.446473477	-0.072026366	0.598818577
ENSG00000176624	*MEX3C*	4.360364877	0.03800033	0.028483593	0.009516738	0.999933887	-0.12318459	-0.201290749	0.078106159	0.598818577
ENSG00000137103	*TMEM8B*	0.601017758	-0.067589983	-0.0896321	0.022042118	0.999933887	-0.048920276	0.131866514	-0.18078679	0.598818577
ENSG00000123064	*DDX54*	3.507821471	0.041289521	0.13721957	-0.09593005	0.999933887	0.128929847	0.219923273	-0.090993426	0.599117516
ENSG00000086062	*B4GALT1*	7.418624243	-0.050656993	-0.007320866	-0.043336126	0.999933887	0.944170985	1.04321597	-0.099044985	0.599293241
ENSG00000269952	*AL117336.1*	1.096836808	0.046708264	0.2012059	-0.154497636	0.999933887	0.326267526	0.570811745	-0.244544219	0.599420618
ENSG00000258571	*PTTG4P*	-0.064779886	-0.016297793	-0.09000194	0.073704147	0.999933887	-0.608796478	-0.26131363	-0.347482848	0.599420618
ENSG00000157483	*MYO1E*	0.348635495	-0.065066758	0.116188537	-0.181255295	0.999933887	-0.361203985	-0.201598251	-0.159605734	0.599434099
ENSG00000106701	*FSD1L*	-0.418197812	-0.053889794	0.021212775	-0.075102569	0.999933887	-0.680178198	-0.450814275	-0.229363922	0.599434099
ENSG00000178814	*OPLAH*	3.61464313	0.026513328	0.086469073	-0.059955745	0.999933887	0.201783938	0.101056395	0.100727543	0.599436077
ENSG00000171150	*SOCS5*	2.686904781	0.051066085	-0.153466266	0.204532351	0.999933887	-0.216526565	-0.328598583	0.112072018	0.599675964
ENSG00000225611	*LINC02158*	2.523892535	0.032251131	0.10678318	-0.074532049	0.999933887	0.620526528	0.739917854	-0.119391326	0.599869649
ENSG00000157637	*SLC38A10*	5.685670794	0.020992544	-0.022993743	0.043986288	0.999933887	-0.079184617	-0.158579689	0.079395072	0.600041958
ENSG00000151779	*NBAS*	2.584871037	-0.004018268	-0.055818114	0.051799846	0.999933887	0.083284235	0.224975352	-0.141691117	0.600236095
ENSG00000241560	*ZBTB20-AS1*	-0.978201944	-0.023464632	0.373284259	-0.396748891	0.999933887	-0.010800168	0.444946877	-0.455747044	0.600433487
ENSG00000229781	*RRN3P4*	-1.298832669	0.212641431	0.003669417	0.208972014	0.999933887	1.859449032	2.212688987	-0.353239955	0.600525512
ENSG00000153044	*CENPH*	-0.732540254	-0.076575974	-0.007754934	-0.06882104	0.999933887	0.084252154	0.336195302	-0.251943148	0.600559409
ENSG00000196735	*HLA-DQA1*	4.230833441	0.026846592	0.096598947	-0.069752354	0.999933887	1.671943346	1.928487497	-0.256544151	0.600588304
ENSG00000177954	*RPS27*	8.428246257	0.027468648	-0.024055591	0.051524239	0.999933887	0.112770562	0.192457517	-0.079686954	0.600731387
ENSG00000108582	*CPD*	10.062907268	-0.083857243	-0.079786191	-0.004071052	0.999933887	0.082153425	0.167206586	-0.085053161	0.600959556
ENSG00000249502	*AC006160.1*	1.487007232	0.104280966	-0.038024636	0.142305602	0.999933887	0.130816041	0.283165015	-0.152348975	0.600959556
ENSG00000231993	*EP300-AS1*	0.748929536	-0.02294896	0.147544912	-0.170493871	0.999933887	-0.184299566	0.04093288	-0.225232446	0.600959556
ENSG00000254531	*FLJ20021*	-0.110324925	0.070194984	0.241488331	-0.171293346	0.999933887	-0.143957381	-0.378350542	0.234393162	0.601053096
ENSG00000100099	*HPS4*	4.900740224	-0.078067841	0.014922704	-0.092990545	0.999933887	-0.204294774	-0.141470798	-0.062823975	0.601199109
ENSG00000242802	*AP5Z1*	8.233450047	0.070761228	0.096950088	-0.02618886	0.999933887	0.86472608	0.954269869	-0.089543789	0.601199109
ENSG00000139354	*GAS2L3*	0.71033557	-0.183935908	0.240158603	-0.424094511	0.999933887	-0.517797451	-0.359388672	-0.158408778	0.601199109
ENSG00000246263	*UBR5-AS1*	4.260375897	-0.005983799	-0.086763119	0.08077932	0.999933887	-0.471421311	-0.379396012	-0.092025299	0.601876191
ENSG00000145425	*RPS3A*	6.664855219	-0.070684182	-0.162305057	0.091620875	0.999933887	0.076518976	0.14472578	-0.068206804	0.601944598
ENSG00000174306	*ZHX3*	0.746015313	0.047238421	0.096565742	-0.049327322	0.999933887	-0.108624658	-0.292749217	0.18412456	0.601944598
ENSG00000184182	*UBE2F*	1.898254286	0.14346096	0.072355616	0.071105344	0.999933887	-0.022608382	0.107417169	-0.130025552	0.6020543
ENSG00000134531	*EMP1*	3.358989355	-0.186530799	-0.123947126	-0.062583673	0.999933887	-1.520181861	-1.660124379	0.139942519	0.6020543
ENSG00000090006	*LTBP4*	4.246233589	0.019625761	0.05545757	-0.035831809	0.999933887	0.091965485	0.006361798	0.085603687	0.602070109
ENSG00000280347	*AC000123.3*	1.62790738	-0.021409027	0.235953456	-0.257362484	0.999933887	-0.35192353	-0.07970033	-0.272223199	0.602070109
ENSG00000168393	*DTYMK*	0.91678955	0.242406828	-0.187743747	0.430150575	0.944094537	0.123970241	-0.056111057	0.180081298	0.602318429
ENSG00000259556	*AC090971.3*	0.649061994	-0.057796995	-0.179404803	0.121607808	0.999933887	0.359365129	0.575423352	-0.216058224	0.602318429
ENSG00000142544	*CTU1*	0.952343729	0.01515744	0.227852771	-0.21269533	0.999933887	0.005442911	-0.143458035	0.148900946	0.602330299
ENSG00000213434	*VTI1BP2*	-2.983975257	0.277834989	0.34715157	-0.069316581	0.999933887	2.053479364	2.32383876	-0.270359396	0.602401185
ENSG00000229106	*BTBD6P1*	0.856262109	-0.246698984	-0.016035535	-0.230663449	0.999933887	-0.113909945	0.039879678	-0.153789622	0.60296518
ENSG00000005059	*MCUB*	3.437798905	0.019319702	-0.06547222	0.084791922	0.999933887	0.037216937	0.119382704	-0.082165767	0.603202521
ENSG00000153561	*RMND5A*	7.642754499	-0.106432196	-0.017045072	-0.089387124	0.999933887	-0.754985601	-0.831618126	0.076632525	0.603374504
ENSG00000187555	*USP7*	7.026176869	-0.038148175	-0.033664887	-0.004483288	0.999933887	0.041221716	0.088590086	-0.04736837	0.603374504
ENSG00000243402	*AC022973.2*	0.016946114	0.068197409	0.271200941	-0.203003531	0.999933887	-0.138938583	0.194993646	-0.333932229	0.60375267
ENSG00000044115	*CTNNA1*	4.800197668	-0.039433592	-0.05682627	0.017392678	0.999933887	-0.189305027	-0.119065732	-0.070239295	0.604037737
ENSG00000204899	*MZT1*	1.084590691	0.027016108	0.000200979	0.026815129	0.999933887	-0.140452255	0.018573434	-0.159025689	0.604253838
ENSG00000255026	*AC136475.3*	4.926351505	0.108362443	0.131211437	-0.022848995	0.999933887	-0.233299837	-0.330196813	0.096896976	0.604265952
ENSG00000167193	*CRK*	6.325955665	-0.027636821	-0.110737392	0.083100572	0.999933887	-0.160585372	-0.106167532	-0.054417841	0.604388135
ENSG00000005020	*SKAP2*	6.663552152	0.022635021	-0.040658064	0.063293085	0.999933887	-0.769771694	-0.868438657	0.098666964	0.604407591
ENSG00000111335	*OAS2*	4.574813922	-0.018531954	-0.032766881	0.014234928	0.999933887	-0.265009373	-0.348727652	0.083718279	0.604407591
ENSG00000116285	*ERRFI1*	0.474112318	0.036037651	0.099726376	-0.063688726	0.999933887	-0.403910198	-0.649258981	0.245348783	0.604407591
ENSG00000139546	*TARBP2*	1.963220239	0.003029257	0.060650213	-0.057620956	0.999933887	-0.279072586	-0.410779238	0.131706652	0.604407591
ENSG00000134198	*TSPAN2*	3.968371707	-0.036840103	-0.102515161	0.065675058	0.999933887	0.010766843	0.119338305	-0.108571462	0.604796361
ENSG00000115365	*LANCL1*	2.256438385	-0.113351013	-0.070145313	-0.0432057	0.999933887	-0.016862819	0.100585888	-0.117448707	0.604950325
ENSG00000013573	*DDX11*	1.532513544	-0.095212808	0.319853827	-0.415066635	0.594252256	0.137496959	0.022902097	0.114594862	0.604950325
ENSG00000166452	*AKIP1*	0.297615756	0.102014131	-0.277408098	0.379422229	0.999933887	0.166103608	0.000383955	0.165719653	0.604950325
ENSG00000162434	*JAK1*	8.679477478	-0.023797925	-0.058863455	0.03506553	0.999933887	0.179144587	0.230621337	-0.05147675	0.604950325
ENSG00000258311	*AC009779.3*	1.982043022	0.152916282	0.001973036	0.150943246	0.999933887	0.037973064	0.168644257	-0.130671193	0.60501947
ENSG00000184611	*KCNH7*	-0.550932213	-0.129822237	0.153858771	-0.283681008	0.999933887	-0.286219817	-0.625934582	0.339714765	0.60501947
ENSG00000108786	*HSD17B1*	0.098624168	-0.147614716	-0.091146086	-0.05646863	0.999933887	-0.640869047	-0.918908338	0.27803929	0.605355737
ENSG00000100522	*GNPNAT1*	0.043599813	-0.100036841	0.133080704	-0.233117545	0.999933887	0.102034547	0.285020781	-0.182986234	0.605355737
ENSG00000170049	*KCNAB3*	1.849915153	0.021527876	-0.160656757	0.182184634	0.999933887	0.173711533	0.032734899	0.140976633	0.605355737
ENSG00000258082	*AL391832.3*	2.883709566	0.172704157	0.117968347	0.054735811	0.999933887	1.88420727	2.046028223	-0.161820953	0.605619432
ENSG00000074842	*MYDGF*	2.954263621	0.12772504	-0.08203047	0.20975551	0.999933887	0.12868113	0.215424184	-0.086743054	0.605619432
ENSG00000059769	*DNAJC25*	1.566975816	-0.12193588	-0.08807922	-0.03385666	0.999933887	0.139386034	0.324023923	-0.184637889	0.605683536
ENSG00000255310	*AF131215.5*	-0.264989721	-0.052304698	-0.060720587	0.008415888	0.999933887	0.090365462	0.338593915	-0.248228453	0.605683536
ENSG00000188321	*ZNF559*	1.249480593	-0.023430132	-0.157400417	0.133970284	0.999933887	-0.31073965	-0.162947932	-0.147791718	0.605707001
ENSG00000156931	*VPS8*	6.76981894	-0.086361677	-0.089626182	0.003264505	0.999933887	-1.079927945	-1.171939195	0.09201125	0.605742635
ENSG00000146757	*ZNF92*	1.47437848	-0.195175777	-0.290407954	0.095232177	0.999933887	-0.448474794	-0.241729107	-0.206745687	0.606158547
ENSG00000180900	*SCRIB*	3.806559034	0.0717835	0.052885334	0.018898167	0.999933887	0.070081563	-0.030961295	0.101042858	0.606158547
ENSG00000130725	*UBE2M*	6.00083289	0.105014909	0.123227049	-0.018212141	0.999933887	-0.315417072	-0.396662641	0.081245569	0.606747961
ENSG00000146872	*TLK2*	5.059086804	-0.064532947	0.025933752	-0.090466699	0.999933887	-0.247308627	-0.186355328	-0.060953298	0.606785865
ENSG00000169710	*FASN*	4.38111666	0.072656535	0.103434436	-0.0307779	0.999933887	0.202555933	0.286780115	-0.084224183	0.606785865
ENSG00000178425	*NT5DC1*	2.011562923	0.077142461	-0.142582617	0.219725078	0.999933887	-0.18765733	-0.332059386	0.144402057	0.606785865
ENSG00000211793	*TRAV9-2*	-0.926180883	0.171345724	0.380716272	-0.209370549	0.999933887	-0.086223158	0.201309001	-0.287532159	0.606864181
ENSG00000115128	*SF3B6*	4.655733033	-0.036701193	-0.040633216	0.003932023	0.999933887	-0.166257463	-0.094249734	-0.072007729	0.607005977
ENSG00000170558	*CDH2*	-0.653644725	-0.065538633	0.046942691	-0.112481324	0.999933887	-0.054291584	0.351014491	-0.405306074	0.607005977
ENSG00000130816	*DNMT1*	4.362298237	-0.011750234	0.03547312	-0.047223354	0.999933887	0.120353845	0.186268225	-0.06591438	0.607005977
ENSG00000158186	*MRAS*	-0.374302923	0.22471631	0.185465754	0.039250556	0.999933887	-0.457829758	-0.679129131	0.221299373	0.607040481
ENSG00000147684	*NDUFB9*	3.585964808	-0.031369574	-0.025191784	-0.00617779	0.999933887	-0.255483491	-0.355401058	0.099917567	0.607040481
ENSG00000213699	*SLC35F6*	4.157157361	-0.156081004	-0.084957053	-0.071123951	0.999933887	-0.682048894	-0.761544194	0.079495299	0.607360188
ENSG00000275532	*AC006449.2*	0.89481715	0.307561414	0.004295668	0.303265746	0.999933887	1.025609776	1.212974664	-0.187364888	0.607360188
ENSG00000261158	*AC109597.2*	-0.396638899	-0.002981667	0.080644241	-0.083625908	0.999933887	-0.401426433	-0.852022649	0.450596216	0.607360188
ENSG00000168564	*CDKN2AIP*	4.034925532	-0.127064701	-0.247176986	0.120112285	0.999933887	0.632421714	0.752533819	-0.120112105	0.60752227
ENSG00000126432	*PRDX5*	6.175369308	0.042044376	0.036404394	0.005639982	0.999933887	-0.033962368	-0.113439953	0.079477585	0.60752227
ENSG00000128928	*IVD*	2.802080549	0.026303312	-0.0398485	0.066151812	0.999933887	0.156413922	0.051937701	0.104476222	0.60752227
ENSG00000126243	*LRFN3*	0.815875758	-0.031635299	-0.167922196	0.136286896	0.999933887	0.024458692	0.193321707	-0.168863015	0.60752227
ENSG00000237254	*TRBV30*	0.141060663	0.035590252	-0.041680952	0.077271205	0.999933887	0.22497263	0.42018869	-0.19521606	0.60752227

ENSG00000171723	*GPHN*	-0.187388933	-0.101746312	-0.08378055	-0.017965761	0.999933887	0.124195441	-0.097819563	0.222015004	0.607804071
ENSG00000211745	*TRBV4-2*	-0.494783668	0.082096335	0.148587049	-0.066490713	0.999933887	0.040831401	0.266801709	-0.225970309	0.607804071
ENSG00000171999	*OR52P2P*	-0.557381468	0.065184051	-0.184272151	0.249456202	0.999933887	-0.111385626	0.32222797	-0.433613596	0.607804071
ENSG00000177728	*TMEM94*	4.162160248	-0.013938534	0.0838914	-0.097829934	0.999933887	0.004294213	-0.08598722	0.090281433	0.60800331
ENSG00000184481	*FOXO4*	5.735714245	0.010386778	-0.020530996	0.030917774	0.999933887	-0.539686045	-0.626640029	0.086953983	0.60800331
ENSG00000156253	*RWDD2B*	-0.384479165	-0.024135334	-0.234512301	0.210376967	0.999933887	0.322729743	0.10114005	0.221589694	0.60800331
ENSG00000279759	*AC118344.2*	0.307373247	-0.22905917	0.126950692	-0.356009861	0.999933887	-0.392135124	-0.196616147	-0.195518977	0.608330037
ENSG00000181513	*ACBD4*	1.653636183	0.06151171	0.08903686	-0.027525149	0.999933887	0.172473541	0.03949354	0.132980001	0.608330037
ENSG00000137947	*GTF2B*	5.139543098	-0.023313333	-0.052980162	0.029666828	0.999933887	-0.128058072	-0.059200219	-0.068857853	0.608330037
ENSG00000264493	*MIR4298*	0.519541961	0.026567051	-0.102542206	0.129109257	0.999933887	-0.261547532	-0.023175885	-0.238371647	0.608330037
ENSG00000119537	*KDSR*	3.306102453	-0.01046596	-0.033575742	0.023109782	0.999933887	-0.624200212	-0.726931593	0.10273138	0.608330037
ENSG00000124203	*ZNF831*	4.014188005	0.100115043	0.1619988	-0.061883757	0.999933887	0.048136202	-0.026355184	0.074491386	0.608740171
ENSG00000223725	*AC009226.1*	0.965700644	-0.047861795	0.302834441	-0.350696236	0.999933887	1.228549133	1.408565808	-0.180016675	0.608740171
ENSG00000111667	*USP5*	2.974926908	0.006617216	0.022532609	-0.015915393	0.999933887	0.170127705	0.073177774	0.096949931	0.608740171
ENSG00000278030	*TRBV7-9*	0.814340236	-0.109661326	-0.242626885	0.13296556	0.999933887	0.136054957	0.271246336	-0.135191379	0.608768489
ENSG00000169682	*SPNS1*	2.638238624	0.043270905	0.193549642	-0.150278737	0.999933887	0.494318359	0.611338065	-0.117019706	0.60923981
ENSG00000140939	*NOL3*	0.209601642	-0.032372888	0.266218234	-0.298591122	0.999933887	0.071136693	0.278610401	-0.207473708	0.609294777
ENSG00000100731	*PCNX1*	7.665626849	0.001583808	-0.034080517	0.035664325	0.999933887	-0.527421898	-0.602186053	0.074764155	0.609347928
ENSG00000139133	*ALG10*	-0.413413717	-0.148839991	-0.133568306	-0.015271685	0.999933887	-0.195087284	0.046599622	-0.241686907	0.609794726
ENSG00000185482	*STAC3*	0.236394616	0.066888733	-0.048363464	0.115252198	0.999933887	0.058849129	0.28205579	-0.223206661	0.610004093
ENSG00000233016	*SNHG7*	2.696861615	0.096228745	-0.076697505	0.172926251	0.999933887	0.140479044	0.038263059	0.102215985	0.610034575
ENSG00000184508	*HDDC3*	-0.200528132	0.156326599	-0.031931048	0.188257647	0.999933887	0.082296394	-0.167794155	0.250090549	0.610034575
ENSG00000268635	*AP003680.1*	0.504694295	0.339110552	0.579680816	-0.240570264	0.999933887	1.726009152	1.970959639	-0.244950487	0.610071544
ENSG00000065029	*ZNF76*	3.988365077	0.039507566	0.113666184	-0.074158618	0.999933887	0.212391689	0.282353857	-0.069962167	0.610143823
ENSG00000276966	*H4C5*	-0.653654868	-0.012235758	-0.253655654	0.241419895	0.999933887	-0.024125613	-0.310462271	0.286336658	0.610155379
ENSG00000241015	*TPM3P9*	1.194591305	0.08087008	0.085752908	-0.004882828	0.999933887	0.123053494	-0.025752692	0.148806186	0.610517183
ENSG00000154743	*TSEN2*	0.012784751	-0.18062034	-0.05206334	-0.128557	0.999933887	0.157240883	0.330441461	-0.173200578	0.610555212
ENSG00000224186	*C5orf66*	1.027649463	-0.172745383	-0.074108368	-0.098637015	0.999933887	-1.731648202	-1.921554339	0.189906137	0.610590829
ENSG00000211778	*TRAV4*	-0.369384614	0.052310759	0.084708498	-0.032397739	0.999933887	0.204157633	0.449119728	-0.244962094	0.610590829
ENSG00000090857	*PDPR*	6.134004082	-0.06218982	-0.081406365	0.019216545	0.999933887	0.09415481	0.173596258	-0.079441448	0.610609895
ENSG00000047597	*XK*	-0.448254931	-0.08998762	0.191636461	-0.281624081	0.999933887	-0.0472657	0.161031127	-0.208296826	0.610756391
ENSG00000135482	*ZC3H10*	2.097971145	-0.029353243	-0.001195594	-0.028157649	0.999933887	-0.419857331	-0.306468515	-0.113388816	0.610756391
ENSG00000072803	*FBXW11*	4.659934112	0.016755239	-0.016178881	0.03293412	0.999933887	0.073383676	0.152229073	-0.078845397	0.610756391
ENSG00000032389	*EIPR1*	1.922558482	0.020757829	-0.019989031	0.040746859	0.999933887	0.039838831	-0.108608158	0.148446989	0.610756391
ENSG00000205133	*TRIQK*	2.597850967	-0.057328794	0.014052659	-0.071381453	0.999933887	-0.495917234	-0.385227952	-0.110689281	0.610823601
ENSG00000239480	*AC073517.1*	-0.028409889	0.089632254	0.150965053	-0.0613328	0.999933887	-0.15559756	-0.376588506	0.220990946	0.611049963
ENSG00000222047	*C10orf55*	5.17407086	0.2634841	0.373451263	-0.109967163	0.999933887	1.42652851	1.553156836	-0.126628326	0.611205775
ENSG00000108064	*TFAM*	3.5913429	-0.043383927	-0.129817721	0.086433794	0.999933887	-0.137578782	-0.232542102	0.094963319	0.611213345
ENSG00000143742	*SRP9*	4.15450263	-0.067727115	-0.09515734	0.027430225	0.999933887	-0.100543174	-0.175618003	0.07507483	0.611533509
ENSG00000168970	*JMJD7-PLA2G*	2.000372841	0.122986609	0.191822721	-0.068836112	0.999933887	-0.044606067	-0.18173503	0.137128964	0.611533509
ENSG00000198855	*FICD*	2.419923046	0.077701691	0.017998342	0.059703349	0.999933887	0.429081558	0.325649605	0.103431953	0.611623385
ENSG00000205352	*PRR13*	6.705289726	-0.042818124	-0.01863064	-0.024187484	0.999933887	-0.152221282	-0.215467297	0.063246015	0.611849286
ENSG00000204228	*HSD17B8*	0.243741802	0.074421159	0.007580619	0.06684054	0.999933887	0.174094461	0.368932865	-0.194838405	0.611961945
ENSG00000101298	*SNPH*	1.388242894	0.037039272	0.043180045	-0.006140774	0.999933887	0.084709981	-0.08079126	0.165501241	0.612051474
ENSG00000141682	*PMAIP1*	7.779261758	0.117658721	0.126901318	-0.009242596	0.999933887	-0.143761473	-0.051860193	-0.09190128	0.612090876
ENSG00000155275	*TRMT44*	1.976633426	0.041057989	-0.050286389	0.091344378	0.999933887	0.240331841	0.371420249	-0.131088408	0.612231005
ENSG00000187642	*PERM1*	-0.885635343	0.065871187	-0.142795022	0.20866621	0.999933887	1.499648497	1.097637229	0.402011268	0.612231005
ENSG00000136720	*HS6ST1*	2.584181588	0.035647328	0.080481129	-0.044833801	0.999933887	0.279107558	0.398565043	-0.119457485	0.612304091
ENSG00000055917	*PUM2*	7.059317844	-0.051079719	-0.05086435	-0.000215369	0.999933887	-0.409497074	-0.339250787	-0.070246287	0.612380734
ENSG00000125885	*MCM8*	-0.446992621	-0.149046643	-0.184780643	0.035734	0.999933887	-0.012040166	-0.331737127	0.319696961	0.612380734
ENSG00000165055	*METTL2B*	1.005674742	0.027771772	0.034493874	-0.006722102	0.999933887	0.138070994	0.284246778	-0.146175784	0.612380734
ENSG00000240853	*RN7SL328P*	-0.224850257	-0.008305101	0.14340076	-0.151705862	0.999933887	0.185346552	0.470882491	-0.285535939	0.612380734
ENSG00000166912	*MTMR10*	6.233384318	-0.093102014	-0.092989029	-0.000112985	0.999933887	-1.363340311	-1.471475981	0.108135669	0.612398915
ENSG00000262766	*AC135050.5*	1.385066809	0.086466361	0.104977085	-0.018510723	0.999933887	-0.284306174	-0.462443472	0.178137298	0.612398915
ENSG00000286982	*AC016931.1*	0.065607502	0.154131919	0.37922314	-0.225091221	0.999933887	-0.14340618	0.084837781	-0.228243961	0.612398915
ENSG00000166123	*GPT2*	-0.995019079	0.172364552	0.585379067	-0.413014514	0.999933887	-0.066809509	0.22779987	-0.29460938	0.612398915
ENSG00000100055	*CYTH4*	9.545040849	-0.023510673	0.002365943	-0.025876616	0.999933887	-0.086787539	-0.153412693	0.066625153	0.612398915
ENSG00000258018	*LINC02457*	-0.568329752	0.051887998	0.513311363	-0.461423365	0.999933887	1.381573364	1.101432799	0.280140565	0.612398915
ENSG00000084093	*REST*	4.690063165	0.098973828	-0.063206843	0.162180672	0.999933887	-0.903251955	-1.04786224	0.144610285	0.612656653
ENSG00000109475	*RPL34*	7.258683233	-0.027093005	-0.042367049	0.015274044	0.999933887	0.071223998	0.142138168	-0.070914169	0.612656653
ENSG00000048028	*USP28*	2.650304349	-0.037640979	-0.088174116	0.050533137	0.999933887	-0.042587363	0.05260165	-0.095189013	0.612656653
ENSG00000175893	*ZDHHC21*	0.76089191	-0.044027241	-0.335968107	0.291940866	0.999933887	-0.163171583	0.061794	-0.224965583	0.612656653
ENSG00000211771	*TRBJ2-7*	-0.041770424	0.181386933	0.214396429	-0.033009496	0.999933887	0.118658367	-0.115771538	0.234429905	0.612770486
ENSG00000180739	*S1PR5*	3.981627603	0.018472743	-0.021023404	0.039496147	0.999933887	0.14765774	0.067093426	0.080564314	0.612770486
ENSG00000225920	*RIMKLBP2*	-0.923002813	-0.144083342	-0.779211848	0.635128507	0.999933887	-1.315897945	-0.96867785	-0.347220096	0.612799775
ENSG00000253214	*AC079209.1*	4.441752603	-0.043934378	0.053819994	-0.097754372	0.999933887	0.388719597	0.534405651	-0.145686054	0.612861895
ENSG00000100652	*SLC10A1*	1.034373775	0.157848217	0.171611908	-0.01376369	0.999933887	0.498004443	0.679977793	-0.18197335	0.613055169
ENSG00000279089	*AC005839.1*	1.52743931	-0.11787884	0.048965092	-0.166843932	0.999933887	-0.319879693	-0.492706895	0.172827202	0.613066909
ENSG00000258102	*MAP1LC3B2*	3.998637172	0.026789738	0.1092063	-0.082416562	0.999933887	0.101966771	0.019544159	0.082422612	0.613066909
ENSG00000184441	*AP001062.1*	0.559176009	0.018408328	0.194388541	-0.175980213	0.999933887	0.063423035	0.238856319	-0.175433284	0.613066909
ENSG00000127526	*SLC35E1*	4.986907643	-0.038059297	-0.020072763	-0.017986535	0.999933887	-0.128712791	-0.066282584	-0.062430207	0.613077409
ENSG00000155975	*VPS37A*	3.657644345	0.025626069	0.038133719	-0.01250765	0.999933887	-0.352937266	-0.433153729	0.080216463	0.613077409
ENSG00000025708	*TYMP*	7.48402929	0.036382616	0.081979582	-0.045596966	0.999933887	-0.73627262	-0.82784574	0.09157312	0.613086441
ENSG00000172456	*FGGY*	2.441512264	0.083582578	-0.037271764	0.120854342	0.999933887	0.642626855	0.772342212	-0.129715357	0.613414867
ENSG00000103111	*MON1B*	6.359896	-0.015935211	-0.03720744	0.02127223	0.999933887	-0.220648857	-0.283205171	0.062556314	0.613421961
ENSG00000267254	*ZNF790-AS1*	-0.93489944	-0.269098801	0.085923926	-0.355022727	0.999933887	-0.186066869	0.09059163	-0.276658499	0.614326908
ENSG00000215580	*BCORP1*	-4.063275687	0.06910035	0.065409141	0.003691209	0.999933887	0.134730954	0.425294478	-0.290563523	0.61453752
ENSG00000211890	*IGHA2*	1.041889351	0.029679274	0.251826798	-0.222147525	0.999933887	0.275262291	0.56049764	-0.285235349	0.61453752
ENSG00000133114	*GPALPP1*	2.838045642	0.089486452	0.08501644	0.004470012	0.999933887	0.252118207	0.382909499	-0.130791293	0.615059068
ENSG00000115421	*PAPOLG*	3.78723529	-0.071210982	-0.015101509	-0.056109473	0.999933887	-0.309834351	-0.228547931	-0.08128642	0.615059068
ENSG00000197603	*CPLANE1*	0.987534428	-0.031810319	-0.023739303	-0.008071017	0.999933887	-0.293818414	-0.061525455	-0.232292959	0.615059068
ENSG00000272812	*AC004908.2*	1.017535109	0.235885454	0.195630397	0.040255057	0.999933887	0.396750019	0.229519866	0.167230153	0.615136953
ENSG00000059804	*SLC2A3*	8.867080036	0.041388803	0.074532956	-0.033144153	0.999933887	-0.361956497	-0.285816521	-0.076139976	0.615178865
ENSG00000253978	*CTB-178M22.1*	-0.87737406	0.06280515	0.062507052	0.000298098	0.999933887	0.908947289	1.123411878	-0.214464589	0.615178865
ENSG00000115947	*ORC4*	3.004205885	-0.035968927	-0.116810874	0.080841947	0.999933887	-0.483051981	-0.618337325	0.135285344	0.61530949
ENSG00000123472	*ATPAF1*	2.074239437	0.075719773	-0.068777085	0.144496858	0.999933887	-0.011749692	-0.119444729	0.107695037	0.615391379
ENSG00000213619	*NDUFS3*	2.761098441	-0.04949747	0.00850451	-0.05800198	0.999933887	0.090654815	-0.005126667	0.095781482	0.615391379
ENSG00000271553	*AC018638.7*	-0.440169791	-0.041958591	0.219746272	-0.261704863	0.999933887	-0.157823175	0.155865369	-0.313688544	0.615391379
ENSG00000120451	*SNX19*	5.079928504	-0.025650229	-0.007370412	-0.018279817	0.999933887	-0.066638104	-0.118968923	0.052330818	0.615597223
ENSG00000258035	*AC123567.2*	3.366850226	-0.004015889	0.184873395	-0.188889284	0.999933887	0.792101025	0.942092397	-0.149991372	0.615597223
ENSG00000140694	*PARN*	3.650241756	-0.045598558	-0.04332889	-0.002269667	0.999933887	-0.15937374	-0.243077363	0.083703623	0.615672042
ENSG00000277149	*TYW1B*	0.989737653	0.05041427	-0.060969833	0.111384103	0.999933887	-0.050719981	0.112420898	-0.16314088	0.615871569
ENSG00000112282	*MED23*	4.480056995	0.025979225	-0.043506575	0.0694858	0.999933887	-0.316386183	-0.43050219	0.114116007	0.615871569
ENSG00000234358	*AC003080.1*	0.623211742	-0.008778204	0.233164982	-0.241943185	0.999933887	0.782701493	1.020762464	-0.238060971	0.615871569
ENSG00000115204	*MPV17*	3.166100231	0.12514604	0.105352054	0.019793986	0.999933887	0.074261746	-0.003046321	0.077308067	0.616120924
ENSG00000158887	*MPZ*	3.201122574	0.071439007	-0.074343443	0.14578245	0.999933887	0.503169693	0.594947672	-0.091777979	0.616120924
ENSG00000167460	*TPM4*	8.036961329	0.043562727	0.026375601	0.017187126	0.999933887	0.086112394	-0.002130291	0.088242685	0.616401862
ENSG00000042753	*AP2S1*	3.337085913	0.034993695	-0.094268345	0.129262039	0.999933887	-0.005055471	-0.106198259	0.101142788	0.616401862
ENSG00000104205	*SGK3*	1.641551007	-0.029794795	0.264008888	-0.293803683	0.999933887	-0.466652497	-0.329228326	-0.137424172	0.616567417
ENSG00000135414	*GDF11*	1.866446159	0.006439777	-0.082023039	0.088462816	0.999933887	-0.110175031	-0.220597455	0.110422424	0.616585207
ENSG00000204677	*FAM153CP*	-1.469592175	-0.392128092	0.145494462	-0.537622554	0.999933887	-0.544278875	-0.121569105	-0.42270977	0.616917648
ENSG00000182552	*RWDD4*	1.410960467	0.000838803	-0.034568771	0.035407574	0.999933887	-0.113768052	-0.26814536	0.154377308	0.616917648
ENSG00000118596	*SLC16A7*	2.677305722	-0.026244994	0.064586757	-0.090831751	0.999933887	-0.522102985	-0.393089528	-0.129013457	0.616921241
ENSG00000197355	*UAP1L1*	3.649661196	0.132440043	0.056571111	0.075868932	0.999933887	-0.349650281	-0.453906604	0.104256323	0.61718784
ENSG00000119523	*ALG2*	2.64072723	0.028093458	-0.085169146	0.113262604	0.999933887	0.135429239	0.042477361	0.092951878	0.617579717

ENSG00000245958	*AC093752.1*	2.550178719	0.104125749	0.035077121	0.069048628	0.999933887	-0.049323391	0.108444028	-0.157767419	0.617774156
ENSG00000160588	*MPZL3*	6.24094965	-0.087334412	-0.025146981	-0.062187431	0.999933887	0.075018068	0.001533534	0.073484534	0.618040165
ENSG00000196576	*PLXNB2*	2.00436746	0.058488329	0.058345379	0.00014295	0.999933887	-1.043808487	-1.190636788	0.146828301	0.618089666
ENSG00000111846	*GCNT2*	-0.192363559	-0.044998014	-0.143408592	0.098410578	0.999933887	0.103024268	-0.128497632	0.231521901	0.618233751
ENSG00000176871	*WSB2*	5.802538331	-0.088781075	0.003211311	-0.091992386	0.999933887	-0.301143676	-0.230684635	-0.070459041	0.618631494
ENSG00000131966	*ACTR10*	4.166738567	-0.061264401	-0.041554577	-0.019709824	0.999933887	-0.212111289	-0.140250523	-0.071860766	0.618631494
ENSG00000178982	*EIF3K*	5.411214132	-0.043338424	-0.042047535	-0.001290889	0.999933887	0.053023127	-0.007484908	0.060508035	0.618633032
ENSG00000243943	*ZNF512*	2.67829872	0.003103882	-0.0557238	0.058827682	0.999933887	-0.173364869	-0.262098438	0.088733569	0.618931069
ENSG00000244617	*ASPRV1*	4.173198539	-0.062430386	0.000328655	-0.062759042	0.999933887	0.301140145	0.400607486	-0.099467341	0.619026847
ENSG00000207248	*RNU6-1005P*	0.363152125	0.143627474	0.265867572	-0.122240097	0.999933887	0.554495429	0.779604296	-0.225108867	0.619134842
ENSG00000070018	*LRP6*	-0.120949814	-0.111473554	0.229678386	-0.341151939	0.999933887	-0.223104942	0.040787819	-0.263892761	0.619232546
ENSG00000116649	*SRM*	2.296555251	0.034210199	0.114942323	-0.080732123	0.999933887	0.211869403	0.313619379	-0.101749976	0.619280996
ENSG00000037280	*FLT4*	0.131621656	0.038659612	0.196722871	-0.158063259	0.999933887	0.085024351	-0.145217283	0.230241634	0.619280996
ENSG00000198960	*ARMCX6*	2.166774812	0.051394545	0.107535967	-0.056141422	0.999933887	-0.040749247	0.069082641	-0.109831888	0.619561375
ENSG00000151461	*UPF2*	5.327315014	-0.015065629	-0.164637926	0.149572297	0.999933887	-0.877933319	-1.003782038	0.125848719	0.619561375
ENSG00000170345	*FOS*	9.075142236	0.526978282	0.448872848	0.078105435	0.999933887	0.135610132	-0.00937729	0.144987422	0.61961244
ENSG00000174917	*MICOS13*	2.17678388	0.099115498	0.075855746	0.023259752	0.999933887	0.093518864	-0.010957818	0.104476682	0.619722041
ENSG00000274215	*AC106028.4*	1.2546104	0.260763301	-0.013124428	0.273887729	0.999933887	2.403173024	2.622781928	-0.219608904	0.620050351
ENSG00000223745	*CCDC18-AS1*	3.052829166	-0.106454822	-0.065769086	-0.040685736	0.999933887	-0.064084578	-0.168981418	0.104896841	0.620050351
ENSG00000145220	*LYAR*	3.284540143	-0.050184339	-0.045849217	-0.004335122	0.999933887	0.065850215	0.147147927	-0.081297712	0.620050351
ENSG00000121680	*PEX16*	4.565621366	-0.031307216	0.074719359	-0.106026575	0.999933887	-0.004241089	-0.073296457	0.069055369	0.620050351
ENSG00000009830	*POMT2*	2.007540934	0.024562484	-0.126195013	0.150757497	0.999933887	-0.20907466	-0.085570723	-0.123503937	0.620050351
ENSG00000261270	*AC012181.2*	0.408069912	0.191964301	0.068448257	0.123516044	0.999933887	0.244248076	-0.142757245	0.387005321	0.620116672
ENSG00000011590	*ZBTB32*	0.672943353	-0.090044976	0.13831277	-0.228357746	0.999933887	0.324110579	0.132179048	0.191931531	0.620116672
ENSG00000138380	*CARF*	0.696887018	0.093292145	-0.2328966	0.326188745	0.999933887	-0.182769466	0.013655746	-0.196425213	0.620116672
ENSG00000147324	*MFHAS1*	3.117479282	-0.025212131	0.063186529	-0.08839866	0.999933887	-0.002932305	-0.098152995	0.09522069	0.620116672
ENSG00000111796	*KLRB1*	3.226213189	-0.021230386	-0.082543033	0.061312646	0.999933887	0.018684773	0.129724299	-0.111039526	0.620116672
ENSG00000148803	*FUOM*	0.021237611	0.038746005	0.128714955	-0.08996895	0.999933887	-0.031340426	-0.223489642	0.192149217	0.620116672
ENSG00000172809	*RPL38*	6.590778915	-0.002216695	-0.010695018	0.008478323	0.999933887	0.081656881	0.157168861	-0.07551198	0.620116672
ENSG00000163728	*TTC14*	4.183632382	-0.098965375	-0.252519208	0.153553833	0.999933887	-0.424399601	-0.246145953	-0.178253649	0.620125905
ENSG00000269961	*AC010359.2*	1.721608286	0.12563656	0.309860691	-0.184224131	0.999933887	1.188092012	1.315310381	-0.127218369	0.620267574
ENSG00000100311	*PDGFB*	-0.041868049	0.086638744	0.518808837	-0.432170092	0.999933887	0.120532966	0.33804114	-0.217508173	0.620267574
ENSG00000272449	*AL139246.5*	7.080264469	0.200679278	0.18908537	0.011593909	0.999933887	0.981329965	1.12860399	-0.147274025	0.620400777
ENSG00000287265	*AL365272.1*	0.980950492	0.152245077	-0.027830372	0.180075449	0.999933887	-0.113358151	-0.247576966	0.134218815	0.620400777
ENSG00000146540	*C7orf50*	3.056600479	0.041693535	0.07915836	-0.037464824	0.999933887	-0.089423239	-0.188690143	0.099266904	0.620400777
ENSG00000274349	*ZNF658*	-0.531345702	-0.043270578	-0.082091022	0.038820444	0.999933887	-0.267236157	-0.571296821	0.304060664	0.620400777
ENSG00000113384	*GOLPH3*	5.280509036	0.001545164	0.004144029	-0.002598865	0.999933887	-0.336236696	-0.403308168	0.067071472	0.620400777
ENSG00000278743	*AC087239.1*	1.688988001	0.181147181	0.345100355	-0.163953174	0.999933887	0.831642931	1.03419548	-0.202552549	0.620519994
ENSG00000184517	*ZFP1*	0.577037826	-0.178681696	-0.075082497	-0.1035992	0.999933887	0.082746561	-0.095646524	0.178393086	0.620531894
ENSG00000112787	*FBRSL1*	6.669479279	0.034504214	0.049069002	-0.014564788	0.999933887	-0.057224953	-0.11993848	0.062713527	0.620531894
ENSG00000177409	*SAMD9L*	6.080398911	-0.078322288	-0.216337633	0.138015344	0.999933887	-1.01753882	-0.826961226	-0.190577594	0.620531894
ENSG00000138246	*DNAJC13*	4.580308515	-0.037793904	-0.186974502	0.149180598	0.999933887	-0.994358962	-1.127070949	0.132711987	0.620531894
ENSG00000160789	*LMNA*	5.873693035	-0.001362397	0.0014016	-0.002763997	0.999933887	0.03402209	0.117961787	-0.083939696	0.620531894
ENSG00000231830	*AC245140.1*	1.325192225	0.048934856	0.026165898	0.022768957	0.999933887	0.32797101	0.501358879	-0.173387869	0.62053791
ENSG00000132604	*TERF2*	3.496515195	0.016369318	-0.010738744	0.027108062	0.999933887	-0.221956558	-0.156634364	-0.065322194	0.62053791
ENSG00000205045	*SLFN12L*	1.67463911	-0.110371073	-0.024482239	-0.085888834	0.999933887	-0.177948615	0.016848574	-0.194797189	0.620719002
ENSG00000145730	*PAM*	3.472280941	-0.02696452	-0.001026527	-0.025937993	0.999933887	-0.008774572	0.078498709	-0.087273282	0.620749372
ENSG00000197050	*ZNF420*	-0.181845981	-0.170952354	-0.125099774	-0.04585258	0.999933887	-0.039367933	0.174859406	-0.214227339	0.620832051
ENSG00000214226	*C17orf67*	1.264101857	-0.100534123	-0.012151202	-0.088382921	0.999933887	-0.394610112	-0.544025778	0.149415666	0.620832051
ENSG00000128654	*MTX2*	-0.081035981	0.079743024	0.07774466	0.001998364	0.999933887	0.287284556	0.476712448	-0.189427892	0.620832051
ENSG00000158711	*ELK4*	4.598026605	-0.018139021	-0.066858948	0.048719927	0.999933887	-0.277893729	-0.150338968	-0.127554761	0.620866835
ENSG00000267046	*E2F3P1*	0.034860694	0.074166313	-0.336881927	0.41104824	0.999933887	-0.326595763	-0.089639298	-0.236956465	0.621204567
ENSG00000077380	*DYNC1I2*	2.352032928	-0.036649375	-0.16073757	0.124088195	0.999933887	0.082481796	0.184709848	-0.102228051	0.621204567
ENSG00000197694	*SPTAN1*	6.487622036	0.000803139	-0.04967189	0.050475029	0.999933887	0.023452495	-0.030113044	0.053565539	0.621204567
ENSG00000089472	*HEPH*	-0.887537564	-0.168653981	-0.199396152	0.03074217	0.999933887	-0.109657771	0.201040045	-0.310697817	0.621345625
ENSG00000213593	*TMX2*	3.033713381	-0.049430117	-0.005098654	-0.044331463	0.999933887	0.062051198	-0.022961644	0.085012842	0.621345625
ENSG00000241170	*AP001992.1*	1.862231272	-0.036992159	0.001921876	-0.038914035	0.999933887	-0.020296545	0.104572372	-0.124868917	0.621942417
ENSG00000119535	*CSF3R*	12.203206831	0.015563037	0.0118306	0.003732437	0.999933887	-0.012656304	-0.06108995	0.048433646	0.621942417
ENSG00000186115	*CYP4F2*	-2.83953	-0.03570801	-0.079358983	0.043650973	0.999933887	0.129231434	-0.209784009	0.339015444	0.621942417
ENSG00000086827	*ZW10*	0.614121482	-0.054738637	-0.11837201	0.063633373	0.999933887	0.151507779	0.309113706	-0.157605927	0.622118117
ENSG00000258839	*MC1R*	1.90303992	-0.032433484	0.056342609	-0.088776093	0.999933887	0.359890199	0.491064345	-0.131174146	0.622118117
ENSG00000036257	*CUL3*	6.16789017	-0.028326851	-0.102257134	0.073930283	0.999933887	-0.368006989	-0.296065603	-0.071941386	0.622133237
ENSG00000169203	*NPIPB12*	-0.035444401	0.075420002	-0.139293589	0.214713591	0.999933887	-0.177847858	0.079474691	-0.257322549	0.622133237
ENSG00000256967	*AC018653.3*	1.531866422	0.078620735	0.024956694	0.053664041	0.999933887	0.178345708	0.007065289	0.171280419	0.62282165
ENSG00000260144	*AC100827.2*	-0.471934839	0.152588383	0.236349574	-0.083761191	0.999933887	0.434578882	0.049849576	0.384729306	0.62282165
ENSG00000232815	*DUX4L50*	1.842790313	0.054791753	0.164816653	-0.110024901	0.999933887	-0.363742364	-0.551110846	0.187368482	0.62282165
ENSG00000225964	*NRIR*	0.492590935	-0.055697268	-0.148580156	0.092882888	0.999933887	0.069869292	0.277592713	-0.207723421	0.62282165
ENSG00000100564	*PIGH*	2.613458405	-0.119890876	0.099698741	-0.219589617	0.999933887	0.313646872	0.449245827	-0.135598954	0.622865118
ENSG00000118162	*KPTN*	2.024207868	0.077846838	0.025863199	0.051983639	0.999933887	0.162545266	0.031935169	0.130610096	0.622865118
ENSG00000100519	*PSMC6*	4.456908935	-0.042595994	-0.054753562	0.012157568	0.999933887	-0.139158642	-0.059431863	-0.079726779	0.622865118
ENSG00000152784	*PRDM8*	5.728352347	0.029834012	0.022824062	0.007009951	0.999933887	-0.243015426	-0.319689161	0.076673736	0.622865118
ENSG00000124588	*NQO2*	5.637092583	-0.053547232	-0.081210818	0.027663586	0.999933887	-0.12525739	-0.209428642	0.084171252	0.623127003
ENSG00000263712	*MIR4639*	-1.396928883	0.064292778	0.018630195	0.045662583	0.999933887	0.076392846	0.564257004	-0.487864157	0.623127003
ENSG00000133398	*MED10*	3.684137336	-0.038521716	-0.022398412	-0.016123304	0.999933887	-0.059269636	-0.128310126	0.069040491	0.623200711
ENSG00000279765	*AC013394.1*	2.456986183	0.089788761	0.193002237	-0.103213476	0.999933887	0.571568988	0.718308437	-0.14673945	0.623200711
ENSG00000136807	*CDK9*	5.59000903	0.0166879	0.024541251	-0.007853352	0.999933887	-0.223558929	-0.287500441	0.063941513	0.623200711
ENSG00000271064	*AC027644.2*	0.388197515	0.262675532	0.449360488	-0.186684955	0.999933887	1.307069885	0.950145308	0.356924577	0.623227113
ENSG00000204622	*HLA-J*	0.827673424	0.193814721	0.132119442	0.061695279	0.999933887	0.738851843	0.572312591	0.166539252	0.623227113
ENSG00000142686	*C1orf216*	1.526137853	-0.14629039	0.139647567	-0.285937958	0.999933887	0.094674747	-0.018790068	0.113464815	0.623227113
ENSG00000102445	*RUBCNL*	6.325069821	0.009600923	0.021678166	-0.012077243	0.999933887	0.000831193	0.068614854	-0.067783661	0.623227113
ENSG00000088812	*ATRN*	2.254954189	0.02662404	0.006437731	0.020186308	0.999933887	-0.101031665	-0.198996948	0.097965283	0.623334108
ENSG00000265908	*AC024267.5*	-0.480383897	0.027127699	0.019924553	0.007203146	0.999933887	-0.416682985	-0.071876225	-0.344806759	0.623334108
ENSG00000132952	*USPL1*	3.782997909	-0.015640701	-0.079241278	0.063600577	0.999933887	-0.193063733	-0.333057575	0.139993842	0.623481954
ENSG00000131944	*FAAP24*	0.248768395	-0.146287035	-0.202209296	0.05592226	0.999933887	-0.519645723	-0.749032122	0.229386399	0.623580882
ENSG00000185347	*TEDC1*	3.01159499	0.174745207	0.113310623	0.061434584	0.999933887	0.144706009	0.003532237	0.141173772	0.623585781
ENSG00000073910	*FRY*	6.87726768	-0.025259781	-0.029273127	0.004013346	0.999933887	-0.159146944	-0.090981366	-0.068165577	0.623585781
ENSG00000081019	*RSBN1*	4.135912506	0.027138116	-0.007532426	0.034670541	0.999933887	-0.370356224	-0.464416335	0.094060111	0.623585781
ENSG00000196305	*IARS1*	4.074470685	-0.023507553	0.053088348	-0.076595902	0.999933887	0.055878576	0.127084946	-0.07120637	0.623585781
ENSG00000139631	*CSAD*	5.556779	-0.013532768	0.01285566	-0.026388429	0.999933887	0.092894112	0.011565449	0.081328663	0.623585781
ENSG00000254415	*SIGLEC14*	6.835167331	-0.002790439	-0.054143564	0.051353125	0.999933887	0.824028418	0.904115692	-0.080087273	0.623585781
ENSG00000040531	*CTNS*	4.398513507	0.003442677	-0.03165801	0.035100687	0.999933887	-0.283008775	-0.206314068	-0.076694708	0.623757916
ENSG00000135048	*CEMIP2*	6.456552264	-0.037127726	-0.020287396	-0.01684033	0.999933887	0.001386849	0.064628635	-0.063241786	0.623778511
ENSG00000145819	*ARHGAP26*	8.683140974	0.029448843	0.026116522	0.003332321	0.999933887	0.107420269	0.063327223	0.044093046	0.624103708
ENSG00000111790	*FGFR1OP2*	4.57380776	-0.148002203	-0.119400887	-0.028601316	0.999933887	-0.646587549	-0.552704446	-0.093883103	0.624161407
ENSG00000167965	*MLST8*	1.954458385	-0.113971398	-0.014122771	-0.099848627	0.999933887	0.157326872	0.050102214	0.107224658	0.62419816
ENSG00000165606	*DRGX*	-1.197269249	0.044574941	-0.084913105	0.129488046	0.999933887	-1.557974187	-1.30368554	-0.254288648	0.62419816
ENSG00000050438	*SLC4A8*	1.255952298	-0.011346266	0.132955681	-0.144301946	0.999933887	-0.214952332	-0.059071566	-0.155880766	0.62419816
ENSG00000085382	*HACE1*	0.999322743	-0.010637921	-0.155429684	0.144791763	0.999933887	0.022089679	0.191977194	-0.169887515	0.62419816
ENSG00000103365	*GGA2*	5.202690512	-0.040280165	0.032357174	-0.072637339	0.999933887	0.001791155	-0.058672482	0.060463637	0.624451984
ENSG00000257838	*OTOAP1*	-0.523039596	-0.023383689	0.115348831	-0.13873252	0.999933887	-0.320010884	-0.501439831	0.181428947	0.624487182
ENSG00000232680	*AC002511.1*	1.18351925	0.053397945	0.058899456	-0.00550151	0.999933887	2.268135246	2.511992349	-0.243857103	0.624553346
ENSG00000256069	*A2MP1*	-0.117368972	0.008539057	0.00816831	0.000370747	0.999933887	-0.363548366	-0.122694582	-0.240853784	0.624601477
ENSG00000134046	*MBD2*	5.954092484	0.016835903	-0.006062448	0.022898351	0.999933887	-0.055065333	0.014815922	-0.069881255	0.624625491
ENSG00000070761	*CFAP20*	3.599026468	0.080429556	-0.005100501	0.085530057	0.999933887	0.309863391	0.411233232	-0.101369841	0.624679278
ENSG00000162415	*ZSWIM5*	-0.523908526	0.185321392	0.212609138	-0.027287747	0.999933887	-0.117733086	0.118901416	-0.236634502	0.624679278

ENSG00000254093	*PINX1*	0.244112806	0.086229467	0.116181564	-0.029952097	0.999933887	0.278841051	0.500420358	-0.221579308	0.624679278
ENSG00000225921	*NOL7*	3.636033594	0.034164002	0.022286682	0.011877321	0.999933887	0.023297715	0.103455107	-0.080157392	0.624679278
ENSG00000164674	*SYTL3*	4.44502437	0.003932112	0.016876984	-0.012944872	0.999933887	0.096485252	0.013185326	0.083299926	0.624679278
ENSG00000251634	*NAIPP4*	-0.769956065	0.22765578	-0.215140662	0.442796442	0.999933887	-0.744377015	-1.111975634	0.367598619	0.624792121
ENSG00000170471	*RALGAPB*	6.082829374	-0.018117825	-0.051889787	0.033771962	0.999933887	-0.345518091	-0.280283075	-0.065235016	0.62487509
ENSG00000135974	*C2orf49*	3.808798658	-0.020165022	-0.07894094	0.058775918	0.999933887	0.005331208	0.086686546	-0.081355338	0.62487509
ENSG00000285667	*AC012291.3*	0.307941624	-0.201633012	-0.390620301	0.188987289	0.999933887	-0.085277425	-0.288419932	0.203142506	0.625127847
ENSG00000170074	*FAM153A*	-0.84538944	0.225908293	-0.137943462	0.363851756	0.999933887	-0.004677159	0.254574666	-0.259251824	0.625127847
ENSG00000115523	*GNLY*	7.000830551	-0.01096925	-0.023236519	0.012267269	0.999933887	0.109664397	0.166106521	-0.056442124	0.625127847
ENSG00000228107	*AP000692.1*	-0.301209231	-0.156203156	0.219026812	-0.375229968	0.999933887	-0.509444022	-0.321921177	-0.187522845	0.625133831
ENSG00000100271	*TTLL1*	-0.71433276	0.264229744	-0.108143202	0.372372946	0.999933887	-0.062803925	-0.351902035	0.289098109	0.625218744
ENSG00000197061	*H4C3*	-0.873871833	-0.229947531	-0.391118796	0.161171265	0.999933887	-0.09152049	-0.586155316	0.494634826	0.625218744
ENSG00000250130	*GAPDHP43*	-0.05480078	0.161304801	-0.157671191	0.318975992	0.999933887	-0.313656265	-0.572272012	0.258615747	0.625218744
ENSG00000261455	*LINC01003*	4.459576469	0.068395372	0.023195835	0.045199537	0.999933887	0.26888972	0.368022074	-0.099132354	0.625218744
ENSG00000159377	*PSMB4*	6.95718056	-0.033295839	-0.023356849	-0.00993899	0.999933887	0.189199974	0.243341835	-0.05414186	0.625218744
ENSG00000258352	*IFITM3P6*	0.10539492	0.106456643	-0.161428589	0.267885232	0.999933887	-0.201408205	-0.005111817	-0.196296388	0.625218744
ENSG00000115286	*NDUFS7*	1.517468544	-0.065849358	0.023317964	-0.089167322	0.999933887	-0.166125441	-0.293734002	0.127608561	0.625218744
ENSG00000118762	*PKD2*	1.634321111	0.062806775	-0.008697983	0.071504759	0.999933887	-0.111468596	-0.265182403	0.153713808	0.625218744
ENSG00000100632	*ERH*	3.225298974	-0.109441482	0.075363027	-0.184804509	0.999933887	-0.090978534	-0.181672697	0.090694163	0.625461441
ENSG00000237489	*C10orf143*	-0.054237951	-0.204960412	-0.140346017	-0.064614395	0.999933887	-0.226307999	-0.027910535	-0.198397464	0.625461441
ENSG00000132128	*LRRC41*	3.381920118	0.082016882	0.005763992	0.076252891	0.999933887	0.026757079	0.098642252	-0.071885173	0.62555875
ENSG00000141012	*GALNS*	5.670330832	0.058639513	-0.014302133	0.072941646	0.999933887	0.146745841	0.22216377	-0.075417929	0.62585404
ENSG00000254649	*AP003086.2*	0.099894104	0.223384259	0.228472959	-0.0050887	0.999933887	-0.198341101	0.1130256	-0.311366701	0.62585404
ENSG00000207870	*MIR221*	-0.372999897	0.12962986	0.029841894	0.099787966	0.999933887	1.275880502	1.56663957	-0.290759069	0.62585404
ENSG00000167747	*C19orf48*	2.000733277	0.055062651	-0.105908187	0.160970838	0.999933887	0.367083623	0.245465537	0.121618085	0.62585404
ENSG00000137628	*DDX60*	4.053993494	-0.055175837	-0.102974196	0.04779836	0.999933887	-0.443104812	-0.286753124	-0.156351688	0.62585404
ENSG00000187134	*AKR1C1*	1.336608669	0.009535477	0.027611277	-0.0180758	0.999933887	0.042163894	0.173679988	-0.131516094	0.62585404
ENSG00000283384	*AL138694.1*	-0.350915837	0.002700385	-0.262895841	0.265596225	0.999933887	1.01905344	1.286269924	-0.267216484	0.62585404
ENSG00000088899	*LZTS3*	1.566234231	0.089823329	0.170342996	-0.080519668	0.999933887	0.207855976	0.349783284	-0.141927308	0.625889246
ENSG00000121933	*TMIGD3*	0.19044422	0.042910511	-0.017277137	0.060187648	0.999933887	-0.038910748	-0.22131412	0.182403372	0.625889246
ENSG00000166428	*PLD4*	0.472981478	-0.040980434	0.061268435	-0.102248869	0.999933887	-0.099125565	-0.323354867	0.224229302	0.626128171
ENSG00000240857	*RDH14*	0.511077512	-0.024011085	-0.006308393	-0.017702692	0.999933887	0.185677355	0.359956661	-0.174279307	0.626210684
ENSG00000214293	*APTR*	2.160782675	0.079694995	-0.018456425	0.098151421	0.999933887	-0.318488902	-0.425888004	0.107399102	0.62621578
ENSG00000174943	*KCTD13*	4.321269609	0.041962491	0.066753434	-0.024790943	0.999933887	0.150247663	0.211326099	-0.061078436	0.626424129
ENSG00000144677	*CTDSPL*	1.321946733	0.24044567	0.17059324	0.06985243	0.999933887	3.265901687	3.047311935	0.218589752	0.62673988
ENSG00000247240	*UBL7-AS1*	0.480078454	-0.125014123	-0.208339958	0.083325835	0.999933887	-0.856329998	-1.03353725	0.177207253	0.626835095
ENSG00000211897	*IGHG3*	0.585752979	0.136377739	-0.009107976	0.145485715	0.999933887	-0.108278485	0.200177044	-0.308455529	0.627162927
ENSG00000173548	*SNX33*	0.245327285	0.004076702	-0.057098941	0.061175643	0.999933887	-0.121269364	0.086600588	-0.207869952	0.62783298
ENSG00000186638	*KIF24*	0.35970916	0.203892019	0.299057457	-0.095165439	0.999933887	0.206751098	0.430098052	-0.223346954	0.628022499
ENSG00000275202	*AL161421.1*	4.20262322	0.079639051	0.085860407	-0.006221356	0.999933887	0.083781482	0.179364463	-0.095582981	0.628022499
ENSG00000185485	*SDHAP1*	2.740471897	0.064078938	-0.008415888	0.072494826	0.999933887	-0.068941601	-0.17033869	0.101397089	0.628022499
ENSG00000078124	*ACER3*	2.715109286	-0.048833121	-0.104166488	0.055333368	0.999933887	-0.275775527	-0.169444379	-0.106331147	0.628022499
ENSG00000145996	*CDKAL1*	1.45689748	-0.042331015	0.157415689	-0.199746704	0.999933887	0.024528283	0.159339499	-0.134811216	0.628022499
ENSG00000108370	*RGS9*	0.299790565	0.018324579	-0.174486498	0.192811077	0.999933887	0.143179964	0.333286672	-0.190106708	0.628022499
ENSG00000173041	*ZNF680*	-0.349070212	-0.002565424	-0.032974502	0.030409078	0.999933887	-0.338978391	-0.05766156	-0.281316831	0.628022499
ENSG00000169635	*HIC2*	3.313754783	0.071919632	0.031205495	0.040714137	0.999933887	-0.128798381	-0.221569541	0.09277116	0.628094334
ENSG00000100804	*PSMB5*	1.8188127	-0.030929294	-0.233620307	0.202691013	0.999933887	-0.204693835	-0.30995892	0.105265085	0.628275522
ENSG00000174791	*RIN1*	-0.42152293	0.195085461	0.038218641	0.15686682	0.999933887	-0.160718207	-0.385649095	0.224930889	0.628305183
ENSG00000254420	*AP003086.1*	0.280297118	-0.070781361	0.267463375	-0.338244736	0.999933887	0.172880289	0.335757314	-0.162877025	0.628305183
ENSG00000145723	*GIN1*	0.466842812	-0.167062418	0.06962161	-0.236684028	0.999933887	-0.413110299	-0.280354605	-0.132755694	0.628846945
ENSG00000280433	*FP565260.6*	0.735900757	-0.076694976	-0.056136264	-0.020558713	0.999933887	-0.271319366	-0.461622331	0.190302964	0.628846945
ENSG00000107185	*RGP1*	4.489295981	0.048755425	0.00383771	0.044917715	0.999933887	-0.007802838	-0.083522705	0.075719867	0.628960316
ENSG00000155906	*RMND1*	1.307873593	-0.205711025	0.168303742	-0.374014767	0.999933887	0.05385387	-0.073281383	0.127135253	0.628971195
ENSG00000172116	*CD8B*	4.133070862	0.056049739	-0.094809036	0.150858775	0.999933887	0.08204735	0.170612884	-0.088565535	0.628971195
ENSG00000204524	*ZNF805*	2.526047118	0.027213692	0.039622944	-0.012409252	0.999933887	-0.17924452	-0.042823281	-0.136421239	0.629018829
ENSG00000257446	*ZNF878*	-1.570383944	-0.528228967	-0.341504146	-0.18672482	0.999933887	-0.849525518	-0.553909391	-0.295616127	0.629316506
ENSG00000101413	*RPRD1B*	4.808700639	-0.053640959	-0.026097832	-0.027543127	0.999933887	-0.031986015	0.020291157	-0.052277172	0.629867844
ENSG00000181789	*COPG1*	5.707317276	-0.030729803	0.050113816	-0.080843619	0.999933887	-0.345962745	-0.402929943	0.056967198	0.629946049
ENSG00000181982	*CCDC149*	-0.719225708	-0.033058342	0.118912836	-0.151971178	0.999933887	-0.753688864	-1.085603223	0.331914358	0.629972333
ENSG00000174106	*LEMD3*	5.391888147	0.013084278	0.030158717	-0.017074438	0.999933887	0.059219677	0.114300996	-0.055081319	0.630027572
ENSG00000041357	*PSMA4*	5.454941224	-0.052872936	-0.119696108	0.066823172	0.999933887	-0.026808771	0.032262506	-0.059071277	0.630084121
ENSG00000047410	*TPR*	6.541844446	-0.107495384	-0.185526966	0.078031582	0.999933887	-0.792676288	-0.574577677	-0.218098611	0.630084121
ENSG00000101417	*PXMP4*	0.259769987	0.068590008	-0.048926408	0.117516416	0.999933887	-0.107463801	0.031344382	-0.138808183	0.630084121
ENSG00000181220	*ZNF746*	7.6497113	0.015844491	0.097011294	-0.081166803	0.999933887	-0.326082588	-0.247512569	-0.078570019	0.630084121
ENSG00000259250	*AC018904.1*	-0.091056412	-0.06919652	-0.332869275	0.263672754	0.999933887	-0.778848959	-0.985025624	0.206176665	0.630114397
ENSG00000244265	*SIAH2-AS1*	-0.717018133	-0.058876176	0.287872777	-0.346748952	0.999933887	0.118987261	0.471113476	-0.352126215	0.630114397
ENSG00000224914	*LINC00863*	1.143845912	0.006329709	0.10072902	-0.094399311	0.999933887	0.019845887	0.165380673	-0.145534785	0.630114397
ENSG00000267575	*AC006504.5*	-0.588703511	0.051739053	0.372799725	-0.321060672	0.999933887	-0.51005443	-0.247478445	-0.262575985	0.630353551
ENSG00000135976	*ANKRD36*	2.413820801	-0.048799652	0.086369096	-0.135168748	0.999933887	0.006240559	0.165901352	-0.159660793	0.630359951
ENSG00000174428	*GTF2IRD2B*	1.425559009	0.070911441	-0.028866564	0.099778005	0.999933887	0.128722275	0.29104045	-0.162318175	0.630407165
ENSG00000142178	*SIK1*	0.78520919	-0.014695882	0.028503808	-0.04319969	0.999933887	0.174415092	0.023031076	0.151384016	0.630407165
ENSG00000279649	*AC020978.9*	-0.164270327	0.007159455	0.05991023	-0.052750776	0.999933887	-0.151771413	0.104326819	-0.256098232	0.630407165
ENSG00000211896	*IGHG1*	2.31450646	0.156510056	0.043152597	0.113357459	0.999933887	0.079681686	-0.124386109	0.204067795	0.630444741
ENSG00000170445	*HARS1*	4.024985104	-0.036063443	0.005465905	-0.041529348	0.999933887	-0.00417181	0.058842644	-0.063014454	0.630444741
ENSG00000157388	*CACNA1D*	0.79635405	-0.070759551	0.035258839	-0.10601839	0.999933887	-0.488187812	-0.693517262	0.20532945	0.630598734
ENSG00000134758	*RNF138*	4.889192866	0.043303748	0.034706113	0.008597635	0.999933887	-0.41237183	-0.532311498	0.119939668	0.630598734
ENSG00000198783	*ZNF830*	4.13908109	-0.009245339	-0.044875037	0.035629698	0.999933887	0.188617647	0.263501325	-0.074883679	0.630598734
ENSG00000243176	*AC092944.1*	1.467952891	0.257874677	0.129935071	0.127939606	0.999933887	0.574776669	0.395637702	0.179138966	0.630667506
ENSG00000163933	*RFT1*	4.253018845	0.043766763	-0.068072597	0.111839359	0.999933887	-0.06447682	0.027014942	-0.091491761	0.630667506
ENSG00000132801	*ZSWIM3*	0.633568711	-0.099001513	-0.3141587	0.215157187	0.999933887	-0.098381988	-0.235458733	0.137076746	0.630932034
ENSG00000178562	*CD28*	4.529557072	-0.029652808	-0.058531615	0.028878807	0.999933887	0.061377649	0.125537577	-0.064159928	0.63100311
ENSG00000025772	*TOMM34*	2.458344273	-0.047186548	-0.069415621	0.022229074	0.999933887	-0.013353343	-0.116179053	0.10282571	0.631166528
ENSG00000033011	*ALG1*	0.588835539	0.052233132	0.191433143	-0.139200011	0.999933887	0.149089437	-0.024802473	0.17389191	0.631333844
ENSG00000124406	*ATP8A1*	4.460038181	-0.031864611	-0.094296991	0.06243238	0.999933887	-0.300636311	-0.119989182	-0.180647129	0.631333844
ENSG00000099246	*RAB18*	5.612519879	-0.029364118	0.004771848	-0.034135966	0.999933887	-0.406891293	-0.339987588	-0.066903706	0.631456537
ENSG00000138050	*THUMPD2*	0.762615832	0.035908244	-0.072420123	0.108328367	0.999933887	0.07003228	0.234147834	-0.164115555	0.631469237
ENSG00000155666	*KDM8*	1.112727253	-0.020616132	0.144476066	-0.165092198	0.999933887	0.003358497	0.136943825	-0.133585328	0.63162154
ENSG00000082516	*GEMIN5*	1.390136919	-0.074062611	0.016176288	-0.090238899	0.999933887	0.184321702	0.067531894	0.116789808	0.63191358
ENSG00000090554	*FLT3LG*	-0.20019809	0.006627671	0.185478421	-0.178850749	0.999933887	-0.114524089	0.144370869	-0.258894958	0.632123954
ENSG00000187688	*TRPV2*	4.203893423	0.06309331	0.050349179	0.012744131	0.999933887	-0.066493832	-0.140695148	0.074201317	0.632204945
ENSG00000108349	*CASC3*	7.808054711	-0.016781476	-0.021493496	0.004712021	0.999933887	-0.38066113	-0.43232735	0.05166622	0.632477705
ENSG00000196262	*PPIA*	6.199775447	-0.027502134	-0.000296019	-0.027206115	0.999933887	0.123901662	0.187341041	-0.063439379	0.63261917
ENSG00000104228	*TRIM35*	2.30094727	0.020361255	0.0746593	-0.054298045	0.999933887	0.008496058	0.114768131	-0.106272073	0.63261917
ENSG00000100316	*RPL3*	8.899835056	0.010551243	-0.033198173	0.043749416	0.999933887	0.105004148	0.172722466	-0.067718319	0.63261917
ENSG00000102390	*PBDC1*	2.392942512	-0.04370399	0.107196346	-0.150900335	0.999933887	-0.148414256	-0.259651153	0.111236897	0.632799056
ENSG00000196110	*ZNF699*	2.642602923	-0.038828365	-0.00057273	-0.038255635	0.999933887	-0.253090411	-0.360904893	0.107814483	0.632799056
ENSG00000139998	*RAB15*	2.171524887	-0.01728614	-0.023737408	0.006451268	0.999933887	-0.143151357	-0.269508962	0.126357605	0.633162104
ENSG00000087191	*PSMC5*	4.159432794	0.030670102	-0.016779791	0.047449893	0.999933887	-0.010360081	-0.073128706	0.062768624	0.633433902
ENSG00000101752	*MIB1*	4.361434262	-0.002061726	-0.070073572	0.068011846	0.999933887	-0.372725651	-0.213374899	-0.159350752	0.633433902
ENSG00000113407	*TARS1*	3.611105242	-0.104021671	-0.187444358	0.083422687	0.999933887	0.089265892	0.168669453	-0.079403561	0.634012437
ENSG00000117724	*CENPF*	-0.644916015	-0.200494181	-0.154426538	-0.046067642	0.999933887	-0.136817752	0.204183699	-0.341001452	0.634012437
ENSG00000135900	*MRPL44*	3.480013647	-0.100304199	-0.052148273	-0.048155926	0.999933887	0.342326851	0.425056179	-0.082729328	0.634012437
ENSG00000274512	*TBC1D3L*	-3.156599491	-0.204774601	0.431000598	-0.635775199	0.594252256	0.104773702	-0.104141729	0.208915432	0.634012437
ENSG00000167210	*LOXHD1*	3.340457676	-0.033053635	-0.045912234	0.012858599	0.999933887	0.691261891	0.550436258	0.140825633	0.634012437
ENSG00000270339	*PPIAL4H*	-2.003125698	-0.09133892	0.059035767	-0.150374687	0.999933887	2.014730748	2.236647307	-0.221916559	0.634012437

ENSG00000186152	*LILRP1*	-0.277266245	0.121736744	0.091932231	0.029804512	0.999933887	-0.428361804	-0.224113432	-0.204248373	0.634225201
ENSG00000228323	*MYADM-AS1*	0.693037145	0.212542914	0.276165218	-0.063622303	0.999933887	0.23312078	-0.141405824	0.374526605	0.634254489
ENSG00000153767	*GTF2E1*	2.119413262	-0.096962345	-0.068473961	-0.028488384	0.999933887	0.074002219	-0.010484761	0.084486979	0.634254489
ENSG00000072682	*P4HA2*	1.079403894	-0.070477711	0.025518751	-0.095996462	0.999933887	-0.396824567	-0.211199229	-0.185625338	0.634254489
ENSG00000134899	*ERCC5*	2.269235673	0.030603685	0.005738422	0.024865263	0.999933887	-0.177207121	-0.310776306	0.133569185	0.634308891
ENSG00000198034	*RPS4X*	7.86380355	-0.011910117	-0.06253234	0.050622223	0.999933887	0.097800817	0.170666616	-0.072865799	0.634521853
ENSG00000208028	*MIR616*	2.61528005	0.222939107	0.428808803	-0.205869697	0.999933887	0.440902078	0.290871919	0.150030159	0.634619576
ENSG00000157110	*RBPMS*	1.185070264	0.058029088	0.148711312	-0.090682224	0.999933887	-0.231138766	-0.407464628	0.176325862	0.634619576
ENSG00000179604	*CDC42EP4*	1.934469637	-0.023896768	0.011043868	-0.034940636	0.999933887	-0.528083105	-0.676179531	0.148096426	0.634619576
ENSG00000167380	*ZNF226*	2.062324331	-0.158159715	-0.173765993	0.015606278	0.999933887	0.035133731	0.130775339	-0.095641608	0.634630933
ENSG00000183813	*CCR4*	2.851041946	-0.011820778	-0.02878758	0.016966802	0.999933887	0.188987398	0.086786542	0.102200857	0.634630933
ENSG00000114631	*PODXL2*	2.009156021	0.184333524	0.292261445	-0.107927921	0.999933887	0.129916713	0.000254403	0.12966231	0.634850136
ENSG00000140326	*CDAN1*	2.50549635	0.097682081	-0.095913318	0.193595399	0.999933887	0.080265593	-0.036490255	0.116755848	0.634850136
ENSG00000154099	*DNAAF1*	-0.228978354	0.27379264	0.127144607	0.146648033	0.999933887	3.920150791	4.346999265	-0.426848474	0.635036997
ENSG00000100246	*DNAL4*	2.024966358	0.042651069	0.009858257	0.032792812	0.999933887	-0.14732913	-0.011382225	-0.135946905	0.635036997
ENSG00000100380	*ST13*	5.314739347	-0.040655348	-0.059082599	0.018427251	0.999933887	-0.216427728	-0.279654688	0.063226961	0.635079479
ENSG00000112759	*SLC29A1*	3.450260893	0.038295401	0.03385186	0.004443541	0.999933887	0.058338339	0.160446334	-0.102107995	0.635079479
ENSG00000100836	*PABPN1*	5.90266472	0.013573273	0.02038298	-0.006809707	0.999933887	-0.128247578	-0.179393495	0.051145916	0.63513379
ENSG00000172215	*CXCR6*	0.904669468	0.1304549	0.087252224	0.043202676	0.999933887	0.078724804	-0.074868378	0.153593182	0.635480584
ENSG00000285420	*PDGFRL2P*	0.848424857	-0.011516956	-0.001712106	-0.00980485	0.999933887	-0.110840384	-0.272872512	0.162032128	0.63556356
ENSG00000228506	*AL513550.1*	-0.081255079	-0.225687747	0.017379687	-0.243067434	0.999933887	0.175034604	0.368279058	-0.193244454	0.635774369
ENSG00000133422	*MORC2*	3.028685498	-0.068177301	0.018867117	-0.087044418	0.999933887	-0.190504243	-0.270605539	0.080101297	0.635774369
ENSG00000198242	*RPL23A*	5.282564223	-0.033307307	8.8949e-05	-0.033396256	0.999933887	0.127359795	0.201936569	-0.074576774	0.635774369
ENSG00000186522	*SEPTIN10*	-1.151296513	-0.124559408	-0.429003253	0.304443844	0.999933887	-0.406008035	-0.214997472	-0.191010563	0.635774369
ENSG00000155868	*MED7*	2.003682263	0.008200846	-0.263580088	0.271780934	0.944094537	-0.167766342	-0.07284293	-0.094923412	0.635774369
ENSG00000117226	*GBP3*	3.278096741	-0.00813801	-0.086186361	0.078048351	0.999933887	0.432936565	0.575107345	-0.14217078	0.635774369
ENSG00000185909	*KLHDC8B*	6.618514717	-0.234654788	-0.199484995	-0.035169793	0.999933887	-1.438028013	-1.572921056	0.134893043	0.635905772
ENSG00000235245	*AL360181.2*	2.318700586	0.024286479	-0.016833092	0.041119572	0.999933887	-0.127195603	-0.027335528	-0.099860075	0.635913613
ENSG00000161057	*PSMC2*	3.643454569	-0.061042633	-0.115903793	0.054861159	0.999933887	-0.050799533	0.020066273	-0.070865806	0.63615745
ENSG00000151093	*OXSM*	1.918229681	-0.028198971	-0.071546736	0.043347765	0.999933887	0.25550765	0.387277123	-0.131769472	0.63615745
ENSG00000120833	*SOCS2*	2.163311102	-0.14842332	-0.031181725	-0.117241595	0.999933887	0.12905841	0.266615925	-0.137557514	0.636466643
ENSG00000166801	*FAM111A*	4.394020077	0.011653214	0.028526115	-0.0168729	0.999933887	-0.215731346	-0.311468805	0.095737459	0.636466643
ENSG00000270426	*AC099343.2*	2.585533511	-0.165718939	-0.194229131	0.028510192	0.999933887	0.466685598	0.380443651	0.086241947	0.636730254
ENSG00000165626	*BEND7*	0.038770691	0.118545092	0.059940271	0.058604821	0.999933887	-0.736574984	-0.943149788	0.206574804	0.63676072
ENSG00000162066	*AMDHD2*	7.849459188	0.078700409	0.109839735	-0.031139327	0.999933887	-0.011614203	-0.108828486	0.097214283	0.63676072
ENSG00000234292	*AC123595.1*	-0.320466727	0.183768543	0.161817048	0.021951496	0.999933887	1.244113857	1.018175044	0.225938814	0.63676072
ENSG00000177981	*ASB8*	4.403525432	-0.04185578	-0.043878287	0.002022506	0.999933887	-0.313359598	-0.238840501	-0.074519097	0.63676072
ENSG00000197062	*ZSCAN26*	1.224444439	0.076482087	0.035131189	0.041350899	0.999933887	-0.025208428	-0.171155145	0.145946717	0.63676072
ENSG00000053372	*MRTO4*	1.504906608	0.058886792	0.036892633	0.021994159	0.999933887	0.302952464	0.418241472	-0.115289008	0.63682425
ENSG00000152601	*MBNL1*	7.481489026	0.01448256	-0.093848896	0.108331456	0.999933887	-0.345908838	-0.201943858	-0.143964979	0.63690542
ENSG00000119661	*DNAL1*	-0.106506986	0.020264938	-0.025637499	0.045902437	0.999933887	-0.154892958	0.058489047	-0.213382005	0.63739471
ENSG00000159216	*RUNX1*	4.988775727	0.01345882	-0.00860735	0.02206617	0.999933887	0.019195293	0.083148616	-0.063953323	0.637701591
ENSG00000128789	*PSMG2*	2.680243572	-0.083754206	0.055871653	-0.139625859	0.999933887	0.038717054	-0.057233212	0.095950266	0.637748687
ENSG00000141664	*ZCCHC2*	6.440000034	-0.001305693	-0.003596921	0.002291228	0.999933887	-0.110347148	-0.031895391	-0.078451757	0.637751768
ENSG00000196365	*LONP1*	5.74921643	0.091546276	0.10333493	-0.011788654	0.999933887	0.341592761	0.439197891	-0.09760513	0.637760383
ENSG00000102007	*PLP2*	5.47664893	0.016165868	0.01962639	-0.003460522	0.999933887	-0.173230787	-0.255433304	0.082202517	0.63785067
ENSG00000114735	*HEMK1*	2.777320738	-0.017044728	-0.028203419	0.011158691	0.999933887	0.116228437	0.030068278	0.086160158	0.637884944
ENSG00000215022	*AL008729.1*	6.12004821	0.209708522	0.308880497	-0.099171975	0.999933887	0.81043141	0.910320633	-0.099889223	0.637904217
ENSG00000152147	*GEMIN6*	-0.563293557	-0.161290653	-0.090827598	-0.070463054	0.999933887	0.01281147	0.199312139	-0.186500669	0.637904217
ENSG00000135945	*REV1*	3.883906388	-0.032611745	-0.004705173	-0.027906571	0.999933887	-0.210326669	-0.290350864	0.080024195	0.637964565
ENSG00000184389	*A3GALT2*	-0.927696641	-0.059060756	0.012411047	-0.071471803	0.999933887	0.00557114	-0.400544256	0.406115396	0.637964565
ENSG00000272078	*AL139423.1*	0.999491	0.082854566	0.074780382	0.008074184	0.999933887	0.566770566	0.743230513	-0.176459947	0.638147309
ENSG00000167625	*ZNF526*	1.883074734	-0.060470684	-0.107880755	0.047410071	0.999933887	-0.245327168	-0.358971359	0.113644191	0.638147309
ENSG00000141101	*NOB1*	2.066453521	-0.080633866	-0.252878022	0.172244156	0.999933887	0.247747657	0.358668927	-0.11092127	0.638266167
ENSG00000255529	*POLR2M*	1.656281041	-0.052050621	0.081426066	-0.133476687	0.999933887	0.041596344	0.170251114	-0.12865477	0.638266167
ENSG00000144118	*RALB*	7.41285209	0.004973507	-0.013410197	0.018383704	0.999933887	0.205213935	0.27690089	-0.071686955	0.638474123
ENSG00000228451	*SDAD1P1*	2.091793707	0.070984424	0.029565518	0.041418906	0.999933887	0.151451157	0.301845035	-0.150393878	0.638614136
ENSG00000234263	*MAP3K5-AS1*	-0.439633228	-0.012352804	-0.073241712	0.060888908	0.999933887	1.472300075	1.726864765	-0.25456469	0.638875898
ENSG00000094804	*CDC6*	0.091333367	0.071321773	0.05336166	0.017960113	0.999933887	0.507052298	0.685742718	-0.178690419	0.63911707
ENSG00000010327	*STAB1*	1.42302964	0.095100013	0.009539342	0.085560671	0.999933887	-0.275520823	-0.412390991	0.136870168	0.639168477
ENSG00000125812	*GZF1*	4.946290429	0.077288805	-0.003695698	0.080984504	0.999933887	-0.521511339	-0.593981432	0.072470094	0.639245285
ENSG00000099330	*OCEL1*	1.489148136	0.054149133	-0.013559988	0.06770912	0.999933887	-0.013245889	-0.178736549	0.16549066	0.639245285
ENSG00000122335	*SERAC1*	1.82539221	-0.039449171	-0.102294994	0.062845823	0.999933887	-0.259011608	-0.153794882	-0.105216727	0.639245285
ENSG00000108256	*NUFIP2*	6.536001146	-0.015245046	-0.073853211	0.058608165	0.999933887	-0.84627122	-0.7316591	-0.114612119	0.63963097
ENSG00000214894	*LINC00243*	1.686315311	0.135129474	0.020838201	0.114291273	0.999933887	1.162480572	1.304697579	-0.142217008	0.639796626
ENSG00000238113	*LINC01410*	2.398521183	-0.051075862	0.039776444	-0.090852306	0.999933887	-0.597443049	-0.736603958	0.139160909	0.639796626
ENSG00000124160	*NCOA5*	3.541256371	-0.038967258	-0.021352128	-0.01761513	0.999933887	-0.099828127	-0.035658576	-0.064169551	0.639796626
ENSG00000037042	*TUBG2*	0.506859286	0.066698737	-0.00959436	0.076293098	0.999933887	0.154298285	0.012835523	0.141462762	0.639796626
ENSG00000101844	*ATG4A*	1.274309047	-0.029335336	0.011869257	-0.041204593	0.999933887	0.122831861	0.300441833	-0.177609972	0.639796626
ENSG00000243797	*AC004917.1*	1.335303667	-0.037776256	0.034895793	-0.072672049	0.999933887	1.237875243	1.440315753	-0.20244051	0.639796626
ENSG00000130299	*GTPBP3*	1.526992752	0.066801069	-0.111094591	0.17789566	0.999933887	0.114654971	0.003921048	0.110733923	0.639815833
ENSG00000235852	*AC005540.1*	-0.688030737	0.091324796	0.240323126	-0.14899833	0.999933887	0.353957359	0.049011012	0.304946347	0.639815833
ENSG00000188305	*PEAK3*	4.409474148	-0.030460507	-0.093614545	0.063154038	0.999933887	-1.183524138	-1.275446067	0.091921929	0.639815833
ENSG00000214655	*ZSWIM8*	7.685773501	0.01254444	0.058660134	-0.046115693	0.999933887	-0.066714564	-0.012743281	-0.053971283	0.639815833
ENSG00000196074	*SYCP2*	1.1659993	-0.204246394	-0.281188709	0.076942316	0.999933887	-0.363973761	-0.066949643	-0.297024118	0.639939242
ENSG00000236104	*ZBTB22*	3.820188856	0.090693423	0.072311402	0.018382021	0.999933887	0.248695279	0.326586611	-0.077891333	0.639997775
ENSG00000206199	*ANKUB1*	1.75316084	-0.08681527	0.063567208	-0.150382478	0.999933887	0.517449258	0.673382756	-0.155933498	0.639997775
ENSG00000287883	*AC093627.22*	-0.209119214	-0.034489109	0.026622497	-0.061111606	0.999933887	-0.091640197	0.088104807	-0.179745005	0.640119526
ENSG00000162408	*NOL9*	2.987061025	-0.075784535	0.045028054	-0.12081259	0.999933887	0.093538319	0.015075934	0.078462385	0.6401624
ENSG00000184613	*NELL2*	3.881228642	-0.033261028	-0.145077607	0.111816579	0.999933887	0.121603325	0.194306643	-0.072703317	0.64022778
ENSG00000084731	*KIF3C*	2.845670173	0.035272181	0.115973517	-0.080701336	0.999933887	-0.053703214	0.037158354	-0.090861568	0.640361976
ENSG00000176022	*B3GALT6*	2.267279561	0.020480935	0.228304446	-0.207823511	0.999933887	0.236582649	0.126011001	0.110571648	0.640427284
ENSG00000067064	*IDI1*	5.513441042	0.029706103	-0.044126633	0.073832736	0.999933887	-0.014535427	-0.084830314	0.070294887	0.640489119
ENSG00000154127	*UBASH3B*	6.117329669	-0.081293979	-0.045153024	-0.036140955	0.999933887	-0.59797782	-0.534027584	-0.063950236	0.640639316
ENSG00000129055	*ANAPC13*	5.728017647	-0.083884796	-0.07892048	-0.004964315	0.999933887	-0.648638374	-0.576255084	-0.07238329	0.640668077
ENSG00000188092	*GPR89B*	0.470459208	-0.15698238	-0.033194108	-0.123788273	0.999933887	-0.094674535	0.064450314	-0.159124849	0.640668077
ENSG00000278540	*ACACA*	1.922864167	-0.080113895	-0.057876488	-0.022237408	0.999933887	0.084615112	0.193948894	-0.109333782	0.640668077
ENSG00000163521	*GLB1L*	1.433865798	-0.093782378	-0.118622642	0.024840265	0.999933887	-0.005075865	-0.128798652	0.123722787	0.640668077
ENSG00000168672	*LRATD2*	2.123480823	-0.031946617	-0.027850765	-0.004095853	0.999933887	0.109224162	0.009352297	0.099871866	0.640668077
ENSG00000197363	*ZNF517*	1.216397421	0.036815666	0.13332953	-0.096513864	0.999933887	0.165786404	0.356512388	-0.190725984	0.640668077
ENSG00000147364	*FBXO25*	1.829930352	-0.075652235	-0.00998176	-0.065670475	0.999933887	-0.042519353	0.07417417	-0.116693523	0.640898651
ENSG00000122550	*KLHL7*	2.37214933	-0.035981306	-0.103968857	0.067987551	0.999933887	-0.217695287	-0.11203692	-0.105658367	0.640898651
ENSG00000179698	*WDR97*	0.471474098	0.060923808	0.193535043	-0.132611235	0.999933887	0.058456766	-0.12718414	0.185640906	0.640898651
ENSG00000150712	*MTMR12*	3.735156685	-0.021641578	-0.054778716	0.033137137	0.999933887	-0.173844826	-0.256757606	0.08291278	0.640898651
ENSG00000075239	*ACAT1*	1.239786612	-0.028042228	-0.018386949	-0.009655279	0.999933887	0.041943882	0.185023521	-0.14307964	0.640898651
ENSG00000206560	*ANKRD28*	5.540791629	-0.072721247	-0.060161017	-0.01256023	0.999933887	-0.47227165	-0.367242066	-0.105029583	0.641106997
ENSG00000134183	*GNAT2*	0.600415698	0.003713713	0.055883708	-0.052169995	0.999933887	-0.362496017	-0.683997782	0.321501766	0.641200635
ENSG00000113312	*TTC1*	4.623439175	-0.020978266	-0.044817381	0.023839116	0.999933887	-0.047419204	0.014329803	-0.061749007	0.641283886
ENSG00000262049	*AC139530.1*	0.637198406	0.048144453	0.02531444	0.022830013	0.999933887	-0.145989933	0.045693828	-0.191683761	0.641283886
ENSG00000089335	*ZNF302*	1.826925628	-0.134562464	-0.176622407	0.042059943	0.999933887	-0.082196723	0.033612108	-0.115808831	0.641338879
ENSG00000131116	*ZNF428*	1.062866763	0.094376164	0.044625916	0.049750248	0.999933887	0.222606029	0.0533121	0.16929393	0.641347779
ENSG00000172687	*ZNF738*	2.596729386	0.027691594	0.046722342	-0.019030748	0.999933887	0.043033127	0.156430444	-0.113397317	0.641385238
ENSG00000173193	*PARP14*	7.833282839	-0.001265818	-0.046315582	0.045049764	0.999933887	-0.160781655	-0.060810975	-0.099970681	0.641385238
ENSG00000197629	*MPEG1*	7.136190188	-0.103345275	-0.072575388	-0.030769888	0.999933887	-0.650006841	-0.75337486	0.103368018	0.641416035
ENSG00000172965	*MIR4435-2HG*	4.572156638	-0.063339328	0.00020068	-0.063540008	0.999933887	-0.286625967	-0.206249084	-0.080376883	0.641416035

ENSG00000271869	*AC026979.2*	2.19179764	0.137488695	-0.090395544	0.227884239	0.999933887	0.04114172	-0.08410873	0.125250451	0.641498543
ENSG00000246273	*SBF2-AS1*	0.183196957	-0.067073942	0.212180437	-0.279254378	0.999933887	0.249607436	0.406779495	-0.157172059	0.641498543
ENSG00000228839	*PIK3IP1-DT*	0.241932784	-0.044684737	-0.337164589	0.292479852	0.999933887	0.889875046	1.040948925	-0.151073879	0.641498543
ENSG00000234420	*ZNF37BP*	3.079862478	0.018244071	-0.058391285	0.076635355	0.999933887	-0.157576336	-0.032827118	-0.124749218	0.641498543
ENSG00000183688	*RFLNB*	6.85790862	-0.104963713	-0.039120344	-0.06584337	0.999933887	-0.960544705	-1.060234875	0.09969017	0.641557918
ENSG00000053371	*AKR7A2*	1.520701353	0.092012965	-0.096116514	0.188129479	0.999933887	0.100422395	-0.034656046	0.13507844	0.641863064
ENSG00000157107	*FCHO2*	4.65907476	-0.006483469	-0.193866071	0.187382601	0.999933887	-1.095910555	-0.923469292	-0.172441264	0.641932599
ENSG00000237883	*DGUOK-AS1*	0.529512097	-0.010730506	-0.185318433	0.174587926	0.999933887	0.054638552	0.256629858	-0.201991305	0.641974983
ENSG00000152256	*PDK1*	4.368038377	-0.055484735	-0.010816721	-0.044668015	0.999933887	-0.419844882	-0.495374199	0.075529317	0.642239382
ENSG00000122512	*PMS2*	1.895453607	-0.026720621	-0.048176545	0.021455924	0.999933887	-0.286932694	-0.174800752	-0.112131942	0.642261589
ENSG00000268218	*AC137932.3*	2.647911143	-0.02074224	-0.017155031	-0.003587209	0.999933887	-0.438023356	-0.562813667	0.12479031	0.642261589
ENSG00000283199	*C13orf46*	1.622070231	0.119253788	-0.021404888	0.140658676	0.999933887	-0.13246514	0.027027873	-0.159493013	0.642486108
ENSG00000109436	*TBC1D9*	0.186243165	0.140335092	0.035858372	0.10447672	0.999933887	0.899616496	0.721403264	0.178213232	0.642543
ENSG00000104524	*PYCR3*	0.013633326	0.025893624	0.009308209	0.016585415	0.999933887	0.170854557	0.342253787	-0.17139923	0.642578561
ENSG00000229692	*SOS1-IT1*	-1.340275865	-0.097846482	-0.824417334	0.726570852	0.999933887	-0.303304937	-0.05017457	-0.253130367	0.642684226
ENSG00000178035	*IMPDH2*	4.117580311	-0.049211502	0.008874625	-0.058086127	0.999933887	0.119258574	0.050277951	0.068980623	0.642776056
ENSG00000187950	*OVCH1*	-0.863158692	-0.232681962	0.351828151	-0.584510113	0.999933887	0.609810539	0.380534284	0.229276255	0.642776056
ENSG00000065615	*CYB5R4*	6.769342319	-0.049916335	-0.062672824	0.012756489	0.999933887	-0.431292768	-0.370908196	-0.060384571	0.642824816
ENSG00000161381	*PLXDC1*	1.330674242	0.023744839	0.087280781	-0.063535942	0.999933887	-0.085424144	0.050709521	-0.136133665	0.642932801
ENSG00000166136	*NDUFB8*	0.701468146	-0.009633144	0.19932531	-0.208958454	0.999933887	0.124308568	0.284764364	-0.160455796	0.643054879
ENSG00000120699	*EXOSC8*	1.765642418	-0.062901792	-0.110754574	0.047852782	0.999933887	0.136218183	0.245656492	-0.109438309	0.64314144
ENSG00000260917	*AL158212.3*	1.374580351	-0.040240085	-0.071278394	0.031038309	0.999933887	-0.101339388	0.019403366	-0.120742754	0.64344373
ENSG00000151687	*ANKAR*	-0.160719439	-0.026600374	0.100887433	-0.127487807	0.999933887	-0.26239117	-0.026904698	-0.235486472	0.643803552
ENSG00000140807	*NKD1*	1.596047882	-0.177527772	0.11730267	-0.294830442	0.999291887	-0.036616044	0.10873682	-0.145352864	0.643868867
ENSG00000171566	*PLRG1*	3.228580543	-0.016316388	-0.106699652	0.090383264	0.999933887	-0.099136797	-0.0216727	-0.077464098	0.643868867
ENSG00000131652	*THOC6*	2.002085795	0.044287368	-0.021714802	0.06600217	0.999933887	-0.184870832	-0.312936515	0.128065683	0.644132422
ENSG00000155016	*CYP2U1*	-0.727952936	0.01505719	-0.09651493	0.111572121	0.999933887	0.046856212	-0.177809349	0.224665561	0.644688125
ENSG00000160226	*CFAP410*	2.146329569	0.124762617	0.224803204	-0.100040587	0.999933887	0.13208448	0.236374916	-0.104290436	0.644854267
ENSG00000130520	*LSM4*	3.197215349	0.021883019	-0.139447272	0.16133029	0.999933887	0.141587546	0.053521688	0.088065858	0.644939107
ENSG00000167004	*PDIA3*	6.014132797	-0.056957308	-0.089456989	0.032499681	0.999933887	0.087624107	0.148653408	-0.061029301	0.645123559
ENSG00000064933	*PMS1*	1.546249882	0.10392523	-0.214503558	0.318428788	0.999933887	-0.268607816	-0.130364155	-0.138243662	0.645793615
ENSG00000234534	*CSNK1G2P1*	-1.767996286	0.033119323	-0.022359025	0.055478348	0.999933887	0.932986126	0.353917832	0.579068294	0.645793615
ENSG00000060491	*OGFR*	7.308877859	0.102929894	0.066079881	0.036850013	0.999933887	0.24828583	0.335063871	-0.086778041	0.645796055
ENSG00000134824	*FADS2*	-0.096963161	0.127254763	0.137965499	-0.010710736	0.999933887	-0.245584369	-0.026767133	-0.218817236	0.645796055
ENSG00000166133	*RPUSD2*	0.640895818	0.061628507	-0.029907232	0.091535739	0.999933887	-0.055718945	0.11259643	-0.168315375	0.645796055
ENSG00000100410	*PHF5A*	3.895323751	-0.014187116	0.093339821	-0.107526937	0.999933887	-0.038647624	-0.112414808	0.073767183	0.645796055
ENSG00000187630	*DHRS4L2*	-0.490091885	-0.022024255	-0.153040807	0.131016552	0.999933887	-0.116507836	0.141128864	-0.257636699	0.645796055
ENSG00000213672	*NCKIPSD*	1.054387179	0.009992409	-0.043148353	0.053140762	0.999933887	0.027791473	-0.123874123	0.151665596	0.645796055
ENSG00000178917	*ZNF852*	0.816521811	-0.018781584	-0.021015871	0.002234288	0.999933887	-0.082229496	0.082055196	-0.164284692	0.645810199
ENSG00000168040	*FADD*	2.24002188	-0.081864665	-0.129933085	0.048068419	0.999933887	-0.643752268	-0.798753065	0.155000797	0.645854873
ENSG00000264772	*AC016876.2*	-0.902787829	0.022491095	-0.066002514	0.088493609	0.999933887	0.721844293	1.027455379	-0.305611086	0.645968214
ENSG00000163041	*H3-3A*	9.350808237	0.036078867	0.047348846	-0.011269979	0.999933887	-0.265480622	-0.209137885	-0.056342737	0.646009477
ENSG00000215256	*DHRS4-AS1*	-0.080159513	-0.297378231	-0.030129758	-0.267248472	0.999933887	0.134729917	-0.07402815	0.208758067	0.646107154
ENSG00000206053	*JPT2*	1.852070129	-0.133288865	0.079307199	-0.212596064	0.999933887	0.010310671	-0.106512734	0.116823405	0.646107154
ENSG00000231500	*RPS18*	8.082746848	-0.000166521	-0.033026128	0.032859607	0.999933887	0.118675677	0.186538796	-0.067863119	0.646107154
ENSG00000175221	*MED16*	5.204430224	0.036276207	-0.00683254	0.043108747	0.999933887	-0.023894831	-0.092224463	0.068329633	0.646118957
ENSG00000012822	*CALCOCO1*	6.887258003	0.005372388	0.011623152	-0.006250764	0.999933887	-0.533483461	-0.59931343	0.065829968	0.646118957
ENSG00000164944	*VIRMA*	4.333099862	-0.037248902	-0.113251157	0.076002255	0.999933887	-0.394305031	-0.318977594	-0.075327437	0.646124752
ENSG00000256053	*COA8*	1.053779895	-0.02360034	-0.052545282	0.028944942	0.999933887	-0.274616494	-0.418157252	0.143540758	0.646124752
ENSG00000230099	*TRBV5-4*	0.010116364	-0.0190569	0.126298802	-0.145355702	0.999933887	0.284987303	0.112466461	0.172520842	0.646124752
ENSG00000171159	*C9orf16*	4.069686893	0.069480695	0.030199751	0.039280944	0.999933887	0.096705193	0.013024243	0.08368095	0.646143318
ENSG00000232859	*LYRM9*	-0.075386915	-0.136272135	0.197552864	-0.333824999	0.999933887	0.039223648	-0.163339371	0.202563019	0.646208399
ENSG00000160447	*PKN3*	0.200449675	0.012886359	0.156454929	-0.14356857	0.999933887	-0.077616092	0.156466579	-0.234082671	0.646270957
ENSG00000212283	*SNORD89*	0.411317059	0.146147513	-0.024063301	0.170210814	0.999933887	-0.674619056	-0.420657933	-0.253961123	0.646383214
ENSG00000280388	*AC006330.1*	-0.038228697	0.13673933	-0.366833826	0.503573156	0.999291887	0.168049806	-0.05672313	0.224772935	0.64651468
ENSG00000124942	*AHNAK*	9.156601098	-0.04660613	-0.086563257	0.039957127	0.999933887	0.039842237	-0.035237211	0.075079448	0.646759335
ENSG00000148700	*ADD3*	6.017231846	0.004442725	-0.087040367	0.091483093	0.999933887	-0.351864948	-0.457596347	0.105731399	0.646759335
ENSG00000234290	*AC116366.1*	4.23726952	0.070667817	0.055723751	0.014944066	0.999933887	0.025884896	-0.062767423	0.088652319	0.647039105
ENSG00000123395	*ATG101*	2.182019925	0.133813765	0.063825989	0.069987776	0.999933887	0.220778606	0.336567356	-0.115788749	0.647039105
ENSG00000236287	*ZBED5*	4.261297962	-0.074937178	-0.055491956	-0.019445222	0.999933887	-0.402446363	-0.500933956	0.098487592	0.647039105
ENSG00000103064	*SLC7A6*	3.093992482	-0.024514612	-0.102549271	0.078034659	0.999933887	0.029652708	-0.043989182	0.07364189	0.647039105
ENSG00000140743	*CDR2*	3.348767923	0.006119369	0.114417178	-0.108297809	0.999933887	-0.042709025	0.05784704	-0.100556065	0.647039105
ENSG00000204099	*NEU4*	0.699709845	-0.008231994	0.072805906	-0.081037901	0.999933887	1.641755456	1.828885035	-0.187129579	0.647039105
ENSG00000129493	*HEATR5A*	2.195400802	-0.039983859	-0.030772149	-0.00921171	0.999933887	-0.408806599	-0.532361489	0.12355489	0.647053388
ENSG00000080839	*RBL1*	1.301147427	-0.142666299	0.055205283	-0.197871582	0.999933887	-0.138300498	-0.003558652	-0.134741846	0.647151965
ENSG00000133030	*MPRIP*	5.724169324	0.041593177	0.056626807	-0.01503363	0.999933887	0.034134519	0.082715278	-0.048580759	0.647151965
ENSG00000166411	*IDH3A*	1.236011137	-0.089944694	0.241599517	-0.331544211	0.981460244	0.092830358	0.210109923	-0.117279565	0.647261534
ENSG00000273828	*AL133227.1*	0.86000109	0.115415033	0.131225979	-0.015810946	0.999933887	-0.064147672	-0.253441846	0.189294174	0.647261534
ENSG00000170854	*RIOX2*	0.794751932	-0.078643675	0.134431541	-0.213075216	0.999933887	0.082519588	0.21978599	-0.137266402	0.647261534
ENSG00000115415	*STAT1*	8.758415737	-0.057733438	-0.076050108	0.01831667	0.999933887	-0.387544323	-0.447045067	0.059500744	0.647307443
ENSG00000255306	*AC004923.4*	-0.134110768	-0.218190584	-0.297922152	0.079731568	0.999933887	-0.458761808	-0.246744399	-0.212017409	0.647307443
ENSG00000257497	*AC121761.1*	0.475260987	0.128269759	0.409016321	-0.280746562	0.999933887	-0.054555972	-0.278620726	0.224064754	0.647307443
ENSG00000230280	*HNRNPA1P59*	-0.211403659	-0.095811565	0.077908988	-0.173720553	0.999933887	0.57361169	0.729985072	-0.156373381	0.647333552
ENSG00000141456	*PELP1*	3.517728239	-0.031175671	0.041082982	-0.072258653	0.999933887	0.150846837	0.085115029	0.065731807	0.647426045
ENSG00000166927	*MS4A7*	0.631089694	0.024640942	0.137167409	-0.112526468	0.999933887	-0.557751764	-0.365877745	-0.191874019	0.647426045
ENSG00000122140	*MRPS2*	1.20416515	-0.00328837	-0.017058584	0.013770214	0.999933887	0.122481895	0.270129593	-0.147647698	0.647426045
ENSG00000229807	*XIST*	4.459609562	-0.073098005	-0.055804666	-0.017293339	0.999933887	-0.078730412	0.084704984	-0.163435396	0.647645376
ENSG00000169432	*SCN9A*	-0.568197481	-0.023965551	0.204587233	-0.228552784	0.999933887	-0.174980742	0.173958757	-0.348939499	0.647645376
ENSG00000188312	*CENPP*	0.848789203	-0.019063011	0.102986628	-0.122049639	0.999933887	0.376783814	0.540525704	-0.163741891	0.647655261
ENSG00000136937	*NCBP1*	3.603804345	-0.027371845	-0.003291331	-0.024080514	0.999933887	0.001213303	0.076136849	-0.074923545	0.647707737
ENSG00000184232	*OAF*	-0.457832383	0.207691698	-0.005071183	0.212762881	0.999933887	-0.383955993	-0.172611415	-0.211344577	0.64772267
ENSG00000170502	*NUDT9*	0.732348744	0.061604755	0.146199661	-0.084594906	0.999933887	0.013085822	0.162638047	-0.149552225	0.64772267
ENSG00000126709	*IFI6*	6.777794604	0.07443717	0.01550315	0.058934021	0.999933887	-0.135770937	-0.225033032	0.089262095	0.647775734
ENSG00000178146	*GK4P*	-0.053158507	-0.176947233	-0.076090751	-0.100856482	0.999933887	0.242214241	0.440940124	-0.198725883	0.648097132
ENSG00000267002	*AC060780.1*	3.005925312	-0.040191362	-0.052357969	0.012166607	0.999933887	-0.104450423	-0.194548251	0.090097828	0.648160502
ENSG00000075702	*WDR62*	0.24539964	0.043360406	0.018049515	0.025310891	0.999933887	0.156877628	0.358823301	-0.201945673	0.648160502
ENSG00000139726	*DENR*	4.773168801	-0.066508867	-0.050264469	-0.016244398	0.999933887	-0.362457723	-0.304186717	-0.058271006	0.648235457
ENSG00000112309	*B3GAT2*	-0.543570228	0.134029212	0.029964006	0.104065206	0.999933887	-0.60461713	-0.300344387	-0.304272743	0.648235457
ENSG00000163958	*ZDHHC19*	0.929678793	0.146687599	-0.20346018	0.350147779	0.999933887	-0.267603385	-0.047333513	-0.220269872	0.648265166
ENSG00000160307	*S100B*	-1.395269519	0.159020745	-0.190639472	0.349660217	0.999933887	0.283536378	0.618006453	-0.334470076	0.64830231
ENSG00000117643	*MAN1C1*	1.810160947	-0.077604856	0.129137459	-0.206742315	0.999933887	-0.131856184	-0.224341559	0.092485375	0.64830231
ENSG00000235313	*HM13-IT1*	1.187640258	-0.067594034	-0.131130485	0.063536452	0.999933887	-0.101016675	0.049994104	-0.151010779	0.64830231
ENSG00000184787	*UBE2G2*	4.813445265	-0.016383159	0.000165012	-0.016548171	0.999933887	0.004027477	-0.043620474	0.047647951	0.64830231
ENSG00000082458	*DLG3*	1.529772908	0.054501941	-0.056429328	0.110931269	0.999933887	-0.124995955	0.009127577	-0.134123532	0.648352867
ENSG00000176102	*CSTF3*	1.84889248	-0.08227305	-0.057129984	-0.025143067	0.999933887	0.118911619	0.228805989	-0.10989437	0.648543107
ENSG00000250831	*AC074131.1*	-0.801550866	0.048365245	0.139911973	-0.091546728	0.999933887	0.545623966	0.74013641	-0.194512444	0.648543107
ENSG00000084676	*NCOA1*	7.828479638	-0.009485665	-0.076101966	0.066616301	0.999933887	-0.097677012	-0.012887035	-0.084789977	0.648543107
ENSG00000137760	*ALKBH8*	0.705566047	-0.06865131	-0.124661181	0.056009871	0.999933887	0.030476756	0.17794651	-0.147469754	0.648616516
ENSG00000185651	*UBE2L3*	4.713173646	-0.01725692	0.008545259	-0.025802179	0.999933887	-0.260320651	-0.330471767	0.070151116	0.648800687
ENSG00000226334	*AL359182.1*	1.291171791	0.122804112	0.394114608	-0.271310496	0.999933887	0.400160424	0.238962502	0.161197922	0.648800716
ENSG00000160957	*RECQL4*	0.416383843	0.096034315	0.04653352	0.049500795	0.999933887	0.356101601	0.1458679	0.210233701	0.648800716
ENSG00000239557	*PPP2R5CP*	0.526011878	0.016510624	-0.024730855	0.041241479	0.999933887	0.159482658	-0.087386865	0.246869524	0.648928032
ENSG00000203730	*TEDDM1*	-0.127311717	0.160857722	0.221421843	-0.060564121	0.999933887	0.806456565	0.552646786	0.25380978	0.648984204
ENSG00000111886	*GABRR2*	2.650123102	-0.055842828	0.003259294	-0.059102122	0.999933887	0.107936247	0.204004413	-0.096068166	0.648984204
ENSG00000159685	*CHCHD6*	-0.458509237	0.119988992	0.255776816	-0.135787824	0.999933887	0.16345895	-0.029431429	0.192890379	0.648984204

ENSG00000236278	*PEBP1P3*	1.503037657	0.100777513	0.318614034	-0.21783652	0.999933887	0.467815004	0.70408337	-0.236268366	0.649147084
ENSG00000151929	*BAG3*	2.156885687	0.038885826	-0.090378664	0.12926449	0.999933887	0.111671813	0.223249234	-0.111577421	0.649147084
ENSG00000198546	*ZNF511*	1.293786453	-0.011216972	-0.079854648	0.068637676	0.999933887	-0.059038085	-0.202651004	0.143612919	0.649147084
ENSG00000164818	*DNAAF5*	1.722178931	-0.083892634	0.020951978	-0.104844612	0.999933887	0.06334861	0.165494642	-0.102146032	0.649273826
ENSG00000225720	*AL031846.1*	-0.732206701	0.004431885	0.201915659	-0.197483774	0.999933887	-0.056238642	0.213105863	-0.269344506	0.649273826
ENSG00000085552	*IGSF9*	0.359985969	0.266688305	0.277264658	-0.010576353	0.999933887	0.586788362	0.920186009	-0.333397647	0.649307821
ENSG00000242689	*CNTF*	-0.419321377	-0.238978626	-0.139500009	-0.099478617	0.999933887	-0.03344826	-0.322556624	0.289108363	0.649380513
ENSG00000101391	*CDK5RAP1*	2.510174956	-0.040172127	0.009146557	-0.049318684	0.999933887	0.055104458	-0.038779889	0.093884347	0.649610992
ENSG00000113719	*ERGIC1*	8.090084261	0.012135442	-0.004522107	0.016657549	0.999933887	0.216759479	0.271525221	-0.054765742	0.649610992
ENSG00000147123	*NDUFB11*	3.473198931	0.06753027	-0.030943934	0.098474204	0.999933887	0.156512623	0.057575682	0.098936941	0.649957049
ENSG00000203705	*TATDN3*	2.183750706	-0.087165686	-0.134809342	0.047643656	0.999933887	-0.122616488	-0.021558912	-0.101057575	0.650077841
ENSG00000259959	*AC107068.1*	0.129016227	-0.136357733	-0.067573564	-0.068784169	0.999933887	-0.362179706	-0.63615969	0.273979984	0.650077841
ENSG00000196504	*PRPF40A*	5.431111407	-0.075037782	-0.145624864	0.070587083	0.999933887	-0.49691437	-0.367040729	-0.129873641	0.650077841
ENSG00000146085	*MMUT*	2.390267251	-0.019393545	0.032423297	-0.051816842	0.999933887	-0.130660898	-0.033360437	-0.09730046	0.650077841
ENSG00000100292	*HMOX1*	8.57530175	-0.024614562	0.007008037	-0.031622599	0.999933887	-0.800574063	-0.654410925	-0.146163138	0.650077841
ENSG00000174282	*ZBTB4*	4.81884118	0.08557869	0.082922696	0.002655994	0.999933887	-0.001018428	-0.073601189	0.072582761	0.65014835
ENSG00000109184	*DCUN1D4*	2.779177967	-0.069359963	0.004934464	-0.074294426	0.999933887	-0.24752587	-0.13425006	-0.11327581	0.65014835
ENSG00000103544	*VPS35L*	2.845035596	-0.056456975	-0.0035691	-0.052887875	0.999933887	0.002930965	0.088595953	-0.085664988	0.650455983
ENSG00000229894	*GK3P*	-0.276270321	0.019353838	0.286885138	-0.2675313	0.999933887	0.072489003	-0.196262243	0.268751246	0.650455983
ENSG00000100079	*LGALS2*	-1.458949269	-0.007184328	0.049179081	-0.056363409	0.999933887	-0.150481769	-0.488383596	0.337901827	0.650455983
ENSG00000128872	*TMOD2*	3.384211376	-0.002143742	0.072661008	-0.07480475	0.999933887	-0.199178977	-0.102118889	-0.097060088	0.650558538
ENSG00000147133	*TAF1*	5.153655437	-0.056875103	-0.049875337	-0.006999766	0.999933887	-0.236162339	-0.168736928	-0.067425412	0.650642339
ENSG00000257365	*FNTB*	0.672027717	-0.004074994	0.142641062	-0.146716056	0.999933887	-0.756100759	-0.922654754	0.166553995	0.650642339
ENSG00000261067	*AC109460.3*	1.002561248	5.3472e-05	0.189304727	-0.189251255	0.999933887	0.391672173	0.512921048	-0.121248875	0.650642339
ENSG00000196118	*CCDC189*	0.083363608	-0.192586472	0.051034231	-0.243620703	0.999933887	-0.381786998	-0.639874362	0.258087364	0.650999113
ENSG00000168612	*ZSWIM1*	2.608808814	-0.026723172	-0.214485945	0.187762773	0.999933887	-0.020063275	0.077211291	-0.097274566	0.651092178
ENSG00000008300	*CELSR3*	0.755765162	0.013172872	-0.051458798	0.064631669	0.999933887	0.110623167	0.270498035	-0.159874868	0.651369014
ENSG00000164512	*ANKRD55*	-0.035356232	0.115183741	0.153775633	-0.038591891	0.999933887	-0.330144429	-0.093318511	-0.236825918	0.651394821
ENSG00000223935	*LGALSL-DT*	0.291276799	0.118081798	-0.076735575	0.194817373	0.999933887	-0.487287081	-0.692452551	0.20516547	0.651470634
ENSG00000004897	*CDC27*	3.522976191	-0.037374316	-0.093076468	0.055702151	0.999933887	-0.285268149	-0.197030081	-0.088238068	0.651853454
ENSG00000168026	*TTC21A*	3.245700948	0.175063227	0.260779392	-0.085716166	0.999933887	0.027263821	-0.100697537	0.127961358	0.651944312
ENSG00000038274	*MAT2B*	5.869903533	-0.087024908	-0.015344955	-0.071679953	0.999933887	-0.287844097	-0.345003528	0.057159431	0.651944312
ENSG00000266891	*AP000902.1*	0.887095683	-0.069545726	0.031497076	-0.101042802	0.999933887	-0.149704465	0.087969569	-0.237674034	0.651944312
ENSG00000164109	*MAD2L1*	-0.220557907	-0.14063813	-0.125039593	-0.015598537	0.999933887	0.125703456	-0.119480081	0.245183537	0.651944312
ENSG00000100218	*RSPH14*	-1.13779651	-0.157514909	0.118415467	-0.275930376	0.999933887	-0.283290371	0.165597624	-0.448887995	0.651944312
ENSG00000128050	*PAICS*	2.317614706	-0.057628009	-0.190700991	0.133072983	0.999933887	0.077453445	0.169676617	-0.092223172	0.651944312
ENSG00000115484	*CCT4*	3.803975425	0.013151349	-0.153406486	0.166557835	0.999933887	0.159085798	0.232589628	-0.07350383	0.651944312
ENSG00000116151	*MORN1*	0.812269303	-0.001180145	-0.05531102	0.054130875	0.999933887	0.037561765	-0.117124806	0.154686571	0.651944312
ENSG00000226332	*AL354836.1*	0.632632672	-0.052207554	0.164865788	-0.217073341	0.999933887	-0.096990255	0.035952446	-0.132942701	0.651950052
ENSG00000280161	*AC022413.1*	-0.633392686	0.255721608	-0.171845585	0.427567192	0.999933887	0.703941017	0.937648609	-0.233707593	0.651987844
ENSG00000260672	*AC100827.4*	-1.302534324	-0.052731981	0.243375019	-0.296107	0.999933887	0.088253026	0.51742252	-0.429169493	0.651987844
ENSG00000159176	*CSRP1*	3.631517535	-0.036791754	0.012405305	-0.049197059	0.999933887	-0.096479408	-0.180813122	0.084333714	0.652295545
ENSG00000186409	*CCDC30*	0.720236681	-0.033102237	0.111238436	-0.144340673	0.999933887	-0.81324197	-0.6201169	-0.193125071	0.652295545
ENSG00000124380	*SNRNP27*	2.168743457	-0.010470897	0.06519906	-0.075669958	0.999933887	0.003316176	0.099713355	-0.096397179	0.652316527
ENSG00000100453	*GZMB*	3.970346005	-0.061730152	-0.031202497	-0.030527656	0.999933887	0.103564863	0.02661107	0.076953793	0.652510724
ENSG00000177943	*MAMDC4*	1.20051602	0.072540587	0.102541301	-0.030000713	0.999933887	0.102613524	0.248402643	-0.145789118	0.652510724
ENSG00000169118	*CSNK1G1*	3.60382041	-0.099604591	-0.129744702	0.030140111	0.999933887	-0.355015127	-0.444883114	0.089867987	0.652525561
ENSG00000175063	*UBE2C*	0.238407075	-0.133253279	0.150009827	-0.283263106	0.999933887	-0.135239724	0.081509956	-0.21674968	0.652560575
ENSG00000147536	*GINS4*	0.534137089	0.058777275	0.102093356	-0.04331608	0.999933887	0.635181668	0.851670602	-0.216488934	0.652739936
ENSG00000260803	*Z84723.1*	-1.717019172	0.016793625	-0.411342845	0.42813647	0.999933887	-1.399466128	-1.693854097	0.294387969	0.65277847
ENSG00000223750	*SIRPB3P*	-0.390626716	0.093057696	0.048744894	0.044312802	0.999933887	0.03953706	0.311400647	-0.271863587	0.652815725
ENSG00000166402	*TUB*	-0.503210484	-0.039540959	0.003857718	-0.043398677	0.999933887	-0.108324561	0.141316168	-0.249640729	0.652869219
ENSG00000168234	*TTC39C*	3.536130985	-0.069146157	0.010428361	-0.079574518	0.999933887	0.140904444	0.207028486	-0.066124042	0.653016674
ENSG00000198363	*ASPH*	7.518241954	-0.067297166	0.042099543	-0.109396709	0.999933887	-0.340767311	-0.26074133	-0.080025981	0.653016674
ENSG00000119333	*DYNC2I2*	0.301857918	-0.052864246	-0.187635137	0.134770891	0.999933887	0.023165176	-0.139195537	0.162360713	0.653016674
ENSG00000125249	*RAP2A*	2.546534619	-0.001079827	-0.160808068	0.15972824	0.999933887	-0.189383555	-0.084658124	-0.104725431	0.653016674
ENSG00000174780	*SRP72*	4.186330489	-0.047171989	-0.138521221	0.091349232	0.999933887	-0.047500352	0.031732295	-0.079232647	0.653017894
ENSG00000279155	*AC233300.1*	2.072104473	-0.009907094	0.010201942	-0.020109036	0.999933887	0.078448555	0.211985728	-0.133537173	0.653145858
ENSG00000205707	*ETFRF1*	2.013652288	-0.053121901	-0.094006161	0.04088426	0.999933887	-0.026981523	0.109328994	-0.136310517	0.653202405
ENSG00000066422	*ZBTB11*	4.690019953	-0.039450564	-0.150656444	0.11120588	0.999933887	-0.225827495	-0.083547664	-0.142279832	0.653202405
ENSG00000226380	*AC016831.1*	5.281532819	0.112768884	0.093709238	0.019059646	0.999933887	1.097285216	1.22163647	-0.124351254	0.653244526
ENSG00000119801	*YPEL5*	9.556938214	-0.007930418	-0.068525059	0.060594641	0.999933887	-0.194295902	-0.143952595	-0.050343307	0.653244526
ENSG00000164168	*TMEM184C*	3.496877105	0.016443832	-0.152637423	0.169081255	0.999933887	-0.29887393	-0.389716074	0.090842144	0.653477175
ENSG00000115641	*FHL2*	-0.130955886	0.026007129	-0.100009694	0.126016823	0.999933887	-0.109180039	0.093652977	-0.202833016	0.653696064
ENSG00000085465	*OVGP1*	-0.18370849	0.066755033	0.257271092	-0.190516059	0.999933887	-0.020239055	-0.231265954	0.211026899	0.653931093
ENSG00000166579	*NDEL1*	7.121622275	0.064259259	0.040312546	0.023946713	0.999933887	-0.519894691	-0.57769061	0.057795919	0.654164192
ENSG00000231128	*AL137856.1*	-0.621881791	0.238804122	0.116761264	0.122042857	0.999933887	-0.119056153	0.22230881	-0.341364963	0.654164192
ENSG00000075188	*NUP37*	0.200048938	-0.140091507	-0.180438186	0.040346679	0.999933887	-0.218442946	-0.043774266	-0.17466868	0.654519161
ENSG00000035862	*TIMP2*	7.264975449	0.013201194	0.011141937	0.002059257	0.999933887	-1.334552095	-1.433465222	0.098913128	0.654552881
ENSG00000129422	*MTUS1*	-0.905956295	-0.18630146	-0.104146874	-0.082154587	0.999933887	-0.208039319	0.042925477	-0.250964796	0.65464143
ENSG00000261490	*AC005674.1*	4.165060299	-0.109684498	-0.076768992	-0.032915506	0.999933887	-0.121955522	-0.209293719	0.087338197	0.654731381
ENSG00000146066	*HIGD2A*	3.045190203	-0.007899641	0.002884028	-0.010783668	0.999933887	-0.050458056	-0.149659171	0.099201115	0.655007297
ENSG00000101166	*PRELID3B*	4.150040879	-0.175040611	-0.08608072	-0.08895989	0.999933887	-0.27809608	-0.209117817	-0.068978263	0.655199663
ENSG00000115318	*LOXL3*	1.416920622	0.05416436	-0.048289584	0.102453944	0.999933887	0.484926554	0.327176021	0.157750533	0.655199663
ENSG00000237940	*LINC01238*	0.352679795	-0.055864812	-0.204355506	0.148490694	0.999933887	0.056985897	0.226227078	-0.169241181	0.655199663
ENSG00000156853	*ZNF689*	1.820027752	-0.097018711	-0.106644505	0.009625795	0.999933887	0.121991568	0.006463371	0.115528197	0.655201416
ENSG00000278784	*AL136295.7*	2.024938968	-0.013872758	-0.016825049	0.002952291	0.999933887	-0.020159465	0.085014391	-0.105173856	0.655497244
ENSG00000124357	*NAGK*	8.657108335	-0.059745772	-0.053235048	-0.006510723	0.999933887	-0.35428423	-0.295422958	-0.058861272	0.655781868
ENSG00000129559	*NEDD8*	3.42742781	-0.074263182	0.00579397	-0.080057152	0.999933887	-0.031610659	-0.122498364	0.090887705	0.655781868
ENSG00000179941	*BBS10*	0.914819718	0.146415328	-0.122266325	0.268681653	0.999933887	-0.267423379	-0.397370486	0.129947107	0.655781868
ENSG00000164885	*CDK5*	0.172212322	-0.121302809	0.287483665	-0.408786473	0.999933887	0.093410362	-0.093459203	0.186869565	0.655781868
ENSG00000135624	*CCT7*	4.469420927	-0.037667358	-0.024280087	-0.013387271	0.999933887	0.128900406	0.068788354	0.060112052	0.655781868
ENSG00000246922	*UBAP1L*	1.456930951	0.013579336	0.247896155	-0.234316819	0.999933887	-0.017191328	0.085481444	-0.102672772	0.655781868
ENSG00000108298	*RPL19*	8.128588081	0.001126851	-0.061256333	0.062383184	0.999933887	0.050569505	0.111018018	-0.060448513	0.655781868
ENSG00000177685	*CRACR2B*	0.295315238	0.118543821	0.07742518	0.041118641	0.999933887	-0.059110965	-0.256531088	0.197420123	0.655786268
ENSG00000117280	*RAB29*	4.394165636	-0.02793047	0.082127912	-0.110058382	0.999933887	0.032156582	-0.024193598	0.05635018	0.655811725
ENSG00000187676	*B3GLCT*	1.022392985	0.024953126	-0.159277138	0.184230264	0.999933887	-0.037067726	-0.199545953	0.162478227	0.655811725
ENSG00000183682	*BMP8A*	0.09546316	0.127557815	0.176397309	-0.048839494	0.999933887	-0.071191071	0.112917556	-0.184108627	0.656252247
ENSG00000204272	*NBDY*	1.090425542	-0.032971298	-0.155998201	0.123026903	0.999933887	0.119019371	0.257569501	-0.13855013	0.656252247
ENSG00000143190	*POU2F1*	5.345889546	-0.09330034	0.001975818	-0.095276158	0.999933887	-0.444279359	-0.34714872	-0.097130638	0.656365763
ENSG00000095002	*MSH2*	1.165827863	0.000479184	-0.013935774	0.014414958	0.999933887	-0.036824798	0.120715544	-0.157540342	0.656636771
ENSG00000182557	*SPNS3*	2.654005015	-0.022074133	0.003899839	-0.025973972	0.999933887	-0.068652542	-0.178322262	0.10966972	0.656642886
ENSG00000258365	*AC073655.2*	1.618699283	0.207615083	-0.021738902	0.229353985	0.999933887	0.737965838	0.895693899	-0.157728062	0.65683246
ENSG00000113812	*ACTR8*	2.481819265	0.087063908	-0.024865075	0.111928983	0.999933887	-0.06954646	-0.157817873	0.088271413	0.65683246
ENSG00000169131	*ZNF354A*	1.717858984	-0.114876232	-0.125822323	0.010946092	0.999933887	-0.302705359	-0.173519858	-0.129185501	0.65683246
ENSG00000205918	*PDPK2P*	0.552346092	0.103079899	0.083846311	0.019233588	0.999933887	-0.213216952	-0.378776559	0.165559607	0.65683246
ENSG00000197713	*RPE*	1.334751328	-0.108552029	0.061568626	-0.170120654	0.999933887	-0.075693203	-0.214781677	0.139088474	0.657025986
ENSG00000171604	*CXXC5*	0.695517473	0.067363354	0.061265941	0.006097412	0.999933887	-0.056896985	-0.22567177	0.168774785	0.657046329
ENSG00000100483	*VCPKMT*	3.674423188	-0.066621715	-0.062072458	-0.004549257	0.999933887	-0.336136844	-0.456397933	0.120261088	0.657263189
ENSG00000271605	*MILR1*	1.130178573	-0.053925697	0.002484122	-0.056409819	0.999933887	0.054242778	0.264640129	-0.210397351	0.657356768
ENSG00000171469	*ZNF561*	2.312707967	0.121874707	0.027916155	0.093958552	0.999933887	-0.168802973	-0.278032839	0.109229867	0.657589896
ENSG00000162543	*UBXN10*	-0.984186913	-0.167181187	0.068983672	-0.236164858	0.999933887	-0.330543185	-0.044041106	-0.28650208	0.657589896
ENSG00000186376	*ZNF75D*	2.768941091	0.073504002	0.006966825	0.066537177	0.999933887	-0.144831645	-0.241826479	0.096994835	0.657589896
ENSG00000188021	*UBQLN2*	5.445039427	0.029996121	0.015117535	0.014878587	0.999933887	-0.285905611	-0.346571692	0.060666081	0.657589896
ENSG00000014641	*MDH1*	2.97380758	-0.006190445	-0.216867413	0.210676968	0.999933887	0.039106763	0.123702481	-0.084595719	0.657589896

ENSG00000143458	*GABPB2*	2.722653036	-0.003768357	0.121966731	-0.125735088	0.999933887	-0.014658903	-0.108838156	0.094179253	0.657589896
ENSG00000164008	*C1orf50*	0.367269793	-0.005680819	0.327621692	-0.333302511	0.999933887	-0.01906601	0.159679468	-0.178745478	0.657589896
ENSG00000170571	*EMB*	5.623312011	0.040283127	-0.124010174	0.164293301	0.999933887	-0.179797848	-0.087363272	-0.092434576	0.657887078
ENSG00000271888	*AL136162.1*	0.240067882	-0.024053759	0.017750027	-0.041803786	0.999933887	0.358501299	0.122886782	0.235614518	0.658248169
ENSG00000235903	*CPB2-AS1*	3.192798625	0.24343985	0.412998364	-0.169558513	0.999933887	0.830534727	1.029072611	-0.198537883	0.658767384
ENSG00000230539	*AOAH-IT1*	0.347287595	0.216643129	0.000427832	0.216215297	0.999933887	0.750691091	1.037791741	-0.28710065	0.65878179
ENSG00000008869	*HEATR5B*	4.478705033	0.042656114	-0.140646259	0.183302373	0.999933887	-0.10612691	-0.184205811	0.078078901	0.65878179
ENSG00000205250	*E2F4*	5.813035564	0.01922799	0.001793093	0.017434897	0.999933887	-0.024629394	-0.071439295	0.046809901	0.65878179
ENSG00000241945	*PWP2*	1.443343374	-0.040191997	0.152112379	-0.192304376	0.999933887	-0.034489492	-0.193291632	0.15880214	0.65878179
ENSG00000149428	*HYOU1*	4.357973185	-0.005461423	0.032820299	-0.038281722	0.999933887	0.164608035	0.227579307	-0.062971272	0.65878179
ENSG00000161929	*SCIMP*	-0.97168561	-0.179870878	-0.01845988	-0.161410998	0.999933887	-0.461128769	-0.217298232	-0.243830537	0.658878298
ENSG00000232295	*AL589935.1*	0.321687428	0.045270571	-0.147694891	0.192965462	0.999933887	0.190421052	-0.004502578	0.19492363	0.658878298
ENSG00000107758	*PPP3CB*	4.029557981	-0.032568577	0.109004284	-0.14157286	0.999933887	-0.138159465	-0.203156199	0.064996734	0.658920515
ENSG00000139182	*CLSTN3*	4.873323779	-0.006448059	0.061916137	-0.068364196	0.999933887	0.112580125	0.053893201	0.058686924	0.658920515
ENSG00000282173	*TRBJ1-5*	0.494570503	-0.258885772	0.146417307	-0.405303079	0.999933887	0.245507473	0.084126096	0.161381377	0.659114583
ENSG00000147155	*EBP*	1.670088875	-0.112147573	-0.109511825	-0.002635748	0.999933887	0.031985792	0.213495721	-0.181509929	0.659114583
ENSG00000279108	*AC008537.3*	0.717614055	-0.097579128	0.102821084	-0.200400212	0.999933887	-0.103993232	0.046087209	-0.150080441	0.659114583
ENSG00000033867	*SLC4A7*	3.804425761	-0.003654142	-0.106586416	0.102932274	0.999933887	-0.413897627	-0.269419814	-0.144477813	0.659146694
ENSG00000153487	*ING1*	3.981123551	0.143597161	0.099398178	0.044198983	0.999933887	-0.186620513	-0.25344729	0.066826777	0.659465202
ENSG00000205903	*ZNF316*	9.737443674	0.086072965	0.049610363	0.036462601	0.999933887	0.51914286	0.608244254	-0.089101393	0.659483874
ENSG00000080819	*CPOX*	1.757777016	0.105936096	-0.041066003	0.1470021	0.999933887	0.086753331	0.180642985	-0.093889654	0.659508442
ENSG00000120662	*MTRF1*	1.801769166	-0.060952541	-0.104412063	0.043459522	0.999933887	-0.19706326	-0.096440181	-0.100623079	0.659508442
ENSG00000158290	*CUL4B*	5.696572547	-0.034187089	-0.106166433	0.071979344	0.999933887	-0.700936511	-0.622389104	-0.078547408	0.659508442
ENSG00000123739	*PLA2G12A*	0.208490972	0.07778966	-0.261592153	0.339381813	0.999933887	0.155883011	-0.059910898	0.215793909	0.659508442
ENSG00000186470	*BTN3A2*	6.569840275	-0.004864589	-0.051458316	0.046593727	0.999933887	0.015091541	0.072115036	-0.057023495	0.659508442
ENSG00000145349	*CAMK2D*	2.536232156	-0.005438317	-0.108221849	0.102783532	0.999933887	-0.100846968	-0.00354408	-0.097302888	0.659531961
ENSG00000242190	*AC017015.2*	-0.771405418	0.43639207	0.292984007	0.143408063	0.999933887	2.082116402	1.778910788	0.303205614	0.659546756
ENSG00000198121	*LPAR1*	-0.904468749	0.064791719	0.303458528	-0.238666809	0.999933887	-0.575948979	-0.268793132	-0.307155848	0.659546756
ENSG00000117691	*NENF*	1.362670267	0.052256146	-0.088227805	0.140483951	0.999933887	0.277483573	0.158112234	0.119371339	0.659596906
ENSG00000198468	*FLVCR1-DT*	0.594407727	-0.018824665	-0.043175935	0.02435127	0.999933887	0.030063115	0.19267141	-0.162608296	0.659596906
ENSG00000249353	*NPM1P27*	0.402773162	-0.002645939	0.338304946	-0.340950885	0.999933887	0.400596865	0.609420677	-0.208823812	0.659724928
ENSG00000244480	*AC005154.2*	-0.080989955	-0.105842511	-0.134441219	0.028598707	0.999933887	-0.114400948	-0.3119317	0.197530752	0.65989362
ENSG00000181016	*LSMEM1*	4.342935557	-0.058118334	0.005288101	-0.063406436	0.999933887	0.082049985	0.15381226	-0.071762275	0.659966331
ENSG00000090372	*STRN4*	6.62401897	0.043576078	0.075067017	-0.031490939	0.999933887	-0.277219699	-0.344604129	0.067384429	0.660013785
ENSG00000089012	*SIRPG*	2.333021905	-0.001213402	0.087872767	-0.089086169	0.999933887	0.085578762	0.182827736	-0.097248974	0.660275889
ENSG00000140264	*SERF2*	6.564945684	0.036182638	0.033701746	0.002480892	0.999933887	0.340744625	0.287654746	0.053089879	0.660307865
ENSG00000267365	*KCNJ2-AS1*	2.928509551	-0.131757745	-0.110716557	-0.021041187	0.999933887	3.647757798	3.835259138	-0.18750134	0.660333969
ENSG00000269243	*AC008894.2*	-0.094902256	0.058098345	-0.152605876	0.210704221	0.999933887	-0.358015768	-0.559345724	0.201329956	0.660333969
ENSG00000276819	*TRBV15*	-0.993696509	-0.232675236	-0.372979838	0.140304602	0.999933887	0.250523233	0.502495534	-0.251972301	0.660417326
ENSG00000157881	*PANK4*	5.73087863	0.006039437	-0.057018097	0.063057533	0.999933887	0.084679069	0.143545139	-0.058866069	0.660417326
ENSG00000248971	*KRT8P46*	2.095175469	-0.093346735	-0.020528496	-0.072818239	0.999933887	0.209014265	0.324915038	-0.115900774	0.660518281
ENSG00000196843	*ARID5A*	6.984454138	0.024258106	0.056025103	-0.031766998	0.999933887	0.0949032	0.025532204	0.069370997	0.660518281
ENSG00000177483	*RBM44*	0.765051577	0.056016452	-0.124808742	0.180825194	0.999933887	-0.431327414	-0.138183028	-0.293144386	0.660569379
ENSG00000278730	*AC005332.6*	3.171393207	-0.11194567	-0.023583999	-0.088361671	0.999933887	-0.292150173	-0.226393586	-0.065756587	0.660849849
ENSG00000250687	*NAIPP1*	-0.121810139	0.129950828	0.020472209	0.109478619	0.999933887	-0.391997242	-0.661594761	0.269597519	0.660849849
ENSG00000275709	*AC090527.3*	-0.86580323	0.323818802	0.295370124	0.028448679	0.999933887	0.846755934	0.518190355	0.328565579	0.661056314
ENSG00000273445	*AC133644.1*	-0.842020042	0.174428519	0.196059708	-0.021631189	0.999933887	0.149942303	-0.137669733	0.287612036	0.661056314
ENSG00000223642	*AC008277.1*	-0.604277237	0.15693183	0.064335938	0.092595892	0.999933887	-0.763743019	-0.467939872	-0.295803147	0.661056314
ENSG00000049449	*RCN1*	-0.466330998	-0.08800758	0.180591148	-0.268598727	0.999933887	0.223410146	0.004785298	0.218624848	0.661056314
ENSG00000178913	*TAF7*	8.198807485	0.018816901	-0.086633553	0.105450454	0.999933887	0.163659822	0.216685818	-0.053025996	0.661056314
ENSG00000280067	*AC023818.1*	1.04904606	-0.049151892	-0.040052968	-0.009098924	0.999933887	-0.020312324	-0.166765676	0.146453352	0.661056314
ENSG00000060688	*SNRNP40*	2.481057681	0.016663743	-0.212673945	0.229337688	0.999933887	0.090078899	0.005551063	0.084527837	0.661056314
ENSG00000279259	*AC087741.3*	0.587393569	-0.018305151	-0.086599909	0.068294757	0.999933887	-0.164239821	-0.348680537	0.184440715	0.661056314
ENSG00000106211	*HSPB1*	2.05364166	-0.000704061	-0.040948539	0.040244478	0.999933887	0.176974779	0.301029225	-0.124054446	0.661056314
ENSG00000130770	*ATP5IF1*	4.095983805	0.056191632	-0.071438411	0.127630042	0.999933887	0.108236511	0.17997785	-0.071741338	0.661164008
ENSG00000124782	*RREB1*	6.182428254	-0.021219786	0.1013031	-0.122522886	0.999933887	-0.873225099	-0.792089956	-0.081135143	0.661164008
ENSG00000189283	*FHIT*	0.965051689	-0.01023491	-0.005180475	-0.005054436	0.999933887	-0.040990962	0.100089465	-0.141080427	0.661164008
ENSG00000147251	*DOCK11*	7.012625751	-0.009331713	-0.126823749	0.117492036	0.999933887	-0.806585956	-0.683757397	-0.122828559	0.661328112
ENSG00000287510	*AL137857.1*	-0.682733818	0.155072905	0.153800488	0.001272417	0.999933887	-0.135924268	0.191746474	-0.327670742	0.661500843
ENSG00000163736	*PPBP*	-0.107000213	-0.044334633	-0.061224571	0.016889938	0.999933887	0.019686162	0.241950562	-0.2222644	0.661752189
ENSG00000152642	*GPD1L*	1.523112729	0.013576653	0.11899546	-0.105418807	0.999933887	0.0716936	-0.049256715	0.120950316	0.661781861
ENSG00000100014	*SPECC1L*	2.639736305	0.002678908	-0.071844573	0.074523481	0.999933887	0.278509382	0.390277832	-0.11176845	0.661781861
ENSG00000181804	*SLC9A9*	0.99625866	0.048082778	-0.037375713	0.085458491	0.999933887	0.012538029	0.156778106	-0.144240077	0.661822893
ENSG00000160439	*RDH13*	2.059884879	0.07848749	0.06589463	0.012592861	0.999933887	0.332905209	0.433357109	-0.1004519	0.662010314
ENSG00000220205	*VAMP2*	7.034849066	0.047606532	0.102733667	-0.055127135	0.999933887	0.150251235	0.098222514	0.052028721	0.662300017
ENSG00000260804	*LINC01963*	0.328144947	-0.227534078	0.037722678	-0.265256756	0.999933887	-0.101984146	0.088750222	-0.190734368	0.662371654
ENSG00000111801	*BTN3A3*	4.953248049	0.022717243	-0.053017487	0.075734729	0.999933887	-0.013464764	-0.092584767	0.079120003	0.662489176
ENSG00000119655	*NPC2*	7.283350073	-0.060512036	-0.08462872	0.024116684	0.999933887	-0.128061392	-0.069205089	-0.058856303	0.662529401
ENSG00000120334	*CENPL*	-0.089434479	-0.072491441	0.098566901	-0.171058342	0.999933887	-0.014391857	0.16985118	-0.184243036	0.662529401
ENSG00000119689	*DLST*	5.544167659	-0.005773855	0.006179211	-0.011953066	0.999933887	-0.070171143	-0.113679405	0.043508262	0.662529401
ENSG00000123737	*EXOSC9*	3.00534474	-0.047805117	-0.031480294	-0.016324823	0.999933887	0.100024038	0.17573273	-0.075708691	0.662735971
ENSG00000120509	*PDZD11*	0.65273707	-0.055802762	0.024656399	-0.08045916	0.999933887	0.194897304	0.022821851	0.172075453	0.662894619
ENSG00000087258	*GNAO1*	1.363559294	0.072575158	-0.049029423	0.121604581	0.999933887	-0.05479788	-0.172159967	0.117362087	0.66294882
ENSG00000262339	*AC130371.1*	-1.096181115	0.121251427	-0.195167469	0.316418896	0.999933887	-0.244351536	-0.627819666	0.383468131	0.66294882
ENSG00000234585	*CCT6P3*	2.841212977	-0.150730086	-0.184893867	0.034163781	0.999933887	-0.829448239	-0.911726152	0.082277912	0.663113282
ENSG00000141293	*SKAP1*	3.350004817	-0.078450839	0.001992692	-0.080443531	0.999933887	0.064725665	0.143495715	-0.07877005	0.663113282
ENSG00000050748	*MAPK9*	3.470645672	-0.020350164	0.090504108	-0.110854271	0.999933887	0.083923519	0.018382934	0.065540586	0.663113282
ENSG00000017483	*SLC38A5*	1.459375771	-0.027796234	0.050832874	-0.078629108	0.999933887	0.347284897	0.231623161	0.115661736	0.663113282
ENSG00000162004	*CCDC78*	0.341257453	-0.039974905	0.171723724	-0.211698629	0.999933887	0.035999384	0.207733522	-0.171734138	0.663113282
ENSG00000008283	*CYB561*	3.548782082	0.001405515	0.003589204	-0.002183689	0.999933887	0.072711502	-0.012147994	0.084859496	0.663113282
ENSG00000173757	*STAT5B*	9.079457406	-0.002366705	0.006140023	-0.008506729	0.999933887	0.174247084	0.22272178	-0.048474696	0.663318747
ENSG00000221946	*FXYD7*	-0.212339296	0.065302213	0.207903907	-0.142601693	0.999933887	0.16057088	-0.032944939	0.193515819	0.663329206
ENSG00000248213	*CICP16*	-0.004835715	-0.026012044	0.03830719	-0.064319235	0.999933887	0.072393152	0.311204184	-0.238811032	0.663597828
ENSG00000253439	*CDC42P5*	0.974916931	0.082749642	0.238918512	-0.156168871	0.999933887	0.519078854	0.692048641	-0.172969787	0.663642706
ENSG00000187187	*ZNF546*	-0.502020686	-0.295658311	-0.11928006	-0.176378251	0.999933887	-0.022137712	0.198321192	-0.220458904	0.664882905
ENSG00000177707	*NECTIN3*	-0.269201337	-0.195407173	-0.383982083	0.18857491	0.999933887	-0.026764504	0.195459702	-0.222224206	0.664882905
ENSG00000149474	*KAT14*	0.682046784	-0.140517411	0.056614877	-0.197132289	0.999933887	0.062173489	-0.08454393	0.146717418	0.664882905
ENSG00000173950	*XXYLT1*	0.425519564	0.012499611	-0.102329265	0.114828876	0.999933887	-0.017486289	0.193793664	-0.211279954	0.664882905
ENSG00000107317	*PTGDS*	0.107528242	0.252494503	0.187283634	0.065210869	0.999933887	0.07091658	-0.097196192	0.168112772	0.66497446
ENSG00000143514	*TP53BP2*	5.964912739	0.010121422	-0.008548026	0.018669448	0.999933887	0.45844276	0.511248677	-0.052805918	0.66497446
ENSG00000112200	*ZNF451*	3.761212934	-0.001618385	0.023487977	-0.025106362	0.999933887	-0.199434606	-0.073552951	-0.125881655	0.66497446
ENSG00000128268	*MGAT3*	2.119261636	-0.052050521	-0.001490971	-0.050559549	0.999933887	-0.266898171	-0.381216304	0.114318133	0.665283328
ENSG00000268903	*AL627309.6*	5.211222976	0.080645481	-0.010261772	0.090907252	0.999933887	0.279485592	0.190930076	0.088555516	0.665321775
ENSG00000140830	*TXNL4B*	2.852751834	-0.061275247	-0.058296126	-0.00297912	0.999933887	0.039629718	-0.039797978	0.079427696	0.665321775
ENSG00000185829	*ARL17A*	-0.481672676	0.112197381	-0.204623289	0.31682067	0.999933887	-0.022258611	0.17271864	-0.194977251	0.665321775
ENSG00000213626	*LBH*	5.030197523	-0.02738022	-0.010809745	-0.016570475	0.999933887	0.117781894	0.053988825	0.06379307	0.665321775
ENSG00000254019	*AC011676.2*	2.128165168	0.054471975	0.137156382	-0.082684408	0.999933887	0.05023683	0.22977925	-0.17954242	0.665321775
ENSG00000236056	*GAPDHP14*	-0.700018413	-0.04598248	0.141358577	-0.187341057	0.999933887	0.126507106	-0.117644973	0.244152078	0.665321775
ENSG00000057252	*SOAT1*	4.130586994	0.012542197	-0.005017661	0.017559858	0.999933887	-0.082550093	-0.011236156	-0.071313938	0.665321775
ENSG00000004766	*VPS50*	1.421030959	-0.01066644	-0.299316667	0.288650227	0.999933887	-0.279616729	-0.134678196	-0.144938533	0.665321775
ENSG00000229622	*MTND5P2*	-0.345269604	0.01530131	-0.138734984	0.154036294	0.999933887	0.82094432	1.070969917	-0.250025597	0.665321775
ENSG00000175198	*PCCA*	0.059101798	-0.027876086	-0.293977001	0.266100915	0.999933887	0.071066021	0.275042776	-0.203976755	0.665415477
ENSG00000102900	*NUP93*	3.400152047	0.026599256	0.016314278	0.010284977	0.999933887	0.040887963	0.128812392	-0.087924429	0.665471716
ENSG00000177463	*NR2C2*	6.262344892	-0.06069997	-0.041571167	-0.019128803	0.999933887	-0.260112208	-0.311472076	0.051359868	0.665541467
ENSG00000197016	*ZNF470*	-0.83633238	-0.033844961	-0.296258857	0.262413896	0.999933887	-0.056096214	0.18185307	-0.237949285	0.666208268

ENSG00000008128	*CDK11A*	2.723676315	0.070937622	0.032939777	0.037997844	0.999933887	-0.001767759	-0.096185701	0.094417942	0.666721038
ENSG00000132170	*PPARG*	0.309527971	-0.137569152	-0.040550257	-0.097018895	0.999933887	-1.280823673	-1.426539076	0.145715403	0.666721038
ENSG00000248508	*SRP14-AS1*	-0.632128978	0.095630392	-0.357277654	0.452908046	0.999933887	0.092286605	0.317603161	-0.225316557	0.666721038
ENSG00000123144	*TRIR*	6.754359055	0.077593608	0.105067614	-0.027474006	0.999933887	0.135590303	0.193379916	-0.057789613	0.666832243
ENSG00000100385	*IL2RB*	5.00901077	0.035652124	-0.097232744	0.132884868	0.999933887	0.098499216	0.03136807	0.067131146	0.666832243
ENSG00000166289	*PLEKHF1*	2.446510035	0.055448332	0.137073491	-0.081625159	0.999933887	0.039977801	-0.073924323	0.113902124	0.666832243
ENSG00000235381	*AL596202.1*	-0.436793771	0.047002888	-0.218840683	0.265843571	0.999933887	-0.108736168	-0.363652365	0.254916197	0.666832243
ENSG00000066827	*ZFAT*	3.550221496	0.08315634	0.106196706	-0.023040366	0.999933887	0.021346498	0.094017151	-0.072670653	0.666955471
ENSG00000127884	*ECHS1*	2.098745177	-0.048339029	-0.032306762	-0.016032267	0.999933887	0.101135679	-0.016376669	0.117512348	0.667068916
ENSG00000267491	*AC100788.1*	0.770509457	0.062903548	-0.290621502	0.35352505	0.999933887	-0.682898185	-0.878415358	0.195517173	0.667068916
ENSG00000160284	*SPATC1L*	-0.872689116	-0.015207749	0.129256903	-0.144464652	0.999933887	0.218789513	-0.066346033	0.285135546	0.667068916
ENSG00000089639	*GMIP*	8.700862555	0.012826907	0.042118489	-0.029291581	0.999933887	-0.298322122	-0.351416251	0.053094129	0.6672222
ENSG00000197989	*SNHG12*	2.954079167	-0.053921514	-0.104680291	0.050758778	0.999933887	0.133503706	0.041963746	0.09153996	0.667318469
ENSG00000139168	*ZCRB1*	2.385741818	-0.128863027	-0.022650423	-0.106212605	0.999933887	-0.084612912	0.020360152	-0.104973064	0.667351474
ENSG00000104731	*KLHDC4*	3.803558585	-0.014447706	-0.004221785	-0.010225921	0.999933887	-0.020517255	0.045825953	-0.066343208	0.667351474
ENSG00000266714	*MYO15B*	6.017895373	0.045317398	0.080453267	-0.035135869	0.999933887	0.068576285	0.010194078	0.058382207	0.667479294
ENSG00000083844	*ZNF264*	3.793159393	-0.024692725	0.039841389	-0.064534114	0.999933887	-0.343039721	-0.412272	0.069232279	0.667479294
ENSG00000272529	*AC090948.3*	-1.065903574	0.0497813	0.434365258	-0.384583959	0.999933887	0.00431693	0.279815923	-0.275498993	0.667508814
ENSG00000124251	*TP53TG5*	0.854231171	0.143919963	0.0375304	0.106389564	0.999933887	0.530749727	0.727258145	-0.196508417	0.667605762
ENSG00000197279	*ZNF165*	4.367102363	0.091153946	0.097188769	-0.006034823	0.999933887	-0.204820392	-0.111279399	-0.093540993	0.667605762
ENSG00000100575	*TIMM9*	0.581090072	-0.06554369	0.349890276	-0.415433966	0.999933887	0.111316291	0.257445146	-0.146128855	0.667605762
ENSG00000254126	*CD8B2*	-1.108096457	0.250935936	-0.261455211	0.512391147	0.999933887	-0.072633748	0.171484032	-0.24411778	0.667624066
ENSG00000086102	*NFX1*	5.158373514	-0.055154869	-0.074657349	0.01950248	0.999933887	-0.047983447	0.002116391	-0.050099837	0.667624066
ENSG00000184319	*RPL23AP82*	0.733731687	0.105805151	0.225466234	-0.119661083	0.999933887	-0.278506424	-0.392020481	0.113514057	0.667624066
ENSG00000110013	*SIAE*	-0.375805121	-0.06662092	-0.07058527	0.00396435	0.999933887	0.016868654	-0.191222002	0.208090656	0.667624066
ENSG00000137656	*BUD13*	3.03424419	-0.003183597	0.037263254	-0.040446851	0.999933887	-0.188266646	-0.112855336	-0.075411311	0.667652347
ENSG00000003987	*MTMR7*	0.279713889	-0.011343983	0.039673731	-0.051017713	0.999933887	-0.208354799	-0.437190259	0.228835461	0.667763485
ENSG00000149136	*SSRP1*	3.680539728	-0.056675181	0.041007007	-0.097682188	0.999933887	0.157867278	0.230317988	-0.07245071	0.667919245
ENSG00000107854	*TNKS2*	6.301449213	-0.046970949	-0.026554303	-0.020416646	0.999933887	-0.413983434	-0.330640273	-0.083343161	0.667919245
ENSG00000065427	*KARS1*	4.905300158	-0.018557093	-0.104373743	0.085816651	0.999933887	0.16013797	0.215483978	-0.055346009	0.667919245
ENSG00000128944	*KNSTRN*	-0.292130348	-0.030877154	-0.152250557	0.121373403	0.999933887	0.176981118	0.365593343	-0.188612225	0.667919245
ENSG00000178074	*C2orf69*	2.464672944	0.201353215	0.05296044	0.148392775	0.999933887	-0.421313002	-0.239292261	-0.182020741	0.668047926
ENSG00000130590	*SAMD10*	1.351930715	0.165182531	0.151765064	0.013417467	0.999933887	0.207447152	0.335734201	-0.128287049	0.668047926
ENSG00000287950	*AC009432.2*	-1.078130984	0.130511463	0.011702363	0.118809101	0.999933887	-0.256452432	0.153362637	-0.409815069	0.668047926
ENSG00000279166	*AC009951.4*	4.021460777	0.079116409	0.466236796	-0.387120387	0.999933887	1.380152861	1.581848024	-0.201695162	0.668047926
ENSG00000162517	*PEF1*	4.990244646	0.017204334	-0.058558083	0.075762417	0.999933887	0.2646715	0.325696085	-0.061024585	0.668047926
ENSG00000115541	*HSPE1*	1.641875553	-0.028625927	0.125600561	-0.154226489	0.999933887	0.175995055	0.275233147	-0.099238093	0.668047926
ENSG00000251034	*AC087854.1*	0.179346798	0.01966878	0.011565226	0.008103555	0.999933887	-0.137356696	0.095284645	-0.232641341	0.668047926
ENSG00000163795	*ZNF513*	4.567078514	0.119899588	0.140546964	-0.020647375	0.999933887	-0.152224342	-0.231733349	0.079509006	0.668222915
ENSG00000285938	*AC072022.2*	5.473181781	0.112048769	0.052765127	0.059283641	0.999933887	0.735978503	0.818734256	-0.082755752	0.668222915
ENSG00000198853	*RUSC2*	1.161394868	0.259859158	-0.124244913	0.384104071	0.999933887	-0.236696937	-0.134039357	-0.102657579	0.668222915
ENSG00000011021	*CLCN6*	5.472810351	0.002394211	-0.00726803	0.00966224	0.999933887	-0.22717552	-0.161086274	-0.066089247	0.668222915
ENSG00000164574	*GALNT10*	4.473697826	0.033218154	0.012131893	0.02108626	0.999933887	-0.033094303	-0.090001393	0.05690709	0.668350767
ENSG00000145725	*PPIP5K2*	4.267774493	-0.066625711	-0.116722492	0.050096782	0.999933887	-0.626477788	-0.787663542	0.161185754	0.668350767
ENSG00000183978	*COA3*	0.819993255	0.052827064	-0.153242364	0.206069428	0.999933887	0.022308609	0.181111516	-0.158802907	0.668350767
ENSG00000163946	*TASOR*	4.394960914	-0.016943201	-0.050620289	0.033677088	0.999933887	-0.368543335	-0.260991534	-0.107551802	0.668350767
ENSG00000216937	*CCDC7*	-0.007116963	-0.102266926	-0.467349276	0.36508235	0.999933887	-0.174696399	0.012885294	-0.187581694	0.668440194
ENSG00000162402	*USP24*	5.705129532	0.010265758	-0.08583393	0.096099688	0.999933887	-0.232906532	-0.319579639	0.086673107	0.668638977
ENSG00000273096	*AL021707.8*	1.157328518	0.144557677	0.013120624	0.131437053	0.999933887	-0.053942751	0.148748149	-0.2026909	0.668718863
ENSG00000197813	*AC011450.1*	1.336997772	0.208989792	-0.004573333	0.213563124	0.999933887	0.334133692	0.227186647	0.106947045	0.668718863
ENSG00000168228	*ZCCHC4*	0.712724728	0.050823702	0.29015374	-0.239330038	0.999933887	0.218281519	0.06325073	0.155030789	0.668718863
ENSG00000127540	*UQCR11*	2.506637846	0.027116866	0.026922093	0.000194773	0.999933887	-0.294415186	-0.397932496	0.10351731	0.668718863
ENSG00000279457	*WASH9P*	1.177870967	-0.030497792	0.000311466	-0.030809258	0.999933887	-0.118066582	-0.258347554	0.140280972	0.668718863
ENSG00000243811	*APOBEC3D*	-0.779059498	0.038364084	-0.06527614	0.103640224	0.999933887	-0.117973267	-0.402986221	0.285012955	0.668718863
ENSG00000120942	*UBIAD1*	2.334753781	0.043878369	-0.163938369	0.207816739	0.999933887	0.096129721	-0.001351475	0.097481196	0.668739999
ENSG00000103356	*EARS2*	0.826626618	0.001925292	-0.037485687	0.039410979	0.999933887	0.128068776	0.001988384	0.126080392	0.668739999
ENSG00000227827	*PKD1P2*	-1.696455889	-0.005165136	0.213022925	-0.218188061	0.999933887	-0.097740429	0.122618652	-0.220359081	0.668781179
ENSG00000204044	*SLC12A5-AS1*	0.609878303	0.136190898	0.118173253	0.018017646	0.999933887	0.369161305	0.567528089	-0.198366784	0.668865529
ENSG00000172967	*XKR3*	-0.158680643	0.042310533	0.09851103	-0.056200497	0.999933887	-0.888847431	-1.036013417	0.147165986	0.668891171
ENSG00000185127	*C6orf120*	3.473330988	-0.067797828	-0.121041162	0.053243334	0.999933887	-0.270688241	-0.192521339	-0.078166902	0.66907171
ENSG00000204136	*GGTA1*	-0.25216484	-0.192668166	-0.179205803	-0.013462363	0.999933887	0.602996448	0.756615442	-0.153618994	0.66907171
ENSG00000185627	*PSMD13*	5.86112101	-0.026582205	-0.027984274	0.001402069	0.999933887	-0.204598043	-0.255814884	0.05121684	0.66907171
ENSG00000132478	*UNK*	4.489421983	-0.009412025	0.112887103	-0.122299127	0.999933887	0.065101909	0.01222403	0.052877879	0.66907171
ENSG00000197747	*S100A10*	5.059192863	0.004143222	-0.156700257	0.160843479	0.999933887	-0.046845034	-0.111783157	0.064938123	0.66907171
ENSG00000185522	*LMNTD2*	0.331181916	0.261707221	0.345824369	-0.084117148	0.999933887	0.157252093	0.302640191	-0.145388097	0.669112222
ENSG00000273373	*AL355488.1*	-0.380893617	0.162025713	0.080540307	0.081485406	0.999933887	-0.192954672	-0.43931069	0.246356018	0.669112222
ENSG00000248503	*AL356235.1*	1.092221385	-0.038326654	0.072419036	-0.110745689	0.999933887	-0.290608355	-0.424825849	0.134217495	0.669112222
ENSG00000235978	*AC018816.1*	-0.158757394	-0.234403826	0.22104786	-0.455415686	0.999933887	1.025736782	1.218271114	-0.192534331	0.669318589
ENSG00000211698	*TRGV4*	-0.142102223	0.118121091	-0.210235259	0.32835635	0.999933887	-0.119071971	0.03056843	-0.149640401	0.669318589
ENSG00000204370	*SDHD*	3.757897545	-0.079736241	-0.031203945	-0.048532296	0.999933887	-0.151335281	-0.083480734	-0.067854547	0.669487821
ENSG00000169696	*ASPSCR1*	3.31784797	0.067288955	0.074268524	-0.00697957	0.999933887	0.012766723	0.08243366	-0.069666937	0.669786652
ENSG00000129317	*PUS7L*	2.032503299	-0.108484649	-0.133820067	0.025335417	0.999933887	-0.295886892	-0.098953437	-0.196933455	0.669981909
ENSG00000138346	*DNA2*	0.95081428	-0.15274167	-0.261876506	0.109134836	0.999933887	-0.352930902	-0.199116987	-0.153813914	0.670097666
ENSG00000154174	*TOMM70*	2.887510794	-0.143955645	-0.022742499	-0.121213146	0.999933887	0.071379301	0.145110536	-0.073731236	0.670108316
ENSG00000275432	*AC022149.1*	0.19049706	-0.228589933	0.062403672	-0.290993606	0.999933887	-0.962021672	-0.758751471	-0.203270201	0.670108316
ENSG00000162413	*KLHL21*	9.434332019	-8.1347e-05	-0.000219811	0.000138465	0.999933887	0.459158834	0.396038748	0.063120087	0.670108316
ENSG00000230107	*AL022316.1*	0.841318426	-0.124105977	-0.507023812	0.382917835	0.999933887	-0.056778567	-0.179821403	0.123042835	0.67014134
ENSG00000107443	*CCNJ*	1.386456535	-0.077781464	-0.178800187	0.101018723	0.999933887	-0.096642368	-0.23767922	0.141036852	0.67014134
ENSG00000085721	*RRN3*	3.266365293	0.001694534	-0.055310412	0.057004946	0.999933887	0.179299687	0.258707289	-0.079407602	0.67014134
ENSG00000255031	*AP002807.1*	3.473636897	0.181396025	0.235759919	-0.054363894	0.999933887	0.035152577	-0.083901889	0.119054466	0.670226823
ENSG00000232022	*FAAHP1*	-0.053984514	-0.037950038	0.119484352	-0.15743439	0.999933887	-0.107405328	0.089218208	-0.196623537	0.670273791
ENSG00000108821	*COL1A1*	0.010950407	0.037403044	-0.211637379	0.249040423	0.999933887	0.354282515	0.619194513	-0.264911999	0.670393263
ENSG00000188277	*C15orf62*	0.86197269	-0.010593208	0.063201557	-0.073794764	0.999933887	-0.930698318	-1.120981101	0.190282782	0.670393263
ENSG00000198556	*ZNF789*	1.874661677	-0.031559446	0.139076433	-0.170635879	0.999933887	0.069934674	-0.061237246	0.13117192	0.670509697
ENSG00000247271	*ZBED5-AS1*	0.325264198	-0.004688212	0.039433239	-0.044121452	0.999933887	-0.016386372	0.143983995	-0.160370367	0.670509697
ENSG00000198825	*INPP5F*	1.894200082	-0.025678448	0.009285757	-0.034964205	0.999933887	-0.0226416	0.081468803	-0.104110403	0.670524981
ENSG00000126062	*TMEM115*	4.240356562	-0.099353188	0.011264954	-0.110618143	0.999933887	-0.20128386	-0.130478731	-0.070805129	0.670528597
ENSG00000196422	*PPP1R26*	-0.046200794	-0.108660327	0.201753316	-0.310413643	0.999933887	-0.270680122	-0.089526428	-0.181153693	0.670528597
ENSG00000182796	*TMEM198B*	1.404006961	-0.051625827	0.279897317	-0.331523144	0.944094537	-0.109905279	-0.001521744	-0.108383536	0.670528597
ENSG00000266598	*AC037487.2*	-0.115837122	0.056456688	0.194159129	-0.137702441	0.999933887	0.313777879	0.557688868	-0.243910989	0.670528597
ENSG00000133460	*SLC2A11*	0.274530363	-0.005008499	0.174846296	-0.179854795	0.999933887	0.058642381	0.234852887	-0.176210506	0.670528597
ENSG00000187522	*HSPA14*	1.953243899	-0.060727796	-0.124091725	0.063363929	0.999933887	0.041977624	-0.072261089	0.114238713	0.670797675
ENSG00000116903	*EXOC8*	5.236791014	-0.083397333	-0.072612041	-0.010785292	0.999933887	-0.168635703	-0.104450152	-0.064185551	0.670844631
ENSG00000149600	*COMMD7*	1.67877977	-0.237320804	0.023722318	-0.261043122	0.999933887	0.087716396	-0.024104941	0.111821338	0.670865676
ENSG00000258944	*AC004846.2*	-0.052512258	-0.275125849	0.006005652	-0.281131501	0.999933887	0.64078632	0.905301228	-0.264514908	0.670865676
ENSG00000131969	*ABHD12B*	-0.012768413	0.089309932	0.146523667	-0.057213735	0.999933887	-0.448235662	-0.19562166	-0.252614002	0.670865676
ENSG00000172943	*PHF8*	5.457816079	-0.01214032	-0.040087903	0.027947583	0.999933887	-0.08385832	-0.129782172	0.045923851	0.670865676
ENSG00000160294	*MCM3AP*	4.870348877	-0.012394884	0.003839773	-0.016234657	0.999933887	0.047725684	-0.009954242	0.057679926	0.670865676
ENSG00000251364	*AC107884.1*	-0.180277581	-0.351665466	-0.051551099	-0.300114367	0.999933887	-0.005495096	0.190099994	-0.19559509	0.671068626
ENSG00000166848	*TERF2IP*	6.487792592	0.008467743	0.040959878	-0.032492135	0.999933887	0.649016651	0.716481539	-0.067464887	0.671108401
ENSG00000004939	*SLC4A1*	-1.9555603 48	7.6348e-05	-0.312159781	0.312236129	0.999933887	-0.302173584	0.112726151	-0.414899735	0.671108401
ENSG00000134369	*NAV1*	1.426042803	-0.144786518	-0.268461356	0.123674838	0.999933887	-0.386601773	-0.494162271	0.107560498	0.671190602
ENSG00000132749	*TESMIN*	0.554058564	-0.062972623	-0.028106584	-0.034866039	0.999933887	-0.500377893	-0.648937885	0.148559993	0.671236011
ENSG00000062598	*ELMO2*	4.829217399	-0.030070482	0.00019851	-0.030268992	0.999933887	-0.052438299	0.008485445	-0.060923744	0.671247242
ENSG00000204338	*CYP21A1P*	-0.81804969	-0.093292696	-0.121347019	0.028054323	0.999933887	0.532170591	0.793407806	-0.261237215	0.671247242

ENSG00000117151	*CTBS*	4.364131344	-0.020848729	-0.069193069	0.04834434	0.999933887	-0.199197046	-0.124077801	-0.075119245	0.671247242
ENSG00000145491	*ROPN1L*	2.647269482	0.014516488	0.025351429	-0.01083494	0.999933887	0.062307898	-0.073020089	0.135327987	0.671247242
ENSG00000103160	*HSDL1*	4.362702616	-0.01302621	-0.041463948	0.028437738	0.999933887	-0.064206054	-0.137235462	0.073029408	0.671736188
ENSG00000104231	*ZFAND1*	1.62475608	0.018344831	-0.051274503	0.069619334	0.999933887	0.131287526	0.270033203	-0.138745676	0.671736188
ENSG00000079134	*THOC1*	2.939327173	-0.118546201	-0.104077191	-0.01446901	0.999933887	-0.123690582	0.008444399	-0.132134981	0.671985995
ENSG00000144559	*TAMM41*	0.652153171	-0.13392147	0.024407405	-0.158328876	0.999933887	0.126662994	0.260429576	-0.133766582	0.671985995
ENSG00000114126	*TFDP2*	3.084549317	0.048087631	0.053883384	-0.005795753	0.999933887	0.016550671	-0.0677332	0.08428387	0.671985995
ENSG00000255717	*SNHG1*	4.328437422	-0.038668275	0.072470243	-0.111138519	0.999933887	0.292782478	0.222717941	0.070064537	0.671985995
ENSG00000198625	*MDM4*	7.489797552	-0.029886018	0.015323035	-0.045209052	0.999933887	-0.027067715	0.031773454	-0.058841169	0.671985995
ENSG00000087086	*FTL*	12.612230121	0.02643692	-0.028418916	0.054855836	0.999933887	0.326550223	0.269611424	0.0569388	0.671985995
ENSG00000189238	*LINC00943*	-0.183107547	-0.07503171	0.06543889	-0.1404706	0.999933887	0.049508081	0.261016451	-0.21150837	0.671985995
ENSG00000140395	*WDR61*	1.772069182	-0.032131913	-0.048553428	0.016421515	0.999933887	0.061142657	-0.05527234	0.116414997	0.671985995
ENSG00000100351	*GRAP2*	3.982261113	-0.014685582	0.055399061	-0.070084644	0.999933887	0.117157006	0.053938376	0.06321863	0.671985995
ENSG00000127586	*CHTF18*	2.841255225	0.093912187	0.06800762	0.025904567	0.999933887	0.144724834	0.043135813	0.10158902	0.672028791
ENSG00000158246	*TENT5B*	-0.891476472	-0.130270838	-0.164831011	0.034560173	0.999933887	-0.278499946	-0.546107938	0.267607992	0.672028791
ENSG00000211789	*TRAV12-2*	0.247815526	0.062055395	-0.070112988	0.132168384	0.999933887	-0.014828548	0.14894585	-0.163774398	0.672028791
ENSG00000085377	*PREP*	2.287368545	0.022046829	0.033080006	-0.011033178	0.999933887	0.039946311	0.139374002	-0.09942769	0.672248319
ENSG00000181381	*DDX60L*	8.08183235	-0.042094159	-0.090490014	0.048395855	0.999933887	0.430052745	0.594778616	-0.164725871	0.672253052
ENSG00000286288	*AL109809.4*	3.914865338	0.023037438	0.050223966	-0.027186528	0.999933887	0.014604235	0.090114503	-0.075510267	0.672397217
ENSG00000027697	*IFNGR1*	9.229828696	-0.080333209	-0.094234997	0.013901788	0.999933887	-0.200137292	-0.135709752	-0.06442754	0.672459188
ENSG00000130595	*TNNT3*	4.449020312	0.070447482	0.059275034	0.011172448	0.999933887	0.285485708	0.218869335	0.066616373	0.672459188
ENSG00000100097	*LGALS1*	4.170834359	0.06317832	0.02176436	0.04141396	0.999933887	0.17459559	0.259725888	-0.085130298	0.672459188
ENSG00000204381	*LAYN*	-2.069562678	0.352316412	-0.453283758	0.80560017	0.999933887	0.250313771	-0.079375105	0.329688876	0.672565626
ENSG00000142089	*IFITM3*	9.021279981	0.057995914	0.000523297	0.057472617	0.999933887	0.037429394	-0.023553208	0.060982601	0.672793712
ENSG00000124596	*OARD1*	2.869455825	-0.047301784	-0.052598786	0.005297001	0.999933887	-0.047653871	0.044606818	-0.092260689	0.673239288
ENSG00000149311	*ATM*	6.234715954	0.003275996	-0.058863147	0.062139143	0.999933887	-0.579600546	-0.428248073	-0.151352473	0.67328765
ENSG00000171596	*NMUR1*	0.613257427	0.043834511	0.196190969	-0.152356458	0.999933887	0.125302067	-0.022206729	0.147508796	0.673698737
ENSG00000061936	*SFSWAP*	5.512350329	-0.03566034	0.046911001	-0.08257134	0.999933887	0.034352846	0.073552648	-0.039199802	0.673706665
ENSG00000163481	*RNF25*	2.684368011	-0.059416673	-0.041406816	-0.018009857	0.999933887	0.134662051	0.218690959	-0.084028908	0.673805553
ENSG00000275801	*AL121985.3*	-1.580440243	0.200530843	1.056747707	-0.856216864	0.999933887	0.45551564	0.645251165	-0.189735525	0.673805553
ENSG00000239377	*ZNF775-AS1*	-1.870139901	0.450426007	-0.758533783	1.20895979	0.999933887	2.327628917	2.081515409	0.246113508	0.67396893
ENSG00000165752	*STK32C*	1.099284096	0.13929517	0.137106501	0.00218867	0.999933887	-0.381073749	-0.529442927	0.148369177	0.67396893
ENSG00000185418	*TARS3*	1.951503267	-0.016061738	0.036455729	-0.052517467	0.999933887	-0.009029732	0.083545117	-0.092574849	0.67396893
ENSG00000251179	*TMEM92-AS1*	1.319058371	-0.067850767	-0.106146523	0.038295757	0.999933887	-0.609351377	-0.773165419	0.163814042	0.674396067
ENSG00000213339	*QTRT1*	2.164680971	0.011827695	-0.021731233	0.033558929	0.999933887	0.165551382	0.255649433	-0.090098051	0.674396067
ENSG00000181218	*H2AW*	0.631917305	0.076875775	0.253849593	-0.176973818	0.999933887	0.092570111	-0.06725346	0.159823571	0.674595269
ENSG00000102145	*GATA1*	1.110895202	0.071846472	0.145463425	-0.073616953	0.999933887	0.108331857	0.000741333	0.107590524	0.674595269
ENSG00000184675	*AMER1*	0.209530803	0.041839609	-0.27062842	0.312468029	0.999933887	0.104480052	-0.062157122	0.166637174	0.674595269
ENSG00000279434	*AL049776.1*	0.711790735	-0.028439586	0.011087494	-0.03952708	0.999933887	0.045610231	0.187577644	-0.141967413	0.674595269
ENSG00000128191	*DGCR8*	3.535189767	0.012637493	0.030281556	-0.017644063	0.999933887	-0.043369298	-0.1215438	0.078174502	0.674595269
ENSG00000255438	*AL354813.1*	0.131830335	-0.026927543	-0.054498088	0.027570545	0.999933887	-1.555222052	-1.865528768	0.310306716	0.674595269
ENSG00000253200	*AC037459.2*	2.294131581	-0.004124434	0.13882374	-0.142948174	0.999933887	0.195757415	0.323245966	-0.127488551	0.674595269
ENSG00000256582	*LINC02390*	-0.302661225	-0.053011969	0.091247164	-0.144259133	0.999933887	-0.043328254	0.184971276	-0.22829953	0.674637544
ENSG00000254419	*AL139349.1*	-0.030543122	0.067655059	-0.067422706	0.135077765	0.999933887	-0.052534234	-0.273318753	0.22078452	0.674646554
ENSG00000263941	*RN7SL32P*	-0.88429156	0.345463027	-0.163183393	0.50864642	0.999933887	0.326148092	-0.052830678	0.378978769	0.674679182
ENSG00000124191	*TOX2*	0.330961426	0.130528574	0.142034218	-0.011505644	0.999933887	-0.058878578	-0.262074955	0.203196377	0.674679182
ENSG00000198182	*ZNF607*	-0.702427141	0.020914774	0.009144414	0.01177036	0.999933887	0.081423396	-0.096864937	0.178288332	0.674679182
ENSG00000244682	*FCGR2C*	4.405372092	0.004441068	-0.186111834	0.190552902	0.999933887	-0.001637278	0.143629209	-0.145266487	0.674679182
ENSG00000119777	*TMEM214*	3.591783632	0.114325625	0.059892	0.054433625	0.999933887	-0.101406769	-0.181391371	0.079984603	0.675385419
ENSG00000125868	*DSTN*	3.533208005	-0.016781804	-0.098221547	0.081439742	0.999933887	-0.045347112	0.035844895	-0.081192007	0.675385419
ENSG00000115514	*TXNDC9*	1.600815348	-0.013785756	0.045790349	-0.059576105	0.999933887	-0.482665585	-0.595389156	0.112723571	0.675385419
ENSG00000182154	*MRPL41*	2.334944301	0.165404034	0.078535151	0.086868883	0.999933887	0.050999341	-0.053615282	0.104614623	0.675414204
ENSG00000139531	*SUOX*	1.40275835	-0.10022091	0.028775044	-0.128995954	0.999933887	-0.056720765	-0.192831386	0.13611062	0.675414204
ENSG00000046647	*GEMIN8*	1.326886994	-0.025210582	0.048488309	-0.073698891	0.999933887	-0.100625971	0.01310393	-0.113729901	0.675414204
ENSG00000235677	*NPM1P26*	1.00179452	0.088609036	0.027517757	0.061091279	0.999933887	0.442881504	0.275980657	0.166900847	0.675624471
ENSG00000264659	*AC064805.2*	-0.038945765	-0.0093429	-0.27999332	0.27065042	0.999933887	-0.403773285	-0.667582262	0.263808977	0.675624471
ENSG00000231826	*LINC01819*	-0.827918471	0.086881039	-0.050489452	0.137370491	0.999933887	-0.49291026	-0.212405892	-0.280504368	0.675816724
ENSG00000211803	*TRAV23DV6*	-0.934357943	-0.158231109	-0.157389822	-0.000841287	0.999933887	-0.173388691	0.09739505	-0.270783741	0.675909668
ENSG00000204428	*LY6G5C*	0.348699347	-0.068808908	0.201081949	-0.269890857	0.999933887	-0.377290479	-0.175208837	-0.202081642	0.675979601
ENSG00000213442	*RPL18AP3*	-0.232185307	0.30215299	-0.253684596	0.555837586	0.999933887	0.2762881	0.051345927	0.224942174	0.676107316
ENSG00000108384	*RAD51C*	0.072629447	-0.116010943	-0.098848096	-0.017162847	0.999933887	0.124639008	0.297488329	-0.172849321	0.676107316
ENSG00000099992	*TBC1D10A*	3.271980695	0.15922126	0.029051889	0.130169371	0.999933887	0.639997096	0.727714241	-0.087717145	0.676130112
ENSG00000101363	*MANBAL*	2.748431924	-0.093377637	-0.07651201	-0.016865627	0.999933887	0.055808336	0.141003471	-0.085195135	0.676130112
ENSG00000106624	*AEBP1*	-0.060203262	0.132549641	0.389616682	-0.257067041	0.999933887	0.166328647	0.332930017	-0.16660137	0.676130112
ENSG00000108175	*ZMIZ1*	9.328852869	0.030755714	0.072793648	-0.042037935	0.999933887	0.005774131	0.059440905	-0.053666774	0.676130112
ENSG00000178202	*POGLUT3*	0.660937405	0.04126809	-0.124393201	0.16566129	0.999933887	-0.076116083	0.070709723	-0.146825805	0.676130112
ENSG00000180096	*SEPTIN1*	4.316765123	0.014659837	0.043222632	-0.028562795	0.999933887	0.149781985	0.073099169	0.076682816	0.676130112
ENSG00000165178	*NCF1C*	7.182877581	0.010324008	-0.008649264	0.018973273	0.999933887	0.907832334	0.825956524	0.08187581	0.676130112
ENSG00000172409	*CLP1*	3.677788262	-0.164451475	-0.067389039	-0.097062436	0.999933887	-0.436222872	-0.51168554	0.075462669	0.676187625
ENSG00000079332	*SAR1A*	6.362694495	-0.047283448	-0.080488368	0.03320492	0.999933887	0.059362688	0.100430204	-0.041067515	0.676296849
ENSG00000082213	*C5orf22*	1.615461606	-0.204259769	0.205779308	-0.410039077	0.944094537	0.017448788	-0.097618793	0.115067581	0.677133433
ENSG00000176182	*MYPOP*	1.509281139	0.039353149	0.039740647	-0.000387497	0.999933887	0.032361849	0.147656517	-0.115294668	0.677133433
ENSG00000218891	*ZNF579*	0.585907218	-0.019193526	0.262181913	-0.281375439	0.999933887	-0.136286419	-0.310840208	0.174553789	0.677133433
ENSG00000116138	*DNAJC16*	2.122429039	0.002858175	-0.115625798	0.118483973	0.999933887	-0.009604981	0.094975487	-0.104580468	0.677133433
ENSG00000234426	*AL139042.1*	-2.687131414	0.076596148	-0.044172878	0.120769027	0.999933887	0.135495791	-0.083467569	0.21896336	0.677163557
ENSG00000134987	*WDR36*	2.499721811	-0.15026911	-0.327908314	0.177639204	0.999933887	0.050477063	0.169008578	-0.118531515	0.677350633
ENSG00000133706	*LARS1*	3.49606069	-0.098892706	-0.033706441	-0.065186265	0.999933887	0.071355876	0.139958091	-0.068602215	0.677350633
ENSG00000286327	*AL035427.2*	0.361649902	0.349534649	0.101113083	0.248421566	0.999933887	0.05401994	0.206813126	-0.152793186	0.677350633
ENSG00000135905	*DOCK10*	5.114686868	-0.076881466	-0.081065638	0.004184171	0.999933887	-0.035677599	0.070616983	-0.106294582	0.677350633
ENSG00000103121	*CMC2*	1.703650133	-0.0538652	0.083371295	-0.137236495	0.999933887	-0.154619312	-0.033374535	-0.121244777	0.677350633
ENSG00000280734	*LINC01232*	1.464351377	0.055034492	-0.126788777	0.181823269	0.999933887	-0.204652101	-0.074801034	-0.129851068	0.677350633
ENSG00000100304	*TTLL12*	3.457512326	0.007136531	0.023693254	-0.016556723	0.999933887	0.083265355	0.150799488	-0.067534133	0.677350633
ENSG00000160460	*SPTBN4*	0.845025803	0.147074122	0.201282895	-0.054208773	0.999933887	0.084605441	0.202917639	-0.118312198	0.677546374
ENSG00000131470	*PSMC3IP*	1.105875667	-0.254576374	-0.13374942	-0.120826954	0.999933887	-0.630102023	-0.799270515	0.169168492	0.677630807
ENSG00000197714	*ZNF460*	2.400783937	0.168209766	0.050548827	0.117660938	0.999933887	-0.120004446	0.01984138	-0.139845827	0.677727287
ENSG00000230795	*HLA-K*	0.302222598	0.069115687	-0.412994099	0.482109787	0.999933887	0.674309343	0.482972059	0.191337284	0.677727287
ENSG00000140750	*ARHGAP17*	4.15064343	-0.003769354	-0.005359572	0.001590217	0.999933887	-0.05492244	-0.002642981	-0.052279458	0.677727287
ENSG00000116918	*TSNAX*	2.975927851	-0.05706163	0.01595252	-0.07301415	0.999933887	-0.377412563	-0.498280576	0.120868013	0.677812555
ENSG00000105135	*ILVBL*	2.155760934	0.034412541	0.089497712	-0.055085171	0.999933887	0.139026168	0.248904491	-0.109878322	0.677812555
ENSG00000162946	*DISC1*	2.548202411	-0.014854091	0.00184674	-0.016700831	0.999933887	0.191354565	0.312864897	-0.121510332	0.677812555
ENSG00000006453	*BAIAP2L1*	3.527818649	0.07320749	0.176661731	-0.103454241	0.999933887	0.206993595	0.297286611	-0.090293015	0.678371616
ENSG00000170037	*CNTROB*	3.528089369	0.026829795	-0.008455546	0.035285341	0.999933887	0.234780036	0.163268933	0.071511103	0.678371616
ENSG00000232533	*AC093673.1*	3.575811979	0.033451925	0.063492177	-0.030040252	0.999933887	-0.331733138	-0.432013049	0.100279911	0.678371616
ENSG00000136699	*SMPD4*	4.058514846	0.001410795	0.028997183	-0.027586388	0.999933887	0.135676755	0.071029947	0.064646808	0.678486244
ENSG00000118007	*STAG1*	4.374043054	0.027017093	-0.102464803	0.129481896	0.999933887	-0.310305075	-0.405718025	0.09541295	0.678646799
ENSG00000150967	*ABCB9*	0.364062672	0.056894143	0.062115397	-0.005221254	0.999933887	-0.373420142	-0.513303254	0.139883112	0.678646799
ENSG00000175348	*TMEM9B*	4.438839497	0.00750074	0.02662199	-0.01912125	0.999933887	-0.117624447	-0.057110168	-0.060514279	0.678646799
ENSG00000164815	*ORC5*	0.526841096	-0.002860885	-0.062512012	0.059651127	0.999933887	0.040738302	0.186840132	-0.14610183	0.678686162
ENSG00000164241	*C5orf63*	-0.706615272	0.057370294	-0.133590699	0.190960993	0.999933887	-0.133031227	0.077824848	-0.210856076	0.678748637
ENSG00000278607	*AC015819.2*	4.645783137	0.315585135	0.37622564	-0.060640504	0.999933887	0.19606878	0.294152947	-0.098084167	0.679058539
ENSG00000177565	*TBL1XR1*	6.119895621	-0.068902945	-0.032483299	-0.036419646	0.999933887	-0.451426624	-0.526629076	0.075202452	0.679058539
ENSG00000272447	*AL135925.1*	0.549237917	0.135070634	-0.05557244	0.190643074	0.999933887	-0.08485285	-0.260245938	0.175393088	0.67908631
ENSG00000273017	*AP000240.1*	0.897040017	0.058450418	-0.00360835	0.062058768	0.999933887	-0.159239624	-0.025427519	-0.133812105	0.67934924
ENSG00000139438	*FAM222A*	0.02103104	0.083090782	0.034526045	0.048564737	0.999933887	0.065236552	-0.176497531	0.241734082	0.679352723
ENSG00000254288	*AC087672.2*	-0.46712427	0.082711732	-0.282391795	0.365103527	0.999933887	-0.253309582	-0.549663502	0.29635392	0.679352723

ENSG00000203721	*LINC00862*	1.421394173	-0.053712947	-0.189802647	0.1360897	0.999933887	0.165413576	0.282614253	-0.117200677	0.679352723
ENSG00000108187	*PBLD*	3.245646819	0.011510466	-0.119828819	0.131339285	0.999933887	-0.339759354	-0.232261162	-0.107498192	0.679352723
ENSG00000090013	*BLVRB*	3.558244158	0.120988502	0.039632292	0.081356209	0.999933887	-0.243571065	-0.331073109	0.087502044	0.67977545
ENSG00000197782	*ZNF780A*	1.871134375	-0.116484197	0.066003396	-0.182487594	0.999933887	-0.250272448	-0.138046904	-0.112225544	0.67977545
ENSG00000162241	*SLC25A45*	3.73131849	-0.05252297	0.011925903	-0.064448873	0.999933887	-0.074536085	-0.140448663	0.065912578	0.67977545
ENSG00000235314	*LINC00957*	0.283519638	0.123768437	-0.061749185	0.185517622	0.999933887	-0.376048051	-0.571370841	0.195322791	0.67977545
ENSG00000166503	*HDGFL3*	3.467512697	-0.079914223	-0.128912773	0.04899855	0.999933887	-0.611182688	-0.733037098	0.12185441	0.67977545
ENSG00000103260	*METRN*	0.502938002	0.079446313	0.055025361	0.024420952	0.999933887	-0.050047375	0.103893294	-0.153940669	0.67977545
ENSG00000141127	*PRPSAP2*	2.202237872	0.049164927	0.004780248	0.044384679	0.999933887	0.047906033	-0.062392254	0.110298287	0.67977545
ENSG00000126214	*KLC1*	4.958708813	0.017472701	0.051347563	-0.033874862	0.999933887	-0.20164352	-0.252316629	0.050673109	0.67977545
ENSG00000086598	*TMED2*	5.47752161	-0.017564244	-0.032586983	0.015022739	0.999933887	-0.233485807	-0.300159257	0.06667345	0.67977545
ENSG00000269506	*AC110792.2*	-0.045020713	0.05037274	0.11375126	-0.06337852	0.999933887	-0.068804324	0.216250352	-0.285054676	0.67977545
ENSG00000157870	*PRXL2B*	5.215221037	0.030479245	0.010240325	0.02023892	0.999933887	-0.291096281	-0.190931647	-0.100164634	0.67977545
ENSG00000136861	*CDK5RAP2*	5.393006898	-0.006498205	-0.08730993	0.080811725	0.999933887	0.204717933	0.270736123	-0.066018191	0.67977545
ENSG00000102753	*KPNA3*	4.077217279	0.01717037	-0.001087046	0.018257416	0.999933887	-0.460644579	-0.377608259	-0.08303632	0.679916907
ENSG00000140092	*FBLN5*	-0.492996743	0.021642544	0.325828014	-0.30418547	0.999933887	0.078567954	-0.120131031	0.198698985	0.679916907
ENSG00000164253	*WDR41*	2.437395899	-0.038780895	-0.014550267	-0.024230628	0.999933887	-0.182053404	-0.270255276	0.088201872	0.679977744
ENSG00000198851	*CD3E*	6.26700514	-0.019996389	0.001949391	-0.02194578	0.999933887	0.094422276	0.147670862	-0.053248586	0.680027391
ENSG00000247315	*ZCCHC3*	3.722974124	0.104007463	0.01558997	0.088417492	0.999933887	-0.3034831	-0.376919237	0.073436137	0.680150505
ENSG00000065060	*UHRF1BP1*	3.062824012	-0.0364672	-0.031546845	-0.004920355	0.999933887	-0.030988186	0.05795785	-0.088946036	0.680150505
ENSG00000162650	*ATXN7L2*	2.41946232	0.013901891	0.098676148	-0.084774256	0.999933887	0.063393833	-0.021657136	0.085050968	0.680193581
ENSG00000076321	*KLHL20*	2.074448266	-0.042461683	0.028507352	-0.070969035	0.999933887	-0.059275307	0.032893108	-0.092168416	0.680513084
ENSG00000173275	*ZNF449*	0.691989547	0.000465037	-0.042038719	0.042503756	0.999933887	-0.160920717	-0.041956123	-0.118964594	0.680513084
ENSG00000201988	*Y_RNA*	-0.755292338	0.22233291	0.154860569	0.067472341	0.999933887	1.199084521	1.416498488	-0.217413967	0.680579633
ENSG00000166260	*COX11*	1.841347819	-0.032618869	-0.062400956	0.029782087	0.999933887	-0.034665709	0.071132207	-0.105797916	0.680579633
ENSG00000101104	*PABPC1L*	4.326343934	-0.033999318	0.005575598	-0.039574916	0.999933887	0.027917533	0.082331161	-0.054413628	0.680643223
ENSG00000203485	*INF2*	2.60814538	0.041478698	0.231288755	-0.189810057	0.999933887	0.166777994	0.255984816	-0.089206822	0.680643223
ENSG00000141736	*ERBB2*	0.773274919	0.217877349	0.202322556	0.015554793	0.999933887	0.09601023	0.233477278	-0.137467048	0.680897978
ENSG00000173295	*FAM86B3P*	4.189872013	0.063963934	0.105174808	-0.041210874	0.999933887	-0.09222237	-0.164396104	0.072173734	0.680945984
ENSG00000272533	*SNORA28*	-0.327209902	0.147705714	0.50015056	-0.352444846	0.999933887	0.469885943	0.687126264	-0.217240321	0.680945984
ENSG00000225873	*C3orf86*	2.06118523	0.045029331	0.040195106	0.004834224	0.999933887	0.073346028	-0.109308499	0.182654527	0.680945984
ENSG00000137449	*CPEB2*	5.557477881	0.069363331	0.036893723	0.032469608	0.999933887	-0.750901619	-0.828047987	0.077146368	0.681209137
ENSG00000263272	*AC004148.1*	0.490930795	0.182606919	-0.12225018	0.304857099	0.999933887	0.076855298	-0.074344802	0.1512001	0.681432495
ENSG00000160256	*FAM207A*	2.701863908	0.073061811	0.151025789	-0.077963979	0.999933887	-0.080142404	-0.200149327	0.120006924	0.681499305
ENSG00000260528	*FAM157C*	4.109456675	0.081769902	-0.006129666	0.087899568	0.999933887	0.414350923	0.498404553	-0.08405363	0.681616608
ENSG00000064787	*BCAS1*	0.223549227	-0.044168884	0.143983592	-0.188152476	0.999933887	-0.13962004	0.050519492	-0.190139531	0.681616608
ENSG00000158092	*NCK1*	3.307350122	-0.119307047	-0.101525029	-0.017782017	0.999933887	-0.234210507	-0.311025967	0.07681546	0.681990499
ENSG00000270820	*AC016727.1*	1.055003317	0.024413396	0.084818521	-0.060405125	0.999933887	-0.048570707	0.077942356	-0.126513064	0.681990499
ENSG00000199695	*RNU6-1128P*	0.060031795	0.007432194	0.007874807	-0.000442614	0.999933887	0.74430531	1.036473734	-0.292168424	0.682067745
ENSG00000279430	*AL590560.4*	1.656383699	0.079587429	-0.005124745	0.084712174	0.999933887	0.212555304	0.401776213	-0.189220909	0.682107742
ENSG00000124214	*STAU1*	6.130429679	-0.056312876	-0.034518153	-0.021794723	0.999933887	-0.395520237	-0.448049297	0.05252906	0.682381691
ENSG00000151247	*EIF4E*	3.453802574	0.048773969	0.070607123	-0.021833155	0.999933887	0.063113442	0.145764304	-0.082650863	0.682381691
ENSG00000120725	*SIL1*	1.098790399	-0.123624409	-0.050398995	-0.073225414	0.999933887	0.139177521	-0.035751025	0.174928546	0.682433034
ENSG00000241112	*RPL29P14*	-0.283266934	0.116539217	-0.117883879	0.234423096	0.999933887	0.515702328	0.320927233	0.194775095	0.682511211
ENSG00000171444	*MCC*	1.8220693	-0.050748285	-0.191890358	0.141142073	0.999933887	-0.216486107	-0.103088554	-0.113397553	0.682511211
ENSG00000249662	*LINC02218*	1.171524362	-0.022783434	-0.018794235	-0.003989199	0.999933887	0.095290971	-0.035579397	0.130870368	0.682511211
ENSG00000100207	*TCF20*	6.851922539	-0.039723358	0.069315364	-0.109038722	0.999933887	-0.297946182	-0.35445671	0.056510528	0.682694126
ENSG00000143294	*PRCC*	5.168932857	-0.014010246	-0.041012513	0.027002267	0.999933887	0.119075332	0.167431989	-0.048356656	0.68281505
ENSG00000111605	*CPSF6*	4.635659424	-0.002639449	-0.052551462	0.049912013	0.999933887	-0.26701735	-0.333041554	0.066024205	0.68281505
ENSG00000237840	*FAM21FP*	1.365805944	0.260307294	0.136687133	0.123620161	0.999933887	0.623022684	0.490143985	0.132878699	0.682939866
ENSG00000169814	*BTD*	3.588960103	-0.053334424	-0.034329616	-0.019004808	0.999933887	-0.349512037	-0.2693321	-0.080179937	0.683158785
ENSG00000283849	*AC092053.2*	3.116261645	0.447643884	0.361078458	0.086565426	0.999933887	0.986963432	1.157578576	-0.170615144	0.683214994
ENSG00000227191	*TRGC2*	3.164865977	-0.118707729	-0.114152194	-0.004555535	0.999933887	0.087168493	0.169102144	-0.081933651	0.683214994
ENSG00000287032	*AC018647.3*	-0.370707902	0.151981654	0.179275274	-0.02729362	0.999933887	-0.282256069	0.040734477	-0.322990546	0.683309705
ENSG00000176896	*TCEANC*	1.65323815	-0.054297839	-0.099078916	0.044781076	0.999933887	0.146052106	0.268067491	-0.122015385	0.683309705
ENSG00000118412	*CASP8AP2*	2.321629835	-0.057798552	0.006977934	-0.064776487	0.999933887	-0.515831281	-0.334466576	-0.181364705	0.683309705
ENSG00000172269	*DPAGT1*	1.396958765	0.024446309	-0.361112232	0.385558541	0.944094537	0.054457092	-0.053855037	0.108312129	0.683309705
ENSG00000148057	*IDNK*	1.308758387	-0.009588581	-0.311366203	0.301777623	0.999933887	0.181029665	0.293470065	-0.1124404	0.683309705
ENSG00000178342	*KCNG2*	-0.082447352	0.046300214	0.203683746	-0.157383532	0.999933887	0.099996923	0.384671152	-0.284674229	0.683407282
ENSG00000186352	*ANKRD37*	1.58632922	0.092847263	0.246909871	-0.154062607	0.999933887	0.112240451	0.261184971	-0.14894452	0.683892654
ENSG00000265871	*MIR3174*	-0.367666068	-0.137775615	0.139688821	-0.277464436	0.999933887	0.139433977	0.312122786	-0.172688809	0.683892654
ENSG00000175274	*TP53I11*	6.379124849	-0.022447415	0.034624006	-0.057071421	0.999933887	-0.712776163	-0.793735759	0.080959595	0.683952946
ENSG00000189134	*NKAPL*	-0.033017174	0.147147129	-0.14278394	0.289931069	0.999933887	-0.367447833	-0.207503487	-0.159944346	0.684943293
ENSG00000161642	*ZNF385A*	4.46128849	-0.017275016	-0.027314794	0.010039778	0.999933887	-1.178904049	-1.271088303	0.092184254	0.684943293
ENSG00000131069	*ACSS2*	3.831384366	0.011333483	-0.00058633	0.011919813	0.999933887	-0.562045356	-0.65458726	0.092541904	0.685227502
ENSG00000264548	*AC132872.2*	0.936793032	0.209250381	0.040207592	0.169042789	0.999933887	1.124506358	1.336603754	-0.212097395	0.68528968
ENSG00000186532	*SMYD4*	1.626845094	-0.059198514	0.101671965	-0.160870479	0.999933887	0.080756614	-0.019995668	0.100752281	0.68528968
ENSG00000089063	*TMEM230*	4.614088016	0.019167178	-0.031394006	0.050561184	0.999933887	-0.275400884	-0.332280381	0.056879497	0.685592485
ENSG00000184922	*FMNL1*	9.529647839	0.006149193	0.031226493	-0.0250773	0.999933887	-0.277023902	-0.323712624	0.046688722	0.685679556
ENSG00000119509	*INVS*	1.899375898	0.048399005	-0.079737657	0.128136663	0.999933887	-0.123196375	-0.021330106	-0.101866269	0.685817175
ENSG00000178966	*RMI1*	1.155775244	0.057012769	-0.045631507	0.102644277	0.999933887	-0.819488148	-0.953240533	0.133752384	0.685862631
ENSG00000104219	*ZDHHC2*	3.594458821	0.090575335	-0.164717206	0.255292541	0.594252256	-0.321076888	-0.395051399	0.07397451	0.686009881
ENSG00000138190	*EXOC6*	2.962799698	-0.080909804	-0.043217812	-0.037691992	0.999933887	-0.470970927	-0.373907166	-0.097063761	0.686009881
ENSG00000233954	*UQCRHL*	0.192418932	0.069368333	-0.063829917	0.133198249	0.999933887	-0.049043633	-0.220118725	0.171075092	0.686150482
ENSG00000278053	*DDX52*	2.587926835	-0.067341526	-0.065039215	-0.002302311	0.999933887	0.060359123	-0.023812989	0.084172112	0.686178796
ENSG00000117410	*ATP6V0B*	9.212110946	0.018720565	-0.008419018	0.027139583	0.999933887	-0.073655273	-0.122870626	0.049215353	0.686183138
ENSG00000188243	*COMMD6*	3.927147935	-0.013295741	-0.005110651	-0.00818509	0.999933887	0.030310113	-0.037903626	0.068213739	0.686505991
ENSG00000225972	*MTND1P23*	4.243129426	0.080581002	0.212749145	-0.132168143	0.999933887	0.016133897	0.162951126	-0.146817228	0.686802703
ENSG00000092847	*AGO1*	5.925348281	-0.043921875	0.014435327	-0.058357203	0.999933887	-0.239745927	-0.319547487	0.07980156	0.686802703
ENSG00000270127	*AC027020.2*	-0.595531985	0.043010105	0.003244476	0.039765629	0.999933887	-0.101294891	0.121878166	-0.223173058	0.686802703
ENSG00000083544	*TDRD3*	1.255452484	0.016863506	-0.043343869	0.060207375	0.999933887	-0.010854244	0.12932322	-0.140177464	0.686802703
ENSG00000135363	*LMO2*	6.563250644	-0.093576186	-0.14508738	0.051511194	0.999933887	-0.468298086	-0.395643752	-0.072654334	0.686819895
ENSG00000164916	*FOXK1*	4.630952914	0.004022826	0.082513327	-0.078490501	0.999933887	0.177035507	0.238940388	-0.061904881	0.687016877
ENSG00000129521	*EGLN3*	-0.729742969	0.007701096	0.285572698	-0.277871601	0.999933887	0.100936958	-0.113668373	0.214605331	0.68708419
ENSG00000146247	*PHIP*	6.153176602	-0.094003999	-0.198436577	0.104432578	0.999933887	-0.746447888	-0.54518566	-0.201262228	0.687193209
ENSG00000170315	*UBB*	6.358999558	-0.031496784	-0.082078744	0.05058196	0.999933887	0.348859083	0.408939937	-0.060080854	0.687339205
ENSG00000185158	*LRRC37B*	4.246636988	-0.024709308	0.056894702	-0.081604011	0.999933887	-0.377222306	-0.42209593	0.044873624	0.687389849
ENSG00000100744	*GSKIP*	2.994731192	-0.032345646	-0.122759308	0.090413662	0.999933887	-0.490150641	-0.600157996	0.110007355	0.687389849
ENSG00000186834	*HEXIM1*	5.679226905	0.088779113	0.104378815	-0.015599702	0.999933887	-0.123963172	-0.187896516	0.063933343	0.687513419
ENSG00000184216	*IRAK1*	8.321991587	0.079326632	0.103376648	-0.024050016	0.999933887	0.456252229	0.527063215	-0.070810985	0.687513419
ENSG00000221968	*FADS3*	2.324732462	0.054725348	0.081159073	-0.026433726	0.999933887	0.032064221	-0.040887229	0.07295145	0.687513419
ENSG00000183569	*SERHL2*	0.06620416	0.107389377	0.103315168	0.004074208	0.999933887	0.209542619	0.026092934	0.183449685	0.687513419
ENSG00000173559	*NABP1*	9.91505249	0.050426046	0.052298204	-0.001872158	0.999933887	0.034416852	0.119919265	-0.085502414	0.687513419
ENSG00000089692	*LAG3*	2.648865734	0.032611805	0.107168218	-0.074556413	0.999933887	0.040705505	-0.042869805	0.08357531	0.687513419
ENSG00000259096	*SHLD2P2*	-0.472845666	0.00742423	0.232256446	-0.224832216	0.999933887	1.43613375	1.791252716	-0.355118966	0.687513419
ENSG00000269925	*Z98884.2*	-0.493661461	0.241024336	0.19478978	0.046234556	0.999933887	-0.392249163	-0.635913722	0.243664559	0.687553138
ENSG00000189190	*ZNF600*	1.779508754	-0.092599571	-0.145740401	0.053140829	0.999933887	-0.159126853	-0.043529053	-0.1155978	0.687705809
ENSG00000277734	*TRAC*	6.169816769	0.011058907	-0.035126703	0.04618561	0.999933887	0.139920682	0.195825709	-0.055905027	0.687777738
ENSG00000185591	*SP1*	7.3166999	-0.068361343	-0.004767118	-0.063594225	0.999933887	-0.763733818	-0.830812356	0.067078538	0.687939058
ENSG00000021300	*PLEKHB1*	1.402412897	0.047933438	-0.121990031	0.169923469	0.999933887	0.028593314	0.169616754	-0.14102344	0.688381207
ENSG00000173482	*PTPRM*	1.284370685	-0.040307915	-0.276008135	0.23570022	0.999933887	-0.063522279	0.057719565	-0.121241844	0.688536872
ENSG00000181315	*ZNF322*	1.032088199	-0.038235011	-0.051259576	0.013024565	0.999933887	-0.41250084	-0.517783008	0.105282168	0.688536872
ENSG00000185000	*DGAT1*	5.896170672	0.059741257	0.052978779	0.006762478	0.999933887	0.220018961	0.147898339	0.072120621	0.688607397
ENSG00000119688	*ABCD4*	3.418085916	-0.04470968	0.001325744	-0.046035424	0.999933887	0.084501895	0.003609158	0.080892737	0.688607397
ENSG00000182487	*NCF1B*	6.149492079	0.034607466	-0.011461792	0.046069258	0.999933887	0.990501522	0.908685955	0.081815567	0.688607397

ENSG00000142632	*ARHGEF19*	0.485828419	0.059233715	-0.113062813	0.172296528	0.999933887	0.079467965	-0.092440386	0.171908351	0.688607397
ENSG00000007237	*GAS7*	7.674061614	-0.048149135	-0.003035812	-0.045113324	0.999933887	-0.447335189	-0.390108742	-0.057226447	0.688749815
ENSG00000260278	*AC098818.2*	0.697855032	0.365184884	0.214535661	0.150649224	0.999933887	0.721292312	0.488800393	0.232491919	0.688824508
ENSG00000206172	*HBA1*	3.88306826	-0.102045946	-0.116342481	0.014296535	0.999933887	-0.038877463	0.064346333	-0.103223796	0.688824508
ENSG00000237711	*MTCO3P11*	-1.515198471	-0.167256152	-0.197280002	0.03002385	0.999933887	0.419031658	0.908950733	-0.489919075	0.688824508
ENSG00000233527	*ZNF529-AS1*	0.093375148	0.068833924	-0.016262009	0.085095933	0.999933887	-0.265168664	-0.114147191	-0.151021472	0.688824508
ENSG00000099203	*TMED1*	1.442499162	0.053502897	0.151208609	-0.097705712	0.999933887	-0.564438656	-0.706136823	0.141698168	0.688824508
ENSG00000104979	*C19orf53*	4.548506111	0.02054726	0.052570637	-0.032023376	0.999933887	0.043152434	-0.011314994	0.054467429	0.688824508
ENSG00000287356	*AL590822.3*	-1.052489368	0.126166639	0.221099738	-0.094933099	0.999933887	0.203286039	-0.113227898	0.316513936	0.689308684
ENSG00000108797	*CNTNAP1*	1.403015712	-0.021965881	0.124274288	-0.146240168	0.999933887	-0.131093877	-0.253366578	0.122272701	0.689308684
ENSG00000080298	*RFX3*	3.279087945	-0.052035772	-0.093083916	0.041048145	0.999933887	-0.373670741	-0.28077698	-0.092893762	0.689630372
ENSG00000196378	*ZNF34*	0.219039869	-0.186848401	0.20332765	-0.390176051	0.999933887	0.175710816	0.000306947	0.175403869	0.689764298
ENSG00000163235	*TGFA*	2.728045295	0.115604817	0.10859878	0.007006037	0.999933887	-0.071052255	0.040923853	-0.111976109	0.689847258
ENSG00000264769	*AC145207.8*	2.550084281	0.234231447	0.227157549	0.007073898	0.999933887	0.737680267	0.884344322	-0.146664054	0.689847258
ENSG00000178896	*EXOSC4*	1.36107855	0.104804488	0.090425949	0.014378538	0.999933887	0.462032408	0.304861248	0.15717116	0.689847258
ENSG00000070182	*SPTB*	0.671418838	-0.06155458	-0.186321059	0.12476648	0.999933887	-0.172676999	-0.367126651	0.194449652	0.689847258
ENSG00000114744	*COMMD2*	3.133039717	-0.010648031	-0.048680513	0.038032481	0.999933887	-0.137325759	-0.070762403	-0.066563356	0.689942301
ENSG00000147400	*CETN2*	2.251437601	-0.112846328	-0.18272611	0.069879782	0.999933887	-0.059576597	0.06018878	-0.119765377	0.6899623
ENSG00000040199	*PHLPP2*	1.812151509	-0.182576656	-0.09009325	-0.092483406	0.999933887	0.03900574	0.148700569	-0.109694829	0.690308776
ENSG00000158089	*GALNT14*	2.833156486	0.042547275	-0.021569443	0.064116718	0.999933887	0.910584892	1.001040485	-0.090455593	0.690308776
ENSG00000188263	*IL17REL*	0.374066966	0.179650605	0.049386388	0.130264218	0.999933887	1.475443557	1.259468911	0.215974646	0.690419027
ENSG00000155729	*KCTD18*	2.819897217	-0.002930204	-0.106546224	0.10361602	0.999933887	-0.291862184	-0.374800725	0.082938541	0.690449116
ENSG00000138829	*FBN2*	0.532563793	-0.068543538	-0.278016405	0.209472867	0.999933887	-0.450820091	-0.282337221	-0.168482869	0.690871994
ENSG00000211746	*TRBV19*	0.976306277	-0.150993111	0.178864187	-0.329857298	0.999933887	0.10135733	0.255998177	-0.154640846	0.690964141
ENSG00000088827	*SIGLEC1*	-1.107608039	0.101894114	-0.151137094	0.253031208	0.999933887	-0.136411412	0.221010825	-0.357422237	0.690964141
ENSG00000128000	*ZNF780B*	1.139930928	-0.079384782	-0.118145793	0.038761011	0.999933887	-0.084212628	0.061761507	-0.145974134	0.690964141
ENSG00000137135	*ARHGEF39*	-0.600821537	-0.050268158	-0.232619637	0.18235148	0.999933887	0.243466733	0.446112504	-0.202645771	0.690964141
ENSG00000110888	*CAPRIN2*	2.913396616	-0.010060614	-0.11918233	0.109121717	0.999933887	-0.084554733	-0.002060315	-0.082494418	0.690964141
ENSG00000226752	*CUTALP*	4.772948111	-0.056902202	-0.018834305	-0.038067897	0.999933887	0.06822778	0.116532081	-0.048304301	0.691052878
ENSG00000152766	*ANKRD22*	1.034612892	-0.188422699	0.115106096	-0.303528794	0.944094537	0.302200899	0.53985127	-0.237650371	0.691106759
ENSG00000285728	*AC098484.4*	0.20975356	-0.144048932	0.324982877	-0.46903181	0.999933887	0.019704314	0.195059652	-0.175355339	0.691116569
ENSG00000165802	*NSMF*	3.900523958	0.063348627	0.075730733	-0.012382106	0.999933887	0.213852008	0.288302758	-0.074450751	0.691334481
ENSG00000083290	*ULK2*	1.000950864	0.138421368	0.098048296	0.040373072	0.999933887	0.192939097	0.33497289	-0.142033793	0.691634344
ENSG00000146856	*AGBL3*	0.381277898	-0.171784336	0.113924768	-0.285709104	0.999933887	-0.127533378	-0.275701921	0.148168543	0.691634344
ENSG00000150093	*ITGB1*	5.657278119	-0.016242678	-0.125648044	0.109405365	0.999933887	-0.196705234	-0.139842376	-0.056862858	0.691634344
ENSG00000272512	*AL645608.7*	4.115178893	0.299157204	0.297251952	0.001905252	0.999933887	1.329098203	1.180537756	0.148560447	0.69173477
ENSG00000067182	*TNFRSF1A*	7.617693086	-0.065662451	-0.032078323	-0.033584128	0.999933887	-0.600992147	-0.676166159	0.075174012	0.69173477
ENSG00000124783	*SSR1*	5.351768087	0.032119431	-0.081765707	0.113885138	0.999933887	-0.274754233	-0.33536224	0.060608007	0.69173477
ENSG00000079277	*MKNK1*	6.008281351	0.024050726	-0.014392147	0.038442873	0.999933887	-0.301326056	-0.233834608	-0.067491447	0.691754538
ENSG00000280202	*AC005831.1*	1.110931801	-0.009970834	-0.008007056	-0.001963779	0.999933887	-0.045383445	-0.191463044	0.1460796	0.691791423
ENSG00000023318	*ERP44*	6.0477495	-0.046520134	-0.038699829	-0.007820305	0.999933887	-0.083711749	-0.034342744	-0.049369005	0.692220631
ENSG00000136643	*RPS6KC1*	2.600300625	-0.067896342	-0.095289059	0.027392718	0.999933887	-0.179799666	-0.092897821	-0.086901846	0.692296529
ENSG00000100941	*PNN*	5.614635858	-0.059541867	-0.14816821	0.088626343	0.999933887	-0.269600772	-0.362016207	0.092415435	0.692296529
ENSG00000123095	*BHLHE41*	1.745439197	-0.072276269	-0.280479315	0.208203045	0.999933887	-1.015916704	-0.904818624	-0.11109808	0.692296529
ENSG00000164104	*HMGB2*	6.525645118	-0.017357148	-0.045839958	0.02848281	0.999933887	0.336944065	0.385391617	-0.048447552	0.692296529
ENSG00000012174	*MBTPS2*	-0.112421818	0.028911666	-0.043021193	0.07193286	0.999933887	0.033781868	0.19363081	-0.159848942	0.692296529
ENSG00000245293	*CYP2U1-AS1*	-1.630288447	0.158903544	0.361134271	-0.202230727	0.999933887	0.045282631	0.478579707	-0.433297076	0.692496561
ENSG00000100599	*RIN3*	7.183769543	0.026444778	0.026465709	-2.0931e-05	0.999933887	-0.089281588	-0.15063607	0.061354481	0.692950142
ENSG00000136936	*XPA*	1.674125829	-0.062317586	-0.169463932	0.107146346	0.999933887	0.108636701	0.218747045	-0.110110344	0.692969073
ENSG00000113971	*NPHP3*	1.009911176	-0.189395031	-0.111438746	-0.077956285	0.999933887	-0.277554648	-0.412543516	0.134988868	0.692988431
ENSG00000262655	*SPON1*	0.072206724	0.197833082	0.023617362	0.17421572	0.999933887	0.072177434	0.218215179	-0.146037745	0.693049579
ENSG00000240731	*AL139287.1*	0.324764069	0.131955019	-0.202490197	0.334445217	0.999933887	-0.052814333	-0.208080201	0.155265868	0.69330527
ENSG00000120129	*DUSP1*	10.107318972	0.515455262	0.583749325	-0.068294063	0.999933887	1.122876046	1.247931578	-0.125055532	0.69338341
ENSG00000176834	*VSIG10*	0.788373457	0.113794349	0.143215506	-0.029421157	0.999933887	-0.166620916	-0.374676772	0.208055857	0.69338341
ENSG00000286025	*FAM246C*	-0.637703783	0.155633399	-0.015860483	0.171493882	0.999933887	-0.331906602	-0.015702494	-0.316204108	0.693426661
ENSG00000145244	*CORIN*	-0.763333089	-0.338094019	-0.068477937	-0.269616082	0.999933887	-0.352921702	-0.027922974	-0.324998728	0.693550514
ENSG00000108551	*RASD1*	0.818789007	0.272096365	0.542288694	-0.270192329	0.999933887	1.546605898	1.382466194	0.164139704	0.693550514
ENSG00000175575	*PAAF1*	0.434092168	-0.175209909	-0.105635499	-0.06957441	0.999933887	0.031261961	0.190942399	-0.159680438	0.693550514
ENSG00000067704	*IARS2*	3.100694942	-0.060910866	-0.047274206	-0.013636659	0.999933887	-0.081535404	-0.163369395	0.081833991	0.693550514
ENSG00000196177	*ACADSB*	1.88690936	0.073083947	-0.090672494	0.163756442	0.999933887	-0.152949106	-0.036209643	-0.116739463	0.693550514
ENSG00000239636	*TFAP2E-AS1*	-0.197440042	0.093376848	-0.041380325	0.134757173	0.999933887	-0.417921871	-0.62114103	0.203219159	0.693550514
ENSG00000171763	*SPATA5L1*	3.416456733	0.025820795	0.064116901	-0.038296106	0.999933887	0.122995039	0.044492927	0.078502112	0.693550514
ENSG00000120805	*ARL1*	1.73723532	-0.261995868	-0.069132113	-0.192863755	0.999933887	0.08555521	0.183101324	-0.097546113	0.693620812
ENSG00000228655	*AC079793.1*	2.296485839	0.409278311	0.464236682	-0.054958371	0.999933887	0.124601118	-0.180042686	0.304643804	0.693620812
ENSG00000260229	*PPIAP51*	-0.164452106	-0.207246463	-0.084217304	-0.12302916	0.999933887	0.251777823	0.465643724	-0.213865901	0.693620812
ENSG00000156265	*MAP3K7CL*	0.401514377	-0.093009982	-0.027574327	-0.065435654	0.999933887	-0.588370953	-0.755804888	0.167433935	0.693620812
ENSG00000110442	*COMMD9*	2.914745303	-0.025023523	-0.041250518	0.016226995	0.999933887	-0.298774745	-0.373494305	0.07471956	0.693620812
ENSG00000160908	*ZNF394*	6.866811346	0.010763412	-0.024269336	0.035032748	0.999933887	-0.020783439	0.026260467	-0.047043905	0.693620812
ENSG00000103326	*CAPN15*	4.169027883	0.058523794	0.047440518	0.011083276	0.999933887	0.037107131	-0.032253601	0.069360732	0.693675188
ENSG00000110721	*CHKA*	5.060508166	0.034164665	0.047844897	-0.013680233	0.999933887	-0.533988221	-0.478084572	-0.055903649	0.693675188
ENSG00000100346	*CACNA1I*	2.428003676	-0.028984808	0.168072043	-0.197056851	0.999933887	0.054958046	0.136958912	-0.082000867	0.693675188
ENSG00000038002	*AGA*	1.824503011	0.009322509	-0.000217854	0.009540363	0.999933887	-0.08070471	-0.181779133	0.101074423	0.694314083
ENSG00000118113	*MMP8*	-0.740406166	0.262393683	0.180332664	0.082061019	0.999933887	0.147889326	0.353178437	-0.205289111	0.694448072
ENSG00000170500	*LONRF2*	-1.88379473	-0.106260567	0.484567837	-0.590828403	0.999933887	-0.329039377	-0.147006562	-0.182032815	0.694712812
ENSG00000181817	*LSM10*	5.251867697	0.052947833	-0.045318001	0.098265834	0.999933887	-0.357332904	-0.421521527	0.064188623	0.694738216
ENSG00000226121	*AHCTF1P1*	-0.667051486	0.146553048	0.087707932	0.058845116	0.999933887	-1.145974861	-1.428734095	0.282759234	0.694738216
ENSG00000068885	*IFT80*	0.812960005	-0.126127059	-0.0286889	-0.097438159	0.999933887	-0.046947874	0.161663075	-0.208610949	0.694738216
ENSG00000068400	*GRIPAP1*	6.172494843	0.02976954	0.043902123	-0.014132582	0.999933887	0.131304418	0.181902304	-0.050597886	0.694738216
ENSG00000167850	*CD300C*	1.010009826	0.070440699	-0.016491224	0.086931923	0.999933887	-0.69738125	-0.539987669	-0.157393581	0.694738216
ENSG00000119760	*SUPT7L*	3.915543369	-0.0373747	-0.061241173	0.023866474	0.999933887	0.154702888	0.220625448	-0.065922559	0.694738216
ENSG00000230069	*LRRC37A15P*	-0.278225462	-0.123285081	0.184996938	-0.30828202	0.999933887	0.283185871	0.058484319	0.224701552	0.694738216
ENSG00000273145	*BX537318.1*	0.004871363	-0.136811889	-0.175872212	0.039060323	0.999933887	0.38009095	0.251749009	0.128341941	0.694738216
ENSG00000148688	*RPP30*	1.544646798	0.046003986	0.052610579	-0.006606593	0.999933887	0.025279051	0.125385866	-0.100106814	0.694738216
ENSG00000137764	*MAP2K5*	1.992445706	0.033489872	0.042243553	-0.00875368	0.999933887	-0.048730906	0.056082015	-0.10481292	0.694738216
ENSG00000213071	*LPAL2*	-0.808793969	0.067445625	-0.032977688	0.100423313	0.999933887	-0.395423673	-0.163253442	-0.232170231	0.694738216
ENSG00000141741	*MIEN1*	4.006670755	0.013327523	0.048990271	-0.035662748	0.999933887	0.191238924	0.258755653	-0.067516729	0.694738216
ENSG00000166004	*CEP295*	2.654222308	-0.02720371	-0.094081665	0.066877955	0.999933887	-0.813078514	-0.660596983	-0.152481531	0.694738216
ENSG00000082074	*FYB1*	9.093732544	-0.01589984	-0.10376285	0.08786301	0.999933887	-0.901292122	-0.810817024	-0.090475098	0.694738216
ENSG00000177034	*MTX3*	2.169778625	-0.079138207	0.036709246	-0.115847453	0.999933887	-0.181063312	-0.046841892	-0.134221421	0.695161219
ENSG00000273599	*AL731571.1*	2.720722404	0.060247184	0.18665035	-0.126403167	0.999933887	-0.400746554	-0.483985696	0.083239142	0.695282216
ENSG00000239672	*NME1*	-0.714907141	0.027042253	-0.379422977	0.40646523	0.999933887	0.030872748	0.239965553	-0.209092806	0.695282216
ENSG00000070669	*ASNS*	-0.399698222	-0.131393866	0.411888636	-0.543282502	0.944094537	0.257070102	0.090194502	0.1668756	0.695654624
ENSG00000204977	*TRIM13*	3.589039198	-0.055737936	0.033287517	-0.089025453	0.999933887	0.002503276	0.063062986	-0.060559711	0.695753396
ENSG00000198856	*OSTC*	2.404370551	0.041681492	-0.025962703	0.067644195	0.999933887	-0.015891432	0.080245494	-0.096136926	0.695753396
ENSG00000165244	*ZNF367*	0.139138299	0.082810624	0.189203298	-0.106392674	0.999933887	-0.016723118	0.18673234	-0.203455458	0.695769076
ENSG00000182158	*CREB3L2*	3.315002158	-0.043996093	-0.024330421	-0.019665673	0.999933887	-0.443312495	-0.533771397	0.090458902	0.695901677
ENSG00000117593	*DARS2*	1.034128086	-0.028222883	0.075730815	-0.103953698	0.999933887	-0.00360712	0.111378443	-0.114985563	0.695901677
ENSG00000177000	*MTHFR*	5.933167442	-0.007492108	-0.002674014	-0.004818095	0.999933887	-0.393827104	-0.460076278	0.066249174	0.695901677
ENSG00000176148	*TCP11L1*	2.179633969	0.058834548	0.033476703	0.025357844	0.999933887	0.175111398	0.253492115	-0.078380717	0.696047436
ENSG00000180353	*HCLS1*	10.043929813	-0.041155162	-0.011916223	-0.029238939	0.999933887	-0.265619888	-0.310769851	0.045149963	0.696072702
ENSG00000144524	*COPS7B*	2.756467838	0.002174466	-0.054713076	0.056887542	0.999933887	-0.04163349	-0.119887716	0.078254226	0.69620461
ENSG00000130844	*ZNF331*	4.339649294	0.01307441	-0.008192562	0.021266972	0.999933887	0.045146145	0.105260313	-0.060114169	0.696298887
ENSG00000088356	*PDRG1*	2.319086372	-0.069516077	-0.147404041	0.077887964	0.999933887	0.106995283	0.01663764	0.090357643	0.696364375
ENSG00000066027	*PPP2R5A*	6.136905705	-0.008271163	0.030572867	-0.03884403	0.999933887	-0.629784703	-0.679906833	0.05012213	0.696364375
ENSG00000100196	*KDELR3*	2.061047413	0.008689124	0.134911303	-0.12622218	0.999933887	0.530375625	0.62978073	-0.099405106	0.696364375

ENSG00000107796	*ACTA2*	-0.117344576	-0.104074566	0.168252223	-0.272326789	0.999933887	0.295794054	0.501889601	-0.206095547	0.696582156
ENSG00000279413	*AC112497.2*	-0.44099141	-0.039687026	0.218994595	-0.258681621	0.999933887	-0.239179514	0.044180525	-0.283360039	0.696783691
ENSG00000172007	*RAB33B*	4.470890646	-0.108684252	-0.048617219	-0.060067033	0.999933887	-1.258414444	-1.350962733	0.092548288	0.696943586
ENSG00000179222	*MAGED1*	2.036912188	-0.045243057	-0.089502103	0.044259046	0.999933887	0.087341871	0.183012144	-0.095670272	0.696943586
ENSG00000272734	*ADIRF-AS1*	-0.088087251	-0.080742224	0.254258049	-0.335000273	0.999933887	0.05760432	0.233375062	-0.175770743	0.696943586
ENSG00000105676	*ARMC6*	1.82699951	-0.029621997	0.063836782	-0.093458779	0.999933887	0.015295562	-0.095775857	0.111071419	0.696943586
ENSG00000179082	*C9orf106*	0.79863968	-0.13343616	0.041454201	-0.174890362	0.999933887	-0.183964933	-0.042142745	-0.141822188	0.698004666
ENSG00000109762	*SNX25*	1.086877817	-0.067988863	-0.190059903	0.12207104	0.999933887	-0.018999938	-0.132083079	0.11308314	0.698004666
ENSG00000279476	*AC092139.2*	0.602704664	-0.008662582	-0.073772845	0.065110263	0.999933887	-0.070250258	0.138512625	-0.208762883	0.698004666
ENSG00000177301	*KCNA2*	-0.847337923	-0.099224473	0.029315691	-0.128540164	0.999933887	-0.023452726	0.214847445	-0.238300171	0.6981342
ENSG00000071246	*VASH1*	0.736993874	0.042295511	0.056577513	-0.014282002	0.999933887	0.096663395	-0.056603261	0.153266656	0.6981342
ENSG00000176809	*LRRC37A3*	0.584531094	-0.019498574	0.209100046	-0.228598619	0.999933887	-0.405309308	-0.226634195	-0.178675113	0.698212223
ENSG00000137815	*RTF1*	6.11990783	-0.075235176	-0.057648352	-0.017586824	0.999933887	-0.909527705	-0.976342836	0.066815131	0.698221285
ENSG00000181467	*RAP2B*	5.495051977	-0.075112049	-0.094568985	0.019456936	0.999933887	-0.442650139	-0.516880752	0.074230614	0.698437315
ENSG00000188906	*LRRK2*	8.324888565	-0.094537942	-0.153603665	0.059065724	0.999933887	-1.181733396	-1.311431638	0.129698242	0.698437315
ENSG00000243056	*EIF4EBP3*	1.041749917	0.046881819	0.097564114	-0.050682295	0.999933887	0.066304309	0.229299982	-0.162995673	0.698437315
ENSG00000272072	*AC004492.1*	1.863679924	-0.019054071	0.000266868	-0.019320939	0.999933887	0.116033464	0.299684175	-0.183650711	0.698437315
ENSG00000084693	*AGBL5*	1.896489432	0.006565859	0.168590991	-0.162025132	0.999933887	-0.068991501	-0.179308578	0.110317077	0.698437315
ENSG00000109083	*IFT20*	2.780792901	0.019350412	-0.030204296	0.049554708	0.999933887	-0.176325314	-0.256425972	0.080100658	0.698507828
ENSG00000109180	*OCIAD1*	4.59268772	-0.018246109	-0.056342441	0.038096332	0.999933887	-0.142934488	-0.189834304	0.046899816	0.698600678
ENSG00000163947	*ARHGEF3*	4.505540807	-0.048291942	-0.045174769	-0.003117173	0.999933887	0.059175368	0.005131367	0.054044002	0.69861252
ENSG00000204371	*EHMT2*	3.002138475	0.062814369	0.010218989	0.052595379	0.999933887	0.060736888	-0.017210382	0.07794727	0.69861252
ENSG00000110448	*CD5*	5.791137786	-0.007507942	-0.001505815	-0.006002127	0.999933887	0.134947431	0.185551912	-0.05060448	0.699044989
ENSG00000252061	*RNU6-415P*	-0.857556795	-0.087390117	0.129753472	-0.217143589	0.999933887	-0.081996634	0.186653971	-0.268650606	0.699066671
ENSG00000158863	*FAM160B2*	4.08975605	-0.010980547	0.148547376	-0.159527923	0.999933887	0.045813529	-0.01793847	0.063751999	0.699066671
ENSG00000197226	*TBC1D9B*	5.429299752	-0.007149801	0.016158305	-0.023308106	0.999933887	-0.32856288	-0.385084702	0.056521822	0.699066671
ENSG00000226167	*AP4B1-AS1*	-0.273908476	-0.149808993	0.016276189	-0.166085182	0.999933887	-0.154011208	0.068912569	-0.222923777	0.699248394
ENSG00000171657	*GPR82*	-0.174040738	-0.025585437	-0.274716172	0.249130734	0.999933887	-0.251929212	-0.075000905	-0.176928307	0.699248394
ENSG00000175556	*LONRF3*	5.110881304	0.002815206	-0.039878021	0.042693227	0.999933887	-0.23759516	-0.169378129	-0.068217031	0.699248394
ENSG00000256092	*SBNO1-AS1*	0.689176624	-0.0300451	0.255072137	-0.285117237	0.999933887	0.298608829	0.167152805	0.131456024	0.699314154
ENSG00000221914	*PPP2R2A*	6.102186003	-0.036913536	-0.047416655	0.010503119	0.999933887	-0.056573892	-0.095376118	0.038802226	0.699620142
ENSG00000142892	*PIGK*	1.662191951	0.032496436	0.01822511	0.014271326	0.999933887	-0.017592791	0.120681639	-0.13827443	0.699620142
ENSG00000180229	*HERC2P3*	0.065233566	-0.010594995	0.043437321	-0.054032316	0.999933887	-0.025513228	0.119891274	-0.145404503	0.699620142
ENSG00000138018	*SELENOI*	2.606460863	-0.036682434	-0.112347119	0.075664684	0.999933887	-0.099677062	-0.011850998	-0.087826065	0.699627404
ENSG00000133818	*RRAS2*	1.492727333	-0.084640586	-0.184255666	0.09961508	0.999933887	0.069372316	0.164967079	-0.095594763	0.699628575
ENSG00000066084	*DIP2B*	7.155654088	0.005438091	-0.02075029	0.026188381	0.999933887	-0.377893284	-0.429362992	0.051469707	0.699628575
ENSG00000165699	*TSC1*	5.828999197	-0.10380458	-0.14524271	0.04143813	0.999933887	-0.420780114	-0.368289777	-0.052490337	0.699647601
ENSG00000232160	*RAP2C-AS1*	0.337922936	0.21225864	-0.035405396	0.247664037	0.999933887	1.019077641	1.166825767	-0.147748125	0.699647601
ENSG00000213190	*MLLT11*	1.19513841	0.090783526	0.225470075	-0.134686548	0.999933887	1.417956868	1.595974553	-0.178017684	0.699647601
ENSG00000180138	*CSNK1A1L*	0.913011538	-0.061408063	-0.013284345	-0.048123718	0.999933887	0.417837769	0.228776188	0.189061581	0.699647601
ENSG00000164056	*SPRY1*	0.588881134	0.08080183	-0.043170685	0.123972515	0.999933887	-0.257343617	-0.043510834	-0.213832783	0.699647601
ENSG00000164124	*TMEM144*	-0.701690392	0.131320392	0.091160321	0.04016007	0.999933887	-0.975883691	-0.74245217	-0.233431521	0.699647601
ENSG00000175416	*CLTB*	5.351732029	0.028783752	0.027988858	0.000794894	0.999933887	0.098181402	0.034924177	0.063257224	0.699647601
ENSG00000109084	*TMEM97*	-0.773178739	-0.08559302	0.582859813	-0.668452833	0.969206975	0.054794383	0.277575057	-0.222780674	0.699647601
ENSG00000154945	*ANKRD40*	2.499125988	-0.029430972	-0.074579582	0.045148611	0.999933887	-0.009887399	0.066360804	-0.076248203	0.699647601
ENSG00000145623	*OSMR*	0.488299996	-0.073470407	0.35623094	-0.429701347	0.999933887	-0.57151398	-0.406895642	-0.164618339	0.699649141
ENSG00000244482	*LILRA6*	7.127842414	0.014287834	0.040531144	-0.02624331	0.999933887	-0.497694258	-0.451142656	-0.046551601	0.699649141
ENSG00000266777	*SH3GL1P1*	1.800172742	-0.027700913	0.065421853	-0.093122766	0.999933887	-0.448276819	-0.35918331	-0.089093509	0.699649141
ENSG00000142676	*RPL11*	7.71857764	-0.004113287	-0.063766012	0.059652725	0.999933887	0.086760232	0.145287467	-0.058527235	0.699649141
ENSG00000188897	*AC099489.1*	7.908405687	0.064712442	0.064684001	2.8441e-05	0.999933887	0.412107044	0.463360761	-0.051253716	0.699810976
ENSG00000179299	*NSUN7*	2.419921245	0.097033471	-0.103651518	0.200684989	0.999933887	-0.41377916	-0.297472684	-0.116306476	0.699810976
ENSG00000185163	*DDX51*	2.963045373	0.063797025	0.165269452	-0.101472428	0.999933887	0.132009117	0.210004754	-0.077995638	0.699810976
ENSG00000048162	*NOP16*	1.128776568	0.02407742	0.110114084	-0.086036663	0.999933887	0.217490375	0.346976934	-0.129486558	0.699810976
ENSG00000187583	*PLEKHN1*	-0.252300608	0.026007622	-0.065012564	0.091020186	0.999933887	0.248079444	0.444975695	-0.196896252	0.699810976
ENSG00000234771	*SLC25A25-AS*	2.254866235	0.010658915	-0.053401121	0.064060036	0.999933887	0.022319428	-0.066013758	0.088333186	0.699810976
ENSG00000067221	*STOML1*	0.951101677	0.076194903	-0.023060369	0.099255272	0.999933887	1.250666933	1.403126075	-0.152459142	0.70001643
ENSG00000273176	*AL008635.1*	-0.072520924	0.271253765	0.394219747	-0.122965981	0.999933887	1.947278664	2.12721384	-0.179935177	0.700056161
ENSG00000142733	*MAP3K6*	1.09693977	0.096785115	0.02957496	0.067210154	0.999933887	-0.223285293	-0.382369727	0.159084434	0.700056161
ENSG00000280211	*AC106886.4*	0.112786327	0.023433544	0.030795695	-0.007362151	0.999933887	0.138828009	-0.08856216	0.227390168	0.700056161
ENSG00000168824	*NSG1*	0.868160577	-0.062632032	-0.126269867	0.063637835	0.999933887	-0.076120191	-0.207287214	0.131167023	0.700068994
ENSG00000116874	*WARS2*	0.414577409	0.163954924	-0.111381099	0.275336022	0.999933887	0.306990065	0.45520882	-0.148218755	0.700101361
ENSG00000102189	*EEA1*	2.106812136	-0.167417349	-0.081793909	-0.085623441	0.999933887	-0.458271879	-0.285477915	-0.172793964	0.700101361
ENSG00000277494	*GPIHBP1*	-2.419676222	0.397250413	0.13982502	0.257425393	0.999933887	1.311003475	1.57631508	-0.265311606	0.700101361
ENSG00000101474	*APMAP*	6.885565823	-0.025217173	-0.039084996	0.013867823	0.999933887	0.118860387	0.166321105	-0.047460718	0.700101361
ENSG00000151500	*THYN1*	2.377915071	-0.029829739	-0.176819076	0.146989338	0.999933887	0.016457818	-0.071612771	0.08807059	0.700483506
ENSG00000230424	*EMC1-AS1*	-0.294940233	0.026536893	-0.213238443	0.239775336	0.999933887	0.79660333	1.015574771	-0.218971441	0.700505597
ENSG00000279447	*AL118508.3*	-0.89867552	-0.149181387	0.037133837	-0.186315224	0.999933887	-0.834068657	-1.155009837	0.32094118	0.700641867
ENSG00000106049	*HIBADH*	0.766286953	-0.028237758	-0.008098902	-0.020138856	0.999933887	-0.106889675	0.022884694	-0.129774369	0.701096703
ENSG00000159674	*SPON2*	3.473622232	0.002367055	0.065504905	-0.06313785	0.999933887	0.103579121	0.031387833	0.072191289	0.701096703
ENSG00000114767	*RRP9*	1.389873879	-0.009292207	0.164683845	-0.173976052	0.999933887	0.298654613	0.424001998	-0.125347384	0.701132326
ENSG00000270419	*CAHM*	2.139977689	0.108355227	0.137862844	-0.029507617	0.999933887	0.606078619	0.499478822	0.106599797	0.701295049
ENSG00000245164	*LINC00861*	3.419057897	-0.050901424	0.039870602	-0.090772026	0.999933887	-0.040744944	0.045205013	-0.085949957	0.701295049
ENSG00000205269	*TMEM170B*	5.06733645	-0.071450708	-0.095411929	0.023961221	0.999933887	-1.125270702	-1.022455238	-0.102815464	0.701563912
ENSG00000226891	*LINC01359*	0.84536877	-0.071627829	-0.182892428	0.1112646	0.999933887	-0.053627437	0.048478424	-0.102105861	0.701563912
ENSG00000120875	*DUSP4*	3.512267072	-0.012569557	-0.017950308	0.005380751	0.999933887	0.055968256	0.131429937	-0.075461681	0.702045013
ENSG00000264653	*MIR5194*	0.678874794	0.16789274	0.090712028	0.077180711	0.999933887	0.630427643	0.436688786	0.193738857	0.702213768
ENSG00000087365	*SF3B2*	7.202161306	-0.064850064	-0.045185165	-0.019664899	0.999933887	-0.187351131	-0.230080471	0.04272934	0.702297849
ENSG00000172954	*LCLAT1*	1.812289195	-0.14951142	-0.058880812	-0.090630608	0.999933887	-0.385229081	-0.495762356	0.110533275	0.702297849
ENSG00000006715	*VPS41*	4.944700035	-0.103366146	-0.00497086	-0.098395286	0.999933887	-0.41345774	-0.345355432	-0.068102308	0.702297849
ENSG00000081665	*ZNF506*	3.560195244	-6.2026e-05	0.026168112	-0.026230138	0.999933887	-0.200339908	-0.11128932	-0.089050589	0.702297849
ENSG00000081177	*EXD2*	0.020883324	0.163807147	-0.007792118	0.171599265	0.999933887	0.050469879	-0.098291832	0.148761711	0.702623613
ENSG00000129197	*RPAIN*	2.924223247	-0.071136878	-0.161924506	0.090787628	0.999933887	0.100358886	0.165240692	-0.064881806	0.702791892
ENSG00000225975	*LINC01534*	-0.175451408	-0.156186512	0.006275701	-0.162462214	0.999933887	-0.050957497	-0.212382496	0.161425	0.702791892
ENSG00000108387	*SEPTIN4*	-0.830324544	0.103955368	-0.15832616	0.262281528	0.999933887	0.139983345	-0.169753785	0.309737129	0.702791892
ENSG00000211677	*IGLC2*	0.940151179	-0.06878485	0.014879332	-0.083664181	0.999933887	0.211198731	0.384771146	-0.173572415	0.702791892
ENSG00000142556	*ZNF614*	0.831989678	-0.037965115	-0.00928838	-0.028676735	0.999933887	-0.156884508	-0.015973093	-0.140911415	0.702791892
ENSG00000087903	*RFX2*	5.240818004	0.009939504	0.053686572	-0.043747067	0.999933887	-0.412040747	-0.485980676	0.073939929	0.702791892
ENSG00000101280	*ANGPT4*	-0.654802809	0.000763825	-0.07901721	0.079781035	0.999933887	0.290952666	0.468317473	-0.177364807	0.702791892
ENSG00000285646	*AL021155.4*	0.335087651	0.178541388	0.352974689	-0.174433301	0.999933887	0.496898893	0.321563108	0.175335785	0.702853727
ENSG00000111962	*UST*	0.401425021	0.097929208	-0.297819239	0.395748447	0.999933887	0.003065003	-0.149248573	0.152313576	0.702853727
ENSG00000105443	*CYTH2*	4.761327284	0.099248376	0.125984924	-0.026736548	0.999933887	-0.252966192	-0.313749125	0.060782933	0.703215102
ENSG00000224043	*CCNT2-AS1*	-0.53998441	0.024430379	0.10934675	-0.084916371	0.999933887	-0.364050635	-0.195106065	-0.16894457	0.703215102
ENSG00000109133	*TMEM33*	5.313649083	0.018603945	0.052469482	-0.033865537	0.999933887	-0.528610883	-0.460588858	-0.068022025	0.70323376
ENSG00000269937	*AC093525.7*	0.569160418	0.17708298	-0.132128182	0.309211162	0.999933887	-0.614652685	-0.428073345	-0.18657934	0.703286791
ENSG00000070010	*UFD1*	5.587950266	-0.03412627	-0.079519092	0.045392822	0.999933887	-0.176517201	-0.219721626	0.043204425	0.703286791
ENSG00000212719	*LINC02693*	0.249304622	0.044325493	-0.224140517	0.26846601	0.999933887	-0.128557519	-0.258424628	0.129867109	0.703286791
ENSG00000224707	*E2F3-IT1*	-0.153775781	-0.028090355	-0.113395654	0.085305298	0.999933887	0.682059994	0.867657831	-0.185597837	0.703286791
ENSG00000116977	*LGALS8*	5.093220164	-0.047700593	-0.064009508	0.016308915	0.999933887	-0.472666148	-0.528570001	0.055903853	0.703298385
ENSG00000124226	*RNF114*	5.539944802	-0.020714467	-0.017175981	-0.003538487	0.999933887	0.190398513	0.142077253	0.04832126	0.70334096
ENSG00000186104	*CYP2R1*	2.485801439	-0.04367588	-0.202184648	0.158508768	0.999933887	-0.125361945	-0.218597687	0.093235742	0.703395006
ENSG00000277763	*AL138995.1*	-0.08128298	-0.092271397	-0.031148592	-0.061122805	0.999933887	-0.620556442	-0.797474315	0.176917873	0.703418589
ENSG00000285230	*RALY-AS1*	0.861166268	-0.094734052	-0.151049559	0.056315507	0.999933887	-0.072082051	-0.186504768	0.114422716	0.703492087
ENSG00000127412	*TRPV5*	1.329562721	-0.098260375	-0.073009082	-0.025251293	0.999933887	-0.667028594	-0.758899799	0.091871205	0.703523063
ENSG00000163349	*HIPK1*	7.493365046	-0.022168827	0.021954124	-0.044122952	0.999933887	-0.23546691	-0.280952515	0.045485604	0.703785226
ENSG00000005961	*ITGA2B*	0.776948581	0.110645952	0.123618517	-0.012972565	0.999933887	-0.043602847	0.129614224	-0.173217071	0.704199972

ENSG00000097007	*ABL1*	3.577225283	0.047646454	0.049114528	-0.001468074	0.999933887	0.011213607	-0.054411689	0.065625296	0.704310298
ENSG00000161395	*PGAP3*	2.198347015	0.005415715	-0.029417664	0.034833379	0.999933887	0.140254672	0.049713967	0.090540705	0.704312182
ENSG00000264773	*MIR4420*	1.327115852	0.157485876	0.340028981	-0.182543106	0.999933887	0.068266584	0.282248519	-0.213981934	0.70431238
ENSG00000196247	*ZNF107*	2.975775595	-0.093385678	-0.070373499	-0.023012179	0.999933887	-0.493776146	-0.336359313	-0.157416834	0.704514451
ENSG00000188706	*ZDHHC9*	1.407366013	0.084681711	0.036800394	0.047881317	0.999933887	0.243495218	0.379979454	-0.136484236	0.704514451
ENSG00000180376	*CCDC66*	2.019608598	0.072025489	-0.029602588	0.101628077	0.999933887	-0.028387243	0.081201341	-0.109588584	0.704514451
ENSG00000136935	*GOLGA1*	4.167435949	0.015042491	-0.021100897	0.036143388	0.999933887	-0.212770068	-0.26648801	0.053717942	0.704514451
ENSG00000213047	*DENND1B*	2.897626446	0.027346403	-0.220286908	0.247633311	0.999933887	-0.444372199	-0.294356221	-0.150015978	0.704514451
ENSG00000225205	*AC078883.1*	0.216473288	-0.092186352	-0.07712393	-0.015062422	0.999933887	-0.287939643	-0.476332736	0.188393093	0.704573185
ENSG00000245648	*KLRK1-AS1*	1.867892699	-0.080644199	0.031228439	-0.111872638	0.999933887	-0.05636241	0.061508253	-0.117870664	0.704718003
ENSG00000127804	*METTL16*	3.220582665	-0.04393915	-0.075356579	0.031417429	0.999933887	0.020839209	0.10298606	-0.082146851	0.70482229
ENSG00000182831	*C16orf72*	7.095011372	-0.01123937	0.002374086	-0.013613456	0.999933887	-0.313409647	-0.361876739	0.048467092	0.70482229
ENSG00000130270	*ATP8B3*	0.918106637	0.040537778	0.115429444	-0.074891665	0.999933887	-0.354790141	-0.51458112	0.159790979	0.704854919
ENSG00000222011	*FAM185A*	0.845699373	0.09410902	-0.203685459	0.297794479	0.999933887	-0.188277441	-0.323562108	0.135284667	0.705650154
ENSG00000204516	*MICB*	3.671973046	0.052248596	-0.066276506	0.118525101	0.999933887	0.326623637	0.400151441	-0.073527804	0.705707991
ENSG00000142453	*CARM1*	2.235483893	-0.02671668	0.020057897	-0.046774577	0.999933887	0.051442842	-0.03396423	0.085407073	0.705707991
ENSG00000140479	*PCSK6*	-0.47135876	-0.07371216	-0.529363318	0.455651158	0.999933887	-0.908483008	-1.066699015	0.158216007	0.705707991
ENSG00000128908	*INO80*	4.689052575	-0.036690153	-0.050889303	0.01419915	0.999933887	-0.016924829	-0.064772856	0.047848027	0.706398965
ENSG00000203497	*PDCD4-AS1*	4.9032263	0.053865865	0.041107399	0.012758466	0.999933887	0.452373197	0.513772379	-0.061399181	0.706430199
ENSG00000224579	*AC245052.2*	-0.181580618	0.304005886	0.269977792	0.034028094	0.999933887	0.461546048	0.129806266	0.331739782	0.706479445
ENSG00000188807	*TMEM201*	1.362914848	0.078193572	0.026857806	0.051335766	0.999933887	0.158159738	0.262314318	-0.10415458	0.706479445
ENSG00000003509	*NDUFAF7*	2.757072085	0.061291516	0.063007095	-0.001715579	0.999933887	0.007623736	0.081629033	-0.074005297	0.706479445
ENSG00000184924	*PTRHD1*	0.189660758	0.070638089	-0.235561142	0.306199232	0.999933887	-0.037858592	0.150111543	-0.187970135	0.706479445
ENSG00000248334	*WHAMMP2*	0.140053002	-0.204016368	0.45712892	-0.661145288	0.944094537	0.120303848	0.274738122	-0.154434275	0.706503649
ENSG00000054523	*KIF1B*	6.341885927	-0.037679422	-0.032752604	-0.004926818	0.999933887	0.202255905	0.26280731	-0.060551406	0.706606723
ENSG00000181027	*FKRP*	3.31547442	0.060835977	0.116367606	-0.055531628	0.999933887	-0.136601574	-0.07079506	-0.065806514	0.706727281
ENSG00000196440	*ARMCX4*	0.971502825	0.013089508	-0.235459969	0.248549477	0.999933887	0.043907241	0.188819058	-0.144911817	0.706727281
ENSG00000168036	*CTNNB1*	6.882105153	-0.066108593	-0.059152712	-0.006955881	0.999933887	-0.050478497	-0.016506762	-0.033971735	0.706959635
ENSG00000109107	*ALDOC*	2.506148369	-0.118011567	-0.057858218	-0.060153349	0.999933887	0.538931925	0.63632392	-0.097391995	0.706959635
ENSG00000283378	*CNTNAP3C*	-0.434576135	0.187418899	0.085967394	0.101451504	0.999933887	0.157604826	-0.079616561	0.237221387	0.706959635
ENSG00000050165	*DKK3*	-0.21557177	0.192457666	-0.130365548	0.322823214	0.999933887	0.145273513	0.296998988	-0.151725475	0.706959635
ENSG00000108424	*KPNB1*	7.28309505	-0.037576135	-0.068243942	0.030667808	0.999933887	0.090108779	0.136919246	-0.046810468	0.706959635
ENSG00000124172	*ATP5F1E*	6.417780424	-0.015310774	-0.033890588	0.018579815	0.999933887	-0.068729587	-0.12802837	0.059298783	0.706959635
ENSG00000215533	*LINC00189*	-0.741554854	-0.023051634	0.124188857	-0.147240491	0.999933887	-0.097643746	-0.352439764	0.254796018	0.706959635
ENSG00000118946	*PCDH17*	-1.804060607	0.207860688	0.2702585	-0.062397813	0.999933887	-0.18718564	0.016499548	-0.203685187	0.707003148
ENSG00000271119	*AC026412.3*	-0.773719498	-0.123276507	0.072243826	-0.195520333	0.999933887	-0.274623672	-0.044436809	-0.230186864	0.707003148
ENSG00000132254	*ARFIP2*	3.531385395	0.017962574	0.032014444	-0.01405187	0.999933887	0.160047715	0.101431244	0.058616471	0.707003148
ENSG00000145916	*RMND5B*	2.773180701	-0.005203018	0.140620589	-0.145823607	0.999933887	-0.038167291	-0.105392271	0.06722498	0.707003148
ENSG00000141030	*COPS3*	3.68312434	-0.002775721	-0.090753807	0.087978086	0.999933887	0.035655878	-0.029041367	0.064697245	0.707003148
ENSG00000115419	*GLS*	5.941133883	-0.001793492	-0.020340314	0.018546822	0.999933887	-0.4366821	-0.307839732	-0.128842368	0.707003148
ENSG00000198551	*ZNF627*	1.465103198	-0.013298513	-0.123256818	0.109958305	0.999933887	-0.276411638	-0.383532898	0.10712126	0.707015634
ENSG00000151882	*CCL28*	0.061035541	-0.047786363	-0.256359536	0.208573173	0.999933887	0.138548255	-0.000341602	0.138889857	0.70715721
ENSG00000171045	*TSNARE1*	4.534791615	0.021214869	0.078987619	-0.05777275	0.999933887	0.53859922	0.598554229	-0.059955009	0.70715721
ENSG00000158292	*GPR153*	0.7042171	0.153809094	0.150880586	0.002928507	0.999933887	0.158808348	0.009700907	0.149107441	0.707355059
ENSG00000106591	*MRPL32*	2.827441254	-0.065218978	-0.19806638	0.132847402	0.999933887	0.139512597	0.058034018	0.081478579	0.707355059
ENSG00000105738	*SIPA1L3*	2.993005571	0.0053299	0.052334659	-0.047004759	0.999933887	-0.091372117	-0.174419171	0.083047054	0.707355059
ENSG00000127084	*FGD3*	8.642832282	-0.00791354	-0.024870132	0.016956592	0.999933887	-0.658275594	-0.713594815	0.05531922	0.707513631
ENSG00000280798	*LINC00294*	0.785490023	-0.12585552	0.03116837	-0.157023889	0.999933887	-0.001821801	0.122249765	-0.124071566	0.707711822
ENSG00000176055	*MBLAC2*	-0.224244983	-0.131298074	0.211188236	-0.34248631	0.999933887	-0.050800586	0.129458373	-0.180258959	0.707711822
ENSG00000175073	*VCPIP1*	6.283958482	-0.170224933	-0.118053061	-0.052171872	0.999933887	-0.876638731	-0.949231519	0.072592788	0.707816219
ENSG00000287817	*AL096701.4*	0.276818705	-0.137780234	-0.030281698	-0.107498536	0.999933887	0.941501305	0.75684615	0.184655155	0.707990782
ENSG00000185187	*SIGIRR*	4.892617325	0.030880853	0.046915304	-0.016034451	0.999933887	0.078350145	0.019176903	0.059173241	0.707990782
ENSG00000169246	*NPIPB3*	1.761768481	0.04431311	0.101985269	-0.05767216	0.999933887	0.017125201	0.126939057	-0.109813857	0.707990782
ENSG00000261026	*AC105046.1*	5.703527611	0.202358057	0.064738025	0.137620032	0.999933887	0.955203709	1.047283082	-0.092079373	0.708061694
ENSG00000257878	*AC007298.2*	1.451405172	-0.119932569	-0.148509453	0.028576884	0.999933887	-0.474398039	-0.605763651	0.131365612	0.708189091
ENSG00000272054	*AC007390.1*	-0.404348461	-0.053101829	0.034896667	-0.087998496	0.999933887	-0.272416901	-0.052972142	-0.219444759	0.708189091
ENSG00000113161	*HMGCR*	5.591817522	-0.083982787	-0.139612524	0.055629737	0.999933887	0.080863028	0.029964505	0.050898523	0.708301254
ENSG00000250461	*AC122718.1*	0.194537023	0.255031614	0.14084766	0.114183954	0.999933887	0.293477532	0.518902333	-0.225424801	0.708301254
ENSG00000281103	*TRG-AS1*	2.406494585	-0.071325148	-0.15720819	0.085883042	0.999933887	0.06270309	0.14647122	-0.08376813	0.708301254
ENSG00000168566	*SNRNP48*	3.20213475	0.003829138	-0.063187494	0.067016632	0.999933887	-0.328693151	-0.447233336	0.118540185	0.708301254
ENSG00000214063	*TSPAN4*	1.202439821	0.005547307	-0.04530744	0.050854747	0.999933887	-0.936548858	-1.089087013	0.152538155	0.708323483
ENSG00000049089	*COL9A2*	2.573023145	0.079114342	0.251779237	-0.172664895	0.999933887	0.136513884	0.029625153	0.106888731	0.708679475
ENSG00000186205	*MTARC1*	1.431800531	-0.105652832	0.073597304	-0.179250135	0.999933887	-0.648772405	-0.832889315	0.18411691	0.708909311
ENSG00000198736	*MSRB1*	7.865855305	-0.073656927	-0.056012608	-0.017644318	0.999933887	-1.007307556	-1.096462884	0.089155328	0.708909311
ENSG00000160633	*SAFB*	7.240821712	-0.035061404	0.060378135	-0.095439539	0.999933887	-0.219547359	-0.263805603	0.044258244	0.708909311
ENSG00000242609	*CCDC137P*	0.06263823	0.106558636	0.253909175	-0.147350538	0.999933887	1.989566892	2.277940987	-0.288374095	0.708909311
ENSG00000106366	*SERPINE1*	0.855675773	0.152859128	0.112576821	0.040282307	0.999933887	0.22071948	0.350026998	-0.129307518	0.708909311
ENSG00000173085	*COQ2*	3.428939077	0.04182087	0.070392912	-0.028572041	0.999933887	-0.657616023	-0.729547879	0.071931856	0.708909311
ENSG00000166974	*MAPRE2*	5.53653362	-0.003911426	-0.05168506	0.047773634	0.999933887	-0.202288423	-0.145367031	-0.056921392	0.708909311
ENSG00000029364	*SLC39A9*	4.210828284	-0.013369626	-0.057120545	0.043750919	0.999933887	-0.143977795	-0.09250343	-0.051474365	0.708945503
ENSG00000281162	*LINC01127*	2.162752563	-0.021576733	0.017215468	-0.038792201	0.999933887	-0.336730013	-0.200290636	-0.136439377	0.709000628
ENSG00000004139	*SARM1*	1.440624232	0.013845745	0.043824539	-0.029978795	0.999933887	0.030412733	0.131231666	-0.100818933	0.709000628
ENSG00000116544	*DLGAP3*	-1.152017648	0.227537144	0.345459324	-0.11792218	0.999933887	0.505864397	0.241059664	0.264804733	0.709056264
ENSG00000159374	*M1AP*	-0.543979046	-0.138709778	-0.120284668	-0.01842511	0.999933887	-0.806044974	-1.031050543	0.225005569	0.709056264
ENSG00000279738	*AL022311.1*	0.861823208	0.024785899	-0.070585702	0.095371601	0.999933887	-0.306298999	-0.150483991	-0.155815008	0.709056264
ENSG00000101407	*TTI1*	3.024686051	-0.036109048	-0.042178965	0.006069916	0.999933887	-0.029603723	0.035364521	-0.064968244	0.709261655
ENSG00000170871	*KIAA0232*	6.82156945	-0.070778966	-0.10086469	0.030085724	0.999933887	-0.758065857	-0.688186263	-0.069879594	0.709331256
ENSG00000214022	*REPIN1*	7.345674947	-0.0728686	-0.065631624	-0.007236976	0.999933887	1.59485886	1.688250823	-0.093391963	0.709331256
ENSG00000148400	*NOTCH1*	9.847176335	0.047759619	0.065353084	-0.017593466	0.999933887	-0.723916462	-0.653193464	-0.070722998	0.709331256
ENSG00000242154	*AC004884.2*	0.103128282	-0.038753704	0.149882514	-0.188636218	0.999933887	0.400221248	0.587516369	-0.18729512	0.709331256
ENSG00000235655	*H3P6*	5.681533236	0.016439894	-0.016839413	0.033279307	0.999933887	-0.248003636	-0.182714079	-0.065289557	0.709331256
ENSG00000143740	*SNAP47*	1.798766536	-0.008011308	0.038079764	-0.046091072	0.999933887	0.035049275	-0.063215568	0.098264843	0.709331256
ENSG00000119138	*KLF9*	3.385867348	-0.025731143	-0.0695051	0.043773957	0.999933887	-0.104353389	-0.174010617	0.069657229	0.709611495
ENSG00000135318	*NT5E*	0.507817288	-0.150864132	-0.341516444	0.190652312	0.999933887	-0.515719043	-0.630394484	0.114675441	0.709657571
ENSG00000147099	*HDAC8*	0.49052478	-0.05210708	-0.000192838	-0.051914242	0.999933887	-0.104048149	0.059317198	-0.163365346	0.709657571
ENSG00000287926	*AC113208.5*	1.654538695	0.170966102	0.203357803	-0.032391701	0.999933887	1.231918029	1.40504541	-0.173127381	0.709705873
ENSG00000110060	*PUS3*	0.307189019	-0.156823444	0.131032625	-0.287856069	0.999933887	-0.072727984	0.090787494	-0.163515478	0.709780507
ENSG00000116459	*ATP5PB*	4.826247705	-0.069381474	-0.101348356	0.031966882	0.999933887	-0.183823997	-0.129950558	-0.053873439	0.70979478
ENSG00000103148	*NPRL3*	2.52565128	-0.083755692	0.093176867	-0.176932559	0.999933887	-0.07461544	-0.155394839	0.080779399	0.709898512
ENSG00000182308	*DCAF4L1*	-0.534683484	0.009209316	-0.259412632	0.268621948	0.999933887	-0.039648582	-0.238271558	0.198622976	0.709921595
ENSG00000214367	*HAUS3*	2.920276721	0.007981332	-0.056312493	0.064293825	0.999933887	-0.195437542	-0.291171584	0.095720042	0.710091958
ENSG00000132275	*RRP8*	3.063102942	-0.038047951	-0.023603229	-0.014444722	0.999933887	0.0230266	-0.041557301	0.0645839	0.710150025
ENSG00000153896	*ZNF599*	-0.64550272	0.022238638	0.036946604	-0.014707966	0.999933887	0.243096493	0.052589672	0.190506821	0.710150025
ENSG00000171848	*RRM2*	0.659284054	0.148653785	0.234206088	-0.085552303	0.999933887	0.10453829	-0.110940989	0.215479279	0.710292241
ENSG00000174292	*TNK1*	-0.153182826	-0.048701537	0.202478794	-0.251180332	0.999933887	-0.016351955	0.160158444	-0.176510398	0.71056236
ENSG00000197043	*ANXA6*	5.510069467	-0.016329555	-0.014694325	-0.001635229	0.999933887	0.132258322	0.184756185	-0.052497863	0.710611985
ENSG00000211767	*TRBJ2-3*	-0.511319474	0.139764849	0.089880337	0.049884512	0.999933887	0.143783912	-0.058123348	0.201907259	0.710658471
ENSG00000175592	*FOSL1*	4.346665732	0.309443158	0.156027519	0.153415639	0.999933887	1.349421233	1.457421996	-0.108000763	0.710703329
ENSG00000260196	*AC124798.1*	3.414109259	-0.121086019	-0.009392118	-0.111693901	0.999933887	3.165477385	3.282444503	-0.116967118	0.710703329
ENSG00000100983	*GSS*	2.352749157	-0.050951332	0.177852215	-0.228803547	0.999933887	0.005760579	0.081018924	-0.075258345	0.710703329
ENSG00000100503	*NIN*	7.351647122	-0.034224709	-0.079553531	0.045328823	0.999933887	-0.52446441	-0.615450058	0.090985648	0.710703329
ENSG00000283063	*TRBV6-2*	-0.591751435	0.059705719	0.264748657	-0.205042939	0.999933887	0.078563916	0.242712364	-0.164148448	0.710918659
ENSG00000006712	*PAF1*	6.214147497	0.048376897	0.040106675	0.008270221	0.999933887	0.196444563	0.247682631	-0.051238068	0.710966342
ENSG00000198954	*KIFBP*	1.907607726	0.063217614	-0.057534691	0.120752305	0.999933887	0.187875627	0.295724833	-0.107849205	0.710966342
ENSG00000281649	*EBLN3P*	5.632499423	0.024948587	-0.000742697	0.025691283	0.999933887	-0.411567835	-0.464281622	0.052713787	0.711137209

ENSG00000050426	*LETMD1*	3.265427427	-0.026879237	-0.033449699	0.006570462	0.999933887	0.23240103	0.290130273	-0.057729243	0.711159094
ENSG00000288082	*AC023787.3*	-0.00729563	0.033944795	-0.124525081	0.158469877	0.999933887	0.030632345	0.169054412	-0.138422068	0.711198505
ENSG00000142102	*PGGHG*	8.719245924	0.058554992	0.101719458	-0.043164466	0.999933887	-0.133855036	-0.184985387	0.051130351	0.711226767
ENSG00000104691	*UBXN8*	0.144962378	-0.148210548	-0.042414467	-0.105796081	0.999933887	-0.356382699	-0.507336631	0.150953932	0.711280613
ENSG00000186814	*ZSCAN30*	2.514548216	-0.096523381	-0.159451713	0.062928332	0.999933887	-0.355888204	-0.437806269	0.081918065	0.711364786
ENSG00000181045	*SLC26A11*	7.253574779	0.036680708	-0.024984555	0.061665264	0.999933887	0.252102657	0.312500033	-0.060397376	0.711364786
ENSG00000067057	*PFKP*	3.964391772	-0.025729945	0.167317465	-0.19304741	0.999933887	-0.05308024	-0.112585445	0.059505205	0.711364786
ENSG00000165669	*FAM204A*	2.954663128	-0.08284056	-0.037966491	-0.044874069	0.999933887	-0.090555547	-0.165747781	0.075192234	0.711436654
ENSG00000165702	*GFI1B*	1.437852787	0.114395197	0.150193878	-0.035798681	0.999933887	-0.025805434	0.061530731	-0.087336166	0.711508653
ENSG00000281571	*AC241585.2*	0.81122814	0.091867915	-0.224610596	0.316478511	0.999933887	0.319958779	0.184956489	0.13500229	0.711635182
ENSG00000184083	*FAM120C*	0.430320268	0.037989051	0.18398672	-0.145997669	0.999933887	0.027174931	-0.129787359	0.15696229	0.711762869
ENSG00000196873	*CBWD3*	0.950957393	0.1310137	-0.173193561	0.304207261	0.944094537	-0.019549559	-0.195122545	0.175572986	0.71182171
ENSG00000231329	*AL031772.1*	2.473582387	0.090650085	0.149663799	-0.059013714	0.999933887	-0.061537001	0.078793469	-0.14033047	0.71182171
ENSG00000143367	*TUFT1*	3.586024667	-0.029177893	0.063075638	-0.09225353	0.999933887	-0.173898233	-0.248960289	0.075062056	0.71182171
ENSG00000152443	*ZNF776*	3.959943161	-0.09130276	-0.142407029	0.051104269	0.999933887	-0.304587644	-0.244094025	-0.060493619	0.71184253
ENSG00000076716	*GPC4*	-0.045224031	-0.149116884	-0.069626354	-0.07949053	0.999933887	-0.913478716	-1.165457003	0.251978287	0.71196344
ENSG00000266401	*AP002478.1*	-0.835640852	-0.324569908	-0.274466301	-0.050103607	0.999933887	-0.549334151	-0.389007094	-0.160327056	0.712155083
ENSG00000196754	*S100A2*	0.289699311	0.126071017	0.089003821	0.037067196	0.999933887	-0.280027816	-0.118551415	-0.161476401	0.712155083
ENSG00000239483	*RPS15AP16*	0.650872372	-0.055304692	-0.12311838	0.067813688	0.999933887	0.33329235	0.147428954	0.185863396	0.712158566
ENSG00000101846	*STS*	1.048226579	-0.018577798	-0.094255278	0.07567748	0.999933887	-0.235948747	-0.406175862	0.170227115	0.712386439
ENSG00000268573	*AC011815.1*	0.020696802	-0.092847853	-0.032329566	-0.060518287	0.999933887	-0.541794432	-0.6915984	0.149803968	0.712484477
ENSG00000259699	*HMGB1P8*	-0.797536823	0.073673617	0.137087768	-0.063414151	0.999933887	-0.710671903	-0.379566735	-0.331105168	0.712735674
ENSG00000260563	*AC132872.1*	4.831397424	0.081613692	0.031320693	0.050292999	0.999933887	0.382558082	0.43883925	-0.056281168	0.7130115
ENSG00000104765	*BNIP3L*	6.868409788	-0.017589756	-0.073406117	0.055816361	0.999933887	-0.424690555	-0.379118831	-0.045571724	0.713105643
ENSG00000163138	*PACRGL*	0.239863802	-0.02429623	-0.045041559	0.020745328	0.999933887	-0.038114087	0.123743486	-0.161857573	0.713295252
ENSG00000187790	*FANCM*	0.324245189	-0.07322238	-0.062129314	-0.011093066	0.999933887	-0.311165483	-0.11656282	-0.194602663	0.713383291
ENSG00000132323	*ILKAP*	3.909470336	-0.038749909	0.054664171	-0.09341408	0.999933887	0.113328715	0.163639063	-0.050310348	0.713639931
ENSG00000226012	*AP001434.1*	3.054618408	-0.018636612	0.181375244	-0.200011856	0.999933887	-0.459902384	-0.290241749	-0.169660635	0.713791131
ENSG00000164307	*ERAP1*	5.09749908	0.042758603	-0.03008689	0.072845494	0.999933887	-0.457179791	-0.382833535	-0.074346256	0.714003342
ENSG00000164187	*LMBRD2*	2.128877447	0.03021606	-0.134250183	0.164466243	0.999933887	-0.385174002	-0.225700163	-0.159473838	0.714127373
ENSG00000188522	*FAM83G*	2.749714734	0.125571469	-0.032131743	0.157703212	0.999933887	-0.059339485	0.017314413	-0.076653898	0.714206623
ENSG00000276248	*AL442125.1*	1.20490478	-0.242600065	-0.184572338	-0.058027727	0.999933887	-0.870430456	-0.73448782	-0.135942636	0.714774142
ENSG00000117595	*IRF6*	-0.169345995	-0.097156578	-0.003954317	-0.09320226	0.999933887	-0.441341703	-0.638423505	0.197081802	0.714785395
ENSG00000164610	*RP9*	0.247005269	-0.033737242	-0.238171394	0.204434152	0.999933887	0.048844526	0.204168834	-0.155324308	0.714881642
ENSG00000170606	*HSPA4*	5.238308429	-0.118549739	-0.17318177	0.054632031	0.999933887	-0.241003515	-0.191742819	-0.049260695	0.714929328
ENSG00000110871	*COQ5*	0.630247844	-0.06755658	-0.061468419	-0.006088161	0.999933887	0.061273274	0.180083763	-0.11881049	0.714929328
ENSG00000180182	*MED14*	5.043353414	-0.029496613	-0.058009938	0.028513325	0.999933887	-0.165041591	-0.219974798	0.054933206	0.714929328
ENSG00000258315	*C17orf49*	1.704010611	0.2379052	0.251862492	-0.013957292	0.999933887	0.626116714	0.744367709	-0.118250995	0.715137131
ENSG00000264270	*AC087749.1*	0.284006533	0.138199831	0.10535759	0.032842241	0.999933887	0.714079996	0.508064362	0.206015634	0.715137131
ENSG00000215302	*AC127502.1*	-0.199530621	0.088355735	0.214885914	-0.12653018	0.999933887	0.028507287	0.223639203	-0.195131916	0.715137131
ENSG00000198785	*GRIN3A*	-0.177919295	-0.03094406	-0.005374064	-0.025569996	0.999933887	-0.57516481	-0.377879811	-0.197284999	0.715137131
ENSG00000180626	*ZNF594*	-0.764690342	-0.122948879	0.145770786	-0.268719665	0.999933887	-0.155969354	0.016899757	-0.172869111	0.715282433
ENSG00000237798	*AC010894.3*	0.239798183	-0.031345894	0.01160419	-0.042950084	0.999933887	-0.168931747	0.02855788	-0.197489627	0.715282433
ENSG00000103319	*EEF2K*	2.473996927	-0.012487914	0.127489754	-0.139977668	0.999933887	0.050930908	0.136651348	-0.08572044	0.715282433
ENSG00000225101	*OR52K3P*	4.130524214	0.058605277	-0.030876054	0.08948133	0.999933887	-0.236551515	-0.294243651	0.057692136	0.715309836
ENSG00000249086	*AC051649.1*	-1.169033137	0.241091781	0.118375205	0.122716576	0.999933887	-0.493216709	-0.036143779	-0.45707293	0.715598951
ENSG00000183527	*PSMG1*	0.996293301	0.07379192	-0.000974188	0.074766108	0.999933887	0.177070973	0.312091294	-0.135020321	0.715853122
ENSG00000115806	*GORASP2*	3.790888884	-0.022855083	0.017689376	-0.040544459	0.999933887	0.131963945	0.183136907	-0.051172962	0.715853122
ENSG00000188352	*FOCAD*	2.51401443	-0.01657882	-0.100702857	0.084124036	0.999933887	-0.013344841	-0.100357316	0.087012475	0.715853122
ENSG00000088451	*TGDS*	0.571696496	-0.021651798	-0.170061408	0.14840961	0.999933887	-0.028521862	0.092343422	-0.120865285	0.715878481
ENSG00000155893	*PXYLP1*	1.933188804	0.000559507	0.124023697	-0.12346419	0.999933887	-0.044015968	0.056764772	-0.100780741	0.715878481
ENSG00000165282	*PIGO*	2.720508613	0.068773121	0.000647895	0.068125226	0.999933887	0.105340976	0.037689711	0.067651265	0.715883211
ENSG00000156299	*TIAM1*	2.812480059	-0.05123736	-0.075645185	0.024407825	0.999933887	0.099803408	0.035681759	0.06412165	0.716194876
ENSG00000094880	*CDC23*	1.774421216	-0.042962011	-0.116345217	0.073383206	0.999933887	0.177889913	0.091226434	0.086663479	0.716194876
ENSG00000106772	*PRUNE2*	-0.108149358	-0.033151042	0.190217657	-0.223368699	0.999933887	-0.916324873	-0.735435217	-0.180889655	0.716194876
ENSG00000153885	*KCTD15*	0.618540073	0.002781033	0.08204754	-0.079266508	0.999933887	-0.186891169	-0.321917804	0.135026635	0.716194876
ENSG00000148814	*LRRC27*	-0.728889868	0.004105796	0.241766733	-0.237660937	0.999933887	-0.121371562	-0.291698066	0.170326504	0.716194876
ENSG00000171747	*LGALS4*	-0.849003354	-0.181963482	-0.09784677	-0.084116713	0.999933887	-0.110875671	0.118157405	-0.229033076	0.716319206
ENSG00000185875	*THNSL1*	-1.175917046	0.143298667	-0.299097568	0.442396235	0.999933887	0.197692911	0.406088741	-0.20839583	0.716611625
ENSG00000105366	*SIGLEC8*	1.718356557	0.062890276	0.014975982	0.047914294	0.999933887	0.020708844	0.107331537	-0.086622693	0.71664492
ENSG00000284735	*AL139424.3*	-0.331062308	-0.201586737	-0.318237209	0.116650472	0.999933887	-0.322141148	-0.582376161	0.260235014	0.716799959
ENSG00000276291	*FRG1HP*	1.530329179	0.129404395	-0.137645754	0.267050149	0.999933887	-0.202754714	-0.315281297	0.112526583	0.716799959
ENSG00000142655	*PEX14*	1.753983111	0.025994122	0.008606982	0.017387141	0.999933887	0.092912656	0.181500609	-0.088587953	0.716799959
ENSG00000143793	*C1orf35*	2.403532402	0.090603543	0.058307996	0.032295547	0.999933887	0.021696759	-0.076035639	0.097732398	0.71697534
ENSG00000173409	*ARV1*	-0.240898319	-0.082666611	-0.235446305	0.152779694	0.999933887	-0.002170423	0.149113586	-0.15128401	0.716975034
ENSG00000273576	*AC009283.1*	0.258423405	-0.031412202	-0.195184142	0.16377194	0.999933887	0.246452492	0.117818047	0.128634446	0.716975034
ENSG00000173706	*HEG1*	2.96284901	-0.129942336	0.015494082	-0.145436418	0.999933887	0.086639338	0.017016205	0.069623132	0.717011264
ENSG00000263370	*AC104564.1*	-0.427850296	-0.118834098	-0.009331156	-0.109502942	0.999933887	0.088050107	-0.13018896	0.218239067	0.717178857
ENSG00000175283	*DOLK*	0.748431727	0.030469292	0.009115019	0.021354273	0.999933887	0.417004639	0.549345611	-0.132340972	0.717178857
ENSG00000244731	*C4A*	-0.091176797	0.068391309	0.00796649	0.060424819	0.999933887	0.326295271	0.502242728	-0.175947457	0.717198526
ENSG00000034533	*ASTE1*	1.692819798	-0.008850553	-0.120043412	0.111192859	0.999933887	0.027936208	0.135151755	-0.107215547	0.717271954
ENSG00000188818	*ZDHHC11*	-0.275617124	-0.05294694	-0.219742162	0.166795222	0.999933887	0.162957527	0.345560263	-0.182602736	0.717399994
ENSG00000270157	*AC004918.3*	0.438285612	-0.015095199	0.159122582	-0.174217781	0.999933887	0.015757081	-0.111153498	0.12691058	0.717540724
ENSG00000106415	*GLCCI1*	3.788025843	0.037281571	-0.058675795	0.095957366	0.999933887	-0.143150998	-0.212119983	0.068968984	0.717686568
ENSG00000132300	*PTCD3*	3.819797603	-0.073618727	-0.145643496	0.072024769	0.999933887	-0.000169144	-0.055517292	0.055348148	0.717758594
ENSG00000260059	*AC092620.2*	0.045654247	-0.226578726	0.042855243	-0.269433969	0.999933887	0.392209087	0.585766381	-0.193557295	0.717758594
ENSG00000187862	*TTC24*	0.599613005	-0.01904222	0.070442358	-0.089484578	0.999933887	0.043182795	-0.075501628	0.118684423	0.717758594
ENSG00000137500	*CCDC90B*	2.567994004	0.045841031	-0.026382443	0.072223474	0.999933887	-0.131851547	-0.061576212	-0.070275335	0.717838345
ENSG00000143333	*RGS16*	0.680692543	-0.149988708	-0.051422477	-0.098566231	0.999933887	0.428075696	0.55448648	-0.126410784	0.718035779
ENSG00000174669	*SLC29A2*	0.506616492	0.055628239	-0.128726343	0.184354582	0.999933887	0.089429697	-0.03685091	0.126280608	0.718132503
ENSG00000142864	*SERBP1*	5.537526612	-0.065267304	-0.04236712	-0.022900184	0.999933887	-0.154785109	-0.192076338	0.037291229	0.71823322
ENSG00000259953	*AL138756.1*	1.011861979	0.04306834	-0.008898362	0.051966702	0.999933887	0.018797961	0.122268683	-0.103470722	0.718342662
ENSG00000107338	*SHB*	-0.494363612	0.205222614	0.225348309	-0.020125696	0.999933887	-0.623071086	-0.366059296	-0.257011789	0.718491445
ENSG00000145736	*GTF2H2*	-0.147854792	0.220502689	-0.152306671	0.37280936	0.999933887	-0.723668086	-0.927760906	0.204092821	0.718491445
ENSG00000107798	*LIPA*	3.794693599	0.12410995	0.112914662	0.011195288	0.999933887	-0.000781826	-0.126997232	0.126215406	0.718491445
ENSG00000267216	*ZNF8-ERVK3-*	0.138576573	-0.07195357	0.024812507	-0.096766077	0.999933887	-0.211971601	-0.365901822	0.153930221	0.718491445
ENSG00000179761	*PIPOX*	-0.231932295	-0.146494369	-0.200253785	0.053759417	0.999933887	-0.00220046	0.173007509	-0.175207969	0.718574235
ENSG00000135116	*HRK*	0.532258698	0.015111825	0.020235051	-0.005123226	0.999933887	-0.194894125	-0.377405907	0.182511782	0.71871303
ENSG00000136682	*CBWD2*	2.605509413	-0.004695796	-0.082429631	0.077733835	0.999933887	-0.335611206	-0.43675027	0.101139064	0.718883132
ENSG00000231551	*AC245100.4*	1.57088492	0.055500073	-0.019862505	0.075362578	0.999933887	-0.144525983	-0.038082645	-0.106443338	0.718897111
ENSG00000250312	*ZNF718*	0.121951347	-0.139908234	-0.290088571	0.150180338	0.999933887	-0.007407774	-0.168608987	0.161201212	0.718930633
ENSG00000260651	*AF213884.3*	0.487681069	0.296246303	0.18277506	0.113471242	0.999933887	2.172392073	1.991384889	0.181007184	0.719034408
ENSG00000242616	*GNG10*	0.135084271	-0.011889471	0.14795034	-0.159839811	0.999933887	0.451800944	0.693903329	-0.242102386	0.719060268
ENSG00000166333	*ILK*	0.812401472	-0.078045838	0.185248106	-0.263293944	0.999933887	0.160422632	0.252285196	-0.091862564	0.719138809
ENSG00000155085	*AK9*	1.27297519	-0.002830631	0.014781588	-0.017612219	0.999933887	-0.579196324	-0.474155459	-0.105040864	0.719138809
ENSG00000107201	*DDX58*	5.354510849	-0.120077139	-0.124813436	0.004736297	0.999933887	-0.444163057	-0.372847268	-0.071315789	0.719276327
ENSG00000204618	*RNF39*	2.538005309	0.042698274	0.037290237	0.005408037	0.999933887	0.422726211	0.32761968	0.095106531	0.719276327
ENSG00000272849	*AC084018.1*	0.897677659	-0.137317747	0.000582464	-0.137900211	0.999933887	-0.192626067	-0.094223737	-0.09840233	0.719339337
ENSG00000284194	*SCO2*	1.917511511	0.088855496	0.038537942	0.050317554	0.999933887	-0.246175939	-0.405431703	0.159255764	0.719471144
ENSG00000286536	*AL591506.1*	-0.279481316	-0.230093625	-0.089546372	-0.140547254	0.999933887	-0.059735287	-0.182336079	0.122600792	0.719471144
ENSG00000109534	*GAR1*	1.911125448	0.060967691	0.020310711	0.040656979	0.999933887	-0.015655162	-0.123129393	0.10747423	0.719471144
ENSG00000226824	*AC006001.2*	0.737955401	0.056737491	0.049978383	0.006759108	0.999933887	-0.108340998	0.018812833	-0.127153831	0.719471144
ENSG00000119650	*IFT43*	0.930958903	0.185657565	-0.169847665	0.355505231	0.999933887	0.221019177	0.083421991	0.137597186	0.719471625
ENSG00000215190	*LINC00680*	0.04926727	-0.11933162	0.130825513	-0.250157133	0.999933887	-0.120489643	0.038658392	-0.159148035	0.719471625
ENSG00000171858	*RPS21*	6.513293907	0.029966611	-0.016738448	0.04670506	0.999933887	0.113766693	0.171282166	-0.057515473	0.719471625

ENSG00000144659	*SLC25A38*	3.29167427	-0.028617754	-0.114556872	0.085939117	0.999933887	0.153340347	0.217019833	-0.063679487	0.719471625
ENSG00000170145	*SIK2*	3.377472042	-0.010482251	0.006234602	-0.016716853	0.999933887	-0.026320331	0.035846811	-0.062167142	0.719471625
ENSG00000174547	*MRPL11*	1.393919376	0.055573643	0.029265487	0.026308156	0.999933887	0.157428702	0.03900243	0.118426272	0.720039791
ENSG00000256987	*AC023050.5*	1.259813475	-0.081266331	0.030323267	-0.111589597	0.999933887	-0.011498175	0.159228084	-0.170726259	0.720150793
ENSG00000136206	*SPDYE1*	0.014937352	-0.086206502	-0.133815574	0.047609073	0.999933887	0.023325009	0.19385583	-0.170530821	0.720487228
ENSG00000115758	*ODC1*	5.463890749	-0.00400753	-0.023135835	0.019128305	0.999933887	0.141087986	0.194448392	-0.053360406	0.720495923
ENSG00000273702	*AC091271.1*	2.409291301	0.22777614	0.11534828	0.11242786	0.999933887	0.736459346	0.833175843	-0.096716497	0.720998031
ENSG00000183624	*HMCES*	4.892282401	0.061466195	-0.039141948	0.100608143	0.999933887	-0.111210853	-0.05532634	-0.055884513	0.720998031
ENSG00000228264	*PSMD8P1*	1.429408515	0.142609899	0.099963037	0.042646862	0.999933887	0.858608431	1.03851479	-0.179906359	0.720998031
ENSG00000186854	*TRABD2A*	2.631552551	0.047099742	0.054746981	-0.007647238	0.999933887	0.051276708	-0.009215548	0.060492257	0.720998031
ENSG00000138131	*LOXL4*	-0.780620559	0.115095557	0.067047341	0.048048215	0.999933887	0.121721331	-0.107026971	0.228748302	0.720998031
ENSG00000277879	*AL391988.1*	0.7218305	-0.068899634	-0.103519124	0.03461949	0.999933887	-0.316622078	-0.524835798	0.20821372	0.720998031
ENSG00000008083	*JARID2*	8.29506645	-0.028197648	0.031036346	-0.059233994	0.999933887	-0.360823328	-0.319219877	-0.041603451	0.720998031
ENSG00000249790	*AC092490.1*	-1.290004301	0.061337107	-0.249037452	0.310374559	0.999933887	-0.731935849	-0.518428958	-0.213506891	0.720998031
ENSG00000185436	*IFNLR1*	0.625844193	-0.023134025	-0.11774715	0.094613125	0.999933887	0.238288353	0.12448176	0.113806593	0.720998031
ENSG00000259207	*ITGB3*	-1.038186922	-0.042546423	-0.145581507	0.103035083	0.999933887	-0.508922039	-0.245668018	-0.263254022	0.720998031
ENSG00000253948	*VPS13B-DT*	0.217786323	0.014569645	0.054819819	-0.040250174	0.999933887	-0.122502759	0.081719768	-0.204222527	0.720998031
ENSG00000173744	*AGFG1*	5.773051851	-0.000357624	-0.027028228	0.026670604	0.999933887	-0.175685872	-0.130683226	-0.045002646	0.720998031
ENSG00000227039	*ITGB2-AS1*	5.170472667	0.020660822	0.0181417	0.002519123	0.999933887	0.215741035	0.267668593	-0.051927559	0.721243048
ENSG00000141447	*OSBPL1A*	2.843187851	-0.002094579	0.045476098	-0.047570677	0.999933887	0.062234579	-0.02486626	0.087100839	0.721379435
ENSG00000120885	*CLU*	1.118689784	-0.04643479	-0.075741062	0.029306272	0.999933887	-0.089810997	0.0591688	-0.148979797	0.72143778
ENSG00000107874	*CUEDC2*	2.806493183	0.025862498	-0.022447388	0.048309886	0.999933887	0.189298258	0.281225916	-0.091927658	0.721686147
ENSG00000197892	*KIF13B*	3.482458475	-0.070841056	0.000852897	-0.071693953	0.999933887	0.049069212	-0.004908773	0.053977985	0.721798134
ENSG00000163214	*DHX57*	1.065748398	-0.069094058	0.116182586	-0.185276645	0.999933887	-0.129317975	-0.000964964	-0.128353011	0.721798134
ENSG00000174611	*KY*	7.856388323	0.104851768	0.141574421	-0.036722653	0.999933887	0.163483037	0.228574318	-0.065091281	0.721921655
ENSG00000177380	*PPFIA3*	-0.635255808	-0.10479743	-0.04791257	-0.05688486	0.999933887	0.025221049	-0.17210958	0.197330628	0.721921655
ENSG00000226660	*TRBV2*	0.650520428	-0.084020564	-0.118996973	0.034976408	0.999933887	0.020494625	0.135223055	-0.11472843	0.722053337
ENSG00000154165	*GPR15*	1.442777815	-0.078093313	-0.010741035	-0.067352278	0.999933887	0.204631015	0.089289871	0.115341144	0.722053337
ENSG00000180189	*HMGB1P14*	-0.587194416	0.063819603	0.058419979	0.005399624	0.999933887	0.222815476	0.395407065	-0.17259159	0.722166577
ENSG00000079785	*DDX1*	2.547291043	-0.057204131	-0.09887709	0.041672959	0.999933887	0.093370279	0.00880047	0.084569808	0.722314013
ENSG00000093183	*SEC22C*	4.131481887	-0.056251256	0.024443992	-0.080695248	0.999933887	-0.045038844	-0.098618845	0.053580001	0.722677185
ENSG00000107959	*PITRM1*	3.897068586	-0.02566402	0.121504607	-0.147168627	0.999933887	0.003476118	0.066801753	-0.063325635	0.722677185
ENSG00000119318	*RAD23B*	6.679208385	0.008677147	0.004753811	0.003923337	0.999933887	-0.336423586	-0.295856652	-0.040566934	0.722677185
ENSG00000105329	*TGFB1*	8.590452757	0.026302139	0.016528756	0.009773383	0.999933887	-0.2447255	-0.304722604	0.059997104	0.722832028
ENSG00000089157	*RPLP0*	7.48249216	-0.002317327	-0.040357203	0.038039876	0.999933887	0.131137736	0.182929741	-0.051792005	0.723093989
ENSG00000001460	*STPG1*	-0.490226796	-0.199532581	-0.314379387	0.114846807	0.999933887	-0.146837905	0.044260182	-0.191098087	0.723275948
ENSG00000204642	*HLA-F*	8.800286993	0.075294614	0.037820063	0.037474551	0.999933887	0.818450137	0.871035919	-0.052585782	0.723546784
ENSG00000116251	*RPL22*	3.733343003	-0.017570969	-0.110953682	0.093382713	0.999933887	0.084454728	0.155139693	-0.070684966	0.723546784
ENSG00000161298	*ZNF382*	-0.101014963	-0.004433554	-0.0936155	0.089181946	0.999933887	-0.026333559	-0.20552411	0.179190551	0.723546784
ENSG00000266970	*AC061992.2*	1.454127587	0.188130109	0.260030688	-0.071900579	0.999933887	0.360897532	0.498561421	-0.137663889	0.723582544
ENSG00000167037	*SGSM1*	-0.508701097	0.106084017	-0.071182232	0.177266249	0.999933887	-0.065733474	0.132753972	-0.198487446	0.723582544
ENSG00000177628	*GBA*	6.259669333	0.025159348	-0.075214211	0.10037356	0.999933887	-0.515790658	-0.574841915	0.059051257	0.723582544
ENSG00000235618	*FAM21EP*	0.237589523	-0.055478847	-0.036333822	-0.019145025	0.999933887	0.376494913	0.511394585	-0.134899671	0.723582544
ENSG00000237541	*HLA-DQA2*	0.429479265	-0.033805861	0.197461343	-0.231267204	0.999933887	0.730602817	0.894141502	-0.163538685	0.723582544
ENSG00000136243	*NUP42*	1.441974814	-0.019297524	-0.000829919	-0.018467605	0.999933887	-0.267623421	-0.379948547	0.112325126	0.72358557
ENSG00000115364	*MRPL19*	1.033618745	-0.110808689	-0.129687302	0.018878613	0.999933887	0.164704696	0.279040977	-0.114336281	0.72366304
ENSG00000154760	*SLFN13*	3.645254113	-0.004930104	-0.042854089	0.037923985	0.999933887	-0.141993078	-0.082403274	-0.059589804	0.724121613
ENSG00000106244	*PDAP1*	5.368198945	1.1729e-05	-0.016253433	0.016265162	0.999933887	0.035783849	-0.01084914	0.046632989	0.724255343
ENSG00000120262	*CCDC170*	0.81399594	0.134168278	-0.216762878	0.350931157	0.944094537	-0.059968595	0.099269902	-0.159238497	0.724511441
ENSG00000215193	*PEX26*	3.46780992	-0.01402814	-0.005200216	-0.008827923	0.999933887	-0.128592504	-0.185635838	0.057043334	0.724587559
ENSG00000143353	*LYPLAL1*	2.004802403	-0.187351041	-0.013684215	-0.173666826	0.999933887	-0.923710065	-1.019513358	0.095803293	0.724645386
ENSG00000117305	*HMGCL*	3.447739872	0.071698847	-0.009256969	0.080955816	0.999933887	-0.356272068	-0.286981605	-0.069290464	0.724645386
ENSG00000056736	*IL17RB*	-0.927103605	0.016645956	0.493546377	-0.476900422	0.999933887	0.384917488	0.205953307	0.178964181	0.724645386
ENSG00000081692	*JMJD4*	-0.738820978	0.190036956	0.063262645	0.12677431	0.999933887	0.228491947	0.427745787	-0.199253839	0.724791677
ENSG00000119392	*GLE1*	4.056491576	-0.014837251	0.044428769	-0.059266019	0.999933887	-0.379855132	-0.437606594	0.057751462	0.724791677
ENSG00000107371	*EXOSC3*	1.797221323	-0.179876249	-0.00195033	-0.177925919	0.999933887	-0.289998875	-0.193711918	-0.096286958	0.72495461
ENSG00000236609	*ZNF853*	0.72596596	0.091876903	-0.055583243	0.147460147	0.999933887	-0.021237734	-0.128203693	0.106965958	0.724966782
ENSG00000259476	*AC018904.2*	1.772617384	0.281959695	0.421209596	-0.139249901	0.999933887	0.802657277	0.943774401	-0.141117124	0.72502786
ENSG00000165548	*TMEM63C*	0.375333353	0.085157672	-0.067868234	0.153025906	0.999933887	-0.276438838	-0.445196874	0.168758035	0.72502786
ENSG00000069248	*NUP133*	3.545795198	-0.020912786	-0.016816141	-0.004096646	0.999933887	-0.102698733	-0.033873497	-0.068825237	0.72502786
ENSG00000228474	*OST4*	4.419979283	0.016315927	-0.049938844	0.06625477	0.999933887	0.094763724	0.041987041	0.052776683	0.72502786
ENSG00000204438	*GPANK1*	3.814709772	-0.003004692	0.093269377	-0.096274068	0.999933887	-0.371970197	-0.445613538	0.073643341	0.72502786
ENSG00000137207	*YIPF3*	6.745971581	-0.051734993	-0.094879839	0.043144846	0.999933887	-0.190100622	-0.146039312	-0.04406131	0.725265521
ENSG00000148337	*CIZ1*	4.257051601	0.034357455	-0.020421008	0.054778462	0.999933887	0.097821302	0.151974769	-0.054153467	0.725421435
ENSG00000143870	*PDIA6*	3.773972494	-0.040044127	-0.093375107	0.05333098	0.999933887	0.018164152	-0.041580446	0.059744598	0.725464872
ENSG00000117751	*PPP1R8*	3.043806197	-0.050948045	-0.076293715	0.02534567	0.999933887	0.012859782	-0.056291268	0.06915105	0.725464872
ENSG00000063601	*MTMR1*	4.70816589	0.015574163	0.013732086	0.001842077	0.999933887	-0.118735452	-0.174439086	0.055703634	0.725464872
ENSG00000160087	*UBE2J2*	5.501623059	-0.000999235	-0.068480239	0.067481004	0.999933887	-0.121994491	-0.079084299	-0.042910192	0.725464872
ENSG00000280326	*AC067931.2*	-0.115262023	-0.002111833	-0.001358687	-0.000753146	0.999933887	0.836879466	1.026727828	-0.189848362	0.725464872
ENSG00000262823	*AC127521.1*	-0.351608402	0.095217776	-0.131582064	0.22679984	0.999933887	-0.081661203	-0.245810538	0.164149336	0.725584758
ENSG00000108559	*NUP88*	3.079617464	-0.017426463	-0.173150459	0.155723997	0.999933887	0.151966969	0.213142277	-0.061175309	0.725584758
ENSG00000067601	*PMS2P4*	-0.026912845	-0.010889487	-0.180954723	0.170065236	0.999933887	0.073094789	0.237995953	-0.164901164	0.725584758
ENSG00000172803	*SNX32*	1.3018888	0.147069663	0.185556369	-0.038486706	0.999933887	0.254822922	0.361802179	-0.106979258	0.725627872
ENSG00000197162	*ZNF785*	-0.582551363	0.287263511	0.509096882	-0.22183337	0.999933887	-0.071481461	0.10783108	-0.17931254	0.725627872
ENSG00000099899	*TRMT2A*	5.557007557	0.029331263	0.008665067	0.020666196	0.999933887	0.194734823	0.258777036	-0.064042213	0.725627872
ENSG00000106351	*AGFG2*	2.640750896	-0.003135844	0.089755125	-0.092890969	0.999933887	0.037691834	-0.03570461	0.073396444	0.725627872
ENSG00000259456	*ADNP-AS1*	0.259342346	-0.153044286	-0.160543883	0.007499597	0.999933887	0.035006446	-0.104232552	0.139238998	0.725672966
ENSG00000155189	*AGPAT5*	2.119929028	0.043958708	-0.006034654	0.049993363	0.999933887	0.313937246	0.227358792	0.086578454	0.725672966
ENSG00000171928	*TVP23B*	3.148332108	-0.071417471	-0.220851256	0.149433785	0.999933887	-0.23153464	-0.15569285	-0.075841789	0.725873022
ENSG00000079739	*PGM1*	3.048839818	-0.052693429	0.01602728	-0.068720709	0.999933887	-0.120704604	-0.215744385	0.095039781	0.725964017
ENSG00000183570	*PCBP3*	2.652778051	-0.022001569	0.037565725	-0.059567294	0.999933887	0.2850424	0.35178947	-0.06674707	0.726022074
ENSG00000228956	*SATB1-AS1*	1.07035655	0.178923796	0.179134509	-0.000210713	0.999933887	-0.069160342	0.066340226	-0.135500569	0.72604531
ENSG00000103044	*HAS3*	-0.029445483	0.254541004	-0.167664185	0.422205189	0.999933887	-0.154824119	0.017939594	-0.172763712	0.72604531
ENSG00000161558	*TMEM143*	0.73299561	0.089155758	0.004828267	0.084327492	0.999933887	-0.06518862	0.070382556	-0.135571177	0.72604531
ENSG00000286330	*AL353660.1*	-0.735411079	-0.077799756	-0.512792274	0.434992518	0.999933887	-0.219660152	-0.006668799	-0.212991352	0.72604531
ENSG00000164038	*SLC9B2*	1.416534918	-0.026506979	-0.119744529	0.093237549	0.999933887	0.291977435	0.395312557	-0.103335122	0.72604531
ENSG00000154144	*TBRG1*	4.982545246	-0.019905031	-0.003163669	-0.016741362	0.999933887	-0.029949516	0.015398866	-0.045348382	0.726425623
ENSG00000240891	*PLCXD2*	3.236293149	-0.094623003	-0.009898246	-0.084724757	0.999933887	-0.064558986	-0.132293983	0.067734997	0.726525992
ENSG00000116191	*RALGPS2*	3.063682915	-0.160432844	-0.071537211	-0.088895633	0.999933887	-0.505944166	-0.615976286	0.11003212	0.726535264
ENSG00000211807	*TRAV26-1*	-1.034573697	0.058282272	0.330331955	-0.272049683	0.999933887	0.243015192	0.448213536	-0.205198344	0.726535264
ENSG00000169397	*RNASE3*	-0.568852878	0.04037245	0.25479312	-0.214420671	0.999933887	0.194750241	0.478921886	-0.284171646	0.726535264
ENSG00000134909	*ARHGAP32*	-0.219852675	-0.289523861	0.228751492	-0.518275353	0.999933887	0.026968179	0.194667092	-0.167698913	0.72663729
ENSG00000088826	*SMOX*	-0.749495471	-0.023418752	-0.127968358	0.104549606	0.999933887	-0.307681118	-0.500785492	0.193104373	0.72663729
ENSG00000234750	*AC012618.2*	0.270005386	-0.010886184	0.201399792	-0.212285976	0.999933887	0.197667519	0.425658914	-0.227991395	0.72663729
ENSG00000183023	*SLC8A1*	4.013204786	0.020870351	-0.015990319	0.03686067	0.999933887	-0.039842314	-0.126454959	0.086612645	0.726776388
ENSG00000160233	*LRRC3*	1.074450977	-0.029499478	-0.060306689	0.030807211	0.999933887	0.566194764	0.683941818	-0.117747054	0.726776388
ENSG00000243696	*AC006254.1*	-0.268767268	-0.113542814	0.281556081	-0.395098895	0.999933887	0.234277108	0.421944567	-0.18766746	0.726806515
ENSG00000025434	*NR1H3*	0.087679997	0.120424194	0.080967233	0.039456961	0.999933887	0.087051547	-0.092509126	0.179560673	0.726976067
ENSG00000123643	*SLC36A1*	6.176686765	0.015367152	0.025616474	-0.010249321	0.999933887	-0.768005847	-0.710524395	-0.057481452	0.727251847
ENSG00000239350	*AC105265.1*	-0.945483643	-0.034026586	0.114773155	-0.148799741	0.999933887	2.064864432	1.767407508	0.297456924	0.727251847
ENSG00000189067	*LITAF*	12.030693643	-0.056925377	-0.045304321	-0.011621056	0.999933887	-0.085030948	-0.041656338	-0.04337461	0.727506568
ENSG00000167523	*SPATA33*	0.511267446	0.123987416	0.194168327	-0.07018091	0.999933887	-0.114509658	-0.24223236	0.127722702	0.727506568
ENSG00000008710	*PKD1*	4.035547443	0.080069224	0.132348785	-0.052279562	0.999933887	0.076364006	0.002968519	0.073395486	0.727668678
ENSG00000249252	*C1QTNF7-AS1*	-0.540522694	0.179996928	-0.222814317	0.402811245	0.999933887	-0.158723416	-0.367510745	0.208787329	0.728100856
ENSG00000213753	*CENPBD1P1*	4.646874084	0.051240752	0.037630594	0.013610158	0.999933887	0.003531012	-0.051770795	0.055301807	0.728229216

ENSG00000282508	*LINC01002*	5.163174135	0.05147684	-0.042347216	0.093824056	0.999933887	0.433667269	0.487405044	-0.053737775	0.728437401
ENSG00000220848	*RPS18P9*	-0.13154467	0.043155	-0.034620663	0.077775663	0.999933887	-0.549658628	-0.741107408	0.191448781	0.728437401
ENSG00000173391	*OLR1*	3.838502921	-0.020512684	0.047906253	-0.068418937	0.999933887	1.0449918	1.213019305	-0.168027505	0.728686407
ENSG00000147419	*CCDC25*	1.869747344	-0.060146596	-0.161144689	0.100998092	0.999933887	0.099247711	0.183019517	-0.083771807	0.728692189
ENSG00000104907	*TRMT1*	3.445416871	0.052747625	0.137565969	-0.084818344	0.999933887	0.124938719	0.052554315	0.072384403	0.728791276
ENSG00000155962	*CLIC2*	-0.269809204	-0.030306584	0.134182109	-0.164488693	0.999933887	0.476262331	0.651895486	-0.175633155	0.728802441
ENSG00000283633	*AP000547.3*	0.767016739	0.031722738	0.08663509	-0.054912352	0.999933887	-0.167042691	-0.3106647	0.143622009	0.728902651
ENSG00000287913	*AL132986.1*	1.922341386	0.193907547	0.137628112	0.056279436	0.999933887	0.636892932	0.766416656	-0.129523724	0.729422636
ENSG00000128923	*MINDY2*	4.086208947	0.064332154	-0.065613263	0.129945417	0.999933887	-1.128933519	-1.016833089	-0.11210043	0.729422636
ENSG00000284642	*AL139424.2*	-0.424522766	-0.011190096	0.349035108	-0.360225205	0.999933887	-0.197185964	-0.411467302	0.214281338	0.729422636
ENSG00000164983	*TMEM65*	4.13412656	0.106075001	0.15931642	-0.053241419	0.999933887	-0.603349484	-0.667549954	0.06420047	0.729569564
ENSG00000071082	*RPL31*	7.305313223	0.020014424	-0.06087105	0.080885474	0.999933887	0.097861275	0.15119379	-0.053332515	0.729614564
ENSG00000199753	*SNORD104*	-0.34502939	-0.089065459	0.298229393	-0.387294851	0.999933887	0.076070661	0.303511332	-0.227440671	0.729767348
ENSG00000077721	*UBE2A*	5.726089759	-0.023844191	-0.032718623	0.008874433	0.999933887	-0.168017551	-0.128931313	-0.039086238	0.729974584
ENSG00000113569	*NUP155*	2.950314782	0.032359499	9.4272e-05	0.032265227	0.999933887	0.060451543	-0.011981705	0.072433248	0.730287952
ENSG00000177485	*ZBTB33*	3.797178995	-0.026304234	-0.157253847	0.130949613	0.999933887	-0.890701056	-0.787298184	-0.103402872	0.730376311
ENSG00000260257	*AL035071.1*	3.073154065	-0.101585878	0.06464116	-0.166227038	0.999933887	-0.070900179	0.018419859	-0.089320038	0.730615253
ENSG00000206567	*AC022007.1*	0.894479786	-0.045511603	0.174930293	-0.220441896	0.999933887	-0.938767506	-1.05240742	0.113639914	0.730615253
ENSG00000164105	*SAP30*	0.39362078	0.209506333	0.343180394	-0.133674062	0.999933887	0.095288516	-0.060189738	0.155478254	0.730801973
ENSG00000036549	*ZZZ3*	2.874305253	-0.127047534	-0.177991067	0.050943533	0.999933887	-0.317206848	-0.237189103	-0.080017745	0.730905641
ENSG00000274986	*MIR6750*	-0.143497586	0.165894926	0.164628973	0.001265953	0.999933887	0.26721774	0.059573072	0.207644668	0.730994868
ENSG00000272918	*AC005070.3*	1.738614448	-0.025523553	0.018441301	-0.043964854	0.999933887	-0.143085101	-0.006350187	-0.136734915	0.731020195
ENSG00000130396	*AFDN*	3.338178059	-0.004454164	0.003886759	-0.008340923	0.999933887	-0.097538003	-0.157967106	0.060429103	0.731020195
ENSG00000083642	*PDS5B*	4.365729559	-0.080150909	-0.095761474	0.015610565	0.999933887	-0.485531802	-0.35879889	-0.126732912	0.731160214
ENSG00000163344	*PMVK*	1.172059523	0.030074046	0.010891447	0.019182599	0.999933887	0.073081333	0.207903106	-0.134821773	0.731160214
ENSG00000117614	*SYF2*	6.175771244	-0.095551051	-0.178695023	0.083143972	0.999933887	-0.236767223	-0.18733293	-0.049434293	0.731185865
ENSG00000211749	*TRBV23-1*	-0.55601509	0.110864796	0.358341139	-0.247476343	0.999933887	0.141852719	-0.020138582	0.161991302	0.731185865
ENSG00000217644	*AL355864.1*	-0.167896667	-0.134072115	0.197324204	-0.331396319	0.999933887	1.710445101	1.51326178	0.197183321	0.731213715
ENSG00000069509	*FUNDC1*	0.326986045	-0.083503908	-0.102801593	0.019297685	0.999933887	-0.225174334	-0.351645353	0.126471018	0.731352183
ENSG00000264269	*AC016866.1*	-1.693017855	0.075551593	0.075538168	1.3425e-05	0.999978571	1.780164515	2.00419642	-0.224031905	0.731560933
ENSG00000233927	*RPS28*	7.064592306	0.057344785	0.008008005	0.049336779	0.999933887	0.221947143	0.268332653	-0.046385509	0.731703475
ENSG00000183530	*PRR14L*	5.378771164	-0.080116743	-0.042299378	-0.037817364	0.999933887	-0.892097534	-0.973107968	0.081010433	0.731703475
ENSG00000205542	*TMSB4X*	11.28846123	0.035224681	-0.003059338	0.038284019	0.999933887	0.372441233	0.421119451	-0.048678219	0.731703475
ENSG00000221025	*MIR1250*	1.19307633	0.073528876	-0.016941206	0.090470082	0.999933887	0.194289022	0.313433308	-0.119144286	0.73197257
ENSG00000101811	*CSTF2*	1.107239815	-0.165828714	0.046481829	-0.212310543	0.999933887	0.15391196	0.268066988	-0.114155027	0.732051921
ENSG00000213859	*KCTD11*	0.676635902	0.210395082	0.105947399	0.104447682	0.999933887	0.031441152	0.147929305	-0.116488153	0.732188445
ENSG00000089234	*BRAP*	4.52032067	-0.068613236	-0.000346525	-0.068266711	0.999933887	-0.310449428	-0.256037632	-0.054411797	0.732188445
ENSG00000250548	*LINC01303*	1.691628028	-0.005721135	-0.136767167	0.131046032	0.999933887	1.100849051	1.22199295	-0.121143899	0.732283679
ENSG00000286314	*AC010359.3*	2.759828499	0.053490368	0.320712928	-0.26722256	0.999933887	1.075646818	1.194726258	-0.11907944	0.732326723
ENSG00000135040	*NAA35*	2.742436751	-0.021639913	-0.065363737	0.043723825	0.999933887	-0.017036492	0.061639459	-0.078675951	0.732326723
ENSG00000129219	*PLD2*	3.36590836	-0.006429454	0.023352774	-0.029782228	0.999933887	-0.124136589	-0.064622293	-0.059514296	0.732368069
ENSG00000218186	*KRT8P43*	0.369775413	0.115518514	0.056184446	0.059334069	0.999933887	-0.534190647	-0.418829408	-0.11536124	0.732410641
ENSG00000196323	*ZBTB44*	5.045737993	-0.038195259	-0.056277429	0.01808217	0.999933887	-0.629369585	-0.70305818	0.073688595	0.732664837
ENSG00000205930	*C21orf62-AS1*	-0.660210275	0.296805763	-0.018804597	0.315610361	0.999933887	-0.217895484	-0.392714831	0.174819347	0.732884013
ENSG00000179988	*PSTK*	1.542932171	0.001849843	-0.014995025	0.016844868	0.999933887	-0.081906634	-0.187282606	0.105375972	0.732884013
ENSG00000188825	*LINC00910*	2.224712381	0.00123033	0.030551649	-0.029321319	0.999933887	0.189291571	0.11784697	0.071444602	0.733161627
ENSG00000110944	*IL23A*	1.483275053	-0.067701516	0.065363955	-0.133065472	0.999933887	1.49298087	1.659812918	-0.166832048	0.733165303
ENSG00000125246	*CLYBL*	-0.778738097	-0.041420259	-0.208448313	0.167028054	0.999933887	0.113211349	-0.112248572	0.225459921	0.733165303
ENSG00000115756	*HPCAL1*	7.726908009	0.075315889	0.043232787	0.032083103	0.999933887	-1.013824323	-0.948229022	-0.065595301	0.733546998
ENSG00000096092	*TMEM14A*	-0.579158289	0.167610718	-0.050782695	0.218393413	0.999933887	0.056933115	0.286747353	-0.229814238	0.733546998
ENSG00000228022	*HCG20*	-0.068618987	0.036139907	0.233536524	-0.197396617	0.999933887	1.057110579	1.192344836	-0.135234257	0.733546998
ENSG00000275993	*SIK1B*	1.573468581	-0.005219329	0.035523206	-0.040742536	0.999933887	0.216970957	0.0961677	0.120803257	0.733546998
ENSG00000001629	*ANKIB1*	3.567337698	0.02671047	-0.113144993	0.139855462	0.999933887	-0.212687835	-0.308603657	0.095915822	0.73373155
ENSG00000059758	*CDK17*	5.727222996	0.01950247	-0.016784753	0.036287224	0.999933887	-0.439382517	-0.504967509	0.065584991	0.733794106
ENSG00000173163	*COMMD1*	0.674071517	0.012619036	-0.21137494	0.223993977	0.999933887	0.151793831	0.278478783	-0.126684952	0.733979837
ENSG00000287405	*AC017028.2*	-0.199919156	0.383008235	0.215109521	0.167898714	0.999933887	-0.429287578	-0.648389806	0.219102227	0.734013876
ENSG00000275835	*TUBGCP5*	1.339110761	0.020309572	0.006709274	0.013600298	0.999933887	0.12085391	0.2467455	-0.12589159	0.734089648
ENSG00000115694	*STK25*	3.94658971	0.022908307	0.054150235	-0.031241928	0.999933887	0.101486727	0.042126444	0.059360283	0.734173815
ENSG00000185164	*NOMO2*	2.195837923	0.102071811	-0.032799363	0.134871174	0.999933887	-0.031904978	-0.125678903	0.093773926	0.73454346
ENSG00000173418	*NAA20*	2.296177075	-0.086878234	-0.020339297	-0.066538937	0.999933887	-0.09396078	-0.011018073	-0.082942707	0.73454346
ENSG00000121989	*ACVR2A*	0.406455676	-0.284640224	0.046339997	-0.330980221	0.999933887	0.126790135	0.250575168	-0.123785033	0.73482732
ENSG00000117139	*KDM5B*	6.391901791	-0.027833884	-0.094291274	0.06645739	0.999933887	-0.411878144	-0.3698309	-0.042047244	0.73482732
ENSG00000172315	*TP53RK*	2.051695891	-0.115749094	-0.087473218	-0.028275875	0.999933887	0.167640073	0.086768399	0.080871674	0.73523807
ENSG00000170430	*MGMT*	0.676910726	0.031757515	-0.085729997	0.117487512	0.999933887	0.127411853	0.006084047	0.121327806	0.735253902
ENSG00000286887	*AL355881.1*	3.828587303	0.13651323	0.067433096	0.069080135	0.999933887	1.115799717	1.212192265	-0.096392548	0.735448247
ENSG00000138434	*ITPRID2*	6.344433352	-0.008125989	-0.005295234	-0.002830755	0.999933887	-0.476996388	-0.537362478	0.06036609	0.735452481
ENSG00000169239	*CA5B*	2.737005241	-0.02142058	-0.018765216	-0.002655364	0.999933887	0.124714169	0.19213968	-0.067425511	0.736218929
ENSG00000232334	*AL683842.1*	0.970851789	-0.106788067	-0.061972902	-0.044815164	0.999933887	-0.467893691	-0.62148712	0.153593429	0.736293823
ENSG00000140548	*ZNF710*	6.601415067	0.027212275	0.049387058	-0.022174784	0.999933887	0.033659696	-0.00996598	0.043625676	0.736293823
ENSG00000223865	*HLA-DPB1*	4.805908025	0.026177154	0.001911424	0.02426573	0.999933887	0.080047691	0.015183668	0.064864023	0.736293823
ENSG00000158526	*TSR2*	3.396680049	0.03502936	0.088175905	-0.053146545	0.999933887	-0.088965753	-0.016119123	-0.072846631	0.736538506
ENSG00000132467	*UTP3*	2.182795741	-0.090877649	-0.206939197	0.116061548	0.999933887	0.092348285	0.171596053	-0.079247768	0.736682452
ENSG00000095485	*CWF19L1*	4.530939352	-0.019322512	0.016660422	-0.035982933	0.999933887	-0.035792283	0.022920905	-0.058713187	0.736682452
ENSG00000007038	*PRSS21*	-0.632737503	0.238601859	-0.066816465	0.305418324	0.999933887	-0.154182243	0.01590888	-0.170091123	0.736764606
ENSG00000114021	*NIT2*	2.031379651	-0.138832172	-0.096294844	-0.042537328	0.999933887	0.125569272	0.050297802	0.07527147	0.737146677
ENSG00000167208	*SNX20*	6.106782438	-0.005830942	0.004345521	-0.010176464	0.999933887	0.292360963	0.340917219	-0.048556255	0.737146677
ENSG00000117560	*FASLG*	1.5304672	-0.049319709	-0.024713842	-0.024605868	0.999933887	0.086164168	0.174708281	-0.088544114	0.737256181
ENSG00000008382	*MPND*	1.039722295	0.088694968	-0.041548682	0.13024365	0.999933887	0.210359517	0.056790025	0.153569491	0.737308639
ENSG00000164346	*NSA2*	4.068089694	-0.017663647	-0.087349376	0.069685728	0.999933887	0.070583891	0.129491872	-0.05890798	0.737308639
ENSG00000043143	*JADE2*	5.372925861	0.013991249	-0.022439949	0.036431199	0.999933887	0.131821812	0.177317591	-0.045495779	0.737308639
ENSG00000171466	*ZNF562*	2.707759359	-0.089420079	-0.033910259	-0.05550982	0.999933887	-0.110267281	-0.166040241	0.05577296	0.737311374
ENSG00000135605	*TEC*	2.144944072	-0.07666579	-0.031033781	-0.04563201	0.999933887	-0.288265986	-0.372002196	0.08373621	0.737311374
ENSG00000215114	*UBXN2B*	5.799142309	-0.051415253	-0.069547308	0.018132054	0.999933887	-0.811843493	-0.87313742	0.061293927	0.737311374
ENSG00000142794	*NBPF3*	1.29660567	-0.091411298	-0.051522234	-0.039889064	0.999933887	0.239058385	0.353886143	-0.114827758	0.737311374
ENSG00000136238	*RAC1*	6.961292704	-0.039730509	-0.030215865	-0.009514644	0.999933887	0.125310006	0.070670679	0.054639327	0.737311374
ENSG00000174516	*PELI3*	1.003970599	0.070589548	-0.044999645	0.115589193	0.999933887	-0.164719155	-0.26770835	0.102989195	0.737311374
ENSG00000198496	*NBR2*	1.226085896	-0.051649142	-0.098533466	0.046884324	0.999933887	-0.122608886	-0.013076281	-0.109532605	0.737311374
ENSG00000196284	*SUPT3H*	0.330970279	0.048938161	-0.111887512	0.160825672	0.999933887	0.065175925	-0.070961761	0.136137686	0.737311374
ENSG00000268947	*AC002128.1*	-0.365782142	0.006117333	0.153242749	-0.147125416	0.999933887	-0.061975536	0.080653836	-0.142629371	0.737311374
ENSG00000155926	*SLA*	7.683759331	-0.063910772	-0.084479712	0.02056894	0.999933887	0.028190689	-0.023342393	0.051533082	0.737334793
ENSG00000134809	*TIMM10*	2.109201991	-0.059453351	0.150291982	-0.209745334	0.999933887	0.133791937	0.0540346	0.079757337	0.737334793
ENSG00000162231	*NXF1*	7.12725089	0.014732878	0.031393667	-0.016660789	0.999933887	0.148357202	0.10266662	0.045690582	0.737334793
ENSG00000171817	*ZNF540*	-0.238609994	0.056941134	-0.256524191	0.313465325	0.999933887	-0.182465178	-0.017838018	-0.16462716	0.737334793
ENSG00000198755	*RPL10A*	7.107225029	-0.004804377	-0.040159716	0.035355339	0.999933887	0.126328981	0.177105208	-0.050776227	0.737334793
ENSG00000134830	*C5AR2*	7.265078062	-0.078390451	-0.104300512	0.02591006	0.999933887	-0.746456216	-0.799973606	0.05351739	0.737431291
ENSG00000223884	*AC068481.1*	0.11615288	-0.189968458	0.100022196	-0.289990654	0.999933887	-0.052848332	-0.204681212	0.15183288	0.737431291
ENSG00000173585	*CCR9*	-1.001412628	-0.013406949	0.132080285	-0.145487235	0.999933887	0.407597327	0.218967057	0.18863027	0.737431291
ENSG00000139083	*ETV6*	6.497421762	-0.022613676	-0.037039957	0.014426281	0.999933887	0.058899826	0.106448646	-0.04754882	0.737694737
ENSG00000085265	*FCN1*	5.773229922	0.05674082	0.03948768	0.01725314	0.999933887	-0.111111811	-0.188873286	0.077761474	0.73769625
ENSG00000204584	*AC027801.1*	0.087329117	0.104111961	0.181688602	-0.077576641	0.999933887	-0.221953944	-0.407494645	0.185540701	0.73769625
ENSG00000175294	*CATSPER1*	0.065408809	-0.044779673	-0.182992639	0.138212966	0.999933887	-0.814310398	-0.973947061	0.159636663	0.73769625
ENSG00000089091	*DZANK1*	-0.160111456	-0.029846635	0.094465746	-0.124312381	0.999933887	0.022909474	0.20213124	-0.179221766	0.737703346
ENSG00000285851	*AL359762.3*	1.203269488	-0.155312254	0.015784001	-0.171096255	0.999933887	-0.373951255	-0.264645573	-0.109305682	0.737933313
ENSG00000162994	*CLHC1*	-0.278011758	-0.226490688	-0.43102107	0.204530382	0.999933887	-0.285290543	-0.111959614	-0.173330928	0.738308588
ENSG00000274963	*RN7SL600P*	1.735790167	0.109976745	0.043835703	0.066141041	0.999933887	0.199614505	0.338282845	-0.13866834	0.738427528

ENSG00000178295	*GEN1*	1.423689809	-0.072139888	-0.061762088	-0.0103778	0.999933887	-0.053494118	0.106238331	-0.15973245	0.738671011
ENSG00000129158	*SERGEF*	0.520640174	0.048237931	-0.008659217	0.056897148	0.999933887	0.07948096	0.195685728	-0.116204769	0.738701296
ENSG00000119772	*DNMT3A*	6.006380137	0.023126879	0.090571313	-0.067444434	0.999933887	-0.440615161	-0.490941998	0.050326837	0.738711268
ENSG00000243836	*WDR86-AS1*	-0.297112308	0.005380427	0.072893541	-0.067513114	0.999933887	0.157109069	-0.01299617	0.170105239	0.738831461
ENSG00000166896	*ATP23*	0.878327472	0.109784434	-0.176199404	0.285983838	0.999933887	0.219944879	0.353863459	-0.13391858	0.739185815
ENSG00000258634	*AL160006.1*	2.294099273	-0.087829948	0.089107505	-0.176937453	0.999933887	-0.141105412	-0.046718294	-0.094387118	0.739542075
ENSG00000254017	*IGHEP2*	-0.193945386	0.046158716	-0.132514926	0.178673642	0.999933887	0.237643464	0.511766916	-0.274123452	0.739577722
ENSG00000229926	*IL9RP1*	-2.098262352	-0.416839765	-0.282242851	-0.134596914	0.999933887	-1.394493035	-1.622096286	0.227603251	0.73978914
ENSG00000047230	*CTPS2*	-0.475824338	-0.113989893	-0.358974785	0.244984892	0.999933887	0.11467189	-0.074351792	0.189023682	0.73978914
ENSG00000125650	*PSPN*	-0.2239958	-0.15842639	0.350066579	-0.508492969	0.999933887	-0.072495544	0.086276166	-0.158771709	0.739797955
ENSG00000085224	*ATRX*	5.400542951	-0.047194683	-0.135534504	0.088339821	0.999933887	-0.606982352	-0.453197905	-0.153784447	0.739934813
ENSG00000099998	*GGT5*	0.440038645	0.033464785	0.187354773	-0.153889988	0.999933887	0.7932002	0.961449228	-0.168249028	0.740189191
ENSG00000259877	*AC009113.1*	1.189723757	-0.193993772	-0.09847433	-0.095519442	0.999933887	-0.27970981	-0.376016841	0.096307031	0.740335636
ENSG00000205927	*OLIG2*	4.892813323	-0.157010777	-0.062528451	-0.094482327	0.999933887	-1.532332468	-1.654757597	0.12242513	0.740430545
ENSG00000159921	*GNE*	3.123382113	0.048099276	0.099332054	-0.051232779	0.999933887	-0.128846473	-0.192583289	0.063736816	0.740621754
ENSG00000165322	*ARHGAP12*	3.033758347	-0.108730456	-0.207842683	0.099112227	0.999933887	-0.547435517	-0.449394083	-0.098041433	0.740710264
ENSG00000253408	*AC083973.1*	-0.743437195	0.313822783	0.478023855	-0.164201072	0.999933887	1.954209787	2.1756278	-0.221418012	0.740783821
ENSG00000130368	*MAS1*	-2.236674706	0.065301007	-0.383747337	0.449048344	0.999933887	2.899413361	3.14508443	-0.245671069	0.740783821
ENSG00000127334	*DYRK2*	4.16935723	0.052766202	-0.031139918	0.08390612	0.999933887	-0.082299584	-0.028076006	-0.054223578	0.740822482
ENSG00000224034	*LINC02561*	-0.381701949	0.138468292	-0.088467109	0.226935401	0.999933887	-0.097675175	0.035238548	-0.132913723	0.740843929
ENSG00000105968	*H2AZ2*	5.618767568	0.013103195	0.005932409	0.007170786	0.999933887	-0.120673375	-0.084168366	-0.03650501	0.740843929
ENSG00000076650	*GPATCH1*	1.558910917	0.018996624	0.004153817	0.014842807	0.999933887	-0.071776161	-0.182988032	0.111211871	0.740880461
ENSG00000159399	*HK2*	6.224262966	0.004357705	0.005004177	-0.000646472	0.999933887	-0.187073857	-0.136946098	-0.050127759	0.741414029
ENSG00000146677	*AC004453.1*	-0.26246464	-0.28508034	-0.191308846	-0.093771494	0.999933887	-0.037254892	0.190273202	-0.227528094	0.741566297
ENSG00000264112	*AC015813.1*	2.298772882	-0.226639772	-0.157935101	-0.068704671	0.999933887	-0.357518598	-0.449944128	0.09242553	0.741821772
ENSG00000137807	*KIF23*	-0.889962658	-0.377434065	-0.137964079	-0.239469987	0.999933887	-0.351052136	-0.590041793	0.238989657	0.741821772
ENSG00000104043	*ATP8B4*	2.88925883	-0.113867552	0.029045257	-0.142912809	0.999933887	-0.359840663	-0.437130019	0.077289356	0.741821772
ENSG00000131724	*IL13RA1*	6.576441799	-0.054059699	-0.020119355	-0.033940345	0.999933887	-0.143716817	-0.215613841	0.071897024	0.741821772
ENSG00000168994	*PXDC1*	1.489221915	0.089371891	0.192033449	-0.102661558	0.999933887	0.702587119	0.766121909	-0.06353479	0.741821772
ENSG00000106554	*CHCHD3*	2.30595024	-0.033116844	-0.093621978	0.060505134	0.999933887	0.100596834	0.175332582	-0.074735748	0.741821772
ENSG00000125966	*MMP24*	2.027710322	-0.036800878	0.087952689	-0.124753567	0.999933887	-0.254855239	-0.162010079	-0.09284516	0.741821772
ENSG00000170473	*PYM1*	1.869011555	-0.037116136	0.082724425	-0.119840561	0.999933887	-0.064310563	-0.156756002	0.092445439	0.741821772
ENSG00000144713	*RPL32*	7.621067456	0.017850862	-0.052069651	0.069920513	0.999933887	0.089334909	0.141846764	-0.052511855	0.741821772
ENSG00000165392	*WRN*	1.9400575	0.009795891	-0.270414271	0.280210162	0.999933887	-0.189364272	-0.051595274	-0.137768999	0.741821772
ENSG00000137267	*TUBB2A*	0.941253888	-0.087757756	-0.05722987	-0.030527886	0.999933887	0.210692923	0.339067249	-0.128374326	0.742002149
ENSG00000242612	*DECR2*	0.034999708	-0.114962016	-0.251346775	0.136384759	0.999933887	0.065245653	-0.072090875	0.137336528	0.742002149
ENSG00000112208	*BAG2*	-0.225893123	-0.108146031	-0.047678337	-0.060467695	0.999933887	0.046034385	0.252588525	-0.20655414	0.742002149
ENSG00000170915	*PAQR8*	1.489483871	0.035278913	0.106762371	-0.071483458	0.999933887	0.124229513	0.204471928	-0.080242414	0.742002149
ENSG00000151503	*NCAPD3*	2.719857623	0.021205905	-0.053846034	0.075051939	0.999933887	0.084307312	0.016525114	0.067782198	0.742058807
ENSG00000075336	*TIMM21*	0.540595554	-0.139489254	-0.147252277	0.007763023	0.999933887	0.091341172	-0.029723393	0.121064564	0.742189262
ENSG00000277548	*AC018926.3*	-0.626710839	-0.362782335	0.058258392	-0.421040727	0.999933887	-0.47953083	-0.691857055	0.212326225	0.742427603
ENSG00000245937	*LINC01184*	0.82433201	-0.031424836	-0.065265188	0.033840352	0.999933887	0.05916173	-0.060504609	0.11966634	0.742427603
ENSG00000213020	*ZNF611*	2.934671497	-0.050294265	0.035006799	-0.085301065	0.999933887	-0.340907912	-0.260589313	-0.080318598	0.742463499
ENSG00000099901	*RANBP1*	2.547560948	0.037450089	-0.112042858	0.149492947	0.999933887	0.21689521	0.289069114	-0.072173904	0.742463499
ENSG00000128016	*ZFP36*	11.093066135	0.57665919	0.639322799	-0.062663609	0.999933887	1.327856465	1.430957302	-0.103100837	0.742570344
ENSG00000238829	*RNU7-45P*	-0.643928455	-0.429123634	0.123927195	-0.553050829	0.999933887	1.264812049	1.440183253	-0.175371203	0.742570344
ENSG00000241527	*CA15P1*	-0.503871483	-0.080227751	-0.178869976	0.098642225	0.999933887	-0.461735186	-0.116488107	-0.345247078	0.742570344
ENSG00000163157	*TMOD4*	-0.617774763	0.117869546	-0.342270296	0.460139841	0.999933887	-0.225424786	-0.407784098	0.182359312	0.742570344
ENSG00000175224	*ATG13*	5.976259194	-0.062579172	0.013737396	-0.076316568	0.999933887	-0.212180862	-0.169027028	-0.043153835	0.742676743
ENSG00000144115	*THNSL2*	-0.261351795	0.173289135	-0.165122023	0.338411158	0.999933887	-0.649771218	-0.828060279	0.178289061	0.742676743
ENSG00000130766	*SESN2*	5.298748203	0.037897952	0.002547211	0.035350741	0.999933887	0.293079613	0.243829264	0.049250349	0.742676743
ENSG00000107537	*PHYH*	0.40871803	-0.071716285	-0.028216087	-0.043500198	0.999933887	0.151912213	0.284301109	-0.132388896	0.742676743
ENSG00000247572	*CKMT2-AS1*	-0.549776226	-0.097514464	0.155577179	-0.253091643	0.999933887	-0.04673115	-0.235723592	0.188992442	0.742676743
ENSG00000127399	*LRRC61*	0.134348636	-0.049477023	-0.005487088	-0.043989934	0.999933887	0.761445638	0.923791154	-0.162345517	0.742676743
ENSG00000232517	*CSPG4BP*	-0.408070152	-0.094727905	0.023177054	-0.11790496	0.999933887	5.253065415	5.005113873	0.247951542	0.742676743
ENSG00000217078	*AL021407.1*	-0.297772842	-0.162168978	0.353384723	-0.515553701	0.944094537	0.172117824	0.371720176	-0.199602351	0.742745048
ENSG00000205629	*LCMT1*	2.081406864	-0.105712892	-0.050494775	-0.055218117	0.999933887	-0.077741633	-0.154821725	0.077080091	0.742794446
ENSG00000164050	*PLXNB1*	0.142528895	-0.037329015	0.254307482	-0.291636497	0.999933887	0.011142997	0.175077252	-0.163934254	0.742860869
ENSG00000154447	*SH3RF1*	3.423904783	0.126094181	0.256211147	-0.130116966	0.999933887	-0.747027666	-0.692480162	-0.054547504	0.742897669
ENSG00000259116	*AL049869.2*	0.552968101	-0.172837074	0.110961089	-0.283798163	0.999933887	0.114497654	0.272333157	-0.157835503	0.74328827
ENSG00000065970	*FOXJ2*	5.396418654	-0.013127324	0.047015257	-0.060142581	0.999933887	0.069905665	0.014682633	0.055223032	0.743419962
ENSG00000109775	*UFSP2*	1.442693156	-0.053153417	-0.191274531	0.138121113	0.999933887	0.205435097	0.312158228	-0.106723131	0.743476618
ENSG00000119231	*SENP5*	5.048940064	-0.055591499	-0.001092962	-0.054498537	0.999933887	-0.02585311	0.016242832	-0.042095942	0.743531951
ENSG00000110583	*NAA40*	2.540187066	0.034448862	0.133752348	-0.099303487	0.999933887	0.057030287	-0.018191296	0.075221583	0.743531951
ENSG00000156414	*TDRD9*	2.707471273	0.013597313	-0.056082583	0.069679896	0.999933887	-0.377454716	-0.310226773	-0.067227943	0.743531951
ENSG00000110066	*KMT5B*	5.196933997	-0.081426269	-0.067044053	-0.014382215	0.999933887	-0.644289755	-0.691545699	0.047255944	0.74354432
ENSG00000175793	*SFN*	1.80526915	-0.091384023	0.093961196	-0.18534522	0.999933887	0.42683532	0.543531781	-0.116696461	0.74354432
ENSG00000198380	*GFPT1*	3.466315925	-0.09305174	-0.128926216	0.035874476	0.999933887	-0.508576429	-0.440550344	-0.068026084	0.74354432
ENSG00000128536	*CDHR3*	-0.356744765	0.227447287	-0.104397533	0.33184482	0.999933887	-0.202152709	-0.386684152	0.184531443	0.74354432
ENSG00000103269	*RHBDL1*	0.151265889	0.109915786	-0.207367638	0.317283425	0.999933887	0.074166824	-0.10353147	0.177698295	0.74354432
ENSG00000243317	*STMP1*	3.930504027	-0.033570597	0.021006568	-0.054577165	0.999933887	-0.091697069	-0.148926581	0.057229513	0.74354432
ENSG00000097021	*ACOT7*	0.513296763	0.038165114	0.002746277	0.035418837	0.999933887	0.096652991	-0.049306786	0.145959776	0.74354432
ENSG00000162909	*CAPN2*	5.735446102	0.010880295	-0.018194344	0.029074638	0.999933887	0.11993555	0.155257711	-0.035322161	0.74354432
ENSG00000265907	*AP000919.1*	1.301092168	0.025477691	0.198324166	-0.172846475	0.999933887	-0.883130214	-1.019078267	0.135948053	0.74354432
ENSG00000234571	*H2BP2*	-1.534917906	0.011558776	0.102167985	-0.090609209	0.999933887	-0.542240875	-0.180713055	-0.36152782	0.74354432
ENSG00000114270	*COL7A1*	1.408614288	0.008048065	0.27174284	-0.263694775	0.999933887	0.231336572	0.10148376	0.129852812	0.74354432
ENSG00000171792	*RHNO1*	1.62466459	-0.003139481	0.06709825	-0.070237731	0.999933887	0.131003856	0.036362638	0.094641218	0.74354432
ENSG00000115355	*CCDC88A*	1.986357974	0.004099664	-0.158153107	0.162252771	0.999933887	-0.450593622	-0.33932623	-0.111267392	0.74354432
ENSG00000049768	*FOXP3*	-0.158494607	0.004358046	0.107845143	-0.103487097	0.999933887	0.334895726	0.131639756	0.20325597	0.74354432
ENSG00000213186	*TRIM59*	1.017625464	0.00341474	-0.132292602	0.135707342	0.999933887	-0.060646334	0.073738705	-0.134385039	0.74354432
ENSG00000171843	*MLLT3*	2.458462262	0.046473712	-0.231607283	0.278080996	0.999933887	-0.058770281	0.024142534	-0.082912815	0.743648207
ENSG00000108666	*C17orf75*	0.576853124	-0.045035239	0.005013616	-0.050048854	0.999933887	0.188695829	0.075939998	0.112755831	0.74372312
ENSG00000123219	*CENPK*	0.781308141	-0.151065991	-0.057354301	-0.093711691	0.999933887	-0.020221791	0.124053298	-0.14427509	0.743873609
ENSG00000205981	*DNAJC19*	1.337042563	0.092057345	-0.169067189	0.261124534	0.999933887	0.182177313	0.085247932	0.096929381	0.743873609
ENSG00000173540	*GMPPB*	2.640050929	0.064467175	0.091423426	-0.026956251	0.999933887	0.135795767	0.210077303	-0.074281536	0.743873609
ENSG00000108469	*RECQL5*	3.352533422	0.011360364	0.097121286	-0.085760922	0.999933887	0.210556735	0.274955703	-0.064398968	0.743873609
ENSG00000133812	*SBF2*	5.555234654	-0.007202106	-0.025493227	0.018291121	0.999933887	0.05434452	0.099364025	-0.045019506	0.743873609
ENSG00000278881	*FP325331.1*	-0.188431281	0.115765324	0.056448728	0.059316595	0.999933887	0.152234781	0.310107693	-0.157872913	0.743991405
ENSG00000129657	*SEC14L1*	10.225921101	-0.035869291	0.009056643	-0.044925933	0.999933887	-0.818914179	-0.761157335	-0.057756845	0.743991405
ENSG00000106367	*AP1S1*	1.484073417	0.168042971	-0.152447307	0.320490278	0.999933887	-0.298446495	-0.397795929	0.099349434	0.744018409
ENSG00000179240	*GVQW3*	0.185215003	-0.185347291	0.16734099	-0.352688281	0.999933887	-0.131192164	0.005736232	-0.136928396	0.744124372
ENSG00000175899	*A2M*	2.152309469	0.324933354	0.498872519	-0.173939165	0.999933887	0.020274705	0.207940876	-0.187666171	0.744291981
ENSG00000176410	*DNAJC30*	0.16777027	-0.204809513	0.094191189	-0.299000702	0.999933887	0.246476431	0.104426677	0.142049754	0.744291981
ENSG00000173727	*AP000769.1*	1.543246615	0.167294005	0.170895265	-0.00360126	0.999933887	-0.374677599	-0.264730334	-0.109947264	0.744291981
ENSG00000156304	*SCAF4*	4.767037284	-0.055415194	-0.093793014	0.038377819	0.999933887	-0.227638969	-0.184714502	-0.042924467	0.744291981
ENSG00000136273	*HUS1*	2.926019749	-0.071486912	0.023157872	-0.094644784	0.999933887	0.083638078	0.024822958	0.05881512	0.744291981
ENSG00000111602	*TIMELESS*	1.071365719	0.114343448	0.132136201	-0.017792753	0.999933887	0.055339729	-0.05342614	0.108765869	0.744291981
ENSG00000187815	*ZFP69*	-0.216242417	-0.109537749	-0.068341278	-0.04119647	0.999933887	0.008660474	0.160524296	-0.151863822	0.744291981
ENSG00000171302	*CANT1*	7.09321679	-0.026417229	-0.035714871	0.009297642	0.999933887	-0.269286533	-0.226478501	-0.042808032	0.744291981
ENSG00000188931	*CFAP126*	-0.151477819	0.088966316	0.010810037	0.078156278	0.999933887	-0.16375901	-0.027742611	-0.136016398	0.744291981
ENSG00000111640	*GAPDH*	8.572733602	0.022779705	-0.033769206	0.056548911	0.999933887	-0.14353617	-0.189763184	0.046227014	0.744291981
ENSG00000121940	*CLCC1*	0.852171628	-0.057395009	0.056730789	-0.114125798	0.999933887	-0.026818172	0.086102325	-0.112920497	0.744291981
ENSG00000134545	*KLRC1*	-0.535367498	-0.094927054	0.032785329	-0.127712383	0.999933887	0.177401272	0.356120971	-0.178719699	0.744291981
ENSG00000250644	*AC068580.4*	5.559826822	0.03930389	0.024566122	0.014737768	0.999933887	0.560970527	0.638388044	-0.077417517	0.744291981
ENSG00000245904	*AC025164.1*	0.373979265	-0.027967201	0.188814056	-0.216781257	0.999933887	-0.051992174	0.098781772	-0.150773946	0.744291981

ENSG00000130921	*C12orf65*	2.087865831	0.008296265	0.040159644	-0.03186338	0.999933887	0.215029982	0.290835823	-0.075805841	0.744291981
ENSG00000205758	*CRYZL1*	1.978367545	-0.004448552	0.148234947	-0.152683499	0.999933887	-0.106726454	-0.193851336	0.087124883	0.74506753
ENSG00000278330	*AC018529.2*	6.147322527	0.115277246	0.205122253	-0.089845007	0.999933887	0.2821945	0.353335055	-0.071140555	0.745248299
ENSG00000115282	*TTC31*	3.105134388	-0.012274315	-0.006001103	-0.006273212	0.999933887	0.001394066	-0.049761394	0.05115546	0.745248299
ENSG00000277452	*RN7SL473P*	1.538756126	0.2114225	0.150288199	0.061134301	0.999933887	0.141017443	0.294031023	-0.153013579	0.745350083
ENSG00000260219	*CD2BP2-DT*	0.235185779	0.184745935	-0.079251734	0.263997669	0.999933887	0.085384635	-0.06194516	0.147329795	0.745627002
ENSG00000151445	*VIPAS39*	3.041088203	-0.042691016	-0.128440103	0.085749086	0.999933887	-0.27197253	-0.327366752	0.055394221	0.745666853
ENSG00000018699	*TTC27*	1.027193887	-0.038851475	0.008339977	-0.047191452	0.999933887	0.159283709	0.251522198	-0.092238489	0.745883035
ENSG00000277290	*AC136475.10*	-0.209701029	-0.040649317	-0.26177275	0.221123433	0.999933887	-0.395585273	-0.203349907	-0.192235366	0.74606879
ENSG00000125755	*SYMPK*	4.845108011	0.022534897	0.013224935	0.009309962	0.999933887	0.009768102	-0.028742824	0.038510926	0.746109065
ENSG00000130433	*CACNG6*	0.363971944	-0.062688347	0.287138504	-0.349826851	0.999933887	-0.04204991	0.080384195	-0.122434105	0.746109065
ENSG00000116514	*RNF19B*	9.00848978	0.005233704	0.038437007	-0.033203303	0.999933887	1.007436333	0.944138477	0.063297856	0.746109065
ENSG00000101445	*PPP1R16B*	5.722513972	-0.00227784	-0.042813047	0.040535208	0.999933887	0.109208497	0.163586992	-0.054378495	0.746109065
ENSG00000184178	*SCFD2*	0.462983508	0.06033219	0.058938348	0.001393843	0.999933887	0.122228916	0.004605573	0.117623343	0.746236609
ENSG00000006282	*SPATA20*	1.294396801	-0.020166295	-0.11468113	0.094514834	0.999933887	0.045721821	0.1621412	-0.116419379	0.746325312
ENSG00000285366	*AC008533.1*	2.09839666	-0.099855012	0.252488108	-0.35234312	0.999933887	0.068877543	0.232521632	-0.16364409	0.746637785
ENSG00000287167	*AL133313.1*	-0.75441628	-0.209649408	0.034176904	-0.243826312	0.999933887	0.455791918	0.278009129	0.177782788	0.746637785
ENSG00000205730	*ITPRIPL2*	0.461748153	0.070025473	-0.084711438	0.154736911	0.999933887	-0.333076562	-0.482326651	0.149250089	0.746954713
ENSG00000049246	*PER3*	3.713891511	0.024508295	0.106837105	-0.08232881	0.999933887	-0.261962442	-0.181218587	-0.080743855	0.747286082
ENSG00000197063	*MAFG*	8.442514275	0.087678806	0.02785949	0.059819316	0.999933887	0.562520218	0.611894149	-0.049373931	0.747394125
ENSG00000254750	*CASP1P2*	0.433207425	0.085781998	-0.285829224	0.371611222	0.999933887	0.462388428	0.573271143	-0.110882715	0.747394125
ENSG00000260425	*AL031709.1*	0.618761717	-0.012306488	0.029343915	-0.041650403	0.999933887	0.428223738	0.521560869	-0.093337132	0.747394125
ENSG00000117758	*STX12*	4.488817198	0.003386422	0.036022105	-0.032635683	0.999933887	-0.534197155	-0.478121126	-0.056076029	0.747394125
ENSG00000156504	*FAM122B*	3.567569643	-0.035197343	-0.035786439	0.000589096	0.999933887	-0.144398312	-0.199348023	0.054949711	0.748041494
ENSG00000243650	*RN7SL834P*	1.265699365	-0.029153357	-0.106884518	0.077731161	0.999933887	-0.224871625	-0.390877118	0.166005493	0.748041494
ENSG00000134333	*LDHA*	6.104695381	-0.016295463	-0.061614444	0.045318982	0.999933887	0.086619975	0.135326911	-0.048706935	0.748377214
ENSG00000163814	*CDCP1*	1.113823529	0.065648584	0.006953227	0.058695358	0.999933887	-0.872180152	-0.972087775	0.099907624	0.748447071
ENSG00000183684	*ALYREF*	5.212341625	0.072749287	-0.024048614	0.096797902	0.999933887	0.405388348	0.465834994	-0.060446646	0.748513037
ENSG00000141013	*GAS8*	0.173265453	-0.086901963	0.361928464	-0.448830427	0.981460244	-0.127309223	0.010912842	-0.138222065	0.748513037
ENSG00000187726	*DNAJB13*	-0.398895037	0.029959083	-0.448891399	0.478850482	0.999933887	7.596e-05	-0.239988401	0.24006436	0.748513037
ENSG00000172936	*MYD88*	8.685984933	-0.03076721	-0.064564322	0.033797112	0.999933887	-0.546595996	-0.488062031	-0.058533965	0.748625517
ENSG00000180425	*C11orf71*	1.738890829	0.016364006	-0.01890155	0.035265556	0.999933887	0.429822451	0.33463806	0.095184392	0.748625517
ENSG00000256525	*POLG2*	2.752914603	-0.097614344	0.060829228	-0.158443572	0.999933887	0.067076745	0.006562729	0.060514016	0.748658397
ENSG00000009724	*MASP2*	0.881851892	-0.209978709	-0.045834201	-0.164144508	0.999933887	-0.315352986	-0.20102035	-0.114332636	0.748658397
ENSG00000231412	*AC005392.2*	0.339170485	-0.105073509	-0.016793618	-0.088279892	0.999933887	-0.746632007	-0.885471551	0.138839544	0.748741297
ENSG00000162438	*CTRC*	0.87860752	0.071196098	0.207919014	-0.136722916	0.999933887	0.675255914	0.797468029	-0.122212116	0.748741297
ENSG00000087077	*TRIP6*	1.581025075	0.041321726	0.059930961	-0.018609235	0.999933887	-0.1509075	-0.258623273	0.107715772	0.748741297
ENSG00000006695	*COX10*	0.851101752	0.023097627	0.35714287	-0.334045243	0.999933887	0.061835032	-0.05910348	0.120938512	0.748741297
ENSG00000245522	*LINC02709*	-0.611106655	0.297107796	0.17465663	0.122451166	0.999933887	1.075960289	1.255245708	-0.179285419	0.748778502
ENSG00000115129	*TP53I3*	0.69130485	0.146526347	0.111255295	0.035271052	0.999933887	-0.322243472	-0.177415575	-0.144827897	0.748851587
ENSG00000227615	*AP001324.1*	1.158716422	0.073954213	-0.228732746	0.30268696	0.999933887	0.066751112	-0.028700777	0.095451889	0.748851587
ENSG00000187961	*KLHL17*	2.287600912	0.020669265	0.139975612	-0.119306347	0.999933887	0.004597797	-0.073738258	0.078336055	0.748851587
ENSG00000163141	*BNIPL*	0.000312969	0.071937967	-0.05737214	0.129310107	0.999933887	-0.067232727	-0.208892582	0.141659855	0.74888971
ENSG00000048392	*RRM2B*	5.214423719	-0.114268812	-0.104387294	-0.009881518	0.999933887	-0.972778318	-0.888269503	-0.084508815	0.748995991
ENSG00000197451	*HNRNPAB*	4.139882821	0.064109841	0.06682618	-0.002716339	0.999933887	0.128900125	0.193453604	-0.064553479	0.748995991
ENSG00000257221	*AC007569.1*	0.815328656	0.051705981	-0.186159449	0.237865429	0.999933887	-0.623668518	-0.78083486	0.157166343	0.748995991
ENSG00000104131	*EIF3J*	4.637565772	-0.033975835	-0.04013246	0.006156625	0.999933887	-0.118082641	-0.161880793	0.043798152	0.749103189
ENSG00000160193	*WDR4*	1.087838689	0.058949214	0.296077963	-0.237128749	0.999933887	0.366654144	0.276097322	0.090556822	0.749103189
ENSG00000176340	*COX8A*	3.847228544	0.047290216	-0.043461994	0.090752209	0.999933887	0.131984742	0.189546102	-0.05756136	0.749149498
ENSG00000122026	*RPL21*	7.461704583	-0.036283104	-0.109685904	0.0734028	0.999933887	0.078423609	0.124483964	-0.046060355	0.749365019
ENSG00000213463	*SYNJ2BP*	1.061674473	-0.18106068	-0.302412653	0.121351973	0.999933887	0.002773812	-0.097970581	0.100744392	0.750058533
ENSG00000090565	*RAB11FIP3*	2.990524604	0.112362365	-0.015652317	0.128014682	0.999933887	0.067593639	0.003692928	0.063900711	0.750058533
ENSG00000066230	*SLC9A3*	-0.788803394	0.486260989	0.36772909	0.118531899	0.999933887	0.123680224	0.295909866	-0.172229642	0.750058533
ENSG00000196466	*ZNF799*	-0.589650558	0.174061672	-0.215673942	0.389735614	0.999933887	-0.116347724	-0.342682812	0.226335089	0.750058533
ENSG00000213563	*C8orf82*	1.134590565	0.035831822	0.076966723	-0.0411349	0.999933887	0.005393087	-0.114523656	0.119916743	0.750058533
ENSG00000168275	*COA6*	3.146726029	-0.019516533	-0.100762821	0.081246288	0.999933887	-0.158344316	-0.099949321	-0.058394996	0.750058533
ENSG00000181555	*SETD2*	6.05969792	-0.002999736	-0.127604979	0.124605244	0.999933887	-0.737097349	-0.820038882	0.082941534	0.750058533
ENSG00000214941	*ZSWIM7*	1.79996716	0.198878368	-0.042697983	0.241576351	0.999933887	-0.061575274	-0.143983701	0.082408426	0.750155195
ENSG00000128185	*DGCR6L*	2.627994681	0.075969802	-0.08362966	0.159599462	0.999933887	0.033575317	-0.050991398	0.084566714	0.751016482
ENSG00000128422	*KRT17*	0.658842524	0.08381369	0.278804779	-0.194991089	0.999933887	1.105675561	1.286946494	-0.181270933	0.751038496
ENSG00000177030	*DEAF1*	3.686811285	-0.012967585	-0.062840951	0.049873366	0.999933887	0.075002739	0.12952151	-0.054518771	0.751173533
ENSG00000267481	*AC011477.1*	-0.550525206	0.047359018	0.078643282	-0.031284264	0.999933887	-0.12893628	-0.306551625	0.177615346	0.751522147
ENSG00000087586	*AURKA*	0.323817738	0.030559938	-0.007000402	0.03756034	0.999933887	0.177375745	0.334466754	-0.157091009	0.751870444
ENSG00000223547	*ZNF844*	2.484630836	-0.125819277	-0.098978473	-0.026840804	0.999933887	-0.141777642	-0.069109187	-0.072668455	0.751915127
ENSG00000018510	*AGPS*	2.796746019	-0.058212267	-0.164949676	0.106737409	0.999933887	-0.155137621	-0.089112955	-0.066024665	0.751915127
ENSG00000132680	*KHDC4*	4.624239623	-0.051505053	0.014461476	-0.065966528	0.999933887	0.015552129	-0.030534163	0.046086293	0.75198023
ENSG00000259238	*AC092755.2*	0.632786164	0.220910562	0.354609354	-0.133698792	0.999933887	0.579083323	0.752700833	-0.173617509	0.752051015
ENSG00000137692	*DCUN1D5*	1.997854246	0.077366106	-0.134856893	0.212222999	0.999933887	-0.000848398	0.075143138	-0.075991536	0.752051015
ENSG00000110031	*LPXN*	4.929260296	-0.043727263	-0.082972394	0.039245131	0.999933887	0.344959178	0.298840753	0.046118425	0.752051015
ENSG00000165025	*SYK*	7.330498164	-0.027804136	-0.03288273	0.005078594	0.999933887	0.471155694	0.541326474	-0.07017078	0.752051015
ENSG00000059122	*FLYWCH1*	3.941513455	0.103133813	0.027859174	0.075274639	0.999933887	0.041458289	0.102290756	-0.060832467	0.752398239
ENSG00000185019	*UBOX5*	3.146580535	-0.084046965	-0.009738313	-0.074308653	0.999933887	-0.138658971	-0.077978437	-0.060680534	0.752398239
ENSG00000146535	*GNA12*	3.295267324	-0.064671252	0.041313684	-0.105984936	0.999933887	0.000385274	0.061770659	-0.061385385	0.752398239
ENSG00000097046	*CDC7*	1.07704031	0.098190616	-0.227790748	0.325981364	0.999933887	0.140300686	0.241842142	-0.101541456	0.752398239
ENSG00000224376	*AC017104.1*	-1.34789945	0.298945962	0.316827593	-0.017881632	0.999933887	1.953212097	2.202688329	-0.249476232	0.752398239
ENSG00000169718	*DUS1L*	4.514198929	0.043635203	0.103886261	-0.060251057	0.999933887	0.151479097	0.097877984	0.053601113	0.752398239
ENSG00000283602	*AC116353.6*	1.669123143	-0.067652096	-0.209996695	0.142344598	0.999933887	-0.74798318	-0.647565178	-0.100418002	0.752398239
ENSG00000255423	*EBLN2*	3.346533619	0.030850193	0.083532215	-0.052682022	0.999933887	0.315525317	0.421764386	-0.106239069	0.752398239
ENSG00000170634	*ACYP2*	1.559467922	-0.028400369	0.116153292	-0.144553661	0.999933887	0.252239697	0.153356762	0.098882935	0.752398239
ENSG00000259070	*LINC00639*	0.173463629	-0.04751856	0.100561828	-0.148080387	0.999933887	-0.491756593	-0.265313641	-0.226442952	0.752398239
ENSG00000239883	*PARGP1*	0.81932471	0.175283751	-0.106966811	0.282250562	0.999933887	0.020612028	0.155853782	-0.135241754	0.752457115
ENSG00000106868	*SUSD1*	3.219550343	-0.005567416	-0.076511474	0.070944058	0.999933887	-0.004926458	0.054128345	-0.059054803	0.752457115
ENSG00000176396	*EID2*	1.355957949	0.173546267	0.019993865	0.153552403	0.999933887	-0.086030006	0.024921934	-0.11095194	0.752605804
ENSG00000153162	*BMP6*	4.889595916	0.073226001	0.026565532	0.046660469	0.999933887	-0.85078288	-0.782336395	-0.068446485	0.752605804
ENSG00000185869	*ZNF829*	-0.31410273	-0.118140768	-0.093376685	-0.024764083	0.999933887	-0.1310059	0.045326591	-0.17633249	0.752605804
ENSG00000196639	*HRH1*	-0.454877821	-0.036346111	-0.116079561	0.07973345	0.999933887	0.895342648	1.060225985	-0.164883337	0.752605804
ENSG00000120029	*ARMH3*	2.84607615	-0.009651714	0.0255945	-0.035246215	0.999933887	-0.026095909	-0.094485203	0.068389294	0.752605804
ENSG00000047579	*DTNBP1*	5.626031365	0.002194708	0.055872503	-0.053677795	0.999933887	-0.109628786	-0.064381056	-0.045247729	0.752605804
ENSG00000198815	*FOXJ3*	5.488593638	-0.055079734	0.009612373	-0.064692107	0.999933887	-0.198722657	-0.236964658	0.038242	0.752966808
ENSG00000155158	*TTC39B*	2.818907661	0.006992188	-0.045169745	0.052161933	0.999933887	-7.3628e-05	0.071436574	-0.071510203	0.752966808
ENSG00000182134	*TDRKH*	0.238564808	0.044476055	-0.06633838	0.110814436	0.999933887	0.286145305	0.436382912	-0.150237607	0.75307934
ENSG00000157837	*SPPL3*	5.649552909	-0.049298686	0.06043896	-0.109737645	0.999933887	-0.302231604	-0.263431848	-0.038799755	0.753204307
ENSG00000254177	*AC079209.2*	0.310309446	0.072624202	0.2593402	-0.186715997	0.999933887	0.375153818	0.588268963	-0.213115145	0.753555378
ENSG00000159214	*CCDC24*	1.00479313	0.085651465	0.08845729	-0.002805826	0.999933887	0.311074339	0.413765003	-0.102690664	0.753838181
ENSG00000104872	*PIH1D1*	3.485948467	-0.034214282	0.002167926	-0.036382207	0.999933887	0.106611972	0.049970928	0.056641043	0.753838181
ENSG00000166037	*CEP57*	3.995854557	-0.05262108	-0.104602632	0.051981552	0.999933887	-0.142239585	-0.048654003	-0.093585582	0.753838181
ENSG00000139193	*CD27*	2.211916832	0.006382555	-0.046998865	0.05338142	0.999933887	0.070079416	0.140346843	-0.070267427	0.753838181
ENSG00000165195	*PIGA*	4.232247172	-0.001248275	-0.040138502	0.038890227	0.999933887	-0.01717343	-0.090643258	0.073469827	0.753838181
ENSG00000166479	*TMX3*	3.407703873	-0.060856381	-0.091981182	0.031124801	0.999933887	-0.35223371	-0.23314011	-0.1190936	0.753934576
ENSG00000167578	*RAB4B*	0.902940887	-0.006100328	-0.083398492	0.077298164	0.999933887	-0.045442377	0.056435348	-0.101877725	0.753958311
ENSG00000176845	*METRNL*	7.980851901	0.109438907	0.14333205	-0.033893142	0.999933887	0.009633982	0.071830883	-0.062196901	0.754035004
ENSG00000141646	*SMAD4*	4.920542241	-0.06813297	-0.010266083	-0.057866887	0.999933887	-0.567858944	-0.625140084	0.05728114	0.754401243
ENSG00000151490	*PTPRO*	-1.033446106	0.131292955	-0.017401605	0.14869456	0.999933887	0.017319173	-0.164218731	0.181537904	0.754829757
ENSG00000127993	*RBM48*	3.345079033	-0.052366334	-0.000543551	-0.051822783	0.999933887	-0.125217033	-0.073163902	-0.05205313	0.754944022
ENSG00000129103	*SUMF2*	3.555246863	-0.018578438	-0.02151204	0.002933602	0.999933887	0.148129448	0.09056191	0.057567538	0.75498522

ENSG00000245146	*MALINC1*	-0.200051172	0.240041829	-0.020412488	0.260454317	0.999933887	-0.289107627	-0.145399784	-0.143707844	0.755028372
ENSG00000099994	*SUSD2*	0.157579036	-0.02231509	0.249567381	-0.27188247	0.999933887	-0.055530899	0.116927712	-0.172458611	0.755458163
ENSG00000164221	*CCDC112*	0.912865974	-0.196296258	-0.094494524	-0.101801734	0.999933887	-0.300510709	-0.411087204	0.110576494	0.755480626
ENSG00000261693	*AC134682.1*	0.059628105	0.154263047	0.029094873	0.125168174	0.999933887	1.234773319	1.393662658	-0.158889339	0.755480626
ENSG00000137076	*TLN1*	9.684438696	0.004133129	0.01862498	-0.014491851	0.999933887	-0.234654995	-0.276049418	0.041394423	0.755480626
ENSG00000074266	*EED*	2.590040875	-0.07175584	-0.150361955	0.078606115	0.999933887	0.182670096	0.246073258	-0.063403163	0.755545306
ENSG00000181929	*PRKAG1*	4.520159554	-0.029977585	-0.073526244	0.04354866	0.999933887	-0.163597247	-0.120918279	-0.042678968	0.755576227
ENSG00000249476	*AC008467.1*	0.399932716	0.053205149	0.041637504	0.011567645	0.999933887	-0.046329743	0.077492724	-0.123822467	0.75567841
ENSG00000153485	*TMEM251*	3.99788103	0.045029323	-0.140231561	0.185260884	0.999933887	0.6800472	0.751562139	-0.07151494	0.75567841
ENSG00000254441	*EIF2S2P6*	-0.828768735	0.065841266	0.104836543	-0.038995277	0.999933887	-0.125500842	0.212821239	-0.338322081	0.75567841
ENSG00000123178	*SPRYD7*	-0.238284684	-0.145556226	0.089922689	-0.235478916	0.999933887	-0.025965612	0.126961824	-0.152927436	0.755946325
ENSG00000185955	*C7orf61*	0.70003742	0.101177669	-0.139684356	0.240862025	0.999933887	0.218774789	0.121701939	0.097072849	0.756076379
ENSG00000277349	*CICP25*	-1.993275889	-0.040189292	-0.007392056	-0.032797236	0.999933887	0.179502183	-0.062993391	0.242495573	0.756076379
ENSG00000245105	*A2M-AS1*	-0.270685861	0.302368351	0.569411878	-0.267043528	0.999933887	0.07594695	0.302172432	-0.226225481	0.756331325
ENSG00000062822	*POLD1*	2.265180134	0.080350565	0.08008531	0.000265256	0.999933887	0.135134164	0.052521476	0.082612688	0.756331325
ENSG00000214021	*TTLL3*	4.953876639	-0.023620284	0.026452047	-0.050072331	0.999933887	-0.030067572	0.009501734	-0.039569305	0.756331325
ENSG00000276853	*AC026124.2*	0.400317502	0.071400842	-0.178783602	0.250184444	0.999933887	0.426474753	0.286832468	0.139642285	0.756331325
ENSG00000197798	*FAM118B*	1.32788154	0.034496181	-0.226785845	0.261282025	0.999933887	-0.096687954	-0.008974196	-0.087713757	0.756331325
ENSG00000167851	*CD300A*	9.324930607	0.013037062	-0.003612511	0.016649573	0.999933887	-0.639668874	-0.697279396	0.057610521	0.756331325
ENSG00000101079	*NDRG3*	3.047040658	-0.003508938	-0.153150731	0.149641793	0.999933887	-0.135889415	-0.051466074	-0.084423341	0.756331325
ENSG00000236567	*TCF3P1*	-0.033502568	0.212825485	0.18053635	0.032289135	0.999933887	0.53024009	0.842480666	-0.312240576	0.756474969
ENSG00000115526	*CHST10*	-0.461059564	0.22583587	0.04028189	0.18555398	0.999933887	0.315663948	0.12926879	0.186395158	0.756657532
ENSG00000147996	*CBWD5*	1.135803731	-0.113934961	0.114316384	-0.228251346	0.999933887	-0.092846644	-0.238592204	0.14574556	0.756657532
ENSG00000138111	*MFSD13A*	2.586359486	-0.054122026	0.012723218	-0.066845244	0.999933887	-0.613254111	-0.718777818	0.105523707	0.756657532
ENSG00000126217	*MCF2L*	0.383620388	0.007053757	-0.067549783	0.07460354	0.999933887	0.209657318	0.33989888	-0.130241561	0.756657532
ENSG00000063046	*EIF4B*	7.419440196	-0.060561936	-0.065830782	0.005268846	0.999933887	0.026685456	0.062898841	-0.036213385	0.75704849
ENSG00000166913	*YWHAB*	7.370567926	-0.064126368	-0.100511289	0.036384921	0.999933887	-0.142400953	-0.179067658	0.036666705	0.757104842
ENSG00000126970	*ZC4H2*	-0.752581462	0.214162112	0.109962579	0.104199533	0.999933887	-0.080924262	0.135308272	-0.216232534	0.757129639
ENSG00000272669	*AL021707.6*	3.971495916	0.031680042	0.07588904	-0.044208997	0.999933887	0.14730705	0.07963904	0.06766801	0.757410555
ENSG00000188917	*TRMT2B*	2.96554986	0.033655884	-0.062922408	0.096578293	0.999933887	-0.12805897	-0.190539964	0.062480994	0.757470306
ENSG00000204519	*ZNF551*	1.178127601	-0.057514288	0.069085042	-0.12659933	0.999933887	0.204254089	0.102238872	0.102015217	0.757810654
ENSG00000206561	*COLQ*	-0.022425909	-0.069856879	-0.091863937	0.022007059	0.999933887	-0.06956951	0.096352665	-0.165922175	0.758401889
ENSG00000140678	*ITGAX*	11.557722995	0.072982687	0.076032655	-0.003049969	0.999933887	-0.059095247	-0.020636652	-0.038458595	0.758464034
ENSG00000236779	*AL512306.1*	-0.005599181	0.084715445	0.051507001	0.033208443	0.999933887	0.155079131	-0.006421339	0.16150047	0.758503008
ENSG00000178974	*FBXO34*	5.064467516	-0.103959953	-0.077772178	-0.026187774	0.999933887	-0.360986716	-0.408776947	0.047790231	0.758864437
ENSG00000258820	*AF111167.1*	-0.199048644	0.248368142	0.079473798	0.168894344	0.999933887	0.578974978	0.352110786	0.226864192	0.758864437
ENSG00000230730	*AC074011.1*	-0.885756324	0.150032523	0.147069269	0.002963255	0.999933887	0.72047375	0.458416178	0.262057572	0.758864437
ENSG00000286488	*AC103858.3*	1.283290537	-0.0838919	-0.075701583	-0.008190317	0.999933887	0.602324918	0.718387537	-0.116062619	0.758864437
ENSG00000235290	*HLA-W*	0.980291828	-0.052370172	0.201518059	-0.253888231	0.999933887	0.252569658	0.116733245	0.135836412	0.758864437
ENSG00000278022	*AC118658.1*	0.092938332	0.000799378	0.025177215	-0.024377837	0.999933887	0.01274316	0.143769299	-0.131026138	0.758864437
ENSG00000092531	*SNAP23*	4.524439737	0.000536917	-0.087153967	0.087690883	0.999933887	0.209550993	0.1467491	0.062801893	0.759143666
ENSG00000115687	*PASK*	3.690306413	0.032616489	0.074396328	-0.041779839	0.999933887	0.20296876	0.248766352	-0.045797592	0.75919366
ENSG00000116990	*MYCL*	0.422842061	-0.471911965	-0.26490693	-0.207005035	0.999933887	-0.834793828	-0.951294058	0.116500229	0.759530027
ENSG00000068878	*PSME4*	6.075219255	-0.066776263	-0.058190909	-0.008585354	0.999933887	-0.146049339	-0.103190555	-0.042858784	0.759530027
ENSG00000179598	*PLD6*	3.958381104	0.08376443	0.159703387	-0.075938957	0.999933887	-0.270056052	-0.34405401	0.073997958	0.759530027
ENSG00000155008	*APOOL*	0.082416781	-0.065029561	-0.08708992	0.022060359	0.999933887	-0.048259552	0.111384725	-0.159644277	0.759530027
ENSG00000188428	*BLOC1S5*	1.051245103	-0.195664967	-0.042426594	-0.153238373	0.999933887	-0.001786779	-0.091145088	0.089358309	0.759772778
ENSG00000184271	*POU6F1*	2.281413058	-0.03689385	-0.088866659	0.051972809	0.999933887	0.146176238	0.215400146	-0.069223908	0.759772778
ENSG00000232470	*AL136368.1*	0.850277976	0.059949803	0.054879688	0.005070115	0.999933887	0.258099956	0.376771984	-0.118672028	0.759811222
ENSG00000175166	*PSMD2*	6.095602356	-0.01543009	0.003990601	-0.019420691	0.999933887	-0.079315116	-0.113551319	0.034236203	0.759811222
ENSG00000128383	*APOBEC3A*	7.635531097	-0.004843198	0.018003617	-0.022846815	0.999933887	-0.116279758	-0.159844205	0.043564446	0.760449504
ENSG00000053770	*AP5M1*	3.559357585	-0.122439555	-0.099246822	-0.023192732	0.999933887	-0.360391775	-0.302811309	-0.057580467	0.760595002
ENSG00000177613	*CSTF2T*	3.467253773	-0.045385128	0.026741413	-0.072126541	0.999933887	0.028583165	-0.02983155	0.058414715	0.760595002
ENSG00000139239	*RPL14P1*	-0.138517695	0.082707536	0.119629559	-0.036922022	0.999933887	0.052949835	0.199287938	-0.146338103	0.760595002
ENSG00000287697	*Z99127.3*	0.319738987	0.086428315	0.051256572	0.035171743	0.999933887	0.771488964	0.620921425	0.15056754	0.760595002
ENSG00000040487	*SLC66A1*	4.088546081	-0.031459358	0.015547037	-0.047006395	0.999933887	-0.512420124	-0.57001841	0.057598286	0.760595002
ENSG00000170464	*DNAJC18*	0.520529068	-0.044166483	-0.023670135	-0.020496348	0.999933887	0.136615425	-0.015317426	0.15193285	0.760595002
ENSG00000130165	*ELOF1*	3.638282168	-0.063172126	0.030858402	-0.094030528	0.999933887	-0.235003197	-0.29376506	0.058761863	0.760718806
ENSG00000104177	*MYEF2*	0.78966472	-0.065776189	-0.297755182	0.231978993	0.999933887	-0.099191306	0.049572048	-0.148763354	0.760729757
ENSG00000150403	*TMCO3*	6.654091783	-0.10364191	-0.109613195	0.005971285	0.999933887	-0.91851216	-0.98160571	0.06309355	0.760842095
ENSG00000107938	*EDRF1*	3.399260816	0.000364752	-0.040202974	0.040567727	0.999933887	-0.289033852	-0.356127648	0.067093797	0.760867608
ENSG00000280206	*AC026401.3*	3.328052283	0.097712896	0.096359481	0.001353415	0.999933887	0.680210988	0.753996538	-0.073785551	0.761040268
ENSG00000204120	*GIGYF2*	5.404852749	-0.021348133	-0.059875506	0.038527373	0.999933887	-0.327232385	-0.287193618	-0.040038767	0.761067819
ENSG00000259972	*AC009120.2*	1.548538784	-0.138175634	0.093425405	-0.231601039	0.999933887	-0.071533441	-0.162584625	0.091051183	0.761234933
ENSG00000119900	*OGFRL1*	6.6489097	-0.011106764	-0.042267985	0.031161221	0.999933887	-0.004384052	-0.093422964	0.089038913	0.761234933
ENSG00000218313	*AL139274.1*	-0.012174295	-0.038963063	0.386232067	-0.42519513	0.999933887	0.303585118	0.546283598	-0.24269848	0.761243888
ENSG00000141295	*SCRN2*	2.381905086	0.042211433	0.08007544	-0.037864008	0.999933887	-0.00392573	-0.091187718	0.087261988	0.761417303
ENSG00000232615	*AC026412.2*	-1.361627934	-0.032063571	0.096912886	-0.128976457	0.999933887	0.260476036	0.526347255	-0.26587122	0.761558439
ENSG00000226266	*AC009961.1*	-0.006314481	0.035596793	0.131588605	-0.095991812	0.999933887	-0.610859316	-0.426126385	-0.184732931	0.761643874
ENSG00000007402	*CACNA2D2*	1.778916996	0.063678923	-0.019811928	0.083490851	0.999933887	0.077603267	-0.009488253	0.087091521	0.761931891
ENSG00000161692	*DBF4B*	0.739529361	-0.061750791	0.022604082	-0.084354873	0.999933887	-0.114793913	0.004796259	-0.119590172	0.761931891
ENSG00000178950	*GAK*	7.953311609	0.040582595	0.006164947	0.034417648	0.999933887	0.215155511	0.174356135	0.040799376	0.761982151
ENSG00000171132	*PRKCE*	2.868718248	0.003861731	0.152672025	-0.148810294	0.999933887	0.14469016	0.077528346	0.067161813	0.761982151
ENSG00000105486	*LIG1*	2.950047825	0.014699094	0.074843667	-0.060144574	0.999933887	0.096141323	0.16077337	-0.064632047	0.762007218
ENSG00000285106	*AC016831.6*	4.716345387	0.0078415	0.107281954	-0.099440454	0.999933887	0.690083688	0.757366021	-0.067282333	0.762007218
ENSG00000167900	*TK1*	0.255780713	0.204720594	0.130314006	0.074406589	0.999933887	0.739090135	0.878085931	-0.138995795	0.762094352
ENSG00000272602	*ZNF595*	-0.640047185	-0.217218369	-0.134618505	-0.082599864	0.999933887	-0.344849652	-0.161688598	-0.183161054	0.762101038
ENSG00000101425	*BPI*	0.245250266	-0.161323862	0.241752542	-0.403076403	0.999933887	-0.015053469	0.123781902	-0.138835371	0.762127896
ENSG00000101751	*POLI*	2.33183107	0.014301486	-0.042030299	0.056331785	0.999933887	-0.319481846	-0.213587709	-0.105894137	0.762127896
ENSG00000197324	*LRP10*	9.363062647	0.026585917	-0.000227468	0.026813386	0.999933887	-0.104830817	-0.149620184	0.044789367	0.762309362
ENSG00000160688	*FLAD1*	2.802300282	0.02810211	-0.079034097	0.107136207	0.999933887	-0.012409135	-0.066891407	0.054482271	0.762433639
ENSG00000178096	*BOLA1*	0.282660428	0.168720672	0.248635401	-0.079914729	0.999933887	0.173244748	0.063118282	0.110126467	0.762692044
ENSG00000225079	*FTH1P22*	-0.633165398	-0.085862889	0.556828132	-0.642691021	0.999933887	-0.089599289	0.113938043	-0.203537332	0.762907579
ENSG00000117569	*PTBP2*	3.130038025	-0.015687031	-0.055698065	0.040011034	0.999933887	-0.11767048	-0.030553469	-0.08711701	0.763253123
ENSG00000149313	*AASDHPPT*	2.135205679	-0.162998698	-0.027082704	-0.135915993	0.999933887	-0.038641533	-0.123448068	0.084806536	0.763356663
ENSG00000184208	*C22orf46*	2.283460372	0.035114655	0.055510835	-0.02039618	0.999933887	0.115586475	0.195973733	-0.080387258	0.763356663
ENSG00000139620	*KANSL2*	3.70470429	-0.034801307	-0.079973596	0.045172289	0.999933887	0.071924233	0.017274525	0.054649708	0.763399227
ENSG00000143337	*TOR1AIP1*	6.653236407	-0.051053354	-0.047563354	-0.00349	0.999933887	-0.17961278	-0.224143021	0.044530241	0.763558653
ENSG00000213397	*HAUS7*	-0.639063774	-0.252659112	0.00848253	-0.261141643	0.999933887	-0.061487568	-0.270771448	0.209283881	0.763589223
ENSG00000141294	*LRRC46*	-0.264141541	-0.094944388	-0.207095988	0.1121516	0.999933887	0.035802647	-0.158532642	0.194335289	0.763589223
ENSG00000221957	*KIR2DS4*	-0.796632257	0.105700147	0.223025034	-0.117324887	0.999933887	0.965505564	1.145680735	-0.180175171	0.763589223
ENSG00000273015	*AC008124.1*	2.670351762	0.014082723	0.059750471	-0.045667748	0.999933887	0.072950827	0.132651468	-0.059700641	0.763589223
ENSG00000185201	*IFITM2*	11.142784142	0.056212202	0.008782528	0.047429674	0.999933887	-0.019516534	-0.066092257	0.046575723	0.763826609
ENSG00000178947	*SMIM10L2A*	-0.952612666	-0.157580049	-0.00208167	-0.155498379	0.999933887	0.006148046	0.171182829	-0.165034783	0.763826711
ENSG00000158445	*KCNB1*	-0.324953821	-0.050535359	0.242537787	-0.293073145	0.999933887	-0.249684487	-0.422037091	0.172352605	0.763826711
ENSG00000151729	*SLC25A4*	0.281619309	-0.050973622	0.026607624	-0.077581246	0.999933887	0.065890396	0.190710284	-0.124819888	0.76395606
ENSG00000233922	*LINC01694*	-0.825996268	0.019205186	0.173700506	-0.154495319	0.999933887	1.112088556	1.292085259	-0.179996703	0.76395606
ENSG00000164402	*SEPTIN8*	1.121516029	0.006476807	0.144333114	-0.137856307	0.999933887	0.201982774	0.115648928	0.086333846	0.76395606
ENSG00000211747	*TRBV20-1*	1.989914248	-0.002423202	-0.198423661	0.196000458	0.999933887	0.239803568	0.164181316	0.075622251	0.76395606
ENSG00000086475	*SEPHS1*	1.964669473	-0.02647897	0.028853904	-0.055332873	0.999933887	0.015051435	0.099131941	-0.084080506	0.763981502
ENSG00000112079	*STK38*	6.076524604	-0.0342539	-0.061495114	0.027241214	0.999933887	-0.027759278	-0.066284643	0.038525366	0.764177051
ENSG00000118785	*SPP1*	2.701361744	0.027533046	-0.132020727	0.159553774	0.999933887	-0.659053292	-0.773540567	0.114487275	0.764209244
ENSG00000183458	*PKD1P3*	1.03649158	0.128049705	0.275326816	-0.147277111	0.999933887	-0.108018717	-0.011463451	-0.096555266	0.764246591
ENSG00000223704	*LINC01422*	-0.722247368	-0.117498334	-0.134563091	0.017064756	0.999933887	0.133172699	0.315455113	-0.182282415	0.764246591
ENSG00000196388	*INCA1*	-0.717243654	-0.325961229	-0.083722987	-0.242238241	0.999933887	0.22444255	0.416031664	-0.191589114	0.764724314

ENSG00000005238	*FAM214B*	6.469031167	0.035565265	0.081853187	-0.046287922	0.999933887	-0.502231632	-0.451438282	-0.05079335	0.76490757
ENSG00000122557	*HERPUD2*	5.964479799	0.057903892	0.014068183	0.043835709	0.999933887	-0.320425194	-0.360258315	0.039833121	0.764940704
ENSG00000159423	*ALDH4A1*	1.84859125	0.010067901	0.046678757	-0.036610857	0.999933887	0.64804363	0.566563832	0.081479798	0.764940704
ENSG00000174721	*FGFBP3*	-0.85410843	0.27366428	0.198651632	0.075012648	0.999933887	0.017231017	-0.181165603	0.19839662	0.765060293
ENSG00000196275	*GTF2IRD2*	0.209478379	-0.069543524	0.000715342	-0.070258866	0.999933887	0.073029223	0.214701742	-0.141672519	0.765060293
ENSG00000101084	*RAB5IF*	4.515220607	0.136802719	0.038193738	0.09860898	0.999933887	-0.099874248	-0.170071618	0.07019737	0.765119766
ENSG00000243466	*IGKV1-5*	-0.926305665	0.084098758	-0.516571561	0.600670319	0.999933887	0.217309121	0.495221232	-0.277912111	0.765119766
ENSG00000266852	*MIR4482*	-0.047739305	0.006845991	0.152840491	-0.1459945	0.999933887	0.774140465	0.634216111	0.139924355	0.765119766
ENSG00000079950	*STX7*	6.426695413	-0.081875123	-0.032927354	-0.048947769	0.999933887	-0.983522443	-1.039230886	0.055708443	0.765533917
ENSG00000233029	*AC244453.2*	1.114360147	0.092291499	-0.007285866	0.099577364	0.999933887	0.119462914	0.271948855	-0.15248594	0.765686682
ENSG00000197872	*CYRIA*	5.889865031	-0.023512457	-0.033687676	0.010175219	0.999933887	0.194491181	0.12827493	0.066216251	0.765686682
ENSG00000104918	*RETN*	-0.951445285	0.160409222	0.112203027	0.048206195	0.999933887	-0.2015754	0.012849889	-0.214425289	0.7658691
ENSG00000123444	*KBTBD4*	1.824313129	-0.164608622	-0.194092648	0.029484026	0.999933887	-0.106381994	-0.024678137	-0.081703858	0.766089155
ENSG00000244267	*RPL34P22*	-0.909130022	-0.038261208	0.280698405	-0.318959612	0.999933887	1.453508826	1.783873232	-0.330364406	0.766294292
ENSG00000145041	*DCAF1*	3.90288842	-0.009817311	-0.010130192	0.000312882	0.999933887	0.055164785	0.105641327	-0.050476542	0.766567572
ENSG00000285437	*POLR2J3*	1.160127516	-0.034808243	0.07273643	-0.107544673	0.999933887	0.049246186	-0.047063827	0.096310013	0.766595554
ENSG00000019485	*PRDM11*	1.501008308	0.059985648	0.003252371	0.056733277	0.999933887	0.114017154	0.010677452	0.103339702	0.767028515
ENSG00000074657	*ZNF532*	0.27202046	-0.096713243	-0.034393553	-0.062319689	0.999933887	0.093012224	0.218198657	-0.125186433	0.767038139
ENSG00000145216	*FIP1L1*	3.107581869	-0.027734507	-0.029382883	0.001648375	0.999933887	-0.467569709	-0.415871913	-0.051697796	0.767038139
ENSG00000284707	*AC079781.5*	0.007507056	-0.03638379	0.088843807	-0.125227598	0.999933887	0.071654562	0.20947019	-0.137815628	0.767038139
ENSG00000151353	*TMEM18*	2.55094211	-0.008353014	-0.044267614	0.0359146	0.999933887	-0.038096932	-0.094480476	0.056383543	0.767038139
ENSG00000139725	*RHOF*	2.958752054	0.014212386	-0.153870624	0.16808301	0.999933887	1.412314728	1.500711422	-0.088396694	0.767038139
ENSG00000181963	*OR52K2*	0.368704375	0.115104994	-0.030569861	0.145674855	0.999933887	0.017940255	0.150647285	-0.132707029	0.767646431
ENSG00000138623	*SEMA7A*	4.839279073	-0.041344302	0.047211715	-0.088556017	0.999933887	0.195578819	0.257242816	-0.061663997	0.767646431
ENSG00000117054	*ACADM*	1.310677229	-0.081067989	0.01392926	-0.094997249	0.999933887	-0.162271247	-0.068275609	-0.093995638	0.767646431
ENSG00000267265	*AC011476.3*	0.131402875	0.101809175	0.000743431	0.101065744	0.999933887	0.520454754	0.643120202	-0.122665447	0.767646431
ENSG00000065268	*WDR18*	1.886554021	0.046849134	0.192160644	-0.14531151	0.999933887	0.27011373	0.353896454	-0.083782724	0.767646431
ENSG00000223804	*AC244669.1*	0.665468129	-0.030766666	-0.310455668	0.279689003	0.999933887	0.32203844	0.444196632	-0.122158192	0.767646431
ENSG00000261556	*SMG1P7*	0.293470314	-0.123199514	0.104446487	-0.227646	0.999933887	0.127655927	0.01323889	0.114417036	0.767750384
ENSG00000135457	*TFCP2*	3.436246067	-0.022876164	0.057336817	-0.080212981	0.999933887	-0.10159815	-0.149434803	0.047836653	0.767750384
ENSG00000188011	*RTP5*	0.372157308	-0.077888227	-0.128914387	0.05102616	0.999933887	0.034242476	0.16768174	-0.133439264	0.76792862
ENSG00000179151	*EDC3*	2.497563599	0.027987442	-0.003716699	0.031704141	0.999933887	0.049332092	0.10979834	-0.060466249	0.76792862
ENSG00000262962	*KARS1P3*	-0.527112949	-0.017666695	-0.335338234	0.317671539	0.999933887	-1.540826536	-1.746666216	0.20583968	0.76792862
ENSG00000251393	*AC005280.1*	1.935429942	0.110240152	0.099274874	0.010965278	0.999933887	1.60684361	1.703861189	-0.097017579	0.768131736
ENSG00000007923	*DNAJC11*	1.911143401	-0.098705523	-0.048884622	-0.049820901	0.999933887	0.041984563	0.120909664	-0.078925102	0.768399916
ENSG00000273802	*H2BC8*	0.55865804	-0.050234756	0.106250471	-0.156485227	0.999933887	-0.339010177	-0.477228052	0.138217875	0.768399916
ENSG00000196230	*TUBB*	5.523953875	-0.013074101	-0.050559698	0.037485597	0.999933887	0.295443373	0.341253842	-0.045810469	0.768399916
ENSG00000135362	*PRR5L*	5.434234991	-0.051467863	0.034549074	-0.086016937	0.999933887	0.038704506	-0.009137069	0.047841575	0.768432203
ENSG00000271270	*TMCC1-AS1*	-0.397558926	-0.119126812	0.085672162	-0.204798974	0.999933887	-0.639841239	-0.503686009	-0.13615523	0.768561749
ENSG00000163629	*PTPN13*	0.086656459	-0.242264558	-0.112948981	-0.129315578	0.999933887	-0.039954941	0.159699692	-0.199654633	0.768581498
ENSG00000100601	*ALKBH1*	1.618342411	-0.114769381	-0.011112969	-0.103656412	0.999933887	-0.075940356	0.002130635	-0.078070991	0.768727503
ENSG00000093072	*ADA2*	3.952111421	-0.041086793	-0.012851689	-0.028235104	0.999933887	-0.037533762	-0.084274626	0.046740864	0.768921362
ENSG00000260105	*AOC4P*	-0.777612631	0.120705346	0.108769763	0.011935584	0.999933887	1.07443053	0.832857588	0.241572943	0.769075238
ENSG00000185880	*TRIM69*	5.848830845	0.042865348	0.036027077	0.006838271	0.999933887	0.238359513	0.281446294	-0.04308678	0.769446972
ENSG00000180011	*ZADH2*	5.172631584	-0.121205466	-0.048526667	-0.072678799	0.999933887	-0.706052874	-0.761267978	0.055215103	0.769702827
ENSG00000258086	*AC079313.1*	0.787433129	-0.153853948	-0.080085071	-0.073768878	0.999933887	-0.151692482	-0.262216371	0.110523889	0.769702827
ENSG00000160883	*HK3*	5.993327473	0.058024601	0.084501148	-0.026476547	0.999933887	0.099773188	0.045757239	0.054015948	0.769702827
ENSG00000080573	*COL5A3*	1.111301755	0.081332415	0.084366324	-0.003033909	0.999933887	-0.095375228	-0.224591145	0.129215918	0.769702827
ENSG00000103199	*ZNF500*	1.07955977	-0.041123448	0.077856259	-0.118979707	0.999933887	0.113558045	0.205295847	-0.091737803	0.769702827
ENSG00000008086	*CDKL5*	3.319089535	0.016924991	-0.074492352	0.091417343	0.999933887	-0.332350252	-0.401668435	0.069318183	0.769702827
ENSG00000280194	*AD000864.1*	2.987513141	0.316205196	0.20499619	0.111209006	0.999933887	1.750922654	1.855130675	-0.104208021	0.769941192
ENSG00000236753	*MKLN1-AS*	0.822068242	-0.097657734	0.109582692	-0.207240426	0.999933887	-0.517533969	-0.647794536	0.130260567	0.769941192
ENSG00000259642	*ST20-AS1*	2.879427295	0.0269727	0.138002347	-0.111029647	0.999933887	0.233103407	0.145565887	0.08753752	0.769941192
ENSG00000170185	*USP38*	5.087261986	0.051452122	0.048320173	0.003131949	0.999933887	-0.408643065	-0.361184387	-0.047458678	0.770036363
ENSG00000132965	*ALOX5AP*	7.934049617	-0.004726872	-0.014856552	0.01012968	0.999933887	0.180254288	0.234602448	-0.054348161	0.770036363
ENSG00000105556	*MIER2*	1.721366935	0.130167235	0.157034349	-0.026867114	0.999933887	-0.104548277	-0.186711806	0.082163529	0.770496597
ENSG00000148925	*BTBD10*	3.548560199	-0.076561906	0.015602505	-0.092164411	0.999933887	-0.462309742	-0.409727059	-0.052582683	0.770496597
ENSG00000169224	*GCSAML*	-0.172006333	-0.230554837	-0.086347114	-0.144207723	0.999933887	-0.168900762	0.019825873	-0.188726636	0.770517278
ENSG00000253385	*AP003696.1*	-0.590588575	0.235888761	0.357755434	-0.121866673	0.999933887	1.391154534	1.638436638	-0.247282104	0.770689411
ENSG00000259943	*AL050341.2*	1.080154599	-0.271875191	-0.167402368	-0.104472824	0.999933887	0.016147575	0.096284674	-0.080137099	0.771024678
ENSG00000129226	*CD68*	0.013238014	0.287668054	0.17148117	0.116186884	0.999933887	0.689823466	0.863761842	-0.173938376	0.771084313
ENSG00000185950	*IRS2*	9.341709573	-0.011227972	0.05038872	-0.061616693	0.999933887	-0.320935697	-0.280053137	-0.04088256	0.771289196
ENSG00000167562	*ZNF701*	3.112089391	-0.046465353	-0.170676425	0.124211072	0.999933887	-0.545481682	-0.485615201	-0.059866481	0.771550797
ENSG00000167984	*NLRC3*	4.450060059	-0.016854238	0.0822097	-0.099063938	0.999933887	0.038442169	-0.001020939	0.039463109	0.771665451
ENSG00000260910	*LINC00565*	-0.35247242	-0.120362623	-0.310626738	0.190264115	0.999933887	-0.034687697	0.124693739	-0.159381435	0.771688263
ENSG00000106263	*EIF3B*	4.830965014	0.007346329	0.08631983	-0.078973502	0.999933887	0.114034867	0.07529537	0.038739497	0.771688263
ENSG00000082512	*TRAF5*	3.15499042	-0.031252627	-0.184276222	0.153023595	0.999933887	-0.051157478	0.011377265	-0.062534743	0.771884068
ENSG00000119714	*GPR68*	3.33169672	0.119892986	0.007308675	0.112584312	0.999933887	0.133677544	0.195951727	-0.062274183	0.771943241
ENSG00000170629	*DPY19L2P2*	-0.03494467	-0.088661812	0.08819114	-0.176852951	0.999933887	0.147525652	0.294873511	-0.147347859	0.771943241
ENSG00000005448	*WDR54*	1.555435951	-0.027705836	-0.021020077	-0.006685758	0.999933887	0.12345809	0.207664582	-0.084206492	0.772314853
ENSG00000168477	*TNXB*	0.450762745	0.244492017	0.010294744	0.234197272	0.999933887	-0.087563674	-0.230882719	0.143319045	0.772512864
ENSG00000279057	*AC141586.4*	-0.292079675	-0.019916566	0.079336138	-0.099252704	0.999933887	-0.652180833	-0.486755805	-0.165425028	0.772512864
ENSG00000135829	*DHX9*	6.498520849	-0.084317027	-0.113534181	0.029217154	0.999933887	-0.165465884	-0.200057748	0.034591864	0.772513638
ENSG00000134321	*RSAD2*	7.056415957	-0.01433639	-0.030742624	0.016406234	0.999933887	0.164991841	0.220858015	-0.055866173	0.772513638
ENSG00000277590	*MIR7845*	-1.288625938	0.444717134	0.166399646	0.278317489	0.999933887	0.679458835	0.981050317	-0.301591482	0.772532367
ENSG00000258922	*AC106028.2*	0.120162997	-0.048886728	0.035055906	-0.083942633	0.999933887	1.050123217	1.178309236	-0.12818602	0.772636878
ENSG00000175414	*ARL10*	1.509921822	-0.07231914	0.099600607	-0.171919747	0.999933887	0.05241303	-0.025547409	0.077960439	0.772891715
ENSG00000204623	*ZNRD1ASP*	1.027597666	-0.044136772	0.157530419	-0.201667191	0.999933887	-0.019994746	0.062788682	-0.082783428	0.772891715
ENSG00000004478	*FKBP4*	2.59775691	0.035419748	0.043599392	-0.008179644	0.999933887	0.330886571	0.260701251	0.070185319	0.77309045
ENSG00000174123	*TLR10*	2.925864448	-0.030182076	-0.156198054	0.126015978	0.999933887	0.130316428	0.012123981	0.118192447	0.77309045
ENSG00000276023	*DUSP14*	0.451303018	-0.023339155	-0.067667343	0.044328188	0.999933887	0.112154129	-0.018847873	0.131002001	0.77309045
ENSG00000038532	*CLEC16A*	3.95793806	-0.004893293	0.005210014	-0.010103307	0.999933887	-0.085353803	-0.131200144	0.045846341	0.77309045
ENSG00000174996	*KLC2*	2.296394975	0.10001265	0.233454575	-0.133441926	0.999933887	0.303087284	0.380405823	-0.07731854	0.773155223
ENSG00000180329	*CCDC43*	2.706554909	-0.128129217	-0.22421071	0.096081493	0.999933887	-0.126152399	-0.062372866	-0.063779533	0.77324581
ENSG00000158411	*MITD1*	4.185293502	-0.063737787	-0.125815711	0.062077924	0.999933887	0.10925192	0.069789227	0.039462693	0.77324581
ENSG00000146574	*CCZ1B*	1.622548199	-0.093270723	-0.043170676	-0.050100048	0.999933887	-0.29723935	-0.368743007	0.071503657	0.77324581
ENSG00000183828	*NUDT14*	0.318199575	0.166289401	0.147403306	0.018886095	0.999933887	-0.176066922	-0.288553719	0.112486797	0.77324581
ENSG00000228663	*PSMD10P1*	-0.14397151	-0.15880242	0.348549876	-0.507352295	0.999933887	0.070129533	-0.128297619	0.198427152	0.77324581
ENSG00000120158	*RCL1*	1.467058684	-0.04976964	0.142995029	-0.192764669	0.999933887	0.192574632	0.300642728	-0.108068097	0.77324581
ENSG00000145715	*RASA1*	4.439224139	0.052412422	-0.022242854	0.074655276	0.999933887	-0.391170974	-0.502543526	0.111372552	0.77324581
ENSG00000215417	*MIR17HG*	0.386201896	-0.10561634	-0.092272034	-0.013344306	0.999933887	0.279270033	0.41509827	-0.135828237	0.773454795
ENSG00000006652	*IFRD1*	8.623401587	0.015321451	0.03362465	-0.0183032	0.999933887	0.06923832	0.112643161	-0.043404841	0.773454795
ENSG00000180628	*PCGF5*	5.08433644	0.010327215	-0.067452312	0.077779527	0.999933887	-0.384713776	-0.324469936	-0.06024384	0.773454795
ENSG00000168556	*ING2*	2.72317397	0.112994966	0.019520522	0.093474444	0.999933887	0.180443415	0.10405894	0.076384475	0.773534781
ENSG00000170633	*RNF34*	4.843818432	-0.043518767	-0.038151278	-0.005367489	0.999933887	-0.063278804	-0.026803564	-0.03647524	0.773635224
ENSG00000105755	*ETHE1*	0.571754916	0.070193038	0.35611248	-0.285919442	0.999933887	-0.024399017	0.079883514	-0.104282531	0.773953894
ENSG00000095564	*BTAF1*	6.309600492	0.003921223	-0.103687811	0.107609034	0.999933887	-0.136166625	-0.062282585	-0.07388404	0.773953894
ENSG00000167699	*GLOD4*	3.64874249	-0.053023158	-0.118988538	0.06596538	0.999933887	0.070403795	0.109157396	-0.038753601	0.774024271
ENSG00000149218	*ENDOD1*	1.435813165	0.101059099	-0.006659155	0.107718254	0.999933887	0.073658841	-0.021514708	0.095173549	0.774024271
ENSG00000110492	*MDK*	0.137011226	0.184678868	0.029129013	0.155549854	0.999933887	0.065784096	0.211192156	-0.14540806	0.77412394
ENSG00000059691	*GATB*	-0.038784151	-0.063537736	-0.37149962	0.307961884	0.999933887	0.223517715	0.356958033	-0.133440318	0.774125697
ENSG00000249412	*AC010285.1*	-0.252582327	-0.040409123	0.183989521	-0.224398643	0.999933887	-0.32815018	-0.225502265	-0.102647915	0.774125697
ENSG00000106399	*RPA3*	1.926950091	-0.138404339	0.133451823	-0.271856162	0.944094537	0.015306228	0.09249268	-0.077186451	0.774293985
ENSG00000197601	*FAR1*	6.158503491	-0.085496111	-0.218077937	0.132581827	0.999933887	-0.902232992	-0.771120481	-0.131112511	0.774293985
ENSG00000099956	*SMARCB1*	3.812624965	0.013377513	-0.061422144	0.074799657	0.999933887	0.107282417	0.151842718	-0.044560301	0.774293985

ENSG00000254363	*AC011379.2*	-0.627973432	-0.008444546	0.082280322	-0.090724868	0.999933887	-0.027116344	0.130232448	-0.157348791	0.774293985
ENSG00000114779	*ABHD14B*	2.900347838	0.07817903	-0.053253692	0.131432722	0.999933887	0.109603174	0.173961233	-0.064358059	0.77442889
ENSG00000248643	*RBM14-RBM4*	0.187996427	0.191175351	0.253173064	-0.061997713	0.999933887	0.167460791	0.284797138	-0.117336347	0.774500371
ENSG00000241157	*AC104763.1*	0.325791819	0.015297234	-0.150468937	0.165766171	0.999933887	-0.36404835	-0.501762427	0.137714077	0.774551411
ENSG00000120709	*FAM53C*	7.953520592	0.056739457	0.015830792	0.040908665	0.999933887	-0.537603741	-0.592196626	0.054592885	0.774563694
ENSG00000139722	*VPS37B*	6.700100258	-0.039969726	-0.021689064	-0.018280662	0.999933887	-0.217921376	-0.258437414	0.040516038	0.774563694
ENSG00000264017	*RN7SL336P*	0.289457937	0.053949808	0.118422101	-0.064472293	0.999933887	1.02286337	0.878005861	0.144857509	0.774563694
ENSG00000213906	*LTB4R2*	0.504661386	0.043099821	-0.05846649	0.101566311	0.999933887	-0.124846584	0.022823801	-0.147670385	0.774563694
ENSG00000120925	*RNF170*	1.083227096	0.015786693	0.028886093	-0.013099401	0.999933887	-0.179762687	-0.285618631	0.105855944	0.774563694
ENSG00000227598	*Z94721.1*	0.457784996	-0.010680009	-0.08581137	0.075131361	0.999933887	-0.709427497	-0.884893929	0.175466433	0.774563694
ENSG00000011478	*QPCTL*	2.945020435	0.078570541	0.12907348	-0.050502939	0.999933887	0.257568583	0.324586119	-0.067017536	0.77459624
ENSG00000240038	*AMY2B*	2.897916141	-0.054103984	-0.173843413	0.119739429	0.999933887	-0.160760804	-0.061743549	-0.099017255	0.77459624
ENSG00000237296	*SMG1P1*	-0.036464529	-0.054375993	0.026367567	-0.08074356	0.999933887	0.413987539	0.546293096	-0.132305556	0.77459624
ENSG00000168758	*SEMA4C*	3.004028737	0.042228368	-0.014669682	0.05689805	0.999933887	0.089983032	0.150098913	-0.060115881	0.774656165
ENSG00000102763	*VWA8*	2.046948951	0.008798594	-0.062052468	0.070851062	0.999933887	-0.036171644	0.061510594	-0.097682239	0.774656165
ENSG00000279433	*AC018529.3*	3.859595812	0.170224597	0.314482585	-0.144257987	0.999933887	0.131121502	0.223281649	-0.092160147	0.774795196
ENSG00000154330	*PGM5*	1.443492929	0.033321754	-0.070242585	0.10356434	0.999933887	-0.147663699	-0.245858975	0.098195276	0.774795196
ENSG00000188739	*RBM34*	-0.454920189	-0.198282278	0.008240542	-0.20652282	0.999933887	0.080368452	-0.088909652	0.169278104	0.775172981
ENSG00000196372	*ASB13*	-0.833616163	-0.071097346	-0.138796619	0.067699273	0.999933887	0.179713446	-0.014032712	0.193746158	0.775172981
ENSG00000157426	*AASDH*	0.872691004	0.014483075	-0.207871383	0.222354458	0.999933887	0.089969467	0.198428312	-0.108458845	0.775172981
ENSG00000105819	*PMPCB*	4.411708509	-0.098139292	-0.10742328	0.009283988	0.999933887	-0.039962024	-0.002833544	-0.037128479	0.775191063
ENSG00000279520	*AC093525.8*	6.224872579	0.143917788	0.116640492	0.027277296	0.999933887	0.595391061	0.536702472	0.058688588	0.775191063
ENSG00000279330	*AJ003147.3*	1.70145014	-0.231535346	0.054160208	-0.285695555	0.999933887	-0.048260438	-0.150240393	0.101979955	0.775191063
ENSG00000156261	*CCT8*	4.100492715	-0.130760118	-0.085174642	-0.045585476	0.999933887	-0.097549592	-0.046359178	-0.051190414	0.775191063
ENSG00000119203	*CPSF3*	2.007298418	-0.166216459	-0.109164643	-0.057051816	0.999933887	0.060853132	0.125811455	-0.064958324	0.775191063
ENSG00000149262	*INTS4*	3.642695593	-0.092135931	0.000854863	-0.092990794	0.999933887	0.050803373	0.005713916	0.045089457	0.775191063
ENSG00000134056	*MRPS36*	2.365360442	0.148635174	0.058770282	0.089864893	0.999933887	0.109110848	0.02829617	0.080814677	0.775191063
ENSG00000132305	*IMMT*	3.408119524	-0.085139242	-0.107161232	0.022021991	0.999933887	0.018112324	-0.031105323	0.049217647	0.775191063
ENSG00000241106	*HLA-DOB*	0.479040589	-0.185135606	0.008199788	-0.193335394	0.999933887	-0.0134026	0.134020848	-0.147423448	0.775191063
ENSG00000139517	*LNX2*	1.938622682	0.111475392	0.048277934	0.063197458	0.999933887	-0.397074573	-0.471654658	0.074580085	0.775191063
ENSG00000141959	*PFKL*	5.626948431	0.079240965	0.038216307	0.041024658	0.999933887	0.266518598	0.321668337	-0.055149739	0.775191063
ENSG00000154328	*NEIL2*	0.730830697	-0.128995406	-0.208971793	0.079976387	0.999933887	0.046642787	0.14593299	-0.099290203	0.775191063
ENSG00000221909	*FAM200A*	0.572158987	-0.179133758	-0.142886786	-0.036246972	0.999933887	0.025240105	0.146380774	-0.121140669	0.775191063
ENSG00000117505	*DR1*	5.655048028	-0.05168978	-0.008063278	-0.043626502	0.999933887	-0.350142529	-0.392338814	0.042196285	0.775191063
ENSG00000134644	*PUM1*	5.540135159	-0.041506654	-0.032654559	-0.008852095	0.999933887	-0.079183673	-0.044408757	-0.034774916	0.775191063
ENSG00000197980	*LEKR1*	-0.528176974	0.151658566	0.330441952	-0.178783386	0.999933887	0.744111398	0.539337141	0.204774256	0.775191063
ENSG00000188242	*PP7080*	0.385160562	0.135763858	0.159282131	-0.023518273	0.999933887	0.066755857	0.18572566	-0.118969802	0.775191063
ENSG00000145555	*MYO10*	3.187458275	-0.074084408	0.041523147	-0.115607555	0.999933887	-0.300617423	-0.362307526	0.061690103	0.775191063
ENSG00000247626	*MARS2*	0.95409941	0.091042823	-0.070605064	0.161647887	0.999933887	0.158888752	0.277235093	-0.118346341	0.775191063
ENSG00000261456	*TUBB8*	-2.158368029	-0.199813953	-0.570411072	0.370597119	0.999933887	-1.629477801	-1.428276344	-0.201201456	0.775191063
ENSG00000163806	*SPDYA*	0.834258067	0.065017151	0.052343966	0.012673185	0.999933887	0.055152831	0.210343178	-0.155190348	0.775191063
ENSG00000269119	*HNRNPA1P52*	0.380709151	-0.065175028	-0.24544751	0.180272483	0.999933887	-0.345649183	-0.506516653	0.16086747	0.775191063
ENSG00000281691	*RBM5-AS1*	-0.156117496	-0.110392098	0.026432372	-0.13682447	0.999933887	-0.027449097	0.12297485	-0.150423947	0.775191063
ENSG00000170515	*PA2G4*	2.969779087	-0.03499681	0.027412496	-0.062409307	0.999933887	0.134755289	0.063773701	0.070981588	0.775191063
ENSG00000229463	*LYST-AS1*	-0.454141792	0.075709001	-0.008243898	0.0839529	0.999933887	-0.118846726	0.104680683	-0.22352741	0.775191063
ENSG00000257103	*LSM14A*	6.350519116	-0.013569312	-0.002710674	-0.010858638	0.999933887	-0.184737639	-0.152260472	-0.032477167	0.775191063
ENSG00000198467	*TPM2*	1.958024173	-0.030271425	-0.01797036	-0.012301065	0.999933887	0.049105356	0.131296888	-0.082191532	0.775191063
ENSG00000066468	*FGFR2*	-0.011944207	0.03156454	0.025857969	0.005706571	0.999933887	-0.142398476	-0.014104179	-0.128294296	0.775191063
ENSG00000163584	*RPL22L1*	2.724345822	0.013373785	-0.009812761	0.023186547	0.999933887	0.288969108	0.354093642	-0.065124535	0.775191063
ENSG00000196557	*CACNA1H*	-0.057080513	-0.029958676	0.392954829	-0.422913506	0.999933887	0.005744016	0.159666048	-0.153922032	0.775191063
ENSG00000144867	*SRPRB*	1.631921791	0.015577943	-0.086930631	0.102508574	0.999933887	0.268828952	0.3456113	-0.076782348	0.775191063
ENSG00000279700	*AC131212.2*	1.427430661	-0.016120114	-0.149056778	0.132936664	0.999933887	-0.254687364	-0.368356183	0.113668818	0.775191063
ENSG00000132386	*SERPINF1*	-0.879520551	0.030069016	-0.395239007	0.425308023	0.999933887	0.110239155	-0.083740228	0.193979383	0.775191063
ENSG00000149573	*MPZL2*	1.419195597	0.011832097	-0.025033708	0.036865805	0.999933887	-0.323039479	-0.414686522	0.091647043	0.775191063
ENSG00000166979	*EVA1C*	0.565234698	0.013784141	0.017692415	-0.003908274	0.999933887	0.21124829	0.334031848	-0.122783558	0.775191063
ENSG00000104884	*ERCC2*	0.933012312	-0.012516895	-0.027402525	0.01488563	0.999933887	0.020364456	0.120806991	-0.100442535	0.775191063
ENSG00000272906	*AL353708.3*	0.554718194	0.015837073	0.079150363	-0.06331329	0.999933887	0.240970818	0.127318759	0.113652059	0.775191063
ENSG00000049883	*PTCD2*	0.16677124	-0.012875365	-0.056108959	0.043233594	0.999933887	-0.059906298	0.078155424	-0.138061722	0.775191063
ENSG00000151665	*PIGF*	0.74487587	-0.005694645	0.249405899	-0.255100544	0.999933887	0.029090996	0.130593908	-0.101502912	0.775191063
ENSG00000129194	*SOX15*	-0.01070459	0.321997104	0.36069852	-0.038701417	0.999933887	0.679539339	0.528309579	0.15122976	0.775233918
ENSG00000164934	*DCAF13*	3.302710286	-0.101125868	0.007850404	-0.108976272	0.999933887	-0.239004038	-0.185914223	-0.053089815	0.775233918
ENSG00000106028	*SSBP1*	3.644128816	-0.072099138	-0.040453328	-0.031645809	0.999933887	-0.069524104	-0.024688045	-0.04483606	0.775233918
ENSG00000251247	*ZNF345*	0.46520598	0.135341503	-0.011579908	0.146921411	0.999933887	-0.148454519	-0.027674224	-0.120780295	0.775233918
ENSG00000247077	*PGAM5*	1.940144427	-0.068303771	0.009159938	-0.07746371	0.999933887	0.23700363	0.306359191	-0.069355561	0.775233918
ENSG00000281958	*TRBJ1-4*	0.222722455	-0.02362256	0.213698525	-0.237321085	0.999933887	0.165901934	0.056588431	0.109313502	0.775233918
ENSG00000226272	*ARHGAP26-A*	0.802436661	0.291908134	0.597106851	-0.305198717	0.999933887	0.364006564	0.084313039	0.279693526	0.775573053
ENSG00000176248	*ANAPC2*	5.244398862	0.024847393	0.110859289	-0.086011895	0.999933887	0.145953228	0.191228168	-0.04527494	0.775573053
ENSG00000060762	*MPC1*	3.72984044	-0.077555691	-0.019406426	-0.058149265	0.999933887	-0.13945789	-0.088371245	-0.051086645	0.775696593
ENSG00000259912	*AC023813.1*	0.198583647	0.020776146	0.148790439	-0.128014293	0.999933887	3.231196873	3.396033242	-0.164836369	0.775860868
ENSG00000122545	*SEPTIN7*	6.47279796	-0.078082867	-0.136583551	0.058500684	0.999933887	-0.486287312	-0.415903452	-0.07038386	0.776183724
ENSG00000136783	*NIPSNAP3A*	0.815323701	-0.110882799	0.040053927	-0.150936726	0.999933887	-0.135430338	-0.027894052	-0.107536286	0.776183724
ENSG00000071205	*ARHGAP10*	1.770584109	-0.076453687	0.129172153	-0.205625839	0.999933887	-0.172840264	-0.25591188	0.083071616	0.776183724
ENSG00000242498	*ARPIN*	-0.371127848	0.016296906	0.30069356	-0.284396654	0.999933887	-1.21776466	-1.370933712	0.153169052	0.776183724
ENSG00000146833	*TRIM4*	3.118498515	-0.052137931	0.005803062	-0.057940993	0.999933887	-0.177762433	-0.126860683	-0.05090175	0.776364097
ENSG00000125484	*GTF3C4*	2.618244121	-0.014826135	0.022568553	-0.037394688	0.999933887	0.074663162	0.011332145	0.063331017	0.776364097
ENSG00000100058	*CRYBB2P1*	1.148447908	0.06638885	-0.065940513	0.132329363	0.999933887	0.151401519	0.241810345	-0.090408826	0.776459199
ENSG00000089220	*PEBP1*	3.872132152	0.002845778	0.020458859	-0.01761308	0.999933887	0.203538132	0.143272515	0.060265617	0.776624297
ENSG00000149503	*INCENP*	2.206664573	0.047849158	-0.067924818	0.115773977	0.999933887	-0.154968226	-0.08787061	-0.067097615	0.776643178
ENSG00000207171	*AC079926.1*	-0.684349619	0.338756845	0.618268807	-0.279511962	0.999933887	1.189289718	0.965949625	0.223340093	0.776674857
ENSG00000109971	*HSPA8*	7.360212634	-0.002713088	-0.069497643	0.066784555	0.999933887	0.095577215	0.051593904	0.04398331	0.776731891
ENSG00000167994	*RAB3IL1*	2.371156415	-0.038882738	-0.101221045	0.062338307	0.999933887	-0.680903658	-0.620570946	-0.060332712	0.776738136
ENSG00000237440	*ZNF737*	1.745556974	-0.060620883	-0.154927658	0.094306775	0.999933887	-0.318185269	-0.414871675	0.096686405	0.776946947
ENSG00000186010	*NDUFA13*	0.174354192	0.125974093	0.062779016	0.063195078	0.999933887	-0.114759537	-0.224268561	0.109509024	0.77766106
ENSG00000102981	*PARD6A*	0.843916288	0.166821509	-0.170075085	0.336896594	0.999933887	-0.442970791	-0.600480349	0.157509558	0.777756211
ENSG00000267416	*AC025048.4*	-0.848604241	-0.209013039	-0.116790027	-0.092223013	0.999933887	-0.143156331	-0.281604108	0.138447777	0.777816269
ENSG00000188827	*SLX4*	2.502326621	0.066631572	0.049653699	0.016977873	0.999933887	-0.103564568	-0.181310936	0.077746368	0.777816269
ENSG00000246223	*LINC01550*	0.335206363	0.097176262	-0.250447196	0.347623458	0.999933887	-0.004763861	0.110864622	-0.115628484	0.777816269
ENSG00000136854	*STXBP1*	-0.780650185	0.078492182	-0.054531563	0.133023744	0.999933887	-0.098641534	0.068518338	-0.167159872	0.777816269
ENSG00000241860	*AL627309.5*	2.201360767	-0.025156175	0.044985155	-0.07014133	0.999933887	0.320743003	0.411919532	-0.091176529	0.777835344
ENSG00000183666	*GUSBP1*	-0.238352722	-0.045175343	0.122138874	-0.167314217	0.999933887	-0.352603951	-0.200796761	-0.15180719	0.778044921
ENSG00000222057	*RNU4-62P*	1.104857787	0.086606568	0.053014095	0.033592473	0.999933887	-0.334681036	-0.493218497	0.158537461	0.778239456
ENSG00000004468	*CD38*	1.254821433	0.028912613	-0.00771558	0.036628193	0.999933887	-0.216919672	-0.120895365	-0.096024306	0.778239456
ENSG00000127774	*EMC6*	-0.447415566	-0.061942129	-0.196345884	0.134403755	0.999933887	-0.157197887	-0.029307727	-0.127890161	0.778255254
ENSG00000157538	*VPS26C*	5.717549955	-0.005658088	-0.060371805	0.054713718	0.999933887	-0.733310566	-0.693020654	-0.040289912	0.778374326
ENSG00000197753	*LHFPL5*	4.668362487	-0.073355253	-0.00974712	-0.063608132	0.999933887	-0.762729216	-0.698791693	-0.063937523	0.778473457
ENSG00000051128	*HOMER3*	3.297554039	0.058627918	0.02647662	0.032151298	0.999933887	0.131719639	0.193499452	-0.061779813	0.778481033
ENSG00000164047	*CAMP*	1.861817241	-0.007505878	0.035950092	-0.04345597	0.999933887	-0.054770766	-0.150964052	0.096193286	0.778481033
ENSG00000211794	*TRAV12-3*	-0.499265577	-0.008228058	0.066857986	-0.075086043	0.999933887	0.18330569	0.322417795	-0.139112104	0.778481033
ENSG00000181191	*PJA1*	1.344532297	0.004237208	-0.233070441	0.237307649	0.999933887	0.091948874	0.175231797	-0.083282923	0.778481033
ENSG00000228649	*SNHG26*	0.138513306	0.155930222	-0.198467397	0.354397619	0.999933887	0.18269202	0.313001313	-0.130309294	0.778608028
ENSG00000178199	*ZC3H12D*	4.448057637	0.037149553	-0.00349275	0.040642303	0.999933887	0.27735648	0.325027259	-0.047670779	0.778652553
ENSG00000198286	*CARD11*	4.907195554	-0.006623458	0.027406901	-0.034030359	0.999933887	0.077814043	0.12259514	-0.044781096	0.778652553
ENSG00000274712	*AC005332.4*	1.753331219	-0.010490802	-0.038147135	0.027656333	0.999933887	0.062846631	-0.020694894	0.083541525	0.778652553
ENSG00000188389	*PDCD1*	1.994286616	0.108886962	0.063045933	0.045841029	0.999933887	0.148863798	0.071808181	0.077055617	0.778803207
ENSG00000198146	*ZNF770*	2.543255963	-0.122767519	-0.042798658	-0.079968861	0.999933887	-0.302566792	-0.196069616	-0.106497176	0.779021065

ENSG00000162928	*PEX13*	1.689467404	-0.087127598	0.007451888	-0.094579485	0.999933887	-0.129616581	-0.203937204	0.074320623	0.779021065
ENSG00000215788	*TNFRSF25*	4.101876276	0.050232661	0.065383921	-0.01515126	0.999933887	0.260976171	0.201893398	0.059082773	0.779126514
ENSG00000161328	*LRRC56*	0.127470843	0.038025631	-0.057101421	0.095127052	0.999933887	0.058878994	0.185970485	-0.127091491	0.779126514
ENSG00000229413	*AC018638.1*	0.744872578	0.057809279	-0.139748522	0.197557801	0.999933887	-0.081097461	-0.261360257	0.180262796	0.779132585
ENSG00000276234	*TADA2A*	0.71127642	-0.001626208	-0.06609727	0.064471062	0.999933887	0.075334297	-0.024561395	0.099895692	0.779132585
ENSG00000170836	*PPM1D*	4.329540299	0.026380447	0.01814463	0.008235817	0.999933887	-0.388733292	-0.339163502	-0.04956979	0.779294013
ENSG00000253111	*AC091114.1*	1.074309684	-0.10619804	0.266239621	-0.37243766	0.999933887	1.512996168	1.636685686	-0.123689518	0.779295515
ENSG00000167107	*ACSF2*	1.762565163	-0.023990888	-0.020834013	-0.003156875	0.999933887	0.024715508	-0.060302125	0.085017633	0.779370715
ENSG00000126870	*DYNC2I1*	2.200663342	-0.012924841	0.037032704	-0.049957544	0.999933887	0.145887209	0.208062755	-0.062175546	0.779370715
ENSG00000089094	*KDM2B*	4.616008508	0.025598464	0.067948146	-0.042349681	0.999933887	0.06863167	0.113213273	-0.044581604	0.779448897
ENSG00000279801	*AC111170.3*	0.42159372	-0.287640602	-0.080796659	-0.206843943	0.999933887	-0.410045443	-0.516358477	0.106313034	0.779611694
ENSG00000288538	*AP002985.1*	0.351535402	-0.053484902	0.029356223	-0.082841126	0.999933887	-0.248203648	-0.120356674	-0.127846974	0.779757629
ENSG00000102030	*NAA10*	4.563915869	0.04994418	0.109257071	-0.059312891	0.999933887	0.15702296	0.095629722	0.061393238	0.780004004
ENSG00000115085	*ZAP70*	6.376097932	0.037205815	0.111372348	-0.074166532	0.999933887	0.131656366	0.084714595	0.04694177	0.78003374
ENSG00000225636	*HNRNPA3P15*	-1.034015715	0.071877876	0.017368297	0.054509579	0.999933887	-0.22580882	-0.492266572	0.266457751	0.78003374
ENSG00000140718	*FTO*	1.880285687	-0.095095701	-0.124075785	0.028980084	0.999933887	0.00388052	0.09210045	-0.08821993	0.780068449
ENSG00000144120	*TMEM177*	-1.366761093	-0.222436482	-0.469034433	0.246597951	0.999933887	-0.022908563	-0.238000144	0.21509158	0.780155261
ENSG00000073536	*NLE1*	0.909274894	0.113015662	0.069845772	0.04316989	0.999933887	0.357364624	0.263124168	0.094240456	0.780257668
ENSG00000197019	*SERTAD1*	5.090920052	0.132637133	0.1547984	-0.022161267	0.999933887	-0.018794302	0.031784635	-0.050578937	0.780350935
ENSG00000168827	*GFM1*	2.606452936	-0.104130866	-0.100289385	-0.003841481	0.999933887	0.00763586	0.066886417	-0.059250557	0.780350935
ENSG00000267430	*AC036176.2*	0.265380239	0.156119357	0.467091017	-0.31097166	0.999933887	0.102567582	0.28895448	-0.186386898	0.780350935
ENSG00000177570	*SAMD12*	-1.100735438	0.123284153	-0.35011687	0.473401024	0.999933887	-0.019371463	-0.209471947	0.190100484	0.780350935
ENSG00000157593	*SLC35B2*	5.324087738	0.065772781	0.100028751	-0.03425597	0.999933887	1.711681986	1.792819826	-0.08113784	0.780350935
ENSG00000248098	*BCKDHA*	-0.360280808	-0.120284962	-0.166997841	0.046712878	0.999933887	-0.486540112	-0.28095296	-0.205587152	0.780350935
ENSG00000204104	*TRAF3IP1*	0.482569319	-0.076143235	0.033713484	-0.109856719	0.999933887	0.106857157	0.221427218	-0.11457006	0.780350935
ENSG00000174206	*C12orf66*	0.389633233	-0.296142261	0.245345848	-0.541488109	0.940218856	0.327992714	0.224222861	0.103769853	0.780471716
ENSG00000180488	*MIGA1*	1.809628528	-0.069847081	-0.005153514	-0.064693568	0.999933887	-0.172272992	-0.070239765	-0.102033227	0.780471716
ENSG00000112763	*BTN2A1*	6.842851475	0.01971567	0.056034683	-0.036319013	0.999933887	0.041870209	0.080136905	-0.038266696	0.780725165
ENSG00000159579	*RSPRY1*	3.798994786	-0.02006945	-0.111469168	0.091399718	0.999933887	-0.403604166	-0.356444138	-0.047160028	0.78086707
ENSG00000149639	*SOGA1*	0.750269728	-0.032465162	0.033925931	-0.066391093	0.999933887	-0.907266554	-1.038665766	0.131399212	0.781110236
ENSG00000222267	*RNU6-892P*	-0.09894163	-0.283898593	-0.019112133	-0.26478646	0.999933887	0.30997085	0.46266797	-0.15269712	0.781457311
ENSG00000171161	*ZNF672*	5.355076078	0.050154344	-0.029675847	0.079830191	0.999933887	-0.197844622	-0.240379462	0.04253484	0.781609694
ENSG00000224032	*EPB41L4A-AS*	2.305165095	-0.039785051	0.039552414	-0.079337465	0.999933887	0.048226995	0.121246165	-0.073019169	0.781609694
ENSG00000188895	*MSL1*	8.562490012	-0.004784107	0.03567692	-0.040461027	0.999933887	-0.584456592	-0.63172779	0.047271198	0.78171069
ENSG00000164855	*TMEM184A*	1.488763337	0.001181584	0.105137617	-0.103956033	0.999933887	0.053985985	0.147578367	-0.093592382	0.781726898
ENSG00000259248	*USP3-AS1*	1.769918608	-0.033994694	0.026563435	-0.060558129	0.999933887	-0.359498225	-0.207332768	-0.152165457	0.781789279
ENSG00000106070	*GRB10*	2.521102493	-0.007038823	0.062914819	-0.069953642	0.999933887	-0.050537133	-0.113042593	0.06250546	0.781805811
ENSG00000107404	*DVL1*	4.18130137	0.056382252	0.053011637	0.003370615	0.999933887	-0.011654948	0.046813283	-0.058468231	0.781859779
ENSG00000215014	*AL645728.1*	0.820993263	-0.015747924	0.064416938	-0.080164862	0.999933887	0.27755586	0.404932297	-0.127376437	0.781966303
ENSG00000083457	*ITGAE*	2.278803532	-0.041393332	-0.20613116	0.164737828	0.999933887	-0.098914038	-0.161118438	0.0622044	0.782228496
ENSG00000154027	*AK5*	-0.568359236	0.075170344	-0.200326442	0.275496785	0.999933887	-0.107821782	0.035414156	-0.143235938	0.782233975
ENSG00000002919	*SNX11*	4.318163308	-0.020915632	-0.036155025	0.015239394	0.999933887	-0.020205926	0.037319893	-0.057525819	0.782336232
ENSG00000139746	*RBM26*	4.843301557	-0.097455793	-0.180099591	0.082643798	0.999933887	-0.514869372	-0.43183138	-0.083037992	0.782410502
ENSG00000151835	*SACS*	2.983441505	-0.15701049	-0.166221843	0.009211353	0.999933887	-0.281884329	-0.155081315	-0.126803014	0.782784339
ENSG00000258987	*AL132642.1*	-0.919314645	-0.052540395	-0.004785012	-0.047755383	0.999933887	-0.330739857	-0.109409503	-0.221330354	0.782849731
ENSG00000171649	*ZIK1*	-0.707152098	-0.435438455	0.236396482	-0.671834937	0.969206975	-0.180654705	-0.039562844	-0.141091861	0.782868513
ENSG00000118894	*EEF2KMT*	-0.991041026	0.120632824	0.332697968	-0.212065145	0.999933887	0.305697815	0.480640046	-0.174942231	0.782868513
ENSG00000217165	*ANKRD18EP*	0.147012147	-0.077166285	-0.043035394	-0.034130892	0.999933887	-0.162945486	-0.028195209	-0.134750277	0.782879338
ENSG00000170989	*S1PR1*	5.132166853	-0.012046368	-0.071975534	0.059929166	0.999933887	0.108048562	0.06236093	0.045687632	0.782879338
ENSG00000136159	*NUDT15*	1.168222449	0.013189964	0.038003762	-0.024813798	0.999933887	0.036073527	-0.077027765	0.113101292	0.782879338
ENSG00000155749	*FLACC1*	-0.216328478	0.085093613	-0.276315824	0.361409438	0.999933887	-0.352929352	-0.538643485	0.185714133	0.78301999
ENSG00000143256	*PFDN2*	2.903984595	0.032690404	0.064434495	-0.031744091	0.999933887	-0.016311993	0.03832819	-0.054640183	0.78301999
ENSG00000126012	*KDM5C*	7.395873093	0.024272475	0.060076681	-0.035804207	0.999933887	-0.082640372	-0.111904861	0.029264488	0.783122487
ENSG00000133639	*BTG1*	8.625523225	0.018630428	0.023390713	-0.004760286	0.999933887	-0.032252697	-0.066969766	0.034717069	0.783166408
ENSG00000168675	*LDLRAD4*	2.574809703	0.029746199	0.133682669	-0.103936471	0.999933887	0.004709732	-0.052661321	0.057371053	0.783355618
ENSG00000149150	*SLC43A1*	0.787077593	-0.054612909	-0.325893148	0.271280239	0.999933887	0.013466971	0.111595429	-0.098128458	0.783826883
ENSG00000230834	*AC068580.2*	-1.070670085	0.255699152	-0.000212106	0.255911259	0.999933887	0.291374295	0.475268865	-0.183894569	0.783856072
ENSG00000214300	*SPDYE3*	0.123867609	0.193760573	-0.008736816	0.202497389	0.999933887	0.370548817	0.262316426	0.108232391	0.784074094
ENSG00000108474	*PIGL*	1.55805361	-0.019053247	0.04204194	-0.061095187	0.999933887	0.066409857	-0.014149647	0.080559505	0.784243831
ENSG00000259479	*SORD2P*	-0.043637674	0.035561303	0.025940337	0.009620966	0.999933887	-0.20060137	-0.319743816	0.119142446	0.784414078
ENSG00000170540	*ARL6IP1*	5.181208092	-0.084205303	-0.077857912	-0.006347391	0.999933887	-0.291883487	-0.341259671	0.049376184	0.784478366
ENSG00000125247	*TMTC4*	1.120341141	-0.0338748	0.061238962	-0.095113763	0.999933887	0.04018869	-0.04742294	0.08761163	0.784478366
ENSG00000142528	*ZNF473*	2.424916891	-0.041758162	-0.059342404	0.017584243	0.999933887	-0.02945375	0.049073699	-0.078527449	0.784537545
ENSG00000214456	*PLIN5*	4.214749954	0.056738954	0.068892636	-0.012153682	0.999933887	0.077736375	0.016468554	0.061267821	0.784861199
ENSG00000279908	*AC026748.3*	0.389493046	-0.015554445	0.018191166	-0.033745611	0.999933887	0.169977945	0.044159328	0.125818617	0.784861199
ENSG00000176083	*ZNF683*	1.711508135	0.110918656	-0.042115933	0.153034589	0.999933887	-0.034705038	0.036867335	-0.071572373	0.784908463
ENSG00000233901	*LINC01503*	1.868512441	0.015208666	-0.095004552	0.110213218	0.999933887	-0.536439396	-0.461341374	-0.075098022	0.784908463
ENSG00000241657	*TRBV11-2*	-0.394277273	0.117344422	0.11322249	0.004121933	0.999933887	0.205022315	0.360226105	-0.15520379	0.785001879
ENSG00000267123	*SCAT1*	-0.721488441	-0.109149953	-0.329617276	0.220467323	0.999933887	0.075249477	-0.098372159	0.173621636	0.785001879
ENSG00000245849	*RAD51-AS1*	2.769723059	-0.000308812	-0.044932318	0.044623506	0.999933887	-0.077839074	-0.02150193	-0.056337144	0.785001879
ENSG00000278434	*AC023830.3*	0.417934507	0.179953323	0.176495357	0.003457965	0.999933887	0.44972705	0.607721161	-0.15799411	0.785083646
ENSG00000138138	*ATAD1*	3.154310668	-0.112391122	-0.04314634	-0.069244781	0.999933887	-0.157342879	-0.220154362	0.062811483	0.78509922
ENSG00000204536	*CCHCR1*	2.174466101	0.084543811	-0.030255515	0.114799326	0.999933887	0.066586705	-0.010366868	0.076953573	0.78509922
ENSG00000185710	*SMG1P4*	-1.073880925	0.135704282	0.014869611	0.120834671	0.999933887	-0.147503757	0.040176007	-0.187679764	0.7851192
ENSG00000189403	*HMGB1*	6.401744522	-0.024312499	-0.042506393	0.018193894	0.999933887	-0.095932017	-0.132708293	0.036776277	0.785227048
ENSG00000113369	*ARRDC3*	7.476025709	-0.316177649	-0.415908295	0.099730646	0.999933887	-0.269143942	-0.203761844	-0.065382098	0.785246073
ENSG00000119041	*GTF3C3*	2.131015961	-0.110106506	-0.2468698	0.136763294	0.999933887	-0.000429656	0.084803436	-0.085233092	0.785246073
ENSG00000196296	*ATP2A1*	0.322595022	0.050261806	0.171190392	-0.120928587	0.999933887	-0.280702395	-0.434862733	0.154160338	0.785246073
ENSG00000182272	*B4GALNT4*	-0.813048531	0.153035369	0.176344292	-0.023308923	0.999933887	0.128478369	-0.087261548	0.215739918	0.785266702
ENSG00000273797	*AL133445.2*	1.559147024	0.126235906	0.286455521	-0.160219615	0.999933887	1.405804189	1.512311248	-0.106507059	0.785460792
ENSG00000213420	*GPC2*	0.947149043	0.116550515	0.035557788	0.080992727	0.999933887	-0.226707292	-0.348617859	0.121910567	0.785489795
ENSG00000184350	*MRGPRE*	-0.076494836	0.013072874	-0.069433593	0.082506466	0.999933887	-0.296971664	-0.387412226	0.090440562	0.785489795
ENSG00000203791	*EEF1AKMT2*	-0.362685391	0.002476639	-0.095459671	0.09793631	0.999933887	-0.3833953	-0.223489248	-0.159906053	0.785489795
ENSG00000184730	*APOBR*	8.787551313	0.074107279	0.16866735	-0.094560071	0.999933887	-0.42437688	-0.504571373	0.080194493	0.78560797
ENSG00000233750	*CICP27*	0.446502011	-0.026966315	-0.086418955	0.05945264	0.999933887	0.094071417	-0.074728113	0.16879953	0.78560797
ENSG00000264247	*LINC00909*	2.592057174	-0.110585901	-0.350615128	0.240029227	0.999933887	-0.203420645	-0.273560462	0.070139817	0.785635975
ENSG00000160691	*SHC1*	5.345628421	0.034956911	0.025379923	0.009576988	0.999933887	-0.157199358	-0.195356767	0.038157409	0.785803675
ENSG00000158234	*FAIM*	-0.285085004	-0.010348785	-0.041512504	0.031163719	0.999933887	-0.07599254	0.092006632	-0.167999172	0.785803675
ENSG00000171067	*C11orf24*	1.31234565	-0.03301718	0.104787263	-0.137804443	0.999933887	0.093825237	0.022439365	0.071385872	0.786138048
ENSG00000182512	*GLRX5*	2.469105098	0.069110663	0.048536646	0.020574017	0.999933887	-0.037512696	-0.110821541	0.073308845	0.786295704
ENSG00000253210	*AC040970.1*	3.899552005	0.056037442	0.033179714	0.022857728	0.999933887	-0.467142184	-0.405959733	-0.061182451	0.786295704
ENSG00000023228	*NDUFS1*	4.033232916	-0.120460703	-0.029026508	-0.091434195	0.999933887	-0.165014173	-0.126029984	-0.03898419	0.786576764
ENSG00000100441	*KHNYN*	6.644599708	0.01307873	-0.003791479	0.016870209	0.999933887	-0.183206296	-0.217471805	0.034265509	0.786660923
ENSG00000265692	*LINC01970*	-0.804894112	0.158967688	0.038291883	0.120675805	0.999933887	-0.269444111	-0.518538139	0.249094028	0.786673465
ENSG00000068394	*GPKOW*	3.120301305	0.024520411	-0.083256742	0.107777153	0.999933887	-0.049075442	0.007205842	-0.056281284	0.786673465
ENSG00000230724	*LINC01001*	1.727821844	0.030431651	-0.005992767	0.036424419	0.999933887	0.267492121	0.400099584	-0.132607463	0.786673465
ENSG00000146530	*VWDE*	-0.485650503	0.039141044	0.171006781	-0.131865738	0.999933887	-0.442688874	-0.565655938	0.122967064	0.786673465
ENSG00000105983	*LMBR1*	4.134941946	-0.008328303	-0.02185836	0.013530058	0.999933887	-0.130126491	-0.170901156	0.040774665	0.786673465
ENSG00000164483	*SAMD3*	2.891042415	0.010231236	-0.141673464	0.1519047	0.999933887	0.081570379	0.13100475	-0.049434371	0.786673465
ENSG00000281831	*HCP5B*	-1.029831128	0.015784154	0.143880825	-0.128096671	0.999933887	0.549763257	0.405852004	0.143911253	0.786673465
ENSG00000234238	*AC022498.1*	-0.451042537	0.364877963	0.647447962	-0.282569999	0.999933887	1.440851627	1.660316779	-0.219465152	0.786790507
ENSG00000105220	*GPI*	4.57647201	0.001950455	-0.01285257	0.014803025	0.999933887	0.005003559	-0.038726941	0.0437305	0.786790507
ENSG00000258831	*AC103996.2*	-0.601179666	-0.205785093	-0.209988758	0.004203665	0.999933887	0.480807393	0.314485938	0.166321455	0.786966529
ENSG00000255571	*MIR9-3HG*	-0.786653033	-0.046039458	-0.250736678	0.20469722	0.999933887	0.363411488	0.201597816	0.161813672	0.787087911
ENSG00000171840	*NINJ2*	3.740983399	0.024285785	0.042597645	-0.01831186	0.999933887	0.062411994	0.125035813	-0.062623819	0.787392875

ENSG00000162065	*TBC1D24*	1.035425485	-0.021927824	-0.135576779	0.113648955	0.999933887	0.004012015	-0.07738639	0.081398405	0.787392875
ENSG00000131401	*NAPSB*	-0.555521928	0.115173806	-0.152399025	0.267572831	0.999933887	-0.216366992	-0.075399876	-0.140967116	0.78748315
ENSG00000102316	*MAGED2*	3.54431536	0.01869578	0.009374834	0.009320946	0.999933887	0.127441872	0.076053948	0.051387924	0.78748315
ENSG00000198931	*APRT*	3.705450256	0.084507494	0.011619432	0.072888062	0.999933887	0.107974025	0.057886598	0.050087428	0.787570852
ENSG00000126368	*NR1D1*	6.088687419	0.024399258	0.114854422	-0.090455164	0.999933887	1.163116304	1.227472807	-0.064356503	0.787766503
ENSG00000132535	*DLG4*	0.824699999	-0.064707277	0.130294232	-0.195001509	0.999933887	-0.061361343	0.034731841	-0.096093184	0.788016404
ENSG00000117394	*SLC2A1*	4.878690898	0.001174931	0.047478847	-0.046303917	0.999933887	-0.396469526	-0.339583806	-0.05688572	0.788636929
ENSG00000236682	*MAP3K2-DT*	3.156991515	-0.065809989	0.043934462	-0.109744451	0.999933887	-0.744737094	-0.683768239	-0.060968855	0.788881431
ENSG00000169442	*CD52*	4.798565273	-0.011321003	-0.042752505	0.031431502	0.999933887	0.059557932	0.00737995	0.052177982	0.788881431
ENSG00000025800	*KPNA6*	5.07820238	-0.017506794	-0.020387809	0.002881016	0.999933887	-0.266317552	-0.307261807	0.040944255	0.78890607
ENSG00000104823	*ECH1*	2.638673146	-0.033167739	0.075893271	-0.10906101	0.999933887	0.068099555	0.123330224	-0.055230669	0.789026724
ENSG00000248092	*NNT-AS1*	-0.325803716	-0.324725759	-0.570698724	0.245972965	0.999933887	-0.198055537	-0.081456291	-0.116599245	0.789080675
ENSG00000125901	*MRPS26*	1.364744243	0.144673637	0.146752707	-0.00207907	0.999933887	0.043360329	-0.046394552	0.089754881	0.789080675
ENSG00000012779	*ALOX5*	7.565188602	0.045358578	0.025715879	0.019642699	0.999933887	0.122260278	0.160447976	-0.038187698	0.789080675
ENSG00000136938	*ANP32B*	5.192782372	-0.047706402	-0.039168794	-0.008537608	0.999933887	-0.038693643	0.000961184	-0.039654828	0.789080675
ENSG00000214176	*PLEKHM1P1*	6.56890235	0.034684024	0.014964832	0.019719192	0.999933887	-0.194947661	-0.225682364	0.030734703	0.789080675
ENSG00000168781	*PPIP5K1*	0.65949413	0.101624576	-0.0963621	0.197986676	0.999933887	0.145945892	0.235702209	-0.089756317	0.789080675
ENSG00000166793	*YPEL4*	0.961336558	0.109204843	-0.079626949	0.188831792	0.999933887	-0.871231213	-0.976263114	0.105031901	0.789080675
ENSG00000215424	*MCM3AP-AS1*	0.738562067	-0.071564541	0.170829691	-0.242394232	0.999933887	0.133100956	0.226435734	-0.093334778	0.789080675
ENSG00000086065	*CHMP5*	5.505707414	0.013654222	0.009846365	0.003807857	0.999933887	0.063787047	0.099734554	-0.035947507	0.789080675
ENSG00000104331	*BPNT2*	3.481339359	6.2674e-05	0.04053017	-0.040467496	0.999933887	-0.256376125	-0.3095479	0.053171774	0.789080675
ENSG00000136826	*KLF4*	5.171002304	0.208727129	0.242373532	-0.033646403	0.999933887	-0.48069985	-0.405728516	-0.074971335	0.789189009
ENSG00000286813	*AC015871.6*	2.012046662	0.109705053	0.278992701	-0.169287648	0.999933887	0.534805281	0.633372792	-0.098567511	0.789189009
ENSG00000241127	*YAE1*	-1.226930791	-0.083549903	-0.370926059	0.287376156	0.999933887	-0.123064114	0.045559129	-0.168623243	0.789309565
ENSG00000158023	*CFAP251*	-0.249726796	0.237168451	-0.194253352	0.431421803	0.999933887	-0.401620159	-0.241727266	-0.159892893	0.789385287
ENSG00000114656	*CFAP92*	3.323893374	0.05741192	0.096331298	-0.038919379	0.999933887	0.127498632	0.073191613	0.054307019	0.789385287
ENSG00000245213	*AC105285.1*	0.062114374	0.040447363	0.263051534	-0.222604171	0.999933887	0.203017611	0.07202486	0.130992751	0.789385287
ENSG00000269821	*KCNQ1OT1*	3.567711747	0.123533804	0.182449507	-0.058915703	0.999933887	0.112607644	0.015506584	0.097101061	0.789601773
ENSG00000170482	*SLC23A1*	0.499761503	-0.236392259	-0.016823317	-0.219568942	0.999933887	-0.137657898	-0.252879909	0.11522201	0.789655594
ENSG00000146007	*ZMAT2*	5.97603116	-0.08763341	-0.115236292	0.027602882	0.999933887	-0.053385573	-0.009099417	-0.044286156	0.789655594
ENSG00000148344	*PTGES*	-0.848622849	-0.314257002	-0.116814386	-0.197442616	0.999933887	3.145966697	2.912726654	0.233240043	0.789821856
ENSG00000159363	*ATP13A2*	3.120604923	-0.027975476	0.014555152	-0.042530628	0.999933887	0.132032161	0.190463554	-0.058431393	0.789821856
ENSG00000232860	*SMG7-AS1*	0.545416954	0.233398673	0.458930741	-0.225532068	0.999933887	1.347153248	1.461435798	-0.11428255	0.789920255
ENSG00000177879	*AP3S1*	5.338434725	-0.016171532	-0.040416652	0.02424512	0.999933887	-0.412248771	-0.45320431	0.040955539	0.789920255
ENSG00000261592	*AC010531.3*	1.913960597	0.222391316	0.301850817	-0.079459501	0.999933887	0.340930001	0.243178395	0.097751606	0.790184597
ENSG00000257267	*ZNF271P*	3.079383411	-0.183545897	-0.114888109	-0.068657787	0.999933887	-0.239308485	-0.298985634	0.059677149	0.79046132
ENSG00000030066	*NUP160*	3.968446988	-0.089102075	0.074982411	-0.164084486	0.970123867	-0.016056246	0.028231507	-0.044287754	0.79057628
ENSG00000092470	*WDR76*	0.222476142	-0.114359065	-0.106952465	-0.007406601	0.999933887	0.101649152	0.000392135	0.101257017	0.79057628
ENSG00000189306	*RRP7A*	3.507612976	0.046999359	0.0334191	0.01358026	0.999933887	-0.028227952	-0.073900234	0.045672282	0.79057628
ENSG00000170962	*PDGFD*	0.842636541	-0.02488813	-0.121454595	0.096566465	0.999933887	-0.183264758	-0.090578591	-0.092686167	0.79057628
ENSG00000229153	*EPHA1-AS1*	-0.100428879	0.157739334	-0.042325436	0.20006477	0.999933887	0.015501488	0.193023244	-0.177521756	0.790627673
ENSG00000239653	*PSMD6-AS2*	1.758354553	-0.131469498	-0.204603677	0.07313418	0.999933887	-0.615691994	-0.764491624	0.14879963	0.790780856
ENSG00000197375	*SLC22A5*	1.5329161	-0.088539658	0.044754613	-0.133294272	0.999933887	0.068253696	0.149005919	-0.080752223	0.790780856
ENSG00000168096	*ANKS3*	1.646062272	0.078645103	0.165091591	-0.086446488	0.999933887	0.045793241	0.118584325	-0.072791084	0.790780856
ENSG00000173660	*UQCRH*	4.003294804	-0.03654163	0.029810477	-0.066352107	0.999933887	0.085408831	0.129319947	-0.043911117	0.790780856
ENSG00000170791	*CHCHD7*	2.801434879	-0.038432386	-0.065038746	0.02660636	0.999933887	0.163707263	0.093676058	0.070031205	0.790780856
ENSG00000234444	*ZNF736*	2.343082558	-0.012470026	0.117644709	-0.130114735	0.999933887	-0.119115419	-0.052718186	-0.066397234	0.790780856
ENSG00000174456	*C12orf76*	2.071158867	0.012555681	0.154447864	-0.141892183	0.999933887	0.049089162	0.125221348	-0.076132186	0.790780856
ENSG00000168924	*LETM1*	3.101735497	0.008460513	-0.03786374	0.046324253	0.999933887	0.102832547	0.047862512	0.054970035	0.790780856
ENSG00000240429	*LRRFIP1P1*	-1.261209313	0.012541199	0.544480127	-0.531938928	0.999933887	-0.128683953	-0.373243149	0.244559196	0.790780856
ENSG00000090266	*NDUFB2*	2.085537104	-0.048211974	0.07050187	-0.118713844	0.999933887	0.049157297	0.109546163	-0.060388866	0.790908938
ENSG00000136463	*TACO1*	1.450528196	-0.102096805	0.043060471	-0.145157276	0.999933887	-0.044102439	-0.138523725	0.094421285	0.790959179
ENSG00000080947	*CROCCP3*	1.777324062	-0.066802174	0.006396048	-0.073198223	0.999933887	0.069784696	0.137891481	-0.068106786	0.790959179
ENSG00000240023	*AL133163.1*	-1.638451977	-0.169151778	0.169089578	-0.338241356	0.999933887	2.290188572	2.520414753	-0.230226181	0.790959179
ENSG00000266232	*MIR3178*	-0.185955935	0.348415118	0.192972503	0.155442616	0.999933887	0.394494673	0.227371048	0.167123625	0.791046379
ENSG00000279861	*AC073548.1*	5.052129707	0.080317353	0.132160591	-0.051843239	0.999933887	0.194278587	0.247462598	-0.053184011	0.791199266
ENSG00000172183	*ISG20*	8.073990997	0.02870254	0.015389659	0.013312881	0.999933887	0.154632301	0.122913834	0.031718467	0.791199266
ENSG00000183690	*EFHC2*	0.879274888	0.002012652	-0.224932916	0.226945568	0.999933887	0.010450324	0.14156808	-0.131117756	0.791199266
ENSG00000213614	*HEXA*	6.076859075	0.008441533	0.07991777	-0.071476237	0.999933887	-0.02179618	0.034904162	-0.056700342	0.79127598
ENSG00000177971	*IMP3*	1.72416057	-0.169144749	0.033469907	-0.202614656	0.999933887	0.080767735	0.148863284	-0.06809555	0.791972687
ENSG00000263826	*AC112907.3*	0.877991006	0.116598583	0.359369494	-0.242770911	0.999933887	0.363903554	0.470548844	-0.10664529	0.791972687
ENSG00000121064	*SCPEP1*	4.601468056	-0.05103277	-0.069175661	0.018142892	0.999933887	-0.525986401	-0.568310286	0.042323885	0.791972687
ENSG00000254428	*AP003392.1*	1.080472439	-0.085039395	-0.007538267	-0.077501128	0.999933887	-0.150979562	-0.060902969	-0.090076594	0.791972687
ENSG00000120647	*CCDC77*	1.04912214	-0.087032135	0.072031944	-0.159064079	0.999933887	-0.333129604	-0.426033119	0.092903515	0.791972687
ENSG00000133169	*BEX1*	0.473831239	-0.240644636	0.073645276	-0.314289912	0.999933887	0.527163833	0.426004171	0.101159662	0.792049449
ENSG00000176438	*SYNE3*	4.814305158	-0.003807213	0.067840525	-0.071647738	0.999933887	-0.05843373	-0.094079299	0.035645569	0.792049449
ENSG00000132466	*ANKRD17*	5.930723809	-0.006586468	0.015285296	-0.021871764	0.999933887	-0.191596473	-0.151685699	-0.039910774	0.792260602
ENSG00000234745	*HLA-B*	13.079654663	0.024812836	0.001453488	0.023359348	0.999933887	0.315819552	0.346492092	-0.03067254	0.792497333
ENSG00000141644	*MBD1*	5.883326724	0.000113108	-0.024490723	0.024603831	0.999933887	-0.061010925	-0.091725715	0.03071479	0.792497333
ENSG00000130956	*HABP4*	1.604300222	-0.07250175	0.100521658	-0.173023408	0.999933887	0.090313906	0.168526225	-0.07821232	0.792672776
ENSG00000157349	*DDX19B*	1.80087464	0.019398668	-0.0039651	0.023363768	0.999933887	-0.053655972	-0.133852619	0.080196647	0.792672776
ENSG00000139372	*TDG*	3.560562204	-0.006622776	0.019427705	-0.026050481	0.999933887	-0.332476118	-0.29285598	-0.039620139	0.792672776
ENSG00000181722	*ZBTB20*	2.372787595	0.051507937	0.09499548	-0.043487543	0.999933887	-0.201777343	-0.064128795	-0.137648548	0.792754982
ENSG00000169021	*UQCRFS1*	4.140479561	-0.016815012	-0.08284358	0.066028568	0.999933887	0.165428503	0.122394256	0.043034247	0.793307038
ENSG00000129757	*CDKN1C*	2.388413596	0.145018348	0.037443628	0.107574721	0.999933887	-0.469143207	-0.564366494	0.095223287	0.793422138
ENSG00000187535	*IFT140*	0.185455434	-0.147108215	0.128923012	-0.276031228	0.999933887	0.096819683	-0.01226502	0.109084703	0.793422138
ENSG00000089280	*FUS*	8.18681964	0.022673186	0.068862155	-0.046188969	0.999933887	0.055929072	0.091330055	-0.035400984	0.793462686
ENSG00000285410	*GABPB1-IT1*	4.792557115	0.07379354	0.10618417	-0.03239063	0.999933887	0.341982908	0.387914953	-0.045932045	0.79349869
ENSG00000258581	*AL157871.3*	1.201404011	-0.083469407	0.132506761	-0.215976168	0.999933887	0.134575744	0.227138198	-0.092562454	0.79349869
ENSG00000287216	*AC106865.2*	-0.315770496	0.090582902	0.202059658	-0.111476755	0.999933887	0.966369227	1.121418118	-0.15504889	0.79349869
ENSG00000196778	*OR52K1*	-0.195031179	0.075674304	0.070004903	0.005669401	0.999933887	-0.460218956	-0.653789772	0.193570816	0.79349869
ENSG00000171621	*SPSB1*	0.919970051	-0.053692595	-0.082295893	0.028603298	0.999933887	0.125031329	0.236525244	-0.111493916	0.79349869
ENSG00000198162	*MAN1A2*	4.012739202	0.124387416	-0.126798911	0.251186327	0.999933887	-0.28267037	-0.358937544	0.076267174	0.793612478
ENSG00000096433	*ITPR3*	4.544745131	0.052753417	0.049415916	0.003337502	0.999933887	0.157078885	0.119013732	0.038065153	0.793765619
ENSG00000106246	*PTCD1*	1.540119347	0.041405588	-0.007424316	0.048829903	0.999933887	0.076158351	0.150032396	-0.073874046	0.793765619
ENSG00000223749	*MIR503HG*	-0.709620187	0.050892415	0.202213975	-0.15132156	0.999933887	-0.396743761	-0.286861666	-0.109882095	0.793979118
ENSG00000073861	*TBX21*	3.830849392	0.003461677	0.023162322	-0.019700645	0.999933887	0.095312213	0.140227307	-0.044915094	0.794249837
ENSG00000135144	*DTX1*	0.073858203	0.050219971	0.351509875	-0.301289905	0.999933887	0.158648902	0.28494433	-0.126295428	0.794302002
ENSG00000170956	*CEACAM3*	6.912031174	0.008925886	0.043595061	-0.034669175	0.999933887	-0.215278895	-0.174007111	-0.041271785	0.794309705
ENSG00000235865	*GSN-AS1*	1.624438556	-0.180365728	-0.002745309	-0.17762042	0.999933887	-0.197681242	-0.122090397	-0.075590845	0.794383629
ENSG00000162882	*HAAO*	0.244376016	0.115493051	0.317702597	-0.202209546	0.999933887	-0.38171788	-0.250632825	-0.131085056	0.794383629
ENSG00000166341	*DCHS1*	0.355649723	-0.11168452	-0.208049019	0.096364499	0.999933887	-0.001587336	0.115114524	-0.11670186	0.794383629
ENSG00000181638	*ZFP41*	-0.055361188	0.112577688	-0.14535952	0.257937208	0.999933887	0.15372756	0.035995616	0.117731944	0.794383629
ENSG00000280303	*ERICD*	-0.216712798	0.084972331	0.228159861	-0.14318753	0.999933887	1.454740859	1.606165824	-0.151424965	0.794383629
ENSG00000165275	*TRMT10B*	1.671397239	-0.0518412	-0.072711181	0.020869982	0.999933887	-0.166783046	-0.233973529	0.067190483	0.794383629
ENSG00000102543	*CDADC1*	3.161789163	-0.024530025	-0.063696287	0.039166262	0.999933887	0.086410823	0.148571168	-0.062160345	0.794383629
ENSG00000164733	*CTSB*	6.940877819	-0.014812258	-0.019232629	0.004420371	0.999933887	-0.118048985	-0.082148276	-0.035900709	0.794383629
ENSG00000124766	*SOX4*	0.046136204	-0.032667987	0.254258044	-0.286926031	0.999933887	-0.135186192	-0.28629414	0.151107948	0.794383629
ENSG00000122376	*SHLD2*	1.957202214	-0.211025104	-0.171750058	-0.039275046	0.999933887	-0.069440216	0.01532353	-0.084763746	0.79465696
ENSG00000260660	*AC113208.3*	2.681106859	0.145966815	0.265468171	-0.119501356	0.999933887	1.072585766	1.175919517	-0.103333751	0.79465696
ENSG00000102804	*TSC22D1*	3.520991129	0.065050461	-0.012739991	0.077790452	0.999933887	0.397759024	0.347992857	0.049766168	0.79465696
ENSG00000205659	*LIN52*	1.090374398	-0.114551455	0.080663704	-0.195215159	0.999933887	-0.113751325	-0.010181521	-0.103569804	0.79465696
ENSG00000173040	*EVC2*	-0.669232116	0.138167523	0.258235816	-0.120068293	0.999933887	0.031860446	0.188063419	-0.156202973	0.79465696
ENSG00000108950	*FAM20A*	1.162816711	-0.093279802	-0.031047392	-0.062232411	0.999933887	-0.164547801	-0.269970985	0.105423184	0.79465696
ENSG00000250903	*GMDS-DT*	0.987603715	0.079261375	-0.03356713	0.112828504	0.999933887	-0.059849055	-0.16952455	0.109675495	0.79465696

ENSG00000228242	*XPC-AS1*	3.542342324	-0.042101265	-0.056835221	0.014733956	0.999933887	-0.839494643	-0.901989205	0.062494562	0.79465696
ENSG00000123989	*CHPF*	0.835937614	0.043137872	-0.093918372	0.137056244	0.999933887	0.08230355	0.190608216	-0.108304666	0.79465696
ENSG00000286885	*AL138824.1*	0.096469295	-0.038149274	0.148602575	-0.186751849	0.999933887	-0.070482954	-0.203643778	0.133160824	0.79465696
ENSG00000233355	*CHRM3-AS2*	2.76761138	-0.002576928	-0.184781441	0.182204513	0.999933887	-0.124321704	-0.058020165	-0.066301539	0.79465696
ENSG00000180917	*CMTR2*	1.834508336	-0.102908139	-0.157195868	0.054287729	0.999933887	0.014446603	0.095747177	-0.081300574	0.794730918
ENSG00000268041	*ERFL*	0.663441861	0.16296102	0.01769428	0.14526674	0.999933887	-0.069921932	0.050440224	-0.120362156	0.795010951
ENSG00000198841	*KTI12*	0.109341202	-0.127496402	0.287255104	-0.414751506	0.999933887	-0.205188945	-0.324444648	0.119255704	0.795010951
ENSG00000160072	*ATAD3B*	3.665347701	0.015850134	0.068109541	-0.052259407	0.999933887	0.13581199	0.078752743	0.057059246	0.795010951
ENSG00000138617	*PARP16*	2.711566917	-0.007207226	-0.006814574	-0.000392652	0.999933887	0.202002171	0.259933134	-0.057930963	0.795010951
ENSG00000176383	*B3GNT4*	-0.803241467	-0.008974068	0.228561101	-0.237535168	0.999933887	0.412513555	0.221567923	0.190945632	0.795010951
ENSG00000229162	*RUNX3-AS1*	-0.745550116	0.333588555	0.092370667	0.241217888	0.999933887	0.019355993	0.175043062	-0.15568707	0.795051595
ENSG00000176349	*AC104129.1*	-0.740358017	0.040556286	0.122316487	-0.081760202	0.999933887	-0.226689071	-0.048699888	-0.177989183	0.795090774
ENSG00000025796	*SEC63*	4.3746354	-0.059382257	-0.161069455	0.101687198	0.999933887	-0.399129323	-0.335708938	-0.063420385	0.795226327
ENSG00000179348	*GATA2*	1.523447875	-0.005080919	0.179225145	-0.184306064	0.999933887	-0.025231162	0.050588552	-0.075819714	0.795226327
ENSG00000166170	*BAG5*	4.649644651	-0.066970761	-0.025650237	-0.041320524	0.999933887	-0.395549863	-0.356096409	-0.039453454	0.795632284
ENSG00000140632	*GLYR1*	5.965152209	-0.035115331	0.014962108	-0.050077439	0.999933887	-0.205580723	-0.235733237	0.030152514	0.795632284
ENSG00000124313	*IQSEC2*	1.652584646	0.061014257	-0.032171536	0.093185793	0.999933887	-0.226894509	-0.143944376	-0.082950133	0.795632284
ENSG00000101400	*SNTA1*	0.416268282	0.038909109	0.197790961	-0.158881852	0.999933887	0.128590274	-0.00573618	0.134326454	0.795632284
ENSG00000277972	*CISD3*	2.212904373	0.117060075	0.019848569	0.097211506	0.999933887	0.216356414	0.160597	0.055759414	0.795799772
ENSG00000144647	*POMGNT2*	0.850301238	-0.067758519	0.054927111	-0.122685629	0.999933887	0.060224551	0.187997917	-0.127773366	0.795799772
ENSG00000214797	*AP002358.1*	-0.853540307	0.064888347	-0.125558147	0.190446494	0.999933887	0.324007156	0.574998267	-0.250991111	0.795799772
ENSG00000165905	*LARGE2*	-0.333908232	0.133231341	0.128376108	0.004855233	0.999933887	-0.075080612	-0.197389842	0.12230923	0.796017408
ENSG00000124549	*BTN2A3P*	0.974628889	0.068227911	0.116502829	-0.048274918	0.999933887	0.166454971	0.077649388	0.088805583	0.796017408
ENSG00000107745	*MICU1*	5.256612376	0.003057183	0.011260046	-0.008202863	0.999933887	0.111871633	0.150877359	-0.039005726	0.796017408
ENSG00000181610	*MRPS23*	1.377260271	0.018958221	-0.163134168	0.18209239	0.999933887	0.005289899	0.085211972	-0.079922073	0.796261006
ENSG00000152102	*FAM168B*	6.737737009	-0.07943005	-0.039942095	-0.039487955	0.999933887	0.007145663	0.036000051	-0.028854388	0.796264074
ENSG00000259950	*AC034105.3*	1.993742778	0.190344853	0.450634439	-0.260289585	0.999933887	0.893203004	1.00811459	-0.114911586	0.796264074
ENSG00000185236	*RAB11B*	5.967949109	0.044681501	0.032471709	0.012209792	0.999933887	-0.164820975	-0.200030757	0.035209782	0.796264074
ENSG00000285053	*AL357556.3*	-0.284204345	-0.146040846	-0.19835374	0.052312893	0.999933887	-0.128863343	-0.25436676	0.125503417	0.796264074
ENSG00000119969	*HELLS*	-0.091193518	0.102226582	0.034471334	0.067755248	0.999933887	-0.140202384	-0.007837053	-0.132365331	0.796264074
ENSG00000235499	*AC073046.1*	3.463376716	0.052239276	0.103063442	-0.050824166	0.999933887	0.641508314	0.694860221	-0.053351907	0.796264074
ENSG00000187866	*FAM122A*	2.65862696	0.019853873	-0.055328442	0.075182315	0.999933887	-0.164856528	-0.23108898	0.066232452	0.796264074
ENSG00000197165	*SULT1A2*	0.261253065	-0.270344743	-0.382693144	0.112348401	0.999933887	-0.943102514	-1.048938916	0.105836402	0.796534478
ENSG00000132356	*PRKAA1*	5.163624479	-0.017325676	-0.060081576	0.042755899	0.999933887	-0.403748473	-0.350177009	-0.053571464	0.796534478
ENSG00000162889	*MAPKAPK2*	9.225812742	0.007329771	-0.004481636	0.011811407	0.999933887	0.405158117	0.436326436	-0.031168319	0.796534478
ENSG00000106524	*ANKMY2*	1.711134212	-0.099935657	0.047686785	-0.147622443	0.999933887	0.06930829	-0.013798824	0.083107113	0.796760477
ENSG00000121152	*NCAPH*	0.256104232	-0.142315085	-0.162371538	0.020056452	0.999933887	0.212068341	0.317988649	-0.105920307	0.796898547
ENSG00000132470	*ITGB4*	2.913020037	0.07115559	0.097598767	-0.026443177	0.999933887	-0.085080753	-0.161023108	0.075942355	0.796898547
ENSG00000273136	*NBPF26*	4.927266504	-0.102688589	-0.050441798	-0.052246792	0.999933887	0.391367094	0.31320632	0.078160775	0.796898547
ENSG00000115112	*TFCP2L1*	-0.145338822	-0.086139682	0.236759297	-0.322898978	0.999933887	-0.173358808	-0.03699706	-0.136361748	0.796898547
ENSG00000163029	*SMC6*	2.391483025	-0.058214203	-0.151047055	0.092832852	0.999933887	-0.201387756	-0.089016884	-0.112370871	0.796898547
ENSG00000148843	*PDCD11*	3.11600284	0.028230935	-0.016551043	0.044781978	0.999933887	0.160633414	0.112084228	0.048549186	0.796898547
ENSG00000153187	*HNRNPU*	8.6420907	-0.014248621	-0.008711234	-0.005537388	0.999933887	-0.003852999	0.024773835	-0.028626834	0.796898547
ENSG00000053702	*NRIP2*	0.662515419	-0.018637378	0.16606453	-0.184701908	0.999933887	0.021445979	-0.080326188	0.101772167	0.796898547
ENSG00000137168	*PPIL1*	0.357970626	-0.019305844	0.029554955	-0.048860798	0.999933887	0.205281394	0.097736531	0.107544863	0.796898547
ENSG00000180423	*HARBI1*	1.08720686	-0.010019785	-0.121437721	0.111417936	0.999933887	0.310069248	0.226955422	0.083113826	0.796898547
ENSG00000283041	*AC008038.1*	0.263631194	-0.117499715	-0.009939627	-0.107560088	0.999933887	0.17202982	0.279697563	-0.107667743	0.797042916
ENSG00000100445	*SDR39U1*	1.445475313	0.051466016	0.074919292	-0.023453275	0.999933887	0.154779916	0.072297513	0.082482402	0.797042916
ENSG00000107263	*RAPGEF1*	8.874505048	0.004047904	0.019934043	-0.015886138	0.999933887	0.0356215	0.066774565	-0.031153065	0.797042916
ENSG00000235194	*PPP1R3E*	4.933549508	0.033032699	0.10590644	-0.072873741	0.999933887	0.190617045	0.237414331	-0.046797287	0.797148008
ENSG00000140463	*BBS4*	2.584944905	-0.070232245	0.031128455	-0.101360701	0.999933887	-0.000753742	-0.051237533	0.050483791	0.797325512
ENSG00000285518	*AC004900.1*	-1.203626724	-0.172986156	-0.082054375	-0.090931781	0.999933887	0.879712215	1.101384109	-0.221671893	0.797619749
ENSG00000136717	*BIN1*	4.382659086	0.049637412	0.020530534	0.029106879	0.999933887	0.050462173	0.004056071	0.046406102	0.797715008
ENSG00000107223	*EDF1*	5.317514901	0.041599816	0.047383381	-0.005783565	0.999933887	0.095628852	0.051394099	0.044234752	0.797715008
ENSG00000198589	*LRBA*	3.706998059	-0.041871558	-0.013042071	-0.028829487	0.999933887	-0.051536289	0.013381346	-0.064917636	0.797715008
ENSG00000163508	*EOMES*	3.297644333	-0.026223698	-0.063143553	0.036919855	0.999933887	0.185573934	0.135817395	0.049756538	0.797715008
ENSG00000164031	*DNAJB14*	4.33078675	-0.03994773	-0.203627142	0.163679412	0.999933887	-0.799247182	-0.694480688	-0.104766494	0.797715008
ENSG00000129824	*RPS4Y1*	-2.454854904	-0.020214717	-0.165283911	0.145069194	0.999933887	-0.084762244	-0.15313365	0.068371406	0.797779716
ENSG00000139914	*FITM1*	0.168451381	0.359851121	-0.289251245	0.649102366	0.144167088	-0.106044461	-0.209757578	0.103713117	0.79779194
ENSG00000132768	*DPH2*	1.621557607	0.10459765	-0.156980453	0.261578104	0.999933887	0.278430265	0.20962986	0.068800405	0.797823174
ENSG00000272555	*AC009974.1*	-0.875105249	-0.19528304	-0.476641723	0.281358683	0.999933887	0.619991936	0.756320201	-0.136328265	0.797823174
ENSG00000255808	*PDE2A-AS1*	-0.762401867	0.128147872	0.043282517	0.084865355	0.999933887	0.575817122	0.830365162	-0.25454804	0.797823174
ENSG00000165494	*PCF11*	7.975816777	0.042152328	-0.066906441	0.109058769	0.999933887	0.264247502	0.309394582	-0.04514708	0.798045325
ENSG00000169057	*MECP2*	7.623209663	0.023656779	0.027798814	-0.004142035	0.999933887	0.129968758	0.163175954	-0.033207196	0.798045325
ENSG00000139679	*LPAR6*	3.347543067	-0.107119668	-0.137244627	0.03012496	0.999933887	-0.323720595	-0.394313728	0.070593133	0.798644963
ENSG00000231889	*TRAF3IP2-AS*	2.049636921	0.066432618	-0.035670647	0.102103265	0.999933887	0.80829399	0.88003202	-0.071738031	0.798644963
ENSG00000143502	*SUSD4*	-0.876690097	0.077763052	-0.149179955	0.226943007	0.999933887	0.091578183	0.239952837	-0.148374654	0.798644963
ENSG00000167700	*MFSD3*	0.650101757	0.042673707	-0.034464516	0.077138223	0.999933887	0.344284165	0.239680208	0.104603957	0.798644963
ENSG00000280135	*AL096816.1*	2.103075187	-0.062071668	-0.083957168	0.0218855	0.999933887	0.000240689	-0.068060448	0.068301137	0.798964436
ENSG00000204685	*STARD7-AS1*	3.371594517	-0.078481539	0.014136386	-0.092617925	0.999933887	0.123963927	0.060684096	0.063279831	0.799190091
ENSG00000126903	*SLC10A3*	4.726698181	0.051898132	-0.025509774	0.077407906	0.999933887	-0.160531965	-0.206081103	0.045549138	0.799197405
ENSG00000186197	*EDARADD*	-0.310783908	-0.018380766	0.046341547	-0.064722314	0.999933887	0.144032967	0.287458833	-0.143425866	0.799197405
ENSG00000112195	*TREML2*	6.815750088	0.005263083	0.010910233	-0.00564715	0.999933887	-0.263848867	-0.210800343	-0.053048524	0.799197405
ENSG00000196497	*IPO4*	0.73136121	0.291164384	0.195359097	0.095805286	0.999933887	0.336141227	0.208960676	0.127180551	0.799352075
ENSG00000128228	*SDF2L1*	2.478198821	0.168863442	0.154132752	0.01473069	0.999933887	0.187446425	0.117101496	0.070344928	0.799352075
ENSG00000186594	*MIR22HG*	7.396713444	0.111451515	0.073359857	0.038091658	0.999933887	0.416313711	0.458533989	-0.042220278	0.799352075
ENSG00000183207	*RUVBL2*	2.312842876	0.106870083	0.020874214	0.085995869	0.999933887	0.117006643	0.054018692	0.062987951	0.799352075
ENSG00000111203	*ITFG2*	2.739221673	-0.039386405	0.126229592	-0.165615997	0.999933887	-0.052213117	0.003237636	-0.055450752	0.799352075
ENSG00000089009	*RPL6*	6.928605291	-0.031314704	-0.151013162	0.119698458	0.999933887	0.044028611	0.085618473	-0.041589863	0.799352075
ENSG00000186162	*CIDECP1*	3.420947627	0.035933386	0.083810506	-0.04787712	0.999933887	-0.128551368	-0.070483502	-0.058067866	0.799352075
ENSG00000114686	*MRPL3*	1.939092697	0.02305923	-0.133505933	0.156565163	0.999933887	0.170076833	0.235716469	-0.065639636	0.799352075
ENSG00000166326	*TRIM44*	4.03437459	-0.012504719	-0.122552981	0.110048262	0.999933887	0.024169715	0.066211066	-0.042041351	0.799352075
ENSG00000276045	*ORAI1*	4.648460775	0.070788705	-0.023857477	0.094646182	0.999933887	0.008268477	-0.038392591	0.046661068	0.799476086
ENSG00000213799	*ZNF845*	1.942762236	-0.155168019	-0.275538129	0.12037011	0.999933887	-0.580183926	-0.484795755	-0.095388171	0.799476086
ENSG00000132676	*DAP3*	4.682122692	-0.063682689	0.022448467	-0.086131156	0.999933887	0.018228453	0.050846354	-0.032617902	0.799490775
ENSG00000280780	*JAKMIP2-AS1*	-2.324042754	-0.628539764	-0.457838018	-0.170701747	0.999933887	-0.764288943	-0.62011899	-0.144169953	0.799490775
ENSG00000214756	*CSKMT*	0.178344712	0.156224331	0.504261563	-0.348037232	0.999933887	0.3637579	0.47469915	-0.11094125	0.799490775
ENSG00000141698	*NT5C3B*	1.149186335	0.084239712	0.124806979	-0.040567267	0.999933887	0.168059483	0.245737884	-0.077678401	0.799490775
ENSG00000142534	*RPS11*	8.636852969	0.025609042	-0.027754103	0.053363145	0.999933887	0.074690927	0.111165961	-0.036475034	0.799490775
ENSG00000279529	*AC008764.8*	1.739199116	-0.020165694	-0.060363229	0.040197536	0.999933887	0.031153808	0.112301288	-0.08114748	0.799490775
ENSG00000182866	*LCK*	5.414612611	0.070731556	-0.003568474	0.07430003	0.999933887	0.096866932	0.058547331	0.038319601	0.799557281
ENSG00000183354	*KIAA2026*	5.052505068	0.175315176	0.00560386	0.169711317	0.999933887	-0.619366036	-0.530401191	-0.088964845	0.799658864
ENSG00000197780	*TAF13*	4.048336812	0.026962337	0.206956968	-0.179994631	0.944094537	0.538254307	0.587462801	-0.049208494	0.799833667
ENSG00000171962	*DRC3*	0.017493355	-0.132289593	-0.183239468	0.050949875	0.999933887	-0.077171317	0.062897379	-0.140068697	0.799890127
ENSG00000072195	*SPEG*	0.33768812	0.073624851	0.239024563	-0.165399712	0.999933887	0.025900138	-0.088153003	0.114053141	0.799890127
ENSG00000168056	*LTBP3*	4.364003824	-0.001820862	0.061338344	-0.063159207	0.999933887	0.11934603	0.072698641	0.046647389	0.80022529
ENSG00000176986	*SEC24C*	5.442945645	0.00044428	0.032601723	-0.032157443	0.999933887	-0.063290283	-0.031707678	-0.031582606	0.8002938
ENSG00000196531	*NACA*	6.743507847	-0.108202808	-0.180801906	0.072599098	0.999933887	-0.163962581	-0.1985381	0.034575519	0.800304159
ENSG00000261253	*AC137932.2*	-0.247733348	0.370312993	0.143067728	0.227245264	0.999933887	-0.138325136	-0.314588119	0.176262983	0.800304159
ENSG00000163069	*SGCB*	-0.41044152	0.196599681	-0.159566782	0.356166463	0.999933887	-0.182941677	-0.036797939	-0.146143738	0.800304159
ENSG00000236901	*MIR600HG*	0.682626667	-0.178394015	-0.044759124	-0.133634891	0.999933887	-0.397664673	-0.498205228	0.100540555	0.800304159
ENSG00000115750	*TAF1B*	0.817798069	-0.114718363	-0.31926768	0.204549317	0.999933887	-0.084623671	0.018161201	-0.102784872	0.800304159
ENSG00000135763	*URB2*	1.50465418	-0.070483465	0.107778277	-0.178261742	0.999933887	0.23108886	0.165393806	0.065695054	0.800304159
ENSG00000137959	*IFI44L*	4.524428345	-0.086330216	-0.214030568	0.127700352	0.999933887	-0.387536269	-0.477485712	0.089949442	0.800304159
ENSG00000276136	*AC016957.2*	3.000436792	-0.059006043	0.027991942	-0.086997985	0.999933887	-0.221149294	-0.291832164	0.07068287	0.800304159

ENSG00000133026	*MYH10*	3.749588668	0.047105701	-0.019830117	0.066935818	0.999933887	-0.029853295	-0.104128837	0.074275542	0.800304159
ENSG00000113758	*DBN1*	4.576108347	0.037470821	0.08373509	-0.046264269	0.999933887	0.41569542	0.458585831	-0.042890411	0.800304159
ENSG00000127952	*STYXL1*	2.849619748	0.043236075	-0.038462934	0.081699009	0.999933887	0.025207575	0.074164277	-0.048956702	0.800304159
ENSG00000179262	*RAD23A*	4.634916244	-0.029034838	0.028940498	-0.057975336	0.999933887	-0.111254214	-0.146567097	0.035312883	0.800304159
ENSG00000286159	*AL035106.1*	3.232844788	-0.030003561	-0.075036529	0.045032968	0.999933887	-0.097553182	-0.028393135	-0.069160047	0.800304159
ENSG00000260231	*KDM7A-DT*	1.966715316	0.030946426	0.155625418	-0.124678992	0.999933887	0.366399494	0.262814072	0.103585421	0.800304159
ENSG00000167286	*CD3D*	4.945029344	0.009388365	-0.027545152	0.036933517	0.999933887	0.12778374	0.168576312	-0.040792572	0.800304159
ENSG00000108433	*GOSR2*	2.964396376	0.010622982	-0.017042424	0.027665407	0.999933887	0.058536677	0.107326171	-0.048789494	0.800304159
ENSG00000080200	*CRYBG3*	-0.506010553	0.00693584	0.034061153	-0.027125314	0.999933887	-0.196377609	0.003046294	-0.199423903	0.800304159
ENSG00000279425	*AC092279.2*	1.232387281	-0.002987763	0.13980243	-0.142790193	0.999933887	-0.481258507	-0.571528085	0.090269578	0.800304159
ENSG00000258521	*AL157871.2*	0.972078824	-0.0014196	-0.040365121	0.03894552	0.999933887	0.168584934	0.282470129	-0.113885195	0.800304159
ENSG00000126458	*RRAS*	0.751082696	0.062550825	0.025526202	0.037024623	0.999933887	-0.133980472	-0.023037963	-0.110942509	0.800326581
ENSG00000138801	*PAPSS1*	3.619233073	0.002834554	-0.09362674	0.096461293	0.999933887	-0.173173485	-0.124571717	-0.048601768	0.800329721
ENSG00000272150	*NBPF25P*	-0.831798722	-0.069442246	-0.459299053	0.389856807	0.999933887	0.341292743	0.191108038	0.150184705	0.800797278
ENSG00000105197	*TIMM50*	2.701888098	0.063293047	0.099237565	-0.035944518	0.999933887	0.099646089	0.160742563	-0.061096474	0.800843298
ENSG00000134186	*PRPF38B*	6.503823642	0.004382404	-0.067749692	0.072132095	0.999933887	-0.047780315	0.011690826	-0.05947114	0.800843298
ENSG00000095383	*TBC1D2*	7.923640771	-0.076342665	-0.068158894	-0.008183771	0.999933887	-0.422179856	-0.371495202	-0.050684653	0.801168876
ENSG00000088766	*CRLS1*	-0.609049975	0.1540938	0.161480315	-0.007386516	0.999933887	-0.090355537	0.088268695	-0.178624232	0.801168876
ENSG00000188536	*HBA2*	5.679225274	-0.067999437	-0.140515965	0.072516529	0.999933887	0.025335023	0.095693776	-0.070358753	0.801168876
ENSG00000204315	*FKBPL*	0.335969345	0.077758452	-0.086442904	0.164201357	0.999933887	-0.334164826	-0.451583579	0.117418753	0.801186071
ENSG00000241721	*SUMO1P1*	2.925022084	-0.018067981	0.038107032	-0.056175013	0.999933887	0.015512982	-0.046729105	0.062242087	0.801300381
ENSG00000205060	*SLC35B4*	1.250462733	-0.132146341	0.048816886	-0.180963227	0.999933887	-0.156736826	-0.07242811	-0.084308716	0.801737273
ENSG00000188647	*PTAR1*	5.09391902	-0.011220974	-0.060086035	0.048865061	0.999933887	-0.543234107	-0.46798611	-0.075247997	0.801737273
ENSG00000166165	*CKB*	1.478152602	0.119415579	-0.03794057	0.157356149	0.999933887	0.911443762	1.025038316	-0.113594554	0.801777601
ENSG00000107819	*SFXN3*	4.753829175	0.037205116	0.011073019	0.026132097	0.999933887	-0.141940645	-0.180545804	0.03860516	0.801777601
ENSG00000151135	*TMEM263*	2.060826642	-0.058500523	-0.171204943	0.11270442	0.999933887	-0.073708395	-0.000699533	-0.073008861	0.801777601
ENSG00000131495	*NDUFA2*	3.456929826	-0.004652208	0.001480913	-0.006133121	0.999933887	-0.135666063	-0.184681385	0.049015322	0.801777601
ENSG00000138231	*DBR1*	1.998013577	-0.00294137	-0.17672076	0.17377939	0.999933887	-0.139728356	-0.21210646	0.072378104	0.801777601
ENSG00000168818	*STX18*	2.576667382	-0.088059668	0.157505407	-0.245565075	0.944094537	0.059373679	0.103026572	-0.043652893	0.801835092
ENSG00000274767	*AC243829.1*	4.06386388	-0.037413043	-0.095327829	0.057914786	0.999933887	0.027983902	-0.022578175	0.050562077	0.801835092
ENSG00000153879	*CEBPG*	3.118519332	-0.028830293	-0.001255585	-0.027574708	0.999933887	0.003843561	0.050261402	-0.04641784	0.801835092
ENSG00000084072	*PPIE*	2.784355735	-0.031920226	0.00334088	-0.035261106	0.999933887	0.097096638	0.041432757	0.055663882	0.801835092
ENSG00000181396	*OGFOD3*	1.538768594	0.013938184	-0.00170415	0.015642334	0.999933887	0.032048318	0.118747821	-0.086699503	0.801835092
ENSG00000131165	*CHMP1A*	7.103806118	-0.00088213	0.021579402	-0.022461532	0.999933887	0.053952034	0.088566449	-0.034614415	0.801835092
ENSG00000153944	*MSI2*	4.260219879	0.030490111	0.085616847	-0.055126735	0.999933887	-0.069024097	-0.106860323	0.037836226	0.801839106
ENSG00000071462	*BUD23*	4.119081051	0.002173171	-0.033024681	0.035197851	0.999933887	0.157460902	0.122590277	0.034870624	0.801839106
ENSG00000138002	*IFT172*	1.482954191	0.015810601	0.009883064	0.005927537	0.999933887	-0.150533622	-0.080253988	-0.070279634	0.802371996
ENSG00000170486	*KRT72*	-1.181968495	0.025846118	-0.0659516	0.091797718	0.999933887	-0.104545894	0.045414759	-0.149960653	0.802485978
ENSG00000176593	*AC008969.1*	1.161620698	-0.163383248	-0.146901608	-0.016481639	0.999933887	-0.124810131	-0.049076789	-0.075733342	0.802495662
ENSG00000117899	*MESD*	2.641688644	-0.064839482	-0.158053499	0.093214017	0.999933887	0.015581938	-0.05274727	0.068329208	0.802495662
ENSG00000218073	*AL021407.2*	0.650703239	0.030714835	-0.01696063	0.047675465	0.999933887	0.244034773	0.391629121	-0.147594347	0.802576406
ENSG00000198835	*GJC2*	-0.191633519	0.10974168	0.25347431	-0.143732629	0.999933887	0.247662721	0.429737055	-0.182074334	0.802650045
ENSG00000204130	*RUFY2*	2.265225527	-0.104031399	-0.083427203	-0.020604196	0.999933887	-0.535647456	-0.453449045	-0.082198411	0.802736683
ENSG00000113522	*RAD50*	-0.828532928	0.156392034	0.094953016	0.061439018	0.999933887	-0.002391096	-0.15485121	0.152460115	0.802736683
ENSG00000141378	*PTRH2*	2.557558412	-0.070562729	-0.02539005	-0.04517268	0.999933887	-0.123677485	-0.188537096	0.064859611	0.802875477
ENSG00000185808	*PIGP*	-0.020403719	0.069826747	0.00743846	0.062388287	0.999933887	0.093492922	-0.025745255	0.119238177	0.802875477
ENSG00000276523	*AC025287.3*	3.613872239	-0.00699357	0.111129427	-0.118122997	0.999933887	0.830828148	0.890771028	-0.05994288	0.802932166
ENSG00000036448	*MYOM2*	1.214913836	-0.095264783	-0.032180395	-0.063084388	0.999933887	0.256077299	0.151297538	0.104779761	0.803029896
ENSG00000134970	*TMED7*	3.914811187	-0.085813675	0.028202688	-0.114016363	0.999933887	-0.672191308	-0.729350348	0.05715904	0.803529217
ENSG00000099940	*SNAP29*	4.075034076	-0.011704866	0.002003723	-0.013708589	0.999933887	-0.450254697	-0.493813862	0.043559165	0.803529217
ENSG00000279865	*AC006511.3*	0.540769327	0.110726341	0.212955737	-0.102229396	0.999933887	1.41545194	1.308444136	0.107007804	0.803637186
ENSG00000196549	*MME*	7.310883757	-0.031985534	-0.144317651	0.112332116	0.999933887	-0.920323549	-0.978873952	0.058550403	0.803860469
ENSG00000260097	*SPDYE6*	0.411560175	-0.186931053	-0.037839668	-0.149091385	0.999933887	0.126495016	0.22877071	-0.102275693	0.804000145
ENSG00000143257	*NR1I3*	2.474798192	0.09057824	0.095384624	-0.004806384	0.999933887	1.32328409	1.407215591	-0.083931501	0.804000145
ENSG00000094631	*HDAC6*	4.258603442	0.021591416	0.114713522	-0.093122106	0.999933887	0.179780378	0.224760605	-0.044980227	0.804000145
ENSG00000274114	*ALOX15P1*	-0.74415804	0.026609981	-0.164646312	0.191256294	0.999933887	-0.129703114	-0.296688734	0.16698562	0.804000145
ENSG00000274750	*H3C6*	-1.203829502	0.334851885	-0.418836048	0.753687932	0.999933887	-0.129432317	-0.273699095	0.144266778	0.804052803
ENSG00000144591	*GMPPA*	2.664262769	0.028785048	-0.005969031	0.034754079	0.999933887	-0.007070065	-0.058187299	0.051117234	0.804070072
ENSG00000002016	*RAD52*	1.612995809	0.008757011	0.004953025	0.003803986	0.999933887	-0.041924068	-0.123768835	0.081844767	0.804070072
ENSG00000112782	*CLIC5*	0.193593642	-0.048242373	0.065139054	-0.113381426	0.999933887	-0.029149483	-0.127995438	0.098845955	0.804092021
ENSG00000177706	*FAM20C*	1.340903292	0.000491198	0.034800439	-0.034309241	0.999933887	-0.578023756	-0.476787134	-0.101236621	0.804092021
ENSG00000171105	*INSR*	0.755927956	-0.117192557	0.133148724	-0.250341281	0.999933887	-0.731672584	-0.610635334	-0.12103725	0.804139901
ENSG00000174032	*SLC25A30*	2.677625215	-0.030249921	-0.019417983	-0.010831938	0.999933887	-0.167726432	-0.109345582	-0.05838085	0.804139901
ENSG00000067365	*METTL22*	4.686384042	0.065745428	0.051096289	0.014649139	0.999933887	-0.145753098	-0.184846794	0.039093695	0.804548534
ENSG00000170092	*SPDYE5*	-0.328313697	-0.134566329	0.167933005	-0.302499334	0.999933887	-0.287366083	-0.418740585	0.131374502	0.804548534
ENSG00000089123	*TASP1*	0.751899011	0.050918045	0.162616808	-0.111698763	0.999933887	0.01386824	0.092206206	-0.078337966	0.804548534
ENSG00000128482	*RNF112*	-0.327336291	0.019942836	0.20565028	-0.185707444	0.999933887	0.173667192	0.322390597	-0.148723405	0.804548534
ENSG00000104921	*FCER2*	0.89785308	0.054757632	0.083531701	-0.028774069	0.999933887	0.536847772	0.449615962	0.08723181	0.804620515
ENSG00000107611	*CUBN*	1.098358086	0.037797088	0.130704535	-0.092907447	0.999933887	-0.220003507	-0.323064602	0.103061095	0.804620515
ENSG00000274828	*AC068473.5*	0.764323158	0.079723303	0.439039111	-0.359315807	0.999933887	-0.153693942	-0.224755584	0.071061642	0.805055589
ENSG00000204420	*MPIG6B*	0.597119478	0.242223871	0.140508068	0.101715803	0.999933887	-0.323578823	-0.17741219	-0.146166633	0.805091454
ENSG00000225178	*RPSAP58*	-0.951166883	0.288230515	-0.113731939	0.401962455	0.999933887	0.096193589	0.234580454	-0.138386865	0.805207607
ENSG00000231047	*GCNT1P3*	1.024498557	0.050149682	0.187013301	-0.136863619	0.999933887	0.212101946	0.462088731	-0.249986785	0.805207607
ENSG00000106799	*TGFBR1*	7.861987281	-0.196638936	-0.12348725	-0.073151686	0.999933887	-1.265991805	-1.201060875	-0.06493093	0.805387762
ENSG00000196150	*ZNF250*	2.921595699	0.093085311	0.131040069	-0.037954758	0.999933887	0.388481619	0.470298322	-0.081816703	0.805400084
ENSG00000124574	*ABCC10*	3.454917914	-0.022030138	0.052812626	-0.074842764	0.999933887	0.061375229	0.014535584	0.046839645	0.805400084
ENSG00000174989	*FBXW8*	1.6613679	0.094477331	0.099066541	-0.004589211	0.999933887	0.013931306	-0.060927394	0.074858699	0.805774356
ENSG00000113851	*CRBN*	3.895846634	-0.041549244	-0.110614842	0.069065598	0.999933887	-0.274869971	-0.224298811	-0.050571159	0.805774356
ENSG00000189227	*C15orf61*	-0.592993104	0.025499658	0.018256984	0.007242674	0.999933887	0.042610815	0.200283735	-0.15767292	0.805774356
ENSG00000143799	*PARP1*	4.230665035	0.005696386	-0.02826874	0.033965126	0.999933887	0.105453114	0.144518763	-0.039065648	0.805774356
ENSG00000154305	*MIA3*	5.061085967	0.022796422	-0.065676334	0.088472757	0.999933887	-0.419032534	-0.474447793	0.055415258	0.805808121
ENSG00000268575	*AL031282.2*	0.157775776	0.107046834	0.179539392	-0.072492558	0.999933887	-0.247685522	-0.355569667	0.107884145	0.805811362
ENSG00000256128	*LINC00944*	0.001757547	-0.077687371	0.359819125	-0.437506496	0.999933887	0.199568026	0.09552954	0.104038486	0.805811362
ENSG00000149273	*RPS3*	8.530397117	-0.025433286	-0.050875585	0.025442299	0.999933887	0.080845044	0.043031506	0.037813538	0.805811362
ENSG00000156928	*MALSU1*	1.338246117	0.045762773	0.133215868	-0.087453095	0.999933887	0.134152467	0.040934697	0.093217769	0.805811362
ENSG00000147224	*PRPS1*	3.243039013	0.00941077	-0.017048884	0.026459653	0.999933887	0.093978033	0.1499883	-0.056010267	0.805811362
ENSG00000116221	*MRPL37*	2.102875385	0.002068579	0.027933139	-0.02586456	0.999933887	0.225932505	0.159936791	0.065995714	0.806223066
ENSG00000103355	*PRSS33*	3.50578363	0.061650143	0.07547267	-0.013822527	0.999933887	-0.028890194	0.026112043	-0.055002237	0.8063262
ENSG00000159228	*CBR1*	-0.12536392	0.087204172	0.156730227	-0.069526055	0.999933887	-0.203660952	-0.318163289	0.114502337	0.8063262
ENSG00000158517	*NCF1*	7.59775237	0.026832459	0.001584652	0.025247807	0.999933887	0.888588638	0.838589155	0.049999483	0.8063262
ENSG00000224546	*EIF4BP3*	-1.24394067	-0.092309285	0.085355464	-0.177664749	0.999933887	0.143803587	0.301115967	-0.157312381	0.80642519
ENSG00000261997	*AC007336.1*	1.774662835	0.002243009	-0.017937884	0.020180893	0.999933887	-0.406412818	-0.268161511	-0.138251308	0.806511713
ENSG00000196458	*ZNF605*	1.124755273	0.051435007	-0.284499891	0.335934897	0.999933887	-0.24346634	-0.331121138	0.087654797	0.806910148
ENSG00000242970	*AC068522.1*	1.905776495	0.309895494	0.429831828	-0.119936334	0.999933887	0.975170273	1.084483995	-0.109313721	0.807003277
ENSG00000213793	*ZNF888*	0.983067572	-0.160930401	-0.04275292	-0.118177481	0.999933887	-0.419150066	-0.320434334	-0.098715732	0.807003277
ENSG00000125845	*BMP2*	1.009468645	0.03983912	0.088153535	-0.048314415	0.999933887	0.855215346	0.773829055	0.081386291	0.807299739
ENSG00000172785	*CBWD1*	1.268767024	-0.155155846	-0.099135754	-0.056020093	0.999933887	-0.200655877	-0.302706196	0.102050319	0.807312114
ENSG00000188997	*KCTD21*	2.158754649	-0.107369749	-0.078340085	-0.029029664	0.999933887	-0.834538525	-0.909427729	0.074889204	0.807312114
ENSG00000189129	*PLAC9*	0.033308883	-0.106263052	0.028720628	-0.13498368	0.999933887	-0.449478205	-0.584982782	0.135504577	0.807312114
ENSG00000167618	*LAIR2*	-1.039736778	-0.102485966	-0.07994095	-0.022545016	0.999933887	0.16540583	0.332968247	-0.167562417	0.807312114
ENSG00000139921	*TMX1*	2.75695553	0.038311797	-0.040302905	0.078614702	0.999933887	-0.148419113	-0.07432714	-0.074091974	0.807312114
ENSG00000137177	*KIF13A*	6.57523732	-0.029992274	-0.034975697	0.004983423	0.999933887	0.290807584	0.356140065	-0.06533248	0.807312114
ENSG00000286646	*AL121933.2*	1.869413209	-0.025386877	0.009415226	-0.034802103	0.999933887	0.472426691	0.403608529	0.068818161	0.807312114
ENSG00000223960	*CHROMR*	1.165948062	0.001612777	-0.187438478	0.189051255	0.999933887	-0.194595627	-0.270044515	0.075448887	0.807312114
ENSG00000150455	*TIRAP*	1.687777367	0.040945646	-0.138821384	0.17976703	0.999933887	0.121632618	0.203052686	-0.081420069	0.807379118

ENSG00000274370	*AC130371.2*	1.903376903	-0.027639424	0.183620128	-0.211259552	0.999933887	-0.158866324	-0.240554172	0.081687848	0.807379118
ENSG00000204267	*TAP2*	5.998480388	0.035022368	0.026029334	0.008993034	0.999933887	-0.034612075	-0.066186603	0.031574528	0.80744505
ENSG00000082515	*MRPL22*	1.362516954	0.013284713	-0.108092913	0.121377626	0.999933887	0.109309052	0.030907067	0.078401985	0.80750109
ENSG00000264853	*AC011933.2*	0.763512965	-0.185221489	0.027972096	-0.213193586	0.999933887	0.705961286	0.537396247	0.168565039	0.807862422
ENSG00000260285	*AL133367.1*	0.771022374	-0.106041226	-0.031432357	-0.074608869	0.999933887	-0.615793657	-0.530496324	-0.085297332	0.80787768
ENSG00000022267	*FHL1*	0.864788164	-0.02081366	0.077641544	-0.098455204	0.999933887	0.054852436	-0.047351699	0.102204135	0.807947581
ENSG00000108963	*DPH1*	2.148060367	0.003779077	0.052160143	-0.048381066	0.999933887	0.146822564	0.215767163	-0.068944599	0.80800488
ENSG00000105655	*ISYNA1*	2.935514982	0.119109402	0.127351167	-0.008241765	0.999933887	0.131024278	0.184261987	-0.053237708	0.808121806
ENSG00000124370	*MCEE*	0.07057171	-0.282059687	-0.208018817	-0.07404087	0.999933887	0.125268477	0.031900407	0.09336807	0.808121806
ENSG00000125257	*ABCC4*	1.232761067	-0.058185228	-0.250319826	0.192134598	0.999933887	-0.036701842	0.030608897	-0.067310739	0.808121806
ENSG00000178409	*BEND3*	0.081948752	0.030295063	0.085342817	-0.055047754	0.999933887	0.375672255	0.254409407	0.121262848	0.808121806
ENSG00000186300	*ZNF555*	2.401148685	-0.095126167	-0.011097562	-0.084028605	0.999933887	0.345041281	0.432584206	-0.087542925	0.808232714
ENSG00000145979	*TBC1D7*	6.584603205	0.017037108	0.053339122	-0.036302014	0.999933887	0.599338347	0.646875434	-0.047537087	0.808232714
ENSG00000102010	*BMX*	1.302539121	-0.019429867	-0.098711542	0.079281674	0.999933887	-0.877500679	-0.765529791	-0.111970888	0.808232714
ENSG00000133103	*COG6*	1.146061356	0.01211883	-0.007822632	0.019941462	0.999933887	-0.013166012	-0.11123909	0.098073077	0.808232714
ENSG00000103671	*TRIP4*	3.384753885	-0.001054867	-0.104784188	0.103729321	0.999933887	-0.141262136	-0.180731537	0.0394694	0.808232714
ENSG00000099797	*TECR*	3.810072559	0.075555439	0.033628821	0.041926619	0.999933887	0.165713241	0.209067397	-0.043354156	0.808241121
ENSG00000279584	*AC005593.1*	0.697344237	0.277484484	0.149800957	0.127683527	0.999933887	0.44242029	0.55611058	-0.11369029	0.808293697
ENSG00000147130	*ZMYM3*	2.234023401	-0.053623893	0.060121271	-0.113745165	0.999933887	0.039242219	-0.014467209	0.053709427	0.808293697
ENSG00000276168	*RN7SL1*	4.704535152	-0.179411901	-0.63527159	0.45585969	0.999933887	0.649505627	0.389223017	0.260282609	0.808351057
ENSG00000171115	*GIMAP8*	2.522939599	-0.084572122	-0.062474323	-0.022097799	0.999933887	0.143018583	0.19983849	-0.056819907	0.808602118
ENSG00000167394	*ZNF668*	2.92806386	0.064928209	0.065988429	-0.00106022	0.999933887	0.006645606	0.067676483	-0.061030878	0.808721468
ENSG00000196290	*NIF3L1*	0.739163821	-0.066798345	-0.054719618	-0.012078727	0.999933887	0.011296299	-0.07944962	0.090745919	0.808788303
ENSG00000279744	*AC132938.5*	0.480345579	0.060790002	-0.088319085	0.149109087	0.999933887	-0.47710351	-0.595319197	0.118215687	0.808788303
ENSG00000110104	*CCDC86*	0.855971862	-0.070662422	0.064758412	-0.135420834	0.999933887	0.115832006	0.03062739	0.085204615	0.80939566
ENSG00000274922	*AL139384.1*	0.843040502	0.006786029	-0.219557718	0.226343747	0.999933887	-0.005539594	-0.085890275	0.080350681	0.809568273
ENSG00000156531	*PHF6*	1.941406712	-0.146957527	-0.128395415	-0.018562112	0.999933887	-0.048884911	0.027442803	-0.076327715	0.809824182
ENSG00000135469	*COQ10A*	1.457010944	-0.118310883	0.223442005	-0.341752887	0.999933887	0.234794423	0.157303431	0.077490992	0.809824182
ENSG00000156500	*FAM122C*	2.539460529	0.038737406	-0.004624498	0.043361904	0.999933887	-0.086197179	-0.143479177	0.057281999	0.809840761
ENSG00000167815	*PRDX2*	2.351291607	-0.039926315	0.024389218	-0.064315534	0.999933887	0.392349227	0.464050124	-0.071700897	0.809840761
ENSG00000134324	*LPIN1*	4.643974785	-0.019724293	0.012339095	-0.032063388	0.999933887	-0.003132982	0.036124664	-0.039257646	0.809840761
ENSG00000188732	*FAM221A*	-0.546500925	-0.084496102	0.018785868	-0.10328197	0.999933887	-0.231836131	-0.083208016	-0.148628116	0.809886795
ENSG00000284024	*AC069544.2*	3.176139743	-0.013037858	0.016999517	-0.030037375	0.999933887	-0.013266148	0.045585759	-0.058851907	0.809886795
ENSG00000204427	*ABHD16A*	1.950411445	-0.059476114	0.106778626	-0.166254741	0.999933887	-0.456755204	-0.380797183	-0.075958021	0.809978815
ENSG00000129038	*LOXL1*	2.139853006	0.078838958	-0.006535863	0.085374821	0.999933887	0.034021956	0.137790324	-0.103768367	0.810245984
ENSG00000240418	*AC020917.1*	0.484513012	-0.070474887	-0.262384918	0.19191003	0.999933887	0.384963068	0.275996533	0.108966535	0.810277914
ENSG00000158966	*CACHD1*	-0.794631116	0.13367276	0.16519942	-0.03152666	0.999933887	-0.788175532	-0.622186334	-0.165989198	0.810615271
ENSG00000115042	*FAHD2A*	0.916503028	0.011850365	0.067683162	-0.055832797	0.999933887	0.151537157	0.069317497	0.08221966	0.810615271
ENSG00000088053	*GP6*	0.23242254	0.248133802	0.223088558	0.025045244	0.999933887	0.660990972	0.505951183	0.155039789	0.810696005
ENSG00000163002	*NUP35*	-0.087730466	-0.032724522	-0.067556956	0.034832434	0.999933887	0.065950255	0.191920075	-0.12596982	0.810696005
ENSG00000047621	*C12orf4*	1.401145487	0.007365354	-0.191758294	0.199123647	0.999933887	-0.024956023	0.082760053	-0.107716076	0.810867265
ENSG00000162244	*RPL29*	6.866358702	0.074148116	-0.013933252	0.088081367	0.999933887	0.06771682	0.111155332	-0.043438512	0.811387251
ENSG00000152475	*ZNF837*	-1.230797086	0.264054438	0.18413499	0.079919448	0.999933887	0.321245017	0.16489724	0.156347778	0.811387251
ENSG00000189091	*SF3B3*	4.710231523	-0.086862544	-0.011874631	-0.074987913	0.999933887	0.086036519	0.119844664	-0.033808145	0.811405788
ENSG00000100330	*MTMR3*	5.005784822	-0.042178119	-0.001144691	-0.041033428	0.999933887	0.019042142	0.053412959	-0.034370817	0.811405788
ENSG00000138796	*HADH*	1.62190349	-0.074966554	-0.224989283	0.150022729	0.999933887	0.024600285	0.087322421	-0.062722136	0.811405788
ENSG00000152284	*TCF7L1*	-0.209408492	-0.130352823	-0.157597233	0.027244409	0.999933887	-0.594273115	-0.445397759	-0.148875356	0.811405788
ENSG00000124215	*CDH26*	-0.426719905	0.095009383	-0.255233523	0.350242906	0.999933887	-0.20101819	-0.391262803	0.190244613	0.811405788
ENSG00000204628	*RACK1*	7.520050607	-0.011166805	-0.027190968	0.016024163	0.999933887	0.076328259	0.111112765	-0.034784506	0.811405788
ENSG00000134318	*ROCK2*	3.312149276	-0.023664644	0.018971494	-0.042636138	0.999933887	-0.79293711	-0.897629574	0.104692464	0.811405788
ENSG00000158691	*ZSCAN12*	0.305336313	0.063985821	-0.222746142	0.286731963	0.999933887	-0.004867154	0.106507271	-0.111374424	0.811723016
ENSG00000125611	*CHCHD5*	2.613405957	-0.077252286	0.061525825	-0.138778111	0.999933887	-0.262057083	-0.316738072	0.054680989	0.811786829
ENSG00000188290	*HES4*	7.305695269	0.104356167	0.091934446	0.012421721	0.999933887	0.794921342	0.726905191	0.068016151	0.81184016
ENSG00000132361	*CLUH*	3.486450031	0.087312376	0.137764404	-0.050452029	0.999933887	0.260586706	0.212214486	0.04837222	0.811851753
ENSG00000172638	*EFEMP2*	1.8881005	-0.010323124	0.257732475	-0.268055599	0.944094537	-0.211021865	-0.274850557	0.063828692	0.811851753
ENSG00000153563	*CD8A*	6.508228586	-0.001187776	-0.048715236	0.04752746	0.999933887	0.128442906	0.093004592	0.035438314	0.811851753
ENSG00000183011	*NAA38*	3.779924598	0.125530518	0.101838026	0.023692492	0.999933887	0.496651619	0.549413204	-0.052761584	0.811963085
ENSG00000077984	*CST7*	6.989523432	0.014756481	-0.029527218	0.044283699	0.999933887	0.046855343	0.084233894	-0.03737855	0.811963085
ENSG00000163507	*CIP2A*	-0.571606617	-0.018232189	-0.088218905	0.069986716	0.999933887	0.102292297	0.268557767	-0.16626547	0.811963085
ENSG00000168916	*ZNF608*	1.288374149	0.125054957	-0.056590987	0.181645945	0.999933887	-1.133582216	-1.238151227	0.104569011	0.812243027
ENSG00000211787	*TRAV8-3*	-0.263019328	0.082244431	0.149091005	-0.066846574	0.999933887	0.208458553	0.302787734	-0.094329181	0.812258339
ENSG00000279693	*AC099521.2*	-0.021766643	-0.046664464	0.09032245	-0.136986914	0.999933887	-0.285011297	-0.429344609	0.144333312	0.812258339
ENSG00000233006	*MIR3936HG*	-0.27525398	0.018581576	-0.043081422	0.061662999	0.999933887	-0.11702465	0.029050951	-0.146075601	0.812258339
ENSG00000100721	*TCL1A*	-1.414133643	0.096539536	-0.117430811	0.213970347	0.999933887	-0.354411736	-0.476229913	0.121818176	0.812315545
ENSG00000141068	*KSR1*	5.093939182	0.033313043	0.05517583	-0.021862787	0.999933887	0.078361722	0.112356221	-0.033994499	0.812329238
ENSG00000172426	*RSPH9*	0.495020128	-0.080994018	-0.126554289	0.045560271	0.999933887	-0.412434772	-0.522541216	0.110106444	0.812349573
ENSG00000073756	*PTGS2*	8.752734756	0.531502347	0.510523294	0.020979053	0.999933887	1.072217417	1.169077004	-0.096859587	0.812382943
ENSG00000150753	*CCT5*	5.018697174	-0.081241192	-0.02993463	-0.051306562	0.999933887	-0.083241137	-0.115906402	0.032665265	0.812382943
ENSG00000207741	*MIR590*	0.041076788	-0.238537119	-0.256161963	0.017624844	0.999933887	-0.042406643	0.086967146	-0.129373789	0.812382943
ENSG00000117308	*GALE*	2.197864034	0.160381864	-0.178347663	0.338729527	0.944094537	-0.56788722	-0.629982632	0.062095412	0.812382943
ENSG00000116273	*PHF13*	4.453096984	-0.129349454	-0.156939192	0.027589737	0.999933887	-0.615442981	-0.564801321	-0.05064166	0.812382943
ENSG00000087842	*PIR*	-1.424665069	0.393814275	-0.088162827	0.481977103	0.999933887	0.551975517	0.621728485	-0.069752968	0.812382943
ENSG00000280543	*ASAP1-IT2*	1.340972728	0.053126057	0.319678617	-0.26655256	0.999933887	0.372642178	0.290757689	0.081884489	0.812382943
ENSG00000188171	*ZNF626*	-0.329213015	0.09712705	0.338234128	-0.241107077	0.999933887	-0.510339916	-0.380452131	-0.129887786	0.812382943
ENSG00000111885	*MAN1A1*	5.960642011	0.015703157	0.012065557	0.0036376	0.999933887	-0.008769609	0.030058231	-0.03882784	0.812382943
ENSG00000184860	*SDR42E1*	-0.151388892	-0.096508149	0.320138341	-0.41664649	0.999933887	-0.122215718	-0.012671245	-0.109544472	0.812383698
ENSG00000241693	*RN7SL704P*	-0.171591913	0.130743002	0.108073968	0.022669034	0.999933887	0.490008854	0.372777574	0.11723128	0.812383698
ENSG00000102606	*ARHGEF7*	4.757650025	-0.00877421	0.054197255	-0.062971465	0.999933887	-0.016652523	-0.046623646	0.029971123	0.812383698
ENSG00000158555	*GDPD5*	3.403515335	0.005708863	0.0389427	-0.033233837	0.999933887	0.070788869	0.110335371	-0.039546502	0.812383698
ENSG00000163568	*AIM2*	2.569301987	0.000696003	-0.07938277	0.080078773	0.999933887	-0.054533102	-0.113994601	0.059461498	0.812447255
ENSG00000214530	*STARD10*	5.835868676	0.040948321	0.062773252	-0.021824931	0.999933887	0.262358731	0.297391982	-0.035033251	0.812498261
ENSG00000135597	*REPS1*	3.485039186	0.015761267	-0.053462419	0.069223686	0.999933887	-0.079439801	-0.120548244	0.041108442	0.812498261
ENSG00000164896	*FASTK*	4.462578518	-0.012311736	0.024161875	-0.036473611	0.999933887	0.319460825	0.362563467	-0.043102642	0.812498261
ENSG00000186204	*CYP4F12*	1.473171049	0.011607896	-0.039034177	0.050642073	0.999933887	-0.168573713	-0.264579087	0.096005373	0.812790453
ENSG00000143748	*NVL*	1.542418961	0.053957615	-0.035744148	0.089701763	0.999933887	-0.143420323	-0.0671757	-0.076244623	0.812982746
ENSG00000111678	*C12orf57*	2.22971254	0.086244694	-0.012297391	0.098542084	0.999933887	0.377215118	0.304565591	0.072649527	0.813016997
ENSG00000108518	*PFN1*	8.645742392	0.021091801	-0.00238429	0.023476092	0.999933887	0.239111392	0.20117764	0.037933752	0.813376742
ENSG00000275342	*PRAG1*	1.578199733	-0.08922679	0.065676738	-0.154903528	0.999933887	0.16126424	0.221809411	-0.06054517	0.813388341
ENSG00000139131	*YARS2*	0.585213495	-0.010578154	-0.079062501	0.068484347	0.999933887	0.075204076	0.174689317	-0.099485241	0.813399962
ENSG00000011638	*TMEM159*	2.976774794	-0.110724133	-0.135930723	0.02520659	0.999933887	-0.437696289	-0.388681739	-0.04901455	0.813542077
ENSG00000225434	*LINC01504*	1.493240005	0.008485608	0.227700749	-0.219215141	0.999933887	-0.150444115	-0.078421166	-0.072022949	0.813564246
ENSG00000143774	*GUK1*	7.239818795	0.080844085	0.050849617	0.029994468	0.999933887	0.051101112	0.017459052	0.03364206	0.813586449
ENSG00000225733	*FGD5-AS1*	6.130759977	-0.055003294	-0.049913402	-0.005089892	0.999933887	-0.40702003	-0.438171063	0.031151033	0.813586449
ENSG00000277511	*AC116407.2*	0.437169235	0.065264563	-0.145430835	0.210695397	0.999933887	-0.1177644	-0.238853418	0.121089018	0.813586449
ENSG00000233621	*LINC01137*	0.920508845	-0.111834386	0.17902522	-0.290859606	0.999933887	0.620051757	0.523488415	0.096563342	0.813586449
ENSG00000213839	*TMX2P1*	0.451543564	-0.026486521	0.215651945	-0.242138466	0.999933887	-0.193707306	-0.054544958	-0.139162348	0.813586449
ENSG00000059145	*UNKL*	5.855708353	-0.017531674	-0.059998479	0.042466804	0.999933887	-0.229845838	-0.266705364	0.036859526	0.813600018
ENSG00000171109	*MFN1*	4.95844509	-0.065899756	0.015639986	-0.081539742	0.999933887	-0.310988367	-0.252387456	-0.05860091	0.813635055
ENSG00000101439	*CST3*	5.525324795	0.03855085	0.024940664	0.013610185	0.999933887	-0.282765164	-0.340034727	0.057269563	0.813668325
ENSG00000139291	*TMEM19*	0.826119358	0.013774059	0.098734535	-0.084960476	0.999933887	0.032101493	-0.052343679	0.084445172	0.813668325
ENSG00000227345	*PARG*	1.981568636	-0.009154157	0.000903494	-0.01005765	0.999933887	-0.129144396	-0.19396337	0.064818974	0.813668325
ENSG00000157985	*AGAP1*	0.916337717	0.044055131	0.070555883	-0.026500753	0.999933887	-0.078108878	0.011710348	-0.089819226	0.814056545
ENSG00000104442	*ARMC1*	1.696609349	-0.077605857	0.023232757	-0.100838614	0.999933887	0.08498313	0.155261378	-0.070278248	0.814298015
ENSG00000196419	*XRCC6*	5.993535247	-0.033427802	-0.02829194	-0.005135862	0.999933887	0.013188132	-0.019184473	0.032372605	0.814403834
ENSG00000187801	*ZFP69B*	0.155135639	0.098349529	-0.164882829	0.263232359	0.999933887	0.103704229	-0.022973574	0.126677803	0.814403834

ENSG00000125084	*WNT1*	-0.178257605	0.007783637	-0.161286533	0.16907017	0.999933887	-0.040982098	0.093179821	-0.134161919	0.814403834
ENSG00000165914	*TTC7B*	1.340458204	0.134431124	0.20143015	-0.066999026	0.999933887	0.001360639	0.079263959	-0.077903319	0.814404314
ENSG00000087157	*PGS1*	7.237747866	0.046661025	0.039570609	0.007090416	0.999933887	-0.195624898	-0.160914783	-0.034710115	0.814404314
ENSG00000168028	*RPSA*	7.189608324	-0.011320192	-0.034384834	0.023064643	0.999933887	0.128517209	0.167731821	-0.039214612	0.814404314
ENSG00000148384	*INPP5E*	2.707709541	-0.012763334	0.110138104	-0.122901438	0.999933887	0.147426725	0.200858872	-0.053432147	0.814404314
ENSG00000247774	*PCED1B-AS1*	4.392808572	0.002159303	-0.029529685	0.031688987	0.999933887	0.076845205	0.044558356	0.032286849	0.814404314
ENSG00000067141	*NEO1*	1.478589732	-0.104377499	0.084953462	-0.189330961	0.999933887	-0.046286254	0.040037199	-0.086323453	0.814466423
ENSG00000006625	*GGCT*	-0.5148304	-0.064451227	-0.000758088	-0.063693139	0.999933887	-0.163514974	-0.032178897	-0.131336076	0.814466423
ENSG00000138074	*SLC5A6*	3.785871069	0.010492039	0.031811217	-0.021319178	0.999933887	0.156557893	0.198353301	-0.041795408	0.814466423
ENSG00000223573	*TINCR*	-0.277293144	0.018238906	0.223119345	-0.204880438	0.999933887	-0.157226092	-0.031433204	-0.125792888	0.814466423
ENSG00000135596	*MICAL1*	8.050807306	0.052178429	0.058352141	-0.006173711	0.999933887	-0.155165755	-0.127326576	-0.02783918	0.814478823
ENSG00000273691	*AC087284.1*	0.87990639	-0.102372098	0.002638062	-0.10501016	0.999933887	0.116893816	0.027030116	0.0898637	0.814478823
ENSG00000074603	*DPP8*	4.708262875	-0.014120652	-0.044265009	0.030144357	0.999933887	-0.384475288	-0.426357083	0.041881795	0.814478823
ENSG00000279088	*AC022400.6*	2.202857685	-0.140277471	-0.180952935	0.040675464	0.999933887	0.093303223	0.039975665	0.053327558	0.814620149
ENSG00000099338	*CATSPERG*	0.656379858	0.021264041	-0.017406263	0.038670304	0.999933887	-0.225741929	-0.352951374	0.127209445	0.814806702
ENSG00000226777	*FAM30A*	-0.190945561	-0.101758846	0.218669638	-0.320428484	0.999933887	-0.457121143	-0.344472248	-0.112648895	0.815287813
ENSG00000145337	*PYURF*	2.927722115	0.087720212	0.083158236	0.004561977	0.999933887	0.049032408	-0.012426994	0.061459402	0.815326412
ENSG00000169813	*HNRNPF*	8.842777912	-0.00679912	0.020495284	-0.027294404	0.999933887	-0.10358364	-0.137145593	0.033561954	0.815522072
ENSG00000135437	*RDH5*	1.736630345	0.051988114	-0.12810212	0.180090233	0.999933887	0.026435778	-0.029141538	0.055577316	0.815644869
ENSG00000227512	*AL592301.1*	2.812315832	-0.195779528	-0.159981223	-0.035798305	0.999933887	-0.476523413	-0.412047365	-0.064476047	0.815662772
ENSG00000117906	*RCN2*	2.668375406	-0.044952536	-0.040397152	-0.004555384	0.999933887	-0.170743108	-0.111858704	-0.058884404	0.815662772
ENSG00000185219	*ZNF445*	3.670728674	-0.013854541	0.004131705	-0.017986246	0.999933887	-0.078967366	-0.034724457	-0.044242909	0.815662772
ENSG00000169714	*CNBP*	8.639849432	-0.066242345	-0.111701689	0.045459344	0.999933887	-0.659340182	-0.695810262	0.036470081	0.815670308
ENSG00000165506	*DNAAF2*	1.898077055	0.160105793	0.017910634	0.142195158	0.999933887	0.186122041	0.115326491	0.070795551	0.81585173
ENSG00000112039	*FANCE*	-0.016790502	-0.116863145	-0.023276613	-0.093586532	0.999933887	0.084736816	-0.024229435	0.10896625	0.81585173
ENSG00000184792	*OSBP2*	-0.139065535	-0.039244726	0.026759542	-0.066004268	0.999933887	0.091182712	-0.019367742	0.110550454	0.81585173
ENSG00000197951	*ZNF71*	0.660948781	0.018894462	0.038953612	-0.020059149	0.999933887	0.067833043	-0.028537167	0.09637021	0.81585173
ENSG00000166263	*STXBP4*	-0.363191599	-0.154281013	-0.409687397	0.255406384	0.999933887	-0.250018854	-0.10852557	-0.141493284	0.815883448
ENSG00000214659	*KRT8P26*	0.501777457	-0.057422068	0.008596186	-0.066018255	0.999933887	-0.446957342	-0.571288401	0.124331059	0.815910874
ENSG00000076924	*XAB2*	5.874799139	-0.003198089	0.050917084	-0.054115173	0.999933887	0.018135124	-0.013934481	0.032069605	0.816132444
ENSG00000128918	*ALDH1A2*	4.077718931	0.275342829	0.366489268	-0.091146439	0.999933887	0.642998006	0.56714503	0.075852975	0.816253279
ENSG00000165502	*RPL36AL*	6.285584732	-0.034779462	-0.075679349	0.040899887	0.999933887	-0.077241212	-0.046847493	-0.030393719	0.816341491
ENSG00000106330	*MOSPD3*	2.320609715	-0.073619716	0.107567393	-0.181187109	0.999933887	-0.21216248	-0.138960178	-0.073202302	0.81637165
ENSG00000203804	*ADAMTSL4-AS*	4.14919227	0.093710438	0.083959794	0.009750644	0.999933887	0.347640329	0.296870853	0.050769476	0.816747124
ENSG00000281183	*NPTN-IT1*	3.002010063	-0.046343105	-0.090196893	0.043853788	0.999933887	-0.427312263	-0.376160272	-0.051151992	0.816747124
ENSG00000233806	*LINC01237*	-0.641050807	0.046699949	-0.058907863	0.105607811	0.999933887	-0.029052426	0.12327973	-0.152332156	0.816747124
ENSG00000217527	*RPS16P5*	0.537697337	0.170903964	0.310888112	-0.139984148	0.999933887	0.162491895	-0.026634321	0.189126216	0.816759945
ENSG00000148606	*POLR3A*	2.497957019	-0.019432221	-0.051010089	0.031577868	0.999933887	-0.034565732	0.014461501	-0.049027233	0.816759945
ENSG00000105088	*OLFM2*	-0.120538541	-0.059984195	0.485183352	-0.545167547	0.999933887	0.023031094	0.125470849	-0.102439755	0.816759945
ENSG00000104361	*NIPAL2*	-1.607339119	0.247860677	0.233387427	0.01447325	0.999933887	0.2160853	0.401783465	-0.185698165	0.816849032
ENSG00000284428	*AC092329.4*	0.530547205	-0.100986003	0.058940687	-0.15992669	0.999933887	-0.014902576	-0.104708826	0.08980625	0.816930482
ENSG00000239961	*LILRA4*	-1.34236507	-0.407034794	-0.605152402	0.198117608	0.999933887	-1.554975379	-1.708832751	0.153857372	0.81733427
ENSG00000166925	*TSC22D4*	8.301568497	0.052051105	0.05203001	2.1094e-05	0.999933887	-0.134084478	-0.170475162	0.036390683	0.81733427
ENSG00000104447	*TRPS1*	2.836050487	-0.094223249	0.087700978	-0.181924226	0.999933887	-0.178699084	-0.113865376	-0.064833708	0.81736372
ENSG00000126001	*CEP250*	3.69072812	-0.05057695	0.012156589	-0.06273354	0.999933887	-0.025898384	-0.062416445	0.036518061	0.81736372
ENSG00000138795	*LEF1*	4.28658501	-0.042367782	-0.041529615	-0.000838166	0.999933887	0.114313496	0.077733772	0.036579725	0.81736372
ENSG00000162148	*PPP1R32*	0.35507298	0.082638347	0.168352634	-0.085714287	0.999933887	-0.13704631	-0.231430343	0.094384033	0.81736372
ENSG00000101842	*VSIG1*	0.215156564	0.074322748	0.220853574	-0.146530826	0.999933887	0.113859087	0.233391939	-0.119532852	0.81736372
ENSG00000235579	*RPL38P5*	-0.681067449	0.12874662	-0.178680002	0.307426622	0.999933887	1.102980547	1.255810813	-0.152830266	0.81736372
ENSG00000197958	*RPL12*	7.251054754	0.018046468	-0.022352857	0.040399325	0.999933887	0.118251809	0.154308132	-0.036056323	0.81736372
ENSG00000254703	*SENCR*	-0.339391428	-0.051159402	-0.041701794	-0.009457608	0.999933887	-0.178570268	-0.347337653	0.168767386	0.81736372
ENSG00000142507	*PSMB6*	3.850863358	-0.019676527	0.049231328	-0.068907856	0.999933887	-0.168566899	-0.124569745	-0.043997154	0.81736372
ENSG00000196872	*CRACDL*	0.671907899	0.03343312	0.168231907	-0.134798787	0.999933887	-0.550267553	-0.438881019	-0.111386534	0.81736372
ENSG00000273218	*AC005776.2*	0.673984304	0.021333754	-0.189185425	0.210519179	0.999933887	0.338927518	0.430188272	-0.091260754	0.81736372
ENSG00000260793	*AC003102.1*	0.791124232	-0.018763281	0.262000313	-0.280763594	0.999933887	-0.072267068	0.021035367	-0.093302436	0.81736372
ENSG00000127184	*COX7C*	4.549938527	0.002464941	-0.080821382	0.083286323	0.999933887	0.147967449	0.190885714	-0.042918265	0.81736372
ENSG00000050393	*MCUR1*	1.415835193	-0.000276899	0.150926167	-0.151203066	0.999933887	-0.054359348	0.01405103	-0.068410378	0.81736372
ENSG00000279583	*AC009086.2*	-0.159487366	0.385135208	-0.211458867	0.596594075	0.594252256	0.101153908	-0.002437378	0.103591286	0.817470877
ENSG00000196923	*PDLIM7*	8.402562308	0.053228352	0.016633499	0.036594853	0.999933887	-0.008693463	0.020809692	-0.029503154	0.817555881
ENSG00000182405	*PGBD4*	2.066661104	-0.039509407	-0.020394678	-0.019114729	0.999933887	0.216695145	0.158612736	0.058082409	0.817555881
ENSG00000215244	*LINC02649*	3.810884766	0.054998692	0.058026508	-0.003027815	0.999933887	0.006426775	-0.042334956	0.048761731	0.818045085
ENSG00000150459	*SAP18*	5.741356273	-0.041119167	0.03420962	-0.075328787	0.999933887	-0.125009785	-0.093814825	-0.03119496	0.818341876
ENSG00000105373	*NOP53*	7.214112423	0.102094673	0.092213863	0.009880809	0.999933887	0.093416981	0.141822859	-0.048405877	0.818859124
ENSG00000285589	*AC010422.8*	-0.484284868	-0.187080149	-0.063747743	-0.123332406	0.999933887	0.098470679	-0.039998217	0.138468896	0.818859124
ENSG00000105447	*GRWD1*	2.759403075	-0.063628146	-0.104275177	0.040647031	0.999933887	0.299748462	0.35530023	-0.055551768	0.818859124
ENSG00000023839	*ABCC2*	1.70168364	-0.064556651	-0.074279951	0.0097233	0.999933887	0.152659206	0.072573639	0.080085567	0.818859124
ENSG00000113441	*LNPEP*	5.444446114	0.048702958	-0.009301095	0.058004052	0.999933887	-0.42816986	-0.361221874	-0.066947986	0.818873089
ENSG00000177084	*POLE*	3.74918338	0.002332957	0.068356196	-0.066023239	0.999933887	0.17391735	0.222283793	-0.048366443	0.819083064
ENSG00000228705	*LINC00659*	1.621986645	0.1412158	0.388029244	-0.246813444	0.999933887	0.595001797	0.666595938	-0.071594141	0.819123372
ENSG00000127418	*FGFRL1*	2.664112423	0.066235233	0.011642438	0.054592795	0.999933887	0.144183995	0.196608556	-0.052424561	0.819269771
ENSG00000166963	*MAP1A*	0.762744632	0.020660981	0.264438878	-0.243777897	0.999933887	-0.081565016	-0.207906179	0.126341163	0.819269771
ENSG00000180884	*ZNF792*	0.912025882	0.18678856	0.019545168	0.167243392	0.999933887	0.041651227	0.131428003	-0.089776775	0.819303199
ENSG00000162980	*ARL5A*	1.915182728	-0.024268496	-0.117160566	0.09289207	0.999933887	-0.192677329	-0.109850535	-0.082826793	0.819303199
ENSG00000151376	*ME3*	-0.677130125	0.030677493	-0.125712229	0.156389722	0.999933887	0.08422766	-0.056731594	0.140959254	0.819303199
ENSG00000261326	*LINC01355*	-1.423880708	-0.063582491	-0.353383253	0.289800762	0.999933887	-0.254851798	-0.107975829	-0.146875969	0.819473353
ENSG00000117448	*AKR1A1*	4.160036749	-0.05276096	-0.147280628	0.094519668	0.999933887	0.124634593	0.084064527	0.040570066	0.819481479
ENSG00000112992	*NNT*	3.015118384	-0.023530651	-0.053045015	0.029514364	0.999933887	-0.03882732	0.000413753	-0.039241073	0.819481479
ENSG00000102794	*ACOD1*	-2.381409195	0.703934944	0.66877877	0.035156173	0.999933887	3.877383152	4.209238458	-0.331855305	0.819815869
ENSG00000123838	*C4BPA*	1.40520972	-0.101833199	-0.17715285	0.075319651	0.999933887	0.041070132	0.121545052	-0.080474919	0.819815869
ENSG00000185245	*GP1BA*	1.397928327	0.040619193	-0.106087293	0.146706486	0.999933887	0.077224542	-0.026520674	0.103745216	0.819815869
ENSG00000059588	*TARBP1*	2.601848046	-0.003478821	0.133647079	-0.1371259	0.999933887	0.043259337	0.095003709	-0.051744372	0.819815869
ENSG00000243179	*AC110769.1*	-0.112677894	-0.280109447	-0.038783606	-0.241325841	0.999933887	1.301395925	1.445492812	-0.144096887	0.820422623
ENSG00000267655	*AC125437.1*	-0.256305451	-0.170508084	-0.173037883	0.002529799	0.999933887	0.110257245	-0.069345864	0.179603109	0.820422623
ENSG00000090861	*AARS1*	3.319282867	-0.06013731	-0.041307132	-0.018830177	0.999933887	0.023969314	-0.018521616	0.04249093	0.820422623
ENSG00000121769	*FABP3*	-0.902979819	-0.170986812	0.377703337	-0.548690149	0.999933887	0.064190307	-0.059803146	0.123993453	0.820422623
ENSG00000173848	*NET1*	1.192835449	-0.058117155	-0.026595465	-0.03152169	0.999933887	-0.222436625	-0.132670853	-0.089765772	0.820422623
ENSG00000205078	*SYCE1L*	-0.222848938	0.050897859	-0.143288424	0.194186283	0.999933887	0.161814344	0.028543235	0.133271109	0.820422623
ENSG00000143183	*TMCO1*	4.172354354	-0.0706579	-0.020307852	-0.050350049	0.999933887	-0.136849753	-0.101330509	-0.035519244	0.820501664
ENSG00000156650	*KAT6B*	4.486045687	0.054505002	-0.052297572	0.106802574	0.999933887	-0.343422743	-0.393517034	0.050094291	0.820501664
ENSG00000123131	*PRDX4*	0.474963855	-0.108967995	0.264033121	-0.373001116	0.999933887	0.163206731	0.26501332	-0.101806588	0.820501664
ENSG00000147576	*ADHFE1*	-0.467402195	-0.123411689	0.070789853	-0.194201542	0.999933887	0.021035578	-0.10314712	0.124182698	0.820501664
ENSG00000235272	*RAMACL*	-1.458160583	-0.416646531	-0.332666477	-0.083980055	0.999933887	-0.479875331	-0.637956636	0.158081304	0.821227343
ENSG00000076108	*BAZ2A*	8.086654949	0.011008993	0.039264641	-0.028255648	0.999933887	-0.235152679	-0.264403529	0.02925085	0.821310504
ENSG00000121864	*ZNF639*	2.55022669	0.009556675	0.036458325	-0.02690165	0.999933887	0.07697252	0.013089906	0.063882614	0.82134123
ENSG00000279699	*AC004264.2*	1.222375128	0.227596298	0.019355936	0.208240363	0.999933887	2.239824951	2.339711647	-0.099886696	0.821355445
ENSG00000173762	*CD7*	5.324792736	0.030409665	0.051058692	-0.020649027	0.999933887	0.193277341	0.229195268	-0.035917926	0.821355445
ENSG00000178015	*GPR150*	-0.812506716	0.101399266	-0.310275581	0.411674847	0.999933887	-1.5039821	-1.368861905	-0.135120195	0.821355445
ENSG00000117461	*PIK3R3*	-0.20666038	-0.218217	-0.323088958	0.104871957	0.999933887	-0.131135022	-0.024243692	-0.106891331	0.82137817
ENSG00000146021	*KLHL3*	1.676706279	-0.026864243	-0.032099312	0.005235069	0.999933887	-0.107133763	-0.043728952	-0.063404811	0.82137817
ENSG00000152942	*RAD17*	2.192398437	-0.049224838	-0.163853317	0.114628479	0.999933887	-0.274814041	-0.221887406	-0.052926635	0.821424876
ENSG00000186193	*SAPCD2*	-1.433882696	0.199030709	-0.130031031	0.32906174	0.999933887	-0.382530688	-0.112939571	-0.269591117	0.8214429
ENSG00000063761	*ADCK1*	-0.03908873	-0.044375976	0.007300622	-0.051676598	0.999933887	-0.050572059	-0.150642261	0.100070202	0.8214429
ENSG00000223117	*RN7SKP296*	0.350870266	-0.129366379	-0.083319575	-0.046046804	0.999933887	-0.394030552	-0.670225008	0.276194457	0.821702014
ENSG00000254060	*AC022778.1*	0.45741044	0.184534024	0.287348293	-0.10281427	0.999933887	0.476754176	0.616658546	-0.139904369	0.82181086
ENSG00000172543	*CTSW*	4.421059812	0.017412076	0.002265361	0.015146716	0.999933887	0.130169792	0.175669552	-0.04549976	0.821842831

ENSG00000254328	*AC008429.4*	-0.068496912	0.242894852	0.117186763	0.125708089	0.999933887	0.810887961	0.675710758	0.135177203	0.821898992
ENSG00000211785	*TRAV12-1*	0.048840817	-0.048354951	0.045293218	-0.093648169	0.999933887	0.042867461	0.130666458	-0.087798996	0.821925232
ENSG00000123607	*TTC21B*	2.236430946	-0.024930192	-0.012642704	-0.012287488	0.999933887	-0.19248089	-0.096359751	-0.096121139	0.821925232
ENSG00000287115	*AC009951.6*	0.678846642	0.192338439	0.016464747	0.175873692	0.999933887	1.541577303	1.463268004	0.078309299	0.821936918
ENSG00000245888	*NSMCE1-DT*	0.985270413	-0.142332481	-0.172995402	0.030662921	0.999933887	-0.365897605	-0.463047668	0.097150063	0.822056577
ENSG00000243609	*RPS2P44*	1.444377633	0.154668008	0.382216607	-0.227548598	0.999933887	1.803248215	1.668162747	0.135085468	0.822099666
ENSG00000099917	*MED15*	7.306884566	0.031197426	0.040158212	-0.008960786	0.999933887	0.130519239	0.153601662	-0.023082423	0.822108744
ENSG00000187049	*TMEM216*	2.966119023	0.046222872	-0.086345296	0.132568168	0.999933887	-0.127267063	-0.175981937	0.048714874	0.822108744
ENSG00000084444	*FAM234B*	0.401081088	-0.134910107	0.089093374	-0.224003481	0.999933887	-0.617773082	-0.722785728	0.105012646	0.822387585
ENSG00000180712	*LINC02363*	2.544561844	0.05289005	0.143404216	-0.090514166	0.999933887	0.586919099	0.530742089	0.056177011	0.822425808
ENSG00000180921	*FAM83H*	-0.171209315	0.316324248	-0.048407834	0.364732082	0.999933887	0.079130255	-0.043266616	0.12239687	0.822453896
ENSG00000164430	*CGAS*	1.791646147	0.038813963	0.185354402	-0.146540439	0.999933887	0.195217858	0.2564277	-0.061209842	0.822456841
ENSG00000083750	*RRAGB*	2.117120305	-0.040344891	-0.074090538	0.033745647	0.999933887	-0.062011056	-0.010437482	-0.051573574	0.822708256
ENSG00000159733	*ZFYVE28*	3.022208455	0.142204551	-0.009525149	0.1517297	0.999933887	0.160764232	0.208370214	-0.047605981	0.822838547
ENSG00000249249	*AC010226.1*	-0.598530131	0.062119264	-0.210187835	0.272307099	0.999933887	0.066268548	0.223027553	-0.156759005	0.823029468
ENSG00000250893	*AC098869.2*	-0.082881986	-0.078578733	0.129488301	-0.208067034	0.999933887	-0.324961625	-0.198280762	-0.126680863	0.823105949
ENSG00000145780	*FEM1C*	5.31927889	-0.015259253	-0.032983949	0.017724696	0.999933887	-0.452799473	-0.498064362	0.045264889	0.823105949
ENSG00000188372	*ZP3*	-0.403945773	-0.00241455	0.478471628	-0.480886178	0.999933887	2.100240053	1.980937635	0.119302418	0.823105949
ENSG00000280402	*AC093525.10*	0.065906214	0.19824217	-0.003983559	0.202225729	0.999933887	-0.776044586	-0.89498559	0.118941004	0.823197465
ENSG00000234545	*FAM133B*	2.519330277	0.078521106	-0.021660338	0.100181444	0.999933887	0.006603204	-0.06879805	0.075401254	0.823197465
ENSG00000223662	*SAMSN1-AS1*	-0.99809824	-0.053711566	-0.846740765	0.793029199	0.999933887	2.188371937	2.378624745	-0.190252808	0.823197465
ENSG00000196421	*C20orf204*	0.128622731	0.01403995	0.143839377	-0.129799427	0.999933887	-0.019509789	0.106276393	-0.125786183	0.823197465
ENSG00000080608	*PUM3*	1.616482847	-0.199597368	0.045623416	-0.245220784	0.999933887	0.037592693	0.10374352	-0.066150827	0.823421468
ENSG00000184361	*SPATA32*	-0.219696742	0.237331395	0.043500734	0.193830661	0.999933887	-0.095505097	0.05360068	-0.149105777	0.823439541
ENSG00000261118	*AC092123.1*	-0.032484094	-0.1237334	-0.102667296	-0.021066104	0.999933887	-0.354014235	-0.451603485	0.09758925	0.823439541
ENSG00000286342	*AC073210.3*	-0.982741265	-0.19446671	0.471773172	-0.666239882	0.999933887	-0.720302736	-0.88247203	0.162169294	0.823439541
ENSG00000149269	*PAK1*	7.775408524	-0.026299308	-0.043857933	0.017558625	0.999933887	0.103280088	0.076639366	0.026640723	0.823439541
ENSG00000146223	*RPL7L1*	3.454764483	0.025854016	-0.049846548	0.075700564	0.999933887	0.084431535	0.047447738	0.036983798	0.823439541
ENSG00000261349	*AL031432.2*	1.398359773	0.053536784	0.114907426	-0.061370642	0.999933887	0.146793297	0.081975014	0.064818283	0.823439541
ENSG00000257242	*LINC01619*	1.077490515	0.046810144	-0.022439169	0.069249312	0.999933887	0.085220015	0.177908698	-0.092688683	0.823439541
ENSG00000164615	*CAMLG*	4.201687004	-0.011950516	-0.007239556	-0.00471096	0.999933887	-0.125922219	-0.090408354	-0.035513866	0.823476749
ENSG00000099783	*HNRNPM*	7.319269227	-0.022602742	0.006346687	-0.028949429	0.999933887	-0.048352535	-0.023303248	-0.025049287	0.823532635
ENSG00000270062	*AL606834.2*	0.635930139	0.057845831	-0.039862139	0.09770797	0.999933887	-0.356378312	-0.485523868	0.129145556	0.823532635
ENSG00000170175	*CHRNB1*	0.863816303	0.064179337	0.184748841	-0.120569504	0.999933887	-0.254458706	-0.357801757	0.103343051	0.823532635
ENSG00000143126	*CELSR2*	0.62721894	0.079282208	0.150632725	-0.071350517	0.999933887	0.027446135	-0.0608697	0.088315834	0.823935864
ENSG00000236911	*AL137789.1*	-0.963010511	0.027667317	0.099321652	-0.071654335	0.999933887	-0.178400659	0.022981045	-0.201381704	0.823935864
ENSG00000174125	*TLR1*	6.742988896	-0.162057805	-0.173108569	0.011050764	0.999933887	-1.324255928	-1.396831397	0.072575469	0.823951105
ENSG00000213891	*RPL3P6*	0.27134132	0.267824061	0.468612097	-0.200788037	0.999933887	0.21464294	0.422150584	-0.207507644	0.823951105
ENSG00000131469	*RPL27*	7.241869032	0.032270539	-0.060851323	0.093121862	0.999933887	0.080325008	0.11935161	-0.039026603	0.823951105
ENSG00000082068	*WDR70*	1.911620612	0.022292704	-0.117722583	0.140015287	0.999933887	0.093379929	0.039850172	0.053529757	0.823951105
ENSG00000006747	*SCIN*	-1.625280405	-0.789432947	0.115947637	-0.905380583	0.999933887	-0.966449253	-0.83788967	-0.128559583	0.82397279
ENSG00000224578	*HNRNPA1P48*	-0.073495687	-0.249868462	-0.337509218	0.087640756	0.999933887	0.205215053	0.113638623	0.091576431	0.82397279
ENSG00000163374	*YY1AP1*	5.869799836	-0.057045273	-0.034178704	-0.022866569	0.999933887	0.293054009	0.321808384	-0.028754375	0.82397279
ENSG00000255760	*LINC02422*	3.194054377	-0.067443581	-0.007631346	-0.059812235	0.999933887	0.280945433	0.21793172	0.063013713	0.82397279
ENSG00000258337	*AC130895.1*	-0.474289991	0.142633984	0.230504872	-0.087870888	0.999933887	-0.008119613	0.163864196	-0.17198381	0.82397279
ENSG00000105700	*KXD1*	4.693426265	0.025517407	0.024371778	0.001145629	0.999933887	-0.101038777	-0.070529981	-0.030508796	0.82397279
ENSG00000139405	*RITA1*	0.407232524	0.089659989	-0.1070721	0.196732089	0.999933887	0.054951649	0.175452378	-0.12050073	0.82397279
ENSG00000130513	*GDF15*	3.262806485	0.049633757	-0.087431484	0.137065241	0.999933887	0.317810231	0.259913029	0.057897202	0.82397279
ENSG00000143297	*FCRL5*	-0.214939702	-0.077593498	-0.02640418	-0.051189318	0.999933887	0.273571767	0.343569159	-0.069997392	0.82397279
ENSG00000254595	*AC084337.1*	-0.10364534	-0.045290246	0.521971508	-0.567261754	0.594252256	-0.155043456	-0.270257921	0.115214465	0.82397279
ENSG00000169629	*RGPD8*	0.269506066	-0.046075266	-0.101400683	0.055325417	0.999933887	-0.388219282	-0.275021263	-0.113198018	0.82397279
ENSG00000234171	*RNASEH1-AS*	-0.193527218	-0.042150617	0.084237845	-0.126388461	0.999933887	0.101129073	-0.013523099	0.114652171	0.82397279
ENSG00000198553	*KCNRG*	-0.700187162	-0.036853604	0.420296814	-0.457150418	0.999933887	0.174604071	0.044845965	0.129758105	0.82397279
ENSG00000204394	*VARS1*	3.150539506	0.074830642	0.164405469	-0.089574827	0.999933887	0.256006064	0.218136082	0.037869983	0.82407504
ENSG00000183426	*NPIPA1*	-0.398219043	-0.105225348	-0.169089895	0.063864546	0.999933887	0.05603138	0.169371479	-0.113340099	0.824113925
ENSG00000099194	*SCD*	2.43821644	0.056179633	-0.07510803	0.131287664	0.999933887	-0.396904973	-0.465584844	0.06867987	0.824279751
ENSG00000211786	*TRAV8-2*	-0.183458824	-0.281955701	0.116515757	-0.398471458	0.999933887	0.1762534	0.306299511	-0.130046111	0.8243847
ENSG00000104824	*HNRNPL*	7.364123204	0.02189601	0.057386543	-0.035490533	0.999933887	-0.09291413	-0.117946714	0.025032584	0.8243847
ENSG00000224288	*MTCYBP11*	-1.453213837	-0.064951832	0.353027657	-0.417979489	0.999933887	0.475902472	0.187398531	0.288503941	0.8243847
ENSG00000119599	*DCAF4*	1.08610372	0.017503606	0.018784044	-0.001280438	0.999933887	0.074412832	0.153124061	-0.078711229	0.824743077
ENSG00000244513	*AC109587.1*	-0.207565377	0.097844785	-0.033817229	0.131662014	0.999933887	-0.056045231	0.072956772	-0.129002003	0.824812614
ENSG00000198720	*ANKRD13B*	-0.505375241	-0.283024974	-0.294879742	0.011854769	0.999933887	0.023846876	-0.097501641	0.121348517	0.824946203
ENSG00000272655	*POLR2J4*	1.022147117	0.133939325	0.006884938	0.127054387	0.999933887	0.278169594	0.203704738	0.074464855	0.824946203
ENSG00000135249	*RINT1*	2.362268022	-0.046017128	0.155550212	-0.20156734	0.999933887	-0.007710099	-0.061931445	0.054221346	0.824947968
ENSG00000133466	*C1QTNF6*	3.083613473	0.037588052	-0.005361443	0.042949495	0.999933887	0.245140491	0.289110172	-0.043969682	0.824947968
ENSG00000185156	*MFSD6L*	0.883980881	0.026010596	0.106239671	-0.080229075	0.999933887	0.385786356	0.481037173	-0.095250817	0.824947968
ENSG00000000460	*C1orf112*	0.449761291	-0.020255713	-0.168482347	0.148226634	0.999933887	-0.050256208	0.046653709	-0.096909917	0.824947968
ENSG00000261644	*AC007728.2*	2.890437953	-0.033267435	0.089868805	-0.12313624	0.999933887	0.553469766	0.602572489	-0.049102723	0.825035313
ENSG00000204220	*PFDN6*	2.831528467	0.028450256	0.061126268	-0.032676012	0.999933887	0.164267249	0.123550157	0.040717092	0.825035313
ENSG00000130193	*THEM6*	0.397198417	0.080717671	0.263570888	-0.182853217	0.999933887	0.156384567	0.043285655	0.113098912	0.825314872
ENSG00000127954	*STEAP4*	6.318469798	-0.11997651	-0.097030289	-0.022946221	0.999933887	-1.335863245	-1.266004026	-0.069859219	0.825349722
ENSG00000276180	*H4C9*	2.331145338	-0.007068915	-0.058505661	0.051436747	0.999933887	-0.104925491	-0.165260072	0.060334581	0.825349722
ENSG00000175611	*LINC00476*	0.605640832	0.249150107	-0.148268053	0.39741816	0.999933887	0.031697924	0.125990011	-0.094292087	0.825516364
ENSG00000022567	*SLC45A4*	9.174283743	-0.00938368	-0.023308493	0.013924813	0.999933887	-0.539505286	-0.576086089	0.036580803	0.825629112
ENSG00000143549	*TPM3*	9.356530333	0.002889277	-0.024600613	0.027489889	0.999933887	-0.171552831	-0.143952157	-0.027600674	0.825660887
ENSG00000088992	*TESC*	2.08246636	0.001908965	-0.103540526	0.105449492	0.999933887	-0.05618776	0.008943069	-0.06513083	0.825747705
ENSG00000238366	*Y_RNA*	-0.030840326	0.129276375	0.192290681	-0.063014307	0.999933887	0.373930379	0.551821633	-0.177891254	0.825750825
ENSG00000042493	*CAPG*	5.222607407	-0.042594615	0.014703459	-0.057298074	0.999933887	0.362414023	0.325776224	0.0366378	0.826212955
ENSG00000133315	*MACROD1*	-0.27900163	0.082714386	0.08531593	-0.002601544	0.999933887	-0.020226663	-0.145291624	0.125064961	0.826212955
ENSG00000260273	*AL359711.2*	0.506348693	0.053146104	-0.030280163	0.083426267	0.999933887	0.279379106	0.379669136	-0.10029003	0.826212955
ENSG00000224616	*RTCA-AS1*	0.365599258	-0.362989378	-0.324252508	-0.038736871	0.999933887	-1.365014668	-1.2494502	-0.115564467	0.826705983
ENSG00000101307	*SIRPB1*	8.463274159	-0.002770037	-0.016747424	0.013977387	0.999933887	-0.365400122	-0.333618465	-0.031781657	0.826705983
ENSG00000198466	*ZNF587*	3.65394616	-0.016281018	0.025805884	-0.042086902	0.999933887	-0.250137874	-0.297649027	0.047511153	0.826798532
ENSG00000013306	*SLC25A39*	3.733676118	0.078755935	-0.001861692	0.080617627	0.999933887	0.1916536	0.153681763	0.037971837	0.826987617
ENSG00000135776	*ABCB10*	2.145310383	0.023002724	0.000267002	0.022735722	0.999933887	-0.086026422	-0.021745047	-0.064281376	0.827543702
ENSG00000156482	*RPL30*	6.760218598	0.022666035	-0.033926976	0.056593011	0.999933887	0.12306347	0.155341417	-0.032277947	0.827557577
ENSG00000082898	*XPO1*	5.259836689	-0.057906642	-0.044189987	-0.013716655	0.999933887	-0.332138034	-0.286064235	-0.046073798	0.827609897
ENSG00000116741	*RGS2*	9.66834472	0.032761469	-0.053292032	0.086053501	0.999933887	-0.424528676	-0.47410949	0.049580815	0.827616964
ENSG00000213759	*UGT2B11*	-0.640141336	-0.001942726	0.339665077	-0.341607803	0.999933887	-0.200722426	-0.087068329	-0.113654098	0.827616964
ENSG00000229891	*LINC01315*	-0.361242493	-0.197648456	-0.298945209	0.101296753	0.999933887	-0.281844853	-0.392567992	0.110723139	0.82771522
ENSG00000122122	*SASH3*	8.178633328	-0.002435924	-0.014539798	0.012103874	0.999933887	-0.212907021	-0.172811188	-0.040095833	0.82771522
ENSG00000087237	*CETP*	2.559081086	-0.072672914	-0.127013553	0.054340638	0.999933887	-0.532094891	-0.485902876	-0.046192015	0.827800208
ENSG00000124788	*ATXN1*	7.342609685	-0.028043339	-0.016469617	-0.011573721	0.999933887	-0.209586465	-0.245466456	0.035879991	0.827800208
ENSG00000104774	*MAN2B1*	6.438797093	0.008868388	-0.058643428	0.067511817	0.999933887	0.046360058	0.016242013	0.030118045	0.827800208
ENSG00000169193	*CCDC126*	2.149704401	0.102538557	-0.004074178	0.106612735	0.999933887	-0.696723615	-0.625816274	-0.070907341	0.827911691
ENSG00000198502	*HLA-DRB5*	2.983195071	0.071109461	0.041188931	0.02992053	0.999933887	0.613672141	0.689582149	-0.075910007	0.827911691
ENSG00000181924	*COA4*	1.374246751	0.046194453	0.155857397	-0.109662943	0.999933887	0.124514817	0.196597454	-0.072082637	0.827911691
ENSG00000107672	*NSMCE4A*	2.424880403	0.082674739	-0.053030966	0.135705705	0.999933887	0.168588987	0.119069608	0.049519379	0.82792607
ENSG00000180221	*TPT1P10*	0.089028526	-0.033264039	0.192506165	-0.225770204	0.999933887	-0.167226689	0.01934835	-0.186575039	0.82792607
ENSG00000260920	*AL031985.3*	-0.315743719	-0.102574148	-0.31079249	0.208218342	0.999933887	-0.138322328	0.014168506	-0.152490835	0.828180877
ENSG00000064102	*INTS13*	1.572873649	0.061918908	-0.029670964	0.091589872	0.999933887	-0.010002149	0.059364076	-0.069366225	0.828290659
ENSG00000225450	*AL021707.1*	-0.348508143	0.18346383	0.180709669	0.002754161	0.999933887	-0.103366736	-0.225796131	0.122429395	0.828573864
ENSG00000043514	*TRIT1*	0.920430565	-0.143334704	0.051214121	-0.194548825	0.999933887	0.057117681	0.129099572	-0.071981891	0.828573864
ENSG00000264553	*MIR4257*	-0.038359581	0.018475982	0.020127326	-0.001651343	0.999933887	0.534858156	0.391664711	0.143193445	0.828642898
ENSG00000103549	*RNF40*	6.418789856	0.028690778	0.026774533	0.001916245	0.999933887	-0.22900832	-0.264463982	0.035455661	0.828679379

ENSG00000145990	*GFOD1*	4.521265332	-0.033229102	0.013438958	-0.04666806	0.999933887	-0.815975899	-0.771841705	-0.044134195	0.82873991
ENSG00000168273	*SMIM4*	0.064501181	0.051133676	-0.088568122	0.139701798	0.999933887	-0.002435499	-0.09853463	0.096099131	0.82873991
ENSG00000267214	*AC092068.1*	-0.510494598	0.052294389	0.140949799	-0.08865541	0.999933887	0.216022842	0.09801874	0.118004102	0.82873991
ENSG00000100979	*PLTP*	-0.136797228	-0.018532381	-0.376677522	0.358145141	0.999933887	-0.274604857	-0.144224513	-0.130380344	0.82873991
ENSG00000061676	*NCKAP1*	0.408642355	0.093894616	-0.052439932	0.146334548	0.999933887	-0.306696763	-0.398771089	0.092074327	0.829083148
ENSG00000141425	*RPRD1A*	4.481935038	-0.050843817	-0.002662221	-0.048181596	0.999933887	-0.717053154	-0.760728676	0.043675522	0.829163952
ENSG00000185883	*ATP6V0C*	3.844390179	0.311177959	0.139869195	0.171308763	0.999933887	0.592219919	0.529775055	0.062444864	0.829365385
ENSG00000100934	*SEC23A*	3.688216665	0.04815361	0.004041245	0.044112365	0.999933887	-0.393179942	-0.437112962	0.04393302	0.829365385
ENSG00000163807	*KIAA1143*	3.197998766	-0.104981478	-0.09872162	-0.006259858	0.999933887	-0.064214395	-0.021743931	-0.042470464	0.829438414
ENSG00000250959	*GLUD1P3*	0.31322239	-0.14439128	-0.093937032	-0.050454249	0.999933887	-0.049915634	0.03759491	-0.087510544	0.829438414
ENSG00000143951	*WDPCP*	0.463881996	-0.112247967	-0.540852544	0.428604578	0.944094537	-0.071006814	-0.166537333	0.095530519	0.829438414
ENSG00000079435	*LIPE*	0.58260875	0.114581459	0.403319865	-0.288738405	0.999933887	-0.063221356	-0.143401957	0.0801806	0.829438414
ENSG00000163528	*CHCHD4*	0.294739047	0.099930163	-0.058766179	0.158696342	0.999933887	0.114794622	0.022585383	0.09220924	0.829438414
ENSG00000100226	*GTPBP1*	8.161634988	0.01056808	0.019487047	-0.008918967	0.999933887	0.031310074	0.057658495	-0.026348421	0.829438414
ENSG00000163681	*SLMAP*	5.092597514	0.007855607	-0.03091854	0.038774147	0.999933887	-0.56165515	-0.617301939	0.055646788	0.829438414
ENSG00000287195	*AC131568.1*	3.491026071	-0.121900076	-0.00153028	-0.120369796	0.999933887	-0.02982291	0.064184297	-0.094007207	0.829479248
ENSG00000249850	*KRT18P31*	-0.164064174	0.102752563	0.07486497	0.027887593	0.999933887	0.222622847	0.08820388	0.134418968	0.829728118
ENSG00000085644	*ZNF213*	3.603238709	0.037935925	-0.046758294	0.084694219	0.999933887	-0.359150717	-0.401053652	0.041902935	0.830041552
ENSG00000113583	*C5orf15*	3.258177661	-0.006354335	-0.064344298	0.057989963	0.999933887	-0.286501455	-0.250147013	-0.036354442	0.830123712
ENSG00000180776	*ZDHHC20*	5.468962223	0.091208646	0.023986039	0.067222607	0.999933887	-0.805026652	-0.864691372	0.059664721	0.830123905
ENSG00000075856	*SART3*	4.63596649	-0.047744177	-0.009663871	-0.038080306	0.999933887	0.082569024	0.051074737	0.031494287	0.830212326
ENSG00000167930	*FAM234A*	2.819477303	0.04199694	0.012188918	0.029808022	0.999933887	-0.057094236	-0.116781201	0.059686965	0.83042248
ENSG00000135736	*CCDC102A*	0.378235776	0.157103694	0.123753138	0.033350556	0.999933887	0.183831796	0.280813074	-0.096981279	0.830484581
ENSG00000168904	*LRRC28*	1.838472849	0.001560372	0.105900929	-0.104340557	0.999933887	-0.03850933	0.023838168	-0.062347498	0.83049125
ENSG00000204227	*RING1*	3.676971141	0.130428966	0.055273542	0.075155424	0.999933887	0.079986037	0.033062884	0.046923152	0.830624989
ENSG00000227201	*CNN2P1*	-0.136449506	0.007012981	0.001853497	0.005159485	0.999933887	0.63057242	0.805273281	-0.174700861	0.830624989
ENSG00000124535	*WRNIP1*	3.460269655	0.084575617	0.21448517	-0.129909553	0.999933887	0.121350237	0.081297184	0.040053053	0.830679665
ENSG00000071894	*CPSF1*	4.746067779	-0.031221192	0.033474359	-0.06469555	0.999933887	0.14769269	0.187376569	-0.039683878	0.830679665
ENSG00000278864	*AC055811.4*	0.504602985	-0.136476119	-0.031603053	-0.104873067	0.999933887	0.128317253	0.043295229	0.085022023	0.830945607
ENSG00000198934	*MAGEE1*	0.071111257	-0.043636155	-0.044441469	0.000805314	0.999933887	0.057992344	-0.043140046	0.101132389	0.830945607
ENSG00000135069	*PSAT1*	0.679128961	-0.024068997	-0.120628252	0.096559255	0.999933887	-0.038770642	0.032560914	-0.071331557	0.831025279
ENSG00000064961	*HMG20B*	5.320171945	0.054873695	0.046613796	0.0082599	0.999933887	0.007036855	0.037539779	-0.030502924	0.831321386
ENSG00000168778	*TCTN2*	0.000782838	0.219521902	0.134187644	0.085334258	0.999933887	-0.11932577	-0.206919654	0.087593885	0.831474723
ENSG00000108523	*RNF167*	6.696968367	0.007207391	0.004394417	0.002812975	0.999933887	0.087330552	0.061149932	0.02618062	0.831569891
ENSG00000261824	*LINC00662*	0.584771398	-0.092986819	0.156392383	-0.249379203	0.999933887	-0.266993247	-0.166402138	-0.100591109	0.832191566
ENSG00000058866	*DGKG*	1.471769858	0.077945734	-0.018017407	0.095963141	0.999933887	0.128047645	0.047711132	0.080336513	0.832524486
ENSG00000149582	*TMEM25*	-0.155590396	0.126730261	-0.132848488	0.25957875	0.999933887	0.159225537	0.04721554	0.112009997	0.832524486
ENSG00000231327	*LINC01816*	0.900634671	0.272749787	0.52959782	-0.256848033	0.999933887	0.957132684	1.055356049	-0.098223365	0.832683956
ENSG00000286511	*Z95118.2*	0.194138429	-0.277999109	-0.398781747	0.120782638	0.999933887	-1.872434216	-1.754458035	-0.117976181	0.832683956
ENSG00000161638	*ITGA5*	7.915851272	-0.057952699	-0.090203632	0.032250933	0.999933887	-0.464577173	-0.504357203	0.03978003	0.832683956
ENSG00000160318	*CLDND2*	0.707643822	-0.018880272	0.093894696	-0.112774968	0.999933887	0.018737465	0.101631246	-0.08289378	0.832683956
ENSG00000103472	*RRN3P2*	-0.117417379	-0.184229333	0.025825693	-0.210055027	0.999933887	0.100457862	0.188479276	-0.088021414	0.832890474
ENSG00000112306	*RPS12*	7.798646061	-0.015336646	-0.034740971	0.019404326	0.999933887	0.114252715	0.149507687	-0.035254972	0.832890474
ENSG00000138032	*PPM1B*	3.883566002	-0.087511557	-0.051873982	-0.035637575	0.999933887	-0.58567783	-0.633128736	0.047450906	0.832914665
ENSG00000259118	*AL139022.1*	1.244974522	0.120556698	0.017638545	0.102918153	0.999933887	-0.380393167	-0.471930038	0.09153687	0.833426347
ENSG00000105205	*CLC*	5.35779413	-0.060971016	-0.007925503	-0.053045513	0.999933887	-0.097553793	-0.147236602	0.049682809	0.833426347
ENSG00000231181	*AL954705.1*	-0.00688185	-0.044751416	-0.094083124	0.049331708	0.999933887	0.544828461	0.679279001	-0.13445054	0.833426347
ENSG00000251562	*MALAT1*	12.524352158	0.055451762	0.254226954	-0.198775192	0.999933887	0.308817285	0.387869453	-0.079052168	0.83349566
ENSG00000188033	*ZNF490*	0.345484144	0.062519182	-0.129267799	0.19178698	0.999933887	-0.238896067	-0.133765162	-0.105130904	0.83349566
ENSG00000189007	*ADAT2*	1.62259409	-0.109142683	-0.048446721	-0.060695962	0.999933887	0.039517888	0.097133303	-0.057615415	0.833629631
ENSG00000157184	*CPT2*	1.26716504	0.083970729	0.080672318	0.003298411	0.999933887	-0.236059014	-0.307209513	0.0711505	0.833629631
ENSG00000111275	*ALDH2*	1.485658808	0.028452255	-0.125646066	0.154098322	0.999933887	-0.101618368	-0.211199145	0.109580777	0.833629631
ENSG00000136144	*RCBTB1*	4.479185145	-0.086141917	-0.083635155	-0.002506762	0.999933887	-0.669989391	-0.700724806	0.030735414	0.833789613
ENSG00000211820	*TRAV41*	-0.991136381	-0.101940137	-0.281647983	0.179707847	0.999933887	0.118252438	0.238178717	-0.119926278	0.833789613
ENSG00000162736	*NCSTN*	6.972441209	-0.018731679	0.00243083	-0.02116251	0.999933887	-0.013999396	0.01221063	-0.026210027	0.833789613
ENSG00000006756	*ARSD*	1.697297042	0.147274781	0.018353572	0.128921209	0.999933887	-0.031332355	-0.104321105	0.072988751	0.833844742
ENSG00000162552	*WNT4*	0.721934676	0.200687302	0.266189555	-0.065502253	0.999933887	0.289913825	0.415510741	-0.125596916	0.834208012
ENSG00000106638	*TBL2*	1.963547182	-0.05304608	0.036427299	-0.089473379	0.999933887	0.104770854	0.052585802	0.052185052	0.834208012
ENSG00000154358	*OBSCN*	3.171492949	0.092570267	0.07777037	0.014799898	0.999933887	0.075399622	0.130292441	-0.054892819	0.834400208
ENSG00000198590	*C3orf35*	0.45063743	0.128382875	-0.116074054	0.24445693	0.999933887	0.039096337	-0.04799763	0.087093967	0.834476616
ENSG00000185215	*TNFAIP2*	10.995204246	0.078718042	0.093799324	-0.015081282	0.999933887	1.096646219	1.13293185	-0.036285632	0.834617168
ENSG00000100216	*TOMM22*	3.067387094	-0.015710279	-0.012281205	-0.003429074	0.999933887	0.103836945	0.149504484	-0.045667539	0.834667298
ENSG00000228327	*AL669831.1*	2.124858119	0.07669023	0.03928527	0.037404959	0.999933887	0.388810786	0.309827477	0.078983309	0.834726473
ENSG00000232187	*FTH1P7*	1.786263196	0.031132872	0.025141329	0.005991543	0.999933887	0.607565	0.664080116	-0.056515116	0.834873341
ENSG00000182700	*IGIP*	-0.099774147	0.025058743	0.124573538	-0.099514795	0.999933887	-0.487518559	-0.588581607	0.101063048	0.834938915
ENSG00000107669	*ATE1*	2.327436101	-0.038954319	-0.063586512	0.024632193	0.999933887	-0.127768031	-0.076193596	-0.051574435	0.834997958
ENSG00000286825	*AL096870.10*	-0.196108402	0.454953515	0.54645439	-0.091500876	0.999933887	1.35072105	1.482944907	-0.132223856	0.835020223
ENSG00000188375	*H3-5*	0.221775709	0.078528453	0.096225512	-0.017697059	0.999933887	0.272718289	0.381195772	-0.108477483	0.835020223
ENSG00000134897	*BIVM*	-0.277969787	0.025634107	-0.057458144	0.083092251	0.999933887	0.156077775	0.020861319	0.135216455	0.835020223
ENSG00000163737	*PF4*	-0.837679687	-0.018495914	0.025620252	-0.044116166	0.999933887	-0.09578744	0.046098508	-0.141885949	0.835020223
ENSG00000188177	*ZC3H6*	3.772931119	0.09567443	-0.108114586	0.203789016	0.999933887	-0.242587633	-0.183518046	-0.059069587	0.835034304
ENSG00000136003	*ISCU*	6.111991189	-0.008734519	-0.058699731	0.049965212	0.999933887	0.047976841	0.019104499	0.028872342	0.835034304
ENSG00000067191	*CACNB1*	1.165045212	0.025821229	-0.134557036	0.160378265	0.999933887	-0.052166382	0.015174905	-0.067341287	0.835306869
ENSG00000165983	*PTER*	1.076350886	0.079625365	-0.066165206	0.145790571	0.999933887	-0.252621789	-0.17746024	-0.075161549	0.835426731
ENSG00000263002	*ZNF234*	0.349997395	-0.213882551	-0.052125159	-0.161757392	0.999933887	-0.034632563	0.040716741	-0.075349304	0.835457339
ENSG00000157927	*RADIL*	-0.702643241	0.031987075	0.387079708	-0.355092632	0.999933887	2.05119794	2.190030326	-0.138832386	0.835457339
ENSG00000110042	*DTX4*	4.585632706	-0.141266085	-0.127558354	-0.013707731	0.999933887	0.257703993	0.189007443	0.06869655	0.83563846
ENSG00000279432	*AC015799.1*	2.313073297	-0.006225919	0.010543783	-0.016769702	0.999933887	0.554417695	0.630576872	-0.076159177	0.835945387
ENSG00000054392	*HHAT*	-0.501924616	-0.16522742	0.090705716	-0.255933135	0.999933887	0.040044731	0.193675008	-0.153630277	0.836072385
ENSG00000173531	*MST1*	0.342055362	0.097373123	0.097952052	-0.000578928	0.999933887	0.061331781	-0.040925754	0.102257535	0.836072385
ENSG00000287812	*AC105046.2*	0.596433626	0.041545751	-0.002627131	0.044172883	0.999933887	0.987466387	0.823863272	0.163603115	0.836072385
ENSG00000272911	*AC005696.3*	-0.01914648	0.324661187	0.365263894	-0.040602707	0.999933887	1.201795265	1.310055467	-0.108260203	0.836097857
ENSG00000204387	*SNHG32*	2.479518049	0.173486737	-0.017969365	0.191456102	0.999933887	0.226259418	0.169020784	0.057238634	0.836097857
ENSG00000125459	*MSTO1*	1.457358863	-0.160436204	-0.10332734	-0.057108864	0.999933887	0.101510511	0.164515115	-0.063004604	0.836097857
ENSG00000096063	*SRPK1*	7.219637542	-0.056828131	-0.007660752	-0.04916738	0.999933887	-0.48570465	-0.521540636	0.035835986	0.836097857
ENSG00000104472	*CHRAC1*	4.294822429	0.022791878	0.007822582	0.014969297	0.999933887	-0.2575232	-0.294348577	0.036825377	0.836097857
ENSG00000189114	*BLOC1S3*	5.905358467	0.017465916	0.069120293	-0.051654377	0.999933887	-0.197256495	-0.226749101	0.029492606	0.836097857
ENSG00000285816	*AP000944.5*	0.37153934	0.005461672	-0.024808227	0.030269899	0.999933887	0.90870185	1.034961972	-0.126260122	0.836097857
ENSG00000127152	*BCL11B*	5.025856738	-0.001629583	0.049359829	-0.050989412	0.999933887	0.042900122	0.006798978	0.036101144	0.836132812
ENSG00000104626	*ERI1*	2.982722351	-0.046090493	-0.038698022	-0.007392471	0.999933887	-0.164604468	-0.226035586	0.061431118	0.836205348
ENSG00000251215	*GOLGA5P1*	0.665142344	-0.404601814	-0.321290434	-0.083311381	0.999933887	-0.676310953	-0.590784071	-0.085526882	0.836212673
ENSG00000096080	*MRPS18A*	1.449385683	0.075937629	0.099371042	-0.023433413	0.999933887	0.190801037	0.134407906	0.056393131	0.836212673
ENSG00000127946	*HIP1*	6.64121787	-0.069553571	-0.038151274	-0.031402297	0.999933887	0.901200814	0.965847124	-0.064646309	0.836377812
ENSG00000272908	*AC006033.2*	-0.642793908	-0.176023106	0.057389924	-0.23341303	0.999933887	0.098041277	-0.048064082	0.146105359	0.836377812
ENSG00000124243	*BCAS4*	1.643293453	0.053896375	0.093774961	-0.039878586	0.999933887	0.028101263	0.096999805	-0.068898542	0.836377812
ENSG00000282420	*TRBJ1-2*	-1.069708116	-0.121033435	-0.096110166	-0.024923269	0.999933887	0.126713595	0.257113901	-0.130400306	0.836377812
ENSG00000168256	*NKIRAS2*	3.934467497	0.034117977	0.136705346	-0.102587369	0.999933887	-0.085609804	-0.126509722	0.040899918	0.836377812
ENSG00000260032	*NORAD*	7.006846731	-0.081804052	-0.09355649	0.011752438	0.999933887	-0.365373762	-0.39657584	0.031202078	0.836511055
ENSG00000089177	*KIF16B*	0.900105969	0.163406863	-0.211584557	0.37499142	0.999933887	0.009918121	0.098617739	-0.088699618	0.836511055
ENSG00000244462	*RBM12*	5.603479609	-0.046089839	0.012510465	-0.058600304	0.999933887	-0.15533952	-0.129279506	-0.026060015	0.836511055
ENSG00000178700	*DHFR2*	0.985001665	-0.119221018	-0.113465461	-0.005755557	0.999933887	-0.206137181	-0.146461341	-0.05967584	0.836511055
ENSG00000042286	*AIFM2*	0.667614734	0.095191564	0.256636909	-0.161445345	0.999933887	0.080318097	0.148238453	-0.067920357	0.836511055
ENSG00000186818	*LILRB4*	2.566757383	-0.058946805	-0.029153112	-0.029793693	0.999933887	-0.495984093	-0.548808481	0.052824387	0.836511055
ENSG00000132205	*EMILIN2*	5.100761019	0.03696214	0.029308646	0.007653495	0.999933887	-0.086476688	-0.129738389	0.0432617	0.836511055
ENSG00000167740	*CYB5D2*	1.626218367	-0.057296147	0.103115677	-0.160411824	0.999933887	0.063393661	0.00232146	0.061072201	0.836511055

ENSG00000176472	*ZNF575*	-0.382337798	-0.089161417	-0.097008366	0.00784695	0.999933887	0.037243627	0.157036576	-0.119792948	0.836511055
ENSG00000159200	*RCAN1*	1.506689428	0.042978144	0.080663302	-0.037685159	0.999933887	-0.313109307	-0.392515159	0.079405851	0.836511055
ENSG00000132664	*POLR3F*	1.343092898	-0.024066172	0.019768499	-0.043834671	0.999933887	0.031137826	-0.03355608	0.064693906	0.836511055
ENSG00000197008	*ZNF138*	0.044851292	-0.040666316	-0.130140222	0.089473906	0.999933887	-0.288000199	-0.417331677	0.129331478	0.836548003
ENSG00000247137	*AP000873.2*	1.656847248	0.161017383	0.025199138	0.135818245	0.999933887	0.100956603	0.032879251	0.068077352	0.836564134
ENSG00000162772	*ATF3*	4.665826984	0.093765894	0.120422955	-0.026657062	0.999933887	-0.016331462	-0.06760454	0.051273078	0.836564134
ENSG00000185684	*EP400P1*	1.393056215	0.096347252	0.062058478	0.034288774	0.999933887	-0.045102333	0.030767357	-0.07586969	0.836564134
ENSG00000120697	*ALG5*	0.976810475	0.044782493	-0.21292672	0.257709213	0.999933887	0.193380059	0.119714006	0.073666054	0.836564134
ENSG00000133256	*PDE6B*	0.532674174	-0.054191114	0.055271104	-0.109462218	0.999933887	0.193147328	0.106971153	0.086176175	0.836564134
ENSG00000163634	*THOC7*	2.773763286	-0.010073226	-0.087491787	0.077418561	0.999933887	-0.012329321	0.042490336	-0.054819657	0.836564134
ENSG00000133835	*HSD17B4*	4.983777026	-0.202221516	-0.049	-0.153221515	0.999933887	-1.272620637	-1.324941301	0.052320664	0.836765116
ENSG00000126860	*EVI2A*	6.480985362	-0.102749578	-0.19600967	0.093260092	0.999933887	-1.103373123	-1.050809513	-0.05256361	0.836944773
ENSG00000163531	*NFASC*	0.001961486	0.009824841	0.122542861	-0.11271802	0.999933887	0.301315152	0.170984649	0.130330502	0.836967234
ENSG00000110906	*KCTD10*	4.282947187	-0.076935778	-0.008544104	-0.068391674	0.999933887	-0.109276802	-0.14030942	0.031032618	0.837019615
ENSG00000271742	*AL121992.2*	-0.240771185	0.301792324	0.188135626	0.113656698	0.999933887	0.939676018	1.050989145	-0.111313128	0.837152973
ENSG00000149328	*GLB1L2*	1.064724972	0.085695416	-0.093106959	0.178802375	0.999933887	-0.043734888	0.03621605	-0.079950938	0.837476921
ENSG00000143842	*SOX13*	1.808312398	-0.056374925	0.033529727	-0.089904651	0.999933887	-0.192297571	-0.24100145	0.048703878	0.837476921
ENSG00000177311	*ZBTB38*	3.994982931	-0.028328652	-0.145717544	0.117388893	0.999933887	-0.224604667	-0.164844372	-0.059760295	0.837476921
ENSG00000234709	*UPF3AP3*	-0.069051062	0.106591855	0.261529272	-0.154937417	0.999933887	-0.001435997	0.161101265	-0.162537262	0.837611723
ENSG00000130772	*MED18*	3.113845905	-0.031627448	-0.171399208	0.13977176	0.999933887	0.204291748	0.155578689	0.048713059	0.837611723
ENSG00000105379	*ETFB*	1.835089444	-0.017355051	0.069820506	-0.087175556	0.999933887	0.192192655	0.2531599	-0.060967245	0.837611723
ENSG00000231535	*LINC00278*	-3.761791396	0.148077183	-0.287697425	0.435774608	0.999933887	-0.458665263	-0.556740845	0.098075582	0.837679461
ENSG00000197461	*PDGFA*	0.236406574	-0.145782512	-0.235360082	0.08957757	0.999933887	0.313506183	0.419476903	-0.105970719	0.837801469
ENSG00000062524	*LTK*	1.236139678	-0.048975306	0.128752319	-0.177727625	0.999933887	0.112287734	0.192677055	-0.080389321	0.837801469
ENSG00000215883	*CYB5RL*	-0.397204683	-0.245483057	0.340321529	-0.585804586	0.999933887	-0.047243087	-0.15626448	0.109021393	0.838062629
ENSG00000175193	*PARL*	0.531531854	-0.129303094	-0.050099932	-0.079203162	0.999933887	0.087451069	-0.015848209	0.103299278	0.8381075
ENSG00000100890	*PRORP*	0.745395159	0.147513988	0.003508068	0.14400592	0.999933887	0.107563332	0.032267409	0.075295923	0.838154449
ENSG00000181061	*HIGD1A*	2.48021113	-0.057114381	0.0609866	-0.118100981	0.999933887	0.062978777	0.009825573	0.053153204	0.838154449
ENSG00000094914	*AAAS*	3.312725466	-0.051235724	0.033549631	-0.084785355	0.999933887	0.007012245	0.048095602	-0.041083358	0.838380616
ENSG00000162086	*ZNF75A*	2.506622089	-0.104543008	-0.124511084	0.019968076	0.999933887	-0.174782115	-0.21999581	0.045213694	0.838628978
ENSG00000170222	*ADPRM*	1.797844632	0.10971294	-0.046653696	0.156366637	0.999933887	-0.029584973	0.029989818	-0.05957479	0.838657809
ENSG00000225792	*AC004540.2*	0.48590996	-0.165589816	0.07917022	-0.244760036	0.999933887	2.278384546	2.416267884	-0.137883338	0.838657809
ENSG00000103995	*CEP152*	1.384211334	-0.060553385	-0.118324393	0.057771008	0.999933887	-0.145249397	-0.046975216	-0.098274181	0.838815532
ENSG00000226287	*TMEM191A*	0.545427565	0.015428267	0.023883722	-0.008455455	0.999933887	0.078204147	-0.010335365	0.088539513	0.839048114
ENSG00000233461	*AL445524.1*	1.537223083	0.049253281	-0.008747386	0.058000668	0.999933887	0.038803781	-0.055289196	0.094092977	0.839058439
ENSG00000104381	*GDAP1*	0.002728631	-0.140637139	0.000843578	-0.141480717	0.999933887	0.134507463	0.024590921	0.109916542	0.839305998
ENSG00000114988	*LMAN2L*	1.159460384	0.060627833	-0.034236362	0.094864195	0.999933887	-0.157764245	-0.103531104	-0.054233142	0.839305998
ENSG00000250739	*LINC01262*	1.199776628	0.221267808	0.298360402	-0.077092594	0.999933887	1.853329963	1.960170499	-0.106840536	0.839476536
ENSG00000280407	*AC132872.4*	4.01591697	0.046393241	0.119509945	-0.073116704	0.999933887	0.012926492	-0.036062058	0.048988551	0.839476536
ENSG00000136404	*TM6SF1*	3.280000294	0.072912681	-0.060059138	0.132971819	0.999933887	-0.576715598	-0.628717545	0.052001947	0.839531134
ENSG00000065491	*TBC1D22B*	2.26277401	-0.0444452	0.006951795	-0.051396995	0.999933887	-0.005865522	0.047189444	-0.053054966	0.839531134
ENSG00000163382	*NAXE*	1.950168005	-0.006711575	-0.017440702	0.010729127	0.999933887	0.13153219	0.064850299	0.066681892	0.839531134
ENSG00000260417	*AC092127.1*	-0.237392886	0.040496825	0.157611989	-0.117115164	0.999933887	0.483907272	0.63636976	-0.152462488	0.839901277
ENSG00000173890	*GPR160*	5.277136815	0.057750858	0.052300293	0.005450565	0.999933887	-0.952768999	-0.904669382	-0.048099618	0.839997455
ENSG00000042062	*RIPOR3*	0.765552032	0.06460444	0.068064153	-0.003459713	0.999933887	0.369613419	0.518445857	-0.148832437	0.840004167
ENSG00000006025	*OSBPL7*	3.564398768	0.018931206	-0.019368693	0.038299898	0.999933887	0.096771628	0.059715807	0.03705582	0.840004167
ENSG00000277283	*AC004812.2*	0.444517406	-0.052440437	0.125349504	-0.177789941	0.999933887	0.478070624	0.374492049	0.103578575	0.840004167
ENSG00000226791	*LINC02611*	-0.613830809	0.045146068	-0.209087077	0.254233145	0.999933887	0.161533212	0.281682307	-0.120149096	0.840004167
ENSG00000196169	*KIF19*	0.5328252	0.081881645	-0.054603917	0.136485562	0.999933887	0.064129832	-0.010427647	0.074557479	0.8400169
ENSG00000230068	*CDC42-IT1*	-0.307006488	0.079154831	-0.010205105	0.089359936	0.999933887	0.181078643	0.311516672	-0.130438028	0.8400169
ENSG00000137460	*FHDC1*	3.456233788	0.016495026	-0.001210246	0.017705272	0.999933887	0.403216426	0.439414372	-0.036197946	0.8400169
ENSG00000254211	*LINC01485*	0.078168792	0.020050522	0.205397019	-0.185346497	0.999933887	0.464428031	0.385288004	0.079140027	0.8400169
ENSG00000051009	*FAM160A2*	6.240408737	0.057464792	0.016244769	0.041220023	0.999933887	1.144311614	1.197644312	-0.053332698	0.840070552
ENSG00000285554	*AC242988.2*	0.468452868	-0.003114808	-0.00492244	0.001807632	0.999933887	0.287711402	0.388255651	-0.100544249	0.840070552
ENSG00000262870	*CYCSP40*	0.407415704	-0.167656086	-0.038734694	-0.128921392	0.999933887	-0.516826342	-0.415406735	-0.101419608	0.840078191
ENSG00000183161	*FANCF*	0.694962108	0.027233465	0.054189623	-0.026956158	0.999933887	0.069749955	-0.002716903	0.072466858	0.840092193
ENSG00000168014	*C2CD3*	4.846131185	-0.014363096	-0.040422407	0.02605931	0.999933887	-0.097936171	-0.068862259	-0.029073912	0.840180281
ENSG00000235172	*LINC01366*	4.352811525	0.177584521	0.160279212	0.017305309	0.999933887	1.479384472	1.533986817	-0.054602345	0.840505524
ENSG00000012963	*UBR7*	2.699787558	-0.08193517	0.032315001	-0.114250171	0.999933887	0.033926631	0.084353263	-0.050426632	0.840585889
ENSG00000077312	*SNRPA*	3.259690121	-0.020367616	-0.017074258	-0.003293358	0.999933887	0.012894959	-0.026920326	0.039815285	0.840585889
ENSG00000109586	*GALNT7*	3.501120413	0.007214305	-0.148560522	0.155774827	0.999933887	-0.090857858	-0.040745875	-0.050111983	0.840585889
ENSG00000269981	*AL627309.7*	4.16487672	0.073946681	-0.03143523	0.10538191	0.999933887	0.312642026	0.261400732	0.051241294	0.840887473
ENSG00000156858	*PRR14*	6.338256423	0.045033793	0.038223652	0.006810141	0.999933887	0.473594133	0.503584851	-0.029990717	0.840887473
ENSG00000211695	*TRGV9*	-0.027409412	-0.030964007	-0.057019912	0.026055905	0.999933887	0.174892098	0.090626432	0.084265666	0.840887473
ENSG00000139737	*SLAIN1*	1.480023109	0.115974585	-0.007610961	0.123585546	0.999933887	0.05422502	-0.008665859	0.062890878	0.840897401
ENSG00000063177	*RPL18*	7.328951388	0.012261725	-0.028659796	0.040921521	0.999933887	0.089924703	0.121693521	-0.031768818	0.841043405
ENSG00000268858	*AL118506.1*	4.384688318	-0.010080192	0.109895205	-0.119975397	0.999933887	-0.342486787	-0.301162773	-0.041324014	0.8412405
ENSG00000198113	*TOR4A*	4.806768508	0.064549704	0.089954698	-0.025404993	0.999933887	-0.462402955	-0.506583589	0.044180633	0.841463086
ENSG00000163913	*IFT122*	2.719709638	-0.037633956	0.015231081	-0.052865037	0.999933887	0.010178414	-0.041250434	0.051428848	0.841624137
ENSG00000226259	*GTF2H2B*	-0.723716482	0.066728645	-0.183737668	0.250466313	0.999933887	-0.581732071	-0.722746681	0.141014611	0.841624137
ENSG00000235954	*TTC28-AS1*	1.61676261	0.023760323	0.203895812	-0.180135489	0.999933887	-0.053748061	-0.119957395	0.066209334	0.841624137
ENSG00000177374	*HIC1*	3.628198081	-0.009149857	-0.009694323	0.000544466	0.999933887	0.181656381	0.237408005	-0.055751624	0.841624137
ENSG00000099810	*MTAP*	0.934872239	-0.006503686	0.289251953	-0.295755639	0.999933887	-0.051285832	0.020272401	-0.071558233	0.841958546
ENSG00000185189	*NRBP2*	1.733601024	0.173612322	0.056816377	0.116795945	0.999933887	0.013699809	0.067552354	-0.053852546	0.841964782
ENSG00000280587	*LINC01348*	-0.717916492	0.108449182	-0.201799667	0.310248849	0.999933887	2.378504417	2.21141497	0.167089447	0.842018816
ENSG00000276961	*MIR7848*	-0.118261659	-0.028630286	-0.095468545	0.06683826	0.999933887	0.155147069	-0.007898999	0.163046069	0.84214854
ENSG00000169189	*NSMCE1*	3.216562569	-0.061070032	-0.095496776	0.034426743	0.999933887	-0.032236069	0.00875569	-0.04099176	0.842151091
ENSG00000005486	*RHBDD2*	5.670805785	0.034032512	-0.000273581	0.034306093	0.999933887	-0.013219418	-0.042283238	0.029063821	0.842158553
ENSG00000129472	*RAB2B*	2.803698822	-0.091165024	-0.075317921	-0.015847103	0.999933887	0.12187064	0.073582735	0.048287906	0.842251015
ENSG00000167840	*ZNF232*	-0.6973944	-0.158967666	0.191280861	-0.350248527	0.999933887	0.300194728	0.417890179	-0.117695452	0.842251015
ENSG00000181894	*ZNF329*	1.29730142	-0.099438106	-0.193499961	0.094061855	0.999933887	-0.074237239	-0.005413723	-0.068823516	0.842408964
ENSG00000091536	*MYO15A*	-0.382639443	0.098957322	-0.019695939	0.118653261	0.999933887	-0.206205747	-0.075654919	-0.130550828	0.842605936
ENSG00000250317	*SMIM20*	1.389609619	0.037675965	0.000951533	0.036724432	0.999933887	0.059538823	0.131309038	-0.071770215	0.842605936
ENSG00000251586	*TET2-AS1*	-0.071896071	0.173229255	0.490857017	-0.317627763	0.999933887	0.021063467	0.176064246	-0.155000779	0.84274191
ENSG00000228719	*AL022313.2*	-1.322920868	0.443040563	0.242094405	0.200946157	0.999933887	0.624753716	0.383606242	0.241147474	0.842866013
ENSG00000175928	*LRRN1*	0.002871492	0.04092968	-0.125060346	0.165990027	0.999933887	-0.742779538	-0.843223045	0.100443507	0.842955874
ENSG00000170949	*ZNF160*	3.778970924	-0.022802509	-0.071987986	0.049185477	0.999933887	-0.482622221	-0.433304414	-0.049317808	0.843007095
ENSG00000175445	*LPL*	0.577297202	-0.001127594	-0.205433087	0.204305493	0.999933887	-0.963415511	-1.041053585	0.077638074	0.843101662
ENSG00000109743	*BST1*	6.303015747	-0.021455515	0.008597027	-0.030052543	0.999933887	-0.352699888	-0.319521437	-0.033178451	0.843140182
ENSG00000198576	*ARC*	-0.176409814	-0.183637518	-0.129984786	-0.053652732	0.999933887	-0.125159599	-0.027443733	-0.097715866	0.843153598
ENSG00000225697	*SLC26A6*	3.522568911	-0.052597647	0.015978737	-0.068576384	0.999933887	-0.063732162	-0.110141632	0.04640947	0.843575364
ENSG00000249673	*NOP14-AS1*	2.920011874	0.061714013	-0.016840667	0.078554679	0.999933887	0.053842521	0.003799205	0.050043316	0.843676263
ENSG00000173933	*RBM4*	3.541897527	-0.013903466	-0.039232883	0.025329417	0.999933887	0.063991214	0.028592048	0.035399165	0.843676263
ENSG00000117362	*APH1A*	6.395187056	-0.010424211	-0.00657459	-0.00384962	0.999933887	-0.115137794	-0.141009382	0.025871588	0.843676263
ENSG00000244734	*HBB*	8.098226389	-0.103908279	-0.108651465	0.004743187	0.999933887	0.082009803	0.140273612	-0.058263809	0.843830609
ENSG00000139350	*NEDD1*	2.275750374	0.101229695	-0.03579753	0.137027224	0.999933887	-0.235871213	-0.131827605	-0.104043608	0.843830609
ENSG00000141376	*BCAS3*	3.254805611	0.007953874	0.044906774	-0.0369529	0.999933887	0.066123817	0.023748842	0.042374975	0.844336319
ENSG00000161970	*RPL26*	3.695693902	-0.101576097	-0.115655701	0.014079604	0.999933887	0.100001295	0.14256634	-0.042565045	0.84435925
ENSG00000137266	*SLC22A23*	0.143508222	0.056017858	0.041315584	0.014702274	0.999933887	0.114722522	0.010838265	0.103884257	0.84435925
ENSG00000135046	*ANXA1*	7.468454852	-0.079879897	-0.187629068	0.107749171	0.999933887	-0.09551932	-0.060213797	-0.035305523	0.84482782
ENSG00000149100	*EIF3M*	4.186076086	-0.048307465	-0.145317877	0.097010412	0.999933887	0.024384247	0.060229814	-0.035845567	0.844939126
ENSG00000268362	*AC092279.1*	5.071329704	-0.011836177	0.058143209	-0.069979386	0.999933887	-0.294385134	-0.356459365	0.062074231	0.844939126
ENSG00000085982	*USP40*	1.168694827	-0.051952289	0.002976248	-0.054928537	0.999933887	0.059396654	0.127290437	-0.067893782	0.8451025
ENSG00000274523	*RCC1L*	2.264657448	0.023554249	0.12854822	-0.104993971	0.999933887	0.206326079	0.16373662	0.042589459	0.8451025
ENSG00000185896	*LAMP1*	8.814551765	0.020237368	-0.003742779	0.023980147	0.999933887	0.148971807	0.172940396	-0.023968589	0.845247275

ENSG00000213277	*MARCKSL1P1*	0.264459812	-0.094593041	-0.037247561	-0.05734548	0.999933887	0.314445609	0.391789942	-0.077344332	0.845280339
ENSG00000251682	*AC122718.2*	0.407609251	0.260148187	0.492271371	-0.232123184	0.999933887	0.450795966	0.279486585	0.171309381	0.845295155
ENSG00000150687	*PRSS23*	1.720830146	-0.047723581	-0.194281573	0.146557992	0.999933887	0.017843778	0.073347953	-0.055504175	0.845342578
ENSG00000184007	*PTP4A2*	7.39635215	-0.04205284	-0.014661975	-0.027390865	0.999933887	-0.174679019	-0.201211152	0.026532133	0.845490491
ENSG00000172428	*COPS9*	2.502503573	0.068493284	-0.055863403	0.124356687	0.999933887	-0.01395911	-0.068650679	0.054691569	0.845490491
ENSG00000099250	*NRP1*	0.317975675	-0.137302269	0.270445733	-0.407748002	0.999933887	-0.706548516	-0.648087366	-0.05846115	0.845706183
ENSG00000196943	*NOP9*	2.966356706	-0.000787378	-0.064005042	0.063217664	0.999933887	0.16503876	0.124185968	0.040852792	0.845706183
ENSG00000151240	*DIP2C*	0.004908462	-0.401087107	-0.166427266	-0.234659841	0.999933887	-0.190259517	-0.08381347	-0.106446047	0.845742665
ENSG00000286724	*AL732509.1*	0.06052086	0.215091744	0.032973614	0.18211813	0.999933887	-0.413138094	-0.522550015	0.109411921	0.845742665
ENSG00000116406	*EDEM3*	4.568783275	0.004274685	-0.12673195	0.131006635	0.999933887	-0.460682013	-0.395921682	-0.064760331	0.845742665
ENSG00000141873	*SLC39A3*	2.596880936	-0.033975517	-0.074335745	0.040360228	0.999933887	-0.359770841	-0.409189978	0.049419136	0.845852696
ENSG00000008130	*NADK*	9.749815879	0.000351641	0.016587051	-0.016235409	0.999933887	0.141884239	0.121664667	0.020219572	0.845852696
ENSG00000179862	*CITED4*	1.973016732	0.273573621	0.100017339	0.173556282	0.999933887	2.264423967	2.155712288	0.108711679	0.845978095
ENSG00000081181	*ARG2*	-0.724913244	-0.042842176	0.079646052	-0.122488228	0.999933887	0.264584327	0.413490388	-0.148906062	0.846040153
ENSG00000256043	*CTSO*	1.328694994	-0.025407382	-0.187353293	0.161945911	0.999933887	-0.117574888	-0.058198079	-0.05937681	0.846173439
ENSG00000260368	*AC027373.1*	0.142371194	0.190168278	0.393749865	-0.203581586	0.999933887	0.034048103	-0.079665463	0.113713565	0.846185861
ENSG00000179364	*PACS2*	3.249900223	0.03429556	0.063995106	-0.029699546	0.999933887	0.01395747	-0.022037503	0.035994973	0.846185861
ENSG00000204599	*TRIM39*	4.675339612	-0.026941626	0.016557014	-0.04349864	0.999933887	0.190068772	0.159330233	0.030738539	0.846185861
ENSG00000159596	*TMEM69*	1.384577869	0.008725335	0.011927686	-0.003202351	0.999933887	0.096028553	0.045549868	0.050478686	0.846185861
ENSG00000213930	*GALT*	2.560787305	-0.035881948	0.152113314	-0.187995263	0.999933887	0.026193071	0.075170565	-0.048977494	0.846442621
ENSG00000261245	*AC093520.2*	3.111455904	0.011825796	0.097166616	-0.085340821	0.999933887	0.017088867	0.07041329	-0.053324423	0.846442621
ENSG00000083817	*ZNF416*	-0.309293956	-0.020639313	0.04138708	-0.062026392	0.999933887	0.302441165	0.401886516	-0.099445351	0.846511676
ENSG00000231527	*FAM27C*	-1.276530719	0.078045268	0.059068437	0.018976831	0.999933887	-0.328783734	-0.435987957	0.107204223	0.846765492
ENSG00000248489	*LINC02062*	1.231053335	0.122487169	0.202887241	-0.080400072	0.999933887	0.413220083	0.488143356	-0.074923273	0.846875389
ENSG00000151881	*TMEM267*	0.353111003	0.137076088	0.33756449	-0.200488402	0.999933887	0.021584987	0.117444475	-0.095859488	0.846918163
ENSG00000140451	*PIF1*	-0.403824079	0.055577155	0.181211291	-0.125634136	0.999933887	0.092310601	0.230064829	-0.137754228	0.846964686
ENSG00000140988	*RPS2*	8.213998115	0.070103502	0.007706852	0.06239665	0.999933887	0.092321108	0.057383611	0.034937496	0.847032
ENSG00000090104	*RGS1*	4.928223245	-0.059576252	-0.094137725	0.034561472	0.999933887	-0.022713488	0.025256361	-0.047969849	0.847032
ENSG00000250479	*CHCHD10*	1.698331848	0.090454646	0.049984241	0.040470404	0.999933887	0.066116946	0.135633464	-0.069516518	0.847032
ENSG00000132394	*EEFSEC*	1.129400735	0.089104254	0.006329566	0.082774688	0.999933887	0.18834191	0.099862761	0.088479149	0.847032
ENSG00000285534	*AL163541.1*	-0.07847177	0.048739995	-0.000825278	0.049565273	0.999933887	0.04375125	0.140788453	-0.097037203	0.847032
ENSG00000211792	*TRAV14DV4*	-0.286831552	0.045883006	0.132994832	-0.087111826	0.999933887	0.284026071	0.18774912	0.096276951	0.847032
ENSG00000120519	*SLC10A7*	0.760539306	-0.021446853	0.054054026	-0.075500879	0.999933887	-0.143747627	-0.06070482	-0.083042807	0.847032
ENSG00000099834	*CDHR5*	-0.5437752	0.021684807	-0.059942409	0.081627216	0.999933887	-0.118060978	0.0072309	-0.125291878	0.847032
ENSG00000135404	*CD63*	10.284401809	0.001842246	-0.011200275	0.013042522	0.999933887	0.089567612	0.061779924	0.027787688	0.847032
ENSG00000178404	*CEP295NL*	3.247866322	-0.002448577	0.001487516	-0.003936093	0.999933887	-0.844906648	-0.787650411	-0.057256236	0.847032
ENSG00000130332	*LSM7*	2.968424268	0.001482482	-0.058649791	0.060132273	0.999933887	0.05992489	0.019119474	0.040805415	0.847032
ENSG00000122188	*LAX1*	3.220070042	0.028170697	0.02409508	0.004075617	0.999933887	0.081026014	0.118750587	-0.037724573	0.847169072
ENSG00000149179	*C11orf49*	1.44999256	0.089679582	0.157475558	-0.067795977	0.999933887	-0.164820569	-0.107770181	-0.057050387	0.847229582
ENSG00000185386	*MAPK11*	0.356046353	0.006858858	0.033702729	-0.026843871	0.999933887	0.310590206	0.228494324	0.082095882	0.847229582
ENSG00000143382	*ADAMTSL4*	6.209249924	0.083538095	0.086167128	-0.002629034	0.999933887	0.190226374	0.154183967	0.036042407	0.84732579
ENSG00000006125	*AP2B1*	6.425233825	-0.046823596	-0.056778631	0.009955036	0.999933887	-0.139558577	-0.111802559	-0.027756019	0.84732579
ENSG00000120533	*ENY2*	4.787325874	-0.038355019	-0.06760592	0.029250901	0.999933887	-0.112115534	-0.0856834	-0.026432134	0.84732579
ENSG00000179841	*AKAP5*	0.000521616	0.099506594	0.359279493	-0.259772899	0.999933887	-0.119873885	-0.019282109	-0.100591776	0.84732579
ENSG00000037241	*RPL26L1*	1.480423717	-0.035418129	-0.010005118	-0.025413011	0.999933887	0.117037049	0.06376571	0.05327134	0.84732579
ENSG00000196700	*ZNF512B*	2.698910201	0.029674262	0.136787362	-0.1071131	0.999933887	0.104819509	0.146348067	-0.041528558	0.847355117
ENSG00000125631	*HTR5BP*	0.445172578	0.096377762	0.059194735	0.037183027	0.999933887	0.12128278	0.042537987	0.078744794	0.847375251
ENSG00000028116	*VRK2*	1.910267306	-0.113287653	-0.011391252	-0.1018964	0.999933887	-0.296204439	-0.376766674	0.080562235	0.847810274
ENSG00000109606	*DHX15*	5.703712606	-0.08359892	-0.115308905	0.031709986	0.999933887	-0.212807401	-0.174219296	-0.038588105	0.847834408
ENSG00000243753	*HLA-L*	4.609554127	0.109670108	0.076865753	0.032804355	0.999933887	1.178129784	1.261657602	-0.083527819	0.847834408
ENSG00000237649	*KIFC1*	-0.660236911	-0.099209559	-0.150304749	0.051095191	0.999933887	0.209399848	0.055487349	0.153912499	0.847834408
ENSG00000287095	*AF228727.1*	1.179865904	-0.072113078	0.129680978	-0.201794056	0.999933887	-0.709840937	-0.606219962	-0.103620976	0.847950961
ENSG00000162069	*BICDL2*	-0.356477856	0.058690253	0.097611625	-0.038921372	0.999933887	-0.355500111	-0.495556836	0.140056725	0.847950961
ENSG00000287185	*AC041005.1*	1.339513047	-0.064274528	-0.029077246	-0.035197282	0.999933887	-0.553719566	-0.484765207	-0.068954359	0.848064383
ENSG00000228436	*AL139260.1*	1.582439681	0.022956197	-0.333305531	0.356261728	0.999933887	0.270512245	0.219201482	0.051310763	0.848192543
ENSG00000136877	*FPGS*	3.569710818	0.046728172	0.115089146	-0.068360973	0.999933887	0.105305812	0.139377113	-0.034071302	0.848370808
ENSG00000153066	*TXNDC11*	5.376778937	-0.016279017	-0.009677045	-0.006601971	0.999933887	-0.028050689	0.003094438	-0.031145127	0.848370808
ENSG00000159199	*ATP5MC1*	1.64300991	-0.003083151	0.143085879	-0.146169031	0.999933887	0.2354695	0.29397743	-0.058507929	0.848370808
ENSG00000255521	*AL356215.1*	2.521464606	0.143810616	0.230382348	-0.086571731	0.999933887	2.561831301	2.636675671	-0.07484437	0.848378316
ENSG00000104613	*INTS10*	3.511338586	0.000578643	-0.002241979	0.002820622	0.999933887	0.089971818	0.05699126	0.032980558	0.848378316
ENSG00000160613	*PCSK7*	5.212731068	0.038920903	0.038828144	9.2759e-05	0.999933887	-0.005199021	0.020687968	-0.025886989	0.848939375
ENSG00000235897	*TM4SF19-AS1*	-0.155756439	-0.077504828	-0.236365622	0.158860793	0.999933887	0.037564729	-0.037440316	0.075005045	0.848939375
ENSG00000109099	*PMP22*	-0.520608422	-0.187318901	0.068487788	-0.255806689	0.999933887	-0.009470639	0.149979649	-0.159450288	0.848972774
ENSG00000151948	*GLT1D1*	7.96626045	-0.017298542	-0.012965057	-0.004333485	0.999933887	-0.371291617	-0.398461818	0.027170201	0.849246891
ENSG00000102034	*ELF4*	7.40808578	-0.005609895	-0.010963992	0.005354097	0.999933887	-0.507366774	-0.480025656	-0.027341118	0.849246891
ENSG00000100852	*ARHGAP5*	1.563560971	-0.040891068	-0.203752055	0.162860988	0.999933887	-0.304011903	-0.194879321	-0.109132582	0.849336503
ENSG00000122674	*CCZ1*	1.560864059	0.032200591	0.030078921	0.00212167	0.999933887	-0.347622025	-0.412465731	0.064843706	0.849992043
ENSG00000054598	*FOXC1*	-0.198459672	0.070339193	0.109021981	-0.038682788	0.999933887	0.113035631	0.233392002	-0.12035637	0.850525955
ENSG00000141551	*CSNK1D*	8.972499483	-0.009512952	0.019385724	-0.028898676	0.999933887	-0.1570026	-0.18104053	0.02403793	0.850525955
ENSG00000124532	*MRS2*	1.254529905	-0.100197601	-0.108183092	0.007985492	0.999933887	-0.057707589	0.00940428	-0.067111868	0.850564355
ENSG00000136856	*SLC2A8*	0.64646739	0.021271717	-0.090628393	0.11190011	0.999933887	-0.295883252	-0.214444755	-0.081438497	0.850564355
ENSG00000259826	*AC072061.1*	-0.240566971	0.215434008	-0.061533966	0.276967974	0.999933887	-0.208787009	-0.328162497	0.119375488	0.850861535
ENSG00000115761	*NOL10*	1.67885585	-0.083240003	-0.019760791	-0.063479213	0.999933887	0.012111575	-0.040881221	0.052992796	0.85086913
ENSG00000186642	*PDE2A*	2.98619478	0.137306166	0.114364859	0.022941306	0.999933887	0.626197697	0.575394422	0.050803275	0.851188763
ENSG00000170006	*TMEM154*	8.221837308	-0.029423325	-0.035666988	0.006243663	0.999933887	-0.261707092	-0.301089881	0.039382789	0.851188763
ENSG00000182809	*CRIP2*	1.143384909	-0.036165421	0.191394864	-0.227560284	0.999933887	0.170724313	0.246922971	-0.076198658	0.851188763
ENSG00000111639	*MRPL51*	2.575288396	-0.013931562	-0.058609846	0.044678284	0.999933887	-0.086437865	-0.126646149	0.040208284	0.851272393
ENSG00000129116	*PALLD*	0.542079238	0.347308649	0.133352334	0.213956315	0.999933887	-0.010952878	-0.081800197	0.070847319	0.851351137
ENSG00000166105	*GLB1L3*	0.18770365	0.162985986	0.028686716	0.13429927	0.999933887	0.018044474	-0.077708934	0.095753408	0.851351137
ENSG00000234072	*AC074117.1*	0.322805249	-0.183368299	0.330466741	-0.513835039	0.944094537	-0.423259858	-0.354680056	-0.068579803	0.851351137
ENSG00000177370	*TIMM22*	1.782294367	-0.119824014	0.043089049	-0.162913063	0.999933887	-0.054107838	-0.002769269	-0.051338569	0.851351137
ENSG00000156869	*FRRS1*	1.694115449	-0.104380792	-0.003809764	-0.100571028	0.999933887	-0.431859391	-0.499744826	0.067885436	0.851351137
ENSG00000042317	*SPATA7*	-1.016553854	-0.166810025	0.399211316	-0.566021341	0.999933887	-1.005242161	-0.896533136	-0.108709025	0.851351137
ENSG00000106993	*CDC37L1*	2.483005178	-0.059296209	-0.030355758	-0.028940452	0.999933887	-0.079456843	-0.12406895	0.044612107	0.851351137
ENSG00000105374	*NKG7*	6.666300906	0.035679859	-0.047884451	0.08356431	0.999933887	0.055712431	0.086256907	-0.030544476	0.851351137
ENSG00000100418	*DESI1*	4.531447091	-0.033246355	-0.03688805	0.003641695	0.999933887	-0.099485463	-0.068574347	-0.030911116	0.851351137
ENSG00000232021	*LEF1-AS1*	-0.979118786	0.120270063	-0.189693151	0.309963213	0.999933887	0.119246776	0.024611031	0.094635745	0.851351137
ENSG00000008438	*PGLYRP1*	4.112391816	0.008782544	-0.005343665	0.014126209	0.999933887	0.033569252	-0.010754651	0.044323902	0.851351137
ENSG00000279412	*AC020763.3*	1.001078123	0.061866372	-0.072990366	0.134856737	0.999933887	-0.157279525	-0.216359865	0.05908034	0.851450536
ENSG00000006432	*MAP3K9*	1.244698225	-0.115411131	-0.02496993	-0.0904412	0.999933887	-0.064464496	-0.132721232	0.068256735	0.851479147
ENSG00000170855	*TRIAP1*	1.304018882	-0.072342446	-0.131229248	0.058886802	0.999933887	0.165883351	0.103754359	0.062128991	0.851566615
ENSG00000169288	*MRPL1*	0.803521404	0.016270617	-0.277537873	0.29380849	0.999933887	0.091706793	0.003342295	0.088364498	0.851566615
ENSG00000163257	*DCAF16*	3.999925035	-0.081411721	-0.049373976	-0.032037745	0.999933887	-0.018271909	-0.050730273	0.032458364	0.85164596
ENSG00000198369	*SPRED2*	2.577318954	-0.068663524	-0.017924588	-0.050738935	0.999933887	0.090163539	0.151308327	-0.061144788	0.85164596
ENSG00000285653	*AC006238.2*	-0.51886164	0.023575206	0.148147199	-0.124571993	0.999933887	-0.497596011	-0.361364418	-0.136231593	0.85164596
ENSG00000165389	*SPTSSA*	2.427006143	-0.037611757	0.02020011	-0.057811867	0.999933887	-0.297074596	-0.257167737	-0.03990686	0.851729819
ENSG00000238057	*ZEB2-AS1*	1.909066149	0.29424061	0.252723478	0.041517132	0.999933887	1.773465273	1.845454413	-0.07198914	0.851849833
ENSG00000270673	*YTHDF3-AS1*	0.074276235	0.214967792	-0.056070016	0.271037808	0.999933887	1.012998924	1.154827382	-0.141828457	0.851849833
ENSG00000188001	*TPRG1*	1.008472342	0.087387485	-0.33428007	0.421667555	0.999933887	0.064112269	-0.000891851	0.065004121	0.851849833
ENSG00000177455	*CD19*	-0.641643649	-0.10437393	-0.045179528	-0.059194401	0.999933887	-0.124132154	-0.00630779	-0.117824364	0.852137011
ENSG00000231123	*SPATA20P1*	3.215615601	-0.018031526	0.217760829	-0.235792355	0.999933887	-0.447616114	-0.352504058	-0.095112055	0.852137011
ENSG00000262898	*AC139099.2*	0.159490353	0.281313153	0.135464254	0.145848899	0.999933887	0.274273771	0.372305384	-0.098031613	0.85257964
ENSG00000107815	*TWNK*	0.802161678	-0.087520759	-0.090430048	0.002909289	0.999933887	0.242989649	0.311535819	-0.06854617	0.85257964
ENSG00000140511	*HAPLN3*	2.997170246	-0.028154439	0.009381569	-0.037536008	0.999933887	0.215737635	0.263897922	-0.048160287	0.85257964
ENSG00000120738	*EGR1*	6.622652301	0.639689151	0.674933952	-0.035244801	0.999933887	0.693767569	0.791317444	-0.097549875	0.852709354

ENSG00000160716	*CHRNB2*	0.551710113	0.199666837	0.158492832	0.041174005	0.999933887	0.333111282	0.431891924	-0.098780642	0.852766171
ENSG00000198133	*TMEM229B*	1.70303279	0.10803008	0.084897715	0.023132365	0.999933887	-0.061868562	-0.112450135	0.050581573	0.852774822
ENSG00000117533	*VAMP4*	2.689986526	-0.064636163	-0.143143056	0.078506894	0.999933887	-0.316875209	-0.374806308	0.057931099	0.852864094
ENSG00000149131	*SERPING1*	3.99193876	0.013048346	-0.00745153	0.020499875	0.999933887	-0.224153383	-0.277791686	0.053638303	0.852864094
ENSG00000236935	*AP003774.2*	-0.487356114	0.017581356	0.168835559	-0.151254203	0.999933887	-0.183315083	-0.064320652	-0.118994431	0.852864094
ENSG00000256843	*AC023157.1*	0.90134298	0.371078121	0.380076791	-0.00899867	0.999933887	0.771387021	0.84403857	-0.072651549	0.852885066
ENSG00000013523	*ANGEL1*	1.717653098	-0.026572155	-0.037624754	0.011052599	0.999933887	0.083494044	0.02657479	0.056919254	0.85310696
ENSG00000259293	*LIPC-AS1*	2.41281997	0.103918383	0.066385825	0.037532558	0.999933887	0.235305503	0.281675819	-0.046370316	0.853133046
ENSG00000137261	*KIAA0319*	4.181560159	-0.121964186	0.006851579	-0.128815765	0.999933887	-0.401864028	-0.436437457	0.034573429	0.853220036
ENSG00000182600	*SNORC*	-0.0991895	0.152849641	0.413626693	-0.260777053	0.999933887	-0.329176095	-0.225772653	-0.103403442	0.853220036
ENSG00000152944	*MED21*	3.391155896	0.049949463	-0.069904849	0.119854311	0.999933887	-0.445630512	-0.393246566	-0.052383946	0.853220036
ENSG00000274211	*SOCS7*	2.697480051	-0.041406854	0.156091732	-0.197498586	0.999933887	-0.098724195	-0.147710763	0.048986569	0.853220036
ENSG00000139508	*SLC46A3*	2.16765405	-0.024962943	-0.108484035	0.083521092	0.999933887	0.082855188	0.138743441	-0.055888253	0.853220036
ENSG00000233668	*AL353662.1*	0.739399214	0.01250546	0.035940709	-0.023435249	0.999933887	0.352168569	0.478141545	-0.125972975	0.853220036
ENSG00000197879	*MYO1C*	2.735476688	0.010591694	0.12870224	-0.118110546	0.999933887	0.399359612	0.449815654	-0.050456042	0.853220036
ENSG00000164953	*TMEM67*	-0.11535956	0.210760169	0.219072533	-0.008312364	0.999933887	-0.331240963	-0.213709894	-0.117531069	0.853640353
ENSG00000276758	*AC245884.12*	0.506435193	-0.033155885	-0.071678994	0.038523108	0.999933887	-0.117902759	-0.23151614	0.113613381	0.853726522
ENSG00000165209	*STRBP*	2.009453889	-0.123315469	-0.029264566	-0.094050903	0.999933887	-0.25017168	-0.295657542	0.045485862	0.854372124
ENSG00000280385	*AP000648.4*	0.67780374	-0.092988386	-0.135348146	0.04235976	0.999933887	-0.258166513	-0.120290029	-0.137876484	0.854483321
ENSG00000162076	*FLYWCH2*	0.5950672	0.043330387	0.027749256	0.015581132	0.999933887	0.040770169	0.132089452	-0.091319283	0.854611949
ENSG00000273824	*AC008033.3*	-0.996923859	0.042286023	-0.070451814	0.112737837	0.999933887	0.191200327	0.298457918	-0.107257592	0.854716739
ENSG00000164291	*ARSK*	-0.336317676	-0.217196582	-0.36069972	0.143503139	0.999933887	-0.342187982	-0.431388593	0.089200612	0.854724219
ENSG00000246334	*PRR7-AS1*	2.913756199	0.067966696	0.021191254	0.046775442	0.999933887	-0.209728937	-0.261938066	0.052209129	0.854724219
ENSG00000204219	*TCEA3*	-0.844037481	-0.063731988	0.17698758	-0.240719568	0.999933887	0.204909871	0.31072523	-0.105815358	0.854724219
ENSG00000160055	*TMEM234*	3.340344545	-0.011982698	-0.000380566	-0.011602133	0.999933887	-0.098231159	-0.132535151	0.034303992	0.854724219
ENSG00000259874	*AC034105.2*	-0.814875483	0.225824294	0.514423336	-0.288599042	0.999933887	1.957323737	1.81046858	0.146855157	0.854792353
ENSG00000285781	*AL117340.1*	0.9915231	0.229694479	0.509439113	-0.279744635	0.999933887	0.382227436	0.513894428	-0.131666992	0.854792353
ENSG00000267632	*AC067852.3*	1.510736565	-0.12450321	-0.039991351	-0.084511859	0.999933887	-0.483036894	-0.421858723	-0.061178171	0.854792353
ENSG00000196436	*NPIPB15*	1.017332371	0.086241966	0.153640994	-0.067399028	0.999933887	0.019578019	0.074986842	-0.055408823	0.854792353
ENSG00000113048	*MRPS27*	2.319639686	0.041206613	-0.153961005	0.195167618	0.999933887	0.14819332	0.100270378	0.047922942	0.854792353
ENSG00000182841	*RRP7BP*	1.919107847	-0.046654297	0.239938174	-0.286592471	0.999933887	0.156696588	0.104506797	0.052189792	0.854792353
ENSG00000171130	*ATP6V0E2*	1.975128634	0.025061784	0.041727691	-0.016665907	0.999933887	0.05774445	-0.004981574	0.062726023	0.854792353
ENSG00000124356	*STAMBP*	3.999118464	-0.006265074	-0.018317896	0.012052822	0.999933887	-0.021880727	-0.051895056	0.030014329	0.854792353
ENSG00000234814	*SVIL2P*	1.609567696	0.00732395	0.051946902	-0.044622952	0.999933887	0.293879087	0.348668012	-0.054788925	0.854792353
ENSG00000121289	*CEP89*	1.178151729	0.089525501	-0.095783794	0.185309295	0.999933887	-0.088394933	-0.140158135	0.051763203	0.855025263
ENSG00000162545	*CAMK2N1*	0.311477644	-0.059213865	0.024657562	-0.083871428	0.999933887	0.168318771	0.074827549	0.093491222	0.855025263
ENSG00000128596	*CCDC136*	0.186206453	0.024465655	0.029018109	-0.004552454	0.999933887	0.120148715	0.217087328	-0.096938613	0.855025263
ENSG00000005801	*ZNF195*	2.165999325	-0.007994219	-0.184193058	0.176198839	0.999933887	-0.079234734	-0.016598375	-0.062636359	0.855025263
ENSG00000103404	*USP31*	1.473454222	-0.014706454	-0.192346475	0.177640021	0.999933887	-0.215359084	-0.276908708	0.061549624	0.85524586
ENSG00000171223	*JUNB*	9.742533054	0.439614977	0.632812176	-0.193197199	0.999933887	1.000304674	1.060921551	-0.060616877	0.855377583
ENSG00000153130	*SCOC*	1.366693975	-0.018505661	-0.153728052	0.135222391	0.999933887	-0.356611459	-0.423933413	0.067321954	0.855377583
ENSG00000010270	*STARD3NL*	4.828031222	0.038910267	-0.028229083	0.067139349	0.999933887	0.640175065	0.683208577	-0.043033512	0.855499083
ENSG00000150756	*ATPSCKMT*	-0.03411328	0.205445486	0.046479191	0.158966295	0.999933887	-0.085544193	0.010416616	-0.095960809	0.855718898
ENSG00000116898	*MRPS15*	3.192007487	0.057737362	0.0532855	0.004451862	0.999933887	-0.050959623	-0.088937556	0.037977932	0.855718898
ENSG00000105698	*USF2*	7.216664813	0.037980259	0.04693486	-0.008954601	0.999933887	0.209129256	0.18382733	0.025301926	0.855718898
ENSG00000101447	*FAM83D*	-0.306148603	-0.037777987	-0.214331167	0.17655318	0.999933887	-0.018238092	0.092659379	-0.110897471	0.855718898
ENSG00000287937	*AC079753.2*	0.429859566	0.230706839	0.208361302	0.022345536	0.999933887	1.549902145	1.653805923	-0.103903778	0.85573835
ENSG00000275552	*AC243965.2*	-0.208836254	0.171518874	0.249618731	-0.078099857	0.999933887	1.072634135	0.993777345	0.078856789	0.85573835
ENSG00000106992	*AK1*	0.457266456	-0.039593624	-0.020296641	-0.019296983	0.999933887	0.153824429	0.073939438	0.079884991	0.85573835
ENSG00000110852	*CLEC2B*	7.811631229	-0.144154793	-0.124079711	-0.020075082	0.999933887	-0.412451152	-0.449376263	0.036925111	0.85579564
ENSG00000131871	*SELENOS*	3.328878672	0.026605715	0.019551275	0.007054439	0.999933887	-0.089885884	-0.051383112	-0.038502772	0.85579564
ENSG00000120685	*PROSER1*	3.295969109	-0.011949974	-0.041756694	0.02980672	0.999933887	-0.105821535	-0.064477111	-0.041344424	0.85579564
ENSG00000140577	*CRTC3*	4.221388697	0.018263844	0.079160461	-0.060896617	0.999933887	-0.011349046	0.015712097	-0.027061143	0.855805085
ENSG00000102890	*ELMO3*	2.565611513	-0.043184768	-0.056260191	0.013075424	0.999933887	-0.017414983	-0.058357786	0.040942803	0.855851966
ENSG00000109189	*USP46*	1.173252642	-0.0527232	-0.287593566	0.234870367	0.999933887	0.011297402	-0.045710782	0.057008184	0.855868132
ENSG00000134109	*EDEM1*	7.519487316	-0.063681758	-0.039191517	-0.02449024	0.999933887	-0.070569558	-0.047978792	-0.022590766	0.855868786
ENSG00000137770	*CTDSPL2*	3.72399562	-0.02229026	-0.098475849	0.076185589	0.999933887	-0.308556139	-0.250345022	-0.058211117	0.855868786
ENSG00000279554	*AC130448.2*	0.155021817	0.020865033	0.172966816	-0.152101782	0.999933887	-0.738556229	-0.610986038	-0.127570191	0.855868786
ENSG00000132406	*TMEM128*	1.388658222	-0.066863209	-0.026002187	-0.040861022	0.999933887	-0.168217974	-0.226292425	0.058074451	0.855913955
ENSG00000250031	*AC009927.1*	1.516552741	-0.009984378	0.06325696	-0.073241338	0.999933887	-0.684835539	-0.783874312	0.099038773	0.855913955
ENSG00000285863	*AC009554.2*	-0.440217777	0.133241957	-0.191993489	0.325235446	0.999933887	-0.487599093	-0.62142359	0.133824497	0.856021056
ENSG00000129347	*KRI1*	3.319942821	-0.007445	-0.072187756	0.064742755	0.999933887	0.168608231	0.206557292	-0.037949061	0.856021056
ENSG00000155324	*GRAMD2B*	-0.515033913	-0.228992689	0.063036873	-0.292029562	0.999933887	0.264574984	0.36494422	-0.100369235	0.856033324
ENSG00000119684	*MLH3*	1.30996668	-0.131826686	-0.024026555	-0.107800131	0.999933887	-0.141992382	-0.085268673	-0.056723708	0.856033324
ENSG00000104689	*TNFRSF10A*	1.866626159	-0.093355781	0.165110475	-0.258466256	0.999933887	-0.04700754	0.006496312	-0.053503852	0.856033324
ENSG00000261449	*AC103724.3*	0.701902603	0.050165206	0.310208151	-0.260042944	0.999933887	0.262462084	0.125931103	0.136530981	0.856033324
ENSG00000155438	*NIFK*	1.612069223	0.008026094	-0.188412903	0.196438996	0.999933887	0.144308327	0.200970411	-0.056662084	0.856033324
ENSG00000132153	*DHX30*	4.088988141	0.003418626	0.029902295	-0.026483669	0.999933887	0.149750864	0.18164137	-0.031890506	0.856033324
ENSG00000107438	*PDLIM1*	1.984705299	-0.001540319	-0.066134413	0.064594094	0.999933887	0.137419407	0.192458285	-0.055038878	0.856033324
ENSG00000131269	*ABCB7*	1.765346087	0.038409276	-0.025731871	0.064141147	0.999933887	-0.018995927	0.03556748	-0.054563406	0.856092287
ENSG00000105202	*FBL*	4.374376553	0.055277868	0.018706041	0.036571827	0.999933887	0.21566192	0.253212307	-0.037550387	0.8561034
ENSG00000230225	*MTND5P14*	-1.532206012	-0.046821401	0.132205179	-0.179026581	0.999933887	0.397150093	0.130253981	0.266896112	0.856201435
ENSG00000270060	*AC090589.3*	0.058119099	-0.35129403	-0.210953036	-0.140340994	0.999933887	-0.07974954	-0.169839893	0.090090353	0.856257963
ENSG00000106771	*TMEM245*	3.599306343	-0.111882425	0.0599856	-0.171868025	0.999933887	-0.044924758	-0.010033735	-0.034891023	0.856257963
ENSG00000249173	*LINC01093*	0.960718789	0.121112663	0.008124429	0.112988234	0.999933887	3.037246259	3.170022755	-0.132776496	0.856283934
ENSG00000216490	*IFI30*	6.746826295	0.193450909	0.327467706	-0.134016796	0.999933887	-0.066437643	-0.108956988	0.042519344	0.85644199
ENSG00000257284	*AC013437.1*	-0.924247679	0.066726816	0.168314632	-0.101587817	0.999933887	-0.497172397	-0.673330633	0.176158235	0.856490211
ENSG00000110367	*DDX6*	7.449792405	-0.071474572	-0.045301037	-0.026173536	0.999933887	-0.309467985	-0.286118233	-0.023349752	0.85705485
ENSG00000069974	*RAB27A*	6.286709962	-0.077812108	-0.05039501	-0.027417099	0.999933887	-0.320096491	-0.291961882	-0.028134609	0.85705485
ENSG00000104341	*LAPTM4B*	0.718404877	-0.116261078	-0.104780585	-0.011480493	0.999933887	-0.171891569	-0.10967046	-0.062221109	0.85705485
ENSG00000175182	*FAM131A*	4.784228978	0.04862946	0.022187812	0.026441648	0.999933887	-0.30159826	-0.334706741	0.033108482	0.85705485
ENSG00000203875	*SNHG5*	4.516505653	0.048947701	0.107218901	-0.058271201	0.999933887	0.207489929	0.244953902	-0.037463974	0.85705485
ENSG00000172819	*RARG*	2.699717697	0.060820861	0.058145637	0.002675225	0.999933887	-0.034881412	-0.078487637	0.043606226	0.85705485
ENSG00000204444	*APOM*	0.563380483	0.036876851	0.091071196	-0.054194345	0.999933887	0.147267703	0.067075098	0.080192605	0.85705485
ENSG00000168734	*PKIG*	1.285724574	0.017663784	0.006795131	0.010868653	0.999933887	-0.625424139	-0.677377492	0.051953354	0.85705485
ENSG00000169991	*IFFO2*	3.930637986	0.020342645	0.027662312	-0.007319666	0.999933887	0.138950677	0.106385158	0.032565518	0.85705693
ENSG00000198001	*IRAK4*	5.081620341	-0.0056747	-0.053922112	0.048247412	0.999933887	-0.231466234	-0.268843819	0.037377585	0.857149466
ENSG00000137210	*TMEM14B*	2.844785622	-0.063738833	0.044279444	-0.108018278	0.999933887	-0.012181525	-0.05182768	0.039646154	0.85725693
ENSG00000172432	*GTPBP2*	6.228921633	0.047456003	0.037802634	0.009653369	0.999933887	0.063636604	0.088109422	-0.024472818	0.85762825
ENSG00000140545	*MFGE8*	1.595015367	-0.044982497	0.021423716	-0.066406213	0.999933887	0.016538348	0.077507708	-0.06096936	0.85762825
ENSG00000164530	*PI16*	-0.811147484	0.015593226	0.010886508	0.004706718	0.999933887	0.165592158	0.010368995	0.155223164	0.85762825
ENSG00000196357	*ZNF565*	0.032078623	-0.315219703	-0.320579453	0.005359751	0.999933887	0.177952769	0.070976559	0.10697621	0.85765114
ENSG00000085415	*SEH1L*	2.283581172	-0.138641625	-0.021935515	-0.11670611	0.999933887	0.055425601	0.009207169	0.046218433	0.85765114
ENSG00000168394	*TAP1*	8.778485752	0.055736866	0.039761764	0.015975103	0.999933887	0.303500884	0.327416737	-0.023915853	0.85765114
ENSG00000146453	*PNLDC1*	-1.602799943	-0.432806942	-0.443810619	0.011003676	0.999933887	0.787643705	0.892323412	-0.104679707	0.85765114
ENSG00000271780	*AL118558.3*	3.203199984	0.121666394	0.146203154	-0.024536759	0.999933887	0.686930301	0.633852778	0.053077523	0.85765114
ENSG00000115073	*ACTR1B*	4.269735478	0.065793481	0.087467808	-0.021674327	0.999933887	0.318584288	0.355230384	-0.036646095	0.85765114
ENSG00000100764	*PSMC1*	3.788377109	-0.067192754	-0.172472808	0.105280054	0.999933887	-0.048889057	-0.01373765	-0.035151407	0.85765114
ENSG00000175536	*LIPT2*	-1.099144555	-0.256638314	-0.495255945	0.238617631	0.999933887	0.801942788	0.914118698	-0.11217591	0.85765114
ENSG00000128951	*DUT*	2.872041024	-0.041884041	0.03109718	-0.07298122	0.999933887	0.055635672	0.017333425	0.038302246	0.85765114
ENSG00000254909	*AP003392.2*	-0.033320211	0.100342992	0.052986651	0.047356341	0.999933887	-0.216761611	-0.125165834	-0.091595777	0.85765114
ENSG00000259182	*AC019254.1*	-0.667984772	0.081289035	-0.215537001	0.296826036	0.999933887	-0.179884961	-0.345891627	0.166006666	0.85765114
ENSG00000152117	*SMPD4BP*	4.139312021	0.015055586	0.025515349	-0.010459762	0.999933887	0.117262297	0.085311816	0.03195048	0.85765114
ENSG00000181215	*C4orf50*	0.488611014	0.116017868	-0.082006842	0.19802471	0.999933887	0.000582304	0.092612253	-0.092029948	0.857656328
ENSG00000233559	*LINC00513*	3.376966176	0.150613638	0.190964544	-0.040350906	0.999933887	0.629856635	0.694604284	-0.064747649	0.858871847

ENSG00000138709	*LARP1B*	1.52897632	-0.088598607	0.062192327	-0.150790933	0.999933887	0.125771426	0.073591341	0.052180086	0.858871847
ENSG00000130158	*DOCK6*	0.843817127	-0.097691709	0.012293869	-0.109985579	0.999933887	-0.038395533	0.034377904	-0.072773438	0.858871847
ENSG00000260145	*AC023825.2*	0.439332156	-0.025023035	0.122087587	-0.147110622	0.999933887	-0.535102857	-0.450631677	-0.08447118	0.858871847
ENSG00000115239	*ASB3*	2.31469109	0.082641188	-0.074161376	0.156802564	0.999933887	-0.189736297	-0.139492116	-0.050244181	0.858889646
ENSG00000243449	*C4orf48*	2.342495664	0.023768271	0.043498671	-0.0197304	0.999933887	-0.16265755	-0.230992136	0.068334586	0.859034749
ENSG00000205885	*C1RL-AS1*	2.652771824	0.068371973	0.106763383	-0.038391411	0.999933887	0.019077315	0.066605535	-0.04752822	0.859118881
ENSG00000286733	*LINC02348*	-0.485457216	0.049262949	0.012952328	0.036310621	0.999933887	0.048164129	0.14610201	-0.09793788	0.859118881
ENSG00000170545	*SMAGP*	1.012614872	0.006601117	0.040048956	-0.033447839	0.999933887	-0.056630536	0.010938764	-0.0675693	0.859345841
ENSG00000184743	*ATL3*	2.864424747	-0.074036072	-0.020230316	-0.053805757	0.999933887	-0.202805829	-0.162229912	-0.040575917	0.859567078
ENSG00000141279	*NPEPPS*	6.138158719	-0.043950288	-0.005395763	-0.038554525	0.999933887	-0.367279098	-0.391292081	0.024012982	0.859626219
ENSG00000155657	*TTN*	4.624008388	0.125547071	0.075751316	0.049795756	0.999933887	-0.198199482	-0.134194797	-0.064004686	0.859912545
ENSG00000176422	*SPRYD4*	0.830081989	0.096147325	-0.001807294	0.097954618	0.999933887	-0.031952247	0.026928571	-0.058880818	0.859912545
ENSG00000258056	*AC009779.2*	2.856681318	0.106070328	0.18742727	-0.081356942	0.999933887	0.138532481	0.094881335	0.043651146	0.859912545
ENSG00000170271	*FAXDC2*	0.579121563	-0.12291046	-0.0447781	-0.07813236	0.999933887	-0.05467027	0.02669617	-0.08136644	0.859912545
ENSG00000162441	*LZIC*	2.882369156	-0.064762192	-0.110802867	0.046040676	0.999933887	-0.154571037	-0.11102202	-0.043549016	0.859912545
ENSG00000279048	*AC080080.1*	0.401906539	-0.150432876	0.181459351	-0.331892227	0.999933887	0.509533321	0.425752732	0.083780589	0.859912545
ENSG00000063587	*ZNF275*	3.02072614	-0.044415618	0.061251408	-0.105667026	0.999933887	-0.255273967	-0.288379149	0.033105182	0.859912545
ENSG00000168005	*SPINDOC*	1.306076932	-0.071753026	-0.191239635	0.119486609	0.999933887	0.004490965	-0.054305193	0.058796158	0.859912545
ENSG00000105364	*MRPL4*	1.531820497	0.053526303	0.140639053	-0.08711275	0.999933887	0.224839035	0.171757529	0.053081506	0.859912545
ENSG00000172005	*MAL*	2.519312705	0.03275203	-0.028112885	0.060864915	0.999933887	0.20752651	0.252968487	-0.045441977	0.859912545
ENSG00000269867	*AC010326.3*	-0.003276947	-0.058492616	0.384695898	-0.443188514	0.981460244	-0.182767837	-0.271133555	0.088365718	0.859912545
ENSG00000066427	*ATXN3*	3.402094045	0.019961408	-0.028765783	0.048727192	0.999933887	-0.486602022	-0.52127522	0.034673198	0.859912545
ENSG00000269313	*MAGIX*	-0.278739951	-0.020406663	0.102890322	-0.123296985	0.999933887	-0.06722001	-0.189978277	0.122758267	0.859912545
ENSG00000280387	*AL109806.1*	1.866290057	-0.011959046	0.025889638	-0.037848684	0.999933887	-0.110269919	-0.048762394	-0.061507525	0.859912545
ENSG00000163125	*RPRD2*	4.14097509	0.007437032	0.02690778	-0.019470748	0.999933887	-0.071349109	-0.039677946	-0.031671163	0.859912545
ENSG00000156603	*MED19*	1.985496372	-0.006945588	-0.006624812	-0.000320775	0.999933887	0.033716034	-0.018821607	0.052537642	0.859938144
ENSG00000189362	*NEMP2*	1.552923277	-0.030163529	-0.143187538	0.113024009	0.999933887	-0.074177726	-0.029341537	-0.044836189	0.859968988
ENSG00000287917	*AP000944.7*	0.383294546	0.066636779	-0.002613203	0.069249981	0.999933887	0.963998834	0.887060813	0.076938021	0.85997749
ENSG00000119723	*COQ6*	0.405482022	-0.173381258	-0.070680876	-0.102700381	0.999933887	0.063690154	-0.011189739	0.074879893	0.860019493
ENSG00000186951	*PPARA*	2.67155566	0.041845607	0.034491976	0.007353631	0.999933887	-0.173942065	-0.214732841	0.040790776	0.860019493
ENSG00000152240	*HAUS1*	0.406278016	-0.063835286	-0.159600505	0.095765219	0.999933887	0.082266477	0.166734784	-0.084468307	0.860019493
ENSG00000108826	*MRPL27*	2.039564927	-0.014202508	-0.056812683	0.042610175	0.999933887	0.072567134	0.121177922	-0.048610788	0.860019493
ENSG00000088038	*CNOT3*	5.735681908	-0.004835706	0.000587552	-0.005423259	0.999933887	-0.337401162	-0.362159892	0.02475873	0.860019493
ENSG00000112697	*TMEM30A*	6.389621852	-0.00595945	-0.067707914	0.061748464	0.999933887	-0.647070126	-0.68836636	0.041296234	0.860019493
ENSG00000145358	*DDIT4L*	2.130741671	-0.012607103	-0.122802231	0.110195128	0.999933887	-0.290822171	-0.236370681	-0.05445149	0.86015966
ENSG00000170379	*TCAF2*	2.565083571	-0.123398439	-0.050344909	-0.07305353	0.999933887	0.215588843	0.170634777	0.044954066	0.860161002
ENSG00000120053	*GOT1*	1.367881754	-0.149088165	0.297386788	-0.446474953	0.944094537	0.032545482	-0.016071246	0.048616728	0.860161002
ENSG00000124103	*FAM209A*	1.207242412	-0.126744949	0.039703818	-0.166448767	0.999933887	-0.055961172	-0.126350008	0.070388835	0.860161002
ENSG00000130733	*YIPF2*	1.182885712	0.117810676	0.100239126	0.01757155	0.999933887	0.201216191	0.140136895	0.061079297	0.860161002
ENSG00000234636	*MED14OS*	0.012075826	-0.163948986	-0.005852896	-0.15809609	0.999933887	0.353028151	0.456863128	-0.103834977	0.860161002
ENSG00000025156	*HSF2*	2.030833296	-0.043196953	-0.082313941	0.039116988	0.999933887	-0.093759255	-0.14465332	0.050894065	0.860161002
ENSG00000080603	*SRCAP*	2.498993048	-0.012744412	-0.035275704	0.022531292	0.999933887	-0.216511781	-0.174360672	-0.04215111	0.860161002
ENSG00000133321	*PLAAT4*	4.071923582	0.010045585	-0.045791528	0.055837114	0.999933887	0.038327299	0.08103647	-0.042709172	0.860161002
ENSG00000063241	*ISOC2*	-0.011107482	0.138429932	0.202339598	-0.063909666	0.999933887	0.214286387	0.286713236	-0.072426849	0.860174366
ENSG00000125703	*ATG4C*	-0.294529538	-0.068292027	-0.147451752	0.079159725	0.999933887	-0.297542617	-0.194397739	-0.103144878	0.860214671
ENSG00000168259	*DNAJC7*	4.535905202	-0.081743382	-0.029949316	-0.051794066	0.999933887	-0.158692304	-0.182533404	0.0238411	0.860320981
ENSG00000276568	*Metazoa_SRP*	-0.697847896	0.117926727	0.280216896	-0.16229017	0.999933887	1.319745048	1.428237418	-0.10849237	0.861049176
ENSG00000077549	*CAPZB*	8.251925724	-0.007969048	0.00818041	-0.016149458	0.999933887	0.008740319	-0.011436213	0.020176533	0.861828427
ENSG00000266980	*AC087289.1*	-0.062717416	0.033311878	0.196153457	-0.162841579	0.999933887	0.841309532	0.73802092	0.103288612	0.862256742
ENSG00000185298	*CCDC137*	3.035726102	0.042630504	0.044727623	-0.002097119	0.999933887	0.069876222	0.033232155	0.036644067	0.862349602
ENSG00000164897	*TMUB1*	5.218243457	0.049799555	0.155404742	-0.105605187	0.999933887	0.409658112	0.442785532	-0.033127419	0.862403875
ENSG00000164136	*IL15*	0.656415612	-0.225418434	-0.019703481	-0.205714953	0.999933887	0.012280936	-0.084327895	0.096608831	0.862415006
ENSG00000154134	*ROBO3*	0.206318406	0.219493008	0.050727581	0.168765426	0.999933887	0.206301177	0.110691576	0.095609601	0.862415006
ENSG00000274925	*ZKSCAN2-DT*	0.801534358	-0.121841574	0.149144943	-0.270986517	0.999933887	-0.172419888	-0.110508656	-0.061911232	0.862415006
ENSG00000163900	*TMEM41A*	1.408381356	-0.077779593	-0.072224586	-0.005555007	0.999933887	0.15793185	0.100908839	0.057023011	0.862415006
ENSG00000272595	*OR10AH1P*	-1.16658988	-0.095717502	0.188092372	-0.283809874	0.999933887	0.0326661	0.170187577	-0.137521477	0.862415006
ENSG00000159433	*STARD9*	2.048554372	0.00934299	-0.157252837	0.166595827	0.999933887	-0.060140305	-0.013844938	-0.046295368	0.862415006
ENSG00000173930	*SLCO4C1*	2.613017115	-0.013223258	0.022668082	-0.035891339	0.999933887	-0.646242513	-0.701866063	0.055623549	0.862418012
ENSG00000230454	*U73166.1*	-0.071955598	0.014752641	-0.211509686	0.226262326	0.999933887	-0.632342355	-0.536952805	-0.095389551	0.862418012
ENSG00000146731	*CCT6A*	3.736739832	-0.020722006	-0.031826978	0.011104972	0.999933887	0.072539688	0.103282433	-0.030742745	0.862739093
ENSG00000174080	*CTSF*	0.864883371	0.105260592	-0.023191128	0.12845172	0.999933887	0.185121225	0.121676885	0.06344434	0.86305464
ENSG00000092758	*COL9A3*	1.312976408	0.086511485	0.147291758	-0.060780274	0.999933887	2.920623904	3.017797953	-0.097174049	0.863124721
ENSG00000060971	*ACAA1*	5.948934605	0.010979448	-0.003941198	0.014920647	0.999933887	-0.186222554	-0.224257298	0.038034744	0.863124721
ENSG00000131725	*WDR44*	3.197354481	-0.076215277	-0.077423656	0.001208379	0.999933887	-0.389576287	-0.426673957	0.03709767	0.863147084
ENSG00000169583	*CLIC3*	0.644464003	-0.007263328	0.034657577	-0.041920906	0.999933887	0.243904141	0.166844495	0.077059646	0.863147084
ENSG00000198026	*ZNF335*	5.90164647	0.009716227	0.060296308	-0.050580082	0.999933887	0.043048735	0.065794234	-0.022745499	0.863217447
ENSG00000258172	*AC073655.1*	0.964295954	0.220184736	-0.003250606	0.223435342	0.999933887	0.854971467	0.948501869	-0.093530402	0.863272791
ENSG00000135972	*MRPS9*	0.303325598	-0.108834808	0.078778406	-0.187613214	0.999933887	0.14898247	0.072916876	0.076065594	0.863474728
ENSG00000168517	*HEXIM2*	1.839391201	0.135962936	-0.112829354	0.24879229	0.999933887	0.049614752	0.010989368	0.038625384	0.863494798
ENSG00000118420	*UBE3D*	-1.033121732	-0.282805731	0.153628039	-0.436433769	0.999933887	-0.062232001	-0.178015895	0.115783895	0.863494798
ENSG00000102710	*SUPT20H*	4.761091601	0.057181608	-0.050596887	0.107778495	0.999933887	-0.27703815	-0.249919544	-0.027118606	0.863494798
ENSG00000250848	*AC021087.2*	-0.207371403	0.087721103	-0.039209199	0.126930302	0.999933887	-0.031326008	-0.130209098	0.09888309	0.863494798
ENSG00000168255	*POLR2J3*	0.787545425	0.034615486	0.056257479	-0.021641994	0.999933887	-0.112895096	-0.186407492	0.073512395	0.863494798
ENSG00000198393	*ZNF26*	2.75270236	-0.094132448	0.013344037	-0.107476485	0.999933887	-0.028789176	-0.068241857	0.039452682	0.863530987
ENSG00000106333	*PCOLCE*	0.452951853	-0.030196571	0.051618598	-0.081815169	0.999933887	-0.027815049	-0.116572022	0.088756972	0.863614279
ENSG00000175564	*UCP3*	0.527509291	0.073580288	0.051060339	0.022519949	0.999933887	-0.345686768	-0.42927089	0.083584121	0.863748462
ENSG00000137274	*BPHL*	-0.775299986	0.21147342	0.052486978	0.158986442	0.999933887	0.317195739	0.434331383	-0.117135643	0.863955263
ENSG00000102524	*TNFSF13B*	6.063869307	-0.055357563	-0.030335775	-0.025021788	0.999933887	-0.390030656	-0.435147199	0.045116543	0.863955263
ENSG00000197635	*DPP4*	1.860582055	-0.023474898	-0.219746761	0.196271864	0.999933887	-0.068025223	-0.010314915	-0.057710308	0.863955263
ENSG00000172349	*IL16*	6.686985249	-0.01675598	-0.088580588	0.071824608	0.999933887	0.64131541	0.679371489	-0.038056079	0.863955263
ENSG00000050628	*PTGER3*	-1.0845152	-0.012574984	-0.12556831	0.112993326	0.999933887	-0.2533146	-0.082728357	-0.170586243	0.863955263
ENSG00000213638	*ADAT3*	1.670927305	0.017944066	0.049458588	-0.031514522	0.999933887	-0.838774956	-0.900492071	0.061717115	0.863989143
ENSG00000136100	*VPS36*	3.269967768	0.052316285	-0.037569218	0.089885503	0.999933887	-0.193894675	-0.225835549	0.031940874	0.8640392
ENSG00000187837	*H1-2*	5.596188098	-0.000456312	-0.063281174	0.062824862	0.999933887	0.028057719	-0.007503597	0.035561315	0.864100148
ENSG00000143155	*TIPRL*	3.657796949	0.047515374	0.085720806	-0.038205432	0.999933887	0.182064158	0.154229298	0.027834861	0.864291204
ENSG00000198019	*FCGR1B*	3.601447688	0.040561163	-0.056843691	0.097404794	0.999933887	-0.023165506	0.017184364	-0.04034987	0.864376228
ENSG00000128534	*LSM8*	3.919354653	0.027688276	0.037139088	-0.009450812	0.999933887	-0.174343378	-0.13606545	-0.038277929	0.86442154
ENSG00000214114	*MYCBP*	4.300939723	-0.067372385	-0.127750719	0.060378334	0.999933887	0.121346516	0.090015433	0.031331083	0.864847674
ENSG00000131459	*GFPT2*	-0.118521913	0.212867127	-0.024752528	0.237619654	0.999933887	-0.080142237	0.018952009	-0.099094246	0.86486804
ENSG00000231890	*DARS-AS1*	4.862740777	0.239488988	0.285869371	-0.046380383	0.999933887	0.488887847	0.433181875	0.055705972	0.865053507
ENSG00000085719	*CPNE3*	4.14147453	-0.036906643	-0.006603813	-0.03030283	0.999933887	-0.245370258	-0.279544392	0.034174134	0.865053507
ENSG00000148396	*SEC16A*	7.303505239	-0.018843983	-0.01081824	-0.008025744	0.999933887	-0.064711146	-0.04505325	-0.019657896	0.865053507
ENSG00000100413	*POLR3H*	2.174092058	0.160670453	0.156571644	0.004098809	0.999933887	0.052840829	0.100756781	-0.047915952	0.865098849
ENSG00000247121	*AC009126.1*	0.4068422	0.340174116	0.313488359	0.026685757	0.999933887	-0.441454968	-0.502547814	0.061092846	0.865205799
ENSG00000101194	*SLC17A9*	1.319857303	0.205324503	-0.012867069	0.218191573	0.999933887	0.177307695	0.240665032	-0.063357337	0.865205799
ENSG00000269480	*AC020913.2*	0.402019437	0.139027029	0.076951359	0.06207567	0.999933887	-0.199993906	-0.130668817	-0.069325089	0.865205799
ENSG00000151332	*MBIP*	1.336835839	0.03838162	0.039460556	-0.001078936	0.999933887	-0.103240263	-0.170238848	0.066998585	0.865205799
ENSG00000204745	*AC083899.1*	-1.232262146	-0.084618606	0.355635157	-0.440253763	0.999933887	-0.57813276	-0.739429918	0.161297159	0.865205799
ENSG00000006831	*ADIPOR2*	5.321560239	-0.080056786	-0.040994357	-0.039062429	0.999933887	-0.188263946	-0.164158949	-0.024104997	0.865279085
ENSG00000278949	*AC127070.4*	1.348106368	-0.145987338	-0.231760862	0.085773524	0.999933887	-0.172919972	-0.114579283	-0.058340689	0.865279085
ENSG00000161547	*SRSF2*	7.151805137	0.058114252	0.101485735	-0.043371483	0.999933887	0.426695996	0.460602948	-0.033906952	0.865279085
ENSG00000169020	*ATP5ME*	1.527756054	-0.002954787	0.083723586	-0.086678374	0.999933887	-0.008878048	-0.076182923	0.067304874	0.865279085
ENSG00000134326	*CMPK2*	4.601863175	0.002096576	-0.023841275	0.02593785	0.999933887	0.211646392	0.165854245	0.045792147	0.865486623
ENSG00000137343	*ATAT1*	1.138531572	0.029024165	-0.044530567	0.073554733	0.999933887	0.074016109	0.016189776	0.057826333	0.865609287
ENSG00000110987	*BCL7A*	-0.148387939	0.228548272	-0.059728588	0.288276861	0.999933887	-0.281644788	-0.379001471	0.097356683	0.865832018

ENSG00000109917	*ZPR1*	4.479446061	-0.037632731	0.003328789	-0.04096152	0.999933887	0.188364478	0.215128598	-0.02676412	0.865846334
ENSG00000181104	*F2R*	3.129588889	-0.037214203	-0.113618855	0.076404652	0.999933887	-0.032430645	0.004786284	-0.03721693	0.865846334
ENSG00000169188	*APEX2*	1.910338923	-0.008253426	-0.039429967	0.03117654	0.999933887	0.0143005	0.06105558	-0.04675508	0.86610903
ENSG00000137876	*RSL24D1*	4.843261804	-0.075701721	-0.058091126	-0.017610595	0.999933887	-0.036705592	-0.006703871	-0.030001722	0.866165118
ENSG00000232112	*TMA7*	2.541295955	-0.067396895	0.024639313	-0.092036208	0.999933887	0.12571591	0.087968199	0.037747711	0.866165118
ENSG00000134825	*TMEM258*	3.347958968	0.020667591	-0.026458775	0.047126366	0.999933887	0.151822821	0.120138669	0.031684152	0.866165118
ENSG00000184293	*CLECL1*	-0.079171824	0.033147417	-0.079295018	0.112442435	0.999933887	0.062555127	0.158705377	-0.09615025	0.866165118
ENSG00000244560	*AC004890.2*	-0.332056026	0.008410067	0.049819611	-0.041409544	0.999933887	0.133052543	0.042920747	0.090131796	0.86629096
ENSG00000179869	*ABCA13*	1.130238169	-0.212141836	-0.043331718	-0.168810118	0.999933887	0.224421213	0.27208064	-0.047659427	0.866316984
ENSG00000134250	*NOTCH2*	9.523944468	-0.057348797	-0.022107666	-0.035241131	0.999933887	-0.103734021	-0.081913283	-0.021820738	0.866500806
ENSG00000102078	*SLC25A14*	1.81056972	-0.022128244	0.025767213	-0.047895458	0.999933887	-0.108582153	-0.165955716	0.057373563	0.866500806
ENSG00000158435	*CNOT11*	4.479706305	0.000950783	0.111180396	-0.110229613	0.999933887	-0.191303133	-0.164396555	-0.026906578	0.866500806
ENSG00000171863	*RPS7*	6.92699308	0.00825372	-0.051870337	0.060124057	0.999933887	0.032963163	0.060214176	-0.027251013	0.86657169
ENSG00000106588	*PSMA2*	0.425907421	-0.394659103	-0.131478121	-0.263180982	0.999933887	-0.200923344	-0.272580378	0.071657033	0.86665229
ENSG00000162722	*TRIM58*	-1.061447372	0.067248688	0.140316501	-0.073067813	0.999933887	0.0115673	0.13217717	-0.12060987	0.86665229
ENSG00000135960	*EDAR*	-1.166889064	-0.316751939	0.429415984	-0.746167923	0.969206975	0.281540093	0.394451454	-0.112911361	0.866913574
ENSG00000284930	*AC005280.2*	2.902129358	0.030948251	-0.046444551	0.077392802	0.999933887	1.319557424	1.373809226	-0.054251802	0.866913574
ENSG00000106392	*C1GALT1*	4.107035848	0.028196853	-0.05060675	0.078803603	0.999933887	-0.436060039	-0.37789213	-0.058167909	0.866913574
ENSG00000240449	*REPIN1-AS1*	0.558677366	0.036264511	-0.31544542	0.351709931	0.999933887	1.499747253	1.440621705	0.059125548	0.866913574
ENSG00000249471	*ZNF324B*	-0.348596868	-0.044341242	-0.016832771	-0.027508471	0.999933887	0.360163825	0.260167853	0.099995972	0.867082249
ENSG00000286116	*AL157394.3*	1.782565784	0.16233875	0.190653274	-0.028314524	0.999933887	1.6402878	1.563623976	0.076663824	0.867086377
ENSG00000172985	*SH3RF3*	0.898480318	0.15506988	0.03946132	0.11560856	0.999933887	-0.405300512	-0.476830276	0.071529763	0.867372944
ENSG00000184117	*NIPSNAP1*	2.325449963	0.102083482	-0.029739437	0.131822919	0.999933887	0.179919085	0.124693368	0.055225717	0.8674099
ENSG00000136925	*TSTD2*	1.964102099	-0.098531104	-0.013982054	-0.08454905	0.999933887	0.028784602	0.076579586	-0.047794983	0.8674099
ENSG00000134419	*RPS15A*	5.233280142	-0.046421956	-0.00909354	-0.037328417	0.999933887	0.067543313	0.098006641	-0.030463327	0.8674099
ENSG00000187098	*MITF*	-1.088057202	0.12450624	0.140767435	-0.016261196	0.999933887	-2.080500018	-2.188362892	0.107862874	0.8674099
ENSG00000215154	*AC141586.1*	1.632900114	0.039608134	0.014076719	0.025531415	0.999933887	0.104982054	0.049647014	0.05533504	0.8674099
ENSG00000158623	*COPG2*	0.659947456	0.043479396	-0.080598755	0.124078151	0.999933887	-0.122247544	-0.032902328	-0.089345216	0.8674099
ENSG00000137100	*DCTN3*	4.030696216	0.013446079	-0.018497788	0.031943867	0.999933887	0.04250865	0.0770142	-0.03450555	0.8674099
ENSG00000286616	*AL008730.1*	-0.342037743	-0.005956018	-0.00440921	-0.001546808	0.999933887	0.679792564	0.785653489	-0.105860925	0.8674099
ENSG00000189233	*NUGGC*	0.807857451	-0.00185661	0.035444042	-0.037300652	0.999933887	0.190102053	0.123123905	0.066978148	0.8674099
ENSG00000138442	*WDR12*	0.790200087	-0.02092553	0.056879186	-0.077804716	0.999933887	0.075865587	0.131024608	-0.05515902	0.867924644
ENSG00000198464	*ZNF480*	1.404825384	-0.046903773	-0.124609122	0.077705349	0.999933887	-0.155039738	-0.215558344	0.060518605	0.867932867
ENSG00000140839	*CLEC18B*	-0.455555488	0.055678131	0.124663345	-0.068985213	0.999933887	-0.30165931	-0.184178559	-0.117480751	0.867968552
ENSG00000116157	*GPX7*	0.843921271	-0.090905557	-0.216209309	0.125303752	0.999933887	0.148642556	0.085045031	0.063597524	0.867991655
ENSG00000129595	*EPB41L4A*	-1.185005393	-0.19418994	-0.337960856	0.143770916	0.999933887	-0.064094068	0.049413478	-0.113507545	0.868176764
ENSG00000156599	*ZDHHC5*	6.381065778	-0.039580934	0.027209609	-0.066790542	0.999933887	-0.244764299	-0.218619957	-0.026144342	0.868176764
ENSG00000146373	*RNF217*	-0.871722788	-0.145056602	-0.265457	0.120400398	0.999933887	-0.770136294	-0.920690957	0.150554662	0.868176764
ENSG00000136213	*CHST12*	3.544038014	-0.037218681	-0.025267171	-0.011951511	0.999933887	-0.024133797	-0.05878549	0.034651693	0.868176764
ENSG00000211724	*TRBV6-6*	-0.383724366	-0.061518393	-0.049045711	-0.012472682	0.999933887	0.259310786	0.148793755	0.110517031	0.868176764
ENSG00000131373	*HACL1*	2.094238441	-0.017445069	-0.089546417	0.072101348	0.999933887	0.052282857	0.09353124	-0.041248383	0.868176764
ENSG00000162383	*SLC1A7*	-1.130791794	0.021832621	0.03356615	-0.011733529	0.999933887	0.036565446	0.1239069	-0.087341454	0.868176764
ENSG00000011143	*MKS1*	-0.130037812	-0.196548153	-0.032540082	-0.164008071	0.999933887	0.163309737	0.241123977	-0.07781424	0.868266185
ENSG00000196155	*PLEKHG4*	1.191628198	0.00993096	0.255532943	-0.245601983	0.999933887	0.167796994	0.11328755	0.054509444	0.868266185
ENSG00000128626	*MRPS12*	1.44010144	-0.005702136	-0.012511771	0.006809635	0.999933887	0.132462276	0.185378898	-0.052916622	0.868411217
ENSG00000151062	*CACNA2D4*	1.758237285	0.0440131	0.049359057	-0.005345957	0.999933887	0.161876647	0.208563451	-0.046686804	0.868414455
ENSG00000152455	*SUV39H2*	-0.648273037	0.341747171	-0.135178235	0.476925406	0.999933887	-0.453820743	-0.564812429	0.110991687	0.868471221
ENSG00000066933	*MYO9A*	2.878735619	0.063229019	-0.132862349	0.196091367	0.999933887	-0.288288329	-0.230825219	-0.05746311	0.868471221
ENSG00000135002	*RFK*	1.757298693	0.035392414	-0.014632405	0.050024819	0.999933887	-0.17700142	-0.121567334	-0.055434086	0.868471221
ENSG00000077232	*DNAJC10*	3.69512517	-0.013106299	-0.179324501	0.166218202	0.999933887	-0.131848127	-0.078679199	-0.053168929	0.868471221
ENSG00000165632	*TAF3*	2.96690152	-0.001304369	-0.068827241	0.067522872	0.999933887	0.076850003	0.04134345	0.035506553	0.868471221
ENSG00000224083	*MTCO1P11*	-0.791665378	0.004033747	0.159783732	-0.155749985	0.999933887	0.189659991	0.408468622	-0.218808631	0.868471221
ENSG00000272092	*AC087623.2*	0.17842775	0.28483382	0.062375853	0.222457967	0.999933887	-0.222843053	-0.296393882	0.073550829	0.868532214
ENSG00000079462	*PAFAH1B3*	0.246346955	-0.050568635	0.004435749	-0.055004384	0.999933887	0.130391745	0.204687111	-0.074295366	0.868532214
ENSG00000276405	*TRBV13*	-1.451871609	-0.036995891	-0.414108552	0.377112661	0.999933887	0.034892891	0.189433243	-0.154540352	0.868532214
ENSG00000162576	*MXRA8*	-0.361415164	0.02784016	0.18774442	-0.15990426	0.999933887	0.320472118	0.211632246	0.108839872	0.868532214
ENSG00000286872	*AC024270.4*	0.258092218	0.04460573	-0.155392954	0.199998684	0.999933887	-0.3891084	-0.495801495	0.106693095	0.868561919
ENSG00000112941	*TENT4A*	4.786317017	-0.019102246	0.015912965	-0.035015211	0.999933887	-0.045577289	-0.070321153	0.024743864	0.86915984
ENSG00000264577	*AC010761.1*	0.070633172	-0.024835052	-0.047434148	0.022599096	0.999933887	0.1022055	0.011135849	0.091069651	0.86915984
ENSG00000123411	*IKZF4*	1.04438932	0.044279392	0.032871547	0.011407846	0.999933887	0.354787319	0.290427981	0.064359338	0.869217266
ENSG00000265185	*SNORD3B-1*	-0.496219848	0.00076065	0.086072034	-0.085311384	0.999933887	0.72404525	0.838107531	-0.114062281	0.869217266
ENSG00000120280	*CXorf21*	4.437488314	-0.116373607	-0.128671564	0.012297957	0.999933887	-0.050343418	-0.087975242	0.037631824	0.869245515
ENSG00000246067	*RAB30-DT*	0.808830351	0.055233281	0.015896307	0.039336974	0.999933887	0.208628227	0.152108107	0.05652012	0.869245515
ENSG00000204390	*HSPA1L*	0.934111446	0.02279431	0.219605571	-0.196811261	0.999933887	0.460169266	0.397688047	0.06248122	0.869245515
ENSG00000173421	*IHO1*	1.540087539	-0.147870589	0.013542339	-0.161412928	0.999933887	-0.468700521	-0.408348842	-0.060351679	0.869440195
ENSG00000119682	*AREL1*	7.346100093	-0.046651319	-0.007060618	-0.039590701	0.999933887	0.088312876	0.107463688	-0.019150813	0.869440195
ENSG00000066739	*ATG2B*	5.239226636	-0.004735193	-0.104672942	0.099937749	0.999933887	-0.431153948	-0.371273402	-0.059880546	0.869472072
ENSG00000235488	*JARID2-AS1*	3.067692473	-0.033591036	0.115576026	-0.149167062	0.999933887	0.375318164	0.421363468	-0.046045304	0.869534476
ENSG00000019991	*HGF*	0.311829016	-0.082565127	-0.040022814	-0.042542314	0.999933887	-1.216080479	-1.314941761	0.098861282	0.869619484
ENSG00000135164	*DMTF1*	5.014241217	-0.047639192	-0.010049387	-0.037589805	0.999933887	-0.403839168	-0.45667655	0.052837383	0.869630296
ENSG00000179639	*FCER1A*	0.337786363	0.194840373	-0.304561762	0.499402135	0.944094537	-0.188464824	-0.123266204	-0.06519862	0.869661709
ENSG00000135709	*KIAA0513*	6.741315294	-0.041748979	-0.043221824	0.001472845	0.999933887	-0.905049407	-0.86420997	-0.040839436	0.869925767
ENSG00000256553	*TRAV1-2*	-0.772768909	-0.050779282	-0.058813316	0.008034034	0.999933887	0.394051783	0.287802078	0.106249705	0.869971828
ENSG00000198157	*HMGN5*	-0.283008497	-0.14474302	-0.1122406	-0.03250242	0.999933887	-0.970933277	-1.081814351	0.110881074	0.870212834
ENSG00000088808	*PPP1R13B*	1.98609554	-0.034957216	0.032755227	-0.067712443	0.999933887	0.005150562	-0.044512561	0.049663124	0.870212834
ENSG00000124635	*H2BC11*	0.155637458	-0.046615916	-0.061522334	0.014906418	0.999933887	0.017590777	-0.085678596	0.103269372	0.870415729
ENSG00000197540	*GZMM*	4.267391712	0.100629676	-0.028192555	0.128822231	0.999933887	0.160637133	0.123623409	0.037013724	0.870505375
ENSG00000162929	*KIAA1841*	0.22536293	0.29056495	-0.384384573	0.674949523	0.144167088	-0.447338289	-0.531590533	0.084252244	0.870526104
ENSG00000108561	*C1QBP*	2.781063398	0.084742225	0.05031349	0.034428735	0.999933887	0.188282089	0.232336822	-0.044054733	0.87053105
ENSG00000178802	*MPI*	2.146525671	0.0090419	0.006249	0.002792899	0.999933887	0.17812365	0.225903678	-0.047780028	0.87053105
ENSG00000168395	*ING5*	3.442464574	0.004107394	0.081056884	-0.076949489	0.999933887	0.176206898	0.13871841	0.037488488	0.87053105
ENSG00000134030	*CTIF*	0.899019037	0.010847248	-0.008759393	0.01960664	0.999933887	-0.065322934	0.002829055	-0.068151989	0.870670963
ENSG00000132465	*JCHAIN*	0.44134625	0.02288801	-0.059410294	0.082298304	0.999933887	-0.043854297	0.053354054	-0.097208351	0.870747641
ENSG00000071564	*TCF3*	3.576486062	0.100079366	0.143109582	-0.043030216	0.999933887	0.084860995	0.052969619	0.031891376	0.870899874
ENSG00000136813	*ECPAS*	5.1902988	-0.036623518	0.007984468	-0.044607986	0.999933887	-0.022654908	-0.046550656	0.023895748	0.870899874
ENSG00000232098	*AC012313.1*	0.805300618	0.260166429	-0.162607845	0.422774274	0.999933887	0.062665732	0.011219926	0.051445806	0.870988098
ENSG00000124207	*CSE1L*	2.924325002	-0.087984217	-0.002620527	-0.08536369	0.999933887	0.006705525	-0.032413751	0.039119276	0.870988098
ENSG00000122034	*GTF3A*	4.786063546	-0.024279413	-0.097001292	0.072721879	0.999933887	0.044207634	0.017231307	0.026976327	0.870988098
ENSG00000267758	*AC099811.4*	1.560347019	-0.042447346	0.119792036	-0.162239382	0.999933887	1.32621715	1.422045102	-0.095827952	0.870988098
ENSG00000116062	*MSH6*	3.336464522	0.015353682	0.01229887	0.003054813	0.999933887	-0.112018078	-0.076463918	-0.035554161	0.870988098
ENSG00000137509	*PRCP*	6.294895802	-0.003403881	-0.026931833	0.023527952	0.999933887	-0.271776923	-0.232151215	-0.039625708	0.870988098
ENSG00000140450	*ARRDC4*	4.735276148	-0.145948087	-0.112627719	-0.033320369	0.999933887	-0.233516868	-0.181903433	-0.051613435	0.871003879
ENSG00000180574	*EIF2S3B*	-1.770112482	0.274647295	0.006934395	0.2677129	0.999933887	-0.004678668	0.103923161	-0.108601829	0.871003879
ENSG00000010219	*DYRK4*	-0.454052378	-0.112002859	-0.018363825	-0.093639034	0.999933887	0.20312507	0.108453155	0.094671916	0.871003879
ENSG00000233170	*AC138356.2*	0.120778108	-0.062483112	-0.014248618	-0.048234494	0.999933887	-0.439476142	-0.560970681	0.12149454	0.871003879
ENSG00000269984	*AC078795.1*	-0.443888	0.109509852	-0.0057153	0.115225152	0.999933887	-0.033726257	0.08465137	-0.118377627	0.871124176
ENSG00000261573	*AL157402.2*	1.219140718	0.047228054	0.452683807	-0.405455753	0.999933887	0.871686428	0.725937259	0.145749169	0.871124176
ENSG00000169330	*MINAR1*	-0.682890308	0.025598317	0.08245597	-0.056857652	0.999933887	0.018421596	-0.064841801	0.083263397	0.871124176
ENSG00000166405	*RIC3*	1.269731295	0.010305854	0.148831198	-0.138525343	0.999933887	-0.02298566	0.031583776	-0.054569436	0.871124176
ENSG00000143434	*SEMA6C*	0.715680753	0.008174611	0.133248332	-0.125073721	0.999933887	-0.118335406	-0.189988605	0.0716532	0.871124176
ENSG00000125355	*TMEM255A*	0.118950551	0.161628576	-0.316641892	0.478270468	0.999933887	-0.372512983	-0.310874834	-0.061638149	0.871177781
ENSG00000102241	*HTATSF1*	3.397068239	-0.035645415	-0.178630498	0.142985083	0.999933887	0.043867473	0.004652467	0.039215006	0.871177781
ENSG00000204514	*ZNF814*	2.337900813	-0.064054019	-0.09521414	0.031160121	0.999933887	-0.196418336	-0.157818173	-0.038600162	0.871224564
ENSG00000100321	*SYNGR1*	-0.189471933	0.067366138	-0.021470452	0.08883659	0.999933887	0.40790596	0.326281292	0.081624668	0.871224564
ENSG00000137494	*ANKRD42*	3.964670463	0.077830604	0.043660674	0.03416993	0.999933887	0.437341829	0.473733969	-0.03639214	0.871231854

ENSG00000171680	*PLEKHG5*	-0.565976959	0.03570503	0.027008608	0.008696422	0.999933887	-0.079012703	-0.191213661	0.112200958	0.871231854
ENSG00000244242	*IFITM10*	4.655531423	0.1377672	0.243334685	-0.105567485	0.999933887	0.798328914	0.869718758	-0.071389844	0.871288673
ENSG00000237264	*FTH1P11*	0.818960404	0.059435975	-0.009436569	0.068872543	0.999933887	0.529211797	0.585669325	-0.056457528	0.871288673
ENSG00000230701	*FBXW4P1*	0.281047965	-0.044221066	-0.060050286	0.01582922	0.999933887	0.246205399	0.144474431	0.101730968	0.871288673
ENSG00000158710	*TAGLN2*	10.79183884	0.015803203	-0.015823433	0.031626636	0.999933887	0.026101689	0.04943311	-0.023331421	0.871288673
ENSG00000145014	*TMEM44*	0.26192638	-0.122341191	-0.113233728	-0.009107464	0.999933887	0.15013076	0.080577305	0.069553454	0.871347823
ENSG00000130783	*CCDC62*	-0.075407067	-0.215742155	-0.063994128	-0.151748027	0.999933887	-0.38383811	-0.46966865	0.08583054	0.87146824
ENSG00000174606	*ANGEL2*	3.654949531	-0.048027277	0.004154176	-0.052181453	0.999933887	-0.207584797	-0.168827271	-0.038757526	0.87146824
ENSG00000146281	*PM20D2*	3.448879949	-0.058590507	0.022628594	-0.081219101	0.999933887	0.263808602	0.223202788	0.040605813	0.87146824
ENSG00000258777	*HIF1A-AS1*	-2.141432959	0.006067397	0.266307313	-0.260239916	0.999933887	2.377314463	2.516008621	-0.138694157	0.87146824
ENSG00000160199	*PKNOX1*	4.524079712	-0.068318199	-0.072127341	0.003809142	0.999933887	-0.369858764	-0.395685065	0.025826301	0.871572807
ENSG00000121390	*PSPC1*	4.908999388	-0.015610294	-0.008409468	-0.007200826	0.999933887	-0.026099452	-0.048298248	0.022198796	0.871572807
ENSG00000110171	*TRIM3*	2.552018725	0.144847477	0.022861707	0.12198577	0.999933887	0.171945836	0.134075154	0.037870682	0.871647603
ENSG00000111716	*LDHB*	0.657698427	-0.070809986	-0.185929343	0.115119357	0.999933887	-0.001488071	0.074285766	-0.075773838	0.871647603
ENSG00000103047	*TANGO6*	1.619011112	-0.038129872	0.095211623	-0.133341494	0.999933887	0.088681172	0.143623078	-0.054941905	0.871647603
ENSG00000134153	*EMC7*	4.575860675	0.009042696	0.011803247	-0.002760551	0.999933887	-0.254115166	-0.283293681	0.029178515	0.871647603
ENSG00000125743	*SNRPD2*	4.370729279	0.021272204	0.019922914	0.00134929	0.999933887	0.14325566	0.174690486	-0.031434826	0.871686358
ENSG00000212743	*LINC02656*	2.24226644	-0.004082414	0.197841901	-0.201924315	0.999933887	-0.159942073	-0.105032561	-0.054909513	0.871728514
ENSG00000135297	*MTO1*	2.564314601	0.011602705	0.030280513	-0.018677808	0.999933887	0.085217573	0.121055228	-0.035837655	0.871991797
ENSG00000153814	*JAZF1*	3.015574528	-0.092074825	-0.004162825	-0.087912	0.999933887	-0.144975492	-0.179010652	0.03403516	0.872017737
ENSG00000166135	*HIF1AN*	5.546204341	-0.059206313	-0.063359277	0.004152964	0.999933887	-0.081269564	-0.056661746	-0.024607818	0.872017737
ENSG00000228049	*POLR2J2*	-0.157873125	0.086954263	0.017481413	0.069472849	0.999933887	-0.06059929	0.020620693	-0.081219983	0.872017737
ENSG00000255328	*AC136475.5*	-0.695501038	-0.06033412	0.036699828	-0.097033947	0.999933887	0.112146614	0.233974689	-0.121828075	0.872017737
ENSG00000199349	*Y_RNA*	-1.474817383	-0.069435517	0.018094224	-0.087529741	0.999933887	0.481184106	0.245302283	0.235881823	0.872017737
ENSG00000149636	*DSN1*	1.308055495	-0.019623264	0.088872054	-0.108495318	0.999933887	-0.009168003	0.053491361	-0.062659364	0.872017737
ENSG00000163803	*PLB1*	5.192095237	-0.009106128	0.025106062	-0.03421219	0.999933887	0.109645741	0.134315709	-0.024669968	0.872017737
ENSG00000169217	*CD2BP2*	6.081635973	0.0104824	0.044855757	-0.034373357	0.999933887	-0.183230467	-0.212179235	0.028948768	0.872017737
ENSG00000078618	*NRDC*	7.081661471	-0.000602654	-0.083452983	0.082850329	0.999933887	-0.160953561	-0.139018551	-0.02193501	0.872017737
ENSG00000251441	*RTEL1P1*	1.694170596	0.066972423	0.057429579	0.009542844	0.999933887	0.634032094	0.539339847	0.094692247	0.872078866
ENSG00000120215	*MLANA*	-0.16233898	0.035289508	-0.126048365	0.161337873	0.999933887	-0.387736242	-0.49811266	0.110376418	0.872109379
ENSG00000150316	*CWC15*	3.912794529	-0.143482954	-0.165405299	0.021922345	0.999933887	-0.112125551	-0.083091719	-0.029033832	0.872212032
ENSG00000272462	*U91328.1*	0.915577271	-0.037418125	-0.161495989	0.124077864	0.999933887	-0.016422534	0.061064182	-0.077486715	0.872278976
ENSG00000211750	*TRBV24-1*	-0.427691773	-0.12846519	-0.437670025	0.309204835	0.999933887	0.22154672	0.31227262	-0.090725899	0.872449293
ENSG00000111537	*IFNG*	2.090062162	-0.057039984	-0.083547484	0.0265075	0.999933887	0.028860672	-0.020395122	0.049255795	0.872449293
ENSG00000279348	*AC012513.3*	-0.369988246	0.03117557	-0.335189548	0.366365118	0.999933887	0.094244909	0.173161412	-0.078916503	0.872449293
ENSG00000163694	*RBM47*	7.66009476	0.016404411	0.03455022	-0.018145809	0.999933887	-0.395164619	-0.368385513	-0.026779106	0.872490196
ENSG00000242522	*KLHL6-AS1*	0.038112784	-0.099524669	-0.008018394	-0.091506276	0.999933887	0.369829709	0.292365504	0.077464206	0.872493431
ENSG00000171236	*LRG1*	6.107296672	0.087964846	0.017335104	0.070629742	0.999933887	0.568367169	0.538238377	0.030128792	0.872579034
ENSG00000161999	*JMJD8*	1.374894351	0.140747026	0.064950632	0.075796393	0.999933887	0.036410737	-0.022014932	0.058425669	0.872579034
ENSG00000260971	*AC119674.1*	-1.060615546	0.439744683	-0.123059404	0.562804087	0.999933887	0.091058473	0.152010207	-0.060951734	0.872579034
ENSG00000237945	*LINC00649*	2.942873793	0.052822356	-0.036944573	0.089766929	0.999933887	0.096866787	0.132787165	-0.035920377	0.872579034
ENSG00000224001	*AL360081.1*	0.311117561	0.096113862	-0.024001242	0.120115104	0.999933887	-0.115586815	-0.034314073	-0.081272742	0.872579034
ENSG00000253982	*AC100810.1*	2.169021921	-0.072175487	-0.120995386	0.048819899	0.999933887	-0.229479018	-0.267460283	0.037981265	0.872579034
ENSG00000250251	*PKD1P6*	1.525491694	-0.058719233	0.107791764	-0.166510997	0.999933887	0.060379909	0.119688325	-0.059308416	0.872579034
ENSG00000107281	*NPDC1*	2.579156932	0.041430617	0.165035763	-0.123605146	0.999933887	0.061050091	0.011725948	0.049324142	0.872579034
ENSG00000161800	*RACGAP1*	0.904885899	-0.044215088	-0.128525215	0.084310127	0.999933887	-0.083366285	-0.138878505	0.05551222	0.872579034
ENSG00000134061	*CD180*	-0.647765667	0.092795219	0.134679799	-0.04188458	0.999933887	-0.947884715	-0.85060452	-0.097280195	0.872579034
ENSG00000004142	*POLDIP2*	4.145225149	-0.016205514	0.015274031	-0.031479546	0.999933887	-0.088331164	-0.116212636	0.027881472	0.872579034
ENSG00000279407	*AC007191.1*	1.175190071	0.024443851	0.315418158	-0.290974306	0.944094537	0.007685511	-0.051014844	0.058700356	0.872579034
ENSG00000256448	*AP000763.3*	-0.32354979	0.018487913	0.0799902	-0.061502287	0.999933887	-0.963435122	-1.10573113	0.142296009	0.872579034
ENSG00000125971	*DYNLRB1*	1.425525	0.009385903	0.16452656	-0.155140658	0.999933887	-0.009294549	0.049711917	-0.059006465	0.872579034
ENSG00000226564	*FTH1P20*	3.02412795	0.008614396	0.009483451	-0.000869054	0.999933887	0.660708177	0.617094889	0.043613289	0.872579034
ENSG00000280195	*AC245140.2*	0.74003558	0.155062839	0.460460105	-0.305397266	0.999933887	0.767447128	0.692428671	0.075018457	0.87287534
ENSG00000267858	*MZF1-AS1*	1.705345011	0.206143903	0.303172997	-0.097029094	0.999933887	-0.050371368	-0.130169654	0.079798286	0.872910206
ENSG00000163421	*PROK2*	8.910788501	0.039681398	0.057231704	-0.017550306	0.999933887	-0.031560296	-0.070684287	0.039123991	0.872975794
ENSG00000137992	*DBT*	1.333683032	-0.025079883	0.050251088	-0.075330971	0.999933887	-0.226106012	-0.291118865	0.065012853	0.872992028
ENSG00000175197	*DDIT3*	7.802334442	0.111690051	0.146027068	-0.034337017	0.999933887	0.08779052	0.060236666	0.027553854	0.873177034
ENSG00000259151	*CAP2P1*	-0.168103733	0.186387893	0.059079654	0.127308239	0.999933887	0.036400414	0.126064497	-0.089664083	0.873355089
ENSG00000163602	*RYBP*	7.521928472	-0.053937102	-0.029799216	-0.024137886	0.999933887	0.082611485	0.108689321	-0.026077835	0.873355089
ENSG00000284686	*AC119674.2*	-1.536810601	-0.276887362	-0.513814672	0.236927309	0.999933887	-0.081958995	-0.015976251	-0.065982744	0.873355089
ENSG00000117479	*SLC19A2*	2.996285416	-0.024452297	-0.046929249	0.022476952	0.999933887	-0.075979871	-0.024425114	-0.051554757	0.873355089
ENSG00000027869	*SH2D2A*	4.330846318	-0.035852123	-0.006009255	-0.029842868	0.999933887	0.165229334	0.193000293	-0.027770958	0.873370287
ENSG00000138640	*FAM13A*	2.093724923	-0.03290455	-0.115813797	0.082909247	0.999933887	-0.298496014	-0.236297251	-0.062198763	0.873370287
ENSG00000261172	*AC133919.2*	1.119424237	0.030961955	-0.035895872	0.066857827	0.999933887	0.337438959	0.260273861	0.077165098	0.873370287
ENSG00000253676	*TAGLN2P1*	-0.195032815	-0.002551204	-0.126167276	0.123616072	0.999933887	0.202000917	0.103530555	0.098470362	0.873370287
ENSG00000132600	*PRMT7*	2.748808541	-0.000583171	-0.041293478	0.040710306	0.999933887	0.026348823	0.060956182	-0.034607359	0.873370287
ENSG00000054611	*TBC1D22A*	5.134608127	0.038216071	-0.0176652	0.055881271	0.999933887	0.273233192	0.296673846	-0.023440655	0.873375936
ENSG00000172824	*CES4A*	-0.147964072	-0.055936799	0.399384371	-0.45532117	0.999933887	-0.061417704	-0.137850709	0.076433005	0.8733966
ENSG00000204472	*AIF1*	6.819632149	-0.032937377	-0.039939936	0.007002559	0.999933887	0.098422785	0.0676827	0.030740085	0.873437224
ENSG00000177383	*MAGEF1*	-0.011070319	0.224205497	0.030990861	0.193214636	0.999933887	-0.012414451	0.061106703	-0.073521155	0.87375036
ENSG00000138363	*ATIC*	2.456725618	-0.047014359	-0.192124265	0.145109906	0.999933887	0.17186843	0.205848812	-0.033980382	0.87375036
ENSG00000100918	*REC8*	6.85845196	0.020394527	-0.018216587	0.038611114	0.999933887	0.193867484	0.217452645	-0.023585161	0.87375036
ENSG00000280047	*AC091825.3*	0.505484138	-0.078519843	-0.231127135	0.152607291	0.999933887	-0.45556516	-0.508268145	0.052702985	0.87375036
ENSG00000101132	*PFDN4*	0.596501123	-0.017118877	-0.346707052	0.329588174	0.999933887	-0.093468242	-0.03421728	-0.059250963	0.87375036
ENSG00000012817	*KDM5D*	-2.468918478	-0.15448107	0.06372029	-0.21820136	0.999933887	-0.094441908	-0.04928639	-0.045155518	0.874199859
ENSG00000136240	*KDELR2*	3.846800832	-0.030160857	0.04555357	-0.075714427	0.999933887	-0.074668973	-0.044503636	-0.030165338	0.874199859
ENSG00000279727	*LINC02033*	-0.144511405	0.060088565	0.15272033	-0.092631765	0.999933887	0.219663177	0.111186793	0.108476385	0.874199859
ENSG00000004534	*RBM6*	5.041751726	-0.011725774	-0.042575182	0.030849407	0.999933887	0.046041757	0.024534861	0.021506896	0.874199859
ENSG00000114054	*PCCB*	1.193231824	0.024611216	0.149068768	-0.124457551	0.999933887	-0.038897277	0.041315109	-0.080212386	0.874199859
ENSG00000224631	*RPS27AP16*	-0.407682211	0.008847598	0.23672195	-0.227874352	0.999933887	0.255079627	0.348349974	-0.093270347	0.874199859
ENSG00000231305	*AC112484.1*	1.226247386	-0.005890948	-0.019918195	0.014027247	0.999933887	0.580321334	0.633800301	-0.053478966	0.874199859
ENSG00000115998	*C2orf42*	2.464674995	0.062153426	-0.126788412	0.188941838	0.999933887	-0.004324454	-0.039444638	0.035120185	0.874212347
ENSG00000145293	*ENOPH1*	2.093984181	-0.138588456	-0.085837049	-0.052751407	0.999933887	-0.113780831	-0.149061548	0.035280718	0.874267086
ENSG00000280046	*AC104581.4*	1.871628506	0.256724261	0.335983437	-0.079259176	0.999933887	1.078463759	1.143025462	-0.064561703	0.874774864
ENSG00000213145	*CRIP1*	2.624448114	0.069940816	0.020053958	0.049886858	0.999933887	0.157752116	0.108585643	0.049166472	0.874774864
ENSG00000171984	*SHLD1*	1.106182123	-0.095643618	-0.029322612	-0.066321006	0.999933887	-0.247633996	-0.299402544	0.051768548	0.874774864
ENSG00000141543	*EIF4A3*	6.861728749	0.027509521	-0.012278775	0.039788296	0.999933887	0.051220291	0.075298074	-0.024077783	0.874774864
ENSG00000123096	*SSPN*	-0.200925082	-0.158034834	0.063622438	-0.221657272	0.999933887	-1.783115428	-1.864271772	0.081156344	0.874774864
ENSG00000232629	*HLA-DQB2*	-0.628673582	0.083890356	-0.1486971	0.232587457	0.999933887	0.323422379	0.229328698	0.094093681	0.874774864
ENSG00000049167	*ERCC8*	0.318857304	-0.013557534	-0.09795765	0.084400116	0.999933887	0.004633999	0.095809013	-0.091175014	0.874774864
ENSG00000280011	*AL031595.2*	-0.252378924	-0.016089655	-0.069472647	0.053382992	0.999933887	-0.179573814	-0.28351265	0.103938836	0.874774864
ENSG00000214425	*LRRC37A4P*	2.403204157	-0.007754404	0.091716425	-0.099470829	0.999933887	-0.207031438	-0.150377219	-0.05665422	0.874774864
ENSG00000236144	*TMEM147-AS1*	-0.297793979	-0.301759678	0.061804716	-0.363564393	0.999933887	0.179743223	0.104548044	0.075195179	0.874914592
ENSG00000273382	*AL356488.3*	1.526660617	-0.132645352	-0.10533569	-0.027309663	0.999933887	-0.218284505	-0.158827563	-0.059456942	0.874914592
ENSG00000138439	*FAM117B*	2.710134811	-0.054270398	0.059865416	-0.114135815	0.999933887	-0.16296785	-0.211469318	0.048501467	0.874914592
ENSG00000028839	*TBPL1*	2.609277194	0.04993924	0.027138576	0.022800664	0.999933887	-0.189128688	-0.222172008	0.033043319	0.874914592
ENSG00000196233	*LCOR*	6.125425463	-0.043789292	-0.075922692	0.032133399	0.999933887	-0.408202831	-0.368595753	-0.039607078	0.874914592
ENSG00000114867	*EIF4G1*	7.163199786	0.005531337	0.027352049	-0.021820712	0.999933887	-0.049237737	-0.066763648	0.017525911	0.874914592
ENSG00000200814	*RNU6-595P*	0.459389177	0.001352632	0.103790305	-0.102437674	0.999933887	-0.026730493	-0.117661243	0.09093075	0.874914592
ENSG00000176623	*RMDN1*	2.342821154	-0.080764659	0.055917917	-0.136682576	0.999933887	-0.089697074	-0.050195953	-0.039501121	0.875189859
ENSG00000253893	*FAM85B*	-1.049250251	0.169706037	0.007585108	0.162120929	0.999933887	0.187936522	0.06097301	0.126963512	0.875189859
ENSG00000177156	*TALDO1*	9.555420377	-0.020817099	-0.034401838	0.013584739	0.999933887	-0.345092588	-0.314954106	-0.030138481	0.875189859
ENSG00000124098	*FAM210B*	2.566342298	0.018706425	-0.126612416	0.145318841	0.999933887	-0.171367611	-0.214890361	0.04352275	0.875189859
ENSG00000067596	*DHX8*	6.025597608	-0.010442546	-0.019843216	0.009400671	0.999933887	-0.309366976	-0.283006644	-0.026360332	0.875189859
ENSG00000117411	*B4GALT2*	0.273106971	0.028596621	0.224769545	-0.196172925	0.999933887	0.113488047	0.188390877	-0.07490283	0.875189859

ENSG00000152213	*ARL11*	5.369348871	-0.011733519	-0.114625447	0.102891927	0.999933887	-0.769466939	-0.73557841	-0.033888529	0.875189859
ENSG00000110063	*DCPS*	2.217391479	0.007309283	0.120805969	-0.113496686	0.999933887	0.425754335	0.476803656	-0.051049321	0.875189859
ENSG00000144744	*UBA3*	4.39887586	-0.039556467	-0.00067253	-0.038883938	0.999933887	-0.083613598	-0.10856566	0.024952062	0.87528306
ENSG00000162885	*B3GALNT2*	0.757280602	0.091760929	-0.179616091	0.27137702	0.999933887	-0.267049963	-0.200638296	-0.066411667	0.875420763
ENSG00000184260	*H2AC20*	1.545001731	0.08982348	0.186714971	-0.096891491	0.999933887	0.28245254	0.212315216	0.070137324	0.875470424
ENSG00000116857	*TMEM9*	1.424171383	0.087055223	-0.059497199	0.146552422	0.999933887	-0.017004239	0.047947588	-0.064951827	0.875470424
ENSG00000287576	*AC067930.9*	-0.230839528	0.083048154	0.188203931	-0.105155778	0.999933887	0.361630684	0.458912528	-0.097281844	0.875470424
ENSG00000263466	*AC006441.1*	-0.023673076	0.056328779	-0.178184983	0.234513762	0.999933887	-0.257264212	-0.335446974	0.078182762	0.875470424
ENSG00000225217	*HSPA7*	3.986263416	0.017965808	-0.047754857	0.065720665	0.999933887	0.032725721	-0.018701089	0.051426809	0.875470424
ENSG00000278918	*AC080112.3*	3.574536641	0.146616781	0.098224205	0.048392576	0.999933887	0.369518956	0.40852805	-0.039009094	0.875529166
ENSG00000165724	*ZMYND19*	1.289663903	-0.0916441	0.114101615	-0.205745715	0.999933887	0.221052948	0.171654715	0.049398233	0.875583315
ENSG00000158428	*CATIP*	1.549193347	0.189109904	0.264273997	-0.075164092	0.999933887	0.262391265	0.336736589	-0.074345324	0.875696431
ENSG00000108439	*PNPO*	-0.548914975	-0.115498993	-0.548428525	0.432929532	0.999933887	0.291538624	0.204940927	0.086597698	0.875696431
ENSG00000163576	*EFHB*	-0.672490781	-0.190120516	0.081136215	-0.271256732	0.999933887	0.64986409	0.739634735	-0.089770645	0.875814662
ENSG00000235481	*UBE2R2-AS1*	2.515576204	-0.052682293	-0.121177528	0.068495235	0.999933887	-0.05984177	-0.015786358	-0.044055412	0.87592751
ENSG00000130787	*HIP1R*	4.755317623	0.057355842	0.141955086	-0.084599244	0.999933887	0.110044288	0.137732126	-0.027687838	0.876025873
ENSG00000254838	*GVINP1*	4.510254878	0.104659581	-0.092412015	0.197071596	0.999933887	-0.347404532	-0.405105571	0.057701039	0.876025873
ENSG00000147854	*UHRF2*	4.820571231	-0.048748378	-0.100390463	0.051642085	0.999933887	-0.164247164	-0.201252335	0.037005171	0.876025873
ENSG00000198746	*GPATCH3*	4.173427624	-0.045645533	-0.045934396	0.000288863	0.999933887	0.039086066	0.067118733	-0.028032668	0.876025873
ENSG00000143891	*GALM*	2.852900847	-0.027858917	0.068546284	-0.096405201	0.999933887	0.014077195	-0.037019199	0.051096395	0.876025873
ENSG00000258819	*LINC02289*	0.574521167	-0.01751311	-0.025267873	0.007754763	0.999933887	-0.938266188	-0.849238608	-0.089027579	0.876025873
ENSG00000213741	*RPS29*	7.278744652	0.010100077	-0.016014709	0.026114786	0.999933887	0.130851013	0.159222185	-0.028371172	0.876025873
ENSG00000198276	*UCKL1*	3.414291795	0.026117601	0.036469141	-0.010351539	0.999933887	0.072669254	0.105998302	-0.033329048	0.876207006
ENSG00000100478	*AP4S1*	1.771702151	0.013728392	0.083148046	-0.069419654	0.999933887	-0.134688121	-0.088529298	-0.046158823	0.876214382
ENSG00000282608	*ADORA3*	1.359959232	-0.142440747	-0.074672514	-0.067768233	0.999933887	-0.017602403	0.028288015	-0.045890417	0.876286569
ENSG00000181007	*ZFP82*	-0.008023285	0.113446141	-0.365083831	0.478529972	0.999933887	-0.004648827	0.080932616	-0.085581443	0.876286569
ENSG00000005108	*THSD7A*	-1.452159367	0.147051264	0.049157916	0.097893348	0.999933887	-0.722336823	-0.628303104	-0.094033719	0.8764804
ENSG00000115232	*ITGA4*	6.122764319	0.011085042	-0.15974494	0.170829982	0.999933887	-0.296038408	-0.346798415	0.050760007	0.8764804
ENSG00000159882	*ZNF230*	3.983240978	-0.032215925	-0.08626576	0.054049835	0.999933887	-0.510262782	-0.539891384	0.029628602	0.876862464
ENSG00000119401	*TRIM32*	0.556290096	-0.108620663	0.018221064	-0.126841727	0.999933887	0.233271835	0.291903343	-0.058631508	0.876872998
ENSG00000196417	*ZNF765*	1.383176708	-0.089963749	-0.082118739	-0.007845009	0.999933887	-0.621131757	-0.557203081	-0.063928676	0.876872998
ENSG00000075945	*KIFAP3*	1.341388512	-0.022081114	-0.174007847	0.151926732	0.999933887	0.174744925	0.232440936	-0.057696011	0.876872998
ENSG00000236700	*LINC01010*	1.374134193	-0.396073391	-0.100674789	-0.295398602	0.999933887	-1.290310703	-1.242024589	-0.048286115	0.876941556
ENSG00000124688	*MAD2L1BP*	4.097185709	-0.069273458	-0.109576085	0.040302627	0.999933887	-0.142462755	-0.115237731	-0.027225025	0.87704154
ENSG00000130812	*ANGPTL6*	-0.787647703	-0.025785654	-0.021964002	-0.003821652	0.999933887	-0.160346962	-0.260610893	0.100263931	0.877427697
ENSG00000172059	*KLF11*	4.020923079	0.004496715	0.067496619	-0.062999904	0.999933887	-0.524737667	-0.489725563	-0.035012104	0.877653249
ENSG00000080823	*MOK*	1.62814637	0.14873257	0.163058758	-0.014326187	0.999933887	0.056997652	0.114612391	-0.057614739	0.877736607
ENSG00000153823	*PID1*	0.30512064	-0.167084126	0.034923562	-0.202007687	0.999933887	-0.020986758	0.021411041	-0.042397799	0.877875145
ENSG00000124562	*SNRPC*	2.866403826	0.029401893	-0.045043427	0.07444532	0.999933887	0.127317739	0.093192246	0.034125493	0.877875145
ENSG00000185986	*SDHAP3*	2.193048215	-0.042545705	0.094024151	-0.136569856	0.999933887	-0.095677694	-0.138576687	0.042898993	0.877875145
ENSG00000140563	*MCTP2*	6.508432242	-0.007359414	-0.039578117	0.032218703	0.999933887	-0.390218404	-0.360562407	-0.029655998	0.877875145
ENSG00000188976	*NOC2L*	3.668410643	0.043947527	0.033128279	0.010819248	0.999933887	0.117361045	0.086510562	0.030850483	0.877961825
ENSG00000010803	*SCMH1*	1.663943757	0.076234806	-0.007437028	0.083671834	0.999933887	0.02634503	-0.019645079	0.04599011	0.8779643
ENSG00000184575	*XPOT*	4.137872049	-0.04190736	-0.027217884	-0.014689477	0.999933887	-0.051871733	-0.025810474	-0.026061259	0.8779643
ENSG00000253729	*PRKDC*	6.4780967	-0.003621162	-0.017942035	0.014320872	0.999933887	-0.074155066	-0.04410527	-0.030049797	0.8779643
ENSG00000119514	*GALNT12*	0.115707768	0.049978305	0.060039635	-0.010061331	0.999933887	0.134682164	0.058307751	0.076374413	0.878002278
ENSG00000175854	*SWI5*	0.765151398	-0.041756	-0.09568532	0.05392932	0.999933887	-0.17930578	-0.249652243	0.070346463	0.878002278
ENSG00000119986	*AVPI1*	0.114846996	-0.071134112	-0.045478889	-0.025655223	0.999933887	-0.319752231	-0.409097865	0.089345634	0.878118476
ENSG00000149089	*APIP*	0.908075374	-0.032296482	-0.022309773	-0.009986709	0.999933887	-0.084941607	-0.034964282	-0.049977324	0.878280398
ENSG00000165233	*CARD19*	7.077661649	-0.015760176	0.019401399	-0.035161576	0.999933887	-0.434989714	-0.408345418	-0.026644295	0.878280398
ENSG00000232373	*MTCYBP3*	-0.211874779	0.045907976	0.175688719	-0.129780743	0.999933887	1.282168352	1.395862507	-0.113694155	0.878280398
ENSG00000269845	*CCNYL6*	1.099545545	0.216594329	0.267792613	-0.051198284	0.999933887	0.107422592	0.17856584	-0.071143248	0.878383974
ENSG00000196459	*TRAPPC2*	3.173783447	-0.100964213	-0.073073736	-0.027890476	0.999933887	0.051837018	0.022362381	0.029474636	0.878383974
ENSG00000078589	*P2RY10*	4.963380054	-0.022874836	-0.114288398	0.091413561	0.999933887	-0.130705208	-0.161742356	0.031037149	0.878383974
ENSG00000176658	*MYO1D*	0.326719981	0.010130891	0.092309563	-0.082178672	0.999933887	0.043416915	0.11867639	-0.075259475	0.879001341
ENSG00000153531	*ADPRHL1*	0.671389132	-0.087717827	0.168872367	-0.256590193	0.999933887	0.827667432	0.756251458	0.071415974	0.879174793
ENSG00000175104	*TRAF6*	4.83671591	-0.062050974	-0.022138006	-0.039912968	0.999933887	-0.197590342	-0.174683383	-0.022906959	0.879379165
ENSG00000149782	*PLCB3*	3.082797452	0.035633857	0.03251204	0.003121817	0.999933887	0.281640717	0.320898895	-0.039258179	0.879379165
ENSG00000105048	*TNNT1*	-0.486037783	-0.069150195	0.151196845	-0.22034704	0.999933887	-0.373978341	-0.485109406	0.111131065	0.879453338
ENSG00000129515	*SNX6*	5.674562051	-0.049917435	-0.119496015	0.069578579	0.999933887	-0.488729739	-0.463003181	-0.025726558	0.879494188
ENSG00000171169	*NAIF1*	3.770326936	-0.020976273	0.023193979	-0.044170252	0.999933887	-0.181848755	-0.144225344	-0.03762341	0.879535626
ENSG00000009954	*BAZ1B*	4.297611216	-0.029531043	-0.13568214	0.106151097	0.999933887	0.00494245	0.031918551	-0.026976101	0.879719912
ENSG00000138172	*CALHM2*	1.768501356	-0.03577955	-0.00713661	-0.02864294	0.999933887	-0.020889477	0.015659742	-0.036549219	0.879719912
ENSG00000102910	*LONP2*	4.094809493	-0.014997415	-0.047796095	0.03279868	0.999933887	-0.022125135	0.006800214	-0.028925349	0.879719912
ENSG00000272498	*AC090948.2*	-1.039151882	0.060132992	0.064353562	-0.004220571	0.999933887	-0.065947609	0.067628049	-0.133575658	0.879719912
ENSG00000279349	*AC092692.1*	-0.164716607	-0.053867337	0.277604313	-0.33147165	0.999933887	0.232505786	0.169102287	0.063403499	0.879719912
ENSG00000196696	*PDXDC2P-NPIPB14P*	1.416907143	0.009641928	0.07022593	-0.060584002	0.999933887	-0.055345611	-0.103595313	0.048249702	0.879719912
ENSG00000258424	*AL512791.1*	0.4059889	-0.120991236	-0.086018344	-0.034972892	0.999933887	-0.046443871	-0.121004961	0.07456109	0.879732831
ENSG00000169252	*ADRB2*	3.015150938	0.05247916	0.073286516	-0.020807356	0.999933887	0.260673091	0.301604211	-0.040931121	0.879792787
ENSG00000128965	*CHAC1*	-0.533442368	-0.041369614	-0.075177743	0.033808129	0.999933887	0.284572648	0.377273902	-0.092701254	0.879864151
ENSG00000224513	*AC109309.1*	-1.792367188	-0.262458507	0.126598568	-0.389057074	0.999933887	-0.168907592	-0.272488035	0.103580443	0.879883218
ENSG00000188747	*NOXA1*	0.109346857	0.168160389	0.284897353	-0.116736964	0.999933887	-0.042360389	-0.122905553	0.080545164	0.879883218
ENSG00000174231	*PRPF8*	7.741840091	-0.016042175	0.004958758	-0.021000932	0.999933887	-0.010013977	0.007534691	-0.017548668	0.879883218
ENSG00000214439	*FAM185BP*	-0.16908774	-0.057541631	-0.189882292	0.132340662	0.999933887	-0.069165477	-0.164176021	0.095010543	0.879883218
ENSG00000090382	*LYZ*	7.247352636	0.015503258	-0.024955579	0.040458837	0.999933887	0.018096271	-0.008796384	0.026892655	0.879883218
ENSG00000253307	*AC011676.1*	4.147661957	-0.004029642	-0.005756272	0.00172663	0.999933887	-0.123530296	-0.085028956	-0.038501339	0.880247472
ENSG00000174652	*ZNF266*	5.155370587	-0.024611692	-0.070571403	0.045959711	0.999933887	0.009775016	-0.011155918	0.020930934	0.880552569
ENSG00000249207	*AC079921.1*	-0.275092456	0.023101192	0.171694644	-0.148593452	0.999933887	0.179359815	0.26208994	-0.082730125	0.880570465
ENSG00000229512	*AC068580.1*	5.050922949	0.157073883	0.150846363	0.00622752	0.999933887	0.229923908	0.189773361	0.040150547	0.880707408
ENSG00000244932	*AL449212.1*	0.034789404	-0.057385535	-0.248333687	0.190948152	0.999933887	-0.313902365	-0.253009198	-0.060893167	0.880707408
ENSG00000145375	*SPATA5*	0.57892197	-0.036100853	-0.007079897	-0.029020956	0.999933887	-0.064951413	0.00016325	-0.065114663	0.880707408
ENSG00000286177	*AC011462.5*	1.182415397	-0.01645457	0.005140649	-0.021595219	0.999933887	-0.213994816	-0.262886043	0.048891227	0.880707408
ENSG00000106258	*CYP3A5*	-0.17799084	-0.020795108	-0.646050567	0.625255459	0.999933887	-0.283356011	-0.192064246	-0.091291765	0.880707408
ENSG00000107521	*HPS1*	6.566220092	-0.000479124	0.014433849	-0.014912973	0.999933887	0.037267049	0.01963334	0.017633709	0.880707408
ENSG00000156017	*CARNMT1*	1.47579602	-0.058173395	-0.048322976	-0.009850419	0.999933887	-0.153686154	-0.100327541	-0.053358612	0.881602629
ENSG00000047932	*GOPC*	2.618822689	-0.005870973	-0.078292389	0.072421416	0.999933887	-0.12341824	-0.085853463	-0.037564777	0.881776236
ENSG00000140105	*WARS1*	7.68236929	-0.029719821	-0.021961	-0.007758821	0.999933887	-0.105742464	-0.085644207	-0.020098257	0.882009998
ENSG00000169490	*TM2D2*	3.042640567	-0.091074404	-0.029194725	-0.061879679	0.999933887	-0.326687943	-0.291342684	-0.03534526	0.882014155
ENSG00000173726	*TOMM20*	4.807193336	-0.048607534	-0.079264562	0.030657028	0.999933887	0.076156982	0.101474422	-0.025317441	0.882014155
ENSG00000072756	*TRNT1*	3.250121617	0.075939595	0.010110653	0.065828942	0.999933887	0.214897083	0.251355497	-0.036458414	0.882014155
ENSG00000232973	*CYP1B1-AS1*	0.522331138	0.054446205	0.10643327	-0.051987065	0.999933887	0.330103192	0.274335177	0.055768015	0.882451287
ENSG00000168591	*TMUB2*	6.84493152	0.046664314	0.013916578	0.032747736	0.999933887	-0.127551996	-0.107056753	-0.020495243	0.882496451
ENSG00000115598	*IL1RL2*	1.700663389	-0.065938121	0.216898952	-0.282837073	0.999933887	0.591662447	0.64370503	-0.052042582	0.882496451
ENSG00000225399	*AC121247.1*	-0.275032161	0.052883151	-0.005033658	0.057916809	0.999933887	-0.430368976	-0.325000807	-0.105368169	0.882496451
ENSG00000002822	*MAD1L1*	1.718922575	-0.029588013	0.108338051	-0.137926064	0.999933887	0.283218497	0.334021989	-0.050803493	0.882496451
ENSG00000174353	*STAG3L3*	0.572892537	0.014842115	-0.065875126	0.080717241	0.999933887	-0.050142126	0.013953277	-0.064095402	0.882496451
ENSG00000087095	*NLK*	2.135514419	-0.009402308	0.180705292	-0.1901076	0.999933887	-0.09712918	-0.146726237	0.049597057	0.882496451
ENSG00000166780	*BMERB1*	0.9298983	0.013958294	0.140433768	-0.126475474	0.999933887	-0.327117707	-0.257858271	-0.069259436	0.882496451
ENSG00000139194	*RBP5*	1.48796362	0.089649341	-0.029169861	0.118819202	0.999933887	0.117399156	0.058258289	0.059140867	0.882886751
ENSG00000165526	*RPUSD4*	2.373235253	-0.06602896	0.087215965	-0.153244925	0.999933887	0.193983016	0.160702756	0.03328026	0.882886751
ENSG00000074706	*IPCEF1*	4.21088452	-0.024372709	-0.015992369	-0.008380341	0.999933887	-0.215338472	-0.18855734	-0.026781131	0.882886751
ENSG00000287643	*AC004825.3*	0.498630052	0.13849966	0.079672931	0.058826729	0.999933887	-0.092715173	0.001769022	-0.094484195	0.882970447
ENSG00000270554	*AC069431.2*	-0.327396397	0.059548609	-0.049949023	0.109497631	0.999933887	-0.45459297	-0.316866782	-0.137726188	0.882970447
ENSG00000164830	*OXR1*	3.088262798	0.019724962	-0.009033068	0.02875803	0.999933887	-0.533833554	-0.49103179	-0.042801763	0.882970447
ENSG00000287261	*AL139243.1*	0.802098401	0.192305311	0.218242894	-0.025937583	0.999933887	0.441633741	0.393450358	0.048183383	0.883207378

ENSG00000188603	*CLN3*	4.580791284	0.037770607	-0.019367635	0.057138242	0.999933887	-0.00391984	0.018567537	-0.022487378	0.883207378
ENSG00000204498	*NFKBIL1*	2.411690374	0.041769855	-0.141397924	0.183167779	0.999933887	-0.028547593	0.012617223	-0.041164816	0.883207378
ENSG00000101294	*HM13*	7.83017233	0.018428819	-0.042977974	0.061406793	0.999933887	-0.238590207	-0.258655363	0.020065156	0.883207378
ENSG00000162813	*BPNT1*	0.467675966	-0.045651267	0.179504396	-0.225155663	0.999933887	0.168751248	0.23033444	-0.061583192	0.883207378
ENSG00000155858	*LSM11*	0.618729916	-0.398438738	0.006518527	-0.404957265	0.999933887	0.050546171	-0.012090145	0.062636316	0.883461887
[truncated: 338,611 more chars]
