# Supplementary material for: Neutrophil extracellular trap formation and gene programs distinguish TST/IGRA sensitization outcomes among Mycobacterium tuberculosis exposed persons living with HIV
Source: PLoS Genet. 2023 Aug 24;19(8):e1010888. doi: 10.1371/journal.pgen.1010888 (PMC10470897; doi:10.1371/journal.pgen.1010888)
Supplement: S4 Table — (PDF) [file pgen.1010888.s004.pdf]

S4 Table: Sample characteristics

Characteristic	$\mathbf{PMN_{HITTIN}}$ [a], N = 17[*]	$\mathbf{PMN_{HIT}}$ [b], N = 11[*]	p-value[**]
**Sequencer**			0.4
HiSeq	3/ 17 (18%)	4/ 11 (36%)	
NovaSeq	14/ 17 (82%)	7/ 11 (64%)	

[*]n/ N (%); Mean (SD) [**]Fisher's exact test; Wilcoxon rank sum test

[a] PMN_{HITTIN} (neutrophils from HIV-1-infected persistently TB, tuberculin and IGRA negative), [b] PMN_{HIT} (neutrophils from HIV-1-infected IGRA positive tuberculin positive))
