# Supplementary material for: Neutrophil extracellular trap formation and gene programs distinguish TST/IGRA sensitization outcomes among Mycobacterium tuberculosis exposed persons living with HIV
Source: PLoS Genet. 2023 Aug 24;19(8):e1010888. doi: 10.1371/journal.pgen.1010888 (PMC10470897; doi:10.1371/journal.pgen.1010888)
Supplement: S1 Fig — Volcano plot for transcriptional responses to Mtb challenge for neutrophils from HITTIN (PMNHITTIN) and HIT (PMNHIT) participants at 1h (A-C) post Mtb infection. The y-axis shows the negative log10 unadjusted P value and the x-axis the log2 fold change (FC). The vertical dashed lines represent log2 FC thresholds of -0.2 and 0.2. Each gene is represented by a dot. Genes which are downregulated or upregulated as determined by the FDR ≤ 5% are shown in blue and red, respectively. Genes with non-significant expression changes and below the log2 FC threshold are shown in grey. Differentially expressed genes at 1h post-infection compared to 1h uninfected PMN from HITTIN (PMNHITTIN) (A) and HIT participants (PMNHIT) (B). Significant differentially triggered genes between PMNHITTIN and PMNHIT at 1h post infection (C). (PDF) [file pgen.1010888.s008.pdf]

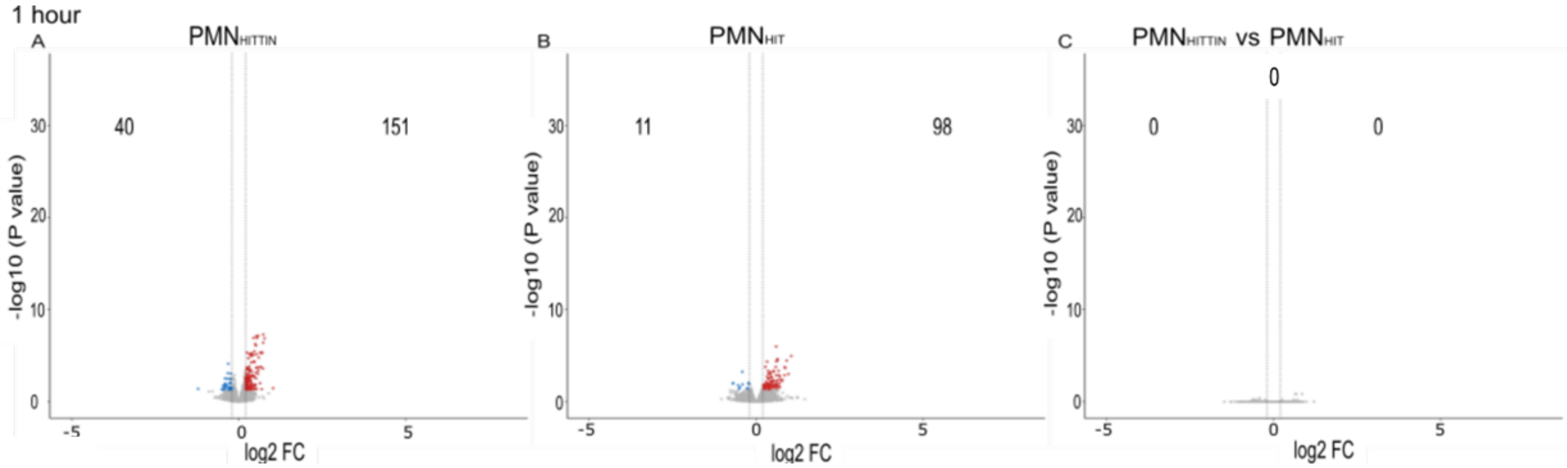

S1 Fig: Volcano plots of differential gene expression at 1h infection by PMN from HITTIN and HIT

Volcano plot for transcriptional responses to *Mtb* challenge for neutrophils from HITTIN (PMN_{HITTIN}) and HIT (PMN_{HIT}) participants at 1h **(A-C)** post *Mtb* infection. The y-axis shows the negative log10 unadjusted P value and the x-axis the log_2 fold change (FC). The vertical dashed lines represent log_2 FC thresholds of -0.2 and 0.2. Each gene is represented by a dot. Genes which are downregulated or upregulated as determined by the FDR ≤ 5% are shown in blue and red, respectively. Genes with non-significant expression changes and below the log_2 FC threshold are shown in grey. Differentially expressed genes at 1h post-infection compared to 1h uninfected PMN from HITTIN (PMN_{HITTIN}) **(A)** and HIT participants (PMN_{HIT}) **(B)**. Significant differentially triggered genes between PMN_{HITTIN} and PMN_{HIT} at 1h post infection **(C)**.
