# Supplementary material for: Neutrophil extracellular trap formation and gene programs distinguish TST/IGRA sensitization outcomes among Mycobacterium tuberculosis exposed persons living with HIV
Source: PLoS Genet. 2023 Aug 24;19(8):e1010888. doi: 10.1371/journal.pgen.1010888 (PMC10470897; doi:10.1371/journal.pgen.1010888)
Supplement: S2 Fig — “HITTIN 1h infected” represents the log2FC gene expression effect when comparing the 1h Mtb infection to the 1h uninfected response in neutrophils (PMN) from HIV-1-positive persistently TB, tuberculin and IGRA negative individuals (PMNHITTIN)(x-axis). “HITTIN 6h infected” is the same but for 6h. “HIT 1h infected” represents the log2FC gene expression effect when comparing the 1h Mtb infection to the 1h uninfected response in PMN from HIV-1-positive, IGRA positive, tuberculin positive individuals (PMNHIT)(y-axis). “HIT 6h infected” is the same but for 6h. Scatterplots display the correlation of the log2FC gene expression as defined for each group at 1h (A and B) or 6h (C and D). Each grey dot represents a differentially expressed gene (DEGs). The grey dots show combined DEGs from both groups represented on the x and y axis. Blue coloured dots indicate DEGs from the x-axis group and red dots indicate the y-axis group. Pearson correlation (R value) was calculated for the coloured DEGs. The log2FC for each group is plotted with the y-axis as the reference group. Differential gene expression is more pronounced in genes with the highest log2FC, with the majority of genes displaying good correlation between phenotype groups. (PDF) [file pgen.1010888.s009.pdf]

DEGs from the x-axis group **DEGs from the y-axis group**

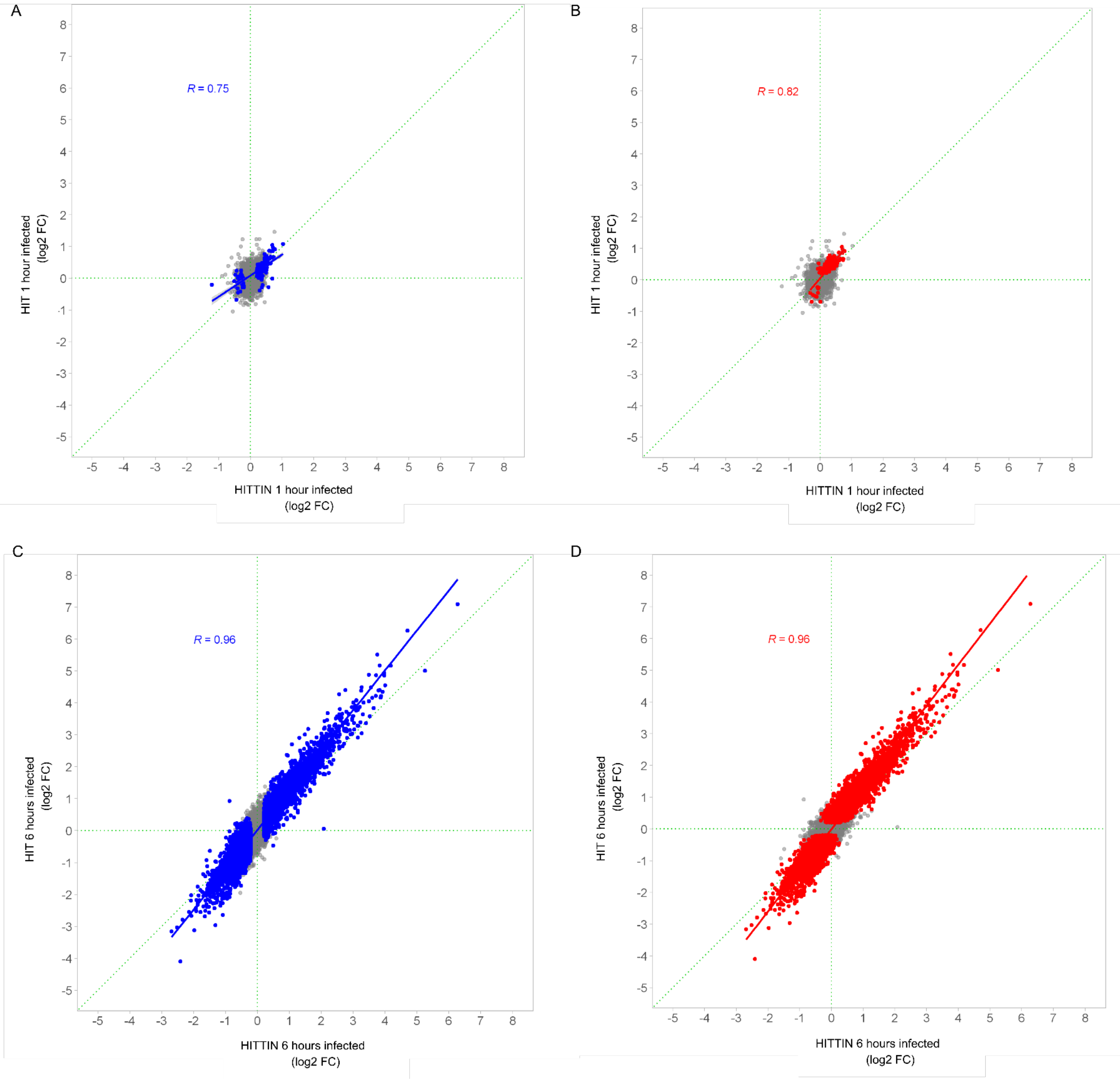

S2 Fig: Correlation plots of log_2FC gene expression after *Mtb* infection of PMN from HITTIN and HIT

"HITTIN 1h infected" represents the log_2FC gene expression effect when comparing the 1h *Mtb* infection to the 1h uninfected response in neutrophils (PMN) from HIV-1-positive persistently TB, tuberculin and IGRA negative individuals (PMN_{HITTIN})(x-axis). "HITTIN 6h infected" is the same but for 6h. "HIT 1h infected" represents the log_2FC gene expression effect when comparing the 1h Mtb infection to the 1h uninfected response in PMN from HIV-1-positive, IGRA positive, tuberculin positive individuals (PMN_{HIT})(y-axis). "HIT 6h infected" is the same but for 6h. Scatterplots display the correlation of the log_2FC gene expression as defined for each group at 1h **(A and B)** or 6h **(C and D)**. Each grey dot represents a differentially expressed gene (DEGs). The grey dots show combined DEGs from both groups represented on the x and y axis. Blue coloured dots indicate DEGs from the x-axis group and red dots indicate the y-axis group. Pearson correlation (R value) was calculated for the coloured DEGs. The log_2FC for each group is plotted with the y-axis as the reference group. Differential gene expression is more pronounced in genes with the highest log_2FC, with the majority of genes displaying good correlation between phenotype groups.
