# Supplementary material for: Neutrophil extracellular trap formation and gene programs distinguish TST/IGRA sensitization outcomes among Mycobacterium tuberculosis exposed persons living with HIV
Source: PLoS Genet. 2023 Aug 24;19(8):e1010888. doi: 10.1371/journal.pgen.1010888 (PMC10470897; doi:10.1371/journal.pgen.1010888)
Supplement: S3 Fig — Scale bars: 100 μm (A-C) and 50 μm (D-I). Isolated neutrophils were stimulated with Mtb and stained with Hoechst 33342 (A and boxed area D) and PL2-3 (B and boxed area E). C and the boxed area F show the overlap (blue, DNA; yellow, chromatin). The segmentation of fluorescent signals used to calculate the neutrophil nuclei area (G) and neutrophil extracellular trap area (H) with the overlap shown in (I). The scale bars represent 100 μm (A-C) and 50 μm (D-I). (PDF) [file pgen.1010888.s010.pdf]

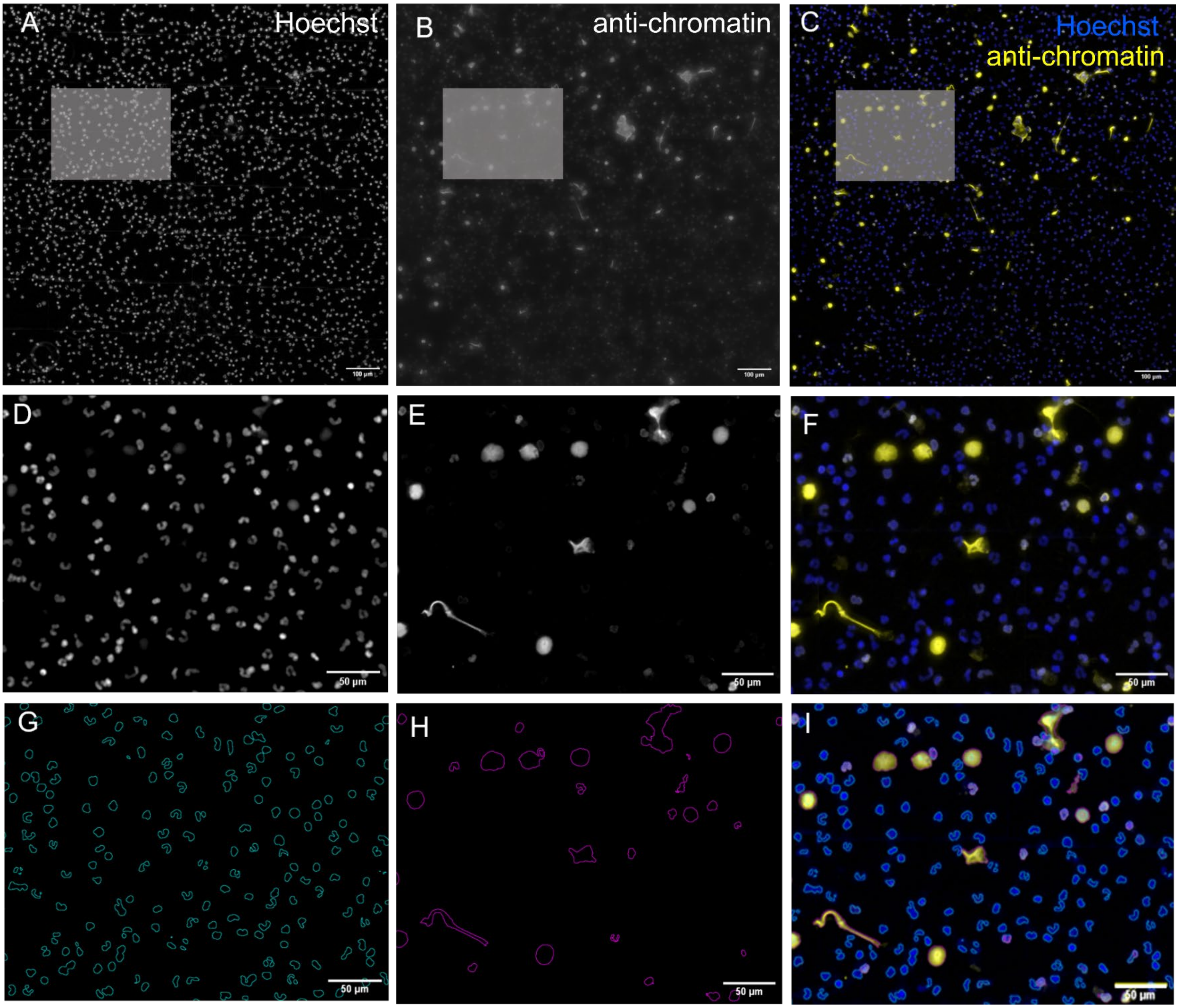

S3 Fig: Fluorescence imaging process overview to calculate neutrophil nuclei and neutrophil extracellular trap (chromatin) area. Scale bars: 100 µm (A-C) and 50 µm (D-I).

Isolated neutrophils were stimulated with *Mtb* and stained with Hoechst 33342 **(A and boxed area D)** and PL2-3 **(B and boxed area E)**. **C and the boxed area F** show the overlap (blue, DNA; yellow, chromatin). The segmentation of fluorescent signals used to calculate the neutrophil nuclei area **(G)** and neutrophil extracellular trap area **(H)** with the overlap shown in **(I)**. The scale bars represent 100 µm **(A-C)** and 50 µm **(D-I)**.
