# Supplementary material for: Neutrophil extracellular trap formation and gene programs distinguish TST/IGRA sensitization outcomes among Mycobacterium tuberculosis exposed persons living with HIV
Source: PLoS Genet. 2023 Aug 24;19(8):e1010888. doi: 10.1371/journal.pgen.1010888 (PMC10470897; doi:10.1371/journal.pgen.1010888)
Supplement: S4 Fig — The first gate was applied to exclude debris (the low SSC-H, FSC-H values in the bottom left corner). Single cells were separated and gated by a CD45+ marker for leukocytes. After single cell gating, CD45+ cells were then grouped into CD15+CD66b+ (granulocytes) and CD15-CD66b-(non-granulocytes) cells. The CD15+CD66b+ cells were further classified as CD14- CD16+ (Neutrophils) and CD14- CD16- (Eosinophils). CD15-CD66b- were stratified as CD3+ (T-cells), CD3- CD14+ (Monocytes) and CD14- CD3- (Other) cells. (PDF) [file pgen.1010888.s011.pdf]

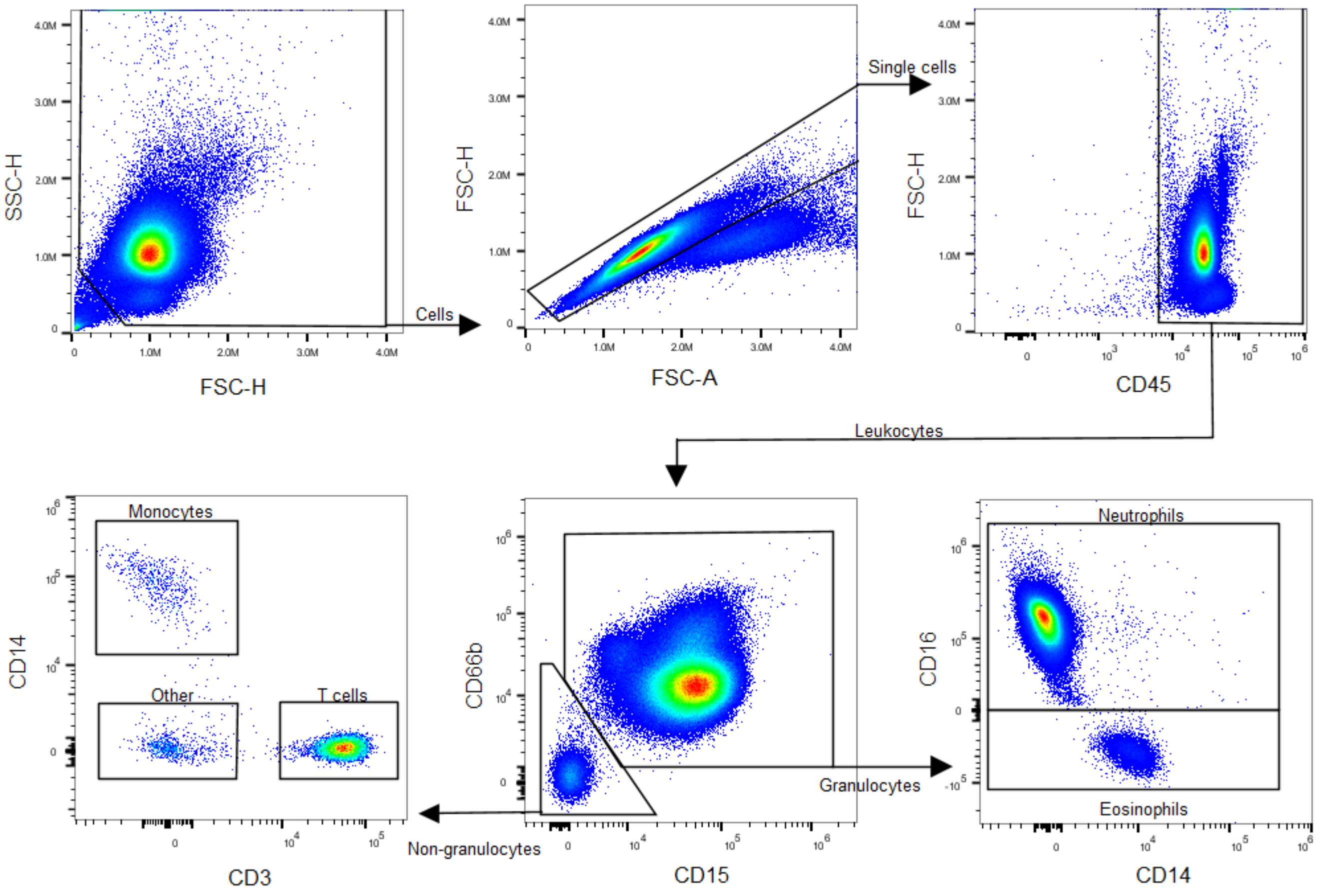

S4 Fig: Flow Cytometry Analysis of Cell Populations

The first gate was applied to exclude debris (the low SSC-H, FSC-H values in the bottom left corner). Single cells were separated and gated by a CD45+ marker for leukocytes. After single cell gating, CD45+ cells were then grouped into CD15+CD66b+ (granulocytes) and CD15-CD66b-(non-granulocytes) cells. The CD15+CD66b+ cells were further classified as CD14- CD16+ (Neutrophils) and CD14- CD16- (Eosinophils). CD15-CD66b- were stratified as CD3+ (T-cells), CD3- CD14+ (Monocytes) and CD14- CD3- (Other) cells.
