# Supplementary material for: Neutrophil extracellular trap formation and gene programs distinguish TST/IGRA sensitization outcomes among Mycobacterium tuberculosis exposed persons living with HIV
Source: PLoS Genet. 2023 Aug 24;19(8):e1010888. doi: 10.1371/journal.pgen.1010888 (PMC10470897; doi:10.1371/journal.pgen.1010888)
Supplement: S5 Fig — The multidimensional scaling (MDS) plots the Euclidian distances between samples with the x and y axis representing the sample distances between samples of read counts normalized by depth but not covariates. Each row of plots in (A) and (B) represent dimension 1 to 5 respectively (represented by the x-axis) and shown with the combination of the other dimensions on the y-axis. Samples are colored for batch effect with green showing samples sequenced on Illumina HiSeq2500 and blue for samples sequenced on Illumina NovaSeq6000. (A) shows the multiple combination of plots by dimension for the normalized counts prior to batch correction. Clear separation of samples by batch can be seen and is most notable in dimensions 4 and 5. (B) Appropriate batch correction after correction of raw counts with ComBat-seq. After batch correct outlier samples are most notably observed in dimension 3. (PDF) [file pgen.1010888.s012.pdf]

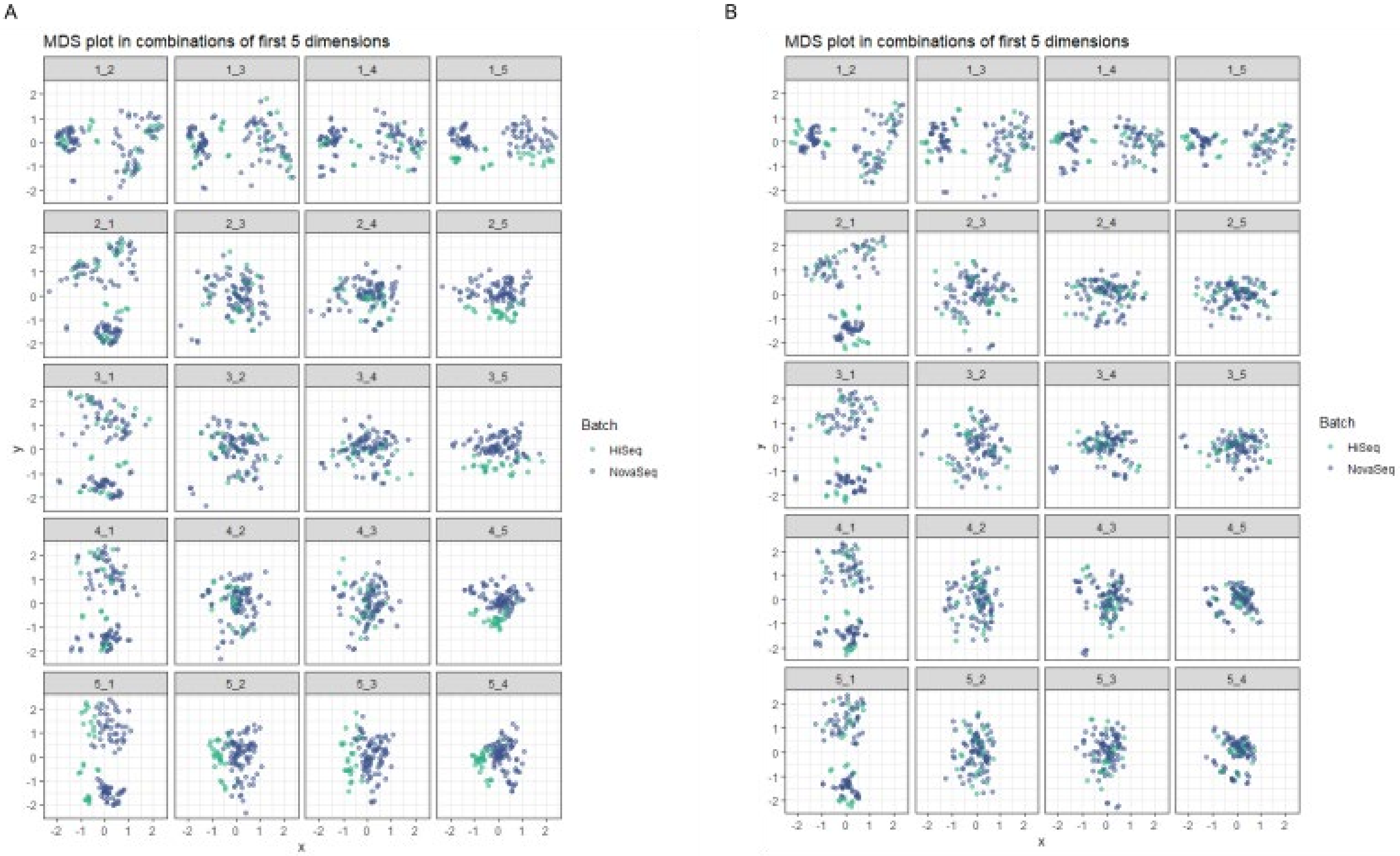

S5 Fig: Multidimensional scaling (MDS) plot before (A) and after (B) ComBat-seq batch correction

The multidimensional scaling (MDS) plots the Euclidian distances between samples with the x and y axis representing the sample distances between samples of read counts normalized by depth but not covariates. Each row of plots in **(A)** and **(B)** represent dimension 1 to 5 respectively (represented by the x-axis) and shown with the combination of the other dimensions on the y-axis. Samples are colored for batch effect with green showing samples sequenced on Illumina HiSeq2500 and blue for samples sequenced on Illumina NovaSeq6000. **(A)** shows the multiple combination of plots by dimension for the normalized counts prior to batch correction. Clear separation of samples by batch can be seen and is most notable in dimensions 4 and 5. **(B)** Appropriate batch correction after correction of raw counts with ComBat-seq. After batch correct outlier samples are most notably observed in dimension 3.
