# Supplementary material for: Neutrophil extracellular trap formation and gene programs distinguish TST/IGRA sensitization outcomes among Mycobacterium tuberculosis exposed persons living with HIV
Source: PLoS Genet. 2023 Aug 24;19(8):e1010888. doi: 10.1371/journal.pgen.1010888 (PMC10470897; doi:10.1371/journal.pgen.1010888)
Supplement: S6 Fig — Density plot with the density represented on the y-axis for the log-cpm values before (A) and after (B) filtering. The dotted vertical line is equivalent to the counts per million (CPM) threshold of 1.32 which was used in the filtering step. (PDF) [file pgen.1010888.s013.pdf]

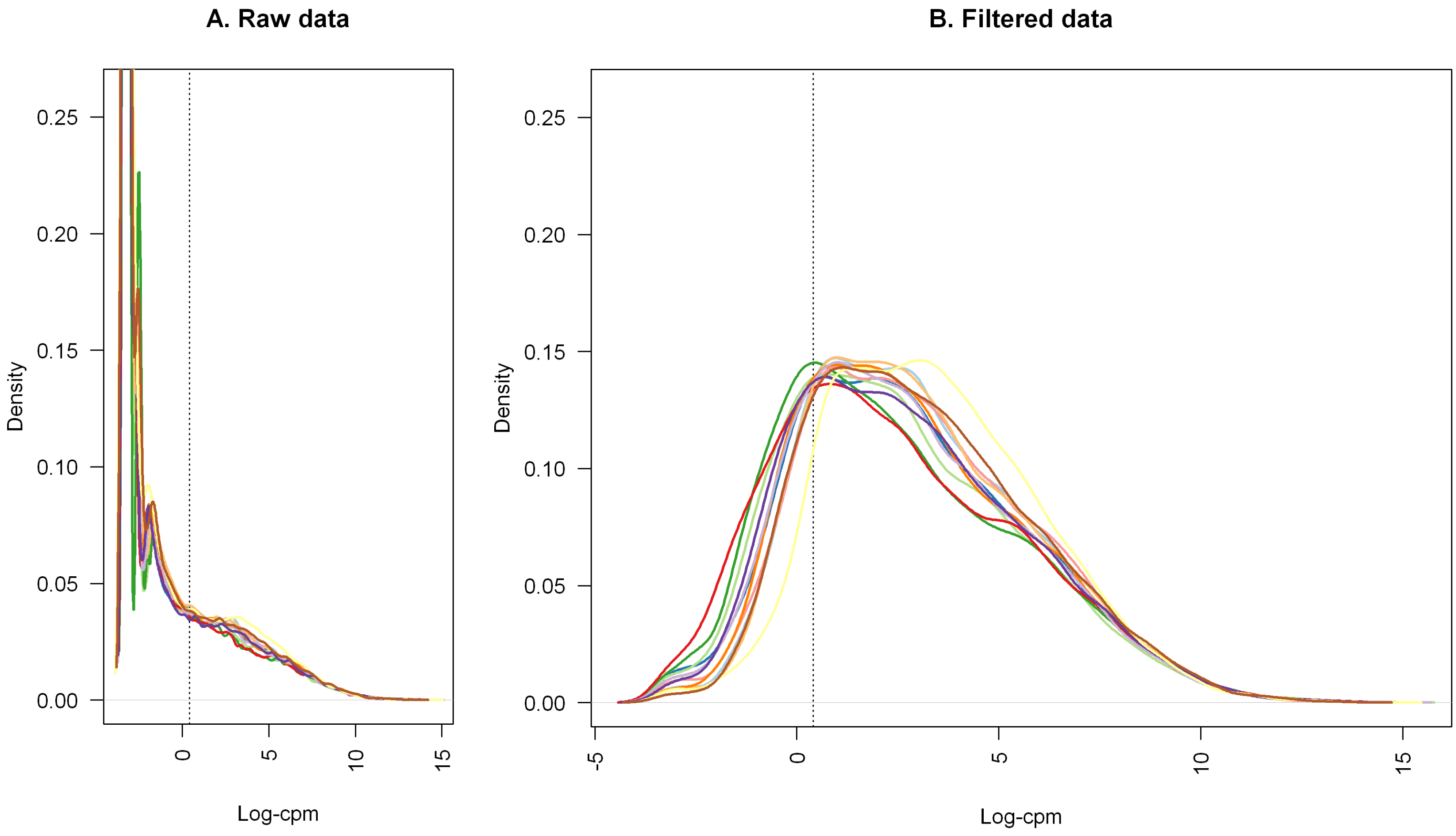

S6 Fig: Density plot showing successful filtering applied to counts.

Density plot with the density represented on the y-axis for the log-cpm values before **(A)** and after **(B)** filtering. The dotted vertical line is equivalent to the counts per million (CPM) threshold of 1.32 which was used in the filtering step.
