# Supplementary material for: Neutrophil extracellular trap formation and gene programs distinguish TST/IGRA sensitization outcomes among Mycobacterium tuberculosis exposed persons living with HIV
Source: PLoS Genet. 2023 Aug 24;19(8):e1010888. doi: 10.1371/journal.pgen.1010888 (PMC10470897; doi:10.1371/journal.pgen.1010888)
Supplement: S7 Fig — (A) shows rescaled (square-root of standard deviations) residual variances plotted against the mean expression (log2 transformed with an offset of 2) of each gene. A decreasing trend is seen between the mean gene expression and the variance with higher expressed genes showing less variation. The sample specific weight for each sample used in the analysis is shown in (B). A total of 112 samples were analysed (28 participants and 4 conditions, uninfected and infected after 1 and 6h). (PDF) [file pgen.1010888.s014.pdf]

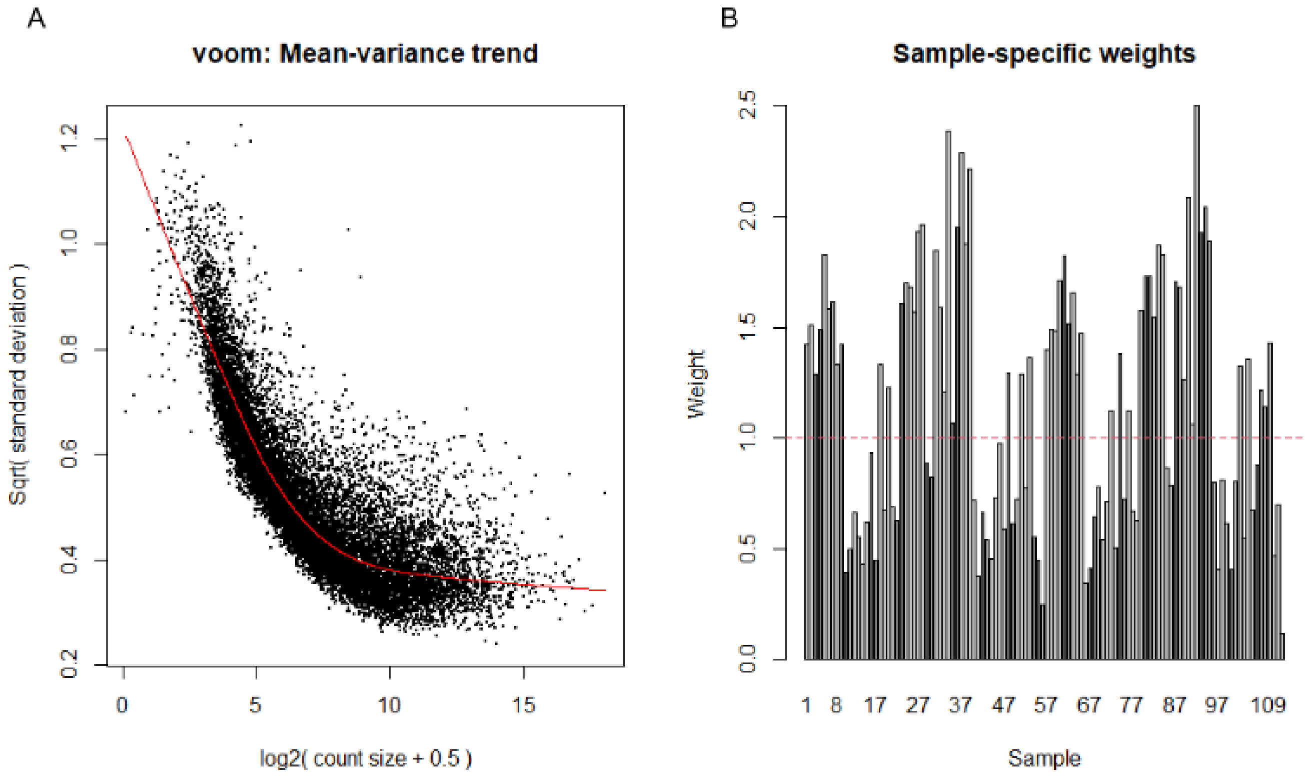

S7 Fig: Voom observational mean-variance trend and sample-specific weights

(A) shows rescaled (square-root of standard deviations) residual variances plotted against the mean expression ($\log_2$ transformed with an offset of 2) of each gene. A decreasing trend is seen between the mean gene expression and the variance with higher expressed genes showing less variation. The sample specific weight for each sample used in the analysis is shown in **(B)**. A total of 112 samples were analysed (28 participants and 4 conditions, uninfected and infected after 1 and 6h).
