# Supplementary material for: Neutrophil extracellular trap formation and gene programs distinguish TST/IGRA sensitization outcomes among Mycobacterium tuberculosis exposed persons living with HIV
Source: PLoS Genet. 2023 Aug 24;19(8):e1010888. doi: 10.1371/journal.pgen.1010888 (PMC10470897; doi:10.1371/journal.pgen.1010888)
Supplement: S8 Fig — The multidimensional scaling (MDS) plots the Euclidian distances between samples with the x and y axis representing the sample distances between samples of read counts normalized by depth but not covariates. Each row of plots in represent dimension 1 to 5 respectively (represented by the x-axis) and shown with the combination of the other dimensions on the y-axis. Samples are colored for phenotype (HITTIN and HIT), timepoint (1h and 6h) and infection status (uninfected [NEG] and infected [INF]) and as depicted in the legend. Separation of the groups by time can be seen in dimension 1, by infection in dimension 2 and the phenotype in dimension 3. Additional separation is seen in dimension 4 and 5 due to sex and possibly smoking (see S8 and S9 Figs). (PDF) [file pgen.1010888.s015.pdf]

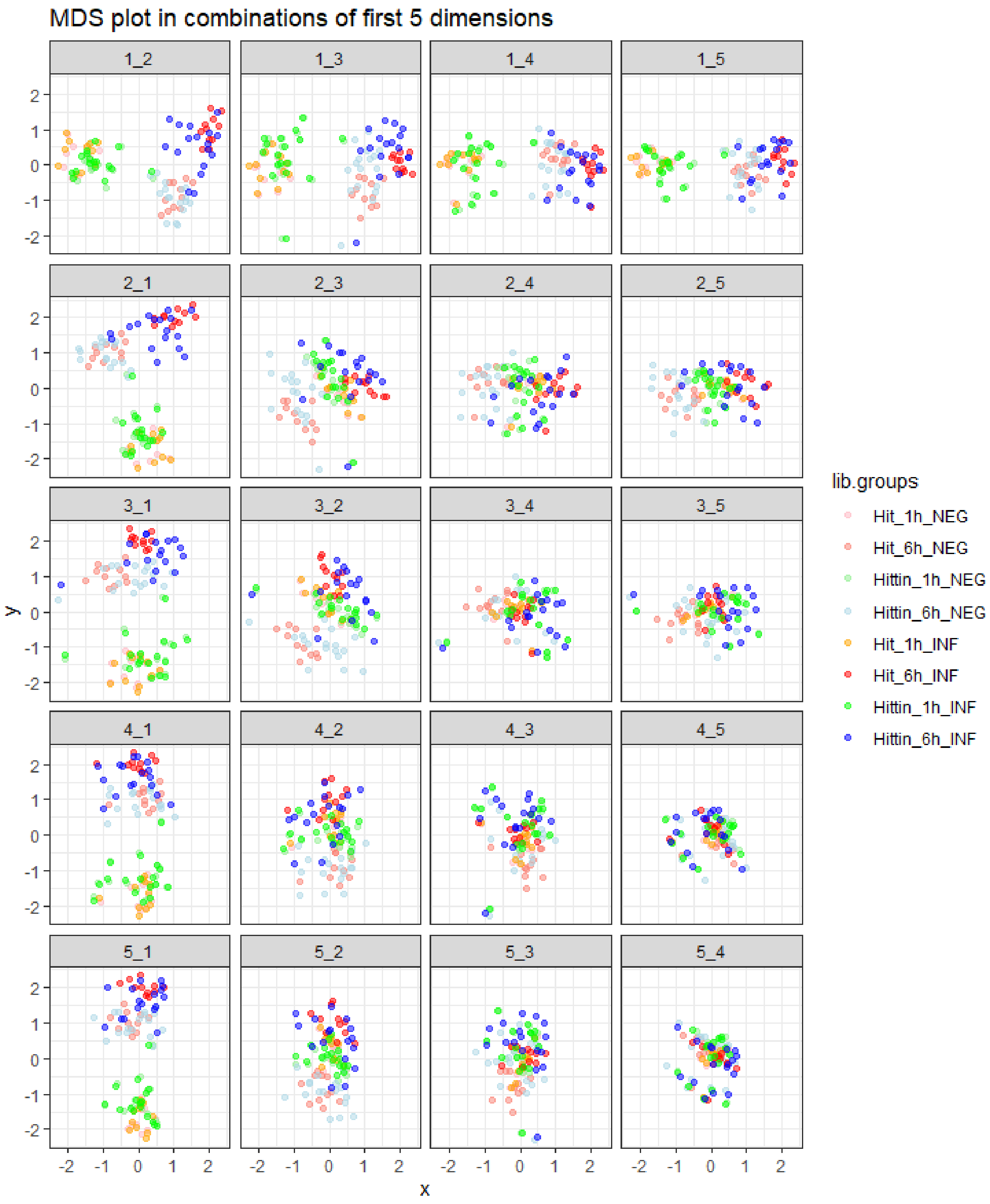

S8 Fig: Multidimensional scaling (MDS) plot of group separation

The multidimensional scaling (MDS) plots the Euclidian distances between samples with the x and y axis representing the sample distances between samples of read counts normalized by depth but not covariates. Each row of plots in represent dimension 1 to 5 respectively (represented by the x-axis) and shown with the combination of the other dimensions on the y-axis. Samples are colored for phenotype (HITTIN and HIT), timepoint (1h and 6h) and infection status (uninfected [NEG] and infected [INF]) and as depicted in the legend. Separation of the groups by time can be seen in

dimension 1, by infection in dimension 2 and the phenotype in dimension 3. Additional separation is seen in dimension 4 and 5 due to sex and possibly smoking (see S8 and S9 Figs).
