# Supplementary material for: Neutrophil extracellular trap formation and gene programs distinguish TST/IGRA sensitization outcomes among Mycobacterium tuberculosis exposed persons living with HIV
Source: PLoS Genet. 2023 Aug 24;19(8):e1010888. doi: 10.1371/journal.pgen.1010888 (PMC10470897; doi:10.1371/journal.pgen.1010888)
Supplement: S9 Fig — The top 5 principal components are represented on the x-axis and the variance explained by each component on the y-axis. Principal component (PC) 1 contributes to most of the variance seen and represents time. PC2 represent infection effect and PC3 the phenotype groups. (PDF) [file pgen.1010888.s016.pdf]

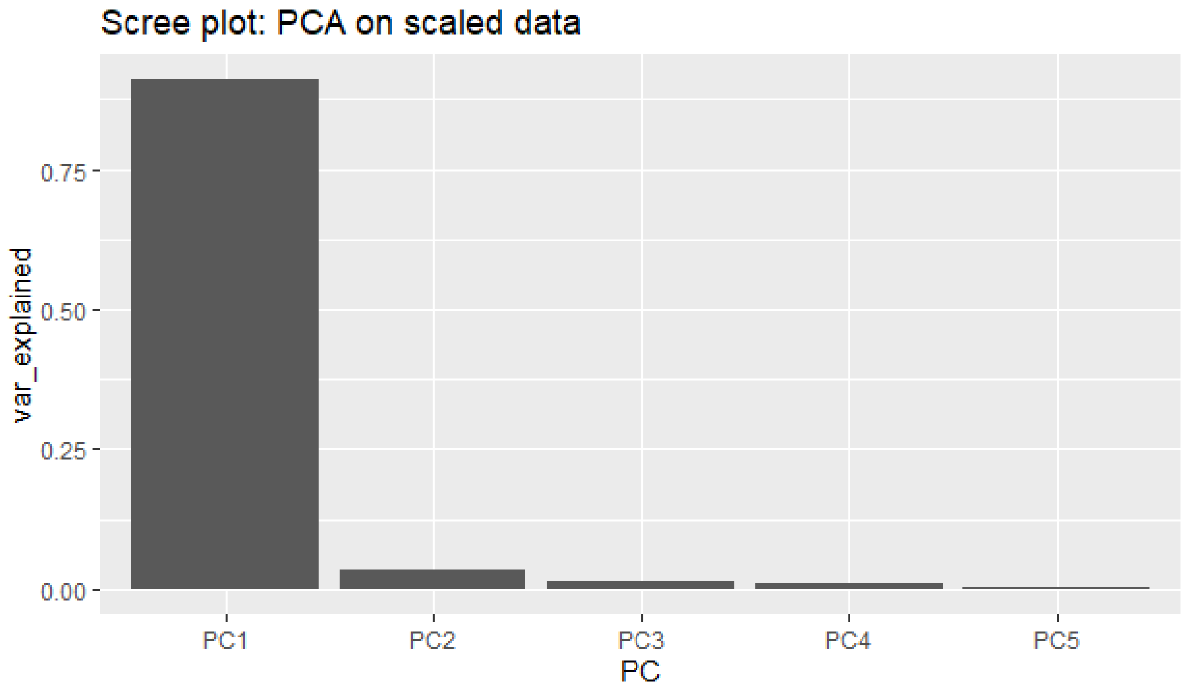

S9 Fig: Scree plot

The top 5 principal components are represented on the x-axis and the variance explained by each component on the y-axis. Principal component (PC) 1 contributes to most of the variance seen and represents time. PC2 represent infection effect and PC3 the phenotype groups.
