# Supplementary material for: Neutrophil extracellular trap formation and gene programs distinguish TST/IGRA sensitization outcomes among Mycobacterium tuberculosis exposed persons living with HIV
Source: PLoS Genet. 2023 Aug 24;19(8):e1010888. doi: 10.1371/journal.pgen.1010888 (PMC10470897; doi:10.1371/journal.pgen.1010888)
Supplement: S11 Fig — The multidimensional scaling (MDS) plots the Euclidian distances between samples with the x and y axis representing the sample distances between samples of read counts normalized by depth but not covariates. Each row of plots in represent dimension 1 to 5 respectively (represented by the x-axis) and shown with the combination of the other dimensions on the y-axis. Samples are colored for participants classified as smokers or not as depicted in the legend. Each participant is represented by 4 dots (1h uninfected and infected; 6h uninfected and infected). There are only two smokers making it difficult to comment on separation due to smoking. (PDF) [file pgen.1010888.s018.pdf]

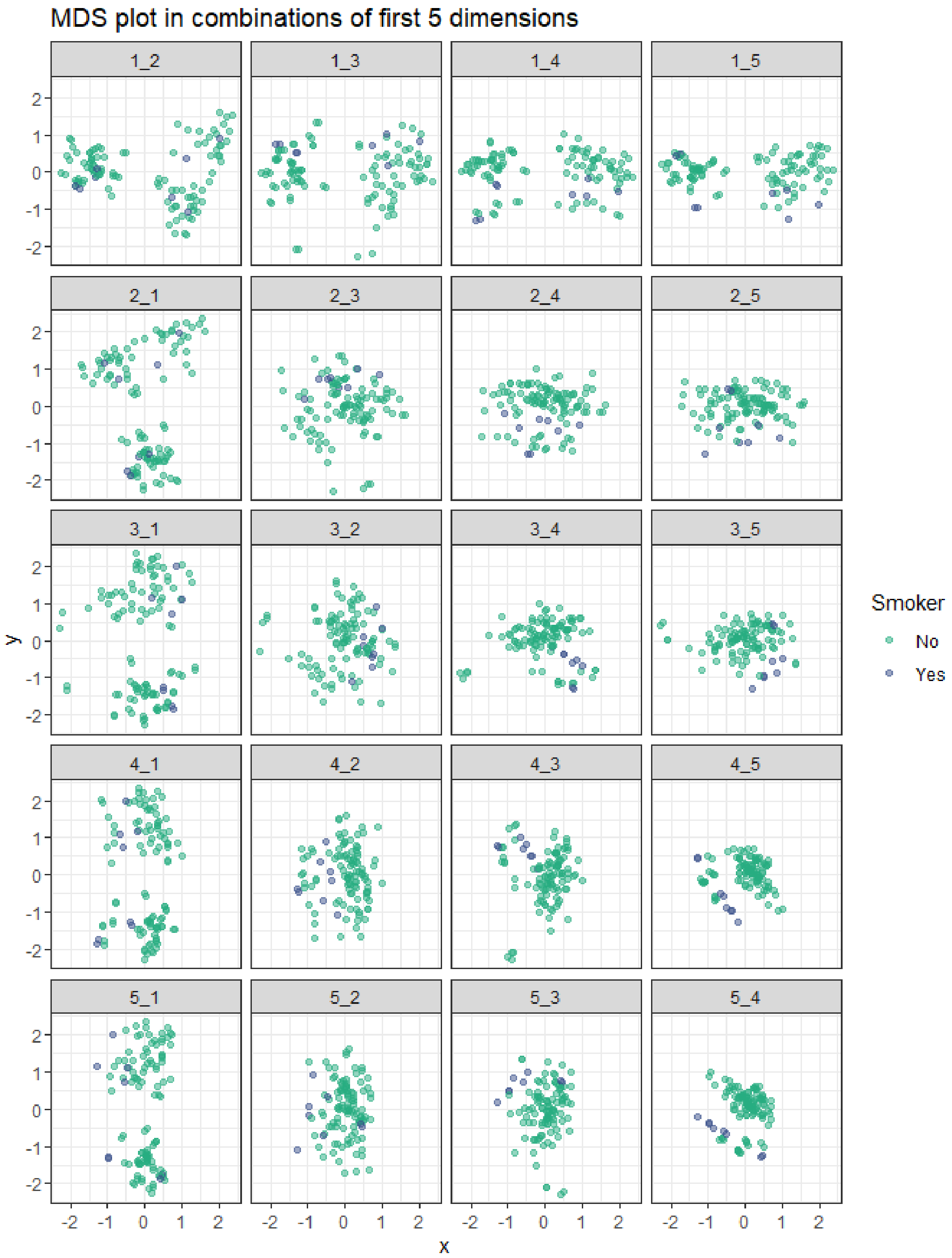

S11 Fig: Multidimensional scaling (MDS) plot of participant smoking habit

The multidimensional scaling (MDS) plots the Euclidian distances between samples with the x and y axis representing the sample distances between samples of read counts normalized by depth but not covariates. Each row of plots in represent dimension 1 to 5 respectively (represented by the x-axis) and shown with the combination of the other dimensions on the y-axis. Samples are colored for participants classified as smokers or not as depicted in the legend. Each participant is represented by 4 dots (1h uninfected and infected; 6h uninfected and infected). There are only two smokers making it difficult to comment on separation due to smoking.
