# Supplementary material for: Neutrophil extracellular trap formation and gene programs distinguish TST/IGRA sensitization outcomes among Mycobacterium tuberculosis exposed persons living with HIV
Source: PLoS Genet. 2023 Aug 24;19(8):e1010888. doi: 10.1371/journal.pgen.1010888 (PMC10470897; doi:10.1371/journal.pgen.1010888)
Supplement: S13 Fig — The multidimensional scaling (MDS) plots the Euclidian distances between samples with the x and y axis representing the sample distances between samples of read counts normalized by depth but not covariates. Each row of plots in represent dimension 1 to 5 respectively (represented by the x-axis) and shown with the combination of the other dimensions on the y-axis. Samples are colored for the numbers of months participants previously used INH (0 or none, 2, 6, 12, 24 or 36 months) or participants who are currently using INH, as depicted in the legend. There is no clear separation based on duration of INH prophylaxis use. (PDF) [file pgen.1010888.s020.pdf]

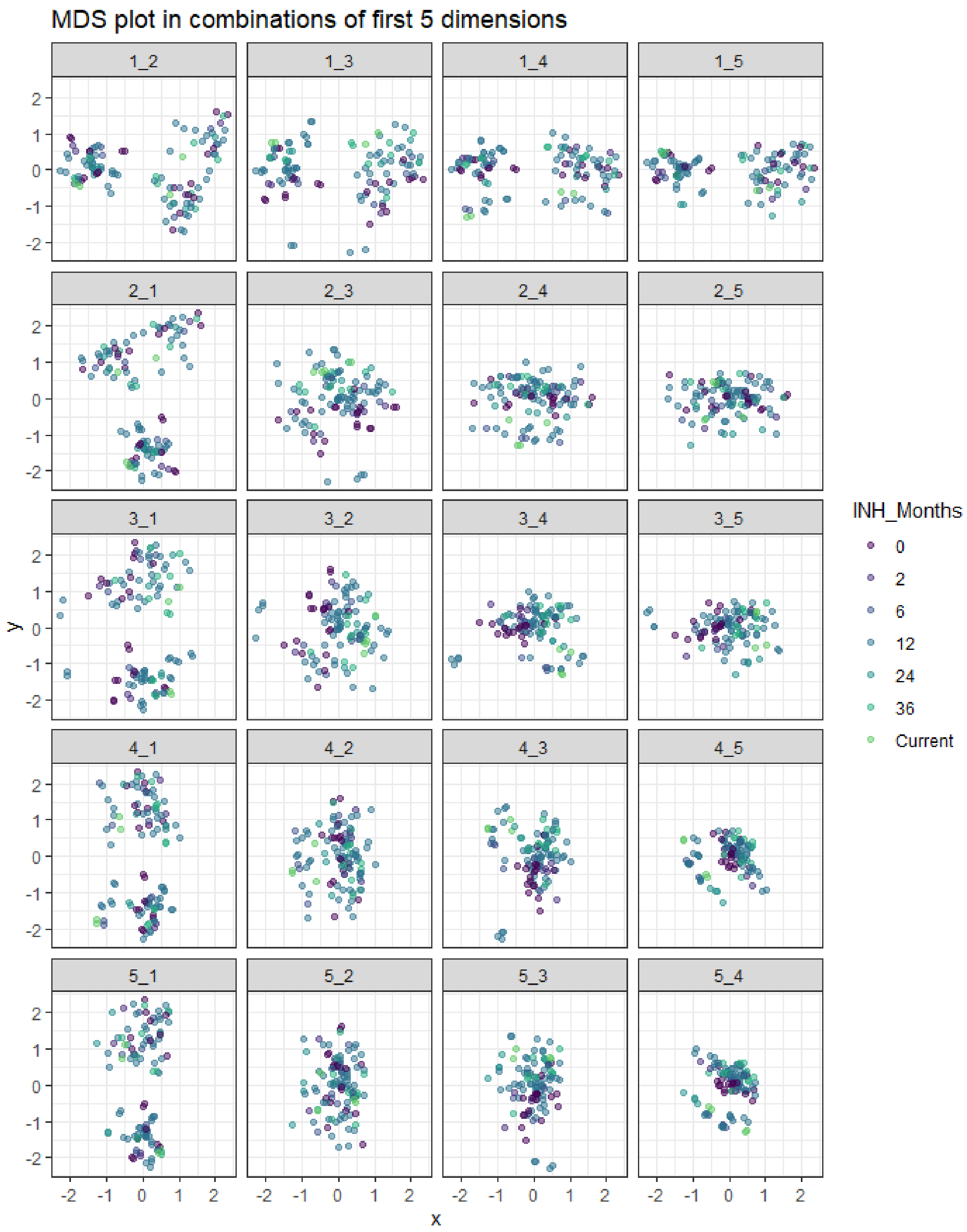

S13 Fig: Multidimensional scaling (MDS) plot of duration of participant isoniazid (INH) prophylaxis use in months

The multidimensional scaling (MDS) plots the Euclidian distances between samples with the x and y axis representing the sample distances between samples of read counts normalized by depth but not covariates. Each row of plots in represent dimension 1 to 5 respectively (represented by the x-axis) and shown with the combination of the other dimensions on the y-axis. Samples are colored for the numbers of months participants previously used INH (0 or none, 2, 6, 12, 24 or 36 months) or participants who are currently using INH, as depicted in the legend. There is no clear separation based on duration of INH prophylaxis use.
