# Supplementary material for: Neutrophil extracellular trap formation and gene programs distinguish TST/IGRA sensitization outcomes among Mycobacterium tuberculosis exposed persons living with HIV
Source: PLoS Genet. 2023 Aug 24;19(8):e1010888. doi: 10.1371/journal.pgen.1010888 (PMC10470897; doi:10.1371/journal.pgen.1010888)
Supplement: Attachment — Submitted filename: CoverLetter_ReviewerResponse_Jul23.pdf [file pgen.1010888.s025.pdf]

Stellenbosch
UNIVERSITY
IYUNIVESITHI
UNIVERSITEIT

Medicine and Health Sciences
EzoNyango nezeeNzululwazi kwezeMpilo
Geneeskunde en Gesondheidswetenskappe

**The Editor**
***PLOS Genetics***

**06 July 2023**

**Dear Professors Stein and Williams,**

Thank you for this opportunity to revise our manuscript. Please find our point-by-point response to the Reviewers comments below.

**Reviewer #1:** In their manuscript "Altered neutrophil extracellular traps in response to Mycobacterium tuberculosis in persons living with HIV with no previous TB and negative TST and IGRA", Kroon and colleagues explore neutrophil gene programs that distinguish a clinical phenotype of great interest. They compare well-defined cohorts of PLWH in South Africa who have presumed exposure to M. tuberculosis, which is based both on residence in a high endemic setting and Mtb-specific antibody responses, in order to explore potential mechanisms by which some immune-reconstituted PLWH lack canonical Mtb sensitization (TST/IGRA reactivity) with the hope to identify non-canonical mechanisms that protect them from TB progression. The manuscript is well crafted and written, and while it builds on a growing literature that explores IFNg independent mechanisms of TB protection among similarly defined clinical phenotypes (TST/IGRA based), the authors add novelty by focusing on PLWH where protective mechanisms may be distinct. Another strength is the inclusion of immunofluorescent microscopy evidence of NET formation to validate transcriptional findings.

While overall the composition is clear, there are a few areas where the narrative that builds from global transcriptional profiling (e.g. DEG identification and pathway enrichments) to experimental NET data could be made more clear.

Major comments:

Title:
- Title is confusing, perhaps because TST and IGRA syntax is awkward. Consider an alternative such as "Neutrophil extracellular trap formation and gene programs distinguish TST/IGRA sensitization outcomes among M. tuberculosis exposed persons living with HIV."

We thank the reviewer for their valuable insight and suggestions. We added the new title as: “**Neutrophil extracellular trap formation and gene programs distinguish TST/IGRA sensitization outcomes among *Mycobacterium tuberculosis* exposed persons living with HIV**”.

Figures 2 and 3 and corresponding section (lines 189-210):
The various analyses lead to some confusion assuming the overall conclusion of this section appears in the section

heading. Examples and suggestions:

1) Figure 2 - Can a statistical statement be made whether the 6hour distributions (HITTIN versus HIT) are different? We thank the reviewer for this comment. We have updated the figure as below, and added the p-value in lines 234-237 : "Consistent with different numbers of DEGs identified for each phenotypic group, when we evaluated the statistical significance of expression changes between the HITTIN and HIT groups, we identified an overall dampened 6h transcriptomic response in $PMN_{HITTIN}$ compared to $PMN_{HIT}$ ($p < 2.2e^{16}$) (Fig 2).".

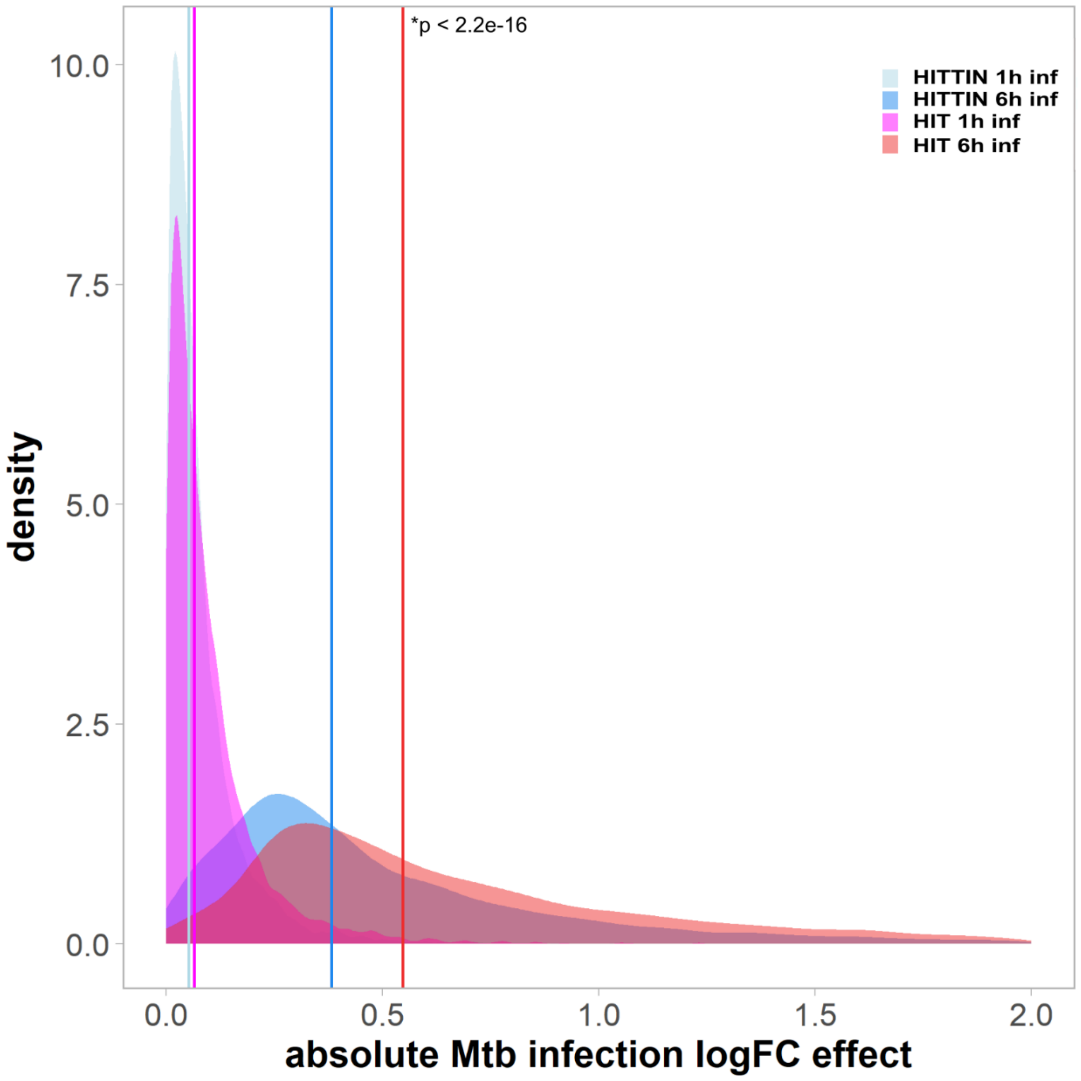


**Fig 2: The absolute log fold change *Mtb* infection effect at 1 and 6 h for $PMN_{HITTIN}$ and $PMN_{HIT}$**

The density plot shows the absolute log fold change (logFC) *Mtb* infection effect of each group at 1h and 6h. All DEGs from Fig 1 had their logFC converted to absolute values and plotted using density function. Absolute values

for neutrophil DEGs from HITTIN participants are shown in light blue at 1h (median = 0.051) and a darker blue at 6h (median = 0.383). Absolute values for neutrophil DEGs from HIT participants are shown in magenta at 1h (median = 0.065) and red at 6h (median = 0.550). Vertical coloured lines indicate the median of absolute values. $PMN_{HITTIN}$ show a significantly lower logFC response to *Mtb* infection at 6h ($p < 2.2e-16$, Wilcoxon rank sum test) compared to 1h of infection with *Mtb* ($p = 0.8255$, Wilcoxon rank sum test).

2) The importance of the log2FC correlations (Fig 3) is not evident and leads to confusion based on the section heading.
- the statement (line 199) "differential expression across phenotypes was correlated with log2FC" needs to be better defined. To what the 'differential expression' refer since log2FC also describes differential expression (media - infection).
- Am I correct in stating the null hypothesis in this sense has not been rejected (e.g. phenotype does not impact the global Mtb PMN response)? If that is true, I would consider moving Fig3 to supplemental and make a simple statement in Results such as 'despite overall lower log2FC among HITTIN, there was a strong correlation of log2FC values for each gene between HITTIN and HIT suggesting that Mtb responses globally are conserved across phenotypes.'

We thank the reviewer for this improvement. We have moved figure 3 to the supplementary material as S2 Fig. In addition, we replaced the section "Next, by correlating the expression changes of up- and downregulated DEGs, identified for each PMN infection phenotype, we observed that differential expression across phenotypes was correlated with the $log_2FC$. Despite differences in $log_2FC$ between groups, we observed a strong correlation of $log_2FC$ values between groups after 6h infection ($R = 0.96$) whilst the 1h correlation was weaker ($R = 0.75$) (Fig 3)." with "*Despite overall lower $log_2FC$ among HITTIN, there was a strong correlation of $log_2FC$ values for each gene between $PMN_{HITTIN}$ and $PMN_{HIT}$ suggesting that Mtb responses globally are conserved across phenotypes (S2 Fig).*"*Lines 237-239..*

3) Suggest removing 1h data in main results section to further streamline main conclusion and avoid unnecessary comparisons. It seems authors have concluded 1h is too early given insufficient transcriptional changes. The statement justifying a focus on 6h time point could be make earlier with reference to supplemental info for 1h timepoint data).

We agree with and thank the reviewer for this comment. We have moved the relevant 1h data as S1 Fig to the supplementary material. In addition, we moved the section on the interaction contrast earlier to now read as follows:

Lines 199-227: "*Mtb* infection triggered significant gene expression changes when compared to uninfected PMN at 1h: 151 up- and 40 downregulated genes for $PMN_{HITTIN}$ while 98 genes were up- and 11 were downregulated for PMN from the HIT group ($PMN_{HIT}$) (S1 Fig A and B, S3 Table). A higher number of *Mtb* activated gene expression

changes were observed at 6h post infection compared to 6h uninfected with 3106 up- and 3548 downregulated DEGs for HITTIN and 3816 up- and 3794 downregulated DEGs for HIT (Figs 1A and B, S3 Table). Since the 1h time point showed limited differences in gene induction by *Mtb*, we focused on the 6h time point differences between PMN phenotypes in subsequent analyses (S1 Fig)."

When comparing transcriptional responses of infected $PMN_{HITTIN}$ and $PMN_{HIT}$ directly, no significant DEG were identified at 1h (S1 Fig C, S3 Table). However, contrasting the effect differences for *Mtb* infection compared to no infection at 6h for each group ($PMN_{HITTIN}$ vs $PMN_{HIT}$), we identified 2285 genes with significant differential response between the two groups (Fig 1C, S3 Table). Since the 1h time point showed limited differences in gene induction by *Mtb*, we focused on the 6h time point differences between PMN phenotypes in subsequent analyses."

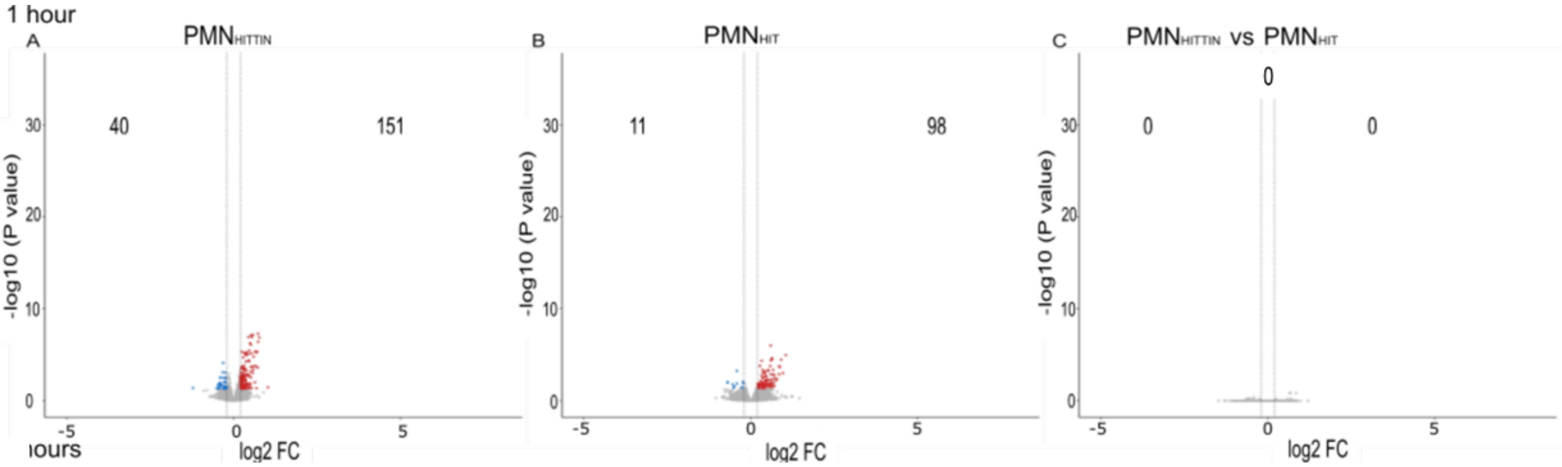


**S1 Fig: Volcano plots of differential gene expression at 1h infection by PMN from HITTIN and HIT**

Volcano plot for transcriptional responses to *Mtb* challenge for neutrophils from HITTIN ($PMN_{HITTIN}$) and HIT ($PMN_{HIT}$) participants at 1h **(A-C)** post *Mtb* infection. The y-axis shows the negative log10 unadjusted P value and the x-axis the $\log_2$ fold change (FC). The vertical dashed lines represent $\log_2$ FC thresholds of -0.2 and 0.2. Each gene is represented by a dot. Genes which are downregulated or upregulated as determined by the FDR ≤ 5% are shown in blue and red, respectively. Genes with non-significant expression changes and below the $\log_2$ FC threshold are shown in grey. Differentially expressed genes at 1h post-infection compared to 1h uninfected PMN from HITTIN ($PMN_{HITTIN}$) **(A)** and HIT participants ($PMN_{HIT}$) **(B)**. Significant differentially triggered genes between $PMN_{HITTIN}$ and $PMN_{HIT}$ at 1h post infection **(C)**.

HITTIN vs HIT interaction DEG model:
(lines 205 - 210) - The model used when comparing HITTIN against HIT needs [a brief] reference in main results

section. Presumably these 2285 DEGs reflect the interaction between (uninfected - Mtb) *(HIT - HITTIN), but is restricted to the 6h timepoint? As written, it suggests a simple contrast model was used that is restricted to Mtb-infected samples (6h) only.

We thank the reviewer for this comment and their suggestion for improvement. The reviewer is correct in stating that these 2285 DEGs reflect the interaction between (uninfected - *Mtb*) *(HIT - HITTIN), but restricted to the 6h timepoint. We have corrected this by changing it to: "However, contrasting the effect differences for *Mtb* infection compared to no infection at 6h for each group ($PMN_{HITTIN}$ vs $PMN_{HIT}$), we identified 2285 genes with significant differential response between the two groups (Fig 1C, S3 Table)." Lines 222-227.

I also note in the methods section that infection time (1h - 6h) was also included in the DEG model that may further lend confusion to interpretation of this main results section (and 2285 DEGs).

We tried to highlight the contrasts between the groups and timepoints by changing it as follows (lines 778-787):

Contrasts were made using makeContrasts and defined as:

i) Group specific *Mtb* infection compared to no infection effect at 1h ($\beta_{HITTIN1inf}, \beta_{HIT1inf}$)

ii) Group specific *Mtb* infection compared to no infection effect at 6h ($\beta_{HITTIN6inf}, \beta_{HIT6inf}$)

iii) Differential response between groups at 1h infected

$$(\beta_{HITTIN1inf}) - (\beta_{HIT1inf})$$

iv) Differential response between groups at 6h infected

$$(\beta_{HITTIN6inf}) - (\beta_{HIT6inf})$$

Directionality of NET term enrichments (and NADPH oxidase DEGs).
(line 229) Reference to 'terms that directly relate to a possible increased microbicidal activity of PMN-HITTIN' leads to a confusing directionality based on subsequent sections that highlight genes that actually have reduced expression in HITTIN.

Thank you for this comment. We changed the paragraph as follows: "Manhattan plots for the three term analyses at 6h are shown in Fig 3 with significantly different terms and pathways of interest indicated. Amongst the enriched terms in $PMN_{HITTIN}$, were "Apoptosis", "Neutrophil extracellular trap formation", and "NADPH regeneration", which are terms that directly relate to microbicidal activity of $PMN_{HITTIN}$ (Fig 3 B). Although these terms are mostly driven by genes with significant positive fold change (less downregulated) (Fig 3 B), the overall

enrichment of the terms is influenced by genes which are less up- and downregulated (Fig 3 A, Table 3). This was also the case for terms with genes triggered relatively less strongly in $PMN_{HITTIN}$ compared to $PMN_{HIT}$. These were dominated by genes involved in neutrophil chemotaxis, neutrophil degranulation, necroptosis and necrotic death (Fig 3 C). It is important to note that the overall response to *Mtb* was always lower in $PMN_{HITTIN}$ compared to $PMN_{HIT}$.

We used fluorescent microscopy to evaluate the biological outcome in the total amount of NETs observed between HITTIN vs HIT and focused on the DEGs associated with the "Neutrophil extracellular trap formation" pathway in KEGG (Table 3)." Lines 280-311

- (line 230) should be 'Fig 4B'.

We thank the reviewer for the comment. The reference was meant for now Fig 3B. We have corrected this in line 284.

- NADPH oxidase genes (RAC2, CYBA, CYBB, NFKB1, NCF2) had decreased expression among HITTIN as described in Results and negative log2FC in Table 3. The positive enrichment among HITTIN of the NET formation pathway (higher NETs among HITTIN?) lends well to the later experimental data but seems contradictory to the gene-level directionality that is highlighted.

The NADPH oxidase genes (*RAC2, CYBA, CYBB, NFKB1, NCF2*) showed positive $log_2$FC response in both HITTIN and HIT. In relation to HIT, HITTIN had a lower positive $log_2$FC response and therefore the contrast shows it as a negative log2FC. Overall, the $log_2$FC response to *Mtb* is lower in HITTIN which corresponds to lower NET formation. We also included an additional statement in lines 284-291 to try clarify the overall response seen. "Although these terms are mostly driven by genes with significant positive FC (less downregulated) (Fig 3 B), the overall enrichment of the terms is influenced by genes which are less up- and downregulated (Fig 3 A, Table 3). This was also the case for terms with genes triggered relatively less strongly in $PMN_{HITTIN}$ compared to $PMN_{HIT}$. These were dominated by genes involved in neutrophil chemotaxis, neutrophil degranulation, necroptosis and necrotic death (Fig 3 C). It is important to note that the overall response to *Mtb* was always lower in $PMN_{HITTIN}$ compared to $PMN_{HIT}$."

In addition, at the end of the section entitled "NET area change difference between HITTIN and HIT from 1 to 6h after *Mtb* infection", we added: "The transcriptional response for "Neutrophil extracellular trap formation" was driven by genes with significant positive FC (less downregulated), however the imaging data revealed overall lower NET formation in $PMN_{HITTIN}$. These findings were in line with the overall lower transcriptional response observed in $PMN_{HITTIN}$.". Lines 357-360

-Reordering these sections by including the NET microscopy data immediately after reporting the positive HITTIN enrichment for the NET formation pathway in 4B may be easier to follow. Once the conclusion that NETs are

lower in HITTIN is made, then further exploration at the gene-level can be made (Table 3 and results section 'DEGs in HITTIN vs HIT...').

We thank the reviewer for this comment and suggestion. We agree that this will improve the flow and understanding of the section, and we have changed it as suggested.

Lines 307-399:" We *used fluorescent microscopy to evaluate the biological outcome in the total amount of NETs observed between HITTIN vs HIT* and focused on the DEGs associated with the *"Neutrophil extracellular trap formation" pathway in KEGG (Table 3).*

**NET area change difference between HITTIN and HIT from 1 to 6h after *Mtb* infection**

We stained fixed cells which were processed in parallel with the cells used for RNAseq. NETs were stained using anti-H2AH2B/DNA (PL2-3) which detects decondensed chromatin and nuclear DNA stained with Hoechst, and the area of both features was quantified.

As a measure of cellular viability over time we compared the total change in cell nuclei area between cells fixed at 1h and 6h of infection with *Mtb.* There was no significant two way interaction between $PMN_{HITTIN}$ and $PMN_{HIT}$ and the infection status, F(1,12)=1.1870, p=0.30, including a similar trend in direction of response for both PMN. However, there was a significant *Mtb* infection effect, F(1,12)=9.7290, p=0.009, with pairwise comparisons showing a significantly greater decrease in total cell nuclei area between 1h and 6h after *Mtb* infection for $PMN_{HIT}$ compared to $PMN_{HITTIN}$ (p=0.04, pairwise t-test) (Fig 4 and S3 Fig).

When then comparing difference in NET area at 1h vs 6h, there was a statistically significant interaction between the PMN groups and infection status, F(1, 10.5924) = 5.3398, p < 0.0421). The simple main effect of phenotype group (considering the Bonferroni adjusted p-value) was significant for *Mtb* infection (p=0.0007), but not for non-infection (p=1). Consistent with the greater viability of $PMN_{HITTIN}$ at 6h of infection, pairwise comparisons show that $PMN_{HITTIN}$ also induce a significantly smaller change in NETs produced between 1h and 6 h of infection, compared to $PMN_{HIT}$ (p=0.0003) (Fig 4 and S3 Fig). Although the transcriptional response for "Neutrophil extracellular trap formation" *was* driven by genes with significant positive FC (less downregulated), the imaging data revealed overall lower NET formation in $PMN_{HITTIN}$. These findings were in line with the overall lower transcriptional response observed in $PMN_{HITTIN}$.

**DEGs in HITTIN vs HIT after 6h *Mtb* infection in the "Neutrophil extracellular trap formation" pathway**

We next investigated the DEGs associated with the *"Neutrophil extracellular trap formation" enriched in the combined KEGG pathway (Fig 3A, Table 3).* In particular, the lower transcriptional and NET response in $PMN_{HITTIN}$ prompted further evaluation of genes which showed a lower upregulated response to *Mtb* in $PMN_{HITTIN}$ compared to $PMN_{HIT}$. Compared to $PMN_{HIT}$, *Mtb* infection of $PMN_{HITTIN}$, triggered a lower upregulation of genes involved in the multiple-protein NADPH oxidase complex including *Rac family small GTPase 2* (*RAC2*), and the transmembrane catalytic [cytochrome b-245 -alpha (*CYBA*) and -beta (*CYBB*)]. NADPH oxidase Nox2 (encoded by CYBB) and other cellular NADPH oxidases is involved with ROS production and NET formation (29–31). CYBB is a

nuclear factor kappa B (NF-κB) transcriptional target and together with NFKB1 was also less upregulated in $PMN_{HITTIN}$. Interestingly, NCF2, the gene encoding neutrophil cytosolic factor 2 (NCF-2 or p67-phox) was downregulated in $PMN_{HITTIN}$ and upregulated in $PMN_{HIT}$. $PMN_{HITTIN}$, also showed enrichment for DEGs related to "NADPH regeneration" (Fig 3A, B). NADPH can dually aid in ROS detoxification or production and is key for ROS mediated NET formation (30,32,33).

Histone deacetylases (HDACs) play a key role in NET formation and allow for peptidylarginine deiminase 4 (PAD4) mediated histone citrullination the initial step in chromatin decondensation (34,35). Compared to $PMN_{HIT,}$ $PMN_{HITTIN}$ had a less downregulated response in *HDAC1*, *HDAC3, HDAC4* and *PADI4* at 6h of *Mtb* infection. *Gasdermin D* (*GSDMD*) which plays a key role perforating the nuclear membrane to aid release of the decondensed chromatic during NET formation (36,37), also displayed the same pattern of expression regulation. *Caspase 1* (*CASP1*) and *4* (*CASP4*) which activate GSDMD have a lower upregulation in $PMN_{HITTIN}$ compared to $PMN_{HIT}$ after 6h *Mtb* infection (38).

Other DEGs enriched in the KEGG NET pathway, are also involved in additional neutrophil functional responses. Cell membrane receptors *TLR2* and *TLR4* were less upregulated in $PMN_{HITTIN}$ in response to 6h *Mtb* infection. Downstream of TLR4, pathway activation of NF-κB, Protein Kinase B (AKT) and phosphoinositide 3-kinase (PI3-K) lead to pro-survival mechanisms (39). Integral to this TLR signaling system is mitogen-activated protein kinase (MAPK) and PI3-K. Dysregulation in especially the PI3-K/AKT signaling system contributes to an imbalance in neutrophil chemotaxis and can heighten inflammation and decrease pathogen clearance (40,41). *MAP2K2* was less upregulated while *MAPK1, MAPK3, AKT1, AKT2* and *PIK3CD* were less downregulated in $PMN_{HITTIN}$. Azurocidin 1 (*AZU1*), the only antimicrobial peptide gene also included in the NET term, was downregulated after 6h *Mtb infection in $PMN_{HITTIN}$ whilst upregulated in infected $PMN_{HIT}$ (Table 3).*"

Limitations
- Considering HITTIN have lower magnitude of transcriptional changes following Mtb infection overall, a contribution from non-PMN populations in the experiment should be considered. Contaminating T cells could plausibly contribute to IFNg-driven PMN transcriptional differences that are detected according to the pre-defined clinical phenotype.
- It would be appropriate to mention this limitation, and that good PMN purity (90%, T cells ~5%) and the 6h timepoint that is short for paracrine effects argue against a significant impact of contaminating cell types.
- Considering the sensitivity of RNAseq it would be further reassuring to confirm there are not other IFNg pathway-related enrichments between HIT vs. HITTIN.
- Note that IFNg even after short stimulation times can impact PMN NADPH oxidase expression (and NADPH production) in vivo:
- https://doi.org/10.1371/journal.pone.0263370
- https://doi.org/10.1182/bloodadvances.2021005776

We thank the reviewer for highlighting this important limitation.

We have added the following to the manuscript:

"The effect of contaminating T cells in PMN cultures cannot be completely excluded. T cells could possibly contribute to IFN-γ driven PMN transcriptional differences with IFN-γ known to upregulate key genes affecting NADPH activity (65,66). Per clinical classification, HITTIN lack IFN-γ T cell responses as measured by IGRA. In addition, good PMN purity (90%) vs 5% in T cells, as well as the 6h timepoint which is short for the IFN-γ driven responses, argue against the significant impact of contaminating cells. Further classification of non IFN-γ T cell subsets in HITTIN is needed. " Lines 514-520.

Below we include Manhattan plots for the reviewer to highlight IFN-γ related pathways from enrichment tests of GO, Kegg and Reactome pathways. In addition, we are including the full GO, KEGG and Reactome results for the interaction as well as group infection responses after 6h *Mtb* infection.

The Manhattan plot shows the **IFN-γ specific pathways and GO terms** for the DEGs triggered significantly differentially by *Mtb* in neutrophils from HITTIN versus neutrophils from HIT participants after 6h of infection.

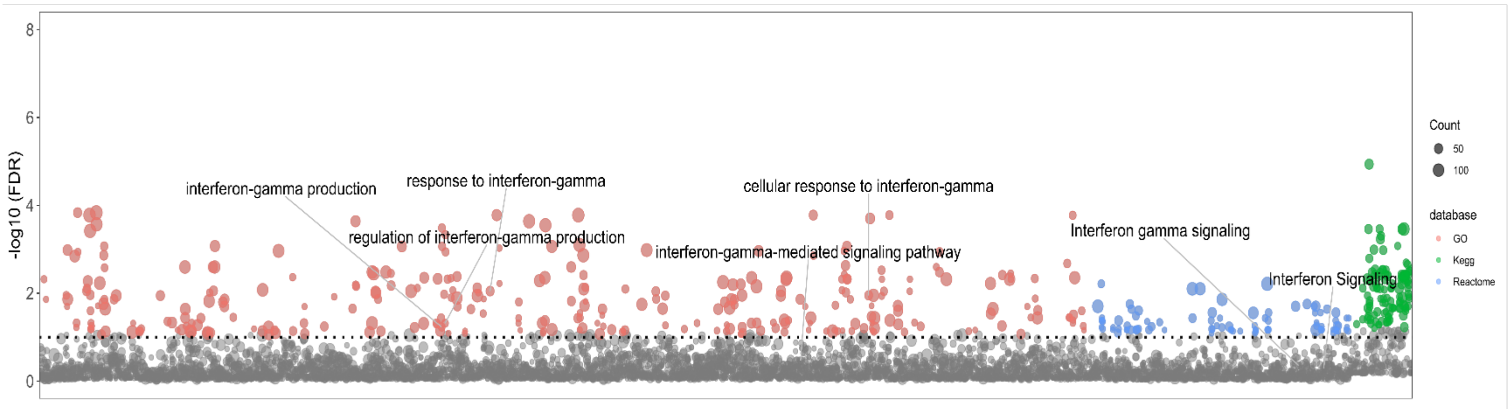


Pathways and plots detected by all signicant DEGs (up and downregulated).

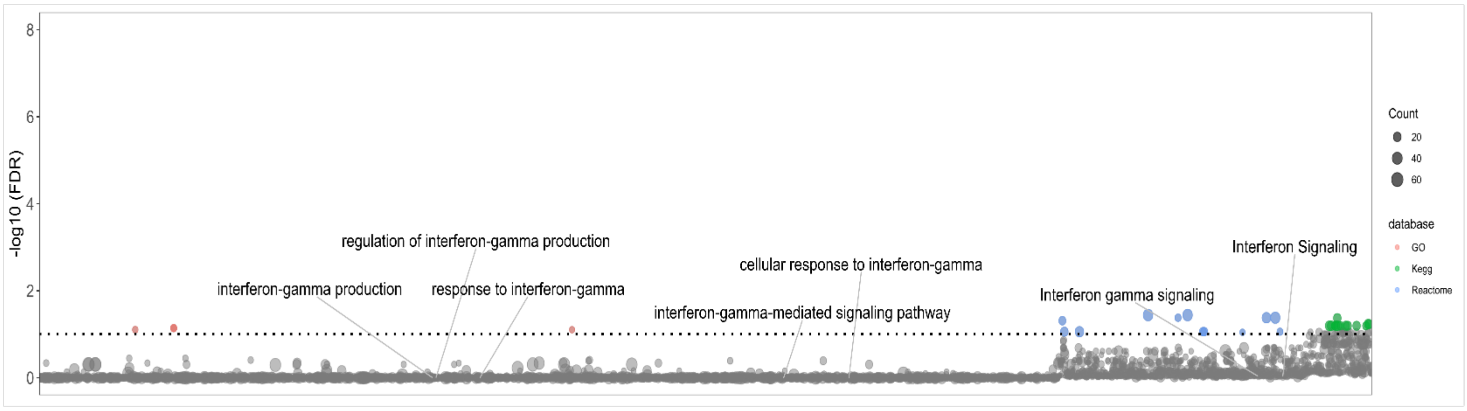


Pathways and plots detected by all signicant DEGs which were less downregulated in interaction/ positive FC.

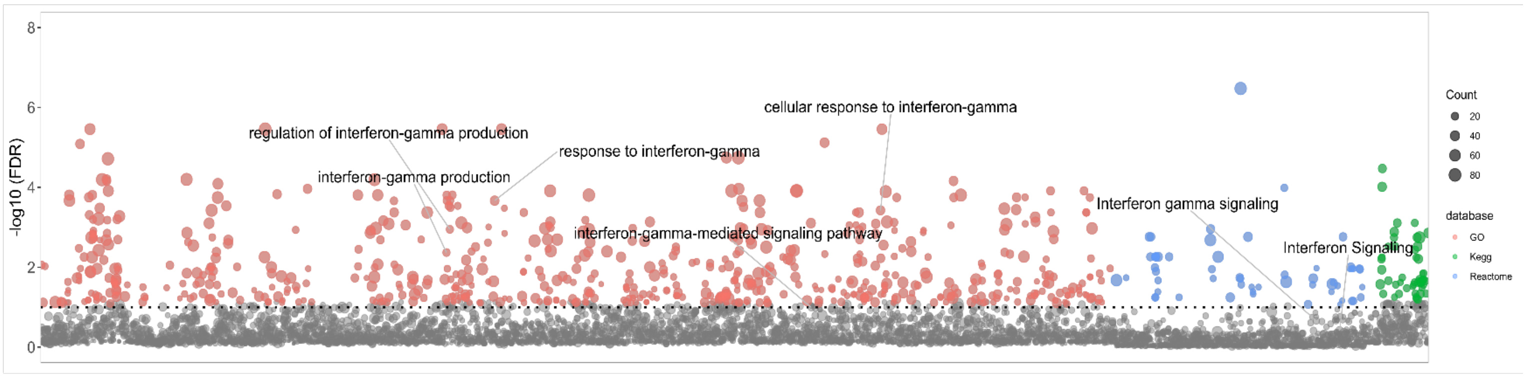


Pathways and plots detected by all signicant DEGs which were less upregulated in interaction/ negative FC.

Minor comments:

- Data access: Submission of data to the European Genome-phenome Archive (EGA) is currently being reviewed. Please confirm submission is processed/finalized.

The data was submitted and is now a closed submission on EGA as "Neutrophils as effector cells in resistance to infection by *Mycobacterium tuberculosis* in HIV- infected individuals" with the study ID EGAS00001007262 (https://ega-archive.org/studies/EGAS00001007262).

- (lines 59-61; awkward syntax); Consider "some PLWH never develop TB and show no evidence of immune sensitization to Mycobacterium tuberculosis (Mtb) as defined by persistently negative tuberculin skin tests (TST) and interferon gamma release assays (IGRA)."
We thank the reviewer for this suggestion. It was incorporated into lines 59-61.

"After prolonged and repeated exposure, some PLWH never develop TB and show no evidence of immune sensitization to *Mycobacterium tuberculosis* (*Mtb*) as defined by persistently negative tuberculin skin tests (TST) and interferon gamma release assays (IGRA)."

- Consider including FDR threshold in Abstract to define significant

We thank the reviewer for the comment and added the following to lines 70-74: "When compared to uninfected PMN, $PMN_{HITTIN}$ displayed 151 significantly upregulated and 40 significantly downregulated differentially expressed genes (DEGs) (absolute cutoff of a log2FC of 0.2, FDR < 0.05) whereas $PMN_{HIT}$ demonstrated 98 significantly upregulated and 11 significantly downregulated DEGs following 1h *Mtb* infection."

- (lines 67 - 72) - differentially expressed is not defined initially and leads to confusion since the primary comparison as defined above is HITTIN versus HIT. Suggest better delineating with "When compared to uninfected PMNs, PMNhittin displayed 151 unregulated and 40 down regulated differentially expressed genes (DEGs) (FDR XX) whereas PMNhit demonstrated 98 upregulated and 11 downregulated DEGs following 1h of Mtb infection.

- (line 72) - As above, consider adding "...3794 significantly downregulated DEGs when comparing Mtb-infected and uninfected PMNs.

We thank the reviewer for the two previous comments. We responded to both as below:

"When compared to uninfected PMN, $PMN_{HITTIN}$ displayed 151 significantly upregulated and 40 significantly downregulated differentially expressed genes (DEGs) (absolute cutoff of a log2FC of 0.2, FDR < 0.05) whereas



Division of Molecular Biology and Human Genetics
Department of Biomedical Science | Faculty of Medicine and Health Sciences
Biomedical Research Institute, Francie Van Zijl Drive, Tygerberg, 7505
·www.sun.ac.za/mbhg

$PMN_{HIT}$ demonstrated 98 significantly upregulated and 11 significantly downregulated DEGs following 1h *Mtb* infection. At the 6h timepoint, $PMN_{HITTIN}$ displayed 3106 significantly upregulated and 3548 significantly downregulated DEGs while $PMN_{HIT}$ had 3816 significantly up- and 3794 significantly downregulated DEGs when comparing *Mtb*-infected and uninfected PMN." Lines 70-77

- (lines 111 - 120) Is there a reason why the PMN abbreviation is not assigned after first neutrophil use, or at least why this sentence is chosen for PMN abbreviation when neutrophil is spelled out later as well?

We thank the reviewer for the comment. This was corrected as follows:

"We hypothesized that the innate immune system, and specifically neutrophils (PMN), play an inherent role in the protective control of *Mtb* infection in HITTIN. PMN are the most abundant leukocytes and among the first responders to *Mtb* infection in the lung in animal models as well as humans (7). They are armed with an arsenal of antimicrobial granules known to restrict *Mtb* growth and are key players in the inflammatory response against *Mtb* (8–11). PMN can control *Mtb* growth during acute infection (8,11,12). Household pulmonary TB contacts with higher initial peripheral neutrophil counts were less likely to become infected with *Mtb* (11). Despite lower RNA expression in PMN compared to other innate immune cells, pathogen-triggered gene expression changes underlie microbial responses by PMN (13,14). " Lines 116-125

- (line 155-158) consider change to "...11 HIT individuals, all of whom were PLWH and on ART, were used in the final analysis (Table 1). These individuals were part of stringently defined cohorts living in a high TB burden community who despite low CD4+ counts before ART initiation never developed TB."

We thank the reviewer for the comment and have corrected it as suggested. Lines 160-163: "Neutrophils obtained from 17 HITTIN and 11 HIT individuals, all of whom were PLWH and on ART, were used in the final analysis (Table 1). These individuals were part of stringently defined cohorts living in a high TB burden community who despite low CD4+counts before ART initiation never developed TB."

- Figure 1 - Legend suggests vertical line reflects log2FC of -/+ 0.02 whereas results section (and visual inspection) suggests this should be -/+ 0.2.

We thank the reviewer for picking up on this error. This has been corrected to 0.2.

- Figure 4 - legend (line 1057) should have 'HIT' not 'HT'.

We thank the reviewer for picking up on this error. This has been corrected to HIT.

- (line 230) reference should be Fig 4B.

We thank the reviewer. This has been addressed as follows: "Amongst the enriched terms in $PMN_{HITTIN}$, were "Apoptosis", "Neutrophil extracellular trap formation", and "NADPH regeneration", which are terms that directly



Division of Molecular Biology and Human Genetics
Department of Biomedical Science | Faculty of Medicine and Health Sciences
Biomedical Research Institute, Francie Van Zijl Drive, Tygerberg, 7505
·www.sun.ac.za/mbhg

relate to microbicidal activity of $PMN_{HITTIN}$ (Fig 3 B).” Lines 281-284

- (line 244-245) change to "NADPH oxidation by Nox2 (encoded by CYBB) and other cellular NADPH oxidases is involved with ROS production and NET formation (29-31)."

We thank the reviewer for this suggestion. We have changed this as suggested. Lines 371-373: “NADPH oxidase Nox2 (encoded by *CYBB*) and other cellular NADPH oxidases is involved with ROS production and NET formation (29–31).”

- (line 277) should be "induced at 1h and 6h post infection".

We thank the reviewer for this comment. Due to changing the order of the results, we have removed this sentence.

- (lines 291 - 292) Incomplete sentence.

We thank the reviewer for the comment. We changed the sentence as follows:

The simple main effect of phenotype group (considering the Bonferroni adjusted p-value) was significant for Mtb infection (p=0.0007), but not for non-infection (p=1). Lines 353-354.

- (line 322) change to "Mtb-induced ROS triggered necrosis in neutrophils..."

We thank the reviewer for the suggestion. The correction was made in lines 450-451.

“*Mtb*-induced ROS triggered necrosis in neutrophils and decreased the ability of macrophages to control *Mtb* growth (18). ”

- (line 349) change to "TLR2/4 signalling could also mediate NET formation independent of ROS" or "ROS-independent mechanisms could also mediate NET formation downstream of TLR2/4."

We thank the reviewer for the comment. We changed it using: “TLR2/4 signaling could also mediate NET formation independent of ROS” Line 477.

- (line 371-372) suggest change to "....maintained by PMN-HITTIN, which demonstrate lower NET formation in response to Mtb infection despite a positive enrichment of NET-related genes as compared to PMN-HIT."

Thank you for this suggestion, we have incorporated the change in lines 500-503: “This intricate balance is likely maintained by $PMN_{HITTIN}$, which demonstrate lower NET formation in response to *Mtb* infection despite a positive enrichment of NET-related genes as compared to $PMN_{HIT}$.”

**Reviewer #2:** In this manuscript, Kroon and colleagues present the results of a RNAseq study on neutrophils in response to Mycobacterium tuberculosis (Mtb) challenge in persons living with HIV who are at high risk of TB. They compared the transcriptome profile of neutrophils after Mtb infection in 17 HIV+ persistently TB, tuberculin and IGRA negative (HITTIN) participants living in a community with high TB burden and 11 individuals living with HIV from the same community with no TB history, but who test persistently IGRA positive, and tuberculin positive (HIT). After 6h of infection, neutrophils of HITTIN participants showed an overall transcriptional impairment as compared to HIT participants, consistent with the lower number of differentially expressed genes (DEGs) in HITTIN vs HIT sujects (3106 vs 3816 up-regulated genes and 3548 vs 3794 down-regulated genes). When comparing the response to Mtb between HITTIN and HIT individuals, the authors identified 2285 significant genes. Genes with a significant positive fold change (less downregulated by neutrophils from HITTIN individuals, N=1068) were enriched in « Apoptosis », « Neutrophil extracellular trap formation », and “NADPH regeneration” pathways. Interestingly, lower neutrophil extracellular trap formation was observed by fluorescence microscopy in HITTIN compared to HIT participants.
This manuscript tackles an important subject in the field of tuberculosis and suggest that NETosis could play a role in the early control of Mtb infection. I do, however, have some concerns regarding the robustness and interpretation of the findings.

1) My main concern pertains to the overall transcriptional impairment after 6h of Mtb infection observed in neutrophils of HITTIN participants compared to HIT participants. I am wondering whether it could be attributed to systematic differences not accounted for between the two groups that influence the transcriptional responsiveness of neutrophils to Mtb. Notably, previous studies by some of the authors have demonstrated that antiretroviral therapy (ART) significantly affects the transcriptional responsiveness of alveolar macrophages to Mtb (Correa-Macedo et al., JCI, 2021). Hence, I am curious if the duration of ART treatment in the participants could partially account for the observed overall impairment in neutrophils. It is essential to investigate, account for, and discuss this possibility.

We thank the reviewer for highlighting the important effect of ART on the transcriptional response of alveolar macrophages to *Mtb*. We take note of this omission and have addressed it we included the following information in the manuscript. In table 1 we specified time on ART as the average time on ART in years. HITTIN were on ART for an average of 7.35(3.15) years and HIT for an average of 8.97 (3.2). Although HITTIN were on ART for a shorter time, the difference was not significant (p=0.2) and therefor it is unlikely to be the driving factors for transcriptional differences observed between the neutrophils from the two groups.

Lines 171-175: “ Participants had controlled viral loads*, with participants in the HITTIN group having been on ART for 7.35 (±3.15) and HIT for 8.97 (±3.2) years. Although HITTIN was on ART for a shorter period there was no significant difference to the time spent in ART in HIT (p=0.2, Wilcoxon rank sum exact test).”*

Lines 509-513: *“Use of ART can significantly affect the transcriptional responsiveness of alveolar macrophages to Mtb. It is important to note that although HITTIN were on ART for a shorter time, there was no significant difference to the time spent on ART in HIT and therefore ART is an unlikely factor driving the transcriptional differences observed between neutrophils from HITTIN and HIT (64). ”*

2) Related to the previous point, I am also concerned about how this transcriptional impairment may have impacted the results of the differential gene expression (DEG) analysis between HITTIN and HIT individuals. The ability to detect differences between the two groups is likely higher for genes that exhibit a robust response in HIT participants following Mtb infection. Consequently, I am wondering whether the enriched pathways could reflect the general response of neutrophils to Mtb rather than a differential expression pattern between HITTIN and HIT groups. It would be helpful to show the results of the pathway enrichment analysis in response to Mtb performed separately for HITTIN and HIT. Are the terms « Apoptosis », « Neutrophil extracellular trap formation », and “NADPH regeneration” significantly enriched in differentially expressed genes (especially down-regulated)? How do they rank in terms of significance in the two groups? This should be discussed.

We thank the reviewer for their comment. The reviewer is correct, a response to *Mtb* infection is expected in both groups and what we measure is indeed a reflection of the overall impaired transcriptional response. Consequently, no claim can be made about a specific effect. Rather what we are reporting is a less pronounced transcriptional response that also effects pathways known to represent anti-mycobacterial activity. With this analysis we show that a quantitative difference reaching a threshold (as determined by the multiple Bonferroni correction FDR) leads to a qualitative difference observed between phenotypes.

The individual infection response per group measures the overall DEG due to *Mtb* infection – no infection (NI) after 6 hours, i.e (6 hr HITTIN *Mtb* infection-NI) and (6 hr HIT *Mtb* infection-NI). With the interaction difference we determine the DEG meeting the significance threshold of genes with a differential response between the two group (6 hr HITTIN *Mtb* infection-NI)- (6 hr HIT *Mtb* infection-NI). We have included the full set of KEGG, GO and Reactome terms for each response to be added as a supplement. In addition, for the purpose of this response, we show separate Manhattan plots figures for HITTIN and HIT with pathways and GO terms for DEGs when comparing the 6h *Mtb* infection vs no infection effect (see below).

We added: “Despite overall lower $log_2$FC among HITTIN, there was a strong correlation of log2FC values for each gene between $PMN_{HITTIN}$ and $PMN_{HIT}$ suggesting that *Mtb* responses globally are conserved across phenotypes (S2 Fig). Irrespective of up- or downregulation of specific genes, the absolute response to *Mtb* after 6h was always smaller in $PMN_{HITTIN}$.” (based on reviewer 1’s suggestion lines 237-241) and “We measure a reflection of the overall impaired transcriptional response and consequently no claim can be made about specific effect. Rather, we are reporting a less pronounced response to *Mtb* infection in HITTIN after 6h that also effects pathways known to represent anti-mycobacterial activity.”(lines 537-540)

The Manhattan plot shows pathways and GO terms for the DEGs triggered significantly differentially by *Mtb* in neutrophils from HITTIN when comparing 6h Mtb infection vs no infection response.

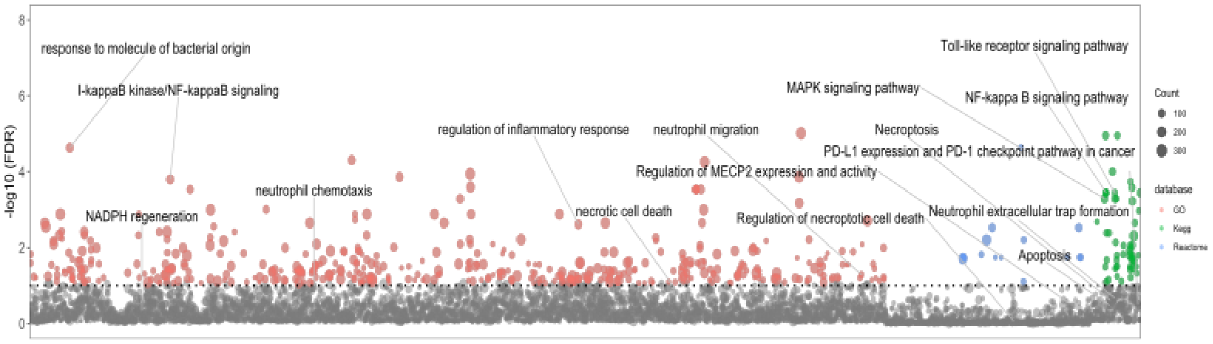


HITTIN Up- and Downregulated 6h *Mtb* infection vs no infection

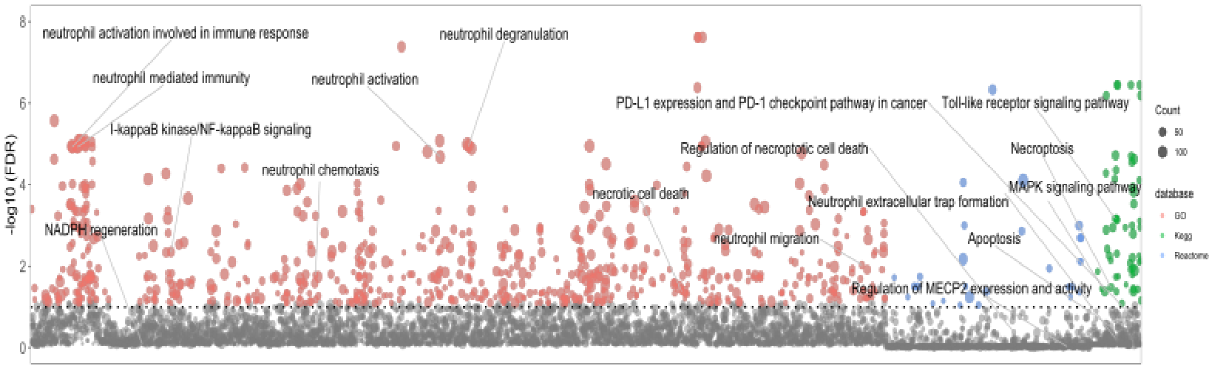


HITTIN Upregulated 6h *Mtb* infection vs no infection

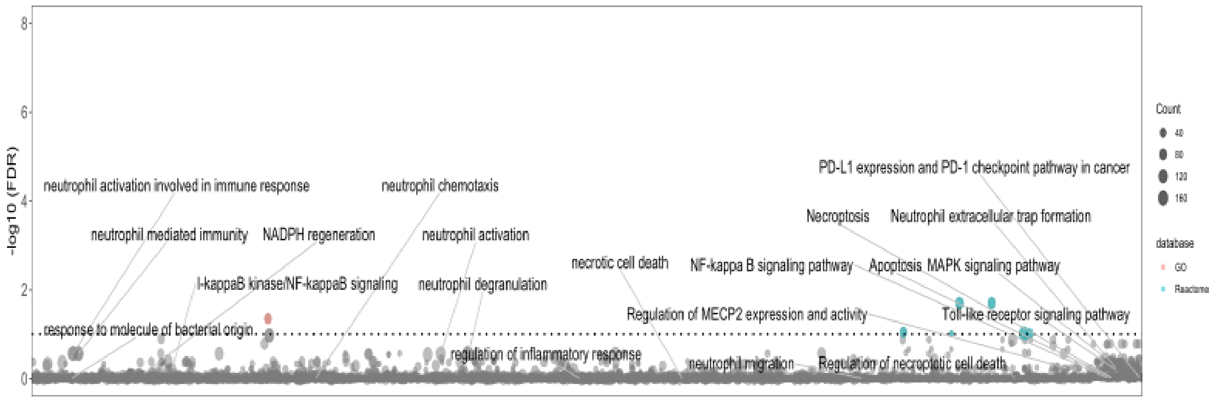


HITTIN Downregulated 6h *Mtb* infection vs no infection

The Manhattan plot shows pathways and GO terms for the DEGs triggered significantly differentially by *Mtb* in neutrophils from HIT when comparing 6h Mtb infection vs no infection response.

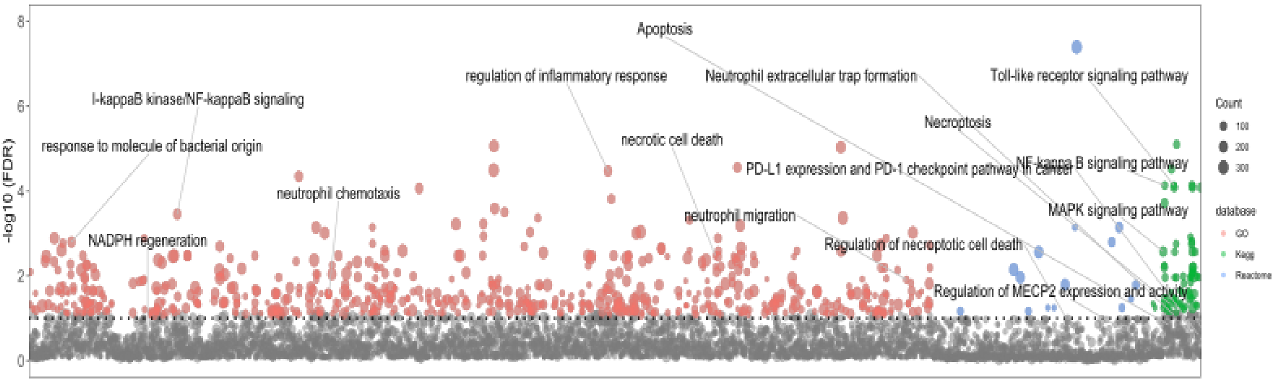


HIT Up- and Downregulated 6h *Mtb* infection vs no infection

| ID | Description | GeneRatio | BgRatio | pvalue | p.adjust | qvalue | Count | fold.enrich | min.hits | sig.GOs |
|---|---|---|---|---|---|---|---|---|---|---|
| GO:0006740 | NADPH regeneration | 10/5708 | 16/10907 | 0.2879698 | 0.5503196 | 0.4496192 | 10 | 1.19426681850035 | TRUE | FALSE |
| hsa04613 | Neutrophil extracellular trap formation | 77/2776 | 121/5083 | 0.0264135 | 0.1103680 | 0.0830355 | 77 | 1.16521482839927 | TRUE | FALSE |
| hsa04210 | Apoptosis | 75/2776 | 121/5083 | 0.0592519 | 0.1909230 | 0.1436411 | 75 | 1.1349495081811 | TRUE | FALSE |

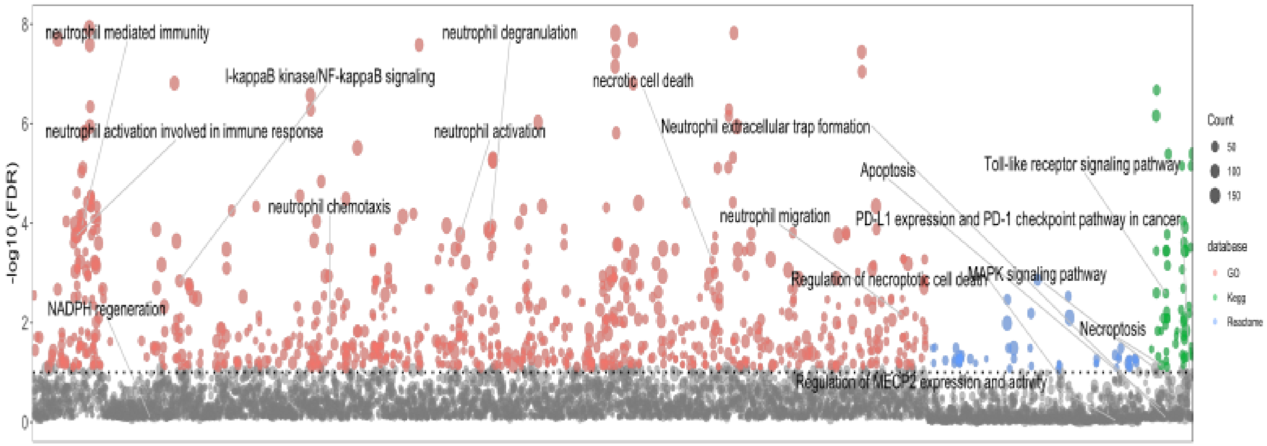


| ID | Description | GeneRatio | BgRatio | pvalue | p.adjust | qvalue | Count | fold.enrich | min.hits | sig.GOs |
|---|---|---|---|---|---|---|---|---|---|---|
| GO:0006740 | NADPH regeneration | 1/2654 | 16/10907 | 0.9884928 | 0.9981486 | 0.7976676 | 1 | 0.25685286360211 | FALSE | FALSE |
| hsa04613 | Neutrophil extracellular trap formation | 30/1339 | 121/5083 | 0.6857386 | 0.9778695 | 0.7962790 | 30 | 0.94118591029447 | TRUE | FALSE |
| hsa04210 | Apoptosis | 36/1339 | 121/5083 | 0.2224707 | 0.6260681 | 0.5098072 | 36 | 1.12942309235337 | TRUE | FALSE |

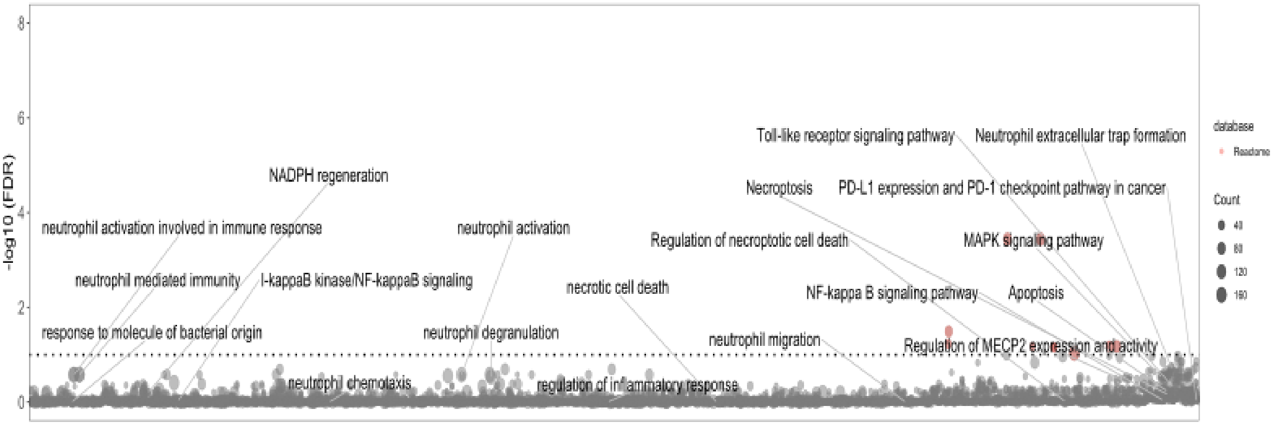


HIT Downregulated 6h *Mtb* infection vs no infection

| ID | Description | GeneRatio | BgRatio | pvalue | p.adjust | qvalue | Count | fold.enrich | min.hits | sig.GOs |
|---|---|---|---|---|---|---|---|---|---|---|
| GO:0006740 | NADPH regeneration | 9/3054 | 16/10907 | 0.0162060 | 0.6191793 | 0.6083655 | 9 | 2.00890225933202 | TRUE | FALSE |
| hsa04613 | Neutrophil extracellular trap formation | 47/1437 | 121/5083 | 0.0071286 | 0.2067309 | 0.1862317 | 47 | 1.37396550435078 | TRUE | FALSE |
| hsa04210 | Apoptosis | 39/1437 | 121/5083 | 0.1893300 | 0.6377112 | 0.5744767 | 39 | 1.14009903552511 | TRUE | FALSE |

3) I am wondering if normalizing the expression profile of the HITTIN subject after 6 hours of Mtb infection to render it more comparable to HIT participants could make sense and how it could impact on the results of the DEG analysis between HITTIN and HIT groups?

Thank you for this comment. Due to large inter-individual expression differences, we did not use expression levels in group comparisons, but rather blocked on subject. The limitation of this approach is that it precludes comparisons of group specific expression levels.

We added under the study limitations (lines 521-522): "To account for large inter-individual differences in response to *Mtb*, we employed subject-specific fold change. This approach precludes comparisons of group expression levels."

Reviewer #3: Very well written paper. This explores an important and relevant topic (understanding the immune response to TB infection in HITTIN and more broadly PLWH) and the authors explain and lay out the justification for this research appropriately. The introduction appropriately lays out the relevant background information on immune responses to TB, and the role of neutrophils/PMN's. Methodologically, the HIT group is an appropriate comparator for this study and the selection of subjects was adequately explored and justifiable, including an exploration of risk factors which would potentially serve as confounders. Differential gene expression in neutrophils/PMN's is an appropriate outcome for this question and the analysis was performed according to accepted standards. The authors further added to their understanding of the neutrophil response by examining the functional role of the DEG's they identified (showing enrichment for extracellular traps). Their microscopy validation was a nice way to wrap this up.

Overall, this answers an important research question and the study is appropriately performed and the paper is well written. The results are impactful and significant. The discussion was also well-written and helps contextualize the importance of these results.

We thank the reviewer for their feedback. It is greatly appreciated.

**Thank you for considering our revised manuscript.**

**Elouise E Kroon**
**MOLECULAR BIOLOGY AND HUMAN GENETICS**
**DIVISION OF MOLECULAR BIOLOGY AND HUMAN GENETICS**
